# NKL Homeobox Genes NKX2-3 and NKX2-4 Deregulate Megakaryocytic-Erythroid Cell Differentiation in AML

**DOI:** 10.3390/ijms222111434

**Published:** 2021-10-22

**Authors:** Stefan Nagel, Claudia Pommerenke, Corinna Meyer, Roderick A. F. MacLeod

**Affiliations:** Department of Human and Animal Cell Lines, Leibniz-Institute DSMZ, German Collection of Microorganisms and Cell Cultures, 38124 Braunschweig, Germany; cpo14@dsmz.de (C.P.); cme@dsmz.de (C.M.); rafmacleod@gmail.com (R.A.F.M.)

**Keywords:** HOX-code, NKL-code, TALE-code, TBX-code

## Abstract

NKL homeobox genes encode transcription factors that impact normal development and hematopoietic malignancies if deregulated. Recently, we established an NKL-code that describes the physiological expression pattern of eleven NKL homeobox genes in the course of hematopoiesis, allowing evaluation of aberrantly activated NKL genes in leukemia/lymphoma. Here, we identify ectopic expression of NKL homeobox gene NKX2-4 in an erythroblastic acute myeloid leukemia (AML) cell line OCI-M2 and describe investigation of its activating factors and target genes. Comparative expression profiling data of AML cell lines revealed in OCI-M2 an aberrantly activated program for endothelial development including master factor ETV2 and the additional endothelial signature genes HEY1, IRF6, and SOX7. Corresponding siRNA-mediated knockdown experiments showed their role in activating NKX2-4 expression. Furthermore, the ETV2 locus at 19p13 was genomically amplified, possibly underlying its aberrant expression. Target gene analyses of NKX2-4 revealed activated ETV2, HEY1, and SIX5 and suppressed FLI1. Comparative expression profiling analysis of public datasets for AML patients and primary megakaryocyte–erythroid progenitor cells showed conspicuous similarities to NKX2-4 activating factors and the target genes we identified, supporting the clinical relevance of our findings and developmental disturbance by NKX2-4. Finally, identification and target gene analysis of aberrantly expressed NKX2-3 in AML patients and a megakaryoblastic AML cell line ELF-153 showed activation of FLI1, contrasting with OCI-M2. FLI1 encodes a master factor for myelopoiesis, driving megakaryocytic differentiation and suppressing erythroid differentiation, thus representing a basic developmental target of these homeo-oncogenes. Taken together, we have identified aberrantly activated NKL homeobox genes NKX2-3 and NKX2-4 in AML, deregulating genes involved in megakaryocytic and erythroid differentiation processes, and thereby contributing to the formation of specific AML subtypes.

## 1. Introduction

Stem and progenitor cells pass through several developmental stages and subsequently differentiate into mature cells and tissues. During early embryogenesis, hematopoietic and endothelial cells share a common progenitor, termed hemangioblast. Later in development, these cell types differentiate separately, starting from hematopoietic and endothelial stem cells, respectively. The process of hematopoiesis generates all types of blood and immune cells, split into the lymphoid and myeloid lineages. The latter produces mature cell types such as erythrocytes and megakaryocytes via the joint megakaryocyte and erythroid progenitor [1]. Megakaryocytes develop subsequently the thrombocytes.

Differentiation processes during hematopoietic and endothelial development are mainly regulated at the transcriptional level [2,3]. The master genes responsible for controlling these processes mostly encode transcription factors (TFs). E26 transformation-specific (ETS) and NKL homeodomain factors represent two types of such developmental TFs and operate at specific stages and lineages. ETS factors share the conserved ETS domain, which forms a winged helix-turn-helix structure and performs both sequence-specific DNA binding and protein–protein interactions [4]. According to sequence similarities, their genes are classified into 13 groups [5]. ETV2 and FLI1 are two basic ETS factors of the ERG group, regulating early steps in hematopoietic and endothelial development. *ETV2* is expressed in the hemangioblast and drives endothelial differentiation in the adult, while FLI1 is active both in hematopoietic stem cells (HSCs) and during megakaryopoiesis [6,7].

Homeobox genes encode a homeodomain that consists of three helices, generating a helix-turn-helix structure. The homeodomain performs an interaction with DNA, cofactors, and chromatin, forming a platform for gene regulation [8]. According to sequence similarities of their conserved homeobox, these genes are arranged into eleven classes and several subclasses [9]. Thus, for example, NKL homeobox genes represent a subclass of the Antennapedia (ANTP) class of homeobox genes. The human genome contains 49 NKL homeobox genes that play fundamental roles in embryonal development of tissues and organs and regulate cell differentiation in the adult. Examples are *NKX2-3*, which is expressed in developing spleen, intestine and in HSCs; *NKX2-4*, which is active in brain and testis development; and *NKX2-5*, which controls development of heart and spleen [10,11,12,13,14]. Recently, we have described the NKL-code that encompasses the gene signature of eleven NKL homeobox genes expressed in specific patterns during hematopoiesis [15]. Basic NKL-code members are *NKX2-3*, in addition to both *HHEX* and *HLX*, which orchestrate differentiation processes in myeloid and lymphoid lineages and are themselves regulated by specific hematopoietic TFs [16].

According to their functions in normal development, deregulated ETS and NKL homeobox genes promote generation of hematopoietic malignancies and are frequently targeted by chromosomal aberrations. For example, the ETS gene *ETV6* is fused with different partner genes by specific translocations in lymphoblastic and myeloid acute leukemias, and NKL homeobox gene *NKX2-5* is aberrantly activated via juxtaposition to an enhancer region of *BCL11B* in T-cell acute lymphoblastic leukemia (T-ALL) [17,18]. Other reported activating mechanisms for NKL homeobox genes are deregulated chromatin components, signaling pathways, and TFs [15]. Furthermore, aberrantly expressed ETS and NKL homeobox genes serve as diagnostic markers for certain hematopoietic cancers, such as *ETV6* in ALL and *TLX1* in T-ALL.

Acute myeloid leukemia (AML) is the most frequent acute leukemia in adults. The tumor cells derive from specific progenitor cells of the myeloid lineage. According to originating cells and stages, phenotypes, chromosomal aberrations, and gene mutations, several subtypes of AML are distinguished that differ in prognosis and treatment. The established French–American–British (FAB) system differentiates eight subtypes, called AML-M0 to -M7 [19]. Thus, for example, AML-M6 represents an erythroblastic subtype and the corresponding tumor cells express several erythropoietic genes and are blocked in development, unable to terminate differentiation. Today, additional criteria including sequencing data basically serve to classify AML [20].

Systematic analysis of NKL homeobox genes in AML has revealed 18 deregulated genes, highlighting the importance of these developmental oncogenes in driving this malignancy [21]. Focused studies in AML have investigated activating factors and downstream functions of the selected deregulated NKL homeobox genes *NANOG*, *HMX2*, and *HMX3* and have shown the oncogenic roles of these genes [21,22]. In this study, we performed detailed analyses of the oncogenically deregulated NKL homeobox gene *NKX2-4* using AML cell line OCI-M2 as model. We found that *NKX2-4* is aberrantly connected with the developmental ETS genes *ETV2* and *FLI1*, generating a leukemogenic network, which impacts myeloid differentiation.

## 2. Results

### 2.1. NKX2-4 Expression in AML Cell Line OCI-M2

**Figure 1:**
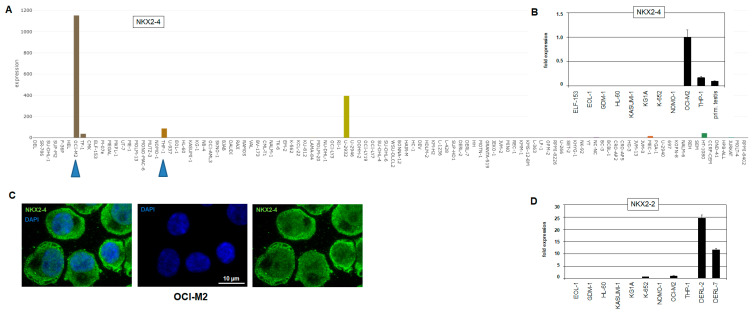
*NKX2-4* expression in hematopoietic cell lines. (**A**) LL-100 RNA-seq data show enhanced expression of NKL homeobox gene *NKX2-4* in AML cell line OCI-M2, while THP-1 expresses low levels (blue arrow heads). Gene expression values are given as DESeq2 normalized count data. (**B**) RQ-PCR analysis confirms high transcript levels in OCI-M2, while THP-1 and primary testis express lower levels. The expression level in OCI-M2 was set as 1. (**C**) Immunostaining of NKX2-4 protein in OCI-M2 cells, showing signals in both cytoplasm and nucleus (green). DAPI was used as nuclear counterstain (blue). (**D**) RQ-PCR analysis of *NKX2-2* in selected AML cell lines and T-cell lymphoma cell lines DERL-2 and DERL-7 shows elevated transcript levels in the latter, while THP-1 tested negative. The expression level in OCI-M2 was set as 1.

Systematic examinations of aberrantly expressed NKL homeobox genes in AML patients and cell lines were performed using public gene expression profiling datasets, highlighting their frequent deregulation and oncogenic impact in this myeloid malignancy [21]. To investigate their pathological function in myeloid in vitro models, we searched for deregulated NKL homeobox genes in our published RNA-seq dataset LL-100, which covers 34 myeloid and 66 lymphoid leukemia/lymphoma cell lines. We detected *NKX2-4* expression in two AML cell lines, conspicuously high in OCI-M2 and low in THP-1 (Figure 1A). Additionally, B-cell lymphoma cell line U-2932 expressed elevated *NKX2-4* levels as well. Importantly, this NKL homeobox gene is not represented in standard expression profiling arrays, and its transcriptional deregulation has, therefore, yet to be reported in leukemia patients or cell lines.

Analyses of additional public RNA-seq datasets containing samples from normal cells and tissues showed an absence of *NKX2-4* expression in developing and mature hematopoietic cells but showed its presence in the hypothalamus, pituitary gland, and testis (Figure S1). RQ-PCR and immunostaining confirmed *NKX2-4* activity in OCI-M2 cells at the RNA and protein levels, respectively (Figure 1B,C). The subcellular distribution of NKX2-4 protein showed localization in both nucleus and cytoplasm, suggesting functional regulation of this TF via nuclear import. Furthermore, combined analysis of *NKX2-4* in a primary testis sample indicated enhanced transcript levels in OCI-M2 (Figure 1B). Thus, *NKX2-4* is ectopically overexpressed in AML cell line OCI-M2, which was therefore used as a model to investigate its oncogenic role in this malignancy, including activating mechanisms and target genes.

### 2.2. Karyotyping and Genomic Profiling of OCI-M2

NKL homeobox genes are frequently deregulated by chromosomal aberrations [15]. To check if the *NKX2-4* locus is targeted by chromosomal rearrangements, we performed karyotyping of OCI-M2. The resultant karyotype was as follows: 51(46–51) < 2n > XX, der(X)t(X;8)(q23;q23), +6, +8, add(9)(p23), del(9)(p12p21), t(10;12)(p12;p12), del(17)(q11q21.1), +20, −21, +3 mar. However, no aberrations of the NKX2-4 locus at 20p11 were detected in OCI-M2. Nevertheless, the observed trisomy of chromosome 20 may boost *NKX2-4* expression by copy number gain.

**Figure 2:**
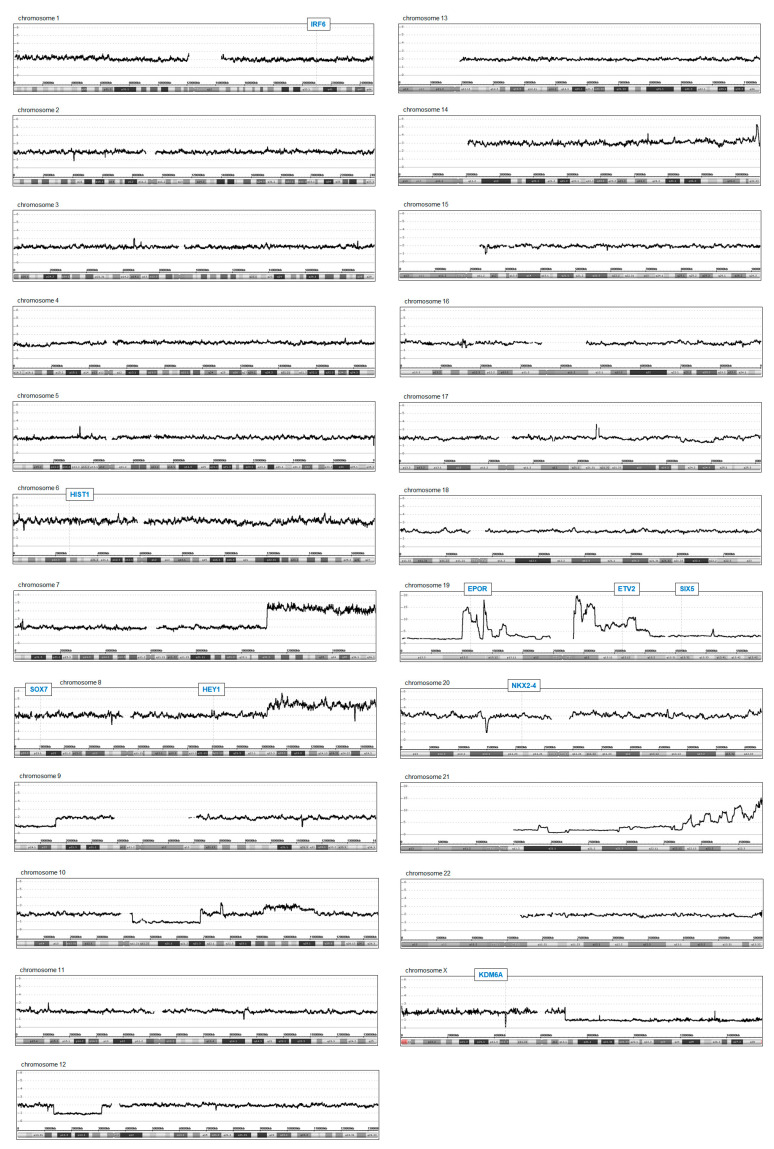
Genomic profiling data of OCI-M2. The data show copy number states for all chromosomes. The y-axis indicates the copy number state, the x-axis the chromosomal position. Selected gene loci are indicated, including *IRF6*, *HIST1*, *SOX7*, *HEY1*, *EPOR*, *ETV2*, *SIX5*, *NKX2-4*, and *KDM6A*.

Furthermore, we performed genomic profiling analysis of OCI-M2 to identify potential copy number alterations. The data for all chromosomes are shown in Figure 2, indicating, however, absence of a focal gain at the *NKX2-4* locus. In contrast, the results show several copy number alterations at other chromosomal positions, including duplications at 7q31-q36 and 8q22-q24, strongly amplified regions at 19p13 and 21q22, and deletions at 9p23-p24, 12p12, Xp11, and Xq12-q22. These regions may be indirectly implemented in *NKX2-4* deregulation. Interestingly, genomic profiling data of THP-1 cells that weakly transcribed *NKX2-4* showed a focal amplification at the *NKX2-4* locus (Figure S2A). However, this aberration neither enhanced the expression level of *NKX2-4* nor of the expression of its gene neighbor *NKX2-2*, as demonstrated by RQ-PCR analysis (Figure 1B,D), indicating absence of cognate activating TFs in THP-1. Of note, T-cell lymphoma cell lines DERL-2 and DERL-7 have been shown to aberrantly express *NKX2-2* and were used here as controls [23].

### 2.3. OCI-M2 Displays an Aberrant Program of Endothelial Development

To identify *NKX2-4* activating factors, we performed comparative expression profiling analysis of AML cell line OCI-M2 versus 31 AML control cell lines using public dataset GSE59808 and the associated online tool GEOR, which calculates the 250 most highly statistically significant differences in gene expression levels. This examination revealed for OCI-M2 differentially expressed up- or downregulated genes (Table S1). Subsequent gene set annotation analysis of these genes using the public online platform DAVID showed several significantly associated GO terms, including activated chromatin/histones and endothelial signaling (Table S2). The latter GO term was of special interest because of the close relationships between endothelial and hematopoietic development.

**Figure 3:**
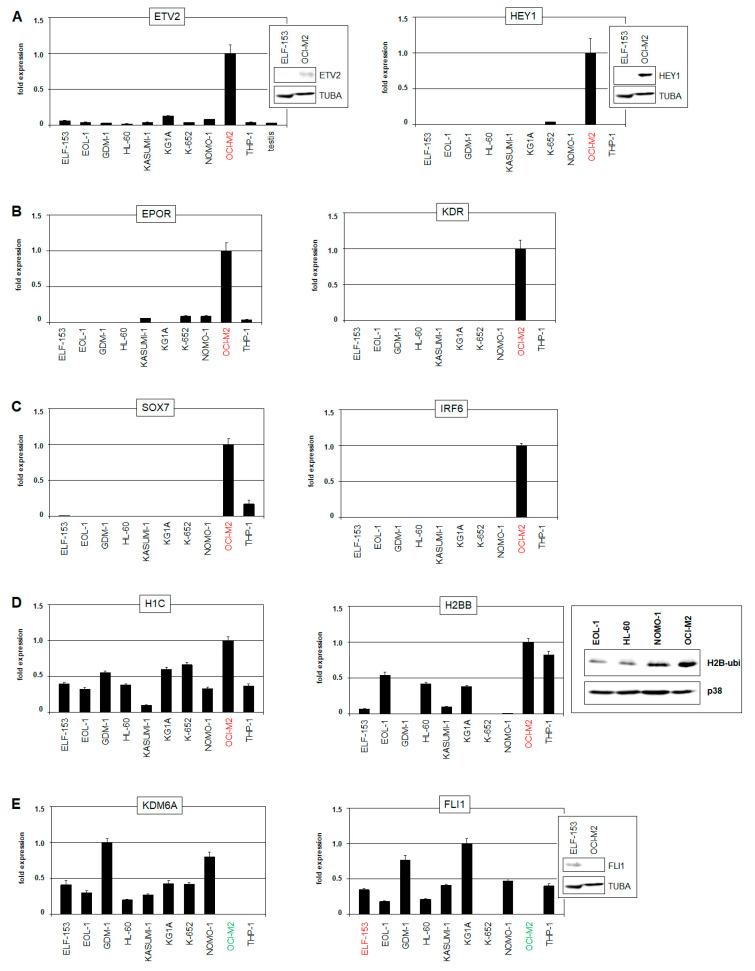
RQ-PCR and Western blot analysis of selected genes in AML cell lines. (**A**) RQ-PCR and Western blot analyses of ETV2 (**left**) and HEY1 (**right**) demonstrate elevated expression levels in OCI-M2. (**B**) RQ-PCR analyses of EPOR (**left**) and KDR (**right**) demonstrate increased expression levels in OCI-M2. (**C**) RQ-PCR analyses of SOX7 (**left**) and IRF6 (**right**) demonstrate elevated expression levels in OCI-M2. (**D**) RQ-PCR analyses of H1C (**left**) and H2BB (**middle**) demonstrate high expression levels in OCI-M2. Raised levels of ubiquinated H2B are shown by Western blot analysis, using p38 as loading control (**right**). (**E**) RQ-PCR and Western blot analyses of KDM6A (**left**) and FLI1 (**right**) demonstrate reduced expression levels in OCI-M2. The cell lines OCI-M2 and ELF-153 are indicated in red and green. The expression levels of OCI-M2 were set as 1, except for analyses of KDM6A (GDM-1 was set as 1) and FLI1 (KG1A was set as 1).

In support of this finding, OCI-M2 overexpressed *ETV2*, encoding a master factor for endothelial development, in addition to members of its associated gene signature, including *EPOR*, *HEY1*, *KDR*, and *SOX7* [24,25,26] (Table S1). RNA-seq data, RQ-PCR, and Western blot analyses of selected AML cell lines confirmed enhanced expression of these genes in OCI-M2 (Figure 3A–C and Figure S3). Furthermore, according to the comparative expression profiling data, *IRF6* and the histone genes *H1C* and *H2BB* from the HIST1-cluster were also overexpressed, while *FLI1* and *KDM6A* were downregulated in OCI-M2 (Figure 3D,E and Figure S3, Table S1). Of note, *IRF6* has been associated with endothelial development as well [27], thus representing an additional overexpressed TF in this context.

Interestingly, overexpressed *ETV2* is located at chromosomal position 19p13, which is targeted by genomic amplification in OCI-M2, while suppressed *KDM6A* is located at Xp11, which is focally deleted (Figure 2 and Figure S2B). Thus, these genomic aberrations may directly cause the deregulated activities observed for the indicated genes. Whole chromosome gains may underlie elevated expression levels of HIST1-genes at 6p22, *SOX7* at 8p23, *HEY1* at 8q21, and *NKX2-4* at 20p11 (Figure 2). However, a potential role for overexpressed histone genes in aberrant *NKX2-4* expression remained unclear, although we additionally detected elevated levels of ubiquitinated H2B in OCI-M2 (Figure 3D), which has been shown to impact NKL homeobox gene activity in B-cell lymphoma [28]. Taken together, AML cell line OCI-M2 displays an aberrantly activated program for endothelial development, which may drive its oncogenic transformation, notably including *NKX2-4* expression. Copy number alterations may underlie aberrant activation of particular endothelial signature genes.

### 2.4. Endothelial Transcription Factors Activate NKX2-4 in OCI-M2

**Figure 4:**
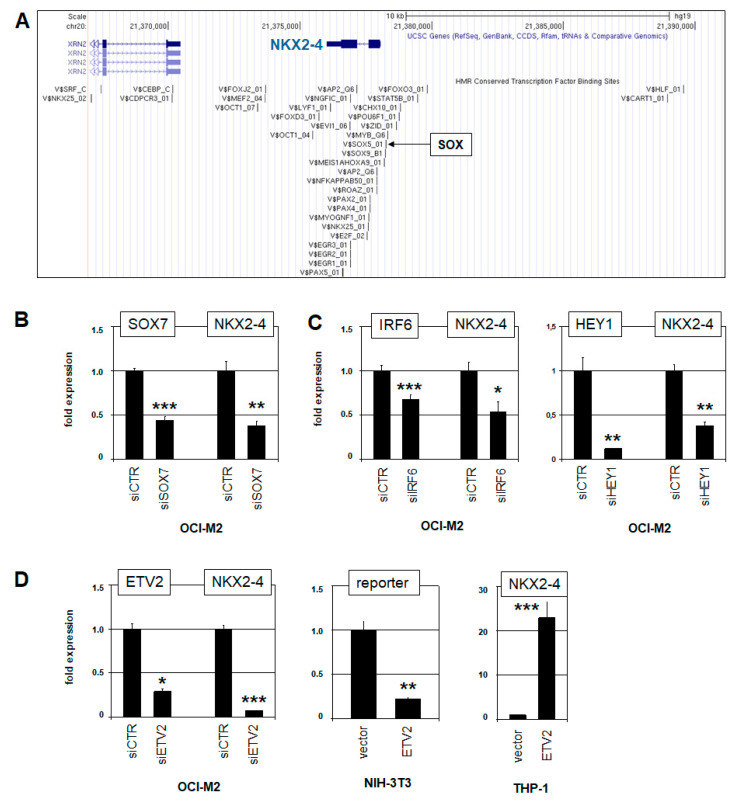
Endothelial TFs activate *NKX2-4* in OCI-M2. (**A**) TF binding sites at the *NKX2-4* locus were obtained from the UCSC genome browser. A consensus binding site for SOX factors is indicated. (**B**) RQ-PCR analysis of OCI-M2 after siRNA-mediated knockdown of *SOX7* shows concomitantly reduced transcript levels of *NKX2-4*. (**C**) RQ-PCR analyses of OCI-M2 after siRNA-mediated knockdown of *IRF6* (**left**) and *HEY1* (**right**) show concomitantly reduced transcript levels of *NKX2-4*. (**D**) RQ-PCR analysis of OCI-M2 after siRNA-mediated knockdown of *ETV2* shows concomitantly reduced transcript levels of *NKX2-4* (**left**). Reporter gene assay for a potential ETV2 binding site at *NKX2-4* was performed in NIH-3T3 cells, showing a suppressive effect. Nevertheless, these context-dependent results may indicate direct regulation of *NKX2-4* by ETV2 (**middle**). Forced expression of ETV2 in THP-1 cells resulted in strongly elevated *NKX2-4* expression (**right**). Statistical significance was assessed by Student’s *t*-test (two-tailed), and the calculated *p*-values are indicated by asterisks (* *p* < 0.05, ** *p* < 0.01, *** *p* < 0.001, n.s. not significant). The expression levels of siCTR-treated and vector-treated cells were set as 1.

To examine whether the overexpressed endothelial TFs contribute to *NKX2-4* expression, we screened its promoter region for potential TF binding sites, using the UCSC genome browser. This approach revealed a SOX consensus site, as shown in Figure 4A. Accordingly, siRNA-mediated knockdown of overexpressed *SOX7* in OCI-M2 resulted in concomitantly reduced *NKX2-4* expression, showing that this endothelial TF activated NKL homeobox gene *NKX2-4* (Figure 4B). Then, a search for additional potential binding sites at *NKX2-4* using the CIS-BP database indicated an IRF6-site at −2648 bp, an ETV2-site at −2021 bp, and a HEY1-site within exon 2. To analyze their regulatory impact on *NKX2-4* expression, we performed corresponding siRNA-mediated knockdown experiments. The results showed that all three factors—IRF6, ETV2 and HEY1—activated expression of *NKX2-4* in OCI-M2 (Figure 4C,D).

To study the role of ETV2 in more detail, we established a reporter gene assay for the identified binding site, showing that ETV2 regulated *NKX2-4* directly (Figure 4D). Furthermore, forced expression of ETV2 in THP-1 cells resulted in strongly elevated transcript levels of *NKX2-4* (Figure 4D), highlighting the activatory power of this TF in AML cells. Taken together, the endothelial TFs SOX7, IRF6, HEY1, and ETV2 are aberrantly overexpressed activators of *NKX2-4* in AML cell line OCI-M2.

### 2.5. NKX2-4 Impacts Erythroid Development

**Figure 5:**
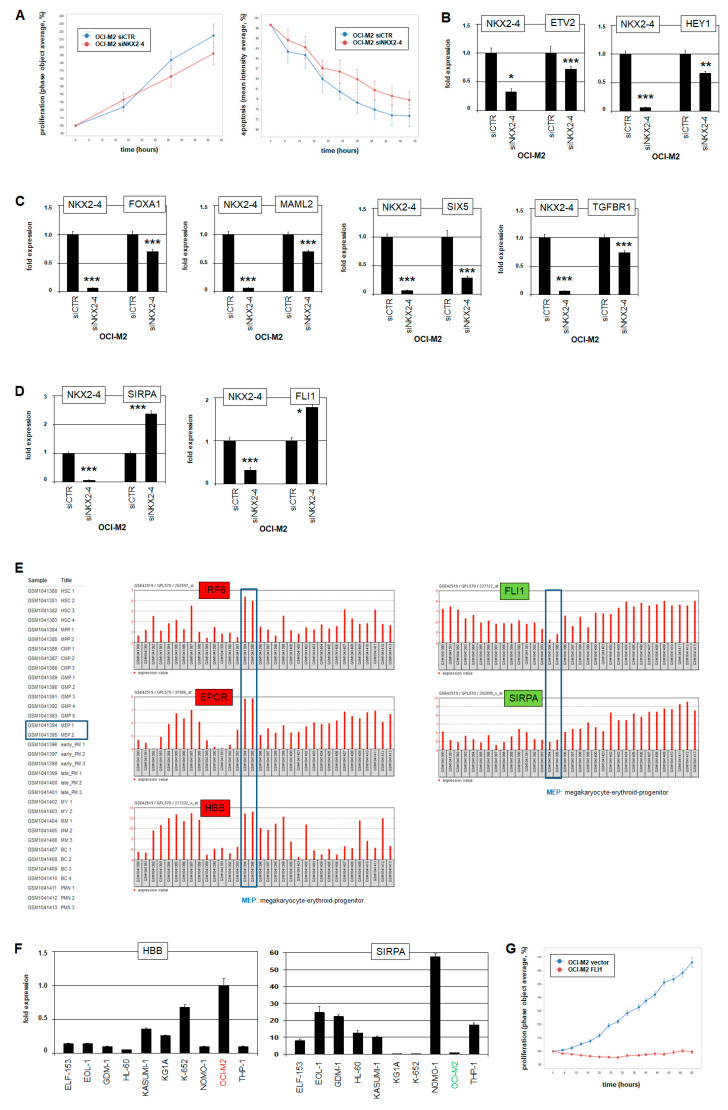
NKX2-4 target gene analyses. (**A**) Life-cell imaging analysis of OCI-M2 cells treated by siRNA-mediated knockdown of *NKX2-4* show no significant impact in proliferation (**left**) or apoptosis (**right**). (**B**) RQ-PCR analyses of *ETV2* (**left**) and *HEY1* (**right**) in OCI-M2 after siRNA-mediated knockdown of *NKX2-4* confirm these genes as activated targets. (**C**) RQ-PCR analyses of *FOXA1*, *MAML2*, *SIX5*, and *TGFBR1* in OCI-M2 after siRNA-mediated knockdown of *NKX2-4* confirm these genes as activated targets. (**D**) RQ-PCR analyses of *SIRPA* (**left**) and *FLI1* (**right**) in OCI-M2 after siRNA-mediated knockdown of *NKX2-4* confirm these genes as suppressed targets. (**E**) Expression profiling analysis of developing myeloid cells using public dataset GSE42519 shows elevated expression levels of *IRF6*, *EPOR*, and *HBB* and reduced levels of *FLI1* and *SIRPA* in megakaryocyte–erythroid progenitor cells (MEP). The y-axis represents the expression levels. (**F**) RQ-PCR analysis of selected AML cell lines for *HBB* (**left**) and *SIRPA* (**right**) show respective elevated and reduced expression levels in OCI-M2. (**G**) Life-cell imaging analysis of OCI-M2 cells treated by forced expression of FLI1 shows significant reduction in proliferation. Statistical significance was assessed by Student’s *t*-test (two-tailed), and the calculated *p*-values are indicated by asterisks (* *p* < 0.05, ** *p* < 0.01, *** *p* < 0.001, n.s. not significant). The expression levels of siCTR-treated cells and of untreated OCI-M2 were set as 1.

Functional analyses of NKX2-4 were performed by life-cell imaging. Accordingly, OCI-M2 cells were treated for *NKX2-4* knockdown and subsequently quantified for proliferation and apoptosis (Figure 5A). However, the results show no significant impact on these processes. The better to understand the potential oncogenic role of TF NKX2-4 in AML, we searched for its target genes. We postulated that the above-identified *NKX2-4* regulators ETV2 and HEY1 may simultaneously represent NKX2-4 target genes because they contain consensus binding sites for NKX2-4 at −1235 bp and −961 bp, respectively. Moreover, indeed, siRNA-mediated knockdown of *NKX2-4* resulted in concurrent downregulation of *ETV2* and *HEY1*, confirming targeting of these genes by NKX2-4 and thus their participation in an aberrant mutually activating network (Figure 5B). The known role of *ETV2* and *HEY1* in endothelial development may indicate that *NKX2-4* deregulates differentiation processes in AML.

To search for NKX2-4 target genes more systematically, we performed expression profiling analysis of OCI-M2 after *NKX2-4* knockdown in comparison with a control (Table S3). Gene set annotation analysis of the top-1000 upregulated and downregulated genes revealed several GO terms associated with tissue and organ development (Table S4). Thus, these results further support that NKX2-4 may deregulate developmental processes in AML. However, this approach showed no GO term specifically associated with myeloid differentiation. Therefore, after inspection of these differentially expressed genes, we selected six myeloid-associated candidates encoding TFs, receptors, and markers for detailed analyses—namely *FLI1*, *FOXA1*, *MAML2*, *SIX5*, *SIRPA*, and *TGFBR1* [7,29,30,31,32,33]. Again, siRNA-mediated knockdown of *NKX2-4* in OCI-M2 and subsequent transcript quantification by RQ-PCR confirmed that NKX2-4 activated *FOXA1*, *MAML2*, and *SIX5* (Figure 5C) and repressed *FLI1* and *SIRPA* (Figure 5D). The identified NKX2-4 target genes may thus play a role in deregulated myeloid development and thus in the leukemogenesis of OCI-M2.

To evaluate our findings, we analyzed gene expression data obtained from primary samples, including peripheral blood cells from AML patients as well as hematopoietic progenitor cells from healthy donors. OCI-M2 is derived from a patient with acute erythroblastic leukemia, AML-M6 [34]. To compare our cell line data with those from patients, we performed comparative expression profiling analysis of samples from six AML-M6 patients versus 429 controls (patients with AML-M0, -M1, -M2, -M3, -M4, and -M5) using public dataset GSE6891 and analysis tool GEOR. Differentially expressed genes revealed by this approach included significantly upregulated *EPOR*, *GATA1*, *HIST1*, *IRF6*, and *SIX5*, and significantly downregulated *FLI1* in AML-M6 (Table S5). Thus, key regulators and target genes of NKX2-4 identified in OCI-M2 are likewise expressed in AML-M6 patients, verifying the clinical relevance of our data obtained from a cell line model.

In addition, we compared expression profiling data of samples from normal megakaryocyte and erythroid progenitors (MEPs) versus more differentiated granulopoietic progenitors, including promyelocytes, myelocytes, metamyelocytes, and band cells, using dataset GSE42519 and analysis tool GEOR. This approach revealed downregulated *FLI1* and *SIRPA* and upregulated *IRF6*, *EPOR*, *GATA1*, and *HBB* in MEPs (Table S6, Figure 5E). FLI1, SIRPA, GATA1 and HBB play basic roles in myeloid development especially in erythropoiesis [7,32,35]. Accordingly, RQ-PCR analysis demonstrated that OCI-M2 expressed elevated *GATA1* and *HBB* and reduced *SIRPA* levels as well (Figure 5F), consistent with its erythroblastic phenotype resembling MEPs.

*FLI1* encodes a key myeloid TF, repressing erythroid while activating megakaryocytic differentiation [7,36]. Therefore, aberrant suppression of *FLI1* by *NKX2-4* in OCI-M2 may promote leukemogenic transformation towards erythroblastic cells. Accordingly, forced expression of FLI1 in OCI-M2 cells inhibited proliferation, suggesting that FLI1-mediated cell differentiation processes are uniformly connected with termination of cell growth (Figure 5G). Taken together, our experimental data for *NKX2-4* in acute erythroblastic leukemia cell line OCI-M2 correspond to expression data from AML-M6 patients and primary MEPs, suggesting that this aberrantly activated NKL homeobox gene may provoke a developmental defect in the process of megakaryocyte–erythroid differentiation.

### 2.6. NKX2-3 Impacts Megakaryocytic Development

**Figure 6:**
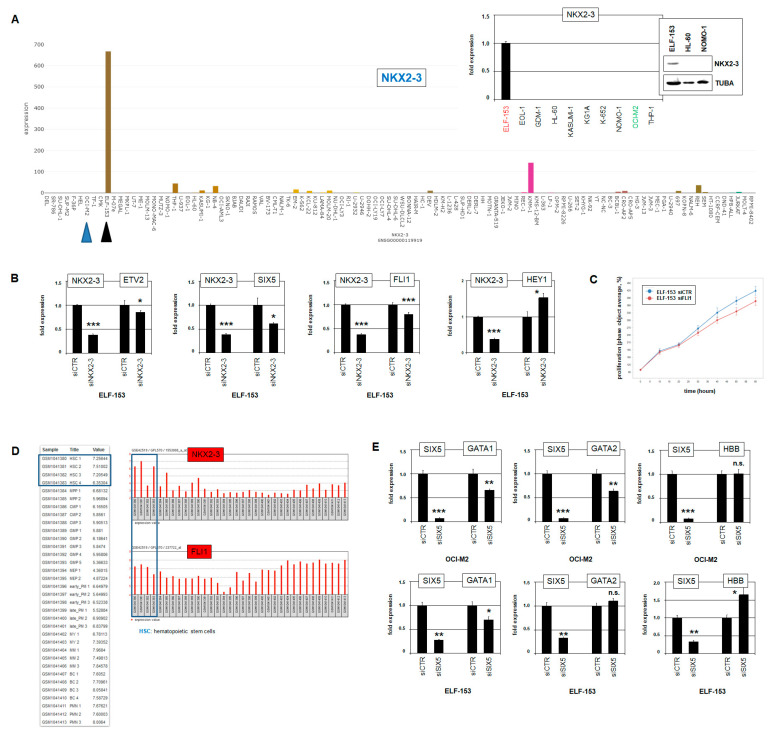
Expression and target gene analysis of NKX2-3. (**A**) LL-100 RNA-seq data show enhanced expression of NKL homeobox gene NKX2-3 in AML cell line ELF-153 (black arrowhead), while this gene is silent in OCI-M2 (blue arrow heads). Gene expression values are given as DESeq2 normalized count data. RQ-PCR and Western blot analyses of selected AML cell lines confirm enhanced expression of NKX2-3 in ELF-153 (insert). The expression level of ELF-153 was set as 1. (**B**) RQ-PCR analyses of ETV2 (**above left**), SIX5 (**above right**), FLI1 (**below left**), and of HEY1 (**below right**) in ELF-153 after siRNA-mediated knockdown of NKX2-3 confirm these genes as targets. Of note, FLI1 is activated by NKX2-3. (**C**) Life-cell imaging analysis of ELF-153 cells treated for knockdown of FLI1 shows reduction in proliferation (*p* = 0.021). (**D**) Expression profiling analysis of developing myeloid cells using public dataset GSE42519 shows elevated expression levels of NKX2-3 and FLI1 in hematopoietic stem cells (HSC). (**E**) RQ-PCR analyses of GATA1, GATA2, and HBB in OCI-M2 (**above**) and ELF-153 (**below**) after siRNA-mediated knockdown of SIX5 show differences in gene regulation between these AML cell lines. Statistical significance was assessed by Student’s *t*-test (two-tailed), and the calculated *p*-values are indicated by asterisks (* *p* < 0.05, ** *p* < 0.01, *** *p* < 0.001, n.s. not significant). The expression levels of siCTR-treated cells were set as 1.

As mentioned above, *NKX2-4* is not represented in standard expression profiling datasets. However, our comparative profiling results of AML patients (M6 versus M0 to M5) showed ectopic expression of NKL homeobox gene *NKX2-3* in AML-M6 patients (Table S5). This finding indicated that NKX2-3 and the closely related TF NKX2-4 may control similar oncogenic processes in this particular AML subtype. To compare their regulatory impacts in vitro, we searched for an *NKX2-3* expressing AML cell line model. Anew screening of RNA-seq dataset LL-100 revealed cell line ELF-153 which, however, derives from acute megakaryoblastic leukemia, AML-M7 [37]. RQ-PCR and Western blot analyses confirmed *NKX2-3* expression in ELF-153 at the RNA and protein level, respectively, validating its suitability for functional tests (Figure 6A).

Subsequent siRNA-mediated knockdown experiments in ELF-153 demonstrated that NKX2-3 activated *ETV2*, *SIX5*, and *FLI1* and suppressed *HEY1* (Figure 6B). Thus, NKX2-3 differs from NKX2-4 in regulation of *HEY1* and *FLI1*, while both NKL-TFs activated *ETV2* and *SIX5*. Accordingly, RQ-PCR and Western blot analysis demonstrated that ELF-153 expressed elevated levels of *FLI1*, and OCI-M2 reduced levels of *FLI1* (Figure 3E). Furthermore, siRNA-mediated knockdown of *FLI1* in ELF-153 resulted in slightly decreased proliferation, as analyzed by life-cell imaging (Figure 6C). Thus, proliferation was promoted by FLI1 in ELF-153, contrasting with OCI-M2. *NKX2-4* is aberrantly expressed in erythroblastic leukemia cell line OCI-M2 and represses *FLI1*, while *NKX2-3* is aberrantly expressed in megakaryoblastic leukemia cell line ELF-153 and activates *FLI1*. *FLI1* contains a consensus binding site for both NKX2-3 and NKX2-4 in its upstream region at −2564 bp, suggesting direct regulation. Moreover, both *NKX2-3* and *FLI1* are physiologically upregulated in HSCs (Figure 6D), indicating aberrant reactivation of their regulatory connection in ELF-153.

GATA1 and GATA2 represent additional regulators in megakaryopoiesis and erythropoiesis [35,38]. Furthermore, GATA1 has been shown to interact with the coactivating TFs SIX1 and SIX2 in erythropoiesis, and *GATA2* is regulated by SIX1 in embryonal development of the placodes [39,40]. Hence, we speculated whether the NKX2-3 and NKX2-4 activated target SIX5 regulates or cooperates with GATA1 and/or GATA2. Thus, we performed *SIX5* knockdown experiments in OCI-M2 and ELF-153 (Figure 6E). The results show that SIX5 activated the expression of *GATA1* in both cell lines, while *GATA2* was activated by SIX5 only in OCI-M2. GATA1-target gene *HBB* was suppressed by SIX5 in ELF-513, while no impact was detectable in OCI-M2. Thus, SIX5 functionally differed in both cell lines. In OCI-M2, *SIX5* supported erythropoiesis via *GATA1* and *GATA2*, while in ELF-153, *SIX5* supported megakaryopoiesis via *GATA1* and inhibited erythropoiesis via *HBB*. Taken together, our findings highlight that the aberrantly expressed NKL homeobox genes *NKX2-3* and *NKX2-4* manipulate developmental lineage decisions in respective megakaryoblastic and erythroblastic AML via *FLI1* deregulation and *SIX5* activation.

## 3. Discussion

**Figure 7:**
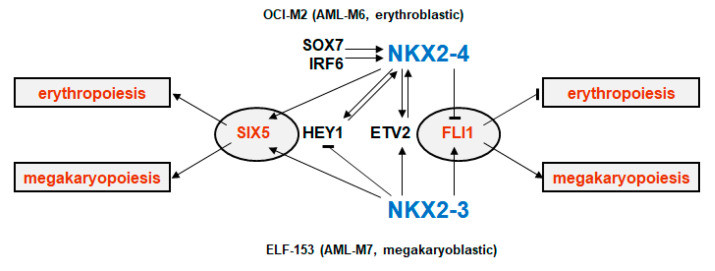
Aberrant gene regulatory network of *NKX2-4* in OCI-M2 (**above**) and *NKX2-3* in ELF-153 (**below**). These NKL homeobox genes promote respective erythroblastic and megakaryoblastic leukemogenesis via their target genes *FLI1* and *SIX5*. Endothelial activators of *NKX2-4* are indicated.

In this study, we report aberrant expression of NKL homeobox genes *NKX2-3* and *NKX2-4* in cell lines derived from two different AML subtypes. *NKX2-4* is ectopically activated in erythroblastic AML-M6 cell line OCI-M2 via the endothelial TFs ETV2, HEY1, IRF6, and SOX7, generating an aberrant developmental gene network. Prominent target genes identified are repressed *FLI1* and activated *SIX5* (Figure 7). In contrast, *NKX2-3* is aberrantly expressed in megakaryoblastic AML-M7 cell line ELF-153 by so far unknown factors and activates both *FLI1* and *SIX5* (Figure 7). In AML-M6 patients, we were also able to find gene activities of endothelial activators and target genes of NKX2-4 first identified in OCI-M2. Thus, this comparison with AML patient data revealed interesting concordances, supporting the clinical relevance of our data. Comparison with primary megakaryocyte–erythroid progenitors indicated deregulation of developmental processes by these aberrantly expressed NKL homeobox genes, operating at that developmental stage. Our data revealed deregulation of master factor FLI1. This ETS factor controls the differentiation of megakaryocytes and erythrocytes and may, therefore, represent a new key target in megakaryoblastic and erythroblastic AML.

*NKX2-3* is a member of the NKL-code and hematopoietically expressed in HSCs [11]. This restricted expression pattern may indicate a physiological role of this NKL homeobox gene in the control of stemness and lineage differentiation, while its deregulation may promote leukemogenesis by disturbing these developmental processes. Aberrant *NKX2-3* activity has been described in AML patients carrying mutations of *NPM1* or aberrations of *KMT2A* [41,42,43]. Enhanced *NKX2-3* expression correlates with *KMT2A*-rearrangements in T-ALL as well [44]. Moreover, *NKX2-3* is a direct target gene of *KMT2A-ENL* and impacts proliferation and cell differentiation [45]. In accordance with these published results, combined analysis of expression profiling data from normal hematopoietic cells and AML patient samples demonstrated physiological *NKX2-3* activity in stem cells and aberrant expression in patients with *KMT2A* rearrangements and complex karyotypes (Figure S4). In addition, *NKX2-3* expression has been detected in myelodysplastic syndrome, diffuse large B-cell lymphoma, T-ALL, and T-cell lymphoma, showing its oncogenic potential in both myeloid and lymphoid cell lineages [15]. Increased colony forming and replating capacity after NKX2-3 overexpression has been shown in murine hematopoietic progenitor cells [42], supporting an impact in early differentiation processes.

In contrast, *NKX2-4* lies without the NKL-code and is, therefore, normally silent throughout hematopoiesis. This gene is not represented on standard expression arrays and thus is less studied in cancer patients. Nevertheless, a screen of T-ALL patients revealed a chromosomal translocation that juxtaposes *NKX2-4* to the *TCRA* gene, supporting its oncogenic activity in hematopoietic malignancies [46].

Our results show that aberrantly expressed endothelial TFs ETV2, HEY1, IRF6, and SOX7 activate *NKX2-4* in AML cell line OCI-M2. The development of endothelial and hematopoietic cell types is closely linked during embryogenesis. The hemangioblast represents an embryonic stem cell that forms the basis of developing blood and endothelial cells. Although this stem cell is absent in the adult, several control genes are shared between these lineages [2]. *ETV2* is a master gene of the hemangioblast and regulates endothelial development in the adult [6]. In the embryo, ETV2 activates myelopoiesis via *RUNX1*, *SPI1*, and *TAL1* [47,48]. In the endothelial development of the heart, *ETV2* is a target gene of the heart master factor NKX2-5 [49]. This physiological connection may be aberrantly captured by the related *NKX2-4* in OCI-M2. Additional endothelial factors are encoded by *FLI1*, *HEY1*, *IRF6*, and *SOX7* [25,27,50,51]. Chromosomal amplifications and chromosomal gains underlie their aberrant activation in OCI-M2. Several of these chromosomal regions are rearranged in erythroleukemic AML patients, including 6p22, 19p13, 20p11, and Xp11, supporting the pathogenic basis of our findings [52].

*FLI1* is a master gene in myelopoiesis, driving megakaryocyte differentiation and repressing erythropoiesis [53]. Furthermore, *FLI1* is prominently expressed in HSCs, alongside *NKX2-3* [7,11]. This coexpression, together with our data showing a regulatory connection between both genes, may indicate that *FLI1* represents a target gene of NKX2-3 in HSCs as well. B-cell lymphoma cell line U-2932 expressed elevated *NKX2-4* and reduced *FLI1* (Figure 1 and Figure S3), suggesting that the repressive impact of NKX2-4 may play a role in malignant lymphoid cells as well. *FLI1* and *IRF6* play a role in both endothelial and erythroid development [53,54]. Additional genes connected with NKL homeobox genes in our study are *HBB* and *SIRPA*. *HBB* encodes beta-globin expressed in erythroid progenitors and *SIRPA* a receptor implicated in innate immunological processes [32]. *SIRPA* is highly expressed in granulocytes and monocytes, while downregulated in megakaryocytes and erythrocytes [55]. Thus, NKX2-4-mediated inhibition of *SIRPA* may shift the myeloid differentiation of granulocytes and monocytes towards megakaryocytes and erythrocytes.

SIX1 is implicated in erythropoiesis by interaction with and activation of *GATA1* [39]. *GATA2* is suppressed by SIX1 and SIX2 during erythropoiesis but activated by SIX1 in Hodgkin lymphoma [39,56]. Furthermore, *SIX1* may promote the development of MEPs [57]. Thus, several studies show a regulatory connection between SIX and GATA factors. Here, our data indicate that aberrantly activated *SIX5* impacts *GATA1* and *GATA2* in AML. Depending on the cell type, *SIX5* can promote erythropoietic or megakaryopoietic processes.

Taken together, this study shows that *FLI1* and *SIX5* represent two target genes of NKX2-3 and NKX2-4 that disturb the differentiation of megakaryocytes and erythrocytes. Overexpression experiments of *NKX2-3* and *NKX2-4* in primary progenitor cells may support this interpretation in the future. However, a developmental impact at the same stage of differentiation was described for NKL homeobox gene *DLX4* [58], highlighting the potential of these genes in lineage decisions. Moreover, we also show that deregulated *NKX2-3* and *NKX2-4* impact developmental gene activities in AML in a context dependent manner. Detection of their aberrant expression may assist the diagnosis and prognosis of certain AML subtypes.

## 4. Materials and Methods

### 4.1. Bioinformatic Analyses of RNA-Seq and Expression Profiling Data

For screening cell lines, we exploited RNA-sequencing data from 100 leukemia/lymphoma cell lines (termed LL-100), available at ArrayExpress (www.ebi.ac.uk/arrayexpress) (accessed on 18 October 2021) via E-MTAB-7721. Gene expression values are given as DESeq2 normalized count data [59]. Expression data for normal cell types were obtained from Gene Expression Omnibus (GEO, www.ncbi.nlm.nih.gov) (accessed on 18 October 2021), using RNA-seq dataset GSE69239 and expression profiling dataset GSE42519 [60,61], in addition to RNA-seq data from The Human Protein Atlas (www.proteinatlas.org) (accessed on 18 October 2021). Gene expression profiling data from AML cell lines and patients were examined using datasets GSE59808 and GSE15434, respectively [62]. Combined expression analysis of normal hematopoietic cells (GSE42519) and AML patients (GSE13159) was performed using the online database BloodSpot [63]. Gene set annotation analysis was performed using the online tool DAVID (www.david.abcc.ncifcrf.gov) (accessed on 18 October 2021) [64]. Consensus binding sites for TFs were obtained from the CIS-BP database (www.cisbp.ccbr.utoronto.ca) (accessed on 18 October 2021) and were used for screening at the UCSC genome browser (www.genome.cse.ucsc.edu) (accessed on 18 October 2021). Expression profiling data from siRNA-treated OCI-M2 cells were generated at the Genome Analytics Facility (Helmholtz Centre for Infection Research, Braunschweig, Germany) using HG U133 Plus 2.0 gene chips (Affymetrix, High Wycombe, UK). The data are available from ArrayExpress via E-MTAB-10941. After RMA background correction and quantile normalization of the spot intensities, the profiling data were expressed as ratios of sample means and subsequently log2 transformed. Data processing was performed via R/Bioconductor using public limma and affy packages.

### 4.2. Cell Lines and Treatments

Cell lines are held by the DSMZ (Braunschweig, Germany) and cultivated as described previously [65]. All cell lines had been authenticated and tested negative for mycoplasma infection. Modification of gene expression levels was performed using gene-specific siRNA oligonucleotides with reference to AllStars negative Control siRNA (siCTR) obtained from Qiagen (Hilden, Germany). The gene expression constructs for ETV2, NKX2-4, and FLI1, in addition to an empty control-vector, were obtained from Origene (Wiesbaden, Germany). SiRNAs (80 pmol) and vector DNA (2 µg) were transfected into 1 × 10^6^ cells by electroporation using the EPI-2500 impulse generator (Fischer, Heidelberg, Germany) at 350 V for 10 ms. Electroporated cells were harvested after 20 h cultivation.

Proliferation and apoptosis were analyzed using the IncuCyte S3 Live-Cell Analysis System (Essen Bioscience, Hertfordshire, UK). For detection of apoptotic cells, we used the IncuCyte Caspase-3/7 Green Apoptosis Assay diluted at 1:2000 (Essen Bioscience, Essen, Germany). Live-cell imaging experiments were performed twice with fourfold parallel tests.

### 4.3. Polymerase Chain-Reaction (PCR) Analyses

Total RNA was extracted from cultivated cell lines using TRIzol reagent (Invitrogen, Darmstadt, Germany). Primary human total RNA derived from testis was purchased from Biochain/BioCat (Heidelberg, Germany). cDNA was synthesized using 5 µg RNA, random priming, and Superscript II (Invitrogen). Real time quantitative (RQ)-PCR analysis was performed using the 7500 Real-time System and commercial buffer and primer sets (Applied Biosystems/Life Technologies, Darmstadt, Germany). For normalization of expression levels, we quantified the transcripts of TATA box binding protein (TBP). Quantitative analyses were performed as biological replicates and measured in triplicate. Standard deviations are presented in the figures as error bars. Statistical significance was assessed by Student’s *t*-test (two-tailed) and the calculated *p*-values are indicated by asterisks (* *p* < 0.05, ** *p* < 0.01, *** *p* < 0.001, n.s. not significant).

### 4.4. Protein Analysis

Western blots were generated by the semi-dry method. Protein lysates from cell lines were prepared using SIGMAFast protease inhibitor cocktail (Sigma, Taufkirchen, Germany). Proteins were transferred onto nitrocellulose membranes (Bio-Rad, Munich, Germany) and blocked with 5% dry milk powder dissolved in phosphate-buffered saline buffer (PBS). The following antibodies were used: alpha-Tubulin (Sigma, #T6199), NKX2-4 (Novus Biologicals, Centennial, CO, USA, #NBP1-91541), ETV2 (MyBioSource, San Diego, CA, USA, #MBS7112932), HEY1 (Novus Biologicals, #NBP2-56068), ubiquinated H2B (Cell Signaling, Frankfurt, Germany, #5546), p38 (Cell Signaling, #8690), FLI1 (Thermo Fisher Scientific, Darmstadt, Germany, #MA1-196), NKX2-3 (Abcam, Cambridge, UK, #ab66366). For loading, control blots were reversibly stained with Poinceau (Sigma) and detection of alpha-Tubulin (TUBA) performed thereafter. Secondary antibodies were linked to peroxidase for detection by Western-Lightning-ECL (Perkin Elmer, Waltham, MA, USA). Documentation was performed using the digital system ChemoStar Imager (INTAS, Göttingen, Germany).

Immuno-cytology was performed as follows: cells were spun onto slides and subsequently air-dried and fixed with methanol/acetic acid for 90 s. Antibodies were diluted 1:20 in PBS containing 5% BSA and incubated for 30 min. Washing was performed 3 times with PBS. Preparations were incubated with secondary antibody (diluted 1:100) for 20 min. After final washing, cells were mounted for nuclear in Vectashield (Vector Laboratories, Burlingame, CA, USA), containing DAPI. Documentation of subcellular protein localization was performed using an Axio-Imager microscope (Zeiss, Göttingen, Germany) configured to a dual Spectral Imaging system (Applied Spectral Imaging, Neckarhausen, Germany).

### 4.5. Karyotyping and Genomic Profiling Analysis

Karyotyping was performed as described previously [66]. For genomic profiling, genomic DNA of AML cell lines was prepared by the Qiagen Gentra Puregene Kit (Qiagen). Labelling, hybridization, and scanning of Cytoscan HD arrays was performed by the Genome Analytics Facility located at the Helmholtz Centre for Infection Research (Braunschweig, Germany), according to the manufacturer’s protocols (Affymetrix, High Wycombe, UK). These arrays base on single nucleotide polymorphisms (SNPs) and allow the determination of copy number states of most gene loci. Data were interpreted using the Chromosome Analysis Suite software version 3.1.0.15 (Affymetrix, High Wycombe, UK) and copy number alterations were determined accordingly.

### 4.6. Reporter-Gene Assay

For creation of reporter gene constructs, we combined a reporter with a regulatory genomic fragment derived from the upstream region of NKX2-4. PCR products of the corresponding genomic region (regulator) and of the HOXA9 gene, comprising exon1-intron1-exon2 (reporter), were cloned into respective HindIII/BamHI and EcoRI sites of expression vector pcDNA3 downstream of the CMV enhancer. Oligonucleotides used for the amplification of the regulator were obtained from Eurofins MWG, Ebersberg, Germany. Their sequences were as follows: NKX2-4-for 5′-GATGAAGCTTATAGCCTGAAAACAGAG-3′, NKX2-4-rev 5′-AACACTTGCTGGGATCCTTCTG-3′. Introduced restriction sites used for cloning are underlined. Constructs were validated by sequence analysis (Eurofins MWG). Transfections of plasmid-DNA into NIH-3T3 cells were performed using SuperFect Transfection Reagent (Qiagen). Commercial HOXA9 and TBP assays were used for RQ-PCR to quantify the spliced reporter–transcript, corresponding to the regulator activity (Thermo Fisher Scientific).

## Data Availability

Data is contained within the article or Supplementary Materials. The data presented in this study are available in [Table S3].

## Supplementary Materials

The following are available online at https://www.mdpi.com/article/10.3390/ijms222111434/s1.
