# Supplementary material for: NKL Homeobox Genes NKX2-3 and NKX2-4 Deregulate Megakaryocytic-Erythroid Cell Differentiation in AML
Source: Int J Mol Sci. 2021 Oct 22;22(21):11434. doi: 10.3390/ijms222111434 (PMC8583893; doi:10.3390/ijms222111434)
Supplement: Supplementary file 1 [file ijms-22-11434-s001.zip › ijms-1414540-supplementary.pdf]

**Figure S1: Public expression data of NKX2-4 and NKX2-3**

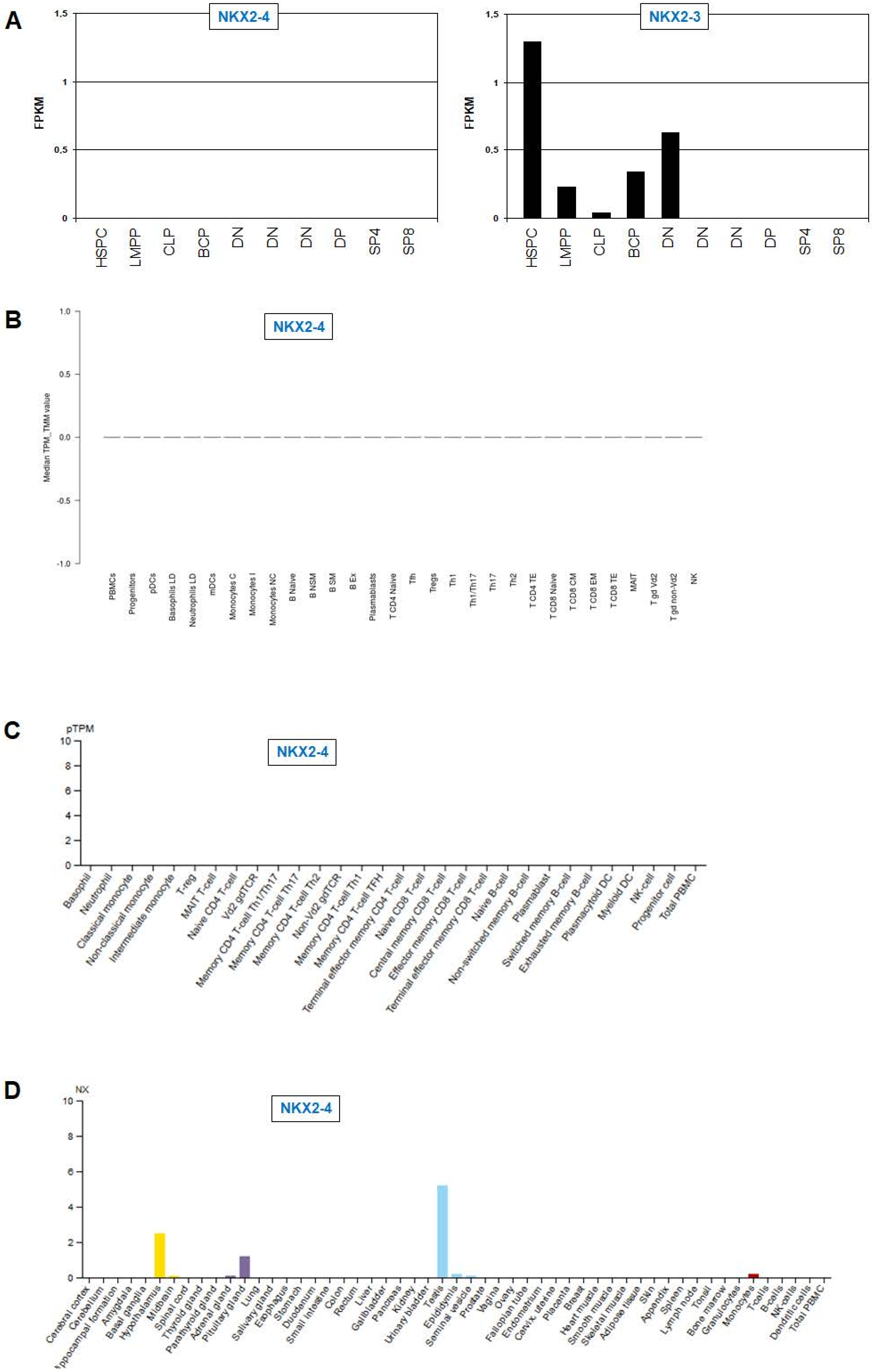

**Figure S2: Copy number alterations in OCI-M2 and THP-1**

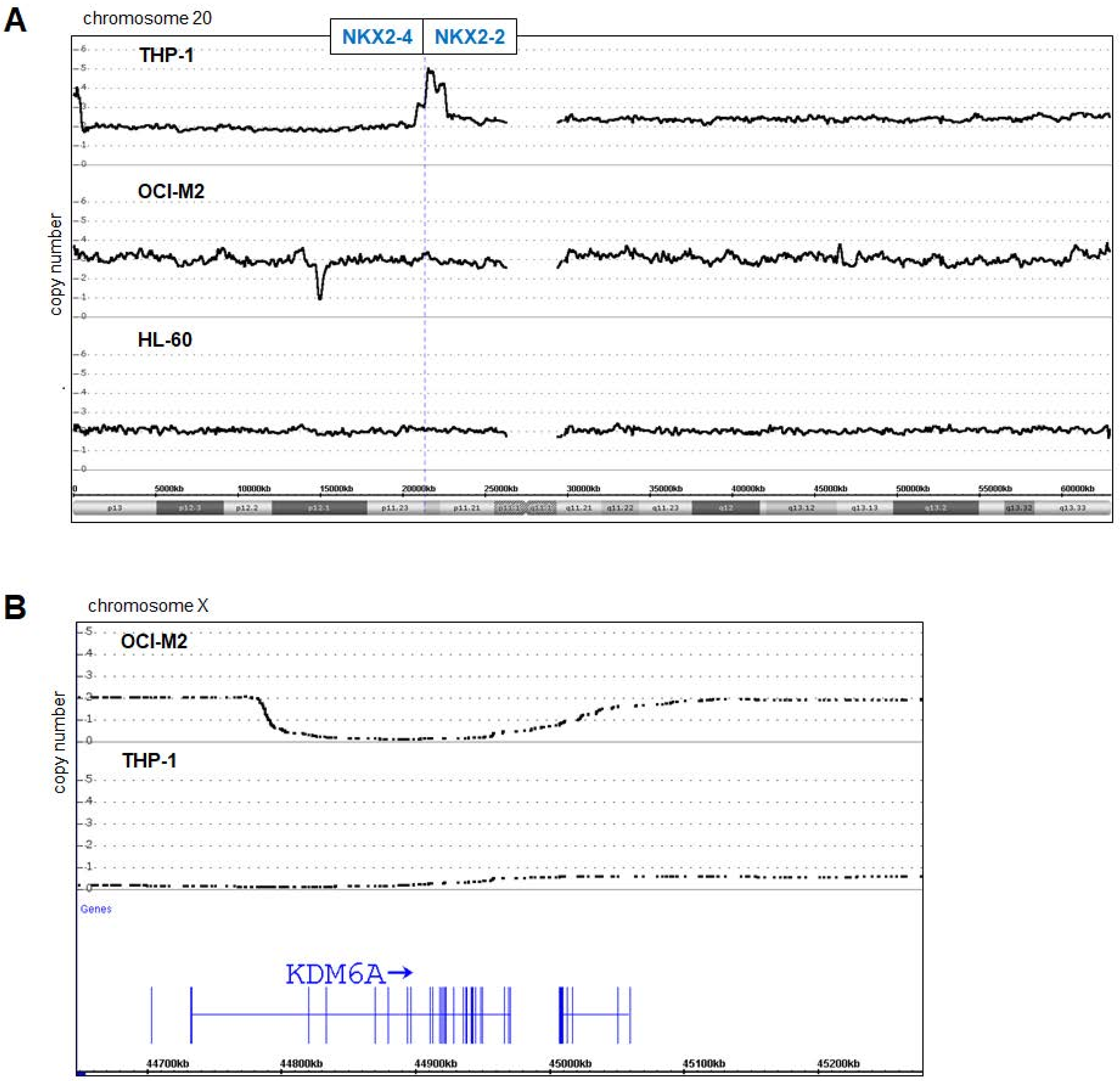

**Figure S3: Expression data from the LL-100 RNA-seq dataset**

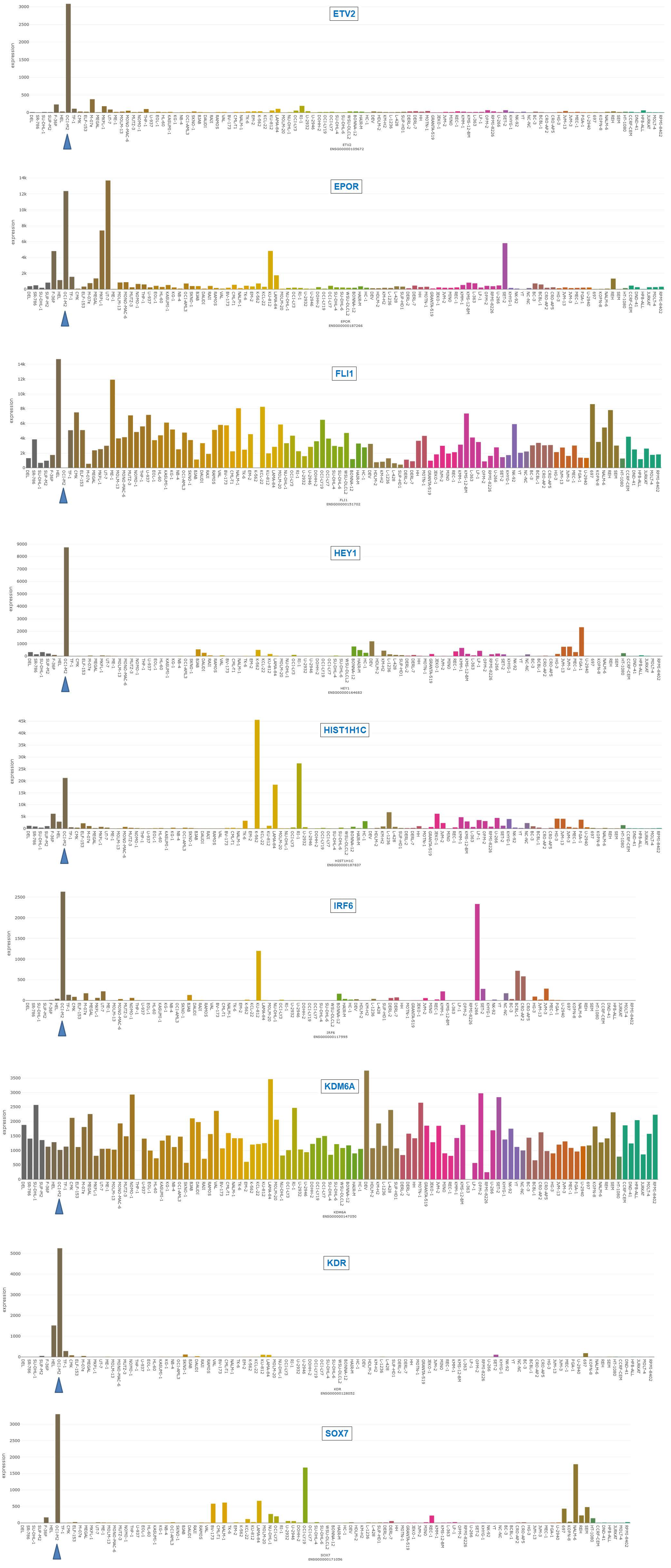

## Figure S4: NKX2-3 expression in normal hematopoietic cells and AML patients

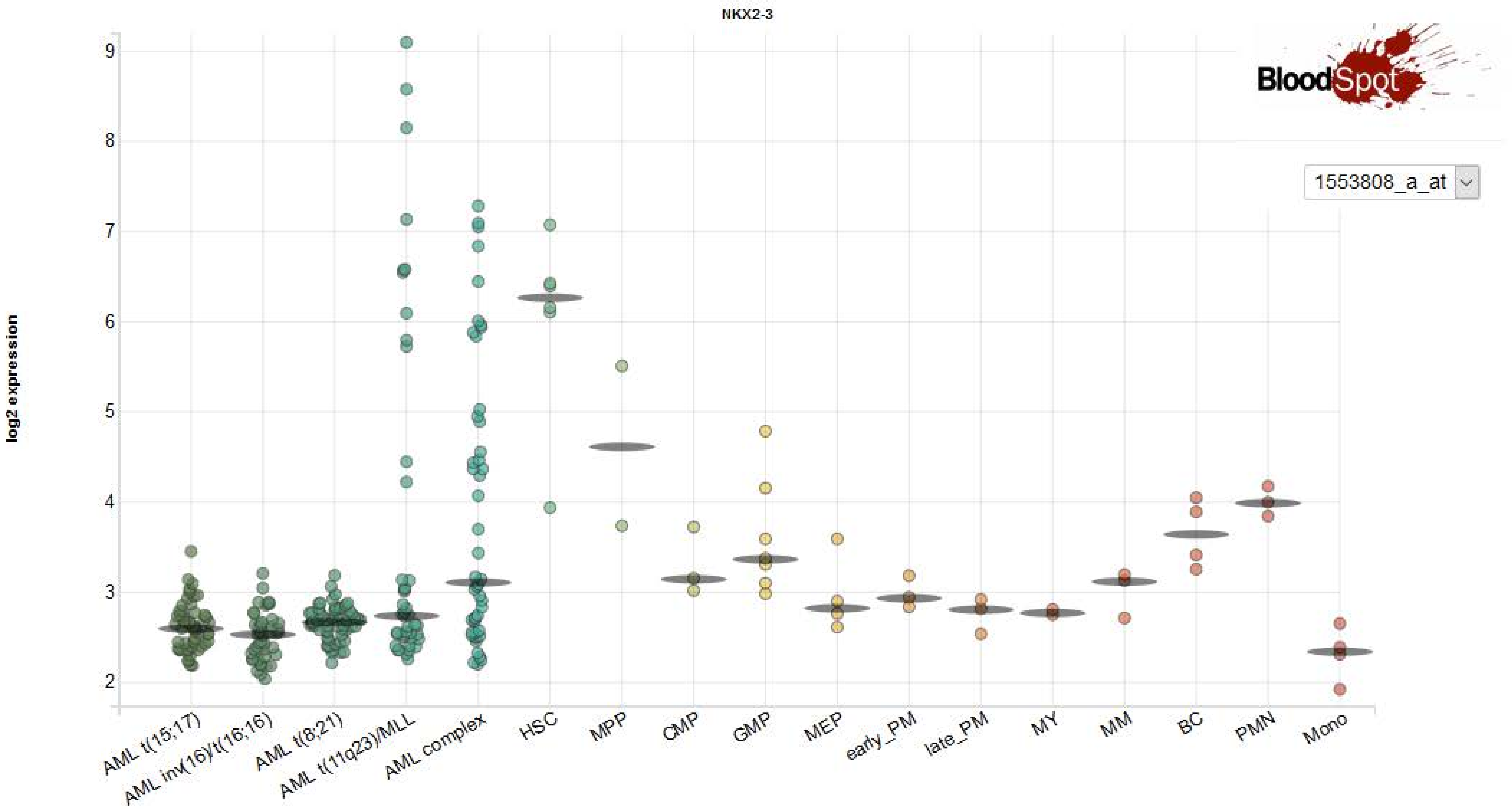


Human Normal Hematopoiesis are cells are from GSE42519
Human AML cells are from GSE13159

| Abbreviation | Name | Immunophenotype |
|---|---|---|
| AML t(15;17) | AML with t(15;17) | Whole BM unsorted |
| AML inv(16)/t(16;16) | AML with inv(16)/t(16;16) | Whole BM unsorted |
| AML t(8;21) | AML with t(8;21) | Whole BM unsorted |
| AML t(11q23)/MLL | AML with t(11q23)/MLL | Whole BM unsorted |
| AML complex | AML with complex aberrant karyotype | Whole BM unsorted |
| HSC | Hematopoietic stem cell | Lin- CD34+ CD38- CD90+ CD45RA- |
| MPP | Multipotential progenitors | Lin- CD34+ CD38- CD90- 45RA- |
| CMP | Common myeloid progenitor cell | Lin- CD34+ CD38+ CD45RA- CD123+ |
| GMP | Granulocyte monocyte progenitors | Lin- CD34+ CD38+ CD45RA+ CD123+ |
| MEP | Megakaryocyte-erythroid progenitor cell | Lin- CD34+ CD38+ CD45RA- CD123- |
| early_PM | Early Promyelocyte | Lin- FSChi SSCint CD34- CD15int CD49dhi CD33hi CD11b- CD16- |
| late_PM | Late Promyelocyte | Lin- FSChi SSChi CD34- CD15hi CD49dhi CD33hi CD11b- CD16- |
| MY | Myelocyte | Lin- FSChi SSChi CD34- CD15hi CD49dhi CD33hi CD11bhi CD16- |
| MM | Metamyelocytes | Lin- FSChi SSChi CD34- CD15hi CD49d- CD33- CD11bhi CD16- |
| BC | Band cell | Lin- FSChi SSChi CD34- CD15hi CD49d- CD33- CD11bhi CD16int |
| PMN | Polymorphonuclear cells | Lin- FSChi SSChi CD34- CD15hi CD49d- CD33- CD11bhi CD16hi |
| Mono | Monocytes | CD14+ CD16- |

**Table S1: Comparative expression profiling data from AML cell lines (GSE59808)**

| ID | adj.P.Val | P.Value | t | B | logFC | Gene.symbol |
|---|---|---|---|---|---|---|
| 203528_at | 0.00000502 | 9.31e-11 | -9.25 | 12.45459 | -6.63 | SEMA4D |
| 213025_at | 0.00000502 | 3.04e-10 | -8.81 | 11.56025 | -5.56 | THUMPD1 |
| 201859_at | 0.00000502 | 3.45e-10 | -8.76 | 11.46491 | -8.38 | SRGN |
| 208933_s_at | 0.00000502 | 3.67e-10 | -8.74 | 11.41708 | -8.83 | LGALS8 |
| 1559584_a_a | 0.00000833 | 7.62e-10 | -8.47 | 10.85751 | -11.28 | C16orf54 |
| 219676_at | 0.00000968 | 1.06e-09 | -8.35 | 10.6006 | -6.51 | ZSCAN16 |
| 210012_s_at | 0.0000275 | 3.52e-09 | -7.91 | 9.66586 | -5.13 | EWSR1 |
| 208934_s_at | 0.00003573 | 5.23e-09 | -7.77 | 9.3544 | -7.61 | LGALS8 |
| 225407_at | 0.00004062 | 6.69e-09 | -7.69 | 9.15998 | -7.25 | MBP |
| 208936_x_at | 0.00012516 | 2.29e-08 | -7.26 | 8.1789 | -5.72 | LGALS8 |
| 243023_at | 0.00037906 | 7.63e-08 | -6.84 | 7.20727 | -5.97 | |
| 1560552_a_a | 0.00047685 | 1.05e-07 | -6.73 | 6.94979 | -5.4 | |
| 209278_s_at | 0.0006454 | 1.53e-07 | 6.6 | 6.63745 | 9.23 | TFPI2 |
| 44783_s_at | 0.00072285 | 1.85e-07 | 6.54 | 6.48409 | 6.8 | **HEY1** |
| 210136_at | 0.00108754 | 2.98e-07 | -6.37 | 6.09227 | -7.5 | MBP |
| 228698_at | 0.0016133 | 4.72e-07 | 6.22 | 5.71418 | 6.32 | **SOX7** |
| 201213_at | 0.00196862 | 6.12e-07 | -6.13 | 5.49961 | -4.42 | PPP1R7 |
| 216516_at | 0.00218751 | 7.20e-07 | -6.08 | 5.36504 | -4.48 | |
| 1562934_at | 0.00275349 | 9.68e-07 | -5.98 | 5.12022 | -3.54 | LOC101927460 |
| 243537_at | 0.00275349 | 1.01e-06 | -5.96 | 5.08686 | -4.92 | |
| 201858_s_at | 0.00276613 | 1.06e-06 | -5.95 | 5.04254 | -10.44 | SRGN |
| 202664_at | 0.00379125 | 1.53e-06 | -5.82 | 4.74166 | -3.34 | WIPF1 |
| 224013_s_at | 0.00427698 | 1.80e-06 | 5.77 | 4.60417 | 7.1 | SOX7 |
| 227783_at | 0.00498109 | 2.19e-06 | -5.7 | 4.44152 | -4.05 | CCDC57 |
| 205305_at | 0.0055321 | 2.53e-06 | 5.65 | 4.31979 | 3.97 | FGL1 |
| 213596_at | 0.00580907 | 2.76e-06 | -5.62 | 4.24613 | -4.49 | CASP4 |
| 206843_at | 0.00580907 | 2.87e-06 | 5.61 | 4.21462 | 7.04 | CRYBA4 |
| 206114_at | 0.00650342 | 3.33e-06 | 5.56 | 4.08972 | 4.76 | EPHA4 |
| 228458_at | 0.00681846 | 3.62e-06 | -5.53 | 4.02073 | -5.46 | C6orf226 |
| 229389_at | 0.00710063 | 3.90e-06 | -5.51 | 3.95836 | -6.14 | ATG16L2 |
| 204236_at | 0.00711197 | 4.03e-06 | -5.5 | 3.92955 | -10.35 | **FLI1** |
| 209086_x_at | 0.01115453 | 6.60e-06 | 5.33 | 3.5156 | 3.88 | MIR6756///MCAM |
| 208180_s_at | 0.01115453 | 6.74e-06 | 5.32 | 3.49858 | 4.27 | **HIST1H4H** |
| 214472_at | 0.01115453 | 6.94e-06 | 5.31 | 3.47423 | 4.49 | **HIST1H3F///HIST1H3B///HI** |
| 1560493_a_a | 0.01151935 | 7.37e-06 | -5.29 | 3.4228 | -4.43 | CPXCR1 |
| 218839_at | 0.01232374 | 8.11e-06 | 5.26 | 3.34232 | 7.84 | HEY1 |
| 222936_s_at | 0.01273329 | 8.62e-06 | -5.24 | 3.29176 | -4.87 | DESI2 |
| 219945_at | 0.01300121 | 9.04e-06 | 5.23 | 3.25178 | 5.9 | DDX25 |
| 1555301_a_a | 0.01323626 | 9.44e-06 | 5.21 | 3.21482 | 3.47 | DIP2A |
| 223323_x_at | 0.01447326 | 1.06e-05 | -5.17 | 3.11825 | -3.78 | TRPM7 |
| 240118_at | 0.01682626 | 1.26e-05 | -5.11 | 2.97046 | -3.23 | |
| 226913_s_at | 0.02160707 | 1.67e-05 | 5.02 | 2.73401 | 7.4 | SOX8 |
| 208527_x_at | 0.02160707 | 1.70e-05 | 5.01 | 2.71925 | 5.16 | **HIST1H2BE** |
| 243563_at | 0.0220975 | 1.81e-05 | -4.99 | 2.66508 | -4.46 | |
| 230263_s_at | 0.0220975 | 1.82e-05 | -4.99 | 2.66191 | -5.72 | DOCK5 |
| 1562957_at | 0.02337664 | 1.97e-05 | -4.96 | 2.59493 | -4.5 | |
| 208546_x_at | 0.02337664 | 2.01e-05 | 4.96 | 2.57763 | 5.44 | HIST1H2BH |

| | | | | | | |
|---|---|---|---|---|---|---|
| 211826_s_at | 0.02667164 | 2.34e-05 | -4.91 | 2.44838 | -3.31 | AFF1 |
| 1560204_at | 0.02780928 | 2.49e-05 | 4.88 | 2.39562 | 3.65 | NT5DC4 |
| 208579_x_at | 0.02824386 | 2.58e-05 | 4.87 | 2.36542 | 5.36 | H2BFS |
| 1555315_a_a | 0.02859566 | 2.71e-05 | 4.86 | 2.32546 | 4.91 | MAK |
| 239501_at | 0.02859566 | 2.72e-05 | -4.86 | 2.32177 | -5.62 | |
| 208490_x_at | 0.03370593 | 3.27e-05 | 4.79 | 2.16651 | 4.48 | **HIST1H2BC///HIST1H2BI///** |
| 230206_at | 0.03473696 | 3.43e-05 | -4.78 | 2.12517 | -5.92 | DOCK5 |
| 222886_at | 0.03769634 | 3.79e-05 | -4.74 | 2.0404 | -2.73 | NSUN3 |
| 1570621_at | 0.03934896 | 4.03e-05 | -4.72 | 1.98879 | -5.29 | |
| 237484_at | 0.03976446 | 4.15e-05 | -4.71 | 1.9649 | -4.71 | SMCO3 |
| 244802_at | 0.03985974 | 4.23e-05 | 4.71 | 1.94813 | 7.39 | |
| 217247_at | 0.04278108 | 4.62e-05 | -4.68 | 1.87371 | -4.61 | |
| 237106_at | 0.04305581 | 4.72e-05 | -4.67 | 1.85404 | -3.67 | SLC11A2 |
| 209087_x_at | 0.04980736 | 5.63e-05 | 4.61 | 1.70612 | 6.66 | MCAM |
| 227790_at | 0.04980736 | 5.65e-05 | 4.61 | 1.70275 | 2.61 | UBE3D |
| 1554520_at | 0.05049638 | 5.89e-05 | -4.59 | 1.66727 | -4.34 | LOC283861 |
| 210481_s_at | 0.05049638 | 5.91e-05 | 4.59 | 1.66418 | 4.88 | CLEC4M |
| 1566720_at | 0.05296609 | 6.51e-05 | -4.56 | 1.58247 | -4.78 | RPS10P7 |
| 210716_s_at | 0.05296609 | 6.53e-05 | -4.56 | 1.58013 | -3.21 | CLIP1 |
| 243335_at | 0.05296609 | 6.56e-05 | -4.56 | 1576 | -3.79 | P4HA1 |
| 224138_at | 0.05296609 | 6.59e-05 | -4.55 | 1.57225 | -2.99 | CBX2 |
| 1552477_a_a | 0.05368575 | 6.78e-05 | 4.55 | 1.54842 | 6.65 | **IRF6** |
| 206110_at | 0.05388343 | 6.90e-05 | 4.54 | 1.53263 | 5.42 | **HIST1H3F///HIST1H3B///HI** |
| 1563656_at | 0.05388343 | 7.15e-05 | -4.53 | 1.50316 | -3.79 | |
| 205239_at | 0.05388343 | 7.18e-05 | 4.53 | 1.4986 | 5.55 | AREG |
| 209398_at | 0.05388343 | 7.19e-05 | 4.52 | 1.49749 | 5.85 | **HIST1H1C** |
| 203992_s_at | 0.05864056 | 7.94e-05 | -4.49 | 1.41415 | -6.9 | **KDM6A** |
| 209858_x_at | 0.06198538 | 8.50e-05 | -4.47 | 1.35568 | -4.24 | MPPE1 |
| 232035_at | 0.06198689 | 8.81e-05 | 4.46 | 1.32543 | 5.24 | **HIST1H4H** |
| 217486_s_at | 0.06198689 | 8.83e-05 | -4.45 | 1.32333 | -3.67 | ZDHHC17 |
| 239016_at | 0.06198689 | 8.90e-05 | -4.45 | 1.3167 | -3.57 | |
| 244559_at | 0.06198689 | 8.96e-05 | -4.45 | 1.31156 | -3.8 | |
| 206374_at | 0.06274903 | 9.18e-05 | -4.44 | 1.2905 | -3.15 | LOC101927562///DUSP8 |
| 230841_at | 0.06390267 | 9.47e-05 | -4.43 | 1.26449 | -3.14 | |
| 1560968_at | 0.06519531 | 9.78e-05 | -4.42 | 1.23707 | -2.84 | |
| 228335_at | 0.07126975 | 1.09e-04 | 4.38 | 1.14403 | 4.61 | CLDN11 |
| 228113_at | 0.07126975 | 1.09e-04 | -4.38 | 1.14097 | -6.47 | RAB37 |
| 231341_at | 0.07145992 | 1.11e-04 | 4.38 | 1.12866 | 5.54 | SLC35D3 |
| 214554_at | 0.07304539 | 1.15e-04 | 4.36 | 1.10009 | 3.89 | HIST1H2AL |
| 238220_at | 0.07426858 | 1.18e-04 | -4.35 | 1.07617 | -5.84 | KDM6A |
| 201136_at | 0.07555602 | 1.23e-04 | -4.34 | 1.0448 | -7.22 | PLP2 |
| 222067_x_at | 0.07555602 | 1.23e-04 | 4.34 | 1.04227 | 4.61 | HIST1H2BD |
| 1554544_a_a | 0.08347396 | 1.37e-04 | -4.3 | 0.94814 | -4.91 | MBP |
| 215510_at | 0.08477632 | 1.42e-04 | 4.29 | 0.92035 | 3.85 | **ETV2** |
| 1561286_a_a | 0.08477632 | 1.43e-04 | 4.29 | 0.91328 | 3.14 | DIP2A |
| 206370_at | 0.08477632 | 1.45e-04 | -4.28 | 0.90095 | -5.29 | PIK3CG |
| 203402_at | 0.08477632 | 1.46e-04 | -4.28 | 0.89805 | -6.19 | KCNAB2 |
| 203991_s_at | 0.08497979 | 1.48e-04 | -4.28 | 0.88702 | -8.44 | KDM6A |
| 1562020_s_a | 0.08579732 | 1.51e-04 | 4.27 | 0.86758 | 6.48 | NT5DC4 |
| 222692_s_at | 0.08579732 | 1.53e-04 | -4.27 | 0.85931 | -3.4 | LOC101928615///FNDC3B |

| | | | | | | |
|---|---|---|---|---|---|---|
| 236539_at | 0.08579732 | 1.54e-04 | -4.26 | 0.85248 | -3.88 | PTPN22 |
| 204953_at | 0.08741484 | 1.58e-04 | 4.25 | 0.82799 | 5.66 | SNAP91 |
| 220974_x_at | 0.08889224 | 1.63e-04 | -4.25 | 0.80521 | -5.9 | SFXN3 |
| 218824_at | 0.09152001 | 1.71e-04 | 4.23 | 0.76417 | 6.55 | PNMAL1 |
| 211340_s_at | 0.09152001 | 1.71e-04 | 4.23 | 0.76364 | 6.53 | MIR6756///MCAM |
| 214957_at | 0.09218787 | 1.74e-04 | 4.22 | 0.74917 | 7.55 | ACTL8 |
| 204401_at | 0.09323089 | 1.77e-04 | -4.22 | 0.73141 | -3.5 | KCNN4 |
| 1554485_s_a | 0.09572214 | 1.84e-04 | 4.2 | 0.70087 | 4.03 | TMEM37 |
| 242761_s_at | 0.09648945 | 1.89e-04 | 4.19 | 0.67824 | 3.01 | ZNF420 |
| 202597_at | 0.09648945 | 1.89e-04 | 4.19 | 0.67806 | 4.92 | IRF6 |
| 227253_at | 0.09802211 | 1.94e-04 | -4.18 | 0.65676 | -3.85 | CP |
| 205880_at | 0.09860679 | 1.97e-04 | 4.18 | 0.64388 | 5.83 | PRKD1 |
| 1556814_a_a | 0.0993747 | 2.00e-04 | -4.17 | 0.62953 | -4.21 | |
| 206555_s_at | 0.10054104 | 2.04e-04 | -4.17 | 0.61193 | -2.26 | THUMPD1 |
| 214455_at | 0.10205353 | 2.09e-04 | 4.16 | 0.59162 | 4.94 | HIST1H2BC///HIST1H2BI///H |
| 1569431_at | 0.103033 | 2.13e-04 | -4.15 | 0.57595 | -3.59 | PAFAH1B2 |
| 202011_at | 0.10338496 | 2.18e-04 | 4.14 | 0.55676 | 5.71 | TJP1 |
| 208071_s_at | 0.10338496 | 2.18e-04 | -4.14 | 0.5541 | -5.51 | LAIR1 |
| 232188_at | 0.10338496 | 2.20e-04 | -4.14 | 0.54935 | -3.28 | AKAP13 |
| 1562019_at | 0.10338496 | 2.21e-04 | 4.14 | 0.5435 | 8.74 | NT5DC4 |
| 223434_at | 0.10685102 | 2.31e-04 | -4.12 | 0.50825 | -6.25 | GBP3 |
| 242904_x_at | 0.10710577 | 2.33e-04 | -4.12 | 0.49906 | -3.36 | |
| 210869_s_at | 0.10835306 | 2.40e-04 | 4.11 | 0.47526 | 7.27 | MIR6756///MCAM |
| 209911_x_at | 0.10835306 | 2.42e-04 | 4.11 | 0.46743 | 5.22 | HIST1H2BD |
| 1564280_x_a | 0.10835306 | 2.44e-04 | -4.1 | 0.46113 | -3.65 | LOC100133920 |
| 205296_at | 0.10835306 | 2.44e-04 | -4.1 | 0.46113 | -2.61 | RBL1 |
| 236684_at | 0.10989777 | 2.50e-04 | -4.1 | 0.4389 | -3.46 | CNOT9 |
| 242276_at | 0.10989777 | 2.51e-04 | 4.09 | 0.4354 | 4.74 | |
| 218921_at | 0.10990877 | 2.54e-04 | -4.09 | 0.42462 | -3.1 | SIGIRR |
| 221138_s_at | 0.10990877 | 2.55e-04 | -4.09 | 0.42183 | -3.82 | |
| 227616_at | 0.11041511 | 2.60e-04 | -4.08 | 0.40665 | -4.06 | BCL9L |
| 244592_at | 0.11041511 | 2.61e-04 | -4.08 | 0.40465 | -6.25 | |
| 1559724_at | 0.11195777 | 2.70e-04 | -4.07 | 0.37521 | -4.48 | |
| 210447_at | 0.11195777 | 2.71e-04 | 4.07 | 0.37092 | 7.12 | GLUD2 |
| 228032_s_at | 0.11195777 | 2.72e-04 | -4.07 | 0.36804 | -3.79 | DENND1B |
| 210644_s_at | 0.11195777 | 2.72e-04 | -4.07 | 0.36691 | -5.43 | LAIR1 |
| 223979_x_at | 0.11196478 | 2.77e-04 | 4.06 | 0.35391 | 3.11 | FTCD |
| 1562253_at | 0.11196478 | 2.77e-04 | -4.06 | 0.35344 | -3.42 | SLC7A11-AS1 |
| 1558289_at | 0.11196478 | 2.79e-04 | -4.06 | 0.34791 | -2.88 | RFT1 |
| 221581_s_at | 0.11301617 | 2.86e-04 | -4.05 | 0.32643 | -7.67 | LAT2 |
| 207677_s_at | 0.11301617 | 2.87e-04 | -4.05 | 0.32322 | -6.22 | NCF4 |
| 204874_x_at | 0.11301617 | 2.87e-04 | 4.05 | 0.32143 | 3.78 | BAIAP3 |
| 1566285_at | 0.11376958 | 2.93e-04 | -4.04 | 0.30456 | -4.52 | |
| 201924_at | 0.11376958 | 2.93e-04 | -4.04 | 0.30365 | -3.09 | AFF1 |
| 230207_s_at | 0.11436306 | 2.97e-04 | -4.04 | 0.29282 | -4.4 | DOCK5 |
| 205226_at | 0.11436306 | 3.00e-04 | 4.03 | 0.28412 | 4.49 | PDGFRL |
| 202663_at | 0.11436306 | 3.01e-04 | -4.03 | 0.28134 | -3.2 | WIPF1 |
| 1556242_a_a | 0.11840364 | 3.14e-04 | -4.02 | 0.24596 | -2.97 | LOC100310756 |
| 223313_s_at | 0.12122392 | 3.27e-04 | 4 | 0.21173 | 7.48 | SNORA11E///SNORA11D///N |
| 220376_at | 0.12122392 | 3.27e-04 | -4 | 0.21167 | -3.73 | LRRC19 |

| | | | | | | |
|---|---|---|---|---|---|---|
| 215235_at | 0.12122392 | 3.28e-04 | -4 | 0.20857 | -4.1 | SPTAN1 |
| 200621_at | 0.12256891 | 3.34e-04 | -3.99 | 0.19347 | -3.82 | CSRP1 |
| 221631_at | 0.12572069 | 3.45e-04 | -3.98 | 0.16622 | -2.66 | CACNA1I |
| 208523_x_at | 0.12910069 | 3.57e-04 | 3.97 | 0.13804 | 4.2 | HIST1H2BC///HIST1H2BI///H |
| 240336_at | 0.12947571 | 3.62e-04 | 3.97 | 0.12599 | 6.5 | HBM |
| 243675_at | 0.12947571 | 3.62e-04 | -3.97 | 0.1244 | -3.89 | |
| 241992_at | 0.13061936 | 3.68e-04 | -3.96 | 0.11118 | -3.31 | |
| 1554929_at | 0.13061936 | 3.74e-04 | -3.95 | 0.09666 | -3.35 | SIK3 |
| 233087_at | 0.13061936 | 3.77e-04 | -3.95 | 0.09164 | -3.93 | FBXL17 |
| 224374_s_at | 0.13061936 | 3.78e-04 | -3.95 | 0.08895 | -4.48 | EMILIN2 |
| 201006_at | 0.13061936 | 3.78e-04 | -3.95 | 0.08816 | -3.07 | PRDX2 |
| 239816_at | 0.13061936 | 3.81e-04 | -3.95 | 0.08075 | -3.43 | POLD3 |
| 208585_at | 0.13061936 | 3.82e-04 | -3.95 | 0.07893 | -4.09 | BTN2A3P |
| 1552703_s_a | 0.13080305 | 3.85e-04 | -3.94 | 0.07245 | -5.77 | CARD16///CASP1 |
| 1555733_s_a | 0.13152792 | 3.90e-04 | -3.94 | 0.06249 | -5.68 | AP1S3 |
| 231367_s_at | 0.13212775 | 3.94e-04 | -3.94 | 0.0534 | -3.94 | |
| 1565706_at | 0.13272156 | 3.98e-04 | -3.93 | 0.0444 | -3.22 | |
| 226796_at | 0.13304824 | 4.02e-04 | -3.93 | 0.03715 | -6.62 | ABHD15 |
| 227196_at | 0.13387015 | 4.07e-04 | 3.93 | 0.02587 | 7.04 | RHPN2 |
| 207995_s_at | 0.13387015 | 4.11e-04 | 3.92 | 0.01747 | 3.57 | CLEC4M |
| 238725_at | 0.13387015 | 4.11e-04 | -3.92 | 0.01661 | -4.16 | IRF1 |
| 221035_s_at | 0.13461775 | 4.17e-04 | 3.92 | 0.00436 | 6.19 | TEX14 |
| 230100_x_at | 0.13461775 | 4.19e-04 | -3.92 | 0.00183 | -3.21 | PAK1 |
| 203263_s_at | 0.13649407 | 4.27e-04 | 3.91 | -0.0149 | 3.1 | ARHGEF9 |
| 212810_s_at | 0.13861196 | 4.36e-04 | -3.9 | -0.03293 | -3.86 | SLC1A4 |
| 209269_s_at | 0.14043807 | 4.44e-04 | -3.89 | -0.04897 | -3.57 | SYK |
| 232307_at | 0.14415492 | 4.59e-04 | -3.88 | -0.07605 | -4.55 | |
| 230673_at | 0.14458215 | 4.63e-04 | 3.88 | -0.08343 | 5.19 | PKHD1L1 |
| 1559884_at | 0.14676042 | 4.74e-04 | 3.87 | -0.10378 | 3.37 | CDKN2B-AS1 |
| 210634_at | 0.14676042 | 4.75e-04 | -3.87 | -0.10577 | -3.22 | KLHL20 |
| 218746_at | 0.14824608 | 4.84e-04 | -3.86 | -0.12081 | -3.47 | TAPBPL |
| 1557529_at | 0.14824608 | 4.85e-04 | -3.86 | -0.12387 | -3.12 | HECTD4 |
| 204967_at | 0.14953313 | 4.92e-04 | 3.86 | -0.13593 | 2.93 | SHROOM2 |
| 203945_at | 0.14999998 | 4.99e-04 | 3.85 | -0.14768 | 3.7 | ARG2 |
| 229137_at | 0.14999998 | 5.00e-04 | -3.85 | -0.14856 | -3.92 | FUCA1 |
| 1569136_at | 0.14999998 | 5.05e-04 | -3.85 | -0.15786 | -6.1 | MGAT4A |
| 207457_s_at | 0.14999998 | 5.07e-04 | 3.85 | -0.16113 | 6.12 | LY6G6F///LY6G6D |
| 207336_at | 0.14999998 | 5.10e-04 | 3.85 | -0.16644 | 5.76 | SOX5 |
| 233275_at | 0.14999998 | 5.17e-04 | -3.84 | -0.17674 | -3.66 | |
| 204882_at | 0.14999998 | 5.17e-04 | -3.84 | -0.17765 | -5.45 | ARHGAP25 |
| 236044_at | 0.14999998 | 5.18e-04 | 3.84 | -0.17996 | 5.55 | PLPP4 |
| 234290_x_at | 0.14999998 | 5.19e-04 | 3.84 | -0.18011 | 2.68 | MYH14 |
| 217582_at | 0.14999998 | 5.23e-04 | -3.84 | -0.18678 | -4.46 | |
| 209310_s_at | 0.14999998 | 5.24e-04 | -3.84 | -0.18894 | -3.63 | CASP4 |
| 231836_at | 0.15467185 | 5.43e-04 | 3.82 | -0.21941 | 2.63 | HKR1 |
| 210731_s_at | 0.1563338 | 5.52e-04 | -3.82 | -0.23387 | -2.68 | LGALS8 |
| 213433_at | 0.1563338 | 5.55e-04 | -3.82 | -0.23728 | -2.8 | ARL3 |
| 225156_at | 0.15679159 | 5.59e-04 | 3.81 | -0.24413 | 2.82 | ELOF1 |
| 206343_s_at | 0.15752478 | 5.65e-04 | 3.81 | -0.25243 | 4.64 | NRG1 |
| 204303_s_at | 0.15904715 | 5.73e-04 | -3.8 | -0.26491 | -4.66 | CTIF |

| | | | | | | |
|---|---|---|---|---|---|---|
| 205875_s_at | 0.15980723 | 5.80e-04 | -3.8 | -0.27454 | -5.13 | ATRIP///TREX1 |
| 242161_at | 0.15980723 | 5.83e-04 | -3.8 | -0.27901 | -4.2 | |
| 228677_s_at | 0.15980723 | 5.85e-04 | -3.8 | -0.28178 | -5.92 | RASAL3 |
| 218280_x_at | 0.16064838 | 5.91e-04 | 3.79 | -0.29047 | 4.02 | HIST2H2AA4///HIST2H2AA3 |
| 238184_at | 0.16368465 | 6.05e-04 | 3.79 | -0.31058 | 5.85 | |
| 238780_s_at | 0.16577839 | 6.22e-04 | -3.78 | -0.3346 | -3.26 | KCNJ5 |
| 221170_at | 0.16577839 | 6.23e-04 | -3.77 | -0.33527 | -5.15 | HRH4 |
| 236977_at | 0.16577839 | 6.24e-04 | -3.77 | -0.33714 | -3.46 | LOC646588 |
| 235328_at | 0.16577839 | 6.25e-04 | -3.77 | -338 | -3.43 | PLXNC1 |
| 1555113_at | 0.16908317 | 6.40e-04 | -3.76 | -0.35886 | -3.73 | PARD3B |
| 1569416_at | 0.17265134 | 6.57e-04 | -3.76 | -0.38067 | -2.94 | |
| 209243_s_at | 0.17462798 | 6.68e-04 | 3.75 | -0.39524 | 6.77 | PEG3 |
| 227919_at | 0.17462798 | 6.73e-04 | 3.75 | -0.40195 | 7.25 | UCA1 |
| 218225_at | 0.17462798 | 6.74e-04 | 3.75 | -0.40248 | 2.42 | ECSIT |
| 1554043_a_a | 0.17697541 | 6.86e-04 | 3.74 | -0.41782 | 3.3 | |
| 201131_s_at | 0.17745328 | 6.91e-04 | 3.74 | -0.4241 | 7.99 | CDH1 |
| 226765_at | 0.17817143 | 6.98e-04 | -3.73 | -0.43233 | -6.04 | SPTBN1 |
| 232663_s_at | 0.17817143 | 7.01e-04 | -3.73 | -0.43545 | -4.28 | UBAP1L |
| 241538_at | 0.18169859 | 7.20e-04 | 3.72 | -0.45885 | 4.95 | |
| 65086_at | 0.18169859 | 7.21e-04 | 3.72 | -0.45993 | 2.29 | YIPF2 |
| 242292_at | 0.18339111 | 7.31e-04 | 3.72 | -0.4717 | 4.59 | FAM226B///FAM226A |
| 1559902_at | 0.18342727 | 7.35e-04 | -3.72 | -0.47575 | -2.94 | MKL1 |
| 230903_s_at | 0.18612034 | 7.49e-04 | 3.71 | -0.49197 | 5.48 | TDRP |
| 205924_at | 0.19031559 | 7.69e-04 | 3.7 | -0.51473 | 2.78 | RAB3B |
| 1554690_a_a | 0.19269883 | 7.82e-04 | -3.69 | -0.52911 | -2.77 | TACC1 |
| 205104_at | 0.19387241 | 7.91e-04 | 3.69 | -0.53807 | 4.83 | SNPH |
| 209933_s_at | 0.19690827 | 8.09e-04 | -3.68 | -0.55786 | -6.74 | CD300A |
| 230574_at | 0.19690827 | 8.10e-04 | 3.68 | -0.55897 | 4.13 | LOC100130938 |
| 238884_at | 0.19690827 | 8.17e-04 | -3.68 | -566 | -5.76 | |
| 1560758_at | 0.19690827 | 8.23e-04 | -3.68 | -0.57186 | -4.43 | |
| 1561161_at | 0.19690827 | 8.24e-04 | -3.67 | -0.57333 | -3.26 | |
| 213122_at | 0.19690827 | 8.25e-04 | 3.67 | -0.57376 | 6.77 | TSPYL5 |
| 241917_at | 0.19712455 | 8.29e-04 | -3.67 | -0.57838 | -3.28 | |
| 1556283_s_a | 0.20024334 | 8.46e-04 | -3.67 | -0.59537 | -2.85 | FGFR1OP2 |
| 218154_at | 0.20054478 | 8.51e-04 | -3.66 | -0.6003 | -3.96 | GSDMD |
| 206060_s_at | 0.20140435 | 8.59e-04 | -3.66 | -0.60827 | -3.2 | PTPN22 |
| 227427_at | 0.20140435 | 8.62e-04 | 3.66 | -0.61121 | 4.75 | ARHGEF25 |
| 37892_at | 0.21325222 | 9.17e-04 | 3.64 | -0.66327 | 8.56 | COL11A1 |
| 236678_at | 0.21343448 | 9.21e-04 | -3.63 | -0.6676 | -3.8 | |
| 1569352_at | 0.21447708 | 9.30e-04 | -3.63 | -0.67531 | -3.65 | FNIP2 |
| 203934_at | 0.21682953 | 9.44e-04 | 3.63 | -0.68812 | 6.24 | **KDR** |
| 1563474_at | 0.21823635 | 9.54e-04 | -3.62 | -0.69716 | -2.88 | ETFBKMT |
| 202856_s_at | 0.21913078 | 9.62e-04 | -3.62 | -0.70443 | -4.74 | MIR6787///SLC16A3 |
| 208076_at | 0.21913078 | 9.66e-04 | 3.62 | -0.70768 | 2.35 | HIST1H4D |
| 1552701_a_a | 0.21921761 | 9.73e-04 | -3.61 | -0.71351 | -5.97 | CARD16 |
| 233210_at | 0.21921761 | 9.74e-04 | -3.61 | -0.71502 | -5.19 | FLJ12120 |
| 211431_s_at | 0.21993402 | 9.82e-04 | -3.61 | -0.72165 | -2.75 | TYRO3 |
| 213725_x_at | 0.21993402 | 9.86e-04 | -3.61 | -0.72473 | -6.41 | XYLT1 |
| 1555728_a_a | 0.22442221 | 1.01e-03 | 3.6 | -0.7453 | 5.42 | MS4A4A |
| 227480_at | 0.22526391 | 1.02e-03 | -3.6 | -0.75559 | -3.56 | SUSD2 |

| | | | | | | |
|---|---|---|---|---|---|---|
| 204320_at | 0.22526391 | 1.02e-03 | 3.6 | -0.75752 | 7.28 | COL11A1 |
| 1567657_at | 0.22526391 | 1.03e-03 | 3.59 | -0.76083 | 4.81 | OR2H1 |
| 205740_s_at | 0.22526391 | 1.03e-03 | 3.59 | -0.76213 | 1.99 | RBM42 |

# Table S2: Gene set annotation analysis

## A

| Sublist | Category | Term | Annotation | RT | Genes | Count | % | P-Value | Benjamini |
|---|---|---|---|---|---|---|---|---|---|
| | GOTERM_BP_DIRECT | nucleosome assembly | chromatin | RT | | 10 | 5,6 | 1,1E-6 | 9,8E-4 |
| | GOTERM_BP_DIRECT | protein autophosphorylation | | RT | | 8 | 4,5 | 8,7E-4 | 3,1E-1 |
| | GOTERM_BP_DIRECT | innate immune response in mucosa | | RT | | 4 | 2,2 | 1,4E-3 | 3,3E-1 |
| | GOTERM_BP_DIRECT | axon ensheathment | | RT | | 3 | 1,7 | 1,6E-3 | 2,9E-1 |
| | GOTERM_BP_DIRECT | defense response to Gram-positive bacterium | | RT | | 5 | 2,8 | 6,9E-3 | 7,0E-1 |
| | GOTERM_BP_DIRECT | antibacterial humoral response | | RT | | 4 | 2,2 | 6,9E-3 | 6,3E-1 |
| | GOTERM_BP_DIRECT | telomere organization | | RT | | 3 | 1,7 | 2,4E-2 | 9,5E-1 |
| | GOTERM_BP_DIRECT | immune system process | | RT | | 3 | 1,7 | 2,7E-2 | 9,5E-1 |
| | GOTERM_BP_DIRECT | positive regulation of GTPase activity | | RT | | 11 | 6,2 | 2,9E-2 | 9,4E-1 |
| | GOTERM_BP_DIRECT | DNA replication-dependent nucleosome assembly | | RT | | 3 | 1,7 | 3,3E-2 | 9,4E-1 |
| | GOTERM_BP_DIRECT | chromatin silencing at rDNA | chromatin | RT | | 3 | 1,7 | 4,3E-2 | 9,7E-1 |
| | GOTERM_BP_DIRECT | adaptive immune response | | RT | | 5 | 2,8 | 4,3E-2 | 9,6E-1 |
| | GOTERM_BP_DIRECT | positive regulation of endothelial cell chemotaxis by VEGF-activated vascular endothelial growth factor receptor signaling pathway | endothel signalling pathway | RT | | 2 | 1,1 | 4,4E-2 | 9,5E-1 |
| | GOTERM_BP_DIRECT | peptidyl-tyrosine phosphorylation | | RT | | 5 | 2,8 | 4,8E-2 | 9,5E-1 |
| | GOTERM_BP_DIRECT | angiogenesis | | RT | | 6 | 3,4 | 4,9E-2 | 9,4E-1 |
| | GOTERM_BP_DIRECT | protein phosphorylation | | RT | | 9 | 5,1 | 5,1E-2 | 9,4E-1 |
| | GOTERM_BP_DIRECT | platelet aggregation | | RT | | 3 | 1,7 | 5,1E-2 | 9,3E-1 |
| | GOTERM_BP_DIRECT | regulation of T cell receptor signaling pathway | | RT | | 2 | 1,1 | 5,2E-2 | 9,2E-1 |
| | GOTERM_BP_DIRECT | protein heterotetramerization | | RT | | 3 | 1,7 | 5,3E-2 | 9,2E-1 |
| | GOTERM_BP_DIRECT | beta-catenin-TCF complex assembly | | RT | | 3 | 1,7 | 5,6E-2 | 9,2E-1 |
| | GOTERM_BP_DIRECT | chromatin silencing | chromatin | RT | | 3 | 1,7 | 6,0E-2 | 9,2E-1 |
| | GOTERM_BP_DIRECT | negative regulation of gene expression, epigenetic | chromatin | RT | | 3 | 1,7 | 7,3E-2 | 9,5E-1 |
| | GOTERM_BP_DIRECT | positive regulation of type I interferon production | | RT | | 3 | 1,7 | 7,5E-2 | 9,5E-1 |
| | GOTERM_BP_DIRECT | gene silencing by RNA | | RT | | 4 | 2,2 | 7,6E-2 | 9,4E-1 |

## B

| Sublist | Category | Term | RT | Genes | Count | % | P-Value |
|---|---|---|---|---|---|---|---|
| | GOTERM_BP_DIRECT | positive regulation of fibroblast migration | RT | | 4 | 1,1 | 7,2E-4 |
| | GOTERM_BP_DIRECT | negative regulation of nucleic acid-templated transcription | RT | | 6 | 1,6 | 1,2E-3 |
| | GOTERM_BP_DIRECT | cellular response to histamine | RT | | 3 | 0,8 | 7,5E-3 |
| | GOTERM_BP_DIRECT | sensory perception of sound | RT | | 8 | 2,2 | 7,6E-3 |
| | GOTERM_BP_DIRECT | extracellular matrix disassembly | RT | | 6 | 1,6 | 9,4E-3 |
| | GOTERM_BP_DIRECT | forelimb morphogenesis | RT | | 3 | 0,8 | 1,2E-2 |
| | GOTERM_BP_DIRECT | cell adhesion | RT | | 16 | 4,3 | 1,2E-2 |
| | GOTERM_BP_DIRECT | cell differentiation | RT | | 16 | 4,3 | 1,3E-2 |
| | GOTERM_BP_DIRECT | regulation of cardiac conduction | RT | | 5 | 1,4 | 1,5E-2 |
| | GOTERM_BP_DIRECT | calcium ion transmembrane transport | RT | | 7 | 1,9 | 1,6E-2 |
| | GOTERM_BP_DIRECT | neurotransmitter biosynthetic process | RT | | 3 | 0,8 | 1,7E-2 |
| | GOTERM_BP_DIRECT | protein localization to organelle | RT | | 3 | 0,8 | 2,9E-2 |
| | GOTERM_BP_DIRECT | innervation | RT | | 3 | 0,8 | 3,7E-2 |
| | GOTERM_BP_DIRECT | response to corticosterone | RT | | 3 | 0,8 | 3,7E-2 |
| | GOTERM_BP_DIRECT | transmission of nerve impulse | RT | | 3 | 0,8 | 4,5E-2 |
| | GOTERM_BP_DIRECT | response to immobilization stress | RT | | 3 | 0,8 | 4,9E-2 |
| | GOTERM_BP_DIRECT | positive regulation of MAPK cascade | RT | | 5 | 1,4 | 4,9E-2 |
| | GOTERM_BP_DIRECT | negative regulation of phosphatase activity | RT | | 4 | 1,1 | 5,6E-2 |
| | GOTERM_BP_DIRECT | regulation of synaptic transmission, glutamatergic | RT | | 3 | 0,8 | 5,7E-2 |
| | GOTERM_BP_DIRECT | cochlea development | RT | | 3 | 0,8 | 6,2E-2 |
| | GOTERM_BP_DIRECT | smooth muscle cell proliferation | RT | | 2 | 0,5 | 6,6E-2 |
| | GOTERM_BP_DIRECT | sprouting angiogenesis | RT | | 3 | 0,8 | 6,7E-2 |
| | GOTERM_BP_DIRECT | protein autophosphorylation | RT | | 7 | 1,9 | 7,3E-2 |
| | GOTERM_BP_DIRECT | endodermal cell differentiation | RT | | 3 | 0,8 | 7,6E-2 |
| | GOTERM_BP_DIRECT | positive regulation of the force of heart contraction | RT | | 2 | 0,5 | 8,2E-2 |
| | GOTERM_BP_DIRECT | negative regulation of gene expression | RT | | 6 | 1,6 | 8,4E-2 |
| | GOTERM_BP_DIRECT | protein phosphorylation | RT | | 13 | 3,5 | 8,9E-2 |
| | GOTERM_BP_DIRECT | collagen-activated tyrosine kinase receptor signaling pathway | RT | | 2 | 0,5 | 9,8E-2 |
| | GOTERM_BP_DIRECT | axonal transport of mitochondrion | RT | | 2 | 0,5 | 9,8E-2 |

## Table S3: Gene expression profiling data of OCI-M2 treated for NK

| Probe_set_ID | Symbol_na33 | OCIM2siCTR | OCIM2siNKX24 | CTR-NKX24 | |
|---|---|---|---|---|---|
| 205419_at | GPR183 | 0,03125738 | -2,690860744 | 2,722118124 | |
| 242136_x_at | MGC70870 | -0,259832361 | -2,735862426 | 2,476030064 | |
| 216710_x_at | ZNF287 | 1,64218396 | -0,765504029 | 2,407687989 | |
| 220039_s_at | CDKAL1 | 0,20027154 | -1,961547147 | 2,161818687 | |
| 1569013_s_at | LOC96610 | 0,457392764 | -1,615132408 | 2,072525172 | |
| 241675_s_at | - | -0,888496123 | -2,847001814 | 1,958505692 | |
| 216395_at | FBXL18 | -0,59053611 | -2,503677622 | 1,913141512 | |
| 206873_at | CA6 | -0,299034815 | -2,211750327 | 1,912715512 | |
| 228756_at | SLC6A6 | 0,103263601 | -1,787089803 | 1,890353405 | |
| 239039_at | - | -0,285837228 | -2,162639771 | 1,876802543 | |
| 236837_x_at | MIPEPP3 | 0,083054335 | -1,787089803 | 1,870144138 | |
| 222224_at | NACA2 | -1,240169874 | -3,037500868 | 1,797330994 | |
| 1563272_at | TBC1D8B | -0,83191556 | -2,621331989 | 1,789416429 | |
| 212354_at | SULF1 | -0,299034815 | -2,086984744 | 1,787949929 | |
| 1553805_at | C3orf49 | -0,79540287 | -2,575129729 | 1,779726859 | |
| 234434_at | - | -0,85085127 | -2,621331989 | 1,770480719 | |
| 235106_at | **MAML2** | -0,655928373 | -2,408705138 | 1,752776764 | |
| 1554549_a_at | WDR20 | -0,325749314 | -2,037551727 | 1,711802412 | |
| 1567681_at | SNORA74A | -0,759588698 | -2,456406426 | 1,696817728 | |
| 206255_at | BLK | -0,467091869 | -2,162639771 | 1,695547903 | |
| 217353_at | - | 0,425644434 | -1,264005341 | 1,689649774 | |
| 235165_at | PARD6B | 1,261018186 | -0,41858459 | 1,679602776 | |
| 242511_at | - | -0,112808516 | -1,787089803 | 1,674281287 | |
| 1558017_s_at | - | -0,741668563 | -2,408705138 | 1,667036575 | |
| 1564017_at | COL18A1-AS1 | -0,496991414 | -2,162639771 | 1,665648358 | |
| 210956_at | PPYR1 | -0,67289328 | -2,335104118 | 1,662210838 | |
| 220103_s_at | LOC100509751 / | 0,152582607 | -1,494668682 | 1,647251289 | |
| 239354_at | - | -0,706943342 | -2,335104118 | 1,628160776 | |
| 214690_at | TAF1B | 0,798390451 | -0,819238336 | 1,617628787 | |
| 231885_at | ZNF451 | -0,172274514 | -1,787089803 | 1,614815289 | |
| 234713_x_at | - | -1,131062212 | -2,735862426 | 1,604800213 | |
| 1562947_x_at | - | -1,353583928 | -2,954576755 | 1,600992827 | |
| 244360_at | FBXL17 | -0,022466681 | -1,615132408 | 1,592665727 | |
| 232193_at | GSTT1 | -0,622910612 | -2,211750327 | 1,588839715 | |
| 1557007_a_at | - | -1,196375121 | -2,780313924 | 1,583938803 | |
| 230706_s_at | CAMK2N2 | 0,181384709 | -1,377419394 | 1,558804103 | |
| 210795_s_at | - | 0,401366113 | -1,154897679 | 1,556263792 | |
| 209937_at | TM4SF4 | -0,741668563 | -2,286583451 | 1,544914888 | |
| 240757_at | CLASP1 | -0,622910612 | -2,162639771 | 1,539729159 | |
| 234431_at | GSN | -0,423248115 | -1,961547147 | 1,538299032 | |
| 229295_at | IL17RA | -0,325749314 | -1,862647763 | 1,536898448 | |
| 218952_at | PCSK1N | 0,518867317 | -1,010192375 | 1,529059692 | |
| 233781_s_at | RIF1 | -0,512251684 | -2,037551727 | 1,525300042 | |
| 228721_at | KRBOX1 | -0,43775896 | -1,961547147 | 1,523788187 | |
| 217019_at | - | 0,95671774 | -0,566887459 | 1,5236052 | |
| 225124_at | PPP1R9B | 0,606430281 | -0,912331589 | 1,51876187 | |
| 1569952_x_at | - | 0,496120222 | -1,010192375 | 1,506312597 | |
| 220519_s_at | LIM2 | -0,83191556 | -2,335104118 | 1,503188558 | |
| 241637_at | - | -0,83191556 | -2,335104118 | 1,503188558 | |
| 216467_s_at | - | -0,285837228 | -1,787089803 | 1,501252575 | |
| 207798_s_at | ATXN2L | -0,366776884 | -1,862647763 | 1,495870879 | |
| 225532_at | CABLES1 | -0,172274514 | -1,663414495 | 1,491139981 | |
| 207987_s_at | GNRH1 | -1,089016378 | -2,575129729 | 1,486113351 | |

| | | | | | |
|---|---|---|---|---|---|
| 1560659_at | - | -0,926850548 | -2,408705138 | 1,481854589 | |
| 213658_at | - | -0,741668563 | -2,211750327 | 1,470081765 | |
| 227073_at | MAP3K2 | 0,822971223 | -0,646746079 | 1,469717302 | |
| 223400_s_at | PBRM1 | 1,143956113 | -0,322870281 | 1,466826394 | |
| 203939_at | NT5E | 0,695652136 | -0,765504029 | 1,461156165 | |
| 220252_x_at | CXorf21 | -0,83191556 | -2,286583451 | 1,454667891 | |
| 1561135_at | - | -0,285837228 | -1,738067356 | 1,452230128 | |
| 237823_at | - | -0,512251684 | -1,961547147 | 1,449295463 | |
| 240227_at | MIER1 | -0,59053611 | -2,037551727 | 1,447015616 | |
| 1564460_at | LOC286442 | -0,423248115 | -1,862647763 | 1,439399647 | |
| 222051_s_at | - | -0,423248115 | -1,862647763 | 1,439399647 | |
| 1569648_at | DACT2 | 0,425644434 | -1,010192375 | 1,435836809 | |
| 228782_at | SCGB3A2 | -0,85085127 | -2,286583451 | 1,435732181 | |
| 205355_at | ACADSB | 0,103263601 | -1,332017329 | 1,435280931 | |
| 205887_x_at | MSH3 | 1,406361373 | -0,024572586 | 1,43093396 | |
| 1559297_at | SNX29P2 | -1,196375121 | -2,621331989 | 1,424956868 | |
| 243550_at | ZDHHC21 | -1,196375121 | -2,621331989 | 1,424956868 | |
| 228926_s_at | SMARCA2 | -0,543051993 | -1,961547147 | 1,418495154 | |
| 237408_at | DCUN1D1 | -0,196756757 | -1,615132408 | 1,418375651 | |
| 205124_at | MEF2B /// MEF2I | -0,496991414 | -1,912089812 | 1,415098398 | |
| 1555183_at | TERF2 | -0,136301282 | -1,541908042 | 1,40560676 | |
| 1559538_at | - | -0,259832361 | -1,663414495 | 1,403582133 | |
| 203130_s_at | KIF5C | 0,181384709 | -1,220210588 | 1,401595297 | |
| 1562845_at | EP400NL | -0,512251684 | -1,912089812 | 1,399838127 | |
| 1560648_s_at | TSPYL1 | -0,888496123 | -2,286583451 | 1,398087328 | |
| 216497_at | - | 0,133056859 | -1,264005341 | 1,397062199 | |
| 206943_at | **TGFBR1** | -0,148196756 | -1,541908042 | 1,393711285 | |
| 221992_at | NPIPL2 | -0,394749123 | -1,787089803 | 1,39234068 | |
| 241345_at | MIR186 /// ZRAN | -1,308181863 | -2,690860744 | 1,382678881 | |
| 244025_at | - | -0,706943342 | -2,086984744 | 1,380041402 | |
| 225996_at | LONRF2 | -1,196375121 | -2,575129729 | 1,378754608 | |
| 225589_at | SH3RF1 | -0,285837228 | -1,663414495 | 1,377577266 | |
| 229689_s_at | DLG5 | -1,089016378 | -2,456406426 | 1,367390048 | |
| 242474_s_at | VMA21 | -1,089016378 | -2,456406426 | 1,367390048 | |
| 209888_s_at | MYL1 | -0,79540287 | -2,162639771 | 1,367236901 | |
| 214337_at | COPA | -0,496991414 | -1,862647763 | 1,365656349 | |
| 227905_s_at | AZI2 | 0,03125738 | -1,332017329 | 1,363274709 | |
| 1560138_at | KIAA0226 | -1,047650486 | -2,408705138 | 1,361054652 | |
| 226342_at | SPTBN1 | -0,85085127 | -2,211750327 | 1,360899057 | |
| 244186_at | - | -0,926850548 | -2,286583451 | 1,359732902 | |
| 204083_s_at | TPM2 | 0,503742507 | -0,855751026 | 1,359493533 | |
| 213498_at | CREB3L1 | 0,401366113 | -0,950686014 | 1,352052127 | |
| 220149_at | C2orf54 | -0,512251684 | -1,862647763 | 1,350396078 | |
| 226420_at | MECOM | -0,512251684 | -1,862647763 | 1,350396078 | |
| 1559681_a_at | TRIM16L | 0,702292136 | -0,646746079 | 1,349038215 | |
| 242472_x_at | FNBP4 | 0,291166226 | -1,050406615 | 1,341572841 | |
| 1562247_at | LOC286058 | -0,946847173 | -2,286583451 | 1,339736277 | |
| 215283_at | LOC339290 | -0,946847173 | -2,286583451 | 1,339736277 | |
| 228940_at | NDUFB4 | -0,622910612 | -1,961547147 | 1,338636535 | |
| 1552814_a_at | KLF14 | -1,2845686 | -2,621331989 | 1,336763389 | |
| 1561099_at | FLJ32756 | -1,2845686 | -2,621331989 | 1,336763389 | |
| 210675_s_at | PTPRR | -1,240169874 | -2,575129729 | 1,334959855 | |
| 215425_at | BTG3 | -1,518072576 | -2,847001814 | 1,328929239 | |
| 224875_at | C5orf24 | 0,760715727 | -0,566887459 | 1,327603186 | |
| 238830_at | - | -1,131062212 | -2,456406426 | 1,325344214 | |
| 204873_at | PEX1 | 2,171078719 | 0,847075464 | 1,324003255 | |

| | | | | | |
|---|---|---|---|---|---|
| 215868_x_at | - | -0,888496123 | -2,211750327 | 1,323254205 | |
| 1555821_a_at | AKT1S1 | 0,209623097 | -1,112851844 | 1,322474941 | |
| 218923_at | CTBS | -0,055687482 | -1,377419394 | 1,321731912 | |
| 1568894_at | - | -0,59053611 | -1,912089812 | 1,321553702 | |
| 205141_at | ANG | -0,59053611 | -1,912089812 | 1,321553702 | |
| 204667_at | **FOXA1** | -0,221656139 | -1,541908042 | 1,320251903 | |
| 1557421_at | - | 0,900083947 | -0,41858459 | 1,318668537 | |
| 1564373_a_at | LOC283887 | 1,269985355 | -0,046302147 | 1,316287501 | |
| 1562091_at | - | -1,639579028 | -2,954576755 | 1,314997727 | |
| 205579_at | HRH1 | -1,308181863 | -2,621331989 | 1,313150126 | |
| 240589_at | LINC00313 | -1,308181863 | -2,621331989 | 1,313150126 | |
| 230095_at | - | -0,022466681 | -1,332017329 | 1,309550649 | |
| 1557066_at | LUC7L | -1,470833215 | -2,780313924 | 1,309480709 | |
| 215595_x_at | - | 0,152582607 | -1,154897679 | 1,307480286 | |
| 215444_s_at | TRIM31 | -0,986356909 | -2,286583451 | 1,300226542 | |
| 215558_at | UBR2 | -0,986356909 | -2,286583451 | 1,300226542 | |
| 207887_s_at | CALCR | -0,078265071 | -1,377419394 | 1,299154323 | |
| 239768_x_at | GPATCH2 | 0,613492853 | -0,679763839 | 1,293256693 | |
| 241822_at | **SIX5** | -0,79540287 | -2,086984744 | 1,291581874 | |
| 222308_x_at | - | -1,399512875 | -2,690860744 | 1,291347869 | |
| 231970_at | C14orf118 | 3,056846434 | 1,765933685 | 1,290912749 | |
| 214294_at | KIAA0485 | -0,622910612 | -1,912089812 | 1,2891792 | |
| 228718_at | ZNF44 | -0,622910612 | -1,912089812 | 1,2891792 | |
| 205364_at | ACOX2 | -0,67289328 | -1,961547147 | 1,288653868 | |
| 1560500_at | - | -0,57445847 | -1,862647763 | 1,288189292 | |
| 244290_at | - | -0,57445847 | -1,862647763 | 1,288189292 | |
| 1559103_s_at | - | -0,926850548 | -2,211750327 | 1,284899779 | |
| 1552980_at | HAS3 | 0,171847695 | -1,112851844 | 1,284699539 | |
| 1554194_at | - | -1,173767127 | -2,456406426 | 1,282639299 | |
| 207931_s_at | PFKFB2 | 0,715481152 | -0,566887459 | 1,282368611 | |
| 1556932_at | - | -1,71423189 | -2,996335703 | 1,282103813 | |
| 234232_at | - | -0,759588698 | -2,037551727 | 1,277963029 | |
| 1554110_at | CDCP1 | -1,131062212 | -2,408705138 | 1,277642925 | |
| 240101_at | - | -1,131062212 | -2,408705138 | 1,277642925 | |
| 205648_at | WNT2 | -0,055687482 | -1,332017329 | 1,276329847 | |
| 229184_at | CCNT2 | -0,888496123 | -2,162639771 | 1,274143649 | |
| 229480_at | MAGI2-AS3 | -0,394749123 | -1,663414495 | 1,268665371 | |
| 1562948_at | - | -1,353583928 | -2,621331989 | 1,267748061 | |
| 207934_at | RFPL1 | -1,353583928 | -2,621331989 | 1,267748061 | |
| 1561052_s_at | - | -1,308181863 | -2,575129729 | 1,266947866 | |
| 1553994_at | NT5E | -1,470833215 | -2,735862426 | 1,26502921 | |
| 1568735_at | - | -1,470833215 | -2,735862426 | 1,26502921 | |
| 229956_at | NR2C1 | -1,470833215 | -2,735862426 | 1,26502921 | |
| 1559340_at | TTLL11-IT1 | -0,946847173 | -2,211750327 | 1,264903154 | |
| 238982_at | DENR | -0,946847173 | -2,211750327 | 1,264903154 | |
| 206616_s_at | ADAM22 | -1,240169874 | -2,503677622 | 1,263507748 | |
| 202663_at | WIPF1 | -0,352951799 | -1,615132408 | 1,262180609 | |
| 239608_at | LPCAT4 | -0,352951799 | -1,615132408 | 1,262180609 | |
| 1552396_at | WFDC6 | -1,196375121 | -2,456406426 | 1,260031305 | |
| 1556533_at | C17orf52 | 0,30867765 | -0,950686014 | 1,259363664 | |
| 232071_at | MRPL19 | 0,103263601 | -1,154897679 | 1,25816128 | |
| 1561223_at | - | -0,83191556 | -2,086984744 | 1,255069184 | |
| 239352_at | SLC6A15 | -0,83191556 | -2,086984744 | 1,255069184 | |
| 208414_s_at | HOXB3 | -0,706943342 | -1,961547147 | 1,254603806 | |
| 219846_at | GON4L | -0,706943342 | -1,961547147 | 1,254603806 | |
| 232764_at | CCNB2 | -0,706943342 | -1,961547147 | 1,254603806 | |

| | | | | | |
|---|---|---|---|---|---|
| 212012_at | PXDN | -0,078265071 | -1,332017329 | 1,253752258 | |
| 1555741_at | MRAP | -0,366776884 | -1,615132408 | 1,248355524 | |
| 231121_at | HPS3 | -0,299034815 | -1,541908042 | 1,242873227 | |
| 234560_at | - | -0,79540287 | -2,037551727 | 1,242148857 | |
| 242302_at | PDS5B | -0,79540287 | -2,037551727 | 1,242148857 | |
| 234332_at | NUB1 | -0,496991414 | -1,738067356 | 1,241075942 | |
| 1562058_at | - | -1,71423189 | -2,954576755 | 1,240344865 | |
| 1562937_at | - | -0,622910612 | -1,862647763 | 1,23973715 | |
| 234586_at | SARDH | -0,67289328 | -1,912089812 | 1,239196532 | |
| 231330_at | LINC00445 | -1,047650486 | -2,286583451 | 1,238932965 | |
| 237706_at | STXBP4 | -1,047650486 | -2,286583451 | 1,238932965 | |
| 204935_at | PTPN2 | 0,702292136 | -0,536087151 | 1,238379287 | |
| 224138_at | CBX2 | 0,083054335 | -1,154897679 | 1,237952014 | |
| 1559062_at | - | -1,173767127 | -2,408705138 | 1,234938011 | |
| 240781_x_at | DCUN1D1 | -1,173767127 | -2,408705138 | 1,234938011 | |
| 239737_at | - | -0,259832361 | -1,494668682 | 1,23483632 | |
| 241128_at | - | -1,763254337 | -2,996335703 | 1,233081366 | |
| 1557022_at | - | -0,148196756 | -1,377419394 | 1,229222638 | |
| 229274_at | GNAS | -0,43775896 | -1,663414495 | 1,225655534 | |
| 1559876_at | - | 0,171847695 | -1,050406615 | 1,222254311 | |
| 1553378_a_at | C17orf66 | -1,399512875 | -2,621331989 | 1,221819114 | |
| 228811_at | - | -1,353583928 | -2,575129729 | 1,221545801 | |
| 221326_s_at | TUBD1 | 1,000476627 | -0,220592223 | 1,221068851 | |
| 202400_s_at | SRF | 0,30867765 | -0,912331589 | 1,221009239 | |
| 236133_x_at | ZNF254 | 0,30867765 | -0,912331589 | 1,221009239 | |
| 37953_s_at | ASIC1 | -0,478090764 | -1,698550632 | 1,220459868 | |
| 1554469_at | ZBTB44 | -0,394749123 | -1,615132408 | 1,220383284 | |
| 1556467_at | LOC100653340 | -1,470833215 | -2,690860744 | 1,220027528 | |
| 233232_at | RHBDL3 | -1,470833215 | -2,690860744 | 1,220027528 | |
| 1553781_at | ZC3HAV1L | -0,741668563 | -1,961547147 | 1,219878585 | |
| 234920_at | ZNF7 | -0,00073712 | -1,220210588 | 1,219473467 | |
| 1552427_at | ZNF485 | -1,2845686 | -2,503677622 | 1,219109022 | |
| 240939_x_at | - | -1,240169874 | -2,456406426 | 1,216236552 | |
| 220020_at | XPNPEP3 | -0,325749314 | -1,541908042 | 1,216158728 | |
| 235784_at | - | -0,946847173 | -2,162639771 | 1,215792598 | |
| 1560590_s_at | - | -0,57445847 | -1,787089803 | 1,212631333 | |
| 209928_s_at | MSC | -1,196375121 | -2,408705138 | 1,212330016 | |
| 1553108_at | C5orf24 | 0,480753764 | -0,730778808 | 1,211532572 | |
| 1559746_a_at | ZNF718 | -0,285837228 | -1,494668682 | 1,208831453 | |
| 218800_at | SRD5A3 | -0,285837228 | -1,494668682 | 1,208831453 | |
| 236849_at | VTI1A | -0,285837228 | -1,494668682 | 1,208831453 | |
| 1570181_a_at | - | -1,639579028 | -2,847001814 | 1,207422786 | |
| 1560094_at | - | -0,655928373 | -1,862647763 | 1,206719389 | |
| 214127_s_at | SRRT | -0,655928373 | -1,862647763 | 1,206719389 | |
| 222723_at | VWA1 | 0,255492549 | -0,950686014 | 1,206178564 | |
| 1553518_at | DEFT1P /// DEFT | -0,83191556 | -2,037551727 | 1,205636167 | |
| 1556175_at | MTSS1L | -0,83191556 | -2,037551727 | 1,205636167 | |
| 1553217_s_at | ZNF41 | -1,131062212 | -2,335104118 | 1,204041905 | |
| 208276_at | - | -1,131062212 | -2,335104118 | 1,204041905 | |
| 217602_at | LOC100288602 / | -0,759588698 | -1,961547147 | 1,201958449 | |
| 243670_at | - | -0,221656139 | -1,423348341 | 1,201692203 | |
| 224166_at | SLC25A2 | -0,888496123 | -2,086984744 | 1,198488621 | |
| 228416_at | ACVR2A | -0,888496123 | -2,086984744 | 1,198488621 | |
| 209544_at | RIPK2 | 0,661985843 | -0,536087151 | 1,198072993 | |
| 213273_at | ODZ4 | -1,089016378 | -2,286583451 | 1,197567073 | |
| 1560661_x_at | - | -0,467091869 | -1,663414495 | 1,196322626 | |

| | | | | | |
|---|---|---|---|---|---|
| 226908_at | LRIG3 | -0,467091869 | -1,663414495 | 1,196322626 | |
| 207648_at | DRP2 | 0,083054335 | -1,112851844 | 1,195906179 | |
| 238559_at | - | -0,136301282 | -1,332017329 | 1,195716047 | |
| 226674_at | SHISA4 | -0,299034815 | -1,494668682 | 1,195633867 | |
| 1554400_at | TCTE3 | -1,308181863 | -2,503677622 | 1,195495759 | |
| 211099_s_at | CNGB1 | -1,308181863 | -2,503677622 | 1,195495759 | |
| 238614_x_at | ZNF430 | 1,504602299 | 0,310717132 | 1,193885167 | |
| 1562319_at | FAM169B | -0,423248115 | -1,615132408 | 1,191884292 | |
| 220056_at | IL22RA1 | -0,423248115 | -1,615132408 | 1,191884292 | |
| 230120_s_at | PLGLB1 /// PLGL | -0,423248115 | -1,615132408 | 1,191884292 | |
| 205301_s_at | OGG1 | 0,181384709 | -1,010192375 | 1,191577084 | |
| 237720_at | ASB4 | -1,763254337 | -2,954576755 | 1,191322418 | |
| 232394_at | - | -0,67289328 | -1,862647763 | 1,189754483 | |
| 210701_at | CFDP1 | -0,85085127 | -2,037551727 | 1,186700457 | |
| 216750_at | APBB2 | -0,85085127 | -2,037551727 | 1,186700457 | |
| 218959_at | HOXC10 | -1,026571149 | -2,211750327 | 1,185179179 | |
| 216422_at | - | -0,148196756 | -1,332017329 | 1,183820573 | |
| 220951_s_at | A1CF | -0,148196756 | -1,332017329 | 1,183820573 | |
| 216681_at | - | -1,71423189 | -2,89013181 | 1,175899921 | |
| 207661_s_at | SH3PXD2A | -1,399512875 | -2,575129729 | 1,175616854 | |
| 235011_at | MAP3K2 | -1,399512875 | -2,575129729 | 1,175616854 | |
| 215922_at | REPS1 | -1,2845686 | -2,456406426 | 1,171837826 | |
| 241043_at | - | -1,2845686 | -2,456406426 | 1,171837826 | |
| 1553644_at | SYNE3 | -1,240169874 | -2,408705138 | 1,168535263 | |
| 226051_at | SELM | -1,240169874 | -2,408705138 | 1,168535263 | |
| 230338_x_at | GSX2 | 0,401366113 | -0,765504029 | 1,166870142 | |
| 241434_at | - | -0,496991414 | -1,663414495 | 1,166423081 | |
| 1555972_s_at | FBXO28 | -0,055687482 | -1,220210588 | 1,164523106 | |
| 219875_s_at | DESI2 | -0,055687482 | -1,220210588 | 1,164523106 | |
| 1554398_at | LYG2 | -0,622910612 | -1,787089803 | 1,164179191 | |
| 234344_at | LOC100288675 | 1,732533761 | 0,568365095 | 1,164168666 | |
| 1564887_at | - | -0,57445847 | -1,738067356 | 1,163608886 | |
| 235129_at | PPP1R1A | -1,173767127 | -2,335104118 | 1,161336991 | |
| 1554283_at | CCRN4L | 1,269985355 | 0,109221392 | 1,160763962 | |
| 206131_at | CLPS | -0,172274514 | -1,332017329 | 1,159742815 | |
| 217181_at | - | -1,688127507 | -2,847001814 | 1,158874308 | |
| 1554544_a_at | MBP | 0,246434561 | -0,912331589 | 1,15876615 | |
| 217344_at | - | -0,221656139 | -1,377419394 | 1,155763256 | |
| 224816_at | GET4 | -0,221656139 | -1,377419394 | 1,155763256 | |
| 1558109_x_at | LOC283788 | -1,131062212 | -2,286583451 | 1,155521238 | |
| 1563539_at | - | -1,131062212 | -2,286583451 | 1,155521238 | |
| 205759_s_at | SULT2B1 | -0,00073712 | -1,154897679 | 1,154160559 | |
| 234751_s_at | NLGN3 | 0,334552598 | -0,819238336 | 1,153790934 | |
| 217112_at | PDGFB | -0,759588698 | -1,912089812 | 1,152501114 | |
| 221828_s_at | FAM125B | -0,512251684 | -1,663414495 | 1,15116281 | |
| 210078_s_at | KCNAB1 | -1,424023738 | -2,575129729 | 1,151105991 | |
| 239527_at | RAB3GAP1 | -1,353583928 | -2,503677622 | 1,150093694 | |
| 236279_at | - | -0,888496123 | -2,037551727 | 1,149055604 | |
| 1559988_at | ZNF483 | -1,308181863 | -2,456406426 | 1,148224563 | |
| 1564632_at | - | -1,308181863 | -2,456406426 | 1,148224563 | |
| 217247_at | - | -1,308181863 | -2,456406426 | 1,148224563 | |
| 238014_at | TMEM194B | -1,308181863 | -2,456406426 | 1,148224563 | |
| 206105_at | AFF2 | -0,59053611 | -1,738067356 | 1,147531246 | |
| 210934_at | BLK | -0,59053611 | -1,738067356 | 1,147531246 | |
| 1557609_s_at | TBC1D12 | -0,394749123 | -1,541908042 | 1,147158919 | |
| 206119_at | BHMT | -0,394749123 | -1,541908042 | 1,147158919 | |

| | | | | | |
|---|---|---|---|---|---|
| 1561429_a_at | C3orf15 /// LOC1 | -1,591296941 | -2,735862426 | 1,144565484 | |
| 1554641_a_at | TET3 | -0,234278768 | -1,377419394 | 1,143140627 | |
| 233522_at | - | -0,352951799 | -1,494668682 | 1,141716883 | |
| 244135_at | - | -0,946847173 | -2,086984744 | 1,140137571 | |
| 1564837_at | LOC151760 | -1,196375121 | -2,335104118 | 1,138728996 | |
| 206585_at | MKRN3 | -1,196375121 | -2,335104118 | 1,138728996 | |
| 206610_s_at | F11 | -0,285837228 | -1,423348341 | 1,137511113 | |
| 228141_at | GPX8 | 0,317354302 | -0,819238336 | 1,136592638 | |
| 204860_s_at | LOC100509323 / | -0,196756757 | -1,332017329 | 1,135260572 | |
| 242433_at | ZBTB11 | -0,196756757 | -1,332017329 | 1,135260572 | |
| 1558041_a_at | KIAA0895L | 1,512180019 | 0,377530647 | 1,134649372 | |
| 216897_s_at | FAM76A | 0,020670649 | -1,112851844 | 1,133522493 | |
| 219800_s_at | THNSL1 | 0,083054335 | -1,050406615 | 1,13346095 | |
| 225279_s_at | C3orf17 | 0,123193849 | -1,010192375 | 1,133386224 | |
| 229215_at | ASCL2 | -0,655928373 | -1,787089803 | 1,13116143 | |
| 236935_at | PTPN4 | -0,83191556 | -1,961547147 | 1,129631587 | |
| 205744_at | DOC2A | 0,273439642 | -0,855751026 | 1,129190668 | |
| 217897_at | FXYD6 | -0,366776884 | -1,494668682 | 1,127891798 | |
| 238776_x_at | OBSL1 | -0,136301282 | -1,264005341 | 1,127704058 | |
| 241333_x_at | - | 0,735041319 | -0,39061235 | 1,125653669 | |
| 243093_at | C2orf49 | -0,299034815 | -1,423348341 | 1,124313527 | |
| 1556831_at | DYNC1H1 | -1,2845686 | -2,408705138 | 1,124136537 | |
| 209765_at | ADAM19 | -1,2845686 | -2,408705138 | 1,124136537 | |
| 223614_at | MMP16 | 0,555999837 | -0,566887459 | 1,122887296 | |
| 214313_s_at | EIF5B | 1,578645857 | 0,456918297 | 1,121727559 | |
| 207774_at | ATG10 | -0,033456564 | -1,154897679 | 1,121441115 | |
| 232521_at | PCSK7 | 0,44160593 | -0,679763839 | 1,121369769 | |
| 235313_at | NRAP | -0,741668563 | -1,862647763 | 1,1209792 | |
| 244797_at | - | -0,741668563 | -1,862647763 | 1,1209792 | |
| 218805_at | GIMAP1-GIMAP5 | 0,900083947 | -0,220592223 | 1,120676171 | |
| 1553133_at | C9orf72 | -0,423248115 | -1,541908042 | 1,118659927 | |
| 226103_at | NEXN | 0,503742507 | -0,614371577 | 1,118114084 | |
| 229368_s_at | ZFAND5 | -0,259832361 | -1,377419394 | 1,117587033 | |
| 206587_at | CCT6B | -0,79540287 | -1,912089812 | 1,116686942 | |
| 230355_at | SEPT7P2 | -0,79540287 | -1,912089812 | 1,116686942 | |
| 244535_at | - | -0,79540287 | -1,912089812 | 1,116686942 | |
| 217660_at | MYH14 | -0,148196756 | -1,264005341 | 1,115808584 | |
| 233630_at | CDS2 | -1,047650486 | -2,162639771 | 1,114989286 | |
| 240520_at | - | -1,047650486 | -2,162639771 | 1,114989286 | |
| 241469_at | HFM1 | -1,047650486 | -2,162639771 | 1,114989286 | |
| 244395_at | FLJ41455 | -2,063149277 | -3,177802286 | 1,114653009 | |
| 1554952_s_at | NLRP12 | -0,67289328 | -1,787089803 | 1,114196523 | |
| 215580_at | MCM3AP | -0,67289328 | -1,787089803 | 1,114196523 | |
| 243364_at | AUTS2 | -0,67289328 | -1,787089803 | 1,114196523 | |
| 202287_s_at | TACSTD2 | -1,173767127 | -2,286583451 | 1,112816324 | |
| 244792_at | - | -1,173767127 | -2,286583451 | 1,112816324 | |
| 219505_at | CECR1 | -0,926850548 | -2,037551727 | 1,110701178 | |
| 221611_s_at | PHF7 | -0,926850548 | -2,037551727 | 1,110701178 | |
| 236520_at | LOC100507495 | -0,926850548 | -2,037551727 | 1,110701178 | |
| 237708_at | - | -0,926850548 | -2,037551727 | 1,110701178 | |
| 217005_at | LDLR | -0,85085127 | -1,961547147 | 1,110695878 | |
| 219543_at | PBLD | -0,85085127 | -1,961547147 | 1,110695878 | |
| 242081_at | ACAP1 | -0,85085127 | -1,961547147 | 1,110695878 | |
| 204437_s_at | FOLR1 | -0,221656139 | -1,332017329 | 1,110361191 | |
| 230774_at | PTGR2 | 1,732533761 | 0,624460759 | 1,108073002 | |
| 230679_at | DCAF10 | 0,376672223 | -0,730778808 | 1,107451031 | |

| | | | | | |
|---|---|---|---|---|---|
| 1554259_at | GPSM1 | 0,822971223 | -0,283667828 | 1,106639051 | |
| 206561_s_at | AKR1B10 | -1,470833215 | -2,575129729 | 1,104296514 | |
| 226641_at | ANKRD44 | -1,470833215 | -2,575129729 | 1,104296514 | |
| 1570276_a_at | GATA4 | -1,399512875 | -2,503677622 | 1,104164747 | |
| 233627_at | - | -1,399512875 | -2,503677622 | 1,104164747 | |
| 1554743_x_at | PMS1 | -0,43775896 | -1,541908042 | 1,104149082 | |
| 232784_at | - | -0,43775896 | -1,541908042 | 1,104149082 | |
| 236953_s_at | NHLRC3 | -0,43775896 | -1,541908042 | 1,104149082 | |
| 210528_at | MR1 | -1,518072576 | -2,621331989 | 1,103259414 | |
| 215181_at | CDH22 | -0,759588698 | -1,862647763 | 1,103059064 | |
| 215688_at | RASGRF1 | -0,512251684 | -1,615132408 | 1,102880723 | |
| 219310_at | SYNDIG1 | -1,353583928 | -2,456406426 | 1,102822498 | |
| 233572_s_at | BCORL1 | 0,052199481 | -1,050406615 | 1,102606096 | |
| 237246_at | - | 0,052199481 | -1,050406615 | 1,102606096 | |
| 242386_x_at | - | 0,052199481 | -1,050406615 | 1,102606096 | |
| 217974_at | TM7SF3 | 0,779676066 | -0,322870281 | 1,102546347 | |
| 205116_at | LAMA2 | -0,986356909 | -2,086984744 | 1,100627835 | |
| 216056_at | - | -0,986356909 | -2,086984744 | 1,100627835 | |
| 1570189_at | LOC388387 | -1,308181863 | -2,408705138 | 1,100523275 | |
| 205559_s_at | PCSK5 | -1,308181863 | -2,408705138 | 1,100523275 | |
| 203563_at | AFAP1 | -1,591296941 | -2,690860744 | 1,099563802 | |
| 240613_at | JAK1 | -1,591296941 | -2,690860744 | 1,099563802 | |
| 224491_at | APOL4 | -0,055687482 | -1,154897679 | 1,099210197 | |
| 1567010_at | - | -0,325749314 | -1,423348341 | 1,097599027 | |
| 228405_at | RHPN1 | -0,325749314 | -1,423348341 | 1,097599027 | |
| 223355_at | ALG1 | 0,747935635 | -0,34958478 | 1,097520416 | |
| 1563345_at | - | -1,639579028 | -2,735862426 | 1,096283397 | |
| 1565856_at | - | -1,639579028 | -2,735862426 | 1,096283397 | |
| 236922_at | - | -1,240169874 | -2,335104118 | 1,094934243 | |
| 235586_at | - | 0,181384709 | -0,912331589 | 1,093716298 | |
| 226759_at | IKZF4 | 0,083054335 | -1,010192375 | 1,09324671 | |
| 224525_s_at | OLA1 | -0,285837228 | -1,377419394 | 1,091582166 | |
| 231607_at | LOC100507480 | -0,946847173 | -2,037551727 | 1,090704553 | |
| 233607_at | - | -0,946847173 | -2,037551727 | 1,090704553 | |
| 238166_s_at | C17orf51 /// FAM | -0,946847173 | -2,037551727 | 1,090704553 | |
| 204112_s_at | HNMT | -1,196375121 | -2,286583451 | 1,090208329 | |
| 208533_at | SOX1 | -1,196375121 | -2,286583451 | 1,090208329 | |
| 236638_at | FAM123C | -1,196375121 | -2,286583451 | 1,090208329 | |
| 1552927_at | TAB3 | -0,57445847 | -1,663414495 | 1,088956024 | |
| 202711_at | EFNB1 | -0,57445847 | -1,663414495 | 1,088956024 | |
| 219914_at | ECEL1 | -0,57445847 | -1,663414495 | 1,088956024 | |
| 1560879_a_at | SYT15 | -0,136301282 | -1,220210588 | 1,083909306 | |
| 216490_x_at | - | -0,136301282 | -1,220210588 | 1,083909306 | |
| 211213_at | ORC5 | -1,763254337 | -2,847001814 | 1,083747477 | |
| 216022_at | - | -1,763254337 | -2,847001814 | 1,083747477 | |
| 204525_at | PHF14 | 0,317354302 | -0,765504029 | 1,082858331 | |
| 229379_at | AHDC1 | 0,03125738 | -1,050406615 | 1,081663995 | |
| 230476_at | - | -1,131062212 | -2,211750327 | 1,080688115 | |
| 213997_at | FAM189A1 | -0,706943342 | -1,787089803 | 1,080146461 | |
| 230156_x_at | - | -0,706943342 | -1,787089803 | 1,080146461 | |
| 205027_s_at | MAP3K8 | 0,728550685 | -0,34958478 | 1,078135466 | |
| 229306_at | C2CD4B | -1,543331864 | -2,621331989 | 1,078000125 | |
| 230548_at | LOC100505942 | -0,467091869 | -1,541908042 | 1,074816173 | |
| 220300_at | RGS3 | -1,089016378 | -2,162639771 | 1,073623394 | |
| 222780_s_at | BAALC | -1,089016378 | -2,162639771 | 1,073623394 | |
| 241225_at | - | -1,089016378 | -2,162639771 | 1,073623394 | |

| | | | | | |
|---|---|---|---|---|---|
| 1561319_at | OTX2-AS1 | -1,964176657 | -3,037500868 | 1,073324211 | |
| 1561358_at | TXLNA | -0,888496123 | -1,961547147 | 1,073051025 | |
| 220263_at | SMAD5-AS1 | -0,888496123 | -1,961547147 | 1,073051025 | |
| 230690_at | TUBB1 | -0,888496123 | -1,961547147 | 1,073051025 | |
| 210626_at | AKAP1 | -0,543051993 | -1,615132408 | 1,072080415 | |
| 1553423_a_at | SLFN13 | -0,148196756 | -1,220210588 | 1,072013831 | |
| 1556589_at | - | -0,423248115 | -1,494668682 | 1,071420566 | |
| 234016_at | LOC90499 | -0,423248115 | -1,494668682 | 1,071420566 | |
| 1564921_at | KRTAP13-1 | 0,020670649 | -1,050406615 | 1,071077264 | |
| 239753_at | ZNF252P | 0,020670649 | -1,050406615 | 1,071077264 | |
| 230697_at | BBS5 | -0,352951799 | -1,423348341 | 1,070396543 | |
| 222262_s_at | ETNK1 | 0,606430281 | -0,461594427 | 1,068024708 | |
| 1555363_s_at | LOC284440 | -0,196756757 | -1,264005341 | 1,067248584 | |
| 230062_at | RIMBP3 /// RIMB | -0,196756757 | -1,264005341 | 1,067248584 | |
| 235149_at | PGM2L1 | -0,196756757 | -1,264005341 | 1,067248584 | |
| 216721_at | SLC25A30 | -0,79540287 | -1,862647763 | 1,067244893 | |
| 241080_at | - | -0,79540287 | -1,862647763 | 1,067244893 | |
| 241327_at | - | -0,79540287 | -1,862647763 | 1,067244893 | |
| 211788_s_at | TREX2 | -0,67289328 | -1,738067356 | 1,065174076 | |
| 222656_at | UBE2W | 1,442503549 | 0,377530647 | 1,064972902 | |
| 205117_at | FGF1 | 0,152582607 | -0,912331589 | 1,064914196 | |
| 237301_at | - | 0,384950573 | -0,679763839 | 1,064714413 | |
| 240091_at | PSMA8 | 1,897847661 | 0,835239052 | 1,062608609 | |
| 213920_at | CUX2 | -0,85085127 | -1,912089812 | 1,061238542 | |
| 239828_at | AKD1 | -0,85085127 | -1,912089812 | 1,061238542 | |
| 214227_at | - | -1,026571149 | -2,086984744 | 1,060413595 | |
| 228589_at | BASP1 | -1,026571149 | -2,086984744 | 1,060413595 | |
| 234371_at | - | -1,026571149 | -2,086984744 | 1,060413595 | |
| 239407_at | GCFC1-AS1 | -0,055687482 | -1,112851844 | 1,057164362 | |
| 234653_at | - | -0,43775896 | -1,494668682 | 1,056909721 | |
| 1552986_at | LOC142937 | -1,399512875 | -2,456406426 | 1,056893551 | |
| 210728_s_at | CALCA | -1,399512875 | -2,456406426 | 1,056893551 | |
| 1554677_s_at | CMTM4 | -0,366776884 | -1,423348341 | 1,056571458 | |
| 1557383_a_at | - | -1,353583928 | -2,408705138 | 1,05512121 | |
| 201864_at | GDI1 | 2,254767157 | 1,200746069 | 1,054021088 | |
| 1562988_at | ZSCAN30 | -1,838812296 | -2,89013181 | 1,051319514 | |
| 1560043_at | CYB5R1 | -1,639579028 | -2,690860744 | 1,051281715 | |
| 1560557_at | - | -1,639579028 | -2,690860744 | 1,051281715 | |
| 228716_at | THRB | -1,639579028 | -2,690860744 | 1,051281715 | |
| 240974_at | LOC100506851 | -1,639579028 | -2,690860744 | 1,051281715 | |
| 206358_at | PRM1 | -0,986356909 | -2,037551727 | 1,051194818 | |
| 233551_at | LOC642776 | -0,986356909 | -2,037551727 | 1,051194818 | |
| 234416_at | - | -0,986356909 | -2,037551727 | 1,051194818 | |
| 240824_at | - | -0,986356909 | -2,037551727 | 1,051194818 | |
| 207222_at | LOC100652777 / | -0,00073712 | -1,050406615 | 1,049669495 | |
| 239855_at | PPM1L | -0,00073712 | -1,050406615 | 1,049669495 | |
| 230713_at | - | 0,82905153 | -0,220592223 | 1,049643754 | |
| 229303_at | - | 1,40228911 | 0,352836757 | 1,049452352 | |
| 216426_at | - | 0,401366113 | -0,646746079 | 1,048112192 | |
| 221703_at | BRIP1 | 0,480753764 | -0,566887459 | 1,047641223 | |
| 228139_at | RIPK3 | 0,228146222 | -0,819238336 | 1,047384558 | |
| 1557607_at | LOC284080 | -1,240169874 | -2,286583451 | 1,046413576 | |
| 1558983_at | LRRC41 | -1,240169874 | -2,286583451 | 1,046413576 | |
| 1564621_a_at | MEI1 | -1,240169874 | -2,286583451 | 1,046413576 | |
| 240567_at | - | -1,240169874 | -2,286583451 | 1,046413576 | |
| 241629_at | - | -0,285837228 | -1,332017329 | 1,046180101 | |

| | | | | | |
|---|---|---|---|---|---|
| 1558791_at | LOC286467 | -0,741668563 | -1,787089803 | 1,04542124 | |
| 240324_at | - | -0,741668563 | -1,787089803 | 1,04542124 | |
| 1555766_a_at | GNG2 | 0,133056859 | -0,912331589 | 1,045388447 | |
| 206336_at | CXCL6 | 0,822971223 | -0,220592223 | 1,043563447 | |
| 218513_at | TMA16 | 2,493593689 | 1,450051551 | 1,043542138 | |
| 1556144_at | DHX30 | -0,221656139 | -1,264005341 | 1,042349202 | |
| 212524_x_at | H2AFX | -0,221656139 | -1,264005341 | 1,042349202 | |
| 243612_at | NSD1 | -0,221656139 | -1,264005341 | 1,042349202 | |
| 233946_at | SMU1 | -0,112808516 | -1,154897679 | 1,042089162 | |
| 220379_at | FSCN3 | 0,03125738 | -1,010192375 | 1,041449755 | |
| 242265_at | BRD8 | 0,03125738 | -1,010192375 | 1,041449755 | |
| 229991_s_at | SYTL4 | 0,995078803 | -0,046302147 | 1,04138095 | |
| 227462_at | ERAP2 | -0,57445847 | -1,615132408 | 1,040673938 | |
| 1556107_at | - | 0,425644434 | -0,614371577 | 1,04001601 | |
| 1563003_at | ITGAX | -1,047650486 | -2,086984744 | 1,039334258 | |
| 219903_s_at | CYP2C8 | -1,047650486 | -2,086984744 | 1,039334258 | |
| 223998_at | TTLL2 | -1,047650486 | -2,086984744 | 1,039334258 | |
| 230038_at | ATXN7L2 | -1,047650486 | -2,086984744 | 1,039334258 | |
| 235455_at | FAM131C | -1,047650486 | -2,086984744 | 1,039334258 | |
| 239970_at | - | -1,047650486 | -2,086984744 | 1,039334258 | |
| 1562908_at | LOC339468 | -2,138804305 | -3,177802286 | 1,038997981 | |
| 206297_at | CTRC | 0,273439642 | -0,765504029 | 1,038943671 | |
| 241920_x_at | SPG11 | -1,173767127 | -2,211750327 | 1,037983201 | |
| 213281_at | JUN | 1,011212064 | -0,024572586 | 1,03578465 | |
| 1562581_at | LOC254028 | -0,078265071 | -1,112851844 | 1,034586773 | |
| 242928_at | - | -0,078265071 | -1,112851844 | 1,034586773 | |
| 1558683_a_at | HMGA2 | -1,812418401 | -2,847001814 | 1,034583413 | |
| 204446_s_at | ALOX5 | -1,812418401 | -2,847001814 | 1,034583413 | |
| 1554274_a_at | SSH1 | -0,299034815 | -1,332017329 | 1,032982514 | |
| 1568814_at | DDX50 | -0,299034815 | -1,332017329 | 1,032982514 | |
| 229760_at | VEPH1 | -0,299034815 | -1,332017329 | 1,032982514 | |
| 228343_at | POU2F2 | -1,470833215 | -2,503677622 | 1,032844407 | |
| 243746_at | IGHMBP2 | -1,470833215 | -2,503677622 | 1,032844407 | |
| 243781_at | - | -1,424023738 | -2,456406426 | 1,032382688 | |
| 221681_s_at | DSPP | -1,964176657 | -2,996335703 | 1,032159046 | |
| 213882_at | TM2D1 | 0,641402394 | -0,39061235 | 1,032014745 | |
| 1558560_s_at | BLZF1 | -1,543331864 | -2,575129729 | 1,031797865 | |
| 220116_at | KCNN2 | -1,543331864 | -2,575129729 | 1,031797865 | |
| 228554_at | PGR | -1,543331864 | -2,575129729 | 1,031797865 | |
| 1565775_at | PIEZO2 | -1,131062212 | -2,162639771 | 1,031577559 | |
| 231066_s_at | CLCN4 | -1,131062212 | -2,162639771 | 1,031577559 | |
| 237635_at | LOC100128164 | -1,131062212 | -2,162639771 | 1,031577559 | |
| 1558855_at | FARP2 | -0,706943342 | -1,738067356 | 1,031124014 | |
| 221186_at | LOC100131532 | -0,706943342 | -1,738067356 | 1,031124014 | |
| 1561973_at | SMARCC2 | -0,83191556 | -1,862647763 | 1,030732203 | |
| 207681_at | CXCR3 | -0,83191556 | -1,862647763 | 1,030732203 | |
| 214329_x_at | TNFSF10 | -0,83191556 | -1,862647763 | 1,030732203 | |
| 238275_at | HAP1 | -0,83191556 | -1,862647763 | 1,030732203 | |
| 240583_at | RIOK3 | -0,83191556 | -1,862647763 | 1,030732203 | |
| 1554216_at | CCDC132 | -1,591296941 | -2,621331989 | 1,030035048 | |
| 211904_x_at | RAD52 | -0,394749123 | -1,423348341 | 1,028599218 | |
| 233520_s_at | CMYA5 | -0,022466681 | -1,050406615 | 1,027939934 | |
| 230600_at | LRRC46 | -0,467091869 | -1,494668682 | 1,027576813 | |
| 231449_at | - | -0,467091869 | -1,494668682 | 1,027576813 | |
| 238616_at | QDPR | -0,467091869 | -1,494668682 | 1,027576813 | |
| 222281_s_at | C1orf186 /// LOC | -0,759588698 | -1,787089803 | 1,027501105 | |

| | | | | | |
|---|---|---|---|---|---|
| 244704_at | NFYB | -0,759588698 | -1,787089803 | 1,027501105 | |
| 239132_at | NOS1 | -1,308181863 | -2,335104118 | 1,026922255 | |
| 241481_at | FAM81A | -1,308181863 | -2,335104118 | 1,026922255 | |
| 1562792_at | NIPAL1 | -0,59053611 | -1,615132408 | 1,024596297 | |
| 230301_at | - | -0,59053611 | -1,615132408 | 1,024596297 | |
| 214593_at | PIAS2 | -2,01371626 | -3,037500868 | 1,023784608 | |
| 1569172_a_at | LOC402160 | -0,888496123 | -1,912089812 | 1,023593689 | |
| 205591_at | OLFM1 | -0,888496123 | -1,912089812 | 1,023593689 | |
| 206089_at | NELL1 | -0,888496123 | -1,912089812 | 1,023593689 | |
| 214175_x_at | PDLIM4 | -0,888496123 | -1,912089812 | 1,023593689 | |
| 236845_at | TRIM62 | -0,888496123 | -1,912089812 | 1,023593689 | |
| 205636_at | SH3GL3 | -0,196756757 | -1,220210588 | 1,023453831 | |
| 243212_at | LOC100505820 | -0,089689931 | -1,112851844 | 1,023161913 | |
| 239161_at | FDX1 | 0,255492549 | -0,765504029 | 1,020996578 | |
| 209135_at | ASPH | 1,515954005 | 0,495031851 | 1,020922155 | |
| 235311_at | FKBP14 | -0,136301282 | -1,154897679 | 1,018596397 | |
| 240919_at | - | -0,136301282 | -1,154897679 | 1,018596397 | |
| 1556803_at | POLR3B | -1,763254337 | -2,780313924 | 1,017059587 | |
| 239533_at | GPR155 | -1,763254337 | -2,780313924 | 1,017059587 | |
| 243497_at | - | -1,937711681 | -2,954576755 | 1,016865074 | |
| 223190_s_at | MLL5 | 3,606946883 | 2,590852465 | 1,016094418 | |
| 65718_at | GPR124 | -0,095377784 | -1,111383214 | 1,016005431 | |
| 207965_at | NEUROG3 | -1,196375121 | -2,211750327 | 1,015375206 | |
| 236020_s_at | TRUB1 | -1,196375121 | -2,211750327 | 1,015375206 | |
| 239550_at | RORA | -1,196375121 | -2,211750327 | 1,015375206 | |
| 204909_at | DDX6 | -0,946847173 | -1,961547147 | 1,014699974 | |
| 205317_s_at | SLC15A2 | -0,946847173 | -1,961547147 | 1,014699974 | |
| 206273_at | SLMO1 | -0,946847173 | -1,961547147 | 1,014699974 | |
| 227933_at | LINGO1 | -0,946847173 | -1,961547147 | 1,014699974 | |
| 211078_s_at | STK3 | 0,334552598 | -0,679763839 | 1,014316438 | |
| 218180_s_at | EPS8L2 | 0,246434561 | -0,765504029 | 1,01193859 | |
| 221841_s_at | KLF4 | -0,85085127 | -1,862647763 | 1,011796493 | |
| 238111_at | SDCCAG3 | -0,85085127 | -1,862647763 | 1,011796493 | |
| 239741_at | PYGO1 | -0,85085127 | -1,862647763 | 1,011796493 | |
| 205434_s_at | AAK1 | 2,361396186 | 1,349621332 | 1,011774854 | |
| 205678_at | AP3B2 | -1,026571149 | -2,037551727 | 1,010980578 | |
| 227589_at | PITPNC1 | -1,026571149 | -2,037551727 | 1,010980578 | |
| 235979_at | C7 | -1,026571149 | -2,037551727 | 1,010980578 | |
| 239929_at | PM20D1 | -1,026571149 | -2,037551727 | 1,010980578 | |
| 242271_at | SLC26A9 | -1,026571149 | -2,037551727 | 1,010980578 | |
| 1566526_at | LOC283688 | -0,366776884 | -1,377419394 | 1,01064251 | |
| 223601_at | OLFM2 | -0,366776884 | -1,377419394 | 1,01064251 | |
| 243451_at | - | -0,366776884 | -1,377419394 | 1,01064251 | |
| 221975_s_at | C21orf2 | -0,00073712 | -1,010192375 | 1,009455255 | |
| 229581_at | ELFN1 | -0,00073712 | -1,010192375 | 1,009455255 | |
| 229569_at | - | -1,399512875 | -2,408705138 | 1,009192262 | |
| 232179_at | LOC158863 | -1,399512875 | -2,408705138 | 1,009192262 | |
| 229839_at | SCARA5 | -1,838812296 | -2,847001814 | 1,008189518 | |
| 205567_at | CHST1 | -0,655928373 | -1,663414495 | 1,007486122 | |
| 207205_at | CEACAM4 | -0,148196756 | -1,154897679 | 1,006700922 | |
| 234747_at | TRAPPC11 | -0,325749314 | -1,332017329 | 1,006268015 | |
| 212364_at | MYO1B | 0,273439642 | -0,730778808 | 1,00421845 | |
| 220905_at | - | 0,052199481 | -0,950686014 | 1,002885495 | |
| 233539_at | NAPEPLD | 0,052199481 | -0,950686014 | 1,002885495 | |
| 1553412_at | AGBL4 | -1,688127507 | -2,690860744 | 1,002733237 | |
| 1563563_at | CCDC40 | -1,688127507 | -2,690860744 | 1,002733237 | |

| | | | | | |
|---|---|---|---|---|---|
| 211303_x_at | FOLH1B | -1,688127507 | -2,690860744 | 1,002733237 | |
| 234440_at | TRDV3 | -1,688127507 | -2,690860744 | 1,002733237 | |
| 1557754_at | LOC401068 | -1,2845686 | -2,286583451 | 1,00201485 | |
| 231268_at | LOC645895 /// M | -1,2845686 | -2,286583451 | 1,00201485 | |
| 231580_at | LOC100506542 | -1,2845686 | -2,286583451 | 1,00201485 | |
| 241059_at | - | -1,2845686 | -2,286583451 | 1,00201485 | |
| 241686_x_at | - | 0,181384709 | -0,819238336 | 1,000623045 | |
| 229027_at | PPM1A | 1,394109935 | 0,393761504 | 1,000348431 | |
| 1560001_at | LOC100131581 | -0,423248115 | -1,423348341 | 1,000100226 | |
| 1570349_at | - | -0,423248115 | -1,423348341 | 1,000100226 | |
| 210324_at | C8G | -0,423248115 | -1,423348341 | 1,000100226 | |
| 1552559_a_at | CDK15 | -0,543051993 | -1,541908042 | 0,998856049 | |
| 206578_at | NKX2-5 | -0,543051993 | -1,541908042 | 0,998856049 | |
| 211238_at | ADAM7 | -0,543051993 | -1,541908042 | 0,998856049 | |
| 228673_s_at | - | -0,543051993 | -1,541908042 | 0,998856049 | |
| 1566900_at | - | -1,089016378 | -2,086984744 | 0,997968366 | |
| 232395_x_at | AGBL3 | -1,089016378 | -2,086984744 | 0,997968366 | |
| 241451_s_at | - | 0,317354302 | -0,679763839 | 0,997118141 | |
| 228751_at | CLK4 | 0,606430281 | -0,39061235 | 0,997042632 | |
| 215955_x_at | ARHGAP26 | -0,741668563 | -1,738067356 | 0,996398793 | |
| 223942_x_at | CHST5 | -0,055687482 | -1,050406615 | 0,994719133 | |
| 240084_at | CBX2 | 0,457392764 | -0,536087151 | 0,993479915 | |
| 1559495_at | - | -0,79540287 | -1,787089803 | 0,991686933 | |
| 237675_at | - | -0,79540287 | -1,787089803 | 0,991686933 | |
| 238843_at | NPHP1 | -0,79540287 | -1,787089803 | 0,991686933 | |
| 244362_at | - | -0,79540287 | -1,787089803 | 0,991686933 | |
| 224475_at | - | 0,171847695 | -0,819238336 | 0,991086032 | |
| 1552541_at | TAGAP | 0,888485433 | -0,102100538 | 0,990585971 | |
| 235844_at | PHTF1 | -0,67289328 | -1,663414495 | 0,990521215 | |
| 227695_at | GLYATL1 /// LOC | -1,047650486 | -2,037551727 | 0,989901241 | |
| 209949_at | NCF2 | -1,173767127 | -2,162639771 | 0,988872645 | |
| 228489_at | TM4SF18 | -1,173767127 | -2,162639771 | 0,988872645 | |
| 1569024_at | FAM13A | -0,022466681 | -1,010192375 | 0,987725694 | |
| 221854_at | PKP1 | -0,022466681 | -1,010192375 | 0,987725694 | |
| 220998_s_at | UNC93B1 | 0,995078803 | 0,007421914 | 0,987656889 | |
| 226962_at | ZBTB41 | 0,496120222 | -0,490927335 | 0,987047557 | |
| 232110_at | GALNT5 | 0,255492549 | -0,730778808 | 0,986271357 | |
| 235635_at | ARHGAP5 | 0,255492549 | -0,730778808 | 0,986271357 | |
| 243815_at | PGBD4 | -0,234278768 | -1,220210588 | 0,98593182 | |
| 244487_at | - | -0,234278768 | -1,220210588 | 0,98593182 | |
| 1556618_at | - | -1,518072576 | -2,503677622 | 0,985605047 | |
| 1568665_at | RNF103 | -1,518072576 | -2,503677622 | 0,985605047 | |
| 1570627_at | TCEB3 | -1,518072576 | -2,503677622 | 0,985605047 | |
| 216198_at | ATF7IP | -1,518072576 | -2,503677622 | 0,985605047 | |
| 228791_at | LOC100129502 | -1,518072576 | -2,503677622 | 0,985605047 | |
| 229543_at | - | -1,518072576 | -2,503677622 | 0,985605047 | |
| 1562532_at | - | -1,470833215 | -2,456406426 | 0,985573211 | |
| 1566783_at | - | -1,470833215 | -2,456406426 | 0,985573211 | |
| 240100_at | LOC100505920 | -1,470833215 | -2,456406426 | 0,985573211 | |
| 1564765_at | - | -0,926850548 | -1,912089812 | 0,985239264 | |
| 217396_at | - | -0,926850548 | -1,912089812 | 0,985239264 | |
| 1570546_a_at | TACC2 | -1,424023738 | -2,408705138 | 0,984681399 | |
| 231332_at | - | -1,424023738 | -2,408705138 | 0,984681399 | |
| 236602_at | - | -1,424023738 | -2,408705138 | 0,984681399 | |
| 241523_at | - | -1,591296941 | -2,575129729 | 0,983832788 | |
| 1552727_s_at | ADAMTS17 | -0,172274514 | -1,154897679 | 0,982623165 | |

| | | | | | |
|---|---|---|---|---|---|
| 210148_at | HIPK3 | -0,172274514 | -1,154897679 | 0,982623165 | |
| 229389_at | ATG16L2 | -0,172274514 | -1,154897679 | 0,982623165 | |
| 236998_at | CCDC56 | -0,172274514 | -1,154897679 | 0,982623165 | |
| 239713_at | CASC2 | -2,01371626 | -2,996335703 | 0,982619443 | |
| 204199_at | RALGPS1 | -0,512251684 | -1,494668682 | 0,982416997 | |
| 221986_s_at | KLHL24 | -0,512251684 | -1,494668682 | 0,982416997 | |
| 239011_at | PRKCE | -1,639579028 | -2,621331989 | 0,981752961 | |
| 208538_at | ANP32C | -1,353583928 | -2,335104118 | 0,98152019 | |
| 227310_at | ADSS | -1,353583928 | -2,335104118 | 0,98152019 | |
| 237524_at | - | -1,353583928 | -2,335104118 | 0,98152019 | |
| 223511_at | SPRTN | 1,274448124 | 0,293518836 | 0,980929288 | |
| 206675_s_at | SKIL | -0,352951799 | -1,332017329 | 0,979065531 | |
| 204470_at | CXCL1 | -0,759588698 | -1,738067356 | 0,978478658 | |
| 205710_at | LRP2 | -0,759588698 | -1,738067356 | 0,978478658 | |
| 206681_x_at | GP2 | -0,759588698 | -1,738067356 | 0,978478658 | |
| 230414_s_at | TMEM170A | -0,759588698 | -1,738067356 | 0,978478658 | |
| 231322_at | - | -0,759588698 | -1,738067356 | 0,978478658 | |
| 1569900_at | FLJ16124 | -1,308181863 | -2,286583451 | 0,978401588 | |
| 217538_at | SGSM2 | -1,308181863 | -2,286583451 | 0,978401588 | |
| 222534_s_at | INF2 | -1,308181863 | -2,286583451 | 0,978401588 | |
| 236230_at | - | -1,308181863 | -2,286583451 | 0,978401588 | |
| 236842_at | LOC100506252 | -1,308181863 | -2,286583451 | 0,978401588 | |
| 237466_s_at | HHIP | -0,285837228 | -1,264005341 | 0,978168112 | |
| 217176_s_at | ZFX | 0,246434561 | -0,730778808 | 0,977213369 | |
| 229861_at | - | 0,984222186 | 0,007421914 | 0,976800272 | |
| 215196_at | - | -0,033456564 | -1,010192375 | 0,976735811 | |
| 207164_s_at | ZNF238 | -0,986356909 | -1,961547147 | 0,975190239 | |
| 230888_at | WDR91 | -0,986356909 | -1,961547147 | 0,975190239 | |
| 231559_at | LOC100506941 | -0,986356909 | -1,961547147 | 0,975190239 | |
| 1567270_at | - | -2,063149277 | -3,037500868 | 0,974351591 | |
| 207554_x_at | TBXA2R | 0,359971644 | -0,614371577 | 0,974343221 | |
| 242456_at | MRE11A | -0,888496123 | -1,862647763 | 0,97415164 | |
| 243706_at | CDO1 | -0,888496123 | -1,862647763 | 0,97415164 | |
| 217604_at | - | 1,177702228 | 0,204310755 | 0,973391472 | |
| 217236_x_at | IGH@ /// IGHA1 / | -1,763254337 | -2,735862426 | 0,972608089 | |
| 205684_s_at | DENND4C | 1,789769151 | 0,817300337 | 0,972468814 | |
| 218637_at | IMPACT | 1,596577845 | 0,624460759 | 0,972117086 | |
| 209543_s_at | CD34 | 0,152582607 | -0,819238336 | 0,971820943 | |
| 1557380_at | AGAP11 | -1,240169874 | -2,211750327 | 0,971580453 | |
| 227225_at | ZNF503 | -1,240169874 | -2,211750327 | 0,971580453 | |
| 244377_at | SLC1A4 | -1,240169874 | -2,211750327 | 0,971580453 | |
| 244525_at | - | -1,240169874 | -2,211750327 | 0,971580453 | |
| 217964_at | TTC19 | 3,925549439 | 2,957221307 | 0,968328133 | |
| 239260_at | CORIN | -1,812418401 | -2,780313924 | 0,967895523 | |
| 243234_at | TBX3 | -1,812418401 | -2,780313924 | 0,967895523 | |
| 231324_at | - | -0,57445847 | -1,541908042 | 0,967449572 | |
| 238072_at | CLK3 | -0,57445847 | -1,541908042 | 0,967449572 | |
| 242577_at | LOC389834 | -0,57445847 | -1,541908042 | 0,967449572 | |
| 1561116_at | - | -1,196375121 | -2,162639771 | 0,96626465 | |
| 237321_at | LOC100506457 | -1,196375121 | -2,162639771 | 0,96626465 | |
| 1554324_s_at | DYNC2LI1 | -0,946847173 | -1,912089812 | 0,965242639 | |
| 212172_at | AK2 | -0,946847173 | -1,912089812 | 0,965242639 | |
| 217634_at | SVIL | -0,946847173 | -1,912089812 | 0,965242639 | |
| 230307_at | LOC100129794 | -0,946847173 | -1,912089812 | 0,965242639 | |
| 240559_at | IZUMO2 | -0,946847173 | -1,912089812 | 0,965242639 | |
| 219368_at | NAP1L2 | -0,366776884 | -1,332017329 | 0,965240445 | |

| | | | | | |
|---|---|---|---|---|---|
| 215421_at | LOC100131510 | -0,299034815 | -1,264005341 | 0,964970526 | |
| 1570108_at | - | -0,148196756 | -1,112851844 | 0,964655088 | |
| 234870_at | TANC1 | -0,148196756 | -1,112851844 | 0,964655088 | |
| 238972_at | - | 0,052199481 | -0,912331589 | 0,964531069 | |
| 241533_at | LOC731656 | 0,767063567 | -0,19610998 | 0,963173547 | |
| 219664_s_at | DECR2 | 2,277177578 | 1,315948728 | 0,96122885 | |
| 219290_x_at | DAPP1 | 0,859074518 | -0,102100538 | 0,961175056 | |
| 1568822_at | GTPBP5 | -0,089689931 | -1,050406615 | 0,960716684 | |
| 203604_at | ZNF516 | -0,259832361 | -1,220210588 | 0,960378226 | |
| 211565_at | SH3GL3 | -0,259832361 | -1,220210588 | 0,960378226 | |
| 228913_at | TPT1-AS1 | -0,259832361 | -1,220210588 | 0,960378226 | |
| 1562945_at | - | -1,543331864 | -2,503677622 | 0,960345758 | |
| 229170_s_at | TTC18 | -1,543331864 | -2,503677622 | 0,960345758 | |
| 232674_at | UCN2 | -1,543331864 | -2,503677622 | 0,960345758 | |
| 233436_at | MTBP | -1,543331864 | -2,503677622 | 0,960345758 | |
| 220316_at | NPAS3 | -0,655928373 | -1,615132408 | 0,959204035 | |
| 1561237_at | - | -1,888254346 | -2,847001814 | 0,958747469 | |
| 230952_at | - | -1,888254346 | -2,847001814 | 0,958747469 | |
| 243571_at | - | -1,888254346 | -2,847001814 | 0,958747469 | |
| 205617_at | PRRG2 | 0,496120222 | -0,461594427 | 0,957714648 | |
| 205879_x_at | RET | 0,496120222 | -0,461594427 | 0,957714648 | |
| 1558604_a_at | SSBP2 | -0,706943342 | -1,663414495 | 0,956471153 | |
| 229866_at | STK32A | -0,706943342 | -1,663414495 | 0,956471153 | |
| 226997_at | ADAMTS12 | -0,467091869 | -1,423348341 | 0,956256473 | |
| 242004_x_at | - | -0,467091869 | -1,423348341 | 0,956256473 | |
| 235178_x_at | ESCO2 | 0,46522187 | -0,490927335 | 0,956149205 | |
| 1568126_at | ANXA2 | -1,131062212 | -2,086984744 | 0,955922531 | |
| 232080_at | HECW2 | -1,131062212 | -2,086984744 | 0,955922531 | |
| 226341_at | LOC100506365 | 1,995690482 | 1,039887232 | 0,955803251 | |
| 202022_at | ALDOC | 0,735041319 | -0,220592223 | 0,955633543 | |
| 1566208_at | TCEA1 | -0,83191556 | -1,787089803 | 0,955174243 | |
| 217153_at | ARHGAP1 | -0,83191556 | -1,787089803 | 0,955174243 | |
| 235620_x_at | ZMYM5 | -0,055687482 | -1,010192375 | 0,954504893 | |
| 220342_x_at | EDEM3 | 1,063722698 | 0,109221392 | 0,954501305 | |
| 227744_s_at | HNRNPD | -0,423248115 | -1,377419394 | 0,954171279 | |
| 214120_at | RFPL1-AS1 | 0,417596971 | -0,536087151 | 0,953684121 | |
| 229075_at | SPATA5 | 1,829737795 | 0,876248481 | 0,953489314 | |
| 1559509_at | - | 0,273439642 | -0,679763839 | 0,953203481 | |
| 231193_s_at | - | -1,937711681 | -2,89013181 | 0,952420129 | |
| 231560_at | - | -1,937711681 | -2,89013181 | 0,952420129 | |
| 244814_at | UGGT1 | 0,133056859 | -0,819238336 | 0,952295195 | |
| 212917_x_at | RECQL | 2,577249059 | 1,6252386 | 0,952010458 | |
| 1559078_at | - | -0,543051993 | -1,494668682 | 0,951616688 | |
| 1565082_x_at | SRC | -0,543051993 | -1,494668682 | 0,951616688 | |
| 206195_x_at | GH2 | -0,543051993 | -1,494668682 | 0,951616688 | |
| 222862_s_at | AK5 | -0,543051993 | -1,494668682 | 0,951616688 | |
| 1562624_at | - | -0,59053611 | -1,541908042 | 0,951371932 | |
| 202036_s_at | SFRP1 | -0,59053611 | -1,541908042 | 0,951371932 | |
| 230393_at | CUL5 | -0,59053611 | -1,541908042 | 0,951371932 | |
| 233513_at | - | -0,59053611 | -1,541908042 | 0,951371932 | |
| 237852_at | - | -0,59053611 | -1,541908042 | 0,951371932 | |
| 217599_s_at | MDFIC | 1,079110292 | 0,128747141 | 0,950363151 | |
| 206130_s_at | ASGR2 | -0,00073712 | -0,950686014 | 0,949948894 | |
| 211830_s_at | CACNA1I | -0,00073712 | -0,950686014 | 0,949948894 | |
| 208016_s_at | AGTR1 | 1,481627361 | 0,532164371 | 0,949462991 | |
| 210353_s_at | SLC6A2 | -1,089016378 | -2,037551727 | 0,948535349 | |

| | | | | | |
|---|---|---|---|---|---|
| 220130_x_at | LTB4R2 | -1,089016378 | -2,037551727 | 0,948535349 | |
| 222377_at | TBX10 | -1,089016378 | -2,037551727 | 0,948535349 | |
| 244190_at | THAP5 | -1,089016378 | -2,037551727 | 0,948535349 | |
| 214612_x_at | MAGEA6 | -2,089474779 | -3,037500868 | 0,948026089 | |
| 223918_at | ACSL6 /// LOC10 | -2,089474779 | -3,037500868 | 0,948026089 | |
| 233465_at | - | -2,089474779 | -3,037500868 | 0,948026089 | |
| 1553970_s_at | CEL | 0,555999837 | -0,39061235 | 0,946612187 | |
| 223846_at | AZI2 | 0,03125738 | -0,912331589 | 0,943588969 | |
| 238191_at | - | 0,03125738 | -0,912331589 | 0,943588969 | |
| 206739_at | HOXC5 | 0,376672223 | -0,566887459 | 0,943559683 | |
| 1569479_at | ZNF718 | -0,79540287 | -1,738067356 | 0,942664486 | |
| 231102_at | CROT | -0,79540287 | -1,738067356 | 0,942664486 | |
| 1552724_at | RHOXF1 | 0,123193849 | -0,819238336 | 0,942432186 | |
| 210375_at | PTGER3 | 0,480753764 | -0,461594427 | 0,94234819 | |
| 1570090_at | - | -0,67289328 | -1,615132408 | 0,942239128 | |
| 216528_at | - | -0,67289328 | -1,615132408 | 0,942239128 | |
| 230956_at | C11orf92 | -0,67289328 | -1,615132408 | 0,942239128 | |
| 237028_at | ENO1-AS1 | -0,67289328 | -1,615132408 | 0,942239128 | |
| 242205_at | - | -0,67289328 | -1,615132408 | 0,942239128 | |
| 236115_at | HTR7P1 | -1,838812296 | -2,780313924 | 0,941501628 | |
| 239639_at | - | -1,838812296 | -2,780313924 | 0,941501628 | |
| 216228_s_at | WDHD1 | 2,466546243 | 1,525647381 | 0,940898863 | |
| 220549_at | FSBP /// RAD54E | 0,209623097 | -0,730778808 | 0,940401905 | |
| 1564473_at | ESCO2 | -1,518072576 | -2,456406426 | 0,93833385 | |
| 221590_s_at | ALDH6A1 | -1,518072576 | -2,456406426 | 0,93833385 | |
| 237248_at | PDE11A | -1,518072576 | -2,456406426 | 0,93833385 | |
| 237629_at | - | -1,518072576 | -2,456406426 | 0,93833385 | |
| 244255_at | LOC286114 | -1,518072576 | -2,456406426 | 0,93833385 | |
| 244735_at | CCDC54 | -1,518072576 | -2,456406426 | 0,93833385 | |
| 1553272_at | SLC36A1 | -0,325749314 | -1,264005341 | 0,938256027 | |
| 1561323_at | LOC339975 | -0,325749314 | -1,264005341 | 0,938256027 | |
| 201265_at | - | -0,325749314 | -1,264005341 | 0,938256027 | |
| 243166_at | SLC30A5 | 0,291166226 | -0,646746079 | 0,937912304 | |
| 207475_at | FABP2 | -1,470833215 | -2,408705138 | 0,937871922 | |
| 210034_s_at | RPL5 /// SNORD: | -0,112808516 | -1,050406615 | 0,937598099 | |
| 222091_at | HPCAL4 | -0,394749123 | -1,332017329 | 0,937268206 | |
| 219003_s_at | MANEA | 2,182996494 | 1,246149888 | 0,936846606 | |
| 1561443_at | - | -0,85085127 | -1,787089803 | 0,936238533 | |
| 1565805_at | - | -0,85085127 | -1,787089803 | 0,936238533 | |
| 1569894_at | PPP2R3C | -0,85085127 | -1,787089803 | 0,936238533 | |
| 240513_at | EIF3M | -0,85085127 | -1,787089803 | 0,936238533 | |
| 243062_at | FLCN | -0,85085127 | -1,787089803 | 0,936238533 | |
| 208080_at | AURKA | 0,715481152 | -0,220592223 | 0,936073375 | |
| 243201_at | RPL36A | -0,926850548 | -1,862647763 | 0,935797214 | |
| 215658_at | LOC100289255 | -1,399512875 | -2,335104118 | 0,935591243 | |
| 1553759_at | MCM9 | -1,639579028 | -2,575129729 | 0,935550701 | |
| 1564590_a_at | OFCC1 | -1,639579028 | -2,575129729 | 0,935550701 | |
| 233720_at | SORBS2 | -1,639579028 | -2,575129729 | 0,935550701 | |
| 235342_at | SPOCK3 | -1,639579028 | -2,575129729 | 0,935550701 | |
| 224997_x_at | H19 /// MIR675 | 0,255492549 | -0,679763839 | 0,935256389 | |
| 207556_s_at | DGKZ | 1,81142822 | 0,876248481 | 0,935179739 | |
| 1568934_at | CX3CR1 | -1,026571149 | -1,961547147 | 0,934975999 | |
| 210315_at | SYN2 | -1,026571149 | -1,961547147 | 0,934975999 | |
| 214551_s_at | CD7 | -1,026571149 | -1,961547147 | 0,934975999 | |
| 242042_s_at | HOXD-AS1 | -1,026571149 | -1,961547147 | 0,934975999 | |
| 1565627_a_at | - | -0,285837228 | -1,220210588 | 0,934373359 | |

| | | | | | |
|---|---|---|---|---|---|
| 205665_at | TSPAN9 | -0,285837228 | -1,220210588 | 0,934373359 | |
| 202650_s_at | KIAA0195 | 1,201332287 | 0,26733076 | 0,934001527 | |
| 1555868_at | LOC100507477 | -1,688127507 | -2,621331989 | 0,933204483 | |
| 232262_at | PIGL | 0,020670649 | -0,912331589 | 0,933002238 | |
| 1552448_a_at | C8orf12 | -1,353583928 | -2,286583451 | 0,932999523 | |
| 1561708_at | MB21D1 | -1,353583928 | -2,286583451 | 0,932999523 | |
| 1563553_at | - | -1,353583928 | -2,286583451 | 0,932999523 | |
| 227282_at | PCDH19 | -1,353583928 | -2,286583451 | 0,932999523 | |
| 229171_at | CENPBD1 | -1,353583928 | -2,286583451 | 0,932999523 | |
| 242005_at | LOC100506377 | -1,353583928 | -2,286583451 | 0,932999523 | |
| 1552279_a_at | SLC46A1 | 0,44160593 | -0,490927335 | 0,932533265 | |
| 1559496_at | PPA2 | -0,078265071 | -1,010192375 | 0,931927304 | |
| 222179_at | CDC5L | 0,541261317 | -0,39061235 | 0,931873667 | |
| 244427_at | KIF23 | 0,606430281 | -0,322870281 | 0,929300563 | |
| 228941_at | ALG10B | 0,882651021 | -0,046302147 | 0,928953168 | |
| 1554692_at | SLC23A2 | -1,763254337 | -2,690860744 | 0,927606407 | |
| 230931_at | PLG | -1,763254337 | -2,690860744 | 0,927606407 | |
| 1565338_x_at | DNAH6 | -1,2845686 | -2,211750327 | 0,927181727 | |
| 216140_at | - | -1,2845686 | -2,211750327 | 0,927181727 | |
| 205357_s_at | AGTR1 | 2,857110344 | 1,930732138 | 0,926378206 | |
| 1568978_s_at | C11orf21 | -0,496991414 | -1,423348341 | 0,926356928 | |
| 228493_at | LOC100506992 | -0,496991414 | -1,423348341 | 0,926356928 | |
| 229378_at | STOX1 | -0,496991414 | -1,423348341 | 0,926356928 | |
| 230790_x_at | - | -0,496991414 | -1,423348341 | 0,926356928 | |
| 1565628_at | - | -1,964176657 | -2,89013181 | 0,925955154 | |
| 205991_s_at | PRRX1 | -0,986356909 | -1,912089812 | 0,925732903 | |
| 244823_at | LOC100129034 | -0,986356909 | -1,912089812 | 0,925732903 | |
| 217267_s_at | RAB7A | -2,311268652 | -3,236070201 | 0,924801549 | |
| 212499_s_at | FCF1 /// MAPK1I | 4,697063183 | 3,7726819 | 0,924381282 | |
| 213087_s_at | EEF1D | 2,247975581 | 1,324440845 | 0,923534736 | |
| 219930_at | KLF8 | 0,30867765 | -0,614371577 | 0,923049226 | |
| 205786_s_at | ITGAM | -1,240169874 | -2,162639771 | 0,922469897 | |
| 220237_at | ATG3 | -1,240169874 | -2,162639771 | 0,922469897 | |
| 228631_s_at | ZNF688 | -1,240169874 | -2,162639771 | 0,922469897 | |
| 1567591_at | - | 0,503742507 | -0,41858459 | 0,922327097 | |
| 219042_at | LZTS1 | -0,741668563 | -1,663414495 | 0,921745932 | |
| 240094_at | - | -0,299034815 | -1,220210588 | 0,921175773 | |
| 241710_at | LOC728819 | 0,384950573 | -0,536087151 | 0,921037724 | |
| 232197_x_at | ARSB | 0,841135803 | -0,079522948 | 0,920658751 | |
| 228412_at | LOC643072 | -0,234278768 | -1,154897679 | 0,920618911 | |
| 231388_at | - | -0,234278768 | -1,154897679 | 0,920618911 | |
| 1562915_at | - | -0,089689931 | -1,010192375 | 0,920502444 | |
| 1555262_a_at | MAGI1 | -0,57445847 | -1,494668682 | 0,920210211 | |
| 243044_at | - | -0,57445847 | -1,494668682 | 0,920210211 | |
| 240421_x_at | SAV1 | 0,57058931 | -0,34958478 | 0,920174091 | |
| 237853_x_at | KRTAP10-12 | 0,661985843 | -0,258114234 | 0,920100076 | |
| 235095_at | CCDC64B | -0,622910612 | -1,541908042 | 0,91899743 | |
| 206233_at | B4GALT6 | 1,556829052 | 0,638150376 | 0,918678675 | |
| 214668_at | SPRYD7 | 0,351548291 | -0,566887459 | 0,91843575 | |
| 206117_at | TPM1 | 0,152582607 | -0,765504029 | 0,918086636 | |
| 203294_s_at | LMAN1 | 1,64218396 | 0,724100169 | 0,918083791 | |
| 234813_at | TMEM108 | -0,033456564 | -0,950686014 | 0,91722945 | |
| 223984_s_at | NUPL1 | 0,945567441 | 0,028364014 | 0,917203427 | |
| 222170_at | - | -0,196756757 | -1,112851844 | 0,916095087 | |
| 235264_at | HCFC2 | -0,196756757 | -1,112851844 | 0,916095087 | |
| 211822_s_at | NLRP1 | -0,946847173 | -1,862647763 | 0,915800589 | |

| | | | | | |
|---|---|---|---|---|---|
| 222076_at | HBEGF | -0,946847173 | -1,862647763 | 0,915800589 | |
| 223731_at | MYCBPAP | -0,946847173 | -1,862647763 | 0,915800589 | |
| 231166_at | GPR155 | 0,496120222 | -0,41858459 | 0,914704812 | |
| 1558755_x_at | ZNF763 | 1,58584542 | 0,67181667 | 0,91402875 | |
| 1554182_at | TRIM73 /// TRIM7 | -1,047650486 | -1,961547147 | 0,913896662 | |
| 1558825_s_at | LOC100653178 / | -1,047650486 | -1,961547147 | 0,913896662 | |
| 208464_at | GRIA4 | -1,047650486 | -1,961547147 | 0,913896662 | |
| 222053_at | TAF6L | -1,047650486 | -1,961547147 | 0,913896662 | |
| 233204_at | - | -1,047650486 | -1,961547147 | 0,913896662 | |
| 243721_at | C18orf61 | -1,047650486 | -1,961547147 | 0,913896662 | |
| 201163_s_at | IGFBP7 | 2,003776245 | 1,090553537 | 0,913222708 | |
| 220520_s_at | NUP62CL | -1,173767127 | -2,086984744 | 0,913217617 | |
| 204915_s_at | SOX11 | -1,543331864 | -2,456406426 | 0,913074562 | |
| 1569569_x_at | - | -1,591296941 | -2,503677622 | 0,912380681 | |
| 240775_at | - | -1,591296941 | -2,503677622 | 0,912380681 | |
| 241749_at | MURC | -1,591296941 | -2,503677622 | 0,912380681 | |
| 1554726_at | ZNF655 | 0,715481152 | -0,19610998 | 0,911591132 | |
| 238768_at | C2orf68 | 0,715481152 | -0,19610998 | 0,911591132 | |
| 234906_at | - | -0,512251684 | -1,423348341 | 0,911096657 | |
| 238814_at | SLC35C2 | -0,512251684 | -1,423348341 | 0,911096657 | |
| 242329_at | CREB5 /// LOC40 | -0,512251684 | -1,423348341 | 0,911096657 | |
| 1566518_at | - | -1,424023738 | -2,335104118 | 0,91108038 | |
| 217651_at | - | -1,424023738 | -2,335104118 | 0,91108038 | |
| 237327_at | APH1A | -1,424023738 | -2,335104118 | 0,91108038 | |
| 240817_at | - | -1,424023738 | -2,335104118 | 0,91108038 | |
| 1566580_at | - | -0,352951799 | -1,264005341 | 0,911053542 | |
| 207444_at | SLC22A13 | -0,352951799 | -1,264005341 | 0,911053542 | |
| 214907_at | CEACAM21 | -0,352951799 | -1,264005341 | 0,911053542 | |
| 219898_at | GPR85 | -0,352951799 | -1,264005341 | 0,911053542 | |
| 239990_at | - | -0,467091869 | -1,377419394 | 0,910327525 | |
| 213839_at | CLMN | -1,937711681 | -2,847001814 | 0,909290133 | |
| 241251_at | - | -1,937711681 | -2,847001814 | 0,909290133 | |
| 208279_s_at | CDRT1 | -0,423248115 | -1,332017329 | 0,908769214 | |
| 1569925_at | LOC100507386 | -0,706943342 | -1,615132408 | 0,908189066 | |
| 237762_at | - | -0,706943342 | -1,615132408 | 0,908189066 | |
| 241350_at | FBXL22 | -0,706943342 | -1,615132408 | 0,908189066 | |
| 209031_at | CADM1 | -1,71423189 | -2,621331989 | 0,9071001 | |
| 235368_at | ADAMTS5 | -1,71423189 | -2,621331989 | 0,9071001 | |
| 235616_at | TSHZ2 | -1,71423189 | -2,621331989 | 0,9071001 | |
| 1562610_at | - | -1,131062212 | -2,037551727 | 0,906489514 | |
| 203841_x_at | MAPRE3 | -1,131062212 | -2,037551727 | 0,906489514 | |
| 232891_at | SIRPD | -1,131062212 | -2,037551727 | 0,906489514 | |
| 234255_at | - | -1,131062212 | -2,037551727 | 0,906489514 | |
| 1557371_a_at | LOC158376 | -0,83191556 | -1,738067356 | 0,906151796 | |
| 222145_at | - | -0,83191556 | -1,738067356 | 0,906151796 | |
| 244069_at | - | -0,83191556 | -1,738067356 | 0,906151796 | |
| 230509_at | SNX22 | 0,934330293 | 0,028364014 | 0,905966278 | |
| 1557350_at | G3BP1 | -0,59053611 | -1,494668682 | 0,904132571 | |
| 216465_at | - | -0,59053611 | -1,494668682 | 0,904132571 | |
| 220027_s_at | RASIP1 | -0,59053611 | -1,494668682 | 0,904132571 | |
| 1563458_at | PARVA | -0,759588698 | -1,663414495 | 0,903825797 | |
| 220709_at | ZNF556 | -0,759588698 | -1,663414495 | 0,903825797 | |
| 227944_at | PTPN3 | -0,759588698 | -1,663414495 | 0,903825797 | |
| 242586_at | FSD1L | -0,759588698 | -1,663414495 | 0,903825797 | |
| 1554836_at | USP36 | -1,308181863 | -2,211750327 | 0,903568464 | |
| 241582_at | - | -1,308181863 | -2,211750327 | 0,903568464 | |

| | | | | | |
|---|---|---|---|---|---|
| 213436_at | CNR1 | 0,44160593 | -0,461594427 | 0,903200356 | |
| 219439_at | C1GALT1 | 2,637386731 | 1,73441697 | 0,902969761 | |
| 221406_s_at | MSH5 /// MSH5-S | 0,083054335 | -0,819238336 | 0,902292671 | |
| 1556678_a_at | - | -0,148196756 | -1,050406615 | 0,902209859 | |
| 204273_at | EDNRB | -0,148196756 | -1,050406615 | 0,902209859 | |
| 205693_at | TNNT3 | -0,148196756 | -1,050406615 | 0,902209859 | |
| 234052_at | - | -0,148196756 | -1,050406615 | 0,902209859 | |
| 235731_at | AIPL1 | -0,148196756 | -1,050406615 | 0,902209859 | |
| 235180_at | STYX | 0,57782909 | -0,322870281 | 0,900699372 | |
| 228944_at | - | 0,480753764 | -0,41858459 | 0,899338353 | |
| 1556350_a_at | EIF4A2 | -2,138804305 | -3,037500868 | 0,898696563 | |
| 1565861_at | - | -2,138804305 | -3,037500868 | 0,898696563 | |
| 203844_at | VHL | -2,138804305 | -3,037500868 | 0,898696563 | |
| 219780_at | ZNF771 | -2,138804305 | -3,037500868 | 0,898696563 | |
| 235976_at | SLITRK6 | -2,138804305 | -3,037500868 | 0,898696563 | |
| 1553918_at | LINC00479 | -0,888496123 | -1,787089803 | 0,898593681 | |
| 218247_s_at | MEX3C | 3,270743649 | 2,372786857 | 0,897956792 | |
| 215187_at | FLJ11292 | -2,504600601 | -3,402185466 | 0,897584865 | |
| 232287_at | PGBD3 | -0,112808516 | -1,010192375 | 0,897383859 | |
| 241936_x_at | - | -0,112808516 | -1,010192375 | 0,897383859 | |
| 1558333_at | C22orf15 | -0,366776884 | -1,264005341 | 0,897228457 | |
| 1570445_a_at | LOC643201 | -0,366776884 | -1,264005341 | 0,897228457 | |
| 208650_s_at | CD24 | -0,366776884 | -1,264005341 | 0,897228457 | |
| 227486_at | NT5E | -0,366776884 | -1,264005341 | 0,897228457 | |
| 221916_at | NEFL | -1,838812296 | -2,735862426 | 0,897050129 | |
| 241175_at | - | -1,838812296 | -2,735862426 | 0,897050129 | |
| 222896_at | TMEM38A | 0,359971644 | -0,536087151 | 0,896058795 | |
| 1559524_at | - | -0,259832361 | -1,154897679 | 0,895065317 | |
| 216882_s_at | NEBL | -0,055687482 | -0,950686014 | 0,894998533 | |
| 1568915_at | - | -0,325749314 | -1,220210588 | 0,894461274 | |
| 244696_at | - | -0,325749314 | -1,220210588 | 0,894461274 | |
| 242312_x_at | - | 0,503742507 | -0,39061235 | 0,894354857 | |
| 1555251_a_at | OTOF | -0,43775896 | -1,332017329 | 0,894258369 | |
| 1557666_s_at | AK8 | 0,57058931 | -0,322870281 | 0,893459591 | |
| 228925_at | ADAM1 | 0,57058931 | -0,322870281 | 0,893459591 | |
| 1553900_s_at | LOC100287541 / | 0,072842263 | -0,819238336 | 0,892080599 | |
| 1557100_s_at | HECTD1 | 0,072842263 | -0,819238336 | 0,892080599 | |
| 1565928_at | - | 0,072842263 | -0,819238336 | 0,892080599 | |
| 211768_at | LAT2 | 0,072842263 | -0,819238336 | 0,892080599 | |
| 1562921_at | - | -1,888254346 | -2,780313924 | 0,892059579 | |
| 202581_at | HSPA1A /// HSPA | 5,025974392 | 4,13442882 | 0,891545572 | |
| 210414_at | FLRT1 | -0,221656139 | -1,112851844 | 0,891195705 | |
| 221978_at | HLA-F | -0,221656139 | -1,112851844 | 0,891195705 | |
| 232406_at | - | -0,221656139 | -1,112851844 | 0,891195705 | |
| 228270_at | ZNF853 | 0,541261317 | -0,34958478 | 0,890846097 | |
| 230544_at | RPS6KA4 | -1,518072576 | -2,408705138 | 0,890632562 | |
| 236354_at | - | -1,518072576 | -2,408705138 | 0,890632562 | |
| 1558790_s_at | ZNF252P-AS1 | -1,196375121 | -2,086984744 | 0,890609622 | |
| 223827_at | TNFRSF19 | -1,196375121 | -2,086984744 | 0,890609622 | |
| 230096_at | - | -1,196375121 | -2,086984744 | 0,890609622 | |
| 230780_at | LOC730091 | -0,022466681 | -0,912331589 | 0,889864908 | |
| 202792_s_at | PPP6R2 | 0,209623097 | -0,679763839 | 0,889386937 | |
| 232702_at | RABGAP1L | 0,209623097 | -0,679763839 | 0,889386937 | |
| 236622_at | PIGM | 0,209623097 | -0,679763839 | 0,889386937 | |
| 1569190_at | SCLT1 | 0,917308734 | 0,028364014 | 0,88894472 | |
| 220991_s_at | RNF32 | 0,273439642 | -0,614371577 | 0,887811219 | |

| | | | | | |
|---|---|---|---|---|---|
| 227024_s_at | MRPL55 | 0,273439642 | -0,614371577 | 0,887811219 | |
| 1555894_s_at | MTSS1L | -0,85085127 | -1,738067356 | 0,887216086 | |
| 1556656_at | - | -0,85085127 | -1,738067356 | 0,887216086 | |
| 229280_s_at | LINC00340 | -0,85085127 | -1,738067356 | 0,887216086 | |
| 239016_at | - | -0,85085127 | -1,738067356 | 0,887216086 | |
| 1560384_a_at | LOC100506387 | -1,399512875 | -2,286583451 | 0,887070575 | |
| 218330_s_at | NAV2 | -1,399512875 | -2,286583451 | 0,887070575 | |
| 244222_at | - | 0,03125738 | -0,855751026 | 0,887008406 | |
| 1557542_at | - | -1,688127507 | -2,575129729 | 0,887002222 | |
| 1560358_at | - | -1,688127507 | -2,575129729 | 0,887002222 | |
| 204931_at | TCF21 | -1,688127507 | -2,575129729 | 0,887002222 | |
| 207273_at | - | -1,688127507 | -2,575129729 | 0,887002222 | |
| 207918_s_at | TSPY1 /// TSPY1 | -1,688127507 | -2,575129729 | 0,887002222 | |
| 230233_at | - | -1,688127507 | -2,575129729 | 0,887002222 | |
| 242950_x_at | - | -1,688127507 | -2,575129729 | 0,887002222 | |
| 1562853_x_at | - | 0,496120222 | -0,39061235 | 0,886732572 | |
| 1559214_at | - | -0,655928373 | -1,541908042 | 0,885979669 | |
| 233495_at | EXOSC3 | -0,655928373 | -1,541908042 | 0,885979669 | |
| 1554122_a_at | HSD17B12 | -1,026571149 | -1,912089812 | 0,885518663 | |
| 240376_s_at | - | -1,026571149 | -1,912089812 | 0,885518663 | |
| 241137_at | DPCR1 | -1,026571149 | -1,912089812 | 0,885518663 | |
| 1567222_x_at | ELOVL5 | 0,46522187 | -0,41858459 | 0,88380646 | |
| 238155_at | - | 0,46522187 | -0,41858459 | 0,88380646 | |
| 235303_at | TRMT10B | -1,964176657 | -2,847001814 | 0,882825158 | |
| 241255_at | - | -1,964176657 | -2,847001814 | 0,882825158 | |
| 208009_s_at | ARHGEF16 | 0,661985843 | -0,220592223 | 0,882578066 | |
| 220603_s_at | MCTP2 | 1,011212064 | 0,128747141 | 0,882464923 | |
| 228464_at | MIR3685 | 0,888485433 | 0,007421914 | 0,88106352 | |
| 213579_s_at | EP300 | 1,274448124 | 0,393761504 | 0,88068662 | |
| 208049_s_at | TACR1 | -0,496991414 | -1,377419394 | 0,88042798 | |
| 1556227_at | VCPIP1 | -0,543051993 | -1,423348341 | 0,880296348 | |
| 221303_at | PCDHB1 | -0,543051993 | -1,423348341 | 0,880296348 | |
| 227339_at | RGMB | -0,543051993 | -1,423348341 | 0,880296348 | |
| 232561_at | ZNF771 | -0,543051993 | -1,423348341 | 0,880296348 | |
| 236897_at | - | -0,543051993 | -1,423348341 | 0,880296348 | |
| 242992_at | ZNF551 | 0,20027154 | -0,679763839 | 0,880035379 | |
| 1556155_at | - | -0,033456564 | -0,912331589 | 0,878875025 | |
| 1565669_at | - | -0,033456564 | -0,912331589 | 0,878875025 | |
| 1570397_x_at | FAM66C | -1,812418401 | -2,690860744 | 0,878442343 | |
| 1556984_at | - | -0,172274514 | -1,050406615 | 0,878132101 | |
| 237712_at | LOC100507562 | -0,172274514 | -1,050406615 | 0,878132101 | |
| 1562413_at | LINC00167 | -1,2845686 | -2,162639771 | 0,878071171 | |
| 1562458_at | UBE2W | -1,2845686 | -2,162639771 | 0,878071171 | |
| 1565777_at | - | -1,2845686 | -2,162639771 | 0,878071171 | |
| 1566499_at | - | -1,2845686 | -2,162639771 | 0,878071171 | |
| 222886_at | NSUN3 | -1,2845686 | -2,162639771 | 0,878071171 | |
| 230896_at | BEND4 | 2,611163891 | 1,73441697 | 0,876746921 | |
| 226380_at | PTPN21 | 0,020670649 | -0,855751026 | 0,876421676 | |
| 231976_at | LINS | 0,020670649 | -0,855751026 | 0,876421676 | |
| 1563750_at | - | -2,01371626 | -2,89013181 | 0,87641555 | |
| 207029_at | KITLG | -2,01371626 | -2,89013181 | 0,87641555 | |
| 236792_at | CFL1 | -2,01371626 | -2,89013181 | 0,87641555 | |
| 1554972_at | - | -0,986356909 | -1,862647763 | 0,876290854 | |
| 1564749_at | LOC100289045 | -0,986356909 | -1,862647763 | 0,876290854 | |
| 1569004_at | LOC100505812 | -0,986356909 | -1,862647763 | 0,876290854 | |
| 207224_s_at | SIGLEC7 | -0,986356909 | -1,862647763 | 0,876290854 | |

| | | | | | |
|---|---|---|---|---|---|
| 210413_x_at | SERPINB3 /// SE | -0,986356909 | -1,862647763 | 0,876290854 | |
| 217659_at | - | -0,986356909 | -1,862647763 | 0,876290854 | |
| 239513_at | C22orf45 | -0,986356909 | -1,862647763 | 0,876290854 | |
| 240045_at | - | -0,986356909 | -1,862647763 | 0,876290854 | |
| 206466_at | ACSBG1 | -2,262747984 | -3,138626879 | 0,875878895 | |
| 235127_at | PMP2 | -2,262747984 | -3,138626879 | 0,875878895 | |
| 228000_at | ADC | 0,384950573 | -0,490927335 | 0,875877909 | |
| 232447_at | - | 0,882651021 | 0,007421914 | 0,875229107 | |
| 221596_s_at | RBM48 | 2,344516784 | 1,469325098 | 0,875191686 | |
| 223131_s_at | TRIM8 | 0,228146222 | -0,646746079 | 0,8748923 | |
| 1552950_at | C15orf26 | -0,136301282 | -1,010192375 | 0,873891093 | |
| 234048_s_at | EPG5 | -0,741668563 | -1,615132408 | 0,873463845 | |
| 242496_at | ART4 | -0,741668563 | -1,615132408 | 0,873463845 | |
| 243684_at | - | -0,741668563 | -1,615132408 | 0,873463845 | |
| 1569953_at | - | -1,089016378 | -1,961547147 | 0,87253077 | |
| 235760_at | NSD1 | -1,089016378 | -1,961547147 | 0,87253077 | |
| 240947_at | ANO6 | -1,089016378 | -1,961547147 | 0,87253077 | |
| 1563507_at | - | -0,622910612 | -1,494668682 | 0,871758069 | |
| 1566814_at | FGF22 | -0,622910612 | -1,494668682 | 0,871758069 | |
| 223499_at | C1QTNF5 /// MFI | 0,052199481 | -0,819238336 | 0,871437817 | |
| 238397_at | - | 0,052199481 | -0,819238336 | 0,871437817 | |
| 209101_at | CTGF | 1,326951551 | 0,456918297 | 0,870033253 | |
| 227132_at | ZNF706 | 0,928678725 | 0,059218869 | 0,869459856 | |
| 207698_at | C6orf123 | -0,394749123 | -1,264005341 | 0,869256217 | |
| 237089_at | - | -0,394749123 | -1,264005341 | 0,869256217 | |
| 244370_at | KIAA2022 | -0,394749123 | -1,264005341 | 0,869256217 | |
| 227014_at | ASPHD2 | -0,285837228 | -1,154897679 | 0,86906045 | |
| 242399_at | - | -0,285837228 | -1,154897679 | 0,86906045 | |
| 222898_s_at | DLL3 | -0,67289328 | -1,541908042 | 0,869014762 | |
| 224399_at | PDCD1LG2 | -0,67289328 | -1,541908042 | 0,869014762 | |
| 239839_at | ZNF555 | -0,67289328 | -1,541908042 | 0,869014762 | |
| 210189_at | HSPA1L | 0,648296226 | -0,220592223 | 0,868888449 | |
| 213462_at | NPAS2 | -0,79540287 | -1,663414495 | 0,868011625 | |
| 205557_at | BPI | -0,352951799 | -1,220210588 | 0,867258789 | |
| 205714_s_at | ZMYND10 | -0,352951799 | -1,220210588 | 0,867258789 | |
| 1564344_at | ATP9B | -1,543331864 | -2,408705138 | 0,865373273 | |
| 209672_s_at | MIOS | -1,543331864 | -2,408705138 | 0,865373273 | |
| 215323_at | LUZP2 | -1,543331864 | -2,408705138 | 0,865373273 | |
| 1554895_a_at | RHBDL2 | -0,512251684 | -1,377419394 | 0,86516771 | |
| 1566721_at | SVEP1 | -0,512251684 | -1,377419394 | 0,86516771 | |
| 202507_s_at | SNAP25 | -0,512251684 | -1,377419394 | 0,86516771 | |
| 213777_s_at | - | -0,512251684 | -1,377419394 | 0,86516771 | |
| 232797_at | - | -0,512251684 | -1,377419394 | 0,86516771 | |
| 1553142_at | LACC1 | -1,591296941 | -2,456406426 | 0,865109485 | |
| 1554765_a_at | LINC00301 | -1,591296941 | -2,456406426 | 0,865109485 | |
| 1558602_a_at | LSAMP-AS3 | -1,591296941 | -2,456406426 | 0,865109485 | |
| 214632_at | NRP2 | -1,591296941 | -2,456406426 | 0,865109485 | |
| 231491_at | LINC00113 | -1,591296941 | -2,456406426 | 0,865109485 | |
| 234760_at | - | -1,591296941 | -2,456406426 | 0,865109485 | |
| 1555314_at | WDR19 | -2,089474779 | -2,954576755 | 0,865101976 | |
| 202966_at | CAPN6 | -2,089474779 | -2,954576755 | 0,865101976 | |
| 241853_at | - | -2,089474779 | -2,954576755 | 0,865101976 | |
| 229317_at | KPNA5 | 1,865674241 | 1,000684521 | 0,86498972 | |
| 215562_at | TTC39A | -0,467091869 | -1,332017329 | 0,86492546 | |
| 214190_x_at | GGA2 | 1,114389003 | 0,249604176 | 0,864784828 | |
| 1564838_a_at | LOC151760 | -1,047650486 | -1,912089812 | 0,864439326 | |

| | | | | | |
|---|---|---|---|---|---|
| 1553523_at | NLRP14 | -1,470833215 | -2,335104118 | 0,864270902 | |
| 1569104_a_at | - | -1,470833215 | -2,335104118 | 0,864270902 | |
| 205925_s_at | RAB3B | -1,639579028 | -2,503677622 | 0,864098594 | |
| 208155_x_at | GAGE1 /// GAGE | -1,639579028 | -2,503677622 | 0,864098594 | |
| 236164_at | FLJ10038 | 0,133056859 | -0,730778808 | 0,863835667 | |
| 205867_at | PTPN11 | -1,173767127 | -2,037551727 | 0,8637846 | |
| 235807_at | - | -1,173767127 | -2,037551727 | 0,8637846 | |
| 238992_at | POLI | -1,173767127 | -2,037551727 | 0,8637846 | |
| 240655_at | - | -1,173767127 | -2,037551727 | 0,8637846 | |
| 241424_at | ELAVL4 | -1,173767127 | -2,037551727 | 0,8637846 | |
| 216715_at | - | 0,87091093 | 0,007421914 | 0,863489016 | |
| 1552521_a_at | TMEM74 | -1,424023738 | -2,286583451 | 0,862559712 | |
| 1554214_at | - | -1,424023738 | -2,286583451 | 0,862559712 | |
| 1561389_at | - | -1,424023738 | -2,286583451 | 0,862559712 | |
| 214105_at | SOCS3 | -1,424023738 | -2,286583451 | 0,862559712 | |
| 215174_at | FMO6P | -1,424023738 | -2,286583451 | 0,862559712 | |
| 216662_at | MYO7B | -1,424023738 | -2,286583451 | 0,862559712 | |
| 223981_at | NIN | -1,424023738 | -2,286583451 | 0,862559712 | |
| 222117_s_at | ADCK2 | -0,148196756 | -1,010192375 | 0,861995619 | |
| 235564_at | ZNF117 | -0,148196756 | -1,010192375 | 0,861995619 | |
| 210234_at | GRM4 | 0,57782909 | -0,283667828 | 0,861496918 | |
| 1553074_at | ASB11 | -1,71423189 | -2,575129729 | 0,860897839 | |
| 206864_s_at | HRK | -1,71423189 | -2,575129729 | 0,860897839 | |
| 234095_at | - | -1,71423189 | -2,575129729 | 0,860897839 | |
| 240354_at | C12orf54 | -1,71423189 | -2,575129729 | 0,860897839 | |
| 219964_at | ST7L | 0,246434561 | -0,614371577 | 0,860806138 | |
| 210745_at | ONECUT1 | -0,926850548 | -1,787089803 | 0,860239255 | |
| 231414_at | - | -0,926850548 | -1,787089803 | 0,860239255 | |
| 1566086_at | - | -1,353583928 | -2,211750327 | 0,858166399 | |
| 1567358_at | NAV2 | -1,353583928 | -2,211750327 | 0,858166399 | |
| 203724_s_at | RUFY3 | -1,353583928 | -2,211750327 | 0,858166399 | |
| 239126_at | CIRBP-AS1 | -1,763254337 | -2,621331989 | 0,858077652 | |
| 242189_at | - | -1,763254337 | -2,621331989 | 0,858077652 | |
| 243930_x_at | - | -1,763254337 | -2,621331989 | 0,858077652 | |
| 1554233_at | C1QTNF9 | -2,138804305 | -2,996335703 | 0,857531398 | |
| 1559991_s_at | - | -0,299034815 | -1,154897679 | 0,855862864 | |
| 216047_x_at | SEZ6L | -0,299034815 | -1,154897679 | 0,855862864 | |
| 238443_at | TFAM | -0,299034815 | -1,154897679 | 0,855862864 | |
| 1552696_at | NIPA1 | -0,759588698 | -1,615132408 | 0,85554371 | |
| 1570511_at | ARHGEF10L | -0,759588698 | -1,615132408 | 0,85554371 | |
| 205920_at | SLC6A6 | -0,759588698 | -1,615132408 | 0,85554371 | |
| 215176_x_at | LOC642838 | -0,759588698 | -1,615132408 | 0,85554371 | |
| 225590_at | SH3RF1 | -0,759588698 | -1,615132408 | 0,85554371 | |
| 1554212_s_at | KCNS2 | -0,00073712 | -0,855751026 | 0,855013906 | |
| 210511_s_at | INHBA | -1,308181863 | -2,162639771 | 0,854457908 | |
| 216986_s_at | IRF4 | -1,308181863 | -2,162639771 | 0,854457908 | |
| 220031_at | OTUD7B | -1,308181863 | -2,162639771 | 0,854457908 | |
| 229225_at | NRP2 | -1,308181863 | -2,162639771 | 0,854457908 | |
| 237843_at | - | -1,308181863 | -2,162639771 | 0,854457908 | |
| 1569487_at | - | 0,123193849 | -0,730778808 | 0,853972657 | |
| 204732_s_at | TRIM23 | 0,123193849 | -0,730778808 | 0,853972657 | |
| 217279_x_at | MMP14 | 0,123193849 | -0,730778808 | 0,853972657 | |
| 230856_at | - | -0,196756757 | -1,050406615 | 0,853649858 | |
| 1565823_at | - | -0,366776884 | -1,220210588 | 0,853433704 | |
| 208191_x_at | PSG4 | -0,366776884 | -1,220210588 | 0,853433704 | |
| 1554966_a_at | FILIP1L | -0,259832361 | -1,112851844 | 0,853019483 | |

| | | | | | |
|---|---|---|---|---|---|
| 219739_at | RNF186 | -0,259832361 | -1,112851844 | 0,853019483 | |
| 220210_at | CHRNA10 | -0,259832361 | -1,112851844 | 0,853019483 | |
| 1557523_at | ATP6AP1L /// FLJ | -1,838812296 | -2,690860744 | 0,852048448 | |
| 1562348_at | LOC400680 | -1,838812296 | -2,690860744 | 0,852048448 | |
| 1562633_at | RMST | -1,838812296 | -2,690860744 | 0,852048448 | |
| 212806_at | PRUNE2 | -1,838812296 | -2,690860744 | 0,852048448 | |
| 224401_s_at | FCRL4 | -1,838812296 | -2,690860744 | 0,852048448 | |
| 231204_at | C4orf21 | -1,838812296 | -2,690860744 | 0,852048448 | |
| 243328_at | - | -1,838812296 | -2,690860744 | 0,852048448 | |
| 1566980_at | - | 0,171847695 | -0,679763839 | 0,851611535 | |
| 242293_at | ING3 | 1,655931423 | 0,805216064 | 0,850715359 | |
| 1562657_a_at | C10orf90 | 0,03125738 | -0,819238336 | 0,850495716 | |
| 1555938_x_at | VIM | 0,592200562 | -0,258114234 | 0,850314795 | |
| 1553890_s_at | NTN5 | -0,888496123 | -1,738067356 | 0,849571233 | |
| 210202_s_at | BIN1 | -0,888496123 | -1,738067356 | 0,849571233 | |
| 233301_at | OXCT2 | -0,888496123 | -1,738067356 | 0,849571233 | |
| 221974_at | IPW /// LOC1005 | 3,062018438 | 2,212749286 | 0,849269152 | |
| 1561834_a_at | - | -0,57445847 | -1,423348341 | 0,848889871 | |
| 1562044_at | - | -0,57445847 | -1,423348341 | 0,848889871 | |
| 237835_at | - | -0,57445847 | -1,423348341 | 0,848889871 | |
| 222558_at | RPRD1A | 1,305306849 | 0,456918297 | 0,848388552 | |
| 243661_at | ZNF273 | 0,457392764 | -0,39061235 | 0,848005114 | |
| 220648_at | ADARB2 | -1,888254346 | -2,735862426 | 0,84760808 | |
| 225983_s_at | VWA1 | -1,888254346 | -2,735862426 | 0,84760808 | |
| 241063_at | - | -1,888254346 | -2,735862426 | 0,84760808 | |
| 1566402_at | SNORA68 | 0,20027154 | -0,646746079 | 0,847017618 | |
| 1557126_a_at | PLD1 | -1,240169874 | -2,086984744 | 0,846814869 | |
| 224147_at | PARPBP | -1,240169874 | -2,086984744 | 0,846814869 | |
| 227677_at | JAK3 | -1,240169874 | -2,086984744 | 0,846814869 | |
| 233956_at | - | -1,240169874 | -2,086984744 | 0,846814869 | |
| 237279_at | - | -1,240169874 | -2,086984744 | 0,846814869 | |
| 240070_at | TIGIT | -1,240169874 | -2,086984744 | 0,846814869 | |
| 203059_s_at | PAPSS2 | 0,496120222 | -0,34958478 | 0,845705002 | |
| 229804_x_at | CBWD1 /// CBWI | 2,943717636 | 2,098562556 | 0,84515508 | |
| 226014_at | EIF3F | 0,30867765 | -0,536087151 | 0,844764801 | |
| 221994_at | PDLIM5 | 0,425644434 | -0,41858459 | 0,844229023 | |
| 224223_s_at | PDE11A | -1,937711681 | -2,780313924 | 0,842602243 | |
| 240423_at | LOC441204 | -1,937711681 | -2,780313924 | 0,842602243 | |
| 1555695_a_at | CLRN1 | 0,228146222 | -0,614371577 | 0,842517798 | |
| 232563_at | ZNF684 | 0,228146222 | -0,614371577 | 0,842517798 | |
| 210177_at | TRIM15 | 0,351548291 | -0,490927335 | 0,842475626 | |
| 241036_at | - | 0,351548291 | -0,490927335 | 0,842475626 | |
| 225494_at | - | 2,882181479 | 2,040470494 | 0,841710984 | |
| 1559254_at | LINC00162 | -1,196375121 | -2,037551727 | 0,841176605 | |
| 1564598_a_at | VWA3B | -1,196375121 | -2,037551727 | 0,841176605 | |
| 238593_at | C11orf80 | -1,196375121 | -2,037551727 | 0,841176605 | |
| 206512_at | SRP19 /// ZRSR1 | -0,423248115 | -1,264005341 | 0,840757225 | |
| 238421_at | RC3H2 | -0,423248115 | -1,264005341 | 0,840757225 | |
| 213740_s_at | LOC100130348 | -0,946847173 | -1,787089803 | 0,84024263 | |
| 215193_x_at | HLA-DRB1 /// HL | -0,946847173 | -1,787089803 | 0,84024263 | |
| 220365_at | ALLC | -0,946847173 | -1,787089803 | 0,84024263 | |
| 234511_at | ANKRD60 | -0,946847173 | -1,787089803 | 0,84024263 | |
| 236235_at | - | -0,946847173 | -1,787089803 | 0,84024263 | |
| 242484_at | TTC9B | -0,946847173 | -1,787089803 | 0,84024263 | |
| 205212_s_at | ACAP1 | 0,020670649 | -0,819238336 | 0,839908986 | |
| 1553178_a_at | SSTR3 | 0,555999837 | -0,283667828 | 0,839667664 | |

| | | | | | |
|---|---|---|---|---|---|
| 228207_at | LOC100499489 | -0,655928373 | -1,494668682 | 0,838740309 | |
| 230090_at | GDNF | -0,655928373 | -1,494668682 | 0,838740309 | |
| 230877_at | IGHD | -0,655928373 | -1,494668682 | 0,838740309 | |
| 232895_s_at | - | -0,655928373 | -1,494668682 | 0,838740309 | |
| 242094_at | - | 1,496984567 | 0,658444279 | 0,838540288 | |
| 230649_at | C1orf86 | 0,072842263 | -0,765504029 | 0,838346292 | |
| 204051_s_at | SFRP4 | -0,172274514 | -1,010192375 | 0,837917861 | |
| 60084_at | CYLD | -1,725201958 | -2,563029977 | 0,837828019 | |
| 210359_at | MTSS1 | 0,641402394 | -0,19610998 | 0,837512374 | |
| 238348_x_at | - | -2,262747984 | -3,098858316 | 0,836110332 | |
| 1552932_at | NLRP6 | -1,026571149 | -1,862647763 | 0,836076614 | |
| 211407_at | NDUFB7 | -1,026571149 | -1,862647763 | 0,836076614 | |
| 241847_at | - | -1,026571149 | -1,862647763 | 0,836076614 | |
| 1563513_at | SYTL4 | -0,496991414 | -1,332017329 | 0,835025915 | |
| 214861_at | KDM4C | -0,706943342 | -1,541908042 | 0,8349647 | |
| 227888_at | RRBP1 | -0,706943342 | -1,541908042 | 0,8349647 | |
| 232511_at | - | -0,706943342 | -1,541908042 | 0,8349647 | |
| 220853_at | GTDC1 | -0,543051993 | -1,377419394 | 0,834367401 | |
| 238571_at | HOXA13 | -0,543051993 | -1,377419394 | 0,834367401 | |
| 205848_at | GAS2 | -0,078265071 | -0,912331589 | 0,834066517 | |
| 236448_at | UNC5A | -0,078265071 | -0,912331589 | 0,834066517 | |
| 241125_at | - | -0,078265071 | -0,912331589 | 0,834066517 | |
| 234109_x_at | ONECUT3 | 0,103263601 | -0,730778808 | 0,834042409 | |
| 236704_at | LOC100505971 | 0,103263601 | -0,730778808 | 0,834042409 | |
| 1565577_s_at | - | 0,841135803 | 0,007421914 | 0,833713889 | |
| 226289_at | CAPRIN1 | 1,638726528 | 0,805216064 | 0,833510464 | |
| 205157_s_at | JUP /// KRT17 | -2,01371626 | -2,847001814 | 0,833285554 | |
| 1559835_at | - | -0,59053611 | -1,423348341 | 0,832812231 | |
| 211657_at | CEACAM6 | -0,59053611 | -1,423348341 | 0,832812231 | |
| 217498_at | - | -0,59053611 | -1,423348341 | 0,832812231 | |
| 218901_at | PLSCR4 | -0,59053611 | -1,423348341 | 0,832812231 | |
| 1558522_at | - | 0,152582607 | -0,679763839 | 0,832346446 | |
| 205409_at | FOSL2 | 0,152582607 | -0,679763839 | 0,832346446 | |
| 222710_at | AMIGO3 /// GMP | 0,44160593 | -0,39061235 | 0,83221828 | |
| 1558807_at | ATAD2B | -0,83191556 | -1,663414495 | 0,831498935 | |
| 1565918_a_at | - | -0,83191556 | -1,663414495 | 0,831498935 | |
| 201438_at | COL6A3 | -0,83191556 | -1,663414495 | 0,831498935 | |
| 208733_at | RAB2A | -0,83191556 | -1,663414495 | 0,831498935 | |
| 213767_at | KSR1 | -0,83191556 | -1,663414495 | 0,831498935 | |
| 230801_at | RPRD1B | -0,83191556 | -1,663414495 | 0,831498935 | |
| 243977_at | LOC541472 | -0,83191556 | -1,663414495 | 0,831498935 | |
| 1570420_at | STXBP2 | 1,434549838 | 0,603679647 | 0,830870191 | |
| 225717_at | KIAA1715 | 1,224581536 | 0,393761504 | 0,830820031 | |
| 233191_at | RUFY2 | 0,859074518 | 0,028364014 | 0,830710504 | |
| 227175_at | MCL1 | 0,728550685 | -0,102100538 | 0,830651223 | |
| 1556113_at | DKFZp451A211 | -1,131062212 | -1,961547147 | 0,830484935 | |
| 1559171_at | MGC57346 | -1,131062212 | -1,961547147 | 0,830484935 | |
| 232595_at | - | -1,131062212 | -1,961547147 | 0,830484935 | |
| 210458_s_at | TANK | 0,480753764 | -0,34958478 | 0,830338544 | |
| 204602_at | DKK1 | 2,2992452 | 1,469325098 | 0,829920103 | |
| 204008_at | DNAL4 | 1,454352496 | 0,624460759 | 0,829891737 | |
| 224018_s_at | SCD5 | -0,325749314 | -1,154897679 | 0,829148365 | |
| 238045_at | TMEM65 | -0,221656139 | -1,050406615 | 0,828750476 | |
| 235452_at | - | 0,181384709 | -0,646746079 | 0,828130788 | |
| 234645_at | - | -2,311268652 | -3,138626879 | 0,827358227 | |
| 229455_at | - | 0,291166226 | -0,536087151 | 0,827253376 | |

| | | | | | |
|---|---|---|---|---|---|
| 1565641_at | C16orf45 | -0,285837228 | -1,112851844 | 0,827014616 | |
| 242753_x_at | AP1AR | -0,285837228 | -1,112851844 | 0,827014616 | |
| 243851_at | AURKAPS1 /// R/ | -0,285837228 | -1,112851844 | 0,827014616 | |
| 1556256_a_at | LOC100505908 | -2,063149277 | -2,89013181 | 0,826982533 | |
| 1552283_s_at | ZDHHC11 | -0,43775896 | -1,264005341 | 0,82624638 | |
| 1553686_at | C18orf25 | -0,43775896 | -1,264005341 | 0,82624638 | |
| 205362_s_at | PFDN4 | 1,04816921 | 0,222599095 | 0,825570116 | |
| 1566039_a_at | - | -0,394749123 | -1,220210588 | 0,825461464 | |
| 202908_at | WFS1 | -0,394749123 | -1,220210588 | 0,825461464 | |
| 219517_at | ELL3 | -0,394749123 | -1,220210588 | 0,825461464 | |
| 227311_at | SNX25 | -0,394749123 | -1,220210588 | 0,825461464 | |
| 239680_at | WDR76 | 0,541261317 | -0,283667828 | 0,824929144 | |
| 223656_s_at | TMEM234 | 1,177702228 | 0,352836757 | 0,82486547 | |
| 220945_x_at | MANSC1 | 0,882651021 | 0,059218869 | 0,823432152 | |
| 1552349_a_at | PRSS33 | -1,089016378 | -1,912089812 | 0,823073434 | |
| 1559102_at | - | -1,089016378 | -1,912089812 | 0,823073434 | |
| 205838_at | GYPA | -1,089016378 | -1,912089812 | 0,823073434 | |
| 214824_at | - | -1,089016378 | -1,912089812 | 0,823073434 | |
| 232319_at | - | -1,089016378 | -1,912089812 | 0,823073434 | |
| 206188_at | ZNF623 | 2,014487247 | 1,191491281 | 0,822995966 | |
| 242947_at | - | -0,089689931 | -0,912331589 | 0,822641658 | |
| 1563655_at | TNNT2 | 0,255492549 | -0,566887459 | 0,822380009 | |
| 236239_at | XPNPEP1 | 0,255492549 | -0,566887459 | 0,822380009 | |
| 214425_at | AMBP | -0,033456564 | -0,855751026 | 0,822294462 | |
| 244379_at | - | -0,033456564 | -0,855751026 | 0,822294462 | |
| 1552862_at | RUSC1-AS1 | -0,67289328 | -1,494668682 | 0,821775402 | |
| 210834_s_at | PTGER3 | -0,67289328 | -1,494668682 | 0,821775402 | |
| 214523_at | CEBPE | -0,67289328 | -1,494668682 | 0,821775402 | |
| 215850_s_at | NDUFA5 | -0,67289328 | -1,494668682 | 0,821775402 | |
| 239626_x_at | TM9SF3 | -0,67289328 | -1,494668682 | 0,821775402 | |
| 218352_at | RCBTB1 | 2,979690938 | 2,159161028 | 0,820529911 | |
| 1559031_at | EXOSC10 | -0,512251684 | -1,332017329 | 0,819765645 | |
| 238521_at | FGF12 | -0,512251684 | -1,332017329 | 0,819765645 | |
| 1561472_at | LOC728805 | -0,79540287 | -1,615132408 | 0,819729538 | |
| 213103_at | STARD13 | -0,79540287 | -1,615132408 | 0,819729538 | |
| 234124_at | - | -0,79540287 | -1,615132408 | 0,819729538 | |
| 230306_at | VPS26B | 1,129248302 | 0,310717132 | 0,81853117 | |
| 241982_at | - | -0,00073712 | -0,819238336 | 0,818501216 | |
| 201324_at | EMP1 | 0,533835206 | -0,283667828 | 0,817503034 | |
| 1561673_at | - | -1,591296941 | -2,408705138 | 0,817408196 | |
| 1567250_at | OR10A3 | -1,591296941 | -2,408705138 | 0,817408196 | |
| 204596_s_at | STC1 | -1,591296941 | -2,408705138 | 0,817408196 | |
| 211451_s_at | KCNJ4 | -1,591296941 | -2,408705138 | 0,817408196 | |
| 229557_at | MEG3 | -1,591296941 | -2,408705138 | 0,817408196 | |
| 230261_at | ST8SIA4 | -1,591296941 | -2,408705138 | 0,817408196 | |
| 229441_at | PRSS23 | 1,897847661 | 1,080561626 | 0,817286035 | |
| 1564306_at | - | -1,518072576 | -2,335104118 | 0,817031542 | |
| 222313_at | - | -1,518072576 | -2,335104118 | 0,817031542 | |
| 231080_at | CDAN1 | -1,518072576 | -2,335104118 | 0,817031542 | |
| 239209_at | - | -1,518072576 | -2,335104118 | 0,817031542 | |
| 244790_at | MTCP1 | -1,518072576 | -2,335104118 | 0,817031542 | |
| 1558549_s_at | VNN1 | -1,639579028 | -2,456406426 | 0,816827398 | |
| 1559313_at | - | -1,639579028 | -2,456406426 | 0,816827398 | |
| 1570229_at | - | -1,639579028 | -2,456406426 | 0,816827398 | |
| 216193_at | - | -1,639579028 | -2,456406426 | 0,816827398 | |
| 228462_at | IRX2 | -1,639579028 | -2,456406426 | 0,816827398 | |

| | | | | | |
|---|---|---|---|---|---|
| 238685_at | STXBP5-AS1 | -1,639579028 | -2,456406426 | 0,816827398 | |
| 37512_at | HSD17B6 | 0,376672238 | -0,439929237 | 0,816601475 | |
| 238728_at | - | -0,234278768 | -1,050406615 | 0,816127847 | |
| 243837_x_at | - | -0,234278768 | -1,050406615 | 0,816127847 | |
| 1558680_s_at | PDE1A | -1,470833215 | -2,286583451 | 0,815750235 | |
| 1562942_at | - | -1,470833215 | -2,286583451 | 0,815750235 | |
| 213352_at | TMCC1 | -1,470833215 | -2,286583451 | 0,815750235 | |
| 214947_at | FAM105A | -1,470833215 | -2,286583451 | 0,815750235 | |
| 216195_at | ANK2 | -1,470833215 | -2,286583451 | 0,815750235 | |
| 229218_at | COL1A2 | -1,470833215 | -2,286583451 | 0,815750235 | |
| 233036_at | - | -1,470833215 | -2,286583451 | 0,815750235 | |
| 241671_x_at | LINC00340 | -1,470833215 | -2,286583451 | 0,815750235 | |
| 235218_x_at | THAP6 | 0,97328325 | 0,157549243 | 0,815734007 | |
| 240351_at | - | -1,688127507 | -2,503677622 | 0,815550116 | |
| 1558754_at | ZNF763 | -1,047650486 | -1,862647763 | 0,814997277 | |
| 214079_at | DHRS2 | -1,047650486 | -1,862647763 | 0,814997277 | |
| 213984_at | PDS5A | 0,46522187 | -0,34958478 | 0,814806651 | |
| 206931_at | ZNF141 | -0,136301282 | -0,950686014 | 0,814384732 | |
| 216315_x_at | - | -0,136301282 | -0,950686014 | 0,814384732 | |
| 204653_at | TFAP2A | 0,083054335 | -0,730778808 | 0,813833143 | |
| 1562463_at | - | -0,299034815 | -1,112851844 | 0,813817029 | |
| 225867_at | VASN | -0,196756757 | -1,010192375 | 0,813435618 | |
| 243860_at | - | -0,196756757 | -1,010192375 | 0,813435618 | |
| 228975_at | SP6 | 0,246434561 | -0,566887459 | 0,81332202 | |
| 234360_at | - | 0,246434561 | -0,566887459 | 0,81332202 | |
| 238963_at | - | 0,246434561 | -0,566887459 | 0,81332202 | |
| 219208_at | FBXO11 | 1,269985355 | 0,456918297 | 0,813067057 | |
| 201117_s_at | CPE | 0,592200562 | -0,220592223 | 0,812792785 | |
| 1557924_s_at | ALPL | -0,85085127 | -1,663414495 | 0,812563225 | |
| 235015_at | ZDHHC9 | -0,85085127 | -1,663414495 | 0,812563225 | |
| 1562589_at | - | -1,399512875 | -2,211750327 | 0,812237452 | |
| 208359_s_at | KCNJ4 | -1,399512875 | -2,211750327 | 0,812237452 | |
| 211441_x_at | CYP3A43 | -1,399512875 | -2,211750327 | 0,812237452 | |
| 223826_s_at | KIAA1432 | -1,399512875 | -2,211750327 | 0,812237452 | |
| 236746_at | GALNT1 | -1,399512875 | -2,211750327 | 0,812237452 | |
| 242444_at | C1QTNF6 | -1,399512875 | -2,211750327 | 0,812237452 | |
| 1562771_at | - | -1,763254337 | -2,575129729 | 0,811875392 | |
| 214712_at | SNX29P2 | -1,763254337 | -2,575129729 | 0,811875392 | |
| 238408_at | - | -1,763254337 | -2,575129729 | 0,811875392 | |
| 241662_x_at | - | -1,763254337 | -2,575129729 | 0,811875392 | |
| 243128_at | ZNF175 | -1,763254337 | -2,575129729 | 0,811875392 | |
| 212210_at | INTS1 | -0,926850548 | -1,738067356 | 0,811216808 | |
| 215436_at | HSDL2 | -0,926850548 | -1,738067356 | 0,811216808 | |
| 217518_at | MYOF | -0,926850548 | -1,738067356 | 0,811216808 | |
| 229534_at | ACOT4 | -0,926850548 | -1,738067356 | 0,811216808 | |
| 61732_r_at | IFT74 | -0,333022385 | -1,143195856 | 0,810173472 | |
| 231852_at | ERI1 | 1,434549838 | 0,624460759 | 0,810089078 | |
| 227090_at | PHF21A | 0,613492853 | -0,19610998 | 0,809602833 | |
| 1570328_s_at | C20orf62 | 0,273439642 | -0,536087151 | 0,809526793 | |
| 217663_at | ZNF234 | 0,273439642 | -0,536087151 | 0,809526793 | |
| 228300_at | CCDC103 /// FAM | 0,273439642 | -0,536087151 | 0,809526793 | |
| 1560172_at | INTS10 | -1,353583928 | -2,162639771 | 0,809055843 | |
| 206448_at | ZNF365 | -1,353583928 | -2,162639771 | 0,809055843 | |
| 207725_at | POU4F2 | -1,353583928 | -2,162639771 | 0,809055843 | |
| 229506_at | PPM1L | -1,353583928 | -2,162639771 | 0,809055843 | |
| 232401_at | KCNS2 | -1,353583928 | -2,162639771 | 0,809055843 | |

| | | | | | |
|---|---|---|---|---|---|
| 235469_at | FAM133B /// FAM | -1,353583928 | -2,162639771 | 0,809055843 | |
| 240777_at | SYNE2 | -1,353583928 | -2,162639771 | 0,809055843 | |
| 223550_s_at | CA10 | -1,812418401 | -2,621331989 | 0,808913588 | |
| 224313_at | AASDHPPT | -1,812418401 | -2,621331989 | 0,808913588 | |
| 234745_at | - | -1,812418401 | -2,621331989 | 0,808913588 | |
| 240261_at | TOM1L1 | -1,812418401 | -2,621331989 | 0,808913588 | |
| 240404_at | - | -1,812418401 | -2,621331989 | 0,808913588 | |
| 216348_at | - | 3,849821959 | 3,042049843 | 0,807772115 | |
| 213601_at | ARHGAP19 /// SL | 0,457392764 | -0,34958478 | 0,806977545 | |
| 229417_at | - | 0,457392764 | -0,34958478 | 0,806977545 | |
| 212587_s_at | PTPRC | 1,805273023 | 1,000684521 | 0,804588502 | |
| 200796_s_at | MCL1 | 2,365585359 | 1,562009954 | 0,803575405 | |
| 1405_i_at | CCL5 | -0,768285559 | -1,57175725 | 0,803471691 | |
| 1564379_at | - | -0,57445847 | -1,377419394 | 0,802960924 | |
| 1570351_at | ADAMTS6 | -0,57445847 | -1,377419394 | 0,802960924 | |
| 211544_s_at | GHRHR | -0,57445847 | -1,377419394 | 0,802960924 | |
| 222187_x_at | G3BP1 | 1,139070151 | 0,336136178 | 0,802933973 | |
| 1552789_at | SEC62 | -1,888254346 | -2,690860744 | 0,802606398 | |
| 1563978_at | LOC728690 | -1,888254346 | -2,690860744 | 0,802606398 | |
| 238117_at | PPOX | -1,888254346 | -2,690860744 | 0,802606398 | |
| 1558841_at | CWC27 | -1,2845686 | -2,086984744 | 0,802416143 | |
| 215466_at | - | -1,2845686 | -2,086984744 | 0,802416143 | |
| 229386_at | ID4 | -1,2845686 | -2,086984744 | 0,802416143 | |
| 231175_at | BEND6 | -1,2845686 | -2,086984744 | 0,802416143 | |
| 237373_at | RAB20 | -1,2845686 | -2,086984744 | 0,802416143 | |
| 244369_at | C21orf59 | -1,2845686 | -2,086984744 | 0,802416143 | |
| 226675_s_at | LOC100507645 / | 3,352164686 | 2,549796788 | 0,802367898 | |
| 212827_at | IGHM | -0,352951799 | -1,154897679 | 0,80194588 | |
| 235446_at | - | -0,352951799 | -1,154897679 | 0,80194588 | |
| 238556_at | - | -0,352951799 | -1,154897679 | 0,80194588 | |
| 205753_at | CRP | -0,986356909 | -1,787089803 | 0,800732894 | |
| 208468_at | SOX21 | -0,986356909 | -1,787089803 | 0,800732894 | |
| 1565320_at | RBMY3AP | -2,089474779 | -2,89013181 | 0,800657031 | |
| 232119_at | SYNPO2 | -2,089474779 | -2,89013181 | 0,800657031 | |
| 239148_at | MARVELD3 | -2,089474779 | -2,89013181 | 0,800657031 | |
| 204299_at | SRSF10 | 3,984619786 | 3,184136378 | 0,800483409 | |
| 234730_s_at | RIPK4 | -0,622910612 | -1,423348341 | 0,800437729 | |
| 235031_at | - | -0,741668563 | -1,541908042 | 0,800239479 | |
| 235790_at | CTAGE5 | -0,741668563 | -1,541908042 | 0,800239479 | |
| 244249_at | - | -0,741668563 | -1,541908042 | 0,800239479 | |
| 238933_at | IRS1 | -0,112808516 | -0,912331589 | 0,799523072 | |
| 217465_at | NCKAP1 | 0,541261317 | -0,258114234 | 0,79937555 | |
| 232091_s_at | ZDHHC24 | 0,152582607 | -0,646746079 | 0,799328685 | |
| 235304_at | LOC100507486 | 2,491678455 | 1,692388143 | 0,799290313 | |
| 1554010_at | NDST1 | 0,95671774 | 0,157549243 | 0,799168497 | |
| 215226_at | EXPH5 | -1,937711681 | -2,735862426 | 0,798150744 | |
| 233945_at | UGGT2 | -1,937711681 | -2,735862426 | 0,798150744 | |
| 234131_at | - | -1,937711681 | -2,735862426 | 0,798150744 | |
| 1552608_at | WFDC11 | -1,240169874 | -2,037551727 | 0,797381852 | |
| 221394_at | TAAR2 | -1,240169874 | -2,037551727 | 0,797381852 | |
| 224340_at | - | -1,240169874 | -2,037551727 | 0,797381852 | |
| 229005_at | MCTP2 | -1,240169874 | -2,037551727 | 0,797381852 | |
| 237197_at | - | -1,240169874 | -2,037551727 | 0,797381852 | |
| 207185_at | SLC10A1 | -0,423248115 | -1,220210588 | 0,796962472 | |
| 209815_at | PTCH1 | -0,423248115 | -1,220210588 | 0,796962472 | |
| 233294_at | DENND2C | -0,423248115 | -1,220210588 | 0,796962472 | |

| | | | | | |
|---|---|---|---|---|---|
| 237421_at | - | -0,423248115 | -1,220210588 | 0,796962472 | |
| 240360_at | BRD4 | -0,423248115 | -1,220210588 | 0,796962472 | |
| 209277_at | TFPI2 | 5,483409962 | 4,686464402 | 0,79694556 | |
| 220749_at | C10orf68 | -0,467091869 | -1,264005341 | 0,796913472 | |
| 223686_at | TPK1 | -0,022466681 | -0,819238336 | 0,796771656 | |
| 231259_s_at | - | -0,022466681 | -0,819238336 | 0,796771656 | |
| 203507_at | CD68 | 0,334552598 | -0,461594427 | 0,796147025 | |
| 203784_s_at | DDX28 | 0,334552598 | -0,461594427 | 0,796147025 | |
| 1552467_at | DSCR10 | 0,181384709 | -0,614371577 | 0,795756286 | |
| 205859_at | LY86 | 0,181384709 | -0,614371577 | 0,795756286 | |
| 231255_at | MPRIP | 0,181384709 | -0,614371577 | 0,795756286 | |
| 1561760_s_at | - | -2,384869671 | -3,177802286 | 0,792932615 | |
| 1562338_at | 01.03.15 | -2,384869671 | -3,177802286 | 0,792932615 | |
| 221211_s_at | C21orf7 | 1,946200473 | 1,153866761 | 0,792333711 | |
| 236769_at | LOC158402 | 0,401366113 | -0,39061235 | 0,791978463 | |
| 1563540_at | - | -1,543331864 | -2,335104118 | 0,791772253 | |
| 210057_at | LOC100506060 / | -1,543331864 | -2,335104118 | 0,791772253 | |
| 220191_at | GKN1 | -1,543331864 | -2,335104118 | 0,791772253 | |
| 231526_at | - | -1,543331864 | -2,335104118 | 0,791772253 | |
| 237909_at | ADAM6 | -1,543331864 | -2,335104118 | 0,791772253 | |
| 238464_at | ANKRD36 /// ANK | -1,543331864 | -2,335104118 | 0,791772253 | |
| 239103_at | - | -1,543331864 | -2,335104118 | 0,791772253 | |
| 1553089_a_at | WFDC2 | -0,946847173 | -1,738067356 | 0,791220183 | |
| 1560855_at | - | -0,946847173 | -1,738067356 | 0,791220183 | |
| 1569979_at | HKR1 | -0,946847173 | -1,738067356 | 0,791220183 | |
| 202566_s_at | SVIL | -0,946847173 | -1,738067356 | 0,791220183 | |
| 209400_at | SLC12A4 | -0,946847173 | -1,738067356 | 0,791220183 | |
| 216549_s_at | TBC1D22B | -0,946847173 | -1,738067356 | 0,791220183 | |
| 224215_s_at | DLL1 | -0,946847173 | -1,738067356 | 0,791220183 | |
| 225280_x_at | ARSD | -0,946847173 | -1,738067356 | 0,791220183 | |
| 236665_at | CCDC18 | -0,946847173 | -1,738067356 | 0,791220183 | |
| 243487_at | AFF4 | -0,946847173 | -1,738067356 | 0,791220183 | |
| 1554346_at | GNB5 | 0,798390451 | 0,007421914 | 0,790968537 | |
| 1555205_at | - | -0,259832361 | -1,050406615 | 0,790574254 | |
| 1559316_at | - | -1,71423189 | -2,503677622 | 0,789445733 | |
| 207237_at | KCNA3 | -1,71423189 | -2,503677622 | 0,789445733 | |
| 231707_at | - | -0,543051993 | -1,332017329 | 0,788965336 | |
| 233336_at | - | -0,543051993 | -1,332017329 | 0,788965336 | |
| 212924_s_at | LSM4 | -0,221656139 | -1,010192375 | 0,788536237 | |
| 204296_at | DCTN1 | -0,366776884 | -1,154897679 | 0,788120795 | |
| 207900_at | CCL17 | -0,366776884 | -1,154897679 | 0,788120795 | |
| 218273_s_at | PDP1 | -0,366776884 | -1,154897679 | 0,788120795 | |
| 226595_at | SNX21 | -0,366776884 | -1,154897679 | 0,788120795 | |
| 228886_at | LRRC27 | -0,366776884 | -1,154897679 | 0,788120795 | |
| 230430_at | ENTPD2 | -0,366776884 | -1,154897679 | 0,788120795 | |
| 243368_at | - | -0,366776884 | -1,154897679 | 0,788120795 | |
| 217461_x_at | - | 1,305306849 | 0,51742585 | 0,787880999 | |
| 1553003_at | PKHD1 | -1,173767127 | -1,961547147 | 0,787780021 | |
| 222924_at | LOC100287789 / | -1,173767127 | -1,961547147 | 0,787780021 | |
| 234896_at | - | -1,173767127 | -1,961547147 | 0,787780021 | |
| 243260_x_at | XKR6 | -1,173767127 | -1,961547147 | 0,787780021 | |
| 243758_at | CCDC37 | -1,173767127 | -1,961547147 | 0,787780021 | |
| 1557190_at | - | -1,424023738 | -2,211750327 | 0,787726589 | |
| 222079_at | ERG | -1,424023738 | -2,211750327 | 0,787726589 | |
| 239927_at | LOC100506259 | -1,424023738 | -2,211750327 | 0,787726589 | |
| 1556182_x_at | ANKRD65 | -0,706943342 | -1,494668682 | 0,78772534 | |

| | | | | | |
|---|---|---|---|---|---|
| 209373_at | MALL | -0,706943342 | -1,494668682 | 0,78772534 | |
| 234024_at | CBLN4 | -0,706943342 | -1,494668682 | 0,78772534 | |
| 235157_at | - | -0,706943342 | -1,494668682 | 0,78772534 | |
| 242710_at | - | -2,311268652 | -3,098858316 | 0,787589665 | |
| 221480_at | HNRNPD | 3,628024221 | 2,840694075 | 0,787330146 | |
| 1559332_at | - | -0,59053611 | -1,377419394 | 0,786883284 | |
| 204686_at | IRS1 | -0,59053611 | -1,377419394 | 0,786883284 | |
| 214245_at | RPS14 | -0,59053611 | -1,377419394 | 0,786883284 | |
| 233412_x_at | - | -0,59053611 | -1,377419394 | 0,786883284 | |
| 235216_at | ESCO1 | -0,59053611 | -1,377419394 | 0,786883284 | |
| 239563_at | - | -0,59053611 | -1,377419394 | 0,786883284 | |
| 241037_at | - | -0,59053611 | -1,377419394 | 0,786883284 | |
| 242109_at | - | -0,59053611 | -1,377419394 | 0,786883284 | |
| 214628_at | NHLH1 | 0,171847695 | -0,614371577 | 0,786219272 | |
| 217326_x_at | IL23A /// TRBV19 | 0,171847695 | -0,614371577 | 0,786219272 | |
| 237731_at | LOC154092 | 0,171847695 | -0,614371577 | 0,786219272 | |
| 236213_at | - | 0,020670649 | -0,765504029 | 0,786174678 | |
| 238520_at | TRERF1 | 0,020670649 | -0,765504029 | 0,786174678 | |
| 242194_at | - | 0,020670649 | -0,765504029 | 0,786174678 | |
| 239289_x_at | FAN1 | 1,163336153 | 0,377530647 | 0,785805507 | |
| 244294_at | GTF2H5 | -0,033456564 | -0,819238336 | 0,785781772 | |
| 203299_s_at | AP1S2 | 5,352607629 | 4,567200037 | 0,785407592 | |
| 206047_at | GNB3 | 0,792179257 | 0,007421914 | 0,784757343 | |
| 235520_at | ZNF280C | 0,682279745 | -0,102100538 | 0,784380283 | |
| 219512_at | DSN1 | 2,589836943 | 1,805902329 | 0,783934614 | |
| 212637_s_at | WWP1 | 1,937784532 | 1,153866761 | 0,78391777 | |
| 1552438_a_at | ANKAR | -2,063149277 | -2,847001814 | 0,783852537 | |
| 217534_at | FAM49B | -2,063149277 | -2,847001814 | 0,783852537 | |
| 1553874_a_at | ZSCAN10 | -0,83191556 | -1,615132408 | 0,783216848 | |
| 1569523_a_at | - | -0,83191556 | -1,615132408 | 0,783216848 | |
| 217572_at | - | -0,83191556 | -1,615132408 | 0,783216848 | |
| 230145_at | DUS3L | -0,83191556 | -1,615132408 | 0,783216848 | |
| 235776_x_at | LINC00475 | -0,83191556 | -1,615132408 | 0,783216848 | |
| 236015_at | - | -0,83191556 | -1,615132408 | 0,783216848 | |
| 240230_s_at | AGAP9 | -0,83191556 | -1,615132408 | 0,783216848 | |
| 1555785_a_at | XRN1 | 0,052199481 | -0,730778808 | 0,782978288 | |
| 206596_s_at | NRL | 0,246434561 | -0,536087151 | 0,782521712 | |
| 1553446_at | C6orf183 | -1,838812296 | -2,621331989 | 0,782519693 | |
| 1557677_a_at | TRIML1 | -1,838812296 | -2,621331989 | 0,782519693 | |
| 232939_at | - | -1,838812296 | -2,621331989 | 0,782519693 | |
| 243143_at | FAM24A | -1,838812296 | -2,621331989 | 0,782519693 | |
| 204075_s_at | CEP104 | -0,43775896 | -1,220210588 | 0,782451627 | |
| 219518_s_at | ELL3 | -0,43775896 | -1,220210588 | 0,782451627 | |
| 220226_at | TRPM8 | -0,43775896 | -1,220210588 | 0,782451627 | |
| 224514_x_at | IL17RC | -0,43775896 | -1,220210588 | 0,782451627 | |
| 237075_at | LOC100506762 | -0,43775896 | -1,220210588 | 0,782451627 | |
| 208035_at | GRM6 | -0,759588698 | -1,541908042 | 0,782319344 | |
| 208484_at | HIST1H1A | -0,759588698 | -1,541908042 | 0,782319344 | |
| 220061_at | ACSM5 | -0,759588698 | -1,541908042 | 0,782319344 | |
| 1553960_at | SNX21 | -1,131062212 | -1,912089812 | 0,781027599 | |
| 1560550_at | - | -1,131062212 | -1,912089812 | 0,781027599 | |
| 213716_s_at | SECTM1 | -1,131062212 | -1,912089812 | 0,781027599 | |
| 223136_at | AIG1 | -1,131062212 | -1,912089812 | 0,781027599 | |
| 44790_s_at | KIAA0226L | -1,590652973 | -2,371435322 | 0,780782349 | |
| 219589_s_at | TMEM143 | 0,457392764 | -0,322870281 | 0,780263046 | |
| 209145_s_at | CBFA2T2 | 0,133056859 | -0,646746079 | 0,779802937 | |

| | | | | | |
|---|---|---|---|---|---|
| 216665_s_at | TTTY2 /// TTTY2 | -2,359436556 | -3,138626879 | 0,779190323 | |
| 238497_at | TMEM136 | 0,317354302 | -0,461594427 | 0,778948728 | |
| 1553818_x_at | LOC100653171 / | -1,308181863 | -2,086984744 | 0,778802881 | |
| 204884_s_at | HUS1 | -1,308181863 | -2,086984744 | 0,778802881 | |
| 207235_s_at | GRM5 | -1,308181863 | -2,086984744 | 0,778802881 | |
| 209955_s_at | FAP | -1,308181863 | -2,086984744 | 0,778802881 | |
| 237058_x_at | SLC6A13 | -1,308181863 | -2,086984744 | 0,778802881 | |
| 241359_at | TLCD2 | -1,308181863 | -2,086984744 | 0,778802881 | |
| 224618_at | PTBP3 | 2,259277178 | 1,480766833 | 0,778510345 | |
| 1562487_at | - | -0,172274514 | -0,950686014 | 0,778411501 | |
| 208373_s_at | P2RY6 | -0,078265071 | -0,855751026 | 0,777485955 | |
| 211867_s_at | PCDHA10 | -0,078265071 | -0,855751026 | 0,777485955 | |
| 227283_at | EFR3B | -0,078265071 | -0,855751026 | 0,777485955 | |
| 230519_at | FAM124A | -0,078265071 | -0,855751026 | 0,777485955 | |
| 202695_s_at | STK17A | 0,934330293 | 0,157549243 | 0,77678105 | |
| 1565836_at | - | -0,234278768 | -1,010192375 | 0,775913608 | |
| 231023_at | CARS2 | -0,234278768 | -1,010192375 | 0,775913608 | |
| 236046_at | FLJ44896 | -0,234278768 | -1,010192375 | 0,775913608 | |
| 1557599_a_at | - | -2,262747984 | -3,037500868 | 0,774752884 | |
| 234205_at | - | -2,262747984 | -3,037500868 | 0,774752884 | |
| 216218_s_at | PLCL2 | 0,57782909 | -0,19610998 | 0,773939071 | |
| 1564931_at | - | -1,089016378 | -1,862647763 | 0,773631385 | |
| 205894_at | ARSE | -1,089016378 | -1,862647763 | 0,773631385 | |
| 222833_at | LPCAT2 | -1,089016378 | -1,862647763 | 0,773631385 | |
| 230705_at | SLC2A5 | -1,089016378 | -1,862647763 | 0,773631385 | |
| 204500_s_at | AGTPBP1 | 1,649074067 | 0,876248481 | 0,772825586 | |
| 1554264_at | CKAP2 | -1,964176657 | -2,735862426 | 0,771685769 | |
| 203477_at | COL15A1 | -1,964176657 | -2,735862426 | 0,771685769 | |
| 216146_at | - | -1,964176657 | -2,735862426 | 0,771685769 | |
| 227644_at | RIMS4 | -1,964176657 | -2,735862426 | 0,771685769 | |
| 244831_at | - | -1,964176657 | -2,735862426 | 0,771685769 | |
| 205862_at | GREB1 | 1,442503549 | 0,67181667 | 0,770686879 | |
| 213206_at | GOSR2 | 0,351548291 | -0,41858459 | 0,770132881 | |
| 235300_x_at | RCHY1 | 0,798390451 | 0,028364014 | 0,770026437 | |
| 244052_at | CBR4 | 1,799091453 | 1,029536893 | 0,76955456 | |
| 222974_at | IL22 | -2,504600601 | -3,273861381 | 0,76926078 | |
| 207739_s_at | GAGE1 /// GAGE | -1,639579028 | -2,408705138 | 0,769126109 | |
| 228938_at | MBP | -1,639579028 | -2,408705138 | 0,769126109 | |
| 1557314_at | DPY19L2P3 | -1,518072576 | -2,286583451 | 0,768510875 | |
| 1561607_at | - | -1,518072576 | -2,286583451 | 0,768510875 | |
| 214270_s_at | MAPRE3 | -1,518072576 | -2,286583451 | 0,768510875 | |
| 241263_at | - | -1,518072576 | -2,286583451 | 0,768510875 | |
| 1552779_a_at | SLC44A5 | -1,688127507 | -2,456406426 | 0,768278919 | |
| 215617_at | SPATS2L | -1,688127507 | -2,456406426 | 0,768278919 | |
| 241018_at | TMEM59 | -1,688127507 | -2,456406426 | 0,768278919 | |
| 233254_x_at | PTEN | 0,417596971 | -0,34958478 | 0,767181751 | |
| 209477_at | EMD | 2,752227654 | 1,98530622 | 0,766921434 | |
| 208031_s_at | RFX2 | 0,57058931 | -0,19610998 | 0,76669929 | |
| 1555502_at | NPSA | -2,187914861 | -2,954576755 | 0,766661894 | |
| 1562938_at | - | -2,187914861 | -2,954576755 | 0,766661894 | |
| 215282_at | ANAPC13 | -2,187914861 | -2,954576755 | 0,766661894 | |
| 219466_s_at | APOA2 | -2,187914861 | -2,954576755 | 0,766661894 | |
| 242571_at | REPS2 | -2,187914861 | -2,954576755 | 0,766661894 | |
| 206482_at | PTK6 | -2,01371626 | -2,780313924 | 0,766597664 | |
| 227081_at | DNALI1 | -2,01371626 | -2,780313924 | 0,766597664 | |
| 233565_s_at | FKBP1A-SDCBP | 1,119359127 | 0,352836757 | 0,76652237 | |

| | | | | | |
|---|---|---|---|---|---|
| 1554704_at | ATP8B3 | 1,534677255 | 0,768343791 | 0,766333465 | |
| 226960_at | CXCL17 | -0,089689931 | -0,855751026 | 0,766061095 | |
| 1555347_at | PDXDC1 | -1,196375121 | -1,961547147 | 0,765172026 | |
| 1559871_s_at | LOC100129129 | -1,196375121 | -1,961547147 | 0,765172026 | |
| 207217_s_at | NOX1 | -1,196375121 | -1,961547147 | 0,765172026 | |
| 208497_x_at | NEUROG1 | -1,196375121 | -1,961547147 | 0,765172026 | |
| 215495_s_at | SAMD4A | -1,196375121 | -1,961547147 | 0,765172026 | |
| 220136_s_at | CRYBA2 | -1,196375121 | -1,961547147 | 0,765172026 | |
| 220461_at | PCNXL2 | -1,196375121 | -1,961547147 | 0,765172026 | |
| 226559_at | IER5L | -1,196375121 | -1,961547147 | 0,765172026 | |
| 232298_at | LOC401093 | -1,196375121 | -1,961547147 | 0,765172026 | |
| 232781_at | LHX4 | -1,196375121 | -1,961547147 | 0,765172026 | |
| 235780_at | PRKACB | -1,196375121 | -1,961547147 | 0,765172026 | |
| 236387_at | LOC100129961 | -1,196375121 | -1,961547147 | 0,765172026 | |
| 239282_at | CCDC41 | -1,196375121 | -1,961547147 | 0,765172026 | |
| 242076_at | - | -1,196375121 | -1,961547147 | 0,765172026 | |
| 212207_at | MED13L | 1,90937145 | 1,144305291 | 0,765066159 | |
| 224843_at | SLAIN2 | -0,00073712 | -0,765504029 | 0,764766909 | |
| 1554129_a_at | ADIG | -0,285837228 | -1,050406615 | 0,764569387 | |
| 1560765_a_at | - | -0,285837228 | -1,050406615 | 0,764569387 | |
| 230001_at | 09.03.15 | -0,285837228 | -1,050406615 | 0,764569387 | |
| 204210_s_at | PCYT1A | 0,44160593 | -0,322870281 | 0,764476211 | |
| 216961_s_at | RPAIN | 0,480753764 | -0,283667828 | 0,764421591 | |
| 206891_at | ACTN3 | 0,273439642 | -0,490927335 | 0,764366977 | |
| 221941_at | PAOX | 0,273439642 | -0,490927335 | 0,764366977 | |
| 242590_at | - | 0,273439642 | -0,490927335 | 0,764366977 | |
| 1552773_at | CLEC4D | -0,85085127 | -1,615132408 | 0,764281138 | |
| 210739_x_at | SLC4A4 | -0,85085127 | -1,615132408 | 0,764281138 | |
| 233472_at | TCP11L1 | -0,85085127 | -1,615132408 | 0,764281138 | |
| 233507_at | - | -0,85085127 | -1,615132408 | 0,764281138 | |
| 241066_at | ZNF449 | -0,85085127 | -1,615132408 | 0,764281138 | |
| 243432_at | CHL1-AS2 | 0,228146222 | -0,536087151 | 0,764233372 | |
| 214158_s_at | PRDM10 | -0,148196756 | -0,912331589 | 0,764134832 | |
| 1564539_at | LOC647323 | -1,399512875 | -2,162639771 | 0,763126896 | |
| 235722_at | SYNJ2BP | -1,399512875 | -2,162639771 | 0,763126896 | |
| 243302_at | - | -1,399512875 | -2,162639771 | 0,763126896 | |
| 204230_s_at | SLC17A7 | 0,083054335 | -0,679763839 | 0,762818174 | |
| 1553652_a_at | C18orf54 | -1,812418401 | -2,575129729 | 0,762711328 | |
| 1568981_at | - | -1,812418401 | -2,575129729 | 0,762711328 | |
| 242835_s_at | LOC728730 | -1,812418401 | -2,575129729 | 0,762711328 | |
| 243052_at | MOB3C | 0,03125738 | -0,730778808 | 0,762036188 | |
| 215997_s_at | CUL4B | 2,305800198 | 1,543943227 | 0,76185697 | |
| 232684_at | ZNF503-AS1 | 0,682279745 | -0,079522948 | 0,761802694 | |
| 207103_at | KCND2 | -1,026571149 | -1,787089803 | 0,760518654 | |
| 210659_at | CMKLR1 | -1,026571149 | -1,787089803 | 0,760518654 | |
| 226182_s_at | WISP3 | -1,026571149 | -1,787089803 | 0,760518654 | |
| 233220_at | GRIN3A | -1,026571149 | -1,787089803 | 0,760518654 | |
| 233431_x_at | - | -1,026571149 | -1,787089803 | 0,760518654 | |
| 237021_at | LOC144486 | -1,026571149 | -1,787089803 | 0,760518654 | |
| 238185_at | - | -1,026571149 | -1,787089803 | 0,760518654 | |
| 1558181_at | - | -0,394749123 | -1,154897679 | 0,760148555 | |
| 235164_at | ZNF25 | -0,394749123 | -1,154897679 | 0,760148555 | |
| 244299_at | - | -0,394749123 | -1,154897679 | 0,760148555 | |
| 1570631_at | - | -0,352951799 | -1,112851844 | 0,759900045 | |
| 235460_at | SNX22 | -0,352951799 | -1,112851844 | 0,759900045 | |
| 216771_at | - | -2,236765673 | -2,996335703 | 0,75957003 | |

| | | | | | |
|---|---|---|---|---|---|
| 229138_at | PARP11 | 0,962260738 | 0,204310755 | 0,757949983 | |
| 232419_at | TMEM132A | 0,934330293 | 0,176436073 | 0,757894219 | |
| 216869_at | PDE1C | -0,57445847 | -1,332017329 | 0,757558859 | |
| 231909_x_at | ODF2L | -0,57445847 | -1,332017329 | 0,757558859 | |
| 1563898_at | - | -2,089474779 | -2,847001814 | 0,757527035 | |
| 1564253_at | LOC285766 | -2,089474779 | -2,847001814 | 0,757527035 | |
| 215118_s_at | IGHA1 | -2,089474779 | -2,847001814 | 0,757527035 | |
| 242161_at | - | -2,089474779 | -2,847001814 | 0,757527035 | |
| 208116_s_at | MAN1A1 | 1,274448124 | 0,51742585 | 0,757022274 | |
| 214348_at | TACR2 | -0,622910612 | -1,377419394 | 0,754508782 | |
| 243291_at | - | -0,622910612 | -1,377419394 | 0,754508782 | |
| 226226_at | TMEM45B | -0,196756757 | -0,950686014 | 0,753929257 | |
| 239881_at | - | -0,196756757 | -0,950686014 | 0,753929257 | |
| 237866_at | PID1 | -2,384869671 | -3,138626879 | 0,753757208 | |
| 1562373_at | - | -1,937711681 | -2,690860744 | 0,753149063 | |
| 1564295_at | FLJ25917 | -1,937711681 | -2,690860744 | 0,753149063 | |
| 1566947_at | - | -1,937711681 | -2,690860744 | 0,753149063 | |
| 226765_at | SPTBN1 | -1,937711681 | -2,690860744 | 0,753149063 | |
| 230231_at | FGF14 | -1,937711681 | -2,690860744 | 0,753149063 | |
| 233023_at | - | -1,937711681 | -2,690860744 | 0,753149063 | |
| 243558_at | - | -1,937711681 | -2,690860744 | 0,753149063 | |
| 232407_at | MUC17 | -0,467091869 | -1,220210588 | 0,753118719 | |
| 240329_at | PTP4A1 | -0,467091869 | -1,220210588 | 0,753118719 | |
| 228945_s_at | SLC39A8 | -0,741668563 | -1,494668682 | 0,753000119 | |
| 229224_x_at | LOC643085 | -0,741668563 | -1,494668682 | 0,753000119 | |
| 236268_at | SEC22C | -0,741668563 | -1,494668682 | 0,753000119 | |
| 240136_at | - | -0,741668563 | -1,494668682 | 0,753000119 | |
| 1557252_at | - | -1,2845686 | -2,037551727 | 0,752983126 | |
| 200606_at | DSP | -1,2845686 | -2,037551727 | 0,752983126 | |
| 204762_s_at | GNAO1 | -1,2845686 | -2,037551727 | 0,752983126 | |
| 223505_s_at | DNAJC27 | -1,2845686 | -2,037551727 | 0,752983126 | |
| 226931_at | TMTC1 | -1,2845686 | -2,037551727 | 0,752983126 | |
| 227535_at | C15orf24 | 0,291166226 | -0,461594427 | 0,752760652 | |
| 89977_at | ACSM5 | -0,945897783 | -1,698550632 | 0,752652849 | |
| 1557613_at | FLJ39534 | 0,072842263 | -0,679763839 | 0,752606102 | |
| 202525_at | PRSS8 | -0,512251684 | -1,264005341 | 0,751753656 | |
| 220557_s_at | PACS1 | -0,512251684 | -1,264005341 | 0,751753656 | |
| 237765_at | SLC25A47 | -0,512251684 | -1,264005341 | 0,751753656 | |
| 241148_at | ACTG2 | -0,512251684 | -1,264005341 | 0,751753656 | |
| 205732_s_at | NCOA2 | -0,986356909 | -1,738067356 | 0,751710447 | |
| 207301_at | EFNA5 | -0,986356909 | -1,738067356 | 0,751710447 | |
| 208008_at | TBC1D29 | -0,986356909 | -1,738067356 | 0,751710447 | |
| 210744_s_at | IL5RA | -0,986356909 | -1,738067356 | 0,751710447 | |
| 238144_s_at | - | -0,986356909 | -1,738067356 | 0,751710447 | |
| 240699_at | SEC14L3 | -0,986356909 | -1,738067356 | 0,751710447 | |
| 244161_at | - | -0,986356909 | -1,738067356 | 0,751710447 | |
| 1553156_at | LHX4 | 0,020670649 | -0,730778808 | 0,751449457 | |
| 219812_at | PVRIG | 0,020670649 | -0,730778808 | 0,751449457 | |
| 229508_at | U2AF2 | 0,020670649 | -0,730778808 | 0,751449457 | |
| 237463_at | ZFPM1 | 0,020670649 | -0,730778808 | 0,751449457 | |
| 241501_at | - | 0,020670649 | -0,730778808 | 0,751449457 | |
| 212310_at | MIA3 | -0,299034815 | -1,050406615 | 0,7513718 | |
| 231620_at | LOC100507190 | -0,299034815 | -1,050406615 | 0,7513718 | |
| 1569275_at | - | -2,138804305 | -2,89013181 | 0,751327505 | |
| 210800_at | TIMM8A | -2,138804305 | -2,89013181 | 0,751327505 | |
| 234826_at | - | -2,138804305 | -2,89013181 | 0,751327505 | |

| | | | | | |
|---|---|---|---|---|---|
| 240821_at | - | -2,138804305 | -2,89013181 | 0,751327505 | |
| 243478_at | CHST2 | -2,138804305 | -2,89013181 | 0,751327505 | |
| 1553120_at | CLSPN | 0,779676066 | 0,028364014 | 0,751312051 | |
| 1568834_s_at | CCDC90B | 0,401366113 | -0,34958478 | 0,750950893 | |
| 218833_at | ZAK | 0,401366113 | -0,34958478 | 0,750950893 | |
| 1555655_at | OR10A4 | -0,67289328 | -1,423348341 | 0,750455062 | |
| 215336_at | AKAP11 | -0,67289328 | -1,423348341 | 0,750455062 | |
| 223822_at | SUSD4 | -0,67289328 | -1,423348341 | 0,750455062 | |
| 234188_at | - | -0,67289328 | -1,423348341 | 0,750455062 | |
| 243575_at | MAST4 | -0,259832361 | -1,010192375 | 0,750360014 | |
| 202903_at | LSM5 | 1,614289682 | 0,864649967 | 0,749639714 | |
| 204221_x_at | GLIPR1 | 0,82905153 | 0,079428135 | 0,749623395 | |
| 231920_s_at | CSNK1G1 | 0,702292136 | -0,046302147 | 0,748594283 | |
| 221578_at | RASSF4 | 0,181384709 | -0,566887459 | 0,748272169 | |
| 202720_at | TES | 1,534677255 | 0,786897721 | 0,747779534 | |
| 227004_at | - | 1,534677255 | 0,786897721 | 0,747779534 | |
| 228812_at | - | 1,515954005 | 0,768343791 | 0,747610215 | |
| 1555964_at | ARL17A /// ARL1 | -0,79540287 | -1,541908042 | 0,746505172 | |
| 1556564_at | HHIPL1 | -0,79540287 | -1,541908042 | 0,746505172 | |
| 1561785_at | MUC4 | -0,79540287 | -1,541908042 | 0,746505172 | |
| 221724_s_at | CLEC4A | -0,79540287 | -1,541908042 | 0,746505172 | |
| 234888_at | CACHD1 | -0,79540287 | -1,541908042 | 0,746505172 | |
| 240020_at | - | -0,366776884 | -1,112851844 | 0,74607496 | |
| 1554820_at | AGBL3 | -2,43257096 | -3,177802286 | 0,745231326 | |
| 236175_at | TRIM55 | -2,43257096 | -3,177802286 | 0,745231326 | |
| 1557529_at | C12orf51 | -1,591296941 | -2,335104118 | 0,743807176 | |
| 208849_at | - | -1,591296941 | -2,335104118 | 0,743807176 | |
| 221901_at | KIAA1644 | -1,591296941 | -2,335104118 | 0,743807176 | |
| 225242_s_at | CCDC80 | -1,591296941 | -2,335104118 | 0,743807176 | |
| 230508_at | DKK3 | -1,591296941 | -2,335104118 | 0,743807176 | |
| 231899_at | ZC3H12C | -1,591296941 | -2,335104118 | 0,743807176 | |
| 232570_s_at | ADAM33 | -1,591296941 | -2,335104118 | 0,743807176 | |
| 241900_at | - | -1,591296941 | -2,335104118 | 0,743807176 | |
| 214220_s_at | ALMS1 | 2,305800198 | 1,562009954 | 0,743790244 | |
| 228907_at | LOC100507110 | 0,641402394 | -0,102100538 | 0,743502932 | |
| 222626_at | RBM26 | 1,578645857 | 0,835239052 | 0,743406804 | |
| 1554582_a_at | METTL20 | -1,543331864 | -2,286583451 | 0,743251586 | |
| 1566517_at | - | -1,543331864 | -2,286583451 | 0,743251586 | |
| 206372_at | MYF6 | -1,543331864 | -2,286583451 | 0,743251586 | |
| 210133_at | CCL11 | -1,543331864 | -2,286583451 | 0,743251586 | |
| 214533_at | CMA1 | -1,543331864 | -2,286583451 | 0,743251586 | |
| 217086_at | CHRNB3 | -1,543331864 | -2,286583451 | 0,743251586 | |
| 225911_at | NPNT | -1,543331864 | -2,286583451 | 0,743251586 | |
| 234058_at | - | -1,543331864 | -2,286583451 | 0,743251586 | |
| 240216_at | - | -1,543331864 | -2,286583451 | 0,743251586 | |
| 243597_at | FANCB | -0,022466681 | -0,765504029 | 0,743037348 | |
| 1554203_at | GRIK1-AS1 | -1,71423189 | -2,456406426 | 0,742174536 | |
| 219234_x_at | SCRN3 | -1,71423189 | -2,456406426 | 0,742174536 | |
| 220479_at | CPS1-IT1 | -1,71423189 | -2,456406426 | 0,742174536 | |
| 220872_at | PRO2964 | -1,71423189 | -2,456406426 | 0,742174536 | |
| 244259_s_at | - | -1,71423189 | -2,456406426 | 0,742174536 | |
| 202751_at | TFIP11 | 1,446464021 | 0,704715219 | 0,741748801 | |
| 225941_at | EIF4E3 | -0,59053611 | -1,332017329 | 0,741481219 | |
| 233181_at | - | -0,59053611 | -1,332017329 | 0,741481219 | |
| 236054_at | - | -0,59053611 | -1,332017329 | 0,741481219 | |
| 243180_at | - | -0,59053611 | -1,332017329 | 0,741481219 | |

| | | | | | |
|---|---|---|---|---|---|
| 243809_at | HELQ | -0,59053611 | -1,332017329 | 0,741481219 | |
| 1560901_at | - | 0,457392764 | -0,283667828 | 0,741060592 | |
| 224030_s_at | FAM115A | 0,457392764 | -0,283667828 | 0,741060592 | |
| 217300_at | - | -0,078265071 | -0,819238336 | 0,740973265 | |
| 230224_at | ZCCHC18 | -0,078265071 | -0,819238336 | 0,740973265 | |
| 243740_at | - | -0,078265071 | -0,819238336 | 0,740973265 | |
| 1556619_at | SHISA9 | -1,470833215 | -2,211750327 | 0,740917112 | |
| 204797_s_at | EML1 | -1,470833215 | -2,211750327 | 0,740917112 | |
| 206341_at | IL2RA | -1,470833215 | -2,211750327 | 0,740917112 | |
| 217048_at | - | -1,470833215 | -2,211750327 | 0,740917112 | |
| 227253_at | CP | -1,470833215 | -2,211750327 | 0,740917112 | |
| 229409_s_at | - | -1,470833215 | -2,211750327 | 0,740917112 | |
| 230360_at | GLDN | -1,470833215 | -2,211750327 | 0,740917112 | |
| 230635_at | - | -1,470833215 | -2,211750327 | 0,740917112 | |
| 232533_at | METTL8 | -1,470833215 | -2,211750327 | 0,740917112 | |
| 239297_at | KIAA1456 | -1,470833215 | -2,211750327 | 0,740917112 | |
| 242529_x_at | - | -1,470833215 | -2,211750327 | 0,740917112 | |
| 214917_at | PRKAA1 | 0,417596971 | -0,322870281 | 0,740467252 | |
| 228289_at | BRD7 | 0,417596971 | -0,322870281 | 0,740467252 | |
| 230986_at | KLF8 | 0,417596971 | -0,322870281 | 0,740467252 | |
| 209355_s_at | PPAP2B | -1,763254337 | -2,503677622 | 0,740423285 | |
| 215478_at | RIMS2 | -1,763254337 | -2,503677622 | 0,740423285 | |
| 201269_s_at | NUDCD3 | -0,172274514 | -0,912331589 | 0,740057075 | |
| 213148_at | C2orf72 | -0,172274514 | -0,912331589 | 0,740057075 | |
| 1557257_at | BCL10 | -1,047650486 | -1,787089803 | 0,739439318 | |
| 1561453_at | - | -1,047650486 | -1,787089803 | 0,739439318 | |
| 214021_x_at | ITGB5 | -1,047650486 | -1,787089803 | 0,739439318 | |
| 224389_s_at | COL25A1 | -1,047650486 | -1,787089803 | 0,739439318 | |
| 1568820_a_at | BEND6 | -2,359436556 | -3,098858316 | 0,73942176 | |
| 243800_at | NR1H4 | -2,359436556 | -3,098858316 | 0,73942176 | |
| 207503_at | TCP10 | 0,171847695 | -0,566887459 | 0,738735155 | |
| 1557598_at | - | -1,424023738 | -2,162639771 | 0,738616033 | |
| 216527_at | - | -1,424023738 | -2,162639771 | 0,738616033 | |
| 230000_at | RNF213 | -1,424023738 | -2,162639771 | 0,738616033 | |
| 234509_at | - | -1,424023738 | -2,162639771 | 0,738616033 | |
| 242825_at | LPPR5 | -1,424023738 | -2,162639771 | 0,738616033 | |
| 242943_at | ST8SIA4 | -1,424023738 | -2,162639771 | 0,738616033 | |
| 244836_at | - | -1,424023738 | -2,162639771 | 0,738616033 | |
| 1562240_at | PLXNA4 | -1,173767127 | -1,912089812 | 0,738322685 | |
| 1564317_at | - | -1,173767127 | -1,912089812 | 0,738322685 | |
| 206921_at | GLE1 | -1,173767127 | -1,912089812 | 0,738322685 | |
| 209758_s_at | MFAP5 | -1,173767127 | -1,912089812 | 0,738322685 | |
| 211884_s_at | CIITA | -1,173767127 | -1,912089812 | 0,738322685 | |
| 238466_at | - | -1,173767127 | -1,912089812 | 0,738322685 | |
| 242192_at | - | -1,173767127 | -1,912089812 | 0,738322685 | |
| 230026_at | MRPL43 | 1,292162421 | 0,553993624 | 0,738168797 | |
| 230930_at | LOC338620 | 0,57782909 | -0,160136748 | 0,737965839 | |
| 205156_s_at | ASIC1 | 0,123193849 | -0,614371577 | 0,737565426 | |
| 1561225_at | LOC338579 | -0,926850548 | -1,663414495 | 0,736563946 | |
| 1561409_at | - | -0,926850548 | -1,663414495 | 0,736563946 | |
| 215272_at | OGG1 | -0,926850548 | -1,663414495 | 0,736563946 | |
| 223597_at | ITLN1 | -0,926850548 | -1,663414495 | 0,736563946 | |
| 228048_at | ZNF503-AS2 | -0,926850548 | -1,663414495 | 0,736563946 | |
| 233987_at | TFAP2D | -0,926850548 | -1,663414495 | 0,736563946 | |
| 236583_at | GIMAP1 | -0,926850548 | -1,663414495 | 0,736563946 | |
| 237226_at | - | -0,926850548 | -1,663414495 | 0,736563946 | |

| | | | | | |
|---|---|---|---|---|---|
| 244217_at | - | -0,926850548 | -1,663414495 | 0,736563946 | |
| 1553749_at | FAM76B | 2,708372067 | 1,971855016 | 0,736517051 | |
| 202793_at | LPCAT3 | 1,360921491 | 0,624460759 | 0,736460732 | |
| 216984_x_at | CKAP2 /// IGLC1 | 0,20027154 | -0,536087151 | 0,73635869 | |
| 1552563_a_at | - | -1,838812296 | -2,575129729 | 0,736317433 | |
| 1559161_at | - | -1,838812296 | -2,575129729 | 0,736317433 | |
| 1563860_at | LOC400965 | -1,838812296 | -2,575129729 | 0,736317433 | |
| 207286_at | CEP135 | -1,838812296 | -2,575129729 | 0,736317433 | |
| 221576_at | LOC100653010 | -1,838812296 | -2,575129729 | 0,736317433 | |
| 238429_at | TMEM71 | -1,838812296 | -2,575129729 | 0,736317433 | |
| 235864_at | ATP6V1D | 0,317354302 | -0,41858459 | 0,735938891 | |
| 227802_at | RUFY3 | 1,153678675 | 0,417770464 | 0,735908212 | |
| 1552572_a_at | MIPOL1 | -0,759588698 | -1,494668682 | 0,735079984 | |
| 1568847_at | - | -0,759588698 | -1,494668682 | 0,735079984 | |
| 236055_at | DQX1 | -0,759588698 | -1,494668682 | 0,735079984 | |
| 238335_at | DNAJC21 | -0,759588698 | -1,494668682 | 0,735079984 | |
| 241368_at | PLIN5 | -0,759588698 | -1,494668682 | 0,735079984 | |
| 241730_at | MYNN | -0,759588698 | -1,494668682 | 0,735079984 | |
| 242596_at | - | -0,759588698 | -1,494668682 | 0,735079984 | |
| 202704_at | TOB1 | 3,245961699 | 2,511262629 | 0,734699069 | |
| 234661_at | CCDC57 | 0,384950573 | -0,34958478 | 0,734535354 | |
| 222309_at | LOC100506935 | 2,845159691 | 2,110887591 | 0,7342721 | |
| 1557506_a_at | - | -2,262747984 | -2,996335703 | 0,733587719 | |
| 1553336_a_at | MIER3 | -1,353583928 | -2,086984744 | 0,733400816 | |
| 211526_s_at | RTEL1 /// RTEL1 | -1,353583928 | -2,086984744 | 0,733400816 | |
| 238367_s_at | C1orf228 | -1,353583928 | -2,086984744 | 0,733400816 | |
| 243217_at | - | -1,353583928 | -2,086984744 | 0,733400816 | |
| 204176_at | KLHL20 | 2,027765132 | 1,294497133 | 0,733267999 | |
| 1562475_at | DKFZp686O1327 | -1,888254346 | -2,621331989 | 0,733077644 | |
| 1564220_a_at | LOC100506465 | -1,888254346 | -2,621331989 | 0,733077644 | |
| 1568899_at | - | -1,888254346 | -2,621331989 | 0,733077644 | |
| 1569759_at | - | -1,888254346 | -2,621331989 | 0,733077644 | |
| 233462_at | TBC1D28 | -1,888254346 | -2,621331989 | 0,733077644 | |
| 234100_at | - | -1,888254346 | -2,621331989 | 0,733077644 | |
| 234409_at | ZNF354C | -1,888254346 | -2,621331989 | 0,733077644 | |
| 237413_at | MAPK10 | -1,888254346 | -2,621331989 | 0,733077644 | |
| 237941_at | - | -1,888254346 | -2,621331989 | 0,733077644 | |
| 213072_at | CYHR1 | 0,760715727 | 0,028364014 | 0,732351712 | |
| 224619_at | CASC4 | 4,646745381 | 3,914426433 | 0,732318948 | |
| 1555573_at | TTC40 | -0,033456564 | -0,765504029 | 0,732047465 | |
| 237431_at | LOC100506882 | -0,033456564 | -0,765504029 | 0,732047465 | |
| 213688_at | CALM1 /// CALM2 | 0,841135803 | 0,109221392 | 0,731914411 | |
| 222007_s_at | FKBP8 | -0,423248115 | -1,154897679 | 0,731649563 | |
| 238506_at | LRRC58 | -0,423248115 | -1,154897679 | 0,731649563 | |
| 1553553_at | TAS2R39 | -1,131062212 | -1,862647763 | 0,73158555 | |
| 1560977_a_at | BCL2L13 | -1,131062212 | -1,862647763 | 0,73158555 | |
| 1564010_at | CAST | -1,131062212 | -1,862647763 | 0,73158555 | |
| 203619_s_at | FAIM2 | -1,131062212 | -1,862647763 | 0,73158555 | |
| 214374_s_at | PPFIBP1 | -1,131062212 | -1,862647763 | 0,73158555 | |
| 221656_s_at | ARHGEF10L | -1,131062212 | -1,862647763 | 0,73158555 | |
| 234876_at | - | -1,131062212 | -1,862647763 | 0,73158555 | |
| 1557206_at | C17orf104 | -2,504600601 | -3,236070201 | 0,7314696 | |
| 243942_at | - | -2,504600601 | -3,236070201 | 0,7314696 | |
| 243965_at | - | -2,504600601 | -3,236070201 | 0,7314696 | |
| 224431_s_at | SUV420H2 | 1,187200746 | 0,456918297 | 0,730282449 | |
| 1556978_a_at | TAF8 | -0,00073712 | -0,730778808 | 0,730041688 | |

| | | | | | |
|---|---|---|---|---|---|
| 218085_at | CHMP5 | 4,482391326 | 3,75237043 | 0,730020896 | |
| 1554606_at | CEP120 | 0,083054335 | -0,646746079 | 0,729800413 | |
| 207366_at | KCNS1 | 0,083054335 | -0,646746079 | 0,729800413 | |
| 225531_at | CABLES1 | 0,083054335 | -0,646746079 | 0,729800413 | |
| 220784_s_at | UTS2 | 0,627515113 | -0,102100538 | 0,729615651 | |
| 226469_s_at | GGT7 | -0,089689931 | -0,819238336 | 0,729548405 | |
| 229055_at | GPR68 | -0,089689931 | -0,819238336 | 0,729548405 | |
| 234895_at | CTLA4 | -0,089689931 | -0,819238336 | 0,729548405 | |
| 229712_at | SNAPC3 | -1,308181863 | -2,037551727 | 0,729369864 | |
| 234362_s_at | CTLA4 | -1,308181863 | -2,037551727 | 0,729369864 | |
| 236563_at | RD3 | -1,308181863 | -2,037551727 | 0,729369864 | |
| 239439_at | AFF4 | -1,308181863 | -2,037551727 | 0,729369864 | |
| 244869_at | - | -1,308181863 | -2,037551727 | 0,729369864 | |
| 223764_x_at | NIPSNAP3B | -0,221656139 | -0,950686014 | 0,729029876 | |
| 220102_at | FOXL2 | 0,592200562 | -0,136643983 | 0,728844544 | |
| 235294_at | SIKE1 | 0,592200562 | -0,136643983 | 0,728844544 | |
| 226944_at | HTRA3 | 0,682279745 | -0,046302147 | 0,728581892 | |
| 1554341_a_at | HELQ | 0,735041319 | 0,007421914 | 0,727619406 | |
| 1563719_a_at | - | -1,964176657 | -2,690860744 | 0,726684087 | |
| 1566127_at | - | -1,964176657 | -2,690860744 | 0,726684087 | |
| 204010_s_at | KRAS | -1,964176657 | -2,690860744 | 0,726684087 | |
| 206799_at | SCGB1D2 | -1,964176657 | -2,690860744 | 0,726684087 | |
| 215895_x_at | PLIN2 | -1,964176657 | -2,690860744 | 0,726684087 | |
| 220842_at | AHI1 | -1,964176657 | -2,690860744 | 0,726684087 | |
| 228353_x_at | UBASH3B | -1,964176657 | -2,690860744 | 0,726684087 | |
| 236503_at | - | -1,964176657 | -2,690860744 | 0,726684087 | |
| 240957_at | - | -1,964176657 | -2,690860744 | 0,726684087 | |
| 244423_at | - | -1,964176657 | -2,690860744 | 0,726684087 | |
| 215880_at | NAGLU | -0,888496123 | -1,615132408 | 0,726636285 | |
| 228952_at | ENPP1 | -0,888496123 | -1,615132408 | 0,726636285 | |
| 231923_at | TMEM150C | -0,888496123 | -1,615132408 | 0,726636285 | |
| 236895_at | - | -0,888496123 | -1,615132408 | 0,726636285 | |
| 244034_at | - | -0,888496123 | -1,615132408 | 0,726636285 | |
| 242610_x_at | - | 0,376672223 | -0,34958478 | 0,726257004 | |
| 1552721_a_at | FGF1 | -2,311268652 | -3,037500868 | 0,726232217 | |
| 234866_s_at | ZNRD1-AS1 | -2,311268652 | -3,037500868 | 0,726232217 | |
| 215127_s_at | RBMS1 | 4,029534322 | 3,303845028 | 0,725689294 | |
| 238929_at | SRSF8 | 1,512180019 | 0,786897721 | 0,725282297 | |
| 1559585_at | DDX60L | 0,44160593 | -0,283667828 | 0,725273757 | |
| 204182_s_at | ZBTB43 | 0,882651021 | 0,157549243 | 0,725101778 | |
| 226730_s_at | USP37 | 2,410880861 | 1,685812099 | 0,725068762 | |
| 224831_at | CPEB4 | 2,261526912 | 1,536652708 | 0,724874204 | |
| 204637_at | CGA | -0,325749314 | -1,050406615 | 0,724657301 | |
| 242147_at | - | -0,325749314 | -1,050406615 | 0,724657301 | |
| 204333_s_at | AGA | 0,401366113 | -0,322870281 | 0,724236394 | |
| 239026_x_at | AGAP3 | 2,303618506 | 1,579853228 | 0,723765278 | |
| 236165_at | MSL3 | 0,97328325 | 0,249604176 | 0,723679074 | |
| 1558723_at | LOC284014 | -0,496991414 | -1,220210588 | 0,723219174 | |
| 219167_at | RASL12 | -0,496991414 | -1,220210588 | 0,723219174 | |
| 222941_at | USP46 | -0,496991414 | -1,220210588 | 0,723219174 | |
| 228575_at | IL20RB | -0,496991414 | -1,220210588 | 0,723219174 | |
| 240202_x_at | PFKP | -0,496991414 | -1,220210588 | 0,723219174 | |
| 244744_at | LOC100130502 | -0,496991414 | -1,220210588 | 0,723219174 | |
| 204006_s_at | FCGR3A /// FCG | -2,01371626 | -2,735862426 | 0,722146165 | |
| 239152_at | - | -2,01371626 | -2,735862426 | 0,722146165 | |
| 214385_s_at | MUC5AC | -0,655928373 | -1,377419394 | 0,721491021 | |

| | | | | | |
|---|---|---|---|---|---|
| 215715_at | SLC6A2 | -0,655928373 | -1,377419394 | 0,721491021 | |
| 216679_at | DDR1-AS1 | -0,655928373 | -1,377419394 | 0,721491021 | |
| 227714_s_at | - | -0,655928373 | -1,377419394 | 0,721491021 | |
| 231201_at | PTGER1 | -0,655928373 | -1,377419394 | 0,721491021 | |
| 1553726_s_at | C6orf170 | -1,240169874 | -1,961547147 | 0,721377273 | |
| 1560856_at | ARHGAP26-AS1 | -1,240169874 | -1,961547147 | 0,721377273 | |
| 214997_at | SCAI | -1,240169874 | -1,961547147 | 0,721377273 | |
| 228100_at | C1orf88 | -1,240169874 | -1,961547147 | 0,721377273 | |
| 229008_at | WDR60 | -1,240169874 | -1,961547147 | 0,721377273 | |
| 233590_at | LOC100506457 | -1,240169874 | -1,961547147 | 0,721377273 | |
| 243634_at | - | -1,240169874 | -1,961547147 | 0,721377273 | |
| 244850_at | - | -1,240169874 | -1,961547147 | 0,721377273 | |
| 207423_s_at | ADAM20 | -0,543051993 | -1,264005341 | 0,720953347 | |
| 210310_s_at | FGF5 | -0,543051993 | -1,264005341 | 0,720953347 | |
| 234099_at | - | -0,543051993 | -1,264005341 | 0,720953347 | |
| 242242_at | USP6 | -0,543051993 | -1,264005341 | 0,720953347 | |
| 229970_at | KBTBD7 | 0,641402394 | -0,079522948 | 0,720925343 | |
| 203845_at | KAT2B | 1,177702228 | 0,456918297 | 0,72078393 | |
| 1556911_at | - | -1,688127507 | -2,408705138 | 0,720577631 | |
| 1559145_at | RNF144A-AS1 | -1,688127507 | -2,408705138 | 0,720577631 | |
| 1562038_at | - | -1,688127507 | -2,408705138 | 0,720577631 | |
| 1570385_at | - | -1,688127507 | -2,408705138 | 0,720577631 | |
| 230569_at | KIAA1430 | -1,688127507 | -2,408705138 | 0,720577631 | |
| 233020_at | - | -1,688127507 | -2,408705138 | 0,720577631 | |
| 240782_at | - | -1,688127507 | -2,408705138 | 0,720577631 | |
| 235029_at | GINS4 | 1,64218396 | 0,921731975 | 0,720451985 | |
| 64900_at | TMEM231 | 1,986199457 | 1,266124526 | 0,720074931 | |
| 210883_x_at | EFNB3 | 0,82905153 | 0,109221392 | 0,719830138 | |
| 216852_x_at | IGLJ3 | 0,152582607 | -0,566887459 | 0,719470066 | |
| 1562731_s_at | MDS2 | -0,136301282 | -0,855751026 | 0,719449744 | |
| 204774_at | EVI2A | -0,136301282 | -0,855751026 | 0,719449744 | |
| 220198_s_at | EIF5A2 | -0,136301282 | -0,855751026 | 0,719449744 | |
| 237979_at | - | 0,228146222 | -0,490927335 | 0,719073557 | |
| 217851_s_at | SLMO2 | 3,89689195 | 3,178292062 | 0,718599888 | |
| 215139_at | ARHGEF10 | -0,394749123 | -1,112851844 | 0,718102721 | |
| 219608_s_at | FBXO38 | -0,394749123 | -1,112851844 | 0,718102721 | |
| 238202_at | - | -0,394749123 | -1,112851844 | 0,718102721 | |
| 244533_at | PTPN14 | -0,394749123 | -1,112851844 | 0,718102721 | |
| 1564125_at | LOC285857 | -2,236765673 | -2,954576755 | 0,717811082 | |
| 205230_at | RPH3A | 0,103263601 | -0,614371577 | 0,717635178 | |
| 238104_at | - | 0,103263601 | -0,614371577 | 0,717635178 | |
| 240761_at | - | 0,103263601 | -0,614371577 | 0,717635178 | |
| 223932_at | - | 0,181384709 | -0,536087151 | 0,71747186 | |
| 213550_s_at | IK /// TMCO6 | 1,917954284 | 1,200746069 | 0,717208215 | |
| 1555116_s_at | SLC11A1 | -2,063149277 | -2,780313924 | 0,717164647 | |
| 1556891_at | SORCS1 | -2,063149277 | -2,780313924 | 0,717164647 | |
| 1559374_at | - | -2,063149277 | -2,780313924 | 0,717164647 | |
| 220664_at | SPRR2C | -2,063149277 | -2,780313924 | 0,717164647 | |
| 233871_at | - | -2,063149277 | -2,780313924 | 0,717164647 | |
| 238970_at | - | -2,063149277 | -2,780313924 | 0,717164647 | |
| 1553565_s_at | DDAH1 | -0,43775896 | -1,154897679 | 0,717138718 | |
| 223733_s_at | PPP4R1L | -0,43775896 | -1,154897679 | 0,717138718 | |
| 224417_at | - | -0,43775896 | -1,154897679 | 0,717138718 | |
| 233860_s_at | OBP2A /// OBP2I | -0,43775896 | -1,154897679 | 0,717138718 | |
| 239738_at | DACH2 | -0,43775896 | -1,154897679 | 0,717138718 | |
| 209961_s_at | HGF | 0,255492549 | -0,461594427 | 0,717086976 | |

| | | | | | |
|---|---|---|---|---|---|
| 228363_at | XIAP | 3,670124012 | 2,953119918 | 0,717004094 | |
| 203533_s_at | CUL5 | 1,638726528 | 0,921731975 | 0,716994553 | |
| 1552504_a_at | BRSK1 | -0,946847173 | -1,663414495 | 0,716567321 | |
| 1553325_at | POU5F2 | -0,946847173 | -1,663414495 | 0,716567321 | |
| 1554116_s_at | PARP11 | -0,946847173 | -1,663414495 | 0,716567321 | |
| 215275_at | TRAF3IP3 | -0,946847173 | -1,663414495 | 0,716567321 | |
| 218293_x_at | NUP50 | -0,946847173 | -1,663414495 | 0,716567321 | |
| 236890_at | GTPBP5 | -0,946847173 | -1,663414495 | 0,716567321 | |
| 238687_x_at | ZNF770 | -0,946847173 | -1,663414495 | 0,716567321 | |
| 205972_at | SLC38A3 | -0,234278768 | -0,950686014 | 0,716407247 | |
| 210184_at | ITGAX | -0,234278768 | -0,950686014 | 0,716407247 | |
| 232713_at | - | -0,234278768 | -0,950686014 | 0,716407247 | |
| 1559469_s_at | SIPA1L2 | -0,706943342 | -1,423348341 | 0,716405 | |
| 235687_at | ZNF626 | -0,706943342 | -1,423348341 | 0,716405 | |
| 242731_x_at | - | -0,706943342 | -1,423348341 | 0,716405 | |
| 1557681_s_at | SAMD15 | -1,196375121 | -1,912089812 | 0,71571469 | |
| 205878_at | POU6F1 | -1,196375121 | -1,912089812 | 0,71571469 | |
| 213131_at | OLFM1 | -1,196375121 | -1,912089812 | 0,71571469 | |
| 228553_at | ENAH | -1,196375121 | -1,912089812 | 0,71571469 | |
| 228911_at | ADAMTS7 /// LO( | -1,196375121 | -1,912089812 | 0,71571469 | |
| 1553346_a_at | TNRC6A | -0,196756757 | -0,912331589 | 0,715574832 | |
| 206955_at | AQP7 /// LOC100 | -0,196756757 | -0,912331589 | 0,715574832 | |
| 226570_at | LOC100507375 | 0,457392764 | -0,258114234 | 0,715506998 | |
| 213139_at | SNAI2 | 0,518867317 | -0,19610998 | 0,714977297 | |
| 203763_at | DYNC2LI1 | 2,030406107 | 1,315948728 | 0,714457379 | |
| 1554733_at | LOC728175 | -2,384869671 | -3,098858316 | 0,713988645 | |
| 204409_s_at | EIF1AY | -2,384869671 | -3,098858316 | 0,713988645 | |
| 237435_at | - | -2,597496523 | -3,311072652 | 0,713576129 | |
| 217823_s_at | UBE2J1 | 3,397876879 | 2,684536601 | 0,713340278 | |
| 226711_at | FOXN2 | 3,503346991 | 2,791007185 | 0,712339806 | |
| 218179_s_at | TRAPPC11 | 2,577249059 | 1,865308541 | 0,711940517 | |
| 205539_at | AVIL | -1,026571149 | -1,738067356 | 0,711496207 | |
| 209692_at | EYA2 | -1,026571149 | -1,738067356 | 0,711496207 | |
| 214833_at | TMEM63A | -1,026571149 | -1,738067356 | 0,711496207 | |
| 216294_s_at | KIAA1109 | -1,026571149 | -1,738067356 | 0,711496207 | |
| 230456_at | - | -1,026571149 | -1,738067356 | 0,711496207 | |
| 233634_at | MARVELD3 | -1,026571149 | -1,738067356 | 0,711496207 | |
| 238471_at | - | -1,026571149 | -1,738067356 | 0,711496207 | |
| 239615_at | SLC22A5 | -1,026571149 | -1,738067356 | 0,711496207 | |
| 217024_x_at | - | -0,299034815 | -1,010192375 | 0,71115756 | |
| 232127_at | CLCN5 | -0,299034815 | -1,010192375 | 0,71115756 | |
| 235393_at | - | -0,299034815 | -1,010192375 | 0,71115756 | |
| 238115_at | DNAJC18 | -0,299034815 | -1,010192375 | 0,71115756 | |
| 1557062_at | LOC100129195 | 0,03125738 | -0,679763839 | 0,711021219 | |
| 1569963_at | - | 0,03125738 | -0,679763839 | 0,711021219 | |
| 205773_at | CPEB3 | 0,03125738 | -0,679763839 | 0,711021219 | |
| 226837_at | SPRED1 | 1,575032557 | 0,864649967 | 0,710382589 | |
| 211188_at | CD84 | -0,83191556 | -1,541908042 | 0,709992482 | |
| 243961_at | LOC100505622 | -0,83191556 | -1,541908042 | 0,709992482 | |
| 244685_at | ANGEL1 | -0,83191556 | -1,541908042 | 0,709992482 | |
| 1554157_a_at | WFDC8 | -0,622910612 | -1,332017329 | 0,709106717 | |
| 207437_at | NOVA1 | -0,622910612 | -1,332017329 | 0,709106717 | |
| 207862_at | UPK2 | -0,622910612 | -1,332017329 | 0,709106717 | |
| 223752_at | CFC1 | -0,622910612 | -1,332017329 | 0,709106717 | |
| 226822_at | STOX2 | -0,622910612 | -1,332017329 | 0,709106717 | |
| 239412_at | IRF5 | -0,622910612 | -1,332017329 | 0,709106717 | |

| | | | | | |
|---|---|---|---|---|---|
| 201928_at | PKP4 | 3,406034785 | 2,697670403 | 0,708364382 | |
| 234923_at | RALGAPA1 | -0,022466681 | -0,730778808 | 0,708312127 | |
| 241540_at | - | -0,022466681 | -0,730778808 | 0,708312127 | |
| 1556683_x_at | - | -2,138804305 | -2,847001814 | 0,708197509 | |
| 1556997_a_at | LEPR | -2,138804305 | -2,847001814 | 0,708197509 | |
| 1559266_s_at | SKIDA1 | -2,138804305 | -2,847001814 | 0,708197509 | |
| 1562215_at | - | -2,138804305 | -2,847001814 | 0,708197509 | |
| 229623_at | - | -2,138804305 | -2,847001814 | 0,708197509 | |
| 235182_at | ISM1 | -2,138804305 | -2,847001814 | 0,708197509 | |
| 1563181_a_at | - | -0,512251684 | -1,220210588 | 0,707958903 | |
| 1567686_at | CECR9 | -0,512251684 | -1,220210588 | 0,707958903 | |
| 202273_at | PDGFRB | -0,512251684 | -1,220210588 | 0,707958903 | |
| 204800_s_at | DHRS12 | -0,512251684 | -1,220210588 | 0,707958903 | |
| 231430_at | FAM181B | -0,512251684 | -1,220210588 | 0,707958903 | |
| 214896_at | - | -0,148196756 | -0,855751026 | 0,70755427 | |
| 223326_s_at | AGSK1 /// LOC64 | -0,148196756 | -0,855751026 | 0,70755427 | |
| 223627_at | MEX3B | -0,148196756 | -0,855751026 | 0,70755427 | |
| 208181_at | HIST1H4A /// HIS | 1,542099044 | 0,835239052 | 0,706859991 | |
| 236488_s_at | - | 0,682279745 | -0,024572586 | 0,706852332 | |
| 236514_at | ACOT8 | 1,331241794 | 0,624460759 | 0,706781035 | |
| 1556480_a_at | - | -0,112808516 | -0,819238336 | 0,70642982 | |
| 209172_s_at | CENPF | 1,987559146 | 1,281471383 | 0,706087763 | |
| 1562543_at | - | -2,43257096 | -3,138626879 | 0,706055919 | |
| 230593_at | GRIK3 | -2,43257096 | -3,138626879 | 0,706055919 | |
| 1553021_s_at | BICD2 | -0,67289328 | -1,377419394 | 0,704526114 | |
| 1569133_x_at | ARSK | -0,67289328 | -1,377419394 | 0,704526114 | |
| 202856_s_at | SLC16A3 | -0,67289328 | -1,377419394 | 0,704526114 | |
| 206400_at | LGALS7 /// LGAL | -0,67289328 | -1,377419394 | 0,704526114 | |
| 208287_at | HCG9 | -0,67289328 | -1,377419394 | 0,704526114 | |
| 224099_at | KCNH7 | -0,67289328 | -1,377419394 | 0,704526114 | |
| 241270_at | - | -0,67289328 | -1,377419394 | 0,704526114 | |
| 214048_at | MBD4 | 2,794789337 | 2,091116676 | 0,703672662 | |
| 208319_s_at | RBM3 | 4,41122478 | 3,707626508 | 0,703598272 | |
| 228765_at | GTF2IRD2 | 2,669932407 | 1,966439221 | 0,703493186 | |
| 224873_s_at | MRPS25 | 1,567778694 | 0,864649967 | 0,703128726 | |
| 1556459_at | ARHGAP22-IT1 | -2,187914861 | -2,89013181 | 0,702216949 | |
| 207053_at | SLC8A1 | -2,187914861 | -2,89013181 | 0,702216949 | |
| 210772_at | FPR2 | -2,187914861 | -2,89013181 | 0,702216949 | |
| 214688_at | TLE4 | -2,187914861 | -2,89013181 | 0,702216949 | |
| 227543_at | RNASEH2C | 1,233777334 | 0,532164371 | 0,701612963 | |
| 221786_at | C6orf120 | 4,034147209 | 3,332545245 | 0,701601964 | |
| 226142_at | GLIPR1 | 1,079110292 | 0,377530647 | 0,701579645 | |
| 220510_at | RHBG | 0,417596971 | -0,283667828 | 0,701264798 | |
| 1555078_at | ZNF843 | 0,351548291 | -0,34958478 | 0,701133071 | |
| 213403_at | - | 0,351548291 | -0,34958478 | 0,701133071 | |
| 239431_at | TICAM2 /// TMED | 0,020670649 | -0,679763839 | 0,700434489 | |
| 243604_at | - | 0,020670649 | -0,679763839 | 0,700434489 | |
| 218586_at | C20orf20 | 3,41414682 | 2,713921281 | 0,700225539 | |
| 1555960_at | HINT1 | 0,133056859 | -0,566887459 | 0,699944318 | |
| 203789_s_at | SEMA3C | 0,503742507 | -0,19610998 | 0,699852487 | |
| 222616_s_at | USP16 | 2,331726354 | 1,632095957 | 0,699630397 | |
| 1431_at | CYP2E1 | -2,113928714 | -2,813272577 | 0,699343862 | |
| 1554537_at | TMEM126B | 0,30867765 | -0,39061235 | 0,69929 | |
| 201108_s_at | THBS1 | -0,79540287 | -1,494668682 | 0,699265812 | |
| 230397_at | SAR1B | -0,79540287 | -1,494668682 | 0,699265812 | |
| 238413_at | RRN3P3 | -0,79540287 | -1,494668682 | 0,699265812 | |

| | | | | | |
|---|---|---|---|---|---|
| 205982_x_at | SFTPC | 0,052199481 | -0,646746079 | 0,698945559 | |
| 212701_at | TLN2 | 0,052199481 | -0,646746079 | 0,698945559 | |
| 1556743_at | - | -1,089016378 | -1,787089803 | 0,698073425 | |
| 216830_at | HERC2 /// HERC | -1,089016378 | -1,787089803 | 0,698073425 | |
| 221362_at | HTR5A | -1,089016378 | -1,787089803 | 0,698073425 | |
| 224245_at | INGX | -1,089016378 | -1,787089803 | 0,698073425 | |
| 1557683_at | - | -2,479842156 | -3,177802286 | 0,69796013 | |
| 240959_at | - | -2,479842156 | -3,177802286 | 0,69796013 | |
| 235744_at | PPTC7 | -0,352951799 | -1,050406615 | 0,697454817 | |
| 1559166_at | - | 0,083054335 | -0,614371577 | 0,697425911 | |
| 212606_at | WDFY3 | 1,560488174 | 0,864649967 | 0,695838207 | |
| 236093_at | - | 0,853119696 | 0,157549243 | 0,695570453 | |
| 1553069_at | BRWD1-IT2 | -1,639579028 | -2,335104118 | 0,695525089 | |
| 1559843_s_at | - | -1,639579028 | -2,335104118 | 0,695525089 | |
| 215135_at | DNPEP | -1,639579028 | -2,335104118 | 0,695525089 | |
| 215237_at | DOCK9 | -1,639579028 | -2,335104118 | 0,695525089 | |
| 216666_at | LOC93432 | -1,639579028 | -2,335104118 | 0,695525089 | |
| 223772_s_at | TMEM87A | -1,639579028 | -2,335104118 | 0,695525089 | |
| 224131_at | HCAR1 | -1,639579028 | -2,335104118 | 0,695525089 | |
| 233355_at | LINC00176 | -1,639579028 | -2,335104118 | 0,695525089 | |
| 234087_at | - | -1,639579028 | -2,335104118 | 0,695525089 | |
| 243225_at | LOC283481 | -1,639579028 | -2,335104118 | 0,695525089 | |
| 243322_at | - | -1,639579028 | -2,335104118 | 0,695525089 | |
| 243870_at | - | -1,639579028 | -2,335104118 | 0,695525089 | |
| 217655_at | LOC100127972 | 1,089278259 | 0,393761504 | 0,695516755 | |
| 219847_at | HDAC11 | 1,04816921 | 0,352836757 | 0,695332453 | |
| 200938_s_at | RERE | -1,591296941 | -2,286583451 | 0,695286509 | |
| 205187_at | SMAD5 | -1,591296941 | -2,286583451 | 0,695286509 | |
| 205747_at | CBLN1 | -1,591296941 | -2,286583451 | 0,695286509 | |
| 221416_at | PLA2G2F | -1,591296941 | -2,286583451 | 0,695286509 | |
| 222901_s_at | KCNJ16 | -1,591296941 | -2,286583451 | 0,695286509 | |
| 229263_at | IL17RD | -1,591296941 | -2,286583451 | 0,695286509 | |
| 236333_at | - | -1,591296941 | -2,286583451 | 0,695286509 | |
| 243333_at | - | -1,591296941 | -2,286583451 | 0,695286509 | |
| 1559026_at | LOC100506599 | -1,71423189 | -2,408705138 | 0,694473248 | |
| 213543_at | SGCD | -1,71423189 | -2,408705138 | 0,694473248 | |
| 215344_at | - | -1,71423189 | -2,408705138 | 0,694473248 | |
| 232600_at | - | -1,71423189 | -2,408705138 | 0,694473248 | |
| 242395_at | - | -1,71423189 | -2,408705138 | 0,694473248 | |
| 242620_at | - | -1,71423189 | -2,408705138 | 0,694473248 | |
| 202913_at | ARHGEF11 | 0,822971223 | 0,128747141 | 0,694224083 | |
| 218700_s_at | RAB7L1 | 0,822971223 | 0,128747141 | 0,694224083 | |
| 223738_s_at | PGM2 | 3,152077493 | 2,458228496 | 0,693848997 | |
| 1553847_a_at | SPERT | -1,518072576 | -2,211750327 | 0,693677752 | |
| 1554818_s_at | DZANK1 | -1,518072576 | -2,211750327 | 0,693677752 | |
| 204200_s_at | PDGFB | -1,518072576 | -2,211750327 | 0,693677752 | |
| 208588_at | FKSG2 | -1,518072576 | -2,211750327 | 0,693677752 | |
| 220822_at | - | -1,518072576 | -2,211750327 | 0,693677752 | |
| 239700_at | ZNF710 | -1,518072576 | -2,211750327 | 0,693677752 | |
| 242053_at | - | -1,518072576 | -2,211750327 | 0,693677752 | |
| 1562687_x_at | - | -1,763254337 | -2,456406426 | 0,693152089 | |
| 1562904_s_at | FLJ10661 | -1,763254337 | -2,456406426 | 0,693152089 | |
| 206496_at | FMO3 | -1,763254337 | -2,456406426 | 0,693152089 | |
| 220932_at | - | -1,763254337 | -2,456406426 | 0,693152089 | |
| 230694_at | SLC35E2B | -1,763254337 | -2,456406426 | 0,693152089 | |
| 222612_at | PSPC1 | 0,613492853 | -0,079522948 | 0,693015801 | |

| | | | | | |
|---|---|---|---|---|---|
| 1564362_x_at | ZNF843 | 1,783520648 | 1,090553537 | 0,692967111 | |
| 1438_at | EPHB3 | 0,190865712 | -0,50192623 | 0,692791942 | |
| 236526_x_at | C5orf44 | 0,496120222 | -0,19610998 | 0,692230202 | |
| 1552818_a_at | BRS3 | -2,262747984 | -2,954576755 | 0,691828771 | |
| 1557517_a_at | - | -2,262747984 | -2,954576755 | 0,691828771 | |
| 1560646_at | METTL21CP1 | -2,262747984 | -2,954576755 | 0,691828771 | |
| 1564932_at | - | -2,262747984 | -2,954576755 | 0,691828771 | |
| 208027_s_at | TLL2 | -2,262747984 | -2,954576755 | 0,691828771 | |
| 217282_at | MAN1A2 | -2,262747984 | -2,954576755 | 0,691828771 | |
| 217558_at | CYP2C9 | -2,262747984 | -2,954576755 | 0,691828771 | |
| 232504_at | LOC285628 /// M | -2,262747984 | -2,954576755 | 0,691828771 | |
| 1553734_at | AK7 | -1,470833215 | -2,162639771 | 0,691806556 | |
| 1555052_a_at | SYT9 | -1,470833215 | -2,162639771 | 0,691806556 | |
| 1566221_at | - | -1,470833215 | -2,162639771 | 0,691806556 | |
| 207204_at | FSCN2 | -1,470833215 | -2,162639771 | 0,691806556 | |
| 211640_x_at | IGHG1 /// IGHM | -1,470833215 | -2,162639771 | 0,691806556 | |
| 213475_s_at | ITGAL | -1,470833215 | -2,162639771 | 0,691806556 | |
| 217016_x_at | TMEM212 | -1,470833215 | -2,162639771 | 0,691806556 | |
| 221130_s_at | - | -1,470833215 | -2,162639771 | 0,691806556 | |
| 233857_s_at | ASB2 | -1,470833215 | -2,162639771 | 0,691806556 | |
| 234835_at | LOC100506667 | -1,470833215 | -2,162639771 | 0,691806556 | |
| 235888_at | GUSBP1 | -1,470833215 | -2,162639771 | 0,691806556 | |
| 236728_at | LNPEP | -1,470833215 | -2,162639771 | 0,691806556 | |
| 238405_at | - | -1,470833215 | -2,162639771 | 0,691806556 | |
| 239852_at | MMAA | -1,470833215 | -2,162639771 | 0,691806556 | |
| 208809_s_at | C6orf62 | 6,16251088 | 5,47085447 | 0,69165641 | |
| 223588_at | THAP2 | -1,812418401 | -2,503677622 | 0,691259221 | |
| 233241_at | PLK1S1 | -1,812418401 | -2,503677622 | 0,691259221 | |
| 241863_x_at | - | -1,812418401 | -2,503677622 | 0,691259221 | |
| 1570219_at | LOXL4 | 0,20027154 | -0,490927335 | 0,691198875 | |
| 207855_s_at | CLCC1 | 1,868628927 | 1,177496821 | 0,691132106 | |
| 1563445_x_at | CTSL1P8 | -0,85085127 | -1,541908042 | 0,691056772 | |
| 205805_s_at | ROR1 | -0,85085127 | -1,541908042 | 0,691056772 | |
| 209933_s_at | CD300A | -0,85085127 | -1,541908042 | 0,691056772 | |
| 210113_s_at | NLRP1 | -0,85085127 | -1,541908042 | 0,691056772 | |
| 216346_at | SEC14L3 | -0,85085127 | -1,541908042 | 0,691056772 | |
| 224258_at | DBIL5P2 | -0,85085127 | -1,541908042 | 0,691056772 | |
| 235000_at | LPP | -0,85085127 | -1,541908042 | 0,691056772 | |
| 235649_at | ADAMTS8 | -0,85085127 | -1,541908042 | 0,691056772 | |
| 1557772_at | - | -2,089474779 | -2,780313924 | 0,690839145 | |
| 1559614_at | FLJ38773 | -2,089474779 | -2,780313924 | 0,690839145 | |
| 204840_s_at | EEA1 | -2,089474779 | -2,780313924 | 0,690839145 | |
| 211829_s_at | GPER | -2,089474779 | -2,780313924 | 0,690839145 | |
| 232081_at | - | -2,089474779 | -2,780313924 | 0,690839145 | |
| 240801_at | C21orf37 | -2,089474779 | -2,780313924 | 0,690839145 | |
| 244614_at | TFG | -2,089474779 | -2,780313924 | 0,690839145 | |
| 212889_x_at | GADD45GIP1 | -0,221656139 | -0,912331589 | 0,69067545 | |
| 215611_at | TCF12 | -0,221656139 | -0,912331589 | 0,69067545 | |
| 224171_at | LSM14B | -0,221656139 | -0,912331589 | 0,69067545 | |
| 241396_at | NEDD4L | -0,221656139 | -0,912331589 | 0,69067545 | |
| 1558847_at | LINC00565 | -1,047650486 | -1,738067356 | 0,69041687 | |
| 1561887_at | - | -1,047650486 | -1,738067356 | 0,69041687 | |
| 1570274_at | PRSS55 | -1,047650486 | -1,738067356 | 0,69041687 | |
| 205727_at | TEP1 | -1,047650486 | -1,738067356 | 0,69041687 | |
| 213125_at | OLFML2B | -1,047650486 | -1,738067356 | 0,69041687 | |
| 215100_at | ADTRP | -1,047650486 | -1,738067356 | 0,69041687 | |

| | | | | | |
|---|---|---|---|---|---|
| 217017_at | OSBPL10 | -1,047650486 | -1,738067356 | 0,69041687 | |
| 221124_s_at | VSX1 | -1,047650486 | -1,738067356 | 0,69041687 | |
| 233042_at | - | -1,047650486 | -1,738067356 | 0,69041687 | |
| 237424_at | - | -1,047650486 | -1,738067356 | 0,69041687 | |
| 244547_at | SGK494 | -1,047650486 | -1,738067356 | 0,69041687 | |
| 1569500_at | - | 0,123193849 | -0,566887459 | 0,690081309 | |
| 240698_s_at | - | 0,123193849 | -0,566887459 | 0,690081309 | |
| 1555912_at | ST7-AS1 | 0,228146222 | -0,461594427 | 0,689740648 | |
| 233645_s_at | C1RL | 0,228146222 | -0,461594427 | 0,689740648 | |
| 215209_at | SEC24D | -0,423248115 | -1,112851844 | 0,689603729 | |
| 222210_at | KIAA0195 | -0,423248115 | -1,112851844 | 0,689603729 | |
| 229316_at | - | -0,423248115 | -1,112851844 | 0,689603729 | |
| 238330_s_at | MPRIP | -0,423248115 | -1,112851844 | 0,689603729 | |
| 244099_at | CACNG2 | -0,423248115 | -1,112851844 | 0,689603729 | |
| 209775_x_at | SLC19A1 | -0,57445847 | -1,264005341 | 0,68954687 | |
| 210943_s_at | LYST | -0,57445847 | -1,264005341 | 0,68954687 | |
| 216894_x_at | CDKN1C | -0,57445847 | -1,264005341 | 0,68954687 | |
| 230771_at | NKAIN4 | -0,57445847 | -1,264005341 | 0,68954687 | |
| 242707_at | MED23 | -0,57445847 | -1,264005341 | 0,68954687 | |
| 1555799_at | FCRL5 | -1,173767127 | -1,862647763 | 0,688880636 | |
| 202202_s_at | LAMA4 | -1,173767127 | -1,862647763 | 0,688880636 | |
| 215191_at | - | -1,173767127 | -1,862647763 | 0,688880636 | |
| 221195_at | RNFT1 | -1,173767127 | -1,862647763 | 0,688880636 | |
| 231309_at | GNA12 | -1,173767127 | -1,862647763 | 0,688880636 | |
| 234447_at | - | -1,173767127 | -1,862647763 | 0,688880636 | |
| 1557203_at | PABPC1L2A /// P | 1,326951551 | 0,638150376 | 0,688801174 | |
| 209774_x_at | CXCL2 | 0,152582607 | -0,536087151 | 0,688669758 | |
| 1553619_a_at | TRIM43 /// TRIM4 | -0,926850548 | -1,615132408 | 0,688281859 | |
| 1561611_at | - | -0,926850548 | -1,615132408 | 0,688281859 | |
| 206719_at | SYNGR4 | -0,926850548 | -1,615132408 | 0,688281859 | |
| 210388_at | PLCB2 | -0,926850548 | -1,615132408 | 0,688281859 | |
| 216675_at | - | -0,926850548 | -1,615132408 | 0,688281859 | |
| 220224_at | HAO1 | -0,926850548 | -1,615132408 | 0,688281859 | |
| 227509_x_at | - | -0,926850548 | -1,615132408 | 0,688281859 | |
| 230658_at | SLC7A2 | -0,926850548 | -1,615132408 | 0,688281859 | |
| 1555452_at | RALGPS1 | 0,695652136 | 0,007421914 | 0,688230223 | |
| 229701_at | DNAJC9 | -0,467091869 | -1,154897679 | 0,68780581 | |
| 239139_at | CPNE9 | -0,467091869 | -1,154897679 | 0,68780581 | |
| 240210_at | ATAD3C | -0,467091869 | -1,154897679 | 0,68780581 | |
| 1552584_at | IL12RB1 | -1,399512875 | -2,086984744 | 0,687471868 | |
| 1556004_at | LOC100506777 | -1,399512875 | -2,086984744 | 0,687471868 | |
| 1562390_at | - | -1,399512875 | -2,086984744 | 0,687471868 | |
| 1568906_at | LOC728196 | -1,399512875 | -2,086984744 | 0,687471868 | |
| 205932_s_at | MSX1 | -1,399512875 | -2,086984744 | 0,687471868 | |
| 207726_at | ESRRB | -1,399512875 | -2,086984744 | 0,687471868 | |
| 228010_at | PPP2R2C | -1,399512875 | -2,086984744 | 0,687471868 | |
| 207961_x_at | MYH11 | -0,078265071 | -0,765504029 | 0,687238958 | |
| 210204_s_at | CNOT4 | -0,078265071 | -0,765504029 | 0,687238958 | |
| 233531_at | SLC46A1 | -0,078265071 | -0,765504029 | 0,687238958 | |
| 238425_at | PSMB2 | 0,715481152 | 0,028364014 | 0,687117137 | |
| 39318_at | TCL1A | -1,469630988 | -2,1567009 | 0,687069912 | |
| 1553470_at | DNAH17 | -1,888254346 | -2,575129729 | 0,686875383 | |
| 206043_s_at | ATP2C2 | -1,888254346 | -2,575129729 | 0,686875383 | |
| 206045_s_at | NOL4 | -1,888254346 | -2,575129729 | 0,686875383 | |
| 216093_at | - | -1,888254346 | -2,575129729 | 0,686875383 | |
| 227188_at | FAM176C | -1,888254346 | -2,575129729 | 0,686875383 | |

| | | | | | |
|---|---|---|---|---|---|
| 234060_at | - | -1,888254346 | -2,575129729 | 0,686875383 | |
| 241444_at | - | -1,888254346 | -2,575129729 | 0,686875383 | |
| 242132_x_at | - | -1,888254346 | -2,575129729 | 0,686875383 | |
| 231106_at | BMS1P2 /// BMS1 | 0,606430281 | -0,079522948 | 0,68595323 | |
| 218158_s_at | APPL1 | 2,064305961 | 1,378453643 | 0,685852317 | |
| 206235_at | LIG4 | 1,90937145 | 1,223626587 | 0,685744862 | |
| 213686_at | VPS13A | 1,079110292 | 0,393761504 | 0,685348788 | |
| 1558397_at | PECAM1 | -2,311268652 | -2,996335703 | 0,685067051 | |
| 236917_at | LRRC34 | 0,401366113 | -0,283667828 | 0,68503394 | |
| 241734_at | SRFBP1 | 1,037706275 | 0,352836757 | 0,684869518 | |
| 234749_s_at | POC1A | 0,934330293 | 0,249604176 | 0,684726117 | |
| 1554834_a_at | RASSF5 | -0,325749314 | -1,010192375 | 0,684443061 | |
| 1556986_at | OR2H1 | -0,325749314 | -1,010192375 | 0,684443061 | |
| 203769_s_at | STS | -0,325749314 | -1,010192375 | 0,684443061 | |
| 240410_at | - | -0,325749314 | -1,010192375 | 0,684443061 | |
| 219487_at | BBS10 | 1,238353345 | 0,553993624 | 0,684359721 | |
| 1559642_a_at | - | -1,353583928 | -2,037551727 | 0,683967799 | |
| 1568695_s_at | INTS6 | -1,353583928 | -2,037551727 | 0,683967799 | |
| 222080_s_at | SIRT5 | -1,353583928 | -2,037551727 | 0,683967799 | |
| 229834_at | NFIX | -1,353583928 | -2,037551727 | 0,683967799 | |
| 233115_at | - | -1,353583928 | -2,037551727 | 0,683967799 | |
| 238924_at | BMS1P1 /// BMS1 | -1,353583928 | -2,037551727 | 0,683967799 | |
| 239385_at | TFG | -1,353583928 | -2,037551727 | 0,683967799 | |
| 241021_at | - | -1,353583928 | -2,037551727 | 0,683967799 | |
| 229167_at | PURA | 1,251994932 | 0,568365095 | 0,683629837 | |
| 1555308_at | CARD14 | -0,366776884 | -1,050406615 | 0,683629731 | |
| 1555448_at | AP5M1 | -0,366776884 | -1,050406615 | 0,683629731 | |
| 1557878_at | - | -0,366776884 | -1,050406615 | 0,683629731 | |
| 1563244_at | - | -0,366776884 | -1,050406615 | 0,683629731 | |
| 212810_s_at | SLC1A4 | -0,366776884 | -1,050406615 | 0,683629731 | |
| 244559_at | - | -0,366776884 | -1,050406615 | 0,683629731 | |
| 1559344_at | - | -1,937711681 | -2,621331989 | 0,683620308 | |
| 1563469_at | - | -1,937711681 | -2,621331989 | 0,683620308 | |
| 1566664_at | CCDC168 | -1,937711681 | -2,621331989 | 0,683620308 | |
| 1570424_at | - | -1,937711681 | -2,621331989 | 0,683620308 | |
| 206312_at | GUCY2C | -1,937711681 | -2,621331989 | 0,683620308 | |
| 214539_at | SERPINB10 | -1,937711681 | -2,621331989 | 0,683620308 | |
| 229923_at | ZDHHC21 | -1,937711681 | -2,621331989 | 0,683620308 | |
| 232026_at | HERC4 | -1,937711681 | -2,621331989 | 0,683620308 | |
| 240967_at | KRTAP19-3 | -1,937711681 | -2,621331989 | 0,683620308 | |
| 219138_at | RPL14 | 2,37600538 | 1,692388143 | 0,683617237 | |
| 232085_at | MAPK8IP3 | -0,172274514 | -0,855751026 | 0,683476512 | |
| 237222_at | FSD2 | -0,172274514 | -0,855751026 | 0,683476512 | |
| 219104_at | RNF141 | 1,163336153 | 0,479907041 | 0,683429112 | |
| 1553693_s_at | CBR4 | -0,136301282 | -0,819238336 | 0,682937054 | |
| 1557720_s_at | MYO16 | -0,136301282 | -0,819238336 | 0,682937054 | |
| 204555_s_at | PPP1R3D | -0,136301282 | -0,819238336 | 0,682937054 | |
| 229362_at | PUS10 | 1,683039483 | 1,000684521 | 0,682354962 | |
| 230399_at | - | 1,139070151 | 0,456918297 | 0,682151853 | |
| 220952_s_at | PLEKHA5 | 0,810733188 | 0,128747141 | 0,681986047 | |
| 221519_at | FBXW4 | 1,64218396 | 0,960386719 | 0,681797241 | |
| 213325_at | PVRL3 | 0,291166226 | -0,39061235 | 0,681778576 | |
| 1554406_a_at | CLEC7A | -0,741668563 | -1,423348341 | 0,681679779 | |
| 1559293_x_at | LINC00032 | -0,741668563 | -1,423348341 | 0,681679779 | |
| 210309_at | RECQL5 | -0,741668563 | -1,423348341 | 0,681679779 | |
| 210356_x_at | MS4A1 | -0,741668563 | -1,423348341 | 0,681679779 | |

| | | | | | |
|---|---|---|---|---|---|
| 210546_x_at | CTAG1A /// CTA( | -0,741668563 | -1,423348341 | 0,681679779 | |
| 210656_at | EED | -0,741668563 | -1,423348341 | 0,681679779 | |
| 228540_at | QKI | -0,741668563 | -1,423348341 | 0,681679779 | |
| 236783_at | KCNIP4 | -0,741668563 | -1,423348341 | 0,681679779 | |
| 240634_x_at | - | -0,741668563 | -1,423348341 | 0,681679779 | |
| 229123_at | - | 1,575032557 | 0,893473268 | 0,681559289 | |
| 224261_at | - | 0,760715727 | 0,079428135 | 0,681287591 | |
| 243927_x_at | KIAA1429 | 1,795990708 | 1,115234684 | 0,680756023 | |
| 204829_s_at | FOLR2 | 0,57782909 | -0,102100538 | 0,679929628 | |
| 227277_at | MTDH | 1,69307511 | 1,013870809 | 0,679204301 | |
| 225840_at | TEF | 0,928678725 | 0,249604176 | 0,679074549 | |
| 242208_at | ZNF37BP | -0,00073712 | -0,679763839 | 0,679026719 | |
| 1557293_at | LOC440993 | 0,518867317 | -0,160136748 | 0,679004065 | |
| 223249_at | CLDN12 | 2,069451339 | 1,390636111 | 0,678815228 | |
| 206894_at | APOA4 | -0,234278768 | -0,912331589 | 0,678052821 | |
| 224328_s_at | LCE3D | -0,234278768 | -0,912331589 | 0,678052821 | |
| 244631_at | LOC389834 | 0,03125738 | -0,646746079 | 0,678003459 | |
| 208347_at | - | 0,900083947 | 0,222599095 | 0,677484853 | |
| 1554187_at | LOC554206 | -2,01371626 | -2,690860744 | 0,677144484 | |
| 207636_at | SERPINI2 | -2,01371626 | -2,690860744 | 0,677144484 | |
| 211286_x_at | CSF2RA | -2,01371626 | -2,690860744 | 0,677144484 | |
| 232271_at | HNF4G | -2,01371626 | -2,690860744 | 0,677144484 | |
| 206040_s_at | MAPK11 | -0,986356909 | -1,663414495 | 0,677057586 | |
| 215145_s_at | CNTNAP2 | -0,986356909 | -1,663414495 | 0,677057586 | |
| 221384_at | UCP1 | -0,986356909 | -1,663414495 | 0,677057586 | |
| 230190_at | NDFIP2 | -0,986356909 | -1,663414495 | 0,677057586 | |
| 230858_at | LOC100499467 | -0,986356909 | -1,663414495 | 0,677057586 | |
| 234174_at | - | -0,986356909 | -1,663414495 | 0,677057586 | |
| 234284_at | GNG8 | -0,986356909 | -1,663414495 | 0,677057586 | |
| 237559_at | GPR55 | -0,986356909 | -1,663414495 | 0,677057586 | |
| 1554852_a_at | KIAA1257 | -1,2845686 | -1,961547147 | 0,676978547 | |
| 1558941_at | ZNF704 | -1,2845686 | -1,961547147 | 0,676978547 | |
| 1559535_s_at | - | -1,2845686 | -1,961547147 | 0,676978547 | |
| 1560537_at | LOC100129662 | -1,2845686 | -1,961547147 | 0,676978547 | |
| 1560968_at | - | -1,2845686 | -1,961547147 | 0,676978547 | |
| 208222_at | ACVR1B | -1,2845686 | -1,961547147 | 0,676978547 | |
| 229124_at | PROK1 | -1,2845686 | -1,961547147 | 0,676978547 | |
| 231695_at | - | -1,2845686 | -1,961547147 | 0,676978547 | |
| 239446_x_at | DCBLD2 | -1,2845686 | -1,961547147 | 0,676978547 | |
| 239557_at | - | -1,2845686 | -1,961547147 | 0,676978547 | |
| 243951_at | ABCB1 | -1,2845686 | -1,961547147 | 0,676978547 | |
| 244278_at | - | -1,2845686 | -1,961547147 | 0,676978547 | |
| 1554807_a_at | SPIRE1 | 0,480753764 | -0,19610998 | 0,676863744 | |
| 212824_at | FUBP3 | 4,574391988 | 3,898162764 | 0,676229225 | |
| 215278_at | - | -0,655928373 | -1,332017329 | 0,676088956 | |
| 235829_at | CAHM | -0,655928373 | -1,332017329 | 0,676088956 | |
| 239903_at | TPBG | -0,655928373 | -1,332017329 | 0,676088956 | |
| 241134_at | - | -0,655928373 | -1,332017329 | 0,676088956 | |
| 227091_at | CCDC146 | -0,089689931 | -0,765504029 | 0,675814098 | |
| 235502_at | MIR3661 /// PPP2 | -0,089689931 | -0,765504029 | 0,675814098 | |
| 240325_x_at | - | -0,089689931 | -0,765504029 | 0,675814098 | |
| 210768_x_at | TMCO1 | 3,75400739 | 3,078904771 | 0,675102619 | |
| 219746_at | DPF3 | 1,011212064 | 0,336136178 | 0,675075886 | |
| 200632_s_at | NDRG1 | 0,682279745 | 0,007421914 | 0,674857832 | |
| 234982_at | UBR3 | 4,260221813 | 3,585762986 | 0,674458827 | |
| 229038_at | CWF19L1 | 0,351548291 | -0,322870281 | 0,674418572 | |

| | | | | | |
|---|---|---|---|---|---|
| 234441_at | - | 0,351548291 | -0,322870281 | 0,674418572 | |
| 220185_at | SPTBN4 | 1,027166904 | 0,352836757 | 0,674330147 | |
| 214785_at | VPS13A | 1,567778694 | 0,893473268 | 0,674305426 | |
| 211610_at | KLF6 | 0,255492549 | -0,41858459 | 0,674077139 | |
| 225856_at | CLOCK | 0,255492549 | -0,41858459 | 0,674077139 | |
| 233612_at | - | 0,255492549 | -0,41858459 | 0,674077139 | |
| 217508_s_at | C18orf25 | 0,702292136 | 0,028364014 | 0,673928122 | |
| 214278_s_at | NDRG2 | -0,59053611 | -1,264005341 | 0,67346923 | |
| 215820_x_at | SNX13 | -0,59053611 | -1,264005341 | 0,67346923 | |
| 216631_s_at | - | -0,59053611 | -1,264005341 | 0,67346923 | |
| 219592_at | MCPH1 | -0,59053611 | -1,264005341 | 0,67346923 | |
| 238693_at | PHC3 | -0,59053611 | -1,264005341 | 0,67346923 | |
| 241477_at | MIR631 /// NEIL1 | -0,59053611 | -1,264005341 | 0,67346923 | |
| 222426_at | MAPKAP1 | 1,850809354 | 1,177496821 | 0,673312534 | |
| 1561544_at | - | -2,063149277 | -2,735862426 | 0,672713148 | |
| 1570327_at | C20orf62 | -2,063149277 | -2,735862426 | 0,672713148 | |
| 206285_at | NPHP1 | -2,063149277 | -2,735862426 | 0,672713148 | |
| 216027_at | TMX4 | -2,063149277 | -2,735862426 | 0,672713148 | |
| 220907_at | GPR110 | -2,063149277 | -2,735862426 | 0,672713148 | |
| 230324_at | - | -2,063149277 | -2,735862426 | 0,672713148 | |
| 222283_at | ZNF480 | 0,181384709 | -0,490927335 | 0,672312044 | |
| 225875_s_at | NIPAL3 | 0,181384709 | -0,490927335 | 0,672312044 | |
| 238228_at | - | 0,181384709 | -0,490927335 | 0,672312044 | |
| 242679_at | TTN-AS1 | 0,181384709 | -0,490927335 | 0,672312044 | |
| 233899_x_at | ZBTB10 | 1,296557217 | 0,624460759 | 0,672096458 | |
| 1559517_a_at | SPIRE1 | -1,240169874 | -1,912089812 | 0,671919938 | |
| 215521_at | PHC3 | -1,240169874 | -1,912089812 | 0,671919938 | |
| 215869_at | - | -1,240169874 | -1,912089812 | 0,671919938 | |
| 232843_s_at | DOCK8 | 1,261018186 | 0,589657387 | 0,671360799 | |
| 211203_s_at | CNTN1 | 0,209623097 | -0,461594427 | 0,671217524 | |
| 243983_at | - | 0,209623097 | -0,461594427 | 0,671217524 | |
| 1566884_at | - | -0,148196756 | -0,819238336 | 0,67104158 | |
| 222362_at | AGFG2 | -0,148196756 | -0,819238336 | 0,67104158 | |
| 232828_at | NALCN-AS1 | -0,148196756 | -0,819238336 | 0,67104158 | |
| 242473_at | TRAF4 | -0,148196756 | -0,819238336 | 0,67104158 | |
| 219536_s_at | ZFP64 | 0,533835206 | -0,136643983 | 0,670479189 | |
| 1557292_a_at | MCOLN3 | -0,706943342 | -1,377419394 | 0,670476052 | |
| 1558289_at | RFT1 | -0,706943342 | -1,377419394 | 0,670476052 | |
| 207395_at | BTN1A1 | -0,706943342 | -1,377419394 | 0,670476052 | |
| 224176_s_at | AXIN2 | -0,706943342 | -1,377419394 | 0,670476052 | |
| 230431_at | - | -0,706943342 | -1,377419394 | 0,670476052 | |
| 234040_at | HELLS | -0,706943342 | -1,377419394 | 0,670476052 | |
| 1564155_x_at | - | 0,103263601 | -0,566887459 | 0,670151061 | |
| 204655_at | CCL5 | 0,103263601 | -0,566887459 | 0,670151061 | |
| 220471_s_at | MYCT1 | 1,406361373 | 0,73688026 | 0,669481113 | |
| 223481_s_at | MRPL47 | 0,728550685 | 0,059218869 | 0,669331817 | |
| 228971_at | - | 1,799091453 | 1,129843209 | 0,669248245 | |
| 213871_s_at | C6orf108 | 0,133056859 | -0,536087151 | 0,669144009 | |
| 236476_at | - | 0,384950573 | -0,283667828 | 0,668618401 | |
| 204889_s_at | NEURL | 0,747935635 | 0,079428135 | 0,6685075 | |
| 1554594_at | ARHGAP27 | -1,543331864 | -2,211750327 | 0,668418463 | |
| 1569832_at | LOC100131655 | -1,543331864 | -2,211750327 | 0,668418463 | |
| 1570190_at | LSAMP | -1,543331864 | -2,211750327 | 0,668418463 | |
| 205403_at | IL1R2 | -1,543331864 | -2,211750327 | 0,668418463 | |
| 208412_s_at | RARB | -1,543331864 | -2,211750327 | 0,668418463 | |
| 237214_at | TFRC | -1,543331864 | -2,211750327 | 0,668418463 | |

| | | | | | |
|---|---|---|---|---|---|
| 241227_at | - | -1,543331864 | -2,211750327 | 0,668418463 | |
| 1553061_at | OR6W1P | -0,946847173 | -1,615132408 | 0,668285235 | |
| 1553122_s_at | LOC389458 /// RI | -0,946847173 | -1,615132408 | 0,668285235 | |
| 1553531_at | PCSK6 | -0,946847173 | -1,615132408 | 0,668285235 | |
| 1562764_at | LOC100507351 | -0,946847173 | -1,615132408 | 0,668285235 | |
| 217670_at | RPLP2 | -0,946847173 | -1,615132408 | 0,668285235 | |
| 229469_at | XAGE2 /// XAGE: | -0,946847173 | -1,615132408 | 0,668285235 | |
| 220208_at | ADAMTS13 | 0,917308734 | 0,249604176 | 0,667704558 | |
| 222475_at | SAP30BP | 1,454352496 | 0,786897721 | 0,667454775 | |
| 240429_at | ZNF546 | 0,317354302 | -0,34958478 | 0,666939082 | |
| 213963_s_at | SAP30 | 0,87091093 | 0,204310755 | 0,666600175 | |
| 1557091_at | MAMSTR | 0,052199481 | -0,614371577 | 0,666571057 | |
| 236079_at | LOC202025 | 0,052199481 | -0,614371577 | 0,666571057 | |
| 237855_at | ZNF777 | 0,052199481 | -0,614371577 | 0,666571057 | |
| 229174_at | C3orf38 | 3,149650293 | 2,483094137 | 0,666556157 | |
| 229169_at | TTC18 | -2,43257096 | -3,098858316 | 0,666287356 | |
| 1555439_at | GTF3C3 | -1,196375121 | -1,862647763 | 0,666272641 | |
| 214376_at | - | -1,196375121 | -1,862647763 | 0,666272641 | |
| 217195_at | - | -1,196375121 | -1,862647763 | 0,666272641 | |
| 235619_at | ASB4 | -1,196375121 | -1,862647763 | 0,666272641 | |
| 236610_at | - | -1,196375121 | -1,862647763 | 0,666272641 | |
| 239764_at | - | -1,196375121 | -1,862647763 | 0,666272641 | |
| 1554383_a_at | TRAM2 | 0,641402394 | -0,024572586 | 0,665974981 | |
| 230005_at | SVIP | 3,565667132 | 2,900133867 | 0,665533265 | |
| 217430_x_at | COL1A1 | 2,573632254 | 1,908311039 | 0,665321215 | |
| 1563259_at | - | 0,246434561 | -0,41858459 | 0,665019151 | |
| 210251_s_at | RUFY3 | 0,246434561 | -0,41858459 | 0,665019151 | |
| 215681_at | KIAA1654 | 0,246434561 | -0,41858459 | 0,665019151 | |
| 219272_at | TRIM62 | 0,246434561 | -0,41858459 | 0,665019151 | |
| 1556465_at | - | -1,838812296 | -2,503677622 | 0,664865326 | |
| 1559633_a_at | CHRM3 | -1,838812296 | -2,503677622 | 0,664865326 | |
| 1564820_at | - | -1,838812296 | -2,503677622 | 0,664865326 | |
| 207478_at | - | -1,838812296 | -2,503677622 | 0,664865326 | |
| 220360_at | THAP9 | -1,838812296 | -2,503677622 | 0,664865326 | |
| 231142_at | - | -1,838812296 | -2,503677622 | 0,664865326 | |
| 230208_at | HCN4 | -0,285837228 | -0,950686014 | 0,664848786 | |
| 243116_at | PIP5KL1 | -0,285837228 | -0,950686014 | 0,664848786 | |
| 37004_at | SFTPB | -0,414543702 | -1,079163354 | 0,664619651 | |
| 214408_s_at | RFPL1-AS1 /// RI | -0,759588698 | -1,423348341 | 0,663759643 | |
| 216968_at | MASP2 | -0,759588698 | -1,423348341 | 0,663759643 | |
| 228890_at | ATOH8 | -0,759588698 | -1,423348341 | 0,663759643 | |
| 236228_at | LOC100130744 | -0,759588698 | -1,423348341 | 0,663759643 | |
| 239582_at | PML | -0,759588698 | -1,423348341 | 0,663759643 | |
| 240077_at | - | -0,759588698 | -1,423348341 | 0,663759643 | |
| 208547_at | HIST1H2BB | 1,596577845 | 0,932882274 | 0,663695571 | |
| 32625_at | NPR1 | -0,345897123 | -1,009110727 | 0,663213604 | |
| 1561896_at | - | -1,424023738 | -2,086984744 | 0,662961005 | |
| 1566503_at | - | -1,424023738 | -2,086984744 | 0,662961005 | |
| 202270_at | GBP1 | -1,424023738 | -2,086984744 | 0,662961005 | |
| 202995_s_at | FBLN1 | -1,424023738 | -2,086984744 | 0,662961005 | |
| 217483_at | FOLH1 | -1,424023738 | -2,086984744 | 0,662961005 | |
| 226304_at | HSPB6 | -1,424023738 | -2,086984744 | 0,662961005 | |
| 233769_at | - | -1,424023738 | -2,086984744 | 0,662961005 | |
| 236440_at | NETO1 | -1,424023738 | -2,086984744 | 0,662961005 | |
| 238713_at | - | -1,424023738 | -2,086984744 | 0,662961005 | |
| 1569502_s_at | TP73-AS1 | 0,171847695 | -0,490927335 | 0,662775031 | |

| | | | | | |
|---|---|---|---|---|---|
| 1560296_at | - | -0,83191556 | -1,494668682 | 0,662753122 | |
| 1561318_at | - | -0,83191556 | -1,494668682 | 0,662753122 | |
| 1562834_at | - | -0,83191556 | -1,494668682 | 0,662753122 | |
| 1569608_x_at | - | -0,83191556 | -1,494668682 | 0,662753122 | |
| 204575_s_at | MMP19 | -0,83191556 | -1,494668682 | 0,662753122 | |
| 215315_at | ZNF549 | -0,83191556 | -1,494668682 | 0,662753122 | |
| 216774_at | - | -0,83191556 | -1,494668682 | 0,662753122 | |
| 227578_at | TMPO-AS1 | -0,83191556 | -1,494668682 | 0,662753122 | |
| 240744_at | CPA5 | -0,83191556 | -1,494668682 | 0,662753122 | |
| 242261_at | IREB2 | -0,83191556 | -1,494668682 | 0,662753122 | |
| 244810_at | - | -0,83191556 | -1,494668682 | 0,662753122 | |
| 227092_at | LOC100507448 | 0,97328325 | 0,310717132 | 0,662566118 | |
| 223608_at | EFCAB2 | 2,338135744 | 1,675891488 | 0,662244256 | |
| 227038_at | SGMS2 | 0,911589958 | 0,249604176 | 0,661985783 | |
| 208227_x_at | ADAM22 | 0,20027154 | -0,461594427 | 0,661865966 | |
| 229492_at | VANGL1 | 0,20027154 | -0,461594427 | 0,661865966 | |
| 232913_at | TMED8 | 0,20027154 | -0,461594427 | 0,661865966 | |
| 236920_at | RHOXF2 /// RHO | 0,20027154 | -0,461594427 | 0,661865966 | |
| 241907_at | - | 0,20027154 | -0,461594427 | 0,661865966 | |
| 212800_at | STX6 | 1,079110292 | 0,417770464 | 0,661339829 | |
| 1562449_s_at | LOC100506195 | 0,46522187 | -0,19610998 | 0,66133185 | |
| 1555377_at | OR4D2 | 0,376672223 | -0,283667828 | 0,660340051 | |
| 208576_s_at | HIST1H3A /// HIS | 0,376672223 | -0,283667828 | 0,660340051 | |
| 216333_x_at | TNXA /// TNXB | 0,376672223 | -0,283667828 | 0,660340051 | |
| 1560402_at | GAS5 | 0,882651021 | 0,222599095 | 0,660051926 | |
| 201289_at | CYR61 | 3,503346991 | 2,843651104 | 0,659695887 | |
| 239263_at | LOC100506813 | 0,401366113 | -0,258114234 | 0,659480347 | |
| 231036_at | LOC100505774 | 0,123193849 | -0,536087151 | 0,659281 | |
| 233854_x_at | KLK4 | 0,123193849 | -0,536087151 | 0,659281 | |
| 238613_at | ZAK | 0,123193849 | -0,536087151 | 0,659281 | |
| 1558501_at | DNM3 | -0,67289328 | -1,332017329 | 0,659124049 | |
| 1564263_at | LINC00330 | -0,67289328 | -1,332017329 | 0,659124049 | |
| 215569_at | GTF2IRD2B | -0,67289328 | -1,332017329 | 0,659124049 | |
| 223820_at | RBP5 | -0,67289328 | -1,332017329 | 0,659124049 | |
| 233730_at | FAM135A | -2,187914861 | -2,847001814 | 0,659086953 | |
| 241238_at | - | -2,187914861 | -2,847001814 | 0,659086953 | |
| 244441_at | - | -2,187914861 | -2,847001814 | 0,659086953 | |
| 1555561_a_at | UGGT2 | -0,196756757 | -0,855751026 | 0,658994269 | |
| 1561978_at | LOC284798 | -0,196756757 | -0,855751026 | 0,658994269 | |
| 229589_x_at | BIVM | -0,196756757 | -0,855751026 | 0,658994269 | |
| 242530_at | SV2C | -0,196756757 | -0,855751026 | 0,658994269 | |
| 1561860_at | - | -2,479842156 | -3,138626879 | 0,658784723 | |
| 241120_s_at | CDC20B | -2,479842156 | -3,138626879 | 0,658784723 | |
| 237034_at | - | 1,153678675 | 0,495031851 | 0,658646824 | |
| 1566207_at | TCEA1 | 1,011212064 | 0,352836757 | 0,658375307 | |
| 223994_s_at | SLC12A9 | 0,30867765 | -0,34958478 | 0,65826243 | |
| 1566974_at | - | -0,496991414 | -1,154897679 | 0,657906265 | |
| 222695_s_at | AXIN2 | -0,496991414 | -1,154897679 | 0,657906265 | |
| 210764_s_at | CYR61 | 2,247975581 | 1,590454215 | 0,657521366 | |
| 240598_at | - | 0,334552598 | -0,322870281 | 0,657422879 | |
| 204865_at | CA3 | -0,022466681 | -0,679763839 | 0,657297159 | |
| 236726_at | RGS6 | -0,022466681 | -0,679763839 | 0,657297159 | |
| 223908_at | HDAC8 | -0,352951799 | -1,010192375 | 0,657240577 | |
| 228563_at | GJC1 | -0,352951799 | -1,010192375 | 0,657240577 | |
| 236991_at | - | -0,352951799 | -1,010192375 | 0,657240577 | |
| 237875_at | - | -0,352951799 | -1,010192375 | 0,657240577 | |

| | | | | | |
|---|---|---|---|---|---|
| 243079_x_at | - | -0,352951799 | -1,010192375 | 0,657240577 | |
| 1563116_at | - | -1,964176657 | -2,621331989 | 0,657155333 | |
| 1566767_at | - | -1,964176657 | -2,621331989 | 0,657155333 | |
| 223775_at | HHIP | -1,964176657 | -2,621331989 | 0,657155333 | |
| 226057_at | ARHGAP31 | -1,964176657 | -2,621331989 | 0,657155333 | |
| 229542_at | C20orf85 | -1,964176657 | -2,621331989 | 0,657155333 | |
| 236850_at | CAPRIN1 | -1,964176657 | -2,621331989 | 0,657155333 | |
| 237758_at | LOC100505685 | -1,964176657 | -2,621331989 | 0,657155333 | |
| 241848_x_at | - | -1,964176657 | -2,621331989 | 0,657155333 | |
| 242381_x_at | - | -1,964176657 | -2,621331989 | 0,657155333 | |
| 244276_at | KLB | -1,964176657 | -2,621331989 | 0,657155333 | |
| 228955_at | LRP8 | 1,348276312 | 0,691645685 | 0,656630626 | |
| 242235_x_at | - | 1,360921491 | 0,704715219 | 0,656206272 | |
| 1555168_a_at | CALN1 | -1,131062212 | -1,787089803 | 0,656027591 | |
| 1559964_at | FLJ38717 | -1,131062212 | -1,787089803 | 0,656027591 | |
| 1566093_at | ARHGEF12 | -1,131062212 | -1,787089803 | 0,656027591 | |
| 207084_at | POU3F2 | -1,131062212 | -1,787089803 | 0,656027591 | |
| 209897_s_at | SLIT2 | -1,131062212 | -1,787089803 | 0,656027591 | |
| 215777_at | - | -1,131062212 | -1,787089803 | 0,656027591 | |
| 220364_at | FLJ11235 | -1,131062212 | -1,787089803 | 0,656027591 | |
| 236102_at | LOC100126784 | -1,131062212 | -1,787089803 | 0,656027591 | |
| 238008_at | PRR18 | -1,131062212 | -1,787089803 | 0,656027591 | |
| 206029_at | ANKRD1 | -0,394749123 | -1,050406615 | 0,655657492 | |
| 218651_s_at | LARP6 | -0,394749123 | -1,050406615 | 0,655657492 | |
| 213114_at | - | 0,518867317 | -0,136643983 | 0,6555113 | |
| 235775_at | TMTC2 | 0,518867317 | -0,136643983 | 0,6555113 | |
| 208030_s_at | ADD1 | 3,484209953 | 2,828804976 | 0,655404977 | |
| 1554019_s_at | CEP57L1 | 0,457392764 | -0,19610998 | 0,653502744 | |
| 240651_at | - | 0,457392764 | -0,19610998 | 0,653502744 | |
| 1553629_a_at | FAM71B | -0,888496123 | -1,541908042 | 0,653411919 | |
| 1556309_s_at | C1orf86 | -0,888496123 | -1,541908042 | 0,653411919 | |
| 1570625_at | TCEB3 | -0,888496123 | -1,541908042 | 0,653411919 | |
| 205656_at | PCDH17 | -0,888496123 | -1,541908042 | 0,653411919 | |
| 210367_s_at | PTGES | -0,888496123 | -1,541908042 | 0,653411919 | |
| 211411_at | - | -0,888496123 | -1,541908042 | 0,653411919 | |
| 215394_at | PIK3C3 | -0,888496123 | -1,541908042 | 0,653411919 | |
| 221299_at | GPR173 | -0,888496123 | -1,541908042 | 0,653411919 | |
| 231516_at | LOC100507146 | -0,888496123 | -1,541908042 | 0,653411919 | |
| 234175_at | - | -0,888496123 | -1,541908042 | 0,653411919 | |
| 234564_at | - | -0,888496123 | -1,541908042 | 0,653411919 | |
| 234886_at | - | -0,888496123 | -1,541908042 | 0,653411919 | |
| 240999_at | LOC100287704 | -0,888496123 | -1,541908042 | 0,653411919 | |
| 1552705_at | DUSP19 | -2,236765673 | -2,89013181 | 0,653366137 | |
| 1563771_a_at | - | -2,236765673 | -2,89013181 | 0,653366137 | |
| 1567986_at | - | -2,236765673 | -2,89013181 | 0,653366137 | |
| 205363_at | BBOX1 | -2,236765673 | -2,89013181 | 0,653366137 | |
| 220622_at | LRRC31 | -2,236765673 | -2,89013181 | 0,653366137 | |
| 240695_at | - | -2,236765673 | -2,89013181 | 0,653366137 | |
| 241832_at | FAM98A | -2,236765673 | -2,89013181 | 0,653366137 | |
| 242392_at | UBE2U | -2,236765673 | -2,89013181 | 0,653366137 | |
| 206359_at | SOCS3 | -1,308181863 | -1,961547147 | 0,653365284 | |
| 210164_at | GZMB | -1,308181863 | -1,961547147 | 0,653365284 | |
| 217259_at | - | -1,308181863 | -1,961547147 | 0,653365284 | |
| 228586_at | - | -1,308181863 | -1,961547147 | 0,653365284 | |
| 228656_at | PROX1 | -1,308181863 | -1,961547147 | 0,653365284 | |
| 235973_at | - | -1,308181863 | -1,961547147 | 0,653365284 | |

| | | | | | |
|---|---|---|---|---|---|
| 235988_at | GPR110 | -1,308181863 | -1,961547147 | 0,653365284 | |
| 240851_at | - | -1,308181863 | -1,961547147 | 0,653365284 | |
| 214590_s_at | UBE2D1 | -0,112808516 | -0,765504029 | 0,652695512 | |
| 223840_s_at | SPATA9 | -2,384869671 | -3,037500868 | 0,652631197 | |
| 233126_s_at | OLAH | -2,384869671 | -3,037500868 | 0,652631197 | |
| 234600_at | - | -2,384869671 | -3,037500868 | 0,652631197 | |
| 235947_at | - | -2,384869671 | -3,037500868 | 0,652631197 | |
| 207914_x_at | EVX1 | -0,078265071 | -0,730778808 | 0,652513737 | |
| 209133_s_at | COMMD4 | -0,078265071 | -0,730778808 | 0,652513737 | |
| 228518_at | IGHG1 /// IGHM | -0,078265071 | -0,730778808 | 0,652513737 | |
| 1559478_at | - | -0,259832361 | -0,912331589 | 0,652499228 | |
| 1562586_at | - | -0,259832361 | -0,912331589 | 0,652499228 | |
| 1570347_at | MLX | -0,259832361 | -0,912331589 | 0,652499228 | |
| 213401_s_at | TBL1X | -0,259832361 | -0,912331589 | 0,652499228 | |
| 235108_at | KCNK3 | -0,259832361 | -0,912331589 | 0,652499228 | |
| 208958_at | ERP44 | -0,299034815 | -0,950686014 | 0,6516512 | |
| 218934_s_at | HSPB7 | -0,299034815 | -0,950686014 | 0,6516512 | |
| 219294_at | CENPQ | 2,194816625 | 1,543943227 | 0,650873398 | |
| 239143_x_at | RNF138 | 2,247975581 | 1,59747851 | 0,650497071 | |
| 216017_s_at | NAB2 | 0,57058931 | -0,079522948 | 0,650112258 | |
| 228634_s_at | CSDA | 0,083054335 | -0,566887459 | 0,649941794 | |
| 231919_at | DBT | 0,083054335 | -0,566887459 | 0,649941794 | |
| 204106_at | TESK1 | 1,027166904 | 0,377530647 | 0,649636258 | |
| 222459_at | AKIRIN1 | 4,313236089 | 3,663961904 | 0,649274185 | |
| 223292_s_at | MRPS15 | 0,728550685 | 0,079428135 | 0,64912255 | |
| 207553_at | OPRK1 | -1,089016378 | -1,738067356 | 0,649050978 | |
| 210436_at | CCT8 | -1,089016378 | -1,738067356 | 0,649050978 | |
| 214880_x_at | CALD1 | -1,089016378 | -1,738067356 | 0,649050978 | |
| 219851_at | ZNF613 | -1,089016378 | -1,738067356 | 0,649050978 | |
| 221422_s_at | MIR600 /// MIR60 | -1,089016378 | -1,738067356 | 0,649050978 | |
| 223127_s_at | C1orf21 | -1,089016378 | -1,738067356 | 0,649050978 | |
| 230060_at | CDCA7 | -1,089016378 | -1,738067356 | 0,649050978 | |
| 230854_at | BCAR4 | -1,089016378 | -1,738067356 | 0,649050978 | |
| 233403_x_at | TM6SF2 | -1,089016378 | -1,738067356 | 0,649050978 | |
| 236417_at | - | -1,089016378 | -1,738067356 | 0,649050978 | |
| 236810_at | - | -1,089016378 | -1,738067356 | 0,649050978 | |
| 240566_at | - | -1,089016378 | -1,738067356 | 0,649050978 | |
| 244361_at | - | -1,089016378 | -1,738067356 | 0,649050978 | |
| 228835_at | - | 0,87091093 | 0,222599095 | 0,648311835 | |
| 231011_at | LARP1B | 0,67554678 | 0,028364014 | 0,647182766 | |
| 215452_x_at | SUMO4 | 4,234684448 | 3,587527995 | 0,647156453 | |
| 1561354_at | - | -1,639579028 | -2,286583451 | 0,647004422 | |
| 1561670_at | - | -1,639579028 | -2,286583451 | 0,647004422 | |
| 1562979_at | - | -1,639579028 | -2,286583451 | 0,647004422 | |
| 234043_at | - | -1,639579028 | -2,286583451 | 0,647004422 | |
| 235098_at | PEX26 | -1,639579028 | -2,286583451 | 0,647004422 | |
| 236668_at | - | -1,639579028 | -2,286583451 | 0,647004422 | |
| 240552_at | - | -1,639579028 | -2,286583451 | 0,647004422 | |
| 241123_at | - | -1,639579028 | -2,286583451 | 0,647004422 | |
| 1569044_at | CDC42BPG | -1,688127507 | -2,335104118 | 0,646976611 | |
| 207325_x_at | MAGEA1 | -1,688127507 | -2,335104118 | 0,646976611 | |
| 207800_at | AKAP5 | -1,688127507 | -2,335104118 | 0,646976611 | |
| 215917_at | RAD21L1 | -1,688127507 | -2,335104118 | 0,646976611 | |
| 217204_at | MTRF1L | -1,688127507 | -2,335104118 | 0,646976611 | |
| 220831_at | GCNT4 | -1,688127507 | -2,335104118 | 0,646976611 | |
| 230757_at | - | -1,688127507 | -2,335104118 | 0,646976611 | |

| | | | | | |
|---|---|---|---|---|---|
| 234166_at | UBE3A | -1,688127507 | -2,335104118 | 0,646976611 | |
| 234689_at | PTCHD4 | -1,688127507 | -2,335104118 | 0,646976611 | |
| 244275_at | CISD2 | -1,688127507 | -2,335104118 | 0,646976611 | |
| 1566136_at | - | -0,172274514 | -0,819238336 | 0,646963822 | |
| 203509_at | SORL1 | -0,172274514 | -0,819238336 | 0,646963822 | |
| 1570402_at | KLC3 | 0,822971223 | 0,176436073 | 0,64653515 | |
| 221765_at | UGCG | 1,481627361 | 0,835239052 | 0,646388309 | |
| 1560542_at | MCM3AP-AS1 | -2,089474779 | -2,735862426 | 0,646387647 | |
| 220493_at | DMRT1 | -2,089474779 | -2,735862426 | 0,646387647 | |
| 233954_at | HIATL1 | -2,089474779 | -2,735862426 | 0,646387647 | |
| 239380_at | C5orf27 | -2,089474779 | -2,735862426 | 0,646387647 | |
| 204876_at | ZNF646 | -0,033456564 | -0,679763839 | 0,646307275 | |
| 205846_at | PTPRB | -0,033456564 | -0,679763839 | 0,646307275 | |
| 210673_x_at | NKX2-1 | -0,033456564 | -0,679763839 | 0,646307275 | |
| 224424_x_at | ACTR3BP2 /// AC | -0,033456564 | -0,679763839 | 0,646307275 | |
| 227336_at | DTX1 | -0,033456564 | -0,679763839 | 0,646307275 | |
| 1566145_s_at | LOC644450 | -0,00073712 | -0,646746079 | 0,646008958 | |
| 232293_at | LCORL | 1,063722698 | 0,417770464 | 0,645952234 | |
| 221987_s_at | TSR1 | 3,262904562 | 2,617011906 | 0,645892656 | |
| 1557827_at | DNAJC9-AS1 | -0,467091869 | -1,112851844 | 0,645759975 | |
| 243861_at | - | -0,467091869 | -1,112851844 | 0,645759975 | |
| 206785_s_at | KLRC1 /// KLRC2 | -0,57445847 | -1,220210588 | 0,645752118 | |
| 214178_s_at | SOX2 | -0,57445847 | -1,220210588 | 0,645752118 | |
| 215927_at | ARFGEF2 | -0,57445847 | -1,220210588 | 0,645752118 | |
| 219901_at | FGD6 | -0,57445847 | -1,220210588 | 0,645752118 | |
| 223874_at | ACTR3C | -0,57445847 | -1,220210588 | 0,645752118 | |
| 235805_at | - | -0,57445847 | -1,220210588 | 0,645752118 | |
| 242887_at | KCMF1 | -0,57445847 | -1,220210588 | 0,645752118 | |
| 228474_s_at | KLF9 | 0,03125738 | -0,614371577 | 0,645628957 | |
| 1552952_at | RBMY2FP | -1,763254337 | -2,408705138 | 0,645450801 | |
| 1553736_at | ZFC3H1 | -1,763254337 | -2,408705138 | 0,645450801 | |
| 1560222_at | - | -1,763254337 | -2,408705138 | 0,645450801 | |
| 1566688_at | - | -1,763254337 | -2,408705138 | 0,645450801 | |
| 1568888_at | LOC100507283 | -1,763254337 | -2,408705138 | 0,645450801 | |
| 1569954_at | - | -1,763254337 | -2,408705138 | 0,645450801 | |
| 226814_at | ADAMTS9 | -1,763254337 | -2,408705138 | 0,645450801 | |
| 230014_at | - | -1,763254337 | -2,408705138 | 0,645450801 | |
| 240245_at | - | -1,763254337 | -2,408705138 | 0,645450801 | |
| 1558473_at | - | -1,518072576 | -2,162639771 | 0,644567196 | |
| 213094_at | GPR126 | -1,518072576 | -2,162639771 | 0,644567196 | |
| 214277_at | COX11 | -1,518072576 | -2,162639771 | 0,644567196 | |
| 216979_at | NR4A3 | -1,518072576 | -2,162639771 | 0,644567196 | |
| 220695_at | - | -1,518072576 | -2,162639771 | 0,644567196 | |
| 233794_at | - | -1,518072576 | -2,162639771 | 0,644567196 | |
| 234666_at | - | -1,518072576 | -2,162639771 | 0,644567196 | |
| 235283_at | INTS6 | -1,518072576 | -2,162639771 | 0,644567196 | |
| 243792_x_at | PTPN13 | -1,518072576 | -2,162639771 | 0,644567196 | |
| 1566804_at | - | -1,812418401 | -2,456406426 | 0,643988025 | |
| 211163_s_at | TNFRSF10C | -1,812418401 | -2,456406426 | 0,643988025 | |
| 217013_at | AZGP1P1 | -1,812418401 | -2,456406426 | 0,643988025 | |
| 222344_at | - | -1,812418401 | -2,456406426 | 0,643988025 | |
| 231786_at | HOXA13 | -1,812418401 | -2,456406426 | 0,643988025 | |
| 232695_at | KIF6 | -1,812418401 | -2,456406426 | 0,643988025 | |
| 242325_at | YWHAH | -1,812418401 | -2,456406426 | 0,643988025 | |
| 210926_at | POTEKP | 1,968405881 | 1,324440845 | 0,643965036 | |
| 1552334_at | TRIOBP | -0,85085127 | -1,494668682 | 0,643817412 | |

| | | | | | |
|---|---|---|---|---|---|
| 1562772_a_at | DAND5 | -0,85085127 | -1,494668682 | 0,643817412 | |
| 208199_s_at | ZFP161 | -0,85085127 | -1,494668682 | 0,643817412 | |
| 208274_at | OCLM | -0,85085127 | -1,494668682 | 0,643817412 | |
| 214267_s_at | CADM4 | -0,85085127 | -1,494668682 | 0,643817412 | |
| 216137_s_at | MAPK8IP3 | -0,85085127 | -1,494668682 | 0,643817412 | |
| 220541_at | MMP26 | -0,85085127 | -1,494668682 | 0,643817412 | |
| 233643_at | FAM86B1 /// FAM | -0,85085127 | -1,494668682 | 0,643817412 | |
| 243443_at | PPME1 | -0,85085127 | -1,494668682 | 0,643817412 | |
| 207220_at | ART4 | 0,359971644 | -0,283667828 | 0,643639471 | |
| 227751_at | PDCD5 | 1,593009234 | 0,949447784 | 0,64356145 | |
| 213529_at | ZNF688 | -0,366776884 | -1,010192375 | 0,643415491 | |
| 214164_x_at | CA12 | -0,366776884 | -1,010192375 | 0,643415491 | |
| 230535_s_at | ATP5E | -0,366776884 | -1,010192375 | 0,643415491 | |
| 230662_at | RNF187 | -0,366776884 | -1,010192375 | 0,643415491 | |
| 234883_x_at | - | -0,366776884 | -1,010192375 | 0,643415491 | |
| 243553_x_at | TRAF3IP2-AS1 | -0,366776884 | -1,010192375 | 0,643415491 | |
| 233423_at | KIAA1609 | -2,311268652 | -2,954576755 | 0,643308104 | |
| 240601_at | - | -2,311268652 | -2,954576755 | 0,643308104 | |
| 241057_x_at | - | -2,311268652 | -2,954576755 | 0,643308104 | |
| 236906_x_at | IPO5 | 0,702292136 | 0,059218869 | 0,643073268 | |
| 208037_s_at | MADCAM1 | 0,384950573 | -0,258114234 | 0,643064807 | |
| 1552410_at | CLEC4F | 0,181384709 | -0,461594427 | 0,642979136 | |
| 220412_x_at | KCNK7 | 0,181384709 | -0,461594427 | 0,642979136 | |
| 230769_at | DENND2C | 0,181384709 | -0,461594427 | 0,642979136 | |
| 240576_at | TBC1D26 /// ZNF | 0,181384709 | -0,461594427 | 0,642979136 | |
| 1552928_s_at | TAB3 | -0,512251684 | -1,154897679 | 0,642645994 | |
| 235148_at | KRTCAP3 | -0,512251684 | -1,154897679 | 0,642645994 | |
| 238023_at | - | -0,512251684 | -1,154897679 | 0,642645994 | |
| 231146_at | FAM24B | 2,675004741 | 2,032717854 | 0,642286888 | |
| 210568_s_at | RECQL | 1,795990708 | 1,153866761 | 0,642123946 | |
| 1560821_at | ARHGAP22 | -2,138804305 | -2,780313924 | 0,641509619 | |
| 1565613_at | - | -2,138804305 | -2,780313924 | 0,641509619 | |
| 217384_x_at | IGHV3-48 | -2,138804305 | -2,780313924 | 0,641509619 | |
| 220828_s_at | FLJ11292 | -2,138804305 | -2,780313924 | 0,641509619 | |
| 235356_at | NHLRC2 | -2,138804305 | -2,780313924 | 0,641509619 | |
| 238364_x_at | GLI4 | -2,138804305 | -2,780313924 | 0,641509619 | |
| 243566_at | LOC100506238 | -2,138804305 | -2,780313924 | 0,641509619 | |
| 207488_at | - | -0,622910612 | -1,264005341 | 0,641094728 | |
| 215426_at | ZCCHC14 | -0,622910612 | -1,264005341 | 0,641094728 | |
| 223603_at | RNF112 | -0,622910612 | -1,264005341 | 0,641094728 | |
| 244020_at | - | -0,622910612 | -1,264005341 | 0,641094728 | |
| 225721_at | SYNPO2 | -0,089689931 | -0,730778808 | 0,641088877 | |
| 232048_at | FAM76B | 0,648296226 | 0,007421914 | 0,640874312 | |
| 205762_s_at | DUS4L | 0,291166226 | -0,34958478 | 0,640751006 | |
| 217541_x_at | ZNF816 /// ZNF8 | 0,291166226 | -0,34958478 | 0,640751006 | |
| 216380_x_at | - | 3,986661073 | 3,346162854 | 0,640498219 | |
| 226704_at | UBE2J2 | 0,072842263 | -0,566887459 | 0,639729722 | |
| 1558557_at | C16orf62 | -2,597496523 | -3,236070201 | 0,638573678 | |
| 237904_at | - | -2,597496523 | -3,236070201 | 0,638573678 | |
| 218502_s_at | TRPS1 | 0,592200562 | -0,046302147 | 0,638502708 | |
| 1552908_at | C1orf150 | 0,767063567 | 0,128747141 | 0,638316426 | |
| 216302_at | HNRNPCL1 /// LO | 0,767063567 | 0,128747141 | 0,638316426 | |
| 234859_at | PLXNA4 | 0,417596971 | -0,220592223 | 0,638189194 | |
| 223263_s_at | FGFR1OP2 | 1,296557217 | 0,658444279 | 0,638112938 | |
| 1552842_at | HS6ST3 /// LOC1 | -1,399512875 | -2,037551727 | 0,638038851 | |
| 1554295_x_at | TTBK2 | -1,399512875 | -2,037551727 | 0,638038851 | |

| | | | | | |
|---|---|---|---|---|---|
| 1566874_at | - | -1,399512875 | -2,037551727 | 0,638038851 | |
| 1569833_at | - | -1,399512875 | -2,037551727 | 0,638038851 | |
| 200878_at | EPAS1 /// LOC10 | -1,399512875 | -2,037551727 | 0,638038851 | |
| 214465_at | ORM1 /// ORM2 | -1,399512875 | -2,037551727 | 0,638038851 | |
| 219612_s_at | FGG | -1,399512875 | -2,037551727 | 0,638038851 | |
| 223897_at | ZNF765 | -1,399512875 | -2,037551727 | 0,638038851 | |
| 242258_at | - | -1,399512875 | -2,037551727 | 0,638038851 | |
| 243445_at | BNC2 | -1,399512875 | -2,037551727 | 0,638038851 | |
| 227444_at | ARMCX4 | 1,191926657 | 0,553993624 | 0,637933032 | |
| 224971_at | C2orf15 /// MRPL | 4,173278271 | 3,535445117 | 0,637833154 | |
| 1556414_at | LINC00515 | -1,937711681 | -2,575129729 | 0,637418048 | |
| 1563389_at | LOC100506030 | -1,937711681 | -2,575129729 | 0,637418048 | |
| 210687_at | CPT1A | -1,937711681 | -2,575129729 | 0,637418048 | |
| 222188_at | C9orf156 | -1,937711681 | -2,575129729 | 0,637418048 | |
| 242976_at | - | -1,937711681 | -2,575129729 | 0,637418048 | |
| 244531_at | NNT | -1,937711681 | -2,575129729 | 0,637418048 | |
| 1554290_at | HERC3 | -2,359436556 | -2,996335703 | 0,636899147 | |
| 1570534_a_at | ZNF483 | -2,359436556 | -2,996335703 | 0,636899147 | |
| 1552637_at | PTPN11 | -1,026571149 | -1,663414495 | 0,636843346 | |
| 1553447_at | AGBL1 | -1,026571149 | -1,663414495 | 0,636843346 | |
| 1555277_a_at | SLC4A5 | -1,026571149 | -1,663414495 | 0,636843346 | |
| 211659_at | GPR135 | -1,026571149 | -1,663414495 | 0,636843346 | |
| 222081_at | SIRT5 | -1,026571149 | -1,663414495 | 0,636843346 | |
| 231064_s_at | - | -1,026571149 | -1,663414495 | 0,636843346 | |
| 232782_at | - | -1,026571149 | -1,663414495 | 0,636843346 | |
| 235684_s_at | SESN3 | -1,026571149 | -1,663414495 | 0,636843346 | |
| 244496_at | - | -1,026571149 | -1,663414495 | 0,636843346 | |
| 244572_at | KY | -1,026571149 | -1,663414495 | 0,636843346 | |
| 225726_s_at | PLEKHH1 | 0,859074518 | 0,222599095 | 0,636475424 | |
| 1554625_at | BCL6B | 0,695652136 | 0,059218869 | 0,636433268 | |
| 224441_s_at | USP45 | 0,715481152 | 0,079428135 | 0,636053016 | |
| 211671_s_at | NR3C1 | 3,078700536 | 2,442710777 | 0,635989759 | |
| 40524_at | PTPN21 | -1,612820892 | -2,248681741 | 0,635860849 | |
| 218929_at | CDKN2AIP | 2,344516784 | 1,708698295 | 0,635818489 | |
| 1559169_at | LOC100507013 | -0,741668563 | -1,377419394 | 0,635750831 | |
| 1563512_at | NOS1AP | -0,741668563 | -1,377419394 | 0,635750831 | |
| 205612_at | MMRN1 | -0,741668563 | -1,377419394 | 0,635750831 | |
| 206215_at | OPCML | -0,741668563 | -1,377419394 | 0,635750831 | |
| 214846_s_at | ALPK3 | -0,741668563 | -1,377419394 | 0,635750831 | |
| 219842_at | ARL15 | -0,741668563 | -1,377419394 | 0,635750831 | |
| 224388_s_at | COL25A1 | -0,741668563 | -1,377419394 | 0,635750831 | |
| 230684_at | GTPBP10 | -0,741668563 | -1,377419394 | 0,635750831 | |
| 237048_at | - | 0,555999837 | -0,079522948 | 0,635522785 | |
| 230185_at | THAP9 | 0,928678725 | 0,293518836 | 0,635159889 | |
| 201069_at | MMP2 | 0,376672223 | -0,258114234 | 0,634786457 | |
| 212575_at | C19orf6 | 1,129248302 | 0,495031851 | 0,634216451 | |
| 235338_s_at | SETDB2 | 1,390002893 | 0,755840599 | 0,634162294 | |
| 219154_at | TMEM120B | -0,221656139 | -0,855751026 | 0,634094888 | |
| 227683_x_at | NUDT4 | -0,221656139 | -0,855751026 | 0,634094888 | |
| 228251_at | UBXN6 | -0,221656139 | -0,855751026 | 0,634094888 | |
| 243537_at | - | -0,221656139 | -0,855751026 | 0,634094888 | |
| 231674_at | - | -2,504600601 | -3,138626879 | 0,634026278 | |
| 232325_at | - | -2,504600601 | -3,138626879 | 0,634026278 | |
| 219482_at | SETD4 | 1,305306849 | 0,67181667 | 0,633490179 | |
| 206929_s_at | NFIC | 0,171847695 | -0,461594427 | 0,633442122 | |
| 239525_at | CTTNBP2NL | 0,171847695 | -0,461594427 | 0,633442122 | |

| | | | | | |
|---|---|---|---|---|---|
| 206469_x_at | AKR7A3 | 1,027166904 | 0,393761504 | 0,6334054 | |
| 1553244_at | FANCB | 0,882651021 | 0,249604176 | 0,633046845 | |
| 216114_at | NCKIPSD | 0,496120222 | -0,136643983 | 0,632764205 | |
| 202769_at | CCNG2 | 3,167755526 | 2,53523821 | 0,632517316 | |
| 235432_at | NPHP3 | 0,760715727 | 0,128747141 | 0,631968586 | |
| 241285_at | - | 0,30867765 | -0,322870281 | 0,631547931 | |
| 232950_s_at | PITPNM2 | 1,063722698 | 0,433557298 | 0,6301654 | |
| 217473_x_at | SLC11A1 | 1,233777334 | 0,603679647 | 0,630097687 | |
| 214835_s_at | SUCLG2 | 2,901063789 | 2,271023131 | 0,630040658 | |
| 205524_s_at | HAPLN1 | -0,59053611 | -1,220210588 | 0,629674477 | |
| 210341_at | MYT1 | -0,59053611 | -1,220210588 | 0,629674477 | |
| 224461_s_at | AIFM2 | -0,59053611 | -1,220210588 | 0,629674477 | |
| 226494_at | CAMSAP3 | -0,59053611 | -1,220210588 | 0,629674477 | |
| 231893_at | KIAA1755 | -0,59053611 | -1,220210588 | 0,629674477 | |
| 236593_at | - | -0,59053611 | -1,220210588 | 0,629674477 | |
| 243437_at | GCC1 | -0,59053611 | -1,220210588 | 0,629674477 | |
| 225872_at | SLC35F5 | 3,769898242 | 3,140317145 | 0,629581097 | |
| 213548_s_at | CDV3 | 2,254767157 | 1,6252386 | 0,629528557 | |
| 1561762_s_at | - | -0,136301282 | -0,765504029 | 0,629202747 | |
| 206371_at | FOLR3 | -0,136301282 | -0,765504029 | 0,629202747 | |
| 238396_at | - | -0,136301282 | -0,765504029 | 0,629202747 | |
| 204222_s_at | GLIPR1 | 1,709647566 | 1,080561626 | 0,62908594 | |
| 1569583_at | EREG | -0,986356909 | -1,615132408 | 0,628775499 | |
| 213962_s_at | ANKLE2 | -0,986356909 | -1,615132408 | 0,628775499 | |
| 219252_s_at | GEMIN8 | -0,986356909 | -1,615132408 | 0,628775499 | |
| 221885_at | DENND2A | -0,986356909 | -1,615132408 | 0,628775499 | |
| 236257_at | CD2AP | -0,986356909 | -1,615132408 | 0,628775499 | |
| 237486_at | - | -0,986356909 | -1,615132408 | 0,628775499 | |
| 238759_at | CCDC88A | -0,986356909 | -1,615132408 | 0,628775499 | |
| 244219_at | - | -0,986356909 | -1,615132408 | 0,628775499 | |
| 208762_at | SUMO1 | 1,965648824 | 1,337086025 | 0,628562799 | |
| 204516_at | ATXN7 | 2,243430036 | 1,614891062 | 0,628538975 | |
| 238290_at | - | 0,209623097 | -0,41858459 | 0,628207687 | |
| 202027_at | TMEM184B | 2,586251598 | 1,958277216 | 0,627974382 | |
| 1558450_at | A2M | -0,79540287 | -1,423348341 | 0,627945471 | |
| 1561392_at | - | -0,79540287 | -1,423348341 | 0,627945471 | |
| 222295_x_at | - | -0,79540287 | -1,423348341 | 0,627945471 | |
| 237320_at | FAM71F2 | -0,79540287 | -1,423348341 | 0,627945471 | |
| 1552740_at | C2orf15 /// MRPL | -2,063149277 | -2,690860744 | 0,627711466 | |
| 1552899_at | MGC34034 | -2,063149277 | -2,690860744 | 0,627711466 | |
| 1554889_at | TIA1 | -2,063149277 | -2,690860744 | 0,627711466 | |
| 1556204_a_at | LOC100506523 / | -2,063149277 | -2,690860744 | 0,627711466 | |
| 205765_at | CYP3A5 | -2,063149277 | -2,690860744 | 0,627711466 | |
| 217669_s_at | AKAP6 | -2,063149277 | -2,690860744 | 0,627711466 | |
| 1563612_at | - | -1,2845686 | -1,912089812 | 0,627521212 | |
| 208455_at | PVRL1 | -1,2845686 | -1,912089812 | 0,627521212 | |
| 213732_at | TCF3 | -1,2845686 | -1,912089812 | 0,627521212 | |
| 236459_at | PRKCE | -1,2845686 | -1,912089812 | 0,627521212 | |
| 237120_at | KRT77 | -1,2845686 | -1,912089812 | 0,627521212 | |
| 238211_at | LOC643714 | -1,2845686 | -1,912089812 | 0,627521212 | |
| 239255_at | - | -1,2845686 | -1,912089812 | 0,627521212 | |
| 1556834_at | LOC100652770 | -2,262747984 | -2,89013181 | 0,627383826 | |
| 1560147_at | WDR86-AS1 | -2,262747984 | -2,89013181 | 0,627383826 | |
| 216814_at | - | -2,262747984 | -2,89013181 | 0,627383826 | |
| 238705_at | - | -2,262747984 | -2,89013181 | 0,627383826 | |
| 241856_at | IMPG2 | -2,262747984 | -2,89013181 | 0,627383826 | |

| | | | | | |
|---|---|---|---|---|---|
| 240803_at | - | -0,423248115 | -1,050406615 | 0,6271585 | |
| 242624_at | ABLIM2 | -0,423248115 | -1,050406615 | 0,6271585 | |
| 224027_at | CCL28 | -2,551294263 | -3,177802286 | 0,626508023 | |
| 214701_s_at | FN1 | -0,285837228 | -0,912331589 | 0,626494361 | |
| 220576_at | PGAP1 | -0,285837228 | -0,912331589 | 0,626494361 | |
| 236441_at | - | -0,285837228 | -0,912331589 | 0,626494361 | |
| 202843_at | DNAJB9 | 1,655931423 | 1,029536893 | 0,62639453 | |
| 211220_s_at | HSF2 | 1,381753566 | 0,755840599 | 0,625912967 | |
| 217921_at | MAN1A2 | 0,46522187 | -0,160136748 | 0,625358619 | |
| 219774_at | CCDC93 | 0,46522187 | -0,160136748 | 0,625358619 | |
| 1557589_a_at | - | -0,706943342 | -1,332017329 | 0,625073987 | |
| 209483_s_at | NSL1 | -0,706943342 | -1,332017329 | 0,625073987 | |
| 211113_s_at | ABCG1 | -0,706943342 | -1,332017329 | 0,625073987 | |
| 223537_s_at | WNT5B | -0,706943342 | -1,332017329 | 0,625073987 | |
| 227532_at | LRRC39 | -0,706943342 | -1,332017329 | 0,625073987 | |
| 227795_at | NDUFV1 | -0,706943342 | -1,332017329 | 0,625073987 | |
| 236425_at | - | -0,706943342 | -1,332017329 | 0,625073987 | |
| 240169_at | - | -0,706943342 | -1,332017329 | 0,625073987 | |
| 232249_at | FMNL3 | -0,325749314 | -0,950686014 | 0,6249367 | |
| 236546_at | POLA2 | -0,325749314 | -0,950686014 | 0,6249367 | |
| 218525_s_at | HIF1AN | 1,940595304 | 1,315948728 | 0,624646576 | |
| 1570315_at | HTA | -0,022466681 | -0,646746079 | 0,624279398 | |
| 202091_at | LOC100652989 / | -0,022466681 | -0,646746079 | 0,624279398 | |
| 214133_at | MUC6 | -0,022466681 | -0,646746079 | 0,624279398 | |
| 219340_s_at | CLN8 | 1,62481334 | 1,000684521 | 0,624128819 | |
| 238071_at | LCN10 /// LCN6 | -0,055687482 | -0,679763839 | 0,624076357 | |
| 214666_x_at | IREB2 | 1,360921491 | 0,73688026 | 0,624041231 | |
| 211305_x_at | FCAR | 0,133056859 | -0,490927335 | 0,623984194 | |
| 232240_at | DNHD1 | 0,273439642 | -0,34958478 | 0,623024422 | |
| 230604_at | - | 0,702292136 | 0,079428135 | 0,622864001 | |
| 205442_at | MFAP3L | 1,859746653 | 1,23718272 | 0,622563933 | |
| 224548_at | HES7 | -0,196756757 | -0,819238336 | 0,622481579 | |
| 235331_x_at | PCGF5 | -0,196756757 | -0,819238336 | 0,622481579 | |
| 1554364_at | PPP2R5C | -1,240169874 | -1,862647763 | 0,622477888 | |
| 1555898_at | ANKRD36C | -1,240169874 | -1,862647763 | 0,622477888 | |
| 1559109_a_at | VPS53 | -1,240169874 | -1,862647763 | 0,622477888 | |
| 1561194_at | - | -1,240169874 | -1,862647763 | 0,622477888 | |
| 210117_at | SPAG1 | -1,240169874 | -1,862647763 | 0,622477888 | |
| 230465_at | HS2ST1 | -1,240169874 | -1,862647763 | 0,622477888 | |
| 232308_at | TTC21A | -1,240169874 | -1,862647763 | 0,622477888 | |
| 235972_at | TMEM131 | -1,240169874 | -1,862647763 | 0,622477888 | |
| 236509_at | - | -1,240169874 | -1,862647763 | 0,622477888 | |
| 237051_at | - | -1,240169874 | -1,862647763 | 0,622477888 | |
| 237291_at | PRORSD1P | -1,240169874 | -1,862647763 | 0,622477888 | |
| 241074_at | - | -1,240169874 | -1,862647763 | 0,622477888 | |
| 212467_at | DNAJC13 | 2,693452197 | 2,071071048 | 0,622381149 | |
| 225134_at | SPRYD3 | 0,779676066 | 0,157549243 | 0,622126823 | |
| 212920_at | REST | 3,674351634 | 3,052311115 | 0,622040519 | |
| 205577_at | PYGM | -0,234278768 | -0,855751026 | 0,621472259 | |
| 208041_at | GRK1 | -0,234278768 | -0,855751026 | 0,621472259 | |
| 236145_at | - | -0,234278768 | -0,855751026 | 0,621472259 | |
| 230759_at | SNX14 | 1,210676994 | 0,589657387 | 0,621019607 | |
| 216582_at | POM121L2 | 0,518867317 | -0,102100538 | 0,620967855 | |
| 1563362_at | D21S2090E | -1,71423189 | -2,335104118 | 0,620872228 | |
| 219985_at | HS3ST3A1 | -1,71423189 | -2,335104118 | 0,620872228 | |
| 222834_s_at | GNG12 | -1,71423189 | -2,335104118 | 0,620872228 | |

| | | | | | |
|---|---|---|---|---|---|
| 223973_at | MIR7-3HG | -1,71423189 | -2,335104118 | 0,620872228 | |
| 238195_at | - | -1,71423189 | -2,335104118 | 0,620872228 | |
| 211382_s_at | TACC2 | 0,541261317 | -0,079522948 | 0,620784265 | |
| 207781_s_at | ZNF711 | 1,496984567 | 0,876248481 | 0,620736086 | |
| 204037_at | LPAR1 | -1,591296941 | -2,211750327 | 0,620453386 | |
| 208026_at | HIST1H4A /// HIS | -1,591296941 | -2,211750327 | 0,620453386 | |
| 217323_at | HLA-DRB6 | -1,591296941 | -2,211750327 | 0,620453386 | |
| 233866_at | KLHL5 | -1,591296941 | -2,211750327 | 0,620453386 | |
| 234387_at | COL4A5 | -1,591296941 | -2,211750327 | 0,620453386 | |
| 235851_s_at | GNAS | -1,591296941 | -2,211750327 | 0,620453386 | |
| 236741_at | WDR72 | -1,591296941 | -2,211750327 | 0,620453386 | |
| 237983_at | - | -1,591296941 | -2,211750327 | 0,620453386 | |
| 239057_at | LMOD2 | -1,591296941 | -2,211750327 | 0,620453386 | |
| 244679_at | - | -1,591296941 | -2,211750327 | 0,620453386 | |
| 215919_s_at | MRPS11 | 0,627515113 | 0,007421914 | 0,620093199 | |
| 205479_s_at | PLAU | 3,205636958 | 2,585563171 | 0,620073787 | |
| 228744_at | HAUS2 | 2,038300149 | 1,418668082 | 0,619632067 | |
| 206811_at | ADCY8 | -1,543331864 | -2,162639771 | 0,619307907 | |
| 208356_x_at | CSH1 | -1,543331864 | -2,162639771 | 0,619307907 | |
| 216864_at | - | -1,543331864 | -2,162639771 | 0,619307907 | |
| 222953_at | GPR83 | -1,543331864 | -2,162639771 | 0,619307907 | |
| 233074_at | - | -1,543331864 | -2,162639771 | 0,619307907 | |
| 240557_at | - | -1,543331864 | -2,162639771 | 0,619307907 | |
| 243117_at | - | -1,543331864 | -2,162639771 | 0,619307907 | |
| 1553698_a_at | CCSAP | 0,083054335 | -0,536087151 | 0,619141486 | |
| 1565903_at | - | 0,083054335 | -0,536087151 | 0,619141486 | |
| 230852_at | STAC3 | 0,083054335 | -0,536087151 | 0,619141486 | |
| 237754_at | LOC100506559 | 0,052199481 | -0,566887459 | 0,61908694 | |
| 215133_s_at | FAM153A /// FAM | -2,479842156 | -3,098858316 | 0,61901616 | |
| 211660_at | POU2F2 | 0,20027154 | -0,41858459 | 0,618856129 | |
| 232751_at | RBBP9 | 0,20027154 | -0,41858459 | 0,618856129 | |
| 202066_at | PPFIA1 | 3,308224834 | 2,689475799 | 0,618749035 | |
| 36499_at | CELSR2 | 0,228150971 | -0,390416179 | 0,61856715 | |
| 218043_s_at | AZI2 | 0,841135803 | 0,222599095 | 0,618536708 | |
| 224204_x_at | ARNTL2 | 0,334552598 | -0,283667828 | 0,618220426 | |
| 228161_at | RAB32 | 0,334552598 | -0,283667828 | 0,618220426 | |
| 221215_s_at | RIPK4 | 0,359971644 | -0,258114234 | 0,618085878 | |
| 227351_at | C16orf52 | 0,359971644 | -0,258114234 | 0,618085878 | |
| 235027_at | FLJ43489 | 2,589836943 | 1,971855016 | 0,617981927 | |
| 204778_x_at | HOXB7 | -0,112808516 | -0,730778808 | 0,617970292 | |
| 207280_at | RNF185-AS1 | -0,112808516 | -0,730778808 | 0,617970292 | |
| 216391_s_at | KLHL1 | -0,112808516 | -0,730778808 | 0,617970292 | |
| 231189_at | - | -0,112808516 | -0,730778808 | 0,617970292 | |
| 1559565_x_at | - | -0,759588698 | -1,377419394 | 0,617830696 | |
| 211798_x_at | IGLJ3 | -0,759588698 | -1,377419394 | 0,617830696 | |
| 243881_at | SHC3 | -0,759588698 | -1,377419394 | 0,617830696 | |
| 1552752_a_at | CADM2 | -1,838812296 | -2,456406426 | 0,61759413 | |
| 1552940_at | TEDDM1 | -1,838812296 | -2,456406426 | 0,61759413 | |
| 1556369_a_at | PHKG2 | -1,838812296 | -2,456406426 | 0,61759413 | |
| 1557166_at | PDCD4 | -1,838812296 | -2,456406426 | 0,61759413 | |
| 1558748_at | - | -1,838812296 | -2,456406426 | 0,61759413 | |
| 1560788_at | MYO3B | -1,838812296 | -2,456406426 | 0,61759413 | |
| 1568795_at | - | -1,838812296 | -2,456406426 | 0,61759413 | |
| 207790_at | LRRC1 | -1,838812296 | -2,456406426 | 0,61759413 | |
| 215391_at | MAP1A | -1,838812296 | -2,456406426 | 0,61759413 | |
| 215998_at | - | -1,838812296 | -2,456406426 | 0,61759413 | |

| | | | | | |
|---|---|---|---|---|---|
| 230752_at | - | -1,838812296 | -2,456406426 | 0,61759413 | |
| 233514_x_at | TEX11 | -1,838812296 | -2,456406426 | 0,61759413 | |
| 239585_at | - | -1,838812296 | -2,456406426 | 0,61759413 | |
| 241372_at | ZC3H6 | -1,838812296 | -2,456406426 | 0,61759413 | |
| 242894_at | - | -1,838812296 | -2,456406426 | 0,61759413 | |
| 243293_at | - | -1,838812296 | -2,456406426 | 0,61759413 | |
| 243826_at | - | -1,838812296 | -2,456406426 | 0,61759413 | |
| 210050_at | TPI1 | 0,457392764 | -0,160136748 | 0,617529513 | |
| 218712_at | C1orf109 | 2,688444421 | 2,071071048 | 0,617373373 | |
| 210804_x_at | SLC8A1 | -0,148196756 | -0,765504029 | 0,617307273 | |
| 227719_at | SMAD9 | -0,148196756 | -0,765504029 | 0,617307273 | |
| 239577_at | - | -0,148196756 | -0,765504029 | 0,617307273 | |
| 241556_at | - | -0,148196756 | -0,765504029 | 0,617307273 | |
| 223651_x_at | CDC23 | 1,481627361 | 0,864649967 | 0,616977394 | |
| 237002_at | NCDN | 0,592200562 | -0,024572586 | 0,616773148 | |
| 231252_at | KANSL1L | 0,695652136 | 0,079428135 | 0,616224001 | |
| 1555988_a_at | LOC126536 | -1,470833215 | -2,086984744 | 0,616151528 | |
| 1563805_a_at | FAM83C | -1,470833215 | -2,086984744 | 0,616151528 | |
| 1569743_at | - | -1,470833215 | -2,086984744 | 0,616151528 | |
| 204722_at | SCN3B | -1,470833215 | -2,086984744 | 0,616151528 | |
| 209763_at | CHRDL1 | -1,470833215 | -2,086984744 | 0,616151528 | |
| 227742_at | CLIC6 | -1,470833215 | -2,086984744 | 0,616151528 | |
| 231244_at | CASD1 | -1,470833215 | -2,086984744 | 0,616151528 | |
| 231482_at | - | -1,470833215 | -2,086984744 | 0,616151528 | |
| 232687_at | - | -1,470833215 | -2,086984744 | 0,616151528 | |
| 233925_at | - | -1,470833215 | -2,086984744 | 0,616151528 | |
| 242491_at | - | -1,470833215 | -2,086984744 | 0,616151528 | |
| 243336_at | LOC100132292 | -1,470833215 | -2,086984744 | 0,616151528 | |
| 201300_s_at | PRNP | 5,026966555 | 4,411005377 | 0,615961178 | |
| 207370_at | IBSP | -0,496991414 | -1,112851844 | 0,61586043 | |
| 218218_at | APPL2 | -0,496991414 | -1,112851844 | 0,61586043 | |
| 220289_s_at | AIM1L | -0,496991414 | -1,112851844 | 0,61586043 | |
| 236994_at | - | -0,496991414 | -1,112851844 | 0,61586043 | |
| 1556808_at | - | -1,047650486 | -1,663414495 | 0,615764009 | |
| 1558722_at | ZNF252P | -1,047650486 | -1,663414495 | 0,615764009 | |
| 1565818_s_at | IKZF1 | -1,047650486 | -1,663414495 | 0,615764009 | |
| 213921_at | SST | -1,047650486 | -1,663414495 | 0,615764009 | |
| 215876_at | - | -1,047650486 | -1,663414495 | 0,615764009 | |
| 235994_s_at | PLAC2 | -1,047650486 | -1,663414495 | 0,615764009 | |
| 239536_at | - | -1,047650486 | -1,663414495 | 0,615764009 | |
| 244771_at | KBTBD12 | -1,047650486 | -1,663414495 | 0,615764009 | |
| 209803_s_at | PHLDA2 | 2,886560922 | 2,271023131 | 0,61553779 | |
| 1557669_at | LOC100132005 | -0,394749123 | -1,010192375 | 0,615443252 | |
| 1559127_x_at | RRP12 | -0,394749123 | -1,010192375 | 0,615443252 | |
| 206122_at | SOX15 | -0,394749123 | -1,010192375 | 0,615443252 | |
| 215712_s_at | IGFALS | -0,394749123 | -1,010192375 | 0,615443252 | |
| 219776_s_at | - | -0,394749123 | -1,010192375 | 0,615443252 | |
| 236087_at | ABLIM2 | -0,394749123 | -1,010192375 | 0,615443252 | |
| 1555273_at | GALNTL6 | -1,888254346 | -2,503677622 | 0,615423277 | |
| 1558076_at | ANKRD32 | -1,888254346 | -2,503677622 | 0,615423277 | |
| 1561342_at | LOC150005 | -1,888254346 | -2,503677622 | 0,615423277 | |
| 207514_s_at | GNAT1 | -1,888254346 | -2,503677622 | 0,615423277 | |
| 231555_at | - | -1,888254346 | -2,503677622 | 0,615423277 | |
| 236774_at | - | -1,888254346 | -2,503677622 | 0,615423277 | |
| 236868_at | - | -1,888254346 | -2,503677622 | 0,615423277 | |
| 237932_at | - | -1,888254346 | -2,503677622 | 0,615423277 | |

| | | | | | |
|---|---|---|---|---|---|
| 216186_at | - | 1,462198073 | 0,847075464 | 0,61512261 | |
| 217554_at | - | -0,926850548 | -1,541908042 | 0,615057494 | |
| 236559_at | YWHAH | -0,926850548 | -1,541908042 | 0,615057494 | |
| 237833_s_at | SNCAIP | -0,926850548 | -1,541908042 | 0,615057494 | |
| 238248_at | - | -0,926850548 | -1,541908042 | 0,615057494 | |
| 243323_s_at | ZFHX3 | -0,926850548 | -1,541908042 | 0,615057494 | |
| 218224_at | PNMA1 | 4,893714114 | 4,279061697 | 0,614652417 | |
| 204635_at | RPS6KA5 | 1,094335495 | 0,479907041 | 0,614428454 | |
| 231975_s_at | MIER3 | 2,064305961 | 1,450051551 | 0,61425441 | |
| 211839_s_at | CSF1 | 0,291166226 | -0,322870281 | 0,614036507 | |
| 221707_s_at | VPS53 | -0,00073712 | -0,614371577 | 0,613634456 | |
| 225768_at | NR1D2 | -0,00073712 | -0,614371577 | 0,613634456 | |
| 240365_at | LOC647946 | -0,00073712 | -0,614371577 | 0,613634456 | |
| 224320_s_at | MCM8 | 1,859746653 | 1,246149888 | 0,613596765 | |
| 1557657_a_at | LOC400238 | -1,424023738 | -2,037551727 | 0,613527988 | |
| 1559656_a_at | LOC100507244 | -1,424023738 | -2,037551727 | 0,613527988 | |
| 1561257_at | LOC286083 | -1,424023738 | -2,037551727 | 0,613527988 | |
| 210195_s_at | PSG1 | -1,424023738 | -2,037551727 | 0,613527988 | |
| 216487_at | - | -1,424023738 | -2,037551727 | 0,613527988 | |
| 217222_at | IGHG1 | -1,424023738 | -2,037551727 | 0,613527988 | |
| 217539_at | C18orf25 | -1,424023738 | -2,037551727 | 0,613527988 | |
| 224385_s_at | MOV10L1 | -1,424023738 | -2,037551727 | 0,613527988 | |
| 229152_at | FDCSP | -1,424023738 | -2,037551727 | 0,613527988 | |
| 231077_at | C1orf192 | -1,424023738 | -2,037551727 | 0,613527988 | |
| 234324_at | NHSL1 | -1,424023738 | -2,037551727 | 0,613527988 | |
| 238889_at | AGBL5 | -1,424023738 | -2,037551727 | 0,613527988 | |
| 240389_at | TRPM6 | -1,424023738 | -2,037551727 | 0,613527988 | |
| 240823_at | - | -1,424023738 | -2,037551727 | 0,613527988 | |
| 241442_at | - | -1,424023738 | -2,037551727 | 0,613527988 | |
| 243334_at | CACNA1D | -1,424023738 | -2,037551727 | 0,613527988 | |
| 237792_at | - | 0,533835206 | -0,079522948 | 0,613358154 | |
| 1552969_a_at | ZMYM6 | -1,173767127 | -1,787089803 | 0,613322676 | |
| 206024_at | HPD | -1,173767127 | -1,787089803 | 0,613322676 | |
| 226989_at | RGMB | -1,173767127 | -1,787089803 | 0,613322676 | |
| 234592_at | - | -1,173767127 | -1,787089803 | 0,613322676 | |
| 242627_at | - | -1,173767127 | -1,787089803 | 0,613322676 | |
| 1569380_a_at | HERPUD1 | -0,299034815 | -0,912331589 | 0,613296774 | |
| 211634_x_at | IGHM | -0,299034815 | -0,912331589 | 0,613296774 | |
| 218207_s_at | STMN3 | -0,299034815 | -0,912331589 | 0,613296774 | |
| 221508_at | TAOK3 | -0,299034815 | -0,912331589 | 0,613296774 | |
| 229003_x_at | FAM69B | -0,299034815 | -0,912331589 | 0,613296774 | |
| 214488_at | RAP2B | -0,033456564 | -0,646746079 | 0,613289514 | |
| 232097_at | TOX4 | -0,033456564 | -0,646746079 | 0,613289514 | |
| 201755_at | MCM5 | 2,671625168 | 2,058399602 | 0,613225566 | |
| 227786_at | MED30 | 2,959046717 | 2,345926937 | 0,613119781 | |
| 225055_at | LOC100499466 | 0,641402394 | 0,028364014 | 0,61303838 | |
| 241904_at | - | 0,641402394 | 0,028364014 | 0,61303838 | |
| 1553288_a_at | NYAP1 | -0,43775896 | -1,050406615 | 0,612647655 | |
| 208252_s_at | CHST3 | -0,43775896 | -1,050406615 | 0,612647655 | |
| 217053_x_at | ETV1 | -0,43775896 | -1,050406615 | 0,612647655 | |
| 229904_at | - | -0,43775896 | -1,050406615 | 0,612647655 | |
| 231534_at | CDK1 | -0,43775896 | -1,050406615 | 0,612647655 | |
| 237585_at | C4orf47 | -0,43775896 | -1,050406615 | 0,612647655 | |
| 229501_s_at | USP8 | 1,64218396 | 1,029536893 | 0,612647067 | |
| 215783_s_at | ALPL | -0,543051993 | -1,154897679 | 0,611845686 | |
| 220988_s_at | C1QTNF3 | -0,543051993 | -1,154897679 | 0,611845686 | |

| | | | | | |
|---|---|---|---|---|---|
| 233298_at | CCDC169 | -0,543051993 | -1,154897679 | 0,611845686 | |
| 226090_x_at | RABL3 | 2,166283906 | 1,55481039 | 0,611473515 | |
| 1566832_at | TOP1P2 | -2,384869671 | -2,996335703 | 0,611466032 | |
| 208498_s_at | ACTG1P4 /// AMY | -2,384869671 | -2,996335703 | 0,611466032 | |
| 1563495_at | SLC9C2 | -1,964176657 | -2,575129729 | 0,610953072 | |
| 1564561_at | - | -1,964176657 | -2,575129729 | 0,610953072 | |
| 1566215_at | - | -1,964176657 | -2,575129729 | 0,610953072 | |
| 1566924_at | - | -1,964176657 | -2,575129729 | 0,610953072 | |
| 1570629_at | - | -1,964176657 | -2,575129729 | 0,610953072 | |
| 205959_at | MMP13 | -1,964176657 | -2,575129729 | 0,610953072 | |
| 207450_s_at | POU6F2 | -1,964176657 | -2,575129729 | 0,610953072 | |
| 215585_at | - | -1,964176657 | -2,575129729 | 0,610953072 | |
| 216628_at | - | -1,964176657 | -2,575129729 | 0,610953072 | |
| 1552933_at | AKNAD1 | -2,236765673 | -2,847001814 | 0,610236141 | |
| 1561339_at | - | -2,236765673 | -2,847001814 | 0,610236141 | |
| 226612_at | UBE2QL1 | -2,236765673 | -2,847001814 | 0,610236141 | |
| 240120_at | - | -2,236765673 | -2,847001814 | 0,610236141 | |
| 210733_at | - | 2,799441827 | 2,189694216 | 0,609747611 | |
| 1553668_at | LRCH3 | 0,351548291 | -0,258114234 | 0,609662525 | |
| 1556747_a_at | - | 1,027166904 | 0,417770464 | 0,609396441 | |
| 215256_x_at | ARHGAP33 | 0,072842263 | -0,536087151 | 0,608929413 | |
| 1556279_at | - | -0,655928373 | -1,264005341 | 0,608076968 | |
| 216269_s_at | ELN | -0,655928373 | -1,264005341 | 0,608076968 | |
| 229162_s_at | ABTB1 | -0,655928373 | -1,264005341 | 0,608076968 | |
| 229495_at | PM20D2 | -0,655928373 | -1,264005341 | 0,608076968 | |
| 229953_x_at | LCA5 | -0,655928373 | -1,264005341 | 0,608076968 | |
| 240172_at | ERGIC2 | -0,655928373 | -1,264005341 | 0,608076968 | |
| 1553392_at | EFCAB3 | -1,353583928 | -1,961547147 | 0,607963219 | |
| 1558401_at | - | -1,353583928 | -1,961547147 | 0,607963219 | |
| 1562507_at | - | -1,353583928 | -1,961547147 | 0,607963219 | |
| 1564257_at | LOC100132116 | -1,353583928 | -1,961547147 | 0,607963219 | |
| 205531_s_at | GLS2 | -1,353583928 | -1,961547147 | 0,607963219 | |
| 215477_at | - | -1,353583928 | -1,961547147 | 0,607963219 | |
| 215872_at | - | -1,353583928 | -1,961547147 | 0,607963219 | |
| 216926_s_at | MAU2 | -1,353583928 | -1,961547147 | 0,607963219 | |
| 217688_at | - | -1,353583928 | -1,961547147 | 0,607963219 | |
| 227747_at | MPZL3 | -1,353583928 | -1,961547147 | 0,607963219 | |
| 228892_at | SH3RF2 | -1,353583928 | -1,961547147 | 0,607963219 | |
| 234918_at | GLTSCR2 | -1,353583928 | -1,961547147 | 0,607963219 | |
| 239194_at | DNAJC30 | -1,353583928 | -1,961547147 | 0,607963219 | |
| 241755_at | UQCRC2 | -1,353583928 | -1,961547147 | 0,607963219 | |
| 1560028_at | C11orf57 | -2,01371626 | -2,621331989 | 0,607615729 | |
| 1561895_at | - | -2,01371626 | -2,621331989 | 0,607615729 | |
| 1569183_a_at | CHM | -2,01371626 | -2,621331989 | 0,607615729 | |
| 213591_at | ALDH7A1 | -2,01371626 | -2,621331989 | 0,607615729 | |
| 215149_at | - | -2,01371626 | -2,621331989 | 0,607615729 | |
| 236582_at | - | -2,01371626 | -2,621331989 | 0,607615729 | |
| 240941_at | - | -2,01371626 | -2,621331989 | 0,607615729 | |
| 243811_at | HOXB1 | -2,01371626 | -2,621331989 | 0,607615729 | |
| 218487_at | ALAD | 1,567778694 | 0,960386719 | 0,607391974 | |
| 1553286_at | ZNF555 | 1,556829052 | 0,949447784 | 0,607381268 | |
| 203757_s_at | CEACAM6 | -1,131062212 | -1,738067356 | 0,607005143 | |
| 209505_at | NR2F1 | -1,131062212 | -1,738067356 | 0,607005143 | |
| 217248_s_at | SLC7A8 | -1,131062212 | -1,738067356 | 0,607005143 | |
| 217350_at | KRT19P2 /// MIR | -1,131062212 | -1,738067356 | 0,607005143 | |
| 219514_at | ANGPTL2 | -1,131062212 | -1,738067356 | 0,607005143 | |

| | | | | | |
|---|---|---|---|---|---|
| 221340_at | CDX4 | -1,131062212 | -1,738067356 | 0,607005143 | |
| 221360_s_at | GHSR | -1,131062212 | -1,738067356 | 0,607005143 | |
| 225631_at | EEPD1 | -1,131062212 | -1,738067356 | 0,607005143 | |
| 231903_x_at | ARHGAP23 | -1,131062212 | -1,738067356 | 0,607005143 | |
| 235101_at | FNBP4 | -1,131062212 | -1,738067356 | 0,607005143 | |
| 235910_x_at | ZNF316 | -1,131062212 | -1,738067356 | 0,607005143 | |
| 238916_at | LOC400027 | -1,131062212 | -1,738067356 | 0,607005143 | |
| 243332_at | - | -1,131062212 | -1,738067356 | 0,607005143 | |
| 234650_at | - | -2,667025278 | -3,273861381 | 0,606836104 | |
| 218629_at | SMO | 0,917308734 | 0,310717132 | 0,606591602 | |
| 238662_at | ATPBD4 | 0,82905153 | 0,222599095 | 0,606452436 | |
| 1552470_a_at | ABHD11 | -0,888496123 | -1,494668682 | 0,606172559 | |
| 1553044_at | GJA10 | -0,888496123 | -1,494668682 | 0,606172559 | |
| 1559425_at | - | -0,888496123 | -1,494668682 | 0,606172559 | |
| 214637_at | OSM | -0,888496123 | -1,494668682 | 0,606172559 | |
| 231126_at | C2orf70 | -0,888496123 | -1,494668682 | 0,606172559 | |
| 238752_at | GPLD1 | -0,888496123 | -1,494668682 | 0,606172559 | |
| 243999_at | SLFN5 | -0,888496123 | -1,494668682 | 0,606172559 | |
| 201822_at | TIMM17A | 0,384950573 | -0,220592223 | 0,605542797 | |
| 204024_at | OSGIN2 | 0,384950573 | -0,220592223 | 0,605542797 | |
| 224519_at | LOC100132167 | 0,384950573 | -0,220592223 | 0,605542797 | |
| 240181_at | ZSCAN12 | 0,255492549 | -0,34958478 | 0,60507733 | |
| 1569790_at | - | -2,43257096 | -3,037500868 | 0,604929908 | |
| 207523_at | C6orf10 | -2,43257096 | -3,037500868 | 0,604929908 | |
| 1553456_at | - | -1,308181863 | -1,912089812 | 0,603907949 | |
| 1558444_at | - | -1,308181863 | -1,912089812 | 0,603907949 | |
| 1563079_at | LOC100289090 | -1,308181863 | -1,912089812 | 0,603907949 | |
| 1563296_at | LINC00572 | -1,308181863 | -1,912089812 | 0,603907949 | |
| 1568768_s_at | BRE-AS1 | -1,308181863 | -1,912089812 | 0,603907949 | |
| 205297_s_at | CD79B | -1,308181863 | -1,912089812 | 0,603907949 | |
| 213525_at | - | -1,308181863 | -1,912089812 | 0,603907949 | |
| 214641_at | COL4A3 | -1,308181863 | -1,912089812 | 0,603907949 | |
| 217471_at | - | -1,308181863 | -1,912089812 | 0,603907949 | |
| 219845_at | BARX1 | -1,308181863 | -1,912089812 | 0,603907949 | |
| 244668_at | - | -1,308181863 | -1,912089812 | 0,603907949 | |
| 222360_at | DPH5 | 0,95671774 | 0,352836757 | 0,603880983 | |
| 204211_x_at | EIF2AK2 | 3,237986416 | 2,634191677 | 0,603794739 | |
| 240166_x_at | TRMT10B | 0,87091093 | 0,26733076 | 0,60358017 | |
| 235156_at | BRWD3 | 0,760715727 | 0,157549243 | 0,603166484 | |
| 201598_s_at | INPPL1 | 2,352981171 | 1,750261389 | 0,602719782 | |
| 230116_at | KRT8P12 | 0,57782909 | -0,024572586 | 0,602401677 | |
| 221550_at | COX15 | 2,001086023 | 1,398700978 | 0,602385045 | |
| 204465_s_at | INA | 0,46522187 | -0,136643983 | 0,601865853 | |
| 239252_at | COX7B | 0,44160593 | -0,160136748 | 0,601742678 | |
| 214450_at | CTSW | -0,078265071 | -0,679763839 | 0,601498768 | |
| 1553079_at | TRIM40 | -2,089474779 | -2,690860744 | 0,601385965 | |
| 1562742_at | - | -2,089474779 | -2,690860744 | 0,601385965 | |
| 1569864_at | SERAC1 | -2,089474779 | -2,690860744 | 0,601385965 | |
| 1570357_at | STX8 | -2,089474779 | -2,690860744 | 0,601385965 | |
| 221438_s_at | TEX12 | -2,089474779 | -2,690860744 | 0,601385965 | |
| 229662_at | - | -2,089474779 | -2,690860744 | 0,601385965 | |
| 237684_at | LOC100507461 | -2,089474779 | -2,690860744 | 0,601385965 | |
| 204025_s_at | PDCD2 | 3,873548912 | 3,272369332 | 0,601179579 | |
| 213481_at | S100A13 | 0,317354302 | -0,283667828 | 0,601022129 | |
| 227575_s_at | C14orf102 | 1,655931423 | 1,055274826 | 0,600656597 | |
| 204231_s_at | FAAH | -0,512251684 | -1,112851844 | 0,600600159 | |

| | | | | | |
|---|---|---|---|---|---|
| 204945_at | PTPRN | -0,512251684 | -1,112851844 | 0,600600159 | |
| 219726_at | NLGN3 | -0,512251684 | -1,112851844 | 0,600600159 | |
| 235970_at | LCORL | -0,512251684 | -1,112851844 | 0,600600159 | |
| 238100_at | AAK1 | -0,512251684 | -1,112851844 | 0,600600159 | |
| 239154_at | - | -0,512251684 | -1,112851844 | 0,600600159 | |
| 1552882_a_at | FAM123B | 0,209623097 | -0,39061235 | 0,600235447 | |
| 1564229_at | LOC729173 | 0,209623097 | -0,39061235 | 0,600235447 | |
| 208395_s_at | URB1 | 0,209623097 | -0,39061235 | 0,600235447 | |
| 237013_at | - | 0,209623097 | -0,39061235 | 0,600235447 | |
| 230696_at | LOC100289092 | 1,560488174 | 0,960386719 | 0,600101455 | |
| 209028_s_at | ABI1 | 2,427005622 | 1,826973888 | 0,600031734 | |
| 221334_s_at | FOXP3 | 0,181384709 | -0,41858459 | 0,599969299 | |
| 215532_x_at | ZNF492 | 1,504602299 | 0,904843258 | 0,59975904 | |
| 217744_s_at | PERP | 0,627515113 | 0,028364014 | 0,599151099 | |
| 47571_at | ZNF236 | 1,764611101 | 1,16573017 | 0,59888093 | |
| 1553084_at | STARD6 | -1,688127507 | -2,286583451 | 0,598455944 | |
| 1558001_s_at | ARID5B | -1,688127507 | -2,286583451 | 0,598455944 | |
| 1558414_at | FRRS1L | -1,688127507 | -2,286583451 | 0,598455944 | |
| 1561589_a_at | NBEAL1 | -1,688127507 | -2,286583451 | 0,598455944 | |
| 207234_at | RFX3 | -1,688127507 | -2,286583451 | 0,598455944 | |
| 215571_at | - | -1,688127507 | -2,286583451 | 0,598455944 | |
| 231916_at | NOS1 | -1,688127507 | -2,286583451 | 0,598455944 | |
| 232498_at | HEATR7A /// LO( | -1,688127507 | -2,286583451 | 0,598455944 | |
| 234850_at | MOGAT3 | -1,688127507 | -2,286583451 | 0,598455944 | |
| 237322_at | MIAT | -1,688127507 | -2,286583451 | 0,598455944 | |
| 242830_at | - | -1,688127507 | -2,286583451 | 0,598455944 | |
| 1562098_at | - | 0,03125738 | -0,566887459 | 0,598144839 | |
| 210051_at | RAPGEF3 | 0,03125738 | -0,566887459 | 0,598144839 | |
| 235088_at | C4orf46 | 3,142344098 | 2,544354523 | 0,597989575 | |
| 203583_at | UNC50 | 3,785615967 | 3,187631636 | 0,597984331 | |
| 214297_at | CSPG4 | -0,352951799 | -0,950686014 | 0,597734216 | |
| 243895_x_at | - | -0,352951799 | -0,950686014 | 0,597734216 | |
| 229217_at | SP3 | -0,221656139 | -0,819238336 | 0,597582198 | |
| 1553051_s_at | ODF3 | -0,622910612 | -1,220210588 | 0,597299975 | |
| 1555468_at | NRP2 | -0,622910612 | -1,220210588 | 0,597299975 | |
| 1558569_at | LOC100131541 | -0,622910612 | -1,220210588 | 0,597299975 | |
| 200724_at | RPL10 | -0,622910612 | -1,220210588 | 0,597299975 | |
| 206541_at | KLKB1 | -0,622910612 | -1,220210588 | 0,597299975 | |
| 209312_x_at | HLA-DRB1 /// HL | -0,622910612 | -1,220210588 | 0,597299975 | |
| 212874_at | APOE | -0,622910612 | -1,220210588 | 0,597299975 | |
| 215500_at | SNX29 | -0,622910612 | -1,220210588 | 0,597299975 | |
| 215921_at | NPIPL3 | -0,622910612 | -1,220210588 | 0,597299975 | |
| 236606_at | - | -0,622910612 | -1,220210588 | 0,597299975 | |
| 238925_at | SNTB2 | -0,622910612 | -1,220210588 | 0,597299975 | |
| 229488_at | OTUD7B | 0,376672223 | -0,220592223 | 0,597264447 | |
| 209649_at | STAM2 | 2,305800198 | 1,708698295 | 0,597101903 | |
| 1556903_at | - | -2,138804305 | -2,735862426 | 0,59705812 | |
| 1562424_at | LOC285889 | -2,138804305 | -2,735862426 | 0,59705812 | |
| 1566461_at | - | -2,138804305 | -2,735862426 | 0,59705812 | |
| 213267_at | DOPEY1 | -2,138804305 | -2,735862426 | 0,59705812 | |
| 216424_at | CD4 | -2,138804305 | -2,735862426 | 0,59705812 | |
| 227210_at | SFMBT2 | -2,138804305 | -2,735862426 | 0,59705812 | |
| 240531_at | - | -2,138804305 | -2,735862426 | 0,59705812 | |
| 242334_at | NLRP4 | -2,138804305 | -2,735862426 | 0,59705812 | |
| 244446_at | LOC100505525 | -2,138804305 | -2,735862426 | 0,59705812 | |
| 203543_s_at | KLF9 | 1,114389003 | 0,51742585 | 0,596963153 | |

| | | | | | |
|---|---|---|---|---|---|
| 209206_at | SEC22B | 3,21379258 | 2,617011906 | 0,596780674 | |
| 236281_x_at | HTR7 | 0,273439642 | -0,322870281 | 0,596309923 | |
| 228468_at | MASTL | 1,912238072 | 1,315948728 | 0,596289344 | |
| 1561516_at | - | -1,812418401 | -2,408705138 | 0,596286737 | |
| 1563059_at | LOC340581 | -1,812418401 | -2,408705138 | 0,596286737 | |
| 1569102_at | ABCA4 | -1,812418401 | -2,408705138 | 0,596286737 | |
| 205112_at | PLCE1 | -1,812418401 | -2,408705138 | 0,596286737 | |
| 216907_x_at | KIR3DL1 /// KIR3 | -1,812418401 | -2,408705138 | 0,596286737 | |
| 220877_at | - | -1,812418401 | -2,408705138 | 0,596286737 | |
| 228113_at | RAB37 | -1,812418401 | -2,408705138 | 0,596286737 | |
| 231051_at | M1 | -1,812418401 | -2,408705138 | 0,596286737 | |
| 232753_at | ZNF346 | -1,812418401 | -2,408705138 | 0,596286737 | |
| 236987_at | - | -1,812418401 | -2,408705138 | 0,596286737 | |
| 1557154_at | - | 0,246434561 | -0,34958478 | 0,596019341 | |
| 1569001_at | BMP1 | 0,246434561 | -0,34958478 | 0,596019341 | |
| 229289_at | FAM71E1 | -0,259832361 | -0,855751026 | 0,595918665 | |
| 239682_at | - | -0,259832361 | -0,855751026 | 0,595918665 | |
| 215207_x_at | NUS1 /// NUS1P3 | 1,973904239 | 1,378453643 | 0,595450595 | |
| 1561448_at | - | -2,359436556 | -2,954576755 | 0,595140199 | |
| 1562847_at | - | -2,359436556 | -2,954576755 | 0,595140199 | |
| 1569808_at | - | -2,359436556 | -2,954576755 | 0,595140199 | |
| 221470_s_at | IL37 | -2,359436556 | -2,954576755 | 0,595140199 | |
| 234529_at | PCGEM1 | -2,359436556 | -2,954576755 | 0,595140199 | |
| 1562955_at | - | -0,946847173 | -1,541908042 | 0,595060869 | |
| 1568951_at | ZNF280D | -0,946847173 | -1,541908042 | 0,595060869 | |
| 214165_s_at | HS6ST1 | -0,946847173 | -1,541908042 | 0,595060869 | |
| 230050_at | NACC2 | -0,946847173 | -1,541908042 | 0,595060869 | |
| 243692_at | GATA4 | -0,946847173 | -1,541908042 | 0,595060869 | |
| 221561_at | SOAT1 | 2,983784569 | 2,389070873 | 0,594713696 | |
| 207565_s_at | MR1 | -0,136301282 | -0,730778808 | 0,594477526 | |
| 211320_s_at | PTPRU | -0,136301282 | -0,730778808 | 0,594477526 | |
| 214074_s_at | CTTN | 1,85379461 | 1,259496994 | 0,594297615 | |
| 209318_x_at | PLAGL1 | 4,229522279 | 3,635238397 | 0,594283882 | |
| 227826_s_at | - | -2,504600601 | -3,098858316 | 0,594257715 | |
| 232722_at | RNASET2 | -2,504600601 | -3,098858316 | 0,594257715 | |
| 234739_at | - | -2,504600601 | -3,098858316 | 0,594257715 | |
| 205530_at | ETFDH | 1,089278259 | 0,495031851 | 0,594246409 | |
| 1569732_at | - | 0,103263601 | -0,490927335 | 0,594190937 | |
| 201882_x_at | B4GALT1 | 0,103263601 | -0,490927335 | 0,594190937 | |
| 206064_s_at | PPIL2 | 0,103263601 | -0,490927335 | 0,594190937 | |
| 222302_at | LOC100507009 | 0,457392764 | -0,136643983 | 0,594036747 | |
| 201181_at | GNAI3 | 2,493593689 | 1,899812471 | 0,593781218 | |
| 202319_at | SENP6 | 1,251994932 | 0,658444279 | 0,593550653 | |
| 223898_at | ZNF670 | 3,381421359 | 2,787940144 | 0,593481215 | |
| 229704_at | PDS5B | 1,90937145 | 1,315948728 | 0,593422722 | |
| 1553694_a_at | PIK3C2A | -0,172274514 | -0,765504029 | 0,593229515 | |
| 200951_s_at | CCND2 | -0,172274514 | -0,765504029 | 0,593229515 | |
| 220713_at | FAM116B | -0,172274514 | -0,765504029 | 0,593229515 | |
| 238381_x_at | - | -2,64318869 | -3,236070201 | 0,592881511 | |
| 214877_at | CDKAL1 | 1,348276312 | 0,755840599 | 0,592435712 | |
| 1552318_at | GIMAP1 | -2,187914861 | -2,780313924 | 0,592399063 | |
| 1553448_at | FLJ34503 | -2,187914861 | -2,780313924 | 0,592399063 | |
| 1562432_at | - | -2,187914861 | -2,780313924 | 0,592399063 | |
| 1566033_at | - | -2,187914861 | -2,780313924 | 0,592399063 | |
| 229147_at | RASSF6 | -2,187914861 | -2,780313924 | 0,592399063 | |
| 241942_at | PXDNL | -2,187914861 | -2,780313924 | 0,592399063 | |

| | | | | | |
|---|---|---|---|---|---|
| 228316_at | C2orf63 | 0,30867765 | -0,283667828 | 0,592345477 | |
| 244367_at | - | 0,30867765 | -0,283667828 | 0,592345477 | |
| 201689_s_at | TPD52 | 1,783520648 | 1,191491281 | 0,592029367 | |
| 224335_s_at | BACE1 | -0,022466681 | -0,614371577 | 0,591904896 | |
| 230346_x_at | - | -0,022466681 | -0,614371577 | 0,591904896 | |
| 1554261_at | KLHL29 | -0,83191556 | -1,423348341 | 0,591432781 | |
| 1555529_at | RNH1 | -0,83191556 | -1,423348341 | 0,591432781 | |
| 1569416_at | - | -0,83191556 | -1,423348341 | 0,591432781 | |
| 221594_at | RBM48 | -0,83191556 | -1,423348341 | 0,591432781 | |
| 230105_at | HOXB13 | -0,83191556 | -1,423348341 | 0,591432781 | |
| 233215_s_at | ZDHHC21 | -0,83191556 | -1,423348341 | 0,591432781 | |
| 244054_at | - | -0,83191556 | -1,423348341 | 0,591432781 | |
| 1554103_at | - | -0,67289328 | -1,264005341 | 0,591112061 | |
| 211778_s_at | OVOL2 | -0,67289328 | -1,264005341 | 0,591112061 | |
| 213679_at | TTC30A | -0,67289328 | -1,264005341 | 0,591112061 | |
| 223739_at | PADI1 | -0,67289328 | -1,264005341 | 0,591112061 | |
| 232622_at | - | -0,67289328 | -1,264005341 | 0,591112061 | |
| 219916_s_at | RNF39 | -0,055687482 | -0,646746079 | 0,591058597 | |
| 229588_at | DNAJC10 | -0,055687482 | -0,646746079 | 0,591058597 | |
| 237151_s_at | PCDP1 | -0,055687482 | -0,646746079 | 0,591058597 | |
| 202738_s_at | PHKB | 1,940595304 | 1,349621332 | 0,590973972 | |
| 226681_at | UBE2H | -1,196375121 | -1,787089803 | 0,590714682 | |
| 228985_at | - | -1,196375121 | -1,787089803 | 0,590714682 | |
| 240343_at | - | -1,196375121 | -1,787089803 | 0,590714682 | |
| 243246_at | - | -1,196375121 | -1,787089803 | 0,590714682 | |
| 217606_at | - | 0,171847695 | -0,41858459 | 0,590432285 | |
| 1562295_at | ADAMTS9-AS2 | -0,741668563 | -1,332017329 | 0,590348766 | |
| 1563715_at | - | -0,741668563 | -1,332017329 | 0,590348766 | |
| 1569481_s_at | SNX22 | -0,741668563 | -1,332017329 | 0,590348766 | |
| 203424_s_at | IGFBP5 | -0,741668563 | -1,332017329 | 0,590348766 | |
| 208067_x_at | UTY | -0,741668563 | -1,332017329 | 0,590348766 | |
| 222461_s_at | HERC2 /// LOC10 | -0,741668563 | -1,332017329 | 0,590348766 | |
| 227513_s_at | LRRFIP1 | -0,741668563 | -1,332017329 | 0,590348766 | |
| 229493_at | HOXD-AS2 | -0,741668563 | -1,332017329 | 0,590348766 | |
| 233206_at | SLC22A23 | -0,741668563 | -1,332017329 | 0,590348766 | |
| 233693_at | C1orf201 | -0,741668563 | -1,332017329 | 0,590348766 | |
| 236553_at | LOC100507520 | -0,741668563 | -1,332017329 | 0,590348766 | |
| 239362_at | NAPA-AS1 | -0,741668563 | -1,332017329 | 0,590348766 | |
| 240151_at | HOXB-AS3 | -0,741668563 | -1,332017329 | 0,590348766 | |
| 216971_s_at | PLEC | -0,089689931 | -0,679763839 | 0,590073908 | |
| 225163_at | FRMD4A | -0,089689931 | -0,679763839 | 0,590073908 | |
| 203132_at | RB1 | 4,28697245 | 3,697038015 | 0,589934435 | |
| 204001_at | SNAPC3 | 0,900083947 | 0,310717132 | 0,589366815 | |
| 216321_s_at | NR3C1 | 2,808702036 | 2,21959457 | 0,589107466 | |
| 1558233_s_at | ATF1 | 3,410096504 | 2,821324225 | 0,588772279 | |
| 1563654_at | C8orf66 | -1,026571149 | -1,615132408 | 0,588561259 | |
| 1568643_a_at | - | -1,026571149 | -1,615132408 | 0,588561259 | |
| 205428_s_at | CALB2 | -1,026571149 | -1,615132408 | 0,588561259 | |
| 207463_x_at | PRSS3 | -1,026571149 | -1,615132408 | 0,588561259 | |
| 234406_at | RGMA | -1,026571149 | -1,615132408 | 0,588561259 | |
| 237305_at | - | -1,026571149 | -1,615132408 | 0,588561259 | |
| 238016_s_at | - | -1,026571149 | -1,615132408 | 0,588561259 | |
| 240926_at | LRCH3 | -1,026571149 | -1,615132408 | 0,588561259 | |
| 241259_at | GAB3 | -1,026571149 | -1,615132408 | 0,588561259 | |
| 224416_s_at | MED28 | 3,021441862 | 2,432926755 | 0,588515107 | |
| 213990_s_at | PAK7 | 0,052199481 | -0,536087151 | 0,588286631 | |

| | | | | | |
|---|---|---|---|---|---|
| 226614_s_at | FAM167A | 0,052199481 | -0,536087151 | 0,588286631 | |
| 242857_at | - | 0,052199481 | -0,536087151 | 0,588286631 | |
| 232517_s_at | PRIC285 | 1,177702228 | 0,589657387 | 0,588044841 | |
| 218951_s_at | PLCXD1 | 2,981056773 | 2,39311332 | 0,587943453 | |
| 231725_at | PCDHB2 | 0,792179257 | 0,204310755 | 0,587868501 | |
| 36612_at | FAM168A | 1,148825584 | 0,56119757 | 0,587628015 | |
| 206437_at | S1PR4 | 0,541261317 | -0,046302147 | 0,587563464 | |
| 243730_at | - | 0,541261317 | -0,046302147 | 0,587563464 | |
| 210082_at | ABCA4 | 0,020670649 | -0,566887459 | 0,587558109 | |
| 229814_at | - | 0,020670649 | -0,566887459 | 0,587558109 | |
| 216378_at | - | -2,551294263 | -3,138626879 | 0,587332616 | |
| 243211_at | - | -2,551294263 | -3,138626879 | 0,587332616 | |
| 202393_s_at | KLF10 | 5,127212336 | 4,540112936 | 0,587099399 | |
| 229036_at | TNRC6B | 1,104397092 | 0,51742585 | 0,586971241 | |
| 1561883_at | - | -0,423248115 | -1,010192375 | 0,58694426 | |
| 205014_at | FGFBP1 | -0,423248115 | -1,010192375 | 0,58694426 | |
| 210306_at | L3MBTL1 | -0,423248115 | -1,010192375 | 0,58694426 | |
| 225102_at | MGLL | -0,423248115 | -1,010192375 | 0,58694426 | |
| 233122_at | KRTCAP2 | -0,423248115 | -1,010192375 | 0,58694426 | |
| 238270_x_at | - | -0,423248115 | -1,010192375 | 0,58694426 | |
| 210601_at | CDH6 | -0,325749314 | -0,912331589 | 0,586582275 | |
| 211210_x_at | SH2D1A | -0,325749314 | -0,912331589 | 0,586582275 | |
| 212703_at | TLN2 | -0,325749314 | -0,912331589 | 0,586582275 | |
| 216159_s_at | - | -0,325749314 | -0,912331589 | 0,586582275 | |
| 219509_at | MYOZ1 | -0,325749314 | -0,912331589 | 0,586582275 | |
| 221385_s_at | FFAR3 | -0,325749314 | -0,912331589 | 0,586582275 | |
| 226504_at | FAM109B | -0,325749314 | -0,912331589 | 0,586582275 | |
| 236146_at | SYNCRIP | -0,325749314 | -0,912331589 | 0,586582275 | |
| 244688_at | - | -0,325749314 | -0,912331589 | 0,586582275 | |
| 226873_at | FAM63B | 0,853119696 | 0,26733076 | 0,585788936 | |
| 208841_s_at | G3BP2 | 5,381596876 | 4,796236302 | 0,585360575 | |
| 1553885_x_at | ZNF99 | -0,234278768 | -0,819238336 | 0,584959569 | |
| 215844_at | TNPO2 | -0,234278768 | -0,819238336 | 0,584959569 | |
| 217469_at | IGHA1 | -0,234278768 | -0,819238336 | 0,584959569 | |
| 231480_at | SLC6A19 | -0,234278768 | -0,819238336 | 0,584959569 | |
| 238120_at | RPH3AL | -0,234278768 | -0,819238336 | 0,584959569 | |
| 1557593_at | SPAG17 | -2,262747984 | -2,847001814 | 0,58425383 | |
| 1568554_x_at | - | -2,262747984 | -2,847001814 | 0,58425383 | |
| 240704_at | - | -2,262747984 | -2,847001814 | 0,58425383 | |
| 242181_at | - | -2,262747984 | -2,847001814 | 0,58425383 | |
| 1555974_a_at | - | -0,366776884 | -0,950686014 | 0,583909131 | |
| 237041_x_at | RCOR1 | -0,366776884 | -0,950686014 | 0,583909131 | |
| 240689_at | C15orf60 | -0,366776884 | -0,950686014 | 0,583909131 | |
| 1556690_s_at | - | -0,467091869 | -1,050406615 | 0,583314746 | |
| 228472_at | CCDC61 | -0,467091869 | -1,050406615 | 0,583314746 | |
| 237931_at | - | -0,467091869 | -1,050406615 | 0,583314746 | |
| 244391_at | TSEN2 | 0,503742507 | -0,079522948 | 0,583265455 | |
| 209920_at | BMPR2 | -0,148196756 | -0,730778808 | 0,582582052 | |
| 216409_at | ACSL6 | -0,148196756 | -0,730778808 | 0,582582052 | |
| 224221_s_at | VAV3 | -0,148196756 | -0,730778808 | 0,582582052 | |
| 241417_at | - | -0,148196756 | -0,730778808 | 0,582582052 | |
| 205695_at | SDS | 0,661985843 | 0,079428135 | 0,582557707 | |
| 215930_s_at | CTAGE5 | 0,661985843 | 0,079428135 | 0,582557707 | |
| 226261_at | ZNRF2 | 1,369290453 | 0,786897721 | 0,582392731 | |
| 205505_at | GCNT1 | 2,724770722 | 2,14244844 | 0,582322283 | |
| 205923_at | RELN | 0,641402394 | 0,059218869 | 0,582183526 | |

| | | | | | |
|---|---|---|---|---|---|
| 220535_at | FAM90A1 | 0,641402394 | 0,059218869 | 0,582183526 | |
| 1562540_at | LOC339978 | -0,79540287 | -1,377419394 | 0,582016524 | |
| 1565858_at | SNORA71A | -0,79540287 | -1,377419394 | 0,582016524 | |
| 210888_s_at | ITIH1 | -0,79540287 | -1,377419394 | 0,582016524 | |
| 218501_at | ARHGEF3 | -0,79540287 | -1,377419394 | 0,582016524 | |
| 231859_at | C14orf132 | -0,79540287 | -1,377419394 | 0,582016524 | |
| 234796_at | CABP7 | -0,79540287 | -1,377419394 | 0,582016524 | |
| 238359_at | - | -0,79540287 | -1,377419394 | 0,582016524 | |
| 225861_at | FAM195A | 3,541801785 | 2,959949103 | 0,581852682 | |
| 230297_x_at | SYNGAP1 | 0,934330293 | 0,352836757 | 0,581493536 | |
| 235610_at | ALKBH8 | 1,621313976 | 1,039887232 | 0,581426745 | |
| 232616_at | LOC100129935 | -0,033456564 | -0,614371577 | 0,580915012 | |
| 223577_x_at | MALAT1 | 3,152077493 | 2,571362843 | 0,580714649 | |
| 221185_s_at | IQCG | 0,555999837 | -0,024572586 | 0,580572423 | |
| 216164_at | LRRN2 | 0,359971644 | -0,220592223 | 0,580563867 | |
| 232335_at | - | 0,359971644 | -0,220592223 | 0,580563867 | |
| 1555358_a_at | ENTPD4 | -0,57445847 | -1,154897679 | 0,580439209 | |
| 1557279_at | ITSN1 | -0,57445847 | -1,154897679 | 0,580439209 | |
| 232391_at | ZNF461 | -0,57445847 | -1,154897679 | 0,580439209 | |
| 243911_at | - | -0,57445847 | -1,154897679 | 0,580439209 | |
| 1560148_at | - | -2,597496523 | -3,177802286 | 0,580305763 | |
| 1568647_at | LOC100505851 | -2,597496523 | -3,177802286 | 0,580305763 | |
| 241126_at | - | -2,597496523 | -3,177802286 | 0,580305763 | |
| 206342_x_at | IDS | 0,533835206 | -0,046302147 | 0,580137353 | |
| 218913_s_at | GMIP | 0,533835206 | -0,046302147 | 0,580137353 | |
| 1564637_a_at | FAM98B | 3,294008537 | 2,713921281 | 0,580087256 | |
| 212475_at | AVL9 | 0,95671774 | 0,377530647 | 0,579187093 | |
| 226868_at | GXYLT1 | 1,894952274 | 1,315948728 | 0,579003545 | |
| 212393_at | SBF1 | 1,073999308 | 0,495031851 | 0,578967457 | |
| 1569348_at | MIR4500HG /// T | -2,311268652 | -2,89013181 | 0,578863159 | |
| 207342_at | CNGB1 | -2,311268652 | -2,89013181 | 0,578863159 | |
| 220220_at | LRRC37A4P | -2,311268652 | -2,89013181 | 0,578863159 | |
| 233162_at | - | -2,311268652 | -2,89013181 | 0,578863159 | |
| 236365_at | AMACR | -2,311268652 | -2,89013181 | 0,578863159 | |
| 242426_at | NRG4 | -2,311268652 | -2,89013181 | 0,578863159 | |
| 201065_s_at | GTF2I /// GTF2IP | 4,354920338 | 3,77655538 | 0,578364958 | |
| 203852_s_at | SMN1 /// SMN2 | 3,954344268 | 3,376085218 | 0,57825905 | |
| 233014_at | - | 0,44160593 | -0,136643983 | 0,578249912 | |
| 1553407_at | MACF1 | -1,2845686 | -1,862647763 | 0,578079162 | |
| 1559421_at | SNED1 | -1,2845686 | -1,862647763 | 0,578079162 | |
| 1564274_at | C9orf47 | -1,2845686 | -1,862647763 | 0,578079162 | |
| 1569146_s_at | KIAA0408 /// SO( | -1,2845686 | -1,862647763 | 0,578079162 | |
| 205439_at | GSTT2 | -1,2845686 | -1,862647763 | 0,578079162 | |
| 206350_at | APCS | -1,2845686 | -1,862647763 | 0,578079162 | |
| 217594_at | ZCCHC11 | -1,2845686 | -1,862647763 | 0,578079162 | |
| 218790_s_at | TMLHE | -1,2845686 | -1,862647763 | 0,578079162 | |
| 220101_x_at | - | -1,2845686 | -1,862647763 | 0,578079162 | |
| 222323_at | - | -1,2845686 | -1,862647763 | 0,578079162 | |
| 239675_at | LOC283143 | -1,2845686 | -1,862647763 | 0,578079162 | |
| 240973_s_at | - | -1,2845686 | -1,862647763 | 0,578079162 | |
| 244143_at | - | -1,2845686 | -1,862647763 | 0,578079162 | |
| 225847_at | NCEH1 | 1,69307511 | 1,115234684 | 0,577840426 | |
| 204908_s_at | BCL3 | 0,228146222 | -0,34958478 | 0,577731002 | |
| 232310_at | LOC100507637 | 0,228146222 | -0,34958478 | 0,577731002 | |
| 208701_at | APLP2 | 0,87091093 | 0,293518836 | 0,577392094 | |
| 203815_at | GSTT1 | 0,995078803 | 0,417770464 | 0,577308339 | |

| | | | | | |
|---|---|---|---|---|---|
| 1558014_s_at | FAR1 | 2,382221458 | 1,805902329 | 0,576319128 | |
| 206989_s_at | SCAF11 | 3,941773349 | 3,365837114 | 0,575936234 | |
| 1320_at | PTPN21 | -1,124406268 | -1,700258111 | 0,575851843 | |
| 209211_at | KLF5 | 0,496120222 | -0,079522948 | 0,57564317 | |
| 217540_at | NXPE3 | 0,496120222 | -0,079522948 | 0,57564317 | |
| 241366_at | LOC389458 /// RI | 0,317354302 | -0,258114234 | 0,575468536 | |
| 217915_s_at | RSL24D1 | 5,643563313 | 5,068098141 | 0,575465172 | |
| 210635_s_at | KLHL20 | 0,911589958 | 0,336136178 | 0,575453781 | |
| 211080_s_at | NEK2 | 1,331241794 | 0,755840599 | 0,575401195 | |
| 223921_s_at | GBA2 | 1,655931423 | 1,080561626 | 0,575369797 | |
| 206157_at | PTX3 | 2,734521183 | 2,159161028 | 0,575360155 | |
| 218067_s_at | ARGLU1 | 5,116377229 | 4,541024081 | 0,575353148 | |
| 212136_at | ATP2B4 | 3,137452663 | 2,562416132 | 0,575036531 | |
| 211967_at | TMEM123 | 4,932352627 | 4,357702907 | 0,57464972 | |
| 1552829_at | - | -1,089016378 | -1,663414495 | 0,574398117 | |
| 1558525_at | - | -1,089016378 | -1,663414495 | 0,574398117 | |
| 1559195_at | - | -1,089016378 | -1,663414495 | 0,574398117 | |
| 1559291_at | LINC00032 | -1,089016378 | -1,663414495 | 0,574398117 | |
| 205776_at | FMO5 | -1,089016378 | -1,663414495 | 0,574398117 | |
| 208381_s_at | SGPL1 | -1,089016378 | -1,663414495 | 0,574398117 | |
| 221081_s_at | DENND2D | -1,089016378 | -1,663414495 | 0,574398117 | |
| 228339_at | ECSCR | -1,089016378 | -1,663414495 | 0,574398117 | |
| 232418_at | LZTFL1 | -1,089016378 | -1,663414495 | 0,574398117 | |
| 238232_at | - | -1,089016378 | -1,663414495 | 0,574398117 | |
| 208893_s_at | DUSP6 | 3,159334685 | 2,585563171 | 0,573771514 | |
| 1556009_at | PEX13 | 1,05337236 | 0,479907041 | 0,573465319 | |
| 32540_at | PPP3CC | -0,584387095 | -1,157156127 | 0,572769032 | |
| 1555632_at | PIK3IP1 | -0,85085127 | -1,423348341 | 0,572497072 | |
| 205499_at | SRPX2 | -0,85085127 | -1,423348341 | 0,572497072 | |
| 206666_at | GZMK | -0,85085127 | -1,423348341 | 0,572497072 | |
| 207137_at | TONSL | -0,85085127 | -1,423348341 | 0,572497072 | |
| 208145_at | - | -0,85085127 | -1,423348341 | 0,572497072 | |
| 240371_at | LOC647107 | -0,85085127 | -1,423348341 | 0,572497072 | |
| 241318_at | - | -0,85085127 | -1,423348341 | 0,572497072 | |
| 1554953_a_at | C21orf90 | -0,43775896 | -1,010192375 | 0,572433415 | |
| 1556620_at | - | -0,43775896 | -1,010192375 | 0,572433415 | |
| 1569713_at | SEC24B-AS1 | -0,43775896 | -1,010192375 | 0,572433415 | |
| 233977_at | GREB1L | -0,43775896 | -1,010192375 | 0,572433415 | |
| 238598_s_at | - | -0,43775896 | -1,010192375 | 0,572433415 | |
| 241045_at | KDM8 | -0,43775896 | -1,010192375 | 0,572433415 | |
| 1556658_a_at | - | -0,759588698 | -1,332017329 | 0,572428631 | |
| 1557664_at | LOC340239 | -0,759588698 | -1,332017329 | 0,572428631 | |
| 1566696_at | - | -0,759588698 | -1,332017329 | 0,572428631 | |
| 215986_at | - | -0,759588698 | -1,332017329 | 0,572428631 | |
| 225186_at | RAPH1 | -0,759588698 | -1,332017329 | 0,572428631 | |
| 227631_at | ABI2 | -0,759588698 | -1,332017329 | 0,572428631 | |
| 231459_at | LOC100506397 | -0,759588698 | -1,332017329 | 0,572428631 | |
| 231467_at | - | -0,759588698 | -1,332017329 | 0,572428631 | |
| 1553618_at | TRIM43 /// TRIM4 | -1,71423189 | -2,286583451 | 0,572351561 | |
| 1557807_a_at | - | -1,71423189 | -2,286583451 | 0,572351561 | |
| 1564805_a_at | OFCC1 | -1,71423189 | -2,286583451 | 0,572351561 | |
| 1566256_s_at | GPR180 | -1,71423189 | -2,286583451 | 0,572351561 | |
| 202878_s_at | CD93 | -1,71423189 | -2,286583451 | 0,572351561 | |
| 206254_at | EGF | -1,71423189 | -2,286583451 | 0,572351561 | |
| 219955_at | L1TD1 | -1,71423189 | -2,286583451 | 0,572351561 | |
| 227048_at | LAMA1 | -1,71423189 | -2,286583451 | 0,572351561 | |

| | | | | | |
|---|---|---|---|---|---|
| 229747_x_at | LOC146880 | -1,71423189 | -2,286583451 | 0,572351561 | |
| 231231_at | - | -1,71423189 | -2,286583451 | 0,572351561 | |
| 234271_at | OTOP2 | -1,71423189 | -2,286583451 | 0,572351561 | |
| 234566_at | - | -1,71423189 | -2,286583451 | 0,572351561 | |
| 240703_s_at | HERC1 | -1,71423189 | -2,286583451 | 0,572351561 | |
| 208876_s_at | PAK2 | 1,104397092 | 0,532164371 | 0,572232721 | |
| 225555_x_at | AURKAIP1 | 1,104397092 | 0,532164371 | 0,572232721 | |
| 1556436_at | EXTL3-AS1 | -1,639579028 | -2,211750327 | 0,572171299 | |
| 1557753_at | - | -1,639579028 | -2,211750327 | 0,572171299 | |
| 1566737_at | LOC728716 | -1,639579028 | -2,211750327 | 0,572171299 | |
| 219896_at | CALY | -1,639579028 | -2,211750327 | 0,572171299 | |
| 221254_s_at | PITPNM3 | -1,639579028 | -2,211750327 | 0,572171299 | |
| 230317_x_at | TMCO3 | -1,639579028 | -2,211750327 | 0,572171299 | |
| 238263_at | EPHA1-AS1 | -1,639579028 | -2,211750327 | 0,572171299 | |
| 226540_at | CCDC42B | 0,181384709 | -0,39061235 | 0,571997059 | |
| 227928_at | PARPBP | 2,197169074 | 1,6252386 | 0,571930474 | |
| 1560023_x_at | - | -1,763254337 | -2,335104118 | 0,571849781 | |
| 1563260_at | LINC00587 | -1,763254337 | -2,335104118 | 0,571849781 | |
| 202437_s_at | CYP1B1 | -1,763254337 | -2,335104118 | 0,571849781 | |
| 216502_at | ISG20L2 | -1,763254337 | -2,335104118 | 0,571849781 | |
| 221361_at | OMP | -1,763254337 | -2,335104118 | 0,571849781 | |
| 223899_at | C3orf78 | -1,763254337 | -2,335104118 | 0,571849781 | |
| 228004_at | LINC00261 | -1,763254337 | -2,335104118 | 0,571849781 | |
| 230596_at | - | -1,763254337 | -2,335104118 | 0,571849781 | |
| 241181_x_at | - | -1,763254337 | -2,335104118 | 0,571849781 | |
| 242061_at | - | -1,763254337 | -2,335104118 | 0,571849781 | |
| 32088_at | BLZF1 | 0,181391373 | -0,390416179 | 0,571807552 | |
| 209427_at | SMTN | 1,339784194 | 0,768343791 | 0,571440404 | |
| 231995_at | CAAP1 | 2,305800198 | 1,73441697 | 0,571383228 | |
| 1560964_at | - | -1,591296941 | -2,162639771 | 0,57134283 | |
| 1561508_at | - | -1,591296941 | -2,162639771 | 0,57134283 | |
| 1561612_at | - | -1,591296941 | -2,162639771 | 0,57134283 | |
| 1564474_at | ZBED3-AS1 | -1,591296941 | -2,162639771 | 0,57134283 | |
| 206806_at | DGKI | -1,591296941 | -2,162639771 | 0,57134283 | |
| 230302_at | - | -1,591296941 | -2,162639771 | 0,57134283 | |
| 231390_at | - | -1,591296941 | -2,162639771 | 0,57134283 | |
| 231676_s_at | - | -1,591296941 | -2,162639771 | 0,57134283 | |
| 231737_at | CACNG4 | -1,591296941 | -2,162639771 | 0,57134283 | |
| 236731_at | LUZP3P | -1,591296941 | -2,162639771 | 0,57134283 | |
| 238182_at | - | -1,591296941 | -2,162639771 | 0,57134283 | |
| 226060_at | RFT1 | 1,865674241 | 1,294497133 | 0,571177108 | |
| 1555269_a_at | ANO1 | 0,152582607 | -0,41858459 | 0,571167196 | |
| 207890_s_at | MMP25 | 0,152582607 | -0,41858459 | 0,571167196 | |
| 208115_x_at | C10orf137 | 0,152582607 | -0,41858459 | 0,571167196 | |
| 231830_x_at | RAB11FIP1 | 0,152582607 | -0,41858459 | 0,571167196 | |
| 232417_x_at | ZDHHC11 | 0,728550685 | 0,157549243 | 0,571001442 | |
| 204325_s_at | NF1 | -0,285837228 | -0,855751026 | 0,569913798 | |
| 205431_s_at | BMP5 | -0,285837228 | -0,855751026 | 0,569913798 | |
| 220241_at | TMCO3 | -0,285837228 | -0,855751026 | 0,569913798 | |
| 222225_at | RPL23AP53 | -0,285837228 | -0,855751026 | 0,569913798 | |
| 232509_at | PDE4DIP | -0,285837228 | -0,855751026 | 0,569913798 | |
| 239728_at | - | -0,285837228 | -0,855751026 | 0,569913798 | |
| 1554306_at | ITPKB | -1,838812296 | -2,408705138 | 0,569892841 | |
| 1555472_at | SORBS2 | -1,838812296 | -2,408705138 | 0,569892841 | |
| 1556469_s_at | - | -1,838812296 | -2,408705138 | 0,569892841 | |
| 1562629_a_at | KRT40 | -1,838812296 | -2,408705138 | 0,569892841 | |

| | | | | | |
|---|---|---|---|---|---|
| 1565597_at | - | -1,838812296 | -2,408705138 | 0,569892841 | |
| 1569673_at | - | -1,838812296 | -2,408705138 | 0,569892841 | |
| 215316_at | - | -1,838812296 | -2,408705138 | 0,569892841 | |
| 216372_at | - | -1,838812296 | -2,408705138 | 0,569892841 | |
| 220431_at | TMPRSS11E | -1,838812296 | -2,408705138 | 0,569892841 | |
| 233309_at | - | -1,838812296 | -2,408705138 | 0,569892841 | |
| 238550_at | RUFY2 | -1,838812296 | -2,408705138 | 0,569892841 | |
| 1568877_a_at | ACBD5 | -0,543051993 | -1,112851844 | 0,569799851 | |
| 208212_s_at | ALK | -0,543051993 | -1,112851844 | 0,569799851 | |
| 215407_s_at | ASTN2 | -0,543051993 | -1,112851844 | 0,569799851 | |
| 220825_s_at | KIRREL | -0,543051993 | -1,112851844 | 0,569799851 | |
| 224476_s_at | MESP1 | -0,543051993 | -1,112851844 | 0,569799851 | |
| 235110_at | PLA2G16 | -0,543051993 | -1,112851844 | 0,569799851 | |
| 236260_at | LOC100287598 | -0,543051993 | -1,112851844 | 0,569799851 | |
| 1553721_at | RNF152 | -2,384869671 | -2,954576755 | 0,569707084 | |
| 1554675_a_at | SYCE1 | -2,384869671 | -2,954576755 | 0,569707084 | |
| 1559901_s_at | LINC00478 | -2,384869671 | -2,954576755 | 0,569707084 | |
| 201430_s_at | DPYSL3 | -2,384869671 | -2,954576755 | 0,569707084 | |
| 209816_at | PTCH1 | -2,384869671 | -2,954576755 | 0,569707084 | |
| 230323_s_at | TMEM45B | -2,384869671 | -2,954576755 | 0,569707084 | |
| 204569_at | ICK | 0,792179257 | 0,222599095 | 0,569580162 | |
| 235659_at | - | 1,62481334 | 1,055274826 | 0,569538514 | |
| 1562914_a_at | FLJ25328 | 0,246434561 | -0,322870281 | 0,569304842 | |
| 208089_s_at | TDRD3 | 0,246434561 | -0,322870281 | 0,569304842 | |
| 210787_s_at | CAMKK2 | 0,246434561 | -0,322870281 | 0,569304842 | |
| 216044_x_at | FAM69A | 0,246434561 | -0,322870281 | 0,569304842 | |
| 217198_x_at | IGH@ /// IGHA2 / | 0,246434561 | -0,322870281 | 0,569304842 | |
| 226322_at | TMTC1 | 0,246434561 | -0,322870281 | 0,569304842 | |
| 1560101_at | SYDE2 | -2,667025278 | -3,236070201 | 0,569044923 | |
| 1559073_at | KDM4B | -1,518072576 | -2,086984744 | 0,568912168 | |
| 1561741_at | - | -1,518072576 | -2,086984744 | 0,568912168 | |
| 206771_at | UPK3A | -1,518072576 | -2,086984744 | 0,568912168 | |
| 210319_x_at | MSX2 | -1,518072576 | -2,086984744 | 0,568912168 | |
| 226654_at | MUC12 | -1,518072576 | -2,086984744 | 0,568912168 | |
| 228110_x_at | - | -1,518072576 | -2,086984744 | 0,568912168 | |
| 229133_s_at | ZNF397 | -1,518072576 | -2,086984744 | 0,568912168 | |
| 230384_at | ANKRD23 | -1,518072576 | -2,086984744 | 0,568912168 | |
| 235161_at | LOC100506451 | -1,518072576 | -2,086984744 | 0,568912168 | |
| 236415_at | DENND5B | -1,518072576 | -2,086984744 | 0,568912168 | |
| 236446_at | - | -1,518072576 | -2,086984744 | 0,568912168 | |
| 239786_at | - | -1,518072576 | -2,086984744 | 0,568912168 | |
| 240551_at | ZBTB45 | -1,518072576 | -2,086984744 | 0,568912168 | |
| 240722_at | LOC100505838 | -1,518072576 | -2,086984744 | 0,568912168 | |
| 207323_s_at | MBP | -0,196756757 | -0,765504029 | 0,568747272 | |
| 234753_x_at | - | -0,196756757 | -0,765504029 | 0,568747272 | |
| 221568_s_at | LIN7C | 4,379468341 | 3,810956512 | 0,568511829 | |
| 214848_at | YWHAZ | 0,962260738 | 0,393761504 | 0,568499234 | |
| 1554042_s_at | TMEM239 | -0,078265071 | -0,646746079 | 0,568481007 | |
| 1562733_at | LINC00092 | -0,078265071 | -0,646746079 | 0,568481007 | |
| 204152_s_at | MFNG | -0,078265071 | -0,646746079 | 0,568481007 | |
| 234086_at | - | -0,078265071 | -0,646746079 | 0,568481007 | |
| 235483_at | STX3 | -0,078265071 | -0,646746079 | 0,568481007 | |
| 214724_at | DIXDC1 | 1,04816921 | 0,479907041 | 0,568262169 | |
| 1555710_at | - | -2,779459409 | -3,347710726 | 0,568251317 | |
| 1555291_at | TRPV3 | -1,888254346 | -2,456406426 | 0,56815208 | |
| 1559607_s_at | GBP6 | -1,888254346 | -2,456406426 | 0,56815208 | |

| | | | | | |
|---|---|---|---|---|---|
| 1564070_s_at | HOTTIP | -1,888254346 | -2,456406426 | 0,56815208 | |
| 1564933_at | - | -1,888254346 | -2,456406426 | 0,56815208 | |
| 228368_at | ARHGAP20 | -1,888254346 | -2,456406426 | 0,56815208 | |
| 238838_at | TCERG1 | -1,888254346 | -2,456406426 | 0,56815208 | |
| 225290_at | ETNK1 | 2,182996494 | 1,614891062 | 0,568105433 | |
| 225471_s_at | AKT2 | 3,22766727 | 2,659583735 | 0,568083534 | |
| 203603_s_at | ZEB2 | 3,393780562 | 2,825817328 | 0,567963233 | |
| 1555076_at | ARHGAP25 | -0,926850548 | -1,494668682 | 0,567818133 | |
| 207938_at | PI15 | -0,926850548 | -1,494668682 | 0,567818133 | |
| 211322_s_at | SARDH | -0,926850548 | -1,494668682 | 0,567818133 | |
| 215142_at | CXorf27 | -0,926850548 | -1,494668682 | 0,567818133 | |
| 220863_at | MIP | -0,926850548 | -1,494668682 | 0,567818133 | |
| 222720_x_at | C1orf27 | -0,926850548 | -1,494668682 | 0,567818133 | |
| 244245_at | ANKRD9 | -0,926850548 | -1,494668682 | 0,567818133 | |
| 1554849_at | TOR1A | -1,047650486 | -1,615132408 | 0,567481922 | |
| 207314_x_at | KIR3DL2 /// LOC | -1,047650486 | -1,615132408 | 0,567481922 | |
| 209550_at | NDN | -1,047650486 | -1,615132408 | 0,567481922 | |
| 221289_at | DLX6 | -1,047650486 | -1,615132408 | 0,567481922 | |
| 233203_at | ROPN1 | -1,047650486 | -1,615132408 | 0,567481922 | |
| 225262_at | FOSL2 | 1,191926657 | 0,624460759 | 0,567465897 | |
| 204954_s_at | DYRK1B | 0,03125738 | -0,536087151 | 0,567344531 | |
| 209757_s_at | MYCN | 0,46522187 | -0,102100538 | 0,567322408 | |
| 244307_s_at | LOC100507539 | 0,46522187 | -0,102100538 | 0,567322408 | |
| 211818_s_at | PDE4C | -0,112808516 | -0,679763839 | 0,566955323 | |
| 229966_at | EWSR1 | -0,112808516 | -0,679763839 | 0,566955323 | |
| 242569_at | STAM2 | 0,30867765 | -0,258114234 | 0,566791884 | |
| 1558948_a_at | - | -1,470833215 | -2,037551727 | 0,566718511 | |
| 1559427_at | MCF2L | -1,470833215 | -2,037551727 | 0,566718511 | |
| 1567271_at | OR4C1P | -1,470833215 | -2,037551727 | 0,566718511 | |
| 1568903_at | - | -1,470833215 | -2,037551727 | 0,566718511 | |
| 205385_at | MDM2 | -1,470833215 | -2,037551727 | 0,566718511 | |
| 208292_at | BMP10 | -1,470833215 | -2,037551727 | 0,566718511 | |
| 215166_at | IGSF9B | -1,470833215 | -2,037551727 | 0,566718511 | |
| 222346_at | LAMA1 | -1,470833215 | -2,037551727 | 0,566718511 | |
| 235650_at | CDHR3 /// LOC1( | -1,470833215 | -2,037551727 | 0,566718511 | |
| 239242_at | SLC25A5-AS1 | -1,470833215 | -2,037551727 | 0,566718511 | |
| 1553719_s_at | ZNF548 | 0,67554678 | 0,109221392 | 0,566325388 | |
| 222741_s_at | SAYSD1 | 2,632180155 | 2,066015817 | 0,566164338 | |
| 1554478_a_at | HEATR3 | -0,00073712 | -0,566887459 | 0,566150339 | |
| 1562924_at | LOC340357 | -0,00073712 | -0,566887459 | 0,566150339 | |
| 233182_x_at | ATXN3 | -0,00073712 | -0,566887459 | 0,566150339 | |
| 237744_at | - | -0,00073712 | -0,566887459 | 0,566150339 | |
| 219105_x_at | ORC6 | 4,506303097 | 3,940211012 | 0,566092085 | |
| 1553811_at | FAM222A-AS1 | -1,937711681 | -2,503677622 | 0,565965941 | |
| 1557403_s_at | - | -1,937711681 | -2,503677622 | 0,565965941 | |
| 1561096_at | LOC285419 | -1,937711681 | -2,503677622 | 0,565965941 | |
| 222835_at | THSD4 | -1,937711681 | -2,503677622 | 0,565965941 | |
| 227061_at | - | -1,937711681 | -2,503677622 | 0,565965941 | |
| 233096_at | KIAA1109 | -1,937711681 | -2,503677622 | 0,565965941 | |
| 235467_s_at | KCNC4 | -1,937711681 | -2,503677622 | 0,565965941 | |
| 233486_at | - | 0,859074518 | 0,293518836 | 0,565555683 | |
| 218769_s_at | ANKRA2 | 2,611163891 | 2,045615873 | 0,565548018 | |
| 237817_at | SSR3 | 0,518867317 | -0,046302147 | 0,565169464 | |
| 1552997_a_at | IQCF1 | -0,59053611 | -1,154897679 | 0,564361568 | |
| 1556864_at | TCTN1 | -0,59053611 | -1,154897679 | 0,564361568 | |
| 1557248_at | ZNF587 | -0,59053611 | -1,154897679 | 0,564361568 | |

| | | | | | |
|---|---|---|---|---|---|
| 1564630_at | EDN1 | -0,59053611 | -1,154897679 | 0,564361568 | |
| 215507_x_at | - | -0,59053611 | -1,154897679 | 0,564361568 | |
| 216283_s_at | PVR | -0,59053611 | -1,154897679 | 0,564361568 | |
| 222787_s_at | TMEM106B | -0,59053611 | -1,154897679 | 0,564361568 | |
| 1557117_at | - | -1,173767127 | -1,738067356 | 0,564300229 | |
| 1557879_at | LOC100129175 | -1,173767127 | -1,738067356 | 0,564300229 | |
| 1561097_at | - | -1,173767127 | -1,738067356 | 0,564300229 | |
| 1561528_at | - | -1,173767127 | -1,738067356 | 0,564300229 | |
| 234231_at | CASP16 | -1,173767127 | -1,738067356 | 0,564300229 | |
| 234505_at | - | -1,173767127 | -1,738067356 | 0,564300229 | |
| 242153_at | LARP1B | -1,173767127 | -1,738067356 | 0,564300229 | |
| 1557804_at | - | -0,655928373 | -1,220210588 | 0,564282215 | |
| 1561258_at | TMEM151B | -0,655928373 | -1,220210588 | 0,564282215 | |
| 1568924_a_at | IQUB | -0,655928373 | -1,220210588 | 0,564282215 | |
| 206112_at | ANKRD7 | -0,655928373 | -1,220210588 | 0,564282215 | |
| 206277_at | P2RY2 | -0,655928373 | -1,220210588 | 0,564282215 | |
| 209542_x_at | IGF1 | -0,655928373 | -1,220210588 | 0,564282215 | |
| 211053_at | KCNG1 | -0,655928373 | -1,220210588 | 0,564282215 | |
| 221968_s_at | ZNF771 | -0,655928373 | -1,220210588 | 0,564282215 | |
| 235194_at | TPCN2 | -0,655928373 | -1,220210588 | 0,564282215 | |
| 236189_at | ANKRD50 | -0,655928373 | -1,220210588 | 0,564282215 | |
| 207165_at | HMMR | 4,695400025 | 4,131407181 | 0,563992844 | |
| 207387_s_at | GK | 1,764611101 | 1,200746069 | 0,563865031 | |
| 233333_x_at | AVIL | 0,592200562 | 0,028364014 | 0,563836547 | |
| 210963_s_at | GYG2 | 0,072842263 | -0,490927335 | 0,563769598 | |
| 219323_s_at | IL18BP | 0,072842263 | -0,490927335 | 0,563769598 | |
| 1560662_s_at | WHAMMP2 /// W | -2,43257096 | -2,996335703 | 0,563764743 | |
| 220351_at | CCRL1 | -2,43257096 | -2,996335703 | 0,563764743 | |
| 241365_at | SATB1 | -2,43257096 | -2,996335703 | 0,563764743 | |
| 209551_at | YIPF4 | 1,844820248 | 1,281471383 | 0,563348865 | |
| 229851_s_at | C11orf54 | 2,44295215 | 1,879785595 | 0,563166554 | |
| 225510_at | OAF | 1,331241794 | 0,768343791 | 0,562898003 | |
| 230935_at | LOC100506798 | 0,767063567 | 0,204310755 | 0,562752811 | |
| 211607_x_at | EGFR | 0,171847695 | -0,39061235 | 0,562460046 | |
| 244084_at | AIFM3 | 0,171847695 | -0,39061235 | 0,562460046 | |
| 232036_at | KLC1 | 0,425644434 | -0,136643983 | 0,562288416 | |
| 237747_at | - | 0,425644434 | -0,136643983 | 0,562288416 | |
| 211547_s_at | PAFAH1B1 | 1,094335495 | 0,532164371 | 0,562171124 | |
| 237052_x_at | GIGYF2 | 1,094335495 | 0,532164371 | 0,562171124 | |
| 1556735_at | - | -1,399512875 | -1,961547147 | 0,562034272 | |
| 1570571_at | CCDC91 | -1,399512875 | -1,961547147 | 0,562034272 | |
| 210366_at | SLCO1B1 | -1,399512875 | -1,961547147 | 0,562034272 | |
| 211490_at | ADRA1A | -1,399512875 | -1,961547147 | 0,562034272 | |
| 220722_s_at | SLC5A7 | -1,399512875 | -1,961547147 | 0,562034272 | |
| 230864_at | NIM1 | -1,399512875 | -1,961547147 | 0,562034272 | |
| 234633_at | KRTAP4-11 | -1,399512875 | -1,961547147 | 0,562034272 | |
| 236264_at | LPHN3 | -1,399512875 | -1,961547147 | 0,562034272 | |
| 240184_at | - | -1,399512875 | -1,961547147 | 0,562034272 | |
| 221014_s_at | RAB33B | 0,641402394 | 0,079428135 | 0,561974259 | |
| 1554619_at | MBLAC2 | -2,01371626 | -2,575129729 | 0,561413469 | |
| 1560917_at | - | -2,01371626 | -2,575129729 | 0,561413469 | |
| 1568755_a_at | - | -2,01371626 | -2,575129729 | 0,561413469 | |
| 1570272_at | - | -2,01371626 | -2,575129729 | 0,561413469 | |
| 221444_at | TAS2R16 | -2,01371626 | -2,575129729 | 0,561413469 | |
| 222925_at | DCDC2 | -2,01371626 | -2,575129729 | 0,561413469 | |
| 213786_at | TAX1BP1 | 1,841816345 | 1,281471383 | 0,560344962 | |

| | | | | | |
|---|---|---|---|---|---|
| 228047_at | SNORA72 | 0,87091093 | 0,310717132 | 0,560193798 | |
| 238406_x_at | SEZ6L2 | 1,163336153 | 0,603679647 | 0,559656507 | |
| 221757_at | PIK3IP1 | 0,853119696 | 0,293518836 | 0,55960086 | |
| 238147_at | TRIM46 | 0,853119696 | 0,293518836 | 0,55960086 | |
| 238551_at | FUT11 | 0,457392764 | -0,102100538 | 0,559493302 | |
| 242684_at | ZNF425 | 0,457392764 | -0,102100538 | 0,559493302 | |
| 207697_x_at | LILRB2 | -0,259832361 | -0,819238336 | 0,559405975 | |
| 233031_at | ZEB2 | -0,259832361 | -0,819238336 | 0,559405975 | |
| 206878_at | DAO | -0,352951799 | -0,912331589 | 0,55937979 | |
| 221286_s_at | MZB1 | -0,352951799 | -0,912331589 | 0,55937979 | |
| 229932_at | - | -0,352951799 | -0,912331589 | 0,55937979 | |
| 243017_at | LOC158572 | -0,352951799 | -0,912331589 | 0,55937979 | |
| 244737_at | - | -0,352951799 | -0,912331589 | 0,55937979 | |
| 207850_at | CXCL3 | 1,62481334 | 1,065442793 | 0,559370547 | |
| 242370_at | MTHFD2L | 1,283332461 | 0,724100169 | 0,559232292 | |
| 205913_at | PLIN1 | 0,209623097 | -0,34958478 | 0,559207878 | |
| 202422_s_at | ACSL4 | 2,755423794 | 2,196649505 | 0,558774289 | |
| 1559563_at | - | -0,055687482 | -0,614371577 | 0,558684095 | |
| 205374_at | SLN | -0,055687482 | -0,614371577 | 0,558684095 | |
| 210392_x_at | NR6A1 | -0,055687482 | -0,614371577 | 0,558684095 | |
| 225575_at | LIFR | -0,055687482 | -0,614371577 | 0,558684095 | |
| 227710_s_at | TPT1-AS1 | -0,055687482 | -0,614371577 | 0,558684095 | |
| 232112_at | RALGPS2 | -0,055687482 | -0,614371577 | 0,558684095 | |
| 1559105_at | LEKR1 | -1,353583928 | -1,912089812 | 0,558505884 | |
| 1568876_a_at | - | -1,353583928 | -1,912089812 | 0,558505884 | |
| 207378_at | TREH | -1,353583928 | -1,912089812 | 0,558505884 | |
| 207413_s_at | SCN5A | -1,353583928 | -1,912089812 | 0,558505884 | |
| 208201_at | DUX2 /// DUX3 /// | -1,353583928 | -1,912089812 | 0,558505884 | |
| 209983_s_at | NRXN2 | -1,353583928 | -1,912089812 | 0,558505884 | |
| 210723_x_at | - | -1,353583928 | -1,912089812 | 0,558505884 | |
| 211272_s_at | DGKA | -1,353583928 | -1,912089812 | 0,558505884 | |
| 230889_at | LOC645321 | -1,353583928 | -1,912089812 | 0,558505884 | |
| 239487_at | - | -1,353583928 | -1,912089812 | 0,558505884 | |
| 242860_at | - | -1,353583928 | -1,912089812 | 0,558505884 | |
| 244150_at | - | -1,353583928 | -1,912089812 | 0,558505884 | |
| 244821_at | - | -1,353583928 | -1,912089812 | 0,558505884 | |
| 235378_at | FAM161B | -0,172274514 | -0,730778808 | 0,558504294 | |
| 1563945_at | LOC284100 | 0,533835206 | -0,024572586 | 0,558407793 | |
| 209875_s_at | SPP1 | 0,533835206 | -0,024572586 | 0,558407793 | |
| 203840_at | BLZF1 | 1,434549838 | 0,876248481 | 0,558301357 | |
| 1552736_a_at | NETO1 | -2,063149277 | -2,621331989 | 0,558182712 | |
| 1559354_a_at | - | -2,063149277 | -2,621331989 | 0,558182712 | |
| 1563319_at | - | -2,063149277 | -2,621331989 | 0,558182712 | |
| 208531_at | SERPINA2 | -2,063149277 | -2,621331989 | 0,558182712 | |
| 210108_at | CACNA1D | -2,063149277 | -2,621331989 | 0,558182712 | |
| 216124_at | - | -2,063149277 | -2,621331989 | 0,558182712 | |
| 229125_at | KANK4 | -2,063149277 | -2,621331989 | 0,558182712 | |
| 237550_at | - | -2,063149277 | -2,621331989 | 0,558182712 | |
| 244022_at | - | -2,063149277 | -2,621331989 | 0,558182712 | |
| 1556786_at | PDE5A | -2,479842156 | -3,037500868 | 0,557658712 | |
| 204869_at | PCSK2 | -2,479842156 | -3,037500868 | 0,557658712 | |
| 244159_at | - | -2,479842156 | -3,037500868 | 0,557658712 | |
| 226973_at | VSTM2L | 0,273439642 | -0,283667828 | 0,557107469 | |
| 206537_at | XIAP | -0,706943342 | -1,264005341 | 0,557061999 | |
| 208023_at | TNFRSF4 | -0,706943342 | -1,264005341 | 0,557061999 | |
| 228896_at | - | -0,706943342 | -1,264005341 | 0,557061999 | |

| | | | | | |
|---|---|---|---|---|---|
| 1565974_at | - | -0,089689931 | -0,646746079 | 0,557056147 | |
| 217365_at | PRAMEF11 | -0,089689931 | -0,646746079 | 0,557056147 | |
| 220589_s_at | ITFG2 /// LOC10( | -0,089689931 | -0,646746079 | 0,557056147 | |
| 220764_at | PPP4R2 | -0,089689931 | -0,646746079 | 0,557056147 | |
| 229079_at | EHMT2 | -0,089689931 | -0,646746079 | 0,557056147 | |
| 229343_at | GTSE1 | -0,089689931 | -0,646746079 | 0,557056147 | |
| 230438_at | TBX15 | -0,089689931 | -0,646746079 | 0,557056147 | |
| 1560443_at | - | 0,020670649 | -0,536087151 | 0,5567578 | |
| 226766_at | ROBO2 | 0,020670649 | -0,536087151 | 0,5567578 | |
| 226984_at | FGD5 | 0,020670649 | -0,536087151 | 0,5567578 | |
| 207863_at | ADPRH | -0,299034815 | -0,855751026 | 0,556716211 | |
| 210080_x_at | CELA3A | -0,299034815 | -0,855751026 | 0,556716211 | |
| 215454_x_at | SFTPC | -0,299034815 | -0,855751026 | 0,556716211 | |
| 219853_at | FKRP | -0,299034815 | -0,855751026 | 0,556716211 | |
| 224125_at | PLEKHN1 | -0,299034815 | -0,855751026 | 0,556716211 | |
| 234722_x_at | OBP2B | -0,299034815 | -0,855751026 | 0,556716211 | |
| 235550_at | MAP9 | -0,299034815 | -0,855751026 | 0,556716211 | |
| 241774_at | FLJ31306 | -0,299034815 | -0,855751026 | 0,556716211 | |
| 213365_at | ERI2 | 2,352981171 | 1,796776588 | 0,556204583 | |
| 235459_at | RNF41 | 0,359971644 | -0,19610998 | 0,556081624 | |
| 1553517_at | FERD3L | -0,394749123 | -0,950686014 | 0,555936891 | |
| 1562930_at | SRRM2-AS1 | -0,394749123 | -0,950686014 | 0,555936891 | |
| 204821_at | BTN3A3 | -0,394749123 | -0,950686014 | 0,555936891 | |
| 206369_s_at | PIK3CG | -0,394749123 | -0,950686014 | 0,555936891 | |
| 209791_at | PADI2 | -0,394749123 | -0,950686014 | 0,555936891 | |
| 227676_at | FAM3D | -0,394749123 | -0,950686014 | 0,555936891 | |
| 219784_at | C16orf95 | 1,247462054 | 0,691645685 | 0,555816368 | |
| 219121_s_at | ESRP1 | -0,986356909 | -1,541908042 | 0,555551133 | |
| 228912_at | VIL1 | -0,986356909 | -1,541908042 | 0,555551133 | |
| 230845_at | HOXB-AS5 | -0,986356909 | -1,541908042 | 0,555551133 | |
| 236324_at | MBP | -0,986356909 | -1,541908042 | 0,555551133 | |
| 239172_x_at | MDFIC | -0,986356909 | -1,541908042 | 0,555551133 | |
| 207014_at | GABRA2 | -2,756478458 | -3,311072652 | 0,554594194 | |
| 242033_at | RNF180 | -2,756478458 | -3,311072652 | 0,554594194 | |
| 1554978_at | ERICH1-AS1 | -1,308181863 | -1,862647763 | 0,5544659 | |
| 1564193_at | FLJ39061 /// LOC | -1,308181863 | -1,862647763 | 0,5544659 | |
| 207665_at | ADAM21 | -1,308181863 | -1,862647763 | 0,5544659 | |
| 210578_at | TRIM10 | -1,308181863 | -1,862647763 | 0,5544659 | |
| 214388_at | - | -1,308181863 | -1,862647763 | 0,5544659 | |
| 215559_at | ABCC6 | -1,308181863 | -1,862647763 | 0,5544659 | |
| 243319_at | - | -1,308181863 | -1,862647763 | 0,5544659 | |
| 218981_at | ACN9 | 3,493810203 | 2,939363796 | 0,554446407 | |
| 204302_s_at | CTIF | 0,417596971 | -0,136643983 | 0,554240953 | |
| 232589_at | LOC100506029 / | 0,417596971 | -0,136643983 | 0,554240953 | |
| 52651_at | COL8A2 | -0,526600223 | -1,080731507 | 0,554131284 | |
| 1569980_x_at | HKR1 | -0,496991414 | -1,050406615 | 0,553415201 | |
| 213909_at | LRRC15 | -0,496991414 | -1,050406615 | 0,553415201 | |
| 214186_s_at | HCG26 | -0,496991414 | -1,050406615 | 0,553415201 | |
| 218808_at | DALRD3 | -0,496991414 | -1,050406615 | 0,553415201 | |
| 229103_at | WNT3 | -0,496991414 | -1,050406615 | 0,553415201 | |
| 238771_at | - | -0,496991414 | -1,050406615 | 0,553415201 | |
| 240454_at | - | -0,496991414 | -1,050406615 | 0,553415201 | |
| 201568_at | UQCRQ | 5,958628005 | 5,405319208 | 0,553308797 | |
| 223261_at | POLK | 2,089851283 | 1,536652708 | 0,553198576 | |
| 233809_at | C15orf63 | 1,04816921 | 0,495031851 | 0,55313736 | |
| 36920_at | MTM1 | 0,162247775 | -0,390565597 | 0,552813372 | |

| | | | | | |
|---|---|---|---|---|---|
| 212769_at | TLE3 | 0,661985843 | 0,109221392 | 0,55276445 | |
| 236691_at | - | 0,661985843 | 0,109221392 | 0,55276445 | |
| 205191_at | RP2 | 3,217273782 | 2,664608955 | 0,552664827 | |
| 204041_at | MAOB | 1,789769151 | 1,23718272 | 0,552586431 | |
| 236449_at | CSTB | 1,210676994 | 0,658444279 | 0,552232715 | |
| 220762_s_at | GNB1L | 1,473887017 | 0,921731975 | 0,552155042 | |
| 207913_at | CYP2F1 | -2,138804305 | -2,690860744 | 0,552056439 | |
| 210491_at | - | -2,138804305 | -2,690860744 | 0,552056439 | |
| 210913_at | CDH20 | -2,138804305 | -2,690860744 | 0,552056439 | |
| 211492_s_at | ADRA1A | -2,138804305 | -2,690860744 | 0,552056439 | |
| 216185_at | FUT9 | -2,138804305 | -2,690860744 | 0,552056439 | |
| 231911_at | ERMN | -2,138804305 | -2,690860744 | 0,552056439 | |
| 233546_at | LOC283075 | -2,138804305 | -2,690860744 | 0,552056439 | |
| 234684_s_at | KRTAP4-4 | -2,138804305 | -2,690860744 | 0,552056439 | |
| 234943_at | LOC400927 | -2,138804305 | -2,690860744 | 0,552056439 | |
| 239479_x_at | - | -2,138804305 | -2,690860744 | 0,552056439 | |
| 241875_at | - | -2,138804305 | -2,690860744 | 0,552056439 | |
| 215073_s_at | NR2F2 | 1,369290453 | 0,817300337 | 0,551990116 | |
| 1567105_at | - | 0,133056859 | -0,41858459 | 0,551641448 | |
| 208257_x_at | PSG1 | 0,133056859 | -0,41858459 | 0,551641448 | |
| 218744_s_at | PACSIN3 | 0,133056859 | -0,41858459 | 0,551641448 | |
| 1559883_s_at | SAMHD1 | 0,928678725 | 0,377530647 | 0,551148078 | |
| 204953_at | SNAP91 | 0,228146222 | -0,322870281 | 0,551016503 | |
| 239948_at | NUP153 | 0,228146222 | -0,322870281 | 0,551016503 | |
| 1555948_s_at | FAM120A | 2,693452197 | 2,14244844 | 0,551003758 | |
| 209571_at | CIR1 | 1,119359127 | 0,568365095 | 0,550994032 | |
| 222472_at | AFTPH | 2,562727117 | 2,011838132 | 0,550888985 | |
| 60474_at | FERMT1 | -1,511180288 | -2,062056522 | 0,550876233 | |
| 236485_at | LOC100507111 | 2,236584753 | 1,685812099 | 0,550772653 | |
| 218102_at | DERA | 0,984222186 | 0,433557298 | 0,550664887 | |
| 200849_s_at | AHCYL1 | 3,439206878 | 2,888726553 | 0,550480325 | |
| 35846_at | THRA | 0,260000594 | -0,289989561 | 0,549990155 | |
| 213917_at | PAX8 | 0,20027154 | -0,34958478 | 0,54985632 | |
| 203668_at | MAN2C1 | 0,291166226 | -0,258114234 | 0,54928046 | |
| 215654_at | BCAT2 | 0,291166226 | -0,258114234 | 0,54928046 | |
| 235585_at | - | 0,798390451 | 0,249604176 | 0,548786275 | |
| 234347_s_at | DENR | 0,555999837 | 0,007421914 | 0,548577923 | |
| 225962_at | ZNRF1 | 0,859074518 | 0,310717132 | 0,548357386 | |
| 1556925_at | SMC3 | -2,187914861 | -2,735862426 | 0,547947565 | |
| 1556963_at | - | -2,187914861 | -2,735862426 | 0,547947565 | |
| 1561849_at | PKD1L2 | -2,187914861 | -2,735862426 | 0,547947565 | |
| 1563132_at | - | -2,187914861 | -2,735862426 | 0,547947565 | |
| 1569240_at | ZNF93 | -2,187914861 | -2,735862426 | 0,547947565 | |
| 206011_at | CASP1 | -2,187914861 | -2,735862426 | 0,547947565 | |
| 216067_at | - | -2,187914861 | -2,735862426 | 0,547947565 | |
| 225790_at | MSRB3 | -2,187914861 | -2,735862426 | 0,547947565 | |
| 233251_at | STRBP | -2,187914861 | -2,735862426 | 0,547947565 | |
| 242166_at | - | -2,187914861 | -2,735862426 | 0,547947565 | |
| 243949_at | - | -2,187914861 | -2,735862426 | 0,547947565 | |
| 218040_at | PRPF38B | 3,28739962 | 2,739547836 | 0,547851784 | |
| 1560896_a_at | - | -0,946847173 | -1,494668682 | 0,547821509 | |
| 1561181_at | - | -0,946847173 | -1,494668682 | 0,547821509 | |
| 205114_s_at | CCL3 /// CCL3L1 | -0,946847173 | -1,494668682 | 0,547821509 | |
| 207819_s_at | ABCB4 | -0,946847173 | -1,494668682 | 0,547821509 | |
| 210748_at | - | -0,946847173 | -1,494668682 | 0,547821509 | |
| 216654_at | TNXB | -0,946847173 | -1,494668682 | 0,547821509 | |

| | | | | | |
|---|---|---|---|---|---|
| 223609_at | ROPN1L | -0,946847173 | -1,494668682 | 0,547821509 | |
| 236887_at | KIN | -0,946847173 | -1,494668682 | 0,547821509 | |
| 238186_at | - | -0,946847173 | -1,494668682 | 0,547821509 | |
| 221778_at | JHDM1D | 0,351548291 | -0,19610998 | 0,547658271 | |
| 219694_at | FAM105A | 0,841135803 | 0,293518836 | 0,547616967 | |
| 202987_at | TRAF3IP2 | -2,551294263 | -3,098858316 | 0,547564053 | |
| 217206_at | - | -2,551294263 | -3,098858316 | 0,547564053 | |
| 221127_s_at | DKK3 | -2,551294263 | -3,098858316 | 0,547564053 | |
| 230559_x_at | FGD4 | -2,551294263 | -3,098858316 | 0,547564053 | |
| 233377_at | - | -2,551294263 | -3,098858316 | 0,547564053 | |
| 237778_at | - | -2,551294263 | -3,098858316 | 0,547564053 | |
| 240322_at | - | -2,551294263 | -3,098858316 | 0,547564053 | |
| 205006_s_at | NMT2 | 1,841816345 | 1,294497133 | 0,547319212 | |
| 216962_at | RPAIN | 1,841816345 | 1,294497133 | 0,547319212 | |
| 1558438_a_at | IGHA1 | -0,67289328 | -1,220210588 | 0,547317308 | |
| 1570297_at | - | -0,67289328 | -1,220210588 | 0,547317308 | |
| 205199_at | CA9 | -0,67289328 | -1,220210588 | 0,547317308 | |
| 216072_at | - | -0,67289328 | -1,220210588 | 0,547317308 | |
| 229159_at | THSD7A | -0,67289328 | -1,220210588 | 0,547317308 | |
| 214765_s_at | NAAA | 0,606430281 | 0,059218869 | 0,547211413 | |
| 1569443_s_at | - | -1,240169874 | -1,787089803 | 0,546919929 | |
| 209570_s_at | D4S234E | -1,240169874 | -1,787089803 | 0,546919929 | |
| 215400_x_at | ADCY9 | -1,240169874 | -1,787089803 | 0,546919929 | |
| 216857_at | IL23A | -1,240169874 | -1,787089803 | 0,546919929 | |
| 217058_at | GNAS | -1,240169874 | -1,787089803 | 0,546919929 | |
| 218813_s_at | SH3GLB2 | -1,240169874 | -1,787089803 | 0,546919929 | |
| 221317_x_at | PCDHB6 | -1,240169874 | -1,787089803 | 0,546919929 | |
| 221980_at | EMILIN2 | -1,240169874 | -1,787089803 | 0,546919929 | |
| 224550_s_at | MRVI1 | -1,240169874 | -1,787089803 | 0,546919929 | |
| 226950_at | ACVRL1 | -1,240169874 | -1,787089803 | 0,546919929 | |
| 233515_at | - | -1,240169874 | -1,787089803 | 0,546919929 | |
| 227595_at | ZMYM6 | 1,283332461 | 0,73688026 | 0,5464522 | |
| 226468_at | RNF115 | 1,883312263 | 1,337086025 | 0,546226238 | |
| 222538_s_at | APPL1 | 1,314003737 | 0,768343791 | 0,545659946 | |
| 214081_at | PLXDC1 | -0,366776884 | -0,912331589 | 0,545554705 | |
| 220777_at | KIF13A | -0,366776884 | -0,912331589 | 0,545554705 | |
| 223496_s_at | CCDC8 | -0,366776884 | -0,912331589 | 0,545554705 | |
| 233654_at | PIGG | -0,366776884 | -0,912331589 | 0,545554705 | |
| 239403_at | CCDC120 | -0,366776884 | -0,912331589 | 0,545554705 | |
| 1563489_at | LOC285638 | -0,83191556 | -1,377419394 | 0,545503834 | |
| 205637_s_at | SH3GL3 | -0,83191556 | -1,377419394 | 0,545503834 | |
| 207586_at | SHH | -0,83191556 | -1,377419394 | 0,545503834 | |
| 211608_at | - | -0,83191556 | -1,377419394 | 0,545503834 | |
| 230693_at | ATP2A1 | -0,83191556 | -1,377419394 | 0,545503834 | |
| 237103_at | - | -0,83191556 | -1,377419394 | 0,545503834 | |
| 242103_at | TMEM86A | -0,83191556 | -1,377419394 | 0,545503834 | |
| 1556794_at | - | 0,384950573 | -0,160136748 | 0,545087322 | |
| 1565576_at | - | 0,702292136 | 0,157549243 | 0,544742893 | |
| 235902_at | - | 0,702292136 | 0,157549243 | 0,544742893 | |
| 1564190_x_at | ZNF519 | 0,083054335 | -0,461594427 | 0,544648761 | |
| 208536_s_at | BCL2L11 | 0,083054335 | -0,461594427 | 0,544648761 | |
| 1554735_a_at | TRIM7 | -0,022466681 | -0,566887459 | 0,544420779 | |
| 233287_at | SLC6A17 | 1,735773781 | 1,191491281 | 0,5442825 | |
| 202083_s_at | SEC14L1 | 1,62481334 | 1,080561626 | 0,544251714 | |
| 205745_x_at | ADAM17 | 1,767779954 | 1,223626587 | 0,544153366 | |
| 228619_x_at | TIPRL | 1,954567604 | 1,410714371 | 0,543853232 | |

| | | | | | |
|---|---|---|---|---|---|
| 202877_s_at | CD93 | -0,221656139 | -0,765504029 | 0,54384789 | |
| 206270_at | PRKCG | -0,221656139 | -0,765504029 | 0,54384789 | |
| 218174_s_at | C10orf57 | -0,221656139 | -0,765504029 | 0,54384789 | |
| 225241_at | CCDC80 | -0,221656139 | -0,765504029 | 0,54384789 | |
| 228304_at | RBM43 | -0,221656139 | -0,765504029 | 0,54384789 | |
| 231014_at | TRIM50 | -0,221656139 | -0,765504029 | 0,54384789 | |
| 234724_x_at | PCDHB18 | -0,221656139 | -0,765504029 | 0,54384789 | |
| 240464_at | - | -0,221656139 | -0,765504029 | 0,54384789 | |
| 225303_at | KIRREL | 0,44160593 | -0,102100538 | 0,543706467 | |
| 1558621_at | CABLES1 | -1,543331864 | -2,086984744 | 0,543652879 | |
| 1559321_at | - | -1,543331864 | -2,086984744 | 0,543652879 | |
| 1560334_at | MEGF11 | -1,543331864 | -2,086984744 | 0,543652879 | |
| 1565674_at | FCGR2A /// FCG | -1,543331864 | -2,086984744 | 0,543652879 | |
| 1569090_x_at | FLJ35390 | -1,543331864 | -2,086984744 | 0,543652879 | |
| 1570177_at | - | -1,543331864 | -2,086984744 | 0,543652879 | |
| 206295_at | IL18 | -1,543331864 | -2,086984744 | 0,543652879 | |
| 207361_at | HBP1 | -1,543331864 | -2,086984744 | 0,543652879 | |
| 216749_at | - | -1,543331864 | -2,086984744 | 0,543652879 | |
| 217072_at | CD300A | -1,543331864 | -2,086984744 | 0,543652879 | |
| 220111_s_at | ANO2 | -1,543331864 | -2,086984744 | 0,543652879 | |
| 221107_at | CHRNA9 | -1,543331864 | -2,086984744 | 0,543652879 | |
| 222910_s_at | PEX5L | -1,543331864 | -2,086984744 | 0,543652879 | |
| 230193_at | WDR66 | -1,543331864 | -2,086984744 | 0,543652879 | |
| 234454_at | - | -1,543331864 | -2,086984744 | 0,543652879 | |
| 236743_at | AGPAT6 | -1,543331864 | -2,086984744 | 0,543652879 | |
| 238603_at | LOC254559 | -1,543331864 | -2,086984744 | 0,543652879 | |
| 239831_at | TMEM106C | -1,543331864 | -2,086984744 | 0,543652879 | |
| 228188_at | FOSL2 | 0,747935635 | 0,204310755 | 0,54362488 | |
| 1562675_at | C1orf86 | 1,360921491 | 0,817300337 | 0,543621154 | |
| 1569307_s_at | - | -2,236765673 | -2,780313924 | 0,543548252 | |
| 208075_s_at | CCL7 | -2,236765673 | -2,780313924 | 0,543548252 | |
| 217083_at | - | -2,236765673 | -2,780313924 | 0,543548252 | |
| 225491_at | SLC1A2 | -2,236765673 | -2,780313924 | 0,543548252 | |
| 227643_at | TPPP | -2,236765673 | -2,780313924 | 0,543548252 | |
| 228792_at | - | -2,236765673 | -2,780313924 | 0,543548252 | |
| 237790_at | SCN8A | -2,236765673 | -2,780313924 | 0,543548252 | |
| 237946_at | - | -2,236765673 | -2,780313924 | 0,543548252 | |
| 242686_at | STARD13 | -2,236765673 | -2,780313924 | 0,543548252 | |
| 1559007_s_at | - | -0,136301282 | -0,679763839 | 0,543462557 | |
| 208188_at | KRT9 | -0,136301282 | -0,679763839 | 0,543462557 | |
| 221287_at | RNASEL | -0,136301282 | -0,679763839 | 0,543462557 | |
| 232185_at | C20orf132 | -0,136301282 | -0,679763839 | 0,543462557 | |
| 238452_at | FCRLB | 0,810733188 | 0,26733076 | 0,543402428 | |
| 219460_s_at | TMEM127 | 1,880387527 | 1,337086025 | 0,543301502 | |
| 213209_at | TAF6L | 0,052199481 | -0,490927335 | 0,543126816 | |
| 230256_at | RUSC1-AS1 | 0,052199481 | -0,490927335 | 0,543126816 | |
| 239850_at | - | 0,052199481 | -0,490927335 | 0,543126816 | |
| 1566129_at | LIMS1 | -0,467091869 | -1,010192375 | 0,543100506 | |
| 214746_s_at | ZNF467 | -0,467091869 | -1,010192375 | 0,543100506 | |
| 228577_x_at | ODF2L | -0,467091869 | -1,010192375 | 0,543100506 | |
| 230452_at | FLJ42351 | -0,467091869 | -1,010192375 | 0,543100506 | |
| 211038_s_at | CROCCP2 | 3,420201053 | 2,877228323 | 0,54297273 | |
| 228423_at | MAP9 | 1,201332287 | 0,658444279 | 0,542888008 | |
| 235282_at | LOC100506295 | 1,201332287 | 0,658444279 | 0,542888008 | |
| 235330_at | CCDC117 | 0,496120222 | -0,046302147 | 0,542422369 | |
| 227179_at | STAU2 | 2,292660284 | 1,750261389 | 0,542398895 | |

| | | | | | |
|---|---|---|---|---|---|
| 215446_s_at | LOX | 1,073999308 | 0,532164371 | 0,541834937 | |
| 233503_at | CCDC169 | 0,123193849 | -0,41858459 | 0,541778439 | |
| 224994_at | CAMK2D | 1,406361373 | 0,864649967 | 0,541711406 | |
| 1559745_at | - | -1,196375121 | -1,738067356 | 0,541692234 | |
| 204607_at | HMGCS2 | -1,196375121 | -1,738067356 | 0,541692234 | |
| 205949_at | CA1 | -1,196375121 | -1,738067356 | 0,541692234 | |
| 209266_s_at | SLC39A8 | -1,196375121 | -1,738067356 | 0,541692234 | |
| 231908_at | ZDHHC18 | -1,196375121 | -1,738067356 | 0,541692234 | |
| 244439_at | SPRED1 | -1,196375121 | -1,738067356 | 0,541692234 | |
| 222294_s_at | RAB27A | 1,960118853 | 1,418668082 | 0,541450771 | |
| 208245_at | RAB9BP1 | -2,597496523 | -3,138626879 | 0,541130356 | |
| 211592_s_at | CACNA1C | -2,597496523 | -3,138626879 | 0,541130356 | |
| 215475_at | - | -2,597496523 | -3,138626879 | 0,541130356 | |
| 244230_at | - | -2,597496523 | -3,138626879 | 0,541130356 | |
| 209829_at | FAM65B | 1,434549838 | 0,893473268 | 0,54107657 | |
| 220643_s_at | FAIM | 2,206540699 | 1,665902186 | 0,540638514 | |
| 235354_s_at | RSRC1 | 0,934330293 | 0,393761504 | 0,540568788 | |
| 37145_at | GNLY | -1,251195656 | -1,791342178 | 0,540146522 | |
| 219149_x_at | DBR1 | 2,72965419 | 2,189694216 | 0,539959974 | |
| 1557661_at | - | -1,964176657 | -2,503677622 | 0,539500966 | |
| 1559174_at | RAB6A | -1,964176657 | -2,503677622 | 0,539500966 | |
| 1565705_x_at | - | -1,964176657 | -2,503677622 | 0,539500966 | |
| 1569439_at | - | -1,964176657 | -2,503677622 | 0,539500966 | |
| 206354_at | SLCO1B3 | -1,964176657 | -2,503677622 | 0,539500966 | |
| 211523_at | GNRHR | -1,964176657 | -2,503677622 | 0,539500966 | |
| 214899_at | ZNF780B | -1,964176657 | -2,503677622 | 0,539500966 | |
| 216123_x_at | - | -1,964176657 | -2,503677622 | 0,539500966 | |
| 216260_at | DICER1 | -1,964176657 | -2,503677622 | 0,539500966 | |
| 233916_at | NYAP2 | -1,964176657 | -2,503677622 | 0,539500966 | |
| 234279_at | - | -1,964176657 | -2,503677622 | 0,539500966 | |
| 239889_at | SERP2 | -1,964176657 | -2,503677622 | 0,539500966 | |
| 241313_at | DOCK4 | -1,964176657 | -2,503677622 | 0,539500966 | |
| 244557_at | C11orf55 | -1,964176657 | -2,503677622 | 0,539500966 | |
| 212431_at | HMGXB3 | 3,130084275 | 2,590852465 | 0,53923181 | |
| 206709_x_at | GPT | 0,255492549 | -0,283667828 | 0,539160377 | |
| 212700_x_at | PLEKHM1 /// PLE | 0,255492549 | -0,283667828 | 0,539160377 | |
| 221180_at | YSK4 | 0,255492549 | -0,283667828 | 0,539160377 | |
| 205341_at | EHD2 | 0,715481152 | 0,176436073 | 0,539045078 | |
| 210752_s_at | MLX | 0,715481152 | 0,176436073 | 0,539045078 | |
| 222151_s_at | CEP63 | 0,715481152 | 0,176436073 | 0,539045078 | |
| 219147_s_at | NMRK1 | 0,95671774 | 0,417770464 | 0,538947277 | |
| 205600_x_at | HOXB5 | -0,57445847 | -1,112851844 | 0,538393374 | |
| 230793_at | LRRC16A | -0,57445847 | -1,112851844 | 0,538393374 | |
| 236785_at | LOC100507390 | -0,57445847 | -1,112851844 | 0,538393374 | |
| 243552_at | MBTD1 | -0,57445847 | -1,112851844 | 0,538393374 | |
| 243853_at | - | -0,57445847 | -1,112851844 | 0,538393374 | |
| 1552976_at | - | -0,512251684 | -1,050406615 | 0,538154931 | |
| 1562482_at | - | -0,512251684 | -1,050406615 | 0,538154931 | |
| 213433_at | ARL3 | -0,512251684 | -1,050406615 | 0,538154931 | |
| 217690_at | ENOSF1 | -0,512251684 | -1,050406615 | 0,538154931 | |
| 235737_at | TSLP | -0,512251684 | -1,050406615 | 0,538154931 | |
| 235772_at | - | -0,512251684 | -1,050406615 | 0,538154931 | |
| 236167_at | - | -0,512251684 | -1,050406615 | 0,538154931 | |
| 212651_at | RHOBTB1 | 0,760715727 | 0,222599095 | 0,538116632 | |
| 229499_at | CAPN13 | 0,695652136 | 0,157549243 | 0,538102893 | |
| 239364_at | - | 0,695652136 | 0,157549243 | 0,538102893 | |

| | | | | | |
|---|---|---|---|---|---|
| 207100_s_at | VAMP1 | 0,401366113 | -0,136643983 | 0,538010096 | |
| 1559064_at | NUP153 | 1,442503549 | 0,904843258 | 0,53766029 | |
| 214205_x_at | GLRX3 | 3,325531666 | 2,787940144 | 0,537591521 | |
| 1555264_a_at | LINC00598 | -1,424023738 | -1,961547147 | 0,537523409 | |
| 1561967_at | FLNB-AS1 | -1,424023738 | -1,961547147 | 0,537523409 | |
| 206779_s_at | ASMT | -1,424023738 | -1,961547147 | 0,537523409 | |
| 208247_at | ERC2-IT1 | -1,424023738 | -1,961547147 | 0,537523409 | |
| 208552_at | GRIK4 | -1,424023738 | -1,961547147 | 0,537523409 | |
| 217069_at | MLL4 | -1,424023738 | -1,961547147 | 0,537523409 | |
| 232412_at | FBXL20 | -1,424023738 | -1,961547147 | 0,537523409 | |
| 1554940_a_at | LOC388882 | -0,79540287 | -1,332017329 | 0,536614459 | |
| 202285_s_at | TACSTD2 | -0,79540287 | -1,332017329 | 0,536614459 | |
| 208272_at | RANBP3 | -0,79540287 | -1,332017329 | 0,536614459 | |
| 209807_s_at | NFIX | -0,79540287 | -1,332017329 | 0,536614459 | |
| 213781_at | PPP1R37 | -0,79540287 | -1,332017329 | 0,536614459 | |
| 230458_at | SLC45A1 | -0,79540287 | -1,332017329 | 0,536614459 | |
| 242729_at | LOC100506866 | -0,79540287 | -1,332017329 | 0,536614459 | |
| 204399_s_at | EML2 | -0,078265071 | -0,614371577 | 0,536106505 | |
| 235051_at | CCDC50 | -0,078265071 | -0,614371577 | 0,536106505 | |
| 233441_at | ONECUT2 | -2,866296344 | -3,402185466 | 0,535889122 | |
| 1555122_at | GPR125 | -2,311268652 | -2,847001814 | 0,535733163 | |
| 202936_s_at | SOX9 | -2,311268652 | -2,847001814 | 0,535733163 | |
| 211567_at | - | -2,311268652 | -2,847001814 | 0,535733163 | |
| 216468_s_at | ZNF682 | -2,311268652 | -2,847001814 | 0,535733163 | |
| 220132_s_at | CLEC2D | -2,311268652 | -2,847001814 | 0,535733163 | |
| 234094_x_at | DNAJC21 | -2,311268652 | -2,847001814 | 0,535733163 | |
| 239092_at | ITGA8 | -2,311268652 | -2,847001814 | 0,535733163 | |
| 239486_at | - | -2,311268652 | -2,847001814 | 0,535733163 | |
| 239722_at | C5orf49 | -2,311268652 | -2,847001814 | 0,535733163 | |
| 241217_x_at | - | -2,311268652 | -2,847001814 | 0,535733163 | |
| 242764_at | DCHS2 | -2,311268652 | -2,847001814 | 0,535733163 | |
| 242792_at | - | -2,311268652 | -2,847001814 | 0,535733163 | |
| 1555370_a_at | CAMTA1 | 0,888485433 | 0,352836757 | 0,535648676 | |
| 1560588_at | - | -0,00073712 | -0,536087151 | 0,535350031 | |
| 1555788_a_at | TRIB3 | 1,089278259 | 0,553993624 | 0,535284635 | |
| 1553585_a_at | MAGEA10-MAGE | -0,888496123 | -1,423348341 | 0,534852219 | |
| 1559576_at | WDR78 | -0,888496123 | -1,423348341 | 0,534852219 | |
| 1561155_at | - | -0,888496123 | -1,423348341 | 0,534852219 | |
| 207764_s_at | HIPK3 | -0,888496123 | -1,423348341 | 0,534852219 | |
| 215560_x_at | MTRF1L | -0,888496123 | -1,423348341 | 0,534852219 | |
| 230561_s_at | KANSL1L | -0,888496123 | -1,423348341 | 0,534852219 | |
| 240902_at | LOC283624 | -0,888496123 | -1,423348341 | 0,534852219 | |
| 219805_at | CXorf56 | 0,87091093 | 0,336136178 | 0,534774752 | |
| 1557139_at | DDX11-AS1 | -2,64318869 | -3,177802286 | 0,534613596 | |
| 1564767_at | - | -2,64318869 | -3,177802286 | 0,534613596 | |
| 216844_at | ZC3H7B | -2,64318869 | -3,177802286 | 0,534613596 | |
| 225207_at | PDK4 | 0,072842263 | -0,461594427 | 0,534436689 | |
| 238885_at | KIAA1549 | 0,072842263 | -0,461594427 | 0,534436689 | |
| 243957_at | LOC100128108 | 0,072842263 | -0,461594427 | 0,534436689 | |
| 219810_at | VCPIP1 | 0,613492853 | 0,079428135 | 0,534064718 | |
| 227195_at | ZNF503 | 0,613492853 | 0,079428135 | 0,534064718 | |
| 239598_s_at | LPCAT2 | 0,613492853 | 0,079428135 | 0,534064718 | |
| 234984_at | NEDD1 | 2,972842302 | 2,438805127 | 0,534037175 | |
| 1559909_a_at | TIMM17B | -0,196756757 | -0,730778808 | 0,534022051 | |
| 218851_s_at | SFT2D3 /// WDR | -0,196756757 | -0,730778808 | 0,534022051 | |
| 224001_at | FAM135B | -0,196756757 | -0,730778808 | 0,534022051 | |

| | | | | | |
|---|---|---|---|---|---|
| 225542_at | ACAP3 | -0,196756757 | -0,730778808 | 0,534022051 | |
| 232516_x_at | DAP3 | -0,196756757 | -0,730778808 | 0,534022051 | |
| 233148_at | PODN | -0,196756757 | -0,730778808 | 0,534022051 | |
| 235880_at | SRRM3 | -0,196756757 | -0,730778808 | 0,534022051 | |
| 205680_at | MMP10 | -0,112808516 | -0,646746079 | 0,533937562 | |
| 211738_x_at | CELA3A | -0,112808516 | -0,646746079 | 0,533937562 | |
| 234481_at | - | -0,112808516 | -0,646746079 | 0,533937562 | |
| 239266_at | - | -0,112808516 | -0,646746079 | 0,533937562 | |
| 244474_at | - | 0,541261317 | 0,007421914 | 0,533839403 | |
| 213020_at | GOSR1 | 2,26825514 | 1,73441697 | 0,53383817 | |
| 213530_at | RAB3GAP1 | 2,26825514 | 1,73441697 | 0,53383817 | |
| 235903_at | ANKS6 | 1,191926657 | 0,658444279 | 0,533482378 | |
| 229087_s_at | - | -0,033456564 | -0,566887459 | 0,533430895 | |
| 235237_at | VMA21 | -0,033456564 | -0,566887459 | 0,533430895 | |
| 239020_at | FUT10 | -0,033456564 | -0,566887459 | 0,533430895 | |
| 239645_at | - | -0,033456564 | -0,566887459 | 0,533430895 | |
| 221454_at | - | -0,285837228 | -0,819238336 | 0,533401108 | |
| 242167_at | - | -0,285837228 | -0,819238336 | 0,533401108 | |
| 204426_at | TMED2 | 3,927675977 | 3,394350331 | 0,533325646 | |
| 231918_s_at | GFM2 | 2,965960999 | 2,432926755 | 0,533034245 | |
| 241344_at | - | 1,923647937 | 1,390636111 | 0,533011826 | |
| 204204_at | SLC31A2 | 0,592200562 | 0,059218869 | 0,532981693 | |
| 206726_at | HPGDS | 0,592200562 | 0,059218869 | 0,532981693 | |
| 227298_at | FLJ37798 | 0,592200562 | 0,059218869 | 0,532981693 | |
| 1562286_at | - | -2,504600601 | -3,037500868 | 0,532900267 | |
| 207744_at | - | -2,504600601 | -3,037500868 | 0,532900267 | |
| 215468_at | LOC647070 | -2,504600601 | -3,037500868 | 0,532900267 | |
| 242274_x_at | SLC25A42 | -2,504600601 | -3,037500868 | 0,532900267 | |
| 242909_at | - | -2,504600601 | -3,037500868 | 0,532900267 | |
| 212612_at | RCOR1 | 4,823882603 | 4,291026333 | 0,53285627 | |
| 225086_at | FAM98B | 4,449221925 | 3,916534328 | 0,532687596 | |
| 211180_x_at | LOC100506403 / | 0,209623097 | -0,322870281 | 0,532493378 | |
| 212730_at | SYNM | 0,209623097 | -0,322870281 | 0,532493378 | |
| 1557141_at | FAM188B /// INM | -1,131062212 | -1,663414495 | 0,532352282 | |
| 206775_at | CUBN | -1,131062212 | -1,663414495 | 0,532352282 | |
| 207200_at | OTC | -1,131062212 | -1,663414495 | 0,532352282 | |
| 207964_x_at | IFNA4 | -1,131062212 | -1,663414495 | 0,532352282 | |
| 209779_at | LLPH | -1,131062212 | -1,663414495 | 0,532352282 | |
| 213284_at | ZFP36L1 | -1,131062212 | -1,663414495 | 0,532352282 | |
| 220572_at | LOC100506282 | -1,131062212 | -1,663414495 | 0,532352282 | |
| 227276_at | PLXDC2 | -1,131062212 | -1,663414495 | 0,532352282 | |
| 227556_at | NME7 | -1,131062212 | -1,663414495 | 0,532352282 | |
| 230719_at | ING3 | -1,131062212 | -1,663414495 | 0,532352282 | |
| 231218_at | - | -1,131062212 | -1,663414495 | 0,532352282 | |
| 232846_s_at | CDH23 /// LOC10 | -1,131062212 | -1,663414495 | 0,532352282 | |
| 232871_at | - | -1,131062212 | -1,663414495 | 0,532352282 | |
| 235246_at | - | -1,131062212 | -1,663414495 | 0,532352282 | |
| 238255_at | - | -1,131062212 | -1,663414495 | 0,532352282 | |
| 239610_at | - | -1,131062212 | -1,663414495 | 0,532352282 | |
| 240618_at | - | -1,131062212 | -1,663414495 | 0,532352282 | |
| 242243_at | TMF1 | -1,131062212 | -1,663414495 | 0,532352282 | |
| 210665_at | TFPI | 0,641402394 | 0,109221392 | 0,532181002 | |
| 236296_x_at | C8orf58 | 0,641402394 | 0,109221392 | 0,532181002 | |
| 218326_s_at | LGR4 | 2,352981171 | 1,820984782 | 0,531996389 | |
| 206644_at | NR0B1 | -0,622910612 | -1,154897679 | 0,531987066 | |
| 214188_at | - | -0,622910612 | -1,154897679 | 0,531987066 | |

| | | | | | |
|---|---|---|---|---|---|
| 1558666_at | PHEX-AS1 | -2,089474779 | -2,621331989 | 0,53185721 | |
| 1562537_at | FCER1A | -2,089474779 | -2,621331989 | 0,53185721 | |
| 1566491_at | - | -2,089474779 | -2,621331989 | 0,53185721 | |
| 213745_at | ATRNL1 | -2,089474779 | -2,621331989 | 0,53185721 | |
| 217057_s_at | GNAS | -2,089474779 | -2,621331989 | 0,53185721 | |
| 225072_at | ZCCHC3 | -2,089474779 | -2,621331989 | 0,53185721 | |
| 243086_at | - | -2,089474779 | -2,621331989 | 0,53185721 | |
| 202837_at | TRAFD1 | 2,16388051 | 1,632095957 | 0,531784553 | |
| 1554706_at | OR2L13 | -2,779459409 | -3,311072652 | 0,531613243 | |
| 1556354_s_at | RGL3 | -2,779459409 | -3,311072652 | 0,531613243 | |
| 207308_at | SLCO1A2 | -2,779459409 | -3,311072652 | 0,531613243 | |
| 207537_at | PFKFB1 | -0,148196756 | -0,679763839 | 0,531567083 | |
| 208325_s_at | AKAP13 | -0,148196756 | -0,679763839 | 0,531567083 | |
| 214380_at | PRPF31 | -0,148196756 | -0,679763839 | 0,531567083 | |
| 231151_at | DLGAP3 | 0,273439642 | -0,258114234 | 0,531553876 | |
| 225244_at | LOC100130093 / | 3,420201053 | 2,888726553 | 0,5314745 | |
| 216554_s_at | ENO1 | 3,262904562 | 2,731588328 | 0,531316234 | |
| 205122_at | MSANTD3-TMEF | 2,259277178 | 1,728030156 | 0,531247021 | |
| 211237_s_at | FGFR4 | -0,234278768 | -0,765504029 | 0,531225261 | |
| 214330_at | ATPAF2 | -0,234278768 | -0,765504029 | 0,531225261 | |
| 1555645_at | GAFA2 | 0,181384709 | -0,34958478 | 0,53096949 | |
| 235914_at | SYNPO | 0,181384709 | -0,34958478 | 0,53096949 | |
| 242307_at | ZNF789 | 0,735041319 | 0,204310755 | 0,530730564 | |
| 1562236_at | KAT6B | -2,359436556 | -2,89013181 | 0,530695254 | |
| 222124_at | HIF3A | -2,359436556 | -2,89013181 | 0,530695254 | |
| 243767_at | - | -2,359436556 | -2,89013181 | 0,530695254 | |
| 231837_at | USP28 | 1,406361373 | 0,876248481 | 0,530112892 | |
| 201249_at | SLC2A1 | 0,246434561 | -0,283667828 | 0,530102388 | |
| 235515_at | SYNE4 | 0,246434561 | -0,283667828 | 0,530102388 | |
| 202879_s_at | CYTH1 | 0,779676066 | 0,249604176 | 0,53007189 | |
| 232371_at | 07.03.15 | -0,325749314 | -0,855751026 | 0,530001712 | |
| 233891_at | MUC3 | -0,325749314 | -0,855751026 | 0,530001712 | |
| 209606_at | CYTIP | 0,882651021 | 0,352836757 | 0,529814264 | |
| 235566_at | TMF1 | 0,882651021 | 0,352836757 | 0,529814264 | |
| 235280_at | POLR1A | 1,119359127 | 0,589657387 | 0,52970174 | |
| 224132_at | - | 0,822971223 | 0,293518836 | 0,529452388 | |
| 1553114_a_at | PTK6 | 0,30867765 | -0,220592223 | 0,529269873 | |
| 229660_at | C16orf55 | 0,30867765 | -0,220592223 | 0,529269873 | |
| 202131_s_at | RIOK3 | 2,967339888 | 2,438805127 | 0,528534762 | |
| 201486_at | RCN2 | 4,182805865 | 3,654731437 | 0,528074428 | |
| 1552788_a_at | HELB | -0,423248115 | -0,950686014 | 0,527437899 | |
| 1557263_s_at | - | -0,423248115 | -0,950686014 | 0,527437899 | |
| 215455_at | TIMELESS | -0,423248115 | -0,950686014 | 0,527437899 | |
| 244305_at | GGN | -0,423248115 | -0,950686014 | 0,527437899 | |
| 228842_at | - | 0,984222186 | 0,456918297 | 0,527303888 | |
| 209151_x_at | TCF3 | 0,480753764 | -0,046302147 | 0,52705591 | |
| 229037_at | TRIM69 | 0,480753764 | -0,046302147 | 0,52705591 | |
| 210233_at | IL1RAP | -0,85085127 | -1,377419394 | 0,526568124 | |
| 214324_at | GP2 | -0,85085127 | -1,377419394 | 0,526568124 | |
| 221179_at | - | -0,85085127 | -1,377419394 | 0,526568124 | |
| 222272_x_at | SCIN | -0,85085127 | -1,377419394 | 0,526568124 | |
| 229260_at | C5orf15 | -0,85085127 | -1,377419394 | 0,526568124 | |
| 230836_at | ST8SIA4 | -0,85085127 | -1,377419394 | 0,526568124 | |
| 231038_s_at | - | -0,85085127 | -1,377419394 | 0,526568124 | |
| 233806_at | - | -0,85085127 | -1,377419394 | 0,526568124 | |
| 238062_at | GPIHBP1 | -0,85085127 | -1,377419394 | 0,526568124 | |

| | | | | | |
|---|---|---|---|---|---|
| 1561085_at | LOC153910 | -1,089016378 | -1,615132408 | 0,52611603 | |
| 203184_at | FBN2 | -1,089016378 | -1,615132408 | 0,52611603 | |
| 207383_s_at | RHBDL1 | -1,089016378 | -1,615132408 | 0,52611603 | |
| 221098_x_at | UTP14A | -1,089016378 | -1,615132408 | 0,52611603 | |
| 224909_s_at | PREX1 | -1,089016378 | -1,615132408 | 0,52611603 | |
| 228413_s_at | SFRP1 | -1,089016378 | -1,615132408 | 0,52611603 | |
| 229917_at | AGAP2 | -1,089016378 | -1,615132408 | 0,52611603 | |
| 239136_at | LOC728978 | -1,089016378 | -1,615132408 | 0,52611603 | |
| 239571_at | - | -1,089016378 | -1,615132408 | 0,52611603 | |
| 244467_at | SHISA8 | -1,089016378 | -1,615132408 | 0,52611603 | |
| 224111_x_at | KLF16 | 1,331241794 | 0,805216064 | 0,52602573 | |
| 1553264_a_at | SYN1 | 0,702292136 | 0,176436073 | 0,525856063 | |
| 243176_at | ARL5A | 1,360921491 | 0,835239052 | 0,525682439 | |
| 244743_x_at | ZNF138 | 2,069451339 | 1,543943227 | 0,525508112 | |
| 204091_at | PDE6D | 3,304956562 | 2,780243895 | 0,524712667 | |
| 228833_s_at | - | -0,089689931 | -0,614371577 | 0,524681645 | |
| 239175_at | - | -0,089689931 | -0,614371577 | 0,524681645 | |
| 1557456_a_at | - | -2,712026959 | -3,236070201 | 0,524043241 | |
| 206418_at | NOX1 | -2,712026959 | -3,236070201 | 0,524043241 | |
| 237668_at | LOC100507201 | -2,712026959 | -3,236070201 | 0,524043241 | |
| 231871_at | GPR180 | 1,215326747 | 0,691645685 | 0,523681062 | |
| 240623_at | - | 0,133056859 | -0,39061235 | 0,523669209 | |
| 1554376_s_at | PTPLA | -1,688127507 | -2,211750327 | 0,523622821 | |
| 1560274_at | WTAP | -1,688127507 | -2,211750327 | 0,523622821 | |
| 1569824_at | - | -1,688127507 | -2,211750327 | 0,523622821 | |
| 217570_x_at | - | -1,688127507 | -2,211750327 | 0,523622821 | |
| 220403_s_at | TP53AIP1 | -1,688127507 | -2,211750327 | 0,523622821 | |
| 203552_at | MAP4K5 | 2,936695663 | 2,413157372 | 0,523538291 | |
| 1554262_s_at | KLHL29 | -1,763254337 | -2,286583451 | 0,523329114 | |
| 1561485_at | IQCA1 | -1,763254337 | -2,286583451 | 0,523329114 | |
| 220676_at | ADAMTS8 | -1,763254337 | -2,286583451 | 0,523329114 | |
| 222298_at | - | -1,763254337 | -2,286583451 | 0,523329114 | |
| 226427_s_at | B3GALT6 | -1,763254337 | -2,286583451 | 0,523329114 | |
| 230094_at | - | -1,763254337 | -2,286583451 | 0,523329114 | |
| 230501_at | NDUFA10 | -1,763254337 | -2,286583451 | 0,523329114 | |
| 233269_at | - | -1,763254337 | -2,286583451 | 0,523329114 | |
| 234593_at | - | -1,763254337 | -2,286583451 | 0,523329114 | |
| 239425_at | DCUN1D5 | -1,763254337 | -2,286583451 | 0,523329114 | |
| 239553_at | LOC729420 | -1,763254337 | -2,286583451 | 0,523329114 | |
| 201766_at | ELAC2 | 0,20027154 | -0,322870281 | 0,523141821 | |
| 1561665_at | - | -1,639579028 | -2,162639771 | 0,523060743 | |
| 219954_s_at | GBA3 | -1,639579028 | -2,162639771 | 0,523060743 | |
| 228789_at | MTMR6 | -1,639579028 | -2,162639771 | 0,523060743 | |
| 229915_at | FAM26F | -1,639579028 | -2,162639771 | 0,523060743 | |
| 232928_at | - | -1,639579028 | -2,162639771 | 0,523060743 | |
| 237572_at | UGT3A1 | -1,639579028 | -2,162639771 | 0,523060743 | |
| 240471_at | - | -1,639579028 | -2,162639771 | 0,523060743 | |
| 1553226_at | LINC00052 | -1,812418401 | -2,335104118 | 0,522685717 | |
| 1554776_at | ZFP42 | -1,812418401 | -2,335104118 | 0,522685717 | |
| 1557404_at | - | -1,812418401 | -2,335104118 | 0,522685717 | |
| 1563822_at | LOC100131763 | -1,812418401 | -2,335104118 | 0,522685717 | |
| 1569962_at | - | -1,812418401 | -2,335104118 | 0,522685717 | |
| 206386_at | SERPINA7 | -1,812418401 | -2,335104118 | 0,522685717 | |
| 214285_at | FABP3 | -1,812418401 | -2,335104118 | 0,522685717 | |
| 222181_at | CNOT2 | -1,812418401 | -2,335104118 | 0,522685717 | |
| 226137_at | ZFHX3 | -1,812418401 | -2,335104118 | 0,522685717 | |

| | | | | | |
|---|---|---|---|---|---|
| 231105_at | - | -1,812418401 | -2,335104118 | 0,522685717 | |
| 231582_at | CDHR3 /// LOC1( | -1,812418401 | -2,335104118 | 0,522685717 | |
| 240638_at | - | -1,812418401 | -2,335104118 | 0,522685717 | |
| 224346_at | - | 2,396622324 | 1,874012195 | 0,522610129 | |
| 233543_s_at | FAM175A | 0,900083947 | 0,377530647 | 0,522553301 | |
| 235032_at | DNAJC21 | 2,458724341 | 1,936283387 | 0,522440954 | |
| 1556505_at | LINC00605 | -0,741668563 | -1,264005341 | 0,522336778 | |
| 1562496_at | LOC339539 | -0,741668563 | -1,264005341 | 0,522336778 | |
| 1566477_at | - | -0,741668563 | -1,264005341 | 0,522336778 | |
| 240672_at | WDR74 | -0,741668563 | -1,264005341 | 0,522336778 | |
| 243928_s_at | ABCC4 | -0,741668563 | -1,264005341 | 0,522336778 | |
| 1556914_at | - | -0,59053611 | -1,112851844 | 0,522315734 | |
| 1562972_at | LOC503519 | -0,59053611 | -1,112851844 | 0,522315734 | |
| 217488_x_at | PMS2P3 | -0,59053611 | -1,112851844 | 0,522315734 | |
| 220546_at | MLL | -0,59053611 | -1,112851844 | 0,522315734 | |
| 228338_at | C11orf93 | -0,59053611 | -1,112851844 | 0,522315734 | |
| 234598_at | - | -0,59053611 | -1,112851844 | 0,522315734 | |
| 235510_at | USHBP1 | -0,59053611 | -1,112851844 | 0,522315734 | |
| 238339_x_at | LRIG1 | -0,59053611 | -1,112851844 | 0,522315734 | |
| 205607_s_at | SCYL3 | 1,699726954 | 1,177496821 | 0,522230134 | |
| 230789_at | ZNF280B | 1,369290453 | 0,847075464 | 0,522214989 | |
| 1559997_x_at | SAMD14 | 0,03125738 | -0,490927335 | 0,522184715 | |
| 216542_x_at | IGHA1 /// IGHG1 | 0,03125738 | -0,490927335 | 0,522184715 | |
| 239754_at | C17orf76-AS1 /// | 0,03125738 | -0,490927335 | 0,522184715 | |
| 222243_s_at | TOB2 | 2,688444421 | 2,166264721 | 0,5221797 | |
| 1556166_x_at | - | -2,43257096 | -2,954576755 | 0,522005795 | |
| 1556916_a_at | - | -2,43257096 | -2,954576755 | 0,522005795 | |
| 1559249_at | ATXN1 | -2,43257096 | -2,954576755 | 0,522005795 | |
| 1566763_at | - | -2,43257096 | -2,954576755 | 0,522005795 | |
| 1567277_at | CTTN | -2,43257096 | -2,954576755 | 0,522005795 | |
| 1569230_at | - | -2,43257096 | -2,954576755 | 0,522005795 | |
| 205422_s_at | ITGBL1 | -2,43257096 | -2,954576755 | 0,522005795 | |
| 214422_at | RAD23B | -2,43257096 | -2,954576755 | 0,522005795 | |
| 220982_s_at | SPACA1 | -2,43257096 | -2,954576755 | 0,522005795 | |
| 232896_at | ERBB2IP | -2,43257096 | -2,954576755 | 0,522005795 | |
| 240995_at | - | -2,43257096 | -2,954576755 | 0,522005795 | |
| 1556299_s_at | C12orf76 | 0,103263601 | -0,41858459 | 0,521848191 | |
| 228777_at | KBTBD3 | 0,103263601 | -0,41858459 | 0,521848191 | |
| 233055_at | - | 0,103263601 | -0,41858459 | 0,521848191 | |
| 239856_at | - | 0,103263601 | -0,41858459 | 0,521848191 | |
| 244470_at | - | 0,103263601 | -0,41858459 | 0,521848191 | |
| 204271_s_at | EDNRB | 1,426552034 | 0,904843258 | 0,521708776 | |
| 226167_at | SYT7 | 0,384950573 | -0,136643983 | 0,521594556 | |
| 229287_at | PCNX | 0,384950573 | -0,136643983 | 0,521594556 | |
| 242900_at | ALG10B | 0,384950573 | -0,136643983 | 0,521594556 | |
| 206151_x_at | CELA3B | 0,171847695 | -0,34958478 | 0,521432476 | |
| 242217_s_at | FBRS | 0,171847695 | -0,34958478 | 0,521432476 | |
| 227761_at | MYO5A | 1,481627361 | 0,960386719 | 0,521240642 | |
| 236960_at | - | 0,44160593 | -0,079522948 | 0,521128878 | |
| 238604_at | - | 0,44160593 | -0,079522948 | 0,521128878 | |
| 219969_at | TXLNG | 1,089278259 | 0,568365095 | 0,520913164 | |
| 225949_at | LOC100653301 / | 0,496120222 | -0,024572586 | 0,520692808 | |
| 235113_at | LRR1 | 4,533553167 | 4,012941072 | 0,520612096 | |
| 1556800_a_at | - | -1,888254346 | -2,408705138 | 0,520450792 | |
| 1560895_at | LOC645188 | -1,888254346 | -2,408705138 | 0,520450792 | |
| 203697_at | FRZB | -1,888254346 | -2,408705138 | 0,520450792 | |

| | | | | | |
|---|---|---|---|---|---|
| 205528_s_at | RUNX1T1 | -1,888254346 | -2,408705138 | 0,520450792 | |
| 221439_at | RBBP9 | -1,888254346 | -2,408705138 | 0,520450792 | |
| 222361_at | TUBBP5 | -1,888254346 | -2,408705138 | 0,520450792 | |
| 223859_at | EPB41L4B | -1,888254346 | -2,408705138 | 0,520450792 | |
| 237677_at | - | -1,888254346 | -2,408705138 | 0,520450792 | |
| 241292_at | - | -1,888254346 | -2,408705138 | 0,520450792 | |
| 242164_s_at | LRIG2 | -1,888254346 | -2,408705138 | 0,520450792 | |
| 1560680_at | - | -0,299034815 | -0,819238336 | 0,520203521 | |
| 202901_x_at | CTSS | -0,299034815 | -0,819238336 | 0,520203521 | |
| 204841_s_at | EEA1 | -0,299034815 | -0,819238336 | 0,520203521 | |
| 220795_s_at | BEGAIN | -0,299034815 | -0,819238336 | 0,520203521 | |
| 203172_at | FXR2 | 2,478200152 | 1,958277216 | 0,519922936 | |
| 212847_at | - | 2,05655332 | 1,536652708 | 0,519900612 | |
| 227754_at | - | 1,835789711 | 1,315948728 | 0,519840983 | |
| 230362_at | INPP5F | 0,417596971 | -0,102100538 | 0,519697508 | |
| 1563641_a_at | SNX20 | -1,518072576 | -2,037551727 | 0,519479151 | |
| 1564595_at | - | -1,518072576 | -2,037551727 | 0,519479151 | |
| 201474_s_at | ITGA3 | -1,518072576 | -2,037551727 | 0,519479151 | |
| 204485_s_at | TOM1L1 | -1,518072576 | -2,037551727 | 0,519479151 | |
| 205890_s_at | GABBR1 /// UBD | -1,518072576 | -2,037551727 | 0,519479151 | |
| 211880_x_at | PCDHGA1 | -1,518072576 | -2,037551727 | 0,519479151 | |
| 214067_at | TSR3 | -1,518072576 | -2,037551727 | 0,519479151 | |
| 216657_at | ATXN3 | -1,518072576 | -2,037551727 | 0,519479151 | |
| 224026_at | - | -1,518072576 | -2,037551727 | 0,519479151 | |
| 230377_s_at | TBC1D2B | -1,518072576 | -2,037551727 | 0,519479151 | |
| 235565_at | ZNF425 | -1,518072576 | -2,037551727 | 0,519479151 | |
| 237153_at | - | -1,518072576 | -2,037551727 | 0,519479151 | |
| 241164_at | CHCHD3 | -1,518072576 | -2,037551727 | 0,519479151 | |
| 243215_at | - | -1,518072576 | -2,037551727 | 0,519479151 | |
| 244883_at | - | -1,518072576 | -2,037551727 | 0,519479151 | |
| 1552774_a_at | SLC25A27 | -1,937711681 | -2,456406426 | 0,518694745 | |
| 1553492_a_at | PAX1 | -1,937711681 | -2,456406426 | 0,518694745 | |
| 1554476_x_at | ZNF808 | -1,937711681 | -2,456406426 | 0,518694745 | |
| 1557179_s_at | CARS2 | -1,937711681 | -2,456406426 | 0,518694745 | |
| 1559571_a_at | ATP13A4 | -1,937711681 | -2,456406426 | 0,518694745 | |
| 1565599_at | - | -1,937711681 | -2,456406426 | 0,518694745 | |
| 1568690_a_at | LOC100631378 | -1,937711681 | -2,456406426 | 0,518694745 | |
| 217103_at | LDLR | -1,937711681 | -2,456406426 | 0,518694745 | |
| 219563_at | LINC00341 | -1,937711681 | -2,456406426 | 0,518694745 | |
| 222838_at | SLAMF7 | -1,937711681 | -2,456406426 | 0,518694745 | |
| 228672_at | - | -1,937711681 | -2,456406426 | 0,518694745 | |
| 234694_at | CNTROB | -1,937711681 | -2,456406426 | 0,518694745 | |
| 235562_at | C3orf70 | -1,937711681 | -2,456406426 | 0,518694745 | |
| 237485_at | SRSF3 | -1,937711681 | -2,456406426 | 0,518694745 | |
| 215076_s_at | COL3A1 | 0,57782909 | 0,059218869 | 0,518610222 | |
| 219113_x_at | HSD17B14 | 0,57782909 | 0,059218869 | 0,518610222 | |
| 238554_at | CYB5B | 0,57782909 | 0,059218869 | 0,518610222 | |
| 1560868_s_at | - | 0,911589958 | 0,393761504 | 0,517828454 | |
| 204432_at | SOX12 | 0,911589958 | 0,393761504 | 0,517828454 | |
| 244640_at | ZNF850 | 4,465414103 | 3,947788666 | 0,517625436 | |
| 1553697_at | CCSAP | -0,394749123 | -0,912331589 | 0,517582465 | |
| 1554198_at | SH3YL1 | -0,394749123 | -0,912331589 | 0,517582465 | |
| 217573_at | GRIN2C | -0,394749123 | -0,912331589 | 0,517582465 | |
| 218983_at | C1RL | -0,394749123 | -0,912331589 | 0,517582465 | |
| 230933_at | DSTN | -0,394749123 | -0,912331589 | 0,517582465 | |
| 235748_s_at | - | -0,394749123 | -0,912331589 | 0,517582465 | |

| | | | | | |
|---|---|---|---|---|---|
| 1555413_s_at | FBXL21 | -2,262747984 | -2,780313924 | 0,51756594 | |
| 1556804_s_at | POLR3B | -2,262747984 | -2,780313924 | 0,51756594 | |
| 1559664_at | - | -2,262747984 | -2,780313924 | 0,51756594 | |
| 1559975_at | BTG1 | -2,262747984 | -2,780313924 | 0,51756594 | |
| 1569167_at | - | -2,262747984 | -2,780313924 | 0,51756594 | |
| 208121_s_at | PTPRO | -2,262747984 | -2,780313924 | 0,51756594 | |
| 217438_at | - | -2,262747984 | -2,780313924 | 0,51756594 | |
| 220294_at | KCNV1 | -2,262747984 | -2,780313924 | 0,51756594 | |
| 229800_at | DCLK1 | -2,262747984 | -2,780313924 | 0,51756594 | |
| 232573_at | - | -2,262747984 | -2,780313924 | 0,51756594 | |
| 239272_at | MMP28 | -2,262747984 | -2,780313924 | 0,51756594 | |
| 241097_at | - | -2,262747984 | -2,780313924 | 0,51756594 | |
| 231812_x_at | PHAX | 2,114952142 | 1,59747851 | 0,517473632 | |
| 243146_at | - | -2,756478458 | -3,273861381 | 0,517382923 | |
| 202922_at | GCLC | 3,474545391 | 2,957221307 | 0,517324084 | |
| 215159_s_at | NADK | 1,352503698 | 0,835239052 | 0,517264646 | |
| 219151_s_at | RABL2A /// RABL | 1,381753566 | 0,864649967 | 0,517103599 | |
| 225901_at | PTPMT1 | 1,381753566 | 0,864649967 | 0,517103599 | |
| 1554885_a_at | LOC100653079 / | 2,107467633 | 1,590454215 | 0,517013418 | |
| 235717_at | ZNF229 | 0,853119696 | 0,336136178 | 0,516983518 | |
| 224836_at | TP53INP2 | 1,631786706 | 1,115234684 | 0,516552022 | |
| 1552897_a_at | KCNG3 | -2,479842156 | -2,996335703 | 0,516493547 | |
| 1558373_s_at | - | -2,479842156 | -2,996335703 | 0,516493547 | |
| 1562281_at | - | -2,479842156 | -2,996335703 | 0,516493547 | |
| 1563012_x_at | - | -2,479842156 | -2,996335703 | 0,516493547 | |
| 1563460_at | - | -2,479842156 | -2,996335703 | 0,516493547 | |
| 215563_s_at | MST1P9 | -2,479842156 | -2,996335703 | 0,516493547 | |
| 231091_x_at | - | -2,479842156 | -2,996335703 | 0,516493547 | |
| 226432_at | ETNK1 | 0,97328325 | 0,456918297 | 0,516364953 | |
| 204407_at | TTF2 | 3,268508253 | 2,752192409 | 0,516315844 | |
| 59705_at | SCLY | 0,223544425 | -0,292672097 | 0,516216523 | |
| 224433_s_at | DDX54 | 1,04816921 | 0,532164371 | 0,51600484 | |
| 202743_at | PIK3R3 | 1,926486359 | 1,410714371 | 0,515771988 | |
| 225501_at | PHF6 | 4,807809007 | 4,292109123 | 0,515699885 | |
| 1555253_at | COL25A1 | 1,119359127 | 0,603679647 | 0,515679481 | |
| 1555970_at | FBXO28 | 1,187200746 | 0,67181667 | 0,515384076 | |
| 226797_at | MBTD1 | 3,939667505 | 3,424287521 | 0,515379983 | |
| 1553267_a_at | CNOT6L | -1,026571149 | -1,541908042 | 0,515336893 | |
| 1560279_a_at | LOC221122 | -1,026571149 | -1,541908042 | 0,515336893 | |
| 1570135_at | ZNF230 | -1,026571149 | -1,541908042 | 0,515336893 | |
| 210875_s_at | ZEB1 | -1,026571149 | -1,541908042 | 0,515336893 | |
| 216376_x_at | - | -1,026571149 | -1,541908042 | 0,515336893 | |
| 220928_s_at | PRDM16 | -1,026571149 | -1,541908042 | 0,515336893 | |
| 228675_at | USP30-AS1 | -1,026571149 | -1,541908042 | 0,515336893 | |
| 234210_x_at | ACTR2 | -1,026571149 | -1,541908042 | 0,515336893 | |
| 242402_x_at | ARAP2 | -1,026571149 | -1,541908042 | 0,515336893 | |
| 243628_at | COPS7B | -1,026571149 | -1,541908042 | 0,515336893 | |
| 244452_at | ERLEC1 | -1,026571149 | -1,541908042 | 0,515336893 | |
| 1559052_s_at | PAK2 | 1,751865623 | 1,23718272 | 0,514682903 | |
| 205227_at | IL1RAP | 2,206540699 | 1,692388143 | 0,514152557 | |
| 1567213_at | PNN | 3,021441862 | 2,507538456 | 0,513903406 | |
| 1552953_a_at | NEUROD2 | 0,123193849 | -0,39061235 | 0,513806199 | |
| 1556727_at | PRCD | 0,123193849 | -0,39061235 | 0,513806199 | |
| 240530_at | - | 0,123193849 | -0,39061235 | 0,513806199 | |
| 214260_at | COPS8 | 0,052199481 | -0,461594427 | 0,513793907 | |
| 232115_at | SLC39A3 | 0,255492549 | -0,258114234 | 0,513606783 | |

| | | | | | |
|---|---|---|---|---|---|
| 1553193_at | ZNF441 | 0,376672223 | -0,136643983 | 0,513316206 | |
| 213536_s_at | - | 0,376672223 | -0,136643983 | 0,513316206 | |
| 1553301_a_at | TMEM182 | -0,706943342 | -1,220210588 | 0,513267246 | |
| 1558794_at | LOC728190 | -0,706943342 | -1,220210588 | 0,513267246 | |
| 1569537_at | - | -0,706943342 | -1,220210588 | 0,513267246 | |
| 216112_at | - | -0,706943342 | -1,220210588 | 0,513267246 | |
| 216801_at | - | -0,706943342 | -1,220210588 | 0,513267246 | |
| 219476_at | C1orf116 | -0,706943342 | -1,220210588 | 0,513267246 | |
| 238905_at | RHOJ | -0,706943342 | -1,220210588 | 0,513267246 | |
| 239825_at | - | -0,706943342 | -1,220210588 | 0,513267246 | |
| 244695_at | GHRLOS | -0,706943342 | -1,220210588 | 0,513267246 | |
| 226332_at | FAM133B /// FAM | 3,159334685 | 2,646096941 | 0,513237744 | |
| 214609_at | PHOX2A | -0,496991414 | -1,010192375 | 0,513200961 | |
| 216509_x_at | MLLT10 | -0,496991414 | -1,010192375 | 0,513200961 | |
| 220735_s_at | SENP7 | -0,496991414 | -1,010192375 | 0,513200961 | |
| 237154_at | - | -0,496991414 | -1,010192375 | 0,513200961 | |
| 208550_x_at | KCNG2 | -0,43775896 | -0,950686014 | 0,512927054 | |
| 235352_at | MR1 | -0,43775896 | -0,950686014 | 0,512927054 | |
| 204264_at | CPT2 | 2,348755185 | 1,835911187 | 0,512843998 | |
| 208090_s_at | AIRE | 0,592200562 | 0,079428135 | 0,512772426 | |
| 210399_x_at | FUT6 | 0,592200562 | 0,079428135 | 0,512772426 | |
| 226849_at | DENND1A | 0,592200562 | 0,079428135 | 0,512772426 | |
| 1560944_at | SLC25A3P1 | -1,399512875 | -1,912089812 | 0,512576937 | |
| 1567055_at | OR1C1 | -1,399512875 | -1,912089812 | 0,512576937 | |
| 206170_at | ADRB2 | -1,399512875 | -1,912089812 | 0,512576937 | |
| 208304_at | CCR3 | -1,399512875 | -1,912089812 | 0,512576937 | |
| 211111_at | HGC6.3 | -1,399512875 | -1,912089812 | 0,512576937 | |
| 216257_at | SERPINB13 | -1,399512875 | -1,912089812 | 0,512576937 | |
| 220766_at | BTG4 | -1,399512875 | -1,912089812 | 0,512576937 | |
| 231085_s_at | - | -1,399512875 | -1,912089812 | 0,512576937 | |
| 236162_at | LOC100287598 | -1,399512875 | -1,912089812 | 0,512576937 | |
| 237171_at | - | -1,399512875 | -1,912089812 | 0,512576937 | |
| 238698_at | CASK | -1,399512875 | -1,912089812 | 0,512576937 | |
| 239063_at | - | -1,399512875 | -1,912089812 | 0,512576937 | |
| 204429_s_at | SLC2A5 | 0,735041319 | 0,222599095 | 0,512442225 | |
| 1553810_a_at | KIAA1524 | 0,779676066 | 0,26733076 | 0,512345306 | |
| 201801_s_at | SLC29A1 | 1,666156729 | 1,153866761 | 0,512289967 | |
| 205379_at | CBR3 | 0,822971223 | 0,310717132 | 0,512254091 | |
| 226321_at | LYSMD3 | 2,915422315 | 2,403170156 | 0,512252159 | |
| 219287_at | KCNMB4 | 0,945567441 | 0,433557298 | 0,512010143 | |
| 1552771_a_at | LIN9 | -2,063149277 | -2,575129729 | 0,511980452 | |
| 1554185_at | LOC554206 | -2,063149277 | -2,575129729 | 0,511980452 | |
| 1556907_at | ZNF474 | -2,063149277 | -2,575129729 | 0,511980452 | |
| 1562320_at | NAV2-AS5 | -2,063149277 | -2,575129729 | 0,511980452 | |
| 1565680_at | DIP2C | -2,063149277 | -2,575129729 | 0,511980452 | |
| 210279_at | GPR18 | -2,063149277 | -2,575129729 | 0,511980452 | |
| 222290_at | OR2A20P /// OR2 | -2,063149277 | -2,575129729 | 0,511980452 | |
| 224724_at | SULF2 | -2,063149277 | -2,575129729 | 0,511980452 | |
| 225622_at | PAG1 | -2,063149277 | -2,575129729 | 0,511980452 | |
| 230137_at | TMEM155 | -2,063149277 | -2,575129729 | 0,511980452 | |
| 232166_at | KIAA1377 | -2,063149277 | -2,575129729 | 0,511980452 | |
| 232425_at | SNX25 | -2,063149277 | -2,575129729 | 0,511980452 | |
| 234543_at | - | -2,063149277 | -2,575129729 | 0,511980452 | |
| 236921_at | - | -2,063149277 | -2,575129729 | 0,511980452 | |
| 240188_at | - | -2,063149277 | -2,575129729 | 0,511980452 | |
| 242768_at | - | -2,063149277 | -2,575129729 | 0,511980452 | |

| | | | | | |
|---|---|---|---|---|---|
| 225011_at | PRKAR2A | 5,026305189 | 4,514364405 | 0,511940784 | |
| 201905_s_at | CTDSPL | 0,228146222 | -0,283667828 | 0,511814049 | |
| 203373_at | SOCS2 | 0,228146222 | -0,283667828 | 0,511814049 | |
| 1558102_at | - | 0,291166226 | -0,220592223 | 0,511758449 | |
| 205424_at | TBKBP1 | 0,291166226 | -0,220592223 | 0,511758449 | |
| 215240_at | ITGB3 | 0,291166226 | -0,220592223 | 0,511758449 | |
| 238573_at | - | 0,291166226 | -0,220592223 | 0,511758449 | |
| 205214_at | STK17B | 2,531373922 | 2,019703483 | 0,511670439 | |
| 215677_s_at | BRF1 | 0,020670649 | -0,490927335 | 0,511597985 | |
| 219802_at | PYROXD1 | 0,020670649 | -0,490927335 | 0,511597985 | |
| 239844_x_at | C1orf228 | 0,020670649 | -0,490927335 | 0,511597985 | |
| 1569532_a_at | LCN15 | 0,46522187 | -0,046302147 | 0,511524017 | |
| 229754_at | LOC100507297 | 0,46522187 | -0,046302147 | 0,511524017 | |
| 1559210_at | - | 0,518867317 | 0,007421914 | 0,511445403 | |
| 235663_at | - | 0,518867317 | 0,007421914 | 0,511445403 | |
| 216475_at | - | 0,57058931 | 0,059218869 | 0,511370442 | |
| 203002_at | AMOTL2 | -0,055687482 | -0,566887459 | 0,511199977 | |
| 212161_at | AP2A2 | -0,055687482 | -0,566887459 | 0,511199977 | |
| 231234_at | CTSC | -0,055687482 | -0,566887459 | 0,511199977 | |
| 234364_at | CKAP2 | -0,055687482 | -0,566887459 | 0,511199977 | |
| 236246_x_at | LOC653160 | -0,055687482 | -0,566887459 | 0,511199977 | |
| 239197_s_at | EZH1 | -0,055687482 | -0,566887459 | 0,511199977 | |
| 221162_at | HHLA1 | 0,715481152 | 0,204310755 | 0,511170396 | |
| 228341_at | NUDT16 | 0,760715727 | 0,249604176 | 0,511111551 | |
| 230388_s_at | KANSL1-AS1 | 0,760715727 | 0,249604176 | 0,511111551 | |
| 222771_s_at | MYEF2 | 0,888485433 | 0,377530647 | 0,510954787 | |
| 203077_s_at | SMAD2 | 2,842156496 | 2,331254065 | 0,510902431 | |
| 1561927_at | ANKUB1 | -2,667025278 | -3,177802286 | 0,510777009 | |
| 209470_s_at | GPM6A | -2,667025278 | -3,177802286 | 0,510777009 | |
| 205494_at | ZNF821 | -0,136301282 | -0,646746079 | 0,510444796 | |
| 219736_at | TRIM36 | -0,136301282 | -0,646746079 | 0,510444796 | |
| 240846_at | PCBD2 | -0,136301282 | -0,646746079 | 0,510444796 | |
| 202371_at | TCEAL4 | 4,268075451 | 3,757867094 | 0,510208357 | |
| 203711_s_at | HIBCH | 2,983784569 | 2,473581079 | 0,51020349 | |
| 234995_at | SPICE1 | 1,859746653 | 1,349621332 | 0,510125321 | |
| 205310_at | FBXO46 | 3,265148651 | 2,755336312 | 0,509812339 | |
| 238623_at | - | 1,296557217 | 0,786897721 | 0,509659496 | |
| 1555056_at | CCNG2 | -0,221656139 | -0,730778808 | 0,509122669 | |
| 1557569_at | MPDU1 | -0,221656139 | -0,730778808 | 0,509122669 | |
| 221061_at | PKD2L1 | -0,221656139 | -0,730778808 | 0,509122669 | |
| 232647_at | PROCA1 | -0,221656139 | -0,730778808 | 0,509122669 | |
| 1556426_at | HEXA | -1,353583928 | -1,862647763 | 0,509063835 | |
| 208017_s_at | MCF2 | -1,353583928 | -1,862647763 | 0,509063835 | |
| 211718_at | MGC2889 | -1,353583928 | -1,862647763 | 0,509063835 | |
| 216568_x_at | - | -1,353583928 | -1,862647763 | 0,509063835 | |
| 217018_at | - | -1,353583928 | -1,862647763 | 0,509063835 | |
| 219820_at | SLC6A16 | -1,353583928 | -1,862647763 | 0,509063835 | |
| 220249_at | HYAL4 | -1,353583928 | -1,862647763 | 0,509063835 | |
| 221066_at | RXFP3 | -1,353583928 | -1,862647763 | 0,509063835 | |
| 230255_at | GABRD | -1,353583928 | -1,862647763 | 0,509063835 | |
| 233024_at | - | -1,353583928 | -1,862647763 | 0,509063835 | |
| 236182_at | FAM185A | -1,353583928 | -1,862647763 | 0,509063835 | |
| 238298_at | LOC100507058 | -1,353583928 | -1,862647763 | 0,509063835 | |
| 239791_at | HOXB-AS3 | -1,353583928 | -1,862647763 | 0,509063835 | |
| 219074_at | TMEM184C | 3,248232277 | 2,739547836 | 0,508684441 | |
| 220295_x_at | DEPDC1 | 1,81142822 | 1,303116084 | 0,508312135 | |

| | | | | | |
|---|---|---|---|---|---|
| 1560617_at | LOC100128198 | -0,986356909 | -1,494668682 | 0,508311773 | |
| 1564028_s_at | FAM115C | -0,986356909 | -1,494668682 | 0,508311773 | |
| 1569253_at | INTS4 | -0,986356909 | -1,494668682 | 0,508311773 | |
| 1569269_s_at | SRGAP1 | -0,986356909 | -1,494668682 | 0,508311773 | |
| 214262_at | TRIP6 | -0,986356909 | -1,494668682 | 0,508311773 | |
| 217661_x_at | SIX5 | -0,986356909 | -1,494668682 | 0,508311773 | |
| 223938_at | TEX35 | -0,986356909 | -1,494668682 | 0,508311773 | |
| 230163_at | GFRA1 | -0,986356909 | -1,494668682 | 0,508311773 | |
| 231554_at | TPD52L3 | -0,986356909 | -1,494668682 | 0,508311773 | |
| 237880_at | LOC100506457 | -0,986356909 | -1,494668682 | 0,508311773 | |
| 239191_at | - | -0,986356909 | -1,494668682 | 0,508311773 | |
| 240374_at | - | -0,986356909 | -1,494668682 | 0,508311773 | |
| 243565_at | CCDC150 | -0,986356909 | -1,494668682 | 0,508311773 | |
| 214620_x_at | PAM | 2,59162628 | 2,083632167 | 0,507994113 | |
| 225497_at | ATE1 | 2,936695663 | 2,428994491 | 0,507701172 | |
| 202742_s_at | PRKACB | 2,122398022 | 1,614891062 | 0,507506961 | |
| 202790_at | CLDN7 | -0,172274514 | -0,679763839 | 0,507489326 | |
| 227989_at | LTBP4 | -0,172274514 | -0,679763839 | 0,507489326 | |
| 232837_at | KIF13A | -0,172274514 | -0,679763839 | 0,507489326 | |
| 213034_at | SIK3 | 2,654607516 | 2,147243253 | 0,507364264 | |
| 224337_s_at | FZD4 | -0,543051993 | -1,050406615 | 0,507354622 | |
| 231050_at | HRASLS5 | -0,543051993 | -1,050406615 | 0,507354622 | |
| 202806_at | DBN1 | 3,251631457 | 2,744302548 | 0,507328909 | |
| 233558_s_at | TRAPPC11 | 2,348755185 | 1,841838775 | 0,506916411 | |
| 213046_at | PABPN1 | 2,400710591 | 1,894118818 | 0,506591773 | |
| 228851_s_at | ENSA | 3,281868968 | 2,775606361 | 0,506262608 | |
| 226160_at | H6PD | 0,859074518 | 0,352836757 | 0,506237761 | |
| 209884_s_at | SLC4A7 | 2,525769574 | 2,019703483 | 0,506066092 | |
| 232506_s_at | C15orf41 | 0,728550685 | 0,222599095 | 0,505951591 | |
| 232493_at | - | 0,682279745 | 0,176436073 | 0,505843672 | |
| 1556992_at | LOC550112 /// LO | -0,259832361 | -0,765504029 | 0,505671668 | |
| 1564002_a_at | AKD1 | -0,259832361 | -0,765504029 | 0,505671668 | |
| 207670_at | KRT85 | -0,259832361 | -0,765504029 | 0,505671668 | |
| 210056_at | RND1 | -0,259832361 | -0,765504029 | 0,505671668 | |
| 222005_s_at | GNG3 | -0,259832361 | -0,765504029 | 0,505671668 | |
| 231379_at | LOC100507560 | -0,259832361 | -0,765504029 | 0,505671668 | |
| 231955_s_at | HIBADH | 1,073999308 | 0,568365095 | 0,505634213 | |
| 235273_at | DYX1C1 | 1,073999308 | 0,568365095 | 0,505634213 | |
| 225605_at | TP53I13 | 1,000476627 | 0,495031851 | 0,505444777 | |
| 1556684_at | RPPH1 | 0,962260738 | 0,456918297 | 0,505342441 | |
| 211557_x_at | SLCO2B1 | 0,480753764 | -0,024572586 | 0,50532635 | |
| 236634_at | C8orf48 | 0,480753764 | -0,024572586 | 0,50532635 | |
| 1552394_a_at | ENTHD1 | -2,384869671 | -2,89013181 | 0,505262139 | |
| 1554108_at | - | -2,384869671 | -2,89013181 | 0,505262139 | |
| 1563673_a_at | ALS2CR11 | -2,384869671 | -2,89013181 | 0,505262139 | |
| 1569773_at | ATP8A1 | -2,384869671 | -2,89013181 | 0,505262139 | |
| 1570125_at | - | -2,384869671 | -2,89013181 | 0,505262139 | |
| 1570345_at | LINC00474 | -2,384869671 | -2,89013181 | 0,505262139 | |
| 206282_at | NEUROD1 | -2,384869671 | -2,89013181 | 0,505262139 | |
| 234401_at | - | -2,384869671 | -2,89013181 | 0,505262139 | |
| 235874_at | PRSS35 | -2,384869671 | -2,89013181 | 0,505262139 | |
| 237548_at | - | -2,384869671 | -2,89013181 | 0,505262139 | |
| 235983_at | - | 0,425644434 | -0,079522948 | 0,505167382 | |
| 211596_s_at | LRIG1 | 1,764611101 | 1,259496994 | 0,505114106 | |
| 234192_s_at | GKAP1 | 1,163336153 | 0,658444279 | 0,504891874 | |
| 210355_at | PTHLH | 1,883312263 | 1,378453643 | 0,50485862 | |

| | | | | | |
|---|---|---|---|---|---|
| 37117_at | ARHGAP8 /// PR | -1,183481721 | -1,688334834 | 0,504853113 | |
| 1560151_x_at | SLC29A2 | 0,30867765 | -0,19610998 | 0,50478763 | |
| 215978_x_at | ZNF721 | 3,899061119 | 3,394350331 | 0,504710789 | |
| 214658_at | TMED7 /// TMED | 3,723323886 | 3,218713651 | 0,504610235 | |
| 1555862_s_at | MICALL2 | 0,246434561 | -0,258114234 | 0,504548795 | |
| 1560639_at | - | 0,246434561 | -0,258114234 | 0,504548795 | |
| 203281_s_at | UBA7 | 0,246434561 | -0,258114234 | 0,504548795 | |
| 228800_x_at | AURKAIP1 | 0,246434561 | -0,258114234 | 0,504548795 | |
| 243591_at | - | 0,246434561 | -0,258114234 | 0,504548795 | |
| 243950_at | - | 0,246434561 | -0,258114234 | 0,504548795 | |
| 203898_at | CRCP | 1,954567604 | 1,450051551 | 0,504516053 | |
| 210812_at | XRCC4 | -0,759588698 | -1,264005341 | 0,504416643 | |
| 216222_s_at | MYO10 | -0,759588698 | -1,264005341 | 0,504416643 | |
| 234301_s_at | TFB1M | -0,759588698 | -1,264005341 | 0,504416643 | |
| 236813_at | MORN4 | -0,759588698 | -1,264005341 | 0,504416643 | |
| 242338_at | TMEM64 | 3,51093129 | 3,006869115 | 0,504062175 | |
| 229371_at | - | 2,614687931 | 2,110887591 | 0,50380034 | |
| 238800_s_at | ZCCHC6 | 0,401366113 | -0,102100538 | 0,503466651 | |
| 1555038_at | EPB41L4A | -2,187914861 | -2,690860744 | 0,502945883 | |
| 215664_s_at | EPHA5 | -2,187914861 | -2,690860744 | 0,502945883 | |
| 234280_at | REG3A | -2,187914861 | -2,690860744 | 0,502945883 | |
| 236973_at | LOC100131662 | -2,187914861 | -2,690860744 | 0,502945883 | |
| 242220_at | SPTBN1 | -2,187914861 | -2,690860744 | 0,502945883 | |
| 1552761_at | SLC16A11 | -0,352951799 | -0,855751026 | 0,502799228 | |
| 1560410_at | - | -0,352951799 | -0,855751026 | 0,502799228 | |
| 207066_at | HRC | -0,352951799 | -0,855751026 | 0,502799228 | |
| 210286_s_at | SLC4A7 | -0,352951799 | -0,855751026 | 0,502799228 | |
| 217642_at | RNF40 | -0,352951799 | -0,855751026 | 0,502799228 | |
| 229852_at | NMNAT1 | -0,352951799 | -0,855751026 | 0,502799228 | |
| 232481_s_at | SLITRK6 | -0,352951799 | -0,855751026 | 0,502799228 | |
| 1555980_a_at | LOC100130417 | -0,033456564 | -0,536087151 | 0,502630587 | |
| 1559723_s_at | - | -0,033456564 | -0,536087151 | 0,502630587 | |
| 216451_at | STK38 | -0,033456564 | -0,536087151 | 0,502630587 | |
| 235495_at | CCDC97 | -0,033456564 | -0,536087151 | 0,502630587 | |
| 228961_at | MIER3 | 2,573632254 | 2,071071048 | 0,502561206 | |
| 1553327_a_at | BEND7 | -1,2845686 | -1,787089803 | 0,502521203 | |
| 1555740_a_at | MRAP | -1,2845686 | -1,787089803 | 0,502521203 | |
| 1556148_s_at | - | -1,2845686 | -1,787089803 | 0,502521203 | |
| 1556477_a_at | LOC283485 | -1,2845686 | -1,787089803 | 0,502521203 | |
| 1564709_at | LOC286238 | -1,2845686 | -1,787089803 | 0,502521203 | |
| 219670_at | BEND5 | -1,2845686 | -1,787089803 | 0,502521203 | |
| 223877_at | C1QTNF7 | -1,2845686 | -1,787089803 | 0,502521203 | |
| 238479_at | DCUN1D5 | -1,2845686 | -1,787089803 | 0,502521203 | |
| 240503_at | - | -1,2845686 | -1,787089803 | 0,502521203 | |
| 241625_at | - | -1,2845686 | -1,787089803 | 0,502521203 | |
| 241882_at | CAMTA1 | -1,2845686 | -1,787089803 | 0,502521203 | |
| 225984_at | PRKAA1 | 2,600539826 | 2,098562556 | 0,501977269 | |
| 211559_s_at | CCNG2 | 1,434549838 | 0,932882274 | 0,501667564 | |
| 1564803_at | KRTAP11-1 | 0,083054335 | -0,41858459 | 0,501638924 | |
| 1553168_at | GRIK5 | -0,112808516 | -0,614371577 | 0,50156306 | |
| 244149_at | - | -0,112808516 | -0,614371577 | 0,50156306 | |
| 217503_at | STK17B | 1,556829052 | 1,055274826 | 0,501554226 | |
| 1555153_s_at | FCHO2 | -2,597496523 | -3,098858316 | 0,501361793 | |
| 243481_at | RHOJ | -2,597496523 | -3,098858316 | 0,501361793 | |
| 229609_at | LOC728190 | -0,83191556 | -1,332017329 | 0,500101769 | |
| 221871_s_at | TFG | 0,95671774 | 0,456918297 | 0,499799443 | |

| | | | | | |
|---|---|---|---|---|---|
| 227926_s_at | - | 2,201862497 | 1,702196344 | 0,499666152 | |
| 216609_at | TXN | 3,86543671 | 3,365837114 | 0,499599596 | |
| 225619_at | SLAIN1 | 2,779171778 | 2,27978304 | 0,499388738 | |
| 243691_at | - | 1,05337236 | 0,553993624 | 0,499378735 | |
| 217937_s_at | HDAC7 | 1,987559146 | 1,488344553 | 0,499214594 | |
| 210201_x_at | BIN1 | 0,67554678 | 0,176436073 | 0,499110707 | |
| 1552714_at | CREG2 | -2,236765673 | -2,735862426 | 0,499096753 | |
| 1553876_at | SAMD3 | -2,236765673 | -2,735862426 | 0,499096753 | |
| 1562856_at | - | -2,236765673 | -2,735862426 | 0,499096753 | |
| 216777_at | - | -2,236765673 | -2,735862426 | 0,499096753 | |
| 231867_at | ODZ2 | -2,236765673 | -2,735862426 | 0,499096753 | |
| 238168_at | TM4SF1 | -2,236765673 | -2,735862426 | 0,499096753 | |
| 241042_at | - | -2,236765673 | -2,735862426 | 0,499096753 | |
| 241732_at | - | -2,236765673 | -2,735862426 | 0,499096753 | |
| 206310_at | SPINK2 | -0,655928373 | -1,154897679 | 0,498969306 | |
| 211550_at | EGFR | -0,655928373 | -1,154897679 | 0,498969306 | |
| 217643_x_at | - | -0,655928373 | -1,154897679 | 0,498969306 | |
| 227403_at | PIGX | -0,655928373 | -1,154897679 | 0,498969306 | |
| 231290_at | RCCD1 | -0,655928373 | -1,154897679 | 0,498969306 | |
| 238114_at | PCMTD1 | -0,655928373 | -1,154897679 | 0,498969306 | |
| 225385_s_at | HNRPLL | 2,510717331 | 2,011838132 | 0,498879199 | |
| 1554780_a_at | PHTF2 | 0,627515113 | 0,128747141 | 0,498767972 | |
| 1559765_a_at | LOC286254 | 0,627515113 | 0,128747141 | 0,498767972 | |
| 210874_s_at | NAT6 | 0,627515113 | 0,128747141 | 0,498767972 | |
| 229719_s_at | DERL3 | -0,148196756 | -0,646746079 | 0,498549322 | |
| 237088_at | - | -0,148196756 | -0,646746079 | 0,498549322 | |
| 221381_s_at | MORF4L1 | 5,512036874 | 5,013671918 | 0,498364956 | |
| 209748_at | SPAST | 3,722507547 | 3,22439681 | 0,498110737 | |
| 227068_at | LOC100652805 / | 4,562580605 | 4,064545714 | 0,498034891 | |
| 229920_at | - | 0,702292136 | 0,204310755 | 0,497981381 | |
| 218911_at | YEATS4 | 2,16388051 | 1,665902186 | 0,497978324 | |
| 1562906_at | FAM170A | -0,512251684 | -1,010192375 | 0,497940691 | |
| 1564012_at | LOC100132354 | -0,512251684 | -1,010192375 | 0,497940691 | |
| 216706_x_at | IGHG1 /// LOC64 | -0,512251684 | -1,010192375 | 0,497940691 | |
| 222911_s_at | CXorf36 | -0,512251684 | -1,010192375 | 0,497940691 | |
| 230305_at | - | -0,512251684 | -1,010192375 | 0,497940691 | |
| 236904_x_at | TECTA | -0,512251684 | -1,010192375 | 0,497940691 | |
| 237033_at | FAM159A | -0,512251684 | -1,010192375 | 0,497940691 | |
| 238482_at | KLF7 | -0,512251684 | -1,010192375 | 0,497940691 | |
| 240280_at | UFSP1 | -0,512251684 | -1,010192375 | 0,497940691 | |
| 1556983_a_at | - | -1,240169874 | -1,738067356 | 0,497897482 | |
| 1557453_at | - | -1,240169874 | -1,738067356 | 0,497897482 | |
| 1559136_s_at | IDS /// LOC10027 | -1,240169874 | -1,738067356 | 0,497897482 | |
| 1563115_at | - | -1,240169874 | -1,738067356 | 0,497897482 | |
| 210556_at | NFATC3 | -1,240169874 | -1,738067356 | 0,497897482 | |
| 231728_at | CAPS | -1,240169874 | -1,738067356 | 0,497897482 | |
| 231758_at | PRM3 | -1,240169874 | -1,738067356 | 0,497897482 | |
| 233829_at | C20orf118 | -1,240169874 | -1,738067356 | 0,497897482 | |
| 236861_at | - | -1,240169874 | -1,738067356 | 0,497897482 | |
| 240911_at | NOS1 | -1,240169874 | -1,738067356 | 0,497897482 | |
| 1553869_at | SESN3 | -1,71423189 | -2,211750327 | 0,497518438 | |
| 1562741_at | UBXN4 | -1,71423189 | -2,211750327 | 0,497518438 | |
| 202063_s_at | SEL1L | -1,71423189 | -2,211750327 | 0,497518438 | |
| 206749_at | CD1B | -1,71423189 | -2,211750327 | 0,497518438 | |
| 215828_at | - | -1,71423189 | -2,211750327 | 0,497518438 | |
| 220873_at | - | -1,71423189 | -2,211750327 | 0,497518438 | |

| | | | | | |
|---|---|---|---|---|---|
| 228133_s_at | MYH11 | -1,71423189 | -2,211750327 | 0,497518438 | |
| 229226_at | - | -1,71423189 | -2,211750327 | 0,497518438 | |
| 229809_at | POU6F1 | -1,71423189 | -2,211750327 | 0,497518438 | |
| 231265_at | COX7B2 | -1,71423189 | -2,211750327 | 0,497518438 | |
| 232630_at | APIP | -1,71423189 | -2,211750327 | 0,497518438 | |
| 235977_at | LONRF2 | -1,71423189 | -2,211750327 | 0,497518438 | |
| 237351_at | LOC100652994 / | -1,71423189 | -2,211750327 | 0,497518438 | |
| 238919_at | - | -1,71423189 | -2,211750327 | 0,497518438 | |
| 239087_at | ANKS4B | -1,71423189 | -2,211750327 | 0,497518438 | |
| 242209_at | ANKRD33 | -1,71423189 | -2,211750327 | 0,497518438 | |
| 233557_s_at | MON1B | 1,40228911 | 0,904843258 | 0,497445851 | |
| 210531_at | NR2C1 | 0,606430281 | 0,109221392 | 0,497208889 | |
| 238606_at | ZNF747 | 0,417596971 | -0,079522948 | 0,497119919 | |
| 217383_at | PGK1 | 1,233777334 | 0,73688026 | 0,496897073 | |
| 228050_at | UTP15 | 2,396622324 | 1,899812471 | 0,496809853 | |
| 239706_x_at | - | 0,555999837 | 0,059218869 | 0,496780968 | |
| 242116_x_at | - | 0,555999837 | 0,059218869 | 0,496780968 | |
| 226831_at | SLC25A46 | 3,512821151 | 3,016072743 | 0,496748408 | |
| 228773_at | LOC100506100 | 1,201332287 | 0,704715219 | 0,496617068 | |
| 206791_s_at | PDE4C | 1,390002893 | 0,893473268 | 0,496529626 | |
| 233896_s_at | PAPLN | -0,234278768 | -0,730778808 | 0,49650004 | |
| 235559_at | FAM188B /// INM | -0,234278768 | -0,730778808 | 0,49650004 | |
| 241680_at | - | -0,234278768 | -0,730778808 | 0,49650004 | |
| 243118_at | CEP57L1 | -0,234278768 | -0,730778808 | 0,49650004 | |
| 1564684_at | BTN2A2 | -0,926850548 | -1,423348341 | 0,496497793 | |
| 207646_s_at | CDY1 /// CDY1B | -0,926850548 | -1,423348341 | 0,496497793 | |
| 216081_at | LAMA4 | -0,926850548 | -1,423348341 | 0,496497793 | |
| 216324_at | - | -0,926850548 | -1,423348341 | 0,496497793 | |
| 216330_s_at | POU6F1 | -0,926850548 | -1,423348341 | 0,496497793 | |
| 232901_at | RARS2 | -0,926850548 | -1,423348341 | 0,496497793 | |
| 233156_at | RNASEH2B | -0,926850548 | -1,423348341 | 0,496497793 | |
| 200782_at | ANXA5 | 4,295231314 | 3,798818761 | 0,496412553 | |
| 212470_at | SPAG9 | 3,279650757 | 2,783327323 | 0,496323434 | |
| 1553651_at | C18orf54 | -1,838812296 | -2,335104118 | 0,496291822 | |
| 1557857_a_at | - | -1,838812296 | -2,335104118 | 0,496291822 | |
| 1558922_at | - | -1,838812296 | -2,335104118 | 0,496291822 | |
| 1559086_at | - | -1,838812296 | -2,335104118 | 0,496291822 | |
| 210920_x_at | - | -1,838812296 | -2,335104118 | 0,496291822 | |
| 213714_at | CACNB2 | -1,838812296 | -2,335104118 | 0,496291822 | |
| 222948_s_at | LOC100509751 / | -1,838812296 | -2,335104118 | 0,496291822 | |
| 224012_at | ANKRD20A1 /// A | -1,838812296 | -2,335104118 | 0,496291822 | |
| 229669_at | LOC100507263 | -1,838812296 | -2,335104118 | 0,496291822 | |
| 233596_at | - | -1,838812296 | -2,335104118 | 0,496291822 | |
| 233820_at | - | -1,838812296 | -2,335104118 | 0,496291822 | |
| 238733_at | - | -1,838812296 | -2,335104118 | 0,496291822 | |
| 242628_at | - | -1,838812296 | -2,335104118 | 0,496291822 | |
| 243703_x_at | - | -1,838812296 | -2,335104118 | 0,496291822 | |
| 244611_at | MED13 | -1,838812296 | -2,335104118 | 0,496291822 | |
| 222603_at | ERMP1 | 1,360921491 | 0,864649967 | 0,496271524 | |
| 1556201_at | RNASET2 | -1,591296941 | -2,086984744 | 0,495687802 | |
| 1565566_a_at | - | -1,591296941 | -2,086984744 | 0,495687802 | |
| 204570_at | COX7A1 | -1,591296941 | -2,086984744 | 0,495687802 | |
| 215630_at | - | -1,591296941 | -2,086984744 | 0,495687802 | |
| 230007_at | JMJD1C | -1,591296941 | -2,086984744 | 0,495687802 | |
| 234682_at | BTBD9 | -1,591296941 | -2,086984744 | 0,495687802 | |
| 241397_at | - | -1,591296941 | -2,086984744 | 0,495687802 | |

| | | | | | |
|---|---|---|---|---|---|
| 215570_s_at | ZNF780A /// ZNF | 1,187200746 | 0,691645685 | 0,495555061 | |
| 226479_at | KBTBD6 | 1,187200746 | 0,691645685 | 0,495555061 | |
| 239644_at | - | 1,187200746 | 0,691645685 | 0,495555061 | |
| 1566722_a_at | SVEP1 | -2,64318869 | -3,138626879 | 0,495438189 | |
| 211742_s_at | EVI2B | 1,153678675 | 0,658444279 | 0,495234396 | |
| 219603_s_at | ZNF226 | 1,649074067 | 1,153866761 | 0,495207305 | |
| 239891_x_at | RAB12 | 2,691784869 | 2,196649505 | 0,495135364 | |
| 213118_at | UHRF1BP1L | 1,709647566 | 1,214517879 | 0,495129687 | |
| 224658_x_at | PACS1 | 0,928678725 | 0,433557298 | 0,495121427 | |
| 205791_x_at | ZNF230 | 1,560488174 | 1,065442793 | 0,495045381 | |
| 238686_at | FBXO3 | 1,560488174 | 1,065442793 | 0,495045381 | |
| 1554349_at | XRCC6BP1 | 0,171847695 | -0,322870281 | 0,494717977 | |
| 204202_at | IQCE | 0,171847695 | -0,322870281 | 0,494717977 | |
| 217117_x_at | MUC3A | 0,334552598 | -0,160136748 | 0,494689347 | |
| 203735_x_at | PPFIBP1 | 2,438982012 | 1,944570415 | 0,494411597 | |
| 1554934_at | RCBTB1 | -2,779459409 | -3,273861381 | 0,494401973 | |
| 1566251_at | SH3GL1P1 | -2,779459409 | -3,273861381 | 0,494401973 | |
| 1559864_at | LCN6 | -1,047650486 | -1,541908042 | 0,494257556 | |
| 1562844_at | LOC339822 | -1,047650486 | -1,541908042 | 0,494257556 | |
| 206744_s_at | ZMYM5 | -1,047650486 | -1,541908042 | 0,494257556 | |
| 208220_x_at | AMELY | -1,047650486 | -1,541908042 | 0,494257556 | |
| 211118_x_at | ESR2 | -1,047650486 | -1,541908042 | 0,494257556 | |
| 215061_at | METTL10 | -1,047650486 | -1,541908042 | 0,494257556 | |
| 216339_s_at | TNXA /// TNXB | -1,047650486 | -1,541908042 | 0,494257556 | |
| 217111_at | AMACR | -1,047650486 | -1,541908042 | 0,494257556 | |
| 220430_at | FAM110D | -1,047650486 | -1,541908042 | 0,494257556 | |
| 227673_at | ZNRD1 | -1,047650486 | -1,541908042 | 0,494257556 | |
| 228295_at | WDR59 | -1,047650486 | -1,541908042 | 0,494257556 | |
| 240048_at | STRC | -1,047650486 | -1,541908042 | 0,494257556 | |
| 205686_s_at | CD86 | -1,543331864 | -2,037551727 | 0,494219862 | |
| 220699_s_at | KIF2A | -1,543331864 | -2,037551727 | 0,494219862 | |
| 222153_at | MYEF2 | -1,543331864 | -2,037551727 | 0,494219862 | |
| 231527_at | FLJ36840 | -1,543331864 | -2,037551727 | 0,494219862 | |
| 232256_s_at | LOC401321 | -1,543331864 | -2,037551727 | 0,494219862 | |
| 234293_x_at | - | -1,543331864 | -2,037551727 | 0,494219862 | |
| 242772_x_at | - | -1,543331864 | -2,037551727 | 0,494219862 | |
| 243989_at | - | -1,543331864 | -2,037551727 | 0,494219862 | |
| 244263_at | - | -1,543331864 | -2,037551727 | 0,494219862 | |
| 207127_s_at | HNRNPH3 | 4,725454911 | 4,231314909 | 0,494140002 | |
| 1554095_at | RBM33 | 2,410880861 | 1,916759838 | 0,494121023 | |
| 221826_at | ANGEL2 | 1,523472489 | 1,029536893 | 0,493935596 | |
| 209532_at | PLAA | 0,911589958 | 0,417770464 | 0,493819495 | |
| 217635_s_at | POLG | 0,911589958 | 0,417770464 | 0,493819495 | |
| 218167_at | AMZ2 | 3,692807389 | 3,199221717 | 0,493585672 | |
| 1554583_a_at | METTL20 | -0,325749314 | -0,819238336 | 0,493489022 | |
| 219545_at | KCTD14 /// NDUF | -0,325749314 | -0,819238336 | 0,493489022 | |
| 222676_at | BRI3 /// FLJ3006 | -0,325749314 | -0,819238336 | 0,493489022 | |
| 223718_at | ACRBP | -0,325749314 | -0,819238336 | 0,493489022 | |
| 228178_s_at | - | -0,325749314 | -0,819238336 | 0,493489022 | |
| 230731_x_at | ZDHHC8 | -0,325749314 | -0,819238336 | 0,493489022 | |
| 224946_s_at | CCDC115 | 3,105247586 | 2,611817827 | 0,493429759 | |
| 222751_at | HERPUD2 | 3,480351894 | 2,986947093 | 0,493404801 | |
| 1558237_x_at | - | 0,87091093 | 0,377530647 | 0,493380283 | |
| 219262_at | SUV39H2 | 0,87091093 | 0,377530647 | 0,493380283 | |
| 242521_at | LOC100505812 | 3,307136232 | 2,813804482 | 0,49333175 | |
| 1569361_a_at | LOC100129098 | 0,209623097 | -0,283667828 | 0,493290925 | |

| | | | | | |
|---|---|---|---|---|---|
| 204268_at | S100A2 | 0,209623097 | -0,283667828 | 0,493290925 | |
| 227364_at | - | 3,882346871 | 3,38929984 | 0,493047031 | |
| 217368_at | - | 0,82905153 | 0,336136178 | 0,492915353 | |
| 230226_s_at | KDM5A | 1,795990708 | 1,303116084 | 0,492874623 | |
| 204693_at | CDC42EP1 | 0,03125738 | -0,461594427 | 0,492851807 | |
| 232761_at | COX4I2 | 0,03125738 | -0,461594427 | 0,492851807 | |
| 1554915_a_at | PDE12 | 2,236584753 | 1,743944487 | 0,492640265 | |
| 217448_s_at | TOX4 | 1,493160564 | 1,000684521 | 0,492476043 | |
| 1556222_at | SEPT7L | -1,964176657 | -2,456406426 | 0,492229769 | |
| 232023_at | TMEM67 | -1,964176657 | -2,456406426 | 0,492229769 | |
| 236727_at | - | -1,964176657 | -2,456406426 | 0,492229769 | |
| 238833_at | LOC729088 | -1,964176657 | -2,456406426 | 0,492229769 | |
| 242980_at | COMMD10 | -1,964176657 | -2,456406426 | 0,492229769 | |
| 201407_s_at | PPP1CB | 3,976425635 | 3,484256382 | 0,492169253 | |
| 1557044_at | LOC100506930 | -2,504600601 | -2,996335703 | 0,491735102 | |
| 1561229_at | - | -2,504600601 | -2,996335703 | 0,491735102 | |
| 1568589_at | - | -2,504600601 | -2,996335703 | 0,491735102 | |
| 216740_at | - | -2,504600601 | -2,996335703 | 0,491735102 | |
| 222291_at | FAM149A | -2,504600601 | -2,996335703 | 0,491735102 | |
| 231586_at | SRG7 | -2,504600601 | -2,996335703 | 0,491735102 | |
| 237101_at | GHSR | -2,504600601 | -2,996335703 | 0,491735102 | |
| 237149_at | - | -2,504600601 | -2,996335703 | 0,491735102 | |
| 239673_at | - | -2,504600601 | -2,996335703 | 0,491735102 | |
| 207018_s_at | RAB27B | 3,350052045 | 2,858346012 | 0,491706032 | |
| 1553303_at | C16orf46 | 0,072842263 | -0,41858459 | 0,491426852 | |
| 205188_s_at | SMAD5 | 0,072842263 | -0,41858459 | 0,491426852 | |
| 213770_at | KSR1 | 0,072842263 | -0,41858459 | 0,491426852 | |
| 220778_x_at | SEMA6B | 0,072842263 | -0,41858459 | 0,491426852 | |
| 222304_x_at | LOC100509541 / | 0,072842263 | -0,41858459 | 0,491426852 | |
| 213373_s_at | CASP8 | 1,556829052 | 1,065442793 | 0,491386259 | |
| 214223_at | - | 0,695652136 | 0,204310755 | 0,491341381 | |
| 235640_at | - | 0,695652136 | 0,204310755 | 0,491341381 | |
| 226879_at | HVCN1 | 0,57058931 | 0,079428135 | 0,491161175 | |
| 202502_at | ACADM | 4,817780426 | 4,326864571 | 0,490915855 | |
| 235413_at | GGCX | 0,648296226 | 0,157549243 | 0,490746983 | |
| 1557235_at | - | -1,470833215 | -1,961547147 | 0,490713932 | |
| 1557544_at | CCDC147 | -1,470833215 | -1,961547147 | 0,490713932 | |
| 1560024_at | - | -1,470833215 | -1,961547147 | 0,490713932 | |
| 219380_x_at | POLH | -1,470833215 | -1,961547147 | 0,490713932 | |
| 233200_at | - | -1,470833215 | -1,961547147 | 0,490713932 | |
| 233340_at | SPINK13 | -1,470833215 | -1,961547147 | 0,490713932 | |
| 237857_at | - | -1,470833215 | -1,961547147 | 0,490713932 | |
| 240042_at | FIBCD1 | -1,470833215 | -1,961547147 | 0,490713932 | |
| 241857_at | - | -1,470833215 | -1,961547147 | 0,490713932 | |
| 243647_at | - | -1,470833215 | -1,961547147 | 0,490713932 | |
| 214791_at | SP140L | 2,390468117 | 1,899812471 | 0,490655646 | |
| 229832_x_at | SH3TC1 | 0,518867317 | 0,028364014 | 0,490503303 | |
| 235332_at | FAM22A /// FAM2 | 0,518867317 | 0,028364014 | 0,490503303 | |
| 213000_at | MORC3 | 4,122199215 | 3,631821253 | 0,490377962 | |
| 214057_at | MCL1 | 2,122398022 | 1,632095957 | 0,490302066 | |
| 230578_at | - | -0,00073712 | -0,490927335 | 0,490190215 | |
| 234381_at | - | -0,00073712 | -0,490927335 | 0,490190215 | |
| 237709_at | LOC100507513 | -0,00073712 | -0,490927335 | 0,490190215 | |
| 1560482_at | - | -2,01371626 | -2,503677622 | 0,489961362 | |
| 219049_at | CSGALNACT1 | -2,01371626 | -2,503677622 | 0,489961362 | |
| 220197_at | ATP6V0A4 | -2,01371626 | -2,503677622 | 0,489961362 | |

| | | | | | |
|---|---|---|---|---|---|
| 220906_at | - | -2,01371626 | -2,503677622 | 0,489961362 | |
| 233989_at | - | -2,01371626 | -2,503677622 | 0,489961362 | |
| 236539_at | PTPN22 | -2,01371626 | -2,503677622 | 0,489961362 | |
| 240268_at | LOC440117 | -2,01371626 | -2,503677622 | 0,489961362 | |
| 242162_at | WDR69 | -2,01371626 | -2,503677622 | 0,489961362 | |
| 1558421_a_at | C14orf180 | -0,622910612 | -1,112851844 | 0,489941232 | |
| 1568745_at | LOC646268 | -0,622910612 | -1,112851844 | 0,489941232 | |
| 1570507_at | SCAF11 | -0,622910612 | -1,112851844 | 0,489941232 | |
| 205969_at | AADAC | -0,622910612 | -1,112851844 | 0,489941232 | |
| 220516_at | ZSCAN2 | -0,622910612 | -1,112851844 | 0,489941232 | |
| 224143_at | TTTY8 /// TTTY8 | -0,622910612 | -1,112851844 | 0,489941232 | |
| 224990_at | C4orf34 | -0,622910612 | -1,112851844 | 0,489941232 | |
| 228873_at | COL22A1 | -0,622910612 | -1,112851844 | 0,489941232 | |
| 229924_s_at | - | -0,622910612 | -1,112851844 | 0,489941232 | |
| 230893_at | DNAJC21 | -0,622910612 | -1,112851844 | 0,489941232 | |
| 232208_at | ISLR2 | -0,622910612 | -1,112851844 | 0,489941232 | |
| 236754_at | PPP1R2 | -0,622910612 | -1,112851844 | 0,489941232 | |
| 1557296_at | FLJ12825 | -1,173767127 | -1,663414495 | 0,489647368 | |
| 1558144_at | MEG3 | -1,173767127 | -1,663414495 | 0,489647368 | |
| 1561185_at | TTTY7 | -1,173767127 | -1,663414495 | 0,489647368 | |
| 205001_s_at | DDX3Y | -1,173767127 | -1,663414495 | 0,489647368 | |
| 209460_at | ABAT | -1,173767127 | -1,663414495 | 0,489647368 | |
| 219140_s_at | RBP4 | -1,173767127 | -1,663414495 | 0,489647368 | |
| 224020_at | MGC4473 | -1,173767127 | -1,663414495 | 0,489647368 | |
| 229247_at | FBLN7 | -1,173767127 | -1,663414495 | 0,489647368 | |
| 229921_at | - | -1,173767127 | -1,663414495 | 0,489647368 | |
| 230056_at | BPTF | -1,173767127 | -1,663414495 | 0,489647368 | |
| 233538_s_at | CYBB | -1,173767127 | -1,663414495 | 0,489647368 | |
| 200673_at | LAPTM4A | 5,701259584 | 5,211772284 | 0,4894873 | |
| 238523_at | KLHL36 | 1,079110292 | 0,589657387 | 0,489452905 | |
| 1552263_at | MAPK1 | 2,310153708 | 1,820984782 | 0,489168926 | |
| 211410_x_at | KIR2DL2 /// KIR2 | -0,423248115 | -0,912331589 | 0,489083473 | |
| 227503_at | - | -0,423248115 | -0,912331589 | 0,489083473 | |
| 213231_at | DMWD | -0,366776884 | -0,855751026 | 0,488974142 | |
| 228653_at | SAMD5 | -0,366776884 | -0,855751026 | 0,488974142 | |
| 243214_at | ZBTB46 | -0,366776884 | -0,855751026 | 0,488974142 | |
| 204708_at | MAPK4 | -0,888496123 | -1,377419394 | 0,488923272 | |
| 205147_x_at | NCF4 | -0,888496123 | -1,377419394 | 0,488923272 | |
| 210915_x_at | TRBC1 | -0,888496123 | -1,377419394 | 0,488923272 | |
| 220859_at | - | -0,888496123 | -1,377419394 | 0,488923272 | |
| 224924_at | TTC7A | -0,888496123 | -1,377419394 | 0,488923272 | |
| 226418_at | ERGIC2 | -0,888496123 | -1,377419394 | 0,488923272 | |
| 227083_at | B3GALTL | -0,888496123 | -1,377419394 | 0,488923272 | |
| 229820_at | LOC440993 | -0,888496123 | -1,377419394 | 0,488923272 | |
| 229996_s_at | PCGF5 | -0,888496123 | -1,377419394 | 0,488923272 | |
| 223812_at | FAM178B | 0,882651021 | 0,393761504 | 0,488889517 | |
| 226099_at | ELL2 | 3,848325987 | 3,359653127 | 0,488672861 | |
| 226337_at | GORAB | 1,666156729 | 1,177496821 | 0,488659908 | |
| 220689_at | - | -0,078265071 | -0,566887459 | 0,488622388 | |
| 242458_at | RALGPS2 | -0,078265071 | -0,566887459 | 0,488622388 | |
| 224284_x_at | FKSG49 | 1,603688695 | 1,115234684 | 0,48845401 | |
| 205653_at | CTSG | 0,351548291 | -0,136643983 | 0,488192274 | |
| 228029_at | ZNF721 | 3,214953914 | 2,726791458 | 0,488162456 | |
| 226449_at | CEP120 | 2,382221458 | 1,894118818 | 0,48810264 | |
| 1556199_a_at | RGS9BP | -1,424023738 | -1,912089812 | 0,488066074 | |
| 1556761_at | - | -1,424023738 | -1,912089812 | 0,488066074 | |

| | | | | | |
|---|---|---|---|---|---|
| 1559936_at | - | -1,424023738 | -1,912089812 | 0,488066074 | |
| 1565737_at | SCTR | -1,424023738 | -1,912089812 | 0,488066074 | |
| 207464_at | AHCYL1 | -1,424023738 | -1,912089812 | 0,488066074 | |
| 212755_at | MON2 | -1,424023738 | -1,912089812 | 0,488066074 | |
| 219308_s_at | AK5 | -1,424023738 | -1,912089812 | 0,488066074 | |
| 228982_s_at | USP42 | -1,424023738 | -1,912089812 | 0,488066074 | |
| 230043_at | MUC20 | -1,424023738 | -1,912089812 | 0,488066074 | |
| 238276_at | - | -1,424023738 | -1,912089812 | 0,488066074 | |
| 239972_at | - | -1,424023738 | -1,912089812 | 0,488066074 | |
| 207495_at | RAB28 | 0,44160593 | -0,046302147 | 0,487908077 | |
| 240574_at | DNAJC3-AS1 | 0,44160593 | -0,046302147 | 0,487908077 | |
| 206730_at | GRIA3 | -2,823166348 | -3,311072652 | 0,487906304 | |
| 201656_at | ITGA6 | 2,146943689 | 1,659204017 | 0,487739673 | |
| 1552332_at | TRIOBP | 1,224581536 | 0,73688026 | 0,487701275 | |
| 1554812_at | CLDN20 | -2,359436556 | -2,847001814 | 0,487565258 | |
| 1556648_a_at | C10orf40 | -2,359436556 | -2,847001814 | 0,487565258 | |
| 1564331_at | ZNF846 | -2,359436556 | -2,847001814 | 0,487565258 | |
| 206515_at | CYP4F3 | -2,359436556 | -2,847001814 | 0,487565258 | |
| 207033_at | GIF | -2,359436556 | -2,847001814 | 0,487565258 | |
| 220559_at | EN1 | -2,359436556 | -2,847001814 | 0,487565258 | |
| 231138_at | TRPC5OS | -2,359436556 | -2,847001814 | 0,487565258 | |
| 242291_at | EIF4ENIF1 | -2,359436556 | -2,847001814 | 0,487565258 | |
| 227007_at | TMCO4 | 0,291166226 | -0,19610998 | 0,487276206 | |
| 222137_at | CC2D1A | 0,384950573 | -0,102100538 | 0,487051111 | |
| 226550_at | SLC9A7 | 1,292162421 | 0,805216064 | 0,486946357 | |
| 1559190_s_at | RDH13 | 1,210676994 | 0,724100169 | 0,486576825 | |
| 214898_x_at | MUC3B | 0,228146222 | -0,258114234 | 0,486260455 | |
| 218550_s_at | LRRC20 | 0,228146222 | -0,258114234 | 0,486260455 | |
| 231776_at | EOMES | 0,228146222 | -0,258114234 | 0,486260455 | |
| 211636_at | IGHA1 /// IGHA2 | -2,551294263 | -3,037500868 | 0,486206605 | |
| 231841_s_at | KIAA1462 | -2,551294263 | -3,037500868 | 0,486206605 | |
| 233002_at | PPP4R4 | -2,551294263 | -3,037500868 | 0,486206605 | |
| 240711_at | - | -2,551294263 | -3,037500868 | 0,486206605 | |
| 242186_x_at | LPHN3 | -2,551294263 | -3,037500868 | 0,486206605 | |
| 226346_at | MEX3A | 1,177702228 | 0,691645685 | 0,486056542 | |
| 1552979_at | LINC00471 | -2,089474779 | -2,575129729 | 0,48565495 | |
| 1556332_at | - | -2,089474779 | -2,575129729 | 0,48565495 | |
| 1557880_at | PCDH11X /// PCD | -2,089474779 | -2,575129729 | 0,48565495 | |
| 1560286_s_at | - | -2,089474779 | -2,575129729 | 0,48565495 | |
| 1562130_at | IQCA1 | -2,089474779 | -2,575129729 | 0,48565495 | |
| 1562245_a_at | ZNF578 | -2,089474779 | -2,575129729 | 0,48565495 | |
| 1563427_at | - | -2,089474779 | -2,575129729 | 0,48565495 | |
| 1565887_at | TRPM7 | -2,089474779 | -2,575129729 | 0,48565495 | |
| 207632_at | MUSK | -2,089474779 | -2,575129729 | 0,48565495 | |
| 216238_s_at | FGB | -2,089474779 | -2,575129729 | 0,48565495 | |
| 221151_at | PRDM9 | -2,089474779 | -2,575129729 | 0,48565495 | |
| 234065_at | - | -2,089474779 | -2,575129729 | 0,48565495 | |
| 240549_at | - | -2,089474779 | -2,575129729 | 0,48565495 | |
| 241403_at | CLK4 | -2,089474779 | -2,575129729 | 0,48565495 | |
| 208169_s_at | PTGER3 | 0,661985843 | 0,176436073 | 0,485549769 | |
| 235798_at | TMEM170B | 0,661985843 | 0,176436073 | 0,485549769 | |
| 224352_s_at | CFL2 | 2,266015882 | 1,781437557 | 0,484578325 | |
| 1553145_at | FLJ39653 | -1,131062212 | -1,615132408 | 0,484070195 | |
| 1557417_s_at | RSPH10B /// RSP | -1,131062212 | -1,615132408 | 0,484070195 | |
| 1562412_at | - | -1,131062212 | -1,615132408 | 0,484070195 | |
| 1568789_at | UHRF1BP1 | -1,131062212 | -1,615132408 | 0,484070195 | |

| | | | | | |
|---|---|---|---|---|---|
| 205464_at | SCNN1B | -1,131062212 | -1,615132408 | 0,484070195 | |
| 220857_at | - | -1,131062212 | -1,615132408 | 0,484070195 | |
| 227429_at | EFCAB4A | -1,131062212 | -1,615132408 | 0,484070195 | |
| 227863_at | IFITM10 | -1,131062212 | -1,615132408 | 0,484070195 | |
| 230115_at | DKFZp779M0652 | -1,131062212 | -1,615132408 | 0,484070195 | |
| 242486_at | - | 1,549482847 | 1,065442793 | 0,484040054 | |
| 243916_x_at | UBLCP1 | 3,612245157 | 3,128242026 | 0,48400313 | |
| 217904_s_at | BACE1 | 0,20027154 | -0,283667828 | 0,483939367 | |
| 231724_at | MED26 | 0,20027154 | -0,283667828 | 0,483939367 | |
| 243948_at | - | 1,874520207 | 1,390636111 | 0,483884095 | |
| 222637_at | COMMD10 | 2,549899391 | 2,066015817 | 0,483883574 | |
| 214202_at | - | 0,641402394 | 0,157549243 | 0,483853151 | |
| 222844_s_at | SRR | 0,641402394 | 0,157549243 | 0,483853151 | |
| 226394_at | 05.03.15 | 2,027765132 | 1,543943227 | 0,483821905 | |
| 235416_at | LOC643201 | 1,037706275 | 0,553993624 | 0,483712651 | |
| 202486_at | AFG3L2 | 3,593615441 | 3,109937707 | 0,483677734 | |
| 203367_at | DUSP14 | 2,536956583 | 2,053299695 | 0,483656888 | |
| 211300_s_at | TP53 | 1,348276312 | 0,864649967 | 0,483626344 | |
| 1554158_at | ZMYND11 | -0,467091869 | -0,950686014 | 0,483594146 | |
| 1557558_s_at | MATN1-AS1 | -0,467091869 | -0,950686014 | 0,483594146 | |
| 207601_at | SULT1B1 | -0,467091869 | -0,950686014 | 0,483594146 | |
| 208553_at | HIST1H1E | -0,467091869 | -0,950686014 | 0,483594146 | |
| 203332_s_at | INPP5D | 2,325288363 | 1,841838775 | 0,483449589 | |
| 204320_at | COL11A1 | 2,64946283 | 2,166264721 | 0,483198109 | |
| 202553_s_at | SYF2 | 3,767525744 | 3,284389368 | 0,483136376 | |
| 1552863_a_at | CACNG6 | 1,318332599 | 0,835239052 | 0,483093547 | |
| 201228_s_at | ARIH2 | 1,318332599 | 0,835239052 | 0,483093547 | |
| 223493_at | FBXO4 | 1,269985355 | 0,786897721 | 0,483087633 | |
| 215754_at | SCARB2 | -0,196756757 | -0,679763839 | 0,483007082 | |
| 228604_at | FAM76A | -0,196756757 | -0,679763839 | 0,483007082 | |
| 224905_at | WDR26 | 2,433006244 | 1,950068772 | 0,482937472 | |
| 209626_s_at | OSBPL3 | 2,001086023 | 1,518263577 | 0,482822446 | |
| 1554379_a_at | TP73 | 0,133056859 | -0,34958478 | 0,482641639 | |
| 203703_s_at | TTLL4 | 0,133056859 | -0,34958478 | 0,482641639 | |
| 242981_at | - | 0,133056859 | -0,34958478 | 0,482641639 | |
| 1553502_a_at | AKAP2 /// PALM2 | -2,138804305 | -2,621331989 | 0,482527684 | |
| 1561710_at | SNX19 | -2,138804305 | -2,621331989 | 0,482527684 | |
| 205183_at | - | -2,138804305 | -2,621331989 | 0,482527684 | |
| 206140_at | LHX2 | -2,138804305 | -2,621331989 | 0,482527684 | |
| 211108_s_at | JAK3 | -2,138804305 | -2,621331989 | 0,482527684 | |
| 212992_at | AHNAK2 | -2,138804305 | -2,621331989 | 0,482527684 | |
| 217375_at | - | -2,138804305 | -2,621331989 | 0,482527684 | |
| 219871_at | FLJ13197 | -2,138804305 | -2,621331989 | 0,482527684 | |
| 220394_at | FGF20 | -2,138804305 | -2,621331989 | 0,482527684 | |
| 222877_at | - | -2,138804305 | -2,621331989 | 0,482527684 | |
| 231661_at | REG3G | -2,138804305 | -2,621331989 | 0,482527684 | |
| 237094_at | FAM19A5 | -2,138804305 | -2,621331989 | 0,482527684 | |
| 238271_x_at | - | -2,138804305 | -2,621331989 | 0,482527684 | |
| 239744_at | - | -2,138804305 | -2,621331989 | 0,482527684 | |
| 209298_s_at | ITSN1 | 0,962260738 | 0,479907041 | 0,482353697 | |
| 1553525_at | NLRP13 | 0,020670649 | -0,461594427 | 0,482265076 | |
| 210697_at | ZNF257 | 0,020670649 | -0,461594427 | 0,482265076 | |
| 217632_at | GNL3L | 0,020670649 | -0,461594427 | 0,482265076 | |
| 233077_at | NAV2-AS4 | 0,020670649 | -0,461594427 | 0,482265076 | |
| 233912_x_at | - | 0,020670649 | -0,461594427 | 0,482265076 | |
| 212720_at | PAPOLA | 3,734704399 | 3,252481567 | 0,482222833 | |

| | | | | | |
|---|---|---|---|---|---|
| 201784_s_at | C11orf58 | 5,666452598 | 5,184334316 | 0,482118282 | |
| 216563_at | ANKRD12 | 0,541261317 | 0,059218869 | 0,482042448 | |
| 1552698_at | TUBA3FP | -0,67289328 | -1,154897679 | 0,482004399 | |
| 1553281_at | PLCXD2 | -0,67289328 | -1,154897679 | 0,482004399 | |
| 1564369_at | - | -0,67289328 | -1,154897679 | 0,482004399 | |
| 209529_at | PPAP2C | -0,67289328 | -1,154897679 | 0,482004399 | |
| 234120_at | - | -0,67289328 | -1,154897679 | 0,482004399 | |
| 242834_at | - | -0,67289328 | -1,154897679 | 0,482004399 | |
| 216091_s_at | BTRC | 1,119359127 | 0,638150376 | 0,481208751 | |
| 1555476_at | IREB2 | -0,85085127 | -1,332017329 | 0,481166059 | |
| 1561391_at | STAU2-AS1 | -0,85085127 | -1,332017329 | 0,481166059 | |
| 1561919_at | - | -0,85085127 | -1,332017329 | 0,481166059 | |
| 214371_at | TSSK2 | -0,85085127 | -1,332017329 | 0,481166059 | |
| 216009_at | SLC39A9 | -0,85085127 | -1,332017329 | 0,481166059 | |
| 216480_x_at | MLLT10 | -0,85085127 | -1,332017329 | 0,481166059 | |
| 220231_at | PPP1R17 | -0,85085127 | -1,332017329 | 0,481166059 | |
| 227552_at | 37135 | -0,85085127 | -1,332017329 | 0,481166059 | |
| 230978_at | LOC100505659 | -0,85085127 | -1,332017329 | 0,481166059 | |
| 236416_at | ARHGEF7 | -0,85085127 | -1,332017329 | 0,481166059 | |
| 238350_at | UBN2 | -0,85085127 | -1,332017329 | 0,481166059 | |
| 239048_at | - | -0,85085127 | -1,332017329 | 0,481166059 | |
| 239054_at | SFMBT1 | -0,85085127 | -1,332017329 | 0,481166059 | |
| 244835_at | C16orf52 | -0,85085127 | -1,332017329 | 0,481166059 | |
| 206115_at | EGR3 | 2,623460554 | 2,14244844 | 0,481012114 | |
| 211709_s_at | CLEC11A | 3,343695493 | 2,862725455 | 0,480970038 | |
| 201083_s_at | BCLAF1 | 3,27186005 | 2,791007185 | 0,480852865 | |
| 235604_x_at | ZNF493 | 1,139070151 | 0,658444279 | 0,480625872 | |
| 222808_at | ALG13 | 3,088871381 | 2,608344689 | 0,480526692 | |
| 1562428_at | LOC654780 | -0,055687482 | -0,536087151 | 0,480399669 | |
| 208936_x_at | LGALS8 | -0,055687482 | -0,536087151 | 0,480399669 | |
| 216824_at | - | -0,055687482 | -0,536087151 | 0,480399669 | |
| 235135_at | TAF10 | -0,055687482 | -0,536087151 | 0,480399669 | |
| 241601_at | WIPF3 | -0,055687482 | -0,536087151 | 0,480399669 | |
| 243447_at | - | -0,055687482 | -0,536087151 | 0,480399669 | |
| 223675_s_at | VEZT | 2,410880861 | 1,930732138 | 0,480148723 | |
| 238907_at | ZNF780A | 1,104397092 | 0,624460759 | 0,479936333 | |
| 218717_s_at | LEPREL1 | 1,04816921 | 0,568365095 | 0,479804115 | |
| 220129_at | CCDC169-SOHL | -0,285837228 | -0,765504029 | 0,479666801 | |
| 222866_s_at | FLVCR2 | -0,285837228 | -0,765504029 | 0,479666801 | |
| 1559651_at | CSNK1A1P1 | -2,756478458 | -3,236070201 | 0,479591743 | |
| 1562352_at | - | -2,756478458 | -3,236070201 | 0,479591743 | |
| 1567000_at | - | -2,756478458 | -3,236070201 | 0,479591743 | |
| 212395_s_at | EMC1 | 2,59519831 | 2,115788273 | 0,479410037 | |
| 1557103_a_at | LMTK3 | -1,308181863 | -1,787089803 | 0,47890794 | |
| 1558011_at | LOC100506903 / | -1,308181863 | -1,787089803 | 0,47890794 | |
| 1560290_at | - | -1,308181863 | -1,787089803 | 0,47890794 | |
| 1565713_at | - | -1,308181863 | -1,787089803 | 0,47890794 | |
| 1565859_at | SNORA71A | -1,308181863 | -1,787089803 | 0,47890794 | |
| 205994_at | ELK4 | -1,308181863 | -1,787089803 | 0,47890794 | |
| 213173_at | PCNX | -1,308181863 | -1,787089803 | 0,47890794 | |
| 214032_at | ZAP70 | -1,308181863 | -1,787089803 | 0,47890794 | |
| 222085_at | FAM174B | -1,308181863 | -1,787089803 | 0,47890794 | |
| 229600_s_at | CPD | -1,308181863 | -1,787089803 | 0,47890794 | |
| 233631_x_at | KRTAP9-2 /// KR | -1,308181863 | -1,787089803 | 0,47890794 | |
| 233773_at | - | -1,308181863 | -1,787089803 | 0,47890794 | |
| 239511_s_at | SRSF4 | -1,308181863 | -1,787089803 | 0,47890794 | |

| | | | | | |
|---|---|---|---|---|---|
| 242737_at | - | -1,308181863 | -1,787089803 | 0,47890794 | |
| 1553280_at | PUS10 | 0,376672223 | -0,102100538 | 0,478772761 | |
| 233451_at | SLC52A3 | 0,376672223 | -0,102100538 | 0,478772761 | |
| 1554132_a_at | FAM190B | -0,741668563 | -1,220210588 | 0,478542025 | |
| 228532_at | C1orf162 | -0,741668563 | -1,220210588 | 0,478542025 | |
| 228981_at | TMEM169 | -0,741668563 | -1,220210588 | 0,478542025 | |
| 229954_at | CHDH | -0,741668563 | -1,220210588 | 0,478542025 | |
| 1555131_a_at | PER3 | -0,136301282 | -0,614371577 | 0,478070294 | |
| 1558466_at | UGGT2 | -0,136301282 | -0,614371577 | 0,478070294 | |
| 1567457_at | RAC1 | -0,136301282 | -0,614371577 | 0,478070294 | |
| 211795_s_at | FYB | -0,136301282 | -0,614371577 | 0,478070294 | |
| 231890_at | - | -0,136301282 | -0,614371577 | 0,478070294 | |
| 243751_at | CHD2 | -0,136301282 | -0,614371577 | 0,478070294 | |
| 235984_at | - | 0,682279745 | 0,204310755 | 0,47796899 | |
| 225346_at | MTERFD3 | 1,593009234 | 1,115234684 | 0,47777455 | |
| 1554774_at | MINA | 0,317354302 | -0,160136748 | 0,47749105 | |
| 1559132_at | TMEM80 | 0,317354302 | -0,160136748 | 0,47749105 | |
| 240843_at | - | 0,317354302 | -0,160136748 | 0,47749105 | |
| 205398_s_at | SMAD3 | 1,567778694 | 1,090553537 | 0,477225156 | |
| 202730_s_at | MIR4680 /// PDC | 2,929639344 | 2,452428893 | 0,47721045 | |
| 203760_s_at | SLA | -0,089689931 | -0,566887459 | 0,477197528 | |
| 229329_s_at | - | -0,089689931 | -0,566887459 | 0,477197528 | |
| 239574_at | - | -0,089689931 | -0,566887459 | 0,477197528 | |
| 232811_x_at | PRICKLE1 | 0,87091093 | 0,393761504 | 0,477149426 | |
| 211122_s_at | CXCL11 | 3,006764929 | 2,529740643 | 0,477024286 | |
| 235253_at | RAD1 | 1,976645579 | 1,499637023 | 0,477008556 | |
| 205091_x_at | RECQL | 1,381753566 | 0,904843258 | 0,476910308 | |
| 226656_at | CRTAP | 1,722769813 | 1,246149888 | 0,476619925 | |
| 234677_at | - | 0,555999837 | 0,079428135 | 0,476571702 | |
| 1556427_s_at | LRRN4CL | -0,946847173 | -1,423348341 | 0,476501168 | |
| 1561152_a_at | - | -0,946847173 | -1,423348341 | 0,476501168 | |
| 204339_s_at | RGS4 | -0,946847173 | -1,423348341 | 0,476501168 | |
| 210182_at | CORT | -0,946847173 | -1,423348341 | 0,476501168 | |
| 211024_s_at | NKX2-1 | -0,946847173 | -1,423348341 | 0,476501168 | |
| 211455_at | - | -0,946847173 | -1,423348341 | 0,476501168 | |
| 220267_at | KRT24 | -0,946847173 | -1,423348341 | 0,476501168 | |
| 220406_at | TGFB2 | -0,946847173 | -1,423348341 | 0,476501168 | |
| 221723_s_at | SLC4A5 | -0,946847173 | -1,423348341 | 0,476501168 | |
| 237407_at | HS1BP3 | -0,946847173 | -1,423348341 | 0,476501168 | |
| 243516_at | - | -0,946847173 | -1,423348341 | 0,476501168 | |
| 244365_at | - | -0,946847173 | -1,423348341 | 0,476501168 | |
| 203913_s_at | HPGD | 2,817903185 | 2,341749893 | 0,476153292 | |
| 1569745_at | LOC100505783 | 0,255492549 | -0,220592223 | 0,476084773 | |
| 214793_at | DUSP7 | 0,255492549 | -0,220592223 | 0,476084773 | |
| 230246_at | PLAC9 | 0,255492549 | -0,220592223 | 0,476084773 | |
| 231874_at | FAM126B | 0,255492549 | -0,220592223 | 0,476084773 | |
| 242424_at | - | 0,255492549 | -0,220592223 | 0,476084773 | |
| 1554098_at | SPIN3 | -0,57445847 | -1,050406615 | 0,475948145 | |
| 206917_at | GNA13 | -0,57445847 | -1,050406615 | 0,475948145 | |
| 219024_at | PLEKHA1 | -0,57445847 | -1,050406615 | 0,475948145 | |
| 226819_at | LSM11 | -0,57445847 | -1,050406615 | 0,475948145 | |
| 229114_at | GAB1 | -0,57445847 | -1,050406615 | 0,475948145 | |
| 230530_at | - | -0,57445847 | -1,050406615 | 0,475948145 | |
| 234373_x_at | - | -0,57445847 | -1,050406615 | 0,475948145 | |
| 239715_at | - | -0,57445847 | -1,050406615 | 0,475948145 | |
| 239879_at | LOC284998 | -0,57445847 | -1,050406615 | 0,475948145 | |

| | | | | | |
|---|---|---|---|---|---|
| 1555272_at | RSPH10B /// RSF | 1,917954284 | 1,442269454 | 0,47568483 | |
| 204533_at | CXCL10 | 0,853119696 | 0,377530647 | 0,475589049 | |
| 217622_at | RHBDD3 | 0,853119696 | 0,377530647 | 0,475589049 | |
| 222156_x_at | CCPG1 /// DYX1( | 0,152582607 | -0,322870281 | 0,475452888 | |
| 228219_s_at | UPB1 | 0,152582607 | -0,322870281 | 0,475452888 | |
| 211081_s_at | MAP4K5 | 1,079110292 | 0,603679647 | 0,475430645 | |
| 230205_at | ZNF561 | 1,079110292 | 0,603679647 | 0,475430645 | |
| 214711_at | GATC | 2,566371329 | 2,091116676 | 0,475254654 | |
| 222717_at | SDPR | 5,069317741 | 4,594226933 | 0,475090808 | |
| 219188_s_at | MACROD1 | 1,292162421 | 0,817300337 | 0,474862084 | |
| 202203_s_at | AMFR | 2,31665944 | 1,841838775 | 0,474820665 | |
| 1554981_at | C9orf11 | -2,479842156 | -2,954576755 | 0,474734599 | |
| 1569512_at | - | -2,479842156 | -2,954576755 | 0,474734599 | |
| 208193_at | IL9 | -2,479842156 | -2,954576755 | 0,474734599 | |
| 214621_at | GYS2 | -2,479842156 | -2,954576755 | 0,474734599 | |
| 222287_at | TRDN | -2,479842156 | -2,954576755 | 0,474734599 | |
| 229724_at | GABRB3 | -2,479842156 | -2,954576755 | 0,474734599 | |
| 232233_at | SLC22A16 | -2,479842156 | -2,954576755 | 0,474734599 | |
| 237127_at | - | -2,479842156 | -2,954576755 | 0,474734599 | |
| 204288_s_at | SORBS2 | 0,533835206 | 0,059218869 | 0,474616338 | |
| 212984_at | ATF2 | 3,488057722 | 3,013449121 | 0,474608601 | |
| 207067_s_at | HDC | -0,43775896 | -0,912331589 | 0,474572628 | |
| 210214_s_at | BMPR2 | -0,43775896 | -0,912331589 | 0,474572628 | |
| 220698_at | MGC4294 | -0,43775896 | -0,912331589 | 0,474572628 | |
| 226812_at | - | -0,43775896 | -0,912331589 | 0,474572628 | |
| 229968_at | - | -0,43775896 | -0,912331589 | 0,474572628 | |
| 1562327_at | - | -1,688127507 | -2,162639771 | 0,474512265 | |
| 203587_at | ARL4D | -1,688127507 | -2,162639771 | 0,474512265 | |
| 207202_s_at | NR1I2 | -1,688127507 | -2,162639771 | 0,474512265 | |
| 215033_at | TM4SF1 | -1,688127507 | -2,162639771 | 0,474512265 | |
| 217328_at | IL23A | -1,688127507 | -2,162639771 | 0,474512265 | |
| 222964_at | - | -1,688127507 | -2,162639771 | 0,474512265 | |
| 229399_at | C10orf118 | -1,688127507 | -2,162639771 | 0,474512265 | |
| 230251_at | LINC00473 | -1,688127507 | -2,162639771 | 0,474512265 | |
| 231326_s_at | LOC100506234 | -1,688127507 | -2,162639771 | 0,474512265 | |
| 233317_at | CD9 | -1,688127507 | -2,162639771 | 0,474512265 | |
| 237452_at | - | -1,688127507 | -2,162639771 | 0,474512265 | |
| 238200_at | - | -1,688127507 | -2,162639771 | 0,474512265 | |
| 242702_at | MMAA | -1,688127507 | -2,162639771 | 0,474512265 | |
| 207675_x_at | ARTN | -0,172274514 | -0,646746079 | 0,474471565 | |
| 225328_at | FBXO32 | -0,172274514 | -0,646746079 | 0,474471565 | |
| 231964_at | BICD1 | -0,172274514 | -0,646746079 | 0,474471565 | |
| 1559079_at | LINC00567 | -1,812418401 | -2,286583451 | 0,474165049 | |
| 1559616_x_at | ZNF626 | -1,812418401 | -2,286583451 | 0,474165049 | |
| 1562336_at | CASC2 | -1,812418401 | -2,286583451 | 0,474165049 | |
| 1563776_at | LOC100507283 | -1,812418401 | -2,286583451 | 0,474165049 | |
| 1566469_at | - | -1,812418401 | -2,286583451 | 0,474165049 | |
| 1570528_at | XYLT2 | -1,812418401 | -2,286583451 | 0,474165049 | |
| 206039_at | RAB33A | -1,812418401 | -2,286583451 | 0,474165049 | |
| 207242_s_at | GRIK1 | -1,812418401 | -2,286583451 | 0,474165049 | |
| 211481_at | SLCO1A2 | -1,812418401 | -2,286583451 | 0,474165049 | |
| 216020_at | IFIH1 | -1,812418401 | -2,286583451 | 0,474165049 | |
| 222142_at | CYLD | -1,812418401 | -2,286583451 | 0,474165049 | |
| 230517_at | ZNF775 | -1,812418401 | -2,286583451 | 0,474165049 | |
| 236877_at | - | -1,812418401 | -2,286583451 | 0,474165049 | |
| 238137_at | - | -1,812418401 | -2,286583451 | 0,474165049 | |

| | | | | | |
|---|---|---|---|---|---|
| 238726_at | - | -1,812418401 | -2,286583451 | 0,474165049 | |
| 240275_at | ARMC3 | -1,812418401 | -2,286583451 | 0,474165049 | |
| 243657_at | - | -1,812418401 | -2,286583451 | 0,474165049 | |
| 218419_s_at | TMUB2 | 1,063722698 | 0,589657387 | 0,474065311 | |
| 220115_s_at | CDH10 | 0,083054335 | -0,39061235 | 0,473666685 | |
| 225978_at | RIMKLB | 0,767063567 | 0,293518836 | 0,473544731 | |
| 221541_at | CRISPLD2 | 0,480753764 | 0,007421914 | 0,47333185 | |
| 222591_at | STYXL1 | 0,480753764 | 0,007421914 | 0,47333185 | |
| 211760_s_at | VAMP4 | 1,027166904 | 0,553993624 | 0,47317328 | |
| 1568720_at | ZNF506 | -2,262747984 | -2,735862426 | 0,473114441 | |
| 206408_at | LRRTM2 | -2,262747984 | -2,735862426 | 0,473114441 | |
| 206557_at | ZNF702P | -2,262747984 | -2,735862426 | 0,473114441 | |
| 208933_s_at | LGALS8 | -2,262747984 | -2,735862426 | 0,473114441 | |
| 214559_at | DRD3 | -2,262747984 | -2,735862426 | 0,473114441 | |
| 230680_at | SLITRK4 | -2,262747984 | -2,735862426 | 0,473114441 | |
| 230776_at | RNF157-AS1 | -2,262747984 | -2,735862426 | 0,473114441 | |
| 240337_at | RHOA | -2,262747984 | -2,735862426 | 0,473114441 | |
| 240581_at | LOC644135 | -2,262747984 | -2,735862426 | 0,473114441 | |
| 238401_at | ENDOV /// LOC1 | 0,695652136 | 0,222599095 | 0,473053042 | |
| 213637_at | DDX52 | 3,231115193 | 2,758473378 | 0,472641815 | |
| 214172_x_at | RYK | 1,850809354 | 1,378453643 | 0,472355711 | |
| 230814_at | C19orf81 | 1,143956113 | 0,67181667 | 0,472139443 | |
| 1555733_s_at | AP1S3 | -2,667025278 | -3,138626879 | 0,471601601 | |
| 1562381_at | HLA-F-AS1 | -2,667025278 | -3,138626879 | 0,471601601 | |
| 1564107_at | - | -2,667025278 | -3,138626879 | 0,471601601 | |
| 1570111_at | LINC00521 | -2,667025278 | -3,138626879 | 0,471601601 | |
| 212999_x_at | HLA-DQB1 /// LO | -2,667025278 | -3,138626879 | 0,471601601 | |
| 227238_at | MUC15 | -2,667025278 | -3,138626879 | 0,471601601 | |
| 243799_x_at | ANGPTL3 | -2,667025278 | -3,138626879 | 0,471601601 | |
| 240512_x_at | KCTD4 | -2,930741289 | -3,402185466 | 0,471444177 | |
| 229220_x_at | NOM1 | 4,315944589 | 3,844556416 | 0,471388174 | |
| 1570257_x_at | - | 0,67554678 | 0,204310755 | 0,471236025 | |
| 204659_s_at | GFER | 0,67554678 | 0,204310755 | 0,471236025 | |
| 206836_at | SLC6A3 | 0,67554678 | 0,204310755 | 0,471236025 | |
| 243198_at | TEX9 | 0,67554678 | 0,204310755 | 0,471236025 | |
| 215621_s_at | IGHD | 0,334552598 | -0,136643983 | 0,471196581 | |
| 1552599_at | PXT1 | -1,937711681 | -2,408705138 | 0,470993456 | |
| 1553817_at | LOC100653171 / | -1,937711681 | -2,408705138 | 0,470993456 | |
| 1554825_at | - | -1,937711681 | -2,408705138 | 0,470993456 | |
| 1555462_at | PPP1R1C | -1,937711681 | -2,408705138 | 0,470993456 | |
| 1560354_at | - | -1,937711681 | -2,408705138 | 0,470993456 | |
| 1563845_at | FAM153B /// LOC | -1,937711681 | -2,408705138 | 0,470993456 | |
| 1565132_at | RBMY3AP | -1,937711681 | -2,408705138 | 0,470993456 | |
| 1569721_at | - | -1,937711681 | -2,408705138 | 0,470993456 | |
| 203708_at | PDE4B | -1,937711681 | -2,408705138 | 0,470993456 | |
| 211414_at | GLS | -1,937711681 | -2,408705138 | 0,470993456 | |
| 214475_x_at | CAPN3 | -1,937711681 | -2,408705138 | 0,470993456 | |
| 232768_at | CCNB2 | -1,937711681 | -2,408705138 | 0,470993456 | |
| 233331_at | - | -1,937711681 | -2,408705138 | 0,470993456 | |
| 243556_at | NGEF | -1,937711681 | -2,408705138 | 0,470993456 | |
| 1557567_a_at | LOC100507634 | -0,259832361 | -0,730778808 | 0,470946447 | |
| 206738_at | APOC2 /// APOC | -0,259832361 | -0,730778808 | 0,470946447 | |
| 231188_at | ZSCAN2 | -0,259832361 | -0,730778808 | 0,470946447 | |
| 233733_at | - | -0,259832361 | -0,730778808 | 0,470946447 | |
| 206003_at | CEP135 | 2,972842302 | 2,501934108 | 0,470908194 | |
| 221951_at | TMEM80 | 2,043538949 | 1,572742379 | 0,47079657 | |

| | | | | | |
|---|---|---|---|---|---|
| 1556597_a_at | LOC284513 | 0,052199481 | -0,41858459 | 0,47078407 | |
| 202834_at | AGT | 0,052199481 | -0,41858459 | 0,47078407 | |
| 233070_at | ZNF197 | 0,052199481 | -0,41858459 | 0,47078407 | |
| 242855_at | KCP | 0,052199481 | -0,41858459 | 0,47078407 | |
| 209938_at | TADA2A | 0,888485433 | 0,417770464 | 0,47071497 | |
| 1554450_s_at | MIER3 | 1,889144008 | 1,418668082 | 0,470475925 | |
| 222607_s_at | DIS3 | 4,022255566 | 3,551810897 | 0,47044467 | |
| 219661_at | RANBP17 | 1,912238072 | 1,442269454 | 0,469968618 | |
| 227471_at | HACE1 | 2,084778276 | 1,614891062 | 0,469887215 | |
| 242442_x_at | TRMT10A | 1,094335495 | 0,624460759 | 0,469874736 | |
| 203830_at | C17orf75 | 1,446464021 | 0,976641161 | 0,46982286 | |
| 218668_s_at | RAP2C | 3,443176398 | 2,973511251 | 0,469665147 | |
| 222270_at | SMEK2 | 0,273439642 | -0,19610998 | 0,469549622 | |
| 218372_at | MED9 | 1,037706275 | 0,568365095 | 0,46934118 | |
| 1555027_at | P4HA2 | -2,311268652 | -2,780313924 | 0,469045273 | |
| 200795_at | SPARCL1 | -2,311268652 | -2,780313924 | 0,469045273 | |
| 202948_at | IL1R1 | -2,311268652 | -2,780313924 | 0,469045273 | |
| 207516_at | CHRNB4 | -2,311268652 | -2,780313924 | 0,469045273 | |
| 241119_at | - | -2,311268652 | -2,780313924 | 0,469045273 | |
| 243563_at | - | -2,311268652 | -2,780313924 | 0,469045273 | |
| 1567166_at | - | 0,779676066 | 0,310717132 | 0,468958934 | |
| 226097_at | FNDC5 | 0,30867765 | -0,160136748 | 0,468814398 | |
| 227726_at | RNF166 | 0,30867765 | -0,160136748 | 0,468814398 | |
| 201294_s_at | WSB1 | 1,224581536 | 0,755840599 | 0,468740936 | |
| 203640_at | MBNL2 | 4,759679153 | 4,291026333 | 0,46865282 | |
| 218986_s_at | DDX60 | 2,14450783 | 1,675891488 | 0,468616342 | |
| 1569351_at | SOBP | -0,79540287 | -1,264005341 | 0,468602471 | |
| 210397_at | DEFB1 | -0,79540287 | -1,264005341 | 0,468602471 | |
| 219638_at | FBXO22 | -0,79540287 | -1,264005341 | 0,468602471 | |
| 225883_at | ATG16L2 | -0,79540287 | -1,264005341 | 0,468602471 | |
| 229117_s_at | JUND | -0,79540287 | -1,264005341 | 0,468602471 | |
| 235530_at | - | -0,79540287 | -1,264005341 | 0,468602471 | |
| 239578_at | - | -0,79540287 | -1,264005341 | 0,468602471 | |
| 239915_at | HOXA-AS3 | -0,79540287 | -1,264005341 | 0,468602471 | |
| 242378_at | MYADML2 | -0,79540287 | -1,264005341 | 0,468602471 | |
| 205264_at | CD3EAP | 3,479385765 | 3,010820719 | 0,468565046 | |
| 203005_at | LTBR | 2,333865983 | 1,865308541 | 0,468557441 | |
| 207688_s_at | - | 3,03990821 | 2,571362843 | 0,468545366 | |
| 237503_at | SLC5A8 | -0,022466681 | -0,490927335 | 0,468460654 | |
| 239435_x_at | SHROOM1 | -0,022466681 | -0,490927335 | 0,468460654 | |
| 244477_at | - | -0,022466681 | -0,490927335 | 0,468460654 | |
| 203645_s_at | CD163 | -1,026571149 | -1,494668682 | 0,468097533 | |
| 205623_at | ALDH3A1 | -1,026571149 | -1,494668682 | 0,468097533 | |
| 207736_s_at | TNP2 | -1,026571149 | -1,494668682 | 0,468097533 | |
| 209925_at | OCLN | -1,026571149 | -1,494668682 | 0,468097533 | |
| 211156_at | CDKN2A | -1,026571149 | -1,494668682 | 0,468097533 | |
| 214064_at | TF | -1,026571149 | -1,494668682 | 0,468097533 | |
| 215154_at | ULK2 | -1,026571149 | -1,494668682 | 0,468097533 | |
| 224041_at | TTTY6 /// TTTY6 | -1,026571149 | -1,494668682 | 0,468097533 | |
| 229150_at | - | -1,026571149 | -1,494668682 | 0,468097533 | |
| 231706_s_at | EVX1 | -1,026571149 | -1,494668682 | 0,468097533 | |
| 235001_at | DNAJC21 | -1,026571149 | -1,494668682 | 0,468097533 | |
| 236242_at | - | -1,026571149 | -1,494668682 | 0,468097533 | |
| 236578_at | - | -1,026571149 | -1,494668682 | 0,468097533 | |
| 242071_x_at | ITGA8 | -1,026571149 | -1,494668682 | 0,468097533 | |
| 222726_s_at | EXOC5 | 2,834621043 | 2,366632651 | 0,467988392 | |

| | | | | | |
|---|---|---|---|---|---|
| 236667_at | TMEM120B | 1,191926657 | 0,724100169 | 0,467826487 | |
| 206451_at | TBCCD1 | 2,764969948 | 2,297144887 | 0,467825061 | |
| 242923_at | ZNF678 | 0,496120222 | 0,028364014 | 0,467756208 | |
| 1552272_a_at | PRR22 | 0,209623097 | -0,258114234 | 0,467737331 | |
| 206838_at | TBX19 | 0,209623097 | -0,258114234 | 0,467737331 | |
| 213345_at | NFATC4 | 0,209623097 | -0,258114234 | 0,467737331 | |
| 218666_s_at | STX17 | 0,735041319 | 0,26733076 | 0,46771056 | |
| 232397_at | LOC100507039 | 3,643632572 | 3,175947691 | 0,467684881 | |
| 202996_at | POLD4 | 1,496984567 | 1,029536893 | 0,467447674 | |
| 1554485_s_at | TMEM37 | 0,760715727 | 0,293518836 | 0,467196891 | |
| 206414_s_at | ASAP2 | 0,760715727 | 0,293518836 | 0,467196891 | |
| 1553088_a_at | BCL2L11 | 1,748661581 | 1,281471383 | 0,467190198 | |
| 1554916_a_at | JRK | -0,543051993 | -1,010192375 | 0,467140382 | |
| 214622_at | CYP21A2 | -0,543051993 | -1,010192375 | 0,467140382 | |
| 220068_at | VPREB3 | -0,543051993 | -1,010192375 | 0,467140382 | |
| 230537_at | - | -0,543051993 | -1,010192375 | 0,467140382 | |
| 234487_at | - | -0,543051993 | -1,010192375 | 0,467140382 | |
| 236232_at | - | -0,543051993 | -1,010192375 | 0,467140382 | |
| 241615_x_at | - | -0,543051993 | -1,010192375 | 0,467140382 | |
| 242888_at | PRRT3-AS1 | -0,543051993 | -1,010192375 | 0,467140382 | |
| 222424_s_at | NUCKS1 | 2,693452197 | 2,226407528 | 0,467044669 | |
| 1553141_at | LACC1 | -1,196375121 | -1,663414495 | 0,467039373 | |
| 1557768_at | - | -1,196375121 | -1,663414495 | 0,467039373 | |
| 1569310_at | IRGQ | -1,196375121 | -1,663414495 | 0,467039373 | |
| 220644_at | - | -1,196375121 | -1,663414495 | 0,467039373 | |
| 227691_at | AKAP8L | -1,196375121 | -1,663414495 | 0,467039373 | |
| 229659_s_at | - | -1,196375121 | -1,663414495 | 0,467039373 | |
| 233348_at | - | -1,196375121 | -1,663414495 | 0,467039373 | |
| 233788_at | - | -1,196375121 | -1,663414495 | 0,467039373 | |
| 237152_at | PCDP1 | -1,196375121 | -1,663414495 | 0,467039373 | |
| 242665_at | FMNL2 | -1,196375121 | -1,663414495 | 0,467039373 | |
| 205132_at | ACTC1 | 0,246434561 | -0,220592223 | 0,467026784 | |
| 210924_at | OLFM1 | 0,246434561 | -0,220592223 | 0,467026784 | |
| 218703_at | SEC22A | 0,246434561 | -0,220592223 | 0,467026784 | |
| 229204_at | - | 1,331241794 | 0,864649967 | 0,466591827 | |
| 203106_s_at | VPS41 | -0,299034815 | -0,765504029 | 0,466469214 | |
| 216138_at | - | -0,299034815 | -0,765504029 | 0,466469214 | |
| 218451_at | CDCP1 | -0,299034815 | -0,765504029 | 0,466469214 | |
| 237595_at | - | -0,299034815 | -0,765504029 | 0,466469214 | |
| 1552893_at | CAMK2N2 | -0,352951799 | -0,819238336 | 0,466286538 | |
| 1556232_at | KIF6 | -0,352951799 | -0,819238336 | 0,466286538 | |
| 1569316_at | TRIM24 | -0,352951799 | -0,819238336 | 0,466286538 | |
| 222914_s_at | TMEM121 | -0,352951799 | -0,819238336 | 0,466286538 | |
| 240920_at | - | -0,352951799 | -0,819238336 | 0,466286538 | |
| 220257_x_at | NXF2 /// NXF2B | -0,148196756 | -0,614371577 | 0,46617482 | |
| 234986_at | GCLM | 0,715481152 | 0,249604176 | 0,465876976 | |
| 224801_at | NDFIP2 | 2,114952142 | 1,64909809 | 0,465854052 | |
| 202412_s_at | USP1 | 2,325288363 | 1,859476797 | 0,465811566 | |
| 1554994_at | RAG1 | -2,712026959 | -3,177802286 | 0,465775327 | |
| 1556888_at | - | -2,712026959 | -3,177802286 | 0,465775327 | |
| 1561679_at | - | -2,712026959 | -3,177802286 | 0,465775327 | |
| 1561962_at | - | -2,712026959 | -3,177802286 | 0,465775327 | |
| 1569254_s_at | INTS4 | -2,712026959 | -3,177802286 | 0,465775327 | |
| 230276_at | FAM49A | -2,712026959 | -3,177802286 | 0,465775327 | |
| 232786_at | COG6 | -2,712026959 | -3,177802286 | 0,465775327 | |
| 235587_at | LOC202781 | 2,027765132 | 1,562009954 | 0,465755178 | |

| | | | | | |
|---|---|---|---|---|---|
| 219150_s_at | ADAP1 | 0,859074518 | 0,393761504 | 0,465313014 | |
| 215767_at | ZNF804A | 0,181384709 | -0,283667828 | 0,465052537 | |
| 220442_at | GALNT4 /// POC1 | 0,181384709 | -0,283667828 | 0,465052537 | |
| 227209_at | CNTN1 | 0,181384709 | -0,283667828 | 0,465052537 | |
| 221846_s_at | CASKIN2 | 0,641402394 | 0,176436073 | 0,464966321 | |
| 216583_x_at | - | 1,089278259 | 0,624460759 | 0,4648175 | |
| 1554608_at | TGOLN2 | 0,384950573 | -0,079522948 | 0,464473522 | |
| 241964_at | ZNF865 | 0,384950573 | -0,079522948 | 0,464473522 | |
| 213906_at | MYBL1 | 1,201332287 | 0,73688026 | 0,464452026 | |
| 219502_at | NEIL3 | 2,001086023 | 1,536652708 | 0,464433315 | |
| 215054_at | EPOR | 3,801937331 | 3,337798009 | 0,464139322 | |
| 217837_s_at | CHMP3 /// RNF10 | 3,837056482 | 3,373018423 | 0,464038059 | |
| 223536_at | PSD2 | 0,417596971 | -0,046302147 | 0,463899117 | |
| 209006_s_at | C1orf63 | 2,470441349 | 2,006570641 | 0,463870708 | |
| 209850_s_at | CDC42EP2 | 1,874520207 | 1,410714371 | 0,463805835 | |
| 221882_s_at | TMEM8A | 2,238870125 | 1,775255987 | 0,463614137 | |
| 229205_at | LOC100507372 | 0,841135803 | 0,377530647 | 0,463605156 | |
| 213241_at | PLXNC1 | 0,072842263 | -0,39061235 | 0,463454613 | |
| 231567_s_at | CCDC62 | 0,072842263 | -0,39061235 | 0,463454613 | |
| 235819_at | BTF3L4 | 0,072842263 | -0,39061235 | 0,463454613 | |
| 237977_at | - | 0,072842263 | -0,39061235 | 0,463454613 | |
| 228966_at | PANK2 | 0,592200562 | 0,128747141 | 0,463453421 | |
| 217027_x_at | - | 1,951783949 | 1,488344553 | 0,463439396 | |
| 209464_at | AURKB | 2,783874823 | 2,320681318 | 0,463193505 | |
| 1570070_at | C20orf160 | 1,593009234 | 1,129843209 | 0,463166025 | |
| 1552459_a_at | MBD3L1 | -1,399512875 | -1,862647763 | 0,463134887 | |
| 1552948_at | CCDC27 | -1,399512875 | -1,862647763 | 0,463134887 | |
| 213961_s_at | - | -1,399512875 | -1,862647763 | 0,463134887 | |
| 216437_at | - | -1,399512875 | -1,862647763 | 0,463134887 | |
| 217638_at | - | -1,399512875 | -1,862647763 | 0,463134887 | |
| 221088_s_at | PPP1R9A | -1,399512875 | -1,862647763 | 0,463134887 | |
| 229183_at | KCTD1 | -1,399512875 | -1,862647763 | 0,463134887 | |
| 230575_at | MSRB2 | -1,399512875 | -1,862647763 | 0,463134887 | |
| 239627_at | - | -1,399512875 | -1,862647763 | 0,463134887 | |
| 239634_at | - | -1,399512875 | -1,862647763 | 0,463134887 | |
| 239834_at | - | -1,399512875 | -1,862647763 | 0,463134887 | |
| 243980_at | ZNF594 | -1,399512875 | -1,862647763 | 0,463134887 | |
| 200931_s_at | VCL | 5,498032951 | 5,035495453 | 0,462537498 | |
| 223700_at | MND1 | 3,310399576 | 2,848075314 | 0,462324262 | |
| 233993_at | MUC3 | 0,798390451 | 0,336136178 | 0,462254273 | |
| 1553556_at | TAS2R40 | -2,384869671 | -2,847001814 | 0,462132143 | |
| 1553708_at | LINC00525 | -2,384869671 | -2,847001814 | 0,462132143 | |
| 1560790_at | GOLGA6L2 | -2,384869671 | -2,847001814 | 0,462132143 | |
| 1561642_at | - | -2,384869671 | -2,847001814 | 0,462132143 | |
| 1565424_at | LINC00529 | -2,384869671 | -2,847001814 | 0,462132143 | |
| 1565740_at | - | -2,384869671 | -2,847001814 | 0,462132143 | |
| 211480_s_at | SLCO1A2 | -2,384869671 | -2,847001814 | 0,462132143 | |
| 222326_at | - | -2,384869671 | -2,847001814 | 0,462132143 | |
| 230866_at | CYSLTR1 | -2,384869671 | -2,847001814 | 0,462132143 | |
| 234493_at | LOC116437 | -2,384869671 | -2,847001814 | 0,462132143 | |
| 236095_at | NTRK2 | -2,384869671 | -2,847001814 | 0,462132143 | |
| 239716_at | - | -2,384869671 | -2,847001814 | 0,462132143 | |
| 243345_at | RNF14 | -2,384869671 | -2,847001814 | 0,462132143 | |
| 204057_at | IRF8 | 0,359971644 | -0,102100538 | 0,462072182 | |
| 229879_at | - | 0,541261317 | 0,079428135 | 0,461833181 | |
| 243840_at | CLSPN | 1,880387527 | 1,418668082 | 0,461719445 | |

| | | | | | |
|---|---|---|---|---|---|
| 1557300_s_at | - | 0,95671774 | 0,495031851 | 0,461685889 | |
| 213440_at | RAB1A | 2,446911392 | 1,98530622 | 0,461605172 | |
| 219111_s_at | DDX54 | 1,638726528 | 1,177496821 | 0,461229707 | |
| 218409_s_at | DNAJC1 | 2,632180155 | 2,170981159 | 0,461198996 | |
| 223520_s_at | KIF13A | -0,394749123 | -0,855751026 | 0,461001903 | |
| 228508_at | MAML3 | -0,394749123 | -0,855751026 | 0,461001903 | |
| 228787_s_at | BCAS4 | -0,394749123 | -0,855751026 | 0,461001903 | |
| 230799_at | LOC100134259 | -0,394749123 | -0,855751026 | 0,461001903 | |
| 240723_at | - | -0,394749123 | -0,855751026 | 0,461001903 | |
| 243671_at | - | -0,394749123 | -0,855751026 | 0,461001903 | |
| 222097_at | - | -0,00073712 | -0,461594427 | 0,460857306 | |
| 1555259_at | ZAK | -0,759588698 | -1,220210588 | 0,46062189 | |
| 232238_at | ASPM | -0,759588698 | -1,220210588 | 0,46062189 | |
| 241889_at | NFKBID | -0,759588698 | -1,220210588 | 0,46062189 | |
| 244336_at | - | -0,759588698 | -1,220210588 | 0,46062189 | |
| 234971_x_at | PLCD3 | 1,247462054 | 0,786897721 | 0,460564332 | |
| 225732_at | KLHDC5 | 2,817903185 | 2,357351836 | 0,460551349 | |
| 231792_at | MYLK2 | 0,917308734 | 0,456918297 | 0,460390437 | |
| 209451_at | TANK | 2,774453351 | 2,314300277 | 0,460153074 | |
| 223489_x_at | EXOSC3 | 2,879254447 | 2,419116683 | 0,460137764 | |
| 224725_at | MIB1 | 2,458724341 | 1,998633167 | 0,460091173 | |
| 235033_at | NPEPL1 | 1,063722698 | 0,603679647 | 0,460043051 | |
| 204598_at | UBOX5 | 1,473887017 | 1,013870809 | 0,460016208 | |
| 222403_at | MTCH2 | 2,325288363 | 1,865308541 | 0,459979822 | |
| 216038_x_at | DAXX | 2,750626925 | 2,290658654 | 0,45996827 | |
| 226181_at | TUBE1 | 2,822481847 | 2,362515214 | 0,459966633 | |
| 1553527_at | NLRP9 | -0,59053611 | -1,050406615 | 0,459870505 | |
| 1558400_x_at | ANKRD24 | -0,59053611 | -1,050406615 | 0,459870505 | |
| 209768_s_at | GP1BB /// SEPT5 | -0,59053611 | -1,050406615 | 0,459870505 | |
| 214607_at | PAK3 | -0,59053611 | -1,050406615 | 0,459870505 | |
| 221614_s_at | RPH3AL | -0,59053611 | -1,050406615 | 0,459870505 | |
| 229040_at | LOC100505746 | -0,59053611 | -1,050406615 | 0,459870505 | |
| 234712_at | LINC00470 | -0,59053611 | -1,050406615 | 0,459870505 | |
| 235870_at | HS2ST1 /// LOC3 | -0,59053611 | -1,050406615 | 0,459870505 | |
| 238094_at | - | -0,59053611 | -1,050406615 | 0,459870505 | |
| 241846_at | - | -0,59053611 | -1,050406615 | 0,459870505 | |
| 242266_x_at | - | -0,59053611 | -1,050406615 | 0,459870505 | |
| 242398_x_at | - | -0,59053611 | -1,050406615 | 0,459870505 | |
| 225410_at | COA5 | 2,525769574 | 2,066015817 | 0,459753757 | |
| 1552309_a_at | NEXN | 0,682279745 | 0,222599095 | 0,459680651 | |
| 206590_x_at | DRD2 | 0,682279745 | 0,222599095 | 0,459680651 | |
| 231505_s_at | SFXN4 | 0,682279745 | 0,222599095 | 0,459680651 | |
| 244324_at | C18orf54 | 2,014487247 | 1,55481039 | 0,459676857 | |
| 214976_at | RPL13 /// SNORD | 0,518867317 | 0,059218869 | 0,459648448 | |
| 227582_at | KLHDC9 | 0,518867317 | 0,059218869 | 0,459648448 | |
| 221893_s_at | ADCK2 | 2,084778276 | 1,6252386 | 0,459539676 | |
| 220553_s_at | PRPF39 | 3,475514766 | 3,016072743 | 0,459442022 | |
| 207526_s_at | IL1RL1 | 2,05655332 | 1,59747851 | 0,45907481 | |
| 212105_s_at | DHX9 | 2,549899391 | 2,091116676 | 0,458782715 | |
| 225362_at | FAM122B | 2,048758794 | 1,590454215 | 0,458304579 | |
| 234997_x_at | - | 3,117719377 | 2,659583735 | 0,458135641 | |
| 209772_s_at | CD24 | -0,221656139 | -0,679763839 | 0,458107701 | |
| 215841_at | GUCA1B | -0,221656139 | -0,679763839 | 0,458107701 | |
| 224965_at | GNG2 | 0,810733188 | 0,352836757 | 0,45789643 | |
| 206657_s_at | MYOD1 | -0,078265071 | -0,536087151 | 0,457822079 | |
| 213568_at | OSR2 | -0,078265071 | -0,536087151 | 0,457822079 | |

| | | | | | |
|---|---|---|---|---|---|
| 215186_at | TBC1D30 | -0,078265071 | -0,536087151 | 0,457822079 | |
| 229616_s_at | GRAMD2 | -0,078265071 | -0,536087151 | 0,457822079 | |
| 218485_s_at | SLC35C1 | 0,661985843 | 0,204310755 | 0,457675087 | |
| 229700_at | ZNF738 | 2,846658948 | 2,389070873 | 0,457588074 | |
| 1560325_at | - | -2,43257096 | -2,89013181 | 0,45756085 | |
| 1561590_a_at | LOC415056 | -2,43257096 | -2,89013181 | 0,45756085 | |
| 1561627_at | - | -2,43257096 | -2,89013181 | 0,45756085 | |
| 1561951_at | SLC5A12 | -2,43257096 | -2,89013181 | 0,45756085 | |
| 1562811_at | - | -2,43257096 | -2,89013181 | 0,45756085 | |
| 1566698_at | - | -2,43257096 | -2,89013181 | 0,45756085 | |
| 224833_at | ETS1 | -2,43257096 | -2,89013181 | 0,45756085 | |
| 236797_at | - | -2,43257096 | -2,89013181 | 0,45756085 | |
| 244820_at | - | -2,43257096 | -2,89013181 | 0,45756085 | |
| 218511_s_at | PNPO | 2,814842651 | 2,357351836 | 0,457490814 | |
| 1556704_s_at | LOC100133920 / | -0,033456564 | -0,490927335 | 0,457470771 | |
| 1560204_at | NT5DC4 | -0,033456564 | -0,490927335 | 0,457470771 | |
| 240943_at | - | -0,033456564 | -0,490927335 | 0,457470771 | |
| 211756_at | PTHLH | 2,2992452 | 1,841838775 | 0,457406426 | |
| 228700_at | CXorf38 | 2,2992452 | 1,841838775 | 0,457406426 | |
| 224797_at | ARRDC3 | 3,41414682 | 2,957221307 | 0,456925513 | |
| 1553919_at | C9orf62 | -0,655928373 | -1,112851844 | 0,456923471 | |
| 1554783_s_at | ARHGEF2 | -0,655928373 | -1,112851844 | 0,456923471 | |
| 1556163_a_at | IGSF3 | -0,655928373 | -1,112851844 | 0,456923471 | |
| 1556667_at | FONG | -0,655928373 | -1,112851844 | 0,456923471 | |
| 1556685_at | - | -0,655928373 | -1,112851844 | 0,456923471 | |
| 1564052_at | TREML4 | -0,655928373 | -1,112851844 | 0,456923471 | |
| 214600_at | TEAD1 | -0,655928373 | -1,112851844 | 0,456923471 | |
| 214851_at | HNF4A | -0,655928373 | -1,112851844 | 0,456923471 | |
| 217171_at | SMPD1 | -0,655928373 | -1,112851844 | 0,456923471 | |
| 220057_at | XAGE1A /// XAGI | -0,655928373 | -1,112851844 | 0,456923471 | |
| 238688_at | TPM1 | -0,655928373 | -1,112851844 | 0,456923471 | |
| 242459_at | - | -0,655928373 | -1,112851844 | 0,456923471 | |
| 235295_at | - | 1,406361373 | 0,949447784 | 0,456913589 | |
| 222490_at | POLR3E | 1,62481334 | 1,16809119 | 0,45672215 | |
| 213993_at | SPON1 | -2,779459409 | -3,236070201 | 0,456610792 | |
| 240160_x_at | - | -2,779459409 | -3,236070201 | 0,456610792 | |
| 1566471_at | - | 1,814495998 | 1,3579181 | 0,456577898 | |
| 213418_at | HSPA6 | 1,982112682 | 1,525647381 | 0,456465302 | |
| 1559438_at | C21orf58 | 0,767063567 | 0,310717132 | 0,456346435 | |
| 1560767_at | HCG22 | 0,767063567 | 0,310717132 | 0,456346435 | |
| 227559_at | NDUFAF4 | 2,26825514 | 1,811954245 | 0,456300896 | |
| 221141_x_at | EPN1 | 0,376672223 | -0,079522948 | 0,456195172 | |
| 233271_at | - | 0,376672223 | -0,079522948 | 0,456195172 | |
| 208260_at | AVPR1B | 0,792179257 | 0,336136178 | 0,456043079 | |
| 203427_at | ASF1A | 3,413135307 | 2,957221307 | 0,455914 | |
| 222488_s_at | DCTN4 | 3,986661073 | 3,530865925 | 0,455795148 | |
| 1554941_at | KLHL14 | -2,64318869 | -3,098858316 | 0,455669627 | |
| 1561236_at | - | -2,64318869 | -3,098858316 | 0,455669627 | |
| 1562645_at | LOC401176 | -2,64318869 | -3,098858316 | 0,455669627 | |
| 207246_at | ZFY | -2,64318869 | -3,098858316 | 0,455669627 | |
| 207852_at | CXCL5 | -2,64318869 | -3,098858316 | 0,455669627 | |
| 228706_s_at | CLDN23 | -2,64318869 | -3,098858316 | 0,455669627 | |
| 232514_at | KIF27 | -2,64318869 | -3,098858316 | 0,455669627 | |
| 244147_at | - | -2,64318869 | -3,098858316 | 0,455669627 | |
| 39248_at | AQP3 | 5,596442834 | 5,140821732 | 0,455621103 | |
| 202221_s_at | EP300 | 2,38635068 | 1,930732138 | 0,455618542 | |

| | | | | | |
|---|---|---|---|---|---|
| 203283_s_at | HS2ST1 /// LOC3 | 3,497632487 | 3,042049843 | 0,455582643 | |
| 1553928_at | ELMOD2 | 1,897847661 | 1,442269454 | 0,455578207 | |
| 1555009_a_at | SYNJ2 | 0,171847695 | -0,283667828 | 0,455515523 | |
| 207277_at | CD209 | 0,171847695 | -0,283667828 | 0,455515523 | |
| 217596_at | UPF3A | 0,171847695 | -0,283667828 | 0,455515523 | |
| 229397_s_at | ARHGAP35 | 0,171847695 | -0,283667828 | 0,455515523 | |
| 201363_s_at | IVNS1ABP | 2,776027875 | 2,320681318 | 0,455346557 | |
| 213835_x_at | GTPBP3 | 3,450096885 | 2,994948944 | 0,45514794 | |
| 230031_at | HSPA5 | 1,758252436 | 1,303116084 | 0,455136352 | |
| 1316_at | THRA | 0,015503229 | -0,439564623 | 0,455067852 | |
| 1557532_at | - | 0,888485433 | 0,433557298 | 0,454928135 | |
| 224549_x_at | - | 0,888485433 | 0,433557298 | 0,454928135 | |
| 230339_at | - | 0,888485433 | 0,433557298 | 0,454928135 | |
| 231719_at | TOR1AIP2 | 0,888485433 | 0,433557298 | 0,454928135 | |
| 213168_at | SP3 | 4,431847465 | 3,977034308 | 0,454813157 | |
| 37802_r_at | FAM63B | -2,425712529 | -2,880484503 | 0,454771973 | |
| 229746_x_at | LOC100505876 | 2,466546243 | 2,011838132 | 0,454708111 | |
| 204843_s_at | PRKAR2A | 0,911589958 | 0,456918297 | 0,454671661 | |
| 201025_at | EIF5B | 3,579258128 | 3,124599694 | 0,454658433 | |
| 226990_at | CAPRIN1 | 3,869867201 | 3,415371412 | 0,454495789 | |
| 1558692_at | C1orf85 | 0,533835206 | 0,079428135 | 0,454407071 | |
| 209818_s_at | HABP4 | 0,533835206 | 0,079428135 | 0,454407071 | |
| 1552803_a_at | STMN1 | -2,236765673 | -2,690860744 | 0,454095071 | |
| 1560429_at | - | -2,236765673 | -2,690860744 | 0,454095071 | |
| 1569076_a_at | ZNF836 | -2,236765673 | -2,690860744 | 0,454095071 | |
| 204677_at | CDH5 | -2,236765673 | -2,690860744 | 0,454095071 | |
| 230195_at | LOC100131138 | -2,236765673 | -2,690860744 | 0,454095071 | |
| 233760_at | UTP11L | -2,236765673 | -2,690860744 | 0,454095071 | |
| 234348_at | - | -2,236765673 | -2,690860744 | 0,454095071 | |
| 237745_at | TSC22D1-AS1 | -2,236765673 | -2,690860744 | 0,454095071 | |
| 238306_at | - | -2,236765673 | -2,690860744 | 0,454095071 | |
| 204756_at | MAP2K5 | 1,414471577 | 0,960386719 | 0,454084858 | |
| 221150_at | MEPE | -0,112808516 | -0,566887459 | 0,454078943 | |
| 236574_at | - | -0,112808516 | -0,566887459 | 0,454078943 | |
| 238795_at | FAM208B | -0,112808516 | -0,566887459 | 0,454078943 | |
| 242885_at | - | -0,112808516 | -0,566887459 | 0,454078943 | |
| 220682_s_at | - | 0,317354302 | -0,136643983 | 0,453998285 | |
| 208653_s_at | CD164 | 4,916735717 | 4,462889331 | 0,453846387 | |
| 220849_at | LOC79999 | -0,496991414 | -0,950686014 | 0,453694601 | |
| 222842_at | EIF2C4 | -0,496991414 | -0,950686014 | 0,453694601 | |
| 228132_at | ABLIM2 | -0,496991414 | -0,950686014 | 0,453694601 | |
| 228735_s_at | PANK2 | -0,496991414 | -0,950686014 | 0,453694601 | |
| 229424_s_at | ARHGAP27 | -0,496991414 | -0,950686014 | 0,453694601 | |
| 234450_at | PROKR2 | -0,496991414 | -0,950686014 | 0,453694601 | |
| 243633_at | - | -0,496991414 | -0,950686014 | 0,453694601 | |
| 214829_at | AASS | 0,351548291 | -0,102100538 | 0,453648829 | |
| 229250_at | TPCN2 | 0,351548291 | -0,102100538 | 0,453648829 | |
| 235202_x_at | IKBIP | 1,177702228 | 0,724100169 | 0,453602058 | |
| 233068_at | - | 1,021868205 | 0,568365095 | 0,45350311 | |
| 1561361_at | ZNF660 | -1,2845686 | -1,738067356 | 0,453498756 | |
| 206017_at | KIAA0319 | -1,2845686 | -1,738067356 | 0,453498756 | |
| 206759_at | FCER2 | -1,2845686 | -1,738067356 | 0,453498756 | |
| 207138_at | PHF2 | -1,2845686 | -1,738067356 | 0,453498756 | |
| 207567_at | SLC13A2 | -1,2845686 | -1,738067356 | 0,453498756 | |
| 208593_x_at | CRHR1 | -1,2845686 | -1,738067356 | 0,453498756 | |
| 220774_at | DYM | -1,2845686 | -1,738067356 | 0,453498756 | |

| | | | | | |
|---|---|---|---|---|---|
| 227387_at | NSMCE4A | -1,2845686 | -1,738067356 | 0,453498756 | |
| 229745_x_at | DACT3 | -1,2845686 | -1,738067356 | 0,453498756 | |
| 231826_at | RALGAPA2 | -1,2845686 | -1,738067356 | 0,453498756 | |
| 232024_at | GIMAP2 | -1,2845686 | -1,738067356 | 0,453498756 | |
| 232409_x_at | FBXL16 | -1,2845686 | -1,738067356 | 0,453498756 | |
| 240021_at | - | -1,2845686 | -1,738067356 | 0,453498756 | |
| 240903_at | - | -1,2845686 | -1,738067356 | 0,453498756 | |
| 243754_at | - | -1,2845686 | -1,738067356 | 0,453498756 | |
| 214121_x_at | PDLIM7 | 1,493160564 | 1,039887232 | 0,453273333 | |
| 221604_s_at | PEX16 | 1,621313976 | 1,16809119 | 0,453222786 | |
| 228530_at | MZT1 | 0,87091093 | 0,417770464 | 0,453140466 | |
| 228497_at | SLC22A15 | 0,67554678 | 0,222599095 | 0,452947685 | |
| 1553914_at | MGC34800 | -1,089016378 | -1,541908042 | 0,452891664 | |
| 1559567_at | PRDM11 | -1,089016378 | -1,541908042 | 0,452891664 | |
| 1568849_at | LINC00165 | -1,089016378 | -1,541908042 | 0,452891664 | |
| 216280_s_at | DICER1 | -1,089016378 | -1,541908042 | 0,452891664 | |
| 222031_at | LOC100506453 / | -1,089016378 | -1,541908042 | 0,452891664 | |
| 226645_at | KLF2 | -1,089016378 | -1,541908042 | 0,452891664 | |
| 233053_at | - | -1,089016378 | -1,541908042 | 0,452891664 | |
| 234223_at | - | -1,089016378 | -1,541908042 | 0,452891664 | |
| 234460_at | Ndufaf4 | -1,089016378 | -1,541908042 | 0,452891664 | |
| 237726_at | - | -1,089016378 | -1,541908042 | 0,452891664 | |
| 205263_at | BCL10 | 3,479385765 | 3,026519779 | 0,452865985 | |
| 219919_s_at | SSH3 | 0,103263601 | -0,34958478 | 0,452848382 | |
| 220070_at | KDM8 | 0,103263601 | -0,34958478 | 0,452848382 | |
| 1552811_at | WFIKKN1 | 0,702292136 | 0,249604176 | 0,45268796 | |
| 1560081_at | LOC100505648 | -0,366776884 | -0,819238336 | 0,452461452 | |
| 201615_x_at | CALD1 | -0,366776884 | -0,819238336 | 0,452461452 | |
| 205192_at | MAP3K14 | -0,366776884 | -0,819238336 | 0,452461452 | |
| 208372_s_at | LIMK1 | -0,366776884 | -0,819238336 | 0,452461452 | |
| 224355_s_at | MS4A8B | -0,366776884 | -0,819238336 | 0,452461452 | |
| 244011_at | PPM1K | -0,366776884 | -0,819238336 | 0,452461452 | |
| 204095_s_at | ELL | 0,480753764 | 0,028364014 | 0,452389749 | |
| 222335_at | - | 1,940595304 | 1,488344553 | 0,452250751 | |
| 225680_at | LRWD1 /// MIR44 | 2,227406889 | 1,775255987 | 0,452150902 | |
| 238535_at | CEP152 | 0,984222186 | 0,532164371 | 0,452057815 | |
| 209553_at | LOC100505729 / | 1,767779954 | 1,315948728 | 0,451831226 | |
| 226686_at | CISD2 | 3,340506684 | 2,888726553 | 0,451780131 | |
| 218444_at | ALG12 | 0,255492549 | -0,19610998 | 0,451602529 | |
| 225509_at | SAP30L | 1,542099044 | 1,090553537 | 0,451545506 | |
| 227184_at | PTAFR | 0,82905153 | 0,377530647 | 0,451520884 | |
| 201319_at | MYL12A | 5,666452598 | 5,215058591 | 0,451394007 | |
| 213747_at | - | 0,291166226 | -0,160136748 | 0,451302974 | |
| 229914_at | FLJ38717 | 0,291166226 | -0,160136748 | 0,451302974 | |
| 232540_x_at | - | 0,291166226 | -0,160136748 | 0,451302974 | |
| 239196_at | ANKRD22 | 0,291166226 | -0,160136748 | 0,451302974 | |
| 218331_s_at | FAM208B | 4,468825568 | 4,017530898 | 0,45129467 | |
| 225058_at | GPR108 | 1,987559146 | 1,536652708 | 0,450906439 | |
| 211642_at | - | -2,823166348 | -3,273861381 | 0,450695033 | |
| 225642_at | KTI12 /// TXNDC | 2,310153708 | 1,859476797 | 0,450676911 | |
| 1554115_at | C5orf58 | -0,926850548 | -1,377419394 | 0,450568846 | |
| 1560724_at | - | -0,926850548 | -1,377419394 | 0,450568846 | |
| 211312_s_at | WISP1 | -0,926850548 | -1,377419394 | 0,450568846 | |
| 215027_at | RAPGEF3 | -0,926850548 | -1,377419394 | 0,450568846 | |
| 217648_at | - | -0,926850548 | -1,377419394 | 0,450568846 | |
| 223395_at | ABI3BP | -0,926850548 | -1,377419394 | 0,450568846 | |

| | | | | | |
|---|---|---|---|---|---|
| 238241_at | LOC100144602 | -0,926850548 | -1,377419394 | 0,450568846 | |
| 242818_x_at | - | -0,926850548 | -1,377419394 | 0,450568846 | |
| 243071_at | - | -0,926850548 | -1,377419394 | 0,450568846 | |
| 202976_s_at | RHOBTB3 | 5,192337655 | 4,741799874 | 0,450537782 | |
| 243196_s_at | TRAFD1 | 0,945567441 | 0,495031851 | 0,450535591 | |
| 37079_at | NUS1P3 | 1,538392922 | 1,088062085 | 0,450330837 | |
| 212525_s_at | H2AFX | 1,187200746 | 0,73688026 | 0,450320486 | |
| 1563105_at | - | 0,425644434 | -0,024572586 | 0,45021702 | |
| 203217_s_at | ST3GAL5 | 0,425644434 | -0,024572586 | 0,45021702 | |
| 240496_at | - | 0,425644434 | -0,024572586 | 0,45021702 | |
| 213350_at | RPS11 | 3,498586477 | 3,048471686 | 0,450114791 | |
| 205221_at | HGD | -0,196756757 | -0,646746079 | 0,449989321 | |
| 206098_at | ZBTB6 | -0,196756757 | -0,646746079 | 0,449989321 | |
| 208363_s_at | INPP4A | -0,196756757 | -0,646746079 | 0,449989321 | |
| 217193_x_at | - | -0,196756757 | -0,646746079 | 0,449989321 | |
| 222945_x_at | OLAH | -0,196756757 | -0,646746079 | 0,449989321 | |
| 228261_at | MIB2 | -0,196756757 | -0,646746079 | 0,449989321 | |
| 229315_at | - | -0,196756757 | -0,646746079 | 0,449989321 | |
| 234668_at | ALG13 | -0,196756757 | -0,646746079 | 0,449989321 | |
| 1553315_at | SLFNL1 | -2,504600601 | -2,954576755 | 0,449976154 | |
| 1555195_at | FBXO36 | -2,504600601 | -2,954576755 | 0,449976154 | |
| 1555284_at | ALS2 | -2,504600601 | -2,954576755 | 0,449976154 | |
| 1555533_at | QRFPR | -2,504600601 | -2,954576755 | 0,449976154 | |
| 1557520_a_at | - | -2,504600601 | -2,954576755 | 0,449976154 | |
| 1570204_at | ZBED3-AS1 | -2,504600601 | -2,954576755 | 0,449976154 | |
| 205764_at | CSNK1A1 | -2,504600601 | -2,954576755 | 0,449976154 | |
| 224242_at | GALP | -2,504600601 | -2,954576755 | 0,449976154 | |
| 224276_at | ZNF33A | -2,504600601 | -2,954576755 | 0,449976154 | |
| 234163_at | UBE3A | -2,504600601 | -2,954576755 | 0,449976154 | |
| 237395_at | CYP4Z1 | -2,504600601 | -2,954576755 | 0,449976154 | |
| 239503_at | C10orf114 | -2,504600601 | -2,954576755 | 0,449976154 | |
| 241245_at | SRSF4 | -2,504600601 | -2,954576755 | 0,449976154 | |
| 243925_at | - | -2,504600601 | -2,954576755 | 0,449976154 | |
| 226746_s_at | UBE4B | 0,457392764 | 0,007421914 | 0,449970851 | |
| 238647_at | C14orf28 | 0,457392764 | 0,007421914 | 0,449970851 | |
| 207416_s_at | NFATC3 | 1,880387527 | 1,43051703 | 0,449870497 | |
| 1556579_s_at | IGSF10 | 0,03125738 | -0,41858459 | 0,44984197 | |
| 1567257_at | OR1J2 | 0,03125738 | -0,41858459 | 0,44984197 | |
| 202473_x_at | HCFC1 | 0,03125738 | -0,41858459 | 0,44984197 | |
| 209012_at | TRIO | 0,03125738 | -0,41858459 | 0,44984197 | |
| 213432_at | MUC5B | 0,03125738 | -0,41858459 | 0,44984197 | |
| 228494_at | PPP1R9A | 0,03125738 | -0,41858459 | 0,44984197 | |
| 231710_at | CAPS | 0,03125738 | -0,41858459 | 0,44984197 | |
| 232225_at | - | 0,03125738 | -0,41858459 | 0,44984197 | |
| 244018_at | - | 0,03125738 | -0,41858459 | 0,44984197 | |
| 207943_x_at | PLAGL1 | 3,872077355 | 3,42231092 | 0,449766434 | |
| 225810_at | MTMR10 | 1,073999308 | 0,624460759 | 0,449538549 | |
| 216713_at | KRIT1 | 2,852640442 | 2,403170156 | 0,449470286 | |
| 235348_at | ABHD13 | 1,504602299 | 1,055274826 | 0,449327473 | |
| 1555465_at | MCOLN2 | 0,57782909 | 0,128747141 | 0,44908195 | |
| 228594_at | NADKD1 | 2,713311265 | 2,264418116 | 0,448893149 | |
| 218430_s_at | RFX7 | 1,578645857 | 1,129843209 | 0,448802648 | |
| 224987_at | C6orf89 | 1,751865623 | 1,303116084 | 0,448749538 | |
| 1557801_x_at | C11orf31 | 0,228146222 | -0,220592223 | 0,448738445 | |
| 220665_at | LUZP4 | 0,228146222 | -0,220592223 | 0,448738445 | |
| 205393_s_at | CHEK1 | 2,825526235 | 2,376875125 | 0,44865111 | |

| | | | | | |
|---|---|---|---|---|---|
| 1557289_s_at | GTF2IRD2 /// GT | -1,763254337 | -2,211750327 | 0,44849599 | |
| 1561880_a_at | SIGLEC16 | -1,763254337 | -2,211750327 | 0,44849599 | |
| 1562865_at | - | -1,763254337 | -2,211750327 | 0,44849599 | |
| 204124_at | SLC34A2 | -1,763254337 | -2,211750327 | 0,44849599 | |
| 208281_x_at | DAZ1 /// DAZ2 /// | -1,763254337 | -2,211750327 | 0,44849599 | |
| 211151_x_at | GH1 | -1,763254337 | -2,211750327 | 0,44849599 | |
| 213950_s_at | PPP3CC | -1,763254337 | -2,211750327 | 0,44849599 | |
| 222456_s_at | LIMA1 | -1,763254337 | -2,211750327 | 0,44849599 | |
| 234599_at | - | -1,763254337 | -2,211750327 | 0,44849599 | |
| 236603_at | - | -1,763254337 | -2,211750327 | 0,44849599 | |
| 241382_at | PCP4L1 | -1,763254337 | -2,211750327 | 0,44849599 | |
| 215096_s_at | ESD | 5,260285448 | 4,811797157 | 0,448488291 | |
| 218598_at | RINT1 | 2,701760055 | 2,253342112 | 0,448417943 | |
| 1552408_at | ODF4 | -1,71423189 | -2,162639771 | 0,448407882 | |
| 1556573_s_at | LOC286178 | -1,71423189 | -2,162639771 | 0,448407882 | |
| 1566786_at | - | -1,71423189 | -2,162639771 | 0,448407882 | |
| 205876_at | LIFR | -1,71423189 | -2,162639771 | 0,448407882 | |
| 213855_s_at | LIPE | -1,71423189 | -2,162639771 | 0,448407882 | |
| 216904_at | LOC100652958 | -1,71423189 | -2,162639771 | 0,448407882 | |
| 228893_at | - | -1,71423189 | -2,162639771 | 0,448407882 | |
| 231141_at | LOC100506983 | -1,71423189 | -2,162639771 | 0,448407882 | |
| 232982_at | SYNRG | -1,71423189 | -2,162639771 | 0,448407882 | |
| 237454_at | - | -1,71423189 | -2,162639771 | 0,448407882 | |
| 239182_at | HOXD-AS1 | -1,71423189 | -2,162639771 | 0,448407882 | |
| 240110_at | HMGCS2 | -1,71423189 | -2,162639771 | 0,448407882 | |
| 240716_at | TTC23 | -1,71423189 | -2,162639771 | 0,448407882 | |
| 243398_at | - | -1,71423189 | -2,162639771 | 0,448407882 | |
| 1563005_at | - | 0,715481152 | 0,26733076 | 0,448150392 | |
| 212046_x_at | MAPK3 | 2,59519831 | 2,147243253 | 0,447955057 | |
| 216519_s_at | PROSC | -0,706943342 | -1,154897679 | 0,447954337 | |
| 231621_at | MPZL1 | -0,706943342 | -1,154897679 | 0,447954337 | |
| 242203_at | - | -0,706943342 | -1,154897679 | 0,447954337 | |
| 242510_at | - | -0,706943342 | -1,154897679 | 0,447954337 | |
| 219261_at | C7orf26 | 2,156646206 | 1,708698295 | 0,447947911 | |
| 220955_x_at | RAB23 | 2,501229299 | 2,053299695 | 0,447929604 | |
| 230805_at | - | 2,038300149 | 1,590454215 | 0,447845934 | |
| 1553213_a_at | KRT78 | -1,838812296 | -2,286583451 | 0,447771154 | |
| 1555729_a_at | CD209 | -1,838812296 | -2,286583451 | 0,447771154 | |
| 1568375_at | DEFB124 | -1,838812296 | -2,286583451 | 0,447771154 | |
| 203863_at | ACTN2 | -1,838812296 | -2,286583451 | 0,447771154 | |
| 206265_s_at | GPLD1 | -1,838812296 | -2,286583451 | 0,447771154 | |
| 211398_at | FGFR2 | -1,838812296 | -2,286583451 | 0,447771154 | |
| 215945_s_at | TRIM2 | -1,838812296 | -2,286583451 | 0,447771154 | |
| 216496_s_at | - | -1,838812296 | -2,286583451 | 0,447771154 | |
| 216691_at | - | -1,838812296 | -2,286583451 | 0,447771154 | |
| 220268_at | C11orf67 | -1,838812296 | -2,286583451 | 0,447771154 | |
| 222822_s_at | MMRN2 | -1,838812296 | -2,286583451 | 0,447771154 | |
| 230299_s_at | WNT5B | -1,838812296 | -2,286583451 | 0,447771154 | |
| 231030_at | ZRANB2-AS1 | -1,838812296 | -2,286583451 | 0,447771154 | |
| 234638_at | - | -1,838812296 | -2,286583451 | 0,447771154 | |
| 236326_at | HDAC7 | -1,838812296 | -2,286583451 | 0,447771154 | |
| 243352_at | ALPK1 | -1,838812296 | -2,286583451 | 0,447771154 | |
| 244091_at | - | -1,838812296 | -2,286583451 | 0,447771154 | |
| 213167_s_at | SLC5A3 | 0,401366113 | -0,046302147 | 0,44766826 | |
| 221595_at | RBM48 | 0,401366113 | -0,046302147 | 0,44766826 | |
| 207405_s_at | RAD17 | 3,55745065 | 3,109937707 | 0,447512943 | |

| | | | | | |
|---|---|---|---|---|---|
| 1554377_a_at | CNTNAP4 | -1,639579028 | -2,086984744 | 0,447405715 | |
| 1554449_at | MIER3 | -1,639579028 | -2,086984744 | 0,447405715 | |
| 1562829_at | LOC339568 | -1,639579028 | -2,086984744 | 0,447405715 | |
| 207509_s_at | LAIR2 | -1,639579028 | -2,086984744 | 0,447405715 | |
| 207518_at | DGKE | -1,639579028 | -2,086984744 | 0,447405715 | |
| 209074_s_at | FAM107A /// LOC | -1,639579028 | -2,086984744 | 0,447405715 | |
| 220362_at | PSORS1C1 | -1,639579028 | -2,086984744 | 0,447405715 | |
| 220534_at | TRIM48 | -1,639579028 | -2,086984744 | 0,447405715 | |
| 225720_at | SYNPO2 | -1,639579028 | -2,086984744 | 0,447405715 | |
| 231597_x_at | - | -1,639579028 | -2,086984744 | 0,447405715 | |
| 231685_at | - | -1,639579028 | -2,086984744 | 0,447405715 | |
| 234129_at | ARHGEF12 | -1,639579028 | -2,086984744 | 0,447405715 | |
| 237196_at | LOC100506558 / | -1,639579028 | -2,086984744 | 0,447405715 | |
| 242171_at | - | -1,639579028 | -2,086984744 | 0,447405715 | |
| 243515_at | - | -1,639579028 | -2,086984744 | 0,447405715 | |
| 243582_at | SH3RF2 | -1,639579028 | -2,086984744 | 0,447405715 | |
| 215134_at | PI4K2A | 1,805273023 | 1,3579181 | 0,447354923 | |
| 213746_s_at | FLNA | 3,120200849 | 2,672945617 | 0,447255232 | |
| 209207_s_at | SEC22B | 3,191547726 | 2,744302548 | 0,447245178 | |
| 1552917_at | IL29 | -1,047650486 | -1,494668682 | 0,447018196 | |
| 1557789_at | - | -1,047650486 | -1,494668682 | 0,447018196 | |
| 205404_at | HSD11B1 | -1,047650486 | -1,494668682 | 0,447018196 | |
| 206417_at | CNGA1 | -1,047650486 | -1,494668682 | 0,447018196 | |
| 206797_at | NAT2 | -1,047650486 | -1,494668682 | 0,447018196 | |
| 210550_s_at | RASGRF1 | -1,047650486 | -1,494668682 | 0,447018196 | |
| 217009_at | PGK2 | -1,047650486 | -1,494668682 | 0,447018196 | |
| 220790_s_at | MS4A5 | -1,047650486 | -1,494668682 | 0,447018196 | |
| 232416_at | CELF5 | -1,047650486 | -1,494668682 | 0,447018196 | |
| 233555_s_at | SULF2 | -1,047650486 | -1,494668682 | 0,447018196 | |
| 237039_at | LOC100506088 | -1,047650486 | -1,494668682 | 0,447018196 | |
| 241769_at | - | -1,047650486 | -1,494668682 | 0,447018196 | |
| 1553863_at | WDFY4 | -1,888254346 | -2,335104118 | 0,446849772 | |
| 1559270_at | ZFHX4 | -1,888254346 | -2,335104118 | 0,446849772 | |
| 205958_x_at | CSHL1 | -1,888254346 | -2,335104118 | 0,446849772 | |
| 207454_at | GRIK3 | -1,888254346 | -2,335104118 | 0,446849772 | |
| 208410_x_at | AMELX | -1,888254346 | -2,335104118 | 0,446849772 | |
| 215709_at | LOC100653079 / | -1,888254346 | -2,335104118 | 0,446849772 | |
| 230900_at | CCDC110 | -1,888254346 | -2,335104118 | 0,446849772 | |
| 238600_at | JAKMIP1 | -1,888254346 | -2,335104118 | 0,446849772 | |
| 239733_at | DYDC2 | -1,888254346 | -2,335104118 | 0,446849772 | |
| 240646_at | GIMAP8 | -1,888254346 | -2,335104118 | 0,446849772 | |
| 244469_at | - | -1,888254346 | -2,335104118 | 0,446849772 | |
| 244526_at | RASGRP3 | -1,888254346 | -2,335104118 | 0,446849772 | |
| 226112_at | SGCB | 0,555999837 | 0,109221392 | 0,446778445 | |
| 223168_at | RHOU | 1,783520648 | 1,337086025 | 0,446434623 | |
| 227353_at | TMC8 | -0,089689931 | -0,536087151 | 0,446397219 | |
| 242074_at | - | -0,089689931 | -0,536087151 | 0,446397219 | |
| 1561371_at | FLJ39080 | -1,591296941 | -2,037551727 | 0,446254785 | |
| 1570196_at | - | -1,591296941 | -2,037551727 | 0,446254785 | |
| 1570270_at | - | -1,591296941 | -2,037551727 | 0,446254785 | |
| 202952_s_at | ADAM12 | -1,591296941 | -2,037551727 | 0,446254785 | |
| 208307_at | RBMY1A1 /// RBI | -1,591296941 | -2,037551727 | 0,446254785 | |
| 208385_at | NR2E3 | -1,591296941 | -2,037551727 | 0,446254785 | |
| 211362_s_at | SERPINB13 | -1,591296941 | -2,037551727 | 0,446254785 | |
| 211457_at | GABARAPL3 | -1,591296941 | -2,037551727 | 0,446254785 | |
| 214392_at | IRGC | -1,591296941 | -2,037551727 | 0,446254785 | |

| | | | | | |
|---|---|---|---|---|---|
| 214466_at | GJA5 | -1,591296941 | -2,037551727 | 0,446254785 | |
| 215183_at | - | -1,591296941 | -2,037551727 | 0,446254785 | |
| 220544_at | TSKS | -1,591296941 | -2,037551727 | 0,446254785 | |
| 220834_at | MS4A12 | -1,591296941 | -2,037551727 | 0,446254785 | |
| 229188_s_at | ZNRF2 | -1,591296941 | -2,037551727 | 0,446254785 | |
| 231433_at | LOC100506929 | -1,591296941 | -2,037551727 | 0,446254785 | |
| 232879_at | CRTC3 | -1,591296941 | -2,037551727 | 0,446254785 | |
| 233840_at | LOC100130950 | -1,591296941 | -2,037551727 | 0,446254785 | |
| 234361_at | CREB3L3 | -1,591296941 | -2,037551727 | 0,446254785 | |
| 239270_at | PLCXD3 | -1,591296941 | -2,037551727 | 0,446254785 | |
| 239402_at | - | -1,591296941 | -2,037551727 | 0,446254785 | |
| 240800_x_at | - | -1,591296941 | -2,037551727 | 0,446254785 | |
| 240844_at | - | -1,591296941 | -2,037551727 | 0,446254785 | |
| 231698_at | FLJ36848 | 0,123193849 | -0,322870281 | 0,44606413 | |
| 240934_at | - | 0,695652136 | 0,249604176 | 0,446047961 | |
| 231821_x_at | FLJ14186 /// LOC | 1,422536444 | 0,976641161 | 0,445895283 | |
| 1557228_at | EHBP1L1 | 0,798390451 | 0,352836757 | 0,445553694 | |
| 1552602_at | CACNG5 | -0,234278768 | -0,679763839 | 0,445485072 | |
| 1553769_at | SLAMF9 | -0,234278768 | -0,679763839 | 0,445485072 | |
| 1557731_at | LOC400620 | -0,234278768 | -0,679763839 | 0,445485072 | |
| 208457_at | GABRD | -0,234278768 | -0,679763839 | 0,445485072 | |
| 216340_s_at | CYP2A7P1 | -0,234278768 | -0,679763839 | 0,445485072 | |
| 219155_at | PITPNC1 | -0,234278768 | -0,679763839 | 0,445485072 | |
| 209575_at | IL10RB | 2,742596529 | 2,297144887 | 0,445451642 | |
| 1556405_s_at | LOC374890 | -0,467091869 | -0,912331589 | 0,44523972 | |
| 218621_at | HEMK1 | -0,467091869 | -0,912331589 | 0,44523972 | |
| 233137_at | - | -0,467091869 | -0,912331589 | 0,44523972 | |
| 201320_at | SMARCC2 | 1,835789711 | 1,390636111 | 0,4451536 | |
| 1553402_a_at | HFE | -2,551294263 | -2,996335703 | 0,44504144 | |
| 1553454_at | RPTN | -2,551294263 | -2,996335703 | 0,44504144 | |
| 1557674_s_at | EFCAB2 | -2,551294263 | -2,996335703 | 0,44504144 | |
| 1570076_at | - | -2,551294263 | -2,996335703 | 0,44504144 | |
| 203992_s_at | KDM6A | -2,551294263 | -2,996335703 | 0,44504144 | |
| 220812_s_at | HHLA2 | -2,551294263 | -2,996335703 | 0,44504144 | |
| 231993_at | ITGBL1 | -2,551294263 | -2,996335703 | 0,44504144 | |
| 234136_at | - | -2,551294263 | -2,996335703 | 0,44504144 | |
| 235075_at | DSG3 | -2,551294263 | -2,996335703 | 0,44504144 | |
| 242483_at | - | -2,551294263 | -2,996335703 | 0,44504144 | |
| 1570230_at | - | -0,285837228 | -0,730778808 | 0,44494158 | |
| 206890_at | IL12RB1 | -0,285837228 | -0,730778808 | 0,44494158 | |
| 207532_at | CRYGD | -0,285837228 | -0,730778808 | 0,44494158 | |
| 243587_x_at | - | -0,285837228 | -0,730778808 | 0,44494158 | |
| 241814_at | - | -2,866296344 | -3,311072652 | 0,444776308 | |
| 227350_at | HELLS | 3,554701391 | 3,109937707 | 0,444763684 | |
| 1553995_a_at | NT5E | -1,964176657 | -2,408705138 | 0,444528481 | |
| 1557907_x_at | MUC12 | -1,964176657 | -2,408705138 | 0,444528481 | |
| 1559982_s_at | AKR1E2 | -1,964176657 | -2,408705138 | 0,444528481 | |
| 1560220_a_at | CBY1 | -1,964176657 | -2,408705138 | 0,444528481 | |
| 1560941_a_at | SACS-AS1 | -1,964176657 | -2,408705138 | 0,444528481 | |
| 1567252_at | OR10D3 | -1,964176657 | -2,408705138 | 0,444528481 | |
| 1569062_s_at | IQGAP3 | -1,964176657 | -2,408705138 | 0,444528481 | |
| 207592_s_at | HCN2 | -1,964176657 | -2,408705138 | 0,444528481 | |
| 212985_at | APBB2 | -1,964176657 | -2,408705138 | 0,444528481 | |
| 217463_s_at | C11orf9 | -1,964176657 | -2,408705138 | 0,444528481 | |
| 219912_s_at | - | -1,964176657 | -2,408705138 | 0,444528481 | |
| 232482_at | OR51E2 | -1,964176657 | -2,408705138 | 0,444528481 | |

| | | | | | |
|---|---|---|---|---|---|
| 233972_s_at | FEZF2 | -1,964176657 | -2,408705138 | 0,444528481 | |
| 234189_at | - | -1,964176657 | -2,408705138 | 0,444528481 | |
| 235712_at | GAS5-AS1 | -1,964176657 | -2,408705138 | 0,444528481 | |
| 237050_at | - | -1,964176657 | -2,408705138 | 0,444528481 | |
| 237484_at | C12orf69 | -1,964176657 | -2,408705138 | 0,444528481 | |
| 237575_at | - | -1,964176657 | -2,408705138 | 0,444528481 | |
| 244856_at | - | -1,964176657 | -2,408705138 | 0,444528481 | |
| 207193_at | AGRP | 0,503742507 | 0,059218869 | 0,444523639 | |
| 210424_s_at | GOLGA8A /// GO | 0,503742507 | 0,059218869 | 0,444523639 | |
| 218124_at | RETSAT | 1,04816921 | 0,603679647 | 0,444489564 | |
| 229984_at | DTWD1 | 1,04816921 | 0,603679647 | 0,444489564 | |
| 226478_at | TM7SF3 | 1,534677255 | 1,090553537 | 0,444123718 | |
| 233518_at | - | 0,648296226 | 0,204310755 | 0,44398547 | |
| 1556970_at | - | -0,888496123 | -1,332017329 | 0,443521207 | |
| 205057_s_at | IDUA | -0,888496123 | -1,332017329 | 0,443521207 | |
| 207279_s_at | NEBL | -0,888496123 | -1,332017329 | 0,443521207 | |
| 208545_x_at | MIR1257 /// TAF4 | -0,888496123 | -1,332017329 | 0,443521207 | |
| 219872_at | FAM198B | -0,888496123 | -1,332017329 | 0,443521207 | |
| 222128_at | NSUN6 | -0,888496123 | -1,332017329 | 0,443521207 | |
| 226358_at | APH1B | -0,888496123 | -1,332017329 | 0,443521207 | |
| 229795_at | - | -0,888496123 | -1,332017329 | 0,443521207 | |
| 236050_at | C11orf35 | -0,888496123 | -1,332017329 | 0,443521207 | |
| 236652_at | LOC149703 | -0,888496123 | -1,332017329 | 0,443521207 | |
| 242647_at | USP34 | -0,888496123 | -1,332017329 | 0,443521207 | |
| 1567060_at | OR8G1 | -1,518072576 | -1,961547147 | 0,443474572 | |
| 206975_at | LTA | -1,518072576 | -1,961547147 | 0,443474572 | |
| 207149_at | CDH12 | -1,518072576 | -1,961547147 | 0,443474572 | |
| 213964_x_at | C10orf95 /// LOC | -1,518072576 | -1,961547147 | 0,443474572 | |
| 214261_s_at | ADH6 | -1,518072576 | -1,961547147 | 0,443474572 | |
| 231732_at | SMPD3 | -1,518072576 | -1,961547147 | 0,443474572 | |
| 236627_at | - | -1,518072576 | -1,961547147 | 0,443474572 | |
| 236910_at | MRPL39 | -1,518072576 | -1,961547147 | 0,443474572 | |
| 237813_at | PCBP2 | -1,518072576 | -1,961547147 | 0,443474572 | |
| 238438_at | CNOT6L | -1,518072576 | -1,961547147 | 0,443474572 | |
| 238997_at | PSORS1C3 | -1,518072576 | -1,961547147 | 0,443474572 | |
| 212633_at | UFL1 | 2,922548342 | 2,479296438 | 0,443251904 | |
| 238057_at | USP45 | 1,912238072 | 1,469325098 | 0,442912974 | |
| 212634_at | UFL1 | 1,523472489 | 1,080561626 | 0,442910863 | |
| 1558630_at | - | 0,052199481 | -0,39061235 | 0,442811831 | |
| 226194_at | CHAMP1 | 3,245961699 | 2,803210557 | 0,442751142 | |
| 218840_s_at | NADSYN1 | 1,596577845 | 1,153866761 | 0,442711084 | |
| 1554448_at | - | -2,01371626 | -2,456406426 | 0,442690166 | |
| 1556985_at | - | -2,01371626 | -2,456406426 | 0,442690166 | |
| 1558337_at | - | -2,01371626 | -2,456406426 | 0,442690166 | |
| 1559133_at | - | -2,01371626 | -2,456406426 | 0,442690166 | |
| 1561578_s_at | SLC25A53 | -2,01371626 | -2,456406426 | 0,442690166 | |
| 1562719_at | - | -2,01371626 | -2,456406426 | 0,442690166 | |
| 1563341_at | - | -2,01371626 | -2,456406426 | 0,442690166 | |
| 207208_at | RBMXL2 | -2,01371626 | -2,456406426 | 0,442690166 | |
| 208136_s_at | MGC3771 | -2,01371626 | -2,456406426 | 0,442690166 | |
| 211369_at | - | -2,01371626 | -2,456406426 | 0,442690166 | |
| 215787_at | ACTA2 | -2,01371626 | -2,456406426 | 0,442690166 | |
| 216213_at | NEK1 | -2,01371626 | -2,456406426 | 0,442690166 | |
| 223887_at | GPR132 | -2,01371626 | -2,456406426 | 0,442690166 | |
| 244101_at | - | -2,01371626 | -2,456406426 | 0,442690166 | |
| 209756_s_at | MYCN | 0,246434561 | -0,19610998 | 0,442544541 | |

| | | | | | |
|---|---|---|---|---|---|
| 213635_s_at | SAFB | 0,246434561 | -0,19610998 | 0,442544541 | |
| 235791_x_at | CHD1 | 3,304956562 | 2,862725455 | 0,442231107 | |
| 1554578_at | ZNF70 | 0,417596971 | -0,024572586 | 0,442169557 | |
| 1566512_at | - | 0,417596971 | -0,024572586 | 0,442169557 | |
| 1567015_at | NFE2L2 | -0,172274514 | -0,614371577 | 0,442097063 | |
| 210407_at | PPM1A | -0,172274514 | -0,614371577 | 0,442097063 | |
| 224268_x_at | ZAN | -0,172274514 | -0,614371577 | 0,442097063 | |
| 238657_at | UBXN10 | -0,172274514 | -0,614371577 | 0,442097063 | |
| 242298_x_at | - | -0,172274514 | -0,614371577 | 0,442097063 | |
| 235363_at | - | 2,874852734 | 2,432926755 | 0,44192598 | |
| 228256_s_at | EPB41L4A | 3,880884266 | 3,439026336 | 0,44185793 | |
| 225659_at | SPOPL | 1,481627361 | 1,039887232 | 0,44174013 | |
| 210187_at | FKBP1A | 3,24026957 | 2,798646381 | 0,44162319 | |
| 213851_at | TMEM110 | 1,556829052 | 1,115234684 | 0,441594367 | |
| 1558816_at | ZNF664 | 0,735041319 | 0,293518836 | 0,441522484 | |
| 225231_at | CBL | 2,834621043 | 2,39311332 | 0,441507723 | |
| 203482_at | FAM178A | 3,097712429 | 2,656223838 | 0,441488591 | |
| 1558476_at | BEND5 | -1,173767127 | -1,615132408 | 0,441365281 | |
| 206795_at | F2RL2 | -1,173767127 | -1,615132408 | 0,441365281 | |
| 215140_at | - | -1,173767127 | -1,615132408 | 0,441365281 | |
| 231368_at | BOD1L2 | -1,173767127 | -1,615132408 | 0,441365281 | |
| 243054_at | - | -1,173767127 | -1,615132408 | 0,441365281 | |
| 243825_at | BCL6B | 0,859074518 | 0,417770464 | 0,441304055 | |
| 1557550_at | LOC148145 | -1,470833215 | -1,912089812 | 0,441256597 | |
| 1565939_at | C5orf22 | -1,470833215 | -1,912089812 | 0,441256597 | |
| 1566866_at | - | -1,470833215 | -1,912089812 | 0,441256597 | |
| 1567167_at | - | -1,470833215 | -1,912089812 | 0,441256597 | |
| 203393_at | HES1 | -1,470833215 | -1,912089812 | 0,441256597 | |
| 205253_at | PBX1 | -1,470833215 | -1,912089812 | 0,441256597 | |
| 207248_at | KCNA4 | -1,470833215 | -1,912089812 | 0,441256597 | |
| 223756_at | KANSL3 | -1,470833215 | -1,912089812 | 0,441256597 | |
| 232311_at | B2M | -1,470833215 | -1,912089812 | 0,441256597 | |
| 233450_at | - | -1,470833215 | -1,912089812 | 0,441256597 | |
| 236759_at | - | -1,470833215 | -1,912089812 | 0,441256597 | |
| 238664_s_at | MGC12916 | -1,470833215 | -1,912089812 | 0,441256597 | |
| 240115_at | LOC100286925 | -1,470833215 | -1,912089812 | 0,441256597 | |
| 1557034_s_at | LOC100134445 / | 0,97328325 | 0,532164371 | 0,441118879 | |
| 221943_x_at | RPL38 | 3,973683876 | 3,532699346 | 0,44098453 | |
| 1558409_at | - | -2,063149277 | -2,503677622 | 0,440528345 | |
| 1558882_at | HTATSF1P2 | -2,063149277 | -2,503677622 | 0,440528345 | |
| 1560337_at | LOC286184 | -2,063149277 | -2,503677622 | 0,440528345 | |
| 1564760_at | - | -2,063149277 | -2,503677622 | 0,440528345 | |
| 1569503_at | HEATR5B | -2,063149277 | -2,503677622 | 0,440528345 | |
| 210937_s_at | PDX1 | -2,063149277 | -2,503677622 | 0,440528345 | |
| 215627_at | - | -2,063149277 | -2,503677622 | 0,440528345 | |
| 215859_at | NCLN | -2,063149277 | -2,503677622 | 0,440528345 | |
| 224396_s_at | ASPN | -2,063149277 | -2,503677622 | 0,440528345 | |
| 237746_at | - | -2,063149277 | -2,503677622 | 0,440528345 | |
| 238966_at | CELF4 | -2,063149277 | -2,503677622 | 0,440528345 | |
| 239572_at | GJA3 | -2,063149277 | -2,503677622 | 0,440528345 | |
| 241622_at | PTPN2 | -2,063149277 | -2,503677622 | 0,440528345 | |
| 1558292_s_at | PIGW | 3,574742007 | 3,134292219 | 0,440449788 | |
| 222399_s_at | TM9SF3 | 5,201887051 | 4,761478085 | 0,440408966 | |
| 228600_x_at | FAM221A | 1,850809354 | 1,410714371 | 0,440094983 | |
| 1556912_at | GIT2 | -2,597496523 | -3,037500868 | 0,440004345 | |
| 1568856_at | NBR1 | -2,597496523 | -3,037500868 | 0,440004345 | |

| | | | | | |
|---|---|---|---|---|---|
| 214046_at | FUT9 | -2,597496523 | -3,037500868 | 0,440004345 | |
| 220655_at | TNIP3 | -2,597496523 | -3,037500868 | 0,440004345 | |
| 239520_at | - | -2,597496523 | -3,037500868 | 0,440004345 | |
| 241880_x_at | - | -2,597496523 | -3,037500868 | 0,440004345 | |
| 242003_at | ERICH1 | -2,597496523 | -3,037500868 | 0,440004345 | |
| 1556382_a_at | NAA15 | -0,67289328 | -1,112851844 | 0,439958564 | |
| 1556452_a_at | LOC283761 | -0,67289328 | -1,112851844 | 0,439958564 | |
| 1561202_at | - | -0,67289328 | -1,112851844 | 0,439958564 | |
| 1562826_at | - | -0,67289328 | -1,112851844 | 0,439958564 | |
| 1564314_at | LOC219690 | -0,67289328 | -1,112851844 | 0,439958564 | |
| 230119_at | - | -0,67289328 | -1,112851844 | 0,439958564 | |
| 230897_at | CCDC30 /// PPC | -0,67289328 | -1,112851844 | 0,439958564 | |
| 231266_at | LYPD4 | -0,67289328 | -1,112851844 | 0,439958564 | |
| 235102_x_at | SNORD3B-1 /// S | -0,67289328 | -1,112851844 | 0,439958564 | |
| 239190_at | VRK3 | -0,67289328 | -1,112851844 | 0,439958564 | |
| 224321_at | TMEFF2 | 2,030406107 | 1,590454215 | 0,439951892 | |
| 1555114_at | ATP6V0A2 | -0,325749314 | -0,765504029 | 0,439754715 | |
| 1557817_a_at | - | -0,325749314 | -0,765504029 | 0,439754715 | |
| 1560492_at | - | -0,325749314 | -0,765504029 | 0,439754715 | |
| 1563668_at | MORN1 | -0,325749314 | -0,765504029 | 0,439754715 | |
| 206923_at | PRKCA | -0,325749314 | -0,765504029 | 0,439754715 | |
| 233362_at | ZNF341 | -0,325749314 | -0,765504029 | 0,439754715 | |
| 244554_at | - | -0,325749314 | -0,765504029 | 0,439754715 | |
| 243511_at | - | 0,181384709 | -0,258114234 | 0,439498943 | |
| 203783_x_at | POLRMT | 0,359971644 | -0,079522948 | 0,439494592 | |
| 228861_at | CDS2 | 0,359971644 | -0,079522948 | 0,439494592 | |
| 229484_at | PPM1J | 0,359971644 | -0,079522948 | 0,439494592 | |
| 229253_at | THEM4 | 2,956271699 | 2,516830926 | 0,439440774 | |
| 1559245_at | - | 0,518867317 | 0,079428135 | 0,439439182 | |
| 201452_at | RHEB | 0,518867317 | 0,079428135 | 0,439439182 | |
| 231548_at | FOXO3 | 0,518867317 | 0,079428135 | 0,439439182 | |
| 206094_x_at | UGT1A1 /// UGT | 0,661985843 | 0,222599095 | 0,439386748 | |
| 221493_at | TSPYL1 | 4,744881223 | 4,305575871 | 0,439305352 | |
| 1554880_at | DKFZP434K028 | 0,020670649 | -0,41858459 | 0,439255239 | |
| 213672_at | MARS | 0,020670649 | -0,41858459 | 0,439255239 | |
| 215906_at | - | 0,020670649 | -0,41858459 | 0,439255239 | |
| 233325_at | SLC35D2 | 0,020670649 | -0,41858459 | 0,439255239 | |
| 219199_at | AFF4 | -0,022466681 | -0,461594427 | 0,439127746 | |
| 230885_at | SPG7 | -0,022466681 | -0,461594427 | 0,439127746 | |
| 235685_at | - | -0,022466681 | -0,461594427 | 0,439127746 | |
| 47105_at | DUS2L | 1,799091453 | 1,359984858 | 0,439106595 | |
| 202351_at | ITGAV | 3,319065909 | 2,880111483 | 0,438954426 | |
| 1552690_a_at | CACNA2D4 | -1,424023738 | -1,862647763 | 0,438624024 | |
| 1558418_at | - | -1,424023738 | -1,862647763 | 0,438624024 | |
| 1562821_a_at | DSCAM-AS1 | -1,424023738 | -1,862647763 | 0,438624024 | |
| 1563073_at | - | -1,424023738 | -1,862647763 | 0,438624024 | |
| 1566851_at | TRIM42 | -1,424023738 | -1,862647763 | 0,438624024 | |
| 215547_at | TSC22D2 | -1,424023738 | -1,862647763 | 0,438624024 | |
| 216144_at | - | -1,424023738 | -1,862647763 | 0,438624024 | |
| 218863_s_at | TNS1 | -1,424023738 | -1,862647763 | 0,438624024 | |
| 221091_at | INSL5 | -1,424023738 | -1,862647763 | 0,438624024 | |
| 224251_at | WDR96 | -1,424023738 | -1,862647763 | 0,438624024 | |
| 232379_at | SKIL | -1,424023738 | -1,862647763 | 0,438624024 | |
| 232484_at | - | -1,424023738 | -1,862647763 | 0,438624024 | |
| 232607_at | - | -1,424023738 | -1,862647763 | 0,438624024 | |
| 237655_at | - | -1,424023738 | -1,862647763 | 0,438624024 | |

| | | | | | |
|---|---|---|---|---|---|
| 242465_at | LOC100505592 | -1,424023738 | -1,862647763 | 0,438624024 | |
| 244013_at | - | -1,424023738 | -1,862647763 | 0,438624024 | |
| 244210_at | - | -1,424023738 | -1,862647763 | 0,438624024 | |
| 209708_at | MOXD1 | -0,512251684 | -0,950686014 | 0,43843433 | |
| 217995_at | SQRDL | -0,512251684 | -0,950686014 | 0,43843433 | |
| 234125_at | - | -0,512251684 | -0,950686014 | 0,43843433 | |
| 235269_at | FAM83F | -0,512251684 | -0,950686014 | 0,43843433 | |
| 238742_x_at | - | -0,512251684 | -0,950686014 | 0,43843433 | |
| 235767_x_at | PHAX | 2,396622324 | 1,958277216 | 0,438345107 | |
| 233936_s_at | GGNBP2 | 2,166283906 | 1,728030156 | 0,438253749 | |
| 219131_at | UBIAD1 | 2,035673598 | 1,59747851 | 0,438195088 | |
| 205072_s_at | XRCC4 | 1,129248302 | 0,691645685 | 0,437602617 | |
| 212572_at | STK38L | 0,917308734 | 0,479907041 | 0,437401693 | |
| 235103_at | MAN2A1 | 2,069451339 | 1,632095957 | 0,437355382 | |
| 232130_at | - | 2,708372067 | 2,271023131 | 0,437348936 | |
| 244842_x_at | - | 0,747935635 | 0,310717132 | 0,437218503 | |
| 240054_at | - | 0,613492853 | 0,176436073 | 0,43705678 | |
| 1563916_at | WDR11-AS1 | -0,986356909 | -1,423348341 | 0,436991433 | |
| 1568879_a_at | LAMA3 | -0,986356909 | -1,423348341 | 0,436991433 | |
| 204792_s_at | IFT140 | -0,986356909 | -1,423348341 | 0,436991433 | |
| 205833_s_at | PART1 | -0,986356909 | -1,423348341 | 0,436991433 | |
| 214156_at | MYRIP | -0,986356909 | -1,423348341 | 0,436991433 | |
| 217261_at | TTTY2 /// TTTY2 | -0,986356909 | -1,423348341 | 0,436991433 | |
| 229096_at | LIMS3 /// LIMS3-I | -0,986356909 | -1,423348341 | 0,436991433 | |
| 230614_at | - | -0,986356909 | -1,423348341 | 0,436991433 | |
| 233401_at | - | -0,986356909 | -1,423348341 | 0,436991433 | |
| 240670_at | - | -0,986356909 | -1,423348341 | 0,436991433 | |
| 213959_s_at | RPGRIP1L | 0,496120222 | 0,059218869 | 0,436901353 | |
| 229673_at | C14orf118 | 0,496120222 | 0,059218869 | 0,436901353 | |
| 221784_at | WIZ | 0,46522187 | 0,028364014 | 0,436857856 | |
| 236974_at | - | 0,46522187 | 0,028364014 | 0,436857856 | |
| 212916_at | PHF8 | 2,272723257 | 1,835911187 | 0,43681207 | |
| 210630_s_at | RAD52 | 0,334552598 | -0,102100538 | 0,436653136 | |
| 226213_at | ERBB3 | 0,334552598 | -0,102100538 | 0,436653136 | |
| 234954_at | - | 2,102456302 | 1,665902186 | 0,436554116 | |
| 226860_at | TMEM19 | 2,819431021 | 2,382985886 | 0,436445135 | |
| 1553059_at | PGLYRP3 | -2,138804305 | -2,575129729 | 0,436325424 | |
| 1560838_at | FAM217A | -2,138804305 | -2,575129729 | 0,436325424 | |
| 1564504_at | ASIC5 | -2,138804305 | -2,575129729 | 0,436325424 | |
| 1564729_at | - | -2,138804305 | -2,575129729 | 0,436325424 | |
| 1570051_at | RNF144A | -2,138804305 | -2,575129729 | 0,436325424 | |
| 207322_at | ITSN1 | -2,138804305 | -2,575129729 | 0,436325424 | |
| 210611_s_at | DTNA | -2,138804305 | -2,575129729 | 0,436325424 | |
| 223928_s_at | GUCA1C | -2,138804305 | -2,575129729 | 0,436325424 | |
| 233751_at | ZFHX3 | -2,138804305 | -2,575129729 | 0,436325424 | |
| 239514_at | LOC100505784 | -2,138804305 | -2,575129729 | 0,436325424 | |
| 1568613_at | RSPH3 | 2,38635068 | 1,950068772 | 0,436281907 | |
| 209741_x_at | SCAPER | 0,152582607 | -0,283667828 | 0,436250434 | |
| 214244_s_at | LOC100652765 | 0,152582607 | -0,283667828 | 0,436250434 | |
| 227059_at | GPC6 | 0,152582607 | -0,283667828 | 0,436250434 | |
| 227496_at | LOC253842 /// NI | 0,152582607 | -0,283667828 | 0,436250434 | |
| 244713_at | - | 0,152582607 | -0,283667828 | 0,436250434 | |
| 203203_s_at | KRR1 | 2,868962815 | 2,432926755 | 0,43603606 | |
| 218846_at | MED23 | 4,136978698 | 3,701119714 | 0,435858983 | |
| 232194_at | METTL4 | 1,073999308 | 0,638150376 | 0,435848932 | |
| 209931_s_at | FKBP1B /// MFSI | -0,57445847 | -1,010192375 | 0,435733905 | |

| | | | | | |
|---|---|---|---|---|---|
| 212425_at | SCAMP1 | -0,57445847 | -1,010192375 | 0,435733905 | |
| 224529_s_at | NT5C1A | -0,57445847 | -1,010192375 | 0,435733905 | |
| 228198_s_at | FAHD2B /// FAHI | -0,57445847 | -1,010192375 | 0,435733905 | |
| 233091_at | ATAD3B | -0,57445847 | -1,010192375 | 0,435733905 | |
| 236356_at | NDUFS1 | -0,57445847 | -1,010192375 | 0,435733905 | |
| 215499_at | MAP2K3 | 3,179700672 | 2,744302548 | 0,435398124 | |
| 228505_s_at | TMEM170A | 1,515954005 | 1,080561626 | 0,43539238 | |
| 232642_at | VWA5B2 | -0,055687482 | -0,490927335 | 0,435239853 | |
| 235782_at | - | -0,055687482 | -0,490927335 | 0,435239853 | |
| 235958_at | PLA2G4F | -0,055687482 | -0,490927335 | 0,435239853 | |
| 224642_at | FYTTD1 | 2,501229299 | 2,066015817 | 0,435213482 | |
| 213702_x_at | ASAH1 | 5,922207779 | 5,487019892 | 0,435187887 | |
| 223238_s_at | PBRM1 | 4,099113343 | 3,663961904 | 0,435151438 | |
| 208654_s_at | CD164 | 5,38907564 | 4,954036493 | 0,435039147 | |
| 212538_at | DOCK9 | 0,728550685 | 0,293518836 | 0,43503185 | |
| 212662_at | PVR | 0,728550685 | 0,293518836 | 0,43503185 | |
| 226124_at | ZFP90 | 1,805273023 | 1,370274469 | 0,434998554 | |
| 1553962_s_at | RHOB | 0,702292136 | 0,26733076 | 0,434961377 | |
| 228572_at | - | 0,702292136 | 0,26733076 | 0,434961377 | |
| 236961_at | - | 0,702292136 | 0,26733076 | 0,434961377 | |
| 216899_s_at | SKAP2 | 2,077135162 | 1,642321262 | 0,434813899 | |
| 238199_x_at | COX3 | 4,439816047 | 4,005038666 | 0,434777381 | |
| 1555761_x_at | RBM15 | 1,269985355 | 0,835239052 | 0,434746302 | |
| 234734_s_at | TNRC6A | 2,433006244 | 1,998633167 | 0,434373077 | |
| 213810_s_at | AKIRIN2-AS1 | 0,44160593 | 0,007421914 | 0,434184016 | |
| 242241_x_at | - | 0,44160593 | 0,007421914 | 0,434184016 | |
| 229843_at | FAM82B | 1,037706275 | 0,603679647 | 0,434026628 | |
| 1552790_a_at | SEC62 | 0,273439642 | -0,160136748 | 0,43357639 | |
| 210451_at | PKLR | 0,273439642 | -0,160136748 | 0,43357639 | |
| 222068_s_at | DNAAF1 | 0,273439642 | -0,160136748 | 0,43357639 | |
| 1552508_at | KCNE4 | -1,353583928 | -1,787089803 | 0,433505875 | |
| 1557900_at | SIM2 | -1,353583928 | -1,787089803 | 0,433505875 | |
| 1564383_s_at | FLJ35934 | -1,353583928 | -1,787089803 | 0,433505875 | |
| 204345_at | COL16A1 | -1,353583928 | -1,787089803 | 0,433505875 | |
| 210404_x_at | CAMK2B | -1,353583928 | -1,787089803 | 0,433505875 | |
| 214814_at | YTHDC1 | -1,353583928 | -1,787089803 | 0,433505875 | |
| 223652_at | AS3MT | -1,353583928 | -1,787089803 | 0,433505875 | |
| 229902_at | FLT4 | -1,353583928 | -1,787089803 | 0,433505875 | |
| 234671_at | KRTAP4-2 | -1,353583928 | -1,787089803 | 0,433505875 | |
| 236001_at | LOC100289255 | -1,353583928 | -1,787089803 | 0,433505875 | |
| 239695_at | JAK1 | -1,353583928 | -1,787089803 | 0,433505875 | |
| 241156_at | - | -1,353583928 | -1,787089803 | 0,433505875 | |
| 242035_at | - | -1,353583928 | -1,787089803 | 0,433505875 | |
| 212956_at | TBC1D9 | 1,326951551 | 0,893473268 | 0,433478283 | |
| 1556318_s_at | CAND1 | -2,187914861 | -2,621331989 | 0,433417128 | |
| 1563725_at | ZNF583 | -2,187914861 | -2,621331989 | 0,433417128 | |
| 205893_at | NLGN1 | -2,187914861 | -2,621331989 | 0,433417128 | |
| 207349_s_at | UCP3 | -2,187914861 | -2,621331989 | 0,433417128 | |
| 213725_x_at | XYLT1 | -2,187914861 | -2,621331989 | 0,433417128 | |
| 216214_at | - | -2,187914861 | -2,621331989 | 0,433417128 | |
| 217566_s_at | TGM4 | -2,187914861 | -2,621331989 | 0,433417128 | |
| 230468_s_at | C1orf56 | -2,187914861 | -2,621331989 | 0,433417128 | |
| 233095_at | - | -2,187914861 | -2,621331989 | 0,433417128 | |
| 236444_x_at | - | -2,187914861 | -2,621331989 | 0,433417128 | |
| 244606_at | - | -2,187914861 | -2,621331989 | 0,433417128 | |
| 244880_at | - | -2,187914861 | -2,621331989 | 0,433417128 | |

| | | | | | |
|---|---|---|---|---|---|
| 201466_s_at | JUN | 2,327437554 | 1,894118818 | 0,433318736 | |
| 235006_at | CDKN2AIPNL | 1,238353345 | 0,805216064 | 0,433137281 | |
| 218306_s_at | HERC1 | 3,508091848 | 3,075135559 | 0,43295629 | |
| 1558105_a_at | SLC9A7 | 0,083054335 | -0,34958478 | 0,432639115 | |
| 1560818_at | LOC387895 | 0,083054335 | -0,34958478 | 0,432639115 | |
| 1569002_x_at | BMP1 | 0,083054335 | -0,34958478 | 0,432639115 | |
| 204136_at | COL7A1 | 0,083054335 | -0,34958478 | 0,432639115 | |
| 206086_x_at | HFE | 0,083054335 | -0,34958478 | 0,432639115 | |
| 211468_s_at | RECQL5 | 0,083054335 | -0,34958478 | 0,432639115 | |
| 211872_s_at | RGS11 | 0,083054335 | -0,34958478 | 0,432639115 | |
| 230049_at | TAF6L | 0,083054335 | -0,34958478 | 0,432639115 | |
| 237642_at | CTBP1-AS1 | 0,083054335 | -0,34958478 | 0,432639115 | |
| 242332_at | FOXF1-AS1 | 0,083054335 | -0,34958478 | 0,432639115 | |
| 214101_s_at | - | 1,104397092 | 0,67181667 | 0,432580422 | |
| 1552921_a_at | FIGNL1 | 2,134723057 | 1,702196344 | 0,432526713 | |
| 1553313_s_at | SLC5A3 | -0,423248115 | -0,855751026 | 0,432502911 | |
| 1553812_at | TLE6 | -0,423248115 | -0,855751026 | 0,432502911 | |
| 208439_s_at | FCN2 | -0,423248115 | -0,855751026 | 0,432502911 | |
| 209827_s_at | IL16 | -0,423248115 | -0,855751026 | 0,432502911 | |
| 215756_at | LOC730227 | -0,423248115 | -0,855751026 | 0,432502911 | |
| 235519_at | FOXK1 | -0,423248115 | -0,855751026 | 0,432502911 | |
| 235832_at | NKX6-2 | -0,423248115 | -0,855751026 | 0,432502911 | |
| 236288_at | RNF34 | -0,423248115 | -0,855751026 | 0,432502911 | |
| 227894_at | WDR90 | 1,850809354 | 1,418668082 | 0,432141272 | |
| 1554335_at | CYTH4 | -0,83191556 | -1,264005341 | 0,432089781 | |
| 1562425_at | FARP1 | -0,83191556 | -1,264005341 | 0,432089781 | |
| 1570371_a_at | EPT1 | -0,83191556 | -1,264005341 | 0,432089781 | |
| 205295_at | CKMT2 | -0,83191556 | -1,264005341 | 0,432089781 | |
| 206620_at | GRAP | -0,83191556 | -1,264005341 | 0,432089781 | |
| 211643_x_at | IGK@ /// IGKC | -0,83191556 | -1,264005341 | 0,432089781 | |
| 214163_at | HSPB11 | -0,83191556 | -1,264005341 | 0,432089781 | |
| 215173_at | DNAAF1 | -0,83191556 | -1,264005341 | 0,432089781 | |
| 220592_at | CCDC40 | -0,83191556 | -1,264005341 | 0,432089781 | |
| 231367_s_at | - | -0,83191556 | -1,264005341 | 0,432089781 | |
| 231509_at | PPP1R17 | -0,83191556 | -1,264005341 | 0,432089781 | |
| 237186_at | KCNJ5 | -0,83191556 | -1,264005341 | 0,432089781 | |
| 237695_at | - | -0,83191556 | -1,264005341 | 0,432089781 | |
| 242817_at | PGLYRP2 | -0,83191556 | -1,264005341 | 0,432089781 | |
| 243385_at | - | -0,83191556 | -1,264005341 | 0,432089781 | |
| 244834_at | RSG1 | -0,83191556 | -1,264005341 | 0,432089781 | |
| 1552988_at | C11orf65 | -2,667025278 | -3,098858316 | 0,431833039 | |
| 1559258_a_at | CXorf61 | -2,667025278 | -3,098858316 | 0,431833039 | |
| 231677_at | LOC100506165 | -2,667025278 | -3,098858316 | 0,431833039 | |
| 237361_at | - | -2,667025278 | -3,098858316 | 0,431833039 | |
| 243320_at | - | -2,667025278 | -3,098858316 | 0,431833039 | |
| 1560250_s_at | LOC284242 | -0,299034815 | -0,730778808 | 0,431743993 | |
| 215274_at | SLC12A3 | -0,299034815 | -0,730778808 | 0,431743993 | |
| 227909_at | LINC00086 /// LIN | -0,299034815 | -0,730778808 | 0,431743993 | |
| 231727_s_at | MIF4GD | 0,911589958 | 0,479907041 | 0,431682917 | |
| 238709_at | ELP2 | 0,911589958 | 0,479907041 | 0,431682917 | |
| 212464_s_at | FN1 | 2,107467633 | 1,675891488 | 0,431576145 | |
| 225980_at | C14orf43 | 2,644299732 | 2,212749286 | 0,431550445 | |
| 222740_at | ATAD2 | 5,053824781 | 4,622479552 | 0,43134523 | |
| 1555681_at | - | 0,384950573 | -0,046302147 | 0,43125272 | |
| 218348_s_at | ZC3H7A | 2,883642771 | 2,452428893 | 0,431213877 | |
| 214697_s_at | PTBP3 | 2,450859799 | 2,019703483 | 0,431156316 | |

| | | | | | |
|---|---|---|---|---|---|
| 201321_s_at | SMARCC2 | 1,841816345 | 1,410714371 | 0,431101974 | |
| 220091_at | SLC2A6 | 0,767063567 | 0,336136178 | 0,430927389 | |
| 228820_at | XPNPEP3 | 1,089278259 | 0,658444279 | 0,43083398 | |
| 212883_at | APOE | 1,352503698 | 0,921731975 | 0,430771723 | |
| 219493_at | SHCBP1 | 3,600740871 | 3,170070041 | 0,43067083 | |
| 204739_at | CENPC1 | 2,227406889 | 1,796776588 | 0,430630301 | |
| 1559471_s_at | D21S2088E | -0,136301282 | -0,566887459 | 0,430586177 | |
| 217692_at | MAGOH2 | -0,136301282 | -0,566887459 | 0,430586177 | |
| 225322_s_at | C17orf70 | -0,136301282 | -0,566887459 | 0,430586177 | |
| 229575_at | - | -0,136301282 | -0,566887459 | 0,430586177 | |
| 236076_at | LOC257396 | -0,136301282 | -0,566887459 | 0,430586177 | |
| 1558220_at | - | -0,946847173 | -1,377419394 | 0,430572221 | |
| 1569189_at | TTC9C | -0,946847173 | -1,377419394 | 0,430572221 | |
| 211819_s_at | SORBS1 | -0,946847173 | -1,377419394 | 0,430572221 | |
| 214191_at | ICA1 | -0,946847173 | -1,377419394 | 0,430572221 | |
| 215551_at | ESR1 | -0,946847173 | -1,377419394 | 0,430572221 | |
| 215990_s_at | BCL6 | -0,946847173 | -1,377419394 | 0,430572221 | |
| 223957_at | - | -0,946847173 | -1,377419394 | 0,430572221 | |
| 230671_at | - | -0,946847173 | -1,377419394 | 0,430572221 | |
| 238345_at | SLC38A10 | -0,946847173 | -1,377419394 | 0,430572221 | |
| 238582_at | C21orf2 | -0,946847173 | -1,377419394 | 0,430572221 | |
| 238683_at | ZNF524 | -0,946847173 | -1,377419394 | 0,430572221 | |
| 242354_at | - | -0,946847173 | -1,377419394 | 0,430572221 | |
| 242367_at | - | -0,946847173 | -1,377419394 | 0,430572221 | |
| 244371_at | - | -0,946847173 | -1,377419394 | 0,430572221 | |
| 226152_at | TTC7B | 4,08075718 | 3,650516168 | 0,430241012 | |
| 239807_at | - | 0,984222186 | 0,553993624 | 0,430228561 | |
| 1553034_at | SDCCAG8 | 0,209623097 | -0,220592223 | 0,430215321 | |
| 230025_at | GJD3 | 0,209623097 | -0,220592223 | 0,430215321 | |
| 200965_s_at | ABLIM1 | 1,247462054 | 0,817300337 | 0,430161717 | |
| 225786_at | HNRNPU-AS1 | 1,247462054 | 0,817300337 | 0,430161717 | |
| 37996_s_at | DMPK | 0,421626332 | -0,008482952 | 0,430109284 | |
| 212926_at | SMC5 | 0,606430281 | 0,176436073 | 0,429994208 | |
| 230160_x_at | - | 0,606430281 | 0,176436073 | 0,429994208 | |
| 239314_at | NHLRC3 | 0,606430281 | 0,176436073 | 0,429994208 | |
| 224570_s_at | IRF2BP2 | 3,962664437 | 3,532699346 | 0,42996509 | |
| 1557146_a_at | SSTR5-AS1 | -1,308181863 | -1,738067356 | 0,429885493 | |
| 216916_s_at | DLGAP2 | -1,308181863 | -1,738067356 | 0,429885493 | |
| 230128_at | IGLL5 | -1,308181863 | -1,738067356 | 0,429885493 | |
| 233018_at | TMEM134 | -1,308181863 | -1,738067356 | 0,429885493 | |
| 233242_at | WDR73 | -1,308181863 | -1,738067356 | 0,429885493 | |
| 233382_at | - | -1,308181863 | -1,738067356 | 0,429885493 | |
| 239361_at | - | -1,308181863 | -1,738067356 | 0,429885493 | |
| 243014_at | - | -1,308181863 | -1,738067356 | 0,429885493 | |
| 205250_s_at | CEP290 | 1,390002893 | 0,960386719 | 0,429616174 | |
| 202231_at | EIF3M | 6,339652993 | 5,910321048 | 0,429331946 | |
| 220731_s_at | NECAP2 | 2,959046717 | 2,529740643 | 0,429306075 | |
| 201297_s_at | MOB1A | 2,094906514 | 1,665902186 | 0,429004329 | |
| 218251_at | MID1IP1 | 1,666156729 | 1,23718272 | 0,428974009 | |
| 203870_at | USP46 | 1,799091453 | 1,370274469 | 0,428816984 | |
| 227075_at | ELP3 | 2,81177561 | 2,382985886 | 0,428789724 | |
| 201855_s_at | ATMIN | 3,010782559 | 2,582026171 | 0,428756388 | |
| 1552344_s_at | CNOT7 | 3,532516649 | 3,103784293 | 0,428732356 | |
| 230265_at | SEL1L | 2,86748657 | 2,438805127 | 0,428681443 | |
| 214152_at | CCPG1 /// DYX1( | 1,442503549 | 1,013870809 | 0,42863274 | |
| 205527_s_at | GEMIN4 | 3,028064153 | 2,599625088 | 0,428439065 | |

| | | | | | |
|---|---|---|---|---|---|
| 242618_at | HCG18 | 1,215326747 | 0,786897721 | 0,428429026 | |
| 225457_s_at | LINC00263 /// PP | 2,001086023 | 1,572742379 | 0,428343644 | |
| 200684_s_at | UBE2L3 | 2,945117939 | 2,516830926 | 0,428287014 | |
| 208516_at | MTNR1B | -0,033456564 | -0,461594427 | 0,428137862 | |
| 228134_at | MYH11 | -0,033456564 | -0,461594427 | 0,428137862 | |
| 239188_at | PPP2R3C | -0,033456564 | -0,461594427 | 0,428137862 | |
| 1561012_at | - | -2,262747984 | -2,690860744 | 0,42811276 | |
| 1563170_at | - | -2,262747984 | -2,690860744 | 0,42811276 | |
| 1563524_a_at | ITPK1-AS1 | -2,262747984 | -2,690860744 | 0,42811276 | |
| 1570121_at | ZNF365 | -2,262747984 | -2,690860744 | 0,42811276 | |
| 1570279_at | ABCA1 | -2,262747984 | -2,690860744 | 0,42811276 | |
| 219302_s_at | CNTNAP2 | -2,262747984 | -2,690860744 | 0,42811276 | |
| 221683_s_at | CEP290 | -2,262747984 | -2,690860744 | 0,42811276 | |
| 234547_at | - | -2,262747984 | -2,690860744 | 0,42811276 | |
| 238242_at | - | -2,262747984 | -2,690860744 | 0,42811276 | |
| 242047_at | RTKN2 | -2,262747984 | -2,690860744 | 0,42811276 | |
| 243914_at | - | -2,262747984 | -2,690860744 | 0,42811276 | |
| 243959_at | - | -2,262747984 | -2,690860744 | 0,42811276 | |
| 203178_at | GATM | 3,515651309 | 3,087661492 | 0,427989818 | |
| 1552596_at | GAS2L2 | 0,291166226 | -0,136643983 | 0,427810208 | |
| 211820_x_at | GYPA | 0,291166226 | -0,136643983 | 0,427810208 | |
| 243816_at | ZNF70 | 0,291166226 | -0,136643983 | 0,427810208 | |
| 230175_s_at | DCBLD2 | 3,109000457 | 2,681234383 | 0,427766074 | |
| 228899_at | - | 1,119359127 | 0,691645685 | 0,427713442 | |
| 1555661_at | OR8D1 | -0,622910612 | -1,050406615 | 0,427496003 | |
| 1561897_at | - | -0,622910612 | -1,050406615 | 0,427496003 | |
| 207316_at | HAS1 | -0,622910612 | -1,050406615 | 0,427496003 | |
| 209167_at | GPM6B | -0,622910612 | -1,050406615 | 0,427496003 | |
| 211897_s_at | CRHR1 | -0,622910612 | -1,050406615 | 0,427496003 | |
| 214130_s_at | LOC728802 /// PI | -0,622910612 | -1,050406615 | 0,427496003 | |
| 219535_at | HUNK | -0,622910612 | -1,050406615 | 0,427496003 | |
| 234130_at | LDB3 | -0,622910612 | -1,050406615 | 0,427496003 | |
| 234584_s_at | ATE1 | 1,414471577 | 0,987376598 | 0,42709498 | |
| 204702_s_at | NFE2L3 | 2,017152618 | 1,590454215 | 0,426698403 | |
| 1563223_a_at | CENPI | -2,712026959 | -3,138626879 | 0,42659992 | |
| 1564250_at | - | -2,712026959 | -3,138626879 | 0,42659992 | |
| 213994_s_at | SPON1 | -2,712026959 | -3,138626879 | 0,42659992 | |
| 242221_at | - | -2,712026959 | -3,138626879 | 0,42659992 | |
| 244272_s_at | TC2N | -2,712026959 | -3,138626879 | 0,42659992 | |
| 1556042_s_at | LOC338799 | 1,348276312 | 0,921731975 | 0,426544336 | |
| 204133_at | RRP9 | 1,348276312 | 0,921731975 | 0,426544336 | |
| 208855_s_at | STK24 | 6,063886117 | 5,637514587 | 0,42637153 | |
| 227865_at | IDNK | 0,103263601 | -0,322870281 | 0,426133883 | |
| 228677_s_at | RASAL3 | 0,103263601 | -0,322870281 | 0,426133883 | |
| 230150_at | BCAP29 | 0,103263601 | -0,322870281 | 0,426133883 | |
| 1570523_s_at | ATG10 | 0,401366113 | -0,024572586 | 0,425938699 | |
| 223285_s_at | ST6GALNAC4 | 0,401366113 | -0,024572586 | 0,425938699 | |
| 227162_at | ZBTB26 | 1,426552034 | 1,000684521 | 0,425867513 | |
| 236614_at | LOC729683 | 0,882651021 | 0,456918297 | 0,425732724 | |
| 221997_s_at | MRPL52 | 0,648296226 | 0,222599095 | 0,425697131 | |
| 204872_at | TLE4 | 0,859074518 | 0,433557298 | 0,42551722 | |
| 205408_at | MLLT10 | 1,515954005 | 1,090553537 | 0,425400468 | |
| 205348_s_at | DYNC1I1 | -0,221656139 | -0,646746079 | 0,42508994 | |
| 207836_s_at | RBPMS | -0,221656139 | -0,646746079 | 0,42508994 | |
| 214406_s_at | SLC7A4 | -0,221656139 | -0,646746079 | 0,42508994 | |
| 228709_at | TPR | -0,221656139 | -0,646746079 | 0,42508994 | |

| | | | | | |
|---|---|---|---|---|---|
| 230161_at | - | -0,221656139 | -0,646746079 | 0,42508994 | |
| 238250_at | - | -0,221656139 | -0,646746079 | 0,42508994 | |
| 224595_at | SLC44A1 | 2,678376417 | 2,253342112 | 0,425034305 | |
| 220081_x_at | HSD17B7 | 1,593009234 | 1,16809119 | 0,424918044 | |
| 226241_s_at | MRPL52 | 4,746488337 | 4,321571774 | 0,424916562 | |
| 218943_s_at | DDX58 | 1,318332599 | 0,893473268 | 0,424859331 | |
| 228828_at | BZRAP1-AS1 | 2,470441349 | 2,045615873 | 0,424825476 | |
| 1558530_at | LRTM2 | -0,79540287 | -1,220210588 | 0,424807718 | |
| 1559532_at | C3orf71 | -0,79540287 | -1,220210588 | 0,424807718 | |
| 206225_at | ZNF507 | -0,79540287 | -1,220210588 | 0,424807718 | |
| 217209_at | CEACAM3 | -0,79540287 | -1,220210588 | 0,424807718 | |
| 230424_at | NREP | -0,79540287 | -1,220210588 | 0,424807718 | |
| 234449_at | - | -0,79540287 | -1,220210588 | 0,424807718 | |
| 205371_s_at | DBT | 0,533835206 | 0,109221392 | 0,424613814 | |
| 1554294_s_at | TTBK2 | -2,311268652 | -2,735862426 | 0,424593774 | |
| 1554842_at | SLC12A1 | -2,311268652 | -2,735862426 | 0,424593774 | |
| 1557285_at | AREGB | -2,311268652 | -2,735862426 | 0,424593774 | |
| 1558494_at | - | -2,311268652 | -2,735862426 | 0,424593774 | |
| 1561501_s_at | CTU2 | -2,311268652 | -2,735862426 | 0,424593774 | |
| 1562440_at | MAP3K13 | -2,311268652 | -2,735862426 | 0,424593774 | |
| 1564813_at | - | -2,311268652 | -2,735862426 | 0,424593774 | |
| 205573_s_at | SNX7 | -2,311268652 | -2,735862426 | 0,424593774 | |
| 210661_at | GLRA3 | -2,311268652 | -2,735862426 | 0,424593774 | |
| 214856_at | SPTBN1 | -2,311268652 | -2,735862426 | 0,424593774 | |
| 221411_at | HOXD12 | -2,311268652 | -2,735862426 | 0,424593774 | |
| 221530_s_at | BHLHE41 | -2,311268652 | -2,735862426 | 0,424593774 | |
| 225664_at | COL12A1 | -2,311268652 | -2,735862426 | 0,424593774 | |
| 229816_at | WDR78 | -2,311268652 | -2,735862426 | 0,424593774 | |
| 240481_at | - | -2,311268652 | -2,735862426 | 0,424593774 | |
| 1553593_a_at | TAL2 | -0,394749123 | -0,819238336 | 0,424489213 | |
| 218033_s_at | SNN | -0,394749123 | -0,819238336 | 0,424489213 | |
| 229770_at | GLT1D1 | -0,394749123 | -0,819238336 | 0,424489213 | |
| 1552652_at | HPS4 | 0,735041319 | 0,310717132 | 0,424324187 | |
| 202482_x_at | RANBP1 | 0,503742507 | 0,079428135 | 0,424314372 | |
| 242191_at | NBPF10 | 0,503742507 | 0,079428135 | 0,424314372 | |
| 208404_x_at | KCNJ5 | 0,228146222 | -0,19610998 | 0,424256202 | |
| 234491_s_at | SAV1 | 2,109966786 | 1,685812099 | 0,424154687 | |
| 213287_s_at | KRT10 | 1,04816921 | 0,624460759 | 0,423708451 | |
| 229672_at | UQCC | 1,04816921 | 0,624460759 | 0,423708451 | |
| 205303_at | KCNJ8 | 1,027166904 | 0,603679647 | 0,423487258 | |
| 228437_at | CNIH4 | 0,841135803 | 0,417770464 | 0,423365339 | |
| 226772_s_at | SAP30L | -0,112808516 | -0,536087151 | 0,423278634 | |
| 236518_at | KIAA1984 | -0,112808516 | -0,536087151 | 0,423278634 | |
| 238110_at | PPP6R2 | -0,112808516 | -0,536087151 | 0,423278634 | |
| 239679_at | - | -0,112808516 | -0,536087151 | 0,423278634 | |
| 242609_x_at | - | -0,112808516 | -0,536087151 | 0,423278634 | |
| 1559514_at | LOC100132077 | -1,240169874 | -1,663414495 | 0,42324462 | |
| 1565802_at | - | -1,240169874 | -1,663414495 | 0,42324462 | |
| 1566279_at | - | -1,240169874 | -1,663414495 | 0,42324462 | |
| 1566490_at | - | -1,240169874 | -1,663414495 | 0,42324462 | |
| 1567913_at | - | -1,240169874 | -1,663414495 | 0,42324462 | |
| 216837_at | EPHA5 | -1,240169874 | -1,663414495 | 0,42324462 | |
| 221236_s_at | STMN4 | -1,240169874 | -1,663414495 | 0,42324462 | |
| 231501_at | LOC100507162 | -1,240169874 | -1,663414495 | 0,42324462 | |
| 232239_at | LOC643529 | -1,240169874 | -1,663414495 | 0,42324462 | |
| 237355_at | - | -1,240169874 | -1,663414495 | 0,42324462 | |

| | | | | | |
|---|---|---|---|---|---|
| 237943_at | TMCC1 | -1,240169874 | -1,663414495 | 0,42324462 | |
| 244410_at | PKHD1 | -1,240169874 | -1,663414495 | 0,42324462 | |
| 219396_s_at | MIR631 /// NEIL1 | 0,376672223 | -0,046302147 | 0,42297437 | |
| 228245_s_at | LOC100509445 / | 0,376672223 | -0,046302147 | 0,42297437 | |
| 243024_at | ZNF394 | 0,376672223 | -0,046302147 | 0,42297437 | |
| 222041_at | DPH1 /// OVCA2 | 1,114389003 | 0,691645685 | 0,422743318 | |
| 223029_s_at | TRAF7 | 1,114389003 | 0,691645685 | 0,422743318 | |
| 221020_s_at | SLC25A32 | 4,702869182 | 4,2801535 | 0,422715681 | |
| 202340_x_at | NR4A1 | 1,094335495 | 0,67181667 | 0,422518825 | |
| 215266_at | DNAH3 | 0,072842263 | -0,34958478 | 0,422427043 | |
| 217285_at | DGCR14 /// TSSI | 0,072842263 | -0,34958478 | 0,422427043 | |
| 212968_at | RFNG | 0,917308734 | 0,495031851 | 0,422276883 | |
| 223146_at | SFT2D3 /// WDR | 0,917308734 | 0,495031851 | 0,422276883 | |
| 226174_at | USP42 | 0,917308734 | 0,495031851 | 0,422276883 | |
| 212623_at | TMEM41B | 4,392357579 | 3,970285709 | 0,42207187 | |
| 202367_at | CUX1 | 0,715481152 | 0,293518836 | 0,421962316 | |
| 223059_s_at | FAM107B | 0,715481152 | 0,293518836 | 0,421962316 | |
| 230435_at | FAM228B | 0,715481152 | 0,293518836 | 0,421962316 | |
| 1566151_at | - | 0,03125738 | -0,39061235 | 0,42186973 | |
| 221663_x_at | HRH3 | 0,03125738 | -0,39061235 | 0,42186973 | |
| 229825_at | PLK3 | 0,03125738 | -0,39061235 | 0,42186973 | |
| 205249_at | EGR2 | 1,422536444 | 1,000684521 | 0,421851923 | |
| 238510_at | ZNF720 | 2,72965419 | 2,307890888 | 0,421763302 | |
| 204489_s_at | CD44 | 2,814842651 | 2,39311332 | 0,421729331 | |
| 205126_at | VRK2 | 3,63237684 | 3,210719429 | 0,421657411 | |
| 1563796_s_at | EARS2 | 1,011212064 | 0,589657387 | 0,421554677 | |
| 1564109_at | LOC284865 | 0,480753764 | 0,059218869 | 0,421534895 | |
| 214423_x_at | ALDOB | 0,480753764 | 0,059218869 | 0,421534895 | |
| 201162_at | IGFBP7 | 2,227406889 | 1,805902329 | 0,42150456 | |
| 1559573_at | LOC100506229 | -2,756478458 | -3,177802286 | 0,421323828 | |
| 217694_at | - | -2,756478458 | -3,177802286 | 0,421323828 | |
| 220376_at | LRRC19 | -2,756478458 | -3,177802286 | 0,421323828 | |
| 220771_at | LINC00328 | -2,756478458 | -3,177802286 | 0,421323828 | |
| 224034_at | - | -2,756478458 | -3,177802286 | 0,421323828 | |
| 33778_at | TBC1D22A | 2,875587286 | 2,454364686 | 0,4212226 | |
| 220954_s_at | PILRB | 1,238353345 | 0,817300337 | 0,421053008 | |
| 1554890_a_at | TIA1 | 2,365585359 | 1,944570415 | 0,421014944 | |
| 40273_at | SPHK2 | 1,86715234 | 1,44616575 | 0,420986591 | |
| 212934_at | UBXN2B | 2,663141434 | 2,242180416 | 0,420961018 | |
| 1556941_a_at | LOC283484 | -2,359436556 | -2,780313924 | 0,420877368 | |
| 1563595_at | SRGAP3 | -2,359436556 | -2,780313924 | 0,420877368 | |
| 1564003_at | - | -2,359436556 | -2,780313924 | 0,420877368 | |
| 1569817_at | - | -2,359436556 | -2,780313924 | 0,420877368 | |
| 231523_at | FGF14 | -2,359436556 | -2,780313924 | 0,420877368 | |
| 235445_at | LOC100506092 | -2,359436556 | -2,780313924 | 0,420877368 | |
| 239273_s_at | MMP28 | -2,359436556 | -2,780313924 | 0,420877368 | |
| 241616_at | - | -2,359436556 | -2,780313924 | 0,420877368 | |
| 244235_at | IVNS1ABP | -2,359436556 | -2,780313924 | 0,420877368 | |
| 220473_s_at | ZCCHC4 | 0,20027154 | -0,220592223 | 0,420863763 | |
| 207492_at | NGLY1 | 0,798390451 | 0,377530647 | 0,420859804 | |
| 1555826_at | - | 1,296557217 | 0,876248481 | 0,420308736 | |
| 230123_at | NECAP2 | 1,296557217 | 0,876248481 | 0,420308736 | |
| 205832_at | CPA4 | 0,57782909 | 0,157549243 | 0,420279848 | |
| 235045_at | RBM7 | 0,57782909 | 0,157549243 | 0,420279848 | |
| 213741_s_at | KPNA1 | 2,122398022 | 1,702196344 | 0,420201678 | |
| 204291_at | LOC100509474 / | 3,203298287 | 2,783327323 | 0,419970964 | |

| | | | | | |
|---|---|---|---|---|---|
| 212564_at | KCTD2 | 2,16388051 | 1,743944487 | 0,419936022 | |
| 1558670_at | - | -0,259832361 | -0,679763839 | 0,419931478 | |
| 1559139_at | NOC2L | -0,259832361 | -0,679763839 | 0,419931478 | |
| 201203_s_at | RRBP1 | -0,259832361 | -0,679763839 | 0,419931478 | |
| 226724_s_at | - | -0,259832361 | -0,679763839 | 0,419931478 | |
| 230821_at | ZNF148 | -0,259832361 | -0,679763839 | 0,419931478 | |
| 239181_at | - | -0,259832361 | -0,679763839 | 0,419931478 | |
| 243312_at | - | -0,259832361 | -0,679763839 | 0,419931478 | |
| 1570295_at | - | -0,59053611 | -1,010192375 | 0,419656265 | |
| 206530_at | RAB30 | -0,59053611 | -1,010192375 | 0,419656265 | |
| 206691_s_at | PDIA2 | -0,59053611 | -1,010192375 | 0,419656265 | |
| 211670_x_at | SSX3 | -0,59053611 | -1,010192375 | 0,419656265 | |
| 232800_at | - | -0,59053611 | -1,010192375 | 0,419656265 | |
| 237500_at | - | -0,59053611 | -1,010192375 | 0,419656265 | |
| 238046_x_at | PWWP2B | -0,59053611 | -1,010192375 | 0,419656265 | |
| 208820_at | LOC100653024 / | 5,168558587 | 4,748914989 | 0,419643598 | |
| 225329_at | FAM195B | 2,808702036 | 2,389070873 | 0,419631162 | |
| 222646_s_at | ERO1L | 3,726584632 | 3,307062282 | 0,419522351 | |
| 222491_at | HGSNAT | 0,317354302 | -0,102100538 | 0,419454839 | |
| 209037_s_at | EHD1 | 1,596577845 | 1,177496821 | 0,419081025 | |
| 221782_at | DNAJC10 | 2,683419202 | 2,264418116 | 0,419001085 | |
| 213331_s_at | NEK1 | 2,084778276 | 1,665902186 | 0,418876091 | |
| 222587_s_at | GALNT7 | 2,589836943 | 2,170981159 | 0,418855784 | |
| 1553400_a_at | C17orf69 | -1,196375121 | -1,615132408 | 0,418757286 | |
| 1555082_a_at | NEK11 | -1,196375121 | -1,615132408 | 0,418757286 | |
| 1570106_at | - | -1,196375121 | -1,615132408 | 0,418757286 | |
| 205537_s_at | VAV2 | -1,196375121 | -1,615132408 | 0,418757286 | |
| 207529_at | DEFA5 | -1,196375121 | -1,615132408 | 0,418757286 | |
| 215220_s_at | TPR | -1,196375121 | -1,615132408 | 0,418757286 | |
| 220621_at | FOXE3 | -1,196375121 | -1,615132408 | 0,418757286 | |
| 230646_at | FNDC5 | -1,196375121 | -1,615132408 | 0,418757286 | |
| 231518_at | LOC283867 | -1,196375121 | -1,615132408 | 0,418757286 | |
| 231757_at | TAS2R5 | -1,196375121 | -1,615132408 | 0,418757286 | |
| 232878_at | LOC644192 | -1,196375121 | -1,615132408 | 0,418757286 | |
| 236266_at | RORA | -1,196375121 | -1,615132408 | 0,418757286 | |
| 236831_at | CCDC50 | -1,196375121 | -1,615132408 | 0,418757286 | |
| 241112_at | ZNF517 | -1,196375121 | -1,615132408 | 0,418757286 | |
| 243288_at | SMYD2 | -1,196375121 | -1,615132408 | 0,418757286 | |
| 243547_at | FLJ39639 /// ZNF | -1,196375121 | -1,615132408 | 0,418757286 | |
| 225481_at | FRMD6 | 1,283332461 | 0,864649967 | 0,418682493 | |
| 205934_at | PLCL1 | 1,64218396 | 1,223626587 | 0,418557373 | |
| 222382_x_at | NUP205 | 0,425644434 | 0,007421914 | 0,41822252 | |
| 231681_x_at | HIST3H2A | 0,425644434 | 0,007421914 | 0,41822252 | |
| 243649_at | FBXO7 | 0,425644434 | 0,007421914 | 0,41822252 | |
| 204914_s_at | SOX11 | -1,543331864 | -1,961547147 | 0,418215283 | |
| 211022_s_at | ATRX | -1,543331864 | -1,961547147 | 0,418215283 | |
| 224305_s_at | NPC1L1 | -1,543331864 | -1,961547147 | 0,418215283 | |
| 229053_at | SYT17 | -1,543331864 | -1,961547147 | 0,418215283 | |
| 229463_at | NTRK2 | -1,543331864 | -1,961547147 | 0,418215283 | |
| 231591_at | DMGDH | -1,543331864 | -1,961547147 | 0,418215283 | |
| 232113_at | - | -1,543331864 | -1,961547147 | 0,418215283 | |
| 235238_at | SHC4 | -1,543331864 | -1,961547147 | 0,418215283 | |
| 237944_at | - | -1,543331864 | -1,961547147 | 0,418215283 | |
| 243365_s_at | AUTS2 | -1,543331864 | -1,961547147 | 0,418215283 | |
| 226783_at | AGXT2L2 | 1,339784194 | 0,921731975 | 0,418052219 | |
| 1553860_at | DCST1 | -0,43775896 | -0,855751026 | 0,417992066 | |

| | | | | | |
|---|---|---|---|---|---|
| 208524_at | GPR15 | -0,43775896 | -0,855751026 | 0,417992066 | |
| 217511_at | KAZALD1 | -0,43775896 | -0,855751026 | 0,417992066 | |
| 222331_at | - | -0,43775896 | -0,855751026 | 0,417992066 | |
| 240233_at | LOC100506714 | -0,43775896 | -0,855751026 | 0,417992066 | |
| 200961_at | SEPHS2 | 5,305414236 | 4,88753743 | 0,417876806 | |
| 208869_s_at | GABARAPL1 | -0,00073712 | -0,41858459 | 0,417847469 | |
| 229107_at | - | -0,00073712 | -0,41858459 | 0,417847469 | |
| 237793_at | C20orf78 | -0,00073712 | -0,41858459 | 0,417847469 | |
| 1568799_at | - | -0,196756757 | -0,614371577 | 0,41761482 | |
| 207339_s_at | LTB | -0,196756757 | -0,614371577 | 0,41761482 | |
| 235611_at | SREK1 | -0,196756757 | -0,614371577 | 0,41761482 | |
| 236683_at | - | -0,196756757 | -0,614371577 | 0,41761482 | |
| 212838_at | DNMBP | 1,795990708 | 1,378453643 | 0,417537064 | |
| 1559638_at | C1orf200 | 1,394109935 | 0,976641161 | 0,417468774 | |
| 221973_at | LOC100506076 / | 1,089278259 | 0,67181667 | 0,417461589 | |
| 212515_s_at | DDX3X | 5,499701558 | 5,082250729 | 0,417450829 | |
| 219500_at | CLCF1 | 0,810733188 | 0,393761504 | 0,416971683 | |
| 220666_at | - | -2,930741289 | -3,347710726 | 0,416969437 | |
| 203004_s_at | MEF2D | 0,133056859 | -0,283667828 | 0,416724686 | |
| 214256_at | ATP10A | 0,133056859 | -0,283667828 | 0,416724686 | |
| 214353_at | - | 0,133056859 | -0,283667828 | 0,416724686 | |
| 219023_at | AP1AR | 2,559073676 | 2,14244844 | 0,416625237 | |
| 223535_at | NUDT12 | 1,496984567 | 1,080561626 | 0,416422941 | |
| 203433_at | MTHFS /// ST20- | 2,713311265 | 2,297144887 | 0,416166379 | |
| 212836_at | POLD3 | 2,206540699 | 1,790660532 | 0,415880167 | |
| 243252_at | - | 0,984222186 | 0,568365095 | 0,41585709 | |
| 1569098_s_at | TP53BP1 | 0,592200562 | 0,176436073 | 0,415764488 | |
| 207072_at | IL18RAP | 0,592200562 | 0,176436073 | 0,415764488 | |
| 214216_s_at | LARP4B | 0,592200562 | 0,176436073 | 0,415764488 | |
| 215887_at | ZNF277 | 0,592200562 | 0,176436073 | 0,415764488 | |
| 228293_at | DEPDC7 | 2,197169074 | 1,781437557 | 0,415731517 | |
| 222428_s_at | LARS | 5,256624694 | 4,840945211 | 0,415679483 | |
| 216450_x_at | HSP90B1 | 2,782308844 | 2,366632651 | 0,415676193 | |
| 1569879_a_at | MEGF11 | 0,255492549 | -0,160136748 | 0,415629298 | |
| 216845_x_at | MLL2 | 0,255492549 | -0,160136748 | 0,415629298 | |
| 228486_at | SLC44A1 | 0,255492549 | -0,160136748 | 0,415629298 | |
| 232041_at | INADL | 0,255492549 | -0,160136748 | 0,415629298 | |
| 238493_at | ZNF506 | 1,865674241 | 1,450051551 | 0,41562269 | |
| 242994_at | - | 1,073999308 | 0,658444279 | 0,415555029 | |
| 202737_s_at | LSM4 | 4,730742086 | 4,31519468 | 0,415547406 | |
| 242760_x_at | PIGB | 1,348276312 | 0,932882274 | 0,415394038 | |
| 1567069_at | OR4D1 | -0,496991414 | -0,912331589 | 0,415340175 | |
| 208058_s_at | MGAT3 | -0,496991414 | -0,912331589 | 0,415340175 | |
| 228236_at | SLC52A3 | -0,496991414 | -0,912331589 | 0,415340175 | |
| 230965_at | USP2 | -0,496991414 | -0,912331589 | 0,415340175 | |
| 236209_at | - | -0,496991414 | -0,912331589 | 0,415340175 | |
| 244710_at | LRGUK | -0,496991414 | -0,912331589 | 0,415340175 | |
| 213552_at | GLCE | 1,05337236 | 0,638150376 | 0,415221983 | |
| 215067_x_at | PRDX2 | 1,05337236 | 0,638150376 | 0,415221983 | |
| 213296_at | RER1 | 1,709647566 | 1,294497133 | 0,415150433 | |
| 217299_s_at | NBN | 2,294858597 | 1,879785595 | 0,415073002 | |
| 1560327_at | - | 1,40228911 | 0,987376598 | 0,414912512 | |
| 213090_s_at | MIR1257 /// TAF4 | 3,123915073 | 2,709065209 | 0,414849863 | |
| 218101_s_at | NDUFC2 /// NDU | 6,13991327 | 5,725205755 | 0,414707514 | |
| 229390_at | FAM26F | 1,201332287 | 0,786897721 | 0,414434565 | |
| 1552582_at | ABCC13 | -2,43257096 | -2,847001814 | 0,414430854 | |

| | | | | | |
|---|---|---|---|---|---|
| 1554161_at | SLC25A27 | -2,43257096 | -2,847001814 | 0,414430854 | |
| 1555457_at | DBC1 | -2,43257096 | -2,847001814 | 0,414430854 | |
| 1557869_at | - | -2,43257096 | -2,847001814 | 0,414430854 | |
| 1569545_at | - | -2,43257096 | -2,847001814 | 0,414430854 | |
| 206142_at | ZNF135 | -2,43257096 | -2,847001814 | 0,414430854 | |
| 211109_at | JAK3 | -2,43257096 | -2,847001814 | 0,414430854 | |
| 214618_at | CFLAR | -2,43257096 | -2,847001814 | 0,414430854 | |
| 219682_s_at | TBX3 | -2,43257096 | -2,847001814 | 0,414430854 | |
| 220552_at | TRPC5 | -2,43257096 | -2,847001814 | 0,414430854 | |
| 223278_at | GJB2 | -2,43257096 | -2,847001814 | 0,414430854 | |
| 226189_at | ITGB8 | -2,43257096 | -2,847001814 | 0,414430854 | |
| 236287_at | - | -2,43257096 | -2,847001814 | 0,414430854 | |
| 237054_at | ENPP5 | -2,43257096 | -2,847001814 | 0,414430854 | |
| 219183_s_at | CYTH4 | 0,767063567 | 0,352836757 | 0,41422681 | |
| 1555505_a_at | TYR | -2,089474779 | -2,503677622 | 0,414202843 | |
| 1557821_at | LOC283547 | -2,089474779 | -2,503677622 | 0,414202843 | |
| 1561249_a_at | DNM1P35 | -2,089474779 | -2,503677622 | 0,414202843 | |
| 1561251_at | LOC285577 | -2,089474779 | -2,503677622 | 0,414202843 | |
| 202435_s_at | CYP1B1 | -2,089474779 | -2,503677622 | 0,414202843 | |
| 215935_at | FAM75A1 /// FAM | -2,089474779 | -2,503677622 | 0,414202843 | |
| 220230_s_at | CYB5R2 | -2,089474779 | -2,503677622 | 0,414202843 | |
| 222043_at | CLU | -2,089474779 | -2,503677622 | 0,414202843 | |
| 224044_at | RHOT1 | -2,089474779 | -2,503677622 | 0,414202843 | |
| 231214_at | - | -2,089474779 | -2,503677622 | 0,414202843 | |
| 231557_at | - | -2,089474779 | -2,503677622 | 0,414202843 | |
| 244513_at | TMEM161B | -2,089474779 | -2,503677622 | 0,414202843 | |
| 227118_s_at | MRPS26 | 0,334552598 | -0,079522948 | 0,414075546 | |
| 242247_at | METTL15 | 0,334552598 | -0,079522948 | 0,414075546 | |
| 202571_s_at | DLGAP4 | 0,87091093 | 0,456918297 | 0,413992633 | |
| 218254_s_at | SAR1B | 4,053102157 | 3,639498476 | 0,413603681 | |
| 230298_at | MBLAC2 | 2,59162628 | 2,17802703 | 0,41359925 | |
| 219609_at | WDR25 | 0,945567441 | 0,532164371 | 0,413403071 | |
| 1555611_s_at | MBD1 | 1,037706275 | 0,624460759 | 0,413245516 | |
| 231212_x_at | - | 1,037706275 | 0,624460759 | 0,413245516 | |
| 228052_x_at | TCF3 | 0,44160593 | 0,028364014 | 0,413241916 | |
| 1562664_at | LOC286009 | -0,741668563 | -1,154897679 | 0,413229116 | |
| 217411_s_at | RREB1 | -0,741668563 | -1,154897679 | 0,413229116 | |
| 228016_s_at | - | -0,741668563 | -1,154897679 | 0,413229116 | |
| 231162_at | CLDND2 | -0,741668563 | -1,154897679 | 0,413229116 | |
| 236788_at | - | -0,741668563 | -1,154897679 | 0,413229116 | |
| 240595_at | - | -0,741668563 | -1,154897679 | 0,413229116 | |
| 242049_s_at | NBAS | -0,741668563 | -1,154897679 | 0,413229116 | |
| 226467_at | TMCO7 | 1,659347917 | 1,246149888 | 0,413198029 | |
| 220865_s_at | PDSS1 | 3,097712429 | 2,684536601 | 0,413175828 | |
| 1559926_at | LOC728353 | -0,85085127 | -1,264005341 | 0,413154071 | |
| 205440_s_at | NPY1R | -0,85085127 | -1,264005341 | 0,413154071 | |
| 206433_s_at | SPOCK3 | -0,85085127 | -1,264005341 | 0,413154071 | |
| 206916_x_at | TAT | -0,85085127 | -1,264005341 | 0,413154071 | |
| 211803_at | CDK2 | -0,85085127 | -1,264005341 | 0,413154071 | |
| 211848_s_at | CEACAM7 | -0,85085127 | -1,264005341 | 0,413154071 | |
| 212258_s_at | SMARCA2 | -0,85085127 | -1,264005341 | 0,413154071 | |
| 213060_s_at | CHI3L2 | -0,85085127 | -1,264005341 | 0,413154071 | |
| 215415_s_at | LYST | -0,85085127 | -1,264005341 | 0,413154071 | |
| 224454_at | ETNK1 | -0,85085127 | -1,264005341 | 0,413154071 | |
| 231562_at | APOC2 | -0,85085127 | -1,264005341 | 0,413154071 | |
| 233890_at | - | -0,85085127 | -1,264005341 | 0,413154071 | |

| | | | | | |
|---|---|---|---|---|---|
| 202518_at | BCL7B | 2,458724341 | 2,045615873 | 0,413108468 | |
| 222586_s_at | OSBPL11 | 1,373456798 | 0,960386719 | 0,413070079 | |
| 212958_x_at | PAM | 4,340094954 | 3,927027853 | 0,413067101 | |
| 218641_at | C11orf95 | 2,491678455 | 2,078620836 | 0,41305762 | |
| 227193_at | SLC30A4 | 0,57058931 | 0,157549243 | 0,413040067 | |
| 205003_at | DOCK4 | 1,442503549 | 1,029536893 | 0,412966655 | |
| 1558828_s_at | - | -2,823166348 | -3,236070201 | 0,412903853 | |
| 207869_s_at | CACNA1G | -2,823166348 | -3,236070201 | 0,412903853 | |
| 216533_at | PCCA | -2,823166348 | -3,236070201 | 0,412903853 | |
| 215845_x_at | - | 1,426552034 | 1,013870809 | 0,412681225 | |
| 225397_at | C15orf57 | 1,426552034 | 1,013870809 | 0,412681225 | |
| 1555288_s_at | FBF1 | -0,078265071 | -0,490927335 | 0,412662264 | |
| 228758_at | BCL6 | -0,078265071 | -0,490927335 | 0,412662264 | |
| 233496_s_at | CFL2 | -0,078265071 | -0,490927335 | 0,412662264 | |
| 1553466_at | CXorf59 | -0,352951799 | -0,765504029 | 0,41255223 | |
| 1568781_at | - | -0,352951799 | -0,765504029 | 0,41255223 | |
| 203901_at | TAB1 | -0,352951799 | -0,765504029 | 0,41255223 | |
| 223508_at | NOTCH1 | -0,352951799 | -0,765504029 | 0,41255223 | |
| 232670_at | - | -0,352951799 | -0,765504029 | 0,41255223 | |
| 213926_s_at | AGFG1 | 1,556829052 | 1,144305291 | 0,412523761 | |
| 228389_at | - | 0,541261317 | 0,128747141 | 0,412514176 | |
| 1554973_a_at | ZBTB26 | -0,234278768 | -0,646746079 | 0,412467311 | |
| 1561542_at | - | -0,234278768 | -0,646746079 | 0,412467311 | |
| 204861_s_at | LOC100509323 / | -0,234278768 | -0,646746079 | 0,412467311 | |
| 207609_s_at | CYP1A2 | -0,234278768 | -0,646746079 | 0,412467311 | |
| 214783_s_at | ANXA11 | -0,234278768 | -0,646746079 | 0,412467311 | |
| 220389_at | CCDC81 | -0,234278768 | -0,646746079 | 0,412467311 | |
| 226613_at | GATSL3 | -0,234278768 | -0,646746079 | 0,412467311 | |
| 229605_at | CYB5RL | -0,234278768 | -0,646746079 | 0,412467311 | |
| 238126_at | RSBN1L-AS1 | -0,234278768 | -0,646746079 | 0,412467311 | |
| 222462_s_at | BACE1 | 1,542099044 | 1,129843209 | 0,412255835 | |
| 201823_s_at | RNF14 | 3,58196103 | 3,170070041 | 0,411890989 | |
| 225754_at | AP1G1 | 2,361396186 | 1,950068772 | 0,411327414 | |
| 223946_at | MED23 | 0,020670649 | -0,39061235 | 0,411283 | |
| 226484_at | ZBTB47 | 0,020670649 | -0,39061235 | 0,411283 | |
| 233918_at | DCDC2B | 0,020670649 | -0,39061235 | 0,411283 | |
| 238911_at | STARD10 | 0,020670649 | -0,39061235 | 0,411283 | |
| 221108_at | C22orf43 | 0,82905153 | 0,417770464 | 0,411281067 | |
| 212047_s_at | RNF167 | 2,987866617 | 2,576704359 | 0,411162257 | |
| 1557697_at | - | -1,131062212 | -1,541908042 | 0,410845829 | |
| 1558517_s_at | LRRC8C | -1,131062212 | -1,541908042 | 0,410845829 | |
| 1562326_at | FLJ30838 | -1,131062212 | -1,541908042 | 0,410845829 | |
| 1566728_at | - | -1,131062212 | -1,541908042 | 0,410845829 | |
| 201506_at | LOC100652886 / | -1,131062212 | -1,541908042 | 0,410845829 | |
| 206286_s_at | TDGF1 /// TDGF | -1,131062212 | -1,541908042 | 0,410845829 | |
| 206367_at | REN | -1,131062212 | -1,541908042 | 0,410845829 | |
| 208584_at | SNCG | -1,131062212 | -1,541908042 | 0,410845829 | |
| 213697_at | HIPK3 | -1,131062212 | -1,541908042 | 0,410845829 | |
| 216798_at | FLJ23519 /// RNH | -1,131062212 | -1,541908042 | 0,410845829 | |
| 217128_s_at | CAMK1G | -1,131062212 | -1,541908042 | 0,410845829 | |
| 231055_at | - | -1,131062212 | -1,541908042 | 0,410845829 | |
| 231791_at | ASAH2B | -1,131062212 | -1,541908042 | 0,410845829 | |
| 232449_at | BCO2 | -1,131062212 | -1,541908042 | 0,410845829 | |
| 236118_at | LOC100128893 | -1,131062212 | -1,541908042 | 0,410845829 | |
| 52285_f_at | CEP76 | 3,292359144 | 2,881550906 | 0,410808238 | |
| 225587_at | TMEM129 | 0,30867765 | -0,102100538 | 0,410778187 | |

| | | | | | |
|---|---|---|---|---|---|
| 240793_at | TTN | 0,30867765 | -0,102100538 | 0,410778187 | |
| 1553062_at | MOGAT1 | 0,152582607 | -0,258114234 | 0,410696841 | |
| 214300_s_at | TOP3A | 0,152582607 | -0,258114234 | 0,410696841 | |
| 214759_at | WTAP | 0,152582607 | -0,258114234 | 0,410696841 | |
| 200762_at | DPYSL2 | 4,553887643 | 4,143455947 | 0,410431696 | |
| 1556812_a_at | - | -2,479842156 | -2,89013181 | 0,410289654 | |
| 1559018_at | PTPRE | -2,479842156 | -2,89013181 | 0,410289654 | |
| 1566843_at | PER4 | -2,479842156 | -2,89013181 | 0,410289654 | |
| 1567304_at | - | -2,479842156 | -2,89013181 | 0,410289654 | |
| 1570152_at | - | -2,479842156 | -2,89013181 | 0,410289654 | |
| 207424_at | MYF5 | -2,479842156 | -2,89013181 | 0,410289654 | |
| 216590_at | GNAT3 | -2,479842156 | -2,89013181 | 0,410289654 | |
| 217589_at | RAB40A | -2,479842156 | -2,89013181 | 0,410289654 | |
| 229823_at | RIMS2 | -2,479842156 | -2,89013181 | 0,410289654 | |
| 234330_at | - | -2,479842156 | -2,89013181 | 0,410289654 | |
| 234510_at | - | -2,479842156 | -2,89013181 | 0,410289654 | |
| 237812_at | - | -2,479842156 | -2,89013181 | 0,410289654 | |
| 243060_at | LOC100505835 | -2,479842156 | -2,89013181 | 0,410289654 | |
| 1553106_at | C5orf24 | 0,273439642 | -0,136643983 | 0,410083625 | |
| 215099_s_at | RXRB | 0,273439642 | -0,136643983 | 0,410083625 | |
| 229645_at | FAM69C | 0,273439642 | -0,136643983 | 0,410083625 | |
| 236270_at | NFATC4 | 0,273439642 | -0,136643983 | 0,410083625 | |
| 219174_at | IFT74 | 1,726031811 | 1,315948728 | 0,410083082 | |
| 219555_s_at | CENPN | 4,373240364 | 3,963505394 | 0,40973497 | |
| 1555380_at | ADAMTS4 | 0,518867317 | 0,109221392 | 0,409645925 | |
| 229349_at | LIN28B | 5,17304712 | 4,763431224 | 0,409615896 | |
| 238973_s_at | TSNAX | 0,384950573 | -0,024572586 | 0,40952316 | |
| 213513_x_at | ARPC2 | 5,124433928 | 4,715003086 | 0,409430842 | |
| 201653_at | CNIH | 6,191486349 | 5,78209163 | 0,40939472 | |
| 1558164_s_at | PEX13 | 0,613492853 | 0,204310755 | 0,409182098 | |
| 222111_at | FAM63B | 1,314003737 | 0,904843258 | 0,409160478 | |
| 237465_at | USP53 | 0,702292136 | 0,293518836 | 0,408773301 | |
| 1554464_a_at | CRTAP /// LOC1( | 2,688444421 | 2,27978304 | 0,408661381 | |
| 218989_x_at | SLC30A5 | 3,857278636 | 3,448769219 | 0,408509417 | |
| 223346_at | VPS18 | 1,58584542 | 1,177496821 | 0,4083486 | |
| 212322_at | SGPL1 | 1,523472489 | 1,115234684 | 0,408237805 | |
| 206338_at | ELAVL3 | -0,543051993 | -0,950686014 | 0,407634021 | |
| 236406_at | ZNF324B | -0,543051993 | -0,950686014 | 0,407634021 | |
| 237027_at | LSAMP-AS1 | -0,543051993 | -0,950686014 | 0,407634021 | |
| 241362_at | SOGA1 | -0,543051993 | -0,950686014 | 0,407634021 | |
| 206528_at | TRPC6 | -2,866296344 | -3,273861381 | 0,407565037 | |
| 236179_at | CDH11 | -2,866296344 | -3,273861381 | 0,407565037 | |
| 214500_at | H2AFY | 1,163336153 | 0,755840599 | 0,407495554 | |
| 219125_s_at | SLC50A1 | 2,338135744 | 1,930732138 | 0,407403606 | |
| 215541_s_at | DIAPH1 | 2,427005622 | 2,019703483 | 0,407302139 | |
| 223793_at | PRRC2B | 1,079110292 | 0,67181667 | 0,407293622 | |
| 223418_x_at | ANKRD13C | 2,452829957 | 2,045615873 | 0,407214084 | |
| 201873_s_at | ABCE1 | 4,914950325 | 4,507854821 | 0,407095503 | |
| 210892_s_at | GTF2I | 1,575032557 | 1,16809119 | 0,406941366 | |
| 1557246_at | KIDINS220 | 0,123193849 | -0,283667828 | 0,406861677 | |
| 206885_x_at | GH1 | 0,123193849 | -0,283667828 | 0,406861677 | |
| 216934_at | - | 0,123193849 | -0,283667828 | 0,406861677 | |
| 243138_at | - | 0,123193849 | -0,283667828 | 0,406861677 | |
| 203362_s_at | MAD2L1 | 5,721829387 | 5,315121782 | 0,406707606 | |
| 222273_at | PAPOLG | 1,607231019 | 1,200746069 | 0,40648495 | |
| 222849_s_at | SCRN3 | 2,637386731 | 2,230931691 | 0,40645504 | |

| | | | | | |
|---|---|---|---|---|---|
| 1565731_at | ALDH3B1 | 1,496984567 | 1,090553537 | 0,40643103 | |
| 220894_x_at | PRDM12 | 0,359971644 | -0,046302147 | 0,406273791 | |
| 223790_at | KATNAL1 | 0,359971644 | -0,046302147 | 0,406273791 | |
| 235375_x_at | TTC9B | 0,359971644 | -0,046302147 | 0,406273791 | |
| 215483_at | AKAP9 | 0,46522187 | 0,059218869 | 0,406003002 | |
| 235679_at | - | 0,46522187 | 0,059218869 | 0,406003002 | |
| 1554512_a_at | CEP89 | 0,083054335 | -0,322870281 | 0,405924616 | |
| 1566457_at | - | -0,706943342 | -1,112851844 | 0,405908502 | |
| 204043_at | TCN2 | -0,706943342 | -1,112851844 | 0,405908502 | |
| 212122_at | RHOQ | -0,706943342 | -1,112851844 | 0,405908502 | |
| 220888_s_at | CASS4 | -0,706943342 | -1,112851844 | 0,405908502 | |
| 221272_s_at | C1orf21 | -0,706943342 | -1,112851844 | 0,405908502 | |
| 223958_s_at | DNAL1 | -0,706943342 | -1,112851844 | 0,405908502 | |
| 234386_s_at | - | -0,706943342 | -1,112851844 | 0,405908502 | |
| 203958_s_at | ZBTB40 | -0,055687482 | -0,461594427 | 0,405906945 | |
| 221355_at | CHRNG | -0,055687482 | -0,461594427 | 0,405906945 | |
| 227191_at | ITFG1 | -0,055687482 | -0,461594427 | 0,405906945 | |
| 239824_s_at | TMEM107 | -0,055687482 | -0,461594427 | 0,405906945 | |
| 61734_at | RCN3 | 0,343075573 | -0,062816928 | 0,405892501 | |
| 205241_at | SCO2 | 4,056345228 | 3,650516168 | 0,405829061 | |
| 201504_s_at | TSN | 3,559280583 | 3,153484076 | 0,405796508 | |
| 213188_s_at | MINA | 2,940912947 | 2,53523821 | 0,405674737 | |
| 1556154_a_at | MGC23284 | -1,089016378 | -1,494668682 | 0,405652304 | |
| 1557540_at | LOC100507403 | -1,089016378 | -1,494668682 | 0,405652304 | |
| 1562392_at | MRPL23 | -1,089016378 | -1,494668682 | 0,405652304 | |
| 1568601_at | GAD2 | -1,089016378 | -1,494668682 | 0,405652304 | |
| 205940_at | MYH3 | -1,089016378 | -1,494668682 | 0,405652304 | |
| 206776_x_at | ACRV1 | -1,089016378 | -1,494668682 | 0,405652304 | |
| 210103_s_at | FOXA2 | -1,089016378 | -1,494668682 | 0,405652304 | |
| 211020_at | GCNT2 | -1,089016378 | -1,494668682 | 0,405652304 | |
| 220354_at | MCF2L-AS1 | -1,089016378 | -1,494668682 | 0,405652304 | |
| 222004_s_at | DOCK6 | -1,089016378 | -1,494668682 | 0,405652304 | |
| 233374_at | - | -1,089016378 | -1,494668682 | 0,405652304 | |
| 233664_at | - | -1,089016378 | -1,494668682 | 0,405652304 | |
| 241521_at | LOC100507652 | -1,089016378 | -1,494668682 | 0,405652304 | |
| 243134_at | - | -1,089016378 | -1,494668682 | 0,405652304 | |
| 242470_at | EID2B | 1,326951551 | 0,921731975 | 0,405219575 | |
| 222149_x_at | GOLGA8C /// GC | 0,822971223 | 0,417770464 | 0,40520076 | |
| 212594_at | MIR4680 /// PDC | 1,874520207 | 1,469325098 | 0,405195109 | |
| 1552343_s_at | PDE7A | -0,926850548 | -1,332017329 | 0,405166781 | |
| 202765_s_at | FBN1 | -0,926850548 | -1,332017329 | 0,405166781 | |
| 205058_at | SLC26A1 | -0,926850548 | -1,332017329 | 0,405166781 | |
| 207690_at | ALX3 | -0,926850548 | -1,332017329 | 0,405166781 | |
| 220038_at | C8orf44-SGK3 /// | -0,926850548 | -1,332017329 | 0,405166781 | |
| 220472_at | ZCCHC4 | -0,926850548 | -1,332017329 | 0,405166781 | |
| 1555977_at | MYL12A | 0,900083947 | 0,495031851 | 0,405052097 | |
| 1568683_at | MGC23284 | -0,325749314 | -0,730778808 | 0,405029494 | |
| 219554_at | RHCG | -0,325749314 | -0,730778808 | 0,405029494 | |
| 223409_at | - | -0,325749314 | -0,730778808 | 0,405029494 | |
| 223715_at | BRSK2 | -0,325749314 | -0,730778808 | 0,405029494 | |
| 242214_at | RPS27A | 1,64218396 | 1,23718272 | 0,405001241 | |
| 220968_s_at | TSPAN9 | 1,251994932 | 0,847075464 | 0,404919469 | |
| 202459_s_at | LPIN2 | 0,97328325 | 0,568365095 | 0,404918155 | |
| 201963_at | ACSL1 | 0,627515113 | 0,222599095 | 0,404916018 | |
| 212846_at | RRP1B | 6,720728761 | 6,315817527 | 0,404911234 | |
| 228543_at | PET117 | 3,214953914 | 2,810785576 | 0,404168338 | |

| | | | | | |
|---|---|---|---|---|---|
| 227278_at | TAF13 | 5,088452866 | 4,684404159 | 0,404048707 | |
| 232235_at | DSEL | 1,865674241 | 1,461646549 | 0,404027692 | |
| 227801_at | TRIM59 | 3,434229569 | 3,030417996 | 0,403811573 | |
| 1558626_at | - | -2,551294263 | -2,954576755 | 0,403282492 | |
| 1560517_s_at | - | -2,551294263 | -2,954576755 | 0,403282492 | |
| 219196_at | SCG3 | -2,551294263 | -2,954576755 | 0,403282492 | |
| 229281_at | NPAS3 | -2,551294263 | -2,954576755 | 0,403282492 | |
| 232905_at | GTF2H5 | -2,551294263 | -2,954576755 | 0,403282492 | |
| 226635_at | LOC100506710 | 4,608815095 | 4,205686812 | 0,403128283 | |
| 233810_x_at | - | 1,296557217 | 0,893473268 | 0,403083949 | |
| 219384_s_at | ADAT1 | 1,352503698 | 0,949447784 | 0,403055915 | |
| 212990_at | SYNJ1 | 2,325288363 | 1,922365006 | 0,402923357 | |
| 1558027_s_at | PRKAB2 | 0,95671774 | 0,553993624 | 0,402724116 | |
| 235057_at | ITCH | 1,027166904 | 0,624460759 | 0,402706145 | |
| 1554351_a_at | TIPRL | 3,316904202 | 2,914267328 | 0,402636874 | |
| 204729_s_at | STX1A | 1,442503549 | 1,039887232 | 0,402616317 | |
| 1556207_a_at | LOC100652856 | -2,908843709 | -3,311072652 | 0,402228943 | |
| 210196_s_at | PSG1 | -2,908843709 | -3,311072652 | 0,402228943 | |
| 1556136_at | MYLK4 | 1,139070151 | 0,73688026 | 0,40218989 | |
| 223347_at | MUM1 | 0,859074518 | 0,456918297 | 0,402156221 | |
| 229007_at | LOC283788 | 0,779676066 | 0,377530647 | 0,402145419 | |
| 211527_x_at | VEGFA | 0,695652136 | 0,293518836 | 0,402133301 | |
| 226530_at | BMF | 0,695652136 | 0,293518836 | 0,402133301 | |
| 1553688_at | METTL6 | 0,181384709 | -0,220592223 | 0,401976933 | |
| 215088_s_at | SDHC | 3,595400101 | 3,193438316 | 0,401961785 | |
| 228062_at | NAP1L5 | 1,780386218 | 1,378453643 | 0,401932575 | |
| 207319_s_at | CDK13 | 0,052199481 | -0,34958478 | 0,401784261 | |
| 210693_at | SPPL2B | 0,052199481 | -0,34958478 | 0,401784261 | |
| 219705_at | QSER1 | 0,052199481 | -0,34958478 | 0,401784261 | |
| 232234_at | SLA2 | 0,052199481 | -0,34958478 | 0,401784261 | |
| 1554679_a_at | LAPTM4B | 5,37095686 | 4,96934727 | 0,40160959 | |
| 218944_at | PYCRL | 0,57782909 | 0,176436073 | 0,401393017 | |
| 235713_at | ALKBH8 | 0,57782909 | 0,176436073 | 0,401393017 | |
| 223358_s_at | PDE7A | 2,446911392 | 2,045615873 | 0,401295519 | |
| 1553947_at | EXOSC6 | 2,874852734 | 2,473581079 | 0,401271655 | |
| 208309_s_at | MALT1 | 0,376672223 | -0,024572586 | 0,40124481 | |
| 210577_at | CASR | 0,376672223 | -0,024572586 | 0,40124481 | |
| 230617_at | LOC100507675 | 0,376672223 | -0,024572586 | 0,40124481 | |
| 1564336_at | - | -0,089689931 | -0,490927335 | 0,401237404 | |
| 207123_s_at | MATN4 | -0,089689931 | -0,490927335 | 0,401237404 | |
| 232880_at | - | -0,089689931 | -0,490927335 | 0,401237404 | |
| 216689_x_at | ARHGAP1 | 1,515954005 | 1,115234684 | 0,400719321 | |
| 222631_at | LOC285540 /// PI | 2,889473182 | 2,488772006 | 0,400701176 | |
| 234405_s_at | PHAX | 2,516380342 | 2,115788273 | 0,400592069 | |
| 1554428_s_at | NLGN2 | 1,360921491 | 0,960386719 | 0,400534772 | |
| 222811_at | FTSJD1 | 2,864529541 | 2,464004877 | 0,400524664 | |
| 223380_s_at | LATS2 | 1,305306849 | 0,904843258 | 0,400463591 | |
| 201366_at | ANXA7 | 4,286420174 | 3,886022844 | 0,40039733 | |
| 205491_s_at | GJB3 | -0,512251684 | -0,912331589 | 0,400079904 | |
| 208862_s_at | CTNND1 /// TMX | -0,512251684 | -0,912331589 | 0,400079904 | |
| 213896_x_at | FAM149B1 | -0,512251684 | -0,912331589 | 0,400079904 | |
| 214137_at | PTPRJ | -0,512251684 | -0,912331589 | 0,400079904 | |
| 218390_s_at | FAM204A | -0,512251684 | -0,912331589 | 0,400079904 | |
| 227953_at | CMTM6 | -0,512251684 | -0,912331589 | 0,400079904 | |
| 244000_at | - | -0,512251684 | -0,912331589 | 0,400079904 | |
| 210790_s_at | SAR1A | 2,582657321 | 2,182705233 | 0,399952088 | |

| | | | | | |
|---|---|---|---|---|---|
| 202184_s_at | NUP133 | 4,198742362 | 3,798818761 | 0,399923602 | |
| 207765_s_at | FAM214B | 0,917308734 | 0,51742585 | 0,399882884 | |
| 221646_s_at | ZDHHC11 | -0,136301282 | -0,536087151 | 0,399785869 | |
| 231117_at | FAM181A | -0,136301282 | -0,536087151 | 0,399785869 | |
| 236802_at | - | -0,136301282 | -0,536087151 | 0,399785869 | |
| 239281_at | ZDHHC14 | -0,136301282 | -0,536087151 | 0,399785869 | |
| 242911_at | MED13L | -0,136301282 | -0,536087151 | 0,399785869 | |
| 203989_x_at | F2R | 5,136741656 | 4,737036893 | 0,399704763 | |
| 1555985_at | C17orf64 | -1,763254337 | -2,162639771 | 0,399385434 | |
| 1560014_s_at | PDXDC1 | -1,763254337 | -2,162639771 | 0,399385434 | |
| 1560162_at | FLJ40606 | -1,763254337 | -2,162639771 | 0,399385434 | |
| 1560276_at | LOC283403 | -1,763254337 | -2,162639771 | 0,399385434 | |
| 1570100_at | - | -1,763254337 | -2,162639771 | 0,399385434 | |
| 202992_at | C7 | -1,763254337 | -2,162639771 | 0,399385434 | |
| 205220_at | HCAR3 | -1,763254337 | -2,162639771 | 0,399385434 | |
| 205569_at | LAMP3 | -1,763254337 | -2,162639771 | 0,399385434 | |
| 207158_at | APOBEC1 | -1,763254337 | -2,162639771 | 0,399385434 | |
| 208461_at | HIC1 | -1,763254337 | -2,162639771 | 0,399385434 | |
| 208467_at | KLF12 | -1,763254337 | -2,162639771 | 0,399385434 | |
| 220021_at | TMC7 | -1,763254337 | -2,162639771 | 0,399385434 | |
| 232852_at | - | -1,763254337 | -2,162639771 | 0,399385434 | |
| 237644_at | - | -1,763254337 | -2,162639771 | 0,399385434 | |
| 241179_at | - | -1,763254337 | -2,162639771 | 0,399385434 | |
| 241971_at | - | -1,763254337 | -2,162639771 | 0,399385434 | |
| 221564_at | PRMT2 | 4,88279496 | 4,483415907 | 0,399379053 | |
| 1553311_at | C20orf197 | -1,812418401 | -2,211750327 | 0,399331926 | |
| 1559263_s_at | ZC3H12D | -1,812418401 | -2,211750327 | 0,399331926 | |
| 1563035_x_at | GPD1 | -1,812418401 | -2,211750327 | 0,399331926 | |
| 203788_s_at | SEMA3C | -1,812418401 | -2,211750327 | 0,399331926 | |
| 205030_at | FABP7 | -1,812418401 | -2,211750327 | 0,399331926 | |
| 213556_at | LOC390940 | -1,812418401 | -2,211750327 | 0,399331926 | |
| 214631_at | ZBTB33 | -1,812418401 | -2,211750327 | 0,399331926 | |
| 224384_s_at | RNF17 | -1,812418401 | -2,211750327 | 0,399331926 | |
| 227769_at | GPR27 | -1,812418401 | -2,211750327 | 0,399331926 | |
| 236874_at | - | -1,812418401 | -2,211750327 | 0,399331926 | |
| 238085_at | LOC100507653 | -1,812418401 | -2,211750327 | 0,399331926 | |
| 238457_at | DGKE | -1,812418401 | -2,211750327 | 0,399331926 | |
| 242527_at | - | -1,812418401 | -2,211750327 | 0,399331926 | |
| 239043_at | ZNF404 | 1,504602299 | 1,105412836 | 0,399189463 | |
| 1553689_s_at | METTL6 | 2,553576109 | 2,154405725 | 0,399170384 | |
| 227299_at | CCNI | 0,735041319 | 0,336136178 | 0,398905142 | |
| 1562217_at | FLJ34521 | -1,688127507 | -2,086984744 | 0,398857237 | |
| 1563168_at | NRG1-IT1 | -1,688127507 | -2,086984744 | 0,398857237 | |
| 204163_at | EMILIN1 | -1,688127507 | -2,086984744 | 0,398857237 | |
| 233169_at | ZNF350 | -1,688127507 | -2,086984744 | 0,398857237 | |
| 1555579_s_at | PTPRM | -2,597496523 | -2,996335703 | 0,39883918 | |
| 1557363_a_at | PHIP | -2,597496523 | -2,996335703 | 0,39883918 | |
| 1560063_a_at | - | -2,597496523 | -2,996335703 | 0,39883918 | |
| 1561239_at | - | -2,597496523 | -2,996335703 | 0,39883918 | |
| 1561721_a_at | C12orf40 | -2,597496523 | -2,996335703 | 0,39883918 | |
| 1568487_x_at | - | -2,597496523 | -2,996335703 | 0,39883918 | |
| 1569508_at | PRDM5 | -2,597496523 | -2,996335703 | 0,39883918 | |
| 203998_s_at | SYT1 | -2,597496523 | -2,996335703 | 0,39883918 | |
| 209469_at | GPM6A | -2,597496523 | -2,996335703 | 0,39883918 | |
| 214131_at | TXLNG2P | -2,597496523 | -2,996335703 | 0,39883918 | |
| 215665_at | - | -2,597496523 | -2,996335703 | 0,39883918 | |

| | | | | | |
|---|---|---|---|---|---|
| 234041_at | - | -2,597496523 | -2,996335703 | 0,39883918 | |
| 235050_at | SLC2A12 | -2,597496523 | -2,996335703 | 0,39883918 | |
| 239767_at | - | -2,597496523 | -2,996335703 | 0,39883918 | |
| 242815_x_at | - | -2,597496523 | -2,996335703 | 0,39883918 | |
| 243625_at | - | -2,597496523 | -2,996335703 | 0,39883918 | |
| 228229_at | ZNF526 | 1,348276312 | 0,949447784 | 0,398828528 | |
| 1569264_at | LOC400655 | -0,366776884 | -0,765504029 | 0,398727145 | |
| 202831_at | GPX2 | -0,366776884 | -0,765504029 | 0,398727145 | |
| 222334_at | C1orf186 /// LOC | -0,366776884 | -0,765504029 | 0,398727145 | |
| 222861_x_at | FBXO44 | -0,366776884 | -0,765504029 | 0,398727145 | |
| 223703_at | C10orf11 | -0,366776884 | -0,765504029 | 0,398727145 | |
| 237870_at | NQO2 | -0,366776884 | -0,765504029 | 0,398727145 | |
| 242124_at | CHST1 | -0,366776884 | -0,765504029 | 0,398727145 | |
| 205194_at | PSPH | 2,791679321 | 2,39311332 | 0,398566002 | |
| 204877_s_at | TAOK2 | 0,555999837 | 0,157549243 | 0,398450594 | |
| 235725_at | SMAD4 | 0,555999837 | 0,157549243 | 0,398450594 | |
| 1560209_at | - | -2,779459409 | -3,177802286 | 0,398342878 | |
| 215879_at | ITGB1 | -2,779459409 | -3,177802286 | 0,398342878 | |
| 217402_at | - | -2,779459409 | -3,177802286 | 0,398342878 | |
| 235831_at | - | -2,779459409 | -3,177802286 | 0,398342878 | |
| 243548_x_at | - | -2,779459409 | -3,177802286 | 0,398342878 | |
| 1554022_at | CCDC13 | -1,888254346 | -2,286583451 | 0,398329105 | |
| 1557402_at | - | -1,888254346 | -2,286583451 | 0,398329105 | |
| 1562713_a_at | NETO1 | -1,888254346 | -2,286583451 | 0,398329105 | |
| 1563743_at | C1orf180 | -1,888254346 | -2,286583451 | 0,398329105 | |
| 1565821_at | - | -1,888254346 | -2,286583451 | 0,398329105 | |
| 1568791_s_at | LOC100505518 | -1,888254346 | -2,286583451 | 0,398329105 | |
| 1569561_at | - | -1,888254346 | -2,286583451 | 0,398329105 | |
| 216161_at | SBNO1 | -1,888254346 | -2,286583451 | 0,398329105 | |
| 216319_at | - | -1,888254346 | -2,286583451 | 0,398329105 | |
| 216893_s_at | COL4A3 | -1,888254346 | -2,286583451 | 0,398329105 | |
| 220829_s_at | B3GALT1 | -1,888254346 | -2,286583451 | 0,398329105 | |
| 220833_at | - | -1,888254346 | -2,286583451 | 0,398329105 | |
| 221370_at | LOC100287163 / | -1,888254346 | -2,286583451 | 0,398329105 | |
| 221393_at | TAAR3 | -1,888254346 | -2,286583451 | 0,398329105 | |
| 228342_s_at | ALPK3 | -1,888254346 | -2,286583451 | 0,398329105 | |
| 231460_at | LOC100507511 | -1,888254346 | -2,286583451 | 0,398329105 | |
| 232363_at | - | -1,888254346 | -2,286583451 | 0,398329105 | |
| 239222_at | C9orf9 | -1,888254346 | -2,286583451 | 0,398329105 | |
| 240213_at | - | -1,888254346 | -2,286583451 | 0,398329105 | |
| 240510_at | NBN | -1,888254346 | -2,286583451 | 0,398329105 | |
| 207247_s_at | ZFX /// ZFY | 0,457392764 | 0,059218869 | 0,398173896 | |
| 218489_s_at | ALAD | 0,457392764 | 0,059218869 | 0,398173896 | |
| 226003_at | KIF21A | 1,215326747 | 0,817300337 | 0,39802641 | |
| 201687_s_at | API5 | 4,837141808 | 4,439136927 | 0,398004881 | |
| 213017_at | ABHD3 | 2,396622324 | 1,998633167 | 0,397989156 | |
| 1553429_at | FLJ31713 | -1,639579028 | -2,037551727 | 0,397972698 | |
| 1554292_a_at | UHRF1BP1L | -1,639579028 | -2,037551727 | 0,397972698 | |
| 1554409_at | LOC653375 /// W | -1,639579028 | -2,037551727 | 0,397972698 | |
| 1555189_a_at | TAT | -1,639579028 | -2,037551727 | 0,397972698 | |
| 1556425_a_at | LOC284219 | -1,639579028 | -2,037551727 | 0,397972698 | |
| 1566139_at | HOPX | -1,639579028 | -2,037551727 | 0,397972698 | |
| 1569859_at | - | -1,639579028 | -2,037551727 | 0,397972698 | |
| 205922_at | VNN2 | -1,639579028 | -2,037551727 | 0,397972698 | |
| 206598_at | INS | -1,639579028 | -2,037551727 | 0,397972698 | |
| 210299_s_at | FHL1 | -1,639579028 | -2,037551727 | 0,397972698 | |

| | | | | | |
|---|---|---|---|---|---|
| 210437_at | MAGEA9 /// MAG | -1,639579028 | -2,037551727 | 0,397972698 | |
| 214927_at | ITGBL1 | -1,639579028 | -2,037551727 | 0,397972698 | |
| 217467_at | - | -1,639579028 | -2,037551727 | 0,397972698 | |
| 222936_s_at | DESI2 | -1,639579028 | -2,037551727 | 0,397972698 | |
| 234819_at | - | -1,639579028 | -2,037551727 | 0,397972698 | |
| 237392_at | FGF14-IT1 | -1,639579028 | -2,037551727 | 0,397972698 | |
| 244472_at | TRABD2B | -1,639579028 | -2,037551727 | 0,397972698 | |
| 244776_at | - | -1,639579028 | -2,037551727 | 0,397972698 | |
| 230380_at | THAP2 | 1,153678675 | 0,755840599 | 0,397838076 | |
| 219248_at | THUMPD2 | 1,542099044 | 1,144305291 | 0,397793753 | |
| 226806_s_at | NFIA | 1,089278259 | 0,691645685 | 0,397632574 | |
| 207993_s_at | CHP1 | 1,575032557 | 1,177496821 | 0,397535736 | |
| 1552327_at | ARMCX4 | -1,937711681 | -2,335104118 | 0,397392437 | |
| 1563745_a_at | LOC283050 | -1,937711681 | -2,335104118 | 0,397392437 | |
| 216998_s_at | ADAM5P | -1,937711681 | -2,335104118 | 0,397392437 | |
| 222350_at | - | -1,937711681 | -2,335104118 | 0,397392437 | |
| 224067_at | - | -1,937711681 | -2,335104118 | 0,397392437 | |
| 234331_s_at | FAM84A | -1,937711681 | -2,335104118 | 0,397392437 | |
| 237975_at | LOC100505986 | -1,937711681 | -2,335104118 | 0,397392437 | |
| 237986_at | - | -1,937711681 | -2,335104118 | 0,397392437 | |
| 238081_at | WDFY3-AS2 | -1,937711681 | -2,335104118 | 0,397392437 | |
| 1553690_at | SGOL1 | 0,425644434 | 0,028364014 | 0,397280419 | |
| 207022_s_at | LDHC | 0,425644434 | 0,028364014 | 0,397280419 | |
| 213975_s_at | LYZ | 3,464815649 | 3,067567462 | 0,397248187 | |
| 1562329_at | CSMD1 | -3,07502285 | -3,472251727 | 0,397228877 | |
| 241296_at | - | -3,07502285 | -3,472251727 | 0,397228877 | |
| 224436_s_at | NIPSNAP3A | 3,891817836 | 3,49464046 | 0,397177376 | |
| 229787_s_at | - | 1,512180019 | 1,115234684 | 0,396945334 | |
| 204443_at | ARSA | 0,317354302 | -0,079522948 | 0,39687725 | |
| 241928_at | - | 0,317354302 | -0,079522948 | 0,39687725 | |
| 204251_s_at | CEP164 | 1,000476627 | 0,603679647 | 0,396796981 | |
| 213426_s_at | CAV2 | 1,000476627 | 0,603679647 | 0,396796981 | |
| 1553488_at | TEKT5 | -1,026571149 | -1,423348341 | 0,396777193 | |
| 205445_at | PRL | -1,026571149 | -1,423348341 | 0,396777193 | |
| 214572_s_at | INSL3 | -1,026571149 | -1,423348341 | 0,396777193 | |
| 220331_at | CYP46A1 | -1,026571149 | -1,423348341 | 0,396777193 | |
| 230100_x_at | PAK1 | -1,026571149 | -1,423348341 | 0,396777193 | |
| 233796_at | - | -1,026571149 | -1,423348341 | 0,396777193 | |
| 242455_at | POU3F2 | -1,026571149 | -1,423348341 | 0,396777193 | |
| 228091_at | STX17 | 1,318332599 | 0,921731975 | 0,396600624 | |
| 242006_at | LCA5 | 1,318332599 | 0,921731975 | 0,396600624 | |
| 238002_at | GOLIM4 | 3,098971025 | 2,702564916 | 0,396406109 | |
| 204904_at | GJA4 | 0,20027154 | -0,19610998 | 0,39638152 | |
| 231997_at | TBCEL | 0,20027154 | -0,19610998 | 0,39638152 | |
| 1556982_at | - | 0,853119696 | 0,456918297 | 0,396201399 | |
| 223491_at | COMMD2 | 2,972842302 | 2,576704359 | 0,396137943 | |
| 1561985_at | C14orf39 | -0,022466681 | -0,41858459 | 0,396117909 | |
| 217124_at | IQCE | -0,022466681 | -0,41858459 | 0,396117909 | |
| 227867_at | TRABD2A | -0,022466681 | -0,41858459 | 0,396117909 | |
| 230058_at | SDCCAG3 | -0,022466681 | -0,41858459 | 0,396117909 | |
| 236322_at | - | -0,022466681 | -0,41858459 | 0,396117909 | |
| 204242_s_at | ACOX3 | 1,201332287 | 0,805216064 | 0,396116223 | |
| 1564784_at | - | -0,423248115 | -0,819238336 | 0,395990221 | |
| 1569339_s_at | LOC100287558 | -0,423248115 | -0,819238336 | 0,395990221 | |
| 204620_s_at | VCAN | -0,423248115 | -0,819238336 | 0,395990221 | |
| 210047_at | SLC11A2 | -0,423248115 | -0,819238336 | 0,395990221 | |

| | | | | | |
|---|---|---|---|---|---|
| 215327_at | - | -0,423248115 | -0,819238336 | 0,395990221 | |
| 235136_at | ORMDL3 | -0,423248115 | -0,819238336 | 0,395990221 | |
| 236730_at | GIPC3 | -0,423248115 | -0,819238336 | 0,395990221 | |
| 239186_at | MGC39372 | -0,423248115 | -0,819238336 | 0,395990221 | |
| 241529_at | - | -0,423248115 | -0,819238336 | 0,395990221 | |
| 243973_at | - | -0,423248115 | -0,819238336 | 0,395990221 | |
| 212127_at | RANGAP1 | 2,201862497 | 1,805902329 | 0,395960167 | |
| 217975_at | WBP5 | 4,845789115 | 4,449849671 | 0,395939444 | |
| 209144_s_at | CBFA2T2 | 0,072842263 | -0,322870281 | 0,395712544 | |
| 228734_at | - | 0,072842263 | -0,322870281 | 0,395712544 | |
| 230390_at | - | 0,072842263 | -0,322870281 | 0,395712544 | |
| 236273_at | NBPF1 | 1,968405881 | 1,572742379 | 0,395663502 | |
| 1567014_s_at | CSNK2A1 /// CSN | 2,666540916 | 2,271023131 | 0,395517785 | |
| 1554199_at | PTPRO | -2,384869671 | -2,780313924 | 0,395444253 | |
| 1554438_at | KIAA1217 | -2,384869671 | -2,780313924 | 0,395444253 | |
| 1560034_a_at | - | -2,384869671 | -2,780313924 | 0,395444253 | |
| 1560981_a_at | PPARA | -2,384869671 | -2,780313924 | 0,395444253 | |
| 1563002_at | - | -2,384869671 | -2,780313924 | 0,395444253 | |
| 1565107_x_at | - | -2,384869671 | -2,780313924 | 0,395444253 | |
| 203029_s_at | PTPRN2 | -2,384869671 | -2,780313924 | 0,395444253 | |
| 215107_s_at | TTC22 | -2,384869671 | -2,780313924 | 0,395444253 | |
| 215751_at | - | -2,384869671 | -2,780313924 | 0,395444253 | |
| 216322_at | CD58 | -2,384869671 | -2,780313924 | 0,395444253 | |
| 231292_at | EID3 | -2,384869671 | -2,780313924 | 0,395444253 | |
| 233705_at | PACSIN2 | -2,384869671 | -2,780313924 | 0,395444253 | |
| 234732_s_at | EFCAB6 | -2,384869671 | -2,780313924 | 0,395444253 | |
| 237834_at | SNCAIP | -2,384869671 | -2,780313924 | 0,395444253 | |
| 239279_at | LOC730102 | -2,384869671 | -2,780313924 | 0,395444253 | |
| 239936_at | DLEU2 | -2,384869671 | -2,780313924 | 0,395444253 | |
| 240597_at | - | -2,384869671 | -2,780313924 | 0,395444253 | |
| 241657_at | - | -2,384869671 | -2,780313924 | 0,395444253 | |
| 244463_at | ADAM23 | -2,384869671 | -2,780313924 | 0,395444253 | |
| 57588_at | SLC24A3 | -2,173511597 | -2,568876131 | 0,395364534 | |
| 1555407_s_at | FGD3 | -0,759588698 | -1,154897679 | 0,395308981 | |
| 1555822_at | FAM138A /// FAM | -0,759588698 | -1,154897679 | 0,395308981 | |
| 1556409_a_at | - | -0,759588698 | -1,154897679 | 0,395308981 | |
| 1556687_a_at | CLDN10 | -0,759588698 | -1,154897679 | 0,395308981 | |
| 205179_s_at | ADAM8 | -0,759588698 | -1,154897679 | 0,395308981 | |
| 205831_at | CD2 | -0,759588698 | -1,154897679 | 0,395308981 | |
| 210472_at | MT1G | -0,759588698 | -1,154897679 | 0,395308981 | |
| 232986_at | ZNF233 | -0,759588698 | -1,154897679 | 0,395308981 | |
| 239029_at | TMCC1 | -0,759588698 | -1,154897679 | 0,395308981 | |
| 239165_at | - | -0,759588698 | -1,154897679 | 0,395308981 | |
| 240794_at | NPAS4 | -0,759588698 | -1,154897679 | 0,395308981 | |
| 238860_at | C6orf130 | 1,119359127 | 0,724100169 | 0,395258958 | |
| 1553229_at | ZNF572 | 1,163336153 | 0,768343791 | 0,394992363 | |
| 1553461_at | FAM9B | -2,01371626 | -2,408705138 | 0,394988877 | |
| 1553573_s_at | EFNA2 | -2,01371626 | -2,408705138 | 0,394988877 | |
| 1554147_s_at | C3orf15 /// LOC1 | -2,01371626 | -2,408705138 | 0,394988877 | |
| 1556362_at | - | -2,01371626 | -2,408705138 | 0,394988877 | |
| 1561633_at | HMGA2 | -2,01371626 | -2,408705138 | 0,394988877 | |
| 1563104_at | - | -2,01371626 | -2,408705138 | 0,394988877 | |
| 205962_at | PAK2 | -2,01371626 | -2,408705138 | 0,394988877 | |
| 207362_at | SLC30A4 | -2,01371626 | -2,408705138 | 0,394988877 | |
| 210572_at | PCDHA2 | -2,01371626 | -2,408705138 | 0,394988877 | |
| 220450_at | - | -2,01371626 | -2,408705138 | 0,394988877 | |

| | | | | | |
|---|---|---|---|---|---|
| 221720_s_at | - | -2,01371626 | -2,408705138 | 0,394988877 | |
| 231585_at | VPS13A | -2,01371626 | -2,408705138 | 0,394988877 | |
| 236966_at | ARMC8 | -2,01371626 | -2,408705138 | 0,394988877 | |
| 237815_at | - | -2,01371626 | -2,408705138 | 0,394988877 | |
| 244851_at | - | -2,01371626 | -2,408705138 | 0,394988877 | |
| 60794_f_at | ZNF814 | -1,663649063 | -2,058592585 | 0,394943523 | |
| 210220_at | FZD2 | 1,05337236 | 0,658444279 | 0,39492808 | |
| 212275_s_at | SRCAP | 1,05337236 | 0,658444279 | 0,39492808 | |
| 214875_x_at | APLP2 | 4,10413628 | 3,709248632 | 0,394887648 | |
| 227546_x_at | AURKAIP1 | 2,783874823 | 2,389070873 | 0,39480395 | |
| 218818_at | FHL3 | 2,691784869 | 2,297144887 | 0,394639983 | |
| 1559995_at | SAMD14 | -0,172274514 | -0,566887459 | 0,394612946 | |
| 215003_at | DGCR9 | -0,172274514 | -0,566887459 | 0,394612946 | |
| 229545_at | FERMT1 | -0,172274514 | -0,566887459 | 0,394612946 | |
| 233837_at | GGT1 | -0,172274514 | -0,566887459 | 0,394612946 | |
| 210145_at | PLA2G4A | 1,631786706 | 1,23718272 | 0,394603986 | |
| 240079_at | ZNF81 | 1,631786706 | 1,23718272 | 0,394603986 | |
| 235168_at | PIGM | 0,984222186 | 0,589657387 | 0,394564799 | |
| 221553_at | MAGT1 | 2,669932407 | 2,275409734 | 0,394522673 | |
| 209426_s_at | AMACR /// C1QT | 0,503742507 | 0,109221392 | 0,394521115 | |
| 235297_at | CELF1 | 0,503742507 | 0,109221392 | 0,394521115 | |
| 1569891_at | ATP5A1 | -0,655928373 | -1,050406615 | 0,394478242 | |
| 201868_s_at | TBL1X | -0,655928373 | -1,050406615 | 0,394478242 | |
| 211831_s_at | THPO | -0,655928373 | -1,050406615 | 0,394478242 | |
| 218376_s_at | MICAL1 | -0,655928373 | -1,050406615 | 0,394478242 | |
| 220244_at | LINC00312 | -0,655928373 | -1,050406615 | 0,394478242 | |
| 230368_at | ERF | -0,655928373 | -1,050406615 | 0,394478242 | |
| 231575_at | EGR4 | -0,655928373 | -1,050406615 | 0,394478242 | |
| 235325_at | SPG7 | -0,655928373 | -1,050406615 | 0,394478242 | |
| 236889_at | - | -0,655928373 | -1,050406615 | 0,394478242 | |
| 238135_at | AGTRAP | -0,655928373 | -1,050406615 | 0,394478242 | |
| 240786_at | NOTCH4 | -0,655928373 | -1,050406615 | 0,394478242 | |
| 226207_at | RILPL1 | 2,043538949 | 1,64909809 | 0,394440859 | |
| 213982_s_at | RABGAP1L | 2,122398022 | 1,728030156 | 0,394367866 | |
| 238669_at | PTGS1 | 1,58584542 | 1,191491281 | 0,394354139 | |
| 220631_at | OSGEPL1 | 1,764611101 | 1,370274469 | 0,394336631 | |
| 1553376_a_at | AXDND1 | -2,64318869 | -3,037500868 | 0,394312178 | |
| 1553582_a_at | SPAG11A /// SPA | -2,64318869 | -3,037500868 | 0,394312178 | |
| 1554831_x_at | ALS2CR11 | -2,64318869 | -3,037500868 | 0,394312178 | |
| 1559640_at | ANKFN1 | -2,64318869 | -3,037500868 | 0,394312178 | |
| 1560846_at | - | -2,64318869 | -3,037500868 | 0,394312178 | |
| 1562294_x_at | ANKRD30B | -2,64318869 | -3,037500868 | 0,394312178 | |
| 1563721_at | - | -2,64318869 | -3,037500868 | 0,394312178 | |
| 213910_at | IGFBP7 | -2,64318869 | -3,037500868 | 0,394312178 | |
| 238318_at | - | -2,64318869 | -3,037500868 | 0,394312178 | |
| 240089_at | - | -2,64318869 | -3,037500868 | 0,394312178 | |
| 242666_at | - | -2,64318869 | -3,037500868 | 0,394312178 | |
| 222703_s_at | YRDC | 4,268075451 | 3,873779903 | 0,394295548 | |
| 206463_s_at | DHRS2 | 0,57058931 | 0,176436073 | 0,394153237 | |
| 1553137_s_at | KLF11 | -1,518072576 | -1,912089812 | 0,394017236 | |
| 1555250_a_at | CPEB3 | -1,518072576 | -1,912089812 | 0,394017236 | |
| 1555587_at | PDZRN3 | -1,518072576 | -1,912089812 | 0,394017236 | |
| 1560615_a_at | - | -1,518072576 | -1,912089812 | 0,394017236 | |
| 1564886_at | - | -1,518072576 | -1,912089812 | 0,394017236 | |
| 1569690_at | CCDC36 | -1,518072576 | -1,912089812 | 0,394017236 | |
| 216225_at | - | -1,518072576 | -1,912089812 | 0,394017236 | |

| | | | | | |
|---|---|---|---|---|---|
| 216263_s_at | NGDN | -1,518072576 | -1,912089812 | 0,394017236 | |
| 217186_at | ZNF259P1 | -1,518072576 | -1,912089812 | 0,394017236 | |
| 220131_at | FXYD7 | -1,518072576 | -1,912089812 | 0,394017236 | |
| 220855_at | CLTC-IT1 | -1,518072576 | -1,912089812 | 0,394017236 | |
| 228699_at | NRP2 | -1,518072576 | -1,912089812 | 0,394017236 | |
| 230400_s_at | PKN2 | -1,518072576 | -1,912089812 | 0,394017236 | |
| 232191_at | ERVH48-1 | -1,518072576 | -1,912089812 | 0,394017236 | |
| 234933_at | CC2D2A | -1,518072576 | -1,912089812 | 0,394017236 | |
| 239818_x_at | TRIB1 | -1,518072576 | -1,912089812 | 0,394017236 | |
| 244490_at | - | -1,518072576 | -1,912089812 | 0,394017236 | |
| 226447_at | ASH1L | 1,751865623 | 1,3579181 | 0,393947522 | |
| 217001_x_at | HLA-DOA | 0,401366113 | 0,007421914 | 0,393944199 | |
| 228525_at | LRP3 | 0,401366113 | 0,007421914 | 0,393944199 | |
| 236208_at | MOCS2 | 0,401366113 | 0,007421914 | 0,393944199 | |
| 1560911_at | LOC100133461 | -0,285837228 | -0,679763839 | 0,393926611 | |
| 222169_x_at | SH2D3A | -0,285837228 | -0,679763839 | 0,393926611 | |
| 229341_at | TFCP2L1 | -0,285837228 | -0,679763839 | 0,393926611 | |
| 230308_at | FAM120AOS | -0,285837228 | -0,679763839 | 0,393926611 | |
| 230756_at | ZNF683 | -0,285837228 | -0,679763839 | 0,393926611 | |
| 213497_at | ABTB2 | 1,937784532 | 1,543943227 | 0,393841304 | |
| 220363_s_at | ELMO2 | 1,210676994 | 0,817300337 | 0,393376657 | |
| 1560921_at | ZNF169 | 0,291166226 | -0,102100538 | 0,393266763 | |
| 205810_s_at | WASL | 0,291166226 | -0,102100538 | 0,393266763 | |
| 231877_at | TRMT10A | 0,291166226 | -0,102100538 | 0,393266763 | |
| 238639_x_at | - | 0,291166226 | -0,102100538 | 0,393266763 | |
| 1555544_a_at | CADM2 | -2,063149277 | -2,456406426 | 0,393257149 | |
| 1558845_at | LOC100506089 | -2,063149277 | -2,456406426 | 0,393257149 | |
| 1558894_a_at | CCDC67 | -2,063149277 | -2,456406426 | 0,393257149 | |
| 1562699_at | - | -2,063149277 | -2,456406426 | 0,393257149 | |
| 1570415_at | DDX52 | -2,063149277 | -2,456406426 | 0,393257149 | |
| 205225_at | ESR1 | -2,063149277 | -2,456406426 | 0,393257149 | |
| 206808_at | HNRNPA3P1 | -2,063149277 | -2,456406426 | 0,393257149 | |
| 207619_at | HCRTR1 | -2,063149277 | -2,456406426 | 0,393257149 | |
| 208495_at | TLX3 | -2,063149277 | -2,456406426 | 0,393257149 | |
| 210068_s_at | AQP4 | -2,063149277 | -2,456406426 | 0,393257149 | |
| 216831_s_at | RUNX1T1 | -2,063149277 | -2,456406426 | 0,393257149 | |
| 223784_at | TMEM27 | -2,063149277 | -2,456406426 | 0,393257149 | |
| 229881_at | KLF12 | -2,063149277 | -2,456406426 | 0,393257149 | |
| 230683_at | - | -2,063149277 | -2,456406426 | 0,393257149 | |
| 236522_at | - | -2,063149277 | -2,456406426 | 0,393257149 | |
| 237271_at | LOC154872 | -2,063149277 | -2,456406426 | 0,393257149 | |
| 239595_at | GPX2 | -2,063149277 | -2,456406426 | 0,393257149 | |
| 240560_at | - | -2,063149277 | -2,456406426 | 0,393257149 | |
| 242846_at | - | -2,063149277 | -2,456406426 | 0,393257149 | |
| 243202_at | - | -2,063149277 | -2,456406426 | 0,393257149 | |
| 200765_x_at | CTNNA1 | 3,537166687 | 3,14392006 | 0,393246626 | |
| 225338_at | ZYG11B | 3,196259441 | 2,803210557 | 0,393048884 | |
| 228606_at | TCTEX1D2 | 2,776027875 | 2,382985886 | 0,393041989 | |
| 205841_at | JAK2 | 1,422536444 | 1,029536893 | 0,392999551 | |
| 204104_at | SNAPC2 | 0,810733188 | 0,417770464 | 0,392962724 | |
| 220032_at | CPED1 | 0,810733188 | 0,417770464 | 0,392962724 | |
| 1559993_at | SFXN3 | -0,221656139 | -0,614371577 | 0,392715438 | |
| 212970_at | APBB2 | -0,221656139 | -0,614371577 | 0,392715438 | |
| 227992_s_at | LINC00085 | -0,221656139 | -0,614371577 | 0,392715438 | |
| 231765_at | ZFYVE20 | -0,221656139 | -0,614371577 | 0,392715438 | |
| 227772_at | LATS1 | 3,396853889 | 3,004228687 | 0,392625203 | |

| | | | | | |
|---|---|---|---|---|---|
| 222034_at | GNB2L1 /// LOC1 | 1,638726528 | 1,246149888 | 0,39257664 | |
| 224593_at | ZNF664 | 4,256842836 | 3,864347045 | 0,39249579 | |
| 215178_x_at | NAAA | 1,406361373 | 1,013870809 | 0,392490564 | |
| 1554113_a_at | SLC4A8 | 0,171847695 | -0,220592223 | 0,392439919 | |
| 213424_at | KIAA0895 | 0,171847695 | -0,220592223 | 0,392439919 | |
| 227832_at | MBD6 | 0,171847695 | -0,220592223 | 0,392439919 | |
| 239170_at | ACTR3 | 0,171847695 | -0,220592223 | 0,392439919 | |
| 244889_at | LOC388210 | 0,171847695 | -0,220592223 | 0,392439919 | |
| 208616_s_at | PTP4A2 | 6,7596472 | 6,367261321 | 0,39238588 | |
| 225835_at | SLC12A2 | 2,182996494 | 1,790660532 | 0,392335962 | |
| 203368_at | CRELD1 | 0,255492549 | -0,136643983 | 0,392136532 | |
| 234568_at | SCAMP5 | 0,255492549 | -0,136643983 | 0,392136532 | |
| 238893_at | LOC338758 | 0,255492549 | -0,136643983 | 0,392136532 | |
| 241277_x_at | ZNF837 | 0,255492549 | -0,136643983 | 0,392136532 | |
| 223463_at | RAB23 | 4,842787231 | 4,450819624 | 0,391967607 | |
| 222036_s_at | MCM4 | 4,650612883 | 4,258712848 | 0,391900035 | |
| 1566284_at | - | -1,470833215 | -1,862647763 | 0,391814547 | |
| 204563_at | SELL | -1,470833215 | -1,862647763 | 0,391814547 | |
| 205549_at | PCP4 | -1,470833215 | -1,862647763 | 0,391814547 | |
| 206193_s_at | CDSN | -1,470833215 | -1,862647763 | 0,391814547 | |
| 219430_at | GPR137 | -1,470833215 | -1,862647763 | 0,391814547 | |
| 220677_s_at | ADAMTS8 | -1,470833215 | -1,862647763 | 0,391814547 | |
| 228964_at | PRDM1 | -1,470833215 | -1,862647763 | 0,391814547 | |
| 230823_at | - | -1,470833215 | -1,862647763 | 0,391814547 | |
| 233345_at | EFCAB6 | -1,470833215 | -1,862647763 | 0,391814547 | |
| 234488_s_at | GMCL1 /// GMCL | 0,641402394 | 0,249604176 | 0,391798219 | |
| 224565_at | LOC100653017 / | 3,67603922 | 3,284389368 | 0,391649852 | |
| 201295_s_at | WSB1 | 0,702292136 | 0,310717132 | 0,391575004 | |
| 224036_s_at | LMBR1 | 2,611163891 | 2,21959457 | 0,391569321 | |
| 229711_s_at | MDM2 | 3,159334685 | 2,767843855 | 0,39149083 | |
| 229797_at | MCOLN3 | 0,995078803 | 0,603679647 | 0,391399156 | |
| 1553060_at | PSKH2 | 0,133056859 | -0,258114234 | 0,391171092 | |
| 237718_at | EIF4E | 0,133056859 | -0,258114234 | 0,391171092 | |
| 243007_at | TTC5 | 0,133056859 | -0,258114234 | 0,391171092 | |
| 209004_s_at | FBXL5 | 3,171349466 | 2,780243895 | 0,391105571 | |
| 1563065_at | - | -0,986356909 | -1,377419394 | 0,391062485 | |
| 1563483_at | FAM91A2 | -0,986356909 | -1,377419394 | 0,391062485 | |
| 1565825_at | - | -0,986356909 | -1,377419394 | 0,391062485 | |
| 207950_s_at | ANK3 | -0,986356909 | -1,377419394 | 0,391062485 | |
| 211329_x_at | HFE | -0,986356909 | -1,377419394 | 0,391062485 | |
| 214822_at | FAM5B | -0,986356909 | -1,377419394 | 0,391062485 | |
| 215206_at | - | -0,986356909 | -1,377419394 | 0,391062485 | |
| 233015_at | MBNL1 | -0,986356909 | -1,377419394 | 0,391062485 | |
| 238369_s_at | LOC100507186 | -0,986356909 | -1,377419394 | 0,391062485 | |
| 225970_at | DDHD1 | 2,402750389 | 2,011838132 | 0,390912257 | |
| 204156_at | SIK3 | 0,613492853 | 0,222599095 | 0,390893758 | |
| 227098_at | DUSP18 | 0,613492853 | 0,222599095 | 0,390893758 | |
| 208190_s_at | LSR | 1,177702228 | 0,786897721 | 0,390804506 | |
| 204285_s_at | PMAIP1 | 5,187613093 | 4,796999275 | 0,390613819 | |
| 213128_s_at | UBE3A | 3,668429488 | 3,277845403 | 0,390584085 | |
| 238738_at | - | 1,339784194 | 0,949447784 | 0,390336411 | |
| 200617_at | MLEC | 3,010782559 | 2,620464265 | 0,390318294 | |
| 212876_at | B4GALT4 | 2,320980353 | 1,930732138 | 0,390248215 | |
| 214588_s_at | MFAP3 | 0,518867317 | 0,128747141 | 0,390120176 | |
| 222660_s_at | RNF38 | 0,518867317 | 0,128747141 | 0,390120176 | |
| 201121_s_at | PGRMC1 | 4,935173991 | 4,545117128 | 0,390056863 | |

| | | | | | |
|---|---|---|---|---|---|
| 227409_at | PPP1R3E | -0,00073712 | -0,39061235 | 0,38987523 | |
| 236477_at | PRPF40A | -0,00073712 | -0,39061235 | 0,38987523 | |
| 244376_at | METTL7A | -0,00073712 | -0,39061235 | 0,38987523 | |
| 235625_at | VPS41 | 1,926486359 | 1,536652708 | 0,389833652 | |
| 211721_s_at | ZNF551 | 1,780386218 | 1,390636111 | 0,389750107 | |
| 213434_at | STX2 | 1,649074067 | 1,259496994 | 0,389577072 | |
| 224444_s_at | LINC00467 | 0,767063567 | 0,377530647 | 0,38953292 | |
| 1563639_a_at | FHAD1 | 1,504602299 | 1,115234684 | 0,389367615 | |
| 201309_x_at | NREP | 2,139623739 | 1,750261389 | 0,38936235 | |
| 1562211_a_at | ZNF491 | 0,417596971 | 0,028364014 | 0,389232956 | |
| 238146_at | - | 0,417596971 | 0,028364014 | 0,389232956 | |
| 221995_s_at | - | 1,027166904 | 0,638150376 | 0,389016528 | |
| 202611_s_at | MED14 | 2,696781084 | 2,307890888 | 0,388890196 | |
| 223062_s_at | PSAT1 | 4,377913865 | 3,989102799 | 0,388811067 | |
| 225062_at | LOC389831 | 2,472384965 | 2,083632167 | 0,388752798 | |
| 234726_s_at | TMEM168 | 1,556829052 | 1,16809119 | 0,388737861 | |
| 1560978_at | - | -0,467091869 | -0,855751026 | 0,388659157 | |
| 223955_at | EFCAB4B | -0,467091869 | -0,855751026 | 0,388659157 | |
| 235814_at | - | -0,467091869 | -0,855751026 | 0,388659157 | |
| 239552_at | VWDE | -0,467091869 | -0,855751026 | 0,388659157 | |
| 1561025_at | - | -3,013665402 | -3,402185466 | 0,388520064 | |
| 229308_at | ANKRD29 | -3,013665402 | -3,402185466 | 0,388520064 | |
| 243273_at | - | -3,013665402 | -3,402185466 | 0,388520064 | |
| 220261_s_at | ZDHHC4 | 2,652894659 | 2,264418116 | 0,388476542 | |
| 242584_at | FAM161A | 1,40228911 | 1,013870809 | 0,388418301 | |
| 208652_at | PPP2CA | 5,773977476 | 5,38567249 | 0,388304986 | |
| 1555342_a_at | UNC5C | -0,83191556 | -1,220210588 | 0,388295028 | |
| 1556689_a_at | WNT4 | -0,83191556 | -1,220210588 | 0,388295028 | |
| 1562797_at | - | -0,83191556 | -1,220210588 | 0,388295028 | |
| 1565329_at | POLE4 | -0,83191556 | -1,220210588 | 0,388295028 | |
| 1566210_at | - | -0,83191556 | -1,220210588 | 0,388295028 | |
| 216363_at | - | -0,83191556 | -1,220210588 | 0,388295028 | |
| 221001_at | - | -0,83191556 | -1,220210588 | 0,388295028 | |
| 224106_at | - | -0,83191556 | -1,220210588 | 0,388295028 | |
| 229550_at | UNC79 | -0,83191556 | -1,220210588 | 0,388295028 | |
| 232703_at | GLUD1P7 | -0,83191556 | -1,220210588 | 0,388295028 | |
| 233248_at | - | -0,83191556 | -1,220210588 | 0,388295028 | |
| 242037_at | ASPH | -0,83191556 | -1,220210588 | 0,388295028 | |
| 243413_at | TTC30B | -0,83191556 | -1,220210588 | 0,388295028 | |
| 242145_at | - | 0,228146222 | -0,160136748 | 0,38828297 | |
| 212533_at | WEE1 | 4,695400025 | 4,307183466 | 0,388216559 | |
| 232490_s_at | PRUNE | 0,30867765 | -0,079522948 | 0,388200598 | |
| 223011_s_at | OCIAD1 | 5,321676926 | 4,933712985 | 0,387963942 | |
| 210761_s_at | GRB7 | -0,148196756 | -0,536087151 | 0,387890394 | |
| 225654_at | NSD1 | -0,148196756 | -0,536087151 | 0,387890394 | |
| 226491_x_at | PTBP1 | -0,148196756 | -0,536087151 | 0,387890394 | |
| 227088_at | PDE5A | -0,148196756 | -0,536087151 | 0,387890394 | |
| 230235_at | MCTS1 | -0,148196756 | -0,536087151 | 0,387890394 | |
| 234696_at | - | -0,148196756 | -0,536087151 | 0,387890394 | |
| 238806_at | - | -0,148196756 | -0,536087151 | 0,387890394 | |
| 221773_at | ELK3 | 2,663141434 | 2,275409734 | 0,3877317 | |
| 225476_at | GPANK1 | 1,62481334 | 1,23718272 | 0,38763062 | |
| 211552_s_at | ALDH4A1 | 0,882651021 | 0,495031851 | 0,38761917 | |
| 1567282_at | OR1J4 | -1,399512875 | -1,787089803 | 0,387576928 | |
| 1568830_at | IRAK3 | -1,399512875 | -1,787089803 | 0,387576928 | |
| 210507_s_at | AVIL | -1,399512875 | -1,787089803 | 0,387576928 | |

| | | | | | |
|---|---|---|---|---|---|
| 215480_at | KIAA0509 | -1,399512875 | -1,787089803 | 0,387576928 | |
| 223796_at | CNTNAP3 /// CN | -1,399512875 | -1,787089803 | 0,387576928 | |
| 231232_at | LOC100506125 | -1,399512875 | -1,787089803 | 0,387576928 | |
| 232650_at | C12orf65 | -1,399512875 | -1,787089803 | 0,387576928 | |
| 234049_at | FLJ10661 | -1,399512875 | -1,787089803 | 0,387576928 | |
| 1555225_at | C1orf43 | 2,261526912 | 1,874012195 | 0,387514717 | |
| 203495_at | LRRC14 | 1,079110292 | 0,691645685 | 0,387464607 | |
| 225327_at | FAM214A | 2,433006244 | 2,045615873 | 0,387390371 | |
| 226851_at | LYPLAL1 | 2,830080806 | 2,442710777 | 0,387370028 | |
| 1557476_at | PFN4 | -0,622910612 | -1,010192375 | 0,387281763 | |
| 1570567_at | - | -0,622910612 | -1,010192375 | 0,387281763 | |
| 209197_at | SYT11 | -0,622910612 | -1,010192375 | 0,387281763 | |
| 212311_at | SEL1L3 | -0,622910612 | -1,010192375 | 0,387281763 | |
| 216924_s_at | DRD2 | -0,622910612 | -1,010192375 | 0,387281763 | |
| 220506_at | GUCY1B2 | -0,622910612 | -1,010192375 | 0,387281763 | |
| 221156_x_at | CCPG1 | -0,622910612 | -1,010192375 | 0,387281763 | |
| 227966_s_at | CCDC74A /// CC | -0,622910612 | -1,010192375 | 0,387281763 | |
| 229191_at | TBCD | -0,622910612 | -1,010192375 | 0,387281763 | |
| 232086_at | PIK3C3 | -0,622910612 | -1,010192375 | 0,387281763 | |
| 235367_at | MYPN | -0,622910612 | -1,010192375 | 0,387281763 | |
| 237254_at | SLC5A11 | -0,622910612 | -1,010192375 | 0,387281763 | |
| 239907_at | - | -0,622910612 | -1,010192375 | 0,387281763 | |
| 242523_at | - | -0,622910612 | -1,010192375 | 0,387281763 | |
| 1553674_at | LRRIQ3 | -2,187914861 | -2,575129729 | 0,387214868 | |
| 1557444_at | TREML3P | -2,187914861 | -2,575129729 | 0,387214868 | |
| 1559812_at | - | -2,187914861 | -2,575129729 | 0,387214868 | |
| 1563219_at | - | -2,187914861 | -2,575129729 | 0,387214868 | |
| 1563498_s_at | SLC25A45 | -2,187914861 | -2,575129729 | 0,387214868 | |
| 1564337_at | - | -2,187914861 | -2,575129729 | 0,387214868 | |
| 1568635_at | - | -2,187914861 | -2,575129729 | 0,387214868 | |
| 1569518_at | - | -2,187914861 | -2,575129729 | 0,387214868 | |
| 1570163_at | EGFLAM-AS4 | -2,187914861 | -2,575129729 | 0,387214868 | |
| 205381_at | LRRC17 | -2,187914861 | -2,575129729 | 0,387214868 | |
| 207910_at | SCGB1D1 | -2,187914861 | -2,575129729 | 0,387214868 | |
| 211095_at | NF1 | -2,187914861 | -2,575129729 | 0,387214868 | |
| 211161_s_at | COL3A1 | -2,187914861 | -2,575129729 | 0,387214868 | |
| 224189_x_at | EHF | -2,187914861 | -2,575129729 | 0,387214868 | |
| 224423_x_at | PMCHL2 | -2,187914861 | -2,575129729 | 0,387214868 | |
| 233026_s_at | PDZD2 | -2,187914861 | -2,575129729 | 0,387214868 | |
| 234289_x_at | - | -2,187914861 | -2,575129729 | 0,387214868 | |
| 234652_at | - | -2,187914861 | -2,575129729 | 0,387214868 | |
| 236438_at | - | -2,187914861 | -2,575129729 | 0,387214868 | |
| 237549_at | - | -2,187914861 | -2,575129729 | 0,387214868 | |
| 241204_at | - | -2,187914861 | -2,575129729 | 0,387214868 | |
| 244039_x_at | - | -2,187914861 | -2,575129729 | 0,387214868 | |
| 218062_x_at | CDC42EP4 | 1,578645857 | 1,191491281 | 0,387154576 | |
| 211062_s_at | CPZ /// GPR78 | 0,103263601 | -0,283667828 | 0,386931429 | |
| 215114_at | SENP3 | 0,103263601 | -0,283667828 | 0,386931429 | |
| 215379_x_at | IGLV1-44 | 0,103263601 | -0,283667828 | 0,386931429 | |
| 230155_x_at | MSL1 | 0,103263601 | -0,283667828 | 0,386931429 | |
| 240677_at | - | 0,103263601 | -0,283667828 | 0,386931429 | |
| 210486_at | ANKMY1 | -0,259832361 | -0,646746079 | 0,386913717 | |
| 216175_at | - | -0,259832361 | -0,646746079 | 0,386913717 | |
| 237174_at | - | -0,259832361 | -0,646746079 | 0,386913717 | |
| 1563680_at | LOC284950 | -2,712026959 | -3,098858316 | 0,386831357 | |
| 216314_at | CRISP1 | -2,712026959 | -3,098858316 | 0,386831357 | |

| | | | | | |
|---|---|---|---|---|---|
| 229331_at | SPATA18 | -2,712026959 | -3,098858316 | 0,386831357 | |
| 231679_at | - | -2,712026959 | -3,098858316 | 0,386831357 | |
| 234628_at | - | -2,712026959 | -3,098858316 | 0,386831357 | |
| 236670_s_at | - | -2,712026959 | -3,098858316 | 0,386831357 | |
| 243896_at | WDR96 | -2,712026959 | -3,098858316 | 0,386831357 | |
| 228092_at | CREM | 1,233777334 | 0,847075464 | 0,38670187 | |
| 221482_s_at | ARPP19 | 3,999522577 | 3,6128809 | 0,386641678 | |
| 218470_at | YARS2 | 3,815781597 | 3,429217209 | 0,386564388 | |
| 206055_s_at | SNRPA1 | 5,773977476 | 5,387446626 | 0,38653085 | |
| 223269_at | POLR3GL | 3,850569363 | 3,464222256 | 0,386347107 | |
| 208436_s_at | IRF7 | 2,854131948 | 2,467842989 | 0,386288959 | |
| 224521_s_at | CCDC77 | 1,515954005 | 1,129843209 | 0,386110796 | |
| 235482_at | PCBP1-AS1 | 0,779676066 | 0,393761504 | 0,385914561 | |
| 212900_at | SEC24A | 3,345817457 | 2,959949103 | 0,385868355 | |
| 200086_s_at | COX4I1 | 5,931509779 | 5,545849527 | 0,385660251 | |
| 223441_at | SLC17A5 | 2,365585359 | 1,979940779 | 0,38564458 | |
| 1554933_at | PSIP1 | -2,504600601 | -2,89013181 | 0,385531209 | |
| 1556813_at | - | -2,504600601 | -2,89013181 | 0,385531209 | |
| 1564610_at | - | -2,504600601 | -2,89013181 | 0,385531209 | |
| 210173_at | PTPRJ | -2,504600601 | -2,89013181 | 0,385531209 | |
| 216258_s_at | SERPINB13 | -2,504600601 | -2,89013181 | 0,385531209 | |
| 233906_at | - | -2,504600601 | -2,89013181 | 0,385531209 | |
| 236570_at | ZNF366 | -2,504600601 | -2,89013181 | 0,385531209 | |
| 238702_at | SPTSSB | -2,504600601 | -2,89013181 | 0,385531209 | |
| 240637_at | WDR41 | -2,504600601 | -2,89013181 | 0,385531209 | |
| 242091_at | ZNF720 | -2,504600601 | -2,89013181 | 0,385531209 | |
| 1557078_at | SLFN5 | -0,946847173 | -1,332017329 | 0,385170156 | |
| 1560764_at | - | -0,946847173 | -1,332017329 | 0,385170156 | |
| 1567242_at | OR2L1P | -0,946847173 | -1,332017329 | 0,385170156 | |
| 210683_at | NRTN | -0,946847173 | -1,332017329 | 0,385170156 | |
| 213030_s_at | PLXNA2 | -0,946847173 | -1,332017329 | 0,385170156 | |
| 215079_at | - | -0,946847173 | -1,332017329 | 0,385170156 | |
| 216431_at | - | -0,946847173 | -1,332017329 | 0,385170156 | |
| 220556_at | ATP1B4 | -0,946847173 | -1,332017329 | 0,385170156 | |
| 228715_at | ZCCHC12 | -0,946847173 | -1,332017329 | 0,385170156 | |
| 231224_x_at | PRKAG2 | -0,946847173 | -1,332017329 | 0,385170156 | |
| 234495_at | KLK15 | -0,946847173 | -1,332017329 | 0,385170156 | |
| 237060_at | - | -0,946847173 | -1,332017329 | 0,385170156 | |
| 238078_at | SEC22A | -0,946847173 | -1,332017329 | 0,385170156 | |
| 238445_x_at | MGAT5B | -0,946847173 | -1,332017329 | 0,385170156 | |
| 202472_at | MPI | 0,917308734 | 0,532164371 | 0,385144363 | |
| 1555892_s_at | LOC253039 | -0,033456564 | -0,41858459 | 0,385128025 | |
| 212641_at | HIVEP2 | -0,033456564 | -0,41858459 | 0,385128025 | |
| 215461_at | ZNRF4 | -0,033456564 | -0,41858459 | 0,385128025 | |
| 228587_at | FAM83G | -0,033456564 | -0,41858459 | 0,385128025 | |
| 234187_at | - | -0,033456564 | -0,41858459 | 0,385128025 | |
| 242592_at | GPR137C | -0,033456564 | -0,41858459 | 0,385128025 | |
| 213923_at | RAP2B | 5,547270277 | 5,162298023 | 0,384972254 | |
| 243000_at | CDK6 | 0,695652136 | 0,310717132 | 0,384935004 | |
| 242428_at | - | 1,414471577 | 1,029536893 | 0,384934684 | |
| 1555194_at | - | -2,236765673 | -2,621331989 | 0,384566316 | |
| 1557004_at | LOC100505902 / | -2,236765673 | -2,621331989 | 0,384566316 | |
| 1559611_at | TMEM75 | -2,236765673 | -2,621331989 | 0,384566316 | |
| 1560887_a_at | - | -2,236765673 | -2,621331989 | 0,384566316 | |
| 1561212_at | - | -2,236765673 | -2,621331989 | 0,384566316 | |
| 1562380_at | - | -2,236765673 | -2,621331989 | 0,384566316 | |

| | | | | | |
|---|---|---|---|---|---|
| 1564078_at | ADAM29 | -2,236765673 | -2,621331989 | 0,384566316 | |
| 1565927_s_at | - | -2,236765673 | -2,621331989 | 0,384566316 | |
| 1567855_at | ZNF29P | -2,236765673 | -2,621331989 | 0,384566316 | |
| 1570215_at | - | -2,236765673 | -2,621331989 | 0,384566316 | |
| 204198_s_at | RUNX3 | -2,236765673 | -2,621331989 | 0,384566316 | |
| 206159_at | GDF10 | -2,236765673 | -2,621331989 | 0,384566316 | |
| 207284_s_at | ASPH | -2,236765673 | -2,621331989 | 0,384566316 | |
| 219935_at | ADAMTS5 | -2,236765673 | -2,621331989 | 0,384566316 | |
| 220667_at | USP49 | -2,236765673 | -2,621331989 | 0,384566316 | |
| 222253_s_at | POM121L9P | -2,236765673 | -2,621331989 | 0,384566316 | |
| 235675_at | DHFRL1 | -2,236765673 | -2,621331989 | 0,384566316 | |
| 237570_x_at | - | -2,236765673 | -2,621331989 | 0,384566316 | |
| 237957_at | - | -2,236765673 | -2,621331989 | 0,384566316 | |
| 238063_at | TMEM154 | -2,236765673 | -2,621331989 | 0,384566316 | |
| 242779_at | - | -2,236765673 | -2,621331989 | 0,384566316 | |
| 243507_s_at | C20orf196 | 1,089278259 | 0,704715219 | 0,38456304 | |
| 201741_x_at | SRSF1 | 0,359971644 | -0,024572586 | 0,38454423 | |
| 229801_at | C10orf47 | 0,359971644 | -0,024572586 | 0,38454423 | |
| 238545_at | BRD7 | 0,359971644 | -0,024572586 | 0,38454423 | |
| 228401_at | ATAD2 | 4,266956117 | 3,882432751 | 0,384523365 | |
| 204754_at | HLF | -1,353583928 | -1,738067356 | 0,384483428 | |
| 206634_at | SIX3 | -1,353583928 | -1,738067356 | 0,384483428 | |
| 207793_s_at | EPB41 | -1,353583928 | -1,738067356 | 0,384483428 | |
| 214979_at | - | -1,353583928 | -1,738067356 | 0,384483428 | |
| 224085_at | - | -1,353583928 | -1,738067356 | 0,384483428 | |
| 229029_at | CAMK4 | -1,353583928 | -1,738067356 | 0,384483428 | |
| 232111_at | LINC00617 | -1,353583928 | -1,738067356 | 0,384483428 | |
| 232232_s_at | SLC22A16 | -1,353583928 | -1,738067356 | 0,384483428 | |
| 237370_at | - | -1,353583928 | -1,738067356 | 0,384483428 | |
| 240732_at | - | -1,353583928 | -1,738067356 | 0,384483428 | |
| 241002_at | - | -1,353583928 | -1,738067356 | 0,384483428 | |
| 241558_at | - | -1,353583928 | -1,738067356 | 0,384483428 | |
| 212133_at | NIPA2 | 2,134723057 | 1,750261389 | 0,384461668 | |
| 1552510_at | SLC34A3 | 2,705069849 | 2,320681318 | 0,384388531 | |
| 228974_at | - | 2,705069849 | 2,320681318 | 0,384388531 | |
| 201745_at | TWF1 | 2,675004741 | 2,290658654 | 0,384346087 | |
| 238594_x_at | DUSP8 | 1,742232072 | 1,3579181 | 0,384313972 | |
| 218303_x_at | KRCC1 | 2,644299732 | 2,259997914 | 0,384301818 | |
| 205288_at | CDC14A | 0,841135803 | 0,456918297 | 0,384217506 | |
| 239873_at | - | 0,841135803 | 0,456918297 | 0,384217506 | |
| 1554177_a_at | ATP5S | 1,621313976 | 1,23718272 | 0,384131257 | |
| 203677_s_at | TARBP2 | 2,621710292 | 2,237691445 | 0,384018846 | |
| 219793_at | SNX16 | 0,541261317 | 0,157549243 | 0,383712074 | |
| 223744_s_at | SIAE | 0,541261317 | 0,157549243 | 0,383712074 | |
| 244251_at | LCP2 | 0,541261317 | 0,157549243 | 0,383712074 | |
| 221435_x_at | HYI | 1,305306849 | 0,921731975 | 0,383574874 | |
| 202945_at | FPGS | 1,575032557 | 1,191491281 | 0,383541276 | |
| 1559748_at | ADAMTSL3 | -0,078265071 | -0,461594427 | 0,383329355 | |
| 205752_s_at | GSTM5 | -0,078265071 | -0,461594427 | 0,383329355 | |
| 210440_s_at | CDC14A | -0,078265071 | -0,461594427 | 0,383329355 | |
| 221450_x_at | PCDHB13 | -0,078265071 | -0,461594427 | 0,383329355 | |
| 205198_s_at | ATP7A | 1,139070151 | 0,755840599 | 0,383229551 | |
| 229298_at | KBTBD7 | 0,760715727 | 0,377530647 | 0,38318508 | |
| 233729_at | - | 0,246434561 | -0,136643983 | 0,383078544 | |
| 242607_at | - | 0,246434561 | -0,136643983 | 0,383078544 | |
| 242743_at | IL4R | 0,246434561 | -0,136643983 | 0,383078544 | |

| | | | | | |
|---|---|---|---|---|---|
| 243001_at | RBFA | 0,246434561 | -0,136643983 | 0,383078544 | |
| 207358_x_at | MACF1 | 3,402980973 | 3,019999252 | 0,382981721 | |
| 224996_at | ASPH | 1,937784532 | 1,55481039 | 0,382974141 | |
| 204807_at | TMEM5 | 2,048758794 | 1,665902186 | 0,382856609 | |
| 202064_s_at | SEL1L | 1,247462054 | 0,864649967 | 0,382812086 | |
| 226688_at | C3orf23 | 1,247462054 | 0,864649967 | 0,382812086 | |
| 238700_at | PIAS2 | 1,247462054 | 0,864649967 | 0,382812086 | |
| 34846_at | CAMK2B | 1,650791465 | 1,268326995 | 0,38246447 | |
| 1559515_at | - | -2,756478458 | -3,138626879 | 0,382148421 | |
| 206627_s_at | SSX1 | -2,756478458 | -3,138626879 | 0,382148421 | |
| 236404_at | - | -2,756478458 | -3,138626879 | 0,382148421 | |
| 237884_x_at | TRPM7 | -2,756478458 | -3,138626879 | 0,382148421 | |
| 238673_at | SAMD12 | -2,756478458 | -3,138626879 | 0,382148421 | |
| 242604_at | - | -2,756478458 | -3,138626879 | 0,382148421 | |
| 228041_at | AASDH | 1,369290453 | 0,987376598 | 0,381913855 | |
| 1555105_a_at | MIER1 | 1,331241794 | 0,949447784 | 0,38179401 | |
| 222621_at | DNAJC1 | 1,331241794 | 0,949447784 | 0,38179401 | |
| 225107_at | HNRNPA2B1 | 2,967339888 | 2,585563171 | 0,381776717 | |
| 220681_at | C22orf26 | 1,05337236 | 0,67181667 | 0,38155569 | |
| 221220_s_at | SCYL2 | 1,05337236 | 0,67181667 | 0,38155569 | |
| 209073_s_at | NUMB | 2,830080806 | 2,448549499 | 0,381531307 | |
| 1554181_at | SNX32 | -0,43775896 | -0,819238336 | 0,381479376 | |
| 219735_s_at | TFCP2L1 | -0,43775896 | -0,819238336 | 0,381479376 | |
| 227381_at | CERCAM | -0,43775896 | -0,819238336 | 0,381479376 | |
| 234515_at | PCGEM1 | -0,43775896 | -0,819238336 | 0,381479376 | |
| 237438_at | - | -0,43775896 | -0,819238336 | 0,381479376 | |
| 241784_x_at | HELQ | -0,43775896 | -0,819238336 | 0,381479376 | |
| 201772_at | AZIN1 | 5,185541222 | 4,804227461 | 0,381313761 | |
| 1558897_at | PLK5 | 0,123193849 | -0,258114234 | 0,381308083 | |
| 220712_at | C8orf60 | 0,123193849 | -0,258114234 | 0,381308083 | |
| 236982_at | - | 0,123193849 | -0,258114234 | 0,381308083 | |
| 201980_s_at | RSU1 | 4,487686025 | 4,106388091 | 0,381297935 | |
| 221983_at | FAM134A | 2,472384965 | 2,091116676 | 0,381268289 | |
| 203793_x_at | PCGF2 | 1,168140757 | 0,786897721 | 0,381243036 | |
| 208782_at | FSTL1 | 1,168140757 | 0,786897721 | 0,381243036 | |
| 229026_at | - | 1,381753566 | 1,000684521 | 0,381069045 | |
| 226667_x_at | EPN1 | 0,648296226 | 0,26733076 | 0,380965466 | |
| 227036_at | RASAL2 | 0,648296226 | 0,26733076 | 0,380965466 | |
| 230766_at | GART | 2,201862497 | 1,820984782 | 0,380877715 | |
| 204450_x_at | APOA1 | 0,334552598 | -0,046302147 | 0,380854745 | |
| 221836_s_at | TRAPPC9 | 0,334552598 | -0,046302147 | 0,380854745 | |
| 216739_at | - | 0,03125738 | -0,34958478 | 0,38084216 | |
| 223760_s_at | - | 0,03125738 | -0,34958478 | 0,38084216 | |
| 228395_at | GLT8D1 | 0,03125738 | -0,34958478 | 0,38084216 | |
| 231741_at | S1PR3 | 0,03125738 | -0,34958478 | 0,38084216 | |
| 243150_at | - | 0,03125738 | -0,34958478 | 0,38084216 | |
| 225830_at | PDZD8 | 3,164152611 | 2,783327323 | 0,380825288 | |
| 1554741_s_at | FGF7 /// KGFLP1 | -0,299034815 | -0,679763839 | 0,380729024 | |
| 205639_at | AOAH | -0,299034815 | -0,679763839 | 0,380729024 | |
| 210107_at | CLCA1 | -0,299034815 | -0,679763839 | 0,380729024 | |
| 215268_at | KIAA0754 | -0,299034815 | -0,679763839 | 0,380729024 | |
| 220423_at | PLA2G2D | -0,299034815 | -0,679763839 | 0,380729024 | |
| 223628_at | TMEM191A | -0,299034815 | -0,679763839 | 0,380729024 | |
| 230714_s_at | - | -0,299034815 | -0,679763839 | 0,380729024 | |
| 239928_at | TCTN2 | -0,299034815 | -0,679763839 | 0,380729024 | |
| 244127_at | - | -0,299034815 | -0,679763839 | 0,380729024 | |

| | | | | | |
|---|---|---|---|---|---|
| 213409_s_at | RHEB | 0,798390451 | 0,417770464 | 0,380619987 | |
| 215009_s_at | THAP9-AS1 | 3,492853048 | 3,112391743 | 0,380461305 | |
| 1553052_at | WFDC13 | -2,930741289 | -3,311072652 | 0,380331363 | |
| 1562656_at | - | -2,930741289 | -3,311072652 | 0,380331363 | |
| 207703_at | NLGN4Y | -2,930741289 | -3,311072652 | 0,380331363 | |
| 238317_x_at | - | -2,930741289 | -3,311072652 | 0,380331363 | |
| 244403_at | CRB1 | -2,930741289 | -3,311072652 | 0,380331363 | |
| 223335_at | TMEM69 | 4,995887722 | 4,615576224 | 0,380311498 | |
| 211140_s_at | CASP2 | 1,960118853 | 1,579853228 | 0,380265625 | |
| 202932_at | YES1 | 3,938261899 | 3,558125564 | 0,380136335 | |
| 1558050_at | EIF2B5 | -0,234278768 | -0,614371577 | 0,380092809 | |
| 231987_at | MIR143HG | -0,234278768 | -0,614371577 | 0,380092809 | |
| 234928_x_at | RUNX3 | -0,234278768 | -0,614371577 | 0,380092809 | |
| 235070_at | RBFOX1 | -0,234278768 | -0,614371577 | 0,380092809 | |
| 232269_x_at | METRN | 1,215326747 | 0,835239052 | 0,380087695 | |
| 242895_x_at | - | 1,485482016 | 1,105412836 | 0,38006918 | |
| 201092_at | RBBP7 | 6,032283861 | 5,652346853 | 0,379937009 | |
| 207435_s_at | SRRM2 | 3,123915073 | 2,744302548 | 0,379612525 | |
| 1554196_at | IZUMO1 | -2,311268652 | -2,690860744 | 0,379592092 | |
| 1555547_at | - | -2,311268652 | -2,690860744 | 0,379592092 | |
| 1556257_at | LOC100507322 / | -2,311268652 | -2,690860744 | 0,379592092 | |
| 1561914_at | - | -2,311268652 | -2,690860744 | 0,379592092 | |
| 1562137_at | - | -2,311268652 | -2,690860744 | 0,379592092 | |
| 1569617_at | OSBP2 | -2,311268652 | -2,690860744 | 0,379592092 | |
| 216254_at | PARVB | -2,311268652 | -2,690860744 | 0,379592092 | |
| 229824_at | SHC3 | -2,311268652 | -2,690860744 | 0,379592092 | |
| 233724_at | ARNT | -2,311268652 | -2,690860744 | 0,379592092 | |
| 235442_at | CXorf56 | -2,311268652 | -2,690860744 | 0,379592092 | |
| 237918_at | - | -2,311268652 | -2,690860744 | 0,379592092 | |
| 240622_at | - | -2,311268652 | -2,690860744 | 0,379592092 | |
| 241320_at | - | -2,311268652 | -2,690860744 | 0,379592092 | |
| 244793_at | - | -2,311268652 | -2,690860744 | 0,379592092 | |
| 1558699_a_at | HERPUD2 | 0,555999837 | 0,176436073 | 0,379563764 | |
| 1559224_at | LCE1E | 0,555999837 | 0,176436073 | 0,379563764 | |
| 204919_at | PRR4 | 0,555999837 | 0,176436073 | 0,379563764 | |
| 243434_at | - | 0,911589958 | 0,532164371 | 0,379425588 | |
| 1557966_x_at | MTERFD2 | 2,700102307 | 2,320681318 | 0,379420989 | |
| 36830_at | MIPEP | 1,249730273 | 0,87046088 | 0,379269393 | |
| 203556_at | ZHX2 | 1,638726528 | 1,259496994 | 0,379229533 | |
| 225161_at | GFM1 | 3,466766854 | 3,087661492 | 0,379105362 | |
| 218520_at | TBK1 | 3,182077878 | 2,803210557 | 0,378867321 | |
| 1557399_at | LOC284009 | -1,2845686 | -1,663414495 | 0,378845894 | |
| 1563961_at | FHAD1 | -1,2845686 | -1,663414495 | 0,378845894 | |
| 1564131_a_at | BSN-AS2 | -1,2845686 | -1,663414495 | 0,378845894 | |
| 1566507_a_at | FBXO9 | -1,2845686 | -1,663414495 | 0,378845894 | |
| 1566656_a_at | LOC100507654 | -1,2845686 | -1,663414495 | 0,378845894 | |
| 217108_at | - | -1,2845686 | -1,663414495 | 0,378845894 | |
| 221433_at | FGF21 | -1,2845686 | -1,663414495 | 0,378845894 | |
| 223149_s_at | PTPN23 | -1,2845686 | -1,663414495 | 0,378845894 | |
| 228085_at | LOC100507419 / | -1,2845686 | -1,663414495 | 0,378845894 | |
| 232963_at | - | -1,2845686 | -1,663414495 | 0,378845894 | |
| 53071_s_at | C17orf101 | 2,620834364 | 2,242180416 | 0,378653948 | |
| 219865_at | LINC00339 | 2,14450783 | 1,765933685 | 0,378574146 | |
| 220526_s_at | MRPL20 | 5,512036874 | 5,133591331 | 0,378445543 | |
| 227482_at | ADCK1 | 1,748661581 | 1,370274469 | 0,378387112 | |
| 229000_at | ZNF77 | 2,410880861 | 2,032717854 | 0,378163007 | |

| | | | | | |
|---|---|---|---|---|---|
| 212588_at | PTPRC | 3,708558634 | 3,330438773 | 0,378119862 | |
| 208438_s_at | FGR | -0,112808516 | -0,490927335 | 0,378118819 | |
| 206124_s_at | LLGL1 | 0,457392764 | 0,079428135 | 0,377964629 | |
| 1553369_at | FAM129C | -0,352951799 | -0,730778808 | 0,377827009 | |
| 210385_s_at | ERAP1 | -0,352951799 | -0,730778808 | 0,377827009 | |
| 219303_at | RNF219 | 3,628024221 | 3,250254796 | 0,377769425 | |
| 1561355_at | LOC692247 | 0,384950573 | 0,007421914 | 0,37752866 | |
| 216859_x_at | - | 0,384950573 | 0,007421914 | 0,37752866 | |
| 228638_at | - | 0,384950573 | 0,007421914 | 0,37752866 | |
| 1555343_at | MEGF10 | -0,67289328 | -1,050406615 | 0,377513335 | |
| 1564854_at | - | -0,67289328 | -1,050406615 | 0,377513335 | |
| 208306_x_at | HLA-DRB1 /// LO | -0,67289328 | -1,050406615 | 0,377513335 | |
| 210400_at | GRIN2C | -0,67289328 | -1,050406615 | 0,377513335 | |
| 210492_at | MFAP3L | -0,67289328 | -1,050406615 | 0,377513335 | |
| 210780_at | ESR2 | -0,67289328 | -1,050406615 | 0,377513335 | |
| 216256_at | GRM8 | -0,67289328 | -1,050406615 | 0,377513335 | |
| 241514_at | - | -0,67289328 | -1,050406615 | 0,377513335 | |
| 242632_at | FGD2 | -0,67289328 | -1,050406615 | 0,377513335 | |
| 244394_at | - | -0,67289328 | -1,050406615 | 0,377513335 | |
| 225678_at | POLR3H | 1,114389003 | 0,73688026 | 0,377508743 | |
| 235810_at | ZNF182 | 1,865674241 | 1,488344553 | 0,377329688 | |
| 200073_s_at | HNRNPD | 6,571751774 | 6,194681366 | 0,377070407 | |
| 203068_at | KLHL21 | 1,442503549 | 1,065442793 | 0,377060756 | |
| 209729_at | GAS2L1 | 1,406361373 | 1,029536893 | 0,37682448 | |
| 1552758_at | HDAC9 | -2,359436556 | -2,735862426 | 0,37642587 | |
| 1557535_at | PALLD | -2,359436556 | -2,735862426 | 0,37642587 | |
| 1558199_at | FN1 | -2,359436556 | -2,735862426 | 0,37642587 | |
| 1564056_at | - | -2,359436556 | -2,735862426 | 0,37642587 | |
| 1569998_at | MMD2 | -2,359436556 | -2,735862426 | 0,37642587 | |
| 205118_at | FPR1 | -2,359436556 | -2,735862426 | 0,37642587 | |
| 206787_at | BRDT | -2,359436556 | -2,735862426 | 0,37642587 | |
| 215506_s_at | DIRAS3 | -2,359436556 | -2,735862426 | 0,37642587 | |
| 215796_at | - | -2,359436556 | -2,735862426 | 0,37642587 | |
| 227929_at | LIN7A | -2,359436556 | -2,735862426 | 0,37642587 | |
| 232386_at | VPS13C | -2,359436556 | -2,735862426 | 0,37642587 | |
| 234236_at | AMBRA1 | -2,359436556 | -2,735862426 | 0,37642587 | |
| 234827_at | - | -2,359436556 | -2,735862426 | 0,37642587 | |
| 237605_at | - | -2,359436556 | -2,735862426 | 0,37642587 | |
| 238047_at | ARHGAP36 | -2,359436556 | -2,735862426 | 0,37642587 | |
| 241949_at | ACOT6 | -2,359436556 | -2,735862426 | 0,37642587 | |
| 241815_at | - | 1,726031811 | 1,349621332 | 0,376410478 | |
| 1563090_at | CCDC33 | 0,533835206 | 0,157549243 | 0,376285963 | |
| 222709_at | ATG7 | 0,533835206 | 0,157549243 | 0,376285963 | |
| 1566147_a_at | ANKRD20A12P | -0,57445847 | -0,950686014 | 0,376227544 | |
| 204763_s_at | GNAO1 | -0,57445847 | -0,950686014 | 0,376227544 | |
| 208225_at | FGFR2 | -0,57445847 | -0,950686014 | 0,376227544 | |
| 210484_s_at | LOC254896 /// TI | -0,57445847 | -0,950686014 | 0,376227544 | |
| 215334_at | EFR3B | -0,57445847 | -0,950686014 | 0,376227544 | |
| 220309_at | TTC22 | -0,57445847 | -0,950686014 | 0,376227544 | |
| 221077_at | ARMC4 | -0,57445847 | -0,950686014 | 0,376227544 | |
| 226471_at | GGT7 | -0,57445847 | -0,950686014 | 0,376227544 | |
| 227561_at | DDR2 | -0,57445847 | -0,950686014 | 0,376227544 | |
| 229432_at | NAGS | -0,57445847 | -0,950686014 | 0,376227544 | |
| 229959_at | CYP4V2 | -0,57445847 | -0,950686014 | 0,376227544 | |
| 230723_at | SPATA18 | -0,57445847 | -0,950686014 | 0,376227544 | |
| 231170_at | - | -0,57445847 | -0,950686014 | 0,376227544 | |

| | | | | | |
|---|---|---|---|---|---|
| 233560_x_at | MCM8 | -0,57445847 | -0,950686014 | 0,376227544 | |
| 239951_at | - | -0,57445847 | -0,950686014 | 0,376227544 | |
| 242023_at | - | -0,57445847 | -0,950686014 | 0,376227544 | |
| 224367_at | BEX2 | 3,598072957 | 3,222126232 | 0,375946725 | |
| 226948_at | RHBDD1 | 0,87091093 | 0,495031851 | 0,375879079 | |
| 218766_s_at | WARS2 | 1,352503698 | 0,976641161 | 0,375862537 | |
| 204340_at | TMEM187 | 0,728550685 | 0,352836757 | 0,375713928 | |
| 1555436_a_at | AFF4 | -1,047650486 | -1,423348341 | 0,375697856 | |
| 1559009_at | - | -1,047650486 | -1,423348341 | 0,375697856 | |
| 216605_s_at | CEACAM21 | -1,047650486 | -1,423348341 | 0,375697856 | |
| 220847_x_at | ZNF221 | -1,047650486 | -1,423348341 | 0,375697856 | |
| 234898_at | - | -1,047650486 | -1,423348341 | 0,375697856 | |
| 203457_at | STX7 | 1,143956113 | 0,768343791 | 0,375612323 | |
| 214581_x_at | TNFRSF21 | 1,46610492 | 1,090553537 | 0,375551383 | |
| 222397_at | EFTUD2 | 0,273439642 | -0,102100538 | 0,37554018 | |
| 239445_at | - | 0,273439642 | -0,102100538 | 0,37554018 | |
| 1556994_at | - | -0,888496123 | -1,264005341 | 0,375509218 | |
| 1559551_at | - | -0,888496123 | -1,264005341 | 0,375509218 | |
| 1561976_at | C1orf167 /// LOC | -0,888496123 | -1,264005341 | 0,375509218 | |
| 1568971_at | C14orf135 | -0,888496123 | -1,264005341 | 0,375509218 | |
| 207652_s_at | CMKLR1 | -0,888496123 | -1,264005341 | 0,375509218 | |
| 208034_s_at | PROZ | -0,888496123 | -1,264005341 | 0,375509218 | |
| 220092_s_at | ANTXR1 | -0,888496123 | -1,264005341 | 0,375509218 | |
| 232220_at | S100A7A | -0,888496123 | -1,264005341 | 0,375509218 | |
| 232346_at | LOC388692 | -0,888496123 | -1,264005341 | 0,375509218 | |
| 233734_s_at | OSBPL5 | -0,888496123 | -1,264005341 | 0,375509218 | |
| 241214_at | - | -0,888496123 | -1,264005341 | 0,375509218 | |
| 244293_at | UQCRB | -0,888496123 | -1,264005341 | 0,375509218 | |
| 225686_at | SKA2 | 3,591828571 | 3,216434104 | 0,375394467 | |
| 203897_at | LYRM1 | 3,335176289 | 2,959949103 | 0,375227187 | |
| 216981_x_at | SPN | 2,156646206 | 1,781437557 | 0,375208649 | |
| 225268_at | KPNA4 | 5,089402992 | 4,714195481 | 0,375207511 | |
| 218604_at | LEMD3 | 3,525045272 | 3,149904996 | 0,375140277 | |
| 220838_at | EXD3 | 0,052199481 | -0,322870281 | 0,375069762 | |
| 226911_at | EGFLAM | 0,052199481 | -0,322870281 | 0,375069762 | |
| 1556244_s_at | LOC375196 | 0,503742507 | 0,128747141 | 0,374995367 | |
| 215519_x_at | SGSM3 | 0,503742507 | 0,128747141 | 0,374995367 | |
| 228303_at | GALNT6 | 0,503742507 | 0,128747141 | 0,374995367 | |
| 238467_at | - | 0,503742507 | 0,128747141 | 0,374995367 | |
| 1556758_at | FAM208B | -1,240169874 | -1,615132408 | 0,374962533 | |
| 1561228_at | LOC100506122 | -1,240169874 | -1,615132408 | 0,374962533 | |
| 211380_s_at | PRKG1 | -1,240169874 | -1,615132408 | 0,374962533 | |
| 215992_s_at | RAPGEF2 | -1,240169874 | -1,615132408 | 0,374962533 | |
| 237450_at | LOC389332 | -1,240169874 | -1,615132408 | 0,374962533 | |
| 1554472_a_at | PHF20L1 | 0,928678725 | 0,553993624 | 0,3746851 | |
| 218279_s_at | HIST2H2AA3 | 0,928678725 | 0,553993624 | 0,3746851 | |
| 213439_x_at | RUNDC3A | 0,792179257 | 0,417770464 | 0,374408793 | |
| 234761_at | - | 0,792179257 | 0,417770464 | 0,374408793 | |
| 235846_at | FSBP /// RAD54E | 1,079110292 | 0,704715219 | 0,374395073 | |
| 1562577_at | - | 0,641402394 | 0,26733076 | 0,374071635 | |
| 203377_s_at | CDC40 | 1,542099044 | 1,16809119 | 0,374007853 | |
| 218784_s_at | SAYSD1 | 1,689737652 | 1,315948728 | 0,373788924 | |
| 204699_s_at | DIEXF | 2,837639949 | 2,464004877 | 0,373635071 | |
| 210766_s_at | CSE1L | 5,739893061 | 5,366268691 | 0,37362437 | |
| 206544_x_at | SMARCA2 | 1,360921491 | 0,987376598 | 0,373544894 | |
| 226406_at | C18orf25 | 3,301680869 | 2,928263672 | 0,373417197 | |

| | | | | | |
|---|---|---|---|---|---|
| 213200_at | SYP | 0,767063567 | 0,393761504 | 0,373302063 | |
| 219170_at | FSD1 | 0,853119696 | 0,479907041 | 0,373212655 | |
| 227585_at | ATAD1 | 1,67631007 | 1,303116084 | 0,373193986 | |
| 1564166_s_at | LOC100630923 | 0,152582607 | -0,220592223 | 0,37317483 | |
| 210841_s_at | NRP2 | 0,152582607 | -0,220592223 | 0,37317483 | |
| 223519_at | ZAK | 0,152582607 | -0,220592223 | 0,37317483 | |
| 218301_at | RNPEPL1 | 1,011212064 | 0,638150376 | 0,373061688 | |
| 219062_s_at | ZCCHC2 | 1,85379461 | 1,480766833 | 0,373027777 | |
| 215991_s_at | EMC1 | 0,401366113 | 0,028364014 | 0,373002099 | |
| 1558877_at | - | -1,838812296 | -2,211750327 | 0,372938031 | |
| 1560112_at | WDFY2 | -1,838812296 | -2,211750327 | 0,372938031 | |
| 1568891_x_at | FANCD2 | -1,838812296 | -2,211750327 | 0,372938031 | |
| 216990_at | GART | -1,838812296 | -2,211750327 | 0,372938031 | |
| 229056_at | - | -1,838812296 | -2,211750327 | 0,372938031 | |
| 229777_at | CLRN3 | -1,838812296 | -2,211750327 | 0,372938031 | |
| 232164_s_at | EPPK1 | -1,838812296 | -2,211750327 | 0,372938031 | |
| 233201_at | - | -1,838812296 | -2,211750327 | 0,372938031 | |
| 233549_at | PDE1A | -1,838812296 | -2,211750327 | 0,372938031 | |
| 233965_at | TBX5-AS1 | -1,838812296 | -2,211750327 | 0,372938031 | |
| 236901_at | - | -1,838812296 | -2,211750327 | 0,372938031 | |
| 237517_at | - | -1,838812296 | -2,211750327 | 0,372938031 | |
| 237557_at | - | -1,838812296 | -2,211750327 | 0,372938031 | |
| 239755_at | - | -1,838812296 | -2,211750327 | 0,372938031 | |
| 240842_at | - | -1,838812296 | -2,211750327 | 0,372938031 | |
| 241269_at | - | -1,838812296 | -2,211750327 | 0,372938031 | |
| 244806_at | - | -1,838812296 | -2,211750327 | 0,372938031 | |
| 1555031_at | - | -1,71423189 | -2,086984744 | 0,372752854 | |
| 1557734_s_at | LOC100130548 | -1,71423189 | -2,086984744 | 0,372752854 | |
| 1561281_a_at | LEMD1-AS1 | -1,71423189 | -2,086984744 | 0,372752854 | |
| 1564974_at | KRTAP8-1 | -1,71423189 | -2,086984744 | 0,372752854 | |
| 1570014_at | - | -1,71423189 | -2,086984744 | 0,372752854 | |
| 204303_s_at | CTIF | -1,71423189 | -2,086984744 | 0,372752854 | |
| 207754_at | RASSF8 | -1,71423189 | -2,086984744 | 0,372752854 | |
| 229398_at | RAB18 | -1,71423189 | -2,086984744 | 0,372752854 | |
| 230410_at | - | -1,71423189 | -2,086984744 | 0,372752854 | |
| 233598_at | - | -1,71423189 | -2,086984744 | 0,372752854 | |
| 235188_at | - | -1,71423189 | -2,086984744 | 0,372752854 | |
| 236450_at | - | -1,71423189 | -2,086984744 | 0,372752854 | |
| 239438_at | RAPGEF6 | -1,71423189 | -2,086984744 | 0,372752854 | |
| 210685_s_at | UBE4B | 1,40228911 | 1,029536893 | 0,372752216 | |
| 212096_s_at | MTUS1 | 3,95851035 | 3,585762986 | 0,372747365 | |
| 215150_at | YOD1 | 1,987559146 | 1,614891062 | 0,372668085 | |
| 226317_at | PPP4R2 | 2,450859799 | 2,078620836 | 0,372238963 | |
| 1554517_x_at | LINC00537 | 0,82905153 | 0,456918297 | 0,372133233 | |
| 220150_s_at | FAM184A | 1,063722698 | 0,691645685 | 0,372077012 | |
| 1556035_s_at | ZNF207 | 1,549482847 | 1,177496821 | 0,371986026 | |
| 212054_x_at | TBC1D9B | 1,549482847 | 1,177496821 | 0,371986026 | |
| 222129_at | FAM134A | 2,064305961 | 1,692388143 | 0,371917818 | |
| 205275_at | GTPBP1 | -0,089689931 | -0,461594427 | 0,371904495 | |
| 209182_s_at | C10orf10 | -0,089689931 | -0,461594427 | 0,371904495 | |
| 219456_s_at | RIN3 | -0,089689931 | -0,461594427 | 0,371904495 | |
| 221440_s_at | RBBP9 | -0,089689931 | -0,461594427 | 0,371904495 | |
| 203935_at | ACVR1 | 3,484209953 | 3,112391743 | 0,37181821 | |
| 202158_s_at | CELF2 | 3,453052675 | 3,081412119 | 0,371640556 | |
| 234947_s_at | FAM204A | 2,462640593 | 2,091116676 | 0,371523917 | |
| 1552415_a_at | WFDC9 | -0,741668563 | -1,112851844 | 0,371183281 | |

| | | | | | |
|---|---|---|---|---|---|
| 210716_s_at | CLIP1 | -0,741668563 | -1,112851844 | 0,371183281 | |
| 218747_s_at | TAPBPL | -0,741668563 | -1,112851844 | 0,371183281 | |
| 221157_s_at | FBXO24 | -0,741668563 | -1,112851844 | 0,371183281 | |
| 229222_at | ACSS3 | -0,741668563 | -1,112851844 | 0,371183281 | |
| 229702_at | CSNK1G3 | -0,741668563 | -1,112851844 | 0,371183281 | |
| 224957_at | C18orf32 /// RPL1 | 3,154500615 | 2,783327323 | 0,371173292 | |
| 235707_at | C6orf228 | 0,888485433 | 0,51742585 | 0,371059583 | |
| 1566577_at | - | -1,964176657 | -2,335104118 | 0,370927461 | |
| 202953_at | C1QB | -1,964176657 | -2,335104118 | 0,370927461 | |
| 204515_at | HSD3B1 | -1,964176657 | -2,335104118 | 0,370927461 | |
| 204580_at | MMP12 | -1,964176657 | -2,335104118 | 0,370927461 | |
| 205715_at | BST1 | -1,964176657 | -2,335104118 | 0,370927461 | |
| 208462_s_at | ABCC9 | -1,964176657 | -2,335104118 | 0,370927461 | |
| 219497_s_at | BCL11A | -1,964176657 | -2,335104118 | 0,370927461 | |
| 222098_s_at | - | -1,964176657 | -2,335104118 | 0,370927461 | |
| 226344_at | ZMAT1 | -1,964176657 | -2,335104118 | 0,370927461 | |
| 231289_at | - | -1,964176657 | -2,335104118 | 0,370927461 | |
| 231797_at | SIX4 | -1,964176657 | -2,335104118 | 0,370927461 | |
| 232999_at | - | -1,964176657 | -2,335104118 | 0,370927461 | |
| 233171_at | GRIN3A | -1,964176657 | -2,335104118 | 0,370927461 | |
| 237312_at | LOC100506272 | -1,964176657 | -2,335104118 | 0,370927461 | |
| 237645_at | LOC100506577 | -1,964176657 | -2,335104118 | 0,370927461 | |
| 238564_at | FAM171B | -1,964176657 | -2,335104118 | 0,370927461 | |
| 241176_at | - | -1,964176657 | -2,335104118 | 0,370927461 | |
| 204918_s_at | MLLT3 | 2,882181479 | 2,511262629 | 0,370918849 | |
| 231772_x_at | CENPH | 3,25953187 | 2,888726553 | 0,370805317 | |
| 203504_s_at | ABCA1 | -0,394749123 | -0,765504029 | 0,370754906 | |
| 219534_x_at | CDKN1C | -0,394749123 | -0,765504029 | 0,370754906 | |
| 238820_at | TMEM229B | -0,394749123 | -0,765504029 | 0,370754906 | |
| 241818_at | - | -0,394749123 | -0,765504029 | 0,370754906 | |
| 1559865_at | - | -2,667025278 | -3,037500868 | 0,370475591 | |
| 1562341_at | - | -2,667025278 | -3,037500868 | 0,370475591 | |
| 1570366_x_at | ZNF709 | -2,667025278 | -3,037500868 | 0,370475591 | |
| 205694_at | TYRP1 | -2,667025278 | -3,037500868 | 0,370475591 | |
| 210837_s_at | PDE4D | -2,667025278 | -3,037500868 | 0,370475591 | |
| 211522_s_at | GNRHR | -2,667025278 | -3,037500868 | 0,370475591 | |
| 213362_at | PTPRD | -2,667025278 | -3,037500868 | 0,370475591 | |
| 231385_at | DPPA3 | -2,667025278 | -3,037500868 | 0,370475591 | |
| 239904_at | - | -2,667025278 | -3,037500868 | 0,370475591 | |
| 241638_at | - | -2,667025278 | -3,037500868 | 0,370475591 | |
| 242649_x_at | HMGN2P46 | -2,667025278 | -3,037500868 | 0,370475591 | |
| 243406_at | TMCO5B | -2,667025278 | -3,037500868 | 0,370475591 | |
| 201266_at | TXNRD1 | 4,713590151 | 4,343149918 | 0,370440232 | |
| 228633_s_at | CNTROB | 1,292162421 | 0,921731975 | 0,370430446 | |
| 221531_at | WDR61 | 4,020928202 | 3,650516168 | 0,370412034 | |
| 202714_s_at | KIAA0391 | 0,747935635 | 0,377530647 | 0,370404989 | |
| 1563657_at | PLXND1 | 0,020670649 | -0,34958478 | 0,37025543 | |
| 217284_x_at | SERHL2 | 0,020670649 | -0,34958478 | 0,37025543 | |
| 223282_at | TSHZ1 | 0,020670649 | -0,34958478 | 0,37025543 | |
| 239642_at | - | 0,020670649 | -0,34958478 | 0,37025543 | |
| 1554067_at | C12orf66 | -1,591296941 | -1,961547147 | 0,370250206 | |
| 1561668_at | - | -1,591296941 | -1,961547147 | 0,370250206 | |
| 205330_at | MN1 | -1,591296941 | -1,961547147 | 0,370250206 | |
| 215964_at | - | -1,591296941 | -1,961547147 | 0,370250206 | |
| 217484_at | CR1 | -1,591296941 | -1,961547147 | 0,370250206 | |
| 220259_at | PLEKHH3 | -1,591296941 | -1,961547147 | 0,370250206 | |

| | | | | | |
|---|---|---|---|---|---|
| 231881_at | CALD1 | -1,591296941 | -1,961547147 | 0,370250206 | |
| 232040_at | LOC157860 | -1,591296941 | -1,961547147 | 0,370250206 | |
| 235281_x_at | AHNAK | -1,591296941 | -1,961547147 | 0,370250206 | |
| 237897_at | ADAM17 | -1,591296941 | -1,961547147 | 0,370250206 | |
| 238634_x_at | - | -1,591296941 | -1,961547147 | 0,370250206 | |
| 222762_x_at | LIMD1 | 1,748661581 | 1,378453643 | 0,370207938 | |
| 228255_at | TMEM237 | -0,196756757 | -0,566887459 | 0,370130702 | |
| 239235_at | - | -0,196756757 | -0,566887459 | 0,370130702 | |
| 242556_at | - | -0,196756757 | -0,566887459 | 0,370130702 | |
| 242782_x_at | TMEM198 | -0,196756757 | -0,566887459 | 0,370130702 | |
| 244271_at | - | -0,196756757 | -0,566887459 | 0,370130702 | |
| 221761_at | ADSS | 3,628024221 | 3,258033502 | 0,369990719 | |
| 1561699_a_at | - | -2,866296344 | -3,236070201 | 0,369773857 | |
| 234313_at | NCOR1 | -2,866296344 | -3,236070201 | 0,369773857 | |
| 243247_at | - | -2,866296344 | -3,236070201 | 0,369773857 | |
| 206220_s_at | RASA3 | 0,209623097 | -0,160136748 | 0,369759846 | |
| 228573_at | ANTXR2 | 1,274448124 | 0,904843258 | 0,369604866 | |
| 224828_at | CPEB4 | 0,97328325 | 0,603679647 | 0,369603603 | |
| 224597_at | LOC647979 | 6,177257557 | 5,807870843 | 0,369386714 | |
| 225219_at | SMAD5 | 1,593009234 | 1,223626587 | 0,369382647 | |
| 226268_at | RAB21 | 1,593009234 | 1,223626587 | 0,369382647 | |
| 226426_at | ADNP | 2,908260915 | 2,538891651 | 0,369369264 | |
| 1560596_at | - | -0,85085127 | -1,220210588 | 0,369359318 | |
| 1561373_at | PACRG-AS1 | -0,85085127 | -1,220210588 | 0,369359318 | |
| 203325_s_at | COL5A1 | -0,85085127 | -1,220210588 | 0,369359318 | |
| 209293_x_at | ID4 | -0,85085127 | -1,220210588 | 0,369359318 | |
| 217338_at | KRT19P2 | -0,85085127 | -1,220210588 | 0,369359318 | |
| 223137_at | ZDHHC4 | -0,85085127 | -1,220210588 | 0,369359318 | |
| 226075_at | SPSB1 | -0,85085127 | -1,220210588 | 0,369359318 | |
| 229990_at | TSC22D1-AS1 | -0,85085127 | -1,220210588 | 0,369359318 | |
| 234466_at | FAM20C | -0,85085127 | -1,220210588 | 0,369359318 | |
| 236399_at | - | -0,85085127 | -1,220210588 | 0,369359318 | |
| 241609_at | FOXD3 | -0,85085127 | -1,220210588 | 0,369359318 | |
| 211703_s_at | TM2D1 | 2,618203384 | 2,248887791 | 0,369315593 | |
| 1562391_at | B3GALNT2 | -0,543051993 | -0,912331589 | 0,369279596 | |
| 202237_at | NNMT | -0,543051993 | -0,912331589 | 0,369279596 | |
| 206896_s_at | GNG7 | -0,543051993 | -0,912331589 | 0,369279596 | |
| 214762_at | ATP6V1G2 | -0,543051993 | -0,912331589 | 0,369279596 | |
| 217337_at | - | -0,543051993 | -0,912331589 | 0,369279596 | |
| 221717_at | - | -0,543051993 | -0,912331589 | 0,369279596 | |
| 229016_s_at | TRERF1 | -0,543051993 | -0,912331589 | 0,369279596 | |
| 230300_at | PSMA5 | -0,543051993 | -0,912331589 | 0,369279596 | |
| 236394_at | A2MP1 | -0,543051993 | -0,912331589 | 0,369279596 | |
| 217757_at | A2M | 0,376672223 | 0,007421914 | 0,36925031 | |
| 221237_s_at | OSBP2 | 0,376672223 | 0,007421914 | 0,36925031 | |
| 202453_s_at | GTF2H1 | 2,327437554 | 1,958277216 | 0,369160338 | |
| 214934_at | ATP9B | 1,434549838 | 1,065442793 | 0,369107045 | |
| 221192_x_at | MFSD11 | 1,434549838 | 1,065442793 | 0,369107045 | |
| 229318_at | - | 1,767779954 | 1,398700978 | 0,369078976 | |
| 227583_at | POP4 | 2,936695663 | 2,567790814 | 0,368904849 | |
| 1557796_at | FAR2 | -1,543331864 | -1,912089812 | 0,368757947 | |
| 1559298_a_at | FLJ42289 | -1,543331864 | -1,912089812 | 0,368757947 | |
| 1560412_at | LOC100507506 | -1,543331864 | -1,912089812 | 0,368757947 | |
| 1561062_a_at | - | -1,543331864 | -1,912089812 | 0,368757947 | |
| 1569245_at | C8orf74 | -1,543331864 | -1,912089812 | 0,368757947 | |
| 1570566_at | - | -1,543331864 | -1,912089812 | 0,368757947 | |

| | | | | | |
|---|---|---|---|---|---|
| 207899_at | GIP | -1,543331864 | -1,912089812 | 0,368757947 | |
| 216729_at | - | -1,543331864 | -1,912089812 | 0,368757947 | |
| 220112_at | ANKRD55 | -1,543331864 | -1,912089812 | 0,368757947 | |
| 223835_x_at | OTP | -1,543331864 | -1,912089812 | 0,368757947 | |
| 230487_at | C6orf99 | -1,543331864 | -1,912089812 | 0,368757947 | |
| 230514_s_at | LYZL1 /// LYZL2 | -1,543331864 | -1,912089812 | 0,368757947 | |
| 232535_at | - | -1,543331864 | -1,912089812 | 0,368757947 | |
| 234831_at | - | -1,543331864 | -1,912089812 | 0,368757947 | |
| 236222_at | C3orf15 /// LOC1 | -1,543331864 | -1,912089812 | 0,368757947 | |
| 241108_at | - | -1,543331864 | -1,912089812 | 0,368757947 | |
| 241321_at | - | -1,543331864 | -1,912089812 | 0,368757947 | |
| 242027_at | - | -1,543331864 | -1,912089812 | 0,368757947 | |
| 232635_at | CEP128 | 1,027166904 | 0,658444279 | 0,368722625 | |
| 223417_at | RAD18 | 1,69307511 | 1,324440845 | 0,368634265 | |
| 224159_x_at | TRIM4 | 1,69307511 | 1,324440845 | 0,368634265 | |
| 1569206_at | TCP11L2 | 0,661985843 | 0,293518836 | 0,368467007 | |
| 44065_at | C12orf52 | 1,66275634 | 1,294497133 | 0,368259207 | |
| 204564_at | PCGF3 | -0,022466681 | -0,39061235 | 0,368145669 | |
| 239801_at | RNF40 | -0,022466681 | -0,39061235 | 0,368145669 | |
| 1565857_at | - | -1,173767127 | -1,541908042 | 0,368140915 | |
| 1570350_at | LOC100506403 / | -1,173767127 | -1,541908042 | 0,368140915 | |
| 207032_s_at | CRISP1 | -1,173767127 | -1,541908042 | 0,368140915 | |
| 210072_at | CCL19 | -1,173767127 | -1,541908042 | 0,368140915 | |
| 210331_at | HECW1 | -1,173767127 | -1,541908042 | 0,368140915 | |
| 211869_at | - | -1,173767127 | -1,541908042 | 0,368140915 | |
| 212067_s_at | C1R | -1,173767127 | -1,541908042 | 0,368140915 | |
| 214520_at | FOXC2 | -1,173767127 | -1,541908042 | 0,368140915 | |
| 217034_at | - | -1,173767127 | -1,541908042 | 0,368140915 | |
| 232170_at | S100A7A | -1,173767127 | -1,541908042 | 0,368140915 | |
| 233771_at | - | -1,173767127 | -1,541908042 | 0,368140915 | |
| 234292_s_at | ZNF167 | -1,173767127 | -1,541908042 | 0,368140915 | |
| 236140_at | GCLM | -1,173767127 | -1,541908042 | 0,368140915 | |
| 236554_x_at | TMC8 | -1,173767127 | -1,541908042 | 0,368140915 | |
| 242019_at | CERS6 | -1,173767127 | -1,541908042 | 0,368140915 | |
| 208674_x_at | DDOST | 4,895888063 | 4,527755823 | 0,36813224 | |
| 200675_at | CD81 | 5,536859965 | 5,168796961 | 0,368063003 | |
| 205830_at | CLGN | 2,102456302 | 1,73441697 | 0,368039332 | |
| 241215_at | - | 0,171847695 | -0,19610998 | 0,367957675 | |
| 1568954_s_at | C16orf72 | 2,420979937 | 2,053299695 | 0,367680241 | |
| 213628_at | CLCC1 | 2,173470163 | 1,805902329 | 0,367567834 | |
| 227718_at | PURB | 2,173470163 | 1,805902329 | 0,367567834 | |
| 213192_at | THAP3 | 1,104397092 | 0,73688026 | 0,367516831 | |
| 212927_at | SMC5 | 3,173740462 | 2,806245339 | 0,367495123 | |
| 204614_at | SERPINB2 | 3,234554895 | 2,867091644 | 0,367463251 | |
| 202495_at | TBCC | 3,397876879 | 3,030417996 | 0,367458883 | |
| 1552514_at | WBP2NL | -2,479842156 | -2,847001814 | 0,367159658 | |
| 1557733_a_at | CHRM3-AS2 | -2,479842156 | -2,847001814 | 0,367159658 | |
| 1558857_at | - | -2,479842156 | -2,847001814 | 0,367159658 | |
| 1560416_at | DNAH11 | -2,479842156 | -2,847001814 | 0,367159658 | |
| 1562189_at | - | -2,479842156 | -2,847001814 | 0,367159658 | |
| 1566449_at | LRRC40 | -2,479842156 | -2,847001814 | 0,367159658 | |
| 1568916_at | SPIRE2 | -2,479842156 | -2,847001814 | 0,367159658 | |
| 210000_s_at | SOCS1 | -2,479842156 | -2,847001814 | 0,367159658 | |
| 223815_at | CEP95 | -2,479842156 | -2,847001814 | 0,367159658 | |
| 227439_at | ANKS1B | -2,479842156 | -2,847001814 | 0,367159658 | |
| 233534_at | KRTAP3-2 | -2,479842156 | -2,847001814 | 0,367159658 | |

| | | | | | |
|---|---|---|---|---|---|
| 234213_at | - | -2,479842156 | -2,847001814 | 0,367159658 | |
| 235578_at | ABCC9 | -2,479842156 | -2,847001814 | 0,367159658 | |
| 239999_at | LINC00478 | -2,479842156 | -2,847001814 | 0,367159658 | |
| 242107_x_at | - | -2,479842156 | -2,847001814 | 0,367159658 | |
| 244454_at | - | -2,479842156 | -2,847001814 | 0,367159658 | |
| 203572_s_at | TAF6 | 2,28383338 | 1,916759838 | 0,367073542 | |
| 212647_at | RRAS | 0,95671774 | 0,589657387 | 0,367060353 | |
| 64371_at | SUGP2 | 1,714582406 | 1,347539663 | 0,367042743 | |
| 223576_at | C6orf203 | 2,943717636 | 2,576704359 | 0,367013277 | |
| 216976_s_at | RYK | 1,903621061 | 1,536652708 | 0,366968354 | |
| 222509_s_at | ZNF672 | 0,760715727 | 0,393761504 | 0,366954222 | |
| 1554742_at | PMS1 | -2,089474779 | -2,456406426 | 0,366931647 | |
| 1555836_at | POLR2B | -2,089474779 | -2,456406426 | 0,366931647 | |
| 1556122_at | RAB11B-AS1 | -2,089474779 | -2,456406426 | 0,366931647 | |
| 1560556_a_at | PLEKHA8 | -2,089474779 | -2,456406426 | 0,366931647 | |
| 1563361_at | RNF216 | -2,089474779 | -2,456406426 | 0,366931647 | |
| 1564351_at | - | -2,089474779 | -2,456406426 | 0,366931647 | |
| 1567139_at | - | -2,089474779 | -2,456406426 | 0,366931647 | |
| 1569554_at | ESR2 | -2,089474779 | -2,456406426 | 0,366931647 | |
| 203066_at | CHST15 | -2,089474779 | -2,456406426 | 0,366931647 | |
| 206841_at | PDE6H | -2,089474779 | -2,456406426 | 0,366931647 | |
| 208399_s_at | EDN3 | -2,089474779 | -2,456406426 | 0,366931647 | |
| 230351_at | LOC283481 | -2,089474779 | -2,456406426 | 0,366931647 | |
| 233452_at | - | -2,089474779 | -2,456406426 | 0,366931647 | |
| 237888_at | LOC100422737 | -2,089474779 | -2,456406426 | 0,366931647 | |
| 238386_x_at | - | -2,089474779 | -2,456406426 | 0,366931647 | |
| 238964_at | FIGN | -2,089474779 | -2,456406426 | 0,366931647 | |
| 240173_at | - | -2,089474779 | -2,456406426 | 0,366931647 | |
| 240197_at | SYN2 | -2,089474779 | -2,456406426 | 0,366931647 | |
| 242089_at | - | -2,089474779 | -2,456406426 | 0,366931647 | |
| 243761_at | CLDN12 | -2,089474779 | -2,456406426 | 0,366931647 | |
| 201116_s_at | CPE | 1,153678675 | 0,786897721 | 0,366780954 | |
| 223471_at | RAB3IP | 1,153678675 | 0,786897721 | 0,366780954 | |
| 244804_at | SQSTM1 | 0,083054335 | -0,283667828 | 0,366722162 | |
| 219809_at | WDR55 | 2,232003119 | 1,865308541 | 0,366694578 | |
| 238561_s_at | UTP23 | 1,603688695 | 1,23718272 | 0,366505975 | |
| 203517_at | MTX2 | 4,19991587 | 3,833443169 | 0,366472701 | |
| 223780_s_at | MED13 | 0,425644434 | 0,059218869 | 0,366425565 | |
| 237024_at | C3orf45 | 0,425644434 | 0,059218869 | 0,366425565 | |
| 225985_at | PRKAA1 | 0,57058931 | 0,204310755 | 0,366278555 | |
| 233638_s_at | POMGNT1 | 0,57058931 | 0,204310755 | 0,366278555 | |
| 212065_s_at | USP34 | 0,702292136 | 0,336136178 | 0,366155958 | |
| 219538_at | WDR5B | 0,702292136 | 0,336136178 | 0,366155958 | |
| 232014_at | ZNF30 | 2,501229299 | 2,135226207 | 0,366003092 | |
| 213734_at | RFC5 | 3,469688722 | 3,103784293 | 0,36590443 | |
| 226608_at | C16orf87 | 1,446464021 | 1,080561626 | 0,365902395 | |
| 1567079_at | CLN6 | 1,037706275 | 0,67181667 | 0,365889605 | |
| 228972_at | PITPNA-AS1 | 1,85379461 | 1,488344553 | 0,365450057 | |
| 231832_at | GALNT4 /// POC1 | 1,90937145 | 1,543943227 | 0,365428223 | |
| 1554342_s_at | HELQ | 1,089278259 | 0,724100169 | 0,36517809 | |
| 213457_at | MFHAS1 | 1,269985355 | 0,904843258 | 0,365142096 | |
| 1555086_at | STAT5B | 1,352503698 | 0,987376598 | 0,365127101 | |
| 226183_at | GSK3B | 1,352503698 | 0,987376598 | 0,365127101 | |
| 226279_at | PRSS23 | 3,237986416 | 2,87289275 | 0,365093666 | |
| 1555925_at | - | -2,908843709 | -3,273861381 | 0,365017673 | |
| 1561170_at | GOLGA6L1 /// G( | -2,908843709 | -3,273861381 | 0,365017673 | |

| | | | | | |
|---|---|---|---|---|---|
| 231453_at | C17orf105 | -2,908843709 | -3,273861381 | 0,365017673 | |
| 223077_at | TMOD3 | 3,22766727 | 2,862725455 | 0,364941814 | |
| 1553426_at | C5orf64 | -2,138804305 | -2,503677622 | 0,364873317 | |
| 1557993_at | - | -2,138804305 | -2,503677622 | 0,364873317 | |
| 206269_at | GCM1 | -2,138804305 | -2,503677622 | 0,364873317 | |
| 207735_at | RNF125 | -2,138804305 | -2,503677622 | 0,364873317 | |
| 208790_s_at | PTRF | -2,138804305 | -2,503677622 | 0,364873317 | |
| 209010_s_at | TRIO | -2,138804305 | -2,503677622 | 0,364873317 | |
| 216939_s_at | HTR4 | -2,138804305 | -2,503677622 | 0,364873317 | |
| 228557_at | L3MBTL4 | -2,138804305 | -2,503677622 | 0,364873317 | |
| 235321_at | NDUFS1 | -2,138804305 | -2,503677622 | 0,364873317 | |
| 236729_at | - | -2,138804305 | -2,503677622 | 0,364873317 | |
| 240992_at | - | -2,138804305 | -2,503677622 | 0,364873317 | |
| 213693_s_at | MUC1 | 0,798390451 | 0,433557298 | 0,364833153 | |
| 235830_at | - | 0,798390451 | 0,433557298 | 0,364833153 | |
| 1557701_s_at | POLH | 0,228146222 | -0,136643983 | 0,364790204 | |
| 1569515_a_at | - | 0,228146222 | -0,136643983 | 0,364790204 | |
| 204974_at | RAB3A | 0,228146222 | -0,136643983 | 0,364790204 | |
| 210925_at | CIITA | 0,228146222 | -0,136643983 | 0,364790204 | |
| 211728_s_at | HYAL3 | 0,228146222 | -0,136643983 | 0,364790204 | |
| 223677_at | ATG10 | 0,228146222 | -0,136643983 | 0,364790204 | |
| 226665_at | AHSA2 | 0,228146222 | -0,136643983 | 0,364790204 | |
| 236751_at | - | 0,228146222 | -0,136643983 | 0,364790204 | |
| 209404_s_at | TMED7 /// TMED | 3,687797371 | 3,323041804 | 0,364755567 | |
| 219558_at | ATP13A3 | 1,394109935 | 1,029536893 | 0,364573042 | |
| 225580_at | MRPL50 | 3,819603766 | 3,455550298 | 0,364053467 | |
| 201879_at | ARIH1 | 1,844820248 | 1,480766833 | 0,364053415 | |
| 1555125_at | GCFC1 | 0,859074518 | 0,495031851 | 0,364042668 | |
| 206997_s_at | HS6ST1 | -0,366776884 | -0,730778808 | 0,364001924 | |
| 210161_at | - | -0,366776884 | -0,730778808 | 0,364001924 | |
| 216950_s_at | FCGR1A /// FCG | -0,366776884 | -0,730778808 | 0,364001924 | |
| 227240_at | NGEF | -0,366776884 | -0,730778808 | 0,364001924 | |
| 233299_at | RIMS4 | -0,366776884 | -0,730778808 | 0,364001924 | |
| 234308_at | TUBGCP6 | -0,366776884 | -0,730778808 | 0,364001924 | |
| 234376_at | MYCN | -0,366776884 | -0,730778808 | 0,364001924 | |
| 235797_x_at | HMCN2 | -0,366776884 | -0,730778808 | 0,364001924 | |
| 211352_s_at | NCOA3 | 0,613492853 | 0,249604176 | 0,363888677 | |
| 1560386_at | - | -0,172274514 | -0,536087151 | 0,363812637 | |
| 205360_at | PFDN4 | -0,172274514 | -0,536087151 | 0,363812637 | |
| 206851_at | RNASE3 | -0,172274514 | -0,536087151 | 0,363812637 | |
| 221068_at | KANK2 | -0,172274514 | -0,536087151 | 0,363812637 | |
| 213891_s_at | TCF4 | 2,671625168 | 2,307890888 | 0,36373428 | |
| 1554821_a_at | ZBED1 | 0,317354302 | -0,046302147 | 0,363656449 | |
| 1566178_x_at | - | 0,317354302 | -0,046302147 | 0,363656449 | |
| 220616_at | ZNF384 | 0,317354302 | -0,046302147 | 0,363656449 | |
| 227841_at | AMDHD2 /// CEM | 0,317354302 | -0,046302147 | 0,363656449 | |
| 209735_at | ABCG2 | -1,131062212 | -1,494668682 | 0,363606469 | |
| 211030_s_at | SLC6A6 | -1,131062212 | -1,494668682 | 0,363606469 | |
| 214470_at | KLRB1 | -1,131062212 | -1,494668682 | 0,363606469 | |
| 214978_s_at | PPFIA4 | -1,131062212 | -1,494668682 | 0,363606469 | |
| 216726_at | VENTXP1 | -1,131062212 | -1,494668682 | 0,363606469 | |
| 223930_at | - | -1,131062212 | -1,494668682 | 0,363606469 | |
| 228042_at | ADPRH | -1,131062212 | -1,494668682 | 0,363606469 | |
| 228865_at | C1orf116 | -1,131062212 | -1,494668682 | 0,363606469 | |
| 232793_at | - | -1,131062212 | -1,494668682 | 0,363606469 | |
| 233174_at | LOC100287015 | -1,131062212 | -1,494668682 | 0,363606469 | |

| | | | | | |
|---|---|---|---|---|---|
| 234718_at | ANO2 | -1,131062212 | -1,494668682 | 0,363606469 | |
| 234845_at | DKFZp761P0212 | -1,131062212 | -1,494668682 | 0,363606469 | |
| 244281_at | REXO1 | -1,131062212 | -1,494668682 | 0,363606469 | |
| 244499_at | THAP2 | -1,131062212 | -1,494668682 | 0,363606469 | |
| 202702_at | TRIM26 | 1,119359127 | 0,755840599 | 0,363518528 | |
| 209000_s_at | 39692 | 1,493160564 | 1,129843209 | 0,363317355 | |
| 231705_at | HRSP12 | 0,917308734 | 0,553993624 | 0,36331511 | |
| 201747_s_at | SAFB | 2,190100188 | 1,826973888 | 0,363126299 | |
| 211763_s_at | UBE2B | 3,329826127 | 2,966746113 | 0,363080014 | |
| 1554927_at | LINC00598 | -1,424023738 | -1,787089803 | 0,363066065 | |
| 1570650_at | CCBL1 | -1,424023738 | -1,787089803 | 0,363066065 | |
| 206395_at | DGKG | -1,424023738 | -1,787089803 | 0,363066065 | |
| 208711_s_at | CCND1 | -1,424023738 | -1,787089803 | 0,363066065 | |
| 209652_s_at | PGF | -1,424023738 | -1,787089803 | 0,363066065 | |
| 211591_s_at | PDE4A | -1,424023738 | -1,787089803 | 0,363066065 | |
| 211616_s_at | HTR2A | -1,424023738 | -1,787089803 | 0,363066065 | |
| 214689_at | PAPPA2 | -1,424023738 | -1,787089803 | 0,363066065 | |
| 221051_s_at | NMRK2 | -1,424023738 | -1,787089803 | 0,363066065 | |
| 228471_at | ANKRD44 | -1,424023738 | -1,787089803 | 0,363066065 | |
| 230523_at | QSOX1 | -1,424023738 | -1,787089803 | 0,363066065 | |
| 231492_at | LOC100506034 | -1,424023738 | -1,787089803 | 0,363066065 | |
| 237349_at | - | -1,424023738 | -1,787089803 | 0,363066065 | |
| 241626_at | WTAP | -1,424023738 | -1,787089803 | 0,363066065 | |
| 243308_at | - | -1,424023738 | -1,787089803 | 0,363066065 | |
| 243702_at | - | -1,424023738 | -1,787089803 | 0,363066065 | |
| 204165_at | WASF1 | 4,779045678 | 4,41598058 | 0,363065098 | |
| 1555399_a_at | DUSP16 | -0,055687482 | -0,41858459 | 0,362897108 | |
| 1555550_at | ZACN | -0,055687482 | -0,41858459 | 0,362897108 | |
| 1569640_s_at | EEPD1 | -0,055687482 | -0,41858459 | 0,362897108 | |
| 229697_at | HIRIP3 | -0,055687482 | -0,41858459 | 0,362897108 | |
| 228123_s_at | ABHD12 | 2,485917452 | 2,123108223 | 0,362809228 | |
| 212799_at | STX6 | 2,791679321 | 2,428994491 | 0,362684831 | |
| 211050_x_at | LOC100134822 / | 0,715481152 | 0,352836757 | 0,362644394 | |
| 204849_at | DPH3P1 /// TCFL | 2,626954721 | 2,264418116 | 0,362536605 | |
| 224734_at | HMGB1 | 4,224629877 | 3,862161446 | 0,362468432 | |
| 202166_s_at | PPP1R2 | 2,814842651 | 2,452428893 | 0,362413758 | |
| 201933_at | CHMP1A | 2,830080806 | 2,467842989 | 0,362237816 | |
| 1559023_a_at | KIAA0494 | 0,44160593 | 0,079428135 | 0,362177795 | |
| 204005_s_at | PAWR | 1,442503549 | 1,080561626 | 0,361941923 | |
| 227514_at | ITPRIPL2 | 0,779676066 | 0,417770464 | 0,361905602 | |
| 230281_at | C16orf46 | 0,779676066 | 0,417770464 | 0,361905602 | |
| 225827_at | EIF2C2 | 1,05337236 | 0,691645685 | 0,361726674 | |
| 243405_at | - | 1,780386218 | 1,418668082 | 0,361718136 | |
| 208844_at | VDAC3 | 1,923647937 | 1,562009954 | 0,361637983 | |
| 212440_at | SNRNP27 | 3,815015946 | 3,453616097 | 0,361399849 | |
| 207733_x_at | PSG9 | 0,103263601 | -0,258114234 | 0,361377835 | |
| 208931_s_at | ILF3 | 5,970881696 | 5,60955773 | 0,361323966 | |
| 209677_at | PRKCI | 0,518867317 | 0,157549243 | 0,361318074 | |
| 212765_at | CAMSAP2 | 1,751865623 | 1,390636111 | 0,361229511 | |
| 224814_at | DPP7 | 2,406821352 | 2,045615873 | 0,36120548 | |
| 1553983_at | DTYMK | 1,897847661 | 1,536652708 | 0,361194953 | |
| 217822_at | WBP11 | 3,988699475 | 3,62753841 | 0,361161065 | |
| 207513_s_at | ZNF189 | 3,209137871 | 2,848075314 | 0,361062557 | |
| 1557137_at | TMEM17 | -0,285837228 | -0,646746079 | 0,36090885 | |
| 206132_at | MCC | -0,285837228 | -0,646746079 | 0,36090885 | |
| 210247_at | SYN2 | -0,285837228 | -0,646746079 | 0,36090885 | |

| | | | | | |
|---|---|---|---|---|---|
| 213075_at | OLFML2A | -0,285837228 | -0,646746079 | 0,36090885 | |
| 224045_x_at | LINC00470 | -0,285837228 | -0,646746079 | 0,36090885 | |
| 237270_at | LOC100505710 | -0,285837228 | -0,646746079 | 0,36090885 | |
| 238136_at | SHISA7 | -0,285837228 | -0,646746079 | 0,36090885 | |
| 204276_at | TK2 | 1,129248302 | 0,768343791 | 0,360904511 | |
| 201409_s_at | PPP1CB | 3,182077878 | 2,821324225 | 0,360753653 | |
| 227818_at | CEP85 | 2,981056773 | 2,620464265 | 0,360592508 | |
| 205770_at | GSR | 3,399920684 | 3,039473081 | 0,360447603 | |
| 231203_at | LOC100505478 | 0,20027154 | -0,160136748 | 0,360408288 | |
| 240121_x_at | - | 1,177702228 | 0,817300337 | 0,360401891 | |
| 204161_s_at | ENPP4 | 2,438982012 | 2,078620836 | 0,360361176 | |
| 225964_at | ZXDC | 1,504602299 | 1,144305291 | 0,360297008 | |
| 228366_at | - | 1,360921491 | 1,000684521 | 0,36023697 | |
| 205915_x_at | GRIN1 | -0,59053611 | -0,950686014 | 0,360149904 | |
| 205985_x_at | CLCNKB | -0,59053611 | -0,950686014 | 0,360149904 | |
| 206424_at | CYP26A1 | -0,59053611 | -0,950686014 | 0,360149904 | |
| 217544_at | - | -0,59053611 | -0,950686014 | 0,360149904 | |
| 239668_at | - | -0,59053611 | -0,950686014 | 0,360149904 | |
| 244108_at | SYNPO2 | -0,59053611 | -0,950686014 | 0,360149904 | |
| 218942_at | PIP4K2C | 3,641042878 | 3,281121096 | 0,359921783 | |
| 224982_at | AKT1S1 | 2,156646206 | 1,796776588 | 0,359869618 | |
| 1557375_at | - | -0,79540287 | -1,154897679 | 0,359494809 | |
| 203510_at | MET | -0,79540287 | -1,154897679 | 0,359494809 | |
| 205685_at | CD86 | -0,79540287 | -1,154897679 | 0,359494809 | |
| 205874_at | ITPKA | -0,79540287 | -1,154897679 | 0,359494809 | |
| 222245_s_at | FER1L4 | -0,79540287 | -1,154897679 | 0,359494809 | |
| 229621_x_at | EBF3 | -0,79540287 | -1,154897679 | 0,359494809 | |
| 231561_s_at | APOC2 | -0,79540287 | -1,154897679 | 0,359494809 | |
| 236950_s_at | LOC157381 | -0,79540287 | -1,154897679 | 0,359494809 | |
| 237415_at | - | -0,79540287 | -1,154897679 | 0,359494809 | |
| 239916_at | WDR16 | -0,79540287 | -1,154897679 | 0,359494809 | |
| 222478_at | VPS36 | 3,158127685 | 2,798646381 | 0,359481305 | |
| 210098_s_at | - | 2,680059304 | 2,320681318 | 0,359377987 | |
| 218748_s_at | EXOC5 | 2,281618189 | 1,922365006 | 0,359253183 | |
| 1560404_a_at | ATPBD4 | -2,779459409 | -3,138626879 | 0,35916747 | |
| 202196_s_at | DKK3 | -2,779459409 | -3,138626879 | 0,35916747 | |
| 217012_at | - | -2,779459409 | -3,138626879 | 0,35916747 | |
| 228695_at | C8orf46 | -2,779459409 | -3,138626879 | 0,35916747 | |
| 236892_s_at | HOXB-AS3 | -2,779459409 | -3,138626879 | 0,35916747 | |
| 238411_x_at | NREP | -2,779459409 | -3,138626879 | 0,35916747 | |
| 240103_at | - | -2,779459409 | -3,138626879 | 0,35916747 | |
| 241088_at | - | -2,779459409 | -3,138626879 | 0,35916747 | |
| 220583_at | - | 0,334552598 | -0,024572586 | 0,359125185 | |
| 222312_s_at | - | 0,334552598 | -0,024572586 | 0,359125185 | |
| 218978_s_at | SLC25A37 | 2,171078719 | 1,811954245 | 0,359124475 | |
| 214093_s_at | FUBP1 | 2,303618506 | 1,944570415 | 0,359048091 | |
| 235542_at | TET3 | 2,303618506 | 1,944570415 | 0,359048091 | |
| 1564277_a_at | LOC100133920 / | 1,063722698 | 0,704715219 | 0,359007479 | |
| 217303_s_at | ADRB3 | -0,496991414 | -0,855751026 | 0,358759612 | |
| 223642_at | ZIC2 | 2,077135162 | 1,718396606 | 0,358738556 | |
| 1557438_at | - | -2,262747984 | -2,621331989 | 0,358584005 | |
| 1557759_at | ATP5SL | -2,262747984 | -2,621331989 | 0,358584005 | |
| 1559350_at | - | -2,262747984 | -2,621331989 | 0,358584005 | |
| 1561211_at | - | -2,262747984 | -2,621331989 | 0,358584005 | |
| 1562669_at | - | -2,262747984 | -2,621331989 | 0,358584005 | |
| 205751_at | SH3GL2 | -2,262747984 | -2,621331989 | 0,358584005 | |

| | | | | | |
|---|---|---|---|---|---|
| 206033_s_at | DSC3 | -2,262747984 | -2,621331989 | 0,358584005 | |
| 215058_at | DENND5B | -2,262747984 | -2,621331989 | 0,358584005 | |
| 216764_at | - | -2,262747984 | -2,621331989 | 0,358584005 | |
| 217551_at | OR7E14P | -2,262747984 | -2,621331989 | 0,358584005 | |
| 221019_s_at | COLEC12 | -2,262747984 | -2,621331989 | 0,358584005 | |
| 224075_s_at | VSX1 | -2,262747984 | -2,621331989 | 0,358584005 | |
| 224771_at | NAV1 | -2,262747984 | -2,621331989 | 0,358584005 | |
| 237375_at | LOC100506816 | -2,262747984 | -2,621331989 | 0,358584005 | |
| 238091_at | LOC100506388 | -2,262747984 | -2,621331989 | 0,358584005 | |
| 240460_at | - | -2,262747984 | -2,621331989 | 0,358584005 | |
| 240938_at | - | -2,262747984 | -2,621331989 | 0,358584005 | |
| 241455_at | C6orf132 | -2,262747984 | -2,621331989 | 0,358584005 | |
| 242262_x_at | - | -2,262747984 | -2,621331989 | 0,358584005 | |
| 242295_at | FLJ32955 | -2,262747984 | -2,621331989 | 0,358584005 | |
| 242563_at | - | -2,262747984 | -2,621331989 | 0,358584005 | |
| 210758_at | PSIP1 | 0,417596971 | 0,059218869 | 0,358378102 | |
| 222096_x_at | LOC100507284 | 0,417596971 | 0,059218869 | 0,358378102 | |
| 205282_at | LRP8 | 1,512180019 | 1,153866761 | 0,358313257 | |
| 212277_at | MTMR4 | 1,716223609 | 1,3579181 | 0,358305509 | |
| 213606_s_at | ARHGDIA | 1,163336153 | 0,805216064 | 0,358120089 | |
| 229870_at | LOC644656 | 0,853119696 | 0,495031851 | 0,358087845 | |
| 212417_at | SCAMP1 | 1,549482847 | 1,191491281 | 0,357991566 | |
| 200872_at | S100A10 | 4,105389286 | 3,747642277 | 0,357747009 | |
| 203678_at | FAN1 | 1,883312263 | 1,525647381 | 0,357664882 | |
| 1556715_at | PRPSAP1 | 0,255492549 | -0,102100538 | 0,357593087 | |
| 1568838_at | WASIR2 | 0,255492549 | -0,102100538 | 0,357593087 | |
| 1559504_at | MIR137HG /// MI | -3,114791413 | -3,472251727 | 0,357460314 | |
| 215152_at | MYB | 1,094335495 | 0,73688026 | 0,357455234 | |
| 206667_s_at | SCAMP1 | 0,533835206 | 0,176436073 | 0,357399133 | |
| 213389_at | ZNF592 | 0,533835206 | 0,176436073 | 0,357399133 | |
| 216293_at | CLTA | 0,533835206 | 0,176436073 | 0,357399133 | |
| 214574_x_at | LST1 | 1,735773781 | 1,378453643 | 0,357320138 | |
| 1561127_at | ADARB2-AS1 | -0,033456564 | -0,39061235 | 0,357155786 | |
| 215821_x_at | PSG3 | -0,033456564 | -0,39061235 | 0,357155786 | |
| 219663_s_at | TMEM121 | -0,033456564 | -0,39061235 | 0,357155786 | |
| 220915_s_at | - | -0,033456564 | -0,39061235 | 0,357155786 | |
| 233439_at | LETM1 | -0,033456564 | -0,39061235 | 0,357155786 | |
| 244028_at | USP15 | -0,033456564 | -0,39061235 | 0,357155786 | |
| 1557427_at | - | -2,597496523 | -2,954576755 | 0,357080232 | |
| 1560506_at | - | -2,597496523 | -2,954576755 | 0,357080232 | |
| 1561617_at | DNAH6 | -2,597496523 | -2,954576755 | 0,357080232 | |
| 1562094_at | - | -2,597496523 | -2,954576755 | 0,357080232 | |
| 1562420_at | - | -2,597496523 | -2,954576755 | 0,357080232 | |
| 206293_at | SULT2A1 | -2,597496523 | -2,954576755 | 0,357080232 | |
| 211131_s_at | EDA | -2,597496523 | -2,954576755 | 0,357080232 | |
| 217575_s_at | - | -2,597496523 | -2,954576755 | 0,357080232 | |
| 233109_at | COL12A1 | -2,597496523 | -2,954576755 | 0,357080232 | |
| 237354_at | PDXDC1 | -2,597496523 | -2,954576755 | 0,357080232 | |
| 227963_at | - | 1,143956113 | 0,786897721 | 0,357058392 | |
| 203527_s_at | APC | 0,606430281 | 0,249604176 | 0,356826106 | |
| 221743_at | CELF1 | 5,023325284 | 4,666563551 | 0,356761733 | |
| 201542_at | SAR1A | 4,776688188 | 4,419948427 | 0,356739761 | |
| 231320_at | EBP | 0,384950573 | 0,028364014 | 0,356586559 | |
| 210280_at | MPZ | 0,072842263 | -0,283667828 | 0,35651009 | |
| 211350_s_at | KIF25-AS1 | 0,072842263 | -0,283667828 | 0,35651009 | |
| 228354_at | MORN4 | 0,072842263 | -0,283667828 | 0,35651009 | |

| | | | | | |
|---|---|---|---|---|---|
| 238446_at | NAIP | 0,072842263 | -0,283667828 | 0,35651009 | |
| 242408_at | STYX | 0,072842263 | -0,283667828 | 0,35651009 | |
| 209786_at | HMGN4 | 4,667677441 | 4,311461649 | 0,356215793 | |
| 223352_s_at | C17orf80 | 1,556829052 | 1,200746069 | 0,356082982 | |
| 228867_at | TATDN3 | 0,46522187 | 0,109221392 | 0,356000478 | |
| 231700_at | GUCA1A | 0,46522187 | 0,109221392 | 0,356000478 | |
| 218701_at | LACTB2 | 2,626954721 | 2,271023131 | 0,35593159 | |
| 242751_at | - | 1,305306849 | 0,949447784 | 0,355859066 | |
| 200902_at | 42248 | 5,365737993 | 5,010057188 | 0,355680806 | |
| 1552794_a_at | ZNF547 | -1,308181863 | -1,663414495 | 0,355232632 | |
| 1559996_s_at | SAMD14 | -1,308181863 | -1,663414495 | 0,355232632 | |
| 1563118_at | - | -1,308181863 | -1,663414495 | 0,355232632 | |
| 1563793_at | LOC100130278 | -1,308181863 | -1,663414495 | 0,355232632 | |
| 1569337_at | SLC5A9 | -1,308181863 | -1,663414495 | 0,355232632 | |
| 1569885_at | LOC100302640 | -1,308181863 | -1,663414495 | 0,355232632 | |
| 206181_at | SLAMF1 | -1,308181863 | -1,663414495 | 0,355232632 | |
| 206690_at | ASIC2 | -1,308181863 | -1,663414495 | 0,355232632 | |
| 207024_at | CHRND | -1,308181863 | -1,663414495 | 0,355232632 | |
| 215049_x_at | CD163 | -1,308181863 | -1,663414495 | 0,355232632 | |
| 215959_at | - | -1,308181863 | -1,663414495 | 0,355232632 | |
| 217587_at | - | -1,308181863 | -1,663414495 | 0,355232632 | |
| 226272_at | RCAN3 | -1,308181863 | -1,663414495 | 0,355232632 | |
| 233554_at | - | -1,308181863 | -1,663414495 | 0,355232632 | |
| 238712_at | - | -1,308181863 | -1,663414495 | 0,355232632 | |
| 241767_at | - | -1,308181863 | -1,663414495 | 0,355232632 | |
| 243043_at | - | -1,308181863 | -1,663414495 | 0,355232632 | |
| 201016_at | EIF1AX | 5,776339397 | 5,42111841 | 0,355220987 | |
| 205953_at | LRIG2 | 1,835789711 | 1,480766833 | 0,355022878 | |
| 228645_at | SNHG9 /// SNOR | 1,835789711 | 1,480766833 | 0,355022878 | |
| 222419_x_at | UBE2H | 0,30867765 | -0,046302147 | 0,354979797 | |
| 202160_at | CREBBP | 3,416167722 | 3,061230246 | 0,354937476 | |
| 243856_at | LANCL3 | 0,648296226 | 0,293518836 | 0,35477739 | |
| 1557321_a_at | CAPN14 | -2,823166348 | -3,177802286 | 0,354635938 | |
| 1562464_at | - | -2,823166348 | -3,177802286 | 0,354635938 | |
| 1566169_at | - | -2,823166348 | -3,177802286 | 0,354635938 | |
| 207932_at | IFNA8 | -2,823166348 | -3,177802286 | 0,354635938 | |
| 222359_x_at | KDSR | -2,823166348 | -3,177802286 | 0,354635938 | |
| 234453_s_at | C14orf166B | -2,823166348 | -3,177802286 | 0,354635938 | |
| 241083_at | - | -2,823166348 | -3,177802286 | 0,354635938 | |
| 241578_x_at | - | -2,823166348 | -3,177802286 | 0,354635938 | |
| 1569714_at | ZFYVE20 | -0,136301282 | -0,490927335 | 0,354626053 | |
| 204678_s_at | KCNK1 | -0,136301282 | -0,490927335 | 0,354626053 | |
| 211873_s_at | PCDHGA9 | -0,136301282 | -0,490927335 | 0,354626053 | |
| 221558_s_at | LEF1 | -0,136301282 | -0,490927335 | 0,354626053 | |
| 240252_at | - | -0,136301282 | -0,490927335 | 0,354626053 | |
| 210320_s_at | DDX52 | 1,649074067 | 1,294497133 | 0,354576934 | |
| 231124_x_at | LY9 | -0,259832361 | -0,614371577 | 0,354539215 | |
| 239933_x_at | C14orf45 | -0,259832361 | -0,614371577 | 0,354539215 | |
| 212239_at | PIK3R1 | 2,640847372 | 2,286318242 | 0,35452913 | |
| 204513_s_at | ELMO1 | 3,043834719 | 2,689475799 | 0,35435892 | |
| 1556762_a_at | - | -0,655928373 | -1,010192375 | 0,354264002 | |
| 201325_s_at | EMP1 | -0,655928373 | -1,010192375 | 0,354264002 | |
| 205611_at | TNFSF12 /// TNF | -0,655928373 | -1,010192375 | 0,354264002 | |
| 205808_at | ASPH | -0,655928373 | -1,010192375 | 0,354264002 | |
| 208452_x_at | MYO9B | -0,655928373 | -1,010192375 | 0,354264002 | |
| 211705_s_at | SORBS1 | -0,655928373 | -1,010192375 | 0,354264002 | |

| | | | | | |
|---|---|---|---|---|---|
| 221373_x_at | PSPN | -0,655928373 | -1,010192375 | 0,354264002 | |
| 222054_at | PPIEL | -0,655928373 | -1,010192375 | 0,354264002 | |
| 231262_at | - | -0,655928373 | -1,010192375 | 0,354264002 | |
| 232849_at | LOC100128988 | -0,655928373 | -1,010192375 | 0,354264002 | |
| 234162_at | - | -0,655928373 | -1,010192375 | 0,354264002 | |
| 239689_at | - | -0,655928373 | -1,010192375 | 0,354264002 | |
| 202241_at | TRIB1 | 4,082030648 | 3,727773127 | 0,354257521 | |
| 232513_x_at | FAM209B | 0,747935635 | 0,393761504 | 0,354174131 | |
| 222638_s_at | TMEM242 | 3,327680494 | 2,973511251 | 0,354169244 | |
| 1562788_at | LOC254099 | 0,03125738 | -0,322870281 | 0,354127661 | |
| 210823_s_at | PTPRS | 0,03125738 | -0,322870281 | 0,354127661 | |
| 235263_at | STAG3L1 /// STA | 0,03125738 | -0,322870281 | 0,354127661 | |
| 1552813_at | KLF14 | -0,325749314 | -0,679763839 | 0,354014525 | |
| 207788_s_at | SORBS3 | -0,325749314 | -0,679763839 | 0,354014525 | |
| 220047_at | SIRT4 | -0,325749314 | -0,679763839 | 0,354014525 | |
| 228626_at | URM1 | -0,325749314 | -0,679763839 | 0,354014525 | |
| 208511_at | PTTG3P | 2,520143371 | 2,166264721 | 0,353878649 | |
| 209283_at | CRYAB | 0,810733188 | 0,456918297 | 0,35381489 | |
| 208070_s_at | REV3L | 2,912562019 | 2,558821855 | 0,353740165 | |
| 221248_s_at | WHSC1L1 | 0,133056859 | -0,220592223 | 0,353649082 | |
| 235365_at | DFNB59 | 0,133056859 | -0,220592223 | 0,353649082 | |
| 235491_at | ZBTB10 | 0,133056859 | -0,220592223 | 0,353649082 | |
| 236343_at | - | 0,133056859 | -0,220592223 | 0,353649082 | |
| 202541_at | AIMP1 | 2,827046023 | 2,473581079 | 0,353464944 | |
| 210076_x_at | SERBP1 | 4,357025868 | 4,003717379 | 0,353308489 | |
| 1565714_at | - | -0,759588698 | -1,112851844 | 0,353263146 | |
| 1570338_at | - | -0,759588698 | -1,112851844 | 0,353263146 | |
| 204888_s_at | NEURL | -0,759588698 | -1,112851844 | 0,353263146 | |
| 211879_x_at | PCDHGA3 | -0,759588698 | -1,112851844 | 0,353263146 | |
| 220158_at | LGALS14 | -0,759588698 | -1,112851844 | 0,353263146 | |
| 229931_at | ZNF775 | -0,759588698 | -1,112851844 | 0,353263146 | |
| 231686_at | GATM | -0,759588698 | -1,112851844 | 0,353263146 | |
| 233144_s_at | RASAL1 | -0,759588698 | -1,112851844 | 0,353263146 | |
| 242903_at | IFNGR1 | -0,759588698 | -1,112851844 | 0,353263146 | |
| 1558431_at | NHLRC4 | 1,567778694 | 1,214517879 | 0,353260815 | |
| 221737_at | GNA12 | 1,567778694 | 1,214517879 | 0,353260815 | |
| 1554777_at | ZFP42 | -2,64318869 | -2,996335703 | 0,353147013 | |
| 1560104_at | - | -2,64318869 | -2,996335703 | 0,353147013 | |
| 1568868_at | CYP27C1 | -2,64318869 | -2,996335703 | 0,353147013 | |
| 206349_at | LGI1 | -2,64318869 | -2,996335703 | 0,353147013 | |
| 213496_at | LPPR4 | -2,64318869 | -2,996335703 | 0,353147013 | |
| 224043_s_at | UPB1 | -2,64318869 | -2,996335703 | 0,353147013 | |
| 232099_at | PCDHB16 | -2,64318869 | -2,996335703 | 0,353147013 | |
| 239942_at | - | -2,64318869 | -2,996335703 | 0,353147013 | |
| 240246_at | LOC642236 | -2,64318869 | -2,996335703 | 0,353147013 | |
| 243936_x_at | - | -2,64318869 | -2,996335703 | 0,353147013 | |
| 208770_s_at | EIF4EBP2 | 3,042527069 | 2,689475799 | 0,35305127 | |
| 237400_at | - | 0,95671774 | 0,603679647 | 0,353038093 | |
| 229436_x_at | BRCC3 | 1,783520648 | 1,43051703 | 0,353003618 | |
| 1570197_at | - | 0,273439642 | -0,079522948 | 0,35296259 | |
| 210077_s_at | SRSF5 | 0,273439642 | -0,079522948 | 0,35296259 | |
| 211077_s_at | TLK1 | 0,273439642 | -0,079522948 | 0,35296259 | |
| 220022_at | ZNF334 | 0,273439642 | -0,079522948 | 0,35296259 | |
| 230364_at | CHPT1 | 0,273439642 | -0,079522948 | 0,35296259 | |
| 238209_at | - | 0,273439642 | -0,079522948 | 0,35296259 | |
| 226579_at | - | 3,074867907 | 2,721978587 | 0,35288932 | |

| | | | | | |
|---|---|---|---|---|---|
| 202506_at | SSFA2 | 3,166555554 | 2,813804482 | 0,352751072 | |
| 1552478_a_at | IRF6 | 0,359971644 | 0,007421914 | 0,35254973 | |
| 1559426_at | - | 0,359971644 | 0,007421914 | 0,35254973 | |
| 207442_at | CSF3 | 0,359971644 | 0,007421914 | 0,35254973 | |
| 215903_s_at | MAST2 | 1,339784194 | 0,987376598 | 0,352407597 | |
| 227604_at | TMEM185B | 1,089278259 | 0,73688026 | 0,352397999 | |
| 222329_x_at | - | 1,139070151 | 0,786897721 | 0,352172429 | |
| 1566302_at | PPP1R11 | -0,467091869 | -0,819238336 | 0,352146467 | |
| 213873_at | ST3GAL6 | -0,467091869 | -0,819238336 | 0,352146467 | |
| 227655_at | LOC100505806 | -0,467091869 | -0,819238336 | 0,352146467 | |
| 227667_at | CUEDC1 | -0,467091869 | -0,819238336 | 0,352146467 | |
| 239315_at | FAM115C | -0,467091869 | -0,819238336 | 0,352146467 | |
| 241754_at | SCAI | -0,467091869 | -0,819238336 | 0,352146467 | |
| 1554557_at | ATP11B | 1,62481334 | 1,272721751 | 0,352091589 | |
| 214830_at | SLC38A6 | 3,919150955 | 3,56709884 | 0,352052115 | |
| 210387_at | HIST1H2BC /// H | 2,890927111 | 2,538891651 | 0,35203546 | |
| 206614_at | GDF5 | 0,480753764 | 0,128747141 | 0,352006623 | |
| 227943_at | - | 0,480753764 | 0,128747141 | 0,352006623 | |
| 229946_at | FAM168B | 0,480753764 | 0,128747141 | 0,352006623 | |
| 236049_at | WDR90 | 0,480753764 | 0,128747141 | 0,352006623 | |
| 218255_s_at | FBRS | 1,187200746 | 0,835239052 | 0,351961694 | |
| 222763_s_at | SFT2D3 /// WDR | 3,684447666 | 3,332545245 | 0,351902421 | |
| 212051_at | WIPF2 | 2,790121796 | 2,438805127 | 0,351316669 | |
| 211665_s_at | SOS2 | 0,661985843 | 0,310717132 | 0,351268711 | |
| 64438_at | C17orf101 | 1,512180019 | 1,160996507 | 0,351183512 | |
| 1556263_s_at | PWRN1 | -2,384869671 | -2,735862426 | 0,350992754 | |
| 1556410_a_at | KRTAP19-1 | -2,384869671 | -2,735862426 | 0,350992754 | |
| 1563182_at | ACVR1C | -2,384869671 | -2,735862426 | 0,350992754 | |
| 1568882_at | LRTOMT | -2,384869671 | -2,735862426 | 0,350992754 | |
| 211923_s_at | ZNF471 | -2,384869671 | -2,735862426 | 0,350992754 | |
| 215352_at | GIMAP1-GIMAP5 | -2,384869671 | -2,735862426 | 0,350992754 | |
| 225645_at | EHF | -2,384869671 | -2,735862426 | 0,350992754 | |
| 228855_at | NUDT7 | -2,384869671 | -2,735862426 | 0,350992754 | |
| 231503_at | LOC100507193 | -2,384869671 | -2,735862426 | 0,350992754 | |
| 231551_at | LOC729059 | -2,384869671 | -2,735862426 | 0,350992754 | |
| 234523_at | - | -2,384869671 | -2,735862426 | 0,350992754 | |
| 235740_at | MCTP1 | -2,384869671 | -2,735862426 | 0,350992754 | |
| 242653_at | DCC | -2,384869671 | -2,735862426 | 0,350992754 | |
| 243172_at | - | -2,384869671 | -2,735862426 | 0,350992754 | |
| 243254_at | HIVEP2 | -2,384869671 | -2,735862426 | 0,350992754 | |
| 1552293_at | TMEM196 | -1,026571149 | -1,377419394 | 0,350848245 | |
| 1553323_a_at | CATSPER2 | -1,026571149 | -1,377419394 | 0,350848245 | |
| 1558930_at | LINC00460 | -1,026571149 | -1,377419394 | 0,350848245 | |
| 1569052_at | - | -1,026571149 | -1,377419394 | 0,350848245 | |
| 203888_at | THBD | -1,026571149 | -1,377419394 | 0,350848245 | |
| 216277_at | BUB1 | -1,026571149 | -1,377419394 | 0,350848245 | |
| 223834_at | CD274 | -1,026571149 | -1,377419394 | 0,350848245 | |
| 229685_at | LOC100134937 | -1,026571149 | -1,377419394 | 0,350848245 | |
| 236294_at | HUWE1 | -1,026571149 | -1,377419394 | 0,350848245 | |
| 237308_at | - | -1,026571149 | -1,377419394 | 0,350848245 | |
| 240730_at | - | -1,026571149 | -1,377419394 | 0,350848245 | |
| 226339_at | TRUB1 | 3,822654227 | 3,471887144 | 0,350767083 | |
| 216559_x_at | - | 4,982993627 | 4,632345568 | 0,350648058 | |
| 223622_s_at | HYI | 0,882651021 | 0,532164371 | 0,35048665 | |
| 243046_at | - | 0,882651021 | 0,532164371 | 0,35048665 | |
| 225740_x_at | MDM4 | 3,547344317 | 3,196911138 | 0,350433179 | |

| | | | | | |
|---|---|---|---|---|---|
| 210645_s_at | TTC3 /// TTC3P1 | 5,842829726 | 5,492446869 | 0,350382857 | |
| 226389_s_at | RAPGEF1 | 1,326951551 | 0,976641161 | 0,35031039 | |
| 1555180_at | LOC100132686 | -1,812418401 | -2,162639771 | 0,35022137 | |
| 1564238_a_at | WDR49 | -1,812418401 | -2,162639771 | 0,35022137 | |
| 204261_s_at | PSEN2 | -1,812418401 | -2,162639771 | 0,35022137 | |
| 214024_s_at | DGCR6L | -1,812418401 | -2,162639771 | 0,35022137 | |
| 221198_at | SCT | -1,812418401 | -2,162639771 | 0,35022137 | |
| 224216_at | - | -1,812418401 | -2,162639771 | 0,35022137 | |
| 228417_at | ISYNA1 | -1,812418401 | -2,162639771 | 0,35022137 | |
| 232070_at | LOC100506639 | -1,812418401 | -2,162639771 | 0,35022137 | |
| 232123_at | LOC283174 | -1,812418401 | -2,162639771 | 0,35022137 | |
| 233978_at | PTPRE | -1,812418401 | -2,162639771 | 0,35022137 | |
| 234067_at | - | -1,812418401 | -2,162639771 | 0,35022137 | |
| 236148_at | LOC100507568 | -1,812418401 | -2,162639771 | 0,35022137 | |
| 236169_at | - | -1,812418401 | -2,162639771 | 0,35022137 | |
| 237674_at | - | -1,812418401 | -2,162639771 | 0,35022137 | |
| 239544_at | - | -1,812418401 | -2,162639771 | 0,35022137 | |
| 239967_at | - | -1,812418401 | -2,162639771 | 0,35022137 | |
| 240141_at | - | -1,812418401 | -2,162639771 | 0,35022137 | |
| 243593_s_at | - | -1,812418401 | -2,162639771 | 0,35022137 | |
| 224185_at | WRAP53 | 1,666156729 | 1,315948728 | 0,350208 | |
| 225760_at | MYSM1 | 3,217273782 | 2,867091644 | 0,350182137 | |
| 219203_at | EMC9 | 2,348755185 | 1,998633167 | 0,350122018 | |
| 206526_at | RIBC2 | 1,021868205 | 0,67181667 | 0,350051535 | |
| 205356_at | USP13 | 1,073999308 | 0,724100169 | 0,349899139 | |
| 209947_at | UBAP2L | 2,035673598 | 1,685812099 | 0,349861499 | |
| 1558152_at | LOC100131262 | 2,701760055 | 2,352169913 | 0,349590142 | |
| 228142_at | UQCR10 | 2,185368281 | 1,835911187 | 0,349457094 | |
| 1559094_at | FBXO9 | 0,702292136 | 0,352836757 | 0,349455379 | |
| 214309_s_at | C21orf2 | 0,330272223 | -0,019180479 | 0,349452702 | |
| 223178_s_at | NT5DC1 | 2,758612869 | 2,409170778 | 0,349442091 | |
| 1555610_at | AGK | -1,688127507 | -2,037551727 | 0,34942422 | |
| 1559580_at | LRRC39 | -1,688127507 | -2,037551727 | 0,34942422 | |
| 1563894_at | LOC441178 | -1,688127507 | -2,037551727 | 0,34942422 | |
| 1566689_at | - | -1,688127507 | -2,037551727 | 0,34942422 | |
| 208470_s_at | HP /// HPR | -1,688127507 | -2,037551727 | 0,34942422 | |
| 214421_x_at | CYP2C9 | -1,688127507 | -2,037551727 | 0,34942422 | |
| 216648_s_at | RREB1 | -1,688127507 | -2,037551727 | 0,34942422 | |
| 219730_at | MED18 | -1,688127507 | -2,037551727 | 0,34942422 | |
| 222796_at | ATP5J2-PTCD1 / | -1,688127507 | -2,037551727 | 0,34942422 | |
| 230849_at | KCNA1 | -1,688127507 | -2,037551727 | 0,34942422 | |
| 234171_at | - | -1,688127507 | -2,037551727 | 0,34942422 | |
| 235834_at | CALD1 | -1,688127507 | -2,037551727 | 0,34942422 | |
| 238240_at | - | -1,688127507 | -2,037551727 | 0,34942422 | |
| 238302_at | - | -1,688127507 | -2,037551727 | 0,34942422 | |
| 239394_at | SLC6A2 | -1,688127507 | -2,037551727 | 0,34942422 | |
| 243220_at | - | -1,688127507 | -2,037551727 | 0,34942422 | |
| 244321_at | PGAP1 | -1,688127507 | -2,037551727 | 0,34942422 | |
| 211493_x_at | DTNA | 0,767063567 | 0,417770464 | 0,349293103 | |
| 225078_at | EMP2 | 0,767063567 | 0,417770464 | 0,349293103 | |
| 205789_at | CD1D | 0,82905153 | 0,479907041 | 0,349144489 | |
| 209859_at | TRIM9 | 0,82905153 | 0,479907041 | 0,349144489 | |
| 229283_at | LOC728613 | 0,82905153 | 0,479907041 | 0,349144489 | |
| 219109_at | SPAG16 | 1,799091453 | 1,450051551 | 0,349039903 | |
| 1559929_at | - | -1,937711681 | -2,286583451 | 0,348871769 | |
| 1560141_at | LOC100133039 | -1,937711681 | -2,286583451 | 0,348871769 | |

| | | | | | |
|---|---|---|---|---|---|
| 1569701_at | PER3 | -1,937711681 | -2,286583451 | 0,348871769 | |
| 206805_at | SEMA3A | -1,937711681 | -2,286583451 | 0,348871769 | |
| 208554_at | POU4F3 | -1,937711681 | -2,286583451 | 0,348871769 | |
| 215432_at | ACSM1 | -1,937711681 | -2,286583451 | 0,348871769 | |
| 224285_at | GPR174 | -1,937711681 | -2,286583451 | 0,348871769 | |
| 228150_at | SEC16B | -1,937711681 | -2,286583451 | 0,348871769 | |
| 230510_at | HSPB9 | -1,937711681 | -2,286583451 | 0,348871769 | |
| 231980_at | DOK6 | -1,937711681 | -2,286583451 | 0,348871769 | |
| 234558_at | - | -1,937711681 | -2,286583451 | 0,348871769 | |
| 240778_at | GINS4 | -1,937711681 | -2,286583451 | 0,348871769 | |
| 242708_at | - | -1,937711681 | -2,286583451 | 0,348871769 | |
| 243820_at | LOC401463 | -1,937711681 | -2,286583451 | 0,348871769 | |
| 236781_at | - | -0,00073712 | -0,34958478 | 0,34884766 | |
| 238307_at | - | -0,00073712 | -0,34958478 | 0,34884766 | |
| 224090_s_at | TNFRSF19 | -0,112808516 | -0,461594427 | 0,34878591 | |
| 208284_x_at | GGT1 /// GGT2 // | 0,152582607 | -0,19610998 | 0,348692587 | |
| 216579_at | GJB4 | 0,152582607 | -0,19610998 | 0,348692587 | |
| 220692_at | MIR4448 | 0,152582607 | -0,19610998 | 0,348692587 | |
| 228249_at | C11orf74 | 1,973904239 | 1,6252386 | 0,348665638 | |
| 231878_at | C16orf53 | 1,58584542 | 1,23718272 | 0,348662701 | |
| 226680_at | IKZF5 | 1,621313976 | 1,272721751 | 0,348592226 | |
| 221428_s_at | TBL1XR1 | 2,974214633 | 2,625627364 | 0,348587269 | |
| 230034_x_at | MRPL41 | 0,246434561 | -0,102100538 | 0,348535099 | |
| 234858_at | - | 0,246434561 | -0,102100538 | 0,348535099 | |
| 210362_x_at | PML | 1,224581536 | 0,876248481 | 0,348333055 | |
| 1555809_at | CRISPLD2 | 0,376672223 | 0,028364014 | 0,348308209 | |
| 243664_at | TXNL1 | 0,376672223 | 0,028364014 | 0,348308209 | |
| 205377_s_at | ACHE | 0,457392764 | 0,109221392 | 0,348171372 | |
| 238818_at | KIAA1429 | 0,457392764 | 0,109221392 | 0,348171372 | |
| 1553111_a_at | KBTBD6 | 0,57058931 | 0,222599095 | 0,347990216 | |
| 211691_x_at | - | 0,57058931 | 0,222599095 | 0,347990216 | |
| 227821_at | LGI4 | 0,57058931 | 0,222599095 | 0,347990216 | |
| 238996_x_at | ALDOA | 4,208686884 | 3,860702537 | 0,347984347 | |
| 1557424_at | LOC100505878 | -3,054236653 | -3,402185466 | 0,347948813 | |
| 240863_at | CYP19A1 | -3,054236653 | -3,402185466 | 0,347948813 | |
| 205340_at | ZBTB24 | 2,724770722 | 2,376875125 | 0,347895597 | |
| 212981_s_at | FAM115A /// LOC | 4,362800281 | 4,014909928 | 0,347890353 | |
| 226533_at | HINT3 | 0,641402394 | 0,293518836 | 0,347883559 | |
| 231872_at | LRRCC1 | 0,641402394 | 0,293518836 | 0,347883559 | |
| 1556346_at | COTL1 | 1,515954005 | 1,16809119 | 0,347862815 | |
| 1560888_x_at | - | -2,43257096 | -2,780313924 | 0,347742965 | |
| 1561010_a_at | MAOB | -2,43257096 | -2,780313924 | 0,347742965 | |
| 1561567_at | - | -2,43257096 | -2,780313924 | 0,347742965 | |
| 209540_at | IGF1 | -2,43257096 | -2,780313924 | 0,347742965 | |
| 216255_s_at | GRM8 | -2,43257096 | -2,780313924 | 0,347742965 | |
| 222932_at | EHF | -2,43257096 | -2,780313924 | 0,347742965 | |
| 224219_s_at | TRPC4 | -2,43257096 | -2,780313924 | 0,347742965 | |
| 234686_at | SUGT1P1 | -2,43257096 | -2,780313924 | 0,347742965 | |
| 241132_at | - | -2,43257096 | -2,780313924 | 0,347742965 | |
| 241142_at | - | -2,43257096 | -2,780313924 | 0,347742965 | |
| 202823_at | TCEB1 | 3,497632487 | 3,149904996 | 0,347727491 | |
| 1556319_at | LOC283270 | -0,299034815 | -0,646746079 | 0,347711264 | |
| 1556855_a_at | LOC283501 | -0,299034815 | -0,646746079 | 0,347711264 | |
| 1558230_at | SF3B2 | -0,299034815 | -0,646746079 | 0,347711264 | |
| 210002_at | GATA6 | -0,299034815 | -0,646746079 | 0,347711264 | |
| 239396_at | - | -0,299034815 | -0,646746079 | 0,347711264 | |

| | | | | | |
|---|---|---|---|---|---|
| 236852_at | FBXO43 | 1,348276312 | 1,000684521 | 0,34759179 | |
| 212138_at | PDS5A | 4,672757696 | 4,32527877 | 0,347478926 | |
| 222946_s_at | C1orf135 | 1,835789711 | 1,488344553 | 0,347445158 | |
| 225607_at | CCDC43 | 2,600539826 | 2,253342112 | 0,347197713 | |
| 212857_x_at | SUB1 | 6,700972236 | 6,353842334 | 0,347129902 | |
| 201756_at | RPA2 | 5,173943153 | 4,826818428 | 0,347124725 | |
| 222498_at | AZI2 | 1,296557217 | 0,949447784 | 0,347109433 | |
| 200774_at | FAM120A | 5,144076207 | 4,796999275 | 0,347076933 | |
| 221168_at | PRDM13 | 2,776027875 | 2,428994491 | 0,347033385 | |
| 233019_at | CNOT7 | 1,593009234 | 1,246149888 | 0,346859346 | |
| 224781_s_at | RBM17 | 4,584760517 | 4,238072872 | 0,346687645 | |
| 212989_at | SGMS1 | 2,59519831 | 2,248887791 | 0,346310519 | |
| 224945_at | BTBD7 | 3,474545391 | 3,128242026 | 0,346303364 | |
| 212523_s_at | KIAA0146 | 0,209623097 | -0,136643983 | 0,34626708 | |
| 226716_at | PRR12 | 0,209623097 | -0,136643983 | 0,34626708 | |
| 227489_at | SMURF2 | 0,209623097 | -0,136643983 | 0,34626708 | |
| 232756_at | KALRN | 0,209623097 | -0,136643983 | 0,34626708 | |
| 233605_x_at | HNRNPM | 0,209623097 | -0,136643983 | 0,34626708 | |
| 221210_s_at | NPL | 0,425644434 | 0,079428135 | 0,346216298 | |
| 209689_at | CCDC93 | 0,503742507 | 0,157549243 | 0,346193264 | |
| 205857_at | SLC18A2 | 0,613492853 | 0,26733076 | 0,346162094 | |
| 227657_at | RNF150 | 0,613492853 | 0,26733076 | 0,346162094 | |
| 205210_at | TGFBRAP1 | 0,779676066 | 0,433557298 | 0,346118767 | |
| 233754_x_at | ZNF71 | 0,841135803 | 0,495031851 | 0,346103952 | |
| 1558801_at | - | 1,037706275 | 0,691645685 | 0,34606059 | |
| 231913_s_at | BRCC3 | 1,210676994 | 0,864649967 | 0,346027027 | |
| 1557675_at | RAF1 | 1,523472489 | 1,177496821 | 0,345975668 | |
| 229610_at | CKAP2L | 2,064305961 | 1,718396606 | 0,345909355 | |
| 1555279_at | ARMC8 | 2,456762221 | 2,110887591 | 0,34587463 | |
| 214041_x_at | RPL37A | 2,666540916 | 2,320681318 | 0,345859598 | |
| 208926_at | NEU1 | 3,724955181 | 3,379145507 | 0,345809674 | |
| 1555140_a_at | BCL2L2 | -0,986356909 | -1,332017329 | 0,34566042 | |
| 1558195_at | LINC00592 | -0,986356909 | -1,332017329 | 0,34566042 | |
| 206393_at | TNNI2 | -0,986356909 | -1,332017329 | 0,34566042 | |
| 208572_at | HIST3H3 | -0,986356909 | -1,332017329 | 0,34566042 | |
| 211448_s_at | RGS6 | -0,986356909 | -1,332017329 | 0,34566042 | |
| 224197_s_at | C1QTNF1 | -0,986356909 | -1,332017329 | 0,34566042 | |
| 225436_at | FAM108C1 | -0,986356909 | -1,332017329 | 0,34566042 | |
| 231694_at | - | -0,986356909 | -1,332017329 | 0,34566042 | |
| 243677_at | GORASP1 | -0,986356909 | -1,332017329 | 0,34566042 | |
| 1554964_x_at | - | -2,063149277 | -2,408705138 | 0,34555586 | |
| 1561418_at | - | -2,063149277 | -2,408705138 | 0,34555586 | |
| 1570488_at | - | -2,063149277 | -2,408705138 | 0,34555586 | |
| 204597_x_at | STC1 | -2,063149277 | -2,408705138 | 0,34555586 | |
| 205931_s_at | CREB5 /// LOC4( | -2,063149277 | -2,408705138 | 0,34555586 | |
| 210327_s_at | AGXT | -2,063149277 | -2,408705138 | 0,34555586 | |
| 210741_at | ARHGEF12 | -2,063149277 | -2,408705138 | 0,34555586 | |
| 215988_s_at | DLG1 | -2,063149277 | -2,408705138 | 0,34555586 | |
| 216782_at | - | -2,063149277 | -2,408705138 | 0,34555586 | |
| 216829_at | IGK@ /// IGKC /// | -2,063149277 | -2,408705138 | 0,34555586 | |
| 217695_x_at | - | -2,063149277 | -2,408705138 | 0,34555586 | |
| 220065_at | TNMD | -2,063149277 | -2,408705138 | 0,34555586 | |
| 223573_s_at | PPP2R2C | -2,063149277 | -2,408705138 | 0,34555586 | |
| 227519_at | PLAC4 | -2,063149277 | -2,408705138 | 0,34555586 | |
| 230042_at | ITSN1 | -2,063149277 | -2,408705138 | 0,34555586 | |
| 232853_at | - | -2,063149277 | -2,408705138 | 0,34555586 | |

| | | | | | |
|---|---|---|---|---|---|
| 234029_at | PCDHGB8P | -2,063149277 | -2,408705138 | 0,34555586 | |
| 234717_at | - | -2,063149277 | -2,408705138 | 0,34555586 | |
| 238291_at | UFL1 | -2,063149277 | -2,408705138 | 0,34555586 | |
| 238344_at | - | -2,063149277 | -2,408705138 | 0,34555586 | |
| 242412_at | - | -2,063149277 | -2,408705138 | 0,34555586 | |
| 243119_at | - | -2,063149277 | -2,408705138 | 0,34555586 | |
| 243832_at | - | -2,063149277 | -2,408705138 | 0,34555586 | |
| 1562033_at | - | -1,196375121 | -1,541908042 | 0,34553292 | |
| 207221_at | F2RL3 | -1,196375121 | -1,541908042 | 0,34553292 | |
| 209844_at | HOXB13 | -1,196375121 | -1,541908042 | 0,34553292 | |
| 209975_at | CYP2E1 | -1,196375121 | -1,541908042 | 0,34553292 | |
| 210724_at | EMR3 | -1,196375121 | -1,541908042 | 0,34553292 | |
| 211916_s_at | MYO1A | -1,196375121 | -1,541908042 | 0,34553292 | |
| 220522_at | CRB1 | -1,196375121 | -1,541908042 | 0,34553292 | |
| 221074_at | - | -1,196375121 | -1,541908042 | 0,34553292 | |
| 225688_s_at | PHLDB2 | -1,196375121 | -1,541908042 | 0,34553292 | |
| 230343_at | - | -1,196375121 | -1,541908042 | 0,34553292 | |
| 232626_at | - | -1,196375121 | -1,541908042 | 0,34553292 | |
| 235453_at | TOR1AIP2 | -1,196375121 | -1,541908042 | 0,34553292 | |
| 239131_at | - | -1,196375121 | -1,541908042 | 0,34553292 | |
| 241208_at | PDLIM5 | -1,196375121 | -1,541908042 | 0,34553292 | |
| 204194_at | BACH1 | 2,718233611 | 2,372786857 | 0,345446754 | |
| 216862_s_at | MTCP1NB | 1,987559146 | 1,642321262 | 0,345237884 | |
| 207376_at | VENTX | -0,221656139 | -0,566887459 | 0,345231321 | |
| 210610_at | CEACAM1 | -0,221656139 | -0,566887459 | 0,345231321 | |
| 216377_x_at | ALPPL2 | -0,221656139 | -0,566887459 | 0,345231321 | |
| 218173_s_at | WHSC1L1 | -0,221656139 | -0,566887459 | 0,345231321 | |
| 222965_at | PRO2214 | -0,221656139 | -0,566887459 | 0,345231321 | |
| 235288_at | - | -0,221656139 | -0,566887459 | 0,345231321 | |
| 241152_at | - | -0,221656139 | -0,566887459 | 0,345231321 | |
| 244216_at | - | -0,221656139 | -0,566887459 | 0,345231321 | |
| 228769_at | ZSCAN22 | 1,238353345 | 0,893473268 | 0,344880077 | |
| 202050_s_at | ZMYM4 | 2,883642771 | 2,538891651 | 0,34475112 | |
| 217803_at | GOLPH3 | 4,898420203 | 4,553720057 | 0,344700146 | |
| 1558166_at | MGC16275 | -1,518072576 | -1,862647763 | 0,344575187 | |
| 1560208_at | - | -1,518072576 | -1,862647763 | 0,344575187 | |
| 1564157_at | FLJ33544 | -1,518072576 | -1,862647763 | 0,344575187 | |
| 206603_at | SLC2A4 | -1,518072576 | -1,862647763 | 0,344575187 | |
| 210957_s_at | AFF2 | -1,518072576 | -1,862647763 | 0,344575187 | |
| 214562_at | HIST1H4A /// HIS | -1,518072576 | -1,862647763 | 0,344575187 | |
| 215298_at | - | -1,518072576 | -1,862647763 | 0,344575187 | |
| 233583_at | - | -1,518072576 | -1,862647763 | 0,344575187 | |
| 241766_at | LOC100509303 | -1,518072576 | -1,862647763 | 0,344575187 | |
| 243351_at | FLJ31485 | -1,518072576 | -1,862647763 | 0,344575187 | |
| 227040_at | NHLRC3 | 2,402750389 | 2,058399602 | 0,344350787 | |
| 214126_at | - | 1,567778694 | 1,223626587 | 0,344152106 | |
| 233186_s_at | BANP | 2,194816625 | 1,85068474 | 0,344131885 | |
| 204994_at | MX2 | 0,351548291 | 0,007421914 | 0,344126377 | |
| 209968_s_at | NCAM1 | 0,351548291 | 0,007421914 | 0,344126377 | |
| 228640_at | PCDH7 | 0,351548291 | 0,007421914 | 0,344126377 | |
| 226696_at | RBBP9 | 2,134723057 | 1,790660532 | 0,344062525 | |
| 204568_at | ATG14 | 2,716594694 | 2,372786857 | 0,343807837 | |
| 210461_s_at | ABLIM1 | 0,123193849 | -0,220592223 | 0,343786073 | |
| 216535_at | CADM3 | 0,123193849 | -0,220592223 | 0,343786073 | |
| 231816_s_at | UBE2Q1 | 0,123193849 | -0,220592223 | 0,343786073 | |
| 200639_s_at | YWHAZ | 5,963472883 | 5,619727846 | 0,343745037 | |

| | | | | | |
|---|---|---|---|---|---|
| 212097_at | CAV1 | 5,535001352 | 5,191315431 | 0,343685922 | |
| 229419_at | FBXW7 | 1,774096855 | 1,43051703 | 0,343579825 | |
| 210991_s_at | RIMS3 | 0,020670649 | -0,322870281 | 0,343540931 | |
| 227126_at | PTPRG | 0,020670649 | -0,322870281 | 0,343540931 | |
| 228561_at | CDC37L1 | 0,020670649 | -0,322870281 | 0,343540931 | |
| 233266_at | - | 0,020670649 | -0,322870281 | 0,343540931 | |
| 236131_at | - | 0,020670649 | -0,322870281 | 0,343540931 | |
| 238454_at | ZNF540 | 0,020670649 | -0,322870281 | 0,343540931 | |
| 1553139_s_at | PLXNA3 | -0,512251684 | -0,855751026 | 0,343499342 | |
| 1554219_at | - | -0,512251684 | -0,855751026 | 0,343499342 | |
| 1555196_at | LINC00421 | -0,512251684 | -0,855751026 | 0,343499342 | |
| 1560866_at | WNK2 | -0,512251684 | -0,855751026 | 0,343499342 | |
| 210323_at | TEKT2 | -0,512251684 | -0,855751026 | 0,343499342 | |
| 211658_at | PRDX2 | -0,512251684 | -0,855751026 | 0,343499342 | |
| 211791_s_at | KCNAB2 | -0,512251684 | -0,855751026 | 0,343499342 | |
| 214386_at | LOC100653164 | -0,512251684 | -0,855751026 | 0,343499342 | |
| 226359_at | GTPBP1 | -0,512251684 | -0,855751026 | 0,343499342 | |
| 226658_at | PDPN | -0,512251684 | -0,855751026 | 0,343499342 | |
| 229132_at | MINA | -0,512251684 | -0,855751026 | 0,343499342 | |
| 203485_at | RTN1 | 2,994644467 | 2,651169275 | 0,343475192 | |
| 1554769_at | ZNF785 | -0,706943342 | -1,050406615 | 0,343463273 | |
| 1568590_at | ARL3 | -0,706943342 | -1,050406615 | 0,343463273 | |
| 206322_at | SYN3 | -0,706943342 | -1,050406615 | 0,343463273 | |
| 211837_s_at | PTCRA | -0,706943342 | -1,050406615 | 0,343463273 | |
| 215776_at | INSRR | -0,706943342 | -1,050406615 | 0,343463273 | |
| 231868_at | HOMEZ | -0,706943342 | -1,050406615 | 0,343463273 | |
| 234654_at | - | -0,706943342 | -1,050406615 | 0,343463273 | |
| 237319_at | C2orf53 | -0,706943342 | -1,050406615 | 0,343463273 | |
| 239912_at | - | -0,706943342 | -1,050406615 | 0,343463273 | |
| 240339_at | - | -0,706943342 | -1,050406615 | 0,343463273 | |
| 243145_at | - | -0,706943342 | -1,050406615 | 0,343463273 | |
| 243223_at | - | -0,706943342 | -1,050406615 | 0,343463273 | |
| 225971_at | DDHD1 | 2,710020346 | 2,366632651 | 0,343387695 | |
| 224311_s_at | CAB39 | 2,355089531 | 2,011838132 | 0,343251399 | |
| 216028_at | - | 1,534677255 | 1,191491281 | 0,343185974 | |
| 1565633_at | - | -2,930741289 | -3,273861381 | 0,343120092 | |
| 207166_at | GNGT1 | -2,930741289 | -3,273861381 | 0,343120092 | |
| 236035_at | - | -2,930741289 | -3,273861381 | 0,343120092 | |
| 216421_at | - | 1,496984567 | 1,153866761 | 0,343117806 | |
| 231979_at | - | 0,822971223 | 0,479907041 | 0,343064182 | |
| 212904_at | LRRC47 | 5,37849084 | 5,035495453 | 0,342995387 | |
| 202972_s_at | FAM13A | 0,760715727 | 0,417770464 | 0,342945263 | |
| 229544_at | - | 1,823660386 | 1,480766833 | 0,342893553 | |
| 1559220_at | - | 0,695652136 | 0,352836757 | 0,342815379 | |
| 212326_at | VPS13D | 0,695652136 | 0,352836757 | 0,342815379 | |
| 220775_s_at | UEVLD | 0,695652136 | 0,352836757 | 0,342815379 | |
| 221857_s_at | TJAP1 | 0,695652136 | 0,352836757 | 0,342815379 | |
| 229338_at | LOC100289361 | 0,695652136 | 0,352836757 | 0,342815379 | |
| 1553166_at | CDH24 | -0,148196756 | -0,490927335 | 0,342730579 | |
| 205451_at | FOXO4 | -0,148196756 | -0,490927335 | 0,342730579 | |
| 242812_at | HCG18 | -0,148196756 | -0,490927335 | 0,342730579 | |
| 1554518_at | GSTCD | 1,247462054 | 0,904843258 | 0,342618795 | |
| 220306_at | FAM46C | 1,247462054 | 0,904843258 | 0,342618795 | |
| 202980_s_at | SIAH1 | 1,177702228 | 0,835239052 | 0,342463175 | |
| 1553842_at | BEND2 | -2,504600601 | -2,847001814 | 0,342401213 | |
| 1554403_a_at | - | -2,504600601 | -2,847001814 | 0,342401213 | |

| | | | | | |
|---|---|---|---|---|---|
| 1558920_at | SLC8A1-AS1 | -2,504600601 | -2,847001814 | 0,342401213 | |
| 1558934_a_at | GTF2H5 | -2,504600601 | -2,847001814 | 0,342401213 | |
| 1564307_a_at | A2ML1 | -2,504600601 | -2,847001814 | 0,342401213 | |
| 1564372_s_at | CASC2 | -2,504600601 | -2,847001814 | 0,342401213 | |
| 1567703_at | - | -2,504600601 | -2,847001814 | 0,342401213 | |
| 1569208_a_at | - | -2,504600601 | -2,847001814 | 0,342401213 | |
| 1569892_at | - | -2,504600601 | -2,847001814 | 0,342401213 | |
| 213425_at | WNT5A | -2,504600601 | -2,847001814 | 0,342401213 | |
| 217452_s_at | B3GALT2 | -2,504600601 | -2,847001814 | 0,342401213 | |
| 219850_s_at | EHF | -2,504600601 | -2,847001814 | 0,342401213 | |
| 221288_at | GPR22 | -2,504600601 | -2,847001814 | 0,342401213 | |
| 233154_at | - | -2,504600601 | -2,847001814 | 0,342401213 | |
| 234138_at | - | -2,504600601 | -2,847001814 | 0,342401213 | |
| 240712_s_at | - | -2,504600601 | -2,847001814 | 0,342401213 | |
| 243665_s_at | - | -2,504600601 | -2,847001814 | 0,342401213 | |
| 1557277_a_at | LOC100507584 | -2,756478458 | -3,098858316 | 0,342379858 | |
| 1569755_at | LINC00276 | -2,756478458 | -3,098858316 | 0,342379858 | |
| 217705_at | PRKD1 | -2,756478458 | -3,098858316 | 0,342379858 | |
| 224277_at | MOP-1 | -2,756478458 | -3,098858316 | 0,342379858 | |
| 230692_at | LOC157503 | -2,756478458 | -3,098858316 | 0,342379858 | |
| 234544_at | ARHGEF12 | -2,756478458 | -3,098858316 | 0,342379858 | |
| 235380_at | - | -2,756478458 | -3,098858316 | 0,342379858 | |
| 235401_s_at | FCRLA | -2,756478458 | -3,098858316 | 0,342379858 | |
| 1552914_a_at | CD276 | -0,423248115 | -0,765504029 | 0,342255914 | |
| 1557347_at | MCPH1 | -0,423248115 | -0,765504029 | 0,342255914 | |
| 1564339_a_at | CHRM3 | -0,423248115 | -0,765504029 | 0,342255914 | |
| 230310_at | - | -0,423248115 | -0,765504029 | 0,342255914 | |
| 236460_at | - | -0,423248115 | -0,765504029 | 0,342255914 | |
| 242393_x_at | AGAP9 /// BMS1I | -0,423248115 | -0,765504029 | 0,342255914 | |
| 1554839_at | CIDECP | 0,401366113 | 0,059218869 | 0,342147244 | |
| 217702_at | IL27RA | 0,317354302 | -0,024572586 | 0,341926888 | |
| 204716_at | CCDC6 | 1,732533761 | 1,390636111 | 0,34189765 | |
| 235598_at | SMAD2 | 0,945567441 | 0,603679647 | 0,341887795 | |
| 215716_s_at | ATP2B1 | 2,41290634 | 2,071071048 | 0,341835292 | |
| 211764_s_at | UBE2D1 | 2,683419202 | 2,341749893 | 0,341669309 | |
| 214356_s_at | KIAA0368 | 2,683419202 | 2,341749893 | 0,341669309 | |
| 215772_x_at | SUCLG2 | 2,64946283 | 2,307890888 | 0,341571942 | |
| 219353_at | NHLRC2 | 1,274448124 | 0,932882274 | 0,34156585 | |
| 239721_at | - | 0,181384709 | -0,160136748 | 0,341521458 | |
| 208328_s_at | MEF2A | 1,859746653 | 1,518263577 | 0,341483076 | |
| 201097_s_at | ARF4 | 6,563911807 | 6,222493151 | 0,341418656 | |
| 204461_x_at | RAD1 | 3,336243945 | 2,994948944 | 0,341295001 | |
| 232137_at | ZNF616 | 0,735041319 | 0,393761504 | 0,341279815 | |
| 234140_s_at | STIM2 | 1,485482016 | 1,144305291 | 0,341176725 | |
| 1556247_a_at | LOC100506271 | 0,083054335 | -0,258114234 | 0,341168569 | |
| 204562_at | IRF4 | 0,083054335 | -0,258114234 | 0,341168569 | |
| 207566_at | MR1 | 0,083054335 | -0,258114234 | 0,341168569 | |
| 221140_s_at | GPR132 | 0,083054335 | -0,258114234 | 0,341168569 | |
| 228831_s_at | GNG7 | 0,083054335 | -0,258114234 | 0,341168569 | |
| 235447_at | TRUB1 | 0,083054335 | -0,258114234 | 0,341168569 | |
| 225891_at | TPRN | 1,406361373 | 1,065442793 | 0,34091858 | |
| 220947_s_at | TBC1D10B | 2,644299732 | 2,303602088 | 0,340697644 | |
| 225019_at | CAMK2D | 3,046446467 | 2,705818724 | 0,340627743 | |
| 216545_at | - | -0,078265071 | -0,41858459 | 0,340319518 | |
| 220645_at | NXPE4 | -0,078265071 | -0,41858459 | 0,340319518 | |
| 241448_at | - | -0,078265071 | -0,41858459 | 0,340319518 | |

| | | | | | |
|---|---|---|---|---|---|
| 242207_at | CENPP /// LOC1( | -0,078265071 | -0,41858459 | 0,340319518 | |
| 1555311_at | - | 1,233777334 | 0,893473268 | 0,340304066 | |
| 202757_at | COBRA1 | 3,340506684 | 3,000258961 | 0,340247724 | |
| 209627_s_at | OSBPL3 | 1,187200746 | 0,847075464 | 0,340125283 | |
| 218685_s_at | SMUG1 | 1,187200746 | 0,847075464 | 0,340125283 | |
| 226109_at | C21orf91 | 3,804253954 | 3,464222256 | 0,340031698 | |
| 230055_at | KHDC1 | 1,655931423 | 1,315948728 | 0,339982695 | |
| 224953_at | YIPF5 | 3,107750584 | 2,767843855 | 0,339906729 | |
| 1555916_at | RPUSD3 | 1,820612054 | 1,480766833 | 0,339845221 | |
| 225088_at | FOPNL | 2,947914474 | 2,608344689 | 0,339569785 | |
| 225023_at | GOPC | 2,675004741 | 2,335461563 | 0,339543178 | |
| 217211_at | - | 3,219589925 | 2,880111483 | 0,339478442 | |
| 225281_at | C3orf17 | 4,225494449 | 3,886022844 | 0,339471605 | |
| 201282_at | OGDH | 1,709647566 | 1,370274469 | 0,339373096 | |
| 1554258_a_at | DNAJC5B | -0,196756757 | -0,536087151 | 0,339330394 | |
| 1569194_at | ZNF789 | -0,196756757 | -0,536087151 | 0,339330394 | |
| 207004_at | BCL2 | -0,196756757 | -0,536087151 | 0,339330394 | |
| 240434_at | - | 1,493160564 | 1,153866761 | 0,339293803 | |
| 212006_at | UBXN4 | 3,526916747 | 3,187631636 | 0,339285111 | |
| 242131_at | ATP6 | 2,701760055 | 2,362515214 | 0,339244842 | |
| 203335_at | PHYH | 3,517535002 | 3,178292062 | 0,33924294 | |
| 214639_s_at | HOXA1 | 1,67631007 | 1,337086025 | 0,339224046 | |
| 1569091_at | - | 0,606430281 | 0,26733076 | 0,339099522 | |
| 224613_s_at | DNAJC5 | 0,606430281 | 0,26733076 | 0,339099522 | |
| 221808_at | RAB9A | 4,551591289 | 4,212565608 | 0,339025681 | |
| 201572_x_at | DCTD | 3,792633459 | 3,453616097 | 0,339017362 | |
| 1555839_a_at | C3orf79 | -2,551294263 | -2,89013181 | 0,338837547 | |
| 1557434_at | LOC100506497 | -2,551294263 | -2,89013181 | 0,338837547 | |
| 1557864_x_at | - | -2,551294263 | -2,89013181 | 0,338837547 | |
| 1558944_at | LOC100507353 | -2,551294263 | -2,89013181 | 0,338837547 | |
| 1559645_at | LINC00184 | -2,551294263 | -2,89013181 | 0,338837547 | |
| 1563670_at | ZSCAN23 | -2,551294263 | -2,89013181 | 0,338837547 | |
| 1569706_at | MYSM1 | -2,551294263 | -2,89013181 | 0,338837547 | |
| 210170_at | PDLIM3 | -2,551294263 | -2,89013181 | 0,338837547 | |
| 213353_at | ABCA5 | -2,551294263 | -2,89013181 | 0,338837547 | |
| 220026_at | CLCA4 | -2,551294263 | -2,89013181 | 0,338837547 | |
| 228400_at | SHROOM3 | -2,551294263 | -2,89013181 | 0,338837547 | |
| 231186_at | FLJ43390 | -2,551294263 | -2,89013181 | 0,338837547 | |
| 232750_at | - | -2,551294263 | -2,89013181 | 0,338837547 | |
| 232885_at | BBIP1 | -2,551294263 | -2,89013181 | 0,338837547 | |
| 233351_at | - | -2,551294263 | -2,89013181 | 0,338837547 | |
| 235004_at | RBM24 | -2,551294263 | -2,89013181 | 0,338837547 | |
| 241113_at | - | -2,551294263 | -2,89013181 | 0,338837547 | |
| 241247_at | - | -2,551294263 | -2,89013181 | 0,338837547 | |
| 237032_x_at | SIPA1L1 | 0,87091093 | 0,532164371 | 0,338746559 | |
| 217761_at | ADI1 | 4,510092468 | 4,171380634 | 0,338711834 | |
| 1569403_at | - | 1,352503698 | 1,013870809 | 0,338632889 | |
| 242908_x_at | - | -2,972500237 | -3,311072652 | 0,338572415 | |
| 1569839_s_at | LRRC37A5P | -1,399512875 | -1,738067356 | 0,338554481 | |
| 204239_s_at | NNAT | -1,399512875 | -1,738067356 | 0,338554481 | |
| 206062_at | GUCA1A | -1,399512875 | -1,738067356 | 0,338554481 | |
| 207991_x_at | ACRV1 | -1,399512875 | -1,738067356 | 0,338554481 | |
| 211011_at | COL19A1 | -1,399512875 | -1,738067356 | 0,338554481 | |
| 221383_at | NMUR1 | -1,399512875 | -1,738067356 | 0,338554481 | |
| 228313_at | GPRC5B | -1,399512875 | -1,738067356 | 0,338554481 | |
| 229652_s_at | FOXN3 | -1,399512875 | -1,738067356 | 0,338554481 | |

| | | | | | |
|---|---|---|---|---|---|
| 230768_at | - | -1,399512875 | -1,738067356 | 0,338554481 | |
| 231067_s_at | - | -1,399512875 | -1,738067356 | 0,338554481 | |
| 237845_at | - | -1,399512875 | -1,738067356 | 0,338554481 | |
| 239374_at | TRIM65 | -1,399512875 | -1,738067356 | 0,338554481 | |
| 240861_at | - | -1,399512875 | -1,738067356 | 0,338554481 | |
| 243199_at | LOC100505574 | -1,399512875 | -1,738067356 | 0,338554481 | |
| 1555878_at | RPS24 | 3,176127502 | 2,837730973 | 0,338396529 | |
| 1556250_at | - | -2,236765673 | -2,575129729 | 0,338364056 | |
| 1557012_a_at | - | -2,236765673 | -2,575129729 | 0,338364056 | |
| 1561983_at | - | -2,236765673 | -2,575129729 | 0,338364056 | |
| 1566863_at | - | -2,236765673 | -2,575129729 | 0,338364056 | |
| 201617_x_at | CALD1 | -2,236765673 | -2,575129729 | 0,338364056 | |
| 207796_x_at | KLRD1 | -2,236765673 | -2,575129729 | 0,338364056 | |
| 215219_at | DOPEY2 | -2,236765673 | -2,575129729 | 0,338364056 | |
| 216147_at | - | -2,236765673 | -2,575129729 | 0,338364056 | |
| 216367_at | COL4A3 | -2,236765673 | -2,575129729 | 0,338364056 | |
| 220338_at | RALGPS2 | -2,236765673 | -2,575129729 | 0,338364056 | |
| 221386_at | OR3A2 | -2,236765673 | -2,575129729 | 0,338364056 | |
| 221420_at | - | -2,236765673 | -2,575129729 | 0,338364056 | |
| 224537_at | PCDHGC5 | -2,236765673 | -2,575129729 | 0,338364056 | |
| 225846_at | ESRP1 | -2,236765673 | -2,575129729 | 0,338364056 | |
| 227648_at | C22orf32 | -2,236765673 | -2,575129729 | 0,338364056 | |
| 230258_at | GLIS3 | -2,236765673 | -2,575129729 | 0,338364056 | |
| 235642_at | - | -2,236765673 | -2,575129729 | 0,338364056 | |
| 236341_at | CTLA4 | -2,236765673 | -2,575129729 | 0,338364056 | |
| 237471_at | LOC154822 | -2,236765673 | -2,575129729 | 0,338364056 | |
| 242548_x_at | ANKRD37 | -2,236765673 | -2,575129729 | 0,338364056 | |
| 218962_s_at | TMEM168 | 2,516380342 | 2,17802703 | 0,338353312 | |
| 226030_at | ACADSB | 2,396622324 | 2,058399602 | 0,338222722 | |
| 1554430_at | FAM165B | 0,417596971 | 0,079428135 | 0,338168835 | |
| 222859_s_at | DAPP1 | 0,417596971 | 0,079428135 | 0,338168835 | |
| 235301_at | KIAA1324L | 1,780386218 | 1,442269454 | 0,338116764 | |
| 44111_at | VPS33B | 2,36034699 | 2,022315766 | 0,338031224 | |
| 228485_s_at | SLC44A1 | 0,715481152 | 0,377530647 | 0,337950505 | |
| 1566403_at | SNORA68 | -0,57445847 | -0,912331589 | 0,337873119 | |
| 209639_s_at | RGS12 | -0,57445847 | -0,912331589 | 0,337873119 | |
| 215369_at | - | -0,57445847 | -0,912331589 | 0,337873119 | |
| 231906_at | HOXD8 | -0,57445847 | -0,912331589 | 0,337873119 | |
| 237167_at | KIAA1217 | -0,57445847 | -0,912331589 | 0,337873119 | |
| 239293_at | NRSN1 | -0,57445847 | -0,912331589 | 0,337873119 | |
| 239337_at | LOC400768 | -0,57445847 | -0,912331589 | 0,337873119 | |
| 202205_at | VASP | 3,584658878 | 3,246908183 | 0,337750695 | |
| 226327_at | ZNF507 | 3,367911651 | 3,030417996 | 0,337493655 | |
| 210680_s_at | MASP1 | 0,291166226 | -0,046302147 | 0,337468373 | |
| 225869_s_at | UNC93B1 | 0,291166226 | -0,046302147 | 0,337468373 | |
| 229195_at | MESP1 | 0,291166226 | -0,046302147 | 0,337468373 | |
| 209954_x_at | SS18 | 1,799091453 | 1,461646549 | 0,337444904 | |
| 213340_s_at | TP73-AS1 | 2,420979937 | 2,083632167 | 0,33734777 | |
| 1566599_at | - | -0,67289328 | -1,010192375 | 0,337299095 | |
| 204540_at | EEF1A2 | -0,67289328 | -1,010192375 | 0,337299095 | |
| 206753_at | RDH16 | -0,67289328 | -1,010192375 | 0,337299095 | |
| 210684_s_at | DLG4 | -0,67289328 | -1,010192375 | 0,337299095 | |
| 217162_at | - | -0,67289328 | -1,010192375 | 0,337299095 | |
| 229951_x_at | - | -0,67289328 | -1,010192375 | 0,337299095 | |
| 234342_at | FAM20C | -0,67289328 | -1,010192375 | 0,337299095 | |
| 236672_at | ZNF681 | -0,67289328 | -1,010192375 | 0,337299095 | |

| | | | | | |
|---|---|---|---|---|---|
| 238377_s_at | - | -0,67289328 | -1,010192375 | 0,337299095 | |
| 243076_x_at | GLI4 | -0,67289328 | -1,010192375 | 0,337299095 | |
| 244844_at | - | -0,67289328 | -1,010192375 | 0,337299095 | |
| 217876_at | GTF3C5 | 1,631786706 | 1,294497133 | 0,337289573 | |
| 213342_at | YAP1 | -0,926850548 | -1,264005341 | 0,337154792 | |
| 217569_x_at | - | -0,926850548 | -1,264005341 | 0,337154792 | |
| 231346_s_at | - | -0,926850548 | -1,264005341 | 0,337154792 | |
| 232788_at | - | -0,926850548 | -1,264005341 | 0,337154792 | |
| 234770_at | - | -0,926850548 | -1,264005341 | 0,337154792 | |
| 239796_x_at | TIRAP | -0,926850548 | -1,264005341 | 0,337154792 | |
| 244702_at | - | -0,926850548 | -1,264005341 | 0,337154792 | |
| 209062_x_at | NCOA3 | 1,269985355 | 0,932882274 | 0,337103081 | |
| 223555_at | GON4L | 1,269985355 | 0,932882274 | 0,337103081 | |
| 1568815_a_at | DDX50 | 1,442503549 | 1,105412836 | 0,337090713 | |
| 244676_s_at | MTUS2 | 0,541261317 | 0,204310755 | 0,336950561 | |
| 226921_at | UBR1 | 4,279221286 | 3,942281595 | 0,33693969 | |
| 221904_at | FAM131A | 0,20027154 | -0,136643983 | 0,336915522 | |
| 226919_at | LYRM2 | 2,31665944 | 1,979940779 | 0,336718661 | |
| 209370_s_at | SH3BP2 | 1,201332287 | 0,864649967 | 0,33668232 | |
| 209349_at | RAD50 | 0,995078803 | 0,658444279 | 0,336634524 | |
| 213518_at | PRKCI | 4,28917944 | 3,952590169 | 0,336589271 | |
| 224760_at | SP1 | 3,464815649 | 3,128242026 | 0,336573623 | |
| 225128_at | KDELC2 | 1,995690482 | 1,659204017 | 0,336486466 | |
| 213306_at | MPDZ | 2,361396186 | 2,024923328 | 0,336472859 | |
| 204630_s_at | GOSR1 | 3,552865637 | 3,216434104 | 0,336431533 | |
| 241342_at | TMEM65 | 1,46610492 | 1,129843209 | 0,336261711 | |
| 1556507_at | LOC100507274 | 2,038300149 | 1,702196344 | 0,336103805 | |
| 1569074_at | SRRM3 | -0,394749123 | -0,730778808 | 0,336029685 | |
| 210031_at | CD247 | -0,394749123 | -0,730778808 | 0,336029685 | |
| 217413_s_at | TNXB | -0,394749123 | -0,730778808 | 0,336029685 | |
| 223459_s_at | C1orf56 | -0,394749123 | -0,730778808 | 0,336029685 | |
| 229006_at | LOC100507487 | -0,394749123 | -0,730778808 | 0,336029685 | |
| 239785_at | DZIP1L | -0,394749123 | -0,730778808 | 0,336029685 | |
| 242373_at | - | -0,394749123 | -0,730778808 | 0,336029685 | |
| 205233_s_at | PAFAH2 | 0,052199481 | -0,283667828 | 0,335867308 | |
| 205383_s_at | ZBTB20 | 0,052199481 | -0,283667828 | 0,335867308 | |
| 229771_at | MAP3K13 | 0,052199481 | -0,283667828 | 0,335867308 | |
| 234647_at | KCNIP3 | 0,052199481 | -0,283667828 | 0,335867308 | |
| 207350_s_at | VAMP4 | 0,853119696 | 0,51742585 | 0,335693846 | |
| 222164_at | FGFR1 | 0,853119696 | 0,51742585 | 0,335693846 | |
| 212460_at | SPTSSA | 5,543346316 | 5,207761432 | 0,335584884 | |
| 207564_x_at | OGT | 2,355089531 | 2,019703483 | 0,335386048 | |
| 230413_s_at | - | 5,215731134 | 4,880355851 | 0,335375283 | |
| 200749_at | RAN | 4,76563534 | 4,430312399 | 0,335322941 | |
| 210296_s_at | PEX2 | 3,277429131 | 2,942125533 | 0,335303597 | |
| 219012_s_at | C11orf30 | 0,792179257 | 0,456918297 | 0,335260959 | |
| 1552845_at | CLDN15 | 0,97328325 | 0,638150376 | 0,335132874 | |
| 205716_at | SLC25A40 | 0,97328325 | 0,638150376 | 0,335132874 | |
| 1555393_s_at | C21orf67 | 0,255492549 | -0,079522948 | 0,335015497 | |
| 232727_at | UCKL1 | 0,255492549 | -0,079522948 | 0,335015497 | |
| 235896_s_at | SMCR7 | 0,255492549 | -0,079522948 | 0,335015497 | |
| 225679_at | NAA30 | 3,689469311 | 3,354479478 | 0,334989833 | |
| 205668_at | LY75 | 2,146943689 | 1,811954245 | 0,334989445 | |
| 230078_at | RAPGEF6 | 4,056345228 | 3,721356767 | 0,334988461 | |
| 205585_at | ETV6 | -0,055687482 | -0,39061235 | 0,334924868 | |
| 242218_at | LOC100507672 | -0,055687482 | -0,39061235 | 0,334924868 | |

| | | | | | |
|---|---|---|---|---|---|
| 205632_s_at | PIP5K1B | 2,482063962 | 2,147243253 | 0,334820709 | |
| 231164_at | ABCA17P | 3,456984333 | 3,122166354 | 0,334817978 | |
| 213212_x_at | GOLGA6L3 /// G( | 1,607231019 | 1,272721751 | 0,334509268 | |
| 213282_at | APOOL | 2,827046023 | 2,492544876 | 0,334501148 | |
| 223436_s_at | TRPT1 | 1,348276312 | 1,013870809 | 0,334405503 | |
| 1558885_at | - | -1,089016378 | -1,423348341 | 0,334331964 | |
| 1570285_at | - | -1,089016378 | -1,423348341 | 0,334331964 | |
| 206058_at | SLC6A12 | -1,089016378 | -1,423348341 | 0,334331964 | |
| 207303_at | PDE1C | -1,089016378 | -1,423348341 | 0,334331964 | |
| 217676_at | - | -1,089016378 | -1,423348341 | 0,334331964 | |
| 217687_at | ADCY2 | -1,089016378 | -1,423348341 | 0,334331964 | |
| 220334_at | RGS17 | -1,089016378 | -1,423348341 | 0,334331964 | |
| 222963_s_at | IL1RAPL1 | -1,089016378 | -1,423348341 | 0,334331964 | |
| 226618_at | UBE2QL1 | -1,089016378 | -1,423348341 | 0,334331964 | |
| 227062_at | LOC100653017 / | -1,089016378 | -1,423348341 | 0,334331964 | |
| 232011_s_at | MAP1LC3A | -1,089016378 | -1,423348341 | 0,334331964 | |
| 218393_s_at | SMU1 | 2,680059304 | 2,345926937 | 0,334132368 | |
| 205596_s_at | SMURF2 | 1,764611101 | 1,43051703 | 0,33409407 | |
| 213180_s_at | GOSR2 | 1,764611101 | 1,43051703 | 0,33409407 | |
| 213562_s_at | SQLE | 4,279776324 | 3,945725977 | 0,334050346 | |
| 1554071_at | CCDC67 | -3,013665402 | -3,347710726 | 0,334045324 | |
| 1563503_at | - | -3,013665402 | -3,347710726 | 0,334045324 | |
| 206552_s_at | TAC1 | -3,013665402 | -3,347710726 | 0,334045324 | |
| 202250_s_at | DCAF8 | 0,82905153 | 0,495031851 | 0,33401968 | |
| 241415_at | - | 0,627515113 | 0,293518836 | 0,333996277 | |
| 218884_s_at | GUF1 | 1,814495998 | 1,480766833 | 0,333729165 | |
| 218689_at | FANCF | 1,593009234 | 1,259496994 | 0,33351224 | |
| 218028_at | ELOVL1 | 2,546213279 | 2,212749286 | 0,333463993 | |
| 223288_at | USP38 | 3,34687727 | 3,013449121 | 0,333428149 | |
| 1554869_at | WDR37 | 0,555999837 | 0,222599095 | 0,333400742 | |
| 212126_at | CBX5 | 3,069741844 | 2,7363693 | 0,333372544 | |
| 202622_s_at | ATXN2 | 2,416948786 | 2,083632167 | 0,333316619 | |
| 218878_s_at | SIRT1 | 2,352981171 | 2,019703483 | 0,333277688 | |
| 218411_s_at | MBIP | 2,901063789 | 2,567790814 | 0,333272975 | |
| 220024_s_at | PRX | 0,30867765 | -0,024572586 | 0,333250236 | |
| 200608_s_at | RAD21 | 5,816676661 | 5,483469529 | 0,333207132 | |
| 218442_at | TTC4 | 3,408067074 | 3,075135559 | 0,332931515 | |
| 221240_s_at | B3GNT4 | 1,168140757 | 0,835239052 | 0,332901705 | |
| 212536_at | ATP11B | 3,333038605 | 3,000258961 | 0,332779644 | |
| 1569114_at | LOC100506847 | -0,234278768 | -0,566887459 | 0,332608692 | |
| 210542_s_at | SLCO3A1 | -0,234278768 | -0,566887459 | 0,332608692 | |
| 228998_at | TNRC6B | -0,234278768 | -0,566887459 | 0,332608692 | |
| 230525_at | LINC00514 /// LC | -0,234278768 | -0,566887459 | 0,332608692 | |
| 236064_at | SLC25A35 | -0,234278768 | -0,566887459 | 0,332608692 | |
| 240044_x_at | TNRC6B | -0,234278768 | -0,566887459 | 0,332608692 | |
| 241802_x_at | GRM2 | -0,234278768 | -0,566887459 | 0,332608692 | |
| 243580_at | GNA14 | -0,234278768 | -0,566887459 | 0,332608692 | |
| 244086_at | - | -0,234278768 | -0,566887459 | 0,332608692 | |
| 244339_at | - | -0,234278768 | -0,566887459 | 0,332608692 | |
| 244398_x_at | ZNF684 | -0,234278768 | -0,566887459 | 0,332608692 | |
| 223428_s_at | ISY1 | 2,206540699 | 1,874012195 | 0,332528505 | |
| 215622_x_at | PHF7 | 0,44160593 | 0,109221392 | 0,332384538 | |
| 228762_at | LFNG | 0,44160593 | 0,109221392 | 0,332384538 | |
| 229165_at | MRPL12 | 0,95671774 | 0,624460759 | 0,332256981 | |
| 218737_at | SBNO1 | 3,034656185 | 2,702564916 | 0,332091269 | |
| 218919_at | ZFAND1 | 3,621908408 | 3,289820089 | 0,33208832 | |

| | | | | | |
|---|---|---|---|---|---|
| 225633_at | DPY19L3 | 5,179605142 | 4,847588869 | 0,332016273 | |
| 239565_at | LOC100128292 | 0,171847695 | -0,160136748 | 0,331984444 | |
| 210819_x_at | DIO2 | 0,900083947 | 0,568365095 | 0,331718852 | |
| 203591_s_at | CSF3R | -0,888496123 | -1,220210588 | 0,331714465 | |
| 216825_s_at | MPL | -0,888496123 | -1,220210588 | 0,331714465 | |
| 227923_at | SHANK3 | -0,888496123 | -1,220210588 | 0,331714465 | |
| 231441_at | C7orf62 | -0,888496123 | -1,220210588 | 0,331714465 | |
| 239760_at | - | -0,888496123 | -1,220210588 | 0,331714465 | |
| 1559343_at | LOC100506948 / | 0,359971644 | 0,028364014 | 0,33160763 | |
| 1565832_at | - | 0,359971644 | 0,028364014 | 0,33160763 | |
| 1567032_s_at | ZNF160 | 0,359971644 | 0,028364014 | 0,33160763 | |
| 217041_at | NPTXR | 0,359971644 | 0,028364014 | 0,33160763 | |
| 1556474_a_at | FLJ38379 | -2,359436556 | -2,690860744 | 0,331424188 | |
| 1556607_at | EHD4 | -2,359436556 | -2,690860744 | 0,331424188 | |
| 1566438_at | - | -2,359436556 | -2,690860744 | 0,331424188 | |
| 207229_at | KLRAP1 | -2,359436556 | -2,690860744 | 0,331424188 | |
| 227764_at | LYPD6 | -2,359436556 | -2,690860744 | 0,331424188 | |
| 233066_at | - | -2,359436556 | -2,690860744 | 0,331424188 | |
| 234139_s_at | - | -2,359436556 | -2,690860744 | 0,331424188 | |
| 236818_at | - | -2,359436556 | -2,690860744 | 0,331424188 | |
| 240422_at | - | -2,359436556 | -2,690860744 | 0,331424188 | |
| 240439_at | - | -2,359436556 | -2,690860744 | 0,331424188 | |
| 242639_at | NARG2 | -2,359436556 | -2,690860744 | 0,331424188 | |
| 243586_at | - | -2,359436556 | -2,690860744 | 0,331424188 | |
| 243650_at | PLEKHH2 | -2,359436556 | -2,690860744 | 0,331424188 | |
| 212280_x_at | ATG4B | 2,960432227 | 2,629059192 | 0,331373035 | |
| 222201_s_at | CASP8AP2 | 3,717599787 | 3,386261038 | 0,331338749 | |
| 238624_at | - | 1,446464021 | 1,115234684 | 0,331229336 | |
| 227022_at | GNPDA2 | 1,224581536 | 0,893473268 | 0,331108268 | |
| 238075_at | CHEK1 | 2,688444421 | 2,357351836 | 0,331092585 | |
| 216042_at | TNFRSF25 | 0,072842263 | -0,258114234 | 0,330956496 | |
| 219095_at | JMJD7 /// JMJD7 | 0,072842263 | -0,258114234 | 0,330956496 | |
| 232342_at | MTMR14 | 0,072842263 | -0,258114234 | 0,330956496 | |
| 243092_at | PAN3-AS1 | 0,072842263 | -0,258114234 | 0,330956496 | |
| 215438_x_at | GSPT1 | 5,683961059 | 5,353099844 | 0,330861216 | |
| 223208_at | KCTD10 | 2,074578432 | 1,743944487 | 0,330633944 | |
| 236487_at | SCLT1 | 1,874520207 | 1,543943227 | 0,330576979 | |
| 1553208_s_at | - | -1,2845686 | -1,615132408 | 0,330563807 | |
| 1557565_a_at | LOC100507300 | -1,2845686 | -1,615132408 | 0,330563807 | |
| 1559083_x_at | LOC284600 | -1,2845686 | -1,615132408 | 0,330563807 | |
| 1563143_at | LOC100507065 | -1,2845686 | -1,615132408 | 0,330563807 | |
| 1569335_a_at | STRA6 | -1,2845686 | -1,615132408 | 0,330563807 | |
| 1569353_at | CCP110 | -1,2845686 | -1,615132408 | 0,330563807 | |
| 207295_at | SCNN1G | -1,2845686 | -1,615132408 | 0,330563807 | |
| 215216_at | VPS16 | -1,2845686 | -1,615132408 | 0,330563807 | |
| 223842_s_at | SCARA3 | -1,2845686 | -1,615132408 | 0,330563807 | |
| 233988_x_at | SCUBE1 | -1,2845686 | -1,615132408 | 0,330563807 | |
| 237011_at | LOC643837 | -1,2845686 | -1,615132408 | 0,330563807 | |
| 240171_at | - | -1,2845686 | -1,615132408 | 0,330563807 | |
| 225816_at | PHF17 | 2,763383302 | 2,432926755 | 0,330456548 | |
| 228005_at | ZXDB | 2,520143371 | 2,189694216 | 0,330449155 | |
| 229422_at | NRD1 | 1,62481334 | 1,294497133 | 0,330316207 | |
| 1569472_s_at | TTC3 /// TTC3P1 | 1,251994932 | 0,921731975 | 0,330262957 | |
| 215949_x_at | IGHM /// LOC100 | 0,228146222 | -0,102100538 | 0,330246759 | |
| 225758_s_at | TUBGCP6 | 0,228146222 | -0,102100538 | 0,330246759 | |
| 220925_at | NAA35 | 3,24026957 | 2,910041809 | 0,330227762 | |

| | | | | | |
|---|---|---|---|---|---|
| 236227_at | TMEM161B | 1,021868205 | 0,691645685 | 0,33022252 | |
| 220800_s_at | TMOD3 | 0,747935635 | 0,417770464 | 0,330165172 | |
| 208646_at | - | 6,239782398 | 5,90996843 | 0,329813968 | |
| 1554038_at | LARP1B | -1,047650486 | -1,377419394 | 0,329768909 | |
| 1554374_at | - | -1,047650486 | -1,377419394 | 0,329768909 | |
| 1561425_a_at | ZNF568 | -1,047650486 | -1,377419394 | 0,329768909 | |
| 202712_s_at | CKMT1A /// CKM | -1,047650486 | -1,377419394 | 0,329768909 | |
| 207789_s_at | DPP6 | -1,047650486 | -1,377419394 | 0,329768909 | |
| 214844_s_at | DOK5 | -1,047650486 | -1,377419394 | 0,329768909 | |
| 214933_at | CACNA1A | -1,047650486 | -1,377419394 | 0,329768909 | |
| 230459_s_at | SHB | -1,047650486 | -1,377419394 | 0,329768909 | |
| 231249_at | SZT2 | -1,047650486 | -1,377419394 | 0,329768909 | |
| 231521_at | PMS2P4 | -1,047650486 | -1,377419394 | 0,329768909 | |
| 232326_at | C8orf56 | -1,047650486 | -1,377419394 | 0,329768909 | |
| 234059_at | - | -1,047650486 | -1,377419394 | 0,329768909 | |
| 235142_at | ZBTB8A | -1,047650486 | -1,377419394 | 0,329768909 | |
| 239416_at | FBXL6 | -1,047650486 | -1,377419394 | 0,329768909 | |
| 202460_s_at | LPIN2 | 1,799091453 | 1,469325098 | 0,329766356 | |
| 231796_at | EPHA8 | 0,533835206 | 0,204310755 | 0,329524451 | |
| 225916_at | LOC100506639 / | 1,90937145 | 1,579853228 | 0,329518222 | |
| 227113_at | ADHFE1 | 0,682279745 | 0,352836757 | 0,329442988 | |
| 236170_x_at | - | 0,682279745 | 0,352836757 | 0,329442988 | |
| 213341_at | FEM1C | 1,954567604 | 1,6252386 | 0,329329004 | |
| 1554865_at | - | -2,667025278 | -2,996335703 | 0,329310425 | |
| 1557838_at | FAM135B | -2,667025278 | -2,996335703 | 0,329310425 | |
| 1560696_x_at | QTRTD1 | -2,667025278 | -2,996335703 | 0,329310425 | |
| 1562453_at | - | -2,667025278 | -2,996335703 | 0,329310425 | |
| 1564077_at | - | -2,667025278 | -2,996335703 | 0,329310425 | |
| 1564547_x_at | - | -2,667025278 | -2,996335703 | 0,329310425 | |
| 1566161_at | - | -2,667025278 | -2,996335703 | 0,329310425 | |
| 1568646_x_at | ZNF208 | -2,667025278 | -2,996335703 | 0,329310425 | |
| 1569664_at | - | -2,667025278 | -2,996335703 | 0,329310425 | |
| 205945_at | IL6R | -2,667025278 | -2,996335703 | 0,329310425 | |
| 207772_s_at | PRMT8 | -2,667025278 | -2,996335703 | 0,329310425 | |
| 211459_at | - | -2,667025278 | -2,996335703 | 0,329310425 | |
| 217105_at | - | -2,667025278 | -2,996335703 | 0,329310425 | |
| 231879_at | COL12A1 | -2,667025278 | -2,996335703 | 0,329310425 | |
| 234957_at | - | -2,667025278 | -2,996335703 | 0,329310425 | |
| 236161_at | - | -2,667025278 | -2,996335703 | 0,329310425 | |
| 243164_s_at | - | -2,667025278 | -2,996335703 | 0,329310425 | |
| 226323_at | ZNF830 | 2,171078719 | 1,841838775 | 0,329239944 | |
| 203918_at | PCDH1 | 0,133056859 | -0,19610998 | 0,329166839 | |
| 217721_at | 39326 | 0,133056859 | -0,19610998 | 0,329166839 | |
| 205550_s_at | BRE | 2,752227654 | 2,423075926 | 0,329151728 | |
| 224697_at | DCAF5 | 1,233777334 | 0,904843258 | 0,328934075 | |
| 225032_at | FNDC3B | 3,013454777 | 2,684536601 | 0,328918176 | |
| 206072_at | UCN | -0,089689931 | -0,41858459 | 0,328894658 | |
| 222301_at | C1orf61 | -0,089689931 | -0,41858459 | 0,328894658 | |
| 232549_at | RBM11 | -0,089689931 | -0,41858459 | 0,328894658 | |
| 244421_at | - | -0,089689931 | -0,41858459 | 0,328894658 | |
| 223078_s_at | TMOD3 | 1,394109935 | 1,065442793 | 0,328667143 | |
| 238012_at | DPP7 | 1,394109935 | 1,065442793 | 0,328667143 | |
| 1552719_at | CASC4 | -0,285837228 | -0,614371577 | 0,328534348 | |
| 1561044_at | - | -0,285837228 | -0,614371577 | 0,328534348 | |
| 211499_s_at | MAPK11 | -0,285837228 | -0,614371577 | 0,328534348 | |
| 212562_s_at | CTSZ | -0,285837228 | -0,614371577 | 0,328534348 | |

| | | | | | |
|---|---|---|---|---|---|
| 216448_at | - | -0,285837228 | -0,614371577 | 0,328534348 | |
| 220599_s_at | CARD14 | -0,285837228 | -0,614371577 | 0,328534348 | |
| 224122_at | - | -0,285837228 | -0,614371577 | 0,328534348 | |
| 227412_at | PPP1R3E | -0,285837228 | -0,614371577 | 0,328534348 | |
| 228072_at | SYT12 | -0,285837228 | -0,614371577 | 0,328534348 | |
| 229207_x_at | RNF187 | -0,285837228 | -0,614371577 | 0,328534348 | |
| 229792_at | KLHL17 | -0,285837228 | -0,614371577 | 0,328534348 | |
| 201916_s_at | SEC63 | 4,19991587 | 3,871608557 | 0,328307313 | |
| 227214_at | GOPC | 1,85379461 | 1,525647381 | 0,328147229 | |
| 1555501_s_at | RSRC1 | 2,854131948 | 2,526063925 | 0,328068024 | |
| 203676_at | GNS | 0,822971223 | 0,495031851 | 0,327939373 | |
| 213190_at | COG7 | 0,822971223 | 0,495031851 | 0,327939373 | |
| 203143_s_at | KIAA0040 | -0,622910612 | -0,950686014 | 0,327775402 | |
| 214142_at | ZG16 | -0,622910612 | -0,950686014 | 0,327775402 | |
| 218063_s_at | CDC42EP4 | -0,622910612 | -0,950686014 | 0,327775402 | |
| 220074_at | CDHR5 | -0,622910612 | -0,950686014 | 0,327775402 | |
| 220635_at | PSORS1C2 | -0,622910612 | -0,950686014 | 0,327775402 | |
| 224672_x_at | MESDC2 | -0,622910612 | -0,950686014 | 0,327775402 | |
| 234626_at | OR51I1 | -0,622910612 | -0,950686014 | 0,327775402 | |
| 203448_s_at | TERF1 | 3,706908684 | 3,379145507 | 0,327763177 | |
| 221165_s_at | IL22 | -0,43775896 | -0,765504029 | 0,327745069 | |
| 234075_at | - | -0,43775896 | -0,765504029 | 0,327745069 | |
| 243631_at | - | -0,43775896 | -0,765504029 | 0,327745069 | |
| 244698_at | CDRT4 /// FAM18 | -0,43775896 | -0,765504029 | 0,327745069 | |
| 208249_s_at | TGDS | 3,146001821 | 2,81832103 | 0,327680791 | |
| 211930_at | HNRNPA3 | 3,146001821 | 2,81832103 | 0,327680791 | |
| 217087_at | C1orf68 | 0,917308734 | 0,589657387 | 0,327651347 | |
| 213041_s_at | ATP5D | 4,20284547 | 3,875225653 | 0,327619817 | |
| 202609_at | EPS8 | 3,054253462 | 2,726791458 | 0,327462004 | |
| 214429_at | MTMR6 | 3,518475927 | 3,191118448 | 0,327357479 | |
| 203741_s_at | ADCY7 | 3,115233629 | 2,787940144 | 0,327293485 | |
| 203647_s_at | FDX1 | 3,977794563 | 3,650516168 | 0,327278396 | |
| 208816_x_at | ANXA2P2 | 4,325653301 | 3,9984201 | 0,327233201 | |
| 1559879_a_at | - | -2,908843709 | -3,236070201 | 0,327226492 | |
| 1570593_at | - | -2,908843709 | -3,236070201 | 0,327226492 | |
| 1557876_at | LOC340094 | -3,07502285 | -3,402185466 | 0,327162616 | |
| 1561240_at | - | -3,07502285 | -3,402185466 | 0,327162616 | |
| 1567387_at | - | -3,07502285 | -3,402185466 | 0,327162616 | |
| 240742_at | - | 0,760715727 | 0,433557298 | 0,327158429 | |
| 1562305_x_at | ZKSCAN3 | -0,022466681 | -0,34958478 | 0,3271181 | |
| 213569_at | LOC100506603 | -0,022466681 | -0,34958478 | 0,3271181 | |
| 230926_s_at | ODF2L | -0,022466681 | -0,34958478 | 0,3271181 | |
| 233443_at | PSMG4 | -0,022466681 | -0,34958478 | 0,3271181 | |
| 217640_x_at | SKA1 | 2,277177578 | 1,950068772 | 0,327108806 | |
| 214009_at | MSL3 | 1,063722698 | 0,73688026 | 0,326842437 | |
| 206712_at | GRTP1 | -0,352951799 | -0,679763839 | 0,326812041 | |
| 226761_at | IKZF4 | -0,352951799 | -0,679763839 | 0,326812041 | |
| 239389_at | TARBP2 | -0,352951799 | -0,679763839 | 0,326812041 | |
| 218522_s_at | MAP1S | 1,143956113 | 0,817300337 | 0,326655776 | |
| 226754_at | ZNF251 | 1,143956113 | 0,817300337 | 0,326655776 | |
| 1564063_a_at | ATP11B | 1,314003737 | 0,987376598 | 0,326627139 | |
| 227785_at | SDCCAG8 | 1,314003737 | 0,987376598 | 0,326627139 | |
| 226472_at | PPIL4 | 3,565667132 | 3,239069096 | 0,326598036 | |
| 228045_at | - | 1,58584542 | 1,259496994 | 0,326348426 | |
| 225105_at | C12orf75 | 4,848036436 | 4,52176814 | 0,326268296 | |
| 201559_s_at | CLIC4 | 1,326951551 | 1,000684521 | 0,32626703 | |

| | | | | | |
|---|---|---|---|---|---|
| 212200_at | ANKLE2 | 1,814495998 | 1,488344553 | 0,326151446 | |
| 225773_at | RSPRY1 | 3,159334685 | 2,833274877 | 0,326059808 | |
| 213431_x_at | SFI1 | 1,094335495 | 0,768343791 | 0,325991704 | |
| 224497_x_at | HSD17B14 | 0,246434561 | -0,079522948 | 0,325957509 | |
| 206745_at | HOXC11 | 0,984222186 | 0,658444279 | 0,325777906 | |
| 213907_at | EEF1E1 | 2,758612869 | 2,432926755 | 0,325686114 | |
| 213256_at | 03.03.15 | 3,612245157 | 3,28656411 | 0,325681047 | |
| 241972_at | LOC401588 | 1,880387527 | 1,55481039 | 0,325577137 | |
| 1553956_at | TMEM237 | 2,396622324 | 2,071071048 | 0,325551276 | |
| 1558793_at | FRY-AS1 | -2,712026959 | -3,037500868 | 0,325473909 | |
| 1558972_s_at | THEMIS | -2,712026959 | -3,037500868 | 0,325473909 | |
| 1559590_at | CHDH | -2,712026959 | -3,037500868 | 0,325473909 | |
| 1562307_at | - | -2,712026959 | -3,037500868 | 0,325473909 | |
| 1563486_at | - | -2,712026959 | -3,037500868 | 0,325473909 | |
| 1564758_at | LOC643659 | -2,712026959 | -3,037500868 | 0,325473909 | |
| 1566202_at | - | -2,712026959 | -3,037500868 | 0,325473909 | |
| 205337_at | DCT | -2,712026959 | -3,037500868 | 0,325473909 | |
| 228885_at | MAMDC2 | -2,712026959 | -3,037500868 | 0,325473909 | |
| 233636_at | MIR17HG | -2,712026959 | -3,037500868 | 0,325473909 | |
| 234108_at | TAS2R45 | -2,712026959 | -3,037500868 | 0,325473909 | |
| 236339_at | PPM1L | -2,712026959 | -3,037500868 | 0,325473909 | |
| 239477_at | EFHB | -2,712026959 | -3,037500868 | 0,325473909 | |
| 240771_at | C1orf101 | -2,712026959 | -3,037500868 | 0,325473909 | |
| 242880_at | NALCN | -2,712026959 | -3,037500868 | 0,325473909 | |
| 217322_x_at | - | 2,768138014 | 2,442710777 | 0,325427237 | |
| 206698_at | XK | 5,08686793 | 4,761478085 | 0,325389845 | |
| 214001_x_at | - | 2,646022818 | 2,320681318 | 0,3253415 | |
| 215644_at | LOC100509474 / | -0,136301282 | -0,461594427 | 0,325293144 | |
| 217637_at | - | -0,136301282 | -0,461594427 | 0,325293144 | |
| 232320_at | LOC100509205 | -0,136301282 | -0,461594427 | 0,325293144 | |
| 235890_at | TBL1XR1 | -0,136301282 | -0,461594427 | 0,325293144 | |
| 241667_x_at | - | -0,136301282 | -0,461594427 | 0,325293144 | |
| 221007_s_at | FIP1L1 | 2,691784869 | 2,366632651 | 0,325152218 | |
| 235203_at | - | 1,493160564 | 1,16809119 | 0,325069374 | |
| 207824_s_at | MAZ | 1,274448124 | 0,949447784 | 0,32500034 | |
| 201862_s_at | LRRFIP1 | 3,498586477 | 3,173599504 | 0,324986974 | |
| 1552627_a_at | ARHGAP5 | 0,592200562 | 0,26733076 | 0,324869802 | |
| 209574_s_at | C18orf1 | 0,592200562 | 0,26733076 | 0,324869802 | |
| 214782_at | CTTN | 0,592200562 | 0,26733076 | 0,324869802 | |
| 204632_at | RPS6KA4 | 2,344516784 | 2,019703483 | 0,324813301 | |
| 201421_s_at | WDR77 | 2,845159691 | 2,520531219 | 0,324628472 | |
| 226732_at | RBM33 | 3,716780201 | 3,392332256 | 0,324447946 | |
| 223268_at | C11orf54 | 2,166283906 | 1,841838775 | 0,324445131 | |
| 229270_x_at | SSBP4 | 0,962260738 | 0,638150376 | 0,324110362 | |
| 222088_s_at | SLC2A14 /// SLC | 5,219497001 | 4,895396095 | 0,324100906 | |
| 231840_x_at | LYRM7 | 2,218170265 | 1,894118818 | 0,324051447 | |
| 202886_s_at | PPP2R1B | 2,254767157 | 1,930732138 | 0,32403502 | |
| 218622_at | NUP37 | 4,975475611 | 4,651455664 | 0,324019947 | |
| 230264_s_at | AP1S2 | 6,919202954 | 6,595288643 | 0,323914311 | |
| 214869_x_at | GAPVD1 | 2,577249059 | 2,253342112 | 0,323906946 | |
| 34210_at | CD52 | -0,339265673 | -0,663160505 | 0,323894832 | |
| 1554961_at | FGFR4 | 0,103263601 | -0,220592223 | 0,323855825 | |
| 1556769_a_at | - | 0,103263601 | -0,220592223 | 0,323855825 | |
| 1559054_a_at | - | 0,103263601 | -0,220592223 | 0,323855825 | |
| 206743_s_at | ASGR1 | 0,103263601 | -0,220592223 | 0,323855825 | |
| 226800_at | EFCAB7 | 0,103263601 | -0,220592223 | 0,323855825 | |

| | | | | | |
|---|---|---|---|---|---|
| 228391_at | CYP4V2 | 0,103263601 | -0,220592223 | 0,323855825 | |
| 230946_at | FMN2 | 0,103263601 | -0,220592223 | 0,323855825 | |
| 234355_s_at | PTCHD2 | 0,103263601 | -0,220592223 | 0,323855825 | |
| 236812_at | STMN4 | 0,103263601 | -0,220592223 | 0,323855825 | |
| 236965_at | UBQLNL | 0,103263601 | -0,220592223 | 0,323855825 | |
| 1555516_at | FOXP2 | -1,838812296 | -2,162639771 | 0,323827475 | |
| 1559987_at | - | -1,838812296 | -2,162639771 | 0,323827475 | |
| 1562934_at | - | -1,838812296 | -2,162639771 | 0,323827475 | |
| 1566176_at | - | -1,838812296 | -2,162639771 | 0,323827475 | |
| 203528_at | SEMA4D | -1,838812296 | -2,162639771 | 0,323827475 | |
| 203873_at | SMARCA1 | -1,838812296 | -2,162639771 | 0,323827475 | |
| 215538_at | LARGE | -1,838812296 | -2,162639771 | 0,323827475 | |
| 217082_at | - | -1,838812296 | -2,162639771 | 0,323827475 | |
| 222954_at | FBXO40 | -1,838812296 | -2,162639771 | 0,323827475 | |
| 227735_s_at | C10orf99 | -1,838812296 | -2,162639771 | 0,323827475 | |
| 229072_at | RAB30 | -1,838812296 | -2,162639771 | 0,323827475 | |
| 230794_at | PELI2 | -1,838812296 | -2,162639771 | 0,323827475 | |
| 233190_at | - | -1,838812296 | -2,162639771 | 0,323827475 | |
| 233420_at | - | -1,838812296 | -2,162639771 | 0,323827475 | |
| 239326_at | - | -1,838812296 | -2,162639771 | 0,323827475 | |
| 239570_at | RAB1A | -1,838812296 | -2,162639771 | 0,323827475 | |
| 243932_at | - | -1,838812296 | -2,162639771 | 0,323827475 | |
| 1552414_at | WFDC9 | -1,763254337 | -2,086984744 | 0,323730407 | |
| 1553314_a_at | KIF19 | -1,763254337 | -2,086984744 | 0,323730407 | |
| 1553630_at | C10orf107 | -1,763254337 | -2,086984744 | 0,323730407 | |
| 1554277_s_at | FANCM | -1,763254337 | -2,086984744 | 0,323730407 | |
| 1557346_a_at | C17orf51 /// FAM | -1,763254337 | -2,086984744 | 0,323730407 | |
| 1558281_a_at | TMEM184A | -1,763254337 | -2,086984744 | 0,323730407 | |
| 1560486_at | STXBP3 | -1,763254337 | -2,086984744 | 0,323730407 | |
| 1560520_at | LOC401312 | -1,763254337 | -2,086984744 | 0,323730407 | |
| 1563472_at | - | -1,763254337 | -2,086984744 | 0,323730407 | |
| 1563854_s_at | LOC283045 | -1,763254337 | -2,086984744 | 0,323730407 | |
| 1564423_a_at | SEC16B | -1,763254337 | -2,086984744 | 0,323730407 | |
| 1569320_at | GPBP1L1 | -1,763254337 | -2,086984744 | 0,323730407 | |
| 203900_at | SZT2 | -1,763254337 | -2,086984744 | 0,323730407 | |
| 204390_at | - | -1,763254337 | -2,086984744 | 0,323730407 | |
| 211571_s_at | VCAN | -1,763254337 | -2,086984744 | 0,323730407 | |
| 220916_at | - | -1,763254337 | -2,086984744 | 0,323730407 | |
| 220958_at | ULK4 | -1,763254337 | -2,086984744 | 0,323730407 | |
| 227272_at | C15orf52 | -1,763254337 | -2,086984744 | 0,323730407 | |
| 228153_at | RNF144B | -1,763254337 | -2,086984744 | 0,323730407 | |
| 234595_at | CATX-1 | -1,763254337 | -2,086984744 | 0,323730407 | |
| 237245_at | - | -1,763254337 | -2,086984744 | 0,323730407 | |
| 239113_at | LOC100507468 | -1,763254337 | -2,086984744 | 0,323730407 | |
| 240541_at | - | -1,763254337 | -2,086984744 | 0,323730407 | |
| 240725_at | - | -1,763254337 | -2,086984744 | 0,323730407 | |
| 241418_at | LOC344887 | -1,763254337 | -2,086984744 | 0,323730407 | |
| 241884_at | - | -1,763254337 | -2,086984744 | 0,323730407 | |
| 242234_at | XAF1 | -1,763254337 | -2,086984744 | 0,323730407 | |
| 230873_at | ARPC4-TTLL3 /// | 0,841135803 | 0,51742585 | 0,323709953 | |
| 1552623_at | HSH2D | -1,888254346 | -2,211750327 | 0,323495982 | |
| 1554062_at | XG | -1,888254346 | -2,211750327 | 0,323495982 | |
| 1554736_at | ARHGAP29 | -1,888254346 | -2,211750327 | 0,323495982 | |
| 1558532_at | TPM1 | -1,888254346 | -2,211750327 | 0,323495982 | |
| 1560559_at | - | -1,888254346 | -2,211750327 | 0,323495982 | |
| 1561846_s_at | CCDC168 | -1,888254346 | -2,211750327 | 0,323495982 | |

| | | | | | |
|---|---|---|---|---|---|
| 208162_s_at | - | -1,888254346 | -2,211750327 | 0,323495982 | |
| 211102_s_at | LILRA2 | -1,888254346 | -2,211750327 | 0,323495982 | |
| 213411_at | ADAM22 | -1,888254346 | -2,211750327 | 0,323495982 | |
| 219676_at | ZSCAN16 | -1,888254346 | -2,211750327 | 0,323495982 | |
| 230589_at | TRAF3IP2-AS1 | -1,888254346 | -2,211750327 | 0,323495982 | |
| 230744_at | FSTL1 | -1,888254346 | -2,211750327 | 0,323495982 | |
| 231365_at | HOXA-AS4 /// MI | -1,888254346 | -2,211750327 | 0,323495982 | |
| 234354_x_at | ERBB2 | -1,888254346 | -2,211750327 | 0,323495982 | |
| 235962_at | AZI2 | -1,888254346 | -2,211750327 | 0,323495982 | |
| 236071_at | LOC494558 | -1,888254346 | -2,211750327 | 0,323495982 | |
| 238101_at | BEND4 | -1,888254346 | -2,211750327 | 0,323495982 | |
| 238991_at | ASB1 | -1,888254346 | -2,211750327 | 0,323495982 | |
| 240504_at | - | -1,888254346 | -2,211750327 | 0,323495982 | |
| 240639_at | ARMC9 | -1,888254346 | -2,211750327 | 0,323495982 | |
| 241608_at | - | -1,888254346 | -2,211750327 | 0,323495982 | |
| 241810_at | LOC100131180 | -1,888254346 | -2,211750327 | 0,323495982 | |
| 209259_s_at | SMC3 | 4,036776538 | 3,713295978 | 0,32348056 | |
| 210705_s_at | TRIM5 | 2,281618189 | 1,958277216 | 0,323340973 | |
| 1564736_a_at | CASP12 | -1,71423189 | -2,037551727 | 0,323319837 | |
| 1565867_a_at | ZC3H11A | -1,71423189 | -2,037551727 | 0,323319837 | |
| 1566181_at | - | -1,71423189 | -2,037551727 | 0,323319837 | |
| 1568908_at | - | -1,71423189 | -2,037551727 | 0,323319837 | |
| 206099_at | PRKCH | -1,71423189 | -2,037551727 | 0,323319837 | |
| 208606_s_at | WNT4 | -1,71423189 | -2,037551727 | 0,323319837 | |
| 211856_x_at | CD28 | -1,71423189 | -2,037551727 | 0,323319837 | |
| 216166_at | - | -1,71423189 | -2,037551727 | 0,323319837 | |
| 219393_s_at | AKT3 | -1,71423189 | -2,037551727 | 0,323319837 | |
| 232072_at | - | -1,71423189 | -2,037551727 | 0,323319837 | |
| 240269_at | - | -1,71423189 | -2,037551727 | 0,323319837 | |
| 241940_at | - | -1,71423189 | -2,037551727 | 0,323319837 | |
| 243168_at | - | -1,71423189 | -2,037551727 | 0,323319837 | |
| 201581_at | TMX4 | 2,987866617 | 2,664608955 | 0,323257662 | |
| 202336_s_at | PAM | 4,587453139 | 4,264240901 | 0,323212239 | |
| 1557777_at | PDE6B | 0,480753764 | 0,157549243 | 0,323204521 | |
| 205013_s_at | ADORA2A | 0,480753764 | 0,157549243 | 0,323204521 | |
| 215909_x_at | MINK1 | 0,351548291 | 0,028364014 | 0,323184277 | |
| 240656_at | - | 0,351548291 | 0,028364014 | 0,323184277 | |
| 221708_s_at | UNC45A | 2,11992033 | 1,796776588 | 0,323143742 | |
| 1555539_at | SDS | -0,83191556 | -1,154897679 | 0,322982119 | |
| 1560153_at | FRAS1 | -0,83191556 | -1,154897679 | 0,322982119 | |
| 1560966_at | - | -0,83191556 | -1,154897679 | 0,322982119 | |
| 207414_s_at | LOC100507472 / | -0,83191556 | -1,154897679 | 0,322982119 | |
| 220014_at | PRR16 | -0,83191556 | -1,154897679 | 0,322982119 | |
| 221129_at | FAM215A | -0,83191556 | -1,154897679 | 0,322982119 | |
| 229282_at | GATA6 | -0,83191556 | -1,154897679 | 0,322982119 | |
| 232360_at | EHF | -0,83191556 | -1,154897679 | 0,322982119 | |
| 235916_at | YPEL4 | -0,83191556 | -1,154897679 | 0,322982119 | |
| 238779_at | DCP2 | -0,83191556 | -1,154897679 | 0,322982119 | |
| 241702_at | - | -0,83191556 | -1,154897679 | 0,322982119 | |
| 225026_at | CHD6 | 1,283332461 | 0,960386719 | 0,322945741 | |
| 239138_at | NAA50 | 0,779676066 | 0,456918297 | 0,322757768 | |
| 229538_s_at | IQGAP3 | 0,67554678 | 0,352836757 | 0,322710023 | |
| 218065_s_at | TMEM9B | 3,513765154 | 3,191118448 | 0,322646707 | |
| 1567458_s_at | RAC1 | 4,657889997 | 4,335292864 | 0,322597134 | |
| 228324_at | C9orf41 | 3,302773593 | 2,980244813 | 0,322528781 | |
| 224663_s_at | CFL2 | 1,954567604 | 1,632095957 | 0,322471647 | |

| | | | | | |
|---|---|---|---|---|---|
| 1552970_s_at | ZMYM6 | -1,964176657 | -2,286583451 | 0,322406794 | |
| 1553521_at | DEFB104A /// DE | -1,964176657 | -2,286583451 | 0,322406794 | |
| 1556117_at | LOC79015 | -1,964176657 | -2,286583451 | 0,322406794 | |
| 1557543_at | - | -1,964176657 | -2,286583451 | 0,322406794 | |
| 1561114_a_at | DEPDC4 | -1,964176657 | -2,286583451 | 0,322406794 | |
| 1569450_at | CAPZA2 | -1,964176657 | -2,286583451 | 0,322406794 | |
| 202917_s_at | S100A8 | -1,964176657 | -2,286583451 | 0,322406794 | |
| 215106_at | TTC22 | -1,964176657 | -2,286583451 | 0,322406794 | |
| 228193_s_at | RGCC | -1,964176657 | -2,286583451 | 0,322406794 | |
| 229868_s_at | LOC100653010 | -1,964176657 | -2,286583451 | 0,322406794 | |
| 230473_s_at | HMGB4 | -1,964176657 | -2,286583451 | 0,322406794 | |
| 230890_at | LOC100506821 | -1,964176657 | -2,286583451 | 0,322406794 | |
| 232943_at | - | -1,964176657 | -2,286583451 | 0,322406794 | |
| 233106_at | FRMD6-AS1 | -1,964176657 | -2,286583451 | 0,322406794 | |
| 233903_s_at | ARHGEF26 | -1,964176657 | -2,286583451 | 0,322406794 | |
| 239456_at | - | -1,964176657 | -2,286583451 | 0,322406794 | |
| 239502_at | - | -1,964176657 | -2,286583451 | 0,322406794 | |
| 240078_at | SFSWAP | -1,964176657 | -2,286583451 | 0,322406794 | |
| 241011_at | - | -1,964176657 | -2,286583451 | 0,322406794 | |
| 222613_at | C12orf4 | 3,069741844 | 2,747463672 | 0,322278172 | |
| 233329_s_at | KRCC1 | 1,659347917 | 1,337086025 | 0,322261892 | |
| 1555819_s_at | SAMD14 | -0,496991414 | -0,819238336 | 0,322246922 | |
| 1558331_at | SIRT2 | -0,496991414 | -0,819238336 | 0,322246922 | |
| 203472_s_at | SLCO2B1 | -0,496991414 | -0,819238336 | 0,322246922 | |
| 204376_at | VPRBP | -0,496991414 | -0,819238336 | 0,322246922 | |
| 217114_at | SNRNP200 | -0,496991414 | -0,819238336 | 0,322246922 | |
| 230758_at | GEMIN8 | -0,496991414 | -0,819238336 | 0,322246922 | |
| 233176_at | LOC100507642 | -0,496991414 | -0,819238336 | 0,322246922 | |
| 237693_at | - | -0,496991414 | -0,819238336 | 0,322246922 | |
| 239168_at | LOC100652811 / | -0,496991414 | -0,819238336 | 0,322246922 | |
| 224698_at | ESYT2 | 3,21379258 | 2,891586849 | 0,322205731 | |
| 206758_at | EDN2 | -0,00073712 | -0,322870281 | 0,322133161 | |
| 212554_at | CAP2 | -0,00073712 | -0,322870281 | 0,322133161 | |
| 212366_at | ZNF292 | 2,238870125 | 1,916759838 | 0,322110287 | |
| 205202_at | PCMT1 | 5,503745873 | 5,181707664 | 0,322038208 | |
| 1556636_at | - | -1,639579028 | -1,961547147 | 0,321968119 | |
| 1560225_at | CNR1 | -1,639579028 | -1,961547147 | 0,321968119 | |
| 1561491_at | LOC283214 | -1,639579028 | -1,961547147 | 0,321968119 | |
| 1564044_at | KNCN | -1,639579028 | -1,961547147 | 0,321968119 | |
| 1568661_at | - | -1,639579028 | -1,961547147 | 0,321968119 | |
| 206389_s_at | PDE3A | -1,639579028 | -1,961547147 | 0,321968119 | |
| 207691_x_at | ENTPD1 | -1,639579028 | -1,961547147 | 0,321968119 | |
| 215302_at | LOC257152 | -1,639579028 | -1,961547147 | 0,321968119 | |
| 216168_at | - | -1,639579028 | -1,961547147 | 0,321968119 | |
| 219667_s_at | BANK1 | -1,639579028 | -1,961547147 | 0,321968119 | |
| 230796_at | LOC440900 | -1,639579028 | -1,961547147 | 0,321968119 | |
| 233078_at | API5 | -1,639579028 | -1,961547147 | 0,321968119 | |
| 234250_at | LOC100506405 | -1,639579028 | -1,961547147 | 0,321968119 | |
| 234790_at | - | -1,639579028 | -1,961547147 | 0,321968119 | |
| 240678_at | - | -1,639579028 | -1,961547147 | 0,321968119 | |
| 244754_at | - | -1,639579028 | -1,961547147 | 0,321968119 | |
| 1553192_at | ZNF441 | 0,401366113 | 0,079428135 | 0,321937978 | |
| 225979_at | PLEKHG2 | 0,911589958 | 0,589657387 | 0,321932571 | |
| 225202_at | RHOBTB3 | 5,891396388 | 5,569485024 | 0,321911365 | |
| 208875_s_at | PAK2 | 2,833109217 | 2,511262629 | 0,321846588 | |
| 223554_s_at | RANGRF | 1,46610492 | 1,144305291 | 0,321799629 | |

| | | | | | |
|---|---|---|---|---|---|
| 1554705_at | SCARA5 | -0,59053611 | -0,912331589 | 0,321795478 | |
| 206586_at | CNR2 | -0,59053611 | -0,912331589 | 0,321795478 | |
| 210843_s_at | MFAP3L | -0,59053611 | -0,912331589 | 0,321795478 | |
| 215439_x_at | - | -0,59053611 | -0,912331589 | 0,321795478 | |
| 226904_at | SLC6A10P /// SL( | -0,59053611 | -0,912331589 | 0,321795478 | |
| 230971_x_at | GLTPD2 | -0,59053611 | -0,912331589 | 0,321795478 | |
| 244605_at | - | -0,59053611 | -0,912331589 | 0,321795478 | |
| 213547_at | CAND2 | 1,40228911 | 1,080561626 | 0,321727484 | |
| 226615_at | XPR1 | 0,715481152 | 0,393761504 | 0,321719647 | |
| 229285_at | RNASEL | 0,715481152 | 0,393761504 | 0,321719647 | |
| 1552990_at | FAM71E2 | -2,01371626 | -2,335104118 | 0,321387858 | |
| 1553514_a_at | VNN3 | -2,01371626 | -2,335104118 | 0,321387858 | |
| 1555807_a_at | MOG | -2,01371626 | -2,335104118 | 0,321387858 | |
| 1558777_at | MKL2 | -2,01371626 | -2,335104118 | 0,321387858 | |
| 1559347_at | - | -2,01371626 | -2,335104118 | 0,321387858 | |
| 1563038_at | - | -2,01371626 | -2,335104118 | 0,321387858 | |
| 1563466_at | MYLK | -2,01371626 | -2,335104118 | 0,321387858 | |
| 1566098_at | - | -2,01371626 | -2,335104118 | 0,321387858 | |
| 1566585_at | - | -2,01371626 | -2,335104118 | 0,321387858 | |
| 1569322_at | LOC439990 | -2,01371626 | -2,335104118 | 0,321387858 | |
| 1569990_at | NUDT3 | -2,01371626 | -2,335104118 | 0,321387858 | |
| 202986_at | ARNT2 | -2,01371626 | -2,335104118 | 0,321387858 | |
| 207174_at | GPC5 | -2,01371626 | -2,335104118 | 0,321387858 | |
| 216003_at | - | -2,01371626 | -2,335104118 | 0,321387858 | |
| 216753_at | - | -2,01371626 | -2,335104118 | 0,321387858 | |
| 222255_at | PRX | -2,01371626 | -2,335104118 | 0,321387858 | |
| 235100_at | - | -2,01371626 | -2,335104118 | 0,321387858 | |
| 237526_at | - | -2,01371626 | -2,335104118 | 0,321387858 | |
| 237534_at | - | -2,01371626 | -2,335104118 | 0,321387858 | |
| 239253_at | - | -2,01371626 | -2,335104118 | 0,321387858 | |
| 242797_x_at | - | -2,01371626 | -2,335104118 | 0,321387858 | |
| 211406_at | IER3IP1 | 1,751865623 | 1,43051703 | 0,321348592 | |
| 202458_at | PRSS23 | 4,777867415 | 4,456625687 | 0,321241728 | |
| 201992_s_at | KIF5B | 2,314494121 | 1,993317152 | 0,321176968 | |
| 234733_s_at | FANCM | 2,314494121 | 1,993317152 | 0,321176968 | |
| 1773_at | CHURC1-FNTB / | 1,206012206 | 0,884886581 | 0,321125626 | |
| 209187_at | DR1 | 4,399525568 | 4,078419261 | 0,321106307 | |
| 215287_at | STRN | -0,325749314 | -0,646746079 | 0,320996765 | |
| 226002_at | GAB1 | -0,325749314 | -0,646746079 | 0,320996765 | |
| 227773_at | SAMD10 | -0,325749314 | -0,646746079 | 0,320996765 | |
| 234216_at | FLJ21408 | -0,325749314 | -0,646746079 | 0,320996765 | |
| 234239_at | - | -0,325749314 | -0,646746079 | 0,320996765 | |
| 240473_at | - | -0,325749314 | -0,646746079 | 0,320996765 | |
| 213712_at | ELOVL2 | 0,853119696 | 0,532164371 | 0,320955325 | |
| 227094_at | DHTKD1 | 0,853119696 | 0,532164371 | 0,320955325 | |
| 1553801_a_at | C14orf126 | 1,089278259 | 0,768343791 | 0,320934469 | |
| 1564552_at | - | -1,173767127 | -1,494668682 | 0,320901555 | |
| 207561_s_at | ABCB8 /// ASIC3 | -1,173767127 | -1,494668682 | 0,320901555 | |
| 216130_at | - | -1,173767127 | -1,494668682 | 0,320901555 | |
| 217567_at | TGM4 | -1,173767127 | -1,494668682 | 0,320901555 | |
| 220524_at | EPB41L4B | -1,173767127 | -1,494668682 | 0,320901555 | |
| 220756_s_at | SLC52A1 | -1,173767127 | -1,494668682 | 0,320901555 | |
| 220963_s_at | RSG1 | -1,173767127 | -1,494668682 | 0,320901555 | |
| 221367_at | MOS | -1,173767127 | -1,494668682 | 0,320901555 | |
| 221631_at | CACNA1I | -1,173767127 | -1,494668682 | 0,320901555 | |
| 225942_at | NLN | -1,173767127 | -1,494668682 | 0,320901555 | |

| | | | | | |
|---|---|---|---|---|---|
| 229014_at | FLJ42709 | -1,173767127 | -1,494668682 | 0,320901555 | |
| 229148_at | - | -1,173767127 | -1,494668682 | 0,320901555 | |
| 231009_at | PLA2G12B | -1,173767127 | -1,494668682 | 0,320901555 | |
| 233725_at | - | -1,173767127 | -1,494668682 | 0,320901555 | |
| 234705_at | CATX-1 | -1,173767127 | -1,494668682 | 0,320901555 | |
| 236221_at | AP4B1 | -1,173767127 | -1,494668682 | 0,320901555 | |
| 241141_at | BMP6 | -1,173767127 | -1,494668682 | 0,320901555 | |
| 1552629_a_at | JMJD6 | -1,591296941 | -1,912089812 | 0,32079287 | |
| 1553188_s_at | PARD3B | -1,591296941 | -1,912089812 | 0,32079287 | |
| 1556456_at | FLJ39739 | -1,591296941 | -1,912089812 | 0,32079287 | |
| 1559605_a_at | LOC285043 | -1,591296941 | -1,912089812 | 0,32079287 | |
| 1559971_at | BSDC1 | -1,591296941 | -1,912089812 | 0,32079287 | |
| 1562454_at | - | -1,591296941 | -1,912089812 | 0,32079287 | |
| 205852_at | CDK5R2 | -1,591296941 | -1,912089812 | 0,32079287 | |
| 208323_s_at | ANXA13 | -1,591296941 | -1,912089812 | 0,32079287 | |
| 210015_s_at | MAP2 | -1,591296941 | -1,912089812 | 0,32079287 | |
| 210603_at | NAA11 | -1,591296941 | -1,912089812 | 0,32079287 | |
| 211377_x_at | MYCN | -1,591296941 | -1,912089812 | 0,32079287 | |
| 212336_at | EPB41L1 | -1,591296941 | -1,912089812 | 0,32079287 | |
| 221884_at | MECOM | -1,591296941 | -1,912089812 | 0,32079287 | |
| 223557_s_at | TMEFF2 | -1,591296941 | -1,912089812 | 0,32079287 | |
| 223678_s_at | SFTPA1 /// SFTP | -1,591296941 | -1,912089812 | 0,32079287 | |
| 225250_at | STIM2 | -1,591296941 | -1,912089812 | 0,32079287 | |
| 228329_at | DAB1 | -1,591296941 | -1,912089812 | 0,32079287 | |
| 228803_at | PMS1 | -1,591296941 | -1,912089812 | 0,32079287 | |
| 234533_at | BCL2L14 | -1,591296941 | -1,912089812 | 0,32079287 | |
| 235571_at | - | -1,591296941 | -1,912089812 | 0,32079287 | |
| 236809_at | - | -1,591296941 | -1,912089812 | 0,32079287 | |
| 237076_at | NCSTN | -1,591296941 | -1,912089812 | 0,32079287 | |
| 240123_at | - | -1,591296941 | -1,912089812 | 0,32079287 | |
| 240411_at | HEATR7B2 | -1,591296941 | -1,912089812 | 0,32079287 | |
| 243592_at | REV1 | -1,591296941 | -1,912089812 | 0,32079287 | |
| 244578_at | LCP2 | -1,591296941 | -1,912089812 | 0,32079287 | |
| 201513_at | TSN | 2,156646206 | 1,835911187 | 0,320735019 | |
| 1568955_at | SRGAP2 /// SRG | 1,512180019 | 1,191491281 | 0,320688738 | |
| 207724_s_at | SPAST | 1,512180019 | 1,191491281 | 0,320688738 | |
| 226650_at | ZFAND2A | 3,28739962 | 2,966746113 | 0,320653507 | |
| 217800_s_at | NDFIP1 | 4,06796029 | 3,747642277 | 0,320318014 | |
| 213858_at | ZNF250 | 1,534677255 | 1,214517879 | 0,320159376 | |
| 203574_at | NFIL3 | 0,888485433 | 0,568365095 | 0,320120338 | |
| 215809_at | CYP2D6 | 0,888485433 | 0,568365095 | 0,320120338 | |
| 229143_at | CNOT3 | 0,888485433 | 0,568365095 | 0,320120338 | |
| 239038_at | C1orf52 | 0,888485433 | 0,568365095 | 0,320120338 | |
| 236249_at | IKBIP | 1,473887017 | 1,153866761 | 0,320020256 | |
| 201515_s_at | TSN | 4,839402629 | 4,519458551 | 0,319944077 | |
| 204330_s_at | MRPS12 | 2,808702036 | 2,488772006 | 0,31993003 | |
| 218426_s_at | RNF216 | 1,614289682 | 1,294497133 | 0,319792549 | |
| 219707_at | CPNE7 | 0,273439642 | -0,046302147 | 0,319741789 | |
| 220236_at | PDPR | 0,273439642 | -0,046302147 | 0,319741789 | |
| 202842_s_at | DNAJB9 | 2,817903185 | 2,498185745 | 0,31971744 | |
| 220212_s_at | THADA | 2,344516784 | 2,024923328 | 0,319593456 | |
| 218743_at | CHMP6 | 1,011212064 | 0,691645685 | 0,319566379 | |
| 221665_s_at | EPS8L1 | 1,011212064 | 0,691645685 | 0,319566379 | |
| 218538_s_at | MRS2 | 3,439206878 | 3,119728903 | 0,319477975 | |
| 1555141_a_at | SLC9B1 | -2,779459409 | -3,098858316 | 0,319398908 | |
| 1564635_a_at | FHAD1 | -2,779459409 | -3,098858316 | 0,319398908 | |

| | | | | | |
|---|---|---|---|---|---|
| 1564685_a_at | - | -2,779459409 | -3,098858316 | 0,319398908 | |
| 217126_at | - | -2,779459409 | -3,098858316 | 0,319398908 | |
| 220759_at | EDDM3B | -2,779459409 | -3,098858316 | 0,319398908 | |
| 220850_at | MORC1 | -2,779459409 | -3,098858316 | 0,319398908 | |
| 222259_s_at | SPO11 | -2,779459409 | -3,098858316 | 0,319398908 | |
| 241642_x_at | TLK1 | -2,779459409 | -3,098858316 | 0,319398908 | |
| 203465_at | MRPL19 | 3,512821151 | 3,193438316 | 0,319382836 | |
| 1554524_a_at | OLFM3 | -1,543331864 | -1,862647763 | 0,319315898 | |
| 1559568_at | - | -1,543331864 | -1,862647763 | 0,319315898 | |
| 1560812_at | - | -1,543331864 | -1,862647763 | 0,319315898 | |
| 1563157_at | - | -1,543331864 | -1,862647763 | 0,319315898 | |
| 1565495_at | CYAT1 /// IGLC1 | -1,543331864 | -1,862647763 | 0,319315898 | |
| 1569637_at | ZNF736 | -1,543331864 | -1,862647763 | 0,319315898 | |
| 1569680_at | - | -1,543331864 | -1,862647763 | 0,319315898 | |
| 207207_at | RBMXL2 | -1,543331864 | -1,862647763 | 0,319315898 | |
| 207660_at | DMD | -1,543331864 | -1,862647763 | 0,319315898 | |
| 208557_at | HOXA6 | -1,543331864 | -1,862647763 | 0,319315898 | |
| 209047_at | AQP1 | -1,543331864 | -1,862647763 | 0,319315898 | |
| 213478_at | KAZN | -1,543331864 | -1,862647763 | 0,319315898 | |
| 215774_s_at | SUCLG2 | -1,543331864 | -1,862647763 | 0,319315898 | |
| 220769_s_at | WDR78 | -1,543331864 | -1,862647763 | 0,319315898 | |
| 223645_s_at | TXLNG2P | -1,543331864 | -1,862647763 | 0,319315898 | |
| 233416_at | - | -1,543331864 | -1,862647763 | 0,319315898 | |
| 233958_at | - | -1,543331864 | -1,862647763 | 0,319315898 | |
| 234766_at | OR8D2 | -1,543331864 | -1,862647763 | 0,319315898 | |
| 236300_at | PDE3A | -1,543331864 | -1,862647763 | 0,319315898 | |
| 237237_at | - | -1,543331864 | -1,862647763 | 0,319315898 | |
| 242155_x_at | RFFL | -1,543331864 | -1,862647763 | 0,319315898 | |
| 242211_x_at | WDR90 | -1,543331864 | -1,862647763 | 0,319315898 | |
| 207602_at | TMPRSS11D | 0,123193849 | -0,19610998 | 0,319303829 | |
| 227316_at | PET117 | 0,123193849 | -0,19610998 | 0,319303829 | |
| 228615_at | LOC286161 | 0,123193849 | -0,19610998 | 0,319303829 | |
| 1553391_at | CXorf58 | -2,089474779 | -2,408705138 | 0,319230359 | |
| 1555383_a_at | POF1B | -2,089474779 | -2,408705138 | 0,319230359 | |
| 1561956_at | - | -2,089474779 | -2,408705138 | 0,319230359 | |
| 1569588_x_at | - | -2,089474779 | -2,408705138 | 0,319230359 | |
| 203924_at | GSTA1 | -2,089474779 | -2,408705138 | 0,319230359 | |
| 207544_s_at | ADH6 | -2,089474779 | -2,408705138 | 0,319230359 | |
| 207886_s_at | CALCR | -2,089474779 | -2,408705138 | 0,319230359 | |
| 209869_at | ADRA2A | -2,089474779 | -2,408705138 | 0,319230359 | |
| 213056_at | FRMD4B | -2,089474779 | -2,408705138 | 0,319230359 | |
| 215325_x_at | C19orf26 | -2,089474779 | -2,408705138 | 0,319230359 | |
| 221018_s_at | TDRD1 | -2,089474779 | -2,408705138 | 0,319230359 | |
| 223771_at | TMEM87A | -2,089474779 | -2,408705138 | 0,319230359 | |
| 226930_at | FNDC1 | -2,089474779 | -2,408705138 | 0,319230359 | |
| 231096_at | GDEP | -2,089474779 | -2,408705138 | 0,319230359 | |
| 232575_at | PCA3 | -2,089474779 | -2,408705138 | 0,319230359 | |
| 234007_at | DEFB121 | -2,089474779 | -2,408705138 | 0,319230359 | |
| 239286_at | CDH11 | -2,089474779 | -2,408705138 | 0,319230359 | |
| 240958_at | UNC5C | -2,089474779 | -2,408705138 | 0,319230359 | |
| 241290_at | - | -2,089474779 | -2,408705138 | 0,319230359 | |
| 243100_at | NT5C1B | -2,089474779 | -2,408705138 | 0,319230359 | |
| 243557_at | - | -2,089474779 | -2,408705138 | 0,319230359 | |
| 227455_at | C6orf136 | 1,844820248 | 1,525647381 | 0,319172867 | |
| 203608_at | ALDH5A1 | 4,10413628 | 3,785040604 | 0,319095676 | |
| 223482_at | TMEM120A | 2,589836943 | 2,271023131 | 0,318813812 | |

| | | | | | |
|---|---|---|---|---|---|
| 217752_s_at | CNDP2 | 2,956271699 | 2,637603218 | 0,318668481 | |
| 1554616_at | SERPINB8 | 0,541261317 | 0,222599095 | 0,318662222 | |
| 1561683_at | - | -0,172274514 | -0,490927335 | 0,318652821 | |
| 230819_at | C2CD4C | -0,172274514 | -0,490927335 | 0,318652821 | |
| 204975_at | EMP2 | 2,139623739 | 1,820984782 | 0,318638957 | |
| 223592_s_at | RNF135 | 2,531373922 | 2,212749286 | 0,318624636 | |
| 1555715_a_at | HRH3 | 0,798390451 | 0,479907041 | 0,31848341 | |
| 220632_s_at | POMT2 | 0,798390451 | 0,479907041 | 0,31848341 | |
| 242065_x_at | IDI1 | 0,798390451 | 0,479907041 | 0,31848341 | |
| 213704_at | RABGGTB /// SN | 2,571820446 | 2,253342112 | 0,318478334 | |
| 218238_at | GTPBP4 | 4,690399017 | 4,372110559 | 0,318288458 | |
| 232338_at | - | 1,073999308 | 0,755840599 | 0,318158709 | |
| 225947_at | MYO19 | 3,101484927 | 2,783327323 | 0,318157604 | |
| 1555614_at | SUGT1P1 | 0,695652136 | 0,377530647 | 0,31812149 | |
| 214876_s_at | TUBGCP5 | 0,695652136 | 0,377530647 | 0,31812149 | |
| 226898_s_at | SFPQ | 0,695652136 | 0,377530647 | 0,31812149 | |
| 229032_at | WSCD2 | 0,181384709 | -0,136643983 | 0,318028692 | |
| 229784_at | MGC16121 | 0,181384709 | -0,136643983 | 0,318028692 | |
| 244660_at | ELAVL1 | 0,181384709 | -0,136643983 | 0,318028692 | |
| 217266_at | RPL15 | 3,985300536 | 3,667303849 | 0,317996687 | |
| 218381_s_at | U2AF2 | 4,725454911 | 4,407512498 | 0,317942413 | |
| 226269_at | GDAP1 | 1,462198073 | 1,144305291 | 0,317892782 | |
| 204171_at | RPS6KB1 | 3,054253462 | 2,7363693 | 0,317884162 | |
| 228023_x_at | ACTG1P4 /// AM' | 1,767779954 | 1,450051551 | 0,317728403 | |
| 1566165_at | - | -2,138804305 | -2,456406426 | 0,317602121 | |
| 1570299_at | - | -2,138804305 | -2,456406426 | 0,317602121 | |
| 220087_at | BCMO1 | -2,138804305 | -2,456406426 | 0,317602121 | |
| 231217_at | - | -2,138804305 | -2,456406426 | 0,317602121 | |
| 234892_at | - | -2,138804305 | -2,456406426 | 0,317602121 | |
| 237577_at | PCNP | -2,138804305 | -2,456406426 | 0,317602121 | |
| 240614_at | KCNC2 | -2,138804305 | -2,456406426 | 0,317602121 | |
| 229355_at | UBE2D3 | 2,614687931 | 2,297144887 | 0,317543044 | |
| 236019_at | RAB12 | 1,104397092 | 0,786897721 | 0,317499371 | |
| 236274_at | EIF3B | 1,104397092 | 0,786897721 | 0,317499371 | |
| 207219_at | ZNF643 | 0,376672223 | 0,059218869 | 0,317453355 | |
| 206196_s_at | RUNDC3A | -0,79540287 | -1,112851844 | 0,317448974 | |
| 210126_at | PSG9 | -0,79540287 | -1,112851844 | 0,317448974 | |
| 232863_at | ZNF815P /// ZNF | -0,79540287 | -1,112851844 | 0,317448974 | |
| 233459_at | POLR3E | -0,79540287 | -1,112851844 | 0,317448974 | |
| 243621_at | SPACA3 | -0,79540287 | -1,112851844 | 0,317448974 | |
| 228749_at | ZDBF2 | 1,976645579 | 1,659204017 | 0,317441563 | |
| 201500_s_at | PPP1R11 | 4,049200824 | 3,731768909 | 0,317431915 | |
| 225652_at | - | 3,105247586 | 2,787940144 | 0,317307442 | |
| 238599_at | IRAK1BP1 | 0,735041319 | 0,417770464 | 0,317270856 | |
| 225994_at | CPSF2 | 4,13575284 | 3,818491075 | 0,317261765 | |
| 1557242_at | - | -0,946847173 | -1,264005341 | 0,317158168 | |
| 1558208_at | - | -0,946847173 | -1,264005341 | 0,317158168 | |
| 1566603_s_at | RPUSD3 | -0,946847173 | -1,264005341 | 0,317158168 | |
| 206732_at | SLITRK3 | -0,946847173 | -1,264005341 | 0,317158168 | |
| 214571_at | FGF3 | -0,946847173 | -1,264005341 | 0,317158168 | |
| 214778_at | MEGF8 | -0,946847173 | -1,264005341 | 0,317158168 | |
| 220162_s_at | CARD9 | -0,946847173 | -1,264005341 | 0,317158168 | |
| 229314_at | - | -0,946847173 | -1,264005341 | 0,317158168 | |
| 243622_at | LOC145694 | -0,946847173 | -1,264005341 | 0,317158168 | |
| 226543_at | EEF1E1-MUTED | 1,422536444 | 1,105412836 | 0,317123609 | |
| 211952_at | IPO5 | 2,051361651 | 1,73441697 | 0,316944681 | |

| | | | | | |
|---|---|---|---|---|---|
| 202981_x_at | SIAH1 | 3,42923503 | 3,112391743 | 0,316843287 | |
| 238096_at | LOC284023 | 1,238353345 | 0,921731975 | 0,31662137 | |
| 227485_at | DDX26B | 2,122398022 | 1,805902329 | 0,316495693 | |
| 206959_s_at | UPF3A | 1,05337236 | 0,73688026 | 0,316492099 | |
| 213946_s_at | OBSL1 | 1,05337236 | 0,73688026 | 0,316492099 | |
| 204329_s_at | ZNF202 | 0,425644434 | 0,109221392 | 0,316423041 | |
| 234929_s_at | SPATA7 | 0,425644434 | 0,109221392 | 0,316423041 | |
| 1556061_at | RPP30 | 1,889144008 | 1,572742379 | 0,316401629 | |
| 213852_at | RBM8A | 3,130084275 | 2,813804482 | 0,316279793 | |
| 1561387_a_at | NXPE1 | -1,470833215 | -1,787089803 | 0,316256588 | |
| 201428_at | CLDN4 | -1,470833215 | -1,787089803 | 0,316256588 | |
| 206226_at | HRG | -1,470833215 | -1,787089803 | 0,316256588 | |
| 208020_s_at | CACNA1C | -1,470833215 | -1,787089803 | 0,316256588 | |
| 210839_s_at | ENPP2 | -1,470833215 | -1,787089803 | 0,316256588 | |
| 214207_s_at | CARD10 | -1,470833215 | -1,787089803 | 0,316256588 | |
| 217444_at | - | -1,470833215 | -1,787089803 | 0,316256588 | |
| 224047_at | - | -1,470833215 | -1,787089803 | 0,316256588 | |
| 230076_at | PITPNM3 | -1,470833215 | -1,787089803 | 0,316256588 | |
| 230512_x_at | TMEM165 | -1,470833215 | -1,787089803 | 0,316256588 | |
| 231196_x_at | LINC00202 | -1,470833215 | -1,787089803 | 0,316256588 | |
| 233112_at | - | -1,470833215 | -1,787089803 | 0,316256588 | |
| 237767_at | - | -1,470833215 | -1,787089803 | 0,316256588 | |
| 237810_at | CLDN6 | -1,470833215 | -1,787089803 | 0,316256588 | |
| 240859_at | ZFYVE16 | -1,470833215 | -1,787089803 | 0,316256588 | |
| 242562_at | - | -1,470833215 | -1,787089803 | 0,316256588 | |
| 242930_at | - | -1,470833215 | -1,787089803 | 0,316256588 | |
| 243793_at | AHDC1 | -1,470833215 | -1,787089803 | 0,316256588 | |
| 224267_x_at | ZAN | -0,033456564 | -0,34958478 | 0,316128216 | |
| 226404_at | RBM39 | -0,033456564 | -0,34958478 | 0,316128216 | |
| 231400_s_at | - | -0,033456564 | -0,34958478 | 0,316128216 | |
| 232995_at | - | -0,033456564 | -0,34958478 | 0,316128216 | |
| 239508_x_at | CCDC108 | -0,033456564 | -0,34958478 | 0,316128216 | |
| 243946_at | SMOC2 | -0,033456564 | -0,34958478 | 0,316128216 | |
| 223298_s_at | NT5C3 | 4,037433122 | 3,721356767 | 0,316076355 | |
| 59375_at | MYO15B | 0,025976038 | -0,289989561 | 0,315965598 | |
| 225223_at | SMAD5 | 2,151803104 | 1,835911187 | 0,315891917 | |
| 223824_at | RNLS | 2,438982012 | 2,123108223 | 0,315873788 | |
| 1554240_a_at | ITGAL | -2,187914861 | -2,503677622 | 0,315762761 | |
| 1557775_a_at | - | -2,187914861 | -2,503677622 | 0,315762761 | |
| 1559523_at | - | -2,187914861 | -2,503677622 | 0,315762761 | |
| 1561309_x_at | - | -2,187914861 | -2,503677622 | 0,315762761 | |
| 1563907_at | GARNL3 | -2,187914861 | -2,503677622 | 0,315762761 | |
| 1567374_at | - | -2,187914861 | -2,503677622 | 0,315762761 | |
| 204818_at | HSD17B2 | -2,187914861 | -2,503677622 | 0,315762761 | |
| 219429_at | FA2H | -2,187914861 | -2,503677622 | 0,315762761 | |
| 221697_at | MAP1LC3C | -2,187914861 | -2,503677622 | 0,315762761 | |
| 224238_at | - | -2,187914861 | -2,503677622 | 0,315762761 | |
| 231930_at | ELMOD1 /// LOC | -2,187914861 | -2,503677622 | 0,315762761 | |
| 232354_at | - | -2,187914861 | -2,503677622 | 0,315762761 | |
| 240293_at | CCDC153 | -2,187914861 | -2,503677622 | 0,315762761 | |
| 240603_s_at | ERI2 | -2,187914861 | -2,503677622 | 0,315762761 | |
| 242954_at | - | -2,187914861 | -2,503677622 | 0,315762761 | |
| 217767_at | C3 | 0,291166226 | -0,024572586 | 0,315738812 | |
| 216525_x_at | PMS2P3 | 3,042527069 | 2,726791458 | 0,315735611 | |
| 208250_s_at | DMBT1 | 0,810733188 | 0,495031851 | 0,315701337 | |
| 229925_at | SLC6A17 | 0,810733188 | 0,495031851 | 0,315701337 | |

| | | | | | |
|---|---|---|---|---|---|
| 224391_s_at | SIAE | 1,191926657 | 0,876248481 | 0,315678176 | |
| 223046_at | EGLN1 | 3,992090425 | 3,67645448 | 0,315635945 | |
| 1552747_a_at | PP2D1 | -2,823166348 | -3,138626879 | 0,315460531 | |
| 1561763_at | - | -2,823166348 | -3,138626879 | 0,315460531 | |
| 236945_at | CCDC171 | -2,823166348 | -3,138626879 | 0,315460531 | |
| 237540_at | LOC100506767 | -2,823166348 | -3,138626879 | 0,315460531 | |
| 241545_x_at | - | -2,823166348 | -3,138626879 | 0,315460531 | |
| 241926_s_at | ERG | -2,823166348 | -3,138626879 | 0,315460531 | |
| 1568777_at | EML5 | -0,299034815 | -0,614371577 | 0,315336762 | |
| 216126_at | - | -0,299034815 | -0,614371577 | 0,315336762 | |
| 220824_at | - | -0,299034815 | -0,614371577 | 0,315336762 | |
| 228576_s_at | MXRA8 | -0,299034815 | -0,614371577 | 0,315336762 | |
| 242574_at | FKBP15 | -0,299034815 | -0,614371577 | 0,315336762 | |
| 243512_x_at | - | -0,299034815 | -0,614371577 | 0,315336762 | |
| 218609_s_at | NUDT2 | 1,726031811 | 1,410714371 | 0,315317439 | |
| 242809_at | IL1RL1 | 1,726031811 | 1,410714371 | 0,315317439 | |
| 212021_s_at | MKI67 | 2,586251598 | 2,271023131 | 0,315228467 | |
| 235096_at | LEO1 | 3,067171967 | 2,752192409 | 0,314979558 | |
| 220474_at | SLC25A21 | 3,5222335 | 3,207279726 | 0,314953774 | |
| 204411_at | KIF21B | 0,03125738 | -0,283667828 | 0,314925207 | |
| 209877_at | SNCG | 0,03125738 | -0,283667828 | 0,314925207 | |
| 230053_at | - | 0,03125738 | -0,283667828 | 0,314925207 | |
| 232126_at | COQ2 | 0,03125738 | -0,283667828 | 0,314925207 | |
| 236744_at | PHPT1 | 0,03125738 | -0,283667828 | 0,314925207 | |
| 229564_at | RRP7A | 0,97328325 | 0,658444279 | 0,314838971 | |
| 208072_s_at | DGKD | 2,156646206 | 1,841838775 | 0,314807431 | |
| 202770_s_at | CCNG2 | 1,912238072 | 1,59747851 | 0,314759562 | |
| 1557053_s_at | UBE2G2 | 5,834920167 | 5,52020409 | 0,314716077 | |
| 229551_x_at | ZNF367 | 2,782308844 | 2,467842989 | 0,314465855 | |
| 244103_at | SDE2 | 4,895888063 | 4,581441196 | 0,314446866 | |
| 1554213_at | ARHGEF10 | -0,221656139 | -0,536087151 | 0,314431012 | |
| 213776_at | LOC157562 | -0,221656139 | -0,536087151 | 0,314431012 | |
| 235074_at | SPRED1 | -0,221656139 | -0,536087151 | 0,314431012 | |
| 1559716_at | INO80C | 0,747935635 | 0,433557298 | 0,314378337 | |
| 230136_at | LOC400099 | 0,747935635 | 0,433557298 | 0,314378337 | |
| 1555241_at | C8orf59 | 0,882651021 | 0,568365095 | 0,314285926 | |
| 202195_s_at | TMED5 | 2,713311265 | 2,39915583 | 0,314155435 | |
| 223669_at | HEMGN | 1,274448124 | 0,960386719 | 0,314061405 | |
| 1554948_at | - | -1,424023738 | -1,738067356 | 0,314043618 | |
| 1559586_at | LOC728868 | -1,424023738 | -1,738067356 | 0,314043618 | |
| 201442_s_at | ATP6AP2 | -1,424023738 | -1,738067356 | 0,314043618 | |
| 206256_at | CPN1 | -1,424023738 | -1,738067356 | 0,314043618 | |
| 213003_s_at | KIAA0146 | -1,424023738 | -1,738067356 | 0,314043618 | |
| 214419_s_at | CYP2C9 | -1,424023738 | -1,738067356 | 0,314043618 | |
| 216779_at | CYLC1 | -1,424023738 | -1,738067356 | 0,314043618 | |
| 222167_at | - | -1,424023738 | -1,738067356 | 0,314043618 | |
| 228160_at | LOC339290 | -1,424023738 | -1,738067356 | 0,314043618 | |
| 230743_at | HOXB-AS3 | -1,424023738 | -1,738067356 | 0,314043618 | |
| 231042_s_at | - | -1,424023738 | -1,738067356 | 0,314043618 | |
| 233178_at | TGIF2LY | -1,424023738 | -1,738067356 | 0,314043618 | |
| 233706_at | - | -1,424023738 | -1,738067356 | 0,314043618 | |
| 233940_at | - | -1,424023738 | -1,738067356 | 0,314043618 | |
| 236074_at | LOC100506676 | -1,424023738 | -1,738067356 | 0,314043618 | |
| 237544_at | - | -1,424023738 | -1,738067356 | 0,314043618 | |
| 238152_at | C11orf95 | -1,424023738 | -1,738067356 | 0,314043618 | |
| 238222_at | GKN2 | -1,424023738 | -1,738067356 | 0,314043618 | |

| | | | | | |
|---|---|---|---|---|---|
| 238366_at | C1orf228 | -1,424023738 | -1,738067356 | 0,314043618 | |
| 239495_at | - | -1,424023738 | -1,738067356 | 0,314043618 | |
| 239958_at | LOC253039 | -1,424023738 | -1,738067356 | 0,314043618 | |
| 240869_at | - | -1,424023738 | -1,738067356 | 0,314043618 | |
| 242594_at | BOD1L1 | -1,424023738 | -1,738067356 | 0,314043618 | |
| 244168_s_at | ULK4 | -1,424023738 | -1,738067356 | 0,314043618 | |
| 244543_s_at | BCDIN3D-AS1 | -1,424023738 | -1,738067356 | 0,314043618 | |
| 207753_at | ZNF304 | 2,173470163 | 1,859476797 | 0,313993366 | |
| 1553441_at | CNTNAP4 | -3,192553003 | -3,506469152 | 0,31391615 | |
| 221452_s_at | TMEM14B | 6,408712215 | 6,094905529 | 0,313806686 | |
| 225393_at | GATAD2B | 0,917308734 | 0,603679647 | 0,313629087 | |
| 200037_s_at | CBX3 | 6,265930995 | 5,952363964 | 0,313567031 | |
| 223257_at | G2E3 | 2,094906514 | 1,781437557 | 0,313468957 | |
| 1552388_at | FLJ30901 | -0,148196756 | -0,461594427 | 0,31339767 | |
| 1553684_at | PPIL6 | -0,148196756 | -0,461594427 | 0,31339767 | |
| 1559432_at | CCBP2 | -0,148196756 | -0,461594427 | 0,31339767 | |
| 212963_at | TM2D1 | -0,148196756 | -0,461594427 | 0,31339767 | |
| 214296_x_at | IZUMO4 | -0,148196756 | -0,461594427 | 0,31339767 | |
| 204624_at | ATP7B | 3,858765357 | 3,545468469 | 0,313296889 | |
| 201244_s_at | RAF1 | 3,296204798 | 2,982929462 | 0,313275336 | |
| 218385_at | MRPS18A | 3,25953187 | 2,946258252 | 0,313273618 | |
| 205740_s_at | RBM42 | 5,103580305 | 4,790501109 | 0,313079197 | |
| 1555490_s_at | PDZD3 | -0,366776884 | -0,679763839 | 0,312986955 | |
| 1561667_at | - | -0,366776884 | -0,679763839 | 0,312986955 | |
| 1569847_at | CGNL1 | -0,366776884 | -0,679763839 | 0,312986955 | |
| 207026_s_at | ATP2B3 | -0,366776884 | -0,679763839 | 0,312986955 | |
| 217054_at | - | -0,366776884 | -0,679763839 | 0,312986955 | |
| 227104_x_at | ZNF800 | -0,366776884 | -0,679763839 | 0,312986955 | |
| 232749_at | - | -0,366776884 | -0,679763839 | 0,312986955 | |
| 235290_at | ZNF782 | -0,366776884 | -0,679763839 | 0,312986955 | |
| 1556037_s_at | HHIP | 0,606430281 | 0,293518836 | 0,312911446 | |
| 221142_s_at | PECR | 0,606430281 | 0,293518836 | 0,312911446 | |
| 1554096_a_at | RBM33 | 0,44160593 | 0,128747141 | 0,312858789 | |
| 207300_s_at | F7 | 0,44160593 | 0,128747141 | 0,312858789 | |
| 227303_at | ANKS3 | 0,44160593 | 0,128747141 | 0,312858789 | |
| 242589_x_at | - | 0,44160593 | 0,128747141 | 0,312858789 | |
| 1562231_at | - | 0,152582607 | -0,160136748 | 0,312719355 | |
| 208301_at | - | 0,152582607 | -0,160136748 | 0,312719355 | |
| 1559282_at | - | -0,543051993 | -0,855751026 | 0,312699033 | |
| 1559603_at | GPR12 | -0,543051993 | -0,855751026 | 0,312699033 | |
| 206972_s_at | GPR161 | -0,543051993 | -0,855751026 | 0,312699033 | |
| 210079_x_at | KCNAB1 | -0,543051993 | -0,855751026 | 0,312699033 | |
| 215858_at | - | -0,543051993 | -0,855751026 | 0,312699033 | |
| 216704_at | - | -0,543051993 | -0,855751026 | 0,312699033 | |
| 222724_at | VWA1 | -0,543051993 | -0,855751026 | 0,312699033 | |
| 229680_at | TACO1 | -0,543051993 | -0,855751026 | 0,312699033 | |
| 230444_at | PDE7A | -0,543051993 | -0,855751026 | 0,312699033 | |
| 236124_at | LOC153546 | -0,543051993 | -0,855751026 | 0,312699033 | |
| 243546_at | - | -0,543051993 | -0,855751026 | 0,312699033 | |
| 213244_at | SCAMP4 | 1,352503698 | 1,039887232 | 0,312616467 | |
| 205062_x_at | ARID4A | 2,05655332 | 1,743944487 | 0,312608833 | |
| 207939_x_at | RNPS1 | 4,176858496 | 3,864347045 | 0,312511451 | |
| 41553_at | OSGIN2 | 0,465232922 | 0,152806864 | 0,312426058 | |
| 1553363_at | C6orf195 | -2,262747984 | -2,575129729 | 0,312381745 | |
| 1558965_at | PHF21A | -2,262747984 | -2,575129729 | 0,312381745 | |
| 1559172_at | - | -2,262747984 | -2,575129729 | 0,312381745 | |

| | | | | | |
|---|---|---|---|---|---|
| 1566419_at | - | -2,262747984 | -2,575129729 | 0,312381745 | |
| 205399_at | DCLK1 | -2,262747984 | -2,575129729 | 0,312381745 | |
| 206048_at | OVOL2 | -2,262747984 | -2,575129729 | 0,312381745 | |
| 216717_at | FAM48A | -2,262747984 | -2,575129729 | 0,312381745 | |
| 219529_at | CLIC3 | -2,262747984 | -2,575129729 | 0,312381745 | |
| 220123_at | SLC35F5 | -2,262747984 | -2,575129729 | 0,312381745 | |
| 222249_at | - | -2,262747984 | -2,575129729 | 0,312381745 | |
| 228643_at | LOC100506233 | -2,262747984 | -2,575129729 | 0,312381745 | |
| 235658_at | - | -2,262747984 | -2,575129729 | 0,312381745 | |
| 240647_at | - | -2,262747984 | -2,575129729 | 0,312381745 | |
| 240658_at | - | -2,262747984 | -2,575129729 | 0,312381745 | |
| 240857_at | DNAH9 | -2,262747984 | -2,575129729 | 0,312381745 | |
| 241960_at | CSMD1 | -2,262747984 | -2,575129729 | 0,312381745 | |
| 242568_s_at | - | -2,262747984 | -2,575129729 | 0,312381745 | |
| 1562805_at | TLR8-AS1 | -0,078265071 | -0,39061235 | 0,312347279 | |
| 202249_s_at | DCAF8 | 0,792179257 | 0,479907041 | 0,312272216 | |
| 203395_s_at | HES1 | 1,46610492 | 1,153866761 | 0,312238159 | |
| 218908_at | ASPSCR1 | 1,46610492 | 1,153866761 | 0,312238159 | |
| 209175_at | SEC23IP | 3,575646363 | 3,263564154 | 0,312082209 | |
| 64418_at | SYNRG | 2,4830283 | 2,170981159 | 0,312047141 | |
| 238937_at | ZNF420 | 3,536237877 | 3,22439681 | 0,311841067 | |
| 204897_at | PTGER4 | 1,40228911 | 1,090553537 | 0,311735572 | |
| 205574_x_at | BMP1 | 0,209623097 | -0,102100538 | 0,311723635 | |
| 220879_at | - | 0,209623097 | -0,102100538 | 0,311723635 | |
| 233159_at | STARD13 | 0,209623097 | -0,102100538 | 0,311723635 | |
| 243757_at | - | 0,209623097 | -0,102100538 | 0,311723635 | |
| 203706_s_at | FZD7 | 1,742232072 | 1,43051703 | 0,311715042 | |
| 225537_at | TRAPPC6B | 3,711030028 | 3,399383202 | 0,311646826 | |
| 226946_at | NADKD1 | 2,837639949 | 2,526063925 | 0,311576024 | |
| 226070_at | C9orf142 | 1,593009234 | 1,281471383 | 0,311537851 | |
| 1554298_a_at | WDR49 | -2,866296344 | -3,177802286 | 0,311505942 | |
| 1558834_s_at | AKNAD1 | -2,866296344 | -3,177802286 | 0,311505942 | |
| 1570515_a_at | FILIP1 | -2,866296344 | -3,177802286 | 0,311505942 | |
| 221182_at | C1orf129 | -2,866296344 | -3,177802286 | 0,311505942 | |
| 224017_at | TBX22 | -2,866296344 | -3,177802286 | 0,311505942 | |
| 236203_at | HLA-DQA1 /// LO | -2,866296344 | -3,177802286 | 0,311505942 | |
| 239871_at | CLTC | -2,866296344 | -3,177802286 | 0,311505942 | |
| 244817_at | CARS2 | -2,866296344 | -3,177802286 | 0,311505942 | |
| 202028_s_at | RPL38 | 5,136435239 | 4,8249493 | 0,311485939 | |
| 1554307_at | LOC644852 | -2,64318869 | -2,954576755 | 0,311388065 | |
| 1554793_at | UBE3C | -2,64318869 | -2,954576755 | 0,311388065 | |
| 1556967_at | ZDHHC14 | -2,64318869 | -2,954576755 | 0,311388065 | |
| 1557606_at | - | -2,64318869 | -2,954576755 | 0,311388065 | |
| 1560201_at | ZNF713 | -2,64318869 | -2,954576755 | 0,311388065 | |
| 1563139_at | - | -2,64318869 | -2,954576755 | 0,311388065 | |
| 1563339_at | - | -2,64318869 | -2,954576755 | 0,311388065 | |
| 1564144_at | - | -2,64318869 | -2,954576755 | 0,311388065 | |
| 1567183_s_at | - | -2,64318869 | -2,954576755 | 0,311388065 | |
| 1569344_a_at | - | -2,64318869 | -2,954576755 | 0,311388065 | |
| 206211_at | SELE | -2,64318869 | -2,954576755 | 0,311388065 | |
| 213580_at | - | -2,64318869 | -2,954576755 | 0,311388065 | |
| 214837_at | ALB | -2,64318869 | -2,954576755 | 0,311388065 | |
| 216109_at | MED13L | -2,64318869 | -2,954576755 | 0,311388065 | |
| 219552_at | SVEP1 | -2,64318869 | -2,954576755 | 0,311388065 | |
| 222332_at | - | -2,64318869 | -2,954576755 | 0,311388065 | |
| 235382_at | AQPEP | -2,64318869 | -2,954576755 | 0,311388065 | |

| | | | | | |
|---|---|---|---|---|---|
| 240000_at | - | -2,64318869 | -2,954576755 | 0,311388065 | |
| 212064_x_at | MAZ | 2,427005622 | 2,115788273 | 0,311217349 | |
| 203787_at | SSBP2 | 1,614289682 | 1,303116084 | 0,311173597 | |
| 214112_s_at | CXorf40A /// CXo | 2,864529541 | 2,553413592 | 0,311115949 | |
| 214661_s_at | NOP14 | 2,864529541 | 2,553413592 | 0,311115949 | |
| 217910_x_at | MLX | 3,199783157 | 2,888726553 | 0,311056604 | |
| 208451_s_at | C4A /// C4B /// LC | 0,728550685 | 0,417770464 | 0,310780222 | |
| 213840_s_at | MRPS12 | 0,728550685 | 0,417770464 | 0,310780222 | |
| 232475_at | C15orf42 | 0,728550685 | 0,417770464 | 0,310780222 | |
| 227231_at | KIAA1211 | 1,079110292 | 0,768343791 | 0,310766502 | |
| 1554084_a_at | NOL9 | 1,556829052 | 1,246149888 | 0,310679163 | |
| 91816_f_at | MEX3D | 2,407837301 | 2,097324242 | 0,310513059 | |
| 213756_s_at | HSF1 | 0,57782909 | 0,26733076 | 0,310498331 | |
| 226411_at | EVI5L | 0,57782909 | 0,26733076 | 0,310498331 | |
| 230876_at | ZNF883 | 0,57782909 | 0,26733076 | 0,310498331 | |
| 41037_at | TEAD4 | -0,038978033 | -0,349437029 | 0,310458996 | |
| 215739_s_at | TUBGCP3 | 0,052199481 | -0,258114234 | 0,310313714 | |
| 235012_at | LRCH1 | 0,052199481 | -0,258114234 | 0,310313714 | |
| 236426_at | KIF19 | 0,052199481 | -0,258114234 | 0,310313714 | |
| 202515_at | DLG1 | 2,860082603 | 2,549796788 | 0,310285815 | |
| 225462_at | TMEM128 | 2,506929603 | 2,196649505 | 0,310280098 | |
| 235410_at | NPHP3 | 1,339784194 | 1,029536893 | 0,310247301 | |
| 1552509_a_at | CD300LG | -2,311268652 | -2,621331989 | 0,310063338 | |
| 1552809_at | RFX4 | -2,311268652 | -2,621331989 | 0,310063338 | |
| 1562387_at | C5orf42 | -2,311268652 | -2,621331989 | 0,310063338 | |
| 1564235_at | LOC729866 | -2,311268652 | -2,621331989 | 0,310063338 | |
| 1569128_at | C3orf38 | -2,311268652 | -2,621331989 | 0,310063338 | |
| 1569861_at | TRAF5 | -2,311268652 | -2,621331989 | 0,310063338 | |
| 205943_at | TDO2 | -2,311268652 | -2,621331989 | 0,310063338 | |
| 206153_at | CYP4F11 | -2,311268652 | -2,621331989 | 0,310063338 | |
| 207010_at | GABRB1 | -2,311268652 | -2,621331989 | 0,310063338 | |
| 207293_s_at | AGTR2 | -2,311268652 | -2,621331989 | 0,310063338 | |
| 219513_s_at | SH2D3A | -2,311268652 | -2,621331989 | 0,310063338 | |
| 231506_at | - | -2,311268652 | -2,621331989 | 0,310063338 | |
| 237446_at | - | -2,311268652 | -2,621331989 | 0,310063338 | |
| 238368_at | - | -2,311268652 | -2,621331989 | 0,310063338 | |
| 238940_at | KLF12 | -2,311268652 | -2,621331989 | 0,310063338 | |
| 244852_at | DSEL | -2,311268652 | -2,621331989 | 0,310063338 | |
| 212018_s_at | RSL1D1 | 5,163454645 | 4,853468768 | 0,309985877 | |
| 226366_at | SHPRH | 3,292909151 | 2,982929462 | 0,309979689 | |
| 204751_x_at | DSC2 | 0,317354302 | 0,007421914 | 0,309932388 | |
| 232772_at | LOC221272 | 0,317354302 | 0,007421914 | 0,309932388 | |
| 223404_s_at | TRMT1L | 2,886560922 | 2,576704359 | 0,309856562 | |
| 1552468_a_at | DSCR10 | -1,353583928 | -1,663414495 | 0,309830567 | |
| 1561314_at | LOC100506387 | -1,353583928 | -1,663414495 | 0,309830567 | |
| 207637_at | WSCD2 | -1,353583928 | -1,663414495 | 0,309830567 | |
| 207710_at | LCE2B | -1,353583928 | -1,663414495 | 0,309830567 | |
| 208886_at | H1F0 | -1,353583928 | -1,663414495 | 0,309830567 | |
| 210805_x_at | LOC100506403 / | -1,353583928 | -1,663414495 | 0,309830567 | |
| 234658_at | - | -1,353583928 | -1,663414495 | 0,309830567 | |
| 236214_at | FAM71F1 | -1,353583928 | -1,663414495 | 0,309830567 | |
| 239772_x_at | DHX30 | -1,353583928 | -1,663414495 | 0,309830567 | |
| 244853_at | - | -1,353583928 | -1,663414495 | 0,309830567 | |
| 232266_x_at | CDK13 | 3,812716554 | 3,503081281 | 0,309635273 | |
| 209993_at | ABCB1 | 1,269985355 | 0,960386719 | 0,309598635 | |
| 208137_x_at | ZNF611 | 1,751865623 | 1,442269454 | 0,309596169 | |

| | | | | | |
|---|---|---|---|---|---|
| 223295_s_at | LUC7L | 3,58196103 | 3,272369332 | 0,309591698 | |
| 224751_at | C7orf73 /// LOC1 | 5,271770432 | 4,962222456 | 0,309547976 | |
| 208766_s_at | HNRNPR | 6,40037808 | 6,090866876 | 0,309511204 | |
| 231768_at | USF1 | 1,390002893 | 1,080561626 | 0,309441268 | |
| 239250_at | ZNF542 | 3,548266005 | 3,239069096 | 0,30919691 | |
| 226657_at | C17orf103 | 0,661985843 | 0,352836757 | 0,309149085 | |
| 244587_at | ATF7 | 0,661985843 | 0,352836757 | 0,309149085 | |
| 210517_s_at | AKAP12 | 3,604290432 | 3,295230443 | 0,309059989 | |
| 204662_at | CCP110 | 3,115233629 | 2,806245339 | 0,30898829 | |
| 202749_at | WRB | 5,883566792 | 5,574611027 | 0,308955765 | |
| 223724_s_at | STAG3L1 /// STA | 1,62481334 | 1,315948728 | 0,308864612 | |
| 213555_at | RWDD2A | 1,000476627 | 0,691645685 | 0,308830942 | |
| 230927_at | - | 1,000476627 | 0,691645685 | 0,308830942 | |
| 1561219_x_at | - | -0,741668563 | -1,050406615 | 0,308738052 | |
| 1562674_at | - | -0,741668563 | -1,050406615 | 0,308738052 | |
| 1568943_at | INPP5D | -0,741668563 | -1,050406615 | 0,308738052 | |
| 213830_at | YME1L1 | -0,741668563 | -1,050406615 | 0,308738052 | |
| 222293_at | CADM4 | -0,741668563 | -1,050406615 | 0,308738052 | |
| 228209_at | LOC100527964 | -0,741668563 | -1,050406615 | 0,308738052 | |
| 231475_at | TBC1D21 | -0,741668563 | -1,050406615 | 0,308738052 | |
| 236366_at | LOC440149 | -0,741668563 | -1,050406615 | 0,308738052 | |
| 243830_at | - | -0,741668563 | -1,050406615 | 0,308738052 | |
| 1558956_s_at | IFT80 | 2,535098095 | 2,226407528 | 0,308690567 | |
| 208313_s_at | SF1 | 3,825698252 | 3,517040489 | 0,308657762 | |
| 1558508_a_at | C1orf53 | 2,701760055 | 2,39311332 | 0,308646736 | |
| 218000_s_at | PHLDA1 | 0,702292136 | 0,393761504 | 0,308530632 | |
| 219235_s_at | PHACTR4 | 1,940595304 | 1,632095957 | 0,308499347 | |
| 217349_s_at | PRICKLE3 | 0,171847695 | -0,136643983 | 0,308491678 | |
| 228216_at | - | 0,171847695 | -0,136643983 | 0,308491678 | |
| 218334_at | THOC7 | 4,541443681 | 4,23300737 | 0,308436311 | |
| 212242_at | TUBA4A | 1,348276312 | 1,039887232 | 0,30838908 | |
| 204578_at | PPIP5K1 | 0,417596971 | 0,109221392 | 0,308375578 | |
| 239815_at | - | 0,417596971 | 0,109221392 | 0,308375578 | |
| 240279_at | - | 0,417596971 | 0,109221392 | 0,308375578 | |
| 225903_at | PIGU | 3,03990821 | 2,731588328 | 0,308319882 | |
| 208698_s_at | NONO | 5,171253382 | 4,862972786 | 0,308280597 | |
| 212446_s_at | CERS6 | 3,288503211 | 2,980244813 | 0,308258398 | |
| 226501_at | XPNPEP3 | 3,010782559 | 2,702564916 | 0,308217643 | |
| 209365_s_at | ECM1 | 1,485482016 | 1,177496821 | 0,307985195 | |
| 222093_s_at | INO80B | 1,485482016 | 1,177496821 | 0,307985195 | |
| 240199_x_at | ZNF345 | 0,911589958 | 0,603679647 | 0,307910312 | |
| 201048_x_at | RAB6A | 1,063722698 | 0,755840599 | 0,307882098 | |
| 220538_at | ADM2 | 1,201332287 | 0,893473268 | 0,307859019 | |
| 232146_at | NDUFC1 | 1,201332287 | 0,893473268 | 0,307859019 | |
| 207876_s_at | FLNC | 2,44295215 | 2,135226207 | 0,307725942 | |
| 220608_s_at | - | 0,46522187 | 0,157549243 | 0,307672627 | |
| 1552578_a_at | MYO3B | 0,228146222 | -0,079522948 | 0,30766917 | |
| 1555536_at | ANTXR2 | 0,228146222 | -0,079522948 | 0,30766917 | |
| 240398_at | - | 0,228146222 | -0,079522948 | 0,30766917 | |
| 205641_s_at | TRADD | 1,880387527 | 1,572742379 | 0,307645148 | |
| 214677_x_at | IGLC1 | -0,423248115 | -0,730778808 | 0,307530693 | |
| 223653_x_at | CELF4 | -0,423248115 | -0,730778808 | 0,307530693 | |
| 226061_s_at | NUDT3 | -0,423248115 | -0,730778808 | 0,307530693 | |
| 234023_s_at | CENPJ | -0,423248115 | -0,730778808 | 0,307530693 | |
| 239906_at | - | -0,423248115 | -0,730778808 | 0,307530693 | |
| 240145_at | - | -0,423248115 | -0,730778808 | 0,307530693 | |

| | | | | | |
|---|---|---|---|---|---|
| 240479_at | HS3ST5 | -0,423248115 | -0,730778808 | 0,307530693 | |
| 241393_at | IPP | -0,423248115 | -0,730778808 | 0,307530693 | |
| 241841_at | - | -0,423248115 | -0,730778808 | 0,307530693 | |
| 242229_at | NAPEPLD | -0,423248115 | -0,730778808 | 0,307530693 | |
| 209996_x_at | PCM1 | 1,094335495 | 0,786897721 | 0,307437773 | |
| 206020_at | SOCS6 | 0,945567441 | 0,638150376 | 0,307417065 | |
| 225384_at | DOCK7 | 0,945567441 | 0,638150376 | 0,307417065 | |
| 226914_at | ARPC5L | 2,621710292 | 2,314300277 | 0,307410014 | |
| 212043_at | TGOLN2 | 5,01267994 | 4,705281859 | 0,307398081 | |
| 227991_x_at | ZBTB43 | 1,814495998 | 1,507116528 | 0,307379471 | |
| 208118_x_at | LOC100271836 / | 1,631786706 | 1,324440845 | 0,307345861 | |
| 214395_x_at | EEF1D | 1,530951994 | 1,223626587 | 0,307325406 | |
| 1557017_at | - | -3,250025915 | -3,557342128 | 0,307316213 | |
| 200686_s_at | SRSF11 | 5,936622427 | 5,629398466 | 0,30722396 | |
| 1554385_a_at | PADI2 | -0,259832361 | -0,566887459 | 0,307055098 | |
| 210300_at | REM1 | -0,259832361 | -0,566887459 | 0,307055098 | |
| 214187_x_at | CTDSPL | -0,259832361 | -0,566887459 | 0,307055098 | |
| 226266_at | PGS1 | -0,259832361 | -0,566887459 | 0,307055098 | |
| 230044_at | PCYT2 | -0,259832361 | -0,566887459 | 0,307055098 | |
| 233696_at | - | -0,259832361 | -0,566887459 | 0,307055098 | |
| 241845_at | - | -0,259832361 | -0,566887459 | 0,307055098 | |
| 244254_at | - | -0,259832361 | -0,566887459 | 0,307055098 | |
| 206113_s_at | RAB5A | 2,700102307 | 2,39311332 | 0,306988987 | |
| 1564069_at | HOTTIP | -0,512251684 | -0,819238336 | 0,306986652 | |
| 201911_s_at | FARP1 | -0,512251684 | -0,819238336 | 0,306986652 | |
| 212763_at | CAMSAP2 | -0,512251684 | -0,819238336 | 0,306986652 | |
| 220439_at | RIN3 | -0,512251684 | -0,819238336 | 0,306986652 | |
| 221930_at | PHF7 | -0,512251684 | -0,819238336 | 0,306986652 | |
| 235558_at | RBMS2 | -0,512251684 | -0,819238336 | 0,306986652 | |
| 237251_at | LRRC71 | -0,512251684 | -0,819238336 | 0,306986652 | |
| 1557788_a_at | LINC00476 | -1,308181863 | -1,615132408 | 0,306950545 | |
| 1565758_at | - | -1,308181863 | -1,615132408 | 0,306950545 | |
| 1565949_x_at | CHML | -1,308181863 | -1,615132408 | 0,306950545 | |
| 1566837_at | - | -1,308181863 | -1,615132408 | 0,306950545 | |
| 1569243_at | - | -1,308181863 | -1,615132408 | 0,306950545 | |
| 205368_at | FAM131B | -1,308181863 | -1,615132408 | 0,306950545 | |
| 210394_x_at | SSX4 /// SSX4B | -1,308181863 | -1,615132408 | 0,306950545 | |
| 210549_s_at | CCL23 | -1,308181863 | -1,615132408 | 0,306950545 | |
| 216165_at | - | -1,308181863 | -1,615132408 | 0,306950545 | |
| 222174_at | - | -1,308181863 | -1,615132408 | 0,306950545 | |
| 223924_at | TTC25 | -1,308181863 | -1,615132408 | 0,306950545 | |
| 226830_x_at | - | -1,308181863 | -1,615132408 | 0,306950545 | |
| 227450_at | ERP27 | -1,308181863 | -1,615132408 | 0,306950545 | |
| 232093_at | LINC00085 | -1,308181863 | -1,615132408 | 0,306950545 | |
| 232222_at | C18orf49 | -1,308181863 | -1,615132408 | 0,306950545 | |
| 232330_at | C7orf44 | -1,308181863 | -1,615132408 | 0,306950545 | |
| 233790_at | - | -1,308181863 | -1,615132408 | 0,306950545 | |
| 234249_at | - | -1,308181863 | -1,615132408 | 0,306950545 | |
| 235392_at | - | -1,308181863 | -1,615132408 | 0,306950545 | |
| 236408_at | - | -1,308181863 | -1,615132408 | 0,306950545 | |
| 239360_at | - | -1,308181863 | -1,615132408 | 0,306950545 | |
| 242601_at | HEPACAM2 | -1,308181863 | -1,615132408 | 0,306950545 | |
| 243941_at | - | -1,308181863 | -1,615132408 | 0,306950545 | |
| 205899_at | CCNA1 | 2,009141686 | 1,702196344 | 0,306945342 | |
| 222870_s_at | B3GNT2 | 4,378950369 | 4,072129637 | 0,306820731 | |
| 209731_at | NTHL1 | 2,097427503 | 1,790660532 | 0,306766971 | |

| | | | | | |
|---|---|---|---|---|---|
| 209064_x_at | PAIP1 | 3,132544588 | 2,825817328 | 0,30672726 | |
| 204408_at | APEX2 | 3,831009889 | 3,524430539 | 0,30657935 | |
| 35974_at | LRMP | 0,449520941 | 0,143220067 | 0,306300874 | |
| 204034_at | ETHE1 | 2,525769574 | 2,21959457 | 0,306175004 | |
| 227334_at | USP54 | 1,767779954 | 1,461646549 | 0,306133404 | |
| 212753_at | PCGF3 | 2,472384965 | 2,166264721 | 0,306120244 | |
| 1553387_at | ATM | -2,384869671 | -2,690860744 | 0,305991073 | |
| 1555299_s_at | ERVW-1 | -2,384869671 | -2,690860744 | 0,305991073 | |
| 1556521_a_at | - | -2,384869671 | -2,690860744 | 0,305991073 | |
| 1559044_at | EXOSC1 | -2,384869671 | -2,690860744 | 0,305991073 | |
| 1559336_at | - | -2,384869671 | -2,690860744 | 0,305991073 | |
| 1560705_at | - | -2,384869671 | -2,690860744 | 0,305991073 | |
| 1562011_at | LOC100506413 | -2,384869671 | -2,690860744 | 0,305991073 | |
| 1563136_at | - | -2,384869671 | -2,690860744 | 0,305991073 | |
| 1568732_at | - | -2,384869671 | -2,690860744 | 0,305991073 | |
| 217532_x_at | - | -2,384869671 | -2,690860744 | 0,305991073 | |
| 229440_at | RBM47 | -2,384869671 | -2,690860744 | 0,305991073 | |
| 241198_s_at | C11orf70 | -2,384869671 | -2,690860744 | 0,305991073 | |
| 241915_at | ACSM2B | -2,384869671 | -2,690860744 | 0,305991073 | |
| 207833_s_at | HLCS | 1,210676994 | 0,904843258 | 0,305833736 | |
| 225112_at | ABI2 | 2,609398637 | 2,303602088 | 0,30579655 | |
| 223492_s_at | LRRFIP1 | -0,112808516 | -0,41858459 | 0,305776073 | |
| 219247_s_at | ZDHHC14 | 1,073999308 | 0,768343791 | 0,305655518 | |
| 235339_at | SETDB2 | 1,073999308 | 0,768343791 | 0,305655518 | |
| 211681_s_at | PDLIM5 | 1,805273023 | 1,499637023 | 0,305636001 | |
| 230250_at | PTPRB | 0,822971223 | 0,51742585 | 0,305545373 | |
| 212975_at | DENND3 | 0,384950573 | 0,079428135 | 0,305522438 | |
| 229562_at | RPL10A | 0,384950573 | 0,079428135 | 0,305522438 | |
| 231182_at | WIPF1 | 0,384950573 | 0,079428135 | 0,305522438 | |
| 232437_at | CPSF3L | 0,384950573 | 0,079428135 | 0,305522438 | |
| 1556850_at | CEP290 | -1,026571149 | -1,332017329 | 0,30544618 | |
| 209354_at | TNFRSF14 | -1,026571149 | -1,332017329 | 0,30544618 | |
| 211485_s_at | FGF18 | -1,026571149 | -1,332017329 | 0,30544618 | |
| 213014_at | LOC644172 /// M | -1,026571149 | -1,332017329 | 0,30544618 | |
| 214276_at | KLF12 | -1,026571149 | -1,332017329 | 0,30544618 | |
| 231192_at | LPAR3 | -1,026571149 | -1,332017329 | 0,30544618 | |
| 234631_at | KRTAP4-8 | -1,026571149 | -1,332017329 | 0,30544618 | |
| 234818_at | TMEM108 | -1,026571149 | -1,332017329 | 0,30544618 | |
| 239643_at | LOC100129516 | -1,026571149 | -1,332017329 | 0,30544618 | |
| 242883_at | OTOS | -1,026571149 | -1,332017329 | 0,30544618 | |
| 243719_at | STK19 | -1,026571149 | -1,332017329 | 0,30544618 | |
| 1561094_a_at | SLC22A25 | -2,930741289 | -3,236070201 | 0,305328912 | |
| 1562287_at | SNTG1 | -2,930741289 | -3,236070201 | 0,305328912 | |
| 1562616_at | - | -2,930741289 | -3,236070201 | 0,305328912 | |
| 232980_at | LMBRD1 | -2,930741289 | -3,236070201 | 0,305328912 | |
| 234244_at | RPF1 | -2,930741289 | -3,236070201 | 0,305328912 | |
| 235144_at | RASEF | -2,930741289 | -3,236070201 | 0,305328912 | |
| 236089_at | - | -2,930741289 | -3,236070201 | 0,305328912 | |
| 200884_at | CKB | 4,274216313 | 3,968932193 | 0,30528412 | |
| 204159_at | CDKN2C | 2,47626436 | 2,170981159 | 0,305283201 | |
| 209305_s_at | GADD45B | 0,641402394 | 0,336136178 | 0,305266217 | |
| 223114_at | COQ5 | 3,076146581 | 2,770953871 | 0,30519271 | |
| 227600_at | MRPS30 | 0,859074518 | 0,553993624 | 0,305080894 | |
| 224694_at | ANTXR1 | 2,37600538 | 2,071071048 | 0,304934332 | |
| 202752_x_at | SLC7A8 | 1,542099044 | 1,23718272 | 0,304916324 | |
| 213054_at | HAUS5 | 1,542099044 | 1,23718272 | 0,304916324 | |

| | | | | | |
|---|---|---|---|---|---|
| 204123_at | LIG3 | 1,292162421 | 0,987376598 | 0,304785823 | |
| 202514_at | DLG1 | 1,774096855 | 1,469325098 | 0,304771757 | |
| 212074_at | SUN1 | 3,58914411 | 3,284389368 | 0,304754742 | |
| 213293_s_at | TRIM22 | 0,682279745 | 0,377530647 | 0,304749099 | |
| 1555815_a_at | L3MBTL2 | 1,434549838 | 1,129843209 | 0,304706629 | |
| 1560713_a_at | ELFN2 | 0,020670649 | -0,283667828 | 0,304338477 | |
| 219218_at | BAHCC1 | 0,020670649 | -0,283667828 | 0,304338477 | |
| 231752_at | NPBWR1 | 0,020670649 | -0,283667828 | 0,304338477 | |
| 241852_at | - | 0,020670649 | -0,283667828 | 0,304338477 | |
| 243004_at | - | 0,020670649 | -0,283667828 | 0,304338477 | |
| 1559501_at | CBR3-AS1 | 0,480753764 | 0,176436073 | 0,30431769 | |
| 204579_at | FGFR4 | 0,480753764 | 0,176436073 | 0,30431769 | |
| 218879_s_at | MTHFSD | 0,480753764 | 0,176436073 | 0,30431769 | |
| 223661_at | NUCKS1 | 0,480753764 | 0,176436073 | 0,30431769 | |
| 202347_s_at | UBE2K | 4,651470922 | 4,347322916 | 0,304148006 | |
| 209369_at | ANXA3 | 1,722769813 | 1,418668082 | 0,304101731 | |
| 1557192_at | - | -0,85085127 | -1,154897679 | 0,304046409 | |
| 1560255_at | C10orf31 | -0,85085127 | -1,154897679 | 0,304046409 | |
| 203317_at | PSD4 | -0,85085127 | -1,154897679 | 0,304046409 | |
| 204322_at | GOLIM4 | -0,85085127 | -1,154897679 | 0,304046409 | |
| 205028_at | TRO | -0,85085127 | -1,154897679 | 0,304046409 | |
| 205990_s_at | WNT5A | -0,85085127 | -1,154897679 | 0,304046409 | |
| 210298_x_at | FHL1 | -0,85085127 | -1,154897679 | 0,304046409 | |
| 217685_at | SLC16A3 | -0,85085127 | -1,154897679 | 0,304046409 | |
| 230996_at | LPP | -0,85085127 | -1,154897679 | 0,304046409 | |
| 235402_at | PPP1R32 | -0,85085127 | -1,154897679 | 0,304046409 | |
| 241450_at | RSPO1 | -0,85085127 | -1,154897679 | 0,304046409 | |
| 1554082_a_at | NOL9 | 2,344516784 | 2,040470494 | 0,30404629 | |
| 52255_s_at | COL5A3 | 0,295565668 | -0,008476122 | 0,30404179 | |
| 229815_at | TMEM161B-AS1 | 1,504602299 | 1,200746069 | 0,30385623 | |
| 227222_at | FBXO10 | 0,962260738 | 0,658444279 | 0,303816459 | |
| 227296_at | MFSD3 | 0,962260738 | 0,658444279 | 0,303816459 | |
| 223041_at | CD99L2 | 0,760715727 | 0,456918297 | 0,303797429 | |
| 225774_at | RSPRY1 | 2,796341835 | 2,492544876 | 0,303796959 | |
| 227665_at | - | 2,369762403 | 2,066015817 | 0,303746586 | |
| 213857_s_at | CD47 | 5,401369722 | 5,097659882 | 0,30370984 | |
| 1552348_at | PRSS33 | 0,083054335 | -0,220592223 | 0,303646558 | |
| 1562890_at | - | 0,083054335 | -0,220592223 | 0,303646558 | |
| 231071_at | - | 0,083054335 | -0,220592223 | 0,303646558 | |
| 235026_at | C12orf66 | 0,083054335 | -0,220592223 | 0,303646558 | |
| 233748_x_at | PRKAG2 | 1,394109935 | 1,090553537 | 0,303556398 | |
| 219905_at | ERMAP | 1,168140757 | 0,864649967 | 0,30349079 | |
| 53202_at | C7orf25 /// PSMA | -1,583691226 | -1,887156996 | 0,30346577 | |
| 1559920_a_at | CECR5-AS1 | -2,43257096 | -2,735862426 | 0,303291466 | |
| 1562316_at | - | -2,43257096 | -2,735862426 | 0,303291466 | |
| 1569354_at | - | -2,43257096 | -2,735862426 | 0,303291466 | |
| 204052_s_at | SFRP4 | -2,43257096 | -2,735862426 | 0,303291466 | |
| 206757_at | PDE5A | -2,43257096 | -2,735862426 | 0,303291466 | |
| 215589_at | - | -2,43257096 | -2,735862426 | 0,303291466 | |
| 217431_x_at | CYBB | -2,43257096 | -2,735862426 | 0,303291466 | |
| 237588_at | - | -2,43257096 | -2,735862426 | 0,303291466 | |
| 239819_at | - | -2,43257096 | -2,735862426 | 0,303291466 | |
| 241075_at | RIMKLA | -2,43257096 | -2,735862426 | 0,303291466 | |
| 241401_at | WDFY3-AS2 | -2,43257096 | -2,735862426 | 0,303291466 | |
| 212220_at | PSME4 | 0,57058931 | 0,26733076 | 0,303258551 | |
| 1555065_x_at | USP6 | -0,706943342 | -1,010192375 | 0,303249033 | |

| | | | | | |
|---|---|---|---|---|---|
| 1559237_a_at | LOC100507108 | -0,706943342 | -1,010192375 | 0,303249033 | |
| 209466_x_at | LOC100287705 / | -0,706943342 | -1,010192375 | 0,303249033 | |
| 213381_at | VSTM4 | -0,706943342 | -1,010192375 | 0,303249033 | |
| 220446_s_at | CHST4 | -0,706943342 | -1,010192375 | 0,303249033 | |
| 222337_at | - | -0,706943342 | -1,010192375 | 0,303249033 | |
| 225020_at | DAB2IP | -0,706943342 | -1,010192375 | 0,303249033 | |
| 226695_at | PRRX1 | -0,706943342 | -1,010192375 | 0,303249033 | |
| 233641_s_at | FAM167A | -0,706943342 | -1,010192375 | 0,303249033 | |
| 239430_at | IGFL1 | -0,706943342 | -1,010192375 | 0,303249033 | |
| 227693_at | WDR20 | 2,182996494 | 1,879785595 | 0,303210899 | |
| 222620_s_at | DNAJC1 | 2,745814053 | 2,442710777 | 0,303103276 | |
| 235005_at | DIS3L | 1,917954284 | 1,614891062 | 0,303063223 | |
| 209609_s_at | MRPL9 | 4,864780677 | 4,56182315 | 0,302957527 | |
| 241632_x_at | - | 1,224581536 | 0,921731975 | 0,30284956 | |
| 236550_s_at | ZNF311 | 0,613492853 | 0,310717132 | 0,302775721 | |
| 204505_s_at | EPB49 | 1,251994932 | 0,949447784 | 0,302547149 | |
| 234304_s_at | IPO11 | 3,265148651 | 2,962671751 | 0,3024769 | |
| 202905_x_at | NBN | 2,522021211 | 2,21959457 | 0,302426641 | |
| 214352_s_at | KRAS | 0,20027154 | -0,102100538 | 0,302372077 | |
| 230748_at | SLC16A6 | 0,20027154 | -0,102100538 | 0,302372077 | |
| 217249_x_at | - | 4,959281929 | 4,656929558 | 0,302352371 | |
| 222228_s_at | ALKBH4 | 1,119359127 | 0,817300337 | 0,302058791 | |
| 222682_s_at | FAM114A2 | 2,201862497 | 1,899812471 | 0,302050026 | |
| 205407_at | RECK | 2,41290634 | 2,110887591 | 0,302018749 | |
| 230737_s_at | LOC387647 | 0,695652136 | 0,393761504 | 0,301890632 | |
| 242273_at | - | 0,695652136 | 0,393761504 | 0,301890632 | |
| 1556669_a_at | - | -0,234278768 | -0,536087151 | 0,301808383 | |
| 212823_s_at | PLEKHG3 | -0,234278768 | -0,536087151 | 0,301808383 | |
| 222890_at | CCDC113 | -0,234278768 | -0,536087151 | 0,301808383 | |
| 227136_s_at | C10orf46 | -0,234278768 | -0,536087151 | 0,301808383 | |
| 228958_at | ZNF19 | -0,234278768 | -0,536087151 | 0,301808383 | |
| 208382_s_at | DMC1 | 0,255492549 | -0,046302147 | 0,301794696 | |
| 211588_s_at | PML | 0,255492549 | -0,046302147 | 0,301794696 | |
| 214036_at | EFNA5 | 0,255492549 | -0,046302147 | 0,301794696 | |
| 1553053_at | LINC00521 | -1,240169874 | -1,541908042 | 0,301738168 | |
| 1553465_a_at | CES5A | -1,240169874 | -1,541908042 | 0,301738168 | |
| 1565612_at | DYNLRB1 | -1,240169874 | -1,541908042 | 0,301738168 | |
| 203070_at | SEMA3B | -1,240169874 | -1,541908042 | 0,301738168 | |
| 206084_at | PTPRR | -1,240169874 | -1,541908042 | 0,301738168 | |
| 219925_at | ZMYM6 | -1,240169874 | -1,541908042 | 0,301738168 | |
| 221015_s_at | CDADC1 | -1,240169874 | -1,541908042 | 0,301738168 | |
| 223646_s_at | TXLNG2P | -1,240169874 | -1,541908042 | 0,301738168 | |
| 224239_at | DEFB103A /// DE | -1,240169874 | -1,541908042 | 0,301738168 | |
| 228591_at | TNRC6C | -1,240169874 | -1,541908042 | 0,301738168 | |
| 230478_at | OIT3 | -1,240169874 | -1,541908042 | 0,301738168 | |
| 233022_at | TRPM3 | -1,240169874 | -1,541908042 | 0,301738168 | |
| 235672_at | MAP6 | -1,240169874 | -1,541908042 | 0,301738168 | |
| 238360_s_at | LOC100505576 | -1,240169874 | -1,541908042 | 0,301738168 | |
| 209338_at | TFCP2 | 2,724770722 | 2,423075926 | 0,301694796 | |
| 37232_at | KIAA0586 | 1,163336153 | 0,86173571 | 0,301600443 | |
| 239441_at | ZNF780A | 0,97328325 | 0,67181667 | 0,30146658 | |
| 223431_at | CNO | 3,164152611 | 2,862725455 | 0,301427156 | |
| 1556026_at | LOC100131434 | -2,972500237 | -3,273861381 | 0,301361145 | |
| 214282_at | - | -2,972500237 | -3,273861381 | 0,301361145 | |
| 238695_s_at | RAB39B | -2,972500237 | -3,273861381 | 0,301361145 | |
| 244773_at | - | -2,972500237 | -3,273861381 | 0,301361145 | |

| | | | | | |
|---|---|---|---|---|---|
| 202602_s_at | HTATSF1 | 4,008926642 | 3,707626508 | 0,301300133 | |
| 234975_at | GSPT1 | 0,30867765 | 0,007421914 | 0,301255736 | |
| 1553048_a_at | PIP4K2B | 1,381753566 | 1,080561626 | 0,301191941 | |
| 221257_x_at | FBXO38 | 2,943717636 | 2,64270545 | 0,301012186 | |
| 212245_at | MCFD2 | 4,446258267 | 4,145254615 | 0,301003652 | |
| 1560059_at | VPS37C | -0,089689931 | -0,39061235 | 0,300922419 | |
| 209327_s_at | NOP16 | -0,089689931 | -0,39061235 | 0,300922419 | |
| 215233_at | JMJD6 | -0,089689931 | -0,39061235 | 0,300922419 | |
| 215827_x_at | CROCCP3 | -0,089689931 | -0,39061235 | 0,300922419 | |
| 223806_s_at | NAPSA | -0,089689931 | -0,39061235 | 0,300922419 | |
| 227781_x_at | FAM57B | -0,089689931 | -0,39061235 | 0,300922419 | |
| 236784_s_at | - | -0,089689931 | -0,39061235 | 0,300922419 | |
| 243900_at | WDR38 | -0,089689931 | -0,39061235 | 0,300922419 | |
| 224684_at | SNX12 | 3,316904202 | 3,016072743 | 0,300831458 | |
| 206275_s_at | MICAL2 | 0,359971644 | 0,059218869 | 0,300752775 | |
| 214969_at | MAP3K9 | 0,359971644 | 0,059218869 | 0,300752775 | |
| 225395_s_at | FAM120AOS | 3,497632487 | 3,196911138 | 0,300721349 | |
| 231780_at | GBGT1 | 3,111496958 | 2,810785576 | 0,300711382 | |
| 1552590_a_at | ABCC12 | -2,479842156 | -2,780313924 | 0,300471768 | |
| 1560714_at | FLJ37035 | -2,479842156 | -2,780313924 | 0,300471768 | |
| 1561654_at | - | -2,479842156 | -2,780313924 | 0,300471768 | |
| 1561690_at | - | -2,479842156 | -2,780313924 | 0,300471768 | |
| 1563475_s_at | METTL20 | -2,479842156 | -2,780313924 | 0,300471768 | |
| 1564662_at | ZNF852 | -2,479842156 | -2,780313924 | 0,300471768 | |
| 1569100_a_at | - | -2,479842156 | -2,780313924 | 0,300471768 | |
| 1569410_at | FLG2 | -2,479842156 | -2,780313924 | 0,300471768 | |
| 204941_s_at | ALDH3B2 | -2,479842156 | -2,780313924 | 0,300471768 | |
| 216045_at | CCDC144A | -2,479842156 | -2,780313924 | 0,300471768 | |
| 216612_x_at | - | -2,479842156 | -2,780313924 | 0,300471768 | |
| 231703_s_at | ADH4 | -2,479842156 | -2,780313924 | 0,300471768 | |
| 233397_at | - | -2,479842156 | -2,780313924 | 0,300471768 | |
| 236597_at | UGT3A1 | -2,479842156 | -2,780313924 | 0,300471768 | |
| 241404_at | - | -2,479842156 | -2,780313924 | 0,300471768 | |
| 242163_at | THRAP3 | -2,479842156 | -2,780313924 | 0,300471768 | |
| 203287_at | LAD1 | -0,022466681 | -0,322870281 | 0,3004036 | |
| 207868_at | CHRNA2 | -0,022466681 | -0,322870281 | 0,3004036 | |
| 215234_at | - | -0,022466681 | -0,322870281 | 0,3004036 | |
| 219830_at | RAI1 | -0,022466681 | -0,322870281 | 0,3004036 | |
| 234977_at | ZADH2 | -0,022466681 | -0,322870281 | 0,3004036 | |
| 202811_at | STAMBP | 3,702775533 | 3,402394519 | 0,300381014 | |
| 209330_s_at | HNRNPD | 5,394467196 | 5,094094626 | 0,30037257 | |
| 218017_s_at | HGSNAT | 2,478200152 | 2,17802703 | 0,300173122 | |
| 37566_at | KIAA1045 | -2,377296877 | -2,677460985 | 0,300164109 | |
| 201679_at | SRRT | 1,314003737 | 1,013870809 | 0,300132928 | |
| 230379_x_at | C2orf56 | 1,339784194 | 1,039887232 | 0,299896963 | |
| 1558784_at | LOC100133089 | 1,523472489 | 1,223626587 | 0,299845902 | |
| 205127_at | PTGS1 | 0,457392764 | 0,157549243 | 0,299843521 | |
| 209105_at | NCOA1 | 0,457392764 | 0,157549243 | 0,299843521 | |
| 212134_at | PHLDB1 | 0,457392764 | 0,157549243 | 0,299843521 | |
| 230108_at | ERCC6 | 0,457392764 | 0,157549243 | 0,299843521 | |
| 204496_at | STRN3 | 4,232392431 | 3,932593347 | 0,299799084 | |
| 228191_at | FLVCR1 | 3,179700672 | 2,880111483 | 0,299589189 | |
| 201477_s_at | RRM1 | 5,482445879 | 5,182875656 | 0,299570224 | |
| 213264_at | PCBP2 | 1,649074067 | 1,349621332 | 0,299452735 | |
| 205540_s_at | RRAGB | 1,390002893 | 1,090553537 | 0,299449356 | |
| 235618_at | ZNF507 | 1,390002893 | 1,090553537 | 0,299449356 | |

| | | | | | |
|---|---|---|---|---|---|
| 1552717_s_at | CEP170 /// CEP1 | 0,503742507 | 0,204310755 | 0,299431752 | |
| 1562484_at | C17orf104 | 0,503742507 | 0,204310755 | 0,299431752 | |
| 204360_s_at | NAGLU | 0,503742507 | 0,204310755 | 0,299431752 | |
| 237386_at | - | 0,503742507 | 0,204310755 | 0,299431752 | |
| 1555837_s_at | POLR2B | 6,74745965 | 6,448050962 | 0,299408688 | |
| 226508_at | PHC3 | 3,279650757 | 2,980244813 | 0,299405945 | |
| 1556429_a_at | WDR67 | 0,103263601 | -0,19610998 | 0,299373581 | |
| 222112_at | EPS15L1 | 0,103263601 | -0,19610998 | 0,299373581 | |
| 243883_at | MMP15 | 0,103263601 | -0,19610998 | 0,299373581 | |
| 244504_x_at | ARF1 /// MIR362( | 0,103263601 | -0,19610998 | 0,299373581 | |
| 228717_at | - | 1,104397092 | 0,805216064 | 0,299181028 | |
| 222958_s_at | DEPDC1 | 3,154500615 | 2,855418981 | 0,299081635 | |
| 1568619_s_at | ITPRIPL2 | 1,709647566 | 1,410714371 | 0,298933194 | |
| 218772_x_at | TMEM38B | 3,957816838 | 3,658934427 | 0,298882411 | |
| 225539_at | ZNF295 | 3,120200849 | 2,821324225 | 0,298876624 | |
| 236834_at | SCFD2 | 0,888485433 | 0,589657387 | 0,298828047 | |
| 202127_at | PRPF4B | 3,517535002 | 3,218713651 | 0,298821351 | |
| 232122_s_at | VEPH1 | 1,163336153 | 0,864649967 | 0,298686186 | |
| 226376_at | UNK | 0,592200562 | 0,293518836 | 0,298681726 | |
| 215492_x_at | PTCRA | 1,593009234 | 1,294497133 | 0,298512101 | |
| 219189_at | FBXL6 | 1,191926657 | 0,893473268 | 0,298453389 | |
| 1554987_at | GOLGA3 | -0,467091869 | -0,765504029 | 0,29841216 | |
| 210452_x_at | CYP4F2 /// CYP4 | -0,467091869 | -0,765504029 | 0,29841216 | |
| 210497_x_at | SSX2 /// SSX2B | -0,467091869 | -0,765504029 | 0,29841216 | |
| 215511_at | TCF20 | -0,467091869 | -0,765504029 | 0,29841216 | |
| 221155_x_at | - | -0,467091869 | -0,765504029 | 0,29841216 | |
| 200993_at | IPO7 | 4,412743794 | 4,114368048 | 0,298375746 | |
| 224959_at | SLC26A2 | 4,60394751 | 4,305575871 | 0,298371639 | |
| 1553234_at | ADAMTS18 | -1,196375121 | -1,494668682 | 0,29829356 | |
| 1555804_a_at | YSK4 | -1,196375121 | -1,494668682 | 0,29829356 | |
| 1559292_s_at | LINC00032 | -1,196375121 | -1,494668682 | 0,29829356 | |
| 1559355_at | NXPH2 | -1,196375121 | -1,494668682 | 0,29829356 | |
| 1564679_at | ASB15 | -1,196375121 | -1,494668682 | 0,29829356 | |
| 206713_at | NTNG1 | -1,196375121 | -1,494668682 | 0,29829356 | |
| 206720_at | MGAT5 | -1,196375121 | -1,494668682 | 0,29829356 | |
| 213280_at | RAP1GAP2 | -1,196375121 | -1,494668682 | 0,29829356 | |
| 214691_x_at | FAM63B | -1,196375121 | -1,494668682 | 0,29829356 | |
| 215102_at | DPY19L1P1 | -1,196375121 | -1,494668682 | 0,29829356 | |
| 228421_s_at | EFEMP1 | -1,196375121 | -1,494668682 | 0,29829356 | |
| 229110_at | SLC24A2 | -1,196375121 | -1,494668682 | 0,29829356 | |
| 233117_at | MAPT | -1,196375121 | -1,494668682 | 0,29829356 | |
| 241337_at | - | -1,196375121 | -1,494668682 | 0,29829356 | |
| 242833_at | - | -1,196375121 | -1,494668682 | 0,29829356 | |
| 243238_at | PYGB | -1,196375121 | -1,494668682 | 0,29829356 | |
| 243598_at | - | -1,196375121 | -1,494668682 | 0,29829356 | |
| 214960_at | API5 | 1,442503549 | 1,144305291 | 0,298198258 | |
| 239888_at | - | 0,67554678 | 0,377530647 | 0,298016133 | |
| 210169_at | SEC14L5 | 1,46610492 | 1,16809119 | 0,29801373 | |
| 237404_at | - | 0,273439642 | -0,024572586 | 0,298012228 | |
| 228760_at | SRSF8 | 3,62627949 | 3,32832922 | 0,29795027 | |
| 57516_at | ZNF764 | 1,139070151 | 0,841169397 | 0,297900754 | |
| 34063_at | RECQL5 | -0,539157504 | -0,837050727 | 0,297893223 | |
| 226361_at | TMEM42 | 1,274448124 | 0,976641161 | 0,297806963 | |
| 215012_at | ZNF451 | 1,021868205 | 0,724100169 | 0,297768036 | |
| 219999_at | MAN2A2 | 1,021868205 | 0,724100169 | 0,297768036 | |
| 204549_at | IKBKE | 0,715481152 | 0,417770464 | 0,297710688 | |

| | | | | | |
|---|---|---|---|---|---|
| 224894_at | YAP1 | 0,715481152 | 0,417770464 | 0,297710688 | |
| 238142_at | - | 0,715481152 | 0,417770464 | 0,297710688 | |
| 1567912_s_at | CT45A1 /// CT45/ | -3,013665402 | -3,311072652 | 0,29740725 | |
| 212653_s_at | EHBP1 | 3,308224834 | 3,010820719 | 0,297404115 | |
| 244030_at | STYX | 2,64946283 | 2,352169913 | 0,297292917 | |
| 1569775_at | RNF157 | 0,376672223 | 0,079428135 | 0,297244088 | |
| 210200_at | WWP2 | 0,376672223 | 0,079428135 | 0,297244088 | |
| 212705_x_at | PNPLA2 | 0,376672223 | 0,079428135 | 0,297244088 | |
| 226902_at | USP13 | 0,376672223 | 0,079428135 | 0,297244088 | |
| 232774_x_at | ZIK1 | 0,376672223 | 0,079428135 | 0,297244088 | |
| 232866_at | ZSCAN18 | 0,376672223 | 0,079428135 | 0,297244088 | |
| 202577_s_at | DDX19A | 2,277177578 | 1,979940779 | 0,2972368 | |
| 1555523_a_at | FYCO1 | 0,792179257 | 0,495031851 | 0,297147406 | |
| 216385_at | LOC220077 | 0,792179257 | 0,495031851 | 0,297147406 | |
| 211569_s_at | HADH | 1,946200473 | 1,64909809 | 0,297102382 | |
| 209357_at | CITED2 | 4,758484959 | 4,461446286 | 0,297038673 | |
| 218957_s_at | PAAF1 | 2,922548342 | 2,625627364 | 0,296920978 | |
| 207458_at | RHPN1-AS1 | 0,82905153 | 0,532164371 | 0,29688716 | |
| 212419_at | ZCCHC24 | 1,621313976 | 1,324440845 | 0,296873131 | |
| 228351_at | HEATR1 | 2,621710292 | 2,324919719 | 0,296790573 | |
| 204433_s_at | SPATA2 | 2,342392905 | 2,045615873 | 0,296777032 | |
| 210986_s_at | TPM1 | 4,622007457 | 4,32527877 | 0,296728687 | |
| 202058_s_at | KPNA1 | 2,794789337 | 2,498185745 | 0,296603593 | |
| 205609_at | ANGPT1 | 4,447740857 | 4,151234028 | 0,296506829 | |
| 225431_x_at | PM20D2 | 3,102740237 | 2,806245339 | 0,296494898 | |
| 201923_at | PRDX4 | 6,578365491 | 6,281882154 | 0,296483338 | |
| 223239_at | C14orf129 | 4,312693778 | 4,016221008 | 0,29647277 | |
| 1552573_s_at | MIPOL1 | 0,900083947 | 0,603679647 | 0,296404301 | |
| 218424_s_at | STEAP3 | 2,462640593 | 2,166264721 | 0,296375872 | |
| 219371_s_at | KLF2 | 2,132266459 | 1,835911187 | 0,296355273 | |
| 225178_at | TTC14 | 0,518867317 | 0,222599095 | 0,296268222 | |
| 230063_at | ZNF264 | 1,496984567 | 1,200746069 | 0,296238498 | |
| 227815_at | - | 2,294858597 | 1,998633167 | 0,29622543 | |
| 226345_at | ARL5B | 4,236401075 | 3,940211012 | 0,296190062 | |
| 222738_at | CLDN22 /// WW( | 0,934330293 | 0,638150376 | 0,296179916 | |
| 235192_at | TP53RK | 2,014487247 | 1,718396606 | 0,296090641 | |
| 1557172_x_at | NEK8 | 1,283332461 | 0,987376598 | 0,295955863 | |
| 215182_x_at | - | 1,296557217 | 1,000684521 | 0,295872696 | |
| 203732_at | TRIP4 | 3,45992606 | 3,164168347 | 0,295757713 | |
| 1553297_a_at | CSF3R | 0,606430281 | 0,310717132 | 0,295713149 | |
| 1553898_a_at | DKFZp434L192 | -2,551294263 | -2,847001814 | 0,295707551 | |
| 1554163_at | TWIST2 | -2,551294263 | -2,847001814 | 0,295707551 | |
| 1559393_at | ALDH1L2 | -2,551294263 | -2,847001814 | 0,295707551 | |
| 1565769_at | - | -2,551294263 | -2,847001814 | 0,295707551 | |
| 1568805_at | NCOA7 | -2,551294263 | -2,847001814 | 0,295707551 | |
| 1570364_at | ZNF709 | -2,551294263 | -2,847001814 | 0,295707551 | |
| 204719_at | ABCA8 | -2,551294263 | -2,847001814 | 0,295707551 | |
| 206765_at | KCNJ2 | -2,551294263 | -2,847001814 | 0,295707551 | |
| 209990_s_at | GABBR2 | -2,551294263 | -2,847001814 | 0,295707551 | |
| 232961_at | SPATS2L | -2,551294263 | -2,847001814 | 0,295707551 | |
| 237933_at | - | -2,551294263 | -2,847001814 | 0,295707551 | |
| 239292_at | - | -2,551294263 | -2,847001814 | 0,295707551 | |
| 240591_at | - | -2,551294263 | -2,847001814 | 0,295707551 | |
| 240741_x_at | - | -2,551294263 | -2,847001814 | 0,295707551 | |
| 241593_x_at | - | -2,551294263 | -2,847001814 | 0,295707551 | |
| 211985_s_at | CALM1 /// CALM: | 3,81731168 | 3,521663705 | 0,295647975 | |

| | | | | | |
|---|---|---|---|---|---|
| 205226_at | PDGFRL | 1,360921491 | 1,065442793 | 0,295478698 | |
| 223497_at | FAM135A | 2,348755185 | 2,053299695 | 0,29545549 | |
| 204956_at | MTAP | 1,063722698 | 0,768343791 | 0,295378907 | |
| 225644_at | CCDC117 | 4,049200824 | 3,753943043 | 0,29525778 | |
| 203312_x_at | ARF6 | 4,457095376 | 4,161934914 | 0,295160462 | |
| 217445_s_at | GART | 2,979690938 | 2,684536601 | 0,295154337 | |
| 200603_at | PRKAR1A | 5,512273061 | 5,217197809 | 0,295075252 | |
| 57703_at | SENP5 | 1,756658382 | 1,461646549 | 0,295011833 | |
| 225401_at | C1orf85 | 2,194816625 | 1,899812471 | 0,295004154 | |
| 235505_s_at | LRPAP1 | 0,728550685 | 0,433557298 | 0,294993387 | |
| 227548_at | ORMDL1 | 1,820612054 | 1,525647381 | 0,294964673 | |
| 219043_s_at | LOC285359 /// PI | 4,373240364 | 4,078419261 | 0,294821103 | |
| 206589_at | GFI1 | -0,655928373 | -0,950686014 | 0,294757641 | |
| 225442_at | DDR2 | -0,655928373 | -0,950686014 | 0,294757641 | |
| 233812_at | LINC00028 | -0,655928373 | -0,950686014 | 0,294757641 | |
| 237894_at | C3orf22 | -0,655928373 | -0,950686014 | 0,294757641 | |
| 219787_s_at | ECT2 | 3,243687542 | 2,949006836 | 0,294680706 | |
| 226897_s_at | ZC3H7A | 1,874520207 | 1,579853228 | 0,294666978 | |
| 209555_s_at | CD36 | 4,859592153 | 4,564962102 | 0,29463005 | |
| 202498_s_at | SLC2A3 | 4,632475172 | 4,337916639 | 0,294558533 | |
| 201777_s_at | KIAA0494 | 1,672933557 | 1,378453643 | 0,294479913 | |
| 215108_x_at | TOX3 | -3,212234735 | -3,506469152 | 0,294234418 | |
| 205066_s_at | ENPP1 | -0,196756757 | -0,490927335 | 0,294170578 | |
| 206394_at | MYBPC2 | -0,196756757 | -0,490927335 | 0,294170578 | |
| 210828_s_at | ARNT | -0,196756757 | -0,490927335 | 0,294170578 | |
| 211175_at | GPR45 | -0,196756757 | -0,490927335 | 0,294170578 | |
| 212935_at | MCF2L | -0,196756757 | -0,490927335 | 0,294170578 | |
| 215910_s_at | FNDC3A | -0,196756757 | -0,490927335 | 0,294170578 | |
| 230980_x_at | - | -0,196756757 | -0,490927335 | 0,294170578 | |
| 241062_at | - | -0,196756757 | -0,490927335 | 0,294170578 | |
| 244088_at | - | -0,196756757 | -0,490927335 | 0,294170578 | |
| 244385_at | KDM4C | -0,196756757 | -0,490927335 | 0,294170578 | |
| 217092_x_at | - | 5,828105858 | 5,533999386 | 0,294106472 | |
| 1559806_at | - | -0,055687482 | -0,34958478 | 0,293897298 | |
| 229284_at | MAT2B | -0,055687482 | -0,34958478 | 0,293897298 | |
| 235163_at | MOB3A | -0,055687482 | -0,34958478 | 0,293897298 | |
| 228069_at | FAM54A | 4,54260042 | 4,248708657 | 0,293891762 | |
| 209726_at | CA11 | -0,352951799 | -0,646746079 | 0,29379428 | |
| 210642_at | CCIN | -0,352951799 | -0,646746079 | 0,29379428 | |
| 235664_at | - | -0,352951799 | -0,646746079 | 0,29379428 | |
| 241746_at | CUL7 | -0,352951799 | -0,646746079 | 0,29379428 | |
| 238778_at | MPP7 | 1,530951994 | 1,23718272 | 0,293769274 | |
| 202657_s_at | SERTAD2 | 2,159061673 | 1,865308541 | 0,293753132 | |
| 213826_s_at | H3F3A | 1,187200746 | 0,893473268 | 0,293727478 | |
| 216503_s_at | MLLT10 | 1,187200746 | 0,893473268 | 0,293727478 | |
| 219474_at | C3orf52 | 1,215326747 | 0,921731975 | 0,293594772 | |
| 212812_at | SERINC5 | 2,201862497 | 1,908311039 | 0,293551457 | |
| 1569192_at | - | -3,054236653 | -3,347710726 | 0,293474073 | |
| 240292_x_at | ANKS1B | -3,054236653 | -3,347710726 | 0,293474073 | |
| 241306_at | - | -3,054236653 | -3,347710726 | 0,293474073 | |
| 228843_at | ARL10 | 2,987866617 | 2,694398145 | 0,293468472 | |
| 214378_at | TFPI | 0,072842263 | -0,220592223 | 0,293434486 | |
| 230750_at | - | 0,072842263 | -0,220592223 | 0,293434486 | |
| 236483_at | - | 0,072842263 | -0,220592223 | 0,293434486 | |
| 217842_at | LUC7L2 | 4,188728817 | 3,895315489 | 0,293413328 | |
| 228710_at | - | 2,819431021 | 2,526063925 | 0,293367096 | |

| | | | | | |
|---|---|---|---|---|---|
| 1566823_a_at | - | -0,926850548 | -1,220210588 | 0,293360039 | |
| 1570503_at | - | -0,926850548 | -1,220210588 | 0,293360039 | |
| 204898_at | SAP30 | -0,926850548 | -1,220210588 | 0,293360039 | |
| 211089_s_at | NEK3 | -0,926850548 | -1,220210588 | 0,293360039 | |
| 216485_s_at | TPSAB1 | -0,926850548 | -1,220210588 | 0,293360039 | |
| 222828_at | IL20RA | -0,926850548 | -1,220210588 | 0,293360039 | |
| 230218_at | HIC1 | -0,926850548 | -1,220210588 | 0,293360039 | |
| 235597_s_at | RGPD1 /// RGPD | -0,926850548 | -1,220210588 | 0,293360039 | |
| 236954_at | BOLL | -0,926850548 | -1,220210588 | 0,293360039 | |
| 238132_at | LOC100505669 / | -0,926850548 | -1,220210588 | 0,293360039 | |
| 238641_at | C1orf126 | -0,926850548 | -1,220210588 | 0,293360039 | |
| 241229_at | - | -0,926850548 | -1,220210588 | 0,293360039 | |
| 241325_at | LOC100507408 | -0,926850548 | -1,220210588 | 0,293360039 | |
| 217987_at | ASNSD1 | 4,933763998 | 4,640445173 | 0,293318826 | |
| 212969_x_at | EML3 | 0,810733188 | 0,51742585 | 0,293307337 | |
| 1553678_a_at | ITGB1 | 4,431847465 | 4,138648509 | 0,293198955 | |
| 1559840_s_at | TBX18 | -0,43775896 | -0,730778808 | 0,293019848 | |
| 203742_s_at | LOC732360 /// TI | 2,11992033 | 1,826973888 | 0,292946442 | |
| 222543_at | DERL1 | 4,97067094 | 4,677791538 | 0,292879401 | |
| 202193_at | LIMK2 | 0,917308734 | 0,624460759 | 0,292847975 | |
| 213100_at | UNC5B | 0,917308734 | 0,624460759 | 0,292847975 | |
| 243606_at | NXPE3 | 0,917308734 | 0,624460759 | 0,292847975 | |
| 225333_at | ZNF496 | 0,246434561 | -0,046302147 | 0,292736708 | |
| 203177_x_at | TFAM | 3,579258128 | 3,28656411 | 0,292694018 | |
| 1554413_s_at | SNX29P1 /// SNX | -2,597496523 | -2,89013181 | 0,292635287 | |
| 1554922_at | ZNF678 | -2,597496523 | -2,89013181 | 0,292635287 | |
| 1557890_at | STXBP5-AS1 | -2,597496523 | -2,89013181 | 0,292635287 | |
| 1558963_at | - | -2,597496523 | -2,89013181 | 0,292635287 | |
| 1560464_at | TSPAN10 | -2,597496523 | -2,89013181 | 0,292635287 | |
| 1562209_at | DCAF4L1 | -2,597496523 | -2,89013181 | 0,292635287 | |
| 1564285_at | HFM1 | -2,597496523 | -2,89013181 | 0,292635287 | |
| 1568920_at | - | -2,597496523 | -2,89013181 | 0,292635287 | |
| 216459_x_at | DDR1-AS1 | -2,597496523 | -2,89013181 | 0,292635287 | |
| 241440_at | ZIC1 | -2,597496523 | -2,89013181 | 0,292635287 | |
| 242135_at | LOC100509302 | -2,597496523 | -2,89013181 | 0,292635287 | |
| 242964_at | - | -2,597496523 | -2,89013181 | 0,292635287 | |
| 238465_at | SETD9 | 2,691784869 | 2,39915583 | 0,292629039 | |
| 217693_x_at | MAGOH2 | 1,64218396 | 1,349621332 | 0,292562628 | |
| 208456_s_at | RRAS2 | 2,943717636 | 2,651169275 | 0,292548361 | |
| 212410_at | EFHA1 | 4,758484959 | 4,466010984 | 0,292473975 | |
| 205510_s_at | FLJ10038 | 0,351548291 | 0,059218869 | 0,292329422 | |
| 1553954_at | ALG14 | 1,515954005 | 1,223626587 | 0,292327418 | |
| 217416_x_at | - | 2,151803104 | 1,859476797 | 0,292326307 | |
| 1553675_at | KLC4 | -1,131062212 | -1,423348341 | 0,292286129 | |
| 1554443_s_at | BEST1 | -1,131062212 | -1,423348341 | 0,292286129 | |
| 1556657_at | - | -1,131062212 | -1,423348341 | 0,292286129 | |
| 1557727_at | PCBP1-AS1 | -1,131062212 | -1,423348341 | 0,292286129 | |
| 1558398_at | ANKRD24 | -1,131062212 | -1,423348341 | 0,292286129 | |
| 1562282_at | ZNF568 | -1,131062212 | -1,423348341 | 0,292286129 | |
| 204358_s_at | FLRT2 /// LOC10 | -1,131062212 | -1,423348341 | 0,292286129 | |
| 207510_at | BDKRB1 | -1,131062212 | -1,423348341 | 0,292286129 | |
| 223779_at | AFAP1-AS1 | -1,131062212 | -1,423348341 | 0,292286129 | |
| 224531_at | GPR61 | -1,131062212 | -1,423348341 | 0,292286129 | |
| 230284_at | MYOM3 | -1,131062212 | -1,423348341 | 0,292286129 | |
| 230586_s_at | - | -1,131062212 | -1,423348341 | 0,292286129 | |
| 233272_at | - | -1,131062212 | -1,423348341 | 0,292286129 | |

| | | | | | |
|---|---|---|---|---|---|
| 236400_at | IDH1-AS1 | -1,131062212 | -1,423348341 | 0,292286129 | |
| 239475_at | ACPL2 | -1,131062212 | -1,423348341 | 0,292286129 | |
| 217364_x_at | - | 1,722769813 | 1,43051703 | 0,292252783 | |
| 200080_s_at | H3F3A /// H3F3A | 7,375162476 | 7,082929608 | 0,292232869 | |
| 1564431_a_at | LCNL1 | 0,401366113 | 0,109221392 | 0,292144721 | |
| 232500_at | RALGAPA2 | 0,401366113 | 0,109221392 | 0,292144721 | |
| 213104_at | TSR3 | 2,250242994 | 1,958277216 | 0,291965778 | |
| 212215_at | PREPL | 3,420201053 | 3,128242026 | 0,291959026 | |
| 244768_at | DYNC1H1 | 0,496120222 | 0,204310755 | 0,291809467 | |
| 203826_s_at | PITPNM1 | 1,224581536 | 0,932882274 | 0,291699262 | |
| 218568_at | AGK | 3,391728033 | 3,100079607 | 0,291648427 | |
| 1563646_a_at | TMEM67 | 1,251994932 | 0,960386719 | 0,291608213 | |
| 212246_at | MCFD2 | 3,067171967 | 2,775606361 | 0,291565606 | |
| 225117_at | KANSL1 | 3,667581479 | 3,376085218 | 0,291496262 | |
| 228730_s_at | SCRN2 | 1,292162421 | 1,000684521 | 0,2914779 | |
| 203731_s_at | ZKSCAN5 | 1,305306849 | 1,013870809 | 0,29143604 | |
| 210048_at | NAPG | 1,305306849 | 1,013870809 | 0,29143604 | |
| 213936_x_at | SFTPB | 1,305306849 | 1,013870809 | 0,29143604 | |
| 243147_x_at | - | 2,127340679 | 1,835911187 | 0,291429492 | |
| 234512_x_at | - | 6,277510259 | 5,986125033 | 0,291385226 | |
| 227593_at | FLJ37453 | 1,331241794 | 1,039887232 | 0,291354562 | |
| 212918_at | RECQL | 3,356380711 | 3,065035915 | 0,291344796 | |
| 1556001_at | LOC284939 /// M | 1,607231019 | 1,315948728 | 0,291282291 | |
| 223391_at | SGPP1 | 3,794188277 | 3,503081281 | 0,291106996 | |
| 217820_s_at | ENAH | 3,054253462 | 2,763166224 | 0,291087238 | |
| 1563577_at | - | 0,747935635 | 0,456918297 | 0,291017338 | |
| 212638_s_at | WWP1 | 2,700102307 | 2,409170778 | 0,290931529 | |
| 1555270_a_at | WFS1 | -0,759588698 | -1,050406615 | 0,290817917 | |
| 1557373_at | LOC339505 | -0,759588698 | -1,050406615 | 0,290817917 | |
| 1557628_s_at | LOC283745 | -0,759588698 | -1,050406615 | 0,290817917 | |
| 1562433_at | FLJ10489 | -0,759588698 | -1,050406615 | 0,290817917 | |
| 205463_s_at | PDGFA | -0,759588698 | -1,050406615 | 0,290817917 | |
| 215251_at | - | -0,759588698 | -1,050406615 | 0,290817917 | |
| 228712_at | WNK1 | -0,759588698 | -1,050406615 | 0,290817917 | |
| 232900_at | HERC2P7 | -0,759588698 | -1,050406615 | 0,290817917 | |
| 232948_at | - | -0,759588698 | -1,050406615 | 0,290817917 | |
| 233582_at | - | -0,759588698 | -1,050406615 | 0,290817917 | |
| 240358_at | - | -0,759588698 | -1,050406615 | 0,290817917 | |
| 243219_x_at | MRPL50 | -0,759588698 | -1,050406615 | 0,290817917 | |
| 244041_at | STX6 | -0,759588698 | -1,050406615 | 0,290817917 | |
| 202898_at | SDC3 | 0,822971223 | 0,532164371 | 0,290806853 | |
| 212471_at | AVL9 | 0,928678725 | 0,638150376 | 0,290528348 | |
| 217230_at | EZR | 0,928678725 | 0,638150376 | 0,290528348 | |
| 212996_s_at | URB1 | 2,102456302 | 1,811954245 | 0,290502057 | |
| 204645_at | CCNT2 | 2,310153708 | 2,019703483 | 0,290450225 | |
| 203532_x_at | CUL5 | 0,962260738 | 0,67181667 | 0,290444068 | |
| 236044_at | PPAPDC1A | 3,474545391 | 3,184136378 | 0,290409013 | |
| 233085_s_at | NABP1 | 2,503131904 | 2,212749286 | 0,290382618 | |
| 218265_at | SECISBP2 | 0,995078803 | 0,704715219 | 0,290363584 | |
| 229721_x_at | DERL3 | 0,995078803 | 0,704715219 | 0,290363584 | |
| 229969_at | SEC63 | 0,995078803 | 0,704715219 | 0,290363584 | |
| 243259_at | ATXN7 | 0,995078803 | 0,704715219 | 0,290363584 | |
| 225378_at | VPS37A | 3,348994562 | 3,058687544 | 0,290307018 | |
| 212058_at | U2SURP | 5,366783278 | 5,076606343 | 0,290176935 | |
| 204093_at | CCNH | 4,476108798 | 4,186016868 | 0,29009193 | |
| 208745_at | ATP5L | 4,148572682 | 3,858511405 | 0,290061277 | |

| | | | | | |
|---|---|---|---|---|---|
| 200870_at | STRAP | 5,530344319 | 5,240381972 | 0,289962347 | |
| 203138_at | HAT1 | 5,443592265 | 5,153686264 | 0,289906 | |
| 202863_at | SP100 | 2,178241192 | 1,888402606 | 0,289838586 | |
| 212528_at | DESI1 | 1,982112682 | 1,692388143 | 0,28972454 | |
| 238793_at | TIGD7 | 1,982112682 | 1,692388143 | 0,28972454 | |
| 219492_at | CHIC2 | 3,551027544 | 3,261354437 | 0,289673107 | |
| 225479_at | LRRC58 | 3,801937331 | 3,512402411 | 0,28953492 | |
| 1568780_at | - | -0,622910612 | -0,912331589 | 0,289420976 | |
| 217459_at | - | -0,622910612 | -0,912331589 | 0,289420976 | |
| 219325_s_at | ELAC1 | -0,622910612 | -0,912331589 | 0,289420976 | |
| 220733_at | SLC26A1 | -0,622910612 | -0,912331589 | 0,289420976 | |
| 223434_at | GBP3 | -0,622910612 | -0,912331589 | 0,289420976 | |
| 227688_at | LRCH2 | -0,622910612 | -0,912331589 | 0,289420976 | |
| 230534_at | ZNF678 | -0,622910612 | -0,912331589 | 0,289420976 | |
| 1552631_a_at | MAP3K6 | -0,033456564 | -0,322870281 | 0,289413717 | |
| 1560826_at | - | -0,033456564 | -0,322870281 | 0,289413717 | |
| 1561988_at | LOC286068 | -0,033456564 | -0,322870281 | 0,289413717 | |
| 1562910_at | SH3PXD2B | -0,033456564 | -0,322870281 | 0,289413717 | |
| 205692_s_at | CD38 | -0,033456564 | -0,322870281 | 0,289413717 | |
| 221315_s_at | FGF22 | -0,033456564 | -0,322870281 | 0,289413717 | |
| 226485_at | VSIG10 | -0,033456564 | -0,322870281 | 0,289413717 | |
| 227893_at | LINC00476 | -0,033456564 | -0,322870281 | 0,289413717 | |
| 232212_at | PLEKHA8 | -0,033456564 | -0,322870281 | 0,289413717 | |
| 218649_x_at | NEMF | 4,321885451 | 4,032509762 | 0,289375689 | |
| 218404_at | SNX10 | 0,03125738 | -0,258114234 | 0,289371614 | |
| 243203_at | - | 0,03125738 | -0,258114234 | 0,289371614 | |
| 214800_x_at | BTF3 | 6,34364026 | 6,054320155 | 0,289320105 | |
| 216647_at | TCF3 | -0,172274514 | -0,461594427 | 0,289319913 | |
| 236540_at | - | -0,172274514 | -0,461594427 | 0,289319913 | |
| 203197_s_at | C1orf123 | 2,718233611 | 2,428994491 | 0,28923912 | |
| 219441_s_at | LRRK1 | 0,152582607 | -0,136643983 | 0,28922659 | |
| 1562220_at | - | 0,209623097 | -0,079522948 | 0,289146045 | |
| 205575_at | C1QL1 | 0,209623097 | -0,079522948 | 0,289146045 | |
| 210318_at | RBP3 | 0,209623097 | -0,079522948 | 0,289146045 | |
| 220054_at | IL23A | 0,209623097 | -0,079522948 | 0,289146045 | |
| 230047_at | ARHGAP42 | 0,209623097 | -0,079522948 | 0,289146045 | |
| 221486_at | ENSA | 1,094335495 | 0,805216064 | 0,289119431 | |
| 209681_at | SLC19A2 | 3,025418883 | 2,7363693 | 0,289049583 | |
| 201464_x_at | JUN | 4,102254727 | 3,813221015 | 0,289033712 | |
| 200067_x_at | SNX3 | 6,302238787 | 6,013216675 | 0,289022112 | |
| 206357_at | OPA3 | 0,317354302 | 0,028364014 | 0,288990288 | |
| 226678_at | UNC13D | 0,317354302 | 0,028364014 | 0,288990288 | |
| 232470_at | SIK1 | 0,317354302 | 0,028364014 | 0,288990288 | |
| 242479_s_at | MCM4 | 0,317354302 | 0,028364014 | 0,288990288 | |
| 220199_s_at | AIDA | 3,102740237 | 2,813804482 | 0,288935755 | |
| 204402_at | RHBDD3 | 1,758252436 | 1,469325098 | 0,288927339 | |
| 216100_s_at | TOR1AIP1 | 1,795990708 | 1,507116528 | 0,28887418 | |
| 1559529_at | PTK2 | 0,417596971 | 0,128747141 | 0,28884983 | |
| 202044_at | ARHGAP35 | 0,417596971 | 0,128747141 | 0,28884983 | |
| 202103_at | BRD4 | 0,46522187 | 0,176436073 | 0,288785797 | |
| 209611_s_at | SLC1A4 | 0,46522187 | 0,176436073 | 0,288785797 | |
| 223578_x_at | MALAT1 | 0,46522187 | 0,176436073 | 0,288785797 | |
| 235327_x_at | UBXN2A | 3,245961699 | 2,957221307 | 0,288740392 | |
| 225906_at | - | 1,369290453 | 1,080561626 | 0,288728827 | |
| 230556_at | IMMP1L | 1,394109935 | 1,105412836 | 0,2886971 | |
| 227767_at | CSNK1G3 | 2,630440446 | 2,341749893 | 0,288690553 | |

| | | | | | |
|---|---|---|---|---|---|
| 206883_x_at | GP9 | 0,555999837 | 0,26733076 | 0,288669077 | |
| 212092_at | PEG10 | 0,555999837 | 0,26733076 | 0,288669077 | |
| 229197_at | ING5 | 0,555999837 | 0,26733076 | 0,288669077 | |
| 229525_at | - | 0,555999837 | 0,26733076 | 0,288669077 | |
| 225174_at | DNAJC10 | 3,651373876 | 3,362748434 | 0,288625442 | |
| 1556242_a_at | LOC100310756 | -0,325749314 | -0,614371577 | 0,288622263 | |
| 1565703_at | SMAD4 | -0,325749314 | -0,614371577 | 0,288622263 | |
| 219652_s_at | CXorf36 | -0,325749314 | -0,614371577 | 0,288622263 | |
| 223611_s_at | LNX1 | -0,325749314 | -0,614371577 | 0,288622263 | |
| 226402_at | CYP2U1 | -0,325749314 | -0,614371577 | 0,288622263 | |
| 228119_at | LRCH3 | -0,325749314 | -0,614371577 | 0,288622263 | |
| 229396_at | OVOL1 | -0,325749314 | -0,614371577 | 0,288622263 | |
| 240674_at | JARID2 | -0,325749314 | -0,614371577 | 0,288622263 | |
| 243785_at | LOC100272217 | -0,325749314 | -0,614371577 | 0,288622263 | |
| 222705_s_at | SLC25A15 | 0,641402394 | 0,352836757 | 0,288565637 | |
| 1559222_at | - | -1,089016378 | -1,377419394 | 0,288403016 | |
| 1561884_at | CEPT1 | -1,089016378 | -1,377419394 | 0,288403016 | |
| 1565771_at | DKFZp434E1119 | -1,089016378 | -1,377419394 | 0,288403016 | |
| 215721_at | IGHG1 | -1,089016378 | -1,377419394 | 0,288403016 | |
| 217476_at | NR1D1 /// THRA | -1,089016378 | -1,377419394 | 0,288403016 | |
| 219463_at | LAMP5 | -1,089016378 | -1,377419394 | 0,288403016 | |
| 238526_at | RAB3IP | -1,089016378 | -1,377419394 | 0,288403016 | |
| 239931_at | - | -1,089016378 | -1,377419394 | 0,288403016 | |
| 240498_at | - | -1,089016378 | -1,377419394 | 0,288403016 | |
| 219399_at | LIN7C | 2,817903185 | 2,529740643 | 0,288162542 | |
| 202564_x_at | ARL2 | 2,59162628 | 2,303602088 | 0,288024192 | |
| 203566_s_at | AGL | 2,59162628 | 2,303602088 | 0,288024192 | |
| 217739_s_at | NAMPT | 3,800390846 | 3,512402411 | 0,287988435 | |
| 201985_at | KIAA0196 | 3,410096504 | 3,122166354 | 0,28793015 | |
| 226100_at | MLL5 | 2,933877275 | 2,646096941 | 0,287780334 | |
| 222732_at | TRIM39 /// TRIM3 | 1,603688695 | 1,315948728 | 0,287739967 | |
| 228854_at | - | 3,032022986 | 2,744302548 | 0,287720438 | |
| 1561704_at | - | -2,667025278 | -2,954576755 | 0,287551478 | |
| 1562080_at | LINC00424 | -2,667025278 | -2,954576755 | 0,287551478 | |
| 1563690_at | PPP4R1 | -2,667025278 | -2,954576755 | 0,287551478 | |
| 1567575_at | - | -2,667025278 | -2,954576755 | 0,287551478 | |
| 1569297_at | LOC731779 | -2,667025278 | -2,954576755 | 0,287551478 | |
| 205152_at | SLC6A1 | -2,667025278 | -2,954576755 | 0,287551478 | |
| 206786_at | HTN3 | -2,667025278 | -2,954576755 | 0,287551478 | |
| 206960_at | LPAR4 | -2,667025278 | -2,954576755 | 0,287551478 | |
| 209291_at | ID4 | -2,667025278 | -2,954576755 | 0,287551478 | |
| 221126_at | DKK3 | -2,667025278 | -2,954576755 | 0,287551478 | |
| 224116_at | - | -2,667025278 | -2,954576755 | 0,287551478 | |
| 234448_at | - | -2,667025278 | -2,954576755 | 0,287551478 | |
| 240143_at | - | -2,667025278 | -2,954576755 | 0,287551478 | |
| 240787_at | - | -2,667025278 | -2,954576755 | 0,287551478 | |
| 244256_at | CACNA1E | -2,667025278 | -2,954576755 | 0,287551478 | |
| 244511_at | - | -2,667025278 | -2,954576755 | 0,287551478 | |
| 44702_at | SYDE1 | 1,562314261 | 1,274914139 | 0,287400122 | |
| 1556889_s_at | - | -3,114791413 | -3,402185466 | 0,287394053 | |
| 242516_x_at | RBM46 | -3,114791413 | -3,402185466 | 0,287394053 | |
| 242955_x_at | - | -3,114791413 | -3,402185466 | 0,287394053 | |
| 1053_at | RFC2 | 2,831595806 | 2,544354523 | 0,287241283 | |
| 222120_at | ZNF764 | 0,767063567 | 0,479907041 | 0,287156526 | |
| 222536_s_at | ZNF395 | 0,767063567 | 0,479907041 | 0,287156526 | |
| 222078_at | PKLR | 0,841135803 | 0,553993624 | 0,287142179 | |

| | | | | | |
|---|---|---|---|---|---|
| 1555830_s_at | ESYT2 | 0,911589958 | 0,624460759 | 0,287129199 | |
| 205946_at | VIPR2 | 0,911589958 | 0,624460759 | 0,287129199 | |
| 228996_at | RC3H1 | 0,911589958 | 0,624460759 | 0,287129199 | |
| 218392_x_at | SFXN1 | 3,352164686 | 3,065035915 | 0,287128771 | |
| 209984_at | KDM4C | 0,945567441 | 0,658444279 | 0,287123162 | |
| 41329_at | SCYL3 | 1,148825584 | 0,86173571 | 0,287089874 | |
| 1553136_at | KCTD18 | 1,163336153 | 0,876248481 | 0,287087672 | |
| 226778_at | C8orf42 | 1,163336153 | 0,876248481 | 0,287087672 | |
| 238766_at | - | 1,163336153 | 0,876248481 | 0,287087672 | |
| 220985_s_at | RNF170 | 1,191926657 | 0,904843258 | 0,287083398 | |
| 206613_s_at | TAF1A | 1,805273023 | 1,518263577 | 0,287009446 | |
| 210793_s_at | NUP98 | 3,51093129 | 3,22439681 | 0,28653448 | |
| 213058_at | TTC28 | 2,208874124 | 1,922365006 | 0,286509118 | |
| 213501_at | ACOX1 | 2,122398022 | 1,835911187 | 0,286486836 | |
| 218422_s_at | RBM26 | 4,386700496 | 4,100219485 | 0,286481011 | |
| 209258_s_at | SMC3 | 1,523472489 | 1,23718272 | 0,286289769 | |
| 202156_s_at | CELF2 | 3,008105382 | 2,721978587 | 0,286126796 | |
| 225444_at | UBN2 | 2,344516784 | 2,058399602 | 0,286117182 | |
| 207730_x_at | - | 2,331726354 | 2,045615873 | 0,286110481 | |
| 201450_s_at | TIA1 | 3,312571045 | 3,026519779 | 0,286051266 | |
| 201414_s_at | NAP1L4 | 3,266269388 | 2,980244813 | 0,286024575 | |
| 225222_at | HIAT1 | 3,729838025 | 3,443906002 | 0,285932024 | |
| 216383_at | - | 3,670124012 | 3,384231608 | 0,285892404 | |
| 227056_at | KIAA0141 | 1,865674241 | 1,579853228 | 0,285821013 | |
| 209053_s_at | WHSC1 | 2,81177561 | 2,526063925 | 0,285711686 | |
| 238562_at | UTP23 | 1,67631007 | 1,390636111 | 0,285673959 | |
| 221749_at | YTHDF3 | 4,997744463 | 4,712174488 | 0,285569974 | |
| 34260_at | TELO2 | 1,538392922 | 1,252838876 | 0,285554046 | |
| 203017_s_at | SSX2IP | 2,893830579 | 2,608344689 | 0,28548589 | |
| 202101_s_at | RALB | 2,516380342 | 2,230931691 | 0,285448651 | |
| 208828_at | POLE3 | 5,214280103 | 4,928848136 | 0,285431966 | |
| 218432_at | FBXO3 | 2,035673598 | 1,750261389 | 0,28541221 | |
| 204836_at | GLDC | 3,052955227 | 2,767843855 | 0,285111371 | |
| 211698_at | EID1 | 4,072452125 | 3,787346121 | 0,285106004 | |
| 231057_at | - | 1,05337236 | 0,768343791 | 0,285028569 | |
| 232918_at | LOC541471 | 1,05337236 | 0,768343791 | 0,285028569 | |
| 1555282_a_at | PPARGC1B | -0,394749123 | -0,679763839 | 0,285014716 | |
| 1562600_at | - | -0,394749123 | -0,679763839 | 0,285014716 | |
| 205777_at | DUSP9 | -0,394749123 | -0,679763839 | 0,285014716 | |
| 210087_s_at | MPZL1 | -0,394749123 | -0,679763839 | 0,285014716 | |
| 224408_at | MCHR2 | -0,394749123 | -0,679763839 | 0,285014716 | |
| 232294_at | LOC219347 | -0,394749123 | -0,679763839 | 0,285014716 | |
| 235544_x_at | FAM171A2 | -0,394749123 | -0,679763839 | 0,285014716 | |
| 238679_at | MESDC2 | -0,394749123 | -0,679763839 | 0,285014716 | |
| 241475_at | BREA2 | -0,394749123 | -0,679763839 | 0,285014716 | |
| 242832_at | PER1 | -0,394749123 | -0,679763839 | 0,285014716 | |
| 219070_s_at | MOSPD3 | 1,021868205 | 0,73688026 | 0,284987945 | |
| 227375_at | ANKRD13C | 1,021868205 | 0,73688026 | 0,284987945 | |
| 243010_at | MSI2 | 0,95671774 | 0,67181667 | 0,28490107 | |
| 214118_x_at | PCM1 | 4,536342985 | 4,251494565 | 0,28484842 | |
| 32541_at | PPP3CC | 1,769361774 | 1,484560668 | 0,284801106 | |
| 213789_at | - | 1,485482016 | 1,200746069 | 0,284735946 | |
| 241682_at | KLHL23 | 1,462198073 | 1,177496821 | 0,284701253 | |
| 239042_at | TSR1 | 0,779676066 | 0,495031851 | 0,284644215 | |
| 1554574_a_at | CYB5R3 | 1,390002893 | 1,105412836 | 0,284590058 | |
| 1555900_at | - | 1,390002893 | 1,105412836 | 0,284590058 | |

| | | | | | |
|---|---|---|---|---|---|
| 235349_at | FAM82A1 | 0,702292136 | 0,417770464 | 0,284521673 | |
| 1559067_a_at | LOC158402 | 1,314003737 | 1,029536893 | 0,284466843 | |
| 203134_at | PICALM | 1,314003737 | 1,029536893 | 0,284466843 | |
| 214788_x_at | DDN | 0,661985843 | 0,377530647 | 0,284455196 | |
| 228181_at | SLC30A1 | 1,261018186 | 0,976641161 | 0,284377025 | |
| 1556665_at | LOC100652860 / | -1,047650486 | -1,332017329 | 0,284366844 | |
| 1557585_at | ATP6V1H | -1,047650486 | -1,332017329 | 0,284366844 | |
| 1559075_s_at | BAHCC1 | -1,047650486 | -1,332017329 | 0,284366844 | |
| 1561591_at | - | -1,047650486 | -1,332017329 | 0,284366844 | |
| 205518_s_at | CMAHP | -1,047650486 | -1,332017329 | 0,284366844 | |
| 206629_at | ADAMTSL2 | -1,047650486 | -1,332017329 | 0,284366844 | |
| 214781_at | - | -1,047650486 | -1,332017329 | 0,284366844 | |
| 216075_at | - | -1,047650486 | -1,332017329 | 0,284366844 | |
| 216361_s_at | KAT6A | -1,047650486 | -1,332017329 | 0,284366844 | |
| 223709_s_at | WNT10A | -1,047650486 | -1,332017329 | 0,284366844 | |
| 225630_at | EEPD1 | -1,047650486 | -1,332017329 | 0,284366844 | |
| 229012_at | C9orf24 | -1,047650486 | -1,332017329 | 0,284366844 | |
| 230803_s_at | ARHGAP24 | -1,047650486 | -1,332017329 | 0,284366844 | |
| 230951_at | EPB41L5 | -1,047650486 | -1,332017329 | 0,284366844 | |
| 236043_at | LOC100130175 | -1,047650486 | -1,332017329 | 0,284366844 | |
| 237030_at | ACPP | -1,047650486 | -1,332017329 | 0,284366844 | |
| 240056_at | NSMAF | -1,047650486 | -1,332017329 | 0,284366844 | |
| 240891_at | FSTL1 | -1,047650486 | -1,332017329 | 0,284366844 | |
| 244047_at | - | -1,047650486 | -1,332017329 | 0,284366844 | |
| 244344_at | WNK4 | -1,047650486 | -1,332017329 | 0,284366844 | |
| 221664_s_at | F11R | 2,510717331 | 2,226407528 | 0,284309803 | |
| 1553627_s_at | C17orf57 | -2,712026959 | -2,996335703 | 0,284308743 | |
| 1570136_at | - | -2,712026959 | -2,996335703 | 0,284308743 | |
| 1570384_at | TAS2R19 | -2,712026959 | -2,996335703 | 0,284308743 | |
| 217306_at | - | -2,712026959 | -2,996335703 | 0,284308743 | |
| 232461_at | AHI1 | -2,712026959 | -2,996335703 | 0,284308743 | |
| 237912_at | - | -2,712026959 | -2,996335703 | 0,284308743 | |
| 238555_at | - | -2,712026959 | -2,996335703 | 0,284308743 | |
| 238810_at | RFX3 | -2,712026959 | -2,996335703 | 0,284308743 | |
| 240137_at | - | -2,712026959 | -2,996335703 | 0,284308743 | |
| 241705_at | ABCA5 | -2,712026959 | -2,996335703 | 0,284308743 | |
| 243317_at | - | -2,712026959 | -2,996335703 | 0,284308743 | |
| 241703_at | RUNDC3B | 1,177702228 | 0,893473268 | 0,28422896 | |
| 1567081_x_at | CLN6 | 1,119359127 | 0,835239052 | 0,284120075 | |
| 210910_s_at | POMZP3 | 1,119359127 | 0,835239052 | 0,284120075 | |
| 205280_at | GLRB | 1,089278259 | 0,805216064 | 0,284062195 | |
| 221833_at | LOC100507577 / | 1,089278259 | 0,805216064 | 0,284062195 | |
| 239913_at | SLC10A4 | 4,450701473 | 4,166665505 | 0,284035969 | |
| 212628_at | PKN2 | 2,355089531 | 2,071071048 | 0,284018483 | |
| 205005_s_at | NMT2 | 2,11992033 | 1,835911187 | 0,284009143 | |
| 222611_s_at | PSPC1 | 2,089851283 | 1,805902329 | 0,283948954 | |
| 228452_at | C17orf39 | 2,074578432 | 1,790660532 | 0,2839179 | |
| 205136_s_at | NUFIP1 | 1,764611101 | 1,480766833 | 0,283844268 | |
| 228168_at | ATP5G3 | 1,764611101 | 1,480766833 | 0,283844268 | |
| 226882_x_at | WDR4 | 2,466546243 | 2,182705233 | 0,28384101 | |
| 242961_x_at | DDX58 | 2,466546243 | 2,182705233 | 0,28384101 | |
| 1560348_at | RGNEF | 0,291166226 | 0,007421914 | 0,283744312 | |
| 1569986_x_at | TNNT3 | 0,291166226 | 0,007421914 | 0,283744312 | |
| 239044_at | ITFG1 | 0,291166226 | 0,007421914 | 0,283744312 | |
| 206875_s_at | SLK | 3,798842702 | 3,515187047 | 0,283655655 | |
| 89476_r_at | NPEPL1 | -0,298635321 | -0,582258154 | 0,283622833 | |

| | | | | | |
|---|---|---|---|---|---|
| 241653_x_at | - | -3,15396682 | -3,437493426 | 0,283526606 | |
| 1552924_a_at | PITPNM2 | 0,181384709 | -0,102100538 | 0,283485247 | |
| 219413_at | ACBD4 | 0,181384709 | -0,102100538 | 0,283485247 | |
| 241798_at | - | 0,181384709 | -0,102100538 | 0,283485247 | |
| 209674_at | CRY1 | 4,111638046 | 3,828227545 | 0,283410501 | |
| 226151_x_at | CRYZL1 | 2,860082603 | 2,576704359 | 0,283378243 | |
| 205490_x_at | GJB3 | 0,123193849 | -0,160136748 | 0,283330598 | |
| 210365_at | LOC100506403 / | 0,123193849 | -0,160136748 | 0,283330598 | |
| 214873_at | LRP5L | 0,123193849 | -0,160136748 | 0,283330598 | |
| 226698_at | FCHSD1 | 0,123193849 | -0,160136748 | 0,283330598 | |
| 201448_at | TIA1 | 4,018934862 | 3,735753654 | 0,283181208 | |
| 228164_at | AP4E1 | 2,281618189 | 1,998633167 | 0,282985022 | |
| 1567255_at | OR10D1P | -0,00073712 | -0,283667828 | 0,282930707 | |
| 221400_at | MYO3A | -0,00073712 | -0,283667828 | 0,282930707 | |
| 229583_at | - | -0,00073712 | -0,283667828 | 0,282930707 | |
| 230734_x_at | STRN | 2,64946283 | 2,366632651 | 0,282830179 | |
| 223228_at | LDOC1L | 2,361396186 | 2,078620836 | 0,282775351 | |
| 206303_s_at | NUDT4 | 3,133773173 | 2,851017268 | 0,282755905 | |
| 201925_s_at | CD55 | 3,912008086 | 3,629253073 | 0,282755012 | |
| 222207_x_at | - | 3,849821959 | 3,56709884 | 0,282723119 | |
| 225671_at | SPNS2 | 2,001086023 | 1,718396606 | 0,282689417 | |
| 201088_at | KPNA2 | 6,42203549 | 6,139502818 | 0,282532672 | |
| 206383_s_at | G3BP2 | 2,771299138 | 2,488772006 | 0,282527132 | |
| 32723_at | CSTF1 | 3,123915073 | 2,841433901 | 0,282481172 | |
| 221759_at | G6PC3 | 1,473887017 | 1,191491281 | 0,282395736 | |
| 206453_s_at | NDRG2 | 1,187200746 | 0,904843258 | 0,282357488 | |
| 215001_s_at | GLUL | 5,936974356 | 5,654663835 | 0,282310521 | |
| 39705_at | SIN3B | 2,940210922 | 2,657904765 | 0,282306157 | |
| 223650_s_at | NRBF2 | 1,672933557 | 1,390636111 | 0,282297445 | |
| 202551_s_at | CRIM1 | -0,136301282 | -0,41858459 | 0,282283307 | |
| 205718_at | ITGB7 | -0,136301282 | -0,41858459 | 0,282283307 | |
| 219959_at | MOCOS | -0,136301282 | -0,41858459 | 0,282283307 | |
| 229696_at | FECH | -0,136301282 | -0,41858459 | 0,282283307 | |
| 236913_at | - | -0,136301282 | -0,41858459 | 0,282283307 | |
| 239214_at | LOC100130458 | -0,136301282 | -0,41858459 | 0,282283307 | |
| 205002_at | AHDC1 | 1,129248302 | 0,847075464 | 0,282172838 | |
| 229321_s_at | - | 1,631786706 | 1,349621332 | 0,282165374 | |
| 231869_at | KIAA1586 | 4,731959456 | 4,449849671 | 0,282109785 | |
| 201531_at | ZFP36 | 2,68509622 | 2,403170156 | 0,281926065 | |
| 1563485_at | - | 0,715481152 | 0,433557298 | 0,281923853 | |
| 226986_at | WIPI2 | 2,675004741 | 2,39311332 | 0,281891422 | |
| 214449_s_at | RHOQ | 1,037706275 | 0,755840599 | 0,281865676 | |
| 1564021_at | LOC100130456 | 0,67554678 | 0,393761504 | 0,281785276 | |
| 209972_s_at | AIMP2 | 0,67554678 | 0,393761504 | 0,281785276 | |
| 231222_at | - | 0,67554678 | 0,393761504 | 0,281785276 | |
| 202007_at | NID1 | 3,308224834 | 3,026519779 | 0,281705055 | |
| 203933_at | RAB11FIP3 | 0,97328325 | 0,691645685 | 0,281637565 | |
| 208788_at | ELOVL5 | 6,192813132 | 5,911202215 | 0,281610917 | |
| 212476_at | ACAP2 | 3,495722611 | 3,21415095 | 0,281571661 | |
| 219966_x_at | BANP | 3,268508253 | 2,986947093 | 0,28156116 | |
| 212403_at | UBE3B | 2,478200152 | 2,196649505 | 0,281550647 | |
| 222441_x_at | SLMO2 | 4,648465551 | 4,36698147 | 0,28148408 | |
| 226444_at | - | 0,592200562 | 0,310717132 | 0,28148343 | |
| 52741_at | TRMT61A | 1,62481334 | 1,343367293 | 0,281446047 | |
| 219668_at | GDAP1L1 | -0,57445847 | -0,855751026 | 0,281292556 | |
| 220740_s_at | SLC12A6 | -0,57445847 | -0,855751026 | 0,281292556 | |

| | | | | | |
|---|---|---|---|---|---|
| 226972_s_at | CCDC136 | -0,57445847 | -0,855751026 | 0,281292556 | |
| 237872_at | LOC100505697 | -0,57445847 | -0,855751026 | 0,281292556 | |
| 244818_at | ZNF780B | -0,57445847 | -0,855751026 | 0,281292556 | |
| 221643_s_at | RERE | 0,503742507 | 0,222599095 | 0,281143412 | |
| 227606_s_at | STAMBPL1 | 0,503742507 | 0,222599095 | 0,281143412 | |
| 209629_s_at | NXT2 | 2,009141686 | 1,728030156 | 0,28111153 | |
| 233385_x_at | MIA-RAB4B /// RA | 1,85379461 | 1,572742379 | 0,281052231 | |
| 1554609_at | LOC100287896 | -0,285837228 | -0,566887459 | 0,281050231 | |
| 1561868_at | - | -0,285837228 | -0,566887459 | 0,281050231 | |
| 210654_at | TNFRSF10D | -0,285837228 | -0,566887459 | 0,281050231 | |
| 215201_at | REPS1 | -0,285837228 | -0,566887459 | 0,281050231 | |
| 224087_at | - | -0,285837228 | -0,566887459 | 0,281050231 | |
| 236647_at | - | -0,285837228 | -0,566887459 | 0,281050231 | |
| 1555640_at | DAOA | -2,756478458 | -3,037500868 | 0,28102241 | |
| 1563039_at | LOC100506195 | -2,756478458 | -3,037500868 | 0,28102241 | |
| 1566043_at | - | -2,756478458 | -3,037500868 | 0,28102241 | |
| 209335_at | DCN | -2,756478458 | -3,037500868 | 0,28102241 | |
| 210498_at | CLTC | -2,756478458 | -3,037500868 | 0,28102241 | |
| 216057_at | AURKAPS1 /// RA | -2,756478458 | -3,037500868 | 0,28102241 | |
| 217131_at | - | -2,756478458 | -3,037500868 | 0,28102241 | |
| 235915_at | - | -2,756478458 | -3,037500868 | 0,28102241 | |
| 236502_at | - | -2,756478458 | -3,037500868 | 0,28102241 | |
| 237122_at | WDR7 | -2,756478458 | -3,037500868 | 0,28102241 | |
| 237177_at | CNTN4 | -2,756478458 | -3,037500868 | 0,28102241 | |
| 239624_at | - | -2,756478458 | -3,037500868 | 0,28102241 | |
| 242721_at | - | -2,756478458 | -3,037500868 | 0,28102241 | |
| 208386_x_at | DMC1 | 0,798390451 | 0,51742585 | 0,2809646 | |
| 215700_x_at | CPNE6 | 0,457392764 | 0,176436073 | 0,280956691 | |
| 223147_s_at | SFT2D3 /// WDR | 0,457392764 | 0,176436073 | 0,280956691 | |
| 232922_s_at | SLC17A9 | 0,457392764 | 0,176436073 | 0,280956691 | |
| 1562495_at | ANKFY1 | -0,83191556 | -1,112851844 | 0,280936284 | |
| 1566135_at | CARHSP1 | -0,83191556 | -1,112851844 | 0,280936284 | |
| 1570373_at | ZNF746 | -0,83191556 | -1,112851844 | 0,280936284 | |
| 205468_s_at | IRF5 | -0,83191556 | -1,112851844 | 0,280936284 | |
| 207191_s_at | ISLR | -0,83191556 | -1,112851844 | 0,280936284 | |
| 210141_s_at | INHA | -0,83191556 | -1,112851844 | 0,280936284 | |
| 216630_at | - | -0,83191556 | -1,112851844 | 0,280936284 | |
| 216661_x_at | CYP2C9 | -0,83191556 | -1,112851844 | 0,280936284 | |
| 217258_x_at | IGLV1-44 | -0,83191556 | -1,112851844 | 0,280936284 | |
| 217415_at | POLR2A | -0,83191556 | -1,112851844 | 0,280936284 | |
| 219775_s_at | CPLX3 | -0,83191556 | -1,112851844 | 0,280936284 | |
| 220179_at | DPEP3 | -0,83191556 | -1,112851844 | 0,280936284 | |
| 235426_at | GATM | -0,83191556 | -1,112851844 | 0,280936284 | |
| 238338_at | - | -0,83191556 | -1,112851844 | 0,280936284 | |
| 244607_at | - | -0,83191556 | -1,112851844 | 0,280936284 | |
| 1556194_a_at | - | 2,760204767 | 2,479296438 | 0,280908329 | |
| 223118_s_at | USP47 | 2,305800198 | 2,024923328 | 0,28087687 | |
| 220018_at | CBLL1 | 1,799091453 | 1,518263577 | 0,280827876 | |
| 220119_at | EPB41L4A | 0,760715727 | 0,479907041 | 0,280808686 | |
| 212381_at | USP24 | 2,994644467 | 2,713921281 | 0,280723186 | |
| 201685_s_at | TOX4 | 2,238870125 | 1,958277216 | 0,280592908 | |
| 203598_s_at | WBP4 | 2,333865983 | 2,053299695 | 0,280566288 | |
| 213809_x_at | TCF3 | 0,359971644 | 0,079428135 | 0,280543509 | |
| 230606_at | LOC100287525 | 0,359971644 | 0,079428135 | 0,280543509 | |
| 213450_s_at | ICOSLG | 1,575032557 | 1,294497133 | 0,280535424 | |
| 201816_s_at | GBAS | 4,297973795 | 4,017530898 | 0,280442897 | |

| | | | | | |
|---|---|---|---|---|---|
| 219986_s_at | ACAD10 | 0,30867765 | 0,028364014 | 0,280313636 | |
| 240342_at | TRIM61 | 0,30867765 | 0,028364014 | 0,280313636 | |
| 36907_at | MVK | 2,678376417 | 2,398150501 | 0,280225916 | |
| 223213_s_at | ZHX1 | 3,479385765 | 3,199221717 | 0,280164047 | |
| 209296_at | PPM1B | 3,981211211 | 3,701119714 | 0,280091496 | |
| 1554091_a_at | TIRAP | 0,255492549 | -0,024572586 | 0,280065136 | |
| 233686_at | ASXL3 | 0,255492549 | -0,024572586 | 0,280065136 | |
| 238473_at | LOC100216545 | 0,255492549 | -0,024572586 | 0,280065136 | |
| 1559400_s_at | PAPPA | -0,366776884 | -0,646746079 | 0,279969195 | |
| 1561233_at | LOC283387 | -0,366776884 | -0,646746079 | 0,279969195 | |
| 243484_x_at | - | -0,366776884 | -0,646746079 | 0,279969195 | |
| 210649_s_at | ARID1A | 1,965648824 | 1,685812099 | 0,279836725 | |
| 205706_s_at | ANKRD26 | 1,04816921 | 0,768343791 | 0,27982542 | |
| 206892_at | AMHR2 | 0,20027154 | -0,079522948 | 0,279794488 | |
| 1559986_at | LIFR-AS1 /// MIR | -3,192553003 | -3,472251727 | 0,279698724 | |
| 1565800_x_at | RAB3IP | -3,192553003 | -3,472251727 | 0,279698724 | |
| 1566930_at | TFB2M | -3,192553003 | -3,472251727 | 0,279698724 | |
| 225484_at | CEP41 | 2,402750389 | 2,123108223 | 0,279642166 | |
| 224567_x_at | LOC100507645 / | 4,565770118 | 4,286143696 | 0,279626421 | |
| 225087_at | FOPNL | 4,486724797 | 4,207122602 | 0,279602195 | |
| 235189_at | NARG2 | 0,984222186 | 0,704715219 | 0,279506967 | |
| 242233_at | - | 0,984222186 | 0,704715219 | 0,279506967 | |
| 226901_at | C17orf58 | 2,718233611 | 2,438805127 | 0,279428484 | |
| 217707_x_at | SMARCA2 | 2,173470163 | 1,894118818 | 0,279351345 | |
| 209656_s_at | TMEM47 | 2,688444421 | 2,409170778 | 0,279273643 | |
| 51146_at | PIGV | 0,925844617 | 0,646640981 | 0,279203636 | |
| 207634_at | PDCD1 | 0,083054335 | -0,19610998 | 0,279164315 | |
| 229258_at | KIF12 | 0,083054335 | -0,19610998 | 0,279164315 | |
| 230626_at | TSPAN12 | 0,083054335 | -0,19610998 | 0,279164315 | |
| 219050_s_at | ZNHIT2 | 0,917308734 | 0,638150376 | 0,279158358 | |
| 1553780_at | MGC23270 | 1,114389003 | 0,835239052 | 0,279149951 | |
| 202929_s_at | DDT | 3,189186085 | 2,910041809 | 0,279144276 | |
| 218924_s_at | CTBS | 0,882651021 | 0,603679647 | 0,278971374 | |
| 227623_at | CACNA2D1 | 0,020670649 | -0,258114234 | 0,278784883 | |
| 235921_at | - | 0,020670649 | -0,258114234 | 0,278784883 | |
| 212709_at | NUP160 | 3,462861802 | 3,184136378 | 0,278725424 | |
| 227866_at | LOC100505519 / | 1,493160564 | 1,214517879 | 0,278642685 | |
| 213391_at | DPY19L4 | 2,038300149 | 1,759685182 | 0,278614968 | |
| 216484_x_at | HDGF | 3,717599787 | 3,439026336 | 0,278573451 | |
| 226064_s_at | DGAT2 | 1,318332599 | 1,039887232 | 0,278445368 | |
| 202660_at | ITPR2 | 1,446464021 | 1,16809119 | 0,27837283 | |
| 226702_at | CMPK2 | 2,640847372 | 2,362515214 | 0,278332158 | |
| 213404_s_at | RHEB | 5,582338761 | 5,304027206 | 0,278311554 | |
| 207236_at | ZNF345 | 1,292162421 | 1,013870809 | 0,278291612 | |
| 232136_s_at | CTTNBP2 | 1,422536444 | 1,144305291 | 0,278231153 | |
| 244192_x_at | USP4 | 1,422536444 | 1,144305291 | 0,278231153 | |
| 218396_at | VPS13C | 3,158127685 | 2,880111483 | 0,278016202 | |
| 214554_at | HIST1H2AG /// H | 2,745814053 | 2,467842989 | 0,277971064 | |
| 209267_s_at | SLC39A8 | 3,664184451 | 3,386261038 | 0,277923413 | |
| 233421_s_at | NUP133 | 0,695652136 | 0,417770464 | 0,277881673 | |
| 1554063_at | ZHX1-C8ORF76 | 1,814495998 | 1,536652708 | 0,27784329 | |
| 205798_at | IL7R | -0,112808516 | -0,39061235 | 0,277803834 | |
| 209999_x_at | SOCS1 | -0,112808516 | -0,39061235 | 0,277803834 | |
| 221413_at | KCNAB3 | -0,112808516 | -0,39061235 | 0,277803834 | |
| 230571_at | - | -0,112808516 | -0,39061235 | 0,277803834 | |
| 203987_at | FZD6 | 2,074578432 | 1,796776588 | 0,277801844 | |

| | | | | | |
|---|---|---|---|---|---|
| 239859_x_at | ATP5S | 1,210676994 | 0,932882274 | 0,27779472 | |
| 206149_at | CHP2 | -0,67289328 | -0,950686014 | 0,277792735 | |
| 210894_s_at | CEP250 | -0,67289328 | -0,950686014 | 0,277792735 | |
| 227727_at | MRGPRF | -0,67289328 | -0,950686014 | 0,277792735 | |
| 235516_at | SEPSECS | -0,67289328 | -0,950686014 | 0,277792735 | |
| 240633_at | DOK7 | -0,67289328 | -0,950686014 | 0,277792735 | |
| 212238_at | ASXL1 | 1,795990708 | 1,518263577 | 0,27772713 | |
| 225737_s_at | FBXO22 | 1,795990708 | 1,518263577 | 0,27772713 | |
| 222573_s_at | SAV1 | 2,41290634 | 2,135226207 | 0,277680132 | |
| 1554029_a_at | TTC37 | -0,986356909 | -1,264005341 | 0,277648432 | |
| 1564653_s_at | LEKR1 | -0,986356909 | -1,264005341 | 0,277648432 | |
| 209437_s_at | SPON1 | -0,986356909 | -1,264005341 | 0,277648432 | |
| 209589_s_at | EPHB2 | -0,986356909 | -1,264005341 | 0,277648432 | |
| 211262_at | LOC100507472 / | -0,986356909 | -1,264005341 | 0,277648432 | |
| 211301_at | KCND3 | -0,986356909 | -1,264005341 | 0,277648432 | |
| 220366_at | ELSPBP1 | -0,986356909 | -1,264005341 | 0,277648432 | |
| 223648_s_at | FGFRL1 | -0,986356909 | -1,264005341 | 0,277648432 | |
| 231604_at | - | -0,986356909 | -1,264005341 | 0,277648432 | |
| 234549_at | - | -0,986356909 | -1,264005341 | 0,277648432 | |
| 235038_at | KRR1 | -0,986356909 | -1,264005341 | 0,277648432 | |
| 239373_at | - | -0,986356909 | -1,264005341 | 0,277648432 | |
| 240682_at | LINC00293 | -0,986356909 | -1,264005341 | 0,277648432 | |
| 206648_at | ZNF571 | 2,043538949 | 1,765933685 | 0,277605264 | |
| 227026_at | MPHOSPH8 | 3,091402928 | 2,813804482 | 0,277598446 | |
| 204342_at | SLC25A24 | 4,076291169 | 3,798818761 | 0,277472408 | |
| 213198_at | ACVR1B | 2,845159691 | 2,567790814 | 0,277368877 | |
| 1556588_at | C15orf37 | 0,613492853 | 0,336136178 | 0,277356676 | |
| 226676_at | ZNF521 | 0,613492853 | 0,336136178 | 0,277356676 | |
| 204418_x_at | GSTM2 | 1,523472489 | 1,246149888 | 0,277322601 | |
| 203637_s_at | MID1 | 0,57058931 | 0,293518836 | 0,277070475 | |
| 238825_at | ACRC | 1,094335495 | 0,817300337 | 0,277035158 | |
| 219347_at | NUDT15 | 3,495722611 | 3,218713651 | 0,277008959 | |
| 222465_at | RSL24D1 | 5,340953221 | 5,063983634 | 0,276969587 | |
| 214193_s_at | DIEXF | 2,156646206 | 1,879785595 | 0,276860611 | |
| 207650_x_at | PTGER1 | 1,063722698 | 0,786897721 | 0,276824976 | |
| 219833_s_at | EFHC1 | 1,063722698 | 0,786897721 | 0,276824976 | |
| 224064_s_at | DHDDS | 1,063722698 | 0,786897721 | 0,276824976 | |
| 219206_x_at | TMBIM4 | 3,28739962 | 3,010820719 | 0,276578902 | |
| 219362_at | NAA35 | 2,802535172 | 2,526063925 | 0,276471247 | |
| 221627_at | TRIM10 | 0,480753764 | 0,204310755 | 0,276443008 | |
| 229907_at | - | 0,480753764 | 0,204310755 | 0,276443008 | |
| 230988_at | ADD2 | 0,480753764 | 0,204310755 | 0,276443008 | |
| 232145_at | C2orf68 | 0,480753764 | 0,204310755 | 0,276443008 | |
| 222447_at | METTL9 | 2,097427503 | 1,820984782 | 0,276442721 | |
| 211984_at | CALM1 /// CALM2 | 3,882346871 | 3,605931378 | 0,276415493 | |
| 218752_at | ZMAT5 | 1,783520648 | 1,507116528 | 0,27640412 | |
| 1569777_a_at | ZPLD1 | 1,381753566 | 1,105412836 | 0,276340731 | |
| 225371_at | GLE1 | 3,082523011 | 2,806245339 | 0,276277671 | |
| 204823_at | NAV3 | -0,259832361 | -0,536087151 | 0,276254789 | |
| 207969_x_at | ACRV1 | -0,259832361 | -0,536087151 | 0,276254789 | |
| 208599_at | HUWE1 | -0,259832361 | -0,536087151 | 0,276254789 | |
| 213290_at | COL6A2 | -0,259832361 | -0,536087151 | 0,276254789 | |
| 214231_s_at | KIAA0564 | -0,259832361 | -0,536087151 | 0,276254789 | |
| 217494_s_at | PTENP1 | -0,259832361 | -0,536087151 | 0,276254789 | |
| 218266_s_at | NCS1 | -0,259832361 | -0,536087151 | 0,276254789 | |
| 229638_at | IRX3 | -0,259832361 | -0,536087151 | 0,276254789 | |

| | | | | | |
|---|---|---|---|---|---|
| 243264_s_at | C8orf44 /// C8orf4 | -0,259832361 | -0,536087151 | 0,276254789 | |
| 1557638_at | - | -0,543051993 | -0,819238336 | 0,276186343 | |
| 1559065_a_at | CLEC4G | -0,543051993 | -0,819238336 | 0,276186343 | |
| 204543_at | RAPGEF1 | -0,543051993 | -0,819238336 | 0,276186343 | |
| 215680_at | KIAA1654 | -0,543051993 | -0,819238336 | 0,276186343 | |
| 221104_s_at | NIPSNAP3B | -0,543051993 | -0,819238336 | 0,276186343 | |
| 226367_at | KDM5A | -0,543051993 | -0,819238336 | 0,276186343 | |
| 228526_at | - | -0,543051993 | -0,819238336 | 0,276186343 | |
| 235221_at | CBLN3 | -0,543051993 | -0,819238336 | 0,276186343 | |
| 222731_at | ZDHHC2 | 4,171484821 | 3,895315489 | 0,276169332 | |
| 225506_at | KIAA1468 | 1,726031811 | 1,450051551 | 0,27598026 | |
| 229211_at | DUSP28 | 1,331241794 | 1,055274826 | 0,275966968 | |
| 228095_at | PHF14 | 3,875753437 | 3,599822964 | 0,275930472 | |
| 227142_at | PLEKHG5 | 0,934330293 | 0,658444279 | 0,275886014 | |
| 227900_at | CBLB | 1,305306849 | 1,029536893 | 0,275769956 | |
| 205884_at | ITGA4 | 2,825526235 | 2,549796788 | 0,275729447 | |
| 206435_at | B4GALNT1 | 0,384950573 | 0,109221392 | 0,275729181 | |
| 209416_s_at | FZR1 | 0,384950573 | 0,109221392 | 0,275729181 | |
| 221048_x_at | C17orf80 | 0,384950573 | 0,109221392 | 0,275729181 | |
| 1552302_at | TMEM106A | -2,504600601 | -2,780313924 | 0,275713323 | |
| 1553614_a_at | FLJ25694 | -2,504600601 | -2,780313924 | 0,275713323 | |
| 1556272_a_at | - | -2,504600601 | -2,780313924 | 0,275713323 | |
| 1559503_a_at | LOC441666 | -2,504600601 | -2,780313924 | 0,275713323 | |
| 1565105_at | - | -2,504600601 | -2,780313924 | 0,275713323 | |
| 1566772_at | - | -2,504600601 | -2,780313924 | 0,275713323 | |
| 1566916_at | HPYR1 | -2,504600601 | -2,780313924 | 0,275713323 | |
| 1570300_at | - | -2,504600601 | -2,780313924 | 0,275713323 | |
| 205860_x_at | FOLH1 /// FOLH1 | -2,504600601 | -2,780313924 | 0,275713323 | |
| 211496_s_at | PDC | -2,504600601 | -2,780313924 | 0,275713323 | |
| 220523_at | EFHC2 | -2,504600601 | -2,780313924 | 0,275713323 | |
| 220878_at | - | -2,504600601 | -2,780313924 | 0,275713323 | |
| 231239_at | - | -2,504600601 | -2,780313924 | 0,275713323 | |
| 234389_x_at | - | -2,504600601 | -2,780313924 | 0,275713323 | |
| 234540_at | GCNT7 | -2,504600601 | -2,780313924 | 0,275713323 | |
| 237670_at | LOC284801 | -2,504600601 | -2,780313924 | 0,275713323 | |
| 239883_s_at | ANO4 | -2,504600601 | -2,780313924 | 0,275713323 | |
| 240766_at | - | -2,504600601 | -2,780313924 | 0,275713323 | |
| 240815_at | - | -2,504600601 | -2,780313924 | 0,275713323 | |
| 244757_at | - | -2,504600601 | -2,780313924 | 0,275713323 | |
| 200087_s_at | TMED2 | 5,887213751 | 5,611510757 | 0,275702994 | |
| 1554125_a_at | NLGN4Y | -2,823166348 | -3,098858316 | 0,275691968 | |
| 1554724_at | SLC6A11 | -2,823166348 | -3,098858316 | 0,275691968 | |
| 1554912_at | ESYT3 | -2,823166348 | -3,098858316 | 0,275691968 | |
| 1555394_at | PIGK | -2,823166348 | -3,098858316 | 0,275691968 | |
| 1559434_at | - | -2,823166348 | -3,098858316 | 0,275691968 | |
| 1559497_at | - | -2,823166348 | -3,098858316 | 0,275691968 | |
| 1562579_at | - | -2,823166348 | -3,098858316 | 0,275691968 | |
| 1564007_at | - | -2,823166348 | -3,098858316 | 0,275691968 | |
| 1566694_at | - | -2,823166348 | -3,098858316 | 0,275691968 | |
| 215101_s_at | CXCL5 | -2,823166348 | -3,098858316 | 0,275691968 | |
| 238720_at | LOC100506582 | -2,823166348 | -3,098858316 | 0,275691968 | |
| 243979_at | - | -2,823166348 | -3,098858316 | 0,275691968 | |
| 221213_s_at | ZNF280D | 0,900083947 | 0,624460759 | 0,275623188 | |
| 229206_at | - | 0,900083947 | 0,624460759 | 0,275623188 | |
| 239426_at | SLC2A8 | 0,900083947 | 0,624460759 | 0,275623188 | |
| 203621_at | NDUFB5 | 4,920655822 | 4,645113717 | 0,275542105 | |

| | | | | | |
|---|---|---|---|---|---|
| 204396_s_at | GRK5 | 1,666156729 | 1,390636111 | 0,275520617 | |
| 1564064_a_at | ATP11B | 2,320980353 | 2,045615873 | 0,27536448 | |
| 218242_s_at | SUV420H1 | 1,883312263 | 1,60795124 | 0,275361023 | |
| 213919_at | DNAJC4 | 0,334552598 | 0,059218869 | 0,27533373 | |
| 220798_x_at | LPPR3 | 0,334552598 | 0,059218869 | 0,27533373 | |
| 227969_at | PCBP1-AS1 | 0,334552598 | 0,059218869 | 0,27533373 | |
| 240076_at | - | 0,334552598 | 0,059218869 | 0,27533373 | |
| 202632_at | DPH1 /// OVCA2 | 2,972842302 | 2,697670403 | 0,275171899 | |
| 244569_at | C8orf37 | 1,224581536 | 0,949447784 | 0,275133752 | |
| 1558688_at | LOC441461 | 3,203298287 | 2,928263672 | 0,275034615 | |
| 228274_at | SDSL | 2,90394695 | 2,629059192 | 0,274887757 | |
| 210213_s_at | EIF6 | 5,229587283 | 4,954720433 | 0,27486685 | |
| 204346_s_at | RASSF1 | 2,254767157 | 1,979940779 | 0,274826379 | |
| 234675_x_at | - | 2,626954721 | 2,352169913 | 0,274784808 | |
| 1557897_at | - | 0,792179257 | 0,51742585 | 0,274753406 | |
| 226638_at | ARHGAP23 | 0,792179257 | 0,51742585 | 0,274753406 | |
| 213718_at | RBM4 | 0,627515113 | 0,352836757 | 0,274678356 | |
| 226454_at | 09.03.15 | 1,168140757 | 0,893473268 | 0,274667489 | |
| 206953_s_at | LPHN2 | 1,46610492 | 1,191491281 | 0,274613639 | |
| 226887_at | HSPA14 | 2,605861638 | 2,331254065 | 0,274607573 | |
| 1557267_s_at | GEN1 | -1,812418401 | -2,086984744 | 0,274566343 | |
| 1563523_at | - | -1,812418401 | -2,086984744 | 0,274566343 | |
| 1563601_at | - | -1,812418401 | -2,086984744 | 0,274566343 | |
| 1564231_at | IFT80 | -1,812418401 | -2,086984744 | 0,274566343 | |
| 1569590_at | DOCK5 | -1,812418401 | -2,086984744 | 0,274566343 | |
| 1570574_at | - | -1,812418401 | -2,086984744 | 0,274566343 | |
| 206111_at | RNASE2 | -1,812418401 | -2,086984744 | 0,274566343 | |
| 206939_at | DCC | -1,812418401 | -2,086984744 | 0,274566343 | |
| 207977_s_at | DPT | -1,812418401 | -2,086984744 | 0,274566343 | |
| 210116_at | SH2D1A | -1,812418401 | -2,086984744 | 0,274566343 | |
| 215242_at | LOC100505991 / | -1,812418401 | -2,086984744 | 0,274566343 | |
| 215297_at | LOC441204 | -1,812418401 | -2,086984744 | 0,274566343 | |
| 220530_at | - | -1,812418401 | -2,086984744 | 0,274566343 | |
| 233649_at | KATNAL2 | -1,812418401 | -2,086984744 | 0,274566343 | |
| 234168_at | TAF15 | -1,812418401 | -2,086984744 | 0,274566343 | |
| 234257_at | TNS3 | -1,812418401 | -2,086984744 | 0,274566343 | |
| 234750_at | CACNG8 | -1,812418401 | -2,086984744 | 0,274566343 | |
| 239994_at | - | -1,812418401 | -2,086984744 | 0,274566343 | |
| 241695_s_at | - | -1,812418401 | -2,086984744 | 0,274566343 | |
| 244144_at | SYNE1 | -1,812418401 | -2,086984744 | 0,274566343 | |
| 209057_x_at | CDC5L | 2,365585359 | 2,091116676 | 0,274468683 | |
| 225352_at | SEC62 | 1,774096855 | 1,499637023 | 0,274459832 | |
| 209919_x_at | GGT1 /// GGT2 // | 0,228146222 | -0,046302147 | 0,274448369 | |
| 220570_at | RETN | 0,228146222 | -0,046302147 | 0,274448369 | |
| 226144_at | REXO1 | 0,228146222 | -0,046302147 | 0,274448369 | |
| 227292_at | C11orf84 | 1,139070151 | 0,864649967 | 0,274420183 | |
| 1552548_at | BSND | -1,888254346 | -2,162639771 | 0,274385426 | |
| 1560958_s_at | - | -1,888254346 | -2,162639771 | 0,274385426 | |
| 1561728_a_at | LOC400238 | -1,888254346 | -2,162639771 | 0,274385426 | |
| 1562754_at | LOC339260 | -1,888254346 | -2,162639771 | 0,274385426 | |
| 1562835_at | - | -1,888254346 | -2,162639771 | 0,274385426 | |
| 1564403_at | C9orf133 | -1,888254346 | -2,162639771 | 0,274385426 | |
| 1566277_at | OR5E1P | -1,888254346 | -2,162639771 | 0,274385426 | |
| 1569159_at | LZTS1 | -1,888254346 | -2,162639771 | 0,274385426 | |
| 205476_at | CCL20 | -1,888254346 | -2,162639771 | 0,274385426 | |
| 211648_at | IGHG1 /// IGHM | -1,888254346 | -2,162639771 | 0,274385426 | |

| | | | | | |
|---|---|---|---|---|---|
| 213228_at | PDE8B | -1,888254346 | -2,162639771 | 0,274385426 | |
| 215120_s_at | SAMD4A | -1,888254346 | -2,162639771 | 0,274385426 | |
| 219684_at | RTP4 | -1,888254346 | -2,162639771 | 0,274385426 | |
| 224058_s_at | HSD17B7P2 | -1,888254346 | -2,162639771 | 0,274385426 | |
| 230527_at | - | -1,888254346 | -2,162639771 | 0,274385426 | |
| 231485_at | LOC100505540 | -1,888254346 | -2,162639771 | 0,274385426 | |
| 234222_at | - | -1,888254346 | -2,162639771 | 0,274385426 | |
| 236299_at | - | -1,888254346 | -2,162639771 | 0,274385426 | |
| 240896_at | - | -1,888254346 | -2,162639771 | 0,274385426 | |
| 242924_at | - | -1,888254346 | -2,162639771 | 0,274385426 | |
| 243160_at | - | -1,888254346 | -2,162639771 | 0,274385426 | |
| 227025_at | PPHLN1 | 3,285189903 | 3,010820719 | 0,274369184 | |
| 233132_at | LOC96610 | 1,339784194 | 1,065442793 | 0,274341401 | |
| 1554697_at | ADAMTS9 | -1,763254337 | -2,037551727 | 0,27429739 | |
| 1554715_at | LINC00593 | -1,763254337 | -2,037551727 | 0,27429739 | |
| 1555245_s_at | RP1L1 | -1,763254337 | -2,037551727 | 0,27429739 | |
| 1557459_at | - | -1,763254337 | -2,037551727 | 0,27429739 | |
| 1558687_a_at | FOXN1 | -1,763254337 | -2,037551727 | 0,27429739 | |
| 1561192_at | - | -1,763254337 | -2,037551727 | 0,27429739 | |
| 1564083_at | - | -1,763254337 | -2,037551727 | 0,27429739 | |
| 1564963_x_at | ZNF92 | -1,763254337 | -2,037551727 | 0,27429739 | |
| 1565897_at | - | -1,763254337 | -2,037551727 | 0,27429739 | |
| 1570301_at | - | -1,763254337 | -2,037551727 | 0,27429739 | |
| 1570342_at | NKTR | -1,763254337 | -2,037551727 | 0,27429739 | |
| 205903_s_at | KCNN3 | -1,763254337 | -2,037551727 | 0,27429739 | |
| 207148_x_at | MYOZ2 | -1,763254337 | -2,037551727 | 0,27429739 | |
| 210634_at | KLHL20 | -1,763254337 | -2,037551727 | 0,27429739 | |
| 217500_at | TIAL1 | -1,763254337 | -2,037551727 | 0,27429739 | |
| 217666_at | - | -1,763254337 | -2,037551727 | 0,27429739 | |
| 219755_at | CBX8 | -1,763254337 | -2,037551727 | 0,27429739 | |
| 230349_at | XKRX | -1,763254337 | -2,037551727 | 0,27429739 | |
| 237523_at | - | -1,763254337 | -2,037551727 | 0,27429739 | |
| 243920_x_at | - | -1,763254337 | -2,037551727 | 0,27429739 | |
| 244329_at | - | -1,763254337 | -2,037551727 | 0,27429739 | |
| 209019_s_at | PINK1 | 1,314003737 | 1,039887232 | 0,274116505 | |
| 1552440_at | GPR182 | -1,937711681 | -2,211750327 | 0,274038646 | |
| 1561892_at | ZMYM6 | -1,937711681 | -2,211750327 | 0,274038646 | |
| 1569005_at | - | -1,937711681 | -2,211750327 | 0,274038646 | |
| 1569787_at | RFTN1 | -1,937711681 | -2,211750327 | 0,274038646 | |
| 202526_at | SMAD4 | -1,937711681 | -2,211750327 | 0,274038646 | |
| 203535_at | S100A9 | -1,937711681 | -2,211750327 | 0,274038646 | |
| 210089_s_at | LAMA4 | -1,937711681 | -2,211750327 | 0,274038646 | |
| 211333_s_at | FASLG | -1,937711681 | -2,211750327 | 0,274038646 | |
| 211339_s_at | ITK | -1,937711681 | -2,211750327 | 0,274038646 | |
| 214434_at | HSPA12A | -1,937711681 | -2,211750327 | 0,274038646 | |
| 214644_at | HIST1H2AK | -1,937711681 | -2,211750327 | 0,274038646 | |
| 215838_at | LILRA5 | -1,937711681 | -2,211750327 | 0,274038646 | |
| 216877_at | - | -1,937711681 | -2,211750327 | 0,274038646 | |
| 224225_s_at | ETV7 | -1,937711681 | -2,211750327 | 0,274038646 | |
| 226953_at | CADM3 /// DARC | -1,937711681 | -2,211750327 | 0,274038646 | |
| 227458_at | CD274 | -1,937711681 | -2,211750327 | 0,274038646 | |
| 231849_at | KRT80 | -1,937711681 | -2,211750327 | 0,274038646 | |
| 233414_at | - | -1,937711681 | -2,211750327 | 0,274038646 | |
| 234786_at | - | -1,937711681 | -2,211750327 | 0,274038646 | |
| 236009_at | PERP | -1,937711681 | -2,211750327 | 0,274038646 | |
| 237472_at | SOX1 | -1,937711681 | -2,211750327 | 0,274038646 | |

| | | | | | |
|---|---|---|---|---|---|
| 240956_at | - | -1,937711681 | -2,211750327 | 0,274038646 | |
| 241952_at | SLC16A11 | -1,937711681 | -2,211750327 | 0,274038646 | |
| 242212_at | RGS16 | -1,937711681 | -2,211750327 | 0,274038646 | |
| 243952_at | TPTEP1 | -1,937711681 | -2,211750327 | 0,274038646 | |
| 1556616_a_at | - | 0,171847695 | -0,102100538 | 0,273948233 | |
| 1561850_at | LOC100133669 | 0,171847695 | -0,102100538 | 0,273948233 | |
| 206991_s_at | CCR5 | 0,171847695 | -0,102100538 | 0,273948233 | |
| 218810_at | ZC3H12A | 0,171847695 | -0,102100538 | 0,273948233 | |
| 244534_at | - | 0,171847695 | -0,102100538 | 0,273948233 | |
| 204715_at | PANX1 | 1,85379461 | 1,579853228 | 0,273941381 | |
| 218130_at | C17orf62 | 1,85379461 | 1,579853228 | 0,273941381 | |
| 227822_at | ZNF605 | 2,094906514 | 1,820984782 | 0,273921733 | |
| 238990_x_at | TRIM61 | 1,079110292 | 0,805216064 | 0,273894228 | |
| 226019_at | OMA1 | 1,835789711 | 1,562009954 | 0,273779757 | |
| 220176_at | NUBPL | 1,274448124 | 1,000684521 | 0,273763603 | |
| 233378_at | - | 0,945567441 | 0,67181667 | 0,273750771 | |
| 216095_x_at | MTMR1 | 2,963199262 | 2,689475799 | 0,273723463 | |
| 217843_s_at | MED4 | 4,060873315 | 3,787346121 | 0,273527195 | |
| 219130_at | TRMT13 | 3,24026957 | 2,966746113 | 0,273523457 | |
| 206186_at | MPP3 | 0,496120222 | 0,222599095 | 0,273521127 | |
| 226222_at | KIAA1432 | 0,496120222 | 0,222599095 | 0,273521127 | |
| 212814_at | AHCYL2 | 2,048758794 | 1,775255987 | 0,273502807 | |
| 232740_at | MCM3AP-AS1 | 0,911589958 | 0,638150376 | 0,273439582 | |
| 1554545_at | APOBEC4 | -1,688127507 | -1,961547147 | 0,273419641 | |
| 1555722_at | SCAMPER | -1,688127507 | -1,961547147 | 0,273419641 | |
| 1564414_a_at | PNLDC1 | -1,688127507 | -1,961547147 | 0,273419641 | |
| 1569910_at | - | -1,688127507 | -1,961547147 | 0,273419641 | |
| 203540_at | GFAP | -1,688127507 | -1,961547147 | 0,273419641 | |
| 206721_at | C1orf114 | -1,688127507 | -1,961547147 | 0,273419641 | |
| 210636_at | PPARD | -1,688127507 | -1,961547147 | 0,273419641 | |
| 211055_s_at | INVS | -1,688127507 | -1,961547147 | 0,273419641 | |
| 215775_at | THBS1 | -1,688127507 | -1,961547147 | 0,273419641 | |
| 223800_s_at | LIMS3 /// LIMS3L | -1,688127507 | -1,961547147 | 0,273419641 | |
| 226637_at | UBE2H | -1,688127507 | -1,961547147 | 0,273419641 | |
| 227881_s_at | - | -1,688127507 | -1,961547147 | 0,273419641 | |
| 228406_at | SMYD1 | -1,688127507 | -1,961547147 | 0,273419641 | |
| 237212_at | - | -1,688127507 | -1,961547147 | 0,273419641 | |
| 240183_at | TMEM213 | -1,688127507 | -1,961547147 | 0,273419641 | |
| 242512_at | MYO9A | -1,688127507 | -1,961547147 | 0,273419641 | |
| 244008_at | PARP8 | -1,688127507 | -1,961547147 | 0,273419641 | |
| 1552798_a_at | TLR4 | -0,946847173 | -1,220210588 | 0,273363415 | |
| 1554554_at | CCDC57 | -0,946847173 | -1,220210588 | 0,273363415 | |
| 1569912_at | - | -0,946847173 | -1,220210588 | 0,273363415 | |
| 201262_s_at | BGN | -0,946847173 | -1,220210588 | 0,273363415 | |
| 205400_at | WAS | -0,946847173 | -1,220210588 | 0,273363415 | |
| 211184_s_at | USH1C | -0,946847173 | -1,220210588 | 0,273363415 | |
| 217029_at | - | -0,946847173 | -1,220210588 | 0,273363415 | |
| 220543_at | C21orf62 | -0,946847173 | -1,220210588 | 0,273363415 | |
| 224527_at | CDH23 /// LOC10 | -0,946847173 | -1,220210588 | 0,273363415 | |
| 227052_at | - | -0,946847173 | -1,220210588 | 0,273363415 | |
| 231347_at | - | -0,946847173 | -1,220210588 | 0,273363415 | |
| 232754_at | - | -0,946847173 | -1,220210588 | 0,273363415 | |
| 233510_s_at | PARVG | -0,946847173 | -1,220210588 | 0,273363415 | |
| 238169_at | - | -0,946847173 | -1,220210588 | 0,273363415 | |
| 240064_at | - | -0,946847173 | -1,220210588 | 0,273363415 | |
| 212156_at | VPS39 | 1,496984567 | 1,223626587 | 0,27335798 | |

| | | | | | |
|---|---|---|---|---|---|
| 213077_at | YTHDC2 | 2,646022818 | 2,372786857 | 0,273235961 | |
| 223225_s_at | SEH1L | 3,41111015 | 3,137910193 | 0,273199956 | |
| 224824_at | COX20 | 4,411731296 | 4,138648509 | 0,273082786 | |
| 213048_s_at | - | 5,521923587 | 5,249050572 | 0,272873015 | |
| 1559277_at | FLJ35700 | -2,01371626 | -2,286583451 | 0,27286719 | |
| 1562399_at | - | -2,01371626 | -2,286583451 | 0,27286719 | |
| 205366_s_at | HOXB6 | -2,01371626 | -2,286583451 | 0,27286719 | |
| 205472_s_at | DACH1 | -2,01371626 | -2,286583451 | 0,27286719 | |
| 208434_at | MECOM | -2,01371626 | -2,286583451 | 0,27286719 | |
| 209498_at | CEACAM1 | -2,01371626 | -2,286583451 | 0,27286719 | |
| 209686_at | S100B | -2,01371626 | -2,286583451 | 0,27286719 | |
| 210227_at | DLGAP2 | -2,01371626 | -2,286583451 | 0,27286719 | |
| 215249_at | RPL35A | -2,01371626 | -2,286583451 | 0,27286719 | |
| 215893_x_at | - | -2,01371626 | -2,286583451 | 0,27286719 | |
| 222836_at | NAA15 | -2,01371626 | -2,286583451 | 0,27286719 | |
| 228431_at | NFYA | -2,01371626 | -2,286583451 | 0,27286719 | |
| 229476_s_at | THRSP | -2,01371626 | -2,286583451 | 0,27286719 | |
| 229580_at | - | -2,01371626 | -2,286583451 | 0,27286719 | |
| 231257_at | TCERG1L | -2,01371626 | -2,286583451 | 0,27286719 | |
| 231444_at | LOC100506216 | -2,01371626 | -2,286583451 | 0,27286719 | |
| 233699_at | - | -2,01371626 | -2,286583451 | 0,27286719 | |
| 236851_x_at | - | -2,01371626 | -2,286583451 | 0,27286719 | |
| 240526_at | - | -2,01371626 | -2,286583451 | 0,27286719 | |
| 240816_at | - | -2,01371626 | -2,286583451 | 0,27286719 | |
| 241228_at | - | -2,01371626 | -2,286583451 | 0,27286719 | |
| 242654_at | FANCC | -2,01371626 | -2,286583451 | 0,27286719 | |
| 242752_at | - | -2,01371626 | -2,286583451 | 0,27286719 | |
| 244274_at | - | -2,01371626 | -2,286583451 | 0,27286719 | |
| 222175_s_at | MED15 | 1,177702228 | 0,904843258 | 0,272858969 | |
| 1559600_at | - | 0,052199481 | -0,220592223 | 0,272791704 | |
| 208505_s_at | FUT2 | 0,052199481 | -0,220592223 | 0,272791704 | |
| 214474_at | PRKAB2 | 0,052199481 | -0,220592223 | 0,272791704 | |
| 238056_at | SDHC | 0,052199481 | -0,220592223 | 0,272791704 | |
| 239217_x_at | ABCC3 | 0,052199481 | -0,220592223 | 0,272791704 | |
| 202326_at | EHMT2 | 0,841135803 | 0,568365095 | 0,272770708 | |
| 208960_s_at | KLF6 | 0,841135803 | 0,568365095 | 0,272770708 | |
| 201151_s_at | MBNL1 | 3,321224383 | 3,048471686 | 0,272752697 | |
| 48031_r_at | C5orf4 | 1,58225013 | 1,309546674 | 0,272703455 | |
| 1562431_x_at | - | -3,07502285 | -3,347710726 | 0,272687876 | |
| 221573_at | C7orf25 /// PSMA | 0,401366113 | 0,128747141 | 0,272618972 | |
| 201612_at | ALDH9A1 | 4,652328451 | 4,379770162 | 0,272558288 | |
| 1562265_at | - | -1,639579028 | -1,912089812 | 0,272510784 | |
| 1565407_at | LHX9 | -1,639579028 | -1,912089812 | 0,272510784 | |
| 1565868_at | CD44 | -1,639579028 | -1,912089812 | 0,272510784 | |
| 205081_at | CRIP1 | -1,639579028 | -1,912089812 | 0,272510784 | |
| 207341_at | PRTN3 | -1,639579028 | -1,912089812 | 0,272510784 | |
| 214650_x_at | MOG | -1,639579028 | -1,912089812 | 0,272510784 | |
| 215259_s_at | CADM4 | -1,639579028 | -1,912089812 | 0,272510784 | |
| 216046_at | - | -1,639579028 | -1,912089812 | 0,272510784 | |
| 222484_s_at | CXCL14 | -1,639579028 | -1,912089812 | 0,272510784 | |
| 227734_s_at | ZNHIT2 | -1,639579028 | -1,912089812 | 0,272510784 | |
| 231935_at | ARPP21 | -1,639579028 | -1,912089812 | 0,272510784 | |
| 233708_at | - | -1,639579028 | -1,912089812 | 0,272510784 | |
| 237542_at | - | -1,639579028 | -1,912089812 | 0,272510784 | |
| 237916_at | - | -1,639579028 | -1,912089812 | 0,272510784 | |
| 239494_at | - | -1,639579028 | -1,912089812 | 0,272510784 | |

| | | | | | |
|---|---|---|---|---|---|
| 239714_at | - | -1,639579028 | -1,912089812 | 0,272510784 | |
| 243182_at | - | -1,639579028 | -1,912089812 | 0,272510784 | |
| 243357_at | NEGR1 | -1,639579028 | -1,912089812 | 0,272510784 | |
| 244026_at | - | -1,639579028 | -1,912089812 | 0,272510784 | |
| 220171_x_at | KIAA1704 | 2,889473182 | 2,617011906 | 0,272461276 | |
| 1563754_at | GRIK2 | -2,866296344 | -3,138626879 | 0,272330535 | |
| 1564160_at | DTHD1 | -2,866296344 | -3,138626879 | 0,272330535 | |
| 1569566_at | TBC1D1 | -2,866296344 | -3,138626879 | 0,272330535 | |
| 1570302_at | - | -2,866296344 | -3,138626879 | 0,272330535 | |
| 206624_at | USP9Y | -2,866296344 | -3,138626879 | 0,272330535 | |
| 232010_at | FSTL5 | -2,866296344 | -3,138626879 | 0,272330535 | |
| 240050_s_at | - | -2,866296344 | -3,138626879 | 0,272330535 | |
| 204490_s_at | CD44 | 1,683039483 | 1,410714371 | 0,272325111 | |
| 222422_s_at | NDFIP1 | 1,683039483 | 1,410714371 | 0,272325111 | |
| 235588_at | ESCO2 | 1,683039483 | 1,410714371 | 0,272325111 | |
| 226899_at | UNC5B | 2,022468633 | 1,750261389 | 0,272207245 | |
| 1553220_at | FAM117B | 0,351548291 | 0,079428135 | 0,272120156 | |
| 205536_at | VAV2 | 0,351548291 | 0,079428135 | 0,272120156 | |
| 214428_x_at | C4A /// C4B /// LC | 0,351548291 | 0,079428135 | 0,272120156 | |
| 230003_at | - | 0,351548291 | 0,079428135 | 0,272120156 | |
| 242938_s_at | FOXK2 | 0,351548291 | 0,079428135 | 0,272120156 | |
| 226513_at | ASB7 | 1,844820248 | 1,572742379 | 0,272077869 | |
| 200034_s_at | RPL6 | 7,737656461 | 7,465596184 | 0,272060277 | |
| 1554514_at | ACSM5 | -2,063149277 | -2,335104118 | 0,27195484 | |
| 1555298_a_at | VWA3B | -2,063149277 | -2,335104118 | 0,27195484 | |
| 1556381_at | NAA15 | -2,063149277 | -2,335104118 | 0,27195484 | |
| 214018_at | GRIP1 | -2,063149277 | -2,335104118 | 0,27195484 | |
| 217423_at | - | -2,063149277 | -2,335104118 | 0,27195484 | |
| 219858_s_at | MFSD6 | -2,063149277 | -2,335104118 | 0,27195484 | |
| 220148_at | ALDH8A1 | -2,063149277 | -2,335104118 | 0,27195484 | |
| 231128_at | FAM181B | -2,063149277 | -2,335104118 | 0,27195484 | |
| 232998_at | TIGD4 | -2,063149277 | -2,335104118 | 0,27195484 | |
| 236323_at | PVRL3-AS1 | -2,063149277 | -2,335104118 | 0,27195484 | |
| 237936_at | - | -2,063149277 | -2,335104118 | 0,27195484 | |
| 238048_at | - | -2,063149277 | -2,335104118 | 0,27195484 | |
| 238744_at | - | -2,063149277 | -2,335104118 | 0,27195484 | |
| 240047_at | - | -2,063149277 | -2,335104118 | 0,27195484 | |
| 240856_at | O3FAR1 | -2,063149277 | -2,335104118 | 0,27195484 | |
| 241659_at | JMJD1C | -2,063149277 | -2,335104118 | 0,27195484 | |
| 244031_at | - | -2,063149277 | -2,335104118 | 0,27195484 | |
| 223215_s_at | JKAMP | 4,537271727 | 4,265343974 | 0,271927753 | |
| 200854_at | NCOR1 | 2,807162789 | 2,53523821 | 0,271924579 | |
| 227237_x_at | ATAD3B | 3,547344317 | 3,275657468 | 0,271686848 | |
| 1555274_a_at | EPT1 | 3,21379258 | 2,942125533 | 0,271667046 | |
| 227285_at | C1orf51 | 0,728550685 | 0,456918297 | 0,271632388 | |
| 213836_s_at | WIPI1 | 1,789769151 | 1,518263577 | 0,271505574 | |
| 209181_s_at | RABGGTB /// SN | 5,644425553 | 5,372936243 | 0,27148931 | |
| 1553725_s_at | ZNF644 | 1,530951994 | 1,259496994 | 0,271454999 | |
| 228039_at | DDX46 | 1,530951994 | 1,259496994 | 0,271454999 | |
| 226521_s_at | FAM175A | 2,72965419 | 2,458228496 | 0,271425694 | |
| 1553224_at | LUZP1 | -1,591296941 | -1,862647763 | 0,271350821 | |
| 1555392_at | - | -1,591296941 | -1,862647763 | 0,271350821 | |
| 1558607_at | PAPPA | -1,591296941 | -1,862647763 | 0,271350821 | |
| 1558698_at | ZNF264 | -1,591296941 | -1,862647763 | 0,271350821 | |
| 1559686_a_at | LOC100506379 | -1,591296941 | -1,862647763 | 0,271350821 | |
| 1562099_at | - | -1,591296941 | -1,862647763 | 0,271350821 | |

| | | | | | |
|---|---|---|---|---|---|
| 201283_s_at | TRAK1 | -1,591296941 | -1,862647763 | 0,271350821 | |
| 203407_at | PPL | -1,591296941 | -1,862647763 | 0,271350821 | |
| 206605_at | ENDOU | -1,591296941 | -1,862647763 | 0,271350821 | |
| 206663_at | SP4 | -1,591296941 | -1,862647763 | 0,271350821 | |
| 207972_at | GLRA1 | -1,591296941 | -1,862647763 | 0,271350821 | |
| 208228_s_at | FGFR2 | -1,591296941 | -1,862647763 | 0,271350821 | |
| 210791_s_at | ARHGAP32 | -1,591296941 | -1,862647763 | 0,271350821 | |
| 211045_s_at | KCNH6 | -1,591296941 | -1,862647763 | 0,271350821 | |
| 211372_s_at | IL1R2 | -1,591296941 | -1,862647763 | 0,271350821 | |
| 213898_at | RBFOX2 | -1,591296941 | -1,862647763 | 0,271350821 | |
| 214366_s_at | ALOX5 | -1,591296941 | -1,862647763 | 0,271350821 | |
| 222086_s_at | WNT6 | -1,591296941 | -1,862647763 | 0,271350821 | |
| 231687_at | - | -1,591296941 | -1,862647763 | 0,271350821 | |
| 233187_s_at | - | -1,591296941 | -1,862647763 | 0,271350821 | |
| 243133_at | TSC22D1 | -1,591296941 | -1,862647763 | 0,271350821 | |
| 206928_at | ZNF124 | 2,061726373 | 1,790660532 | 0,271065841 | |
| 1557170_at | NEK8 | 0,246434561 | -0,024572586 | 0,271007147 | |
| 207766_at | CDKL1 | 0,246434561 | -0,024572586 | 0,271007147 | |
| 219627_at | ZNF767 | 0,995078803 | 0,724100169 | 0,270978634 | |
| 1555247_a_at | FNIP1 /// RAPGE | 2,59162628 | 2,320681318 | 0,270944962 | |
| 225658_at | SPOPL | 1,732533761 | 1,461646549 | 0,270887212 | |
| 208012_x_at | SP110 | 3,046446467 | 2,775606361 | 0,270840106 | |
| 239346_at | - | 3,569303935 | 3,298466943 | 0,270836991 | |
| 210257_x_at | CUL4B | 2,842156496 | 2,571362843 | 0,270793653 | |
| 226832_at | RNF168 | 2,943717636 | 2,672945617 | 0,270772019 | |
| 204180_s_at | ZBTB43 | 0,648296226 | 0,377530647 | 0,270765579 | |
| 204629_at | PARVB | 0,648296226 | 0,377530647 | 0,270765579 | |
| 219314_s_at | ZNF219 | 0,648296226 | 0,377530647 | 0,270765579 | |
| 219530_at | PALB2 | 2,637386731 | 2,366632651 | 0,27075408 | |
| 204618_s_at | GABPB1 | 2,861566439 | 2,590852465 | 0,270713974 | |
| 222448_s_at | CMPK1 | 4,06796029 | 3,797294336 | 0,270665954 | |
| 208649_s_at | VCP | 4,342753543 | 4,072129637 | 0,270623905 | |
| 235346_at | FUNDC1 | 4,085209408 | 3,814728712 | 0,270480696 | |
| 1554178_a_at | FAM126B | -0,148196756 | -0,41858459 | 0,270387833 | |
| 231481_at | CCNB3 | -0,148196756 | -0,41858459 | 0,270387833 | |
| 229013_at | LOC145783 | 1,360921491 | 1,090553537 | 0,270367954 | |
| 232076_at | ZNF707 | 0,606430281 | 0,336136178 | 0,270294104 | |
| 203739_at | ZNF217 | 3,831009889 | 3,560823412 | 0,270186477 | |
| 222553_x_at | OXR1 | 3,761974695 | 3,491815843 | 0,270158852 | |
| 213459_at | RPL37A | 2,236584753 | 1,966439221 | 0,270145532 | |
| 1555922_at | C10orf114 | 1,758252436 | 1,488344553 | 0,269907884 | |
| 1555963_x_at | B3GNT7 | -2,138804305 | -2,408705138 | 0,269900832 | |
| 1558658_at | ZNF391 | -2,138804305 | -2,408705138 | 0,269900832 | |
| 1560642_at | - | -2,138804305 | -2,408705138 | 0,269900832 | |
| 1562372_at | SV2C | -2,138804305 | -2,408705138 | 0,269900832 | |
| 1563614_at | MTBP | -2,138804305 | -2,408705138 | 0,269900832 | |
| 1569931_at | - | -2,138804305 | -2,408705138 | 0,269900832 | |
| 209396_s_at | CHI3L1 | -2,138804305 | -2,408705138 | 0,269900832 | |
| 211883_x_at | CEACAM1 | -2,138804305 | -2,408705138 | 0,269900832 | |
| 214945_at | FAM153A /// FAM | -2,138804305 | -2,408705138 | 0,269900832 | |
| 214954_at | SUSD5 | -2,138804305 | -2,408705138 | 0,269900832 | |
| 216148_at | - | -2,138804305 | -2,408705138 | 0,269900832 | |
| 229634_at | TMEM139 | -2,138804305 | -2,408705138 | 0,269900832 | |
| 230861_at | DKFZP434L187 | -2,138804305 | -2,408705138 | 0,269900832 | |
| 233497_at | - | -2,138804305 | -2,408705138 | 0,269900832 | |
| 236645_at | LOC100506312 | -2,138804305 | -2,408705138 | 0,269900832 | |

| | | | | | |
|---|---|---|---|---|---|
| 240025_x_at | - | -2,138804305 | -2,408705138 | 0,269900832 | |
| 244670_at | - | -2,138804305 | -2,408705138 | 0,269900832 | |
| 213599_at | OIP5 | 4,00557508 | 3,735753654 | 0,269821426 | |
| 202346_at | UBE2K | 4,256842836 | 3,987098381 | 0,269744455 | |
| 223429_x_at | ISY1 | 3,62627949 | 3,356551164 | 0,269728326 | |
| 223925_s_at | - | 1,894952274 | 1,6252386 | 0,269713673 | |
| 228991_at | CDK13 | 1,894952274 | 1,6252386 | 0,269713673 | |
| 209179_s_at | MBOAT7 | 3,984619786 | 3,714911743 | 0,269708043 | |
| 243280_at | - | 0,133056859 | -0,136643983 | 0,269700841 | |
| 221513_s_at | UTP14A /// UTP1 | 2,310153708 | 2,040470494 | 0,269683214 | |
| 223253_at | EPDR1 | 3,882346871 | 3,6128809 | 0,269465972 | |
| 1561651_s_at | TAL1 | 0,859074518 | 0,589657387 | 0,269417131 | |
| 225919_s_at | C9orf72 | 0,859074518 | 0,589657387 | 0,269417131 | |
| 218215_s_at | NR1H2 | 1,542099044 | 1,272721751 | 0,269377293 | |
| 218278_at | WDR74 | 3,536237877 | 3,266872397 | 0,26936548 | |
| 203165_s_at | SLC33A1 | 2,003776245 | 1,73441697 | 0,269359275 | |
| 203226_s_at | TSPAN31 | 2,003776245 | 1,73441697 | 0,269359275 | |
| 212060_at | U2SURP | 3,373122675 | 3,103784293 | 0,269338382 | |
| 227402_s_at | UTP23 | 3,373122675 | 3,103784293 | 0,269338382 | |
| 203767_s_at | STS | -0,221656139 | -0,490927335 | 0,269271197 | |
| 206703_at | CHRNB1 | -0,221656139 | -0,490927335 | 0,269271197 | |
| 208299_at | CACNA1I | -0,221656139 | -0,490927335 | 0,269271197 | |
| 216887_s_at | LDB3 | -0,221656139 | -0,490927335 | 0,269271197 | |
| 229112_at | SIRT5 | -0,221656139 | -0,490927335 | 0,269271197 | |
| 237388_at | - | -0,221656139 | -0,490927335 | 0,269271197 | |
| 241084_x_at | DYNC1H1 | -0,221656139 | -0,490927335 | 0,269271197 | |
| 232103_at | BPNT1 | 1,104397092 | 0,835239052 | 0,26915804 | |
| 201352_at | YME1L1 | 5,629479193 | 5,360344676 | 0,269134517 | |
| 225094_at | SETD8 | 2,227406889 | 1,958277216 | 0,269129673 | |
| 1555033_a_at | RGS12 | -1,518072576 | -1,787089803 | 0,269017228 | |
| 1556801_at | LOC400794 | -1,518072576 | -1,787089803 | 0,269017228 | |
| 1561056_a_at | - | -1,518072576 | -1,787089803 | 0,269017228 | |
| 1564821_at | - | -1,518072576 | -1,787089803 | 0,269017228 | |
| 1569644_at | - | -1,518072576 | -1,787089803 | 0,269017228 | |
| 207866_at | BMP8A | -1,518072576 | -1,787089803 | 0,269017228 | |
| 219734_at | SIDT1 | -1,518072576 | -1,787089803 | 0,269017228 | |
| 221312_at | GLP2R | -1,518072576 | -1,787089803 | 0,269017228 | |
| 226534_at | KITLG | -1,518072576 | -1,787089803 | 0,269017228 | |
| 228025_s_at | PPP2R2C | -1,518072576 | -1,787089803 | 0,269017228 | |
| 231183_s_at | JAG1 | -1,518072576 | -1,787089803 | 0,269017228 | |
| 232538_at | - | -1,518072576 | -1,787089803 | 0,269017228 | |
| 234931_at | - | -1,518072576 | -1,787089803 | 0,269017228 | |
| 236034_at | ANGPT2 | -1,518072576 | -1,787089803 | 0,269017228 | |
| 237204_at | - | -1,518072576 | -1,787089803 | 0,269017228 | |
| 239705_at | - | -1,518072576 | -1,787089803 | 0,269017228 | |
| 241383_at | ZNF385C | -1,518072576 | -1,787089803 | 0,269017228 | |
| 227096_at | JOSD2 | 0,822971223 | 0,553993624 | 0,268977599 | |
| 1552987_a_at | MGC42157 | -2,908843709 | -3,177802286 | 0,268958577 | |
| 1559930_at | - | -2,908843709 | -3,177802286 | 0,268958577 | |
| 1566551_at | - | -2,908843709 | -3,177802286 | 0,268958577 | |
| 1569213_at | LOC400891 | -2,908843709 | -3,177802286 | 0,268958577 | |
| 215314_at | - | -2,908843709 | -3,177802286 | 0,268958577 | |
| 231969_at | STOX2 | -2,908843709 | -3,177802286 | 0,268958577 | |
| 243281_at | - | -2,908843709 | -3,177802286 | 0,268958577 | |
| 1560986_a_at | - | 0,072842263 | -0,19610998 | 0,268952243 | |
| 200733_s_at | PTP4A1 | 5,647439343 | 5,378554049 | 0,268885294 | |

| | | | | | |
|---|---|---|---|---|---|
| 215980_s_at | IGHMBP2 | 0,702292136 | 0,433557298 | 0,268734838 | |
| 201899_s_at | UBE2A | 4,325653301 | 4,056921713 | 0,268731588 | |
| 202211_at | ARFGAP3 | 4,114752306 | 3,846031735 | 0,268720571 | |
| 223584_s_at | KBTBD2 | 1,422536444 | 1,153866761 | 0,268669683 | |
| 235162_at | MDM4 | 0,97328325 | 0,704715219 | 0,268568031 | |
| 242727_at | ARL5B | 0,97328325 | 0,704715219 | 0,268568031 | |
| 1553430_a_at | EDARADD | -0,741668563 | -1,010192375 | 0,268523812 | |
| 1563560_at | AHNAK | -0,741668563 | -1,010192375 | 0,268523812 | |
| 214251_s_at | NUMA1 | -0,741668563 | -1,010192375 | 0,268523812 | |
| 216879_at | HR44 | -0,741668563 | -1,010192375 | 0,268523812 | |
| 220912_at | - | -0,741668563 | -1,010192375 | 0,268523812 | |
| 223751_x_at | TLR10 | -0,741668563 | -1,010192375 | 0,268523812 | |
| 223953_s_at | ZBTB37 | -0,741668563 | -1,010192375 | 0,268523812 | |
| 230084_at | SLC30A2 | -0,741668563 | -1,010192375 | 0,268523812 | |
| 233998_x_at | - | -0,741668563 | -1,010192375 | 0,268523812 | |
| 236311_at | LOH12CR2 | -0,741668563 | -1,010192375 | 0,268523812 | |
| 239097_at | FRRS1L | -0,741668563 | -1,010192375 | 0,268523812 | |
| 242213_at | - | -0,741668563 | -1,010192375 | 0,268523812 | |
| 242308_at | MCOLN3 | -0,741668563 | -1,010192375 | 0,268523812 | |
| 242593_at | - | -0,741668563 | -1,010192375 | 0,268523812 | |
| 204250_s_at | CEP164 | -0,496991414 | -0,765504029 | 0,268512615 | |
| 207275_s_at | ACSL1 | -0,496991414 | -0,765504029 | 0,268512615 | |
| 216174_at | HCRP1 | -0,496991414 | -0,765504029 | 0,268512615 | |
| 230616_at | LAMB2P1 | -0,496991414 | -0,765504029 | 0,268512615 | |
| 230862_at | - | -0,496991414 | -0,765504029 | 0,268512615 | |
| 232857_at | POLR3H | -0,496991414 | -0,765504029 | 0,268512615 | |
| 1561759_at | LOC100507322 / | -2,187914861 | -2,456406426 | 0,268491565 | |
| 1562822_at | - | -2,187914861 | -2,456406426 | 0,268491565 | |
| 1563171_at | - | -2,187914861 | -2,456406426 | 0,268491565 | |
| 1564067_x_at | TMEM151B | -2,187914861 | -2,456406426 | 0,268491565 | |
| 1564075_a_at | C17orf104 | -2,187914861 | -2,456406426 | 0,268491565 | |
| 1567687_at | CECR9 | -2,187914861 | -2,456406426 | 0,268491565 | |
| 214461_at | LBP | -2,187914861 | -2,456406426 | 0,268491565 | |
| 216643_at | - | -2,187914861 | -2,456406426 | 0,268491565 | |
| 217320_at | - | -2,187914861 | -2,456406426 | 0,268491565 | |
| 225817_at | CGNL1 | -2,187914861 | -2,456406426 | 0,268491565 | |
| 233927_at | - | -2,187914861 | -2,456406426 | 0,268491565 | |
| 234085_at | CCDC169 | -2,187914861 | -2,456406426 | 0,268491565 | |
| 234243_at | RPF1 | -2,187914861 | -2,456406426 | 0,268491565 | |
| 243988_at | - | -2,187914861 | -2,456406426 | 0,268491565 | |
| 36711_at | MAFF | 3,337310811 | 3,068831572 | 0,26847924 | |
| 231864_at | ZNF33A | 1,638726528 | 1,370274469 | 0,268452059 | |
| 1555772_a_at | CDC25A | 1,201332287 | 0,932882274 | 0,268450013 | |
| 208523_x_at | HIST1H2BC /// H | 4,895163777 | 4,626777417 | 0,26838636 | |
| 201798_s_at | MYOF | 0,661985843 | 0,393761504 | 0,268224338 | |
| 206219_s_at | VAV1 | 0,661985843 | 0,393761504 | 0,268224338 | |
| 200979_at | PDHA1 | 3,101484927 | 2,833274877 | 0,26821005 | |
| 201889_at | FAM3C | 5,950110119 | 5,681974705 | 0,268135414 | |
| 206554_x_at | SETMAR | 0,425644434 | 0,157549243 | 0,268095191 | |
| 241411_at | - | 0,425644434 | 0,157549243 | 0,268095191 | |
| 203480_s_at | OTUD4 | 3,69530589 | 3,427247355 | 0,268058535 | |
| 1556739_at | GOLGA8IP | -0,299034815 | -0,566887459 | 0,267852645 | |
| 1563607_x_at | LOC286359 | -0,299034815 | -0,566887459 | 0,267852645 | |
| 230640_at | - | -0,299034815 | -0,566887459 | 0,267852645 | |
| 234135_x_at | - | -0,299034815 | -0,566887459 | 0,267852645 | |
| 237136_at | - | -0,299034815 | -0,566887459 | 0,267852645 | |

| | | | | | |
|---|---|---|---|---|---|
| 226083_at | TMEM70 | 1,960118853 | 1,692388143 | 0,267730711 | |
| 211217_s_at | KCNQ1 | 1,348276312 | 1,080561626 | 0,267714686 | |
| 203289_s_at | NPRL3 | 1,143956113 | 0,876248481 | 0,267707632 | |
| 203542_s_at | KLF9 | 1,143956113 | 0,876248481 | 0,267707632 | |
| 224248_x_at | FAM192A | 2,760204767 | 2,492544876 | 0,267659891 | |
| 211988_at | SMARCE1 | 4,604390697 | 4,336867701 | 0,267522995 | |
| 202069_s_at | IDH3A | 3,621908408 | 3,354479478 | 0,267428931 | |
| 226016_at | CD47 | 3,791855421 | 3,524430539 | 0,267424882 | |
| 219056_at | RNASEH2B | 1,504602299 | 1,23718272 | 0,267419579 | |
| 230141_at | ARID4A | 1,504602299 | 1,23718272 | 0,267419579 | |
| 202090_s_at | UQCR11 | 5,924161742 | 5,656766959 | 0,267394783 | |
| 220943_s_at | C2orf56 | 1,114389003 | 0,847075464 | 0,26731354 | |
| 228090_at | NMNAT3 | 1,926486359 | 1,659204017 | 0,267282343 | |
| 1560706_at | NEDD9 | -1,470833215 | -1,738067356 | 0,267234141 | |
| 1569318_at | LOC284440 | -1,470833215 | -1,738067356 | 0,267234141 | |
| 206021_at | SCAND2 | -1,470833215 | -1,738067356 | 0,267234141 | |
| 207009_at | PHOX2B | -1,470833215 | -1,738067356 | 0,267234141 | |
| 208028_s_at | GPX5 | -1,470833215 | -1,738067356 | 0,267234141 | |
| 210660_at | LILRA1 | -1,470833215 | -1,738067356 | 0,267234141 | |
| 211147_s_at | P2RX6 | -1,470833215 | -1,738067356 | 0,267234141 | |
| 215104_at | NRIP2 | -1,470833215 | -1,738067356 | 0,267234141 | |
| 216118_at | - | -1,470833215 | -1,738067356 | 0,267234141 | |
| 220902_at | - | -1,470833215 | -1,738067356 | 0,267234141 | |
| 229767_at | TBCD | -1,470833215 | -1,738067356 | 0,267234141 | |
| 230499_at | BIRC3 | -1,470833215 | -1,738067356 | 0,267234141 | |
| 230911_at | SIX1 | -1,470833215 | -1,738067356 | 0,267234141 | |
| 234838_at | - | -1,470833215 | -1,738067356 | 0,267234141 | |
| 238881_at | - | -1,470833215 | -1,738067356 | 0,267234141 | |
| 240740_at | LOC100507443 | -1,470833215 | -1,738067356 | 0,267234141 | |
| 243192_at | - | -1,470833215 | -1,738067356 | 0,267234141 | |
| 243456_at | ZNF214 | -1,470833215 | -1,738067356 | 0,267234141 | |
| 244709_at | FAM123C | -1,470833215 | -1,738067356 | 0,267234141 | |
| 222571_at | ST6GALNAC6 | 0,87091093 | 0,603679647 | 0,267231283 | |
| 1556328_at | - | -0,055687482 | -0,322870281 | 0,267182799 | |
| 220808_at | THEG | -0,055687482 | -0,322870281 | 0,267182799 | |
| 230254_at | FAM26E | -0,055687482 | -0,322870281 | 0,267182799 | |
| 235167_at | LOC100190986 | -0,055687482 | -0,322870281 | 0,267182799 | |
| 236807_at | - | -0,055687482 | -0,322870281 | 0,267182799 | |
| 240381_at | ZBED5 | -0,055687482 | -0,322870281 | 0,267182799 | |
| 1554153_a_at | PHF21A | 0,57782909 | 0,310717132 | 0,267111959 | |
| 1554401_a_at | TCTE3 | -2,236765673 | -2,503677622 | 0,26691195 | |
| 1556706_at | - | -2,236765673 | -2,503677622 | 0,26691195 | |
| 1559911_at | - | -2,236765673 | -2,503677622 | 0,26691195 | |
| 1563112_at | - | -2,236765673 | -2,503677622 | 0,26691195 | |
| 1563985_at | - | -2,236765673 | -2,503677622 | 0,26691195 | |
| 1570231_at | LATS1 | -2,236765673 | -2,503677622 | 0,26691195 | |
| 217090_at | - | -2,236765673 | -2,503677622 | 0,26691195 | |
| 228665_at | CYYR1 | -2,236765673 | -2,503677622 | 0,26691195 | |
| 231956_at | RNF213 | -2,236765673 | -2,503677622 | 0,26691195 | |
| 233581_at | - | -2,236765673 | -2,503677622 | 0,26691195 | |
| 237564_at | - | -2,236765673 | -2,503677622 | 0,26691195 | |
| 238891_at | - | -2,236765673 | -2,503677622 | 0,26691195 | |
| 241145_at | - | -2,236765673 | -2,503677622 | 0,26691195 | |
| 213049_at | RALGAPA1 | 2,901063789 | 2,634191677 | 0,266872112 | |
| 225640_at | LOC100506710 | 2,764969948 | 2,498185745 | 0,266784203 | |
| 228652_at | ZNF776 | 3,319065909 | 3,052311115 | 0,266754794 | |

| | | | | | |
|---|---|---|---|---|---|
| 227200_at | ETV3 | 3,188003814 | 2,921282473 | 0,266721341 | |
| 202314_at | CYP51A1 /// LRR | 3,586454644 | 3,319860027 | 0,266594617 | |
| 209804_at | DCLRE1A | 1,381753566 | 1,115234684 | 0,266518882 | |
| 208424_s_at | CIAPIN1 | 3,327680494 | 3,061230246 | 0,266450248 | |
| 227410_at | FAM43A | 1,735773781 | 1,469325098 | 0,266448683 | |
| 1559715_at | LOC100507391 | -0,888496123 | -1,154897679 | 0,266401556 | |
| 1560698_a_at | TRHDE-AS1 | -0,888496123 | -1,154897679 | 0,266401556 | |
| 1564548_at | LOC642620 | -0,888496123 | -1,154897679 | 0,266401556 | |
| 207093_s_at | OMG | -0,888496123 | -1,154897679 | 0,266401556 | |
| 211327_x_at | HFE | -0,888496123 | -1,154897679 | 0,266401556 | |
| 213023_at | UTRN | -0,888496123 | -1,154897679 | 0,266401556 | |
| 220598_at | CARD14 | -0,888496123 | -1,154897679 | 0,266401556 | |
| 229683_s_at | KCTD15 | -0,888496123 | -1,154897679 | 0,266401556 | |
| 231944_at | ERO1LB | -0,888496123 | -1,154897679 | 0,266401556 | |
| 240040_at | PSTK | -0,888496123 | -1,154897679 | 0,266401556 | |
| 241064_at | - | -0,888496123 | -1,154897679 | 0,266401556 | |
| 242896_at | - | -0,888496123 | -1,154897679 | 0,266401556 | |
| 242949_x_at | CCDC157 | -0,888496123 | -1,154897679 | 0,266401556 | |
| 244375_at | - | -0,888496123 | -1,154897679 | 0,266401556 | |
| 205415_s_at | ATXN3 | 2,485917452 | 2,21959457 | 0,266322881 | |
| 205787_x_at | ZC3H11A | 0,798390451 | 0,532164371 | 0,26622608 | |
| 220243_at | ZBTB44 | 0,798390451 | 0,532164371 | 0,26622608 | |
| 241606_s_at | TRUB1 | 0,798390451 | 0,532164371 | 0,26622608 | |
| 227084_at | DTNA | 1,968405881 | 1,702196344 | 0,266209537 | |
| 228365_at | CPNE8 | 1,968405881 | 1,702196344 | 0,266209537 | |
| 202768_at | FOSB | 1,512180019 | 1,246149888 | 0,266030131 | |
| 242304_at | WIBG | 1,512180019 | 1,246149888 | 0,266030131 | |
| 1556560_a_at | LOC100128071 | 0,273439642 | 0,007421914 | 0,266017728 | |
| 205655_at | MDM4 | 0,273439642 | 0,007421914 | 0,266017728 | |
| 232819_s_at | LTBR | 0,273439642 | 0,007421914 | 0,266017728 | |
| 224571_at | IRF2BP2 | 3,013454777 | 2,747463672 | 0,265991105 | |
| 226811_at | FAM46C | 3,590934306 | 3,325159096 | 0,26577521 | |
| 240231_at | - | 2,596981013 | 2,331254065 | 0,265726949 | |
| 226239_at | TMEM150A | 0,760715727 | 0,495031851 | 0,265683876 | |
| 214582_at | PDE3B | 1,187200746 | 0,921731975 | 0,265468771 | |
| 235286_at | - | 1,187200746 | 0,921731975 | 0,265468771 | |
| 220721_at | ZNF614 | 1,305306849 | 1,039887232 | 0,265419618 | |
| 223361_at | ABRACL | 5,053175616 | 4,787816863 | 0,265358754 | |
| 202261_at | VPS72 | 3,248232277 | 2,982929462 | 0,265302814 | |
| 1562271_x_at | ARHGEF7 | -0,59053611 | -0,855751026 | 0,265214916 | |
| 205809_s_at | WASL | -0,59053611 | -0,855751026 | 0,265214916 | |
| 210923_at | SLC1A7 | -0,59053611 | -0,855751026 | 0,265214916 | |
| 216464_x_at | PTGDR2 | -0,59053611 | -0,855751026 | 0,265214916 | |
| 219586_at | WDR59 | -0,59053611 | -0,855751026 | 0,265214916 | |
| 227506_at | SLC16A9 | -0,59053611 | -0,855751026 | 0,265214916 | |
| 230439_at | LOC389458 /// R | -0,59053611 | -0,855751026 | 0,265214916 | |
| 237178_at | LOC100506414 | -0,59053611 | -0,855751026 | 0,265214916 | |
| 239882_at | - | -0,59053611 | -0,855751026 | 0,265214916 | |
| 244882_at | TNRC18 | -0,59053611 | -0,855751026 | 0,265214916 | |
| 1566171_at | RFFL | 0,44160593 | 0,176436073 | 0,265169857 | |
| 223937_at | FOXP1 | 0,44160593 | 0,176436073 | 0,265169857 | |
| 201223_s_at | RAD23B | 4,807039113 | 4,541934651 | 0,265104462 | |
| 217859_s_at | SLC39A9 | 0,95671774 | 0,691645685 | 0,265072055 | |
| 228618_at | PEAR1 | 0,95671774 | 0,691645685 | 0,265072055 | |
| 239010_at | LOC100653149 | 0,95671774 | 0,691645685 | 0,265072055 | |
| 208865_at | CSNK1A1 | 5,055446416 | 4,790501109 | 0,264945307 | |

| | | | | | |
|---|---|---|---|---|---|
| 201019_s_at | EIF1AX | 6,033929877 | 5,769027199 | 0,264902678 | |
| 1558184_s_at | ZNF17 | -0,196756757 | -0,461594427 | 0,264837669 | |
| 209359_x_at | LOC100506403 / | -0,196756757 | -0,461594427 | 0,264837669 | |
| 224530_s_at | KCNIP4 | -0,543019821 | -0,807717948 | 0,264698127 | |
| 215984_s_at | ARFRP1 | 1,614289682 | 1,349621332 | 0,26466835 | |
| 239050_s_at | - | 1,614289682 | 1,349621332 | 0,26466835 | |
| 225166_at | ARHGAP18 | 1,251994932 | 0,987376598 | 0,264618335 | |
| 226136_at | GLIPR1 | 1,251994932 | 0,987376598 | 0,264618335 | |
| 212177_at | PNISR | 1,726031811 | 1,461646549 | 0,264385261 | |
| 225038_s_at | SURF6 | 2,406821352 | 2,14244844 | 0,264372913 | |
| 226886_at | GFPT1 | 2,747420128 | 2,483094137 | 0,264325991 | |
| 1569409_x_at | - | 1,224581536 | 0,960386719 | 0,264194816 | |
| 216049_at | RHOBTB3 | 1,224581536 | 0,960386719 | 0,264194816 | |
| 222517_at | AP3M1 | 1,789769151 | 1,525647381 | 0,26412177 | |
| 225955_at | METRNL | 1,789769151 | 1,525647381 | 0,26412177 | |
| 238218_at | OOEP | 2,16388051 | 1,899812471 | 0,264068039 | |
| 227796_at | ZFP62 | 2,355089531 | 2,091116676 | 0,263972855 | |
| 1560794_at | - | -1,399512875 | -1,663414495 | 0,263901619 | |
| 1562907_at | - | -1,399512875 | -1,663414495 | 0,263901619 | |
| 1563295_at | - | -1,399512875 | -1,663414495 | 0,263901619 | |
| 1563839_at | TBC1D7 | -1,399512875 | -1,663414495 | 0,263901619 | |
| 1568803_at | - | -1,399512875 | -1,663414495 | 0,263901619 | |
| 1568865_at | FNTB | -1,399512875 | -1,663414495 | 0,263901619 | |
| 205336_at | PVALB | -1,399512875 | -1,663414495 | 0,263901619 | |
| 205506_at | VIL1 | -1,399512875 | -1,663414495 | 0,263901619 | |
| 208043_at | - | -1,399512875 | -1,663414495 | 0,263901619 | |
| 208480_s_at | ABCC6 | -1,399512875 | -1,663414495 | 0,263901619 | |
| 217190_x_at | ESR1 | -1,399512875 | -1,663414495 | 0,263901619 | |
| 217429_at | - | -1,399512875 | -1,663414495 | 0,263901619 | |
| 219519_s_at | SIGLEC1 | -1,399512875 | -1,663414495 | 0,263901619 | |
| 219971_at | IL21R | -1,399512875 | -1,663414495 | 0,263901619 | |
| 220596_at | GPATCH4 | -1,399512875 | -1,663414495 | 0,263901619 | |
| 221296_at | TECTA | -1,399512875 | -1,663414495 | 0,263901619 | |
| 228658_at | MIAT | -1,399512875 | -1,663414495 | 0,263901619 | |
| 232236_at | - | -1,399512875 | -1,663414495 | 0,263901619 | |
| 233785_at | ADAMTS9 | -1,399512875 | -1,663414495 | 0,263901619 | |
| 234685_x_at | KRTAP4-9 | -1,399512875 | -1,663414495 | 0,263901619 | |
| 234878_at | ZC3H12B | -1,399512875 | -1,663414495 | 0,263901619 | |
| 235627_at | PFN4 | -1,399512875 | -1,663414495 | 0,263901619 | |
| 235809_at | LIN54 | -1,399512875 | -1,663414495 | 0,263901619 | |
| 237129_at | CEP152 /// LOC1 | -1,399512875 | -1,663414495 | 0,263901619 | |
| 239617_at | GHRLOS | -1,399512875 | -1,663414495 | 0,263901619 | |
| 242771_at | TTN | -1,399512875 | -1,663414495 | 0,263901619 | |
| 1552320_a_at | CCDC65 | -2,311268652 | -2,575129729 | 0,263861077 | |
| 1553036_at | GPR111 | -2,311268652 | -2,575129729 | 0,263861077 | |
| 1560309_s_at | - | -2,311268652 | -2,575129729 | 0,263861077 | |
| 1561208_at | - | -2,311268652 | -2,575129729 | 0,263861077 | |
| 1562048_at | LOC152225 | -2,311268652 | -2,575129729 | 0,263861077 | |
| 1563298_at | - | -2,311268652 | -2,575129729 | 0,263861077 | |
| 1567590_at | - | -2,311268652 | -2,575129729 | 0,263861077 | |
| 1570162_at | - | -2,311268652 | -2,575129729 | 0,263861077 | |
| 1570226_at | - | -2,311268652 | -2,575129729 | 0,263861077 | |
| 204310_s_at | NPR2 | -2,311268652 | -2,575129729 | 0,263861077 | |
| 211517_s_at | IL5RA | -2,311268652 | -2,575129729 | 0,263861077 | |
| 211737_x_at | LOC100287705 / | -2,311268652 | -2,575129729 | 0,263861077 | |
| 214319_at | FRY | -2,311268652 | -2,575129729 | 0,263861077 | |

| | | | | | |
|---|---|---|---|---|---|
| 215703_at | CFTR | -2,311268652 | -2,575129729 | 0,263861077 | |
| 222176_at | - | -2,311268652 | -2,575129729 | 0,263861077 | |
| 230765_at | KIAA1239 | -2,311268652 | -2,575129729 | 0,263861077 | |
| 234283_at | - | -2,311268652 | -2,575129729 | 0,263861077 | |
| 238219_at | C12orf50 | -2,311268652 | -2,575129729 | 0,263861077 | |
| 239100_x_at | PCNX | -2,311268652 | -2,575129729 | 0,263861077 | |
| 239625_at | - | -2,311268652 | -2,575129729 | 0,263861077 | |
| 240090_at | - | -2,311268652 | -2,575129729 | 0,263861077 | |
| 240412_s_at | HEATR7B2 | -2,311268652 | -2,575129729 | 0,263861077 | |
| 240642_at | ZMYM2 | -2,311268652 | -2,575129729 | 0,263861077 | |
| 241588_at | - | -2,311268652 | -2,575129729 | 0,263861077 | |
| 242507_at | - | -2,311268652 | -2,575129729 | 0,263861077 | |
| 243607_at | - | -2,311268652 | -2,575129729 | 0,263861077 | |
| 244267_at | SATB1 | -2,311268652 | -2,575129729 | 0,263861077 | |
| 202006_at | PTPN12 | 4,407166241 | 4,143455947 | 0,263710293 | |
| 216996_s_at | FASTKD2 | 3,81731168 | 3,553617909 | 0,26369377 | |
| 210273_at | PCDH7 | -0,467091869 | -0,730778808 | 0,263686939 | |
| 211825_s_at | FLI1 | -0,467091869 | -0,730778808 | 0,263686939 | |
| 216560_x_at | IGLC1 | -0,467091869 | -0,730778808 | 0,263686939 | |
| 216637_at | - | -0,467091869 | -0,730778808 | 0,263686939 | |
| 219527_at | MARC2 | -0,467091869 | -0,730778808 | 0,263686939 | |
| 219849_at | ZNF671 | -0,467091869 | -0,730778808 | 0,263686939 | |
| 231740_at | KCNJ11 | -0,467091869 | -0,730778808 | 0,263686939 | |
| 232779_at | - | -0,467091869 | -0,730778808 | 0,263686939 | |
| 233905_at | SUN5 | -0,467091869 | -0,730778808 | 0,263686939 | |
| 237541_at | - | -0,467091869 | -0,730778808 | 0,263686939 | |
| 217216_x_at | MLH3 | 1,000476627 | 0,73688026 | 0,263596367 | |
| 1553317_s_at | GPR82 | -2,972500237 | -3,236070201 | 0,263569964 | |
| 1556220_at | - | -2,972500237 | -3,236070201 | 0,263569964 | |
| 1557636_a_at | C7orf57 | -2,972500237 | -3,236070201 | 0,263569964 | |
| 1561926_at | - | -2,972500237 | -3,236070201 | 0,263569964 | |
| 1570452_at | - | -2,972500237 | -3,236070201 | 0,263569964 | |
| 208375_at | IFNA1 | -2,972500237 | -3,236070201 | 0,263569964 | |
| 222227_at | - | -2,972500237 | -3,236070201 | 0,263569964 | |
| 237987_x_at | - | -2,972500237 | -3,236070201 | 0,263569964 | |
| 240773_at | - | -2,972500237 | -3,236070201 | 0,263569964 | |
| 211299_s_at | FLOT2 | 0,853119696 | 0,589657387 | 0,263462309 | |
| 1570071_at | - | 0,103263601 | -0,160136748 | 0,26340035 | |
| 204638_at | ACP5 | 0,103263601 | -0,160136748 | 0,26340035 | |
| 222876_s_at | ADAP2 | 0,103263601 | -0,160136748 | 0,26340035 | |
| 223692_at | NMNAT1 | 0,103263601 | -0,160136748 | 0,26340035 | |
| 226058_at | B3GNT9 | 0,103263601 | -0,160136748 | 0,26340035 | |
| 243710_at | LOC100506175 | 0,103263601 | -0,160136748 | 0,26340035 | |
| 209058_at | EDF1 | 4,959281929 | 4,695903811 | 0,263378118 | |
| 225216_at | FAM199X | 2,783874823 | 2,520531219 | 0,263343604 | |
| 230178_s_at | ELP2 | 1,168140757 | 0,904843258 | 0,263297499 | |
| 224330_s_at | MRPL27 | 4,993521137 | 4,730262306 | 0,263258831 | |
| 218561_s_at | LYRM4 | 4,481426562 | 4,21827299 | 0,263153572 | |
| 223341_s_at | SCOC | 3,354274239 | 3,091398163 | 0,262876076 | |
| 1557167_at | HCG11 | 0,291166226 | 0,028364014 | 0,262802211 | |
| 213999_at | YIPF4 | 0,291166226 | 0,028364014 | 0,262802211 | |
| 222138_s_at | WDR13 | 2,960432227 | 2,697670403 | 0,262761824 | |
| 209943_at | FBXL4 | 1,578645857 | 1,315948728 | 0,262697128 | |
| 217870_s_at | CMPK1 | 4,80549809 | 4,542844647 | 0,262653443 | |
| 224428_s_at | CDCA7 | 4,164288643 | 3,901713973 | 0,26257467 | |
| 224910_at | CARHSP1 | 0,934330293 | 0,67181667 | 0,262513623 | |

| | | | | | |
|---|---|---|---|---|---|
| 230546_at | VASH1 | 0,934330293 | 0,67181667 | 0,262513623 | |
| 209422_at | PHF20 | 2,943717636 | 2,681234383 | 0,262483253 | |
| 218373_at | AKTIP | 0,555999837 | 0,293518836 | 0,262481001 | |
| 226158_at | KLHL24 | 0,555999837 | 0,293518836 | 0,262481001 | |
| 243887_at | MRPL30 | 1,556829052 | 1,294497133 | 0,262331919 | |
| 213470_s_at | HNRNPH1 | 3,25727902 | 2,994948944 | 0,262330076 | |
| 223674_s_at | CDC42SE1 | 0,779676066 | 0,51742585 | 0,262250215 | |
| 223430_at | SIK2 | 0,695652136 | 0,433557298 | 0,262094838 | |
| 232787_at | PRIC285 | 0,695652136 | 0,433557298 | 0,262094838 | |
| 226025_at | ANKRD28 | 4,738838553 | 4,476764847 | 0,262073706 | |
| 221268_s_at | SGPP1 | 2,522021211 | 2,259997914 | 0,262023297 | |
| 1558412_at | LOC113230 | -0,85085127 | -1,112851844 | 0,262000574 | |
| 206396_at | SLC1A1 | -0,85085127 | -1,112851844 | 0,262000574 | |
| 215496_at | SAMD4A | -0,85085127 | -1,112851844 | 0,262000574 | |
| 221309_at | RBM17 | -0,85085127 | -1,112851844 | 0,262000574 | |
| 234199_at | - | -0,85085127 | -1,112851844 | 0,262000574 | |
| 218013_x_at | DCTN4 | 1,841816345 | 1,579853228 | 0,261963117 | |
| 225603_s_at | TRIQK | 1,841816345 | 1,579853228 | 0,261963117 | |
| 226298_at | RUNDC1 | 1,352503698 | 1,090553537 | 0,261950161 | |
| 223061_at | CHID1 | 2,861566439 | 2,599625088 | 0,261941352 | |
| 235010_at | LOC729013 | 0,900083947 | 0,638150376 | 0,261933571 | |
| 1558987_at | - | -2,359436556 | -2,621331989 | 0,261895433 | |
| 1562638_at | LOC339874 | -2,359436556 | -2,621331989 | 0,261895433 | |
| 1562677_at | - | -2,359436556 | -2,621331989 | 0,261895433 | |
| 1564301_a_at | RPAIN | -2,359436556 | -2,621331989 | 0,261895433 | |
| 1564642_at | RUNX1T1 | -2,359436556 | -2,621331989 | 0,261895433 | |
| 201691_s_at | TPD52 | -2,359436556 | -2,621331989 | 0,261895433 | |
| 204646_at | DPYD | -2,359436556 | -2,621331989 | 0,261895433 | |
| 206172_at | IL13RA2 | -2,359436556 | -2,621331989 | 0,261895433 | |
| 213831_at | HLA-DQA1 /// LO | -2,359436556 | -2,621331989 | 0,261895433 | |
| 214858_at | PP14571 | -2,359436556 | -2,621331989 | 0,261895433 | |
| 228067_at | C2orf55 | -2,359436556 | -2,621331989 | 0,261895433 | |
| 230206_at | DOCK5 | -2,359436556 | -2,621331989 | 0,261895433 | |
| 233249_at | - | -2,359436556 | -2,621331989 | 0,261895433 | |
| 237705_at | - | -2,359436556 | -2,621331989 | 0,261895433 | |
| 240028_at | FSIP2 | -2,359436556 | -2,621331989 | 0,261895433 | |
| 240382_at | LOC100505880 | -2,359436556 | -2,621331989 | 0,261895433 | |
| 241886_x_at | LOC338799 | -2,359436556 | -2,621331989 | 0,261895433 | |
| 243110_x_at | NPW | -2,359436556 | -2,621331989 | 0,261895433 | |
| 203243_s_at | PDLIM5 | 3,466766854 | 3,204982027 | 0,261784827 | |
| 201921_at | GNG10 | 4,318648014 | 4,056921713 | 0,2617263 | |
| 217598_at | CINP | 1,238353345 | 0,976641161 | 0,261712184 | |
| 230782_at | SORD | 1,238353345 | 0,976641161 | 0,261712184 | |
| 216920_s_at | TARP /// TRGC2 | 3,787958926 | 3,526272152 | 0,261686774 | |
| 58900_at | UBE2D4 | 2,511662711 | 2,250002661 | 0,26166005 | |
| 1552515_at | HIPK1 | -1,353583928 | -1,615132408 | 0,26154848 | |
| 1553852_at | VPS13B | -1,353583928 | -1,615132408 | 0,26154848 | |
| 1557888_at | ZNF787 | -1,353583928 | -1,615132408 | 0,26154848 | |
| 1559559_at | FAM75E1 | -1,353583928 | -1,615132408 | 0,26154848 | |
| 1559939_at | - | -1,353583928 | -1,615132408 | 0,26154848 | |
| 1561311_at | - | -1,353583928 | -1,615132408 | 0,26154848 | |
| 207106_s_at | LTK | -1,353583928 | -1,615132408 | 0,26154848 | |
| 211786_at | TNFRSF9 | -1,353583928 | -1,615132408 | 0,26154848 | |
| 213680_at | KRT6B | -1,353583928 | -1,615132408 | 0,26154848 | |
| 221167_s_at | CCDC70 | -1,353583928 | -1,615132408 | 0,26154848 | |
| 225142_at | JHDM1D | -1,353583928 | -1,615132408 | 0,26154848 | |

| | | | | | |
|---|---|---|---|---|---|
| 225496_s_at | SYTL2 | -1,353583928 | -1,615132408 | 0,26154848 | |
| 229896_at | - | -1,353583928 | -1,615132408 | 0,26154848 | |
| 230125_at | GUSB | -1,353583928 | -1,615132408 | 0,26154848 | |
| 230441_at | PLEKHG4B | -1,353583928 | -1,615132408 | 0,26154848 | |
| 232806_s_at | FAM131A | -1,353583928 | -1,615132408 | 0,26154848 | |
| 232984_at | HYDIN | -1,353583928 | -1,615132408 | 0,26154848 | |
| 237567_at | STAB2 | -1,353583928 | -1,615132408 | 0,26154848 | |
| 238036_at | SHE | -1,353583928 | -1,615132408 | 0,26154848 | |
| 239803_at | - | -1,353583928 | -1,615132408 | 0,26154848 | |
| 244471_x_at | PANX2 | -1,353583928 | -1,615132408 | 0,26154848 | |
| 244692_at | CYP4F22 | -1,353583928 | -1,615132408 | 0,26154848 | |
| 225366_at | PGM2 | 2,097427503 | 1,835911187 | 0,261516316 | |
| 37226_at | BNIP1 | 1,39615908 | 1,134680029 | 0,261479051 | |
| 204382_at | NAT9 | 1,462198073 | 1,200746069 | 0,261452004 | |
| 1552726_at | ADAMTS17 | -0,352951799 | -0,614371577 | 0,261419778 | |
| 214975_s_at | MTMR1 | -0,352951799 | -0,614371577 | 0,261419778 | |
| 228838_at | - | -0,352951799 | -0,614371577 | 0,261419778 | |
| 232802_at | SYT8 | -0,352951799 | -0,614371577 | 0,261419778 | |
| 212301_at | RTF1 | 4,400036207 | 4,138648509 | 0,261387698 | |
| 220536_at | VRTN | -0,022466681 | -0,283667828 | 0,261201147 | |
| 231278_at | - | -0,022466681 | -0,283667828 | 0,261201147 | |
| 225171_at | ARHGAP18 | 4,246090231 | 3,985091175 | 0,260999056 | |
| 203351_s_at | ORC4 | 4,079482587 | 3,818491075 | 0,260991512 | |
| 230786_at | ZCCHC8 | 0,46522187 | 0,204310755 | 0,260911115 | |
| 236937_at | LOC100505729 / | 0,46522187 | 0,204310755 | 0,260911115 | |
| 239284_at | - | 0,181384709 | -0,079522948 | 0,260907657 | |
| 241779_at | MTX3 | 0,181384709 | -0,079522948 | 0,260907657 | |
| 210357_s_at | SMOX | 3,101484927 | 2,840694075 | 0,260790852 | |
| 201008_s_at | TXNIP | 4,407166241 | 4,146452482 | 0,260713759 | |
| 207187_at | JAK3 | 0,613492853 | 0,352836757 | 0,260656096 | |
| 219057_at | RABEP2 | 0,613492853 | 0,352836757 | 0,260656096 | |
| 212630_at | EXOC3 | 2,331726354 | 2,071071048 | 0,260655306 | |
| 1568900_a_at | ZNF568 | 1,274448124 | 1,013870809 | 0,260577315 | |
| 204573_at | CROT | 1,748661581 | 1,488344553 | 0,260317029 | |
| 216606_x_at | LYPLA2 | 2,44295215 | 2,182705233 | 0,260246916 | |
| 210156_s_at | PCMT1 | 5,494689936 | 5,234479961 | 0,260209975 | |
| 1555504_at | TYR | -3,013665402 | -3,273861381 | 0,260195979 | |
| 215938_s_at | PLA2G6 | 1,390002893 | 1,129843209 | 0,260159685 | |
| 219822_at | MTRF1 | 1,390002893 | 1,129843209 | 0,260159685 | |
| 229219_s_at | - | 0,984222186 | 0,724100169 | 0,260122016 | |
| 214455_at | HIST1H2BC /// H | 3,170152481 | 2,910041809 | 0,260110672 | |
| 200971_s_at | SERP1 | 5,862786464 | 5,60270124 | 0,260085224 | |
| 1554086_at | TUBGCP3 | 0,417596971 | 0,157549243 | 0,260047728 | |
| 1569144_a_at | C9orf169 | 0,417596971 | 0,157549243 | 0,260047728 | |
| 207917_at | - | 0,417596971 | 0,157549243 | 0,260047728 | |
| 230642_at | - | 0,417596971 | 0,157549243 | 0,260047728 | |
| 233517_s_at | HIF3A | 0,417596971 | 0,157549243 | 0,260047728 | |
| 236178_at | C6orf162 | 0,417596971 | 0,157549243 | 0,260047728 | |
| 232038_at | C6orf170 | 0,57058931 | 0,310717132 | 0,259872178 | |
| 1562601_at | UNQ6975 | 0,123193849 | -0,136643983 | 0,259837832 | |
| 203851_at | IGFBP6 | 0,123193849 | -0,136643983 | 0,259837832 | |
| 207692_s_at | ACAN | 0,123193849 | -0,136643983 | 0,259837832 | |
| 220077_at | CCDC134 | 0,123193849 | -0,136643983 | 0,259837832 | |
| 213943_at | TWIST1 | 2,05655332 | 1,796776588 | 0,259776733 | |
| 217936_at | ARHGAP5 | 3,03990821 | 2,780243895 | 0,259664315 | |
| 203447_at | PSMD5 | 4,040056475 | 3,780418488 | 0,259637987 | |

| | | | | | |
|---|---|---|---|---|---|
| 214626_s_at | GANAB | 3,653945107 | 3,394350331 | 0,259594777 | |
| 238021_s_at | CRNDE | 4,070528765 | 3,810956512 | 0,259572253 | |
| 233842_x_at | C20orf43 | 4,396969658 | 4,137444143 | 0,259525516 | |
| 211969_at | HSP90AA1 | 7,637820532 | 7,378342424 | 0,259478108 | |
| 218573_at | MAGEH1 | 3,173740462 | 2,914267328 | 0,259473134 | |
| 217826_s_at | UBE2J1 | 3,266269388 | 3,006869115 | 0,259400273 | |
| 213710_s_at | LOC100652987 | 1,951783949 | 1,692388143 | 0,259395806 | |
| 205115_s_at | RBM19 | 1,339784194 | 1,080561626 | 0,259222569 | |
| 218816_at | LRRC1 | 1,094335495 | 0,835239052 | 0,259096443 | |
| 241933_at | QRSL1 | 1,094335495 | 0,835239052 | 0,259096443 | |
| 241912_at | ZNF814 | 1,575032557 | 1,315948728 | 0,259083828 | |
| 51774_s_at | UBE2D4 | 2,545290278 | 2,286318242 | 0,258972036 | |
| 202634_at | POLR2K | 4,341690695 | 4,082805739 | 0,258884956 | |
| 226395_at | HOOK3 | 2,658027143 | 2,39915583 | 0,258871314 | |
| 223069_s_at | EML4 | 0,917308734 | 0,658444279 | 0,258864455 | |
| 224437_s_at | VTA1 | 4,876570556 | 4,617737065 | 0,258833492 | |
| 209333_at | ULK1 | 1,027166904 | 0,768343791 | 0,258823114 | |
| 201449_at | TIA1 | 2,2992452 | 2,040470494 | 0,258774706 | |
| 211926_s_at | MYH9 | 3,121439986 | 2,862725455 | 0,258714531 | |
| 201435_s_at | EIF4E | 4,998924782 | 4,74021396 | 0,258710822 | |
| 202452_at | ZER1 | 1,063722698 | 0,805216064 | 0,258506634 | |
| 213615_at | LPCAT3 | 1,163336153 | 0,904843258 | 0,258492895 | |
| 221655_x_at | EPS8L1 | 1,163336153 | 0,904843258 | 0,258492895 | |
| 218647_s_at | YRDC | 3,764356332 | 3,505883949 | 0,258472383 | |
| 219403_s_at | HPSE | 1,504602299 | 1,246149888 | 0,258452411 | |
| 205048_s_at | PSPH | 1,649074067 | 1,390636111 | 0,258437955 | |
| 243278_at | FOXP2 | 1,649074067 | 1,390636111 | 0,258437955 | |
| 212568_s_at | DLAT | 4,073092675 | 3,814728712 | 0,258363963 | |
| 210293_s_at | SEC23B | 4,396969658 | 4,138648509 | 0,258321149 | |
| 32128_at | CCL18 | -1,987984448 | -2,246296361 | 0,258311912 | |
| 1555028_at | BRD3 | -2,43257096 | -2,690860744 | 0,258289784 | |
| 1555127_at | MOCS1 | -2,43257096 | -2,690860744 | 0,258289784 | |
| 1555246_a_at | SCN1A | -2,43257096 | -2,690860744 | 0,258289784 | |
| 1561452_at | - | -2,43257096 | -2,690860744 | 0,258289784 | |
| 1563010_at | ITGB8 | -2,43257096 | -2,690860744 | 0,258289784 | |
| 1564130_x_at | - | -2,43257096 | -2,690860744 | 0,258289784 | |
| 1565282_at | - | -2,43257096 | -2,690860744 | 0,258289784 | |
| 208054_at | HERC4 | -2,43257096 | -2,690860744 | 0,258289784 | |
| 211751_at | LOC653513 /// LO | -2,43257096 | -2,690860744 | 0,258289784 | |
| 215132_at | - | -2,43257096 | -2,690860744 | 0,258289784 | |
| 216498_at | - | -2,43257096 | -2,690860744 | 0,258289784 | |
| 231421_at | LOC254312 | -2,43257096 | -2,690860744 | 0,258289784 | |
| 232742_at | - | -2,43257096 | -2,690860744 | 0,258289784 | |
| 234117_at | NPSR1-AS1 | -2,43257096 | -2,690860744 | 0,258289784 | |
| 234636_at | - | -2,43257096 | -2,690860744 | 0,258289784 | |
| 234945_at | FAM54A | -2,43257096 | -2,690860744 | 0,258289784 | |
| 235777_at | ANKRD44 | -2,43257096 | -2,690860744 | 0,258289784 | |
| 238274_at | - | -2,43257096 | -2,690860744 | 0,258289784 | |
| 238871_at | MLLT4 | -2,43257096 | -2,690860744 | 0,258289784 | |
| 240251_at | IFT80 | -2,43257096 | -2,690860744 | 0,258289784 | |
| 241435_at | - | -2,43257096 | -2,690860744 | 0,258289784 | |
| 243179_at | - | -2,43257096 | -2,690860744 | 0,258289784 | |
| 243683_at | MORF4L2 | -2,43257096 | -2,690860744 | 0,258289784 | |
| 200799_at | HSPA1A /// HSPA | 6,507687228 | 6,249430555 | 0,258256673 | |
| 235232_at | GMEB1 | 1,530951994 | 1,272721751 | 0,258230243 | |
| 228222_at | PPP1CB | 4,370116264 | 4,111917374 | 0,25819889 | |

| | | | | | |
|---|---|---|---|---|---|
| 1554814_at | UBE2O | 0,995078803 | 0,73688026 | 0,258198542 | |
| 232317_at | PLXNA4 | 0,995078803 | 0,73688026 | 0,258198542 | |
| 202321_at | GGPS1 | 0,882651021 | 0,624460759 | 0,258190262 | |
| 222507_s_at | TMEM9B | 2,604089881 | 2,345926937 | 0,258162944 | |
| 227877_at | ANXA2R | 0,317354302 | 0,059218869 | 0,258135433 | |
| 1552694_at | SLC2A13 | -2,779459409 | -3,037500868 | 0,25804146 | |
| 1554235_at | CTNNA3 | -2,779459409 | -3,037500868 | 0,25804146 | |
| 1554901_at | GAFA1 | -2,779459409 | -3,037500868 | 0,25804146 | |
| 1560647_at | TSPYL1 | -2,779459409 | -3,037500868 | 0,25804146 | |
| 1560859_at | - | -2,779459409 | -3,037500868 | 0,25804146 | |
| 1561529_at | - | -2,779459409 | -3,037500868 | 0,25804146 | |
| 1569770_x_at | - | -2,779459409 | -3,037500868 | 0,25804146 | |
| 1569840_at | - | -2,779459409 | -3,037500868 | 0,25804146 | |
| 207647_at | CDY1 | -2,779459409 | -3,037500868 | 0,25804146 | |
| 211568_at | BAI3 | -2,779459409 | -3,037500868 | 0,25804146 | |
| 233591_at | - | -2,779459409 | -3,037500868 | 0,25804146 | |
| 234702_x_at | CFTR | -2,779459409 | -3,037500868 | 0,25804146 | |
| 239902_at | - | -2,779459409 | -3,037500868 | 0,25804146 | |
| 241220_at | - | -2,779459409 | -3,037500868 | 0,25804146 | |
| 232367_x_at | ZNF598 | 1,40228911 | 1,144305291 | 0,257983819 | |
| 40255_at | DDX28 | 1,960118853 | 1,702196344 | 0,257922509 | |
| 202302_s_at | RSRC2 | 4,911014665 | 4,653142152 | 0,257872513 | |
| 1553696_s_at | ZNF569 | 3,62365843 | 3,365837114 | 0,257821316 | |
| 202137_s_at | ZMYND11 | 1,348276312 | 1,090553537 | 0,257722774 | |
| 214323_s_at | UPF3A | 3,895444023 | 3,637795954 | 0,25764807 | |
| 226338_at | TMEM55A | 0,962260738 | 0,704715219 | 0,257545519 | |
| 230267_at | - | 0,962260738 | 0,704715219 | 0,257545519 | |
| 204128_s_at | RFC3 | 3,861734212 | 3,604188755 | 0,257545458 | |
| 208829_at | TAPBP | 2,571820446 | 2,314300277 | 0,257520169 | |
| 213301_x_at | TRIM24 | 4,87436728 | 4,616873117 | 0,257494164 | |
| 226603_at | SAMD9L | 1,699726954 | 1,442269454 | 0,2574575 | |
| 202052_s_at | RAI14 | -0,00073712 | -0,258114234 | 0,257377114 | |
| 203796_s_at | BCL7A | -0,00073712 | -0,258114234 | 0,257377114 | |
| 219295_s_at | PCOLCE2 | -0,00073712 | -0,258114234 | 0,257377114 | |
| 228290_at | PLK1S1 | -0,00073712 | -0,258114234 | 0,257377114 | |
| 232095_at | LOC100509683 | -0,00073712 | -0,258114234 | 0,257377114 | |
| 205309_at | SMPDL3B | -1,2845686 | -1,541908042 | 0,257339442 | |
| 212466_at | SPRED2 | -1,2845686 | -1,541908042 | 0,257339442 | |
| 214929_s_at | KIAA1109 | -1,2845686 | -1,541908042 | 0,257339442 | |
| 221584_s_at | KCNMA1 | -1,2845686 | -1,541908042 | 0,257339442 | |
| 224515_at | - | -1,2845686 | -1,541908042 | 0,257339442 | |
| 225809_at | PARM1 | -1,2845686 | -1,541908042 | 0,257339442 | |
| 226436_at | RASSF4 | -1,2845686 | -1,541908042 | 0,257339442 | |
| 229157_at | PRKAG2-AS1 | -1,2845686 | -1,541908042 | 0,257339442 | |
| 231123_at | TRIM36 | -1,2845686 | -1,541908042 | 0,257339442 | |
| 233644_at | KATNAL2 | -1,2845686 | -1,541908042 | 0,257339442 | |
| 234079_at | - | -1,2845686 | -1,541908042 | 0,257339442 | |
| 235937_at | OCLN | -1,2845686 | -1,541908042 | 0,257339442 | |
| 240071_at | - | -1,2845686 | -1,541908042 | 0,257339442 | |
| 240697_at | - | -1,2845686 | -1,541908042 | 0,257339442 | |
| 240764_at | - | -1,2845686 | -1,541908042 | 0,257339442 | |
| 244548_at | - | -1,2845686 | -1,541908042 | 0,257339442 | |
| 244601_at | - | -1,2845686 | -1,541908042 | 0,257339442 | |
| 219343_at | CDC37L1 | 1,104397092 | 0,847075464 | 0,257321628 | |
| 239562_at | MTHFD2L | 1,233777334 | 0,976641161 | 0,257136173 | |
| 228736_at | HELQ | 1,434549838 | 1,177496821 | 0,257053017 | |

| | | | | | |
|---|---|---|---|---|---|
| 201260_s_at | SYPL1 | 4,467851686 | 4,210848982 | 0,257002704 | |
| 203560_at | GGH | 4,713590151 | 4,456625687 | 0,256964464 | |
| 218355_at | KIF4A | 3,966117062 | 3,709248632 | 0,25686843 | |
| 217426_at | - | 0,928678725 | 0,67181667 | 0,256862055 | |
| 1553868_a_at | KIAA0825 | -3,054236653 | -3,311072652 | 0,256835999 | |
| 1558957_s_at | - | -3,054236653 | -3,311072652 | 0,256835999 | |
| 1560540_x_at | - | -3,054236653 | -3,311072652 | 0,256835999 | |
| 220649_at | AGBL3 | -3,054236653 | -3,311072652 | 0,256835999 | |
| 240952_at | - | -3,054236653 | -3,311072652 | 0,256835999 | |
| 244632_at | CNTN5 | -3,054236653 | -3,311072652 | 0,256835999 | |
| 213092_x_at | DNAJC9 | 4,077568583 | 3,820743792 | 0,256824791 | |
| 212779_at | KIAA1109 | 2,281618189 | 2,024923328 | 0,256694862 | |
| 1562055_at | - | -0,234278768 | -0,490927335 | 0,256648568 | |
| 206060_s_at | PTPN22 | -0,234278768 | -0,490927335 | 0,256648568 | |
| 215637_at | CEP41 | -0,234278768 | -0,490927335 | 0,256648568 | |
| 219504_s_at | RPAP2 | -0,234278768 | -0,490927335 | 0,256648568 | |
| 232140_at | - | -0,234278768 | -0,490927335 | 0,256648568 | |
| 241989_at | GRK4 | -0,234278768 | -0,490927335 | 0,256648568 | |
| 225694_at | CDK12 | 3,21379258 | 2,957221307 | 0,256571273 | |
| 1570156_s_at | FMN1 /// LOC10( | -0,423248115 | -0,679763839 | 0,256515724 | |
| 215687_x_at | PLCB1 | -0,423248115 | -0,679763839 | 0,256515724 | |
| 226138_s_at | - | -0,423248115 | -0,679763839 | 0,256515724 | |
| 231822_at | CTTNBP2NL | -0,423248115 | -0,679763839 | 0,256515724 | |
| 224940_s_at | PAPPA | 2,26825514 | 2,011838132 | 0,256417008 | |
| 1562799_at | LOC100130285 | -0,655928373 | -0,912331589 | 0,256403216 | |
| 210801_at | DIMT1 | -0,655928373 | -0,912331589 | 0,256403216 | |
| 210885_s_at | TRIM15 | -0,655928373 | -0,912331589 | 0,256403216 | |
| 213217_at | ADCY2 | -0,655928373 | -0,912331589 | 0,256403216 | |
| 230966_at | IL4I1 | -0,655928373 | -0,912331589 | 0,256403216 | |
| 239051_at | FLJ31813 | -0,655928373 | -0,912331589 | 0,256403216 | |
| 239177_at | IRGQ | -0,655928373 | -0,912331589 | 0,256403216 | |
| 240669_at | - | -0,655928373 | -0,912331589 | 0,256403216 | |
| 228840_at | AMOTL1 | 1,614289682 | 1,3579181 | 0,256371582 | |
| 203481_at | FAM178A | 3,159334685 | 2,902971658 | 0,256363027 | |
| 212997_s_at | TLK2 | 2,739371812 | 2,483094137 | 0,256277676 | |
| 226232_at | GDF11 | 0,384950573 | 0,128747141 | 0,256203433 | |
| 239166_at | - | 0,384950573 | 0,128747141 | 0,256203433 | |
| 1555833_a_at | IRGQ | 2,14450783 | 1,888402606 | 0,256105225 | |
| 204147_s_at | TFDP1 | 2,68509622 | 2,428994491 | 0,25610173 | |
| 1552678_a_at | USP28 | 0,592200562 | 0,336136178 | 0,256064384 | |
| 218176_at | MAGEF1 | 2,371846398 | 2,115788273 | 0,256058125 | |
| 222681_at | POGLUT1 | 2,814842651 | 2,558821855 | 0,256020796 | |
| 1557312_at | C12orf61 | -2,479842156 | -2,735862426 | 0,256020269 | |
| 1557581_x_at | - | -2,479842156 | -2,735862426 | 0,256020269 | |
| 1558301_a_at | EFCAB5 | -2,479842156 | -2,735862426 | 0,256020269 | |
| 1569486_at | - | -2,479842156 | -2,735862426 | 0,256020269 | |
| 1569709_at | SZT2 | -2,479842156 | -2,735862426 | 0,256020269 | |
| 1569923_s_at | LINC00491 | -2,479842156 | -2,735862426 | 0,256020269 | |
| 205974_at | HOXD1 | -2,479842156 | -2,735862426 | 0,256020269 | |
| 206884_s_at | SCEL | -2,479842156 | -2,735862426 | 0,256020269 | |
| 210274_at | MAGEA8 | -2,479842156 | -2,735862426 | 0,256020269 | |
| 213808_at | ADAM23 | -2,479842156 | -2,735862426 | 0,256020269 | |
| 214983_at | TTTY15 | -2,479842156 | -2,735862426 | 0,256020269 | |
| 225016_at | APCDD1 | -2,479842156 | -2,735862426 | 0,256020269 | |
| 228221_at | SLC44A3 | -2,479842156 | -2,735862426 | 0,256020269 | |
| 230248_x_at | - | -2,479842156 | -2,735862426 | 0,256020269 | |

| | | | | | |
|---|---|---|---|---|---|
| 234815_at | - | -2,479842156 | -2,735862426 | 0,256020269 | |
| 234823_at | - | -2,479842156 | -2,735862426 | 0,256020269 | |
| 236811_at | DMRTC2 | -2,479842156 | -2,735862426 | 0,256020269 | |
| 237298_at | FLJ26850 | -2,479842156 | -2,735862426 | 0,256020269 | |
| 238513_at | PRRG4 | -2,479842156 | -2,735862426 | 0,256020269 | |
| 240488_at | - | -2,479842156 | -2,735862426 | 0,256020269 | |
| 241562_x_at | - | -2,479842156 | -2,735862426 | 0,256020269 | |
| 218423_x_at | VPS54 | 2,923969333 | 2,667949403 | 0,25601993 | |
| 201075_s_at | SMARCC1 | 3,480351894 | 3,22439681 | 0,255955083 | |
| 1554279_a_at | TRMT2B | 0,209623097 | -0,046302147 | 0,255925244 | |
| 218184_at | TULP4 | 2,178241192 | 1,922365006 | 0,255876185 | |
| 219515_at | PRDM10 | 1,990274687 | 1,73441697 | 0,255857717 | |
| 204805_s_at | H1FX | 2,061726373 | 1,805902329 | 0,255824044 | |
| 219904_at | ZSCAN5A | 2,618203384 | 2,362515214 | 0,25568817 | |
| 208527_x_at | HIST1H2BC /// H | 6,41042397 | 6,154836421 | 0,255587548 | |
| 202293_at | STAG1 | 2,227406889 | 1,971855016 | 0,255551873 | |
| 216381_x_at | AKR7A3 | 0,859074518 | 0,603679647 | 0,255394872 | |
| 210625_s_at | AKAP1 | 1,011212064 | 0,755840599 | 0,255371464 | |
| 234956_at | LOC93444 | 1,011212064 | 0,755840599 | 0,255371464 | |
| 212887_at | SEC23A | 4,077568583 | 3,822243651 | 0,255324932 | |
| 1555845_at | - | 2,632180155 | 2,376875125 | 0,25530503 | |
| 205339_at | STIL | 3,672662071 | 3,417357537 | 0,255304535 | |
| 202666_s_at | ACTL6A | 4,519522578 | 4,264240901 | 0,255281678 | |
| 226859_at | DNAJC25 /// DNA | 2,213529682 | 1,958277216 | 0,255252466 | |
| 228254_at | STAM2 | 1,799091453 | 1,543943227 | 0,255148226 | |
| 235918_x_at | CEP97 | 1,799091453 | 1,543943227 | 0,255148226 | |
| 223977_s_at | LINC00470 | 0,735041319 | 0,479907041 | 0,255134278 | |
| 213763_at | HIPK2 | 0,334552598 | 0,079428135 | 0,255124463 | |
| 220332_at | CLDN16 | 0,334552598 | 0,079428135 | 0,255124463 | |
| 237211_x_at | MORN3 | 0,334552598 | 0,079428135 | 0,255124463 | |
| 242427_at | WAC | 0,334552598 | 0,079428135 | 0,255124463 | |
| 1552768_at | CAMKK1 | -0,79540287 | -1,050406615 | 0,255003745 | |
| 1562324_a_at | - | -0,79540287 | -1,050406615 | 0,255003745 | |
| 201539_s_at | FHL1 | -0,79540287 | -1,050406615 | 0,255003745 | |
| 219786_at | MTL5 | -0,79540287 | -1,050406615 | 0,255003745 | |
| 220489_s_at | SERINC2 | -0,79540287 | -1,050406615 | 0,255003745 | |
| 220675_s_at | PNPLA3 | -0,79540287 | -1,050406615 | 0,255003745 | |
| 220716_at | GNL3LP1 | -0,79540287 | -1,050406615 | 0,255003745 | |
| 227155_at | LMO4 | -0,79540287 | -1,050406615 | 0,255003745 | |
| 228447_at | AKAP17A | -0,79540287 | -1,050406615 | 0,255003745 | |
| 204193_at | CHKB | 1,549482847 | 1,294497133 | 0,254985714 | |
| 224173_s_at | C2orf15 /// MRPL | 1,549482847 | 1,294497133 | 0,254985714 | |
| 211754_s_at | SLC25A17 | 3,231115193 | 2,976208448 | 0,254906745 | |
| 201424_s_at | CUL4A | 3,831767101 | 3,576905405 | 0,254861696 | |
| 219283_at | C1GALT1C1 | 2,014487247 | 1,759685182 | 0,254802065 | |
| 235451_at | SMAD5 | 1,119359127 | 0,864649967 | 0,25470916 | |
| 1554637_a_at | CBFA2T2 | 0,152582607 | -0,102100538 | 0,254683145 | |
| 1564697_a_at | LOC400752 | 0,152582607 | -0,102100538 | 0,254683145 | |
| 206027_at | S100A3 | 0,152582607 | -0,102100538 | 0,254683145 | |
| 220514_at | LOC100505870 | 0,152582607 | -0,102100538 | 0,254683145 | |
| 231784_s_at | DCAF13 | 4,434840843 | 4,180180181 | 0,254660663 | |
| 202733_at | P4HA2 | 2,11992033 | 1,865308541 | 0,254611788 | |
| 1559038_at | 37500 | 0,822971223 | 0,568365095 | 0,254606128 | |
| 239004_at | SQSTM1 | 0,822971223 | 0,568365095 | 0,254606128 | |
| 228953_at | WHAMM | 1,62481334 | 1,370274469 | 0,254538871 | |
| 201471_s_at | SQSTM1 | 3,50144467 | 3,246908183 | 0,254536487 | |

| | | | | | |
|---|---|---|---|---|---|
| 218078_s_at | ZDHHC3 | 2,338135744 | 2,083632167 | 0,254503577 | |
| 1569647_at | LOC643623 | -1,240169874 | -1,494668682 | 0,254498807 | |
| 207426_s_at | TNFSF4 | -1,240169874 | -1,494668682 | 0,254498807 | |
| 213774_s_at | PPP1R2 | -1,240169874 | -1,494668682 | 0,254498807 | |
| 217063_x_at | YME1L1 | -1,240169874 | -1,494668682 | 0,254498807 | |
| 217169_at | IGHA1 /// IGHG1 | -1,240169874 | -1,494668682 | 0,254498807 | |
| 220055_at | ZNF287 | -1,240169874 | -1,494668682 | 0,254498807 | |
| 220593_s_at | CCDC40 | -1,240169874 | -1,494668682 | 0,254498807 | |
| 223654_s_at | CELF4 | -1,240169874 | -1,494668682 | 0,254498807 | |
| 229900_at | CD109 | -1,240169874 | -1,494668682 | 0,254498807 | |
| 230643_at | WNT9A | -1,240169874 | -1,494668682 | 0,254498807 | |
| 236433_at | LOC100506713 / | -1,240169874 | -1,494668682 | 0,254498807 | |
| 237203_at | LOC100506546 | -1,240169874 | -1,494668682 | 0,254498807 | |
| 242975_s_at | - | -1,240169874 | -1,494668682 | 0,254498807 | |
| 244465_at | - | -1,240169874 | -1,494668682 | 0,254498807 | |
| 222679_s_at | DCUN1D1 | 2,874852734 | 2,620464265 | 0,254388469 | |
| 222652_s_at | GLYR1 | 1,187200746 | 0,932882274 | 0,254318472 | |
| 238054_at | ADPRHL1 | 1,187200746 | 0,932882274 | 0,254318472 | |
| 1553507_a_at | GPR6 | -0,136301282 | -0,39061235 | 0,254311068 | |
| 202426_s_at | RXRA | -0,136301282 | -0,39061235 | 0,254311068 | |
| 203618_at | FAIM2 | -0,136301282 | -0,39061235 | 0,254311068 | |
| 204929_s_at | VAMP5 | -0,136301282 | -0,39061235 | 0,254311068 | |
| 206447_at | CELA2A /// CELA | -0,136301282 | -0,39061235 | 0,254311068 | |
| 222923_s_at | KCNE3 | -0,136301282 | -0,39061235 | 0,254311068 | |
| 229358_at | IHH | -0,136301282 | -0,39061235 | 0,254311068 | |
| 230633_at | TMEM102 | -0,136301282 | -0,39061235 | 0,254311068 | |
| 234351_x_at | TRPS1 | -0,136301282 | -0,39061235 | 0,254311068 | |
| 238451_at | MPP7 | -0,136301282 | -0,39061235 | 0,254311068 | |
| 238745_at | - | -0,136301282 | -0,39061235 | 0,254311068 | |
| 242337_at | - | 1,672933557 | 1,418668082 | 0,254265474 | |
| 214804_at | CENPI | 0,503742507 | 0,249604176 | 0,254138331 | |
| 230598_at | - | 0,503742507 | 0,249604176 | 0,254138331 | |
| 211945_s_at | ITGB1 | 6,497105595 | 6,243145676 | 0,253959919 | |
| 226705_at | FGFR1 | 0,945567441 | 0,691645685 | 0,253921756 | |
| 242539_at | DIS3L2 | 0,945567441 | 0,691645685 | 0,253921756 | |
| 208706_s_at | EIF5 | 5,316276373 | 5,062398002 | 0,253878371 | |
| 213619_at | HNRNPH1 | 6,244908051 | 5,991050893 | 0,253857158 | |
| 225917_at | - | 1,946200473 | 1,692388143 | 0,25381233 | |
| 1561687_a_at | ZNF382 | 1,556829052 | 1,303116084 | 0,253712967 | |
| 219180_s_at | PEX26 | 1,454352496 | 1,200746069 | 0,253606427 | |
| 1557051_s_at | HOTAIRM1 | 0,606430281 | 0,352836757 | 0,253593524 | |
| 214162_at | LOC284244 | 0,606430281 | 0,352836757 | 0,253593524 | |
| 220470_at | BET1L | 0,606430281 | 0,352836757 | 0,253593524 | |
| 221970_s_at | NOL11 | 5,037507384 | 4,783973541 | 0,253533843 | |
| 222736_s_at | TMEM38B | 3,292909151 | 3,039473081 | 0,25343607 | |
| 236196_at | - | 1,631786706 | 1,378453643 | 0,253333063 | |
| 1554830_a_at | STEAP3 | -0,512251684 | -0,765504029 | 0,253252344 | |
| 1559449_a_at | - | -0,512251684 | -0,765504029 | 0,253252344 | |
| 1569838_at | - | -0,512251684 | -0,765504029 | 0,253252344 | |
| 206822_s_at | L3MBTL1 | -0,512251684 | -0,765504029 | 0,253252344 | |
| 207172_s_at | CDH11 | -0,512251684 | -0,765504029 | 0,253252344 | |
| 209447_at | SYNE1 | -0,512251684 | -0,765504029 | 0,253252344 | |
| 211280_s_at | NRF1 | -0,512251684 | -0,765504029 | 0,253252344 | |
| 218864_at | TNS1 | -0,512251684 | -0,765504029 | 0,253252344 | |
| 225495_x_at | GADD45GIP1 | -0,512251684 | -0,765504029 | 0,253252344 | |
| 225822_at | TMEM125 | -0,512251684 | -0,765504029 | 0,253252344 | |

| | | | | | |
|---|---|---|---|---|---|
| 232969_at | CARD8 | -0,512251684 | -0,765504029 | 0,253252344 | |
| 234328_at | - | -0,512251684 | -0,765504029 | 0,253252344 | |
| 235141_at | MARVELD2 | -0,512251684 | -0,765504029 | 0,253252344 | |
| 236061_at | PRDM15 | -0,512251684 | -0,765504029 | 0,253252344 | |
| 238074_at | WDR27 | -0,512251684 | -0,765504029 | 0,253252344 | |
| 240684_at | - | -0,512251684 | -0,765504029 | 0,253252344 | |
| 209029_at | COPS7A | 2,861566439 | 2,608344689 | 0,25322175 | |
| 235882_at | VPS53 | 0,911589958 | 0,658444279 | 0,253145679 | |
| 218327_s_at | SNAP29 | 3,279650757 | 3,026519779 | 0,253130978 | |
| 232491_at | - | 0,457392764 | 0,204310755 | 0,253082009 | |
| 244002_at | - | 0,457392764 | 0,204310755 | 0,253082009 | |
| 238719_at | MIR3661 /// PPP2 | 1,912238072 | 1,659204017 | 0,253034055 | |
| 236314_at | EPM2AIP1 | 1,129248302 | 0,876248481 | 0,252999821 | |
| 1554523_a_at | CNNM2 | 0,747935635 | 0,495031851 | 0,252903785 | |
| 221618_s_at | TAF9B | 1,318332599 | 1,065442793 | 0,252889806 | |
| 230685_at | FLJ33630 | 0,228146222 | -0,024572586 | 0,252718808 | |
| 244136_at | LOC100507261 | 0,228146222 | -0,024572586 | 0,252718808 | |
| 244204_at | - | 0,228146222 | -0,024572586 | 0,252718808 | |
| 211668_s_at | PLAU | 1,512180019 | 1,259496994 | 0,252683024 | |
| 235989_at | - | 1,683039483 | 1,43051703 | 0,252522453 | |
| 224644_at | FGD5-AS1 | 4,244953702 | 3,992437318 | 0,252516384 | |
| 201367_s_at | ZFP36L2 | 1,814495998 | 1,562009954 | 0,252486044 | |
| 200994_at | IPO7 | 4,625505141 | 4,373134192 | 0,252370948 | |
| 208615_s_at | PTP4A2 | 3,734704399 | 3,482360311 | 0,252344088 | |
| 203075_at | SMAD2 | 2,277177578 | 2,024923328 | 0,252254251 | |
| 238637_at | - | 1,751865623 | 1,499637023 | 0,2522286 | |
| 1554080_at | RQCD1 | 2,380152406 | 2,127967638 | 0,252184768 | |
| 212408_at | TOR1AIP1 | 4,587453139 | 4,335292864 | 0,252160276 | |
| 218098_at | ARFGEF2 | 3,936854923 | 3,684723168 | 0,252131754 | |
| 213736_at | COX5B | 1,795990708 | 1,543943227 | 0,25204748 | |
| 229265_at | SKI | 3,994121175 | 3,742106442 | 0,252014733 | |
| 236114_at | - | 0,95671774 | 0,704715219 | 0,252002521 | |
| 242240_at | - | 0,95671774 | 0,704715219 | 0,252002521 | |
| 1556434_at | - | -0,394749123 | -0,646746079 | 0,251996955 | |
| 210666_at | IDS | -0,394749123 | -0,646746079 | 0,251996955 | |
| 226699_at | FCHSD1 | -0,394749123 | -0,646746079 | 0,251996955 | |
| 229229_at | AGXT2 | -0,394749123 | -0,646746079 | 0,251996955 | |
| 211385_x_at | SULT1A2 | 1,381753566 | 1,129843209 | 0,251910358 | |
| 210539_at | TTLL5 | 0,03125738 | -0,220592223 | 0,251849603 | |
| 240873_x_at | DAB2 | 0,03125738 | -0,220592223 | 0,251849603 | |
| 203474_at | IQGAP2 | 5,330008035 | 5,078176444 | 0,25183159 | |
| 204928_s_at | SLC10A3 | 1,732533761 | 1,480766833 | 0,251766929 | |
| 213405_at | RAB22A | 2,919702154 | 2,667949403 | 0,251752751 | |
| 227737_at | SRPRB | 2,218170265 | 1,966439221 | 0,251731044 | |
| 205133_s_at | HSPE1 | 6,34364026 | 6,091955318 | 0,251684942 | |
| 225195_at | DPH3 | 4,365940246 | 4,114368048 | 0,251572199 | |
| 229878_at | KIAA1731 | 1,64218396 | 1,390636111 | 0,251547849 | |
| 222011_s_at | SNORA29 /// TCF | 3,906268218 | 3,654731437 | 0,25153678 | |
| 215718_s_at | PHF3 | 0,518867317 | 0,26733076 | 0,251536557 | |
| 1569077_x_at | ZNF836 | 0,841135803 | 0,589657387 | 0,251478416 | |
| 205160_at | PEX11A | 0,841135803 | 0,589657387 | 0,251478416 | |
| 208047_s_at | NAB1 | 0,841135803 | 0,589657387 | 0,251478416 | |
| 208569_at | HIST1H2AB | 0,841135803 | 0,589657387 | 0,251478416 | |
| 244716_x_at | TMIGD2 | 0,841135803 | 0,589657387 | 0,251478416 | |
| 244808_at | GRAMD1A | 0,841135803 | 0,589657387 | 0,251478416 | |
| 202372_at | AURKAPS1 /// RA | 2,940912947 | 2,689475799 | 0,251437148 | |

| | | | | | |
|---|---|---|---|---|---|
| 1557457_at | - | 0,171847695 | -0,079522948 | 0,251370644 | |
| 232366_at | KIAA0232 | 0,171847695 | -0,079522948 | 0,251370644 | |
| 224206_x_at | MYNN | 2,680059304 | 2,428994491 | 0,251064814 | |
| 56919_at | WDR48 | 2,572726635 | 2,321742086 | 0,250984549 | |
| 213654_at | TAF5L | 1,823660386 | 1,572742379 | 0,250918007 | |
| 223982_s_at | PNPLA8 | 3,292909151 | 3,042049843 | 0,250859308 | |
| 227067_x_at | NOTCH2NL | 1,496984567 | 1,246149888 | 0,250834679 | |
| 222115_x_at | GLYR1 | 1,037706275 | 0,786897721 | 0,250808554 | |
| 230483_at | - | 1,037706275 | 0,786897721 | 0,250808554 | |
| 212748_at | MKL1 | 1,523472489 | 1,272721751 | 0,250750738 | |
| 226309_at | DNAL1 | 1,523472489 | 1,272721751 | 0,250750738 | |
| 207087_x_at | ANK1 | 1,331241794 | 1,080561626 | 0,250680168 | |
| 221613_s_at | ZFAND6 | 4,549751571 | 4,299127518 | 0,250624053 | |
| 1564429_at | - | -0,759588698 | -1,010192375 | 0,250603677 | |
| 205247_at | NOTCH4 | -0,759588698 | -1,010192375 | 0,250603677 | |
| 205568_at | AQP9 | -0,759588698 | -1,010192375 | 0,250603677 | |
| 206486_at | LAG3 | -0,759588698 | -1,010192375 | 0,250603677 | |
| 207446_at | TLR6 | -0,759588698 | -1,010192375 | 0,250603677 | |
| 210939_s_at | GRM1 | -0,759588698 | -1,010192375 | 0,250603677 | |
| 215036_at | IGLC1 | -0,759588698 | -1,010192375 | 0,250603677 | |
| 215110_at | LOC100288974 / | -0,759588698 | -1,010192375 | 0,250603677 | |
| 228742_at | - | -0,759588698 | -1,010192375 | 0,250603677 | |
| 237699_at | LINC00427 | -0,759588698 | -1,010192375 | 0,250603677 | |
| 239415_at | MAP9 | -0,759588698 | -1,010192375 | 0,250603677 | |
| 230177_at | GTF2H2B | 1,926486359 | 1,675891488 | 0,250594871 | |
| 32042_at | ENOX2 | 0,417596972 | 0,167023565 | 0,250573407 | |
| 211953_s_at | IPO5 | 4,546987586 | 4,296432174 | 0,250555412 | |
| 229885_at | RSF1 | 1,143956113 | 0,893473268 | 0,250482845 | |
| 213540_at | HSD17B8 | 1,805273023 | 1,55481039 | 0,250462633 | |
| 225598_at | SLC45A4 | 1,649074067 | 1,398700978 | 0,250373088 | |
| 228061_at | CCDC126 | 0,888485433 | 0,638150376 | 0,250335057 | |
| 232282_at | WNK3 | 2,446911392 | 2,196649505 | 0,250261887 | |
| 201386_s_at | DHX15 | 5,333754759 | 5,083502044 | 0,250252715 | |
| 1554887_at | - | -0,285837228 | -0,536087151 | 0,250249922 | |
| 1560278_at | LOC221122 | -0,285837228 | -0,536087151 | 0,250249922 | |
| 224072_s_at | KCNK9 | -0,285837228 | -0,536087151 | 0,250249922 | |
| 239476_at | - | -0,285837228 | -0,536087151 | 0,250249922 | |
| 243181_at | ANKIB1 | -0,285837228 | -0,536087151 | 0,250249922 | |
| 229650_s_at | C19orf42 | 4,170287948 | 3,920040657 | 0,250247291 | |
| 221190_s_at | C18orf8 | 2,194816625 | 1,944570415 | 0,25024621 | |
| 205517_at | GATA4 | -0,033456564 | -0,283667828 | 0,250211263 | |
| 207530_s_at | CDKN2B | -0,033456564 | -0,283667828 | 0,250211263 | |
| 215651_at | - | -0,033456564 | -0,283667828 | 0,250211263 | |
| 230313_at | - | -0,033456564 | -0,283667828 | 0,250211263 | |
| 234926_s_at | C20orf43 | 5,141940812 | 4,891829281 | 0,250111532 | |
| 201869_s_at | TBL1X | 1,305306849 | 1,055274826 | 0,250032024 | |
| 203725_at | GADD45A | 5,545425037 | 5,295413712 | 0,250011324 | |
| 218091_at | AGFG1 | 3,527851575 | 3,277845403 | 0,250006172 | |
| 34408_at | RTN2 | 2,737756747 | 2,487827245 | 0,249929503 | |
| 223469_at | PGPEP1 | 1,114389003 | 0,864649967 | 0,249739036 | |
| 238725_at | IRF1 | 1,114389003 | 0,864649967 | 0,249739036 | |
| 211961_s_at | RAB7A | 4,346467354 | 4,096815476 | 0,249651878 | |
| 1561402_at | LOC339894 | -1,173767127 | -1,423348341 | 0,249581215 | |
| 204644_at | ENOX2 | -1,173767127 | -1,423348341 | 0,249581215 | |
| 204989_s_at | ITGB4 | -1,173767127 | -1,423348341 | 0,249581215 | |
| 224241_s_at | - | -1,173767127 | -1,423348341 | 0,249581215 | |

| | | | | | |
|---|---|---|---|---|---|
| 231587_at | APOC3 | -1,173767127 | -1,423348341 | 0,249581215 | |
| 233297_s_at | CCDC169 /// CCI | -1,173767127 | -1,423348341 | 0,249581215 | |
| 241830_at | C20orf112 | -1,173767127 | -1,423348341 | 0,249581215 | |
| 1555345_at | SLC38A4 | -2,597496523 | -2,847001814 | 0,249505291 | |
| 1556933_at | - | -2,597496523 | -2,847001814 | 0,249505291 | |
| 1557094_at | LOC100652762 | -2,597496523 | -2,847001814 | 0,249505291 | |
| 1557512_at | - | -2,597496523 | -2,847001814 | 0,249505291 | |
| 1561461_at | - | -2,597496523 | -2,847001814 | 0,249505291 | |
| 215531_s_at | GABRA5 | -2,597496523 | -2,847001814 | 0,249505291 | |
| 217546_at | MT1M | -2,597496523 | -2,847001814 | 0,249505291 | |
| 220468_at | ARL14 | -2,597496523 | -2,847001814 | 0,249505291 | |
| 223949_at | TMPRSS3 | -2,597496523 | -2,847001814 | 0,249505291 | |
| 226237_at | COL8A1 | -2,597496523 | -2,847001814 | 0,249505291 | |
| 229857_s_at | KANSL1-AS1 | -2,597496523 | -2,847001814 | 0,249505291 | |
| 234204_at | - | -2,597496523 | -2,847001814 | 0,249505291 | |
| 234235_at | - | -2,597496523 | -2,847001814 | 0,249505291 | |
| 240059_at | - | -2,597496523 | -2,847001814 | 0,249505291 | |
| 240153_at | ILDR2 | -2,597496523 | -2,847001814 | 0,249505291 | |
| 242318_at | TAPT1 | -2,597496523 | -2,847001814 | 0,249505291 | |
| 242409_at | - | -2,597496523 | -2,847001814 | 0,249505291 | |
| 1556178_x_at | TAF8 | 1,81142822 | 1,562009954 | 0,249418266 | |
| 221050_s_at | GTPBP2 | 1,81142822 | 1,562009954 | 0,249418266 | |
| 208644_at | PARP1 | 4,301804532 | 4,052455702 | 0,24934883 | |
| 238879_at | DCUN1D1 | 2,247975581 | 1,998633167 | 0,249342414 | |
| 210904_s_at | IL13RA1 | 0,97328325 | 0,724100169 | 0,249183081 | |
| 202406_s_at | TIAL1 | 4,820834741 | 4,571665519 | 0,249169222 | |
| 219723_x_at | AGPAT3 | 1,153678675 | 0,904843258 | 0,248835417 | |
| 233021_at | RBM26-AS1 | 1,153678675 | 0,904843258 | 0,248835417 | |
| 218449_at | UFSP2 | 3,598072957 | 3,349287209 | 0,248785748 | |
| 213535_s_at | UBE2I | 5,442105403 | 5,193345242 | 0,248760161 | |
| 1557276_at | LOC100507584 | 1,58584542 | 1,337086025 | 0,248759395 | |
| 205654_at | C4BPA | 0,682279745 | 0,433557298 | 0,248722447 | |
| 219254_at | C17orf101 | 0,682279745 | 0,433557298 | 0,248722447 | |
| 219751_at | SETD6 | 0,682279745 | 0,433557298 | 0,248722447 | |
| 209007_s_at | C1orf63 | 4,328338611 | 4,079673902 | 0,24866471 | |
| 213062_at | NTAN1 | 2,611163891 | 2,362515214 | 0,248648677 | |
| 209857_s_at | SPHK2 | 0,728550685 | 0,479907041 | 0,248643644 | |
| 228118_x_at | C16orf13 | 0,728550685 | 0,479907041 | 0,248643644 | |
| 219124_at | TTI2 | 2,456762221 | 2,208167653 | 0,248594568 | |
| 222686_s_at | CPPED1 | 2,014487247 | 1,765933685 | 0,248553562 | |
| 222039_at | KIF18B | 3,358484112 | 3,109937707 | 0,248546405 | |
| 55081_at | MICALL1 | 1,562314261 | 1,313817865 | 0,248496395 | |
| 229961_x_at | YJEFN3 | 0,052199481 | -0,19610998 | 0,248309461 | |
| 212240_s_at | PIK3R1 | 1,485482016 | 1,23718272 | 0,248299296 | |
| 1556750_at | LOC153577 | -1,838812296 | -2,086984744 | 0,248172447 | |
| 1556753_s_at | - | -1,838812296 | -2,086984744 | 0,248172447 | |
| 1560030_at | LOC283692 | -1,838812296 | -2,086984744 | 0,248172447 | |
| 1562477_at | EBF2 | -1,838812296 | -2,086984744 | 0,248172447 | |
| 1564996_at | - | -1,838812296 | -2,086984744 | 0,248172447 | |
| 1565635_at | - | -1,838812296 | -2,086984744 | 0,248172447 | |
| 1565889_at | - | -1,838812296 | -2,086984744 | 0,248172447 | |
| 1566524_a_at | - | -1,838812296 | -2,086984744 | 0,248172447 | |
| 1570439_at | - | -1,838812296 | -2,086984744 | 0,248172447 | |
| 206522_at | MGAM | -1,838812296 | -2,086984744 | 0,248172447 | |
| 208423_s_at | MSR1 | -1,838812296 | -2,086984744 | 0,248172447 | |
| 209016_s_at | KRT7 | -1,838812296 | -2,086984744 | 0,248172447 | |

| | | | | | |
|---|---|---|---|---|---|
| 214134_at | C2orf55 | -1,838812296 | -2,086984744 | 0,248172447 | |
| 214204_at | PACRG | -1,838812296 | -2,086984744 | 0,248172447 | |
| 214234_s_at | CYP3A5 | -1,838812296 | -2,086984744 | 0,248172447 | |
| 214477_at | MLLT1 | -1,838812296 | -2,086984744 | 0,248172447 | |
| 216268_s_at | JAG1 | -1,838812296 | -2,086984744 | 0,248172447 | |
| 217226_s_at | SFXN3 | -1,838812296 | -2,086984744 | 0,248172447 | |
| 219876_s_at | GOLGA2P5 | -1,838812296 | -2,086984744 | 0,248172447 | |
| 220577_at | GVINP1 | -1,838812296 | -2,086984744 | 0,248172447 | |
| 229487_at | EBF1 | -1,838812296 | -2,086984744 | 0,248172447 | |
| 231174_s_at | - | -1,838812296 | -2,086984744 | 0,248172447 | |
| 234693_at | - | -1,838812296 | -2,086984744 | 0,248172447 | |
| 238090_at | HGSNAT | -1,838812296 | -2,086984744 | 0,248172447 | |
| 238878_at | ARX | -1,838812296 | -2,086984744 | 0,248172447 | |
| 240416_at | - | -1,838812296 | -2,086984744 | 0,248172447 | |
| 240694_at | - | -1,838812296 | -2,086984744 | 0,248172447 | |
| 242239_at | - | 0,255492549 | 0,007421914 | 0,248070635 | |
| 223570_at | MCM10 | 2,819431021 | 2,571362843 | 0,248068177 | |
| 213415_at | CLIC2 | 2,089851283 | 1,841838775 | 0,248012509 | |
| 228244_at | BLOC1S3 | 2,089851283 | 1,841838775 | 0,248012509 | |
| 218895_at | GPATCH3 | 1,709647566 | 1,461646549 | 0,248001016 | |
| 1560297_at | - | 0,376672223 | 0,128747141 | 0,247925083 | |
| 208332_at | LOC100509646 / | 0,376672223 | 0,128747141 | 0,247925083 | |
| 233244_at | - | 0,376672223 | 0,128747141 | 0,247925083 | |
| 204298_s_at | LOX | 0,541261317 | 0,293518836 | 0,247742481 | |
| 226803_at | CHMP4C | 0,641402394 | 0,393761504 | 0,24764089 | |
| 228683_s_at | KCTD15 | 0,641402394 | 0,393761504 | 0,24764089 | |
| 242766_at | - | 0,641402394 | 0,393761504 | 0,24764089 | |
| 201621_at | NBL1 | -0,366776884 | -0,614371577 | 0,247594693 | |
| 210099_at | ABCA2 | -0,366776884 | -0,614371577 | 0,247594693 | |
| 213265_at | PGA3 /// PGA4 // | -0,366776884 | -0,614371577 | 0,247594693 | |
| 217254_s_at | EPO | -0,366776884 | -0,614371577 | 0,247594693 | |
| 232844_at | IFT140 | -0,366776884 | -0,614371577 | 0,247594693 | |
| 234674_at | - | -0,366776884 | -0,614371577 | 0,247594693 | |
| 235219_at | C5orf55 | -0,366776884 | -0,614371577 | 0,247594693 | |
| 239036_at | EIF2C1 | -0,366776884 | -0,614371577 | 0,247594693 | |
| 244176_at | - | -0,366776884 | -0,614371577 | 0,247594693 | |
| 244491_at | - | -0,366776884 | -0,614371577 | 0,247594693 | |
| 1554897_s_at | RHBDL2 | -1,964176657 | -2,211750327 | 0,247573671 | |
| 1555163_at | - | -1,964176657 | -2,211750327 | 0,247573671 | |
| 1556072_at | LINC00528 | -1,964176657 | -2,211750327 | 0,247573671 | |
| 1556239_a_at | - | -1,964176657 | -2,211750327 | 0,247573671 | |
| 1558703_at | SLC46A1 | -1,964176657 | -2,211750327 | 0,247573671 | |
| 1561104_at | - | -1,964176657 | -2,211750327 | 0,247573671 | |
| 1561706_at | - | -1,964176657 | -2,211750327 | 0,247573671 | |
| 1563913_at | - | -1,964176657 | -2,211750327 | 0,247573671 | |
| 1564149_at | - | -1,964176657 | -2,211750327 | 0,247573671 | |
| 1564962_at | ZNF92 | -1,964176657 | -2,211750327 | 0,247573671 | |
| 1565616_at | - | -1,964176657 | -2,211750327 | 0,247573671 | |
| 1566709_at | - | -1,964176657 | -2,211750327 | 0,247573671 | |
| 203868_s_at | VCAM1 | -1,964176657 | -2,211750327 | 0,247573671 | |
| 205092_x_at | ZBTB1 | -1,964176657 | -2,211750327 | 0,247573671 | |
| 210268_at | NFX1 | -1,964176657 | -2,211750327 | 0,247573671 | |
| 213029_at | NFIB | -1,964176657 | -2,211750327 | 0,247573671 | |
| 214811_at | RIMBP2 | -1,964176657 | -2,211750327 | 0,247573671 | |
| 215065_at | PHF8 | -1,964176657 | -2,211750327 | 0,247573671 | |
| 216617_s_at | MAG | -1,964176657 | -2,211750327 | 0,247573671 | |

| | | | | | |
|---|---|---|---|---|---|
| 230835_at | KRTDAP | -1,964176657 | -2,211750327 | 0,247573671 | |
| 230838_s_at | CIAPIN1 | -1,964176657 | -2,211750327 | 0,247573671 | |
| 233808_at | - | -1,964176657 | -2,211750327 | 0,247573671 | |
| 238993_at | MATR3 | -1,964176657 | -2,211750327 | 0,247573671 | |
| 240905_at | - | -1,964176657 | -2,211750327 | 0,247573671 | |
| 241258_at | - | -1,964176657 | -2,211750327 | 0,247573671 | |
| 200835_s_at | MAP4 | 0,779676066 | 0,532164371 | 0,247511695 | |
| 1555725_a_at | RGS5 | -1,71423189 | -1,961547147 | 0,247315258 | |
| 1557770_at | IPO11 | -1,71423189 | -1,961547147 | 0,247315258 | |
| 1567357_at | NAV2 | -1,71423189 | -1,961547147 | 0,247315258 | |
| 206462_s_at | NTRK3 | -1,71423189 | -1,961547147 | 0,247315258 | |
| 207756_at | - | -1,71423189 | -1,961547147 | 0,247315258 | |
| 211524_at | NFKB2 | -1,71423189 | -1,961547147 | 0,247315258 | |
| 214860_at | SLC9A7 | -1,71423189 | -1,961547147 | 0,247315258 | |
| 216935_at | LINC00302 | -1,71423189 | -1,961547147 | 0,247315258 | |
| 222783_s_at | SMOC1 | -1,71423189 | -1,961547147 | 0,247315258 | |
| 228704_s_at | CLDN23 | -1,71423189 | -1,961547147 | 0,247315258 | |
| 232886_at | - | -1,71423189 | -1,961547147 | 0,247315258 | |
| 233592_at | ANKRD18B | -1,71423189 | -1,961547147 | 0,247315258 | |
| 237272_at | LOC100506907 | -1,71423189 | -1,961547147 | 0,247315258 | |
| 238287_at | SLC7A13 | -1,71423189 | -1,961547147 | 0,247315258 | |
| 240720_at | - | -1,71423189 | -1,961547147 | 0,247315258 | |
| 242956_at | - | -1,71423189 | -1,961547147 | 0,247315258 | |
| 221073_s_at | NOD1 | 1,094335495 | 0,847075464 | 0,247260031 | |
| 225711_at | ARL6IP6 | 1,965648824 | 1,718396606 | 0,247252218 | |
| 1552943_at | GABRG1 | -2,930741289 | -3,177802286 | 0,247060997 | |
| 1555357_at | DDX53 | -2,930741289 | -3,177802286 | 0,247060997 | |
| 1559147_at | - | -2,930741289 | -3,177802286 | 0,247060997 | |
| 1559839_at | TBX18 | -2,930741289 | -3,177802286 | 0,247060997 | |
| 1560538_at | - | -2,930741289 | -3,177802286 | 0,247060997 | |
| 1561855_x_at | - | -2,930741289 | -3,177802286 | 0,247060997 | |
| 206893_at | SALL1 | -2,930741289 | -3,177802286 | 0,247060997 | |
| 214587_at | COL8A1 | -2,930741289 | -3,177802286 | 0,247060997 | |
| 216874_at | - | -2,930741289 | -3,177802286 | 0,247060997 | |
| 224989_at | - | -2,930741289 | -3,177802286 | 0,247060997 | |
| 229199_at | SCN9A | -2,930741289 | -3,177802286 | 0,247060997 | |
| 237830_at | - | -2,930741289 | -3,177802286 | 0,247060997 | |
| 238900_at | HLA-DRB1 /// HL | -2,930741289 | -3,177802286 | 0,247060997 | |
| 244130_at | HTR2A | -2,930741289 | -3,177802286 | 0,247060997 | |
| 1553271_at | DIP2B | -2,64318869 | -2,89013181 | 0,246943121 | |
| 1555367_at | ZNF479 | -2,64318869 | -2,89013181 | 0,246943121 | |
| 1560673_at | - | -2,64318869 | -2,89013181 | 0,246943121 | |
| 1560676_at | SIAH3 | -2,64318869 | -2,89013181 | 0,246943121 | |
| 1561777_at | - | -2,64318869 | -2,89013181 | 0,246943121 | |
| 1566217_at | - | -2,64318869 | -2,89013181 | 0,246943121 | |
| 1570180_at | - | -2,64318869 | -2,89013181 | 0,246943121 | |
| 207706_at | USH2A | -2,64318869 | -2,89013181 | 0,246943121 | |
| 215303_at | DCLK1 | -2,64318869 | -2,89013181 | 0,246943121 | |
| 220402_at | TP53AIP1 | -2,64318869 | -2,89013181 | 0,246943121 | |
| 228218_at | LSAMP | -2,64318869 | -2,89013181 | 0,246943121 | |
| 230412_at | NPAS3 | -2,64318869 | -2,89013181 | 0,246943121 | |
| 231875_at | KIF21A | -2,64318869 | -2,89013181 | 0,246943121 | |
| 232839_at | - | -2,64318869 | -2,89013181 | 0,246943121 | |
| 235490_at | TMEM107 | -2,64318869 | -2,89013181 | 0,246943121 | |
| 235800_at | - | -2,64318869 | -2,89013181 | 0,246943121 | |
| 235978_at | FABP4 | -2,64318869 | -2,89013181 | 0,246943121 | |

| | | | | | |
|---|---|---|---|---|---|
| 237020_at | CATSPERD | -2,64318869 | -2,89013181 | 0,246943121 | |
| 240884_at | CCDC14 | -2,64318869 | -2,89013181 | 0,246943121 | |
| 242869_at | - | -2,64318869 | -2,89013181 | 0,246943121 | |
| 243378_at | - | -2,64318869 | -2,89013181 | 0,246943121 | |
| 243775_at | - | -2,64318869 | -2,89013181 | 0,246943121 | |
| 244443_at | CHD2 | -2,64318869 | -2,89013181 | 0,246943121 | |
| 226594_at | ENTPD5 | 2,646022818 | 2,39915583 | 0,246866988 | |
| 225212_at | SLC25A25 | 1,247462054 | 1,000684521 | 0,246777533 | |
| 227692_at | GNAI1 | 3,566577193 | 3,319860027 | 0,246717165 | |
| 224600_at | CGGBP1 | 3,097712429 | 2,851017268 | 0,246695161 | |
| 218243_at | RUFY1 | 4,563948403 | 4,317323511 | 0,246624891 | |
| 213743_at | CCNT2 | 2,522021211 | 2,275409734 | 0,246611477 | |
| 1558969_a_at | RPL32P3 | 0,20027154 | -0,046302147 | 0,246573686 | |
| 236467_at | ERICH1 /// FLJ00 | 0,20027154 | -0,046302147 | 0,246573686 | |
| 230562_at | LOC100507530 | 0,496120222 | 0,249604176 | 0,246516046 | |
| 234464_s_at | EME1 | 4,337431458 | 4,090916839 | 0,246514619 | |
| 233319_x_at | - | 0,87091093 | 0,624460759 | 0,246450171 | |
| 201634_s_at | CYB5B | 3,378314944 | 3,131875186 | 0,246439758 | |
| 220042_x_at | HIVEP3 | 1,168140757 | 0,921731975 | 0,246408782 | |
| 206473_at | MBTPS2 | 1,233777334 | 0,987376598 | 0,246400736 | |
| 1555765_a_at | GNG4 | 1,549482847 | 1,303116084 | 0,246366762 | |
| 1556082_a_at | - | -1,131062212 | -1,377419394 | 0,246357182 | |
| 1557042_at | KCNQ3 | -1,131062212 | -1,377419394 | 0,246357182 | |
| 1559491_at | - | -1,131062212 | -1,377419394 | 0,246357182 | |
| 1560353_at | - | -1,131062212 | -1,377419394 | 0,246357182 | |
| 1563121_at | - | -1,131062212 | -1,377419394 | 0,246357182 | |
| 220562_at | CYP2W1 | -1,131062212 | -1,377419394 | 0,246357182 | |
| 221271_at | IL21 | -1,131062212 | -1,377419394 | 0,246357182 | |
| 234323_at | THSD4 | -1,131062212 | -1,377419394 | 0,246357182 | |
| 241891_at | - | -1,131062212 | -1,377419394 | 0,246357182 | |
| 243132_at | APTX | -1,131062212 | -1,377419394 | 0,246357182 | |
| 1555831_s_at | LRRC41 | 1,932146506 | 1,685812099 | 0,246334406 | |
| 203248_at | ZNF24 | 2,027765132 | 1,781437557 | 0,246327575 | |
| 1566185_at | - | -0,172274514 | -0,41858459 | 0,246310076 | |
| 228003_at | RAB30 | -0,172274514 | -0,41858459 | 0,246310076 | |
| 242069_at | CBX5 | -0,172274514 | -0,41858459 | 0,246310076 | |
| 218020_s_at | ZFAND3 | 2,44295215 | 2,196649505 | 0,246302645 | |
| 219540_at | ZNF267 | 3,037284587 | 2,791007185 | 0,246277403 | |
| 211063_s_at | NCK1 | 3,563845287 | 3,317734938 | 0,246110349 | |
| 224682_at | ANKIB1 | 3,101484927 | 2,855418981 | 0,246065946 | |
| 209025_s_at | SYNCRIP | 5,916776089 | 5,670779353 | 0,245996736 | |
| 236555_at | TRAF3IP2-AS1 | 1,360921491 | 1,115234684 | 0,245686807 | |
| 1553911_at | ZNF663 | -2,089474779 | -2,335104118 | 0,245629339 | |
| 1557862_at | LOC654841 | -2,089474779 | -2,335104118 | 0,245629339 | |
| 1559771_at | - | -2,089474779 | -2,335104118 | 0,245629339 | |
| 1561333_at | - | -2,089474779 | -2,335104118 | 0,245629339 | |
| 1563849_at | SH2D4B | -2,089474779 | -2,335104118 | 0,245629339 | |
| 1566113_at | - | -2,089474779 | -2,335104118 | 0,245629339 | |
| 202565_s_at | SVIL | -2,089474779 | -2,335104118 | 0,245629339 | |
| 205643_s_at | PPP2R2B | -2,089474779 | -2,335104118 | 0,245629339 | |
| 207291_at | PRRG4 | -2,089474779 | -2,335104118 | 0,245629339 | |
| 215802_at | - | -2,089474779 | -2,335104118 | 0,245629339 | |
| 216135_at | IQCK | -2,089474779 | -2,335104118 | 0,245629339 | |
| 216744_at | - | -2,089474779 | -2,335104118 | 0,245629339 | |
| 234050_at | TAGAP | -2,089474779 | -2,335104118 | 0,245629339 | |
| 234259_at | - | -2,089474779 | -2,335104118 | 0,245629339 | |

| | | | | | |
|---|---|---|---|---|---|
| 237072_at | - | -2,089474779 | -2,335104118 | 0,245629339 | |
| 238608_at | - | -2,089474779 | -2,335104118 | 0,245629339 | |
| 240533_at | - | -2,089474779 | -2,335104118 | 0,245629339 | |
| 240679_at | - | -2,089474779 | -2,335104118 | 0,245629339 | |
| 243124_at | RRN3P2 | -2,089474779 | -2,335104118 | 0,245629339 | |
| 243726_at | - | -2,089474779 | -2,335104118 | 0,245629339 | |
| 214440_at | NAT1 | 3,361633475 | 3,116064987 | 0,245568488 | |
| 227594_at | ZMYM6 | 0,917308734 | 0,67181667 | 0,245492064 | |
| 205780_at | BIK | 0,702292136 | 0,456918297 | 0,245373839 | |
| 239603_x_at | - | 0,702292136 | 0,456918297 | 0,245373839 | |
| 241367_at | TEX19 | 0,702292136 | 0,456918297 | 0,245373839 | |
| 203538_at | CAMLG | 3,191547726 | 2,946258252 | 0,245289474 | |
| 218832_x_at | ARRB1 | 0,555999837 | 0,310717132 | 0,245282705 | |
| 212005_at | C1orf144 | 2,225103271 | 1,979940779 | 0,245162492 | |
| 203956_at | MORC2 | 5,027958036 | 4,782818545 | 0,24513949 | |
| 203853_s_at | GAB2 | 1,504602299 | 1,259496994 | 0,245105305 | |
| 1561403_at | SOHLH1 | 0,273439642 | 0,028364014 | 0,245075628 | |
| 220303_at | PDZD3 | 0,273439642 | 0,028364014 | 0,245075628 | |
| 202427_s_at | BRP44 | 3,988699475 | 3,743690278 | 0,245009197 | |
| 203593_at | CD2AP | 3,838564178 | 3,593688577 | 0,244875601 | |
| 228225_at | PEX2 | 1,177702228 | 0,932882274 | 0,244819954 | |
| 1557436_at | XKR6 | -0,57445847 | -0,819238336 | 0,244779866 | |
| 1558167_a_at | MGC16275 | -0,57445847 | -0,819238336 | 0,244779866 | |
| 1566901_at | TGIF1 | -0,57445847 | -0,819238336 | 0,244779866 | |
| 210493_s_at | MFAP3L | -0,57445847 | -0,819238336 | 0,244779866 | |
| 215592_at | - | -0,57445847 | -0,819238336 | 0,244779866 | |
| 216404_at | ATXN8OS | -0,57445847 | -0,819238336 | 0,244779866 | |
| 217715_x_at | - | -0,57445847 | -0,819238336 | 0,244779866 | |
| 220164_s_at | FBXO40 | -0,57445847 | -0,819238336 | 0,244779866 | |
| 220588_at | BCAS4 | -0,57445847 | -0,819238336 | 0,244779866 | |
| 222022_at | DTX3 | -0,57445847 | -0,819238336 | 0,244779866 | |
| 232272_at | ZNF624 | -0,57445847 | -0,819238336 | 0,244779866 | |
| 233469_at | TPTEP1 | -0,57445847 | -0,819238336 | 0,244779866 | |
| 238530_at | NNT | -0,57445847 | -0,819238336 | 0,244779866 | |
| 244396_at | G3BP1 | -0,57445847 | -0,819238336 | 0,244779866 | |
| 214855_s_at | RALGAPA1 | 1,751865623 | 1,507116528 | 0,244749095 | |
| 1558254_s_at | SRPK2 | 2,194816625 | 1,950068772 | 0,244747853 | |
| 201974_s_at | CCZ1 | -0,078265071 | -0,322870281 | 0,24460521 | |
| 222261_at | KIAA1609 | -0,078265071 | -0,322870281 | 0,24460521 | |
| 230072_at | TBC1D15 | -0,078265071 | -0,322870281 | 0,24460521 | |
| 239497_at | - | -0,078265071 | -0,322870281 | 0,24460521 | |
| 203762_s_at | DYNC2LI1 | 2,05655332 | 1,811954245 | 0,244599076 | |
| 206472_s_at | TLE3 | 0,882651021 | 0,638150376 | 0,244500645 | |
| 215292_s_at | MKL1 | 0,882651021 | 0,638150376 | 0,244500645 | |
| 221208_s_at | MSANTD2 | 1,903621061 | 1,659204017 | 0,244417045 | |
| 230158_at | DPY19L2 | 1,903621061 | 1,659204017 | 0,244417045 | |
| 224149_x_at | LOC100287789 / | 0,798390451 | 0,553993624 | 0,244396827 | |
| 225426_at | PPP6C | 2,482063962 | 2,237691445 | 0,244372517 | |
| 225339_at | SPAG9 | 2,094906514 | 1,85068474 | 0,244221774 | |
| 212319_at | SGSM2 | 0,661985843 | 0,417770464 | 0,244215379 | |
| 215253_s_at | RCAN1 | 0,661985843 | 0,417770464 | 0,244215379 | |
| 1554878_a_at | ABCD3 | 2,003776245 | 1,759685182 | 0,244091063 | |
| 241827_at | ZNF615 | 2,003776245 | 1,759685182 | 0,244091063 | |
| 217127_at | CTH | 1,946200473 | 1,702196344 | 0,244004128 | |
| 202034_x_at | RB1CC1 | 4,158264286 | 3,914426433 | 0,243837853 | |
| 222386_s_at | COPZ1 | 4,574391988 | 4,330558009 | 0,243833979 | |

| | | | | | |
|---|---|---|---|---|---|
| 212906_at | GRAMD1B | 0,401366113 | 0,157549243 | 0,24381687 | |
| 214944_at | PHLPP2 | 1,823660386 | 1,579853228 | 0,243807158 | |
| 1553072_at | BNIPL | -1,543331864 | -1,787089803 | 0,243757939 | |
| 1555204_at | - | -1,543331864 | -1,787089803 | 0,243757939 | |
| 1555237_at | - | -1,543331864 | -1,787089803 | 0,243757939 | |
| 1557841_at | - | -1,543331864 | -1,787089803 | 0,243757939 | |
| 1568997_at | POLR1E | -1,543331864 | -1,787089803 | 0,243757939 | |
| 214878_at | ZNF37A | -1,543331864 | -1,787089803 | 0,243757939 | |
| 215066_at | PTPRF | -1,543331864 | -1,787089803 | 0,243757939 | |
| 215885_at | SSX2 | -1,543331864 | -1,787089803 | 0,243757939 | |
| 217000_at | - | -1,543331864 | -1,787089803 | 0,243757939 | |
| 217664_at | - | -1,543331864 | -1,787089803 | 0,243757939 | |
| 222324_at | - | -1,543331864 | -1,787089803 | 0,243757939 | |
| 222895_s_at | BCL11B | -1,543331864 | -1,787089803 | 0,243757939 | |
| 224212_s_at | PCDHA1 /// PCD | -1,543331864 | -1,787089803 | 0,243757939 | |
| 235794_at | MOBP | -1,543331864 | -1,787089803 | 0,243757939 | |
| 236856_x_at | - | -1,543331864 | -1,787089803 | 0,243757939 | |
| 237590_at | - | -1,543331864 | -1,787089803 | 0,243757939 | |
| 240871_at | FAM221B | -1,543331864 | -1,787089803 | 0,243757939 | |
| 241476_at | - | -1,543331864 | -1,787089803 | 0,243757939 | |
| 243399_at | - | -1,543331864 | -1,787089803 | 0,243757939 | |
| 1553973_a_at | SPINK6 | -0,706943342 | -0,950686014 | 0,243742673 | |
| 219827_at | UCP3 | -0,706943342 | -0,950686014 | 0,243742673 | |
| 220590_at | ITFG2 | -0,706943342 | -0,950686014 | 0,243742673 | |
| 221442_at | MC3R | -0,706943342 | -0,950686014 | 0,243742673 | |
| 222274_at | ZDHHC8P1 | -0,706943342 | -0,950686014 | 0,243742673 | |
| 222803_at | PRTFDC1 | 1,373456798 | 1,129843209 | 0,243613589 | |
| 219709_x_at | FAM173A | 2,402750389 | 2,159161028 | 0,243589361 | |
| 203422_at | POLD1 | 2,825526235 | 2,582026171 | 0,243500064 | |
| 226475_at | FAM118A | 1,64218396 | 1,398700978 | 0,243482982 | |
| 235060_at | LOC100190986 | 1,64218396 | 1,398700978 | 0,243482982 | |
| 208643_s_at | XRCC5 | 4,714000909 | 4,470561285 | 0,243439624 | |
| 215544_s_at | UBOX5 | 0,760715727 | 0,51742585 | 0,243289876 | |
| 220707_s_at | FOXRED2 | 0,760715727 | 0,51742585 | 0,243289876 | |
| 209273_s_at | ISCA1 | 3,515651309 | 3,272369332 | 0,243281977 | |
| 227973_at | C2orf69 | 3,482282213 | 3,239069096 | 0,243213117 | |
| 202988_s_at | RGS1 | 0,083054335 | -0,160136748 | 0,243191083 | |
| 228337_at | PWWP2A | 0,083054335 | -0,160136748 | 0,243191083 | |
| 221741_s_at | YTHDF1 | 4,756492437 | 4,51343626 | 0,243056176 | |
| 209666_s_at | CHUK | 3,614006933 | 3,370970266 | 0,243036667 | |
| 212745_s_at | BBS4 | 1,69307511 | 1,450051551 | 0,24302356 | |
| 1557474_at | LOC284578 | -1,089016378 | -1,332017329 | 0,243000951 | |
| 1557783_at | LOC100133991 | -1,089016378 | -1,332017329 | 0,243000951 | |
| 1559494_at | SLC35G2 | -1,089016378 | -1,332017329 | 0,243000951 | |
| 1561413_at | - | -1,089016378 | -1,332017329 | 0,243000951 | |
| 1564868_a_at | FAM117B | -1,089016378 | -1,332017329 | 0,243000951 | |
| 206558_at | SIM2 | -1,089016378 | -1,332017329 | 0,243000951 | |
| 208032_s_at | GRIA3 | -1,089016378 | -1,332017329 | 0,243000951 | |
| 214413_at | TAT | -1,089016378 | -1,332017329 | 0,243000951 | |
| 220779_at | PADI3 | -1,089016378 | -1,332017329 | 0,243000951 | |
| 223725_at | GHRLOS2 | -1,089016378 | -1,332017329 | 0,243000951 | |
| 229172_at | HSPA12B | -1,089016378 | -1,332017329 | 0,243000951 | |
| 229769_at | TMEM242 | -1,089016378 | -1,332017329 | 0,243000951 | |
| 233456_at | - | -1,089016378 | -1,332017329 | 0,243000951 | |
| 233640_x_at | KRTAP9-4 | -1,089016378 | -1,332017329 | 0,243000951 | |
| 236125_at | - | -1,089016378 | -1,332017329 | 0,243000951 | |

| | | | | | |
|---|---|---|---|---|---|
| 238872_at | LOC100128239 | -1,089016378 | -1,332017329 | 0,243000951 | |
| 238935_at | RPS27L | 1,04816921 | 0,805216064 | 0,242953146 | |
| 205181_at | ZNF193 | 2,084778276 | 1,841838775 | 0,242939502 | |
| 218786_at | NT5DC3 | 1,011212064 | 0,768343791 | 0,242868273 | |
| 222778_s_at | WHSC1 | 1,011212064 | 0,768343791 | 0,242868273 | |
| 223134_at | BBX | 1,011212064 | 0,768343791 | 0,242868273 | |
| 219207_at | EDC3 | 1,348276312 | 1,105412836 | 0,242863476 | |
| 227986_at | ZNF343 | 1,850809354 | 1,60795124 | 0,242858115 | |
| 225606_at | BCL2L11 | 3,764356332 | 3,521663705 | 0,242692627 | |
| 227366_at | RILP | 3,24026957 | 2,997606395 | 0,242663175 | |
| 208910_s_at | C1QBP | 6,399037585 | 6,156398017 | 0,242639567 | |
| 1553608_a_at | LINC00189 | -2,712026959 | -2,954576755 | 0,242549796 | |
| 1554329_x_at | STXBP4 | -2,712026959 | -2,954576755 | 0,242549796 | |
| 1554636_at | - | -2,712026959 | -2,954576755 | 0,242549796 | |
| 1561330_at | DSG4 | -2,712026959 | -2,954576755 | 0,242549796 | |
| 1563022_at | CCDC160 | -2,712026959 | -2,954576755 | 0,242549796 | |
| 1569334_at | STRA6 | -2,712026959 | -2,954576755 | 0,242549796 | |
| 216478_at | - | -2,712026959 | -2,954576755 | 0,242549796 | |
| 216999_at | EPOR | -2,712026959 | -2,954576755 | 0,242549796 | |
| 224098_at | - | -2,712026959 | -2,954576755 | 0,242549796 | |
| 229435_at | GLIS3 | -2,712026959 | -2,954576755 | 0,242549796 | |
| 232429_at | - | -2,712026959 | -2,954576755 | 0,242549796 | |
| 236327_at | - | -2,712026959 | -2,954576755 | 0,242549796 | |
| 238176_at | RAPGEF2 | -2,712026959 | -2,954576755 | 0,242549796 | |
| 238314_x_at | - | -2,712026959 | -2,954576755 | 0,242549796 | |
| 242929_at | - | -2,712026959 | -2,954576755 | 0,242549796 | |
| 244014_x_at | - | -2,712026959 | -2,954576755 | 0,242549796 | |
| 244736_at | - | -2,712026959 | -2,954576755 | 0,242549796 | |
| 244780_at | SGPP2 | -2,712026959 | -2,954576755 | 0,242549796 | |
| 55662_at | C10orf76 | 1,46610492 | 1,223626587 | 0,242478333 | |
| 225556_at | VMA21 | 5,078917028 | 4,836499048 | 0,24241798 | |
| 1555420_a_at | KLF7 | -0,148196756 | -0,39061235 | 0,242415594 | |
| 1566134_at | CARHSP1 | -0,148196756 | -0,39061235 | 0,242415594 | |
| 210419_at | BARX2 | -0,148196756 | -0,39061235 | 0,242415594 | |
| 216332_at | POU6F1 | -0,148196756 | -0,39061235 | 0,242415594 | |
| 223435_s_at | PCDHA1 /// PCD | -0,148196756 | -0,39061235 | 0,242415594 | |
| 242552_x_at | ZBED5 | -0,148196756 | -0,39061235 | 0,242415594 | |
| 222327_x_at | OR7E156P | 0,810733188 | 0,568365095 | 0,242368092 | |
| 204262_s_at | PSEN2 | 0,351548291 | 0,109221392 | 0,242326899 | |
| 215723_s_at | PLD1 | 0,351548291 | 0,109221392 | 0,242326899 | |
| 202163_s_at | CNOT8 | 3,352164686 | 3,109937707 | 0,242226979 | |
| 226551_at | RIPK1 | 2,396622324 | 2,154405725 | 0,242216598 | |
| 214299_at | TOP3A | 1,089278259 | 0,847075464 | 0,242202795 | |
| 201197_at | AMD1 | 5,133981558 | 4,891829281 | 0,242152277 | |
| 200890_s_at | SSR1 | 4,329947401 | 4,08780258 | 0,242144822 | |
| 215450_at | SNRPE | 4,497501866 | 4,25538582 | 0,242116047 | |
| 226922_at | RANBP2 | 2,227406889 | 1,98530622 | 0,242100669 | |
| 1555051_at | C10orf53 | -0,43775896 | -0,679763839 | 0,242004879 | |
| 1564658_at | NAT16 | -0,43775896 | -0,679763839 | 0,242004879 | |
| 207491_at | MOGAT2 | -0,43775896 | -0,679763839 | 0,242004879 | |
| 211001_at | TRIM29 | -0,43775896 | -0,679763839 | 0,242004879 | |
| 232988_at | KIAA0182 | -0,43775896 | -0,679763839 | 0,242004879 | |
| 233398_at | - | -0,43775896 | -0,679763839 | 0,242004879 | |
| 240588_at | - | -0,43775896 | -0,679763839 | 0,242004879 | |
| 216092_s_at | SLC7A8 | 1,523472489 | 1,281471383 | 0,242001106 | |
| 201838_s_at | SUPT7L | 0,67554678 | 0,433557298 | 0,241989482 | |

| | | | | | |
|---|---|---|---|---|---|
| 205614_x_at | MST1 | 0,67554678 | 0,433557298 | 0,241989482 | |
| 201285_at | MKRN1 | 4,818544611 | 4,576561638 | 0,241982973 | |
| 204060_s_at | PRKX /// PRKY | 2,178241192 | 1,936283387 | 0,241957804 | |
| 208892_s_at | DUSP6 | 3,517535002 | 3,275657468 | 0,241877534 | |
| 227624_at | TET2 | 1,442503549 | 1,200746069 | 0,241757479 | |
| 1570607_at | - | 0,57782909 | 0,336136178 | 0,241692913 | |
| 241387_at | - | 0,57782909 | 0,336136178 | 0,241692913 | |
| 218853_s_at | MOSPD1 | 2,44295215 | 2,201267805 | 0,241684345 | |
| 235824_at | - | 0,900083947 | 0,658444279 | 0,241639668 | |
| 203816_at | DGUOK | 2,981056773 | 2,739547836 | 0,241508937 | |
| 214291_at | RPL17 /// RPL17- | 2,001086023 | 1,759685182 | 0,241400841 | |
| 213524_s_at | G0S2 | 3,335176289 | 3,09388391 | 0,241292379 | |
| 1552501_a_at | GPBAR1 | 0,020670649 | -0,220592223 | 0,241262873 | |
| 220420_at | LMAN1L | 0,020670649 | -0,220592223 | 0,241262873 | |
| 233030_at | PNPLA3 | 0,020670649 | -0,220592223 | 0,241262873 | |
| 233149_at | - | 0,020670649 | -0,220592223 | 0,241262873 | |
| 237336_at | ADD2 | 0,020670649 | -0,220592223 | 0,241262873 | |
| 211535_s_at | FGFR1 | 2,077135162 | 1,835911187 | 0,241223975 | |
| 212063_at | CD44 | 3,541801785 | 3,300620584 | 0,241181201 | |
| 238597_at | ANKRD13C | 0,417596971 | 0,176436073 | 0,241160897 | |
| 224614_at | DYNC1LI2 | 1,631786706 | 1,390636111 | 0,241150595 | |
| 212231_at | FBXO21 | 2,281618189 | 2,040470494 | 0,241147695 | |
| 1569345_at | - | -0,325749314 | -0,566887459 | 0,241138145 | |
| 206401_s_at | MAPT | -0,325749314 | -0,566887459 | 0,241138145 | |
| 231811_at | LOC100134445 / | -0,325749314 | -0,566887459 | 0,241138145 | |
| 201370_s_at | CUL3 | 2,644299732 | 2,403170156 | 0,241129576 | |
| 212891_s_at | GADD45GIP1 | 3,381421359 | 3,140317145 | 0,241104214 | |
| 1558606_s_at | - | -2,262747984 | -2,503677622 | 0,240929638 | |
| 1560413_at | LOC339788 | -2,262747984 | -2,503677622 | 0,240929638 | |
| 1562802_at | LINC00210 | -2,262747984 | -2,503677622 | 0,240929638 | |
| 201983_s_at | EGFR | -2,262747984 | -2,503677622 | 0,240929638 | |
| 206518_s_at | RGS9 | -2,262747984 | -2,503677622 | 0,240929638 | |
| 208346_at | PPBPP2 | -2,262747984 | -2,503677622 | 0,240929638 | |
| 214307_at | HGD | -2,262747984 | -2,503677622 | 0,240929638 | |
| 216664_at | - | -2,262747984 | -2,503677622 | 0,240929638 | |
| 217582_at | - | -2,262747984 | -2,503677622 | 0,240929638 | |
| 228137_s_at | PPP2R2C | -2,262747984 | -2,503677622 | 0,240929638 | |
| 230730_at | SGCD | -2,262747984 | -2,503677622 | 0,240929638 | |
| 235021_at | KIAA2026 | -2,262747984 | -2,503677622 | 0,240929638 | |
| 237288_at | TGM7 | -2,262747984 | -2,503677622 | 0,240929638 | |
| 240266_at | - | -2,262747984 | -2,503677622 | 0,240929638 | |
| 240582_x_at | - | -2,262747984 | -2,503677622 | 0,240929638 | |
| 244545_at | LOC100652770 | -2,262747984 | -2,503677622 | 0,240929638 | |
| 244551_at | FLJ43489 | -2,262747984 | -2,503677622 | 0,240929638 | |
| 219021_at | RNF121 | 1,556829052 | 1,315948728 | 0,240880324 | |
| 218081_at | C20orf27 | 3,088871381 | 2,848075314 | 0,240796067 | |
| 226125_at | LOC100288152 | 1,331241794 | 1,090553537 | 0,240688257 | |
| 204831_at | CDK8 | 3,821129803 | 3,580454966 | 0,240674837 | |
| 206937_at | SPTA1 | 3,912008086 | 3,671470423 | 0,240537662 | |
| 212508_at | MOAP1 | 3,982575608 | 3,742106442 | 0,240469165 | |
| 222497_x_at | NMD3 | 4,366985384 | 4,126559358 | 0,240426027 | |
| 226634_at | METTL10 | 0,533835206 | 0,293518836 | 0,240316371 | |
| 1560830_a_at | LOC147646 | 1,027166904 | 0,786897721 | 0,240269183 | |
| 225764_at | ETV6 | 3,540875956 | 3,300620584 | 0,240255372 | |
| 226792_s_at | KIFC2 | 1,394109935 | 1,153866761 | 0,240243174 | |
| 209188_x_at | DR1 | 3,50144467 | 3,261354437 | 0,240090233 | |

| | | | | | |
|---|---|---|---|---|---|
| 1861_at | BAD | 0,613492853 | 0,373444244 | 0,240048609 | |
| 232560_at | UROS | 0,735041319 | 0,495031851 | 0,240009469 | |
| 1555908_at | FAM120A | -0,221656139 | -0,461594427 | 0,239938288 | |
| 227323_at | COX4I1 | -0,221656139 | -0,461594427 | 0,239938288 | |
| 239383_at | - | -0,221656139 | -0,461594427 | 0,239938288 | |
| 229780_at | - | 0,103263601 | -0,136643983 | 0,239907584 | |
| 217039_x_at | ELK2AP /// LOC1 | 2,482063962 | 2,242180416 | 0,239883546 | |
| 219178_at | QTRTD1 | 1,305306849 | 1,065442793 | 0,239864056 | |
| 1563591_at | LOC100506403 / | -2,756478458 | -2,996335703 | 0,239857245 | |
| 1565525_a_at | TCP11L2 | -2,756478458 | -2,996335703 | 0,239857245 | |
| 1567174_at | - | -2,756478458 | -2,996335703 | 0,239857245 | |
| 206198_s_at | CEACAM7 | -2,756478458 | -2,996335703 | 0,239857245 | |
| 214887_at | N4BP2L1 | -2,756478458 | -2,996335703 | 0,239857245 | |
| 222213_x_at | - | -2,756478458 | -2,996335703 | 0,239857245 | |
| 231391_at | CTXN3 | -2,756478458 | -2,996335703 | 0,239857245 | |
| 234057_at | - | -2,756478458 | -2,996335703 | 0,239857245 | |
| 234374_at | - | -2,756478458 | -2,996335703 | 0,239857245 | |
| 236650_at | - | -2,756478458 | -2,996335703 | 0,239857245 | |
| 240349_at | PRKAA2 | -2,756478458 | -2,996335703 | 0,239857245 | |
| 241457_at | - | -2,756478458 | -2,996335703 | 0,239857245 | |
| 208623_s_at | EZR | 3,482282213 | 3,242433922 | 0,239848291 | |
| 205794_s_at | NOVA1 | 1,454352496 | 1,214517879 | 0,239834618 | |
| 229872_s_at | LOC100132999 / | 1,454352496 | 1,214517879 | 0,239834618 | |
| 230490_x_at | RSU1 | 2,382221458 | 2,14244844 | 0,239773018 | |
| 200719_at | SKP1 | 1,932146506 | 1,692388143 | 0,239758363 | |
| 225213_at | PPTC7 | 4,445269027 | 4,205686812 | 0,239582214 | |
| 220175_s_at | CBWD1 /// CBWI | 3,484209953 | 3,244672787 | 0,239537166 | |
| 230621_at | IAH1 | 2,470441349 | 2,230931691 | 0,239509658 | |
| 212037_at | PNN | 5,193516382 | 4,954036493 | 0,23947989 | |
| 209154_at | P2RX5-TAX1BP3 | 4,887536743 | 4,648076762 | 0,239459982 | |
| 203467_at | PMM1 | 1,369290453 | 1,129843209 | 0,239447244 | |
| 215011_at | SNHG3 /// SNOR | 1,369290453 | 1,129843209 | 0,239447244 | |
| 225073_at | PPHLN1 | 4,78999665 | 4,550556522 | 0,239440127 | |
| 1557673_at | - | -0,67289328 | -0,912331589 | 0,239438309 | |
| 207112_s_at | GAB1 | -0,67289328 | -0,912331589 | 0,239438309 | |
| 207493_x_at | SSX2 /// SSX2B | -0,67289328 | -0,912331589 | 0,239438309 | |
| 216119_s_at | SPEF1 | -0,67289328 | -0,912331589 | 0,239438309 | |
| 224371_at | ADAMTSL1 | -0,67289328 | -0,912331589 | 0,239438309 | |
| 226034_at | DUSP4 | -0,67289328 | -0,912331589 | 0,239438309 | |
| 229047_at | PLEKHB1 | -0,67289328 | -0,912331589 | 0,239438309 | |
| 230822_at | TMEM61 | -0,67289328 | -0,912331589 | 0,239438309 | |
| 240996_at | - | -0,67289328 | -0,912331589 | 0,239438309 | |
| 243083_at | LOC100287704 / | -0,67289328 | -0,912331589 | 0,239438309 | |
| 244514_at | - | -0,67289328 | -0,912331589 | 0,239438309 | |
| 244604_at | - | -0,67289328 | -0,912331589 | 0,239438309 | |
| 211792_s_at | CDKN2C | 0,82905153 | 0,589657387 | 0,239394143 | |
| 1555746_at | CD79B | -1,424023738 | -1,663414495 | 0,239390756 | |
| 1556110_at | - | -1,424023738 | -1,663414495 | 0,239390756 | |
| 1559405_a_at | TRPV6 | -1,424023738 | -1,663414495 | 0,239390756 | |
| 1559485_at | ATG2B | -1,424023738 | -1,663414495 | 0,239390756 | |
| 1564338_at | - | -1,424023738 | -1,663414495 | 0,239390756 | |
| 207045_at | CCDC132 | -1,424023738 | -1,663414495 | 0,239390756 | |
| 208570_at | WNT1 | -1,424023738 | -1,663414495 | 0,239390756 | |
| 214091_s_at | GPX3 | -1,424023738 | -1,663414495 | 0,239390756 | |
| 217327_at | - | -1,424023738 | -1,663414495 | 0,239390756 | |
| 217581_at | - | -1,424023738 | -1,663414495 | 0,239390756 | |

| | | | | | |
|---|---|---|---|---|---|
| 225293_at | COL27A1 | -1,424023738 | -1,663414495 | 0,239390756 | |
| 231092_s_at | - | -1,424023738 | -1,663414495 | 0,239390756 | |
| 231458_at | LOC100507629 | -1,424023738 | -1,663414495 | 0,239390756 | |
| 234393_at | HDAC9 | -1,424023738 | -1,663414495 | 0,239390756 | |
| 236456_at | PTPN5 | -1,424023738 | -1,663414495 | 0,239390756 | |
| 239398_at | KLHL31 | -1,424023738 | -1,663414495 | 0,239390756 | |
| 243380_at | - | -1,424023738 | -1,663414495 | 0,239390756 | |
| 244699_at | AHI1 | -1,424023738 | -1,663414495 | 0,239390756 | |
| 1558540_s_at | SLC2A11 | 0,592200562 | 0,352836757 | 0,239363804 | |
| 229360_at | ZNF280B | 0,592200562 | 0,352836757 | 0,239363804 | |
| 213012_at | NEDD4 | 1,485482016 | 1,246149888 | 0,239332127 | |
| 225092_at | RABEP1 | 2,994644467 | 2,755336312 | 0,239308156 | |
| 226568_at | FAM102B | 4,076291169 | 3,837157105 | 0,239134064 | |
| 203884_s_at | RAB11FIP2 | 1,143956113 | 0,904843258 | 0,239112855 | |
| 223186_at | TMEM189 /// TMI | 3,663333942 | 3,424287521 | 0,239046421 | |
| 242881_x_at | LOC100506303 / | 2,842156496 | 2,603119255 | 0,239037241 | |
| 215781_s_at | TOP3B | 0,246434561 | 0,007421914 | 0,239012647 | |
| 224669_at | SYS1 | 1,85379461 | 1,614891062 | 0,238903548 | |
| 209473_at | ENTPD1 | 0,695652136 | 0,456918297 | 0,238733839 | |
| 203576_at | BCAT2 | 2,671625168 | 2,432926755 | 0,238698413 | |
| 214173_x_at | URI1 | 6,6397013 | 6,401025631 | 0,238675669 | |
| 238010_at | C1orf174 | 2,250242994 | 2,011838132 | 0,238404862 | |
| 204120_s_at | ADK | 1,940595304 | 1,702196344 | 0,23839896 | |
| 200769_s_at | MAT2A | 3,086335384 | 2,848075314 | 0,23826007 | |
| 233970_s_at | TRMT6 | 4,230671025 | 3,992437318 | 0,238233706 | |
| 213642_at | - | 2,218170265 | 1,979940779 | 0,238229486 | |
| 210620_s_at | GTF3C2 | 0,792179257 | 0,553993624 | 0,238185633 | |
| 231938_at | SGOL1 | 0,962260738 | 0,724100169 | 0,238160569 | |
| 228234_at | TICAM2 /// TMED | 1,251994932 | 1,013870809 | 0,238124123 | |
| 228752_at | EFCAB4B | 2,236584753 | 1,998633167 | 0,237951585 | |
| 217601_at | NUP188 | 0,317354302 | 0,079428135 | 0,237926167 | |
| 220260_at | TBC1D19 | 0,317354302 | 0,079428135 | 0,237926167 | |
| 221410_x_at | PCDHB3 | 0,317354302 | 0,079428135 | 0,237926167 | |
| 239662_x_at | TMCC1 | 0,317354302 | 0,079428135 | 0,237926167 | |
| 206057_x_at | SPN | 2,348755185 | 2,110887591 | 0,237867594 | |
| 219354_at | KLHL26 | 1,318332599 | 1,080561626 | 0,237770973 | |
| 235919_at | CEP78 | 1,318332599 | 1,080561626 | 0,237770973 | |
| 235476_at | TRIM59 | 2,043538949 | 1,805902329 | 0,23763662 | |
| 244779_at | ZDHHC2 | 1,965648824 | 1,728030156 | 0,237618668 | |
| 37796_at | LRCH4 /// SAP25 | 3,183858216 | 2,946258252 | 0,237599964 | |
| 213223_at | RPL28 | 2,666540916 | 2,428994491 | 0,237546426 | |
| 216274_s_at | SEC11A | 5,60687584 | 5,369349853 | 0,237525988 | |
| 229798_s_at | BRI3 | 1,496984567 | 1,259496994 | 0,237487573 | |
| 209660_at | TTR | 0,841135803 | 0,603679647 | 0,237456156 | |
| 211453_s_at | AKT2 | 0,841135803 | 0,603679647 | 0,237456156 | |
| 228881_at | - | 0,841135803 | 0,603679647 | 0,237456156 | |
| 228997_at | TRNAU1AP | 1,774096855 | 1,536652708 | 0,237444147 | |
| 214665_s_at | CHP1 | 5,828864595 | 5,591420757 | 0,237443838 | |
| 1553929_at | ACER1 | -1,026571149 | -1,264005341 | 0,237434192 | |
| 1555305_at | FOXJ2 | -1,026571149 | -1,264005341 | 0,237434192 | |
| 1556092_s_at | HEMK1 | -1,026571149 | -1,264005341 | 0,237434192 | |
| 1562056_at | - | -1,026571149 | -1,264005341 | 0,237434192 | |
| 205627_at | CDA | -1,026571149 | -1,264005341 | 0,237434192 | |
| 207140_at | ALPI | -1,026571149 | -1,264005341 | 0,237434192 | |
| 208102_s_at | PSD | -1,026571149 | -1,264005341 | 0,237434192 | |
| 211739_x_at | CSH1 | -1,026571149 | -1,264005341 | 0,237434192 | |

| | | | | | |
|---|---|---|---|---|---|
| 213544_at | ING2 | -1,026571149 | -1,264005341 | 0,237434192 | |
| 217875_s_at | PMEPA1 | -1,026571149 | -1,264005341 | 0,237434192 | |
| 226417_at | RHOB | -1,026571149 | -1,264005341 | 0,237434192 | |
| 239208_s_at | YBEY | 2,819431021 | 2,582026171 | 0,237404849 | |
| 224963_at | SLC26A2 | 2,320980353 | 2,083632167 | 0,237348186 | |
| 220399_at | LINC00115 | 0,44160593 | 0,204310755 | 0,237295174 | |
| 229071_at | C17orf100 | 0,44160593 | 0,204310755 | 0,237295174 | |
| 1554762_a_at | CLDN22 /// WWC | 1,352503698 | 1,115234684 | 0,237269014 | |
| 226801_s_at | AIDA | 4,961705304 | 4,724458736 | 0,237246568 | |
| 214794_at | PA2G4 | 3,729025365 | 3,491815843 | 0,237209521 | |
| 202690_s_at | SNRPD1 | 6,133171888 | 5,895970644 | 0,237201244 | |
| 224731_at | HMGB1 | 6,54371912 | 6,306604776 | 0,237114344 | |
| 204854_at | LEPREL2 | -0,299034815 | -0,536087151 | 0,237052336 | |
| 206387_at | CDX2 | -0,299034815 | -0,536087151 | 0,237052336 | |
| 215799_at | - | -0,299034815 | -0,536087151 | 0,237052336 | |
| 216299_s_at | XRCC3 | -0,299034815 | -0,536087151 | 0,237052336 | |
| 231637_at | LOC100499194 | -0,299034815 | -0,536087151 | 0,237052336 | |
| 234729_at | PHKG1 | -0,299034815 | -0,536087151 | 0,237052336 | |
| 238739_at | IPMK | -0,299034815 | -0,536087151 | 0,237052336 | |
| 1567107_s_at | TPM4 | 3,902669169 | 3,665633845 | 0,237035324 | |
| 219455_at | C7orf63 | 0,928678725 | 0,691645685 | 0,237033039 | |
| 207545_s_at | NUMB | 1,607231019 | 1,370274469 | 0,23695655 | |
| 213549_at | PDZD8 | 4,814719631 | 4,577894064 | 0,236825567 | |
| 203713_s_at | LLGL2 | -0,112808516 | -0,34958478 | 0,236776264 | |
| 216264_s_at | LAMB2 | -0,112808516 | -0,34958478 | 0,236776264 | |
| 233396_s_at | CSRP2BP | -0,112808516 | -0,34958478 | 0,236776264 | |
| 240359_at | BPIFA3 | -0,112808516 | -0,34958478 | 0,236776264 | |
| 202904_s_at | LSM5 | 2,582657321 | 2,345926937 | 0,236730384 | |
| 218499_at | MST4 | 6,656467298 | 6,419784819 | 0,236682479 | |
| 1552666_a_at | LRRC7 | -2,384869671 | -2,621331989 | 0,236462318 | |
| 1553420_at | SATB2-AS1 | -2,384869671 | -2,621331989 | 0,236462318 | |
| 1555520_at | PTCH1 | -2,384869671 | -2,621331989 | 0,236462318 | |
| 1556221_a_at | - | -2,384869671 | -2,621331989 | 0,236462318 | |
| 1556259_at | - | -2,384869671 | -2,621331989 | 0,236462318 | |
| 1560819_a_at | LOC387895 | -2,384869671 | -2,621331989 | 0,236462318 | |
| 1561003_at | - | -2,384869671 | -2,621331989 | 0,236462318 | |
| 1561462_at | - | -2,384869671 | -2,621331989 | 0,236462318 | |
| 1567078_x_at | CLN6 | -2,384869671 | -2,621331989 | 0,236462318 | |
| 1570596_at | - | -2,384869671 | -2,621331989 | 0,236462318 | |
| 201427_s_at | SEPP1 | -2,384869671 | -2,621331989 | 0,236462318 | |
| 213120_at | UHRF1BP1L | -2,384869671 | -2,621331989 | 0,236462318 | |
| 214029_at | SLC25A42 | -2,384869671 | -2,621331989 | 0,236462318 | |
| 216805_at | - | -2,384869671 | -2,621331989 | 0,236462318 | |
| 220144_s_at | ANKRD5 | -2,384869671 | -2,621331989 | 0,236462318 | |
| 220415_at | FPGT-TNNI3K /// | -2,384869671 | -2,621331989 | 0,236462318 | |
| 237955_at | C5orf58 | -2,384869671 | -2,621331989 | 0,236462318 | |
| 239330_at | DCAF12L2 | -2,384869671 | -2,621331989 | 0,236462318 | |
| 239726_at | ANK3 | -2,384869671 | -2,621331989 | 0,236462318 | |
| 240465_at | C4orf32 | -2,384869671 | -2,621331989 | 0,236462318 | |
| 241311_at | - | -2,384869671 | -2,621331989 | 0,236462318 | |
| 243359_at | - | -2,384869671 | -2,621331989 | 0,236462318 | |
| 211711_s_at | PTEN | 2,433006244 | 2,196649505 | 0,23635674 | |
| 212645_x_at | BRE | 2,462640593 | 2,226407528 | 0,236233065 | |
| 227446_s_at | DHRS4-AS1 | 1,58584542 | 1,349621332 | 0,236224088 | |
| 218311_at | MAP4K3 | 1,390002893 | 1,153866761 | 0,236136132 | |
| 227787_s_at | MED30 | 4,293033569 | 4,056921713 | 0,236111855 | |

| | | | | | |
|---|---|---|---|---|---|
| 228834_at | - | 1,05337236 | 0,817300337 | 0,236072023 | |
| 1562086_at | - | -3,07502285 | -3,311072652 | 0,236049802 | |
| 1567359_at | BDNF-AS | -3,07502285 | -3,311072652 | 0,236049802 | |
| 234755_x_at | - | -3,07502285 | -3,311072652 | 0,236049802 | |
| 235503_at | ASB5 | -3,07502285 | -3,311072652 | 0,236049802 | |
| 237243_at | - | -3,07502285 | -3,311072652 | 0,236049802 | |
| 240717_at | ABCB5 | -3,07502285 | -3,311072652 | 0,236049802 | |
| 244720_at | - | -3,07502285 | -3,311072652 | 0,236049802 | |
| 227766_at | LIG4 | 1,995690482 | 1,759685182 | 0,236005301 | |
| 220990_s_at | MIR21 /// VMP1 | 4,580712136 | 4,344716207 | 0,235995929 | |
| 205492_s_at | DPYSL4 | 0,613492853 | 0,377530647 | 0,235962206 | |
| 230363_s_at | INPP5F | 0,613492853 | 0,377530647 | 0,235962206 | |
| 236106_at | - | 0,613492853 | 0,377530647 | 0,235962206 | |
| 225591_at | FBXO25 | 2,861566439 | 2,625627364 | 0,235939076 | |
| 213838_at | NOL7 | 3,620156261 | 3,384231608 | 0,235924653 | |
| 52940_at | SIGIRR | 1,091809093 | 0,855889476 | 0,235919617 | |
| 1561847_at | NUDT17 | -0,022466681 | -0,258114234 | 0,235647553 | |
| 222075_s_at | OAZ3 | -0,022466681 | -0,258114234 | 0,235647553 | |
| 224096_at | MIR4755 | -0,022466681 | -0,258114234 | 0,235647553 | |
| 212306_at | CLASP2 | 3,890364806 | 3,654731437 | 0,235633368 | |
| 224314_s_at | EGLN1 | 0,715481152 | 0,479907041 | 0,235574111 | |
| 232184_at | ALS2 | 0,715481152 | 0,479907041 | 0,235574111 | |
| 225634_at | ZC3HAV1 | 4,268075451 | 4,032509762 | 0,235565689 | |
| 1569895_at | LOC100133612 | 1,481627361 | 1,246149888 | 0,235477473 | |
| 208351_s_at | MAPK1 | 1,716223609 | 1,480766833 | 0,235456776 | |
| 213225_at | PPM1B | 3,076146581 | 2,840694075 | 0,235452506 | |
| 39966_at | CSPG5 | 2,667389537 | 2,431944693 | 0,235444844 | |
| 224689_at | MANBAL | 2,886560922 | 2,651169275 | 0,235391646 | |
| 208780_x_at | VAPA | 6,552128825 | 6,316881876 | 0,235246949 | |
| 219648_at | MREG | 2,990581579 | 2,755336312 | 0,235245268 | |
| 208262_x_at | MEFV | 0,133056859 | -0,102100538 | 0,235157396 | |
| 230652_at | ARAF | 0,133056859 | -0,102100538 | 0,235157396 | |
| 242092_at | EPB41L2 | 0,133056859 | -0,102100538 | 0,235157396 | |
| 213024_at | TMF1 | 2,001086023 | 1,765933685 | 0,235152338 | |
| 217738_at | NAMPT | 3,107750584 | 2,87289275 | 0,234857834 | |
| 228578_at | RBM45 | 2,846658948 | 2,611817827 | 0,234841121 | |
| 203310_at | STXBP3 | 3,91129185 | 3,67645448 | 0,23483737 | |
| 244650_at | FAM105A | 0,457392764 | 0,222599095 | 0,23479367 | |
| 225475_at | MIER1 | 2,890927111 | 2,656223838 | 0,234703272 | |
| 205552_s_at | OAS1 | 0,859074518 | 0,624460759 | 0,234613759 | |
| 213035_at | ANKRD28 | 2,837639949 | 2,603119255 | 0,234520694 | |
| 224057_s_at | THAP4 | 2,837639949 | 2,603119255 | 0,234520694 | |
| 212698_s_at | 40422 | 3,225364068 | 2,990953566 | 0,234410502 | |
| 233568_x_at | CWF19L1 | 3,234554895 | 3,000258961 | 0,234295934 | |
| 205271_s_at | CDK20 | 1,139070151 | 0,904843258 | 0,234226892 | |
| 1558733_at | ZBTB38 | 0,209623097 | -0,024572586 | 0,234195684 | |
| 229895_s_at | NCK1 | 0,209623097 | -0,024572586 | 0,234195684 | |
| 209113_s_at | HMG20B | 1,841816345 | 1,60795124 | 0,233865105 | |
| 204482_at | CLDN5 | -0,986356909 | -1,220210588 | 0,233853679 | |
| 218874_s_at | ATAT1 | -0,986356909 | -1,220210588 | 0,233853679 | |
| 220663_at | IL1RAPL1 | -0,986356909 | -1,220210588 | 0,233853679 | |
| 231191_at | - | -0,986356909 | -1,220210588 | 0,233853679 | |
| 238921_at | LOC644794 | -0,986356909 | -1,220210588 | 0,233853679 | |
| 240916_x_at | - | -0,986356909 | -1,220210588 | 0,233853679 | |
| 1555775_a_at | ZAR1 | -0,496991414 | -0,730778808 | 0,233787394 | |
| 212736_at | C16orf45 | -0,496991414 | -0,730778808 | 0,233787394 | |

| | | | | | |
|---|---|---|---|---|---|
| 222215_at | SLC38A7 | -0,496991414 | -0,730778808 | 0,233787394 | |
| 232718_at | LINC00589 | -0,496991414 | -0,730778808 | 0,233787394 | |
| 238867_at | TMEM182 | -0,496991414 | -0,730778808 | 0,233787394 | |
| 239107_at | ZNF280D | -0,496991414 | -0,730778808 | 0,233787394 | |
| 239651_at | ANAPC5 | -0,496991414 | -0,730778808 | 0,233787394 | |
| 220079_s_at | USP48 | 4,229522279 | 3,995764148 | 0,23375813 | |
| 205897_at | NFATC4 | 0,627515113 | 0,393761504 | 0,233753609 | |
| 1556553_at | - | -1,308181863 | -1,541908042 | 0,233726179 | |
| 1557780_at | - | -1,308181863 | -1,541908042 | 0,233726179 | |
| 1559375_s_at | - | -1,308181863 | -1,541908042 | 0,233726179 | |
| 210632_s_at | SGCA | -1,308181863 | -1,541908042 | 0,233726179 | |
| 212912_at | RPS6KA2 | -1,308181863 | -1,541908042 | 0,233726179 | |
| 215517_at | PYGO1 | -1,308181863 | -1,541908042 | 0,233726179 | |
| 216358_at | - | -1,308181863 | -1,541908042 | 0,233726179 | |
| 219843_at | IPP | -1,308181863 | -1,541908042 | 0,233726179 | |
| 230269_at | - | -1,308181863 | -1,541908042 | 0,233726179 | |
| 231241_at | - | -1,308181863 | -1,541908042 | 0,233726179 | |
| 231814_at | MUC12 | -1,308181863 | -1,541908042 | 0,233726179 | |
| 236334_at | - | -1,308181863 | -1,541908042 | 0,233726179 | |
| 241658_at | - | -1,308181863 | -1,541908042 | 0,233726179 | |
| 242973_at | CACNA1C | -1,308181863 | -1,541908042 | 0,233726179 | |
| 223320_s_at | ABCB10 | 2,970093718 | 2,7363693 | 0,233724418 | |
| 229828_at | CDC73 | 1,247462054 | 1,013870809 | 0,233591245 | |
| 201861_s_at | LRRFIP1 | 3,032022986 | 2,798646381 | 0,233376605 | |
| 206028_s_at | MERTK | 2,232003119 | 1,998633167 | 0,233369952 | |
| 206037_at | CCBL1 | 0,822971223 | 0,589657387 | 0,233313836 | |
| 203119_at | CCDC86 | 3,834791978 | 3,601570866 | 0,233221112 | |
| 202344_at | HSF1 | 1,823660386 | 1,590454215 | 0,233206171 | |
| 219957_at | RUFY2 | -0,089689931 | -0,322870281 | 0,23318035 | |
| 223468_s_at | RGMA | -0,089689931 | -0,322870281 | 0,23318035 | |
| 224010_at | ANAPC11 | -0,089689931 | -0,322870281 | 0,23318035 | |
| 229102_at | HTATIP2 | -0,089689931 | -0,322870281 | 0,23318035 | |
| 44673_at | SIGLEC1 | 0,181384989 | -0,051772821 | 0,233157809 | |
| 202204_s_at | AMFR | 1,631786706 | 1,398700978 | 0,233085728 | |
| 211863_x_at | HFE | 0,072842263 | -0,160136748 | 0,232979011 | |
| 213723_s_at | IDUA | 0,072842263 | -0,160136748 | 0,232979011 | |
| 215467_x_at | LOC647070 | 0,072842263 | -0,160136748 | 0,232979011 | |
| 221754_s_at | CORO1B | 0,072842263 | -0,160136748 | 0,232979011 | |
| 226234_at | GDF11 | 0,072842263 | -0,160136748 | 0,232979011 | |
| 224747_at | UBE2Q2 | 3,858765357 | 3,625821706 | 0,232943651 | |
| 1554504_at | NAALAD2 | -3,114791413 | -3,347710726 | 0,232919313 | |
| 201299_s_at | MOB1A | 1,732533761 | 1,499637023 | 0,232896739 | |
| 1560047_s_at | - | -0,622910612 | -0,855751026 | 0,232840414 | |
| 1561386_at | - | -0,622910612 | -0,855751026 | 0,232840414 | |
| 1566001_at | - | -0,622910612 | -0,855751026 | 0,232840414 | |
| 206637_at | P2RY14 | -0,622910612 | -0,855751026 | 0,232840414 | |
| 209839_at | DNM3 | -0,622910612 | -0,855751026 | 0,232840414 | |
| 211644_x_at | IGK@ /// IGKC | -0,622910612 | -0,855751026 | 0,232840414 | |
| 221446_at | ADAM30 | -0,622910612 | -0,855751026 | 0,232840414 | |
| 233489_at | TMEM43 | -0,622910612 | -0,855751026 | 0,232840414 | |
| 237759_at | CD48 | -0,622910612 | -0,855751026 | 0,232840414 | |
| 239949_at | THNSL2 | -0,622910612 | -0,855751026 | 0,232840414 | |
| 240813_at | - | -0,622910612 | -0,855751026 | 0,232840414 | |
| 242674_at | EIF4E | -0,622910612 | -0,855751026 | 0,232840414 | |
| 243760_at | MIPEPP3 | -0,622910612 | -0,855751026 | 0,232840414 | |
| 221654_s_at | USP3 | 3,294008537 | 3,061230246 | 0,232778291 | |

| | | | | | |
|---|---|---|---|---|---|
| 220753_s_at | CRYL1 | 0,87091093 | 0,638150376 | 0,232760554 | |
| 227811_at | FGD3 | 0,87091093 | 0,638150376 | 0,232760554 | |
| 207168_s_at | H2AFY | 5,465225326 | 5,232507246 | 0,23271808 | |
| 201573_s_at | ETF1 | 4,984696817 | 4,752066034 | 0,232630782 | |
| 232645_at | LOC153684 | 0,95671774 | 0,724100169 | 0,232617571 | |
| 1552564_at | NUDT9P1 | -2,866296344 | -3,098858316 | 0,232561972 | |
| 1552735_at | PCDHGA4 | -2,866296344 | -3,098858316 | 0,232561972 | |
| 1557431_at | - | -2,866296344 | -3,098858316 | 0,232561972 | |
| 1557703_at | 07.03.15 | -2,866296344 | -3,098858316 | 0,232561972 | |
| 1568366_at | - | -2,866296344 | -3,098858316 | 0,232561972 | |
| 229802_at | WISP1 | -2,866296344 | -3,098858316 | 0,232561972 | |
| 235591_at | SSTR1 | -2,866296344 | -3,098858316 | 0,232561972 | |
| 236037_at | KIAA1244 | -2,866296344 | -3,098858316 | 0,232561972 | |
| 239150_at | SNTN | -2,866296344 | -3,098858316 | 0,232561972 | |
| 218663_at | NCAPG | 3,18563636 | 2,953119918 | 0,232516442 | |
| 236265_at | SP4 | 2,782308844 | 2,549796788 | 0,232512056 | |
| 212944_at | SLC5A3 | 4,635514003 | 4,403009191 | 0,232504812 | |
| 212233_at | MAP1B | 3,008105382 | 2,775606361 | 0,232499022 | |
| 238828_at | KIAA1919 | 1,037706275 | 0,805216064 | 0,232490211 | |
| 240058_at | - | 1,037706275 | 0,805216064 | 0,232490211 | |
| 223191_at | COX16 | 5,492057836 | 5,25960603 | 0,232451807 | |
| 228567_at | MIR4720 | 1,556829052 | 1,324440845 | 0,232388206 | |
| 224837_at | FOXP1 | 2,675004741 | 2,442710777 | 0,232293964 | |
| 212139_at | GCN1L1 | 3,933331467 | 3,701119714 | 0,232211752 | |
| 209328_x_at | HIGD2A | 1,874520207 | 1,642321262 | 0,232198944 | |
| 219361_s_at | AEN | 1,874520207 | 1,642321262 | 0,232198944 | |
| 204215_at | C7orf23 | 0,152582607 | -0,079522948 | 0,232105555 | |
| 207645_s_at | CHD1L | 0,152582607 | -0,079522948 | 0,232105555 | |
| 234062_at | - | 0,152582607 | -0,079522948 | 0,232105555 | |
| 239941_at | - | 0,152582607 | -0,079522948 | 0,232105555 | |
| 228642_at | HOTAIRM1 | 1,079110292 | 0,847075464 | 0,232034828 | |
| 200753_x_at | SRSF2 | 4,986057923 | 4,754031948 | 0,232025974 | |
| 207839_s_at | TMEM8B | 0,291166226 | 0,059218869 | 0,231947357 | |
| 210984_x_at | EGFR | 0,291166226 | 0,059218869 | 0,231947357 | |
| 229614_at | ZNF320 | 1,446464021 | 1,214517879 | 0,231946142 | |
| 220773_s_at | GPHN | 1,940595304 | 1,708698295 | 0,231897009 | |
| 223308_s_at | WDR5 | 3,098971025 | 2,867091644 | 0,231879381 | |
| 229899_s_at | ZNFX1-AS1 | 2,190100188 | 1,958277216 | 0,231822971 | |
| 1563111_a_at | PIGX | 2,225103271 | 1,993317152 | 0,231786119 | |
| 236027_at | SFR1 | 2,225103271 | 1,993317152 | 0,231786119 | |
| 224799_at | NDFIP2 | 3,512821151 | 3,281121096 | 0,231700056 | |
| 225334_at | C10orf32 | 3,512821151 | 3,281121096 | 0,231700056 | |
| 218370_s_at | S100PBP | 2,491678455 | 2,259997914 | 0,231680542 | |
| 223001_at | OSTC | 5,932480812 | 5,700850431 | 0,231630381 | |
| 209676_at | TFPI | 2,822481847 | 2,590852465 | 0,231629382 | |
| 218566_s_at | CHORDC1 | 4,585658616 | 4,354078399 | 0,231580217 | |
| 201302_at | ANXA4 | 2,082235068 | 1,85068474 | 0,231550328 | |
| 202601_s_at | HTATSF1 | 1,191926657 | 0,960386719 | 0,231539937 | |
| 213176_s_at | LTBP4 | 1,191926657 | 0,960386719 | 0,231539937 | |
| 200778_s_at | 37500 | 5,14499041 | 4,913450933 | 0,231539476 | |
| 242565_x_at | YBEY | 2,506929603 | 2,275409734 | 0,231519869 | |
| 1555976_s_at | MYL12A | 1,69307511 | 1,461646549 | 0,231428561 | |
| 223473_at | MPV17L2 | 3,025418883 | 2,794067719 | 0,231351164 | |
| 1554945_x_at | VIL1 | -2,504600601 | -2,735862426 | 0,231261824 | |
| 1555233_at | RHOJ | -2,504600601 | -2,735862426 | 0,231261824 | |
| 1558757_at | - | -2,504600601 | -2,735862426 | 0,231261824 | |

| | | | | | |
|---|---|---|---|---|---|
| 1559322_at | LOC727916 | -2,504600601 | -2,735862426 | 0,231261824 | |
| 1561165_a_at | DEFB108B | -2,504600601 | -2,735862426 | 0,231261824 | |
| 1565877_at | - | -2,504600601 | -2,735862426 | 0,231261824 | |
| 1566482_at | - | -2,504600601 | -2,735862426 | 0,231261824 | |
| 210755_at | HGF | -2,504600601 | -2,735862426 | 0,231261824 | |
| 213355_at | ST3GAL6 | -2,504600601 | -2,735862426 | 0,231261824 | |
| 215300_s_at | FMO5 | -2,504600601 | -2,735862426 | 0,231261824 | |
| 216108_at | - | -2,504600601 | -2,735862426 | 0,231261824 | |
| 221174_at | - | -2,504600601 | -2,735862426 | 0,231261824 | |
| 234357_at | - | -2,504600601 | -2,735862426 | 0,231261824 | |
| 234535_at | - | -2,504600601 | -2,735862426 | 0,231261824 | |
| 236215_at | - | -2,504600601 | -2,735862426 | 0,231261824 | |
| 237930_at | - | -2,504600601 | -2,735862426 | 0,231261824 | |
| 240316_at | C9orf57 | -2,504600601 | -2,735862426 | 0,231261824 | |
| 215579_at | APOBEC3G | 0,359971644 | 0,128747141 | 0,231224503 | |
| 222019_at | PFDN6 | 0,359971644 | 0,128747141 | 0,231224503 | |
| 239147_at | ARSK | 0,359971644 | 0,128747141 | 0,231224503 | |
| 226386_at | MALSU1 | 4,447740857 | 4,216563145 | 0,231177712 | |
| 226347_at | FUT11 | 2,802535172 | 2,571362843 | 0,231172329 | |
| 222410_s_at | SNX6 | 4,710299868 | 4,479143747 | 0,231156122 | |
| 204658_at | TRA2A | 4,288076367 | 4,056921713 | 0,231154654 | |
| 218723_s_at | RGCC | 0,480753764 | 0,249604176 | 0,231149588 | |
| 207927_at | HTR7 | -0,259832361 | -0,490927335 | 0,231094974 | |
| 213108_at | CAMK2A | -0,259832361 | -0,490927335 | 0,231094974 | |
| 220944_at | PGLYRP4 | -0,259832361 | -0,490927335 | 0,231094974 | |
| 233550_s_at | SLC4A11 | -0,259832361 | -0,490927335 | 0,231094974 | |
| 241922_at | LMO4 | -0,259832361 | -0,490927335 | 0,231094974 | |
| 235059_at | RAB12 | 1,422536444 | 1,191491281 | 0,231045163 | |
| 218823_s_at | KCTD9 | 3,53158484 | 3,300620584 | 0,230964256 | |
| 203624_at | AKAP17A | 3,843828743 | 3,6128809 | 0,230947844 | |
| 231825_x_at | ATF7IP | 3,231115193 | 3,000258961 | 0,230856232 | |
| 201432_at | CAT | 5,340953221 | 5,110146091 | 0,230807129 | |
| 212331_at | RBL2 | 3,188003814 | 2,957221307 | 0,230782507 | |
| 203473_at | SLCO2B1 | 1,567778694 | 1,337086025 | 0,230692669 | |
| 225661_at | IFNAR1 | 3,266269388 | 3,035599287 | 0,2306701 | |
| 217673_x_at | GNAS | 4,489366635 | 4,258712848 | 0,230653788 | |
| 203986_at | FAM47E /// FAM4 | 0,541261317 | 0,310717132 | 0,230544185 | |
| 220292_at | ZNF434 | 0,541261317 | 0,310717132 | 0,230544185 | |
| 241416_at | - | 0,541261317 | 0,310717132 | 0,230544185 | |
| 1557480_a_at | PPP1R27 | 0,747935635 | 0,51742585 | 0,230509785 | |
| 212390_at | LOC728802 /// PI | 0,747935635 | 0,51742585 | 0,230509785 | |
| 225915_at | CAB39L | 0,747935635 | 0,51742585 | 0,230509785 | |
| 226184_at | FMNL2 | 1,163336153 | 0,932882274 | 0,230453879 | |
| 217403_s_at | ZNF227 | 1,699726954 | 1,469325098 | 0,230401856 | |
| 235579_at | SCAF11 | 2,456762221 | 2,226407528 | 0,230354693 | |
| 225841_at | HENMT1 | 3,731461974 | 3,501209806 | 0,230252168 | |
| 202239_at | PARP4 | 2,876321464 | 2,646096941 | 0,230224523 | |
| 226585_at | NEIL2 | 1,774096855 | 1,543943227 | 0,230153628 | |
| 200978_at | MDH1 | 5,487980577 | 5,257944504 | 0,230036073 | |
| 211255_x_at | DEDD | 0,798390451 | 0,568365095 | 0,230025356 | |
| 221586_s_at | E2F5 | 3,488057722 | 3,258033502 | 0,23002422 | |
| 233632_s_at | XRN1 | 2,288253583 | 2,058399602 | 0,229853981 | |
| 1559722_at | - | -2,908843709 | -3,138626879 | 0,22978317 | |
| 1563101_at | ARNTL2 | -2,908843709 | -3,138626879 | 0,22978317 | |
| 1569411_at | TMEM67 | -2,908843709 | -3,138626879 | 0,22978317 | |
| 210941_at | PCDH7 | -2,908843709 | -3,138626879 | 0,22978317 | |

| | | | | | |
|---|---|---|---|---|---|
| 214823_at | ZNF204P | -2,908843709 | -3,138626879 | 0,22978317 | |
| 219948_x_at | UGT2A3 | -2,908843709 | -3,138626879 | 0,22978317 | |
| 229598_at | COBLL1 | -2,908843709 | -3,138626879 | 0,22978317 | |
| 231063_at | - | -2,908843709 | -3,138626879 | 0,22978317 | |
| 234296_s_at | TEX11 | -2,908843709 | -3,138626879 | 0,22978317 | |
| 237602_at | - | -2,908843709 | -3,138626879 | 0,22978317 | |
| 237831_x_at | MMAA | -2,908843709 | -3,138626879 | 0,22978317 | |
| 241871_at | CAMK4 | -2,908843709 | -3,138626879 | 0,22978317 | |
| 218304_s_at | OSBPL11 | 2,81177561 | 2,582026171 | 0,229749439 | |
| 209272_at | NAB1 | 3,431234921 | 3,201528602 | 0,229706319 | |
| 222252_x_at | UBQLN4 | 1,094335495 | 0,864649967 | 0,229685528 | |
| 220250_at | ZNF286A | 0,934330293 | 0,704715219 | 0,229615074 | |
| 239069_s_at | - | 4,046594062 | 3,816987307 | 0,229606754 | |
| 203978_at | NUBP1 | 2,693452197 | 2,464004877 | 0,22944732 | |
| 228297_at | - | 2,987866617 | 2,758473378 | 0,229393239 | |
| 220011_at | C1orf135 | 2,779171778 | 2,549796788 | 0,22937499 | |
| 214141_x_at | SRSF7 | 6,247747786 | 6,01846 | 0,229287786 | |
| 231338_at | C15orf55 | 0,30867765 | 0,079428135 | 0,229249515 | |
| 209076_s_at | WDR45L | 4,19815525 | 3,968932193 | 0,229223057 | |
| 201096_s_at | ARF4 | 5,647869371 | 5,418765728 | 0,229103643 | |
| 202097_at | NUP153 | 5,483409962 | 5,254337955 | 0,229072007 | |
| 1554663_a_at | NUMA1 | -2,551294263 | -2,780313924 | 0,229019662 | |
| 1560012_at | - | -2,551294263 | -2,780313924 | 0,229019662 | |
| 1560643_x_at | - | -2,551294263 | -2,780313924 | 0,229019662 | |
| 1562901_at | LOC400456 | -2,551294263 | -2,780313924 | 0,229019662 | |
| 1565900_at | METTL15 | -2,551294263 | -2,780313924 | 0,229019662 | |
| 1566295_at | - | -2,551294263 | -2,780313924 | 0,229019662 | |
| 1566955_at | - | -2,551294263 | -2,780313924 | 0,229019662 | |
| 1569006_at | LOC284379 | -2,551294263 | -2,780313924 | 0,229019662 | |
| 1569570_at | AGBL4 | -2,551294263 | -2,780313924 | 0,229019662 | |
| 216369_at | - | -2,551294263 | -2,780313924 | 0,229019662 | |
| 233231_at | - | -2,551294263 | -2,780313924 | 0,229019662 | |
| 234606_at | - | -2,551294263 | -2,780313924 | 0,229019662 | |
| 234662_at | RAD21L1 | -2,551294263 | -2,780313924 | 0,229019662 | |
| 235185_s_at | LOC388692 | -2,551294263 | -2,780313924 | 0,229019662 | |
| 237169_at | TNC | -2,551294263 | -2,780313924 | 0,229019662 | |
| 237218_at | - | -2,551294263 | -2,780313924 | 0,229019662 | |
| 238127_at | GAS6-AS1 | -2,551294263 | -2,780313924 | 0,229019662 | |
| 238552_at | - | -2,551294263 | -2,780313924 | 0,229019662 | |
| 240152_at | - | -2,551294263 | -2,780313924 | 0,229019662 | |
| 240739_at | - | -2,551294263 | -2,780313924 | 0,229019662 | |
| 241356_at | - | -2,551294263 | -2,780313924 | 0,229019662 | |
| 242018_at | - | -2,551294263 | -2,780313924 | 0,229019662 | |
| 244098_at | - | -2,551294263 | -2,780313924 | 0,229019662 | |
| 202850_at | ABCD3 | 4,010933848 | 3,781960839 | 0,228973009 | |
| 210361_s_at | ELF2 | 1,46610492 | 1,23718272 | 0,228922201 | |
| 227386_s_at | TMEM200B | 0,606430281 | 0,377530647 | 0,228899635 | |
| 227464_at | ACSF3 | 0,606430281 | 0,377530647 | 0,228899635 | |
| 244115_at | FAM126A | 0,606430281 | 0,377530647 | 0,228899635 | |
| 227833_s_at | MBD6 | 1,406361373 | 1,177496821 | 0,228864552 | |
| 235507_at | PCMTD1 | 0,496120222 | 0,26733076 | 0,228789462 | |
| 200687_s_at | SF3B3 | 4,324039713 | 4,09526554 | 0,228774173 | |
| 210508_s_at | KCNQ2 | -0,59053611 | -0,819238336 | 0,228702226 | |
| 216856_s_at | - | -0,59053611 | -0,819238336 | 0,228702226 | |
| 222074_at | UROD | -0,59053611 | -0,819238336 | 0,228702226 | |
| 224263_x_at | ZAN | -0,59053611 | -0,819238336 | 0,228702226 | |

| | | | | | |
|---|---|---|---|---|---|
| 225233_at | MSI2 | -0,59053611 | -0,819238336 | 0,228702226 | |
| 238285_at | SOX5 | -0,59053611 | -0,819238336 | 0,228702226 | |
| 241636_x_at | - | -0,59053611 | -0,819238336 | 0,228702226 | |
| 243843_at | N4BP2L1 | -0,59053611 | -0,819238336 | 0,228702226 | |
| 244843_x_at | - | -0,59053611 | -0,819238336 | 0,228702226 | |
| 223467_at | RASD1 | 0,853119696 | 0,624460759 | 0,228658937 | |
| 220950_s_at | KANSL3 | 0,760715727 | 0,532164371 | 0,228551356 | |
| 1555470_a_at | PPM1F | 1,063722698 | 0,835239052 | 0,228483646 | |
| 1556543_at | - | 0,661985843 | 0,433557298 | 0,228428544 | |
| 218776_s_at | TMEM62 | 0,661985843 | 0,433557298 | 0,228428544 | |
| 236275_at | KRBA1 | 2,194816625 | 1,966439221 | 0,228377404 | |
| 201160_s_at | CSDA | 5,657511266 | 5,429138169 | 0,228373097 | |
| 203323_at | CAV2 | 4,870319181 | 4,642144573 | 0,228174608 | |
| 202787_s_at | MAPKAPK3 | 1,283332461 | 1,055274826 | 0,228057635 | |
| 204665_at | SIKE1 | -0,926850548 | -1,154897679 | 0,22804713 | |
| 215214_at | IGLC1 | -0,926850548 | -1,154897679 | 0,22804713 | |
| 215608_at | - | -0,926850548 | -1,154897679 | 0,22804713 | |
| 216429_at | - | -0,926850548 | -1,154897679 | 0,22804713 | |
| 228235_at | MGC16121 | -0,926850548 | -1,154897679 | 0,22804713 | |
| 231448_at | ADAD1 | -0,926850548 | -1,154897679 | 0,22804713 | |
| 231924_at | LOC100506305 | -0,926850548 | -1,154897679 | 0,22804713 | |
| 234737_at | NT5DC3 | -0,926850548 | -1,154897679 | 0,22804713 | |
| 235752_at | - | -0,926850548 | -1,154897679 | 0,22804713 | |
| 238926_at | LOC100505974 | -0,926850548 | -1,154897679 | 0,22804713 | |
| 239296_at | - | -0,926850548 | -1,154897679 | 0,22804713 | |
| 242907_at | GBP2 | -0,926850548 | -1,154897679 | 0,22804713 | |
| 204671_s_at | ANKRD6 | 1,442503549 | 1,214517879 | 0,22798567 | |
| 205074_at | SLC22A5 | -0,055687482 | -0,283667828 | 0,227980346 | |
| 211198_s_at | ICOSLG | -0,055687482 | -0,283667828 | 0,227980346 | |
| 212570_at | ENDOD1 | -0,055687482 | -0,283667828 | 0,227980346 | |
| 237093_at | - | -0,055687482 | -0,283667828 | 0,227980346 | |
| 244353_s_at | SLC2A12 | -0,055687482 | -0,283667828 | 0,227980346 | |
| 209746_s_at | COQ7 | 1,58584542 | 1,3579181 | 0,22792732 | |
| 234807_x_at | - | 1,58584542 | 1,3579181 | 0,22792732 | |
| 204697_s_at | CHGA | 1,381753566 | 1,153866761 | 0,227886805 | |
| 220223_at | ATAD5 | 1,381753566 | 1,153866761 | 0,227886805 | |
| 223053_x_at | SSU72 | 3,722507547 | 3,49464046 | 0,227867087 | |
| 204643_s_at | ENOX2 | 1,318332599 | 1,090553537 | 0,227779062 | |
| 242364_x_at | LOC100131096 | 1,318332599 | 1,090553537 | 0,227779062 | |
| 206793_at | PNMT | 1,789769151 | 1,562009954 | 0,227759197 | |
| 1555090_x_at | TMEM182 | 0,181384709 | -0,046302147 | 0,227686856 | |
| 207394_at | ZNF137P | 0,181384709 | -0,046302147 | 0,227686856 | |
| 227636_at | THAP5 | 2,127340679 | 1,899812471 | 0,227528208 | |
| 210229_s_at | CSF2 | 0,384950573 | 0,157549243 | 0,22740133 | |
| 242490_at | LOC100653004 | 0,384950573 | 0,157549243 | 0,22740133 | |
| 216307_at | DGKB | 0,03125738 | -0,19610998 | 0,22736736 | |
| 229439_s_at | RBM47 | 0,03125738 | -0,19610998 | 0,22736736 | |
| 237773_at | - | 0,03125738 | -0,19610998 | 0,22736736 | |
| 1553367_a_at | COX6B2 | -0,234278768 | -0,461594427 | 0,227315659 | |
| 209951_s_at | MAP2K7 | -0,234278768 | -0,461594427 | 0,227315659 | |
| 223344_s_at | MS4A7 | -0,234278768 | -0,461594427 | 0,227315659 | |
| 225286_at | ARSD | -0,234278768 | -0,461594427 | 0,227315659 | |
| 226974_at | NEDD4L | -0,234278768 | -0,461594427 | 0,227315659 | |
| 234388_at | - | -0,234278768 | -0,461594427 | 0,227315659 | |
| 1554757_a_at | INPP5A | 0,255492549 | 0,028364014 | 0,227128535 | |
| 208040_s_at | MYBPC3 | 0,255492549 | 0,028364014 | 0,227128535 | |

| | | | | | |
|---|---|---|---|---|---|
| 219082_at | AMDHD2 /// CEM | 0,255492549 | 0,028364014 | 0,227128535 | |
| 221282_x_at | RUNX2 | 0,255492549 | 0,028364014 | 0,227128535 | |
| 224179_s_at | MIOX | 0,255492549 | 0,028364014 | 0,227128535 | |
| 227430_at | ZC3H10 | 0,255492549 | 0,028364014 | 0,227128535 | |
| 229579_s_at | DISP2 | 0,255492549 | 0,028364014 | 0,227128535 | |
| 238716_at | LOC100506990 | 0,255492549 | 0,028364014 | 0,227128535 | |
| 209146_at | MSMO1 | 4,167291414 | 3,940211012 | 0,227080401 | |
| 222369_at | NAA40 | 2,908260915 | 2,681234383 | 0,227026532 | |
| 1556494_at | - | -1,196375121 | -1,423348341 | 0,22697322 | |
| 1568999_at | PSG4 | -1,196375121 | -1,423348341 | 0,22697322 | |
| 205624_at | CPA3 | -1,196375121 | -1,423348341 | 0,22697322 | |
| 208000_at | GML | -1,196375121 | -1,423348341 | 0,22697322 | |
| 209986_at | PAH | -1,196375121 | -1,423348341 | 0,22697322 | |
| 211029_x_at | FGF18 | -1,196375121 | -1,423348341 | 0,22697322 | |
| 211129_x_at | EDA | -1,196375121 | -1,423348341 | 0,22697322 | |
| 224552_s_at | C11orf20 /// KCN | -1,196375121 | -1,423348341 | 0,22697322 | |
| 227642_at | TFCP2L1 | -1,196375121 | -1,423348341 | 0,22697322 | |
| 230383_x_at | - | -1,196375121 | -1,423348341 | 0,22697322 | |
| 230601_s_at | LRRC46 | -1,196375121 | -1,423348341 | 0,22697322 | |
| 231656_x_at | OSBPL10 | -1,196375121 | -1,423348341 | 0,22697322 | |
| 231704_at | CYP3A4 | -1,196375121 | -1,423348341 | 0,22697322 | |
| 232313_at | TMEM132C | -1,196375121 | -1,423348341 | 0,22697322 | |
| 233353_at | FER1L5 | -1,196375121 | -1,423348341 | 0,22697322 | |
| 235543_at | - | -1,196375121 | -1,423348341 | 0,22697322 | |
| 236985_at | - | -1,196375121 | -1,423348341 | 0,22697322 | |
| 237283_at | - | -1,196375121 | -1,423348341 | 0,22697322 | |
| 208843_s_at | GORASP2 | 4,510092468 | 4,283151707 | 0,226940761 | |
| 204012_s_at | LCMT2 | 2,864529541 | 2,637603218 | 0,226926324 | |
| 229513_at | STRBP | 1,073999308 | 0,847075464 | 0,226923844 | |
| 241721_at | - | 1,073999308 | 0,847075464 | 0,226923844 | |
| 232652_x_at | SCAND1 | 3,818840142 | 3,591931093 | 0,226909048 | |
| 227414_at | RHBDD1 | 1,187200746 | 0,960386719 | 0,226814027 | |
| 238496_at | WHSC1L1 | 1,187200746 | 0,960386719 | 0,226814027 | |
| 33579_i_at | GALR3 | 1,187200746 | 0,960386719 | 0,226814027 | |
| 209577_at | PCYT2 | 0,995078803 | 0,768343791 | 0,226735012 | |
| 220335_x_at | CES3 | 1,292162421 | 1,065442793 | 0,226719628 | |
| 218219_s_at | LANCL2 | 2,310153708 | 2,083632167 | 0,226521541 | |
| 222815_at | RLIM | 2,225103271 | 1,998633167 | 0,226470104 | |
| 238813_at | APEX2 | 1,912238072 | 1,685812099 | 0,226425972 | |
| 221158_at | GCFC1 | 2,452829957 | 2,226407528 | 0,226422429 | |
| 219706_at | AP5S1 | 1,596577845 | 1,370274469 | 0,226303376 | |
| 204791_at | NR2C1 | 2,392522438 | 2,166264721 | 0,226257716 | |
| 201179_s_at | GNAI3 | 3,834791978 | 3,608541373 | 0,226250605 | |
| 228515_at | LOC90784 /// PO | 1,751865623 | 1,525647381 | 0,226218242 | |
| 232436_at | ZNF274 | 1,542099044 | 1,315948728 | 0,226150316 | |
| 1569003_at | VMP1 | 1,394109935 | 1,16809119 | 0,226018745 | |
| 206593_s_at | MED22 | 1,394109935 | 1,16809119 | 0,226018745 | |
| 227967_at | TUBGCP5 | 1,394109935 | 1,16809119 | 0,226018745 | |
| 218319_at | PELI1 | 1,485482016 | 1,259496994 | 0,225985021 | |
| 225269_s_at | RBMS1 | 2,456762221 | 2,230931691 | 0,22583053 | |
| 221335_x_at | SMG9 | 2,001086023 | 1,775255987 | 0,225830036 | |
| 227990_at | SLU7 | 2,001086023 | 1,775255987 | 0,225830036 | |
| 201501_s_at | GRSF1 | 3,80887607 | 3,583111417 | 0,225764653 | |
| 204141_at | TUBB2A | 6,001825317 | 5,776106355 | 0,225718962 | |
| 214867_at | LOC100507331 | 0,779676066 | 0,553993624 | 0,225682441 | |
| 236972_at | TRIM63 | 0,779676066 | 0,553993624 | 0,225682441 | |

| | | | | | |
|---|---|---|---|---|---|
| 203048_s_at | TTC37 | 2,348755185 | 2,123108223 | 0,225646962 | |
| 202531_at | IRF1 | 1,706348269 | 1,480766833 | 0,225581436 | |
| 218674_at | C5orf44 | 1,706348269 | 1,480766833 | 0,225581436 | |
| 228171_s_at | PLEKHG4 | 2,266015882 | 2,040470494 | 0,225545388 | |
| 221938_x_at | MED16 | 1,575032557 | 1,349621332 | 0,225411225 | |
| 228967_at | EIF1 | 0,682279745 | 0,456918297 | 0,225361448 | |
| 237919_at | RFFL | 0,334552598 | 0,109221392 | 0,225331206 | |
| 238491_at | LOC100506161 | 0,334552598 | 0,109221392 | 0,225331206 | |
| 242691_at | - | 0,334552598 | 0,109221392 | 0,225331206 | |
| 214005_at | GGCX | 2,197169074 | 1,971855016 | 0,225314058 | |
| 227255_at | PDIK1L | 2,084778276 | 1,859476797 | 0,22530148 | |
| 208128_x_at | KIF25 | 0,123193849 | -0,102100538 | 0,225294387 | |
| 220618_s_at | ZCWPW1 | 0,123193849 | -0,102100538 | 0,225294387 | |
| 229998_x_at | FAM176B | 0,123193849 | -0,102100538 | 0,225294387 | |
| 230065_at | - | 0,123193849 | -0,102100538 | 0,225294387 | |
| 233626_at | - | 0,123193849 | -0,102100538 | 0,225294387 | |
| 234991_at | ZXDC | 0,123193849 | -0,102100538 | 0,225294387 | |
| 1552325_at | CCDC11 | -3,212234735 | -3,437493426 | 0,225258691 | |
| 1553320_s_at | CDC14C | -3,212234735 | -3,437493426 | 0,225258691 | |
| 1568867_x_at | - | -3,212234735 | -3,437493426 | 0,225258691 | |
| 226483_at | TMEM68 | 1,603688695 | 1,378453643 | 0,225235051 | |
| 200815_s_at | PAFAH1B1 | 3,310399576 | 3,08516499 | 0,225234586 | |
| 1560116_a_at | NEDD1 | 3,095191939 | 2,869995113 | 0,225196826 | |
| 223389_s_at | ZNF581 | 3,255587071 | 3,030417996 | 0,225169075 | |
| 1552822_at | TMX3 | -1,812418401 | -2,037551727 | 0,225133325 | |
| 1557751_at | - | -1,812418401 | -2,037551727 | 0,225133325 | |
| 1558262_at | SPTY2D1-AS1 | -1,812418401 | -2,037551727 | 0,225133325 | |
| 1559057_at | - | -1,812418401 | -2,037551727 | 0,225133325 | |
| 1560432_at | CLRN1-AS1 | -1,812418401 | -2,037551727 | 0,225133325 | |
| 1568832_a_at | - | -1,812418401 | -2,037551727 | 0,225133325 | |
| 207649_at | KRT37 | -1,812418401 | -2,037551727 | 0,225133325 | |
| 208295_x_at | CSHL1 | -1,812418401 | -2,037551727 | 0,225133325 | |
| 211647_x_at | IGHG1 /// IGHM | -1,812418401 | -2,037551727 | 0,225133325 | |
| 217098_s_at | ZSCAN12 | -1,812418401 | -2,037551727 | 0,225133325 | |
| 219388_at | GRHL2 | -1,812418401 | -2,037551727 | 0,225133325 | |
| 220813_at | CYSLTR2 | -1,812418401 | -2,037551727 | 0,225133325 | |
| 228484_s_at | FOXO1 | -1,812418401 | -2,037551727 | 0,225133325 | |
| 231945_at | FILIP1 | -1,812418401 | -2,037551727 | 0,225133325 | |
| 234848_at | - | -1,812418401 | -2,037551727 | 0,225133325 | |
| 236756_at | CENPVP1 /// CEI | -1,812418401 | -2,037551727 | 0,225133325 | |
| 239471_at | LRRC28 | -1,812418401 | -2,037551727 | 0,225133325 | |
| 239685_at | - | -1,812418401 | -2,037551727 | 0,225133325 | |
| 240378_at | - | -1,812418401 | -2,037551727 | 0,225133325 | |
| 241008_at | - | -1,812418401 | -2,037551727 | 0,225133325 | |
| 241631_at | - | -1,812418401 | -2,037551727 | 0,225133325 | |
| 241995_at | DGUOK | -1,812418401 | -2,037551727 | 0,225133325 | |
| 244232_at | - | -1,812418401 | -2,037551727 | 0,225133325 | |
| 218213_s_at | C11orf10 | 6,331845983 | 6,10680026 | 0,225045724 | |
| 201732_s_at | CLCN3 | 1,549482847 | 1,324440845 | 0,225042001 | |
| 227356_at | LOC100507015 | 1,549482847 | 1,324440845 | 0,225042001 | |
| 221891_x_at | HSPA8 /// SNOR | 7,5021223 | 7,277120604 | 0,225001697 | |
| 235773_at | ZIK1 | 0,57782909 | 0,352836757 | 0,224992333 | |
| 235939_at | - | 0,57782909 | 0,352836757 | 0,224992333 | |
| 214906_x_at | N4BP2L1 | 0,401366113 | 0,176436073 | 0,22493004 | |
| 237504_at | INTS10 | 0,401366113 | 0,176436073 | 0,22493004 | |
| 243539_at | KIAA1841 | 0,401366113 | 0,176436073 | 0,22493004 | |

| | | | | | |
|---|---|---|---|---|---|
| 1553741_at | LINC00337 | -1,937711681 | -2,162639771 | 0,22492809 | |
| 1555019_at | CDHR1 | -1,937711681 | -2,162639771 | 0,22492809 | |
| 1558300_at | EFCAB5 | -1,937711681 | -2,162639771 | 0,22492809 | |
| 1566505_at | ERVK13-1 | -1,937711681 | -2,162639771 | 0,22492809 | |
| 1570393_at | EML5 | -1,937711681 | -2,162639771 | 0,22492809 | |
| 204380_s_at | FGFR3 | -1,937711681 | -2,162639771 | 0,22492809 | |
| 205772_s_at | - | -1,937711681 | -2,162639771 | 0,22492809 | |
| 214200_s_at | COL6A1 | -1,937711681 | -2,162639771 | 0,22492809 | |
| 216687_x_at | UGT2B15 | -1,937711681 | -2,162639771 | 0,22492809 | |
| 217616_at | - | -1,937711681 | -2,162639771 | 0,22492809 | |
| 223817_at | LRRIQ1 | -1,937711681 | -2,162639771 | 0,22492809 | |
| 224422_x_at | PMCHL2 | -1,937711681 | -2,162639771 | 0,22492809 | |
| 230915_at | DHRS7C | -1,937711681 | -2,162639771 | 0,22492809 | |
| 238480_at | TTC39C | -1,937711681 | -2,162639771 | 0,22492809 | |
| 240791_at | - | -1,937711681 | -2,162639771 | 0,22492809 | |
| 241240_at | - | -1,937711681 | -2,162639771 | 0,22492809 | |
| 241717_at | LOC285281 | -1,937711681 | -2,162639771 | 0,22492809 | |
| 241844_x_at | TMEM156 | -1,937711681 | -2,162639771 | 0,22492809 | |
| 242024_at | - | -1,937711681 | -2,162639771 | 0,22492809 | |
| 243187_at | - | -1,937711681 | -2,162639771 | 0,22492809 | |
| 244164_at | - | -1,937711681 | -2,162639771 | 0,22492809 | |
| 244325_at | - | -1,937711681 | -2,162639771 | 0,22492809 | |
| 244418_at | - | -1,937711681 | -2,162639771 | 0,22492809 | |
| 205742_at | TNNI3 | 0,20027154 | -0,024572586 | 0,224844126 | |
| 212905_at | CSTF2T | 1,201332287 | 0,976641161 | 0,224691126 | |
| 205293_x_at | BAIAP2 | -0,033456564 | -0,258114234 | 0,22465767 | |
| 240235_at | C10orf62 | -0,033456564 | -0,258114234 | 0,22465767 | |
| 240607_at | MIAT | -0,033456564 | -0,258114234 | 0,22465767 | |
| 241919_x_at | WDR31 | -0,033456564 | -0,258114234 | 0,22465767 | |
| 201968_s_at | PGM1 | 1,089278259 | 0,864649967 | 0,224628292 | |
| 216387_x_at | - | 1,089278259 | 0,864649967 | 0,224628292 | |
| 244641_at | MALSU1 | 1,089278259 | 0,864649967 | 0,224628292 | |
| 204668_at | RNF24 | 2,566371329 | 2,341749893 | 0,224621437 | |
| 236492_at | PPP2R2A | 2,146943689 | 1,922365006 | 0,224578683 | |
| 1567410_at | ZNF135 | 1,238353345 | 1,013870809 | 0,224482536 | |
| 213292_s_at | SNX13 | 1,968405881 | 1,743944487 | 0,224461394 | |
| 202051_s_at | ZMYM4 | 3,340506684 | 3,116064987 | 0,224441697 | |
| 237779_at | LOC100505711 | 1,129248302 | 0,904843258 | 0,224405043 | |
| 238007_at | ZNF271 | 1,129248302 | 0,904843258 | 0,224405043 | |
| 1556312_at | C2orf71 | -0,888496123 | -1,112851844 | 0,224355721 | |
| 1557719_at | PIKFYVE | -0,888496123 | -1,112851844 | 0,224355721 | |
| 1565000_a_at | TCP11L2 | -0,888496123 | -1,112851844 | 0,224355721 | |
| 205819_at | MARCO | -0,888496123 | -1,112851844 | 0,224355721 | |
| 208476_s_at | FRMD4A | -0,888496123 | -1,112851844 | 0,224355721 | |
| 219998_at | LGALSL | -0,888496123 | -1,112851844 | 0,224355721 | |
| 221256_s_at | HDHD3 | -0,888496123 | -1,112851844 | 0,224355721 | |
| 224051_at | - | -0,888496123 | -1,112851844 | 0,224355721 | |
| 230318_at | SERPINA1 | -0,888496123 | -1,112851844 | 0,224355721 | |
| 231359_at | APOH | -0,888496123 | -1,112851844 | 0,224355721 | |
| 238531_x_at | - | -0,888496123 | -1,112851844 | 0,224355721 | |
| 239015_at | THAP7-AS1 | -0,888496123 | -1,112851844 | 0,224355721 | |
| 239232_at | MSI2 | -0,888496123 | -1,112851844 | 0,224355721 | |
| 242713_at | - | -0,888496123 | -1,112851844 | 0,224355721 | |
| 224900_at | ANKFY1 | 3,554701391 | 3,330438773 | 0,224262619 | |
| 212232_at | FNBP4 | 3,406034785 | 3,181801492 | 0,224233293 | |
| 227017_at | ERICH1 | 3,541801785 | 3,317734938 | 0,224066847 | |

| | | | | | |
|---|---|---|---|---|---|
| 212693_at | MDN1 | 3,367911651 | 3,14392006 | 0,223991591 | |
| 1570198_x_at | - | 0,928678725 | 0,704715219 | 0,223963506 | |
| 204761_at | USP6NL | 0,928678725 | 0,704715219 | 0,223963506 | |
| 1553752_at | SPATA25 | -1,688127507 | -1,912089812 | 0,223962305 | |
| 1555108_at | SLC10A7 | -1,688127507 | -1,912089812 | 0,223962305 | |
| 1556879_at | - | -1,688127507 | -1,912089812 | 0,223962305 | |
| 1558600_a_at | - | -1,688127507 | -1,912089812 | 0,223962305 | |
| 1561375_at | - | -1,688127507 | -1,912089812 | 0,223962305 | |
| 1561427_at | - | -1,688127507 | -1,912089812 | 0,223962305 | |
| 1569659_at | LOC100506895 | -1,688127507 | -1,912089812 | 0,223962305 | |
| 206655_s_at | GP1BB /// SEPT5 | -1,688127507 | -1,912089812 | 0,223962305 | |
| 213155_at | LOC339166 /// W | -1,688127507 | -1,912089812 | 0,223962305 | |
| 213158_at | - | -1,688127507 | -1,912089812 | 0,223962305 | |
| 215972_at | PART1 | -1,688127507 | -1,912089812 | 0,223962305 | |
| 220646_s_at | KLRF1 | -1,688127507 | -1,912089812 | 0,223962305 | |
| 230660_at | SERTAD4 | -1,688127507 | -1,912089812 | 0,223962305 | |
| 231343_at | - | -1,688127507 | -1,912089812 | 0,223962305 | |
| 233262_at | - | -1,688127507 | -1,912089812 | 0,223962305 | |
| 234742_at | SIRPG | -1,688127507 | -1,912089812 | 0,223962305 | |
| 237406_at | NEK6 | -1,688127507 | -1,912089812 | 0,223962305 | |
| 238999_at | - | -1,688127507 | -1,912089812 | 0,223962305 | |
| 241046_at | LOC100505903 | -1,688127507 | -1,912089812 | 0,223962305 | |
| 244722_at | - | -1,688127507 | -1,912089812 | 0,223962305 | |
| 208832_at | ATXN10 | 1,224581536 | 1,000684521 | 0,223897015 | |
| 218971_s_at | WDR91 | 1,666156729 | 1,442269454 | 0,223887274 | |
| 243643_x_at | SLC30A6 | 2,014487247 | 1,790660532 | 0,223826715 | |
| 222737_s_at | BRD7 | 2,896728217 | 2,672945617 | 0,223782599 | |
| 227286_at | INO80E | 1,951783949 | 1,728030156 | 0,223753792 | |
| 215111_s_at | TSC22D1 | 6,554194938 | 6,330515692 | 0,223679246 | |
| 206187_at | PTGIR | 0,641402394 | 0,417770464 | 0,223631931 | |
| 226781_at | C7orf55 | 4,036776538 | 3,813221015 | 0,223555523 | |
| 222727_s_at | SLC24A6 | -0,423248115 | -0,646746079 | 0,223497963 | |
| 228360_at | LYPD6B | -0,423248115 | -0,646746079 | 0,223497963 | |
| 236041_at | - | -0,423248115 | -0,646746079 | 0,223497963 | |
| 230102_at | ETV5 | 1,314003737 | 1,090553537 | 0,2234502 | |
| 1552772_at | CLEC4D | -2,063149277 | -2,286583451 | 0,223434173 | |
| 1553194_at | NEGR1 | -2,063149277 | -2,286583451 | 0,223434173 | |
| 1555400_at | LOC645261 | -2,063149277 | -2,286583451 | 0,223434173 | |
| 1557835_at | - | -2,063149277 | -2,286583451 | 0,223434173 | |
| 1569033_at | IGF2BP3 | -2,063149277 | -2,286583451 | 0,223434173 | |
| 211907_s_at | PARD6B | -2,063149277 | -2,286583451 | 0,223434173 | |
| 214642_x_at | MAGEA10-MAGE | -2,063149277 | -2,286583451 | 0,223434173 | |
| 215601_at | - | -2,063149277 | -2,286583451 | 0,223434173 | |
| 229708_at | C9orf167 | -2,063149277 | -2,286583451 | 0,223434173 | |
| 229739_s_at | FAM116B | -2,063149277 | -2,286583451 | 0,223434173 | |
| 230607_at | - | -2,063149277 | -2,286583451 | 0,223434173 | |
| 232497_at | ZNF3 | -2,063149277 | -2,286583451 | 0,223434173 | |
| 233578_at | C1orf101 | -2,063149277 | -2,286583451 | 0,223434173 | |
| 234036_x_at | - | -2,063149277 | -2,286583451 | 0,223434173 | |
| 234378_at | - | -2,063149277 | -2,286583451 | 0,223434173 | |
| 235521_at | HOXA3 | -2,063149277 | -2,286583451 | 0,223434173 | |
| 236432_at | - | -2,063149277 | -2,286583451 | 0,223434173 | |
| 236543_at | - | -2,063149277 | -2,286583451 | 0,223434173 | |
| 236952_at | - | -2,063149277 | -2,286583451 | 0,223434173 | |
| 238834_at | MYLK3 | -2,063149277 | -2,286583451 | 0,223434173 | |
| 244581_at | ZBTB20 | -2,063149277 | -2,286583451 | 0,223434173 | |

| | | | | | |
|---|---|---|---|---|---|
| 218775_s_at | CLDN22 /// WW( | 1,560488174 | 1,337086025 | 0,223402149 | |
| 207011_s_at | PTK7 | 1,210676994 | 0,987376598 | 0,223300396 | |
| 219114_at | C3orf18 | 1,210676994 | 0,987376598 | 0,223300396 | |
| 226493_at | KCTD18 | 1,210676994 | 0,987376598 | 0,223300396 | |
| 201643_x_at | KDM3B | 4,10413628 | 3,880994209 | 0,22314207 | |
| 228868_x_at | CDT1 | 2,901063789 | 2,677924589 | 0,2231392 | |
| 1560560_at | - | 1,820612054 | 1,59747851 | 0,223133544 | |
| 226652_at | USP3 | 1,722769813 | 1,499637023 | 0,223132791 | |
| 226609_at | DCBLD1 | 1,504602299 | 1,281471383 | 0,223130916 | |
| 226117_at | TIFA | 0,533835206 | 0,310717132 | 0,223118074 | |
| 1554982_a_at | C9orf11 | -2,667025278 | -2,89013181 | 0,223106533 | |
| 1555057_at | NDUFS4 | -2,667025278 | -2,89013181 | 0,223106533 | |
| 1555571_at | IMMP2L | -2,667025278 | -2,89013181 | 0,223106533 | |
| 1556167_at | HEATR7B1 | -2,667025278 | -2,89013181 | 0,223106533 | |
| 1556288_at | C18orf62 | -2,667025278 | -2,89013181 | 0,223106533 | |
| 1560491_at | LOC100507193 | -2,667025278 | -2,89013181 | 0,223106533 | |
| 1561351_at | - | -2,667025278 | -2,89013181 | 0,223106533 | |
| 1562761_at | NMRK1 | -2,667025278 | -2,89013181 | 0,223106533 | |
| 1562828_at | - | -2,667025278 | -2,89013181 | 0,223106533 | |
| 1564273_at | - | -2,667025278 | -2,89013181 | 0,223106533 | |
| 1569168_at | - | -2,667025278 | -2,89013181 | 0,223106533 | |
| 206638_at | HTR2B | -2,667025278 | -2,89013181 | 0,223106533 | |
| 210516_at | FAM120A | -2,667025278 | -2,89013181 | 0,223106533 | |
| 213317_at | CLIC5 | -2,667025278 | -2,89013181 | 0,223106533 | |
| 217454_at | - | -2,667025278 | -2,89013181 | 0,223106533 | |
| 220359_s_at | ARPP21 | -2,667025278 | -2,89013181 | 0,223106533 | |
| 221152_at | COL8A1 | -2,667025278 | -2,89013181 | 0,223106533 | |
| 1552340_at | SP7 | -1,639579028 | -1,862647763 | 0,223068734 | |
| 1556498_at | FAM69A | -1,639579028 | -1,862647763 | 0,223068734 | |
| 207043_s_at | SLC6A9 | -1,639579028 | -1,862647763 | 0,223068734 | |
| 210118_s_at | IL1A | -1,639579028 | -1,862647763 | 0,223068734 | |
| 217435_x_at | - | -1,639579028 | -1,862647763 | 0,223068734 | |
| 229381_at | C1orf64 | -1,639579028 | -1,862647763 | 0,223068734 | |
| 229967_at | CMTM2 | -1,639579028 | -1,862647763 | 0,223068734 | |
| 231642_at | C6orf201 | -1,639579028 | -1,862647763 | 0,223068734 | |
| 233783_at | - | -1,639579028 | -1,862647763 | 0,223068734 | |
| 234270_at | - | -1,639579028 | -1,862647763 | 0,223068734 | |
| 237004_at | - | -1,639579028 | -1,862647763 | 0,223068734 | |
| 238786_at | ANK3 | -1,639579028 | -1,862647763 | 0,223068734 | |
| 244280_at | LOC100507254 | -1,639579028 | -1,862647763 | 0,223068734 | |
| 244512_at | HOXB-AS3 | -1,639579028 | -1,862647763 | 0,223068734 | |
| 200916_at | TAGLN2 | 6,406236089 | 6,183200519 | 0,22303557 | |
| 208743_s_at | YWHAB | 5,786920447 | 5,563892546 | 0,223027902 | |
| 231406_at | ORAI2 | 1,414471577 | 1,191491281 | 0,222980296 | |
| 1562254_at | - | 0,351548291 | 0,128747141 | 0,22280115 | |
| 210977_s_at | HSF4 | 0,351548291 | 0,128747141 | 0,22280115 | |
| 211135_x_at | LILRA6 /// LILRB: | 0,351548291 | 0,128747141 | 0,22280115 | |
| 212850_s_at | LRP4 | 0,351548291 | 0,128747141 | 0,22280115 | |
| 219133_at | OXSM | 3,223057183 | 3,000258961 | 0,222798223 | |
| 228954_at | LYSMD4 | 1,593009234 | 1,370274469 | 0,222734765 | |
| 201443_s_at | ATP6AP2 | 6,596523732 | 6,373860376 | 0,222663355 | |
| 225228_at | DRAM2 | 1,352503698 | 1,129843209 | 0,22266049 | |
| 201259_s_at | SYPL1 | 4,462972394 | 4,24031851 | 0,222653884 | |
| 226782_at | SLC25A30 | 2,043538949 | 1,820984782 | 0,222554167 | |
| 222442_s_at | ARL8B | 4,170287948 | 3,947788666 | 0,222499281 | |
| 36084_at | CUL7 | 1,82518214 | 1,602724378 | 0,222457762 | |

| | | | | | |
|---|---|---|---|---|---|
| 1563019_at | - | -0,543051993 | -0,765504029 | 0,222452036 | |
| 1565694_at | DTYMK | -0,543051993 | -0,765504029 | 0,222452036 | |
| 206618_at | IL18R1 | -0,543051993 | -0,765504029 | 0,222452036 | |
| 216253_s_at | PARVB | -0,543051993 | -0,765504029 | 0,222452036 | |
| 216888_at | LDB3 | -0,543051993 | -0,765504029 | 0,222452036 | |
| 221038_at | - | -0,543051993 | -0,765504029 | 0,222452036 | |
| 239832_at | - | -0,543051993 | -0,765504029 | 0,222452036 | |
| 239996_x_at | ATP2A2 | -0,543051993 | -0,765504029 | 0,222452036 | |
| 240258_at | ENO1 | -0,543051993 | -0,765504029 | 0,222452036 | |
| 241392_at | TMEM39A | -0,543051993 | -0,765504029 | 0,222452036 | |
| 202484_s_at | MBD2 | 3,966117062 | 3,743690278 | 0,222426784 | |
| 1552826_at | SLC26A7 | -3,013665402 | -3,236070201 | 0,222404799 | |
| 1557644_at | - | -3,013665402 | -3,236070201 | 0,222404799 | |
| 221154_at | LOC100131392 / | -3,013665402 | -3,236070201 | 0,222404799 | |
| 221212_x_at | PBRM1 | -3,013665402 | -3,236070201 | 0,222404799 | |
| 237902_at | - | -3,013665402 | -3,236070201 | 0,222404799 | |
| 240662_at | - | -3,013665402 | -3,236070201 | 0,222404799 | |
| 243274_x_at | - | -3,013665402 | -3,236070201 | 0,222404799 | |
| 1554154_at | GDAP2 | 1,143956113 | 0,921731975 | 0,222224138 | |
| 219017_at | ETNK1 | 1,143956113 | 0,921731975 | 0,222224138 | |
| 213253_at | SMC2 | 1,940595304 | 1,718396606 | 0,222198698 | |
| 202934_at | HK2 | 4,529358299 | 4,307183466 | 0,222174833 | |
| 209229_s_at | PPP6R1 | 2,525769574 | 2,303602088 | 0,222167487 | |
| 227861_at | TMEM161B | 3,489018063 | 3,266872397 | 0,222145666 | |
| 35666_at | SEMA3F | 1,481627361 | 1,259496994 | 0,222130367 | |
| 205352_at | SERPINI1 | 3,809644985 | 3,587527995 | 0,22211699 | |
| 213227_at | LOC100652849 / | 3,604290432 | 3,382199319 | 0,222091113 | |
| 228107_at | LOC100127983 | 4,00557508 | 3,783501543 | 0,222073537 | |
| 212615_at | CHD9 | 2,497416546 | 2,275409734 | 0,222006811 | |
| 221069_s_at | TACO1 | 2,497416546 | 2,275409734 | 0,222006811 | |
| 1569642_at | F2R | 2,886560922 | 2,664608955 | 0,221951967 | |
| 231277_x_at | DTWD2 | 1,027166904 | 0,805216064 | 0,22195084 | |
| 1558425_x_at | LINC00265 /// LC | -0,196756757 | -0,41858459 | 0,221827833 | |
| 203585_at | ZNF185 | -0,196756757 | -0,41858459 | 0,221827833 | |
| 232604_at | ZNF541 | -0,196756757 | -0,41858459 | 0,221827833 | |
| 233836_at | TNRC6A | -0,196756757 | -0,41858459 | 0,221827833 | |
| 236350_at | - | -0,196756757 | -0,41858459 | 0,221827833 | |
| 238578_at | TMEM182 | -0,196756757 | -0,41858459 | 0,221827833 | |
| 1557950_at | NEMF | 3,127619759 | 2,905803877 | 0,221815882 | |
| 212541_at | FLAD1 | 3,088871381 | 2,867091644 | 0,221779737 | |
| 203515_s_at | PMVK | 1,758252436 | 1,536652708 | 0,221599729 | |
| 232278_s_at | DEPDC1 | 2,470441349 | 2,248887791 | 0,221553558 | |
| 202812_at | GAA | 1,326951551 | 1,105412836 | 0,221538715 | |
| 215382_x_at | TPSAB1 | 0,945567441 | 0,724100169 | 0,221467272 | |
| 224881_at | VKORC1L1 | 2,573632254 | 2,352169913 | 0,221462341 | |
| 1558675_s_at | NEMF | 3,75958912 | 3,538185672 | 0,221403448 | |
| 213210_at | TAF6L | 0,425644434 | 0,204310755 | 0,221333678 | |
| 238904_at | NOVA2 | 0,425644434 | 0,204310755 | 0,221333678 | |
| 210175_at | GCFC2 | 2,644299732 | 2,423075926 | 0,221223806 | |
| 209441_at | RHOBTB2 | 0,810733188 | 0,589657387 | 0,221075801 | |
| 201301_s_at | ANXA4 | 2,562727117 | 2,341749893 | 0,220977224 | |
| 1553482_at | C15orf32 | -2,187914861 | -2,408705138 | 0,220790276 | |
| 1555634_a_at | LILRA5 | -2,187914861 | -2,408705138 | 0,220790276 | |
| 1556231_a_at | - | -2,187914861 | -2,408705138 | 0,220790276 | |
| 1558949_at | TNRC18 | -2,187914861 | -2,408705138 | 0,220790276 | |
| 1566780_at | - | -2,187914861 | -2,408705138 | 0,220790276 | |

| | | | | | |
|---|---|---|---|---|---|
| 206924_at | IL11 | -2,187914861 | -2,408705138 | 0,220790276 | |
| 207420_at | COLEC10 | -2,187914861 | -2,408705138 | 0,220790276 | |
| 215078_at | LOC100129518 / | -2,187914861 | -2,408705138 | 0,220790276 | |
| 217562_at | FAM5C | -2,187914861 | -2,408705138 | 0,220790276 | |
| 220456_at | SPTLC3 | -2,187914861 | -2,408705138 | 0,220790276 | |
| 228794_at | XIRP2 | -2,187914861 | -2,408705138 | 0,220790276 | |
| 231593_at | - | -2,187914861 | -2,408705138 | 0,220790276 | |
| 232875_at | - | -2,187914861 | -2,408705138 | 0,220790276 | |
| 232924_at | LRRC17 | -2,187914861 | -2,408705138 | 0,220790276 | |
| 233438_at | - | -2,187914861 | -2,408705138 | 0,220790276 | |
| 233887_at | GPR126 | -2,187914861 | -2,408705138 | 0,220790276 | |
| 237547_at | - | -2,187914861 | -2,408705138 | 0,220790276 | |
| 237798_at | - | -2,187914861 | -2,408705138 | 0,220790276 | |
| 237893_at | - | -2,187914861 | -2,408705138 | 0,220790276 | |
| 238524_at | - | -2,187914861 | -2,408705138 | 0,220790276 | |
| 238697_at | - | -2,187914861 | -2,408705138 | 0,220790276 | |
| 239095_at | - | -2,187914861 | -2,408705138 | 0,220790276 | |
| 223336_s_at | RAB18 | 4,035462473 | 3,814728712 | 0,22073376 | |
| 219763_at | DENND1A | 0,228146222 | 0,007421914 | 0,220724308 | |
| 228278_at | NFIX | 0,228146222 | 0,007421914 | 0,220724308 | |
| 218933_at | SPATA5L1 | 2,2992452 | 2,078620836 | 0,220624365 | |
| 219328_at | DDX31 | 2,506929603 | 2,286318242 | 0,220611361 | |
| 207821_s_at | LOC100653024 / | 3,431234921 | 3,210719429 | 0,220515492 | |
| 52164_at | C11orf24 | 3,317444932 | 3,096985084 | 0,220459848 | |
| 1554171_at | ZMYM3 | 0,715481152 | 0,495031851 | 0,220449301 | |
| 212486_s_at | FYN | 0,715481152 | 0,495031851 | 0,220449301 | |
| 229838_at | NUCB2 | 1,493160564 | 1,272721751 | 0,220438813 | |
| 202152_x_at | USF2 | 3,566577193 | 3,346162854 | 0,220414339 | |
| 221626_at | ZNF506 | 1,037706275 | 0,817300337 | 0,220405938 | |
| 219260_s_at | C17orf81 | 2,779171778 | 2,558821855 | 0,220349923 | |
| 208246_x_at | - | 4,017604437 | 3,797294336 | 0,220310101 | |
| 212441_at | KIAA0232 | 3,336243945 | 3,116064987 | 0,220178958 | |
| 242283_at | DNAH14 | 1,638726528 | 1,418668082 | 0,220058446 | |
| 228378_at | C12orf29 | 3,84757742 | 3,62753841 | 0,22003901 | |
| 225723_at | CCDC167 | 3,268508253 | 3,048471686 | 0,220036567 | |
| 209837_at | AP4M1 | 1,434549838 | 1,214517879 | 0,220031959 | |
| 1558766_at | - | -1,518072576 | -1,738067356 | 0,21999478 | |
| 1560785_at | DYRK3 | -1,518072576 | -1,738067356 | 0,21999478 | |
| 1561226_at | XCR1 | -1,518072576 | -1,738067356 | 0,21999478 | |
| 1565765_x_at | C13orf33 | -1,518072576 | -1,738067356 | 0,21999478 | |
| 1567320_at | - | -1,518072576 | -1,738067356 | 0,21999478 | |
| 1569449_a_at | - | -1,518072576 | -1,738067356 | 0,21999478 | |
| 203634_s_at | CPT1A | -1,518072576 | -1,738067356 | 0,21999478 | |
| 208134_x_at | PSG2 | -1,518072576 | -1,738067356 | 0,21999478 | |
| 214139_at | ARID4B | -1,518072576 | -1,738067356 | 0,21999478 | |
| 217311_at | - | -1,518072576 | -1,738067356 | 0,21999478 | |
| 223995_at | SLC12A9 | -1,518072576 | -1,738067356 | 0,21999478 | |
| 227148_at | PLEKHH2 | -1,518072576 | -1,738067356 | 0,21999478 | |
| 228461_at | SH3RF3 | -1,518072576 | -1,738067356 | 0,21999478 | |
| 228491_at | KRT19 | -1,518072576 | -1,738067356 | 0,21999478 | |
| 231261_at | CATSPERG | -1,518072576 | -1,738067356 | 0,21999478 | |
| 236596_at | - | -1,518072576 | -1,738067356 | 0,21999478 | |
| 238792_at | PCNX | -1,518072576 | -1,738067356 | 0,21999478 | |
| 242179_s_at | TSPAN16 | -1,518072576 | -1,738067356 | 0,21999478 | |
| 243408_at | - | -1,518072576 | -1,738067356 | 0,21999478 | |
| 207719_x_at | CEP170 | 4,282548314 | 4,062643488 | 0,219904826 | |

| | | | | | |
|---|---|---|---|---|---|
| 210211_s_at | HSP90AA1 | 7,668079424 | 7,44818613 | 0,219893294 | |
| 219322_s_at | WRAP73 | 2,782308844 | 2,562416132 | 0,219892712 | |
| 223575_at | KIAA1549 | 0,555999837 | 0,336136178 | 0,219863659 | |
| 210964_s_at | GYG2 | -0,00073712 | -0,220592223 | 0,219855103 | |
| 212283_at | AGRN | -0,00073712 | -0,220592223 | 0,219855103 | |
| 241939_at | IQGAP3 | -0,00073712 | -0,220592223 | 0,219855103 | |
| 243185_at | LOC100507228 | -0,00073712 | -0,220592223 | 0,219855103 | |
| 219873_at | COLEC11 | 0,95671774 | 0,73688026 | 0,21983748 | |
| 238448_at | MRPL19 | 0,95671774 | 0,73688026 | 0,21983748 | |
| 236193_at | HIST1H2BC | 2,819431021 | 2,599625088 | 0,219805933 | |
| 204397_at | EML2 | 0,613492853 | 0,393761504 | 0,219731349 | |
| 211540_s_at | RB1 | 0,613492853 | 0,393761504 | 0,219731349 | |
| 209491_s_at | AMPD3 | 0,083054335 | -0,136643983 | 0,219698317 | |
| 213276_at | CAMK2B | 0,083054335 | -0,136643983 | 0,219698317 | |
| 215668_s_at | PLXNB1 | 0,083054335 | -0,136643983 | 0,219698317 | |
| 231221_at | CLEC16A | 0,083054335 | -0,136643983 | 0,219698317 | |
| 238557_at | LOC100144603 | 0,083054335 | -0,136643983 | 0,219698317 | |
| 202819_s_at | TCEB3 | 3,217273782 | 2,997606395 | 0,219667386 | |
| 1554273_a_at | ERAP2 | -2,236765673 | -2,456406426 | 0,219640753 | |
| 1558216_at | AFAP1-AS1 | -2,236765673 | -2,456406426 | 0,219640753 | |
| 1563279_at | - | -2,236765673 | -2,456406426 | 0,219640753 | |
| 1564443_at | DLEU2 | -2,236765673 | -2,456406426 | 0,219640753 | |
| 1569846_at | - | -2,236765673 | -2,456406426 | 0,219640753 | |
| 1570354_s_at | ZNF169 | -2,236765673 | -2,456406426 | 0,219640753 | |
| 216328_at | SIGLEC8 | -2,236765673 | -2,456406426 | 0,219640753 | |
| 216359_at | MUC7 | -2,236765673 | -2,456406426 | 0,219640753 | |
| 219159_s_at | SLAMF7 | -2,236765673 | -2,456406426 | 0,219640753 | |
| 221605_s_at | PIPOX | -2,236765673 | -2,456406426 | 0,219640753 | |
| 223483_at | SERGEF | -2,236765673 | -2,456406426 | 0,219640753 | |
| 226777_at | ADAM12 | -2,236765673 | -2,456406426 | 0,219640753 | |
| 228143_at | CP | -2,236765673 | -2,456406426 | 0,219640753 | |
| 229373_at | - | -2,236765673 | -2,456406426 | 0,219640753 | |
| 233457_at | - | -2,236765673 | -2,456406426 | 0,219640753 | |
| 233584_at | C20orf62 | -2,236765673 | -2,456406426 | 0,219640753 | |
| 233700_at | - | -2,236765673 | -2,456406426 | 0,219640753 | |
| 237232_at | - | -2,236765673 | -2,456406426 | 0,219640753 | |
| 238170_at | - | -2,236765673 | -2,456406426 | 0,219640753 | |
| 239299_at | DDI1 | -2,236765673 | -2,456406426 | 0,219640753 | |
| 240874_at | - | -2,236765673 | -2,456406426 | 0,219640753 | |
| 241531_at | - | -2,236765673 | -2,456406426 | 0,219640753 | |
| 242339_at | - | -2,236765673 | -2,456406426 | 0,219640753 | |
| 243362_s_at | LEF1-AS1 | -2,236765673 | -2,456406426 | 0,219640753 | |
| 243972_at | BTF3L4 | -2,236765673 | -2,456406426 | 0,219640753 | |
| 1553963_at | RHOB | -3,054236653 | -3,273861381 | 0,219624729 | |
| 1570250_at | LPPR1 | -3,054236653 | -3,273861381 | 0,219624729 | |
| 216089_at | - | -3,054236653 | -3,273861381 | 0,219624729 | |
| 217712_at | - | -3,054236653 | -3,273861381 | 0,219624729 | |
| 234221_at | - | -3,054236653 | -3,273861381 | 0,219624729 | |
| 241151_at | - | -3,054236653 | -3,273861381 | 0,219624729 | |
| 204583_x_at | KLK3 | -0,394749123 | -0,614371577 | 0,219622453 | |
| 209499_x_at | TNFSF12 /// TNF | -0,394749123 | -0,614371577 | 0,219622453 | |
| 209641_s_at | ABCC3 | -0,394749123 | -0,614371577 | 0,219622453 | |
| 220811_at | PRG3 | -0,394749123 | -0,614371577 | 0,219622453 | |
| 39817_s_at | C6orf108 | 2,348755185 | 2,129179938 | 0,219575247 | |
| 208975_s_at | KPNB1 | 5,204666516 | 4,985166919 | 0,219499597 | |
| 209699_x_at | AKR1C2 /// LOC1 | 4,663430187 | 4,44401622 | 0,219413967 | |

| | | | | | |
|---|---|---|---|---|---|
| 202882_x_at | NOL7 | 6,161984103 | 5,942571032 | 0,219413072 | |
| 209346_s_at | PI4K2A | 1,937784532 | 1,718396606 | 0,219387926 | |
| 219231_at | TGS1 | 3,523171367 | 3,303845028 | 0,219326339 | |
| 213416_at | ITGA4 | 4,427846607 | 4,208556964 | 0,219289643 | |
| 224687_at | ANKIB1 | 1,774096855 | 1,55481039 | 0,219286465 | |
| 222482_at | SSBP3 | 3,823415835 | 3,604188755 | 0,219227081 | |
| 202547_s_at | ARHGEF7 | 1,274448124 | 1,055274826 | 0,219173298 | |
| 203912_s_at | DNASE1L1 | 0,376672223 | 0,157549243 | 0,21912298 | |
| 215766_at | GSTA1 | 0,376672223 | 0,157549243 | 0,21912298 | |
| 218600_at | LIMD2 | 0,376672223 | 0,157549243 | 0,21912298 | |
| 224443_at | LINC00467 | 0,376672223 | 0,157549243 | 0,21912298 | |
| 227906_s_at | - | 0,376672223 | 0,157549243 | 0,21912298 | |
| 201576_s_at | GLB1 | 4,041366364 | 3,822243651 | 0,219122713 | |
| 202655_at | MANF | 3,999522577 | 3,780418488 | 0,21910409 | |
| 222229_x_at | - | 6,549714613 | 6,33064743 | 0,219067183 | |
| 209208_at | MPDU1 | 3,872077355 | 3,653046807 | 0,219030548 | |
| 222070_at | DND1 | 0,44160593 | 0,222599095 | 0,219006835 | |
| 231829_at | MAVS | 0,44160593 | 0,222599095 | 0,219006835 | |
| 223450_s_at | COG3 | 1,699726954 | 1,480766833 | 0,218960122 | |
| 209849_s_at | RAD51C | 4,08838118 | 3,869433939 | 0,218947242 | |
| 207551_s_at | MSL3 | 4,191091206 | 3,972313606 | 0,2187776 | |
| 216438_s_at | TMSB4X | 7,938367101 | 7,71961713 | 0,218749971 | |
| 224282_s_at | AGPAT3 | 1,168140757 | 0,949447784 | 0,218692973 | |
| 211084_x_at | PRKD3 | 1,649074067 | 1,43051703 | 0,218557036 | |
| 1552775_at | GPRC6A | -0,512251684 | -0,730778808 | 0,218527123 | |
| 224547_at | - | -0,512251684 | -0,730778808 | 0,218527123 | |
| 238767_at | - | -0,512251684 | -0,730778808 | 0,218527123 | |
| 243430_at | SEZ6 | -0,512251684 | -0,730778808 | 0,218527123 | |
| 228909_at | LOC642852 | 2,804079361 | 2,585563171 | 0,21851619 | |
| 1556542_a_at | - | -0,83191556 | -1,050406615 | 0,218491055 | |
| 1556962_at | - | -0,83191556 | -1,050406615 | 0,218491055 | |
| 217545_at | MYH14 | -0,83191556 | -1,050406615 | 0,218491055 | |
| 219160_s_at | PAPOLG | -0,83191556 | -1,050406615 | 0,218491055 | |
| 221283_at | RUNX2 | -0,83191556 | -1,050406615 | 0,218491055 | |
| 232883_at | - | -0,83191556 | -1,050406615 | 0,218491055 | |
| 237861_at | LOC100506105 | -0,83191556 | -1,050406615 | 0,218491055 | |
| 213170_at | GPX7 | 2,009141686 | 1,790660532 | 0,218481154 | |
| 1569878_at | CCNYL2 | 2,030406107 | 1,811954245 | 0,218451863 | |
| 211932_at | HNRNPA3 | 4,501552344 | 4,283151707 | 0,218400636 | |
| 225691_at | CDK12 | 2,768138014 | 2,549796788 | 0,218341226 | |
| 205396_at | SMAD3 | -0,172274514 | -0,39061235 | 0,218337836 | |
| 215946_x_at | IGLL3P | -0,172274514 | -0,39061235 | 0,218337836 | |
| 238469_at | OGFRL1 | -0,172274514 | -0,39061235 | 0,218337836 | |
| 242969_at | ZNF780B | -0,172274514 | -0,39061235 | 0,218337836 | |
| 217993_s_at | MAT2B | 5,310855527 | 5,092541763 | 0,218313765 | |
| 208696_at | CCT5 | 6,738503329 | 6,520345731 | 0,218157598 | |
| 204651_at | NRF1 | 1,567778694 | 1,349621332 | 0,218157362 | |
| 1556265_at | C20orf202 | 0,171847695 | -0,046302147 | 0,218149842 | |
| 203866_at | NLE1 | 0,171847695 | -0,046302147 | 0,218149842 | |
| 236737_at | C17orf56 | 0,171847695 | -0,046302147 | 0,218149842 | |
| 228292_at | - | 1,05337236 | 0,835239052 | 0,218133307 | |
| 1559675_at | - | 1,094335495 | 0,876248481 | 0,218087014 | |
| 200646_s_at | NUCB1 | 1,094335495 | 0,876248481 | 0,218087014 | |
| 204512_at | HIVEP1 | 0,246434561 | 0,028364014 | 0,218070547 | |
| 226231_at | PAWR | 0,246434561 | 0,028364014 | 0,218070547 | |
| 230149_at | - | 0,246434561 | 0,028364014 | 0,218070547 | |

| | | | | | |
|---|---|---|---|---|---|
| 213152_s_at | SRSF8 | 3,228817493 | 3,010820719 | 0,217996774 | |
| 218492_s_at | THAP7 | 1,247462054 | 1,029536893 | 0,21792516 | |
| 201455_s_at | LOC100653042 / | 3,352164686 | 3,134292219 | 0,217872467 | |
| 218386_x_at | USP16 | 4,544680214 | 4,326864571 | 0,217815643 | |
| 202807_s_at | TOM1 | 0,57058931 | 0,352836757 | 0,217752553 | |
| 238475_at | ALG10B | 0,57058931 | 0,352836757 | 0,217752553 | |
| 203057_s_at | PRDM2 | 2,926807124 | 2,709065209 | 0,217741914 | |
| 220964_s_at | RAB1B | 3,276317033 | 3,058687544 | 0,217629489 | |
| 1557487_at | - | 0,735041319 | 0,51742585 | 0,217615469 | |
| 213512_at | C14orf79 | 0,735041319 | 0,51742585 | 0,217615469 | |
| 228226_s_at | ZNF775 | 0,735041319 | 0,51742585 | 0,217615469 | |
| 1569415_at | - | 1,735773781 | 1,518263577 | 0,217510204 | |
| 215155_at | HEXA | 0,97328325 | 0,755840599 | 0,217442651 | |
| 236347_at | MMAA | 0,97328325 | 0,755840599 | 0,217442651 | |
| 200910_at | CCT3 | 6,140295359 | 5,922958221 | 0,217337138 | |
| 203411_s_at | LMNA | 3,529719415 | 3,312408479 | 0,217310936 | |
| 214263_x_at | POLR2C | 4,288076367 | 4,070868415 | 0,217207952 | |
| 204917_s_at | MLLT3 | 3,492853048 | 3,275657468 | 0,217195579 | |
| 236641_at | KIF14 | 2,825526235 | 2,608344689 | 0,217181546 | |
| 225154_at | SYAP1 | 5,523564805 | 5,306440515 | 0,217124291 | |
| 204071_s_at | TOPORS | 3,665883965 | 3,448769219 | 0,217114746 | |
| 209372_x_at | TUBB2A /// TUBE | 3,207971844 | 2,990953566 | 0,217018278 | |
| 201233_at | PSMD13 | 3,815015946 | 3,598072942 | 0,216943004 | |
| 208988_at | KDM2A | 2,943717636 | 2,726791458 | 0,216926178 | |
| 1558595_at | - | -2,779459409 | -2,996335703 | 0,216876294 | |
| 1562053_at | - | -2,779459409 | -2,996335703 | 0,216876294 | |
| 1569568_at | - | -2,779459409 | -2,996335703 | 0,216876294 | |
| 215692_s_at | MPPED2 | -2,779459409 | -2,996335703 | 0,216876294 | |
| 219073_s_at | OSBPL10 | -2,779459409 | -2,996335703 | 0,216876294 | |
| 220919_s_at | WDR96 | -2,779459409 | -2,996335703 | 0,216876294 | |
| 230540_at | - | -2,779459409 | -2,996335703 | 0,216876294 | |
| 231279_at | PATE1 | -2,779459409 | -2,996335703 | 0,216876294 | |
| 232799_at | - | -2,779459409 | -2,996335703 | 0,216876294 | |
| 233035_at | - | -2,779459409 | -2,996335703 | 0,216876294 | |
| 233332_at | - | -2,779459409 | -2,996335703 | 0,216876294 | |
| 239708_at | - | -2,779459409 | -2,996335703 | 0,216876294 | |
| 240068_at | LINC00323 | -2,779459409 | -2,996335703 | 0,216876294 | |
| 240702_at | HERC1 | -2,779459409 | -2,996335703 | 0,216876294 | |
| 240726_at | - | -2,779459409 | -2,996335703 | 0,216876294 | |
| 240977_at | - | -2,779459409 | -2,996335703 | 0,216876294 | |
| 241328_at | ZMAT1 | -2,779459409 | -2,996335703 | 0,216876294 | |
| 242495_at | - | -2,779459409 | -2,996335703 | 0,216876294 | |
| 243163_at | - | -2,779459409 | -2,996335703 | 0,216876294 | |
| 244715_at | - | -2,779459409 | -2,996335703 | 0,216876294 | |
| 219350_s_at | DIABLO | 4,64373014 | 4,426866007 | 0,216864132 | |
| 202462_s_at | DDX46 | 5,2950189 | 5,078176444 | 0,216842456 | |
| 227178_at | CELF2 | 2,503131904 | 2,286318242 | 0,216813662 | |
| 1555905_a_at | C3orf23 | 0,020670649 | -0,19610998 | 0,216780629 | |
| 1556453_at | LOC100506274 | 0,020670649 | -0,19610998 | 0,216780629 | |
| 210995_s_at | TRIM23 | 0,020670649 | -0,19610998 | 0,216780629 | |
| 224152_s_at | PBRM1 | 0,020670649 | -0,19610998 | 0,216780629 | |
| 231860_at | BRWD1 | 0,020670649 | -0,19610998 | 0,216780629 | |
| 36030_at | IFFO1 | 2,010479935 | 1,793721801 | 0,216758134 | |
| 204634_at | NEK4 | 2,922548342 | 2,705818724 | 0,216729618 | |
| 204354_at | POT1 | 3,950863319 | 3,734161076 | 0,216702243 | |
| 220033_at | - | 0,841135803 | 0,624460759 | 0,216675044 | |

| | | | | | |
|---|---|---|---|---|---|
| 223759_s_at | GSG2 | 0,841135803 | 0,624460759 | 0,216675044 | |
| 204727_at | WDHD1 | 0,888485433 | 0,67181667 | 0,216668763 | |
| 212678_at | NF1 | 0,888485433 | 0,67181667 | 0,216668763 | |
| 239709_at | - | 1,021868205 | 0,805216064 | 0,216652141 | |
| 212223_at | IDS | 1,063722698 | 0,847075464 | 0,216647234 | |
| 237183_at | GALNT5 | 1,360921491 | 1,144305291 | 0,2166162 | |
| 217834_s_at | SYNCRIP | 5,231596898 | 5,014984123 | 0,216612775 | |
| 239482_x_at | ZNF708 | 1,607231019 | 1,390636111 | 0,216594908 | |
| 237364_at | - | 1,841816345 | 1,6252386 | 0,216577745 | |
| 215779_s_at | HIST1H2BC /// H | 2,854131948 | 2,637603218 | 0,216528731 | |
| 1553602_at | MUCL1 | -1,047650486 | -1,264005341 | 0,216354855 | |
| 1553661_a_at | HUS1B | -1,047650486 | -1,264005341 | 0,216354855 | |
| 1557016_a_at | C1orf177 | -1,047650486 | -1,264005341 | 0,216354855 | |
| 1559880_at | LZTS1-AS1 | -1,047650486 | -1,264005341 | 0,216354855 | |
| 200939_s_at | RERE | -1,047650486 | -1,264005341 | 0,216354855 | |
| 206910_x_at | CFHR2 | -1,047650486 | -1,264005341 | 0,216354855 | |
| 207795_s_at | KLRD1 | -1,047650486 | -1,264005341 | 0,216354855 | |
| 217628_at | CLIC5 | -1,047650486 | -1,264005341 | 0,216354855 | |
| 226139_at | CCDC149 | -1,047650486 | -1,264005341 | 0,216354855 | |
| 229245_at | PLEKHA6 | -1,047650486 | -1,264005341 | 0,216354855 | |
| 230222_at | - | -1,047650486 | -1,264005341 | 0,216354855 | |
| 232200_at | HSPA9 | -1,047650486 | -1,264005341 | 0,216354855 | |
| 233395_at | - | -1,047650486 | -1,264005341 | 0,216354855 | |
| 234319_at | STOX2 | -1,047650486 | -1,264005341 | 0,216354855 | |
| 234438_at | - | -1,047650486 | -1,264005341 | 0,216354855 | |
| 236505_at | NUP62 | -1,047650486 | -1,264005341 | 0,216354855 | |
| 237128_at | - | -1,047650486 | -1,264005341 | 0,216354855 | |
| 241310_at | - | -1,047650486 | -1,264005341 | 0,216354855 | |
| 208999_at | 39692 | 3,453052675 | 3,236821511 | 0,216231164 | |
| 225653_at | TGFBRAP1 | 1,795990708 | 1,579853228 | 0,216137479 | |
| 203341_at | CEBPZ | 4,571221501 | 4,355114902 | 0,216106599 | |
| 215359_x_at | ZNF44 | 2,783874823 | 2,567790814 | 0,216084009 | |
| 222774_s_at | NETO2 | 2,582657321 | 2,366632651 | 0,21602467 | |
| 232884_s_at | ZNF853 | 1,331241794 | 1,115234684 | 0,216007109 | |
| 224186_s_at | RNF123 | 1,296557217 | 1,080561626 | 0,215995591 | |
| 212954_at | DYRK4 | 2,446911392 | 2,230931691 | 0,215979701 | |
| 222303_at | - | 0,984222186 | 0,768343791 | 0,215878395 | |
| 209796_s_at | CNPY2 | 4,13575284 | 3,920040657 | 0,215712183 | |
| 219349_s_at | EXOC2 | 1,823660386 | 1,60795124 | 0,215709146 | |
| 235048_at | FAM169A | 1,823660386 | 1,60795124 | 0,215709146 | |
| 1554156_a_at | WFDC8 | -2,359436556 | -2,575129729 | 0,215693173 | |
| 1554368_at | NT5C1B | -2,359436556 | -2,575129729 | 0,215693173 | |
| 1558881_at | LOC145820 | -2,359436556 | -2,575129729 | 0,215693173 | |
| 1561938_at | - | -2,359436556 | -2,575129729 | 0,215693173 | |
| 1562300_at | - | -2,359436556 | -2,575129729 | 0,215693173 | |
| 1564949_at | - | -2,359436556 | -2,575129729 | 0,215693173 | |
| 1565544_at | RNF141 | -2,359436556 | -2,575129729 | 0,215693173 | |
| 1568615_a_at | SRD5A3-AS1 | -2,359436556 | -2,575129729 | 0,215693173 | |
| 1568685_at | - | -2,359436556 | -2,575129729 | 0,215693173 | |
| 1569786_at | - | -2,359436556 | -2,575129729 | 0,215693173 | |
| 208427_s_at | ELAVL2 | -2,359436556 | -2,575129729 | 0,215693173 | |
| 215759_at | ANKRD53 | -2,359436556 | -2,575129729 | 0,215693173 | |
| 215829_at | SHANK2 | -2,359436556 | -2,575129729 | 0,215693173 | |
| 215965_at | - | -2,359436556 | -2,575129729 | 0,215693173 | |
| 221365_at | MLNR | -2,359436556 | -2,575129729 | 0,215693173 | |
| 231468_at | SH3BP4 | -2,359436556 | -2,575129729 | 0,215693173 | |

| | | | | | |
|---|---|---|---|---|---|
| 232840_at | - | -2,359436556 | -2,575129729 | 0,215693173 | |
| 232989_s_at | CCDC136 | -2,359436556 | -2,575129729 | 0,215693173 | |
| 241004_at | - | -2,359436556 | -2,575129729 | 0,215693173 | |
| 226537_at | HINT3 | 2,87191078 | 2,656223838 | 0,215686942 | |
| 1553418_a_at | CNTNAP5 | -1,399512875 | -1,615132408 | 0,215619533 | |
| 1569751_at | TBC1D26 | -1,399512875 | -1,615132408 | 0,215619533 | |
| 1570307_s_at | ST18 | -1,399512875 | -1,615132408 | 0,215619533 | |
| 203876_s_at | MMP11 | -1,399512875 | -1,615132408 | 0,215619533 | |
| 205666_at | FMO1 | -1,399512875 | -1,615132408 | 0,215619533 | |
| 205939_at | CYP3A7 | -1,399512875 | -1,615132408 | 0,215619533 | |
| 206568_at | TNP1 | -1,399512875 | -1,615132408 | 0,215619533 | |
| 207702_s_at | MAGI2 | -1,399512875 | -1,615132408 | 0,215619533 | |
| 210267_at | NIPAL3 | -1,399512875 | -1,615132408 | 0,215619533 | |
| 215805_at | - | -1,399512875 | -1,615132408 | 0,215619533 | |
| 217218_at | WAPAL | -1,399512875 | -1,615132408 | 0,215619533 | |
| 217439_at | - | -1,399512875 | -1,615132408 | 0,215619533 | |
| 217668_at | FAM211B | -1,399512875 | -1,615132408 | 0,215619533 | |
| 230841_at | - | -1,399512875 | -1,615132408 | 0,215619533 | |
| 232567_at | ARHGAP8 /// PR | -1,399512875 | -1,615132408 | 0,215619533 | |
| 236430_at | TMED6 | -1,399512875 | -1,615132408 | 0,215619533 | |
| 236568_at | CC2D1B | -1,399512875 | -1,615132408 | 0,215619533 | |
| 238601_at | PHKB | -1,399512875 | -1,615132408 | 0,215619533 | |
| 239319_at | - | -1,399512875 | -1,615132408 | 0,215619533 | |
| 217920_at | MAN1A2 | 0,46522187 | 0,249604176 | 0,215617694 | |
| 232639_at | C3orf25 | 0,46522187 | 0,249604176 | 0,215617694 | |
| 236278_at | HIST1H3E | 0,46522187 | 0,249604176 | 0,215617694 | |
| 241816_at | - | 0,46522187 | 0,249604176 | 0,215617694 | |
| 203379_at | RPS6KA1 | 1,614289682 | 1,398700978 | 0,215588704 | |
| 226352_at | JMY | 2,208874124 | 1,993317152 | 0,215556972 | |
| 1555882_at | SPIN3 | 1,496984567 | 1,281471383 | 0,215513184 | |
| 244741_s_at | LOC100128252 | 4,579810951 | 4,364410072 | 0,21540088 | |
| 213305_s_at | PPP2R5C | 3,914869478 | 3,69948842 | 0,215381058 | |
| 203006_at | INPP5A | 3,182077878 | 2,966746113 | 0,215331765 | |
| 207114_at | LY6G6C | 1,191926657 | 0,976641161 | 0,215285496 | |
| 223553_s_at | DOK3 | 2,247975581 | 2,032717854 | 0,215257727 | |
| 221263_s_at | SF3B5 | 6,616929358 | 6,401715495 | 0,215213863 | |
| 201086_x_at | SON | 5,2333172 | 5,018259423 | 0,215057777 | |
| 52159_at | HEMK1 | 1,816027445 | 1,600977874 | 0,215049571 | |
| 1552426_a_at | TM2D3 | 4,125908304 | 3,910906416 | 0,215001888 | |
| 241478_at | MICALL2 | 0,853119696 | 0,638150376 | 0,21496932 | |
| 213086_s_at | CSNK1A1 | 5,407731636 | 5,192765587 | 0,214966049 | |
| 212181_s_at | NUDT4 /// NUDT4 | 4,74085559 | 4,525916104 | 0,214939485 | |
| 224750_at | RNF185 | 2,194816625 | 1,979940779 | 0,214875846 | |
| 201017_at | EIF1AX | 4,414766661 | 4,199929321 | 0,214837339 | |
| 226866_at | ESCO1 | 3,872077355 | 3,6572547 | 0,214822655 | |
| 200806_s_at | HSPD1 | 7,160683365 | 6,945862192 | 0,214821172 | |
| 228033_at | E2F7 | 1,805273023 | 1,590454215 | 0,214818808 | |
| 1563532_at | HMCN2 /// LOC1( | -0,79540287 | -1,010192375 | 0,214789505 | |
| 204966_at | BAI2 | -0,79540287 | -1,010192375 | 0,214789505 | |
| 210172_at | SF1 | -0,79540287 | -1,010192375 | 0,214789505 | |
| 211245_x_at | KIR2DL4 | -0,79540287 | -1,010192375 | 0,214789505 | |
| 214019_at | - | -0,79540287 | -1,010192375 | 0,214789505 | |
| 215423_at | - | -0,79540287 | -1,010192375 | 0,214789505 | |
| 224193_s_at | FCRL2 | -0,79540287 | -1,010192375 | 0,214789505 | |
| 239302_s_at | LOC100506922 | -0,79540287 | -1,010192375 | 0,214789505 | |
| 209538_at | ZNF32 | 1,305306849 | 1,090553537 | 0,214753312 | |

| | | | | | |
|---|---|---|---|---|---|
| 213685_at | LOC100506963 | 1,305306849 | 1,090553537 | 0,214753312 | |
| 218686_s_at | RHBDF1 | 1,305306849 | 1,090553537 | 0,214753312 | |
| 204146_at | RAD51AP1 | 4,394921665 | 4,180180181 | 0,214741485 | |
| 200773_x_at | PTMA | 7,449689616 | 7,234985302 | 0,214704314 | |
| 202920_at | ANK2 | 0,592200562 | 0,377530647 | 0,214669915 | |
| 222220_s_at | TSNAXIP1 | 0,592200562 | 0,377530647 | 0,214669915 | |
| 202846_s_at | LOC100505991 / | 3,177319542 | 2,962671751 | 0,214647791 | |
| 207223_s_at | PTBP3 | 1,215326747 | 1,000684521 | 0,214642226 | |
| 212323_s_at | VPS13D | 1,593009234 | 1,378453643 | 0,214555591 | |
| 227757_at | CUL4A | 1,593009234 | 1,378453643 | 0,214555591 | |
| 200786_at | PSMB7 | 6,216561377 | 6,002093644 | 0,214467732 | |
| 230724_s_at | C11orf57 | 1,079110292 | 0,864649967 | 0,214460325 | |
| 202494_at | PPIE | 2,342392905 | 2,127967638 | 0,214425267 | |
| 1554884_at | LOC100653079 / | -2,823166348 | -3,037500868 | 0,21433452 | |
| 1555435_at | AFF4 | -2,823166348 | -3,037500868 | 0,21433452 | |
| 1561713_at | - | -2,823166348 | -3,037500868 | 0,21433452 | |
| 1569504_at | LILRB4 | -2,823166348 | -3,037500868 | 0,21433452 | |
| 205069_s_at | ARHGAP26 | -2,823166348 | -3,037500868 | 0,21433452 | |
| 215339_at | NKTR | -2,823166348 | -3,037500868 | 0,21433452 | |
| 231250_at | HOXB-AS4 | -2,823166348 | -3,037500868 | 0,21433452 | |
| 231678_s_at | ADH4 | -2,823166348 | -3,037500868 | 0,21433452 | |
| 239183_at | ANGPTL1 | -2,823166348 | -3,037500868 | 0,21433452 | |
| 239480_at | - | -2,823166348 | -3,037500868 | 0,21433452 | |
| 242182_x_at | - | -2,823166348 | -3,037500868 | 0,21433452 | |
| 233334_x_at | SLX1A /// SLX1A | 2,272723257 | 2,058399602 | 0,214323655 | |
| 205329_s_at | SNX4 | 2,758612869 | 2,544354523 | 0,214258346 | |
| 1558792_x_at | AP2A1 | 0,273439642 | 0,059218869 | 0,214220773 | |
| 210824_at | - | 0,273439642 | 0,059218869 | 0,214220773 | |
| 231880_at | FAM40B | 2,652894659 | 2,438805127 | 0,214089532 | |
| 239761_at | GCNT1 | 2,102456302 | 1,888402606 | 0,214053696 | |
| 230848_s_at | MGA | 2,860082603 | 2,646096941 | 0,213985662 | |
| 1557003_at | TTC23L | -0,352951799 | -0,566887459 | 0,213935661 | |
| 217597_x_at | RAB40B | -0,352951799 | -0,566887459 | 0,213935661 | |
| 220433_at | PRRG3 | -0,352951799 | -0,566887459 | 0,213935661 | |
| 235091_at | PDE12 | -0,352951799 | -0,566887459 | 0,213935661 | |
| 240535_at | - | -0,352951799 | -0,566887459 | 0,213935661 | |
| 201586_s_at | SFPQ | 5,604662362 | 5,390735672 | 0,213926689 | |
| 229153_at | SLC7A6OS | 1,163336153 | 0,949447784 | 0,213888369 | |
| 236410_x_at | - | 1,163336153 | 0,949447784 | 0,213888369 | |
| 213302_at | PFAS | 4,249494456 | 4,035745559 | 0,213748897 | |
| 204186_s_at | PPID | 4,159471171 | 3,945725977 | 0,213745194 | |
| 217998_at | PHLDA1 | 1,683039483 | 1,469325098 | 0,213714385 | |
| 217436_x_at | HLA-J | 2,763383302 | 2,549796788 | 0,213586514 | |
| 206184_at | CRKL | 1,000476627 | 0,786897721 | 0,213578906 | |
| 223979_x_at | FTCD | 0,480753764 | 0,26733076 | 0,213423004 | |
| 234403_at | OR1I1 | 0,480753764 | 0,26733076 | 0,213423004 | |
| 236451_at | - | 0,480753764 | 0,26733076 | 0,213423004 | |
| 212426_s_at | YWHAQ | 6,483798578 | 6,270390628 | 0,213407949 | |
| 202786_at | STK39 | 0,417596971 | 0,204310755 | 0,213286215 | |
| 205248_at | DOPEY2 | 0,417596971 | 0,204310755 | 0,213286215 | |
| 206347_at | PDK3 | 0,417596971 | 0,204310755 | 0,213286215 | |
| 1557315_a_at | - | -0,136301282 | -0,34958478 | 0,213283498 | |
| 214338_at | DNAJB12 | -0,136301282 | -0,34958478 | 0,213283498 | |
| 230699_at | PGLS | -0,136301282 | -0,34958478 | 0,213283498 | |
| 238342_at | - | -0,136301282 | -0,34958478 | 0,213283498 | |
| 241867_at | - | -0,136301282 | -0,34958478 | 0,213283498 | |

| | | | | | |
|---|---|---|---|---|---|
| 242185_at | - | -0,136301282 | -0,34958478 | 0,213283498 | |
| 212692_s_at | LRBA | 4,73358102 | 4,520382831 | 0,21319819 | |
| 210260_s_at | TNFAIP8 | 5,033234456 | 4,820078204 | 0,213156253 | |
| 206141_at | MOCS3 | 1,631786706 | 1,418668082 | 0,213118624 | |
| 235796_at | - | 1,04816921 | 0,835239052 | 0,212930158 | |
| 212845_at | SAMD4A | 1,318332599 | 1,105412836 | 0,212919763 | |
| 223888_s_at | LARS | 1,515954005 | 1,303116084 | 0,212837921 | |
| 223171_at | DYM | 2,605861638 | 2,39311332 | 0,212748318 | |
| 203320_at | SH2B3 | 3,591828571 | 3,379145507 | 0,212683064 | |
| 211960_s_at | RAB7A | 4,690399017 | 4,477716877 | 0,21268214 | |
| 1561995_at | - | -0,467091869 | -0,679763839 | 0,21267197 | |
| 1562527_at | LOC441666 | -0,467091869 | -0,679763839 | 0,21267197 | |
| 206604_at | OVOL1 | -0,467091869 | -0,679763839 | 0,21267197 | |
| 230907_at | GPRC5C | -0,467091869 | -0,679763839 | 0,21267197 | |
| 232217_at | FAM26E | -0,467091869 | -0,679763839 | 0,21267197 | |
| 234417_at | - | -0,467091869 | -0,679763839 | 0,21267197 | |
| 235153_at | RNF183 | -0,467091869 | -0,679763839 | 0,21267197 | |
| 237018_at | - | -0,467091869 | -0,679763839 | 0,21267197 | |
| 244610_x_at | - | -0,467091869 | -0,679763839 | 0,21267197 | |
| 210887_s_at | EVC | 0,606430281 | 0,393761504 | 0,212668777 | |
| 232765_x_at | SLC22A31 | 0,606430281 | 0,393761504 | 0,212668777 | |
| 1559048_at | - | 0,917308734 | 0,704715219 | 0,212593515 | |
| 216051_x_at | - | 0,917308734 | 0,704715219 | 0,212593515 | |
| 221810_at | RAB15 | 0,917308734 | 0,704715219 | 0,212593515 | |
| 243370_at | CAPRIN1 | 0,917308734 | 0,704715219 | 0,212593515 | |
| 1556701_at | - | 0,133056859 | -0,079522948 | 0,212579807 | |
| 236016_at | SMARCE1 | 0,133056859 | -0,079522948 | 0,212579807 | |
| 209300_s_at | NECAP1 | 1,940595304 | 1,728030156 | 0,212565148 | |
| 227922_x_at | LOC441124 | 1,390002893 | 1,177496821 | 0,212506073 | |
| 204252_at | CDK2 | 3,738747209 | 3,526272152 | 0,212475058 | |
| 224023_s_at | BRK1 | 1,549482847 | 1,337086025 | 0,212396822 | |
| 1558179_at | ATP5J2 | 0,052199481 | -0,160136748 | 0,212336229 | |
| 209843_s_at | SOX10 | 0,052199481 | -0,160136748 | 0,212336229 | |
| 210781_x_at | GRIN1 | 0,052199481 | -0,160136748 | 0,212336229 | |
| 226500_at | ZBTB47 | 0,052199481 | -0,160136748 | 0,212336229 | |
| 241934_at | NTM | 0,052199481 | -0,160136748 | 0,212336229 | |
| 242858_at | - | 0,052199481 | -0,160136748 | 0,212336229 | |
| 31799_at | - | 0,052207609 | -0,160108781 | 0,212316389 | |
| 222500_at | PPIL1 | 4,987757501 | 4,775482021 | 0,21227548 | |
| 213971_s_at | SUZ12 /// SUZ12 | 2,987866617 | 2,775606361 | 0,212260256 | |
| 1566956_at | OR7E104P | 2,520143371 | 2,307890888 | 0,212252483 | |
| 224763_at | LOC100506548 / | 2,520143371 | 2,307890888 | 0,212252483 | |
| 204416_x_at | APOC1 | 6,723026368 | 6,510843436 | 0,212182932 | |
| 215548_s_at | SCFD1 | 3,358484112 | 3,146317015 | 0,212167098 | |
| 202673_at | DPM1 | 6,200528471 | 5,988381327 | 0,212147144 | |
| 212158_at | SDC2 | 3,914869478 | 3,702749166 | 0,212120312 | |
| 224789_at | DCAF12 | 3,12638592 | 2,914267328 | 0,212118592 | |
| 222713_s_at | FANCF | 1,251994932 | 1,039887232 | 0,212107701 | |
| 218264_at | BCCIP | 3,943876123 | 3,731768909 | 0,212107215 | |
| 221637_s_at | C11orf48 | 3,824937847 | 3,6128809 | 0,212056948 | |
| 201091_s_at | CBX3 | 5,024981547 | 4,812929194 | 0,212052353 | |
| 202781_s_at | INPP5K | 1,426552034 | 1,214517879 | 0,212034155 | |
| 222592_s_at | ACSL5 | 1,426552034 | 1,214517879 | 0,212034155 | |
| 240108_at | - | 1,426552034 | 1,214517879 | 0,212034155 | |
| 225101_s_at | SNX14 | 4,056345228 | 3,844556416 | 0,211788813 | |
| 1558821_s_at | - | 0,291166226 | 0,079428135 | 0,211738091 | |

| | | | | | |
|---|---|---|---|---|---|
| 231378_at | - | 0,291166226 | 0,079428135 | 0,211738091 | |
| 216125_s_at | RANBP9 | 2,327437554 | 2,115788273 | 0,211649281 | |
| 203049_s_at | TTC37 | 3,577453376 | 3,365837114 | 0,211616261 | |
| 229025_s_at | IMMP1L | 3,014789032 | 2,803210557 | 0,211578475 | |
| 227522_at | CMBL | 5,751544329 | 5,540164491 | 0,211379837 | |
| 201213_at | PPP1R7 | 1,672933557 | 1,461646549 | 0,211287007 | |
| 47530_at | C9orf156 | 1,601914265 | 1,390636111 | 0,211278154 | |
| 226199_at | UPRT | 2,382221458 | 2,170981159 | 0,211240299 | |
| 31861_at | IGHMBP2 | 2,415939236 | 2,204721854 | 0,211217382 | |
| 203688_at | PKD2 | 0,728550685 | 0,51742585 | 0,211124835 | |
| 229332_at | HPDL | 1,143956113 | 0,932882274 | 0,211073839 | |
| 201877_s_at | PPP2R5C | 4,08266696 | 3,871608557 | 0,211058403 | |
| 1554291_at | UHRF1BP1L | -2,479842156 | -2,690860744 | 0,211018588 | |
| 1556650_at | - | -2,479842156 | -2,690860744 | 0,211018588 | |
| 1559534_at | - | -2,479842156 | -2,690860744 | 0,211018588 | |
| 1560678_at | - | -2,479842156 | -2,690860744 | 0,211018588 | |
| 1561478_at | LINC00560 | -2,479842156 | -2,690860744 | 0,211018588 | |
| 1562576_at | - | -2,479842156 | -2,690860744 | 0,211018588 | |
| 1562588_at | - | -2,479842156 | -2,690860744 | 0,211018588 | |
| 1563098_at | IGFN1 | -2,479842156 | -2,690860744 | 0,211018588 | |
| 202311_s_at | COL1A1 | -2,479842156 | -2,690860744 | 0,211018588 | |
| 206529_x_at | SLC26A4 | -2,479842156 | -2,690860744 | 0,211018588 | |
| 210953_at | TSC22D2 | -2,479842156 | -2,690860744 | 0,211018588 | |
| 213913_s_at | TBC1D30 | -2,479842156 | -2,690860744 | 0,211018588 | |
| 214159_at | PLCE1 | -2,479842156 | -2,690860744 | 0,211018588 | |
| 215784_at | CD1E | -2,479842156 | -2,690860744 | 0,211018588 | |
| 217305_s_at | ADCY10 | -2,479842156 | -2,690860744 | 0,211018588 | |
| 229070_at | ADTRP | -2,479842156 | -2,690860744 | 0,211018588 | |
| 229475_at | MAEL | -2,479842156 | -2,690860744 | 0,211018588 | |
| 230729_at | - | -2,479842156 | -2,690860744 | 0,211018588 | |
| 233994_at | - | -2,479842156 | -2,690860744 | 0,211018588 | |
| 238181_at | - | -2,479842156 | -2,690860744 | 0,211018588 | |
| 238862_at | MFSD4 | -2,479842156 | -2,690860744 | 0,211018588 | |
| 238969_at | C3orf55 | -2,479842156 | -2,690860744 | 0,211018588 | |
| 240226_at | - | -2,479842156 | -2,690860744 | 0,211018588 | |
| 241244_at | - | -2,479842156 | -2,690860744 | 0,211018588 | |
| 243506_at | C18orf42 | -2,479842156 | -2,690860744 | 0,211018588 | |
| 244432_at | - | -2,479842156 | -2,690860744 | 0,211018588 | |
| 244539_at | - | -2,479842156 | -2,690860744 | 0,211018588 | |
| 209934_s_at | ATP2C1 | 1,104397092 | 0,893473268 | 0,210923824 | |
| 203996_s_at | C21orf2 | 0,882651021 | 0,67181667 | 0,210834351 | |
| 222316_at | - | 0,882651021 | 0,67181667 | 0,210834351 | |
| 229630_s_at | WTAP | 4,933058485 | 4,72225126 | 0,210807225 | |
| 230448_at | SLC38A10 | 1,40228911 | 1,191491281 | 0,210797829 | |
| 235572_at | SPC24 | 4,18577038 | 3,975013041 | 0,21075734 | |
| 208738_x_at | SUMO2 | 5,744725693 | 5,533999386 | 0,210726307 | |
| 221771_s_at | MPHOSPH8 | 2,243430036 | 2,032717854 | 0,210712182 | |
| 201973_s_at | CCZ1 /// CCZ1B | 5,51792998 | 5,307244054 | 0,210685926 | |
| 221270_s_at | QTRT1 | 3,771477743 | 3,560823412 | 0,210654331 | |
| 227639_at | PIGK | 1,954567604 | 1,743944487 | 0,210623116 | |
| 218760_at | COQ6 | 1,835789711 | 1,6252386 | 0,21055111 | |
| 202343_x_at | COX5B | 5,470583284 | 5,260159446 | 0,210423837 | |
| 212819_at | ASB1 | -0,325749314 | -0,536087151 | 0,210337837 | |
| 230409_at | MAGI3 | -0,325749314 | -0,536087151 | 0,210337837 | |
| 232359_at | RDH11 | -0,325749314 | -0,536087151 | 0,210337837 | |
| 233195_at | DNAI1 | -0,325749314 | -0,536087151 | 0,210337837 | |

| | | | | | |
|---|---|---|---|---|---|
| 240784_at | NAT16 | -0,325749314 | -0,536087151 | 0,210337837 | |
| 231411_at | LHFP | 0,934330293 | 0,724100169 | 0,210230124 | |
| 212530_at | NEK7 | 5,391902301 | 5,181707664 | 0,210194637 | |
| 212928_at | TSPYL4 | 3,78170256 | 3,571564634 | 0,210137926 | |
| 1556078_at | - | -1,2845686 | -1,494668682 | 0,210100081 | |
| 1556229_at | - | -1,2845686 | -1,494668682 | 0,210100081 | |
| 1563106_at | - | -1,2845686 | -1,494668682 | 0,210100081 | |
| 1569932_at | NHSL2 | -1,2845686 | -1,494668682 | 0,210100081 | |
| 206919_at | ELK4 | -1,2845686 | -1,494668682 | 0,210100081 | |
| 214964_at | - | -1,2845686 | -1,494668682 | 0,210100081 | |
| 220804_s_at | TP73 | -1,2845686 | -1,494668682 | 0,210100081 | |
| 227474_at | LOC654433 | -1,2845686 | -1,494668682 | 0,210100081 | |
| 237686_at | RNF219 | -1,2845686 | -1,494668682 | 0,210100081 | |
| 242340_at | - | -1,2845686 | -1,494668682 | 0,210100081 | |
| 1554749_s_at | CLCNKB | -0,112808516 | -0,322870281 | 0,210061765 | |
| 207835_at | FBLN1 | -0,112808516 | -0,322870281 | 0,210061765 | |
| 212225_at | EIF1 | -0,112808516 | -0,322870281 | 0,210061765 | |
| 213211_s_at | TAF6L | -0,112808516 | -0,322870281 | 0,210061765 | |
| 229098_s_at | C5orf24 | -0,112808516 | -0,322870281 | 0,210061765 | |
| 233971_at | FAM166A | -0,112808516 | -0,322870281 | 0,210061765 | |
| 234769_at | DKFZp547J222 | -0,112808516 | -0,322870281 | 0,210061765 | |
| 212454_x_at | HNRPDL | 4,285314988 | 4,075277877 | 0,210037112 | |
| 1569652_at | MLLT3 | 1,709647566 | 1,499637023 | 0,210010543 | |
| 211977_at | GPR107 | 1,709647566 | 1,499637023 | 0,210010543 | |
| 218779_x_at | EPS8L1 | 1,210676994 | 1,000684521 | 0,209992473 | |
| 212890_at | SLC38A10 | 1,339784194 | 1,129843209 | 0,209940986 | |
| 224414_s_at | CARD6 | 1,789769151 | 1,579853228 | 0,209915923 | |
| 225609_at | GSR | 3,790298086 | 3,580454966 | 0,20984312 | |
| 200602_at | APP | 3,655656719 | 3,445853256 | 0,209803463 | |
| 226473_at | CBX2 | 1,764611101 | 1,55481039 | 0,20980071 | |
| 225724_at | FLJ31306 | 2,250242994 | 2,040470494 | 0,2097725 | |
| 201646_at | SCARB2 | 0,627515113 | 0,417770464 | 0,209744649 | |
| 213204_at | CUL9 | 0,627515113 | 0,417770464 | 0,209744649 | |
| 204306_s_at | CD151 | 2,288253583 | 2,078620836 | 0,209632747 | |
| 1562998_at | - | -3,192553003 | -3,402185466 | 0,209632463 | |
| 1553755_at | NXNL1 | 1,114389003 | 0,904843258 | 0,209545745 | |
| 206519_x_at | SIGLEC6 | 0,072842263 | -0,136643983 | 0,209486245 | |
| 207125_at | ZNF225 | 0,072842263 | -0,136643983 | 0,209486245 | |
| 230092_at | UBXN10 | 0,072842263 | -0,136643983 | 0,209486245 | |
| 233961_at | - | 0,072842263 | -0,136643983 | 0,209486245 | |
| 239687_at | SLX4 | 0,072842263 | -0,136643983 | 0,209486245 | |
| 227981_at | CYB561D1 | 1,868628927 | 1,659204017 | 0,20942491 | |
| 239159_at | GOSR2 | 1,073999308 | 0,864649967 | 0,209349341 | |
| 216908_x_at | RRN3P1 | 2,688444421 | 2,479296438 | 0,209147983 | |
| 1554860_at | PTPN7 | -0,741668563 | -0,950686014 | 0,209017452 | |
| 1558594_at | ZNF219 | -0,741668563 | -0,950686014 | 0,209017452 | |
| 217442_at | LOC100131825 | -0,741668563 | -0,950686014 | 0,209017452 | |
| 231185_at | KIAA1161 | -0,741668563 | -0,950686014 | 0,209017452 | |
| 234909_at | - | -0,741668563 | -0,950686014 | 0,209017452 | |
| 235714_at | GANC | -0,741668563 | -0,950686014 | 0,209017452 | |
| 244128_x_at | GLIS1 | -0,741668563 | -0,950686014 | 0,209017452 | |
| 244765_at | - | -0,741668563 | -0,950686014 | 0,209017452 | |
| 1553397_at | CCDC13 | 1,274448124 | 1,065442793 | 0,209005331 | |
| 229068_at | CCT5 | 1,274448124 | 1,065442793 | 0,209005331 | |
| 239207_at | KDM5C | 1,274448124 | 1,065442793 | 0,209005331 | |
| 202627_s_at | SERPINE1 | 2,159061673 | 1,950068772 | 0,208992901 | |

| | | | | | |
|---|---|---|---|---|---|
| 1557070_at | LOC100130275 | -0,43775896 | -0,646746079 | 0,208987118 | |
| 205766_at | TCAP | -0,43775896 | -0,646746079 | 0,208987118 | |
| 216655_s_at | - | -0,43775896 | -0,646746079 | 0,208987118 | |
| 224016_at | HIPK2 | -0,43775896 | -0,646746079 | 0,208987118 | |
| 231295_at | ME3 | -0,43775896 | -0,646746079 | 0,208987118 | |
| 238618_at | NF2 | -0,43775896 | -0,646746079 | 0,208987118 | |
| 243417_at | ZADH2 | -0,43775896 | -0,646746079 | 0,208987118 | |
| 227033_at | PDIA3 | 1,481627361 | 1,272721751 | 0,20890561 | |
| 227143_s_at | BID | 2,139623739 | 1,930732138 | 0,208891601 | |
| 209231_s_at | DCTN5 | 3,109000457 | 2,900133867 | 0,20886659 | |
| 205260_s_at | ACYP1 | 3,294008537 | 3,08516499 | 0,208843547 | |
| 207133_x_at | ALPK1 | 1,238353345 | 1,029536893 | 0,208816452 | |
| 208860_s_at | ATRX | 2,416948786 | 2,208167653 | 0,208781133 | |
| 219972_s_at | C14orf135 | 3,248232277 | 3,039473081 | 0,208759196 | |
| 223110_at | KIAA1429 | 2,979690938 | 2,770953871 | 0,208737067 | |
| 219495_s_at | ZNF180 | 1,968405881 | 1,759685182 | 0,2087207 | |
| 235573_at | - | 0,945567441 | 0,73688026 | 0,208687181 | |
| 226413_at | LOC400027 | 2,331726354 | 2,123108223 | 0,208618131 | |
| 212930_at | ATP2B1 | 1,314003737 | 1,105412836 | 0,208590901 | |
| 1553112_s_at | CDK8 | 2,014487247 | 1,805902329 | 0,208584918 | |
| 207484_s_at | EHMT2 | 0,384950573 | 0,176436073 | 0,2085145 | |
| 211205_x_at | PIP5K1A | 0,384950573 | 0,176436073 | 0,2085145 | |
| 213703_at | LINC00342 | 0,384950573 | 0,176436073 | 0,2085145 | |
| 215725_at | DGCR11 | 0,384950573 | 0,176436073 | 0,2085145 | |
| 235397_at | LINC00174 | 0,384950573 | 0,176436073 | 0,2085145 | |
| 225152_at | ZNF622 | 3,741167469 | 3,532699346 | 0,208468122 | |
| 226163_at | ZBTB9 | 2,637386731 | 2,428994491 | 0,20839224 | |
| 201133_s_at | PJA2 | 3,453052675 | 3,244672787 | 0,208379888 | |
| 243801_x_at | MRPL30 | 1,578645857 | 1,370274469 | 0,208371387 | |
| 211814_s_at | CCNE2 | 3,203298287 | 2,994948944 | 0,208349343 | |
| 226915_s_at | ARPC5L | 3,279650757 | 3,071356473 | 0,208294285 | |
| 1555812_a_at | ARHGDIB | 3,092667038 | 2,884425449 | 0,208241589 | |
| 209424_s_at | AMACR /// C1QT | 0,518867317 | 0,310717132 | 0,208150185 | |
| 221660_at | MYL10 | 0,518867317 | 0,310717132 | 0,208150185 | |
| 228719_at | ZSWIM7 | 0,518867317 | 0,310717132 | 0,208150185 | |
| 235296_at | EIF5A2 | 0,518867317 | 0,310717132 | 0,208150185 | |
| 241954_at | FDFT1 | 0,518867317 | 0,310717132 | 0,208150185 | |
| 1553190_s_at | PARD3B | 0,317354302 | 0,109221392 | 0,20813291 | |
| 230460_at | MYLK-AS1 | 0,317354302 | 0,109221392 | 0,20813291 | |
| 208161_s_at | ABCC3 | -0,946847173 | -1,154897679 | 0,208050506 | |
| 211430_s_at | IGHG1 /// IGHG2 | -0,946847173 | -1,154897679 | 0,208050506 | |
| 216471_x_at | SSX2 /// SSX2B | -0,946847173 | -1,154897679 | 0,208050506 | |
| 216727_at | STK38 | -0,946847173 | -1,154897679 | 0,208050506 | |
| 221281_at | SRC | -0,946847173 | -1,154897679 | 0,208050506 | |
| 229799_s_at | NCAM1 | -0,946847173 | -1,154897679 | 0,208050506 | |
| 230742_at | RBM5 | -0,946847173 | -1,154897679 | 0,208050506 | |
| 234809_at | ZC4H2 | -0,946847173 | -1,154897679 | 0,208050506 | |
| 236805_at | C9orf96 | -0,946847173 | -1,154897679 | 0,208050506 | |
| 238983_at | NSUN7 | -0,946847173 | -1,154897679 | 0,208050506 | |
| 221965_at | MPHOSPH9 | 1,751865623 | 1,543943227 | 0,207922395 | |
| 229794_at | ZNF585A | 2,640847372 | 2,432926755 | 0,207920617 | |
| 220720_x_at | MZT2B | 2,206540699 | 1,998633167 | 0,207907532 | |
| 227687_at | HYLS1 | 2,86748657 | 2,659583735 | 0,207902835 | |
| 1554634_at | PDS5B | -2,930741289 | -3,138626879 | 0,20788559 | |
| 1555890_at | OR2A20P /// OR2 | -2,930741289 | -3,138626879 | 0,20788559 | |
| 1557343_at | - | -2,930741289 | -3,138626879 | 0,20788559 | |

| | | | | | |
|---|---|---|---|---|---|
| 1557463_at | - | -2,930741289 | -3,138626879 | 0,20788559 | |
| 1558945_s_at | LOC100507353 | -2,930741289 | -3,138626879 | 0,20788559 | |
| 1561720_at | RECQL5 | -2,930741289 | -3,138626879 | 0,20788559 | |
| 1562696_at | - | -2,930741289 | -3,138626879 | 0,20788559 | |
| 1564276_at | C5orf56 | -2,930741289 | -3,138626879 | 0,20788559 | |
| 1569995_at | LOC152586 | -2,930741289 | -3,138626879 | 0,20788559 | |
| 207102_at | AKR1D1 | -2,930741289 | -3,138626879 | 0,20788559 | |
| 211324_s_at | RGPD3 /// RGPD | -2,930741289 | -3,138626879 | 0,20788559 | |
| 211427_s_at | KCNJ13 | -2,930741289 | -3,138626879 | 0,20788559 | |
| 230804_at | NKAPL | -2,930741289 | -3,138626879 | 0,20788559 | |
| 233697_at | - | -2,930741289 | -3,138626879 | 0,20788559 | |
| 235892_at | - | -2,930741289 | -3,138626879 | 0,20788559 | |
| 1560281_a_at | TMEM95 | 0,641402394 | 0,433557298 | 0,207845096 | |
| 200049_at | KAT7 | 2,626954721 | 2,419116683 | 0,207838038 | |
| 208384_s_at | MID2 | 0,457392764 | 0,249604176 | 0,207788589 | |
| 222090_at | NDUFB2-AS1 | 0,457392764 | 0,249604176 | 0,207788589 | |
| 230186_at | TMEM136 | 0,457392764 | 0,249604176 | 0,207788589 | |
| 213224_s_at | BBIP1 | 1,726031811 | 1,518263577 | 0,207768233 | |
| 219900_s_at | ZNF446 | 1,168140757 | 0,960386719 | 0,207754038 | |
| 227319_at | KLHL36 | 3,634982121 | 3,427247355 | 0,207734767 | |
| 1554414_a_at | OSGIN2 | 2,905386372 | 2,697670403 | 0,207715969 | |
| 226947_at | GUSBP1 /// GUS | 2,107467633 | 1,899812471 | 0,207655162 | |
| 201362_at | IVNS1ABP | 3,107750584 | 2,900133867 | 0,207616717 | |
| 1556162_at | IGSF3 | 1,129248302 | 0,921731975 | 0,207516327 | |
| 202598_at | S100A13 | 3,418185797 | 3,210719429 | 0,207466368 | |
| 232931_at | SNRNP200 | 1,883312263 | 1,675891488 | 0,207420775 | |
| 235209_at | SBSPON | 2,427005622 | 2,21959457 | 0,207411052 | |
| 226127_at | ALKBH3 | 2,630440446 | 2,423075926 | 0,20736452 | |
| 218938_at | FBXL15 | 2,742596529 | 2,53523821 | 0,207358319 | |
| 208784_s_at | KLHDC3 | 2,87191078 | 2,664608955 | 0,207301826 | |
| 222027_at | NUCKS1 | 0,702292136 | 0,495031851 | 0,207260285 | |
| 224974_at | SUDS3 | 3,493810203 | 3,28656411 | 0,207246093 | |
| 225048_at | PHF10 | 2,478200152 | 2,271023131 | 0,207177021 | |
| 224823_at | MYLK | 2,342392905 | 2,135226207 | 0,207166698 | |
| 211696_x_at | HBB | 4,748093662 | 4,541024081 | 0,20706958 | |
| 242133_s_at | - | 1,360921491 | 1,153866761 | 0,20705473 | |
| 229299_at | NADKD1 | 2,213529682 | 2,006570641 | 0,206959041 | |
| 225060_at | LRP11 | 3,604290432 | 3,39737216 | 0,206918272 | |
| 238214_at | LRRC69 | 1,732533761 | 1,525647381 | 0,206886381 | |
| 226741_at | SLC12A6 | 3,123915073 | 2,917077481 | 0,206837592 | |
| 213480_at | VAMP4 | 1,649074067 | 1,442269454 | 0,206804613 | |
| 217780_at | WDR83OS | 6,455705735 | 6,248942712 | 0,206763023 | |
| 222879_s_at | POLH | 0,760715727 | 0,553993624 | 0,206722102 | |
| 214481_at | HIST1H2AM | 2,272723257 | 2,066015817 | 0,20670744 | |
| 212176_at | PNISR | 4,017604437 | 3,810956512 | 0,206647925 | |
| 65630_at | TMEM80 | 1,483555976 | 1,2771032 | 0,206452776 | |
| 226460_at | FNIP2 | 0,962260738 | 0,755840599 | 0,206420139 | |
| 1556988_s_at | CHD1L | 2,890927111 | 2,684536601 | 0,206390509 | |
| 205950_s_at | CA1 | 6,31414113 | 6,107799886 | 0,206341244 | |
| 209399_at | HLCS | 1,139070151 | 0,932882274 | 0,206187877 | |
| 215063_x_at | LRRC40 | 1,139070151 | 0,932882274 | 0,206187877 | |
| 212576_at | MGRN1 | 2,764969948 | 2,558821855 | 0,206148093 | |
| 202534_x_at | DHFR | 4,124054951 | 3,917937882 | 0,206117069 | |
| 225611_at | MAST4 | 3,043834719 | 2,837730973 | 0,206103746 | |
| 212726_at | PHF2 | 1,296557217 | 1,090553537 | 0,20600368 | |
| 210244_at | CAMP | 0,181384709 | -0,024572586 | 0,205957296 | |

| | | | | | |
|---|---|---|---|---|---|
| 235120_at | SEC22C | 0,181384709 | -0,024572586 | 0,205957296 | |
| 226238_at | MCEE | 1,596577845 | 1,390636111 | 0,205941734 | |
| 227878_s_at | ALKBH7 | 2,726400382 | 2,520531219 | 0,205869163 | |
| 1558407_at | PLEKHG2 | 2,05655332 | 1,85068474 | 0,20586858 | |
| 218738_s_at | RNF138 | 4,61586603 | 4,410008274 | 0,205857757 | |
| 224935_at | EIF2S3 | 5,869623654 | 5,663790168 | 0,205833486 | |
| 1555371_at | ABCB5 | 0,334552598 | 0,128747141 | 0,205805458 | |
| 202039_at | MYO18A /// TIAF | 0,334552598 | 0,128747141 | 0,205805458 | |
| 214253_s_at | DTNB | 0,334552598 | 0,128747141 | 0,205805458 | |
| 219891_at | PGPEP1 | 0,334552598 | 0,128747141 | 0,205805458 | |
| 214446_at | ELL2 | 1,261018186 | 1,055274826 | 0,20574336 | |
| 209547_s_at | SUGP1 | 2,536956583 | 2,331254065 | 0,205702519 | |
| 53991_at | DENND2A | -1,973053164 | -2,178670297 | 0,205617133 | |
| 204656_at | SHB | 1,406361373 | 1,200746069 | 0,205615304 | |
| 201177_s_at | UBA2 | 6,568916125 | 6,363338863 | 0,205577263 | |
| 203276_at | LMNB1 | 4,149180318 | 3,943660335 | 0,205519983 | |
| 222477_s_at | TM7SF3 | 3,381421359 | 3,175947691 | 0,205473668 | |
| 211549_s_at | HPGD | 1,897847661 | 1,692388143 | 0,205459518 | |
| 207776_s_at | CACNB2 | -0,078265071 | -0,283667828 | 0,205402756 | |
| 210205_at | B3GALT4 | -0,078265071 | -0,283667828 | 0,205402756 | |
| 1570057_x_at | - | -0,706943342 | -0,912331589 | 0,205388247 | |
| 207816_at | LALBA | -0,706943342 | -0,912331589 | 0,205388247 | |
| 212384_at | ATP6V1G2-DDX3 | -0,706943342 | -0,912331589 | 0,205388247 | |
| 220126_at | PRSS50 | -0,706943342 | -0,912331589 | 0,205388247 | |
| 226706_at | FLJ23867 /// QSO | -0,706943342 | -0,912331589 | 0,205388247 | |
| 232082_x_at | SPRR3 | -0,706943342 | -0,912331589 | 0,205388247 | |
| 233935_at | TNFSF14 | -0,706943342 | -0,912331589 | 0,205388247 | |
| 237653_at | - | -0,706943342 | -0,912331589 | 0,205388247 | |
| 238280_at | CYB5RL | -0,706943342 | -0,912331589 | 0,205388247 | |
| 239414_at | - | -0,706943342 | -0,912331589 | 0,205388247 | |
| 244475_at | - | -0,706943342 | -0,912331589 | 0,205388247 | |
| 226315_at | ZNF830 | 2,458724341 | 2,253342112 | 0,205382228 | |
| 235089_at | FBXL20 | 1,373456798 | 1,16809119 | 0,205365608 | |
| 232237_at | MDGA1 | 0,103263601 | -0,102100538 | 0,205364139 | |
| 237249_at | KCNQ1OT1 | 0,103263601 | -0,102100538 | 0,205364139 | |
| 62212_at | C1orf50 /// LOC1 | 2,082235068 | 1,876901783 | 0,205333285 | |
| 219510_at | POLQ | 2,716594694 | 2,511262629 | 0,205332065 | |
| 1553765_a_at | KLHL32 | -2,972500237 | -3,177802286 | 0,20530205 | |
| 1556366_s_at | LY86-AS1 | -2,972500237 | -3,177802286 | 0,20530205 | |
| 1563414_at | - | -2,972500237 | -3,177802286 | 0,20530205 | |
| 1564294_at | - | -2,972500237 | -3,177802286 | 0,20530205 | |
| 1564950_at | - | -2,972500237 | -3,177802286 | 0,20530205 | |
| 1567706_at | - | -2,972500237 | -3,177802286 | 0,20530205 | |
| 217709_at | - | -2,972500237 | -3,177802286 | 0,20530205 | |
| 220724_at | CWH43 | -2,972500237 | -3,177802286 | 0,20530205 | |
| 241764_at | - | -2,972500237 | -3,177802286 | 0,20530205 | |
| 213587_s_at | ATP6V0E2 | 3,082523011 | 2,877228323 | 0,205294688 | |
| 229420_at | - | 3,689469311 | 3,484256382 | 0,205212929 | |
| 229352_at | SPESP1 | 2,540666392 | 2,335461563 | 0,205204828 | |
| 212867_at | NCOA2 | 3,375201825 | 3,170070041 | 0,205131784 | |
| 214349_at | CTCF | 0,541261317 | 0,336136178 | 0,205125139 | |
| 1555694_a_at | KCNIP3 | -0,285837228 | -0,490927335 | 0,205090107 | |
| 207209_at | CETN1 | -0,285837228 | -0,490927335 | 0,205090107 | |
| 211374_x_at | - | -0,285837228 | -0,490927335 | 0,205090107 | |
| 215341_at | DNAH6 | -0,285837228 | -0,490927335 | 0,205090107 | |
| 230899_at | SETD4 | -0,285837228 | -0,490927335 | 0,205090107 | |

| | | | | | |
|---|---|---|---|---|---|
| 242767_at | - | -0,285837228 | -0,490927335 | 0,205090107 | |
| 221770_at | LOC729020 /// RI | 3,188003814 | 2,982929462 | 0,205074351 | |
| 1559629_at | - | 0,661985843 | 0,456918297 | 0,205067545 | |
| 220078_at | USP48 | 0,661985843 | 0,456918297 | 0,205067545 | |
| 230871_at | DHX30 | 0,661985843 | 0,456918297 | 0,205067545 | |
| 1566989_at | ARID1B | 2,303618506 | 2,098562556 | 0,20505595 | |
| 225641_at | MEF2D | 0,97328325 | 0,768343791 | 0,20493946 | |
| 208740_at | SAP18 | 2,663141434 | 2,458228496 | 0,204912938 | |
| 218048_at | COMMD3 | 3,747601714 | 3,542741726 | 0,204859987 | |
| 211121_s_at | DOK1 | 1,575032557 | 1,370274469 | 0,204758087 | |
| 204822_at | TTK | 4,05440026 | 3,849713445 | 0,204686815 | |
| 1555360_a_at | DNAJC11 | 2,288253583 | 2,083632167 | 0,204621416 | |
| 235786_at | - | 0,82905153 | 0,624460759 | 0,204590771 | |
| 221881_s_at | CLIC4 | 0,928678725 | 0,724100169 | 0,204578556 | |
| 205961_s_at | PSIP1 | 2,842156496 | 2,637603218 | 0,204553278 | |
| 203087_s_at | KIF2A | 4,705350332 | 4,500847519 | 0,204502814 | |
| 226815_at | TAMM41 | 1,829737795 | 1,6252386 | 0,204499195 | |
| 215708_s_at | LOC100653079 / | 2,830080806 | 2,625627364 | 0,204453442 | |
| 225131_at | ZRANB1 | 2,931053371 | 2,726791458 | 0,204261913 | |
| 218415_at | VPS33B | 1,381753566 | 1,177496821 | 0,204256746 | |
| 225683_x_at | PHPT1 | 1,381753566 | 1,177496821 | 0,204256746 | |
| 227875_at | KLHL13 | 1,381753566 | 1,177496821 | 0,204256746 | |
| 216181_at | SYNJ2 | 1,233777334 | 1,029536893 | 0,20424044 | |
| 226201_at | DOT1L | 1,233777334 | 1,029536893 | 0,20424044 | |
| 235384_at | NUDT19 | 5,467663228 | 5,263475497 | 0,204187732 | |
| 223203_at | FAM156A /// FAM | 3,819603766 | 3,615478364 | 0,204125402 | |
| 223785_at | FANCI | 1,932146506 | 1,728030156 | 0,204116349 | |
| 36742_at | TRIM15 | 0,273446181 | 0,069360242 | 0,204085939 | |
| 218582_at | 05.03.15 | 3,733084098 | 3,529030171 | 0,204053927 | |
| 218170_at | ISOC1 | 4,273659132 | 4,06960609 | 0,204053042 | |
| 1562474_at | - | 1,485482016 | 1,281471383 | 0,204010632 | |
| 229255_x_at | - | 2,134723057 | 1,930732138 | 0,203990919 | |
| 209648_x_at | SOCS5 | 1,348276312 | 1,144305291 | 0,203971021 | |
| 221903_s_at | CYLD | 1,348276312 | 1,144305291 | 0,203971021 | |
| 211954_s_at | IPO5 | 5,218918276 | 5,014984123 | 0,203934154 | |
| 1554479_a_at | CARD8 | 3,998848507 | 3,795004675 | 0,203843832 | |
| 1553310_at | PLB1 | -2,64318869 | -2,847001814 | 0,203813125 | |
| 1554530_at | VSTM2A | -2,64318869 | -2,847001814 | 0,203813125 | |
| 1557129_a_at | FAM111B | -2,64318869 | -2,847001814 | 0,203813125 | |
| 1562152_at | - | -2,64318869 | -2,847001814 | 0,203813125 | |
| 1563637_at | LOC729652 | -2,64318869 | -2,847001814 | 0,203813125 | |
| 1564152_at | FLJ35816 | -2,64318869 | -2,847001814 | 0,203813125 | |
| 1566126_at | - | -2,64318869 | -2,847001814 | 0,203813125 | |
| 1566441_at | - | -2,64318869 | -2,847001814 | 0,203813125 | |
| 1566881_at | - | -2,64318869 | -2,847001814 | 0,203813125 | |
| 1566896_at | - | -2,64318869 | -2,847001814 | 0,203813125 | |
| 1566966_at | - | -2,64318869 | -2,847001814 | 0,203813125 | |
| 1567376_at | DNAH1 | -2,64318869 | -2,847001814 | 0,203813125 | |
| 1570248_at | - | -2,64318869 | -2,847001814 | 0,203813125 | |
| 1570308_at | - | -2,64318869 | -2,847001814 | 0,203813125 | |
| 204414_at | LARGE | -2,64318869 | -2,847001814 | 0,203813125 | |
| 205951_at | MYH1 | -2,64318869 | -2,847001814 | 0,203813125 | |
| 208231_at | NRG1 | -2,64318869 | -2,847001814 | 0,203813125 | |
| 208283_at | GAGE1 | -2,64318869 | -2,847001814 | 0,203813125 | |
| 211533_at | PDGFRA | -2,64318869 | -2,847001814 | 0,203813125 | |
| 232712_at | - | -2,64318869 | -2,847001814 | 0,203813125 | |

| | | | | | |
|---|---|---|---|---|---|
| 240755_at | - | -2,64318869 | -2,847001814 | 0,203813125 | |
| 241824_at | - | -2,64318869 | -2,847001814 | 0,203813125 | |
| 242009_at | SLC6A4 | -2,64318869 | -2,847001814 | 0,203813125 | |
| 242169_at | BHMT2 | -2,64318869 | -2,847001814 | 0,203813125 | |
| 243127_x_at | DNASE1 | -2,64318869 | -2,847001814 | 0,203813125 | |
| 244849_at | SEMA3A | -2,64318869 | -2,847001814 | 0,203813125 | |
| 211989_at | SMARCE1 | 3,873548912 | 3,669805237 | 0,203743674 | |
| 225583_at | UXS1 | 2,294858597 | 2,091116676 | 0,203741922 | |
| 215891_s_at | GM2A | 1,835789711 | 1,632095957 | 0,203693754 | |
| 1555129_at | - | -1,173767127 | -1,377419394 | 0,203652267 | |
| 206353_at | COX6A2 | -1,173767127 | -1,377419394 | 0,203652267 | |
| 207311_at | DOC2B | -1,173767127 | -1,377419394 | 0,203652267 | |
| 208211_s_at | ALK | -1,173767127 | -1,377419394 | 0,203652267 | |
| 209978_s_at | LPA /// PLG | -1,173767127 | -1,377419394 | 0,203652267 | |
| 212468_at | SPAG9 | -1,173767127 | -1,377419394 | 0,203652267 | |
| 215276_at | WFDC8 | -1,173767127 | -1,377419394 | 0,203652267 | |
| 217260_x_at | IGHG1 /// SKAP2 | -1,173767127 | -1,377419394 | 0,203652267 | |
| 223987_at | CHRDL2 | -1,173767127 | -1,377419394 | 0,203652267 | |
| 224082_at | - | -1,173767127 | -1,377419394 | 0,203652267 | |
| 236909_at | C2orf77 | -1,173767127 | -1,377419394 | 0,203652267 | |
| 237727_at | - | -1,173767127 | -1,377419394 | 0,203652267 | |
| 238233_at | - | -1,173767127 | -1,377419394 | 0,203652267 | |
| 238476_at | CREBRF | -1,173767127 | -1,377419394 | 0,203652267 | |
| 239067_s_at | PANX2 | -1,173767127 | -1,377419394 | 0,203652267 | |
| 239144_at | B3GAT2 | -1,173767127 | -1,377419394 | 0,203652267 | |
| 244201_at | - | -1,173767127 | -1,377419394 | 0,203652267 | |
| 218889_at | NOC3L | 4,863485294 | 4,659868459 | 0,203616836 | |
| 212485_at | GPATCH8 | 2,314494121 | 2,110887591 | 0,20360653 | |
| 202161_at | PKN1 | 1,912238072 | 1,708698295 | 0,203539777 | |
| 36936_at | TSTA3 | 4,358603002 | 4,155107399 | 0,203495603 | |
| 226106_at | RNF141 | 2,030406107 | 1,826973888 | 0,203432219 | |
| 202392_s_at | PISD | 2,456762221 | 2,253342112 | 0,203420109 | |
| 213753_x_at | EIF5A | 4,959281929 | 4,755995187 | 0,203286742 | |
| 200934_at | DEK | 6,137695156 | 5,934445852 | 0,203249304 | |
| 1554423_a_at | FBXO7 | 5,077002274 | 4,873861722 | 0,203140552 | |
| 211395_x_at | FCGR2C | 2,705069849 | 2,501934108 | 0,203135741 | |
| 223475_at | CRISPLD1 | 2,077135162 | 1,874012195 | 0,203122967 | |
| 226883_at | - | 3,981211211 | 3,778101865 | 0,203109346 | |
| 229730_at | SMTNL2 | 0,425644434 | 0,222599095 | 0,203045339 | |
| 222154_s_at | SPATS2L | 5,286482665 | 5,083502044 | 0,202980621 | |
| 212808_at | NFATC2IP | 1,079110292 | 0,876248481 | 0,202861811 | |
| 201571_s_at | DCTD | 3,597182555 | 3,394350331 | 0,202832224 | |
| 206108_s_at | SRSF6 | 2,392522438 | 2,189694216 | 0,202828222 | |
| 203755_at | BUB1B | 4,586556157 | 4,383838717 | 0,20271744 | |
| 1556322_a_at | - | 0,123193849 | -0,079522948 | 0,202716798 | |
| 209546_s_at | APOL1 | 0,123193849 | -0,079522948 | 0,202716798 | |
| 216499_at | - | 0,123193849 | -0,079522948 | 0,202716798 | |
| 222675_s_at | BAIAP2L1 | 0,123193849 | -0,079522948 | 0,202716798 | |
| 233103_at | - | 0,123193849 | -0,079522948 | 0,202716798 | |
| 235217_at | LOC100216546 | 0,123193849 | -0,079522948 | 0,202716798 | |
| 213970_at | RABL3 | 1,462198073 | 1,259496994 | 0,202701079 | |
| 230566_at | MORC2-AS1 | 1,462198073 | 1,259496994 | 0,202701079 | |
| 205089_at | ZNF7 | 3,294008537 | 3,091398163 | 0,202610374 | |
| 227035_x_at | RP9P | 3,102740237 | 2,900133867 | 0,20260637 | |
| 219077_s_at | WWOX | 0,496120222 | 0,293518836 | 0,202601386 | |
| 223248_at | HSDL1 | 2,429008604 | 2,226407528 | 0,202601076 | |

| | | | | | |
|---|---|---|---|---|---|
| 227613_at | ZNF331 | 1,894952274 | 1,692388143 | 0,202564131 | |
| 216600_x_at | ALDOB | 0,792179257 | 0,589657387 | 0,20252187 | |
| 242151_at | - | 0,792179257 | 0,589657387 | 0,20252187 | |
| 201273_s_at | SRP9 | 6,935233779 | 6,732724641 | 0,202509138 | |
| 223376_s_at | BRI3 | 4,026230348 | 3,823741953 | 0,202488395 | |
| 1560074_at | PRKCA | -0,055687482 | -0,258114234 | 0,202426752 | |
| 1560472_at | LOC338588 | -0,055687482 | -0,258114234 | 0,202426752 | |
| 203413_at | LOC100653018 / | -0,055687482 | -0,258114234 | 0,202426752 | |
| 204015_s_at | DUSP4 | -0,055687482 | -0,258114234 | 0,202426752 | |
| 208430_s_at | DTNA | -0,055687482 | -0,258114234 | 0,202426752 | |
| 209701_at | ERAP1 | -0,055687482 | -0,258114234 | 0,202426752 | |
| 237451_x_at | - | -0,055687482 | -0,258114234 | 0,202426752 | |
| 1553679_s_at | VKORC1L1 | 0,359971644 | 0,157549243 | 0,202422401 | |
| 208791_at | CLU | 0,682279745 | 0,479907041 | 0,202372704 | |
| 50965_at | RAB26 | -0,141207927 | -0,343571477 | 0,20236355 | |
| 213386_at | TMEM246 | 2,208874124 | 2,006570641 | 0,202303483 | |
| 220060_s_at | PARPBP | 2,061726373 | 1,859476797 | 0,202249576 | |
| 202523_s_at | SPOCK2 | 0,209623097 | 0,007421914 | 0,202201183 | |
| 218565_at | C9orf114 | 0,209623097 | 0,007421914 | 0,202201183 | |
| 227901_at | LOC648987 | 0,209623097 | 0,007421914 | 0,202201183 | |
| 203790_s_at | HRSP12 | 4,73358102 | 4,531428237 | 0,202152783 | |
| 212826_s_at | SLC25A6 | 6,084046602 | 5,881925611 | 0,202120991 | |
| 225823_at | C19orf70 | 3,684447666 | 3,482360311 | 0,202087355 | |
| 212244_at | GCOM1 /// POLR | 2,713311265 | 2,511262629 | 0,202048636 | |
| 210891_s_at | GTF2I /// GTF2IP | 5,18316972 | 4,981144321 | 0,2020254 | |
| 225406_at | TWSG1 | 4,176858496 | 3,975013041 | 0,201845455 | |
| 1557848_at | - | -0,259832361 | -0,461594427 | 0,201762065 | |
| 202998_s_at | LOXL2 | -0,259832361 | -0,461594427 | 0,201762065 | |
| 222217_s_at | SLC27A3 | -0,259832361 | -0,461594427 | 0,201762065 | |
| 224798_s_at | FAM219B | -0,259832361 | -0,461594427 | 0,201762065 | |
| 227804_at | TLCD1 | -0,259832361 | -0,461594427 | 0,201762065 | |
| 228610_at | TM9SF3 | -0,259832361 | -0,461594427 | 0,201762065 | |
| 230450_at | - | -0,259832361 | -0,461594427 | 0,201762065 | |
| 236244_at | - | -0,259832361 | -0,461594427 | 0,201762065 | |
| 1554271_a_at | CENPL | 2,213529682 | 2,011838132 | 0,20169155 | |
| 224622_at | TBC1D14 | 3,262904562 | 3,061230246 | 0,201674316 | |
| 209870_s_at | APBA2 | 2,109966786 | 1,908311039 | 0,201655747 | |
| 201445_at | CNN3 | 3,902669169 | 3,701119714 | 0,201549454 | |
| 219496_at | SOWAHC | 1,504602299 | 1,303116084 | 0,201486214 | |
| 230212_at | SPRY1 | 1,504602299 | 1,303116084 | 0,201486214 | |
| 203819_s_at | IGF2BP3 | 2,254767157 | 2,053299695 | 0,201467462 | |
| 224719_s_at | C12orf57 | 4,641572531 | 4,440114107 | 0,201458424 | |
| 236497_at | LOC729683 | 1,215326747 | 1,013870809 | 0,201455938 | |
| 212369_at | ZNF384 | 2,890927111 | 2,689475799 | 0,201451312 | |
| 225885_at | EEA1 | 2,630440446 | 2,428994491 | 0,201445955 | |
| 226752_at | FAM174A | 2,787001691 | 2,585563171 | 0,201438519 | |
| 212461_at | AZIN1 | 5,147729547 | 4,946319737 | 0,20140981 | |
| 1556643_at | LOC100507535 | 1,331241794 | 1,129843209 | 0,201398585 | |
| 1552540_s_at | IQCD | -0,148196756 | -0,34958478 | 0,201388024 | |
| 1553883_at | ZNF99 | -0,148196756 | -0,34958478 | 0,201388024 | |
| 204323_x_at | NF1 | -0,148196756 | -0,34958478 | 0,201388024 | |
| 236463_at | ADAD2 | -0,148196756 | -0,34958478 | 0,201388024 | |
| 243178_at | - | -0,148196756 | -0,34958478 | 0,201388024 | |
| 1558304_s_at | TSEN54 | 2,600539826 | 2,39915583 | 0,201383996 | |
| 209009_at | ESD | 5,439623891 | 5,238417317 | 0,201206574 | |
| 243372_at | HSPD1 | 1,369290453 | 1,16809119 | 0,201199262 | |

| | | | | | |
|---|---|---|---|---|---|
| 218968_s_at | ZFP64 | 3,456984333 | 3,255815291 | 0,201169042 | |
| 218458_at | GMCL1 | 3,059434754 | 2,858346012 | 0,201088741 | |
| 201626_at | INSIG1 | 3,96128107 | 3,760216412 | 0,201064658 | |
| 1558507_at | C1orf53 | 1,177702228 | 0,976641161 | 0,201061067 | |
| 204679_at | KCNK1 | 1,177702228 | 0,976641161 | 0,201061067 | |
| 228708_at | RAB27B | 7,312462139 | 7,111425501 | 0,201036638 | |
| 1552491_at | IDI2 | -1,131062212 | -1,332017329 | 0,200955117 | |
| 1555124_at | LOC100129726 | -1,131062212 | -1,332017329 | 0,200955117 | |
| 214996_at | - | -1,131062212 | -1,332017329 | 0,200955117 | |
| 216788_at | - | -1,131062212 | -1,332017329 | 0,200955117 | |
| 219488_at | A4GALT | -1,131062212 | -1,332017329 | 0,200955117 | |
| 224119_at | - | -1,131062212 | -1,332017329 | 0,200955117 | |
| 228352_at | UNC13D | -1,131062212 | -1,332017329 | 0,200955117 | |
| 229227_at | DICER1-AS1 | -1,131062212 | -1,332017329 | 0,200955117 | |
| 238809_at | C5orf51 | -1,131062212 | -1,332017329 | 0,200955117 | |
| 241645_at | - | -1,131062212 | -1,332017329 | 0,200955117 | |
| 206052_s_at | SLBP | 5,382630738 | 5,181707664 | 0,200923074 | |
| 224705_s_at | TNRC6A | 3,158127685 | 2,957221307 | 0,200906379 | |
| 218234_at | ING4 | 1,094335495 | 0,893473268 | 0,200862227 | |
| 207396_s_at | ALG3 | 3,231115193 | 3,030417996 | 0,200697197 | |
| 1552946_at | ZNF114 | 0,859074518 | 0,658444279 | 0,200630239 | |
| 222065_s_at | FLII | 2,922548342 | 2,721978587 | 0,200569755 | |
| 202528_at | GALE | 1,859746653 | 1,659204017 | 0,200542636 | |
| 219698_s_at | METTL4 | 1,780386218 | 1,579853228 | 0,20053299 | |
| 1553528_a_at | TAF5 | 2,947914474 | 2,747463672 | 0,200450802 | |
| 208648_at | VCP | 3,733084098 | 3,532699346 | 0,200384751 | |
| 1552621_at | POLR2J2 /// POL | 1,446464021 | 1,246149888 | 0,200314132 | |
| 236632_at | HHIP-AS1 | 0,57782909 | 0,377530647 | 0,200298444 | |
| 220960_x_at | RPL22 | 7,224239487 | 7,023947199 | 0,200292288 | |
| 207154_at | DIO3 | 0,376672223 | 0,176436073 | 0,20023615 | |
| 216973_s_at | HOXB7 | 0,376672223 | 0,176436073 | 0,20023615 | |
| 230036_at | SAMD9L | 0,376672223 | 0,176436073 | 0,20023615 | |
| 234485_at | - | 0,376672223 | 0,176436073 | 0,20023615 | |
| 211946_s_at | PRRC2C | 5,697110194 | 5,496914678 | 0,200195516 | |
| 201418_s_at | SOX4 | 1,578645857 | 1,378453643 | 0,200192213 | |
| 222968_at | - | 3,043834719 | 2,843651104 | 0,200183615 | |
| 1565786_x_at | FLJ45482 | 1,481627361 | 1,281471383 | 0,200155978 | |
| 223348_x_at | MUM1 | 1,481627361 | 1,281471383 | 0,200155978 | |
| 234983_at | - | 3,769898242 | 3,569779975 | 0,200118267 | |
| 1552520_at | TMEM74 | -0,366776884 | -0,566887459 | 0,200110576 | |
| 232435_at | ALG13 | -0,366776884 | -0,566887459 | 0,200110576 | |
| 233381_at | RUFY1 | -0,366776884 | -0,566887459 | 0,200110576 | |
| 214499_s_at | BCLAF1 | 3,715139632 | 3,515187047 | 0,199952585 | |
| 219410_at | TMEM45A | 3,902669169 | 3,702749166 | 0,199920003 | |
| 222392_x_at | PERP | 1,305306849 | 1,105412836 | 0,199894014 | |
| 220255_at | FANCE | 1,549482847 | 1,349621332 | 0,199861515 | |
| 222665_at | FAM82B | 1,549482847 | 1,349621332 | 0,199861515 | |
| 1555792_a_at | CCDC116 | -0,655928373 | -0,855751026 | 0,199822653 | |
| 1557744_at | - | -0,655928373 | -0,855751026 | 0,199822653 | |
| 1565579_at | - | -0,655928373 | -0,855751026 | 0,199822653 | |
| 205520_at | STRN | -0,655928373 | -0,855751026 | 0,199822653 | |
| 207990_x_at | ACRV1 | -0,655928373 | -0,855751026 | 0,199822653 | |
| 208587_s_at | OR1E1 /// OR1E2 | -0,655928373 | -0,855751026 | 0,199822653 | |
| 216370_s_at | TKTL1 | -0,655928373 | -0,855751026 | 0,199822653 | |
| 220634_at | TBX4 | -0,655928373 | -0,855751026 | 0,199822653 | |
| 221364_at | GRID2 | -0,655928373 | -0,855751026 | 0,199822653 | |

| | | | | | |
|---|---|---|---|---|---|
| 231887_s_at | KIAA1274 | -0,655928373 | -0,855751026 | 0,199822653 | |
| 234462_at | - | -0,655928373 | -0,855751026 | 0,199822653 | |
| 235703_at | PLB1 | -0,655928373 | -0,855751026 | 0,199822653 | |
| 236403_at | THAP7-AS1 | -0,655928373 | -0,855751026 | 0,199822653 | |
| 238059_at | - | -0,655928373 | -0,855751026 | 0,199822653 | |
| 239538_at | ZRANB3 | 1,965648824 | 1,765933685 | 0,19971514 | |
| 1556969_at | CCDC108 | -0,85085127 | -1,050406615 | 0,199555345 | |
| 1561506_at | - | -0,85085127 | -1,050406615 | 0,199555345 | |
| 204196_x_at | PKNOX1 | -0,85085127 | -1,050406615 | 0,199555345 | |
| 208401_s_at | GLP1R | -0,85085127 | -1,050406615 | 0,199555345 | |
| 209739_s_at | PNPLA4 | -0,85085127 | -1,050406615 | 0,199555345 | |
| 209842_at | SOX10 | -0,85085127 | -1,050406615 | 0,199555345 | |
| 210619_s_at | HYAL1 | -0,85085127 | -1,050406615 | 0,199555345 | |
| 216867_s_at | PDGFA | -0,85085127 | -1,050406615 | 0,199555345 | |
| 220125_at | DNAI1 | -0,85085127 | -1,050406615 | 0,199555345 | |
| 221017_s_at | LRRC3 | -0,85085127 | -1,050406615 | 0,199555345 | |
| 223541_at | HAS3 | -0,85085127 | -1,050406615 | 0,199555345 | |
| 224498_x_at | AXIN2 | -0,85085127 | -1,050406615 | 0,199555345 | |
| 232273_at | - | -0,85085127 | -1,050406615 | 0,199555345 | |
| 237325_at | C22orf43 | -0,85085127 | -1,050406615 | 0,199555345 | |
| 238037_at | LMLN | -0,85085127 | -1,050406615 | 0,199555345 | |
| 240150_at | - | -0,85085127 | -1,050406615 | 0,199555345 | |
| 213251_at | SMARCA5 | 4,11599613 | 3,916534328 | 0,199461802 | |
| 1553207_at | ARL10 | 0,30867765 | 0,109221392 | 0,199456258 | |
| 209713_s_at | SLC35D1 | 0,30867765 | 0,109221392 | 0,199456258 | |
| 215022_x_at | ZNF33B | 0,30867765 | 0,109221392 | 0,199456258 | |
| 217314_at | - | 0,30867765 | 0,109221392 | 0,199456258 | |
| 221224_s_at | DCAKD | 0,30867765 | 0,109221392 | 0,199456258 | |
| 229737_at | FAM46A | 0,30867765 | 0,109221392 | 0,199456258 | |
| 202298_at | NDUFA1 | 5,456660079 | 5,257390237 | 0,199269842 | |
| 218259_at | MKL2 | 2,171078719 | 1,971855016 | 0,199223703 | |
| 225025_at | IGSF8 | 0,87091093 | 0,67181667 | 0,19909426 | |
| 219376_at | ZNF322 /// ZNF3 | 2,064305961 | 1,865308541 | 0,198997419 | |
| 201622_at | SND1 | 4,34540724 | 4,146452482 | 0,198954759 | |
| 204209_at | PCYT1A | 1,556829052 | 1,3579181 | 0,198910952 | |
| 222855_s_at | TRPV2 | 1,422536444 | 1,223626587 | 0,198909857 | |
| 227887_at | LOC400236 | 0,152582607 | -0,046302147 | 0,198884754 | |
| 229474_at | MICAL3 | 0,152582607 | -0,046302147 | 0,198884754 | |
| 237265_at | C16orf73 /// LINC | 3,225364068 | 3,026519779 | 0,198844289 | |
| 1559725_at | - | -3,07502285 | -3,273861381 | 0,198838531 | |
| 1561450_at | - | -3,07502285 | -3,273861381 | 0,198838531 | |
| 1562992_at | - | -3,07502285 | -3,273861381 | 0,198838531 | |
| 1568603_at | CADPS | -3,07502285 | -3,273861381 | 0,198838531 | |
| 214776_x_at | XYLB | -3,07502285 | -3,273861381 | 0,198838531 | |
| 232541_at | - | -3,07502285 | -3,273861381 | 0,198838531 | |
| 233755_at | - | -3,07502285 | -3,273861381 | 0,198838531 | |
| 234621_at | - | -3,07502285 | -3,273861381 | 0,198838531 | |
| 244567_at | - | -3,07502285 | -3,273861381 | 0,198838531 | |
| 202277_at | SPTLC1 | 5,158634383 | 4,959839678 | 0,198794705 | |
| 217847_s_at | THRAP3 | 1,314003737 | 1,115234684 | 0,198769052 | |
| 244046_at | URGCP | 1,314003737 | 1,115234684 | 0,198769052 | |
| 1554543_at | SPAG9 | -1,838812296 | -2,037551727 | 0,19873943 | |
| 1555117_at | - | -1,838812296 | -2,037551727 | 0,19873943 | |
| 1555137_a_at | FGD6 | -1,838812296 | -2,037551727 | 0,19873943 | |
| 1562288_at | - | -1,838812296 | -2,037551727 | 0,19873943 | |
| 1564310_a_at | PARP15 | -1,838812296 | -2,037551727 | 0,19873943 | |

| | | | | | |
|---|---|---|---|---|---|
| 1564471_at | LOC100130815 | -1,838812296 | -2,037551727 | 0,19873943 | |
| 1568663_a_at | PWRN2 | -1,838812296 | -2,037551727 | 0,19873943 | |
| 1569597_at | - | -1,838812296 | -2,037551727 | 0,19873943 | |
| 201496_x_at | MYH11 | -1,838812296 | -2,037551727 | 0,19873943 | |
| 205274_at | GTPBP1 | -1,838812296 | -2,037551727 | 0,19873943 | |
| 206677_at | KRT31 | -1,838812296 | -2,037551727 | 0,19873943 | |
| 207546_at | ATP4B | -1,838812296 | -2,037551727 | 0,19873943 | |
| 213482_at | DOCK3 | -1,838812296 | -2,037551727 | 0,19873943 | |
| 216393_at | C10orf12 | -1,838812296 | -2,037551727 | 0,19873943 | |
| 216470_x_at | PRSS2 | -1,838812296 | -2,037551727 | 0,19873943 | |
| 216820_at | - | -1,838812296 | -2,037551727 | 0,19873943 | |
| 219523_s_at | ODZ3 | -1,838812296 | -2,037551727 | 0,19873943 | |
| 220574_at | SEMA6D | -1,838812296 | -2,037551727 | 0,19873943 | |
| 220710_at | ANP32A-IT1 | -1,838812296 | -2,037551727 | 0,19873943 | |
| 229228_at | CREB5 /// LOC4( | -1,838812296 | -2,037551727 | 0,19873943 | |
| 230140_at | PTPN9 | -1,838812296 | -2,037551727 | 0,19873943 | |
| 236881_at | - | -1,838812296 | -2,037551727 | 0,19873943 | |
| 237063_at | - | -1,838812296 | -2,037551727 | 0,19873943 | |
| 239647_at | CHST13 | -1,838812296 | -2,037551727 | 0,19873943 | |
| 241102_at | - | -1,838812296 | -2,037551727 | 0,19873943 | |
| 241726_at | - | -1,838812296 | -2,037551727 | 0,19873943 | |
| 243002_at | - | -1,838812296 | -2,037551727 | 0,19873943 | |
| 227461_at | STON2 | 4,706589309 | 4,507854821 | 0,198734488 | |
| 1554957_at | - | -1,888254346 | -2,086984744 | 0,198730398 | |
| 1555665_at | - | -1,888254346 | -2,086984744 | 0,198730398 | |
| 1561277_at | LOC339298 | -1,888254346 | -2,086984744 | 0,198730398 | |
| 1564872_at | - | -1,888254346 | -2,086984744 | 0,198730398 | |
| 1567064_at | OR1Q1 | -1,888254346 | -2,086984744 | 0,198730398 | |
| 204455_at | DST /// LOC1006 | -1,888254346 | -2,086984744 | 0,198730398 | |
| 204750_s_at | DSC2 | -1,888254346 | -2,086984744 | 0,198730398 | |
| 204855_at | SERPINB5 | -1,888254346 | -2,086984744 | 0,198730398 | |
| 204879_at | PDPN | -1,888254346 | -2,086984744 | 0,198730398 | |
| 206334_at | LIPF | -1,888254346 | -2,086984744 | 0,198730398 | |
| 214925_s_at | SPTAN1 | -1,888254346 | -2,086984744 | 0,198730398 | |
| 219115_s_at | IL20RA | -1,888254346 | -2,086984744 | 0,198730398 | |
| 226900_at | GABPB1-AS1 | -1,888254346 | -2,086984744 | 0,198730398 | |
| 230221_at | ABHD16A | -1,888254346 | -2,086984744 | 0,198730398 | |
| 232165_at | EPPK1 | -1,888254346 | -2,086984744 | 0,198730398 | |
| 233911_s_at | PPM1H | -1,888254346 | -2,086984744 | 0,198730398 | |
| 234829_at | C20orf57 /// DUS | -1,888254346 | -2,086984744 | 0,198730398 | |
| 236158_at | - | -1,888254346 | -2,086984744 | 0,198730398 | |
| 236245_at | ODF3L1 | -1,888254346 | -2,086984744 | 0,198730398 | |
| 237914_s_at | - | -1,888254346 | -2,086984744 | 0,198730398 | |
| 239612_at | LOC100240734 | -1,888254346 | -2,086984744 | 0,198730398 | |
| 213102_at | ACTR3 | 5,511564384 | 5,312856336 | 0,198708047 | |
| 222869_s_at | ELAC1 | 1,352503698 | 1,153866761 | 0,198636937 | |
| 54051_at | PKNOX1 | 1,390002893 | 1,191491281 | 0,198511612 | |
| 1555337_a_at | ZNF317 | 0,822971223 | 0,624460759 | 0,198510464 | |
| 205231_s_at | EPM2A | 0,822971223 | 0,624460759 | 0,198510464 | |
| 1555485_s_at | FAM153B /// LOC | -1,964176657 | -2,162639771 | 0,198463115 | |
| 1556742_at | GUSBP1 | -1,964176657 | -2,162639771 | 0,198463115 | |
| 1559455_at | - | -1,964176657 | -2,162639771 | 0,198463115 | |
| 1559481_at | CHIC1 | -1,964176657 | -2,162639771 | 0,198463115 | |
| 1560554_a_at | C1orf145 | -1,964176657 | -2,162639771 | 0,198463115 | |
| 1564757_a_at | CCDC148 | -1,964176657 | -2,162639771 | 0,198463115 | |
| 210171_s_at | CREM | -1,964176657 | -2,162639771 | 0,198463115 | |

| | | | | | |
|---|---|---|---|---|---|
| 211116_at | SLC9A2 | -1,964176657 | -2,162639771 | 0,198463115 | |
| 215408_at | - | -1,964176657 | -2,162639771 | 0,198463115 | |
| 215835_at | LOC100653174 | -1,964176657 | -2,162639771 | 0,198463115 | |
| 216553_x_at | - | -1,964176657 | -2,162639771 | 0,198463115 | |
| 222258_s_at | SH3BP4 | -1,964176657 | -2,162639771 | 0,198463115 | |
| 222501_s_at | REPIN1 | -1,964176657 | -2,162639771 | 0,198463115 | |
| 228467_at | PURB | -1,964176657 | -2,162639771 | 0,198463115 | |
| 229127_at | JAM2 | -1,964176657 | -2,162639771 | 0,198463115 | |
| 229756_at | - | -1,964176657 | -2,162639771 | 0,198463115 | |
| 230162_s_at | MAPK8IP3 | -1,964176657 | -2,162639771 | 0,198463115 | |
| 230585_at | - | -1,964176657 | -2,162639771 | 0,198463115 | |
| 231392_at | - | -1,964176657 | -2,162639771 | 0,198463115 | |
| 233335_at | - | -1,964176657 | -2,162639771 | 0,198463115 | |
| 237698_at | - | -1,964176657 | -2,162639771 | 0,198463115 | |
| 237976_at | - | -1,964176657 | -2,162639771 | 0,198463115 | |
| 240881_at | - | -1,964176657 | -2,162639771 | 0,198463115 | |
| 241048_at | - | -1,964176657 | -2,162639771 | 0,198463115 | |
| 244719_at | - | -1,964176657 | -2,162639771 | 0,198463115 | |
| 244819_x_at | PSPH | -1,964176657 | -2,162639771 | 0,198463115 | |
| 209940_at | PARP3 | 0,592200562 | 0,393761504 | 0,198439057 | |
| 209965_s_at | RAD51D | 0,592200562 | 0,393761504 | 0,198439057 | |
| 231200_at | LSM14B | 0,592200562 | 0,393761504 | 0,198439057 | |
| 206550_s_at | NUP155 | 3,426229985 | 3,227795991 | 0,198433995 | |
| 224683_at | FBXO18 | 2,156646206 | 1,958277216 | 0,19836899 | |
| 227685_at | TMF1 | 2,156646206 | 1,958277216 | 0,19836899 | |
| 1553467_at | FLJ32742 | -1,763254337 | -1,961547147 | 0,19829281 | |
| 1561014_at | - | -1,763254337 | -1,961547147 | 0,19829281 | |
| 1566248_at | - | -1,763254337 | -1,961547147 | 0,19829281 | |
| 1569108_a_at | ZNF589 | -1,763254337 | -1,961547147 | 0,19829281 | |
| 205473_at | ATP6V1B1 | -1,763254337 | -1,961547147 | 0,19829281 | |
| 210302_s_at | MAB21L2 | -1,763254337 | -1,961547147 | 0,19829281 | |
| 211844_s_at | NRP2 | -1,763254337 | -1,961547147 | 0,19829281 | |
| 213456_at | SOSTDC1 | -1,763254337 | -1,961547147 | 0,19829281 | |
| 226129_at | FAM83H | -1,763254337 | -1,961547147 | 0,19829281 | |
| 228501_at | GALNTL2 | -1,763254337 | -1,961547147 | 0,19829281 | |
| 234071_at | DEPTOR | -1,763254337 | -1,961547147 | 0,19829281 | |
| 236604_at | BAHCC1 | -1,763254337 | -1,961547147 | 0,19829281 | |
| 238780_s_at | - | -1,763254337 | -1,961547147 | 0,19829281 | |
| 239465_at | - | -1,763254337 | -1,961547147 | 0,19829281 | |
| 240415_at | - | -1,763254337 | -1,961547147 | 0,19829281 | |
| 242126_at | - | -1,763254337 | -1,961547147 | 0,19829281 | |
| 244518_at | LOC100130452 | -1,763254337 | -1,961547147 | 0,19829281 | |
| 201193_at | IDH1 | 3,964737003 | 3,766462619 | 0,198274384 | |
| 215146_s_at | TTC28 | 2,410880861 | 2,212749286 | 0,198131575 | |
| 1563878_a_at | LOC338963 | -0,022466681 | -0,220592223 | 0,198125543 | |
| 202449_s_at | RXRA | -0,022466681 | -0,220592223 | 0,198125543 | |
| 210232_at | CDC42 | -0,022466681 | -0,220592223 | 0,198125543 | |
| 210743_s_at | CDC14A | -0,022466681 | -0,220592223 | 0,198125543 | |
| 221095_s_at | KCNE2 | -0,022466681 | -0,220592223 | 0,198125543 | |
| 221136_at | GDF2 | -0,022466681 | -0,220592223 | 0,198125543 | |
| 223668_at | CBLC | -0,022466681 | -0,220592223 | 0,198125543 | |
| 228398_at | SENP8 | -0,022466681 | -0,220592223 | 0,198125543 | |
| 241825_at | TYW5 | -0,022466681 | -0,220592223 | 0,198125543 | |
| 1553938_a_at | STK32A | -2,756478458 | -2,954576755 | 0,198098297 | |
| 1553965_x_at | RHOB | -2,756478458 | -2,954576755 | 0,198098297 | |
| 1554547_at | FAM13C | -2,756478458 | -2,954576755 | 0,198098297 | |

| | | | | | |
|---|---|---|---|---|---|
| 1555166_a_at | ZNF396 | -2,756478458 | -2,954576755 | 0,198098297 | |
| 1556147_at | - | -2,756478458 | -2,954576755 | 0,198098297 | |
| 1556770_a_at | FBXL13 | -2,756478458 | -2,954576755 | 0,198098297 | |
| 1557604_at | LOC401312 | -2,756478458 | -2,954576755 | 0,198098297 | |
| 1559289_at | TADA1 | -2,756478458 | -2,954576755 | 0,198098297 | |
| 1559856_s_at | MLL | -2,756478458 | -2,954576755 | 0,198098297 | |
| 1561488_at | - | -2,756478458 | -2,954576755 | 0,198098297 | |
| 1566219_at | LOC338651 | -2,756478458 | -2,954576755 | 0,198098297 | |
| 1567377_at | DNAH1 | -2,756478458 | -2,954576755 | 0,198098297 | |
| 1568752_s_at | RGS13 | -2,756478458 | -2,954576755 | 0,198098297 | |
| 1570043_at | - | -2,756478458 | -2,954576755 | 0,198098297 | |
| 206370_at | PIK3CG | -2,756478458 | -2,954576755 | 0,198098297 | |
| 210067_at | AQP4 | -2,756478458 | -2,954576755 | 0,198098297 | |
| 217213_at | SLC6A2 | -2,756478458 | -2,954576755 | 0,198098297 | |
| 220889_s_at | CA10 | -2,756478458 | -2,954576755 | 0,198098297 | |
| 220900_at | - | -2,756478458 | -2,954576755 | 0,198098297 | |
| 232073_at | PPFIA2 | -2,756478458 | -2,954576755 | 0,198098297 | |
| 232568_at | MGC24103 | -2,756478458 | -2,954576755 | 0,198098297 | |
| 236871_s_at | IQCF3 /// IQCF4 | -2,756478458 | -2,954576755 | 0,198098297 | |
| 240208_at | - | -2,756478458 | -2,954576755 | 0,198098297 | |
| 240366_at | LHFPL3-AS1 | -2,756478458 | -2,954576755 | 0,198098297 | |
| 241101_at | - | -2,756478458 | -2,954576755 | 0,198098297 | |
| 241655_at | - | -2,756478458 | -2,954576755 | 0,198098297 | |
| 241951_at | SLCO1C1 | -2,756478458 | -2,954576755 | 0,198098297 | |
| 242789_at | PDE1A | -2,756478458 | -2,954576755 | 0,198098297 | |
| 243862_at | RASEF | -2,756478458 | -2,954576755 | 0,198098297 | |
| 1554496_at | RAD51B | -2,01371626 | -2,211750327 | 0,198034067 | |
| 1559252_a_at | - | -2,01371626 | -2,211750327 | 0,198034067 | |
| 1562368_at | CARD11 | -2,01371626 | -2,211750327 | 0,198034067 | |
| 1563635_at | - | -2,01371626 | -2,211750327 | 0,198034067 | |
| 206034_at | SERPINB8 | -2,01371626 | -2,211750327 | 0,198034067 | |
| 206556_at | CLUL1 | -2,01371626 | -2,211750327 | 0,198034067 | |
| 211488_s_at | ITGB8 | -2,01371626 | -2,211750327 | 0,198034067 | |
| 213816_s_at | MET | -2,01371626 | -2,211750327 | 0,198034067 | |
| 214725_at | SBSPON | -2,01371626 | -2,211750327 | 0,198034067 | |
| 216201_at | - | -2,01371626 | -2,211750327 | 0,198034067 | |
| 220876_at | - | -2,01371626 | -2,211750327 | 0,198034067 | |
| 221033_s_at | RNF17 | -2,01371626 | -2,211750327 | 0,198034067 | |
| 227398_s_at | MIDN | -2,01371626 | -2,211750327 | 0,198034067 | |
| 228858_at | - | -2,01371626 | -2,211750327 | 0,198034067 | |
| 233613_x_at | REXO2 | -2,01371626 | -2,211750327 | 0,198034067 | |
| 234522_at | - | -2,01371626 | -2,211750327 | 0,198034067 | |
| 237511_at | - | -2,01371626 | -2,211750327 | 0,198034067 | |
| 237959_at | - | -2,01371626 | -2,211750327 | 0,198034067 | |
| 238577_s_at | TSHZ2 | -2,01371626 | -2,211750327 | 0,198034067 | |
| 239745_at | - | -2,01371626 | -2,211750327 | 0,198034067 | |
| 240267_at | - | -2,01371626 | -2,211750327 | 0,198034067 | |
| 241324_at | - | -2,01371626 | -2,211750327 | 0,198034067 | |
| 243611_at | MICALCL | -2,01371626 | -2,211750327 | 0,198034067 | |
| 244073_at | - | -2,01371626 | -2,211750327 | 0,198034067 | |
| 206854_s_at | MAP3K7 | 4,127142551 | 3,92911743 | 0,198025121 | |
| 222251_s_at | GMEB2 | 2,446911392 | 2,248887791 | 0,198023601 | |
| 1553564_at | MACROD2 | 0,46522187 | 0,26733076 | 0,197891111 | |
| 213595_s_at | CDC42BPA | 0,46522187 | 0,26733076 | 0,197891111 | |
| 1558481_s_at | LOC100507032 | -1,71423189 | -1,912089812 | 0,197857922 | |
| 1558660_at | LOC100507059 | -1,71423189 | -1,912089812 | 0,197857922 | |

| | | | | | |
|---|---|---|---|---|---|
| 1559692_at | - | -1,71423189 | -1,912089812 | 0,197857922 | |
| 1564211_at | C14orf64 | -1,71423189 | -1,912089812 | 0,197857922 | |
| 1566500_at | - | -1,71423189 | -1,912089812 | 0,197857922 | |
| 207456_at | HNF4G | -1,71423189 | -1,912089812 | 0,197857922 | |
| 215635_at | - | -1,71423189 | -1,912089812 | 0,197857922 | |
| 219759_at | ERAP2 | -1,71423189 | -1,912089812 | 0,197857922 | |
| 228079_at | C3orf58 | -1,71423189 | -1,912089812 | 0,197857922 | |
| 229261_at | - | -1,71423189 | -1,912089812 | 0,197857922 | |
| 230287_at | SGSM1 | -1,71423189 | -1,912089812 | 0,197857922 | |
| 234874_at | ATE1 | -1,71423189 | -1,912089812 | 0,197857922 | |
| 235150_at | SESN3 | -1,71423189 | -1,912089812 | 0,197857922 | |
| 237090_at | LOC100506113 | -1,71423189 | -1,912089812 | 0,197857922 | |
| 237115_at | - | -1,71423189 | -1,912089812 | 0,197857922 | |
| 237889_s_at | LOC100422737 | -1,71423189 | -1,912089812 | 0,197857922 | |
| 238930_at | PPM1N | -1,71423189 | -1,912089812 | 0,197857922 | |
| 240236_at | STXBP5L | -1,71423189 | -1,912089812 | 0,197857922 | |
| 240788_at | - | -1,71423189 | -1,912089812 | 0,197857922 | |
| 240790_at | - | -1,71423189 | -1,912089812 | 0,197857922 | |
| 218732_at | PTRH2 | 4,238115661 | 4,040263518 | 0,197852143 | |
| 214083_at | PPP2R5C | 1,073999308 | 0,876248481 | 0,197750827 | |
| 225130_at | ZRANB1 | 1,073999308 | 0,876248481 | 0,197750827 | |
| 235677_at | SRR | 1,073999308 | 0,876248481 | 0,197750827 | |
| 242978_x_at | - | 1,932146506 | 1,73441697 | 0,197729535 | |
| 1554047_at | TXNDC9 | 0,533835206 | 0,336136178 | 0,197699029 | |
| 225246_at | STIM2 | 0,533835206 | 0,336136178 | 0,197699029 | |
| 232079_s_at | PVRL2 | 0,533835206 | 0,336136178 | 0,197699029 | |
| 236588_at | CFDP1 | 0,533835206 | 0,336136178 | 0,197699029 | |
| 209406_at | BAG2 | 4,141261013 | 3,943660335 | 0,197600678 | |
| 243737_at | ATP1B4 | 2,11992033 | 1,922365006 | 0,197555323 | |
| 226283_at | POC1B | 1,567778694 | 1,370274469 | 0,197504224 | |
| 231850_x_at | CEP44 | 1,567778694 | 1,370274469 | 0,197504224 | |
| 202942_at | ETFB | 3,245961699 | 3,048471686 | 0,197490013 | |
| 204610_s_at | CCDC85B | 2,646022818 | 2,448549499 | 0,19747332 | |
| 227853_at | - | 2,16388051 | 1,966439221 | 0,197441289 | |
| 200883_at | UQCRC2 | 4,895888063 | 4,698764458 | 0,197123605 | |
| 1559877_at | LOC100289094 | -2,089474779 | -2,286583451 | 0,197108671 | |
| 1560715_at | P4HA3 | -2,089474779 | -2,286583451 | 0,197108671 | |
| 1561059_a_at | LOC152024 | -2,089474779 | -2,286583451 | 0,197108671 | |
| 1570388_a_at | LOC440896 | -2,089474779 | -2,286583451 | 0,197108671 | |
| 207712_at | BAGE | -2,089474779 | -2,286583451 | 0,197108671 | |
| 207857_at | LILRA2 | -2,089474779 | -2,286583451 | 0,197108671 | |
| 213938_at | ERC2 | -2,089474779 | -2,286583451 | 0,197108671 | |
| 216610_at | SLC6A2 | -2,089474779 | -2,286583451 | 0,197108671 | |
| 223623_at | C2orf40 | -2,089474779 | -2,286583451 | 0,197108671 | |
| 226545_at | CD109 | -2,089474779 | -2,286583451 | 0,197108671 | |
| 237596_at | - | -2,089474779 | -2,286583451 | 0,197108671 | |
| 238262_at | SPDYA | -2,089474779 | -2,286583451 | 0,197108671 | |
| 239405_at | - | -2,089474779 | -2,286583451 | 0,197108671 | |
| 239759_at | - | -2,089474779 | -2,286583451 | 0,197108671 | |
| 241963_at | ZNF704 | -2,089474779 | -2,286583451 | 0,197108671 | |
| 224717_s_at | C19orf42 | 5,084646091 | 4,88753743 | 0,19710866 | |
| 213593_s_at | TRA2A | 0,401366113 | 0,204310755 | 0,197055358 | |
| 238427_at | GRPEL2 | 0,401366113 | 0,204310755 | 0,197055358 | |
| 227247_at | PLEKHA8 | 3,219589925 | 3,022611001 | 0,196978925 | |
| 212141_at | MCM4 | 4,308891884 | 4,111917374 | 0,19697451 | |
| 200915_x_at | KTN1 | 5,265058615 | 5,068098141 | 0,196960475 | |

| | | | | | |
|---|---|---|---|---|---|
| 214745_at | PLCH1 | -0,221656139 | -0,41858459 | 0,196928451 | |
| 220464_at | MCF2L | -0,221656139 | -0,41858459 | 0,196928451 | |
| 221729_at | COL5A2 | -0,221656139 | -0,41858459 | 0,196928451 | |
| 230941_at | LOC728537 | -0,221656139 | -0,41858459 | 0,196928451 | |
| 212383_at | ATP6V0A1 | 2,987866617 | 2,791007185 | 0,196859432 | |
| 215464_s_at | P2RX5-TAX1BP3 | 2,987866617 | 2,791007185 | 0,196859432 | |
| 225888_at | NAA25 | 2,822481847 | 2,625627364 | 0,196854483 | |
| 203014_x_at | SGSM3 | 0,888485433 | 0,691645685 | 0,196839748 | |
| 223143_s_at | AKIRIN2 | 1,210676994 | 1,013870809 | 0,196806185 | |
| 200099_s_at | RPS3A /// SNOR | 8,147865753 | 7,951236818 | 0,196628935 | |
| 222531_s_at | AP5M1 | 3,582860873 | 3,386261038 | 0,196599836 | |
| 225581_s_at | MRPL50 | 4,608815095 | 4,412250787 | 0,196564308 | |
| 1554057_at | ASH1L-AS1 | 1,638726528 | 1,442269454 | 0,196457074 | |
| 1558820_a_at | C18orf34 | 0,171847695 | -0,024572586 | 0,196420282 | |
| 227570_at | TMEM86A | 0,171847695 | -0,024572586 | 0,196420282 | |
| 228816_at | ATP6AP1L /// FLJ | 0,171847695 | -0,024572586 | 0,196420282 | |
| 1569522_at | - | 2,852640442 | 2,656223838 | 0,196416604 | |
| 230434_at | PHOSPHO2 | 0,728550685 | 0,532164371 | 0,196386315 | |
| 234953_x_at | ZNF19 | 0,728550685 | 0,532164371 | 0,196386315 | |
| 218229_s_at | POGK | 2,654607516 | 2,458228496 | 0,196379021 | |
| 228582_x_at | MALAT1 | 1,442503549 | 1,246149888 | 0,19635366 | |
| 1562946_at | - | -0,622910612 | -0,819238336 | 0,196327724 | |
| 202652_at | APBB1 | -0,622910612 | -0,819238336 | 0,196327724 | |
| 207113_s_at | TNF | -0,622910612 | -0,819238336 | 0,196327724 | |
| 211551_at | EGFR | -0,622910612 | -0,819238336 | 0,196327724 | |
| 213351_s_at | TMCC1 | -0,622910612 | -0,819238336 | 0,196327724 | |
| 215025_at | NTRK3 | -0,622910612 | -0,819238336 | 0,196327724 | |
| 223283_s_at | TSHZ1 | -0,622910612 | -0,819238336 | 0,196327724 | |
| 231782_s_at | KLK4 | -0,622910612 | -0,819238336 | 0,196327724 | |
| 234881_at | - | -0,622910612 | -0,819238336 | 0,196327724 | |
| 240721_at | - | -0,622910612 | -0,819238336 | 0,196327724 | |
| 241339_at | - | -0,622910612 | -0,819238336 | 0,196327724 | |
| 1554246_at | C1orf210 | -2,138804305 | -2,335104118 | 0,196299813 | |
| 1557211_a_at | FAM181A-AS1 | -2,138804305 | -2,335104118 | 0,196299813 | |
| 1561064_a_at | - | -2,138804305 | -2,335104118 | 0,196299813 | |
| 1563130_a_at | - | -2,138804305 | -2,335104118 | 0,196299813 | |
| 1567022_at | OR5AK4P | -2,138804305 | -2,335104118 | 0,196299813 | |
| 1568783_at | SREK1 | -2,138804305 | -2,335104118 | 0,196299813 | |
| 1570409_x_at | - | -2,138804305 | -2,335104118 | 0,196299813 | |
| 206164_at | CLCA2 | -2,138804305 | -2,335104118 | 0,196299813 | |
| 208543_at | OR10H2 | -2,138804305 | -2,335104118 | 0,196299813 | |
| 210669_at | TFAP2A | -2,138804305 | -2,335104118 | 0,196299813 | |
| 220121_at | LINS | -2,138804305 | -2,335104118 | 0,196299813 | |
| 220978_at | KRTAP1-3 | -2,138804305 | -2,335104118 | 0,196299813 | |
| 230831_at | FRMD5 | -2,138804305 | -2,335104118 | 0,196299813 | |
| 231028_at | LOC100506082 | -2,138804305 | -2,335104118 | 0,196299813 | |
| 232384_s_at | - | -2,138804305 | -2,335104118 | 0,196299813 | |
| 233950_at | CADPS | -2,138804305 | -2,335104118 | 0,196299813 | |
| 234691_at | KRTAP2-1 | -2,138804305 | -2,335104118 | 0,196299813 | |
| 237562_at | - | -2,138804305 | -2,335104118 | 0,196299813 | |
| 237663_at | - | -2,138804305 | -2,335104118 | 0,196299813 | |
| 241038_at | - | -2,138804305 | -2,335104118 | 0,196299813 | |
| 241449_at | - | -2,138804305 | -2,335104118 | 0,196299813 | |
| 241544_at | - | -2,138804305 | -2,335104118 | 0,196299813 | |
| 241665_x_at | - | -2,138804305 | -2,335104118 | 0,196299813 | |
| 243522_at | - | -2,138804305 | -2,335104118 | 0,196299813 | |

| | | | | | |
|---|---|---|---|---|---|
| 244473_at | - | -2,138804305 | -2,335104118 | 0,196299813 | |
| 244767_at | FRA10AC1 | -2,138804305 | -2,335104118 | 0,196299813 | |
| 209308_s_at | BNIP2 | 4,188728817 | 3,992437318 | 0,196291498 | |
| 1555816_at | THSD4 | -3,114791413 | -3,311072652 | 0,196281239 | |
| 1568848_at | - | -3,114791413 | -3,311072652 | 0,196281239 | |
| 242796_x_at | - | -3,114791413 | -3,311072652 | 0,196281239 | |
| 240066_at | - | 0,255492549 | 0,059218869 | 0,196273681 | |
| 240383_at | UBE2D3 | 0,255492549 | 0,059218869 | 0,196273681 | |
| 240908_at | LOC100507153 | 0,255492549 | 0,059218869 | 0,196273681 | |
| 213153_at | SETD1B | 3,593615441 | 3,39737216 | 0,196243281 | |
| 229465_s_at | - | 1,758252436 | 1,562009954 | 0,196242482 | |
| 208352_x_at | ANK1 | 1,512180019 | 1,315948728 | 0,196231291 | |
| 227508_at | - | 1,512180019 | 1,315948728 | 0,196231291 | |
| 217986_s_at | BAZ1A | 3,919150955 | 3,722963534 | 0,196187421 | |
| 212469_at | NIPBL | 3,120200849 | 2,924079008 | 0,196121841 | |
| 233562_at | LOC84856 | 2,842156496 | 2,646096941 | 0,196059555 | |
| 207483_s_at | CAND1 | 4,153426622 | 3,957375745 | 0,196050878 | |
| 213122_at | TSPYL5 | 5,219497001 | 5,023484486 | 0,196012515 | |
| 217879_at | CDC27 | 3,611363461 | 3,415371412 | 0,19599205 | |
| 224834_at | UBTD2 | 2,132266459 | 1,936283387 | 0,195983072 | |
| 227960_s_at | FAHD1 | 2,531373922 | 2,335461563 | 0,195912359 | |
| 238877_at | EYA4 | 2,531373922 | 2,335461563 | 0,195912359 | |
| 213699_s_at | YWHAQ | 7,080086872 | 6,884191563 | 0,195895309 | |
| 203476_at | TPBG | 3,843828743 | 3,647981081 | 0,195847662 | |
| 242224_at | GPATCH2 | 1,089278259 | 0,893473268 | 0,195804991 | |
| 1553856_s_at | P2RY10 | -1,591296941 | -1,787089803 | 0,195792862 | |
| 1556090_at | - | -1,591296941 | -1,787089803 | 0,195792862 | |
| 1562787_at | - | -1,591296941 | -1,787089803 | 0,195792862 | |
| 1563303_at | - | -1,591296941 | -1,787089803 | 0,195792862 | |
| 207662_at | TBX1 | -1,591296941 | -1,787089803 | 0,195792862 | |
| 207944_at | OCM2 | -1,591296941 | -1,787089803 | 0,195792862 | |
| 213791_at | PENK | -1,591296941 | -1,787089803 | 0,195792862 | |
| 214445_at | ELL2 | -1,591296941 | -1,787089803 | 0,195792862 | |
| 224091_at | - | -1,591296941 | -1,787089803 | 0,195792862 | |
| 232013_at | ERCC6L2 | -1,591296941 | -1,787089803 | 0,195792862 | |
| 234459_at | PPHLN1 | -1,591296941 | -1,787089803 | 0,195792862 | |
| 234524_at | PRDM15 | -1,591296941 | -1,787089803 | 0,195792862 | |
| 235929_s_at | - | -1,591296941 | -1,787089803 | 0,195792862 | |
| 236702_at | - | -1,591296941 | -1,787089803 | 0,195792862 | |
| 238028_at | C6orf132 | -1,591296941 | -1,787089803 | 0,195792862 | |
| 238387_s_at | LINC00226 | -1,591296941 | -1,787089803 | 0,195792862 | |
| 240650_at | CACNA1E | -1,591296941 | -1,787089803 | 0,195792862 | |
| 240660_at | - | -1,591296941 | -1,787089803 | 0,195792862 | |
| 242874_at | - | -1,591296941 | -1,787089803 | 0,195792862 | |
| 243085_at | - | -1,591296941 | -1,787089803 | 0,195792862 | |
| 244348_at | - | -1,591296941 | -1,787089803 | 0,195792862 | |
| 224591_at | HP1BP3 | 5,136741656 | 4,940979636 | 0,19576202 | |
| 225217_s_at | BRPF3 | 2,089851283 | 1,894118818 | 0,195732465 | |
| 224210_s_at | PXMP4 | 0,613492853 | 0,417770464 | 0,19572239 | |
| 234303_s_at | GPR85 | 2,338135744 | 2,14244844 | 0,195687304 | |
| 203829_at | ELP4 | 1,897847661 | 1,702196344 | 0,195651317 | |
| 213847_at | PRPH | 0,67554678 | 0,479907041 | 0,195639739 | |
| 219583_s_at | SPATA7 | 0,67554678 | 0,479907041 | 0,195639739 | |
| 203455_s_at | SAT1 | 6,708412674 | 6,512818573 | 0,1955941 | |
| 219557_s_at | NRIP3 | 1,261018186 | 1,065442793 | 0,195575393 | |
| 235242_at | - | 1,261018186 | 1,065442793 | 0,195575393 | |

| | | | | | |
|---|---|---|---|---|---|
| 205873_at | PIGL | 1,339784194 | 1,144305291 | 0,195478903 | |
| 215731_s_at | MPHOSPH9 | 1,339784194 | 1,144305291 | 0,195478903 | |
| 224492_s_at | ZNF627 | 5,329069831 | 5,133591331 | 0,1954785 | |
| 217725_x_at | SERBP1 | 5,939611094 | 5,744220235 | 0,195390859 | |
| 230182_at | PDCD7 | -0,00073712 | -0,19610998 | 0,19537286 | |
| 230726_at | MRPL38 | -0,00073712 | -0,19610998 | 0,19537286 | |
| 233567_at | IPO9-AS1 | -0,00073712 | -0,19610998 | 0,19537286 | |
| 243407_at | MFSD8 | -0,00073712 | -0,19610998 | 0,19537286 | |
| 205222_at | EHHADH | 0,900083947 | 0,704715219 | 0,195368728 | |
| 210633_x_at | KRT10 | 0,900083947 | 0,704715219 | 0,195368728 | |
| 224327_s_at | DGAT2 | 0,900083947 | 0,704715219 | 0,195368728 | |
| 32699_s_at | PVR | 0,552329299 | 0,356981896 | 0,195347403 | |
| 217743_s_at | TMEM30A | 4,206936942 | 4,011627006 | 0,195309936 | |
| 222687_s_at | ACER3 | 1,000476627 | 0,805216064 | 0,195260563 | |
| 241246_at | - | 1,000476627 | 0,805216064 | 0,195260563 | |
| 202582_s_at | RANBP9 | 5,150463493 | 4,95540405 | 0,195059443 | |
| 210284_s_at | TAB2 | 2,470441349 | 2,275409734 | 0,195031615 | |
| 1569048_s_at | LMF1 | 0,417596971 | 0,222599095 | 0,194997876 | |
| 227502_at | - | 0,417596971 | 0,222599095 | 0,194997876 | |
| 241757_x_at | DYNC2LI1 | 0,417596971 | 0,222599095 | 0,194997876 | |
| 209379_s_at | FAM190B | 1,454352496 | 1,259496994 | 0,194855502 | |
| 1553094_at | FLJ45513 /// TAC | -1,543331864 | -1,738067356 | 0,194735492 | |
| 1569987_at | DLEU7-AS1 | -1,543331864 | -1,738067356 | 0,194735492 | |
| 211120_x_at | ESR2 | -1,543331864 | -1,738067356 | 0,194735492 | |
| 211796_s_at | TRBC1 | -1,543331864 | -1,738067356 | 0,194735492 | |
| 211824_x_at | NLRP1 | -1,543331864 | -1,738067356 | 0,194735492 | |
| 211892_s_at | PTGIS | -1,543331864 | -1,738067356 | 0,194735492 | |
| 213915_at | NKG7 | -1,543331864 | -1,738067356 | 0,194735492 | |
| 220348_at | KLHL29 | -1,543331864 | -1,738067356 | 0,194735492 | |
| 221429_x_at | TEX13A | -1,543331864 | -1,738067356 | 0,194735492 | |
| 224360_s_at | PACSIN1 | -1,543331864 | -1,738067356 | 0,194735492 | |
| 226303_at | PGM5 | -1,543331864 | -1,738067356 | 0,194735492 | |
| 227419_x_at | PLAC9 | -1,543331864 | -1,738067356 | 0,194735492 | |
| 234203_at | - | -1,543331864 | -1,738067356 | 0,194735492 | |
| 235480_at | LOC100506472 | -1,543331864 | -1,738067356 | 0,194735492 | |
| 237795_s_at | SP2 | -1,543331864 | -1,738067356 | 0,194735492 | |
| 240825_at | - | -1,543331864 | -1,738067356 | 0,194735492 | |
| 1555888_at | UBR5 | 0,798390451 | 0,603679647 | 0,194710804 | |
| 235427_at | CFLAR | 0,798390451 | 0,603679647 | 0,194710804 | |
| 226422_at | ERGIC2 | 3,18563636 | 2,990953566 | 0,194682793 | |
| 206770_s_at | SLC35A3 | 2,637386731 | 2,442710777 | 0,194675954 | |
| 230069_at | SFXN1 | 2,637386731 | 2,442710777 | 0,194675954 | |
| 212756_s_at | UBR2 | 2,491678455 | 2,297144887 | 0,194533569 | |
| 201167_x_at | ARHGDIA | 1,348276312 | 1,153866761 | 0,19440955 | |
| 1558511_s_at | ESYT2 | 3,466766854 | 3,272369332 | 0,194397522 | |
| 1568658_at | C2orf74 /// KIAA1 | 2,348755185 | 2,154405725 | 0,19434946 | |
| 209264_s_at | TSPAN4 | 1,593009234 | 1,398700978 | 0,194308256 | |
| 225859_at | XIAP | 1,593009234 | 1,398700978 | 0,194308256 | |
| 223130_s_at | MYLIP | 4,931293191 | 4,737036893 | 0,194256298 | |
| 225289_at | STAT3 | 4,06796029 | 3,873779903 | 0,194180388 | |
| 213119_at | SLC36A1 | 2,102456302 | 1,908311039 | 0,194145263 | |
| 225734_at | FBXO22 | 2,102456302 | 1,908311039 | 0,194145263 | |
| 223073_at | HIATL1 | 4,027552846 | 3,833443169 | 0,194109676 | |
| 217972_at | CHCHD3 | 5,214280103 | 5,02022104 | 0,194059063 | |
| 214741_at | LOC100506639 / | 3,416167722 | 3,222126232 | 0,194041489 | |
| 1555832_s_at | KLF6 | 2,626954721 | 2,432926755 | 0,194027967 | |

| | | | | | |
|---|---|---|---|---|---|
| 204588_s_at | SLC7A7 | 0,273439642 | 0,079428135 | 0,194011507 | |
| 207814_at | DEFA6 | 0,273439642 | 0,079428135 | 0,194011507 | |
| 234885_at | - | 0,273439642 | 0,079428135 | 0,194011507 | |
| 227411_at | WTIP | 0,351548291 | 0,157549243 | 0,193999048 | |
| 238694_at | DGKE | 0,351548291 | 0,157549243 | 0,193999048 | |
| 242723_at | - | 0,351548291 | 0,157549243 | 0,193999048 | |
| 208963_x_at | FADS1 /// MIR19 | -0,089689931 | -0,283667828 | 0,193977896 | |
| 220008_at | PEAK1 | -0,089689931 | -0,283667828 | 0,193977896 | |
| 227341_at | BEND7 | -0,089689931 | -0,283667828 | 0,193977896 | |
| 227965_at | LOC100506848 | -0,089689931 | -0,283667828 | 0,193977896 | |
| 225765_at | TNPO1 | 4,010933848 | 3,816987307 | 0,193946541 | |
| 226053_at | MAP2K7 | 1,011212064 | 0,817300337 | 0,193911727 | |
| 41387_r_at | KDM6B | 1,294361492 | 1,100476722 | 0,193884771 | |
| 1566432_at | - | -0,196756757 | -0,39061235 | 0,193855593 | |
| 213711_at | KRT81 | -0,196756757 | -0,39061235 | 0,193855593 | |
| 224264_x_at | ZAN | -0,196756757 | -0,39061235 | 0,193855593 | |
| 235097_at | TRMT44 | -0,196756757 | -0,39061235 | 0,193855593 | |
| 239400_at | FLJ45513 | -0,196756757 | -0,39061235 | 0,193855593 | |
| 243663_at | - | -0,196756757 | -0,39061235 | 0,193855593 | |
| 225502_at | DOCK8 | 1,912238072 | 1,718396606 | 0,193841466 | |
| 234752_x_at | DMD | -3,15396682 | -3,347710726 | 0,193743906 | |
| 241652_x_at | LIN7A | -3,15396682 | -3,347710726 | 0,193743906 | |
| 242664_at | - | -3,15396682 | -3,347710726 | 0,193743906 | |
| 1556851_at | LOC100506609 | -2,262747984 | -2,456406426 | 0,193658442 | |
| 1557143_at | CSMD2 | -2,262747984 | -2,456406426 | 0,193658442 | |
| 1559462_at | - | -2,262747984 | -2,456406426 | 0,193658442 | |
| 1560533_at | - | -2,262747984 | -2,456406426 | 0,193658442 | |
| 1561405_s_at | CATSPER2 | -2,262747984 | -2,456406426 | 0,193658442 | |
| 1565768_at | ZNF268 | -2,262747984 | -2,456406426 | 0,193658442 | |
| 1566289_at | OR2M4 | -2,262747984 | -2,456406426 | 0,193658442 | |
| 1566958_at | - | -2,262747984 | -2,456406426 | 0,193658442 | |
| 205908_s_at | OMD | -2,262747984 | -2,456406426 | 0,193658442 | |
| 207959_s_at | DNAH9 | -2,262747984 | -2,456406426 | 0,193658442 | |
| 209289_at | NFIB | -2,262747984 | -2,456406426 | 0,193658442 | |
| 213888_s_at | TRAF3IP3 | -2,262747984 | -2,456406426 | 0,193658442 | |
| 221228_s_at | - | -2,262747984 | -2,456406426 | 0,193658442 | |
| 221352_at | - | -2,262747984 | -2,456406426 | 0,193658442 | |
| 227202_at | CNTN1 | -2,262747984 | -2,456406426 | 0,193658442 | |
| 231386_at | FAM228A | -2,262747984 | -2,456406426 | 0,193658442 | |
| 231787_at | SLC25A27 | -2,262747984 | -2,456406426 | 0,193658442 | |
| 233633_at | TBL1XR1 | -2,262747984 | -2,456406426 | 0,193658442 | |
| 234619_at | - | -2,262747984 | -2,456406426 | 0,193658442 | |
| 237102_at | - | -2,262747984 | -2,456406426 | 0,193658442 | |
| 237552_at | LOC100505817 | -2,262747984 | -2,456406426 | 0,193658442 | |
| 241446_at | ADAM28 | -2,262747984 | -2,456406426 | 0,193658442 | |
| 241569_at | - | -2,262747984 | -2,456406426 | 0,193658442 | |
| 243846_x_at | - | -2,262747984 | -2,456406426 | 0,193658442 | |
| 244253_at | - | -2,262747984 | -2,456406426 | 0,193658442 | |
| 1561504_s_at | MYLK4 | -1,026571149 | -1,220210588 | 0,193639439 | |
| 1565581_at | - | -1,026571149 | -1,220210588 | 0,193639439 | |
| 215971_at | - | -1,026571149 | -1,220210588 | 0,193639439 | |
| 216172_at | - | -1,026571149 | -1,220210588 | 0,193639439 | |
| 219804_at | SYNPO2L | -1,026571149 | -1,220210588 | 0,193639439 | |
| 220480_at | HAND2 | -1,026571149 | -1,220210588 | 0,193639439 | |
| 236655_at | TPD52 | -1,026571149 | -1,220210588 | 0,193639439 | |
| 237430_at | - | -1,026571149 | -1,220210588 | 0,193639439 | |

| | | | | | |
|---|---|---|---|---|---|
| 238876_at | - | -1,026571149 | -1,220210588 | 0,193639439 | |
| 238998_x_at | OTUD1 | -1,026571149 | -1,220210588 | 0,193639439 | |
| 239671_at | - | -1,026571149 | -1,220210588 | 0,193639439 | |
| 241414_at | ANKRD10 | -1,026571149 | -1,220210588 | 0,193639439 | |
| 212546_s_at | FRYL | 2,779171778 | 2,585563171 | 0,193608607 | |
| 229099_at | C11orf83 | 2,446911392 | 2,253342112 | 0,19356928 | |
| 209646_x_at | ALDH1B1 | 1,835789711 | 1,642321262 | 0,193468448 | |
| 222212_s_at | CERS2 | 4,102254727 | 3,908790275 | 0,193464452 | |
| 218131_s_at | GATAD2A | 4,681605321 | 4,488147962 | 0,193457359 | |
| 202900_s_at | NUP88 | 4,302897163 | 4,109462531 | 0,193434632 | |
| 224177_s_at | CXorf26 | 3,456984333 | 3,263564154 | 0,193420179 | |
| 202425_x_at | PPP3CA | 3,3845211 | 3,191118448 | 0,193402652 | |
| 205354_at | GAMT | 1,46610492 | 1,272721751 | 0,193383169 | |
| 233933_s_at | OTUD5 | 1,394109935 | 1,200746069 | 0,193363866 | |
| 219419_at | RBFA | 2,501229299 | 2,307890888 | 0,193338411 | |
| 212514_x_at | DDX3X | 4,462972394 | 4,269747853 | 0,193224541 | |
| 226661_at | CDCA2 | 3,776205896 | 3,583111417 | 0,193094479 | |
| 225517_at | ZNF770 | 4,144312047 | 3,951219941 | 0,193092106 | |
| 210334_x_at | BIRC5 | 3,260656977 | 3,067567462 | 0,193089515 | |
| 200942_s_at | HSBP1 | 4,818544611 | 4,625489401 | 0,19305521 | |
| 202762_at | ROCK2 | 3,166555554 | 2,973511251 | 0,193044304 | |
| 202950_at | CRYZ | 3,155710652 | 2,962671751 | 0,193038901 | |
| 228763_at | MDP1 /// NEDD8 | 2,35929703 | 2,166264721 | 0,193032308 | |
| 1557352_at | SQLE | 0,503742507 | 0,310717132 | 0,193025375 | |
| 204184_s_at | ADRBK2 | 0,503742507 | 0,310717132 | 0,193025375 | |
| 232195_at | GPR158 | 0,503742507 | 0,310717132 | 0,193025375 | |
| 227725_at | ST6GALNAC1 | 2,320980353 | 2,127967638 | 0,193012715 | |
| 223017_at | TXNDC12 | 4,495830712 | 4,302892559 | 0,192938153 | |
| 227288_at | SREK1IP1 | 3,199783157 | 3,006869115 | 0,192914042 | |
| 224866_at | FAR1 | 4,19991587 | 4,007018329 | 0,192897541 | |
| 212670_at | ELN | 0,20027154 | 0,007421914 | 0,192849626 | |
| 219025_at | CD248 | 0,20027154 | 0,007421914 | 0,192849626 | |
| 230778_at | - | 0,20027154 | 0,007421914 | 0,192849626 | |
| 240311_at | NANOS3 | 0,20027154 | 0,007421914 | 0,192849626 | |
| 242377_x_at | THUMPD3 | 1,360921491 | 1,16809119 | 0,192830301 | |
| 214472_at | HIST1H2AD /// H | 4,371158382 | 4,17842456 | 0,192733823 | |
| 1557521_a_at | - | 1,699726954 | 1,507116528 | 0,192610427 | |
| 1564511_a_at | FSTL4 | -1,470833215 | -1,663414495 | 0,192581279 | |
| 1568625_at | LOC100505997 | -1,470833215 | -1,663414495 | 0,192581279 | |
| 1569665_at | - | -1,470833215 | -1,663414495 | 0,192581279 | |
| 205554_s_at | DNASE1L3 | -1,470833215 | -1,663414495 | 0,192581279 | |
| 206946_at | HCN4 | -1,470833215 | -1,663414495 | 0,192581279 | |
| 207049_at | SCN8A | -1,470833215 | -1,663414495 | 0,192581279 | |
| 208256_at | EFNA2 | -1,470833215 | -1,663414495 | 0,192581279 | |
| 213966_at | HMG20B | -1,470833215 | -1,663414495 | 0,192581279 | |
| 216133_at | YME1L1 | -1,470833215 | -1,663414495 | 0,192581279 | |
| 217257_at | SH3BP2 | -1,470833215 | -1,663414495 | 0,192581279 | |
| 223862_at | GHRL | -1,470833215 | -1,663414495 | 0,192581279 | |
| 227635_at | RBBP6 | -1,470833215 | -1,663414495 | 0,192581279 | |
| 229788_s_at | - | -1,470833215 | -1,663414495 | 0,192581279 | |
| 230627_at | - | -1,470833215 | -1,663414495 | 0,192581279 | |
| 231076_at | C16orf82 | -1,470833215 | -1,663414495 | 0,192581279 | |
| 235637_s_at | - | -1,470833215 | -1,663414495 | 0,192581279 | |
| 236038_at | - | -1,470833215 | -1,663414495 | 0,192581279 | |
| 239358_at | - | -1,470833215 | -1,663414495 | 0,192581279 | |
| 239546_at | LOC100131053 | -1,470833215 | -1,663414495 | 0,192581279 | |

| | | | | | |
|---|---|---|---|---|---|
| 240424_s_at | LOC441204 | -1,470833215 | -1,663414495 | 0,192581279 | |
| 240444_x_at | CLIP1 | -1,470833215 | -1,663414495 | 0,192581279 | |
| 243590_at | - | -1,470833215 | -1,663414495 | 0,192581279 | |
| 205018_s_at | MBNL2 | 2,400710591 | 2,208167653 | 0,192542939 | |
| 229431_at | RFXAP | 2,400710591 | 2,208167653 | 0,192542939 | |
| 228157_at | ZNF207 | 2,382221458 | 2,189694216 | 0,192527241 | |
| 227335_at | DIDO1 | 1,542099044 | 1,349621332 | 0,192477712 | |
| 225959_s_at | ZNRF1 | 2,774453351 | 2,582026171 | 0,19242718 | |
| 1552359_at | MCMDC2 | -2,311268652 | -2,503677622 | 0,192408971 | |
| 1557755_at | CEP128 | -2,311268652 | -2,503677622 | 0,192408971 | |
| 1564945_at | TBX20 | -2,311268652 | -2,503677622 | 0,192408971 | |
| 1568656_at | - | -2,311268652 | -2,503677622 | 0,192408971 | |
| 203698_s_at | FRZB | -2,311268652 | -2,503677622 | 0,192408971 | |
| 207579_at | MAGEB3 | -2,311268652 | -2,503677622 | 0,192408971 | |
| 211368_s_at | CASP1 | -2,311268652 | -2,503677622 | 0,192408971 | |
| 214912_at | - | -2,311268652 | -2,503677622 | 0,192408971 | |
| 216012_at | - | -2,311268652 | -2,503677622 | 0,192408971 | |
| 216173_at | - | -2,311268652 | -2,503677622 | 0,192408971 | |
| 217075_x_at | MLL4 | -2,311268652 | -2,503677622 | 0,192408971 | |
| 234115_s_at | ZNRD1 | -2,311268652 | -2,503677622 | 0,192408971 | |
| 238286_at | - | -2,311268652 | -2,503677622 | 0,192408971 | |
| 238304_at | - | -2,311268652 | -2,503677622 | 0,192408971 | |
| 238361_s_at | - | -2,311268652 | -2,503677622 | 0,192408971 | |
| 238755_at | RASSF10 | -2,311268652 | -2,503677622 | 0,192408971 | |
| 239636_at | MCF2L | -2,311268652 | -2,503677622 | 0,192408971 | |
| 244146_at | DTWD1 | -2,311268652 | -2,503677622 | 0,192408971 | |
| 1558046_x_at | LOC389906 | 0,760715727 | 0,568365095 | 0,192350631 | |
| 1568640_at | PCBP1-AS1 | 0,760715727 | 0,568365095 | 0,192350631 | |
| 227138_at | CRTAP | 0,760715727 | 0,568365095 | 0,192350631 | |
| 219766_at | B9D2 | 1,247462054 | 1,055274826 | 0,192187228 | |
| 227903_x_at | TPGS1 | 1,247462054 | 1,055274826 | 0,192187228 | |
| 224917_at | MIR21 | 3,825698252 | 3,633530837 | 0,192167415 | |
| 216048_s_at | RHOBTB3 | 4,232392431 | 4,040263518 | 0,192128913 | |
| 1554052_at | CNOT1 | 1,926486359 | 1,73441697 | 0,192069389 | |
| 213138_at | ARID5A | 0,44160593 | 0,249604176 | 0,192001754 | |
| 222191_s_at | B4GALT7 | 0,44160593 | 0,249604176 | 0,192001754 | |
| 226592_at | ZNF618 | 0,44160593 | 0,249604176 | 0,192001754 | |
| 236905_at | - | 0,44160593 | 0,249604176 | 0,192001754 | |
| 207786_at | CYP2R1 | 1,027166904 | 0,835239052 | 0,191927852 | |
| 209044_x_at | SF3B4 | 4,125908304 | 3,933981372 | 0,191926932 | |
| 214635_at | CLDN9 | -0,299034815 | -0,490927335 | 0,19189252 | |
| 237713_at | - | -0,299034815 | -0,490927335 | 0,19189252 | |
| 235728_at | ZFP3 | 1,735773781 | 1,543943227 | 0,191830553 | |
| 203163_at | KATNB1 | 1,369290453 | 1,177496821 | 0,191793632 | |
| 227268_at | RNFT1 | 2,876321464 | 2,684536601 | 0,191784863 | |
| 212893_at | ZZZ3 | 2,979690938 | 2,787940144 | 0,191750794 | |
| 212780_at | SOS1 | 2,369762403 | 2,17802703 | 0,191735372 | |
| 219572_at | CADPS2 | 2,369762403 | 2,17802703 | 0,191735372 | |
| 201156_s_at | RAB5C | 3,89689195 | 3,705189898 | 0,191702052 | |
| 244447_at | - | 2,630440446 | 2,438805127 | 0,191635319 | |
| 201053_s_at | PSMF1 | 3,981211211 | 3,789647959 | 0,191563252 | |
| 221505_at | ANP32E | 5,204228009 | 5,01268698 | 0,191541029 | |
| 213147_at | HOXA10 | 1,168140757 | 0,976641161 | 0,191499596 | |
| 236475_at | MICAL2 | 1,168140757 | 0,976641161 | 0,191499596 | |
| 204353_s_at | POT1 | 3,211467103 | 3,019999252 | 0,19146785 | |
| 203522_at | CCS | 1,982112682 | 1,790660532 | 0,19145215 | |

| | | | | | |
|---|---|---|---|---|---|
| 221984_s_at | FAM134A | 3,101484927 | 2,910041809 | 0,191443118 | |
| 203801_at | MRPS14 | 0,03125738 | -0,160136748 | 0,191394128 | |
| 230704_s_at | ITGB4 | 0,03125738 | -0,160136748 | 0,191394128 | |
| 228670_at | TEP1 | 0,648296226 | 0,456918297 | 0,191377928 | |
| 236605_at | EIF3K | 0,648296226 | 0,456918297 | 0,191377928 | |
| 238596_at | FRA10AC1 | 0,648296226 | 0,456918297 | 0,191377928 | |
| 240286_at | - | 1,191926657 | 1,000684521 | 0,191242136 | |
| 205967_at | HIST1H4A /// HIS | 4,496785894 | 4,305575871 | 0,191210023 | |
| 1557121_s_at | - | 1,296557217 | 1,105412836 | 0,191144381 | |
| 209023_s_at | STAG2 | 1,799091453 | 1,60795124 | 0,191140214 | |
| 223204_at | FAM198B | 2,466546243 | 2,275409734 | 0,191136509 | |
| 218023_s_at | FAM53C | 3,266269388 | 3,075135559 | 0,191133829 | |
| 1553235_at | PCDHAC1 | -0,423248115 | -0,614371577 | 0,191123461 | |
| 1562030_at | LOC284898 | -0,423248115 | -0,614371577 | 0,191123461 | |
| 1566249_at | - | -0,423248115 | -0,614371577 | 0,191123461 | |
| 206814_at | NGF | -0,423248115 | -0,614371577 | 0,191123461 | |
| 207998_s_at | CACNA1D | -0,423248115 | -0,614371577 | 0,191123461 | |
| 215899_at | - | -0,423248115 | -0,614371577 | 0,191123461 | |
| 217208_s_at | DLG1 | -0,423248115 | -0,614371577 | 0,191123461 | |
| 222082_at | ZBTB7A | -0,423248115 | -0,614371577 | 0,191123461 | |
| 228579_at | KCNQ3 | -0,423248115 | -0,614371577 | 0,191123461 | |
| 234573_at | - | -0,423248115 | -0,614371577 | 0,191123461 | |
| 244110_at | MLL | -0,423248115 | -0,614371577 | 0,191123461 | |
| 1554872_a_at | HMGCLL1 | -1,424023738 | -1,615132408 | 0,191108669 | |
| 1566873_at | - | -1,424023738 | -1,615132408 | 0,191108669 | |
| 206206_at | CD180 | -1,424023738 | -1,615132408 | 0,191108669 | |
| 206856_at | LILRB5 | -1,424023738 | -1,615132408 | 0,191108669 | |
| 207502_at | GUCA2B | -1,424023738 | -1,615132408 | 0,191108669 | |
| 212029_s_at | - | -1,424023738 | -1,615132408 | 0,191108669 | |
| 216569_at | - | -1,424023738 | -1,615132408 | 0,191108669 | |
| 217418_x_at | MS4A1 | -1,424023738 | -1,615132408 | 0,191108669 | |
| 220100_at | SLC22A11 | -1,424023738 | -1,615132408 | 0,191108669 | |
| 220959_s_at | OBP2A /// OBP2E | -1,424023738 | -1,615132408 | 0,191108669 | |
| 221321_s_at | KCNIP2 | -1,424023738 | -1,615132408 | 0,191108669 | |
| 229635_at | LOC100505702 | -1,424023738 | -1,615132408 | 0,191108669 | |
| 230747_s_at | TTC39C | -1,424023738 | -1,615132408 | 0,191108669 | |
| 232005_at | DNAH1 | -1,424023738 | -1,615132408 | 0,191108669 | |
| 233669_s_at | TRIM54 | -1,424023738 | -1,615132408 | 0,191108669 | |
| 234624_at | - | -1,424023738 | -1,615132408 | 0,191108669 | |
| 234907_x_at | POLB | -1,424023738 | -1,615132408 | 0,191108669 | |
| 237801_at | - | -1,424023738 | -1,615132408 | 0,191108669 | |
| 244068_at | - | -1,424023738 | -1,615132408 | 0,191108669 | |
| 208232_x_at | NRG1 | -0,759588698 | -0,950686014 | 0,191097316 | |
| 210064_s_at | UPK1B | -0,759588698 | -0,950686014 | 0,191097316 | |
| 216011_at | SLC39A9 | -0,759588698 | -0,950686014 | 0,191097316 | |
| 217654_at | CFLAR | -0,759588698 | -0,950686014 | 0,191097316 | |
| 228837_at | TCF4 | -0,759588698 | -0,950686014 | 0,191097316 | |
| 230597_at | SLC7A3 | -0,759588698 | -0,950686014 | 0,191097316 | |
| 230932_at | - | -0,759588698 | -0,950686014 | 0,191097316 | |
| 231216_at | TMEM179 | -0,759588698 | -0,950686014 | 0,191097316 | |
| 232143_at | DNM1P41 | -0,759588698 | -0,950686014 | 0,191097316 | |
| 239684_at | - | -0,759588698 | -0,950686014 | 0,191097316 | |
| 239922_at | CCDC142 | -0,759588698 | -0,950686014 | 0,191097316 | |
| 240748_at | - | -0,759588698 | -0,950686014 | 0,191097316 | |
| 240998_at | - | -0,759588698 | -0,950686014 | 0,191097316 | |
| 241887_at | UBE2W | -0,759588698 | -0,950686014 | 0,191097316 | |

| | | | | | |
|---|---|---|---|---|---|
| 243699_at | LOC100507006 | -0,759588698 | -0,950686014 | 0,191097316 | |
| 244296_at | - | -0,759588698 | -0,950686014 | 0,191097316 | |
| 1564224_x_at | C1RL-AS1 | -0,57445847 | -0,765504029 | 0,191045559 | |
| 208592_s_at | CD1E | -0,57445847 | -0,765504029 | 0,191045559 | |
| 209855_s_at | KLK2 | -0,57445847 | -0,765504029 | 0,191045559 | |
| 210411_s_at | GRIN2B | -0,57445847 | -0,765504029 | 0,191045559 | |
| 229092_at | NR2F2 | -0,57445847 | -0,765504029 | 0,191045559 | |
| 233599_at | LOC728061 | -0,57445847 | -0,765504029 | 0,191045559 | |
| 236139_at | - | -0,57445847 | -0,765504029 | 0,191045559 | |
| 237065_s_at | - | -0,57445847 | -0,765504029 | 0,191045559 | |
| 238826_x_at | - | -0,57445847 | -0,765504029 | 0,191045559 | |
| 240484_at | - | -0,57445847 | -0,765504029 | 0,191045559 | |
| 242726_at | - | -0,57445847 | -0,765504029 | 0,191045559 | |
| 242769_at | - | -0,57445847 | -0,765504029 | 0,191045559 | |
| 220285_at | FAM108B1 | 1,485482016 | 1,294497133 | 0,190984883 | |
| 227244_s_at | - | 0,82905153 | 0,638150376 | 0,190901154 | |
| 229391_s_at | FAM26F | 0,82905153 | 0,638150376 | 0,190901154 | |
| 214042_s_at | RPL22 | 5,337225169 | 5,146370538 | 0,19085463 | |
| 218877_s_at | TRMT11 | 3,822654227 | 3,631821253 | 0,190832974 | |
| 213738_s_at | ATP5A1 | 7,293554726 | 7,102773699 | 0,190781027 | |
| 201170_s_at | BHLHE40 | 5,023656689 | 4,83315541 | 0,190501279 | |
| 202892_at | CDC23 | 4,246090231 | 4,05564712 | 0,190443111 | |
| 208703_s_at | APLP2 | 2,846658948 | 2,656223838 | 0,190435109 | |
| 211746_x_at | PSMA1 | 5,522627195 | 5,332197571 | 0,190429624 | |
| 224911_s_at | DCBLD2 | 4,138203514 | 3,947788666 | 0,190414848 | |
| 225368_at | HIPK2 | 4,920655822 | 4,730262306 | 0,190393516 | |
| 214109_at | LRBA | 3,805796305 | 3,615478364 | 0,190317942 | |
| 203166_at | CFDP1 | 3,755604375 | 3,565308644 | 0,190295731 | |
| 223148_at | PIGS | 2,609398637 | 2,419116683 | 0,190281954 | |
| 221935_s_at | EOGT | 3,004080279 | 2,813804482 | 0,190275797 | |
| 210448_s_at | P2RX5 | 1,381753566 | 1,191491281 | 0,190262285 | |
| 40850_at | FKBP8 | 1,381753566 | 1,191491281 | 0,190262285 | |
| 1552875_a_at | CD200R1 | -2,384869671 | -2,575129729 | 0,190260058 | |
| 1553746_a_at | OTOGL | -2,384869671 | -2,575129729 | 0,190260058 | |
| 1556677_at | - | -2,384869671 | -2,575129729 | 0,190260058 | |
| 1556945_a_at | - | -2,384869671 | -2,575129729 | 0,190260058 | |
| 1557046_x_at | LOC100506930 | -2,384869671 | -2,575129729 | 0,190260058 | |
| 1562031_at | JAK2 | -2,384869671 | -2,575129729 | 0,190260058 | |
| 1563920_at | FAM45A | -2,384869671 | -2,575129729 | 0,190260058 | |
| 1569975_at | - | -2,384869671 | -2,575129729 | 0,190260058 | |
| 205980_s_at | ARHGAP8 /// PR | -2,384869671 | -2,575129729 | 0,190260058 | |
| 206190_at | GPR17 | -2,384869671 | -2,575129729 | 0,190260058 | |
| 207110_at | KCNJ12 /// LOC1 | -2,384869671 | -2,575129729 | 0,190260058 | |
| 208298_at | EVI5 | -2,384869671 | -2,575129729 | 0,190260058 | |
| 214842_s_at | - | -2,384869671 | -2,575129729 | 0,190260058 | |
| 215080_s_at | AGAP2 | -2,384869671 | -2,575129729 | 0,190260058 | |
| 215790_at | AJAP1 | -2,384869671 | -2,575129729 | 0,190260058 | |
| 217292_at | MTMR7 | -2,384869671 | -2,575129729 | 0,190260058 | |
| 222384_at | DKFZP564C196 | -2,384869671 | -2,575129729 | 0,190260058 | |
| 225759_x_at | CLMN | -2,384869671 | -2,575129729 | 0,190260058 | |
| 229233_at | NRG3 | -2,384869671 | -2,575129729 | 0,190260058 | |
| 230809_at | - | -2,384869671 | -2,575129729 | 0,190260058 | |
| 231426_at | - | -2,384869671 | -2,575129729 | 0,190260058 | |
| 239353_at | STK32A | -2,384869671 | -2,575129729 | 0,190260058 | |
| 240408_at | - | -2,384869671 | -2,575129729 | 0,190260058 | |
| 240605_at | - | -2,384869671 | -2,575129729 | 0,190260058 | |

| | | | | | |
|---|---|---|---|---|---|
| 241858_at | TNNI3K | -2,384869671 | -2,575129729 | 0,190260058 | |
| 242985_x_at | RNF180 | -2,384869671 | -2,575129729 | 0,190260058 | |
| 244522_at | - | -2,384869671 | -2,575129729 | 0,190260058 | |
| 212163_at | KIDINS220 | 3,77777851 | 3,587527995 | 0,190250515 | |
| 201943_s_at | CPD | 2,134723057 | 1,944570415 | 0,190152642 | |
| 226036_x_at | CASP2 | 1,305306849 | 1,115234684 | 0,190072165 | |
| 205456_at | CD3E | 0,457392764 | 0,26733076 | 0,190062005 | |
| 221496_s_at | TOB2 | 0,457392764 | 0,26733076 | 0,190062005 | |
| 1556473_at | FLJ38379 | -2,908843709 | -3,098858316 | 0,190014608 | |
| 1559665_at | LOC100506999 | -2,908843709 | -3,098858316 | 0,190014608 | |
| 1562637_at | SAMD12 | -2,908843709 | -3,098858316 | 0,190014608 | |
| 1567280_at | - | -2,908843709 | -3,098858316 | 0,190014608 | |
| 206977_at | PTH | -2,908843709 | -3,098858316 | 0,190014608 | |
| 216039_at | GABRA2 | -2,908843709 | -3,098858316 | 0,190014608 | |
| 221205_at | - | -2,908843709 | -3,098858316 | 0,190014608 | |
| 231680_at | - | -2,908843709 | -3,098858316 | 0,190014608 | |
| 237879_at | - | -2,908843709 | -3,098858316 | 0,190014608 | |
| 237920_at | SYCP2 | -2,908843709 | -3,098858316 | 0,190014608 | |
| 240448_at | SOGA2 | -2,908843709 | -3,098858316 | 0,190014608 | |
| 240509_s_at | GREM2 | -2,908843709 | -3,098858316 | 0,190014608 | |
| 243779_at | GALNT13 | -2,908843709 | -3,098858316 | 0,190014608 | |
| 1565851_at | - | -3,212234735 | -3,402185466 | 0,189950731 | |
| 1570633_at | - | -3,212234735 | -3,402185466 | 0,189950731 | |
| 214537_at | HIST1H1D | 0,995078803 | 0,805216064 | 0,189862739 | |
| 227027_at | GFPT1 | 2,752227654 | 2,562416132 | 0,189811522 | |
| 209297_at | ITSN1 | 1,995690482 | 1,805902329 | 0,189788153 | |
| 222030_at | SIVA1 | 0,945567441 | 0,755840599 | 0,189726842 | |
| 227626_at | PAQR8 | 2,288253583 | 2,098562556 | 0,189691027 | |
| 226853_at | BMP2K | 4,226646405 | 4,037037849 | 0,189608556 | |
| 203446_s_at | OCRL | 2,247975581 | 2,058399602 | 0,18957598 | |
| 223369_at | NTMT1 | 2,86748657 | 2,677924589 | 0,189561981 | |
| 202089_s_at | SLC39A6 | 2,663141434 | 2,473581079 | 0,189560354 | |
| 1566342_at | - | 2,582657321 | 2,39311332 | 0,189544001 | |
| 216652_s_at | DR1 | 2,972842302 | 2,783327323 | 0,189514979 | |
| 203319_s_at | ZNF148 | 1,094335495 | 0,904843258 | 0,189492236 | |
| 229393_at | L3MBTL3 | 1,094335495 | 0,904843258 | 0,189492236 | |
| 230157_at | CDH24 | 1,426552034 | 1,23718272 | 0,189369315 | |
| 201563_at | SORD | 4,360703168 | 4,171380634 | 0,189322533 | |
| 202262_x_at | DDAH2 | 3,260656977 | 3,071356473 | 0,189300504 | |
| 209134_s_at | RPS6 | 7,867810282 | 7,678598239 | 0,189212043 | |
| 216858_x_at | - | 2,001086023 | 1,811954245 | 0,189131778 | |
| 222857_s_at | KCNMB4 | 2,097427503 | 1,908311039 | 0,189116463 | |
| 202213_s_at | CUL4B | 3,729025365 | 3,540009821 | 0,189015544 | |
| 209974_s_at | BUB3 | 5,802647924 | 5,613677691 | 0,188970233 | |
| 211678_s_at | RNF114 | 4,485281752 | 4,296432174 | 0,188849578 | |
| 200077_s_at | OAZ1 | 7,346969566 | 7,158123196 | 0,18884637 | |
| 1556389_at | CNPY3 | 0,052199481 | -0,136643983 | 0,188843463 | |
| 204311_at | ATP1B2 | 0,052199481 | -0,136643983 | 0,188843463 | |
| 205508_at | SCN1B | 0,052199481 | -0,136643983 | 0,188843463 | |
| 208603_s_at | MAPK8IP2 | 0,052199481 | -0,136643983 | 0,188843463 | |
| 220169_at | TMEM156 | 0,052199481 | -0,136643983 | 0,188843463 | |
| 233000_x_at | DPH3P1 | 0,052199481 | -0,136643983 | 0,188843463 | |
| 1553484_at | LINC00477 | -2,43257096 | -2,621331989 | 0,188761029 | |
| 1555366_at | NSAP11 | -2,43257096 | -2,621331989 | 0,188761029 | |
| 1556881_at | ARHGEF33 | -2,43257096 | -2,621331989 | 0,188761029 | |
| 1558645_at | MIB1 | -2,43257096 | -2,621331989 | 0,188761029 | |

| | | | | | |
|---|---|---|---|---|---|
| 1560743_a_at | - | -2,43257096 | -2,621331989 | 0,188761029 | |
| 1561660_at | SLC25A53 | -2,43257096 | -2,621331989 | 0,188761029 | |
| 1567862_at | DNAH14 | -2,43257096 | -2,621331989 | 0,188761029 | |
| 205979_at | SCGB2A1 | -2,43257096 | -2,621331989 | 0,188761029 | |
| 216291_at | ZNF440 | -2,43257096 | -2,621331989 | 0,188761029 | |
| 225987_at | STEAP4 | -2,43257096 | -2,621331989 | 0,188761029 | |
| 234571_at | - | -2,43257096 | -2,621331989 | 0,188761029 | |
| 237000_at | - | -2,43257096 | -2,621331989 | 0,188761029 | |
| 237442_at | - | -2,43257096 | -2,621331989 | 0,188761029 | |
| 237565_at | - | -2,43257096 | -2,621331989 | 0,188761029 | |
| 239985_at | - | -2,43257096 | -2,621331989 | 0,188761029 | |
| 214719_at | SLC46A3 | 3,088871381 | 2,900133867 | 0,188737514 | |
| 221188_s_at | CIDEB | 0,606430281 | 0,417770464 | 0,188659818 | |
| 228196_s_at | LARP4B | 1,504602299 | 1,315948728 | 0,188653571 | |
| 208242_at | RAX | 0,317354302 | 0,128747141 | 0,188607161 | |
| 210029_at | IDO1 | 0,317354302 | 0,128747141 | 0,188607161 | |
| 213055_at | CD47 | 0,317354302 | 0,128747141 | 0,188607161 | |
| 230948_at | - | 0,317354302 | 0,128747141 | 0,188607161 | |
| 235554_x_at | PACRGL | 0,317354302 | 0,128747141 | 0,188607161 | |
| 221894_at | ADCK2 | 1,318332599 | 1,129843209 | 0,18848939 | |
| 222568_at | UGGT1 | 0,541261317 | 0,352836757 | 0,188424559 | |
| 228823_at | POLR2J2 | 0,541261317 | 0,352836757 | 0,188424559 | |
| 235993_at | PSMF1 | 0,541261317 | 0,352836757 | 0,188424559 | |
| 240991_at | - | 0,541261317 | 0,352836757 | 0,188424559 | |
| 242080_at | - | 0,541261317 | 0,352836757 | 0,188424559 | |
| 203324_s_at | CAV2 | 5,844049959 | 5,65571578 | 0,188334178 | |
| 1557295_a_at | - | -1,353583928 | -1,541908042 | 0,188324114 | |
| 1561526_at | - | -1,353583928 | -1,541908042 | 0,188324114 | |
| 1562253_at | SLC7A11-AS1 | -1,353583928 | -1,541908042 | 0,188324114 | |
| 1564626_at | - | -1,353583928 | -1,541908042 | 0,188324114 | |
| 205659_at | HDAC9 | -1,353583928 | -1,541908042 | 0,188324114 | |
| 207179_at | TLX1 | -1,353583928 | -1,541908042 | 0,188324114 | |
| 214935_at | IL4I1 /// NUP62 // | -1,353583928 | -1,541908042 | 0,188324114 | |
| 220624_s_at | ELF5 | -1,353583928 | -1,541908042 | 0,188324114 | |
| 221316_at | CATSPERG | -1,353583928 | -1,541908042 | 0,188324114 | |
| 222197_s_at | - | -1,353583928 | -1,541908042 | 0,188324114 | |
| 224172_at | - | -1,353583928 | -1,541908042 | 0,188324114 | |
| 228596_at | LOC728377 | -1,353583928 | -1,541908042 | 0,188324114 | |
| 231118_at | ANKRD35 | -1,353583928 | -1,541908042 | 0,188324114 | |
| 231729_s_at | CAPS | -1,353583928 | -1,541908042 | 0,188324114 | |
| 233629_at | - | -1,353583928 | -1,541908042 | 0,188324114 | |
| 236678_at | - | -1,353583928 | -1,541908042 | 0,188324114 | |
| 240785_at | - | -1,353583928 | -1,541908042 | 0,188324114 | |
| 242644_at | TMC8 | -1,353583928 | -1,541908042 | 0,188324114 | |
| 244595_at | - | -1,353583928 | -1,541908042 | 0,188324114 | |
| 244701_at | - | -1,353583928 | -1,541908042 | 0,188324114 | |
| 212648_at | DHX29 | 3,918438257 | 3,730171924 | 0,188266334 | |
| 212206_s_at | H2AFV | 3,472604685 | 3,284389368 | 0,188215317 | |
| 224778_s_at | TAOK1 | 1,932146506 | 1,743944487 | 0,188202018 | |
| 222000_at | C1orf174 | 4,218563327 | 4,030564794 | 0,187998533 | |
| 208619_at | DDB1 | 4,860334514 | 4,672396377 | 0,187938137 | |
| 214259_s_at | AKR7A2 | 3,456984333 | 3,269073685 | 0,187910648 | |
| 204936_at | MAP4K2 | -0,543051993 | -0,730778808 | 0,187726815 | |
| 214536_at | SLURP1 | -0,543051993 | -0,730778808 | 0,187726815 | |
| 229330_at | - | -0,543051993 | -0,730778808 | 0,187726815 | |
| 203470_s_at | PLEK | 0,911589958 | 0,724100169 | 0,187489789 | |

| | | | | | |
|---|---|---|---|---|---|
| 228239_at | FAM165B | 0,911589958 | 0,724100169 | 0,187489789 | |
| 1569719_at | BCL2L14 | -3,250025915 | -3,437493426 | 0,187467511 | |
| 218399_s_at | CDCA4 | 3,829494273 | 3,642048499 | 0,187445774 | |
| 221966_at | GPR137 | 1,649074067 | 1,461646549 | 0,187427517 | |
| 204647_at | HOMER3 | 1,829737795 | 1,642321262 | 0,187416533 | |
| 222530_s_at | MKKS | 4,657889997 | 4,470561285 | 0,187328712 | |
| 222903_s_at | CPEB1 | 0,859074518 | 0,67181667 | 0,187257848 | |
| 235036_at | LIX1L | 0,859074518 | 0,67181667 | 0,187257848 | |
| 209397_at | ME2 | 4,529358299 | 4,34210478 | 0,18725352 | |
| 202106_at | GOLGA3 | 2,758612869 | 2,571362843 | 0,187250025 | |
| 237040_at | CWF19L2 | 0,682279745 | 0,495031851 | 0,187247895 | |
| 206289_at | HOXA4 | 0,480753764 | 0,293518836 | 0,187234928 | |
| 244656_at | RASL10B | 0,480753764 | 0,293518836 | 0,187234928 | |
| 206509_at | PIP | 0,246434561 | 0,059218869 | 0,187215692 | |
| 212038_s_at | VDAC1 | 5,37147771 | 5,184334316 | 0,187143394 | |
| 228825_at | PTGR1 | -0,033456564 | -0,220592223 | 0,187135659 | |
| 236880_at | RAD52 | -0,033456564 | -0,220592223 | 0,187135659 | |
| 221028_s_at | GFOD2 | 1,481627361 | 1,294497133 | 0,187130228 | |
| 213375_s_at | N4BP2L1 | 1,446464021 | 1,259496994 | 0,186967026 | |
| 212329_at | SCAP | 2,68509622 | 2,498185745 | 0,186910476 | |
| 204185_x_at | PPID | 4,731959456 | 4,545117128 | 0,186842328 | |
| 211717_at | ANKRD40 | 1,292162421 | 1,105412836 | 0,186749585 | |
| 202232_s_at | EIF3M | 5,940313411 | 5,753585187 | 0,186728225 | |
| 225493_at | CCNT1 | 3,76356289 | 3,576905405 | 0,186657485 | |
| 216360_x_at | RRP12 | 1,021868205 | 0,835239052 | 0,186629153 | |
| 1553768_a_at | DCBLD1 | -0,136301282 | -0,322870281 | 0,186568999 | |
| 205418_at | FES | -0,136301282 | -0,322870281 | 0,186568999 | |
| 220839_at | METTL5 | -0,136301282 | -0,322870281 | 0,186568999 | |
| 229844_at | FOXP1 | -0,136301282 | -0,322870281 | 0,186568999 | |
| 203968_s_at | CDC6 | 3,431234921 | 3,244672787 | 0,186562134 | |
| 227224_at | RALGPS2 | 1,946200473 | 1,759685182 | 0,186515291 | |
| 1558340_at | DIXDC1 | -1,308181863 | -1,494668682 | 0,186486819 | |
| 1562717_at | LINC00299 | -1,308181863 | -1,494668682 | 0,186486819 | |
| 1569387_at | CSGALNACT1 | -1,308181863 | -1,494668682 | 0,186486819 | |
| 203329_at | PTPRM | -1,308181863 | -1,494668682 | 0,186486819 | |
| 204539_s_at | CELSR1 | -1,308181863 | -1,494668682 | 0,186486819 | |
| 205597_at | SLC44A4 | -1,308181863 | -1,494668682 | 0,186486819 | |
| 232155_at | RNF213 | -1,308181863 | -1,494668682 | 0,186486819 | |
| 235934_at | NAPA-AS1 | -1,308181863 | -1,494668682 | 0,186486819 | |
| 237162_at | KANK1 | -1,308181863 | -1,494668682 | 0,186486819 | |
| 237696_at | - | -1,308181863 | -1,494668682 | 0,186486819 | |
| 238945_at | ACER3 | -1,308181863 | -1,494668682 | 0,186486819 | |
| 239934_x_at | - | -1,308181863 | -1,494668682 | 0,186486819 | |
| 240466_at | - | -1,308181863 | -1,494668682 | 0,186486819 | |
| 228385_at | DDX59 | 1,119359127 | 0,932882274 | 0,186476854 | |
| 226021_at | RDH10 | 2,794789337 | 2,608344689 | 0,186444648 | |
| 227426_at | SOS1 | 2,450859799 | 2,264418116 | 0,186441682 | |
| 227031_at | SNX13 | 1,523472489 | 1,337086025 | 0,186386464 | |
| 1558783_at | - | 0,97328325 | 0,786897721 | 0,186385529 | |
| 205584_at | ALG13 | 0,97328325 | 0,786897721 | 0,186385529 | |
| 227601_at | METTL14 | 2,166283906 | 1,979940779 | 0,186343127 | |
| 1553620_at | TRIM42 | -2,504600601 | -2,690860744 | 0,186260143 | |
| 1555073_at | MGC40069 | -2,504600601 | -2,690860744 | 0,186260143 | |
| 1558402_at | - | -2,504600601 | -2,690860744 | 0,186260143 | |
| 1558950_at | - | -2,504600601 | -2,690860744 | 0,186260143 | |
| 1559265_at | SKIDA1 | -2,504600601 | -2,690860744 | 0,186260143 | |

| | | | | | |
|---|---|---|---|---|---|
| 1560185_at | - | -2,504600601 | -2,690860744 | 0,186260143 | |
| 1560755_at | - | -2,504600601 | -2,690860744 | 0,186260143 | |
| 1561795_at | - | -2,504600601 | -2,690860744 | 0,186260143 | |
| 1564940_at | - | -2,504600601 | -2,690860744 | 0,186260143 | |
| 1566478_at | C17orf104 | -2,504600601 | -2,690860744 | 0,186260143 | |
| 205669_at | NCAM2 | -2,504600601 | -2,690860744 | 0,186260143 | |
| 206641_at | TNFRSF17 | -2,504600601 | -2,690860744 | 0,186260143 | |
| 214884_at | MCF2 | -2,504600601 | -2,690860744 | 0,186260143 | |
| 216276_s_at | ADAM3A | -2,504600601 | -2,690860744 | 0,186260143 | |
| 216588_at | - | -2,504600601 | -2,690860744 | 0,186260143 | |
| 233312_at | ROPN1L | -2,504600601 | -2,690860744 | 0,186260143 | |
| 241023_at | - | -2,504600601 | -2,690860744 | 0,186260143 | |
| 241332_at | - | -2,504600601 | -2,690860744 | 0,186260143 | |
| 243342_at | - | -2,504600601 | -2,690860744 | 0,186260143 | |
| 244594_x_at | - | -2,504600601 | -2,690860744 | 0,186260143 | |
| 244724_at | - | -2,504600601 | -2,690860744 | 0,186260143 | |
| 244866_at | - | -2,504600601 | -2,690860744 | 0,186260143 | |
| 217917_s_at | DYNLRB1 | 5,227862525 | 5,041624605 | 0,186237919 | |
| 223076_s_at | NSUN2 | 3,698630507 | 3,512402411 | 0,186228096 | |
| 211793_s_at | ABI2 | 2,122398022 | 1,936283387 | 0,186114635 | |
| 222504_s_at | EMC8 | 1,783520648 | 1,59747851 | 0,186042138 | |
| 1562488_at | PIEZO2 | -0,926850548 | -1,112851844 | 0,186001296 | |
| 1563228_x_at | SLC38A10 | -0,926850548 | -1,112851844 | 0,186001296 | |
| 206962_x_at | - | -0,926850548 | -1,112851844 | 0,186001296 | |
| 208177_at | SLC34A1 | -0,926850548 | -1,112851844 | 0,186001296 | |
| 228035_at | STK33 | -0,926850548 | -1,112851844 | 0,186001296 | |
| 229818_at | SVOP | -0,926850548 | -1,112851844 | 0,186001296 | |
| 236447_at | - | -0,926850548 | -1,112851844 | 0,186001296 | |
| 236941_at | PRR14L | -0,926850548 | -1,112851844 | 0,186001296 | |
| 236942_at | - | -0,926850548 | -1,112851844 | 0,186001296 | |
| 239176_at | - | -0,926850548 | -1,112851844 | 0,186001296 | |
| 240571_at | - | -0,926850548 | -1,112851844 | 0,186001296 | |
| 243978_at | FAM65C | -0,926850548 | -1,112851844 | 0,186001296 | |
| 1559096_x_at | FBXO9 | 1,339784194 | 1,153866761 | 0,185917433 | |
| 215581_s_at | MCM3AP | 1,339784194 | 1,153866761 | 0,185917433 | |
| 202596_at | ENSA | 4,791943463 | 4,606029888 | 0,185913575 | |
| 217753_s_at | RPS26 | 7,472082308 | 7,286183356 | 0,185898952 | |
| 221543_s_at | ERLIN2 | 3,51093129 | 3,325159096 | 0,185772194 | |
| 218209_s_at | RPRD1A | 3,279650757 | 3,09388391 | 0,185766847 | |
| 223250_at | KLHL7 | 2,771299138 | 2,585563171 | 0,185735967 | |
| 232661_s_at | RBM48 | 1,079110292 | 0,893473268 | 0,185637024 | |
| 202813_at | TARBP1 | 2,531373922 | 2,345926937 | 0,185446986 | |
| 205335_s_at | SRP19 | 5,398050406 | 5,212630303 | 0,185420102 | |
| 217730_at | TMBIM1 | 4,61981709 | 4,434486264 | 0,185330826 | |
| 200866_s_at | PSAP | 4,514815228 | 4,329503706 | 0,185311523 | |
| 209109_s_at | TSPAN6 | 4,419307767 | 4,234134575 | 0,185173192 | |
| 242915_at | ZNF682 | 0,083054335 | -0,102100538 | 0,185154872 | |
| 243053_x_at | LOC100128079 | 0,083054335 | -0,102100538 | 0,185154872 | |
| 227085_at | H2AFV | 3,285189903 | 3,100079607 | 0,185110297 | |
| 223098_s_at | LONP2 | 2,243430036 | 2,058399602 | 0,185030435 | |
| 1554627_a_at | ASCC1 | 3,120200849 | 2,935211251 | 0,184989598 | |
| 218930_s_at | TMEM106B | 2,327437554 | 2,14244844 | 0,184989114 | |
| 215429_s_at | ZNF428 | 2,637386731 | 2,452428893 | 0,184957838 | |
| 218214_at | C12orf44 | 3,211467103 | 3,026519779 | 0,184947324 | |
| 235390_at | SREK1IP1 | 2,178241192 | 1,993317152 | 0,184924039 | |
| 218646_at | C4orf27 | 3,638448528 | 3,453616097 | 0,184832431 | |

| | | | | | |
|---|---|---|---|---|---|
| 219418_at | NHEJ1 /// SLC23 | 1,764611101 | 1,579853228 | 0,184757872 | |
| 239863_at | - | 1,224581536 | 1,039887232 | 0,184694304 | |
| 1555514_a_at | PIAS2 | 2,225103271 | 2,040470494 | 0,184632777 | |
| 217232_x_at | HBB | 4,769592513 | 4,584979629 | 0,184612884 | |
| 216080_s_at | FADS3 | 1,672933557 | 1,488344553 | 0,184589004 | |
| 1552765_x_at | TMEM67 | -2,551294263 | -2,735862426 | 0,184568163 | |
| 1552971_at | SGCZ | -2,551294263 | -2,735862426 | 0,184568163 | |
| 1559670_at | LOC100506489 | -2,551294263 | -2,735862426 | 0,184568163 | |
| 1563522_at | DDX10 | -2,551294263 | -2,735862426 | 0,184568163 | |
| 205590_at | RASGRP1 | -2,551294263 | -2,735862426 | 0,184568163 | |
| 207052_at | HAVCR1 | -2,551294263 | -2,735862426 | 0,184568163 | |
| 228949_at | WLS | -2,551294263 | -2,735862426 | 0,184568163 | |
| 230309_at | BHMT2 | -2,551294263 | -2,735862426 | 0,184568163 | |
| 232144_at | - | -2,551294263 | -2,735862426 | 0,184568163 | |
| 233365_at | - | -2,551294263 | -2,735862426 | 0,184568163 | |
| 234577_at | - | -2,551294263 | -2,735862426 | 0,184568163 | |
| 238379_x_at | - | -2,551294263 | -2,735862426 | 0,184568163 | |
| 240955_at | PANX3 | -2,551294263 | -2,735862426 | 0,184568163 | |
| 241369_at | - | -2,551294263 | -2,735862426 | 0,184568163 | |
| 214359_s_at | HSP90AB1 | 6,567212057 | 6,38265478 | 0,184557277 | |
| 226942_at | PHF20L1 | 3,123915073 | 2,939363796 | 0,184551277 | |
| 218954_s_at | BRF2 | 2,064305961 | 1,879785595 | 0,184520365 | |
| 233960_s_at | LOC100133445 / | 0,641402394 | 0,456918297 | 0,184484097 | |
| 228327_x_at | MEIS3 | 1,089278259 | 0,904843258 | 0,184435001 | |
| 210672_s_at | NPRL3 | 1,990274687 | 1,805902329 | 0,184372358 | |
| 206183_s_at | HERC3 | -0,234278768 | -0,41858459 | 0,184305822 | |
| 213706_at | GPD1 | -0,234278768 | -0,41858459 | 0,184305822 | |
| 217374_x_at | - | -0,234278768 | -0,41858459 | 0,184305822 | |
| 219400_at | CNTNAP1 | -0,234278768 | -0,41858459 | 0,184305822 | |
| 219521_at | B3GAT1 | -0,234278768 | -0,41858459 | 0,184305822 | |
| 223544_at | TMEM79 | -0,234278768 | -0,41858459 | 0,184305822 | |
| 227306_at | - | -0,234278768 | -0,41858459 | 0,184305822 | |
| 239730_at | DGCR14 /// TSSK | -0,234278768 | -0,41858459 | 0,184305822 | |
| 226077_at | RNF145 | 3,67603922 | 3,491815843 | 0,184223377 | |
| 229145_at | ANAPC16 | 2,114952142 | 1,930732138 | 0,184220004 | |
| 202133_at | WWTR1 | 3,877954598 | 3,693764321 | 0,184190277 | |
| 202419_at | KDSR | 3,042527069 | 2,858346012 | 0,184181057 | |
| 201469_s_at | SHC1 | 1,314003737 | 1,129843209 | 0,184160528 | |
| 228951_at | SLC38A7 | 0,57782909 | 0,393761504 | 0,184067586 | |
| 225922_at | FNIP2 | 2,043538949 | 1,859476797 | 0,184062152 | |
| 217812_at | YTHDF2 | 4,689563828 | 4,505522833 | 0,184040994 | |
| 232946_s_at | NADSYN1 | 1,709647566 | 1,525647381 | 0,184000185 | |
| 220235_s_at | LRIF1 | 3,886725813 | 3,702749166 | 0,183976647 | |
| 1555858_at | LOC440944 | 1,274448124 | 1,090553537 | 0,183894587 | |
| 207980_s_at | CITED2 | 3,492853048 | 3,309203139 | 0,183649909 | |
| 205995_x_at | IQCB1 | 2,632180155 | 2,448549499 | 0,183630656 | |
| 203964_at | NMI | 4,413755582 | 4,230185498 | 0,183570084 | |
| 208492_at | RFXAP | 0,359971644 | 0,176436073 | 0,183535571 | |
| 239515_at | - | 0,359971644 | 0,176436073 | 0,183535571 | |
| 202648_at | TCF3 | 2,190100188 | 2,006570641 | 0,183529546 | |
| 218791_s_at | C15orf29 | 1,04816921 | 0,864649967 | 0,183519243 | |
| 228433_at | NFYA | 3,299492934 | 3,116064987 | 0,183427947 | |
| 217543_s_at | MBTPS1 | 1,683039483 | 1,499637023 | 0,18340246 | |
| 208546_x_at | HIST1H2BH | 5,581663686 | 5,398297283 | 0,183366403 | |
| 228978_at | LOC645722 | 0,715481152 | 0,532164371 | 0,183316781 | |
| 233881_s_at | TOLLIP | 0,715481152 | 0,532164371 | 0,183316781 | |

| | | | | | |
|---|---|---|---|---|---|
| 238084_at | PCGF3 | 0,715481152 | 0,532164371 | 0,183316781 | |
| 241826_x_at | ZNF738 | 0,715481152 | 0,532164371 | 0,183316781 | |
| 219110_at | GAR1 | 5,105773197 | 4,922569129 | 0,183204068 | |
| 1555600_s_at | APOL4 | -1,240169874 | -1,423348341 | 0,183178467 | |
| 1556536_at | LOC729224 | -1,240169874 | -1,423348341 | 0,183178467 | |
| 1561207_at | - | -1,240169874 | -1,423348341 | 0,183178467 | |
| 1562984_at | - | -1,240169874 | -1,423348341 | 0,183178467 | |
| 204063_s_at | ULK2 | -1,240169874 | -1,423348341 | 0,183178467 | |
| 205154_at | LRRN2 | -1,240169874 | -1,423348341 | 0,183178467 | |
| 211467_s_at | NFIB | -1,240169874 | -1,423348341 | 0,183178467 | |
| 214255_at | ATP10A | -1,240169874 | -1,423348341 | 0,183178467 | |
| 214384_s_at | DCTN2 | -1,240169874 | -1,423348341 | 0,183178467 | |
| 225807_at | AJUBA | -1,240169874 | -1,423348341 | 0,183178467 | |
| 231440_at | LOC100507206 | -1,240169874 | -1,423348341 | 0,183178467 | |
| 233701_at | - | -1,240169874 | -1,423348341 | 0,183178467 | |
| 234792_x_at | IGHA1 /// IGHV4- | -1,240169874 | -1,423348341 | 0,183178467 | |
| 239613_at | - | -1,240169874 | -1,423348341 | 0,183178467 | |
| 244449_at | LOC100506021 | -1,240169874 | -1,423348341 | 0,183178467 | |
| 207260_at | FEV | 1,000476627 | 0,817300337 | 0,18317629 | |
| 1552275_s_at | PXK | -0,352951799 | -0,536087151 | 0,183135352 | |
| 1554800_at | RAB39A | -0,352951799 | -0,536087151 | 0,183135352 | |
| 204907_s_at | BCL3 | -0,352951799 | -0,536087151 | 0,183135352 | |
| 219171_s_at | ZNF236 | -0,352951799 | -0,536087151 | 0,183135352 | |
| 219712_s_at | SHPK /// TRPV1 | -0,352951799 | -0,536087151 | 0,183135352 | |
| 220678_at | FLJ20712 | -0,352951799 | -0,536087151 | 0,183135352 | |
| 222242_s_at | KLK5 | -0,352951799 | -0,536087151 | 0,183135352 | |
| 236017_at | CDKL3 | -0,352951799 | -0,536087151 | 0,183135352 | |
| 238432_at | FLJ35776 | -0,352951799 | -0,536087151 | 0,183135352 | |
| 242685_at | GTPBP8 | -0,352951799 | -0,536087151 | 0,183135352 | |
| 229192_s_at | TBCD | 1,238353345 | 1,055274826 | 0,183078519 | |
| 218970_s_at | CUTC | 2,950705598 | 2,767843855 | 0,182861743 | |
| 206812_at | ADRB3 | -0,67289328 | -0,855751026 | 0,182857746 | |
| 215221_at | - | -0,67289328 | -0,855751026 | 0,182857746 | |
| 215390_at | - | -0,67289328 | -0,855751026 | 0,182857746 | |
| 231097_at | - | -0,67289328 | -0,855751026 | 0,182857746 | |
| 231474_at | KHDC3L | -0,67289328 | -0,855751026 | 0,182857746 | |
| 232813_s_at | GOLGA6A /// GO | -0,67289328 | -0,855751026 | 0,182857746 | |
| 233352_at | - | -0,67289328 | -0,855751026 | 0,182857746 | |
| 233728_at | - | -0,67289328 | -0,855751026 | 0,182857746 | |
| 235739_at | - | -0,67289328 | -0,855751026 | 0,182857746 | |
| 237782_at | - | -0,67289328 | -0,855751026 | 0,182857746 | |
| 239031_at | SSTR2 | -0,67289328 | -0,855751026 | 0,182857746 | |
| 1554337_at | FARP2 | -2,597496523 | -2,780313924 | 0,182817401 | |
| 1557280_s_at | ITSN1 | -2,597496523 | -2,780313924 | 0,182817401 | |
| 1560723_at | LOC283731 | -2,597496523 | -2,780313924 | 0,182817401 | |
| 1561964_at | - | -2,597496523 | -2,780313924 | 0,182817401 | |
| 1566899_at | - | -2,597496523 | -2,780313924 | 0,182817401 | |
| 205696_s_at | GFRA1 | -2,597496523 | -2,780313924 | 0,182817401 | |
| 212977_at | CXCR7 | -2,597496523 | -2,780313924 | 0,182817401 | |
| 215981_at | LOC100505534 | -2,597496523 | -2,780313924 | 0,182817401 | |
| 219701_at | TMOD2 | -2,597496523 | -2,780313924 | 0,182817401 | |
| 221117_at | - | -2,597496523 | -2,780313924 | 0,182817401 | |
| 225960_at | ZNRF1 | -2,597496523 | -2,780313924 | 0,182817401 | |
| 232365_at | SIAH1 | -2,597496523 | -2,780313924 | 0,182817401 | |
| 232777_s_at | C6orf118 | -2,597496523 | -2,780313924 | 0,182817401 | |
| 233948_at | TBX5-AS1 | -2,597496523 | -2,780313924 | 0,182817401 | |

| | | | | | |
|---|---|---|---|---|---|
| 234596_at | - | -2,597496523 | -2,780313924 | 0,182817401 | |
| 236888_at | CAPZA3 | -2,597496523 | -2,780313924 | 0,182817401 | |
| 237885_at | SOX21-AS1 | -2,597496523 | -2,780313924 | 0,182817401 | |
| 240299_at | ADAD1 | -2,597496523 | -2,780313924 | 0,182817401 | |
| 240796_at | - | -2,597496523 | -2,780313924 | 0,182817401 | |
| 241969_at | ARID5B | -2,597496523 | -2,780313924 | 0,182817401 | |
| 243473_at | - | -2,597496523 | -2,780313924 | 0,182817401 | |
| 244261_at | IL28RA | -2,597496523 | -2,780313924 | 0,182817401 | |
| 227591_at | LOC100505696 | 0,103263601 | -0,079522948 | 0,18278655 | |
| 231291_at | - | 0,103263601 | -0,079522948 | 0,18278655 | |
| 233586_s_at | KLK12 | 0,103263601 | -0,079522948 | 0,18278655 | |
| 242108_at | - | 0,103263601 | -0,079522948 | 0,18278655 | |
| 49452_at | ACACB | 0,103263601 | -0,079520317 | 0,182783919 | |
| 205318_at | KIF5A | -0,496991414 | -0,679763839 | 0,182772426 | |
| 214275_at | MED12 | -0,496991414 | -0,679763839 | 0,182772426 | |
| 218634_at | PHLDA3 | -0,496991414 | -0,679763839 | 0,182772426 | |
| 221008_s_at | AGXT2L1 | -0,496991414 | -0,679763839 | 0,182772426 | |
| 221199_at | GFRA4 | -0,496991414 | -0,679763839 | 0,182772426 | |
| 231910_at | NUDT14 | -0,496991414 | -0,679763839 | 0,182772426 | |
| 232816_s_at | DDX11 | -0,496991414 | -0,679763839 | 0,182772426 | |
| 235788_at | - | -0,496991414 | -0,679763839 | 0,182772426 | |
| 241596_at | NUDT10 | -0,496991414 | -0,679763839 | 0,182772426 | |
| 242083_at | ZNF81 | -0,496991414 | -0,679763839 | 0,182772426 | |
| 218850_s_at | LIMD1 | 0,518867317 | 0,336136178 | 0,182731139 | |
| 236630_at | AQP2 | 0,518867317 | 0,336136178 | 0,182731139 | |
| 237947_at | - | 0,518867317 | 0,336136178 | 0,182731139 | |
| 205034_at | CCNE2 | 4,268634793 | 4,085930792 | 0,182704001 | |
| 204968_at | C6orf47 | 0,841135803 | 0,658444279 | 0,182691524 | |
| 232029_at | - | 0,841135803 | 0,658444279 | 0,182691524 | |
| 225438_at | NUDCD1 | 1,326951551 | 1,144305291 | 0,18264626 | |
| 227741_at | PTPLB | 1,326951551 | 1,144305291 | 0,18264626 | |
| 243964_at | - | 1,326951551 | 1,144305291 | 0,18264626 | |
| 200640_at | YWHAZ | 5,887213751 | 5,704616451 | 0,182597299 | |
| 237520_x_at | - | -3,32387526 | -3,506469152 | 0,182593893 | |
| 217731_s_at | ITM2B | 5,117309148 | 4,934753321 | 0,182555826 | |
| 200840_at | KARS | 6,914385573 | 6,73197677 | 0,182408802 | |
| 37022_at | PRELP | 0,20027154 | 0,01793138 | 0,18234016 | |
| 225348_at | SRSF10 | 1,593009234 | 1,410714371 | 0,182294863 | |
| 203613_s_at | NDUFB6 | 5,844894131 | 5,662639447 | 0,182254684 | |
| 208666_s_at | ST13 | 3,93615092 | 3,753943043 | 0,182207876 | |
| 41386_i_at | KDM6B | 2,132266459 | 1,950068772 | 0,182197687 | |
| 218640_s_at | PLEKHF2 | 4,993182736 | 4,811041972 | 0,182140764 | |
| 223992_x_at | ZCWPW1 | 0,661985843 | 0,479907041 | 0,182078802 | |
| 203903_s_at | HEPH | 5,29611668 | 5,114131207 | 0,181985473 | |
| 223246_s_at | STRBP | 1,373456798 | 1,191491281 | 0,181965517 | |
| 209157_at | DNAJA2 | 4,781007315 | 4,599047207 | 0,181960109 | |
| 206560_s_at | MIA | 0,291166226 | 0,109221392 | 0,181944833 | |
| 214634_at | HIST1H4A /// HIS | 0,291166226 | 0,109221392 | 0,181944833 | |
| 227857_at | - | 0,291166226 | 0,109221392 | 0,181944833 | |
| 238724_at | - | 0,291166226 | 0,109221392 | 0,181944833 | |
| 212910_at | THAP11 | 3,098971025 | 2,917077481 | 0,181893544 | |
| 1554700_at | CDH7 | -3,054236653 | -3,236070201 | 0,181833548 | |
| 1563186_at | - | -3,054236653 | -3,236070201 | 0,181833548 | |
| 1563396_x_at | - | -3,054236653 | -3,236070201 | 0,181833548 | |
| 210729_at | NPY2R | -3,054236653 | -3,236070201 | 0,181833548 | |
| 222966_at | - | -3,054236653 | -3,236070201 | 0,181833548 | |

| | | | | | |
|---|---|---|---|---|---|
| 223315_at | NTN4 | -3,054236653 | -3,236070201 | 0,181833548 | |
| 223681_s_at | INADL | -3,054236653 | -3,236070201 | 0,181833548 | |
| 232125_at | - | -3,054236653 | -3,236070201 | 0,181833548 | |
| 233326_at | CCDC39 | -3,054236653 | -3,236070201 | 0,181833548 | |
| 233772_at | POU6F2-AS2 | -3,054236653 | -3,236070201 | 0,181833548 | |
| 234426_x_at | PIH2 | -3,054236653 | -3,236070201 | 0,181833548 | |
| 237865_x_at | - | -3,054236653 | -3,236070201 | 0,181833548 | |
| 241184_x_at | - | -3,054236653 | -3,236070201 | 0,181833548 | |
| 243681_at | SHANK2 | -3,054236653 | -3,236070201 | 0,181833548 | |
| 217959_s_at | MIR3656 /// TRAI | 4,308347938 | 4,126559358 | 0,18178858 | |
| 238565_at | - | 1,631786706 | 1,450051551 | 0,181735155 | |
| 222742_s_at | RABL5 | 2,987866617 | 2,806245339 | 0,181621277 | |
| 209077_at | TXN2 | 4,040056475 | 3,858511405 | 0,181545069 | |
| 215720_s_at | NFYA | 1,114389003 | 0,932882274 | 0,18150673 | |
| 240854_x_at | - | 1,114389003 | 0,932882274 | 0,18150673 | |
| 211733_x_at | SCP2 | 4,96239695 | 4,780891495 | 0,181505455 | |
| 226161_at | SLC30A6 | 2,908260915 | 2,726791458 | 0,181469457 | |
| 32259_at | EZH1 | 1,617806104 | 1,436405209 | 0,181400895 | |
| 1552330_at | CENPBD1 | 1,530951994 | 1,349621332 | 0,181330662 | |
| 223002_s_at | XRN2 | 4,312693778 | 4,131407181 | 0,181286597 | |
| 1556423_at | VASH1 | 0,209623097 | 0,028364014 | 0,181259083 | |
| 210737_at | TUB | 0,209623097 | 0,028364014 | 0,181259083 | |
| 200712_s_at | MAPRE1 | 4,467364499 | 4,286143696 | 0,181220803 | |
| 218230_at | ARFIP1 | 3,41111015 | 3,23005767 | 0,18105248 | |
| 202744_at | SLC20A2 | 0,735041319 | 0,553993624 | 0,181047695 | |
| 207215_at | GSTTP1 | 0,735041319 | 0,553993624 | 0,181047695 | |
| 225722_at | MIR3658 /// UCK | 0,735041319 | 0,553993624 | 0,181047695 | |
| 1557756_a_at | CEP128 | -1,196375121 | -1,377419394 | 0,181044273 | |
| 1561910_at | - | -1,196375121 | -1,377419394 | 0,181044273 | |
| 1569555_at | GDA | -1,196375121 | -1,377419394 | 0,181044273 | |
| 203838_s_at | TNK2 | -1,196375121 | -1,377419394 | 0,181044273 | |
| 206278_at | PTAFR | -1,196375121 | -1,377419394 | 0,181044273 | |
| 207015_s_at | ALDH1A2 | -1,196375121 | -1,377419394 | 0,181044273 | |
| 209910_at | SLC25A16 | -1,196375121 | -1,377419394 | 0,181044273 | |
| 210255_at | RAD51B | -1,196375121 | -1,377419394 | 0,181044273 | |
| 211722_s_at | HDAC6 | -1,196375121 | -1,377419394 | 0,181044273 | |
| 222560_at | LANCL2 | -1,196375121 | -1,377419394 | 0,181044273 | |
| 222949_at | NXF3 | -1,196375121 | -1,377419394 | 0,181044273 | |
| 232151_at | MACC1 | -1,196375121 | -1,377419394 | 0,181044273 | |
| 232255_at | LOC401321 | -1,196375121 | -1,377419394 | 0,181044273 | |
| 235395_at | SEC63 | -1,196375121 | -1,377419394 | 0,181044273 | |
| 212834_at | DDX52 | 1,381753566 | 1,200746069 | 0,181007497 | |
| 203545_at | ALG8 | 4,421321457 | 4,24031851 | 0,181002947 | |
| 220290_at | AIM1L | 0,533835206 | 0,352836757 | 0,180998449 | |
| 225543_at | GTF3C4 | 2,912562019 | 2,731588328 | 0,180973692 | |
| 219826_at | ZNF419 | 1,735773781 | 1,55481039 | 0,180963391 | |
| 225799_at | LINC00152 /// LC | 5,483891762 | 5,302953328 | 0,180938434 | |
| 200722_s_at | CAPRIN1 | 4,300164033 | 4,119256941 | 0,180907092 | |
| 208634_s_at | MACF1 | 3,906268218 | 3,725370335 | 0,180897883 | |
| 215600_x_at | FBXW12 | 2,213529682 | 2,032717854 | 0,180811828 | |
| 1569142_at | TRIM13 | 0,020670649 | -0,160136748 | 0,180807398 | |
| 207555_s_at | TBXA2R | 0,020670649 | -0,160136748 | 0,180807398 | |
| 217395_at | MT4 | 0,020670649 | -0,160136748 | 0,180807398 | |
| 242725_at | - | 1,168140757 | 0,987376598 | 0,18076416 | |
| 212513_s_at | USP33 | 4,702455242 | 4,52176814 | 0,180687102 | |
| 205401_at | AGPS | 3,248232277 | 3,067567462 | 0,180664814 | |

| | | | | | |
|---|---|---|---|---|---|
| 1559530_at | - | 0,384950573 | 0,204310755 | 0,180639818 | |
| 217164_at | - | 0,384950573 | 0,204310755 | 0,180639818 | |
| 227380_x_at | C16orf13 | 0,384950573 | 0,204310755 | 0,180639818 | |
| 37424_at | CCHCR1 | 1,94340061 | 1,762812816 | 0,180587794 | |
| 220165_at | INO80D | 0,67554678 | 0,495031851 | 0,180514929 | |
| 234728_s_at | DHX35 | 0,67554678 | 0,495031851 | 0,180514929 | |
| 213309_at | PLCL2 | 2,303618506 | 2,123108223 | 0,180510283 | |
| 214151_s_at | CCPG1 /// DYX1( | 1,261018186 | 1,080561626 | 0,18045656 | |
| 203777_s_at | RPS6KB2 | 0,917308734 | 0,73688026 | 0,180428474 | |
| 217051_s_at | - | 0,917308734 | 0,73688026 | 0,180428474 | |
| 234976_x_at | MTHFD2 | 0,917308734 | 0,73688026 | 0,180428474 | |
| 218479_s_at | XPO4 | 1,426552034 | 1,246149888 | 0,180402146 | |
| 201948_at | GNL2 | 3,619279389 | 3,439026336 | 0,180253053 | |
| 212995_x_at | MZT2A /// MZT2E | 4,471257399 | 4,291026333 | 0,180231066 | |
| 212084_at | TEX261 | 1,504602299 | 1,324440845 | 0,180161454 | |
| 202890_at | MAP7 | 2,001086023 | 1,820984782 | 0,180101241 | |
| 1554686_at | STAU2 | -2,667025278 | -2,847001814 | 0,179976537 | |
| 1555210_at | DTWD1 | -2,667025278 | -2,847001814 | 0,179976537 | |
| 1557580_at | - | -2,667025278 | -2,847001814 | 0,179976537 | |
| 1557597_at | LOC100289211 | -2,667025278 | -2,847001814 | 0,179976537 | |
| 1560265_at | GRIK2 | -2,667025278 | -2,847001814 | 0,179976537 | |
| 1560543_at | GRK4 | -2,667025278 | -2,847001814 | 0,179976537 | |
| 1563099_at | LOC100507053 | -2,667025278 | -2,847001814 | 0,179976537 | |
| 1565698_at | HECTD2 | -2,667025278 | -2,847001814 | 0,179976537 | |
| 1566190_at | SUZ12 | -2,667025278 | -2,847001814 | 0,179976537 | |
| 1570020_at | AACSP1 | -2,667025278 | -2,847001814 | 0,179976537 | |
| 211187_at | - | -2,667025278 | -2,847001814 | 0,179976537 | |
| 216136_at | - | -2,667025278 | -2,847001814 | 0,179976537 | |
| 216712_at | SLC25A30 | -2,667025278 | -2,847001814 | 0,179976537 | |
| 240848_at | - | -2,667025278 | -2,847001814 | 0,179976537 | |
| 241873_at | - | -2,667025278 | -2,847001814 | 0,179976537 | |
| 243081_at | LOC100652770 | -2,667025278 | -2,847001814 | 0,179976537 | |
| 243678_at | - | -2,667025278 | -2,847001814 | 0,179976537 | |
| 244295_at | - | -2,667025278 | -2,847001814 | 0,179976537 | |
| 244314_at | - | -2,667025278 | -2,847001814 | 0,179976537 | |
| 226224_at | FOXK2 | 2,410880861 | 2,230931691 | 0,17994917 | |
| 221264_s_at | TARDBP | 1,578645857 | 1,398700978 | 0,179944879 | |
| 203995_at | C21orf2 | 0,613492853 | 0,433557298 | 0,179935555 | |
| 224092_at | BARHL1 | 0,613492853 | 0,433557298 | 0,179935555 | |
| 1556345_s_at | - | 0,30867765 | 0,128747141 | 0,179930509 | |
| 1558782_a_at | LOC100130557 | 0,30867765 | 0,128747141 | 0,179930509 | |
| 214411_x_at | CTRB2 | 0,30867765 | 0,128747141 | 0,179930509 | |
| 222823_at | IPPK | 0,30867765 | 0,128747141 | 0,179930509 | |
| 200004_at | EIF4G2 | 6,906545318 | 6,726680737 | 0,17986458 | |
| 1552335_at | CATSPER1 | -0,078265071 | -0,258114234 | 0,179849162 | |
| 208229_at | FGFR2 | -0,078265071 | -0,258114234 | 0,179849162 | |
| 222648_at | TBC1D16 | -0,078265071 | -0,258114234 | 0,179849162 | |
| 229369_at | VSIG2 | -0,078265071 | -0,258114234 | 0,179849162 | |
| 232619_at | ACTL10 | -0,078265071 | -0,258114234 | 0,179849162 | |
| 202675_at | SDHB | 4,462972394 | 4,283151707 | 0,179820686 | |
| 207620_s_at | CASK | 1,129248302 | 0,949447784 | 0,179800518 | |
| 235589_s_at | MDM4 | 1,649074067 | 1,469325098 | 0,179748969 | |
| 213175_s_at | SNRPB | 6,886141249 | 6,706467992 | 0,179673258 | |
| 1554864_a_at | SDC3 | -0,467091869 | -0,646746079 | 0,17965421 | |
| 1557590_at | PARD6G-AS1 | -0,467091869 | -0,646746079 | 0,17965421 | |
| 201287_s_at | SDC1 | -0,467091869 | -0,646746079 | 0,17965421 | |

| | | | | | |
|---|---|---|---|---|---|
| 209979_at | ADARB1 | -0,467091869 | -0,646746079 | 0,17965421 | |
| 221139_s_at | CSAD | -0,467091869 | -0,646746079 | 0,17965421 | |
| 224501_at | C1orf170 | -0,467091869 | -0,646746079 | 0,17965421 | |
| 234365_at | - | -0,467091869 | -0,646746079 | 0,17965421 | |
| 240012_at | - | -0,467091869 | -0,646746079 | 0,17965421 | |
| 244726_at | - | -0,467091869 | -0,646746079 | 0,17965421 | |
| 209487_at | RBPMS | 0,747935635 | 0,568365095 | 0,17957054 | |
| 216902_s_at | RRN3 /// RRN3P: | 2,993291442 | 2,813804482 | 0,17948696 | |
| 210970_s_at | IBTK | 4,156452062 | 3,977034308 | 0,179417755 | |
| 32062_at | LRRC14 | 1,473887017 | 1,294497133 | 0,179389884 | |
| 1565537_at | NKX1-1 | 0,133056859 | -0,046302147 | 0,179359005 | |
| 1569106_s_at | SETD5 | 0,133056859 | -0,046302147 | 0,179359005 | |
| 213406_at | WSB1 | 0,133056859 | -0,046302147 | 0,179359005 | |
| 224832_at | DUSP16 | 0,133056859 | -0,046302147 | 0,179359005 | |
| 1555762_s_at | RBM15 | 3,416167722 | 3,236821511 | 0,179346211 | |
| 207156_at | HIST1H2AG /// H | 2,416948786 | 2,237691445 | 0,179257341 | |
| 228916_at | CWF19L2 | 2,109966786 | 1,930732138 | 0,179234649 | |
| 204979_s_at | SH3BGR | 1,549482847 | 1,370274469 | 0,179208378 | |
| 209836_x_at | BOLA2 /// BOLA2 | 5,149552753 | 4,970362234 | 0,179190519 | |
| 218382_s_at | U2AF2 | 2,857110344 | 2,677924589 | 0,179185754 | |
| 201885_s_at | CYB5R3 | 4,504404673 | 4,32527877 | 0,179125903 | |
| 214306_at | OPA1 | 2,47626436 | 2,297144887 | 0,179119473 | |
| 225745_at | LRP6 | 2,970093718 | 2,791007185 | 0,179086534 | |
| 1553172_at | ZNF777 | 2,901063789 | 2,721978587 | 0,179085203 | |
| 212505_s_at | MAU2 | 2,671625168 | 2,492544876 | 0,179080292 | |
| 212247_at | NUP205 | 4,933058485 | 4,754031948 | 0,179026537 | |
| 222318_at | ZNF324B | 0,984222186 | 0,805216064 | 0,179006122 | |
| 208691_at | TFRC | 7,787148788 | 7,60818245 | 0,178966338 | |
| 223319_at | GPHN | 3,037284587 | 2,858346012 | 0,178938575 | |
| 220007_at | METTL8 | 1,515954005 | 1,337086025 | 0,17886798 | |
| 226197_at | AR | 2,959046717 | 2,780243895 | 0,178802822 | |
| 214266_s_at | PDLIM7 | 0,401366113 | 0,222599095 | 0,178767018 | |
| 215281_x_at | POGZ | 0,401366113 | 0,222599095 | 0,178767018 | |
| 219499_at | SEC61A2 | 0,401366113 | 0,222599095 | 0,178767018 | |
| 223316_at | CCDC3 | 0,401366113 | 0,222599095 | 0,178767018 | |
| 224864_at | SRA1 | 3,319065909 | 3,140317145 | 0,178748764 | |
| 219382_at | SERTAD3 | 2,232003119 | 2,053299695 | 0,178703424 | |
| 226840_at | H2AFY | 1,40228911 | 1,223626587 | 0,178662522 | |
| 204387_x_at | MRP63 | 2,16388051 | 1,98530622 | 0,17857429 | |
| 214281_s_at | RCHY1 | 4,11599613 | 3,937445604 | 0,178550526 | |
| 224802_at | NDFIP2 | 3,891817836 | 3,713295978 | 0,178521858 | |
| 239386_at | HAVCR1P1 | 0,555999837 | 0,377530647 | 0,17846919 | |
| 236312_at | - | 1,556829052 | 1,378453643 | 0,178375408 | |
| 222990_at | UBQLN1 | 4,864780677 | 4,686464402 | 0,178316275 | |
| 224315_at | DDX20 | 1,820612054 | 1,642321262 | 0,178290792 | |
| 1569793_at | SLC25A18 | -0,83191556 | -1,010192375 | 0,178276815 | |
| 202999_s_at | LOXL2 | -0,83191556 | -1,010192375 | 0,178276815 | |
| 211868_x_at | IGH@ /// IGHA1 / | -0,83191556 | -1,010192375 | 0,178276815 | |
| 216890_at | - | -0,83191556 | -1,010192375 | 0,178276815 | |
| 225739_at | RAB11FIP4 | -0,83191556 | -1,010192375 | 0,178276815 | |
| 225815_at | CPLX2 | -0,83191556 | -1,010192375 | 0,178276815 | |
| 229034_at | SOBP | -0,83191556 | -1,010192375 | 0,178276815 | |
| 233286_at | - | -0,83191556 | -1,010192375 | 0,178276815 | |
| 239262_at | - | -0,83191556 | -1,010192375 | 0,178276815 | |
| 239734_at | - | -0,83191556 | -1,010192375 | 0,178276815 | |
| 241600_at | WIPF3 | -0,83191556 | -1,010192375 | 0,178276815 | |

| | | | | | |
|---|---|---|---|---|---|
| 242184_s_at | - | -0,83191556 | -1,010192375 | 0,178276815 | |
| 244520_at | - | -0,83191556 | -1,010192375 | 0,178276815 | |
| 218799_at | GPN2 | 2,043538949 | 1,865308541 | 0,178230408 | |
| 207499_x_at | UNC45A | 0,695652136 | 0,51742585 | 0,178226286 | |
| 219618_at | IRAK4 | 0,695652136 | 0,51742585 | 0,178226286 | |
| 221529_s_at | PLVAP | 0,695652136 | 0,51742585 | 0,178226286 | |
| 231258_at | LOC100653229 | 0,695652136 | 0,51742585 | 0,178226286 | |
| 208918_s_at | NADK | 4,069245099 | 3,891034012 | 0,178211087 | |
| 1553978_at | MEF2BNB | 1,880387527 | 1,702196344 | 0,178191183 | |
| 217981_s_at | FXC1 | 2,540666392 | 2,362515214 | 0,178151178 | |
| 1557802_at | - | -2,712026959 | -2,89013181 | 0,178104851 | |
| 1568190_at | - | -2,712026959 | -2,89013181 | 0,178104851 | |
| 1569186_at | MYO3A | -2,712026959 | -2,89013181 | 0,178104851 | |
| 1570471_at | ARHGEF12 | -2,712026959 | -2,89013181 | 0,178104851 | |
| 210931_at | RNF6 | -2,712026959 | -2,89013181 | 0,178104851 | |
| 217392_at | - | -2,712026959 | -2,89013181 | 0,178104851 | |
| 217506_at | - | -2,712026959 | -2,89013181 | 0,178104851 | |
| 217537_x_at | - | -2,712026959 | -2,89013181 | 0,178104851 | |
| 223962_at | TTC29 | -2,712026959 | -2,89013181 | 0,178104851 | |
| 230783_at | LOC283713 | -2,712026959 | -2,89013181 | 0,178104851 | |
| 233682_at | - | -2,712026959 | -2,89013181 | 0,178104851 | |
| 236280_at | - | -2,712026959 | -2,89013181 | 0,178104851 | |
| 236575_at | ARHGEF26-AS1 | -2,712026959 | -2,89013181 | 0,178104851 | |
| 240735_at | CDC42BPA | -2,712026959 | -2,89013181 | 0,178104851 | |
| 240980_at | - | -2,712026959 | -2,89013181 | 0,178104851 | |
| 241194_at | - | -2,712026959 | -2,89013181 | 0,178104851 | |
| 243125_x_at | - | -2,712026959 | -2,89013181 | 0,178104851 | |
| 244749_at | - | -2,712026959 | -2,89013181 | 0,178104851 | |
| 235084_x_at | TRIM38 | 0,882651021 | 0,704715219 | 0,177935802 | |
| 51228_at | RBM12B | 1,730911019 | 1,553004872 | 0,177906147 | |
| 201385_at | DHX15 | 6,064530481 | 5,886682232 | 0,177848249 | |
| 226671_at | LAMP2 | 1,912238072 | 1,73441697 | 0,177821102 | |
| 1552257_a_at | TTLL12 | 2,763383302 | 2,585563171 | 0,177820131 | |
| 213437_at | RUFY3 | 1,369290453 | 1,191491281 | 0,177799172 | |
| 226206_at | MAFK | 0,995078803 | 0,817300337 | 0,177778466 | |
| 226270_at | EXOC2 | 2,171078719 | 1,993317152 | 0,177761567 | |
| 209003_at | SLC25A11 | 2,535098095 | 2,357351836 | 0,177746259 | |
| 1555961_a_at | HINT1 | 5,817728115 | 5,640280705 | 0,17744741 | |
| 222846_at | RAB8B | 3,19743497 | 3,019999252 | 0,177435717 | |
| 203234_at | UPP1 | 2,243430036 | 2,066015817 | 0,177414219 | |
| 232591_s_at | TMEM30A | 3,025418883 | 2,848075314 | 0,177343569 | |
| 1554266_at | - | -0,172274514 | -0,34958478 | 0,177310267 | |
| 1560006_a_at | LOC646762 | -0,172274514 | -0,34958478 | 0,177310267 | |
| 1560065_at | PAIP2 | -0,172274514 | -0,34958478 | 0,177310267 | |
| 214443_at | PVR | -0,172274514 | -0,34958478 | 0,177310267 | |
| 219319_at | HIF3A | -0,172274514 | -0,34958478 | 0,177310267 | |
| 226162_at | SLC30A6 | -0,172274514 | -0,34958478 | 0,177310267 | |
| 228436_at | KCNC4 | -0,172274514 | -0,34958478 | 0,177310267 | |
| 234159_at | - | -0,172274514 | -0,34958478 | 0,177310267 | |
| 236224_at | RIT1 | -0,172274514 | -0,34958478 | 0,177310267 | |
| 241385_at | LARP7 | -0,172274514 | -0,34958478 | 0,177310267 | |
| 210418_s_at | IDH3B | 4,363324083 | 4,186016868 | 0,177307216 | |
| 203115_at | FECH | 0,945567441 | 0,768343791 | 0,177223651 | |
| 225685_at | CDC42EP3 | 3,164152611 | 2,986947093 | 0,177205518 | |
| 202123_s_at | ABL1 | 2,688444421 | 2,511262629 | 0,177181792 | |
| 201977_s_at | KIAA0141 | 0,152582607 | -0,024572586 | 0,177155193 | |

| | | | | | |
|---|---|---|---|---|---|
| 203439_s_at | STC2 | 0,152582607 | -0,024572586 | 0,177155193 | |
| 207641_at | TNFRSF13B | 0,152582607 | -0,024572586 | 0,177155193 | |
| 222277_at | C1QTNF9B-AS1 | 0,152582607 | -0,024572586 | 0,177155193 | |
| 243210_at | - | 0,152582607 | -0,024572586 | 0,177155193 | |
| 224200_s_at | RAD18 | 1,567778694 | 1,390636111 | 0,177142583 | |
| 1555867_at | GNG4 | 1,05337236 | 0,876248481 | 0,177123878 | |
| 223179_at | YPEL3 | 1,05337236 | 0,876248481 | 0,177123878 | |
| 237299_at | - | 2,396622324 | 2,21959457 | 0,177027753 | |
| 218175_at | CCDC92 | 1,177702228 | 1,000684521 | 0,177017706 | |
| 227912_s_at | EXOSC3 | 1,177702228 | 1,000684521 | 0,177017706 | |
| 203792_x_at | PCGF2 | 0,334552598 | 0,157549243 | 0,177003355 | |
| 221555_x_at | CDC14B | 0,334552598 | 0,157549243 | 0,177003355 | |
| 235441_at | - | 0,334552598 | 0,157549243 | 0,177003355 | |
| 244498_x_at | LOC100505679 | 0,334552598 | 0,157549243 | 0,177003355 | |
| 221847_at | LOC100129361 | 4,381538373 | 4,204537151 | 0,177001222 | |
| 216342_x_at | - | 6,735067082 | 6,558078739 | 0,176988343 | |
| 203654_s_at | COIL | 3,648798054 | 3,471887144 | 0,17691091 | |
| 235302_at | C14orf118 | 1,534677255 | 1,3579181 | 0,176759155 | |
| 211561_x_at | MAPK14 | 1,607231019 | 1,43051703 | 0,176713989 | |
| 244663_at | - | 1,607231019 | 1,43051703 | 0,176713989 | |
| 225265_at | RBMS1 | 1,67631007 | 1,499637023 | 0,176673048 | |
| 201953_at | CIB1 | 4,044635894 | 3,86798237 | 0,176653524 | |
| 211033_s_at | PEX7 | 2,693452197 | 2,516830926 | 0,176621272 | |
| 1558906_a_at | LOC100505783 | -0,43775896 | -0,614371577 | 0,176612616 | |
| 207028_at | MYCNOS | -0,43775896 | -0,614371577 | 0,176612616 | |
| 213050_at | COBL | -0,43775896 | -0,614371577 | 0,176612616 | |
| 216552_x_at | KIR2DS4 | -0,43775896 | -0,614371577 | 0,176612616 | |
| 223755_at | KIRREL2 | -0,43775896 | -0,614371577 | 0,176612616 | |
| 230736_at | LOC387647 | -0,43775896 | -0,614371577 | 0,176612616 | |
| 234287_at | - | -0,43775896 | -0,614371577 | 0,176612616 | |
| 236636_at | - | -0,43775896 | -0,614371577 | 0,176612616 | |
| 237053_at | SYNDIG1L | -0,43775896 | -0,614371577 | 0,176612616 | |
| 241024_at | C6orf147 /// KHD | -0,43775896 | -0,614371577 | 0,176612616 | |
| 222605_at | RCOR3 | 1,894952274 | 1,718396606 | 0,176555668 | |
| 241751_at | OFD1 | 1,894952274 | 1,718396606 | 0,176555668 | |
| 226795_at | LRCH1 | 1,951783949 | 1,775255987 | 0,176527961 | |
| 226421_at | AMMECR1 | 5,46595713 | 5,289461962 | 0,176495167 | |
| 209679_s_at | SMAGP | 2,084778276 | 1,908311039 | 0,176467237 | |
| 227418_at | MSANTD4 | 3,171349466 | 2,994948944 | 0,176400522 | |
| 205167_s_at | CDC25C | 1,575032557 | 1,398700978 | 0,176331578 | |
| 221873_at | ZNF143 | 3,034656185 | 2,858346012 | 0,176310173 | |
| 223000_s_at | F11R | 4,213343045 | 4,037037849 | 0,176305196 | |
| 200989_at | HIF1A | 5,721216542 | 5,544941426 | 0,176275116 | |
| 200661_at | CTSA | 3,761974695 | 3,585762986 | 0,176211709 | |
| 213532_at | ADAM17 | 2,912562019 | 2,7363693 | 0,176192719 | |
| 202783_at | NNT | 2,208874124 | 2,032717854 | 0,17615627 | |
| 1557265_at | - | 0,255492549 | 0,079428135 | 0,176064414 | |
| 240221_at | CSNK1A1 | 0,255492549 | 0,079428135 | 0,176064414 | |
| 202266_at | TDP2 | 4,798543046 | 4,622479552 | 0,176063495 | |
| 217187_at | MUC5AC | 0,425644434 | 0,249604176 | 0,176040258 | |
| 227829_at | GYLTL1B | 0,425644434 | 0,249604176 | 0,176040258 | |
| 209137_s_at | USP10 | 3,076146581 | 2,900133867 | 0,176012714 | |
| 210778_s_at | MIR4800 /// MXD | 0,779676066 | 0,603679647 | 0,175996419 | |
| 212285_s_at | AGRN | 0,779676066 | 0,603679647 | 0,175996419 | |
| 230521_at | C9orf100 | 0,779676066 | 0,603679647 | 0,175996419 | |
| 215082_at | ELOVL5 | 0,900083947 | 0,724100169 | 0,175983778 | |

| | | | | | |
|---|---|---|---|---|---|
| 229856_s_at | PITHD1 | 1,163336153 | 0,987376598 | 0,175959556 | |
| 235147_at | - | 1,748661581 | 1,572742379 | 0,175919202 | |
| 221490_at | UBAP1 | 1,841816345 | 1,665902186 | 0,17591416 | |
| 212111_at | STX12 | 2,064305961 | 1,888402606 | 0,175903355 | |
| 1553967_at | ADAT3 | -0,285837228 | -0,461594427 | 0,175757198 | |
| 1566949_at | - | -0,285837228 | -0,461594427 | 0,175757198 | |
| 202398_at | AP3S2 /// C15orf: | -0,285837228 | -0,461594427 | 0,175757198 | |
| 207306_at | TCF15 | -0,285837228 | -0,461594427 | 0,175757198 | |
| 211215_x_at | DIO2 | -0,285837228 | -0,461594427 | 0,175757198 | |
| 213930_at | ATG12 | -0,285837228 | -0,461594427 | 0,175757198 | |
| 220122_at | MCTP1 | -0,285837228 | -0,461594427 | 0,175757198 | |
| 238816_at | PSEN1 | -0,285837228 | -0,461594427 | 0,175757198 | |
| 240129_at | - | -0,285837228 | -0,461594427 | 0,175757198 | |
| 218845_at | DUSP22 | 3,506195778 | 3,330438773 | 0,175757005 | |
| 202405_at | TIAL1 | 2,259277178 | 2,083632167 | 0,175645011 | |
| 1557954_at | TXLNG | 3,315822132 | 3,140317145 | 0,175504987 | |
| 1557065_at | YLPM1 | 1,390002893 | 1,214517879 | 0,175485015 | |
| 223072_s_at | INO80B /// INO8( | 1,390002893 | 1,214517879 | 0,175485015 | |
| 224976_at | NFIA | 1,390002893 | 1,214517879 | 0,175485015 | |
| 228283_at | CMC1 | 3,574742007 | 3,399383202 | 0,175358805 | |
| 212183_at | NUDT4 | 2,41290634 | 2,237691445 | 0,175214894 | |
| 236261_at | OSBPL6 | 2,41290634 | 2,237691445 | 0,175214894 | |
| 200736_s_at | GPX1 | 5,828105858 | 5,652979126 | 0,175126732 | |
| 1553654_at | SYT14 | -2,779459409 | -2,954576755 | 0,175117347 | |
| 1554492_at | THADA | -2,779459409 | -2,954576755 | 0,175117347 | |
| 1556417_a_at | - | -2,779459409 | -2,954576755 | 0,175117347 | |
| 1558971_at | THEMIS | -2,779459409 | -2,954576755 | 0,175117347 | |
| 1559890_a_at | ABI1 | -2,779459409 | -2,954576755 | 0,175117347 | |
| 1561289_at | LOC286370 | -2,779459409 | -2,954576755 | 0,175117347 | |
| 1561316_at | GABRB3 | -2,779459409 | -2,954576755 | 0,175117347 | |
| 1561606_at | - | -2,779459409 | -2,954576755 | 0,175117347 | |
| 1563033_x_at | - | -2,779459409 | -2,954576755 | 0,175117347 | |
| 1565346_a_at | ATP1A4 | -2,779459409 | -2,954576755 | 0,175117347 | |
| 1565596_at | - | -2,779459409 | -2,954576755 | 0,175117347 | |
| 1569405_at | - | -2,779459409 | -2,954576755 | 0,175117347 | |
| 1570015_at | RP11-165H20.1 | -2,779459409 | -2,954576755 | 0,175117347 | |
| 215457_at | - | -2,779459409 | -2,954576755 | 0,175117347 | |
| 219934_s_at | SULT1E1 | -2,779459409 | -2,954576755 | 0,175117347 | |
| 224148_at | FYB | -2,779459409 | -2,954576755 | 0,175117347 | |
| 232161_x_at | PTPN4 | -2,779459409 | -2,954576755 | 0,175117347 | |
| 232380_at | FGGY | -2,779459409 | -2,954576755 | 0,175117347 | |
| 232945_at | PUS10 | -2,779459409 | -2,954576755 | 0,175117347 | |
| 233910_at | TMEFF2 | -2,779459409 | -2,954576755 | 0,175117347 | |
| 239661_at | - | -2,779459409 | -2,954576755 | 0,175117347 | |
| 240395_at | LOC100128727 | -2,779459409 | -2,954576755 | 0,175117347 | |
| 1569396_at | RAB40C | 0,351548291 | 0,176436073 | 0,175112218 | |
| 215931_s_at | ARFGEF2 | 0,351548291 | 0,176436073 | 0,175112218 | |
| 1557986_s_at | SMCR8 | -1,089016378 | -1,264005341 | 0,174988963 | |
| 1563117_at | LOC388456 | -1,089016378 | -1,264005341 | 0,174988963 | |
| 227468_at | CPT1C | -1,089016378 | -1,264005341 | 0,174988963 | |
| 229413_s_at | - | -1,089016378 | -1,264005341 | 0,174988963 | |
| 234173_s_at | NXF2 /// NXF2B | -1,089016378 | -1,264005341 | 0,174988963 | |
| 236949_at | - | -1,089016378 | -1,264005341 | 0,174988963 | |
| 237752_at | - | -1,089016378 | -1,264005341 | 0,174988963 | |
| 238749_at | - | -1,089016378 | -1,264005341 | 0,174988963 | |
| 238788_at | LOC494150 | -1,089016378 | -1,264005341 | 0,174988963 | |

| | | | | | |
|---|---|---|---|---|---|
| 241783_at | - | -1,089016378 | -1,264005341 | 0,174988963 | |
| 221497_x_at | EGLN1 | 1,965648824 | 1,790660532 | 0,174988292 | |
| 1553148_a_at | SNX13 | -0,59053611 | -0,765504029 | 0,174967919 | |
| 1567253_at | OR10D3 | -0,59053611 | -0,765504029 | 0,174967919 | |
| 1568742_at | - | -0,59053611 | -0,765504029 | 0,174967919 | |
| 200671_s_at | SPTBN1 | -0,59053611 | -0,765504029 | 0,174967919 | |
| 203541_s_at | KLF9 | -0,59053611 | -0,765504029 | 0,174967919 | |
| 203685_at | BCL2 | -0,59053611 | -0,765504029 | 0,174967919 | |
| 204553_x_at | INPP4A | -0,59053611 | -0,765504029 | 0,174967919 | |
| 209950_s_at | VILL | -0,59053611 | -0,765504029 | 0,174967919 | |
| 212278_x_at | UBE3A | -0,59053611 | -0,765504029 | 0,174967919 | |
| 227287_at | CITED2 | -0,59053611 | -0,765504029 | 0,174967919 | |
| 232030_at | EPG5 | -0,59053611 | -0,765504029 | 0,174967919 | |
| 236501_at | - | -0,59053611 | -0,765504029 | 0,174967919 | |
| 241842_x_at | C19orf45 | -0,59053611 | -0,765504029 | 0,174967919 | |
| 221346_at | OR10J1 | 0,072842263 | -0,102100538 | 0,1749428 | |
| 238340_at | DCAF8 | 0,072842263 | -0,102100538 | 0,1749428 | |
| 241427_x_at | - | 0,072842263 | -0,102100538 | 0,1749428 | |
| 241666_at | C3orf23 | 0,072842263 | -0,102100538 | 0,1749428 | |
| 1553096_s_at | BCL2L11 | 2,266015882 | 2,091116676 | 0,174899206 | |
| 212119_at | RHOQ | 1,789769151 | 1,614891062 | 0,174878089 | |
| 225614_at | SAAL1 | 3,266269388 | 3,091398163 | 0,174871225 | |
| 209375_at | XPC | 2,173470163 | 1,998633167 | 0,174836996 | |
| 218661_at | NAA60 | 2,173470163 | 1,998633167 | 0,174836996 | |
| 222583_s_at | NUP50 | 1,69307511 | 1,518263577 | 0,174811533 | |
| 215227_x_at | ACP1 | 5,278173421 | 5,10337687 | 0,174796551 | |
| 230542_at | ZNF597 | 1,021868205 | 0,847075464 | 0,174792741 | |
| 226532_at | - | 2,710020346 | 2,53523821 | 0,174782136 | |
| 202362_at | RAP1A | 4,94360513 | 4,768885987 | 0,174719143 | |
| 1554286_at | FLJ25758 | -0,148196756 | -0,322870281 | 0,174673525 | |
| 215857_at | NCLN | -0,148196756 | -0,322870281 | 0,174673525 | |
| 217629_at | - | -0,148196756 | -0,322870281 | 0,174673525 | |
| 221945_at | FBXO41 | -0,148196756 | -0,322870281 | 0,174673525 | |
| 232659_at | - | -0,148196756 | -0,322870281 | 0,174673525 | |
| 235058_at | - | -0,148196756 | -0,322870281 | 0,174673525 | |
| 241849_at | - | -0,148196756 | -0,322870281 | 0,174673525 | |
| 242806_at | - | -0,148196756 | -0,322870281 | 0,174673525 | |
| 224654_at | DDX21 | 6,187498665 | 6,012888334 | 0,174610332 | |
| 205269_at | LCP2 | 2,883642771 | 2,709065209 | 0,174577561 | |
| 1552531_a_at | NLRP11 | -1,688127507 | -1,862647763 | 0,174520256 | |
| 1553254_at | MAGEE2 | -1,688127507 | -1,862647763 | 0,174520256 | |
| 1558470_at | VWA3B | -1,688127507 | -1,862647763 | 0,174520256 | |
| 1559309_at | MCFD2 | -1,688127507 | -1,862647763 | 0,174520256 | |
| 1560253_at | LHX9 | -1,688127507 | -1,862647763 | 0,174520256 | |
| 1561894_at | C7orf49 /// LOC6 | -1,688127507 | -1,862647763 | 0,174520256 | |
| 1569789_at | - | -1,688127507 | -1,862647763 | 0,174520256 | |
| 1570158_at | - | -1,688127507 | -1,862647763 | 0,174520256 | |
| 203980_at | FABP4 | -1,688127507 | -1,862647763 | 0,174520256 | |
| 206736_x_at | CHRNA4 | -1,688127507 | -1,862647763 | 0,174520256 | |
| 206815_at | SPAG8 | -1,688127507 | -1,862647763 | 0,174520256 | |
| 206996_x_at | CACNB1 | -1,688127507 | -1,862647763 | 0,174520256 | |
| 216281_at | DICER1 | -1,688127507 | -1,862647763 | 0,174520256 | |
| 220414_at | CALML5 | -1,688127507 | -1,862647763 | 0,174520256 | |
| 220580_at | BICC1 | -1,688127507 | -1,862647763 | 0,174520256 | |
| 222328_x_at | - | -1,688127507 | -1,862647763 | 0,174520256 | |
| 229584_at | LRRK2 | -1,688127507 | -1,862647763 | 0,174520256 | |

| | | | | | |
|---|---|---|---|---|---|
| 231532_at | - | -1,688127507 | -1,862647763 | 0,174520256 | |
| 231535_x_at | ROPN1 | -1,688127507 | -1,862647763 | 0,174520256 | |
| 232006_at | STK35 | -1,688127507 | -1,862647763 | 0,174520256 | |
| 235016_at | REEP3 | -1,688127507 | -1,862647763 | 0,174520256 | |
| 237662_at | - | -1,688127507 | -1,862647763 | 0,174520256 | |
| 239584_at | - | -1,688127507 | -1,862647763 | 0,174520256 | |
| 240945_at | - | -1,688127507 | -1,862647763 | 0,174520256 | |
| 244494_at | - | -1,688127507 | -1,862647763 | 0,174520256 | |
| 203594_at | RTCA | 4,63204053 | 4,457591096 | 0,174449434 | |
| 235514_at | ASPRV1 | 0,592200562 | 0,417770464 | 0,174430098 | |
| 212899_at | CDK19 | 2,400710591 | 2,226407528 | 0,174303063 | |
| 205521_at | EXOG | 0,44160593 | 0,26733076 | 0,17427517 | |
| 226643_s_at | NUDCD2 | 1,079110292 | 0,904843258 | 0,174267034 | |
| 227112_at | TMCC1 | 1,079110292 | 0,904843258 | 0,174267034 | |
| 226816_s_at | KIAA1143 | 4,007586951 | 3,833443169 | 0,174143782 | |
| 211922_s_at | CAT | 3,413135307 | 3,239069096 | 0,174066211 | |
| 206756_at | CHST7 | 1,318332599 | 1,144305291 | 0,174027308 | |
| 228185_at | ZNF25 | 0,181384709 | 0,007421914 | 0,173962795 | |
| 214007_s_at | TWF1 | 0,798390451 | 0,624460759 | 0,173929692 | |
| 233250_x_at | FOXRED2 | 0,798390451 | 0,624460759 | 0,173929692 | |
| 236229_at | - | 0,798390451 | 0,624460759 | 0,173929692 | |
| 212774_at | ZNF238 | 2,750626925 | 2,576704359 | 0,173922565 | |
| 216505_x_at | - | 5,986266443 | 5,812405975 | 0,173860468 | |
| 202678_at | GTF2A2 | 5,015848483 | 4,842054614 | 0,173793869 | |
| 222126_at | AGFG2 | 1,735773781 | 1,562009954 | 0,173763827 | |
| 217945_at | BTBD1 | 5,081147701 | 4,907457724 | 0,173689977 | |
| 211208_s_at | CASK | 2,562727117 | 2,389070873 | 0,173656244 | |
| 1556248_at | FLJ33065 | -0,022466681 | -0,19610998 | 0,173643299 | |
| 1560779_a_at | ZNF568 | -0,022466681 | -0,19610998 | 0,173643299 | |
| 215607_x_at | - | -0,022466681 | -0,19610998 | 0,173643299 | |
| 239017_at | - | -0,022466681 | -0,19610998 | 0,173643299 | |
| 203926_x_at | ATP5D | 2,185368281 | 2,011838132 | 0,173530148 | |
| 226399_at | DNAJB14 | 2,185368281 | 2,011838132 | 0,173530148 | |
| 206976_s_at | HSPH1 | 5,893211151 | 5,719683585 | 0,173527566 | |
| 217858_s_at | ARMCX3 | 2,582657321 | 2,409170778 | 0,173486543 | |
| 209787_s_at | HMGN4 | 5,262252783 | 5,088808057 | 0,173444726 | |
| 1552628_a_at | HERPUD2 | 1,923647937 | 1,750261389 | 0,173386548 | |
| 203140_at | BCL6 | 1,187200746 | 1,013870809 | 0,173329937 | |
| 219237_s_at | DNAJB14 | 1,672933557 | 1,499637023 | 0,173296534 | |
| 201761_at | MTHFD2 | 5,605105329 | 5,431842482 | 0,173262847 | |
| 1561676_at | PRICKLE2-AS3 | -2,823166348 | -2,996335703 | 0,173169355 | |
| 1565743_at | - | -2,823166348 | -2,996335703 | 0,173169355 | |
| 1566101_at | TTLL5 | -2,823166348 | -2,996335703 | 0,173169355 | |
| 1567248_at | OR9A1P | -2,823166348 | -2,996335703 | 0,173169355 | |
| 1569858_at | - | -2,823166348 | -2,996335703 | 0,173169355 | |
| 1570082_x_at | - | -2,823166348 | -2,996335703 | 0,173169355 | |
| 214603_at | MAGEA2 /// MAG | -2,823166348 | -2,996335703 | 0,173169355 | |
| 234097_s_at | ZNRD1-AS1 | -2,823166348 | -2,996335703 | 0,173169355 | |
| 237250_at | - | -2,823166348 | -2,996335703 | 0,173169355 | |
| 238732_at | COL24A1 | -2,823166348 | -2,996335703 | 0,173169355 | |
| 238922_at | - | -2,823166348 | -2,996335703 | 0,173169355 | |
| 240649_at | - | -2,823166348 | -2,996335703 | 0,173169355 | |
| 200624_s_at | MATR3 /// SNHG | 5,710552306 | 5,537427697 | 0,17312461 | |
| 227792_at | ITPRIPL2 | 3,115233629 | 2,942125533 | 0,173108096 | |
| 225838_at | EPC2 | 2,86748657 | 2,694398145 | 0,173088426 | |
| 223569_at | PPAPDC1B | 1,037706275 | 0,864649967 | 0,173056308 | |

| | | | | | |
|---|---|---|---|---|---|
| 217996_at | PHLDA1 | 4,67529113 | 4,502251705 | 0,173039426 | |
| 204604_at | CDK14 | 1,530951994 | 1,3579181 | 0,173033894 | |
| 210676_x_at | RGPD3 /// RGPD | 1,530951994 | 1,3579181 | 0,173033894 | |
| 218854_at | DSE | 1,530951994 | 1,3579181 | 0,173033894 | |
| 201001_s_at | TMEM189 /// TMI | 4,511983428 | 4,338964815 | 0,173018613 | |
| 202646_s_at | CSDE1 | 4,92704765 | 4,754031948 | 0,173015702 | |
| 202283_at | SERPINF1 | 1,709647566 | 1,536652708 | 0,172994858 | |
| 218248_at | FAM111A | 2,166283906 | 1,993317152 | 0,172966754 | |
| 225886_at | DDX5 | 2,605861638 | 2,432926755 | 0,172934883 | |
| 229625_at | GBP5 | 1,238353345 | 1,065442793 | 0,172910552 | |
| 221650_s_at | MED18 | 1,454352496 | 1,281471383 | 0,172881113 | |
| 1553642_at | C9orf163 | 0,606430281 | 0,433557298 | 0,172872983 | |
| 1555779_a_at | CD79A | 0,606430281 | 0,433557298 | 0,172872983 | |
| 214729_at | TWISTNB | 0,606430281 | 0,433557298 | 0,172872983 | |
| 216271_x_at | SYDE1 | 0,606430281 | 0,433557298 | 0,172872983 | |
| 239294_at | - | 0,606430281 | 0,433557298 | 0,172872983 | |
| 220941_s_at | C21orf91 | 0,928678725 | 0,755840599 | 0,172838125 | |
| 233101_at | MTMR9 | 0,928678725 | 0,755840599 | 0,172838125 | |
| 225022_at | GOPC | 3,642769857 | 3,469974737 | 0,17279512 | |
| 202360_at | MAML1 | 3,330897748 | 3,158242412 | 0,172655336 | |
| 205486_at | TESK2 | 2,014487247 | 1,841838775 | 0,172648473 | |
| 222653_at | PNPO | 2,014487247 | 1,841838775 | 0,172648473 | |
| 230504_at | CEACAM19 | 1,094335495 | 0,921731975 | 0,17260352 | |
| 238678_at | FLJ45340 | 1,094335495 | 0,921731975 | 0,17260352 | |
| 203116_s_at | FECH | 2,535098095 | 2,362515214 | 0,172582882 | |
| 1555631_at | - | -1,047650486 | -1,220210588 | 0,172560102 | |
| 1564264_at | - | -1,047650486 | -1,220210588 | 0,172560102 | |
| 205957_at | PLXNB3 | -1,047650486 | -1,220210588 | 0,172560102 | |
| 206532_at | - | -1,047650486 | -1,220210588 | 0,172560102 | |
| 208320_at | CABP1 | -1,047650486 | -1,220210588 | 0,172560102 | |
| 215488_at | - | -1,047650486 | -1,220210588 | 0,172560102 | |
| 221456_at | TAS2R3 | -1,047650486 | -1,220210588 | 0,172560102 | |
| 224307_x_at | MAGI3 | -1,047650486 | -1,220210588 | 0,172560102 | |
| 230124_at | PRKCH | -1,047650486 | -1,220210588 | 0,172560102 | |
| 230816_at | FAM163B | -1,047650486 | -1,220210588 | 0,172560102 | |
| 235850_at | FAM162A | -1,047650486 | -1,220210588 | 0,172560102 | |
| 237434_x_at | MSANTD1 | -1,047650486 | -1,220210588 | 0,172560102 | |
| 244309_at | - | -1,047650486 | -1,220210588 | 0,172560102 | |
| 244417_at | - | -1,047650486 | -1,220210588 | 0,172560102 | |
| 211931_s_at | HNRNPA3 /// HN | 6,057264737 | 5,884709689 | 0,172555048 | |
| 200864_s_at | RAB11A | 3,253893136 | 3,081412119 | 0,172481016 | |
| 228305_at | ZNF565 | 2,55541096 | 2,382985886 | 0,172425074 | |
| 1553163_at | ADPRHL1 | 0,376672223 | 0,204310755 | 0,172361468 | |
| 243263_at | LINC00482 | 0,376672223 | 0,204310755 | 0,172361468 | |
| 244872_at | RBBP4 | 0,376672223 | 0,204310755 | 0,172361468 | |
| 223978_s_at | CRLS1 | 4,84391367 | 4,671564559 | 0,172349111 | |
| 218172_s_at | DERL1 | 4,147356641 | 3,975013041 | 0,1723436 | |
| 217866_at | CPSF7 | 3,912008086 | 3,739727424 | 0,172280662 | |
| 230110_at | MCOLN2 | 1,716223609 | 1,543943227 | 0,172280381 | |
| 33760_at | PEX14 | 2,439975571 | 2,267724404 | 0,172251167 | |
| 217874_at | SUCLG1 | 4,837895809 | 4,665728361 | 0,172167447 | |
| 226896_at | CHCHD1 | 5,044219854 | 4,872052597 | 0,172167257 | |
| 1557174_a_at | - | -0,394749123 | -0,566887459 | 0,172138336 | |
| 1558410_s_at | - | -0,394749123 | -0,566887459 | 0,172138336 | |
| 209619_at | CD74 | -0,394749123 | -0,566887459 | 0,172138336 | |
| 212019_at | RSL1D1 | -0,394749123 | -0,566887459 | 0,172138336 | |

| | | | | | |
|---|---|---|---|---|---|
| 223665_at | ACTRT3 | -0,394749123 | -0,566887459 | 0,172138336 | |
| 237137_at | SCARNA2 | -0,394749123 | -0,566887459 | 0,172138336 | |
| 241985_at | JMY | -0,394749123 | -0,566887459 | 0,172138336 | |
| 36552_at | C2CD3 | 1,684716943 | 1,512700819 | 0,172016124 | |
| 201651_s_at | PACSIN2 | 5,014014911 | 4,842054614 | 0,171960297 | |
| 1552982_a_at | FGF4 | -2,236765673 | -2,408705138 | 0,171939465 | |
| 1556297_a_at | - | -2,236765673 | -2,408705138 | 0,171939465 | |
| 1556602_at | - | -2,236765673 | -2,408705138 | 0,171939465 | |
| 1561604_at | - | -2,236765673 | -2,408705138 | 0,171939465 | |
| 1563478_at | KIAA1671 | -2,236765673 | -2,408705138 | 0,171939465 | |
| 205498_at | GHR | -2,236765673 | -2,408705138 | 0,171939465 | |
| 206134_at | ADAMDEC1 | -2,236765673 | -2,408705138 | 0,171939465 | |
| 210942_s_at | ST3GAL6 | -2,236765673 | -2,408705138 | 0,171939465 | |
| 215625_at | LOC644450 | -2,236765673 | -2,408705138 | 0,171939465 | |
| 215928_at | - | -2,236765673 | -2,408705138 | 0,171939465 | |
| 220141_at | C11orf63 | -2,236765673 | -2,408705138 | 0,171939465 | |
| 222449_at | PMEPA1 | -2,236765673 | -2,408705138 | 0,171939465 | |
| 236375_at | - | -2,236765673 | -2,408705138 | 0,171939465 | |
| 237822_at | - | -2,236765673 | -2,408705138 | 0,171939465 | |
| 239229_at | PHEX | -2,236765673 | -2,408705138 | 0,171939465 | |
| 241888_at | - | -2,236765673 | -2,408705138 | 0,171939465 | |
| 243518_at | - | -2,236765673 | -2,408705138 | 0,171939465 | |
| 244392_at | - | -2,236765673 | -2,408705138 | 0,171939465 | |
| 242297_at | RREB1 | 1,04816921 | 0,876248481 | 0,171920729 | |
| 213013_at | LOC644172 /// M | 0,20027154 | 0,028364014 | 0,171907525 | |
| 226022_at | SASH1 | 0,20027154 | 0,028364014 | 0,171907525 | |
| 236243_at | ZCCHC6 | 0,20027154 | 0,028364014 | 0,171907525 | |
| 207667_s_at | MAP2K3 | 1,937784532 | 1,765933685 | 0,171850847 | |
| 223296_at | SLC25A33 | 4,055697197 | 3,883869861 | 0,171827336 | |
| 205750_at | BPHL | 1,201332287 | 1,029536893 | 0,171795394 | |
| 219506_at | C1orf54 | 0,46522187 | 0,293518836 | 0,171703035 | |
| 222236_s_at | ASAP3 | 0,46522187 | 0,293518836 | 0,171703035 | |
| 223123_s_at | PITHD1 | 0,46522187 | 0,293518836 | 0,171703035 | |
| 231131_at | FAM133A | 0,46522187 | 0,293518836 | 0,171703035 | |
| 37586_at | ZNF142 | 0,722031324 | 0,55037832 | 0,171653004 | |
| 210386_s_at | MTX1 | 3,296204798 | 3,124599694 | 0,171605104 | |
| 223397_s_at | NIP7 | 3,790298086 | 3,618934391 | 0,171363695 | |
| 212908_at | DNAJC16 | 2,724770722 | 2,553413592 | 0,17135713 | |
| 224247_s_at | MRPS10 | 3,418185797 | 3,246908183 | 0,171277614 | |
| 227770_at | COG8 | 1,726031811 | 1,55481039 | 0,17122142 | |
| 201218_at | CTBP2 | 4,538199871 | 4,36698147 | 0,1712184 | |
| 1552587_at | CNBD1 | -2,866296344 | -3,037500868 | 0,171204524 | |
| 1554355_a_at | SIAE | -2,866296344 | -3,037500868 | 0,171204524 | |
| 1557871_at | LOC253573 | -2,866296344 | -3,037500868 | 0,171204524 | |
| 1559124_at | LOC644135 | -2,866296344 | -3,037500868 | 0,171204524 | |
| 1560237_at | - | -2,866296344 | -3,037500868 | 0,171204524 | |
| 1560813_at | - | -2,866296344 | -3,037500868 | 0,171204524 | |
| 1568711_a_at | LOC100507498 | -2,866296344 | -3,037500868 | 0,171204524 | |
| 218804_at | ANO1 | -2,866296344 | -3,037500868 | 0,171204524 | |
| 232108_at | SCRN3 | -2,866296344 | -3,037500868 | 0,171204524 | |
| 243275_at | - | -2,866296344 | -3,037500868 | 0,171204524 | |
| 200691_s_at | HSPA9 | 6,233779363 | 6,062592536 | 0,171186827 | |
| 201979_s_at | PPP5C | 3,095191939 | 2,924079008 | 0,171112931 | |
| 214717_at | PKI55 | 0,760715727 | 0,589657387 | 0,17105834 | |
| 233303_at | - | 0,760715727 | 0,589657387 | 0,17105834 | |
| 230259_at | C10orf125 | 1,549482847 | 1,378453643 | 0,171029204 | |

| | | | | | |
|---|---|---|---|---|---|
| 205110_s_at | FGF13 | 1,659347917 | 1,488344553 | 0,171003365 | |
| 224966_s_at | DUS3L | 1,946200473 | 1,775255987 | 0,170944486 | |
| 33494_at | ETFDH | 1,003167985 | 0,832264713 | 0,170903272 | |
| 223562_at | PARVG | -0,112808516 | -0,283667828 | 0,170859311 | |
| 225314_at | OCIAD2 | -0,112808516 | -0,283667828 | 0,170859311 | |
| 227783_at | CCDC57 | -0,112808516 | -0,283667828 | 0,170859311 | |
| 212973_at | RPIA | 4,636814403 | 4,466010984 | 0,170803418 | |
| 200605_s_at | PRKAR1A | 5,231596898 | 5,060810624 | 0,170786274 | |
| 235315_at | TSC22D1 | 1,348276312 | 1,177496821 | 0,170779491 | |
| 232441_at | KRR1 | 1,473887017 | 1,303116084 | 0,170770932 | |
| 212714_at | LARP4 | 4,211016843 | 4,040263518 | 0,170753325 | |
| 235423_at | ORC2 | 1,976645579 | 1,805902329 | 0,17074325 | |
| 202402_s_at | CARS | 2,41290634 | 2,242180416 | 0,170725924 | |
| 226170_at | EYA3 | 3,796517367 | 3,625821706 | 0,170695661 | |
| 1560189_at | - | -0,741668563 | -0,912331589 | 0,170663026 | |
| 1560445_x_at | ARHGEF1 | -0,741668563 | -0,912331589 | 0,170663026 | |
| 1564319_at | FAM71E2 | -0,741668563 | -0,912331589 | 0,170663026 | |
| 204586_at | BSN | -0,741668563 | -0,912331589 | 0,170663026 | |
| 206540_at | GLB1L | -0,741668563 | -0,912331589 | 0,170663026 | |
| 206971_at | GPR161 | -0,741668563 | -0,912331589 | 0,170663026 | |
| 229836_s_at | NUDT4P1 | -0,741668563 | -0,912331589 | 0,170663026 | |
| 230910_s_at | LOC100288181 | -0,741668563 | -0,912331589 | 0,170663026 | |
| 232518_at | PRIC285 | -0,741668563 | -0,912331589 | 0,170663026 | |
| 241410_at | - | -0,741668563 | -0,912331589 | 0,170663026 | |
| 242587_at | SLC9A9 | -0,741668563 | -0,912331589 | 0,170663026 | |
| 243315_at | - | -0,741668563 | -0,912331589 | 0,170663026 | |
| 226749_at | MRPS9 | 3,455019843 | 3,284389368 | 0,170630475 | |
| 204560_at | FKBP5 | 0,82905153 | 0,658444279 | 0,170607251 | |
| 211505_s_at | STAU1 | 3,671816548 | 3,501209806 | 0,170606742 | |
| 223472_at | WHSC1 | 0,627515113 | 0,456918297 | 0,170596816 | |
| 235528_at | GUCA1B | 0,627515113 | 0,456918297 | 0,170596816 | |
| 219366_at | AVEN | 2,16388051 | 1,993317152 | 0,170563358 | |
| 218498_s_at | ERO1L | 1,732533761 | 1,562009954 | 0,170523807 | |
| 201043_s_at | ANP32A | 4,703696706 | 4,533260944 | 0,170435762 | |
| 212110_at | SLC39A14 | 4,593269972 | 4,422917167 | 0,170352806 | |
| 217857_s_at | RBM8A | 1,767779954 | 1,59747851 | 0,170301444 | |
| 214700_x_at | RIF1 | 2,938102794 | 2,767843855 | 0,170258939 | |
| 225897_at | MARCKS | 1,063722698 | 0,893473268 | 0,17024943 | |
| 226063_at | VAV2 | 1,063722698 | 0,893473268 | 0,17024943 | |
| 226890_at | WDR35 | 1,063722698 | 0,893473268 | 0,17024943 | |
| 203133_at | SEC61B | 6,315968779 | 6,145805576 | 0,170163203 | |
| 224988_at | C6orf89 | 1,982112682 | 1,811954245 | 0,170158438 | |
| 230721_at | C16orf52 | 1,631786706 | 1,461646549 | 0,170140157 | |
| 1557207_s_at | LOC283177 | 0,702292136 | 0,532164371 | 0,170127766 | |
| 201669_s_at | MARCKS | 3,276317033 | 3,106248809 | 0,170068224 | |
| 224756_s_at | ABHD16A /// LY6 | 3,205636958 | 3,035599287 | 0,17003767 | |
| 201397_at | PHGDH | 0,480753764 | 0,310717132 | 0,170036632 | |
| 202088_at | SLC39A6 | 4,445269027 | 4,275233864 | 0,170035163 | |
| 203094_at | MAD2L1BP | 4,333694283 | 4,163710704 | 0,169983579 | |
| 206488_s_at | CD36 | 5,506356754 | 5,336402323 | 0,169954431 | |
| 226336_at | LOC100288602 / | 3,634114217 | 3,464222256 | 0,169891961 | |
| 202631_s_at | APPBP2 | 1,560488174 | 1,390636111 | 0,169852063 | |
| 200696_s_at | GSN | 2,705069849 | 2,53523821 | 0,169831639 | |
| 221879_at | CALML4 | 0,95671774 | 0,786897721 | 0,169820019 | |
| 227337_at | ANKRD37 | 1,897847661 | 1,728030156 | 0,169817505 | |
| 209894_at | LEPR | 4,133297997 | 3,963505394 | 0,169792603 | |

| | | | | | |
|---|---|---|---|---|---|
| 213735_s_at | COX5B | 5,659645186 | 5,489853905 | 0,169791281 | |
| 206834_at | HBD | 6,636346263 | 6,46657202 | 0,169774243 | |
| 202871_at | TRAF4 | 1,706348269 | 1,536652708 | 0,169695561 | |
| 209339_at | SIAH2 | 2,26825514 | 2,098562556 | 0,169692584 | |
| 205031_at | EFNB3 | 2,292660284 | 2,123108223 | 0,169552061 | |
| 230477_at | LOC100130522 | 0,123193849 | -0,046302147 | 0,169495996 | |
| 236122_at | - | 0,123193849 | -0,046302147 | 0,169495996 | |
| 207121_s_at | MAPK6 | 5,48461416 | 5,315121782 | 0,169492378 | |
| 222440_s_at | THRAP3 | 3,136227209 | 2,966746113 | 0,169481096 | |
| 201891_s_at | B2M | 7,339534953 | 7,170099044 | 0,169435909 | |
| 202748_at | GBP2 | 1,360921491 | 1,191491281 | 0,16943021 | |
| 206707_x_at | FAM65B | 1,638726528 | 1,469325098 | 0,16940143 | |
| 208114_s_at | ISG20L2 | 2,466546243 | 2,297144887 | 0,169401357 | |
| 202026_at | SDHD | 4,993521137 | 4,82420097 | 0,169320167 | |
| 225965_at | DDHD1 | 0,841135803 | 0,67181667 | 0,169319133 | |
| 1566548_at | - | -0,366776884 | -0,536087151 | 0,169310267 | |
| 205334_at | S100A1 | -0,366776884 | -0,536087151 | 0,169310267 | |
| 221402_at | OR1F1 | -0,366776884 | -0,536087151 | 0,169310267 | |
| 233931_at | - | -0,366776884 | -0,536087151 | 0,169310267 | |
| 233991_at | - | -0,366776884 | -0,536087151 | 0,169310267 | |
| 240002_at | LOC389634 | -0,366776884 | -0,536087151 | 0,169310267 | |
| 227567_at | LOC100499466 | 1,224581536 | 1,055274826 | 0,16930671 | |
| 202087_s_at | CTSL1 | 4,346467354 | 4,177252958 | 0,169214396 | |
| 223716_s_at | ZRANB2 | 4,998250432 | 4,829058189 | 0,169192242 | |
| 221652_s_at | ASUN | 4,026230348 | 3,857048799 | 0,169181549 | |
| 226767_s_at | FAHD1 | 1,406361373 | 1,23718272 | 0,169178653 | |
| 229307_at | ANKRD28 | 1,81142822 | 1,642321262 | 0,169106958 | |
| 213387_at | ATAD2B | 2,81177561 | 2,64270545 | 0,16907016 | |
| 203313_s_at | TGIF1 | 3,50998543 | 3,340940512 | 0,169044918 | |
| 239960_x_at | LYRM7 | 1,274448124 | 1,105412836 | 0,169035288 | |
| 201999_s_at | DYNLT1 | 5,841890383 | 5,672859131 | 0,169031251 | |
| 1555730_a_at | CFL1 | 5,434648023 | 5,26568197 | 0,168966053 | |
| 203646_at | FDX1 | 2,713311265 | 2,544354523 | 0,168956742 | |
| 214387_x_at | SFTPC | -0,221656139 | -0,39061235 | 0,168956212 | |
| 220827_at | - | -0,221656139 | -0,39061235 | 0,168956212 | |
| 231052_at | GLOD5 | -0,221656139 | -0,39061235 | 0,168956212 | |
| 233315_at | - | -0,221656139 | -0,39061235 | 0,168956212 | |
| 237681_at | - | -0,221656139 | -0,39061235 | 0,168956212 | |
| 243404_at | - | -0,221656139 | -0,39061235 | 0,168956212 | |
| 218398_at | MRPS30 | 4,387215694 | 4,21827299 | 0,168942704 | |
| 220073_s_at | PLEKHG6 | 0,228146222 | 0,059218869 | 0,168927353 | |
| 220418_at | UBASH3A | 0,228146222 | 0,059218869 | 0,168927353 | |
| 226920_at | CSNK1A1 | 0,228146222 | 0,059218869 | 0,168927353 | |
| 227528_s_at | MLL2 | 0,228146222 | 0,059218869 | 0,168927353 | |
| 231697_s_at | - | 0,228146222 | 0,059218869 | 0,168927353 | |
| 222980_at | RAB10 | 5,55279188 | 5,38389617 | 0,16889571 | |
| 212180_at | CRKL | 4,222610527 | 4,053733117 | 0,16887741 | |
| 226098_at | IFT80 | 2,077135162 | 1,908311039 | 0,168824122 | |
| 229644_at | PREP | 1,748661581 | 1,579853228 | 0,168808353 | |
| 217716_s_at | SEC61A1 | 4,253455927 | 4,084681583 | 0,168774344 | |
| 217202_s_at | GLUL | 3,571118904 | 3,402394519 | 0,168724385 | |
| 203205_at | KDM4A | 1,493160564 | 1,324440845 | 0,168719719 | |
| 225953_at | RPRD1A | 3,493810203 | 3,325159096 | 0,168651107 | |
| 203242_s_at | PDLIM5 | 2,433006244 | 2,264418116 | 0,168588128 | |
| 1554008_at | OSMR | -0,986356909 | -1,154897679 | 0,16854077 | |
| 1563014_at | RPS15 | -0,986356909 | -1,154897679 | 0,16854077 | |

| | | | | | |
|---|---|---|---|---|---|
| 205605_at | HOXD9 | -0,986356909 | -1,154897679 | 0,16854077 | |
| 208488_s_at | CR1 | -0,986356909 | -1,154897679 | 0,16854077 | |
| 210152_at | LILRB4 | -0,986356909 | -1,154897679 | 0,16854077 | |
| 212839_s_at | TROVE2 | -0,986356909 | -1,154897679 | 0,16854077 | |
| 220852_at | PRO1768 | -0,986356909 | -1,154897679 | 0,16854077 | |
| 228022_at | CCDC18 | -0,986356909 | -1,154897679 | 0,16854077 | |
| 230664_at | H2BFXP | -0,986356909 | -1,154897679 | 0,16854077 | |
| 230717_at | LCN12 | -0,986356909 | -1,154897679 | 0,16854077 | |
| 232666_at | OAS3 | -0,986356909 | -1,154897679 | 0,16854077 | |
| 237371_at | - | -0,986356909 | -1,154897679 | 0,16854077 | |
| 238349_at | UBN2 | -0,986356909 | -1,154897679 | 0,16854077 | |
| 238374_at | H1FNT | -0,986356909 | -1,154897679 | 0,16854077 | |
| 243536_x_at | ARHGAP27 | -0,986356909 | -1,154897679 | 0,16854077 | |
| 244413_at | CLECL1 | -0,986356909 | -1,154897679 | 0,16854077 | |
| 203343_at | UGDH | 3,207971844 | 3,039473081 | 0,168498763 | |
| 202520_s_at | MLH1 | 4,302897163 | 4,13442882 | 0,168468343 | |
| 202918_s_at | HSPE1-MOB4 /// | 4,647605722 | 4,479143747 | 0,168461976 | |
| 1557342_a_at | - | -0,089689931 | -0,258114234 | 0,168424303 | |
| 204789_at | FMNL1 | -0,089689931 | -0,258114234 | 0,168424303 | |
| 209573_s_at | C18orf1 | -0,089689931 | -0,258114234 | 0,168424303 | |
| 231774_at | KCNIP3 | -0,089689931 | -0,258114234 | 0,168424303 | |
| 236898_at | - | -0,089689931 | -0,258114234 | 0,168424303 | |
| 227012_at | SLC25A40 | 2,525769574 | 2,357351836 | 0,168417738 | |
| 230947_at | 41153 | 0,648296226 | 0,479907041 | 0,168389185 | |
| 221702_s_at | TM2D3 | 4,239257588 | 4,070868415 | 0,168389172 | |
| 216650_at | - | 1,233777334 | 1,065442793 | 0,168334541 | |
| 209941_at | RIPK1 | 1,414471577 | 1,246149888 | 0,168321689 | |
| 200912_s_at | EIF4A2 /// MIR12 | 7,019980367 | 6,851696186 | 0,168284181 | |
| 217437_s_at | TACC1 | 2,327437554 | 2,159161028 | 0,168276526 | |
| 1553415_at | SLC17A8 | -2,930741289 | -3,098858316 | 0,168117027 | |
| 1555355_a_at | ETS1 | -2,930741289 | -3,098858316 | 0,168117027 | |
| 1558846_at | PNLIPRP3 | -2,930741289 | -3,098858316 | 0,168117027 | |
| 1559045_at | LOC100128288 | -2,930741289 | -3,098858316 | 0,168117027 | |
| 1560288_at | - | -2,930741289 | -3,098858316 | 0,168117027 | |
| 1562400_at | LOC283112 | -2,930741289 | -3,098858316 | 0,168117027 | |
| 1562644_at | MTHFD2L | -2,930741289 | -3,098858316 | 0,168117027 | |
| 1567247_at | OR5H1 | -2,930741289 | -3,098858316 | 0,168117027 | |
| 1569756_at | - | -2,930741289 | -3,098858316 | 0,168117027 | |
| 215228_at | NHLH2 | -2,930741289 | -3,098858316 | 0,168117027 | |
| 216722_at | VENTXP1 | -2,930741289 | -3,098858316 | 0,168117027 | |
| 224231_at | PRO0471 | -2,930741289 | -3,098858316 | 0,168117027 | |
| 231856_at | KIAA1244 | -2,930741289 | -3,098858316 | 0,168117027 | |
| 241861_at | SYCP3 | -2,930741289 | -3,098858316 | 0,168117027 | |
| 243958_at | - | -2,930741289 | -3,098858316 | 0,168117027 | |
| 244612_at | - | -2,930741289 | -3,098858316 | 0,168117027 | |
| 213320_at | PRMT3 | 2,943717636 | 2,775606361 | 0,168111276 | |
| 200838_at | CTSB | 3,659926886 | 3,491815843 | 0,168111043 | |
| 218427_at | SDCCAG3 | 2,577249059 | 2,409170778 | 0,168078281 | |
| 214681_at | GK | 0,97328325 | 0,805216064 | 0,168067186 | |
| 205305_at | FGL1 | 2,416948786 | 2,248887791 | 0,168060995 | |
| 1563405_at | ATP4B | 0,417596971 | 0,249604176 | 0,167992795 | |
| 226547_at | KAT6A | 3,030704581 | 2,862725455 | 0,167979126 | |
| 203384_s_at | GOLGA1 | 0,03125738 | -0,136643983 | 0,167901363 | |
| 220573_at | KLK14 | 0,03125738 | -0,136643983 | 0,167901363 | |
| 233230_s_at | SLAIN2 | 0,03125738 | -0,136643983 | 0,167901363 | |
| 238207_at | C3orf78 | 0,03125738 | -0,136643983 | 0,167901363 | |

| | | | | | |
|---|---|---|---|---|---|
| 206205_at | MPHOSPH9 | 1,917954284 | 1,750261389 | 0,167692895 | |
| 204776_at | THBS4 | 0,503742507 | 0,336136178 | 0,167606329 | |
| 206173_x_at | GABPB1 | 2,333865983 | 2,166264721 | 0,167601261 | |
| 52837_at | KIAA1644 | -2,113928714 | -2,281445174 | 0,167516459 | |
| 1555107_a_at | UVSSA | -0,512251684 | -0,679763839 | 0,167512155 | |
| 1559474_at | SPEG | -0,512251684 | -0,679763839 | 0,167512155 | |
| 207684_at | TBX6 | -0,512251684 | -0,679763839 | 0,167512155 | |
| 208458_at | SCNN1D | -0,512251684 | -0,679763839 | 0,167512155 | |
| 210197_at | ITPK1 | -0,512251684 | -0,679763839 | 0,167512155 | |
| 214968_at | DDX51 | -0,512251684 | -0,679763839 | 0,167512155 | |
| 223641_at | - | -0,512251684 | -0,679763839 | 0,167512155 | |
| 237267_at | LOC100506675 | -0,512251684 | -0,679763839 | 0,167512155 | |
| 239216_at | TEKT1 | -0,512251684 | -0,679763839 | 0,167512155 | |
| 239870_at | SPATS1 /// TMEI | -0,512251684 | -0,679763839 | 0,167512155 | |
| 1568796_at | CCDC157 | 0,859074518 | 0,691645685 | 0,167428833 | |
| 225521_at | ANAPC7 | 0,859074518 | 0,691645685 | 0,167428833 | |
| 236817_at | ADAT2 | 1,143956113 | 0,976641161 | 0,167314952 | |
| 212635_at | TNPO1 | 3,899061119 | 3,731768909 | 0,167292211 | |
| 1554065_at | FAM125B | 0,246434561 | 0,079428135 | 0,167006426 | |
| 1568728_s_at | RNF207 | 0,246434561 | 0,079428135 | 0,167006426 | |
| 214140_at | SLC25A16 | 0,246434561 | 0,079428135 | 0,167006426 | |
| 219666_at | MS4A6A | 0,246434561 | 0,079428135 | 0,167006426 | |
| 231583_at | KRT74 | 0,246434561 | 0,079428135 | 0,167006426 | |
| 238470_at | SYS1 | 0,246434561 | 0,079428135 | 0,167006426 | |
| 227476_at | LPGAT1 | 0,661985843 | 0,495031851 | 0,166953992 | |
| 236695_at | STK4-AS1 | 0,661985843 | 0,495031851 | 0,166953992 | |
| 239311_at | - | 0,661985843 | 0,495031851 | 0,166953992 | |
| 213185_at | KIAA0556 | 0,984222186 | 0,817300337 | 0,166921849 | |
| 228517_at | MEAF6 | 0,984222186 | 0,817300337 | 0,166921849 | |
| 236442_at | DPF3 | 0,984222186 | 0,817300337 | 0,166921849 | |
| 242439_s_at | ASXL1 | 0,984222186 | 0,817300337 | 0,166921849 | |
| 217970_s_at | CNOT6 | 3,569303935 | 3,402394519 | 0,166909416 | |
| 200662_s_at | TOMM20 | 5,715894277 | 5,549023389 | 0,166870888 | |
| 1556176_at | TAF8 | 0,735041319 | 0,568365095 | 0,166676224 | |
| 1570362_at | - | 0,735041319 | 0,568365095 | 0,166676224 | |
| 202763_at | CASP3 | 4,244953702 | 4,078419261 | 0,166534441 | |
| 209707_at | PIGK | 3,202127528 | 3,035599287 | 0,166528241 | |
| 201171_at | LOC100652765 | 1,666156729 | 1,499637023 | 0,166519706 | |
| 224447_s_at | MIEN1 | 3,072307152 | 2,905803877 | 0,166503275 | |
| 226660_at | RPS6KB1 | 3,936854923 | 3,770352811 | 0,166502112 | |
| 226601_at | SLC30A7 | 2,151803104 | 1,98530622 | 0,166496884 | |
| 209645_s_at | ALDH1B1 | 1,390002893 | 1,223626587 | 0,166376306 | |
| 226742_at | SAR1B | 3,593615441 | 3,427247355 | 0,166368086 | |
| 226443_at | FAM122A | 2,277177578 | 2,110887591 | 0,166289988 | |
| 201636_at | FXR1 | 3,312571045 | 3,146317015 | 0,16625403 | |
| 1558066_s_at | TBC1D16 | 0,87091093 | 0,704715219 | 0,166195711 | |
| 228190_at | ATG4C | 2,102456302 | 1,936283387 | 0,166172915 | |
| 1552993_at | DYDC1 | -2,972500237 | -3,138626879 | 0,166126642 | |
| 1555341_at | UNC5C | -2,972500237 | -3,138626879 | 0,166126642 | |
| 1560251_at | LOC645485 | -2,972500237 | -3,138626879 | 0,166126642 | |
| 1561210_at | - | -2,972500237 | -3,138626879 | 0,166126642 | |
| 1563001_at | - | -2,972500237 | -3,138626879 | 0,166126642 | |
| 1563133_at | - | -2,972500237 | -3,138626879 | 0,166126642 | |
| 1564777_at | HYALP1 | -2,972500237 | -3,138626879 | 0,166126642 | |
| 1566673_at | - | -2,972500237 | -3,138626879 | 0,166126642 | |
| 206439_at | EPYC | -2,972500237 | -3,138626879 | 0,166126642 | |

| | | | | | |
|---|---|---|---|---|---|
| 206456_at | GABRA5 | -2,972500237 | -3,138626879 | 0,166126642 | |
| 213006_at | CEBPD | -2,972500237 | -3,138626879 | 0,166126642 | |
| 232638_at | COL20A1 | -2,972500237 | -3,138626879 | 0,166126642 | |
| 232872_at | - | -2,972500237 | -3,138626879 | 0,166126642 | |
| 237160_at | CCDC83 | -2,972500237 | -3,138626879 | 0,166126642 | |
| 237837_at | - | -2,972500237 | -3,138626879 | 0,166126642 | |
| 239185_at | ABCA9 | -2,972500237 | -3,138626879 | 0,166126642 | |
| 242057_at | - | -2,972500237 | -3,138626879 | 0,166126642 | |
| 242170_at | - | -2,972500237 | -3,138626879 | 0,166126642 | |
| 243901_at | ALDOB | -2,972500237 | -3,138626879 | 0,166126642 | |
| 217185_s_at | ZNF259 /// ZNF2! | 2,87191078 | 2,705818724 | 0,166092057 | |
| 223251_s_at | ANKRD10 | 2,791679321 | 2,625627364 | 0,166051958 | |
| 33148_at | ZFR | 1,194283819 | 1,028237864 | 0,166045955 | |
| 242292_at | FAM226B | 0,518867317 | 0,352836757 | 0,16603056 | |
| 1557219_at | LOC100506827 | -0,946847173 | -1,112851844 | 0,166004671 | |
| 208014_x_at | - | -0,946847173 | -1,112851844 | 0,166004671 | |
| 216663_s_at | ZMYND10 | -0,946847173 | -1,112851844 | 0,166004671 | |
| 221333_at | FOXP3 | -0,946847173 | -1,112851844 | 0,166004671 | |
| 229731_at | FOXS1 | -0,946847173 | -1,112851844 | 0,166004671 | |
| 230342_at | FOXK1 | -0,946847173 | -1,112851844 | 0,166004671 | |
| 235140_at | SHROOM1 | -0,946847173 | -1,112851844 | 0,166004671 | |
| 244122_at | MGST3 | -0,946847173 | -1,112851844 | 0,166004671 | |
| 203630_s_at | COG5 | 2,808702036 | 2,64270545 | 0,165996586 | |
| 225993_at | EARS2 | 0,934330293 | 0,768343791 | 0,165986502 | |
| 203403_s_at | RNF6 | 4,724639777 | 4,558677353 | 0,165962424 | |
| 215510_at | ETV2 | 1,841816345 | 1,675891488 | 0,165924857 | |
| 200694_s_at | DDX24 | 5,341750837 | 5,175853482 | 0,165897355 | |
| 226157_at | TFDP2 | 2,281618189 | 2,115788273 | 0,165829917 | |
| 210947_s_at | MSH3 | 1,874520207 | 1,708698295 | 0,165821912 | |
| 205522_at | HOXD4 | 1,672933557 | 1,507116528 | 0,165817029 | |
| 205051_s_at | KIT | 6,032283861 | 5,866470968 | 0,165812893 | |
| 201964_at | SETX | 2,691784869 | 2,526063925 | 0,165720944 | |
| 224800_at | WDFY1 | 3,418185797 | 3,252481567 | 0,16570423 | |
| 226642_s_at | NUDCD2 | 4,178645282 | 4,012941072 | 0,165704211 | |
| 201652_at | COPS5 | 5,703122923 | 5,537427697 | 0,165695227 | |
| 223474_at | IRF2BPL | 6,054672518 | 5,889009948 | 0,16566257 | |
| 201681_s_at | DLG5 | 1,523472489 | 1,3579181 | 0,165554389 | |
| 225049_at | BLOC1S2 | 5,311127054 | 5,145621945 | 0,165505108 | |
| 228963_at | - | 1,780386218 | 1,614891062 | 0,165495157 | |
| 40284_at | FOXA2 | -2,570400074 | -2,735862426 | 0,165462352 | |
| 223599_at | TRIM6 | 1,940595304 | 1,775255987 | 0,165339317 | |
| 207740_s_at | NUP62 | 2,956271699 | 2,791007185 | 0,165264514 | |
| 224566_at | LOC100653017 / | 2,799441827 | 2,634191677 | 0,16525015 | |
| 235125_x_at | FAM73A | 1,000476627 | 0,835239052 | 0,165237575 | |
| 221434_s_at | SLIRP | 6,820231911 | 6,655003304 | 0,165228606 | |
| 1555483_x_at | FBLIM1 | -0,325749314 | -0,490927335 | 0,165178021 | |
| 1559240_at | LOC100507053 | -0,325749314 | -0,490927335 | 0,165178021 | |
| 204882_at | ARHGAP25 | -0,325749314 | -0,490927335 | 0,165178021 | |
| 211094_s_at | NF1 | -0,325749314 | -0,490927335 | 0,165178021 | |
| 221376_at | FGF17 | -0,325749314 | -0,490927335 | 0,165178021 | |
| 222125_s_at | P4HTM | -0,325749314 | -0,490927335 | 0,165178021 | |
| 222784_at | SMOC1 | -0,325749314 | -0,490927335 | 0,165178021 | |
| 231542_at | SPAG5-AS1 | -0,325749314 | -0,490927335 | 0,165178021 | |
| 232870_at | HDAC10 | -0,325749314 | -0,490927335 | 0,165178021 | |
| 236103_at | - | -0,325749314 | -0,490927335 | 0,165178021 | |
| 239677_at | - | -0,325749314 | -0,490927335 | 0,165178021 | |

| | | | | | |
|---|---|---|---|---|---|
| 240278_at | RASSF1 | -0,325749314 | -0,490927335 | 0,165178021 | |
| 202380_s_at | NKTR | 2,001086023 | 1,835911187 | 0,165174836 | |
| 218205_s_at | MKNK2 | 4,880967039 | 4,71581024 | 0,165156799 | |
| 201304_at | NDUFA5 | 5,624243949 | 5,459092535 | 0,165151414 | |
| 223107_s_at | ZCCHC17 | 3,547344317 | 3,382199319 | 0,165144998 | |
| 212405_s_at | METTL13 | 3,784051878 | 3,618934391 | 0,165117487 | |
| 205042_at | GNE | 2,912562019 | 2,747463672 | 0,165098348 | |
| 212685_s_at | TBL2 | 3,508091848 | 3,343031718 | 0,165060131 | |
| 225447_at | GPD2 | 4,038745395 | 3,873779903 | 0,164965492 | |
| 205769_at | SLC27A2 | 1,883312263 | 1,718396606 | 0,164915657 | |
| 223637_s_at | FAM160A2 | 1,883312263 | 1,718396606 | 0,164915657 | |
| 209274_s_at | ISCA1 | 4,160677048 | 3,995764148 | 0,1649129 | |
| 1562012_at | LOC100506730 | -0,055687482 | -0,220592223 | 0,164904741 | |
| 1569898_a_at | LOC100132707 | -0,055687482 | -0,220592223 | 0,164904741 | |
| 223371_s_at | DNAJC4 | -0,055687482 | -0,220592223 | 0,164904741 | |
| 236073_at | EPHA10 | -0,055687482 | -0,220592223 | 0,164904741 | |
| 242272_at | ZNF785 | -0,055687482 | -0,220592223 | 0,164904741 | |
| 225351_at | FAM45A | 2,218170265 | 2,053299695 | 0,16487057 | |
| 203102_s_at | MGAT2 | 5,452973635 | 5,288105861 | 0,164867774 | |
| 203875_at | SMARCA1 | 2,243430036 | 2,078620836 | 0,164809201 | |
| 225158_at | GFM1 | 3,540875956 | 3,376085218 | 0,164790738 | |
| 231396_s_at | FAM126A | 1,683039483 | 1,518263577 | 0,164775905 | |
| 209255_at | KLHDC10 | 1,946200473 | 1,781437557 | 0,164762916 | |
| 221436_s_at | CDCA3 | 3,155710652 | 2,990953566 | 0,164757086 | |
| 201123_s_at | EIF5A | 7,300614252 | 7,135905898 | 0,164708354 | |
| 201031_s_at | HNRNPH1 | 6,479154262 | 6,314485986 | 0,164668276 | |
| 202883_s_at | PPP2R1B | 3,780134221 | 3,615478364 | 0,164655857 | |
| 215826_x_at | ZNF835 | 1,269985355 | 1,105412836 | 0,164572519 | |
| 1555185_x_at | TERF2 | 0,822971223 | 0,658444279 | 0,164526944 | |
| 227605_at | AIMP1 | 0,822971223 | 0,658444279 | 0,164526944 | |
| 204812_at | ZW10 | 3,270743649 | 3,106248809 | 0,16449484 | |
| 221056_x_at | EPS15L1 | 1,823660386 | 1,659204017 | 0,164456369 | |
| 210495_x_at | FN1 | 2,197169074 | 2,032717854 | 0,164451221 | |
| 223540_at | PVRL4 | 0,171847695 | 0,007421914 | 0,164425782 | |
| 234664_at | LOC100132062 / | 0,171847695 | 0,007421914 | 0,164425782 | |
| 225704_at | FBRSL1 | 1,534677255 | 1,370274469 | 0,164402786 | |
| 227205_at | TAF1 | 3,037284587 | 2,87289275 | 0,164391837 | |
| 1554192_s_at | TRMT44 | 0,888485433 | 0,724100169 | 0,164385264 | |
| 210188_at | GABPA | 0,888485433 | 0,724100169 | 0,164385264 | |
| 208094_s_at | CCDC130 | 3,052955227 | 2,888726553 | 0,164228673 | |
| 206567_s_at | PHF20 | 2,094906514 | 1,930732138 | 0,164174376 | |
| 1556211_a_at | - | -3,013665402 | -3,177802286 | 0,164136884 | |
| 1561613_at | - | -3,013665402 | -3,177802286 | 0,164136884 | |
| 1564392_at | LINC00320 | -3,013665402 | -3,177802286 | 0,164136884 | |
| 1564469_at | LMOD3 | -3,013665402 | -3,177802286 | 0,164136884 | |
| 1565228_s_at | ALB | -3,013665402 | -3,177802286 | 0,164136884 | |
| 1566716_at | - | -3,013665402 | -3,177802286 | 0,164136884 | |
| 1569591_at | F11 | -3,013665402 | -3,177802286 | 0,164136884 | |
| 205989_s_at | MOG | -3,013665402 | -3,177802286 | 0,164136884 | |
| 217524_x_at | - | -3,013665402 | -3,177802286 | 0,164136884 | |
| 220345_at | LRRTM4 | -3,013665402 | -3,177802286 | 0,164136884 | |
| 231726_at | PCDHB14 | -3,013665402 | -3,177802286 | 0,164136884 | |
| 234433_at | - | -3,013665402 | -3,177802286 | 0,164136884 | |
| 234904_x_at | ELAVL4 | -3,013665402 | -3,177802286 | 0,164136884 | |
| 237233_at | - | -3,013665402 | -3,177802286 | 0,164136884 | |
| 237282_s_at | AKAP14 | -3,013665402 | -3,177802286 | 0,164136884 | |

| | | | | | |
|---|---|---|---|---|---|
| 240921_at | - | -3,013665402 | -3,177802286 | 0,164136884 | |
| 204701_s_at | STOML1 | 1,011212064 | 0,847075464 | 0,1641366 | |
| 223145_s_at | AKIRIN2 | 3,92199823 | 3,757867094 | 0,164131136 | |
| 219704_at | YBX2 | 2,122398022 | 1,958277216 | 0,164120806 | |
| 213338_at | TMEM158 | 1,689737652 | 1,525647381 | 0,164090271 | |
| 205024_s_at | RAD51 | 3,345817457 | 3,181801492 | 0,164015966 | |
| 222204_s_at | RRN3 | 4,6079313 | 4,44401622 | 0,16391508 | |
| 208725_at | EIF2S2 | 1,795990708 | 1,632095957 | 0,163894751 | |
| 212189_s_at | COG4 | 1,795990708 | 1,632095957 | 0,163894751 | |
| 217097_s_at | PHTF2 | 0,457392764 | 0,293518836 | 0,163873929 | |
| 223449_at | SEMA6A | 0,457392764 | 0,293518836 | 0,163873929 | |
| 239034_at | CXorf24 | 0,457392764 | 0,293518836 | 0,163873929 | |
| 202682_s_at | USP4 | 4,240398611 | 4,076535251 | 0,16386336 | |
| 204425_at | ARHGAP4 | 1,177702228 | 1,013870809 | 0,163831419 | |
| 34031_i_at | KRIT1 | 2,675004741 | 2,511262629 | 0,163742112 | |
| 211358_s_at | CIZ1 | 0,541261317 | 0,377530647 | 0,16373067 | |
| 236698_at | - | 0,541261317 | 0,377530647 | 0,16373067 | |
| 237411_at | ADAMTS6 | 0,541261317 | 0,377530647 | 0,16373067 | |
| 212398_at | RDX | 2,693452197 | 2,529740643 | 0,163711555 | |
| 200047_s_at | YY1 | 6,626015835 | 6,462369855 | 0,16364598 | |
| 219128_at | C2orf42 | 1,542099044 | 1,378453643 | 0,1636454 | |
| 227569_at | LNX2 | 2,926807124 | 2,763166224 | 0,1636409 | |
| 204444_at | KIF11 | 3,857278636 | 3,693764321 | 0,163514315 | |
| 227078_at | TMEM110 | 0,695652136 | 0,532164371 | 0,163487766 | |
| 231777_at | CSNK2B /// LY6G | 0,695652136 | 0,532164371 | 0,163487766 | |
| 235526_at | SOX6 | 0,695652136 | 0,532164371 | 0,163487766 | |
| 205395_s_at | MRE11A | 2,621710292 | 2,458228496 | 0,163481796 | |
| 227656_at | C6orf70 | 1,865674241 | 1,702196344 | 0,163477897 | |
| 240928_at | FCF1 | 1,865674241 | 1,702196344 | 0,163477897 | |
| 221877_at | IRGQ | 2,783874823 | 2,620464265 | 0,163410558 | |
| 1554456_a_at | LINS | 0,767063567 | 0,603679647 | 0,16338392 | |
| 1555469_a_at | CLASP2 | 0,767063567 | 0,603679647 | 0,16338392 | |
| 225876_at | NIPAL3 | 0,767063567 | 0,603679647 | 0,16338392 | |
| 1552579_a_at | ADAM21 | -0,655928373 | -0,819238336 | 0,163309963 | |
| 1554488_at | CEP70 | -0,655928373 | -0,819238336 | 0,163309963 | |
| 1557283_a_at | ZNF519 | -0,655928373 | -0,819238336 | 0,163309963 | |
| 217120_s_at | MED14 | -0,655928373 | -0,819238336 | 0,163309963 | |
| 220214_at | ZNF215 | -0,655928373 | -0,819238336 | 0,163309963 | |
| 232758_s_at | - | -0,655928373 | -0,819238336 | 0,163309963 | |
| 237464_at | LOC100506060 / | -0,655928373 | -0,819238336 | 0,163309963 | |
| 241925_x_at | - | -0,655928373 | -0,819238336 | 0,163309963 | |
| 242382_at | LOC100506207 | -0,655928373 | -0,819238336 | 0,163309963 | |
| 209350_s_at | GPS2 | 2,352981171 | 2,189694216 | 0,163286955 | |
| 202369_s_at | TRAM2 | 4,447740857 | 4,284512471 | 0,163228385 | |
| 1554513_s_at | CEP89 | 0,900083947 | 0,73688026 | 0,163203687 | |
| 224460_s_at | L2HGDH | 0,900083947 | 0,73688026 | 0,163203687 | |
| 203867_s_at | NLE1 | 1,62481334 | 1,461646549 | 0,163166791 | |
| 200600_at | MSN | 6,775968267 | 6,612835819 | 0,163132448 | |
| 227171_at | CCT4 | 1,735773781 | 1,572742379 | 0,163031402 | |
| 220329_s_at | RMND1 | 2,422991296 | 2,259997914 | 0,162993383 | |
| 207494_s_at | ZNF76 | 1,46610492 | 1,303116084 | 0,162988836 | |
| 201232_s_at | PSMD13 | 5,100441809 | 4,93752389 | 0,162917919 | |
| 201186_at | LRPAP1 | 3,03990821 | 2,877228323 | 0,162679887 | |
| 1559060_a_at | FNIP1 | -0,033456564 | -0,19610998 | 0,162653416 | |
| 1560841_at | LOC389247 | -0,033456564 | -0,19610998 | 0,162653416 | |
| 208176_at | DUX1 | -0,033456564 | -0,19610998 | 0,162653416 | |

| | | | | | |
|---|---|---|---|---|---|
| 239418_x_at | - | -0,033456564 | -0,19610998 | 0,162653416 | |
| 239532_at | - | -0,033456564 | -0,19610998 | 0,162653416 | |
| 205662_at | B9D1 | 1,163336153 | 1,000684521 | 0,162651632 | |
| 1568609_s_at | FAM91A2 /// FLJ: | 2,35929703 | 2,196649505 | 0,162647525 | |
| 230188_at | NIPAL4 | 0,083054335 | -0,079522948 | 0,162577283 | |
| 234367_x_at | TMPRSS6 | 0,083054335 | -0,079522948 | 0,162577283 | |
| 1561666_a_at | KIAA1908 | -0,299034815 | -0,461594427 | 0,162559612 | |
| 1566504_at | - | -0,299034815 | -0,461594427 | 0,162559612 | |
| 1569961_at | - | -0,299034815 | -0,461594427 | 0,162559612 | |
| 210852_s_at | AASS | -0,299034815 | -0,461594427 | 0,162559612 | |
| 234580_at | TMEM106A | -0,299034815 | -0,461594427 | 0,162559612 | |
| 230466_s_at | RASSF3 | 1,027166904 | 0,864649967 | 0,162516937 | |
| 226294_x_at | FAM91A1 | 3,990735001 | 3,828227545 | 0,162507456 | |
| 202236_s_at | SLC16A1 | 5,386243429 | 5,223738367 | 0,162505062 | |
| 204237_at | GULP1 | 1,593009234 | 1,43051703 | 0,162492204 | |
| 218915_at | NF2 | 3,111496958 | 2,949006836 | 0,162490122 | |
| 228531_at | SAMD9 | 3,290707863 | 3,128242026 | 0,162465837 | |
| 226474_at | NLRC5 | 1,631786706 | 1,469325098 | 0,162461608 | |
| 203223_at | RABEP1 | 1,139070151 | 0,976641161 | 0,16242899 | |
| 236092_at | - | 1,139070151 | 0,976641161 | 0,16242899 | |
| 232227_at | LOC100505976 | 0,291166226 | 0,128747141 | 0,162419085 | |
| 1557690_x_at | - | 0,384950573 | 0,222599095 | 0,162351479 | |
| 230219_at | NDE1 | 0,384950573 | 0,222599095 | 0,162351479 | |
| 225472_at | GPANK1 | 3,819603766 | 3,6572547 | 0,162349066 | |
| 201534_s_at | UBL3 | 2,535098095 | 2,372786857 | 0,162311238 | |
| 233992_x_at | ZNF445 | 1,339784194 | 1,177496821 | 0,162287374 | |
| 236551_at | ZNF311 | 1,339784194 | 1,177496821 | 0,162287374 | |
| 221606_s_at | HMGN5 | 3,268508253 | 3,106248809 | 0,162259444 | |
| 210621_s_at | RASA1 | 3,420201053 | 3,258033502 | 0,162167551 | |
| 223931_s_at | CHFR | 2,830080806 | 2,667949403 | 0,162131403 | |
| 205182_s_at | ZNF324 | 2,433006244 | 2,271023131 | 0,161983113 | |
| 225579_at | PQLC3 | 2,003776245 | 1,841838775 | 0,16193747 | |
| 210157_at | URI1 | 3,296204798 | 3,134292219 | 0,161912579 | |
| 1556038_at | GPR173 | -0,888496123 | -1,050406615 | 0,161910492 | |
| 1559667_at | - | -0,888496123 | -1,050406615 | 0,161910492 | |
| 1560342_at | - | -0,888496123 | -1,050406615 | 0,161910492 | |
| 1562639_at | KIF6 | -0,888496123 | -1,050406615 | 0,161910492 | |
| 207792_at | OPRD1 | -0,888496123 | -1,050406615 | 0,161910492 | |
| 210221_at | CHRNA3 | -0,888496123 | -1,050406615 | 0,161910492 | |
| 217433_at | TACC1 | -0,888496123 | -1,050406615 | 0,161910492 | |
| 222042_x_at | MEX3D | -0,888496123 | -1,050406615 | 0,161910492 | |
| 233930_at | DMRT3 | -0,888496123 | -1,050406615 | 0,161910492 | |
| 234251_at | - | -0,888496123 | -1,050406615 | 0,161910492 | |
| 236040_at | XAGE3 | -0,888496123 | -1,050406615 | 0,161910492 | |
| 236682_at | - | -0,888496123 | -1,050406615 | 0,161910492 | |
| 236847_at | C19orf18 | -0,888496123 | -1,050406615 | 0,161910492 | |
| 236932_s_at | GATAD2A | -0,888496123 | -1,050406615 | 0,161910492 | |
| 237598_at | SEC1P | -0,888496123 | -1,050406615 | 0,161910492 | |
| 242416_at | - | -0,888496123 | -1,050406615 | 0,161910492 | |
| 218529_at | CD320 | 3,579258128 | 3,417357537 | 0,161900591 | |
| 221824_s_at | 08.03.15 | 1,434549838 | 1,272721751 | 0,161828087 | |
| 226839_at | NR2C2AP | 2,146943689 | 1,98530622 | 0,161637469 | |
| 240032_at | - | 0,641402394 | 0,479907041 | 0,161495354 | |
| 1554827_a_at | ADCY7 | 0,715481152 | 0,553993624 | 0,161487527 | |
| 212743_at | RCHY1 | 0,715481152 | 0,553993624 | 0,161487527 | |
| 218849_s_at | PPP1R13L | 0,715481152 | 0,553993624 | 0,161487527 | |

| | | | | | |
|---|---|---|---|---|---|
| 220215_at | ZNF669 | 0,715481152 | 0,553993624 | 0,161487527 | |
| 204764_at | CHURC1-FNTB / | 1,037706275 | 0,876248481 | 0,161457794 | |
| 231022_at | OCLN | 1,201332287 | 1,039887232 | 0,161445055 | |
| 232139_s_at | KIAA1919 | 1,251994932 | 1,090553537 | 0,161441395 | |
| 236220_at | - | 1,603688695 | 1,442269454 | 0,161419241 | |
| 206096_at | ZNF35 | 1,64218396 | 1,480766833 | 0,161417128 | |
| 230653_at | - | 1,716223609 | 1,55481039 | 0,161413219 | |
| 200705_s_at | EEF1B2 /// SNOF | 6,893322346 | 6,73197677 | 0,161345575 | |
| 234981_x_at | CMBL | 4,495352883 | 4,334242016 | 0,161110868 | |
| 218447_at | CMC2 | 5,00682487 | 4,845746475 | 0,161078395 | |
| 1557646_at | LOC100507240 | -3,07502285 | -3,236070201 | 0,161047351 | |
| 1560099_at | LOC339260 | -3,07502285 | -3,236070201 | 0,161047351 | |
| 1560760_s_at | - | -3,07502285 | -3,236070201 | 0,161047351 | |
| 1568686_at | ATP8B5P | -3,07502285 | -3,236070201 | 0,161047351 | |
| 1570046_at | - | -3,07502285 | -3,236070201 | 0,161047351 | |
| 234597_at | - | -3,07502285 | -3,236070201 | 0,161047351 | |
| 234623_x_at | - | -3,07502285 | -3,236070201 | 0,161047351 | |
| 238584_at | IQCA1 | -3,07502285 | -3,236070201 | 0,161047351 | |
| 242098_at | KIAA1244 | -3,07502285 | -3,236070201 | 0,161047351 | |
| 201246_s_at | OTUB1 | 1,442503549 | 1,281471383 | 0,161032165 | |
| 203927_at | NFKBIE | 1,442503549 | 1,281471383 | 0,161032165 | |
| 201138_s_at | SSB | 4,812419766 | 4,651455664 | 0,160964102 | |
| 225546_at | EEF2K | 2,232003119 | 2,071071048 | 0,160932071 | |
| 203440_at | CDH2 | 3,628024221 | 3,467101363 | 0,160922857 | |
| 224723_x_at | LOC401397 | 4,487686025 | 4,326864571 | 0,160821454 | |
| 213944_x_at | GNA11 | 2,742596529 | 2,582026171 | 0,160570357 | |
| 203582_s_at | RAB4A /// SPHAF | 3,32230241 | 3,161800893 | 0,160501516 | |
| 221522_at | ANKRD27 | 5,249275201 | 5,088808057 | 0,160467144 | |
| 34478_at | RAB11B | -1,239798046 | -1,400201104 | 0,160403058 | |
| 212145_at | MRPS27 | 4,034147209 | 3,873779903 | 0,160367307 | |
| 226982_at | ELL2 | 2,671625168 | 2,511262629 | 0,160362538 | |
| 207768_at | EGR4 | 2,238870125 | 2,078620836 | 0,160249289 | |
| 222563_s_at | TNKS2 | 0,798390451 | 0,638150376 | 0,160240075 | |
| 203420_at | FAM8A1 | 3,61576656 | 3,455550298 | 0,160216261 | |
| 203756_at | ARHGEF17 | 1,360921491 | 1,200746069 | 0,160175422 | |
| 218780_at | HOOK2 | 1,360921491 | 1,200746069 | 0,160175422 | |
| 207891_s_at | HAUS7 /// TREX2 | 1,314003737 | 1,153866761 | 0,160136975 | |
| 201489_at | PPIF | 4,526087171 | 4,365953461 | 0,16013371 | |
| 208159_x_at | DDX11 | 2,338135744 | 2,17802703 | 0,160108713 | |
| 218521_s_at | UBE2W | 0,57782909 | 0,417770464 | 0,160058627 | |
| 228269_x_at | KCNIP3 | 0,57782909 | 0,417770464 | 0,160058627 | |
| 218433_at | PANK3 | 1,215326747 | 1,055274826 | 0,160051921 | |
| 219006_at | NDUFAF4 | 5,337625064 | 5,177612232 | 0,160012832 | |
| 242759_at | - | 0,496120222 | 0,336136178 | 0,159984044 | |
| 230241_at | TOR1AIP2 | 1,05337236 | 0,893473268 | 0,159899092 | |
| 214583_at | DDI2 /// RSC1A1 | 2,109966786 | 1,950068772 | 0,159898014 | |
| 205361_s_at | PFDN4 | 4,852893656 | 4,69303748 | 0,159856176 | |
| 226318_at | TBRG1 | 2,819431021 | 2,659583735 | 0,159847285 | |
| 212507_at | TMEM131 | 3,410096504 | 3,250254796 | 0,159841708 | |
| 201029_s_at | CD99 | 2,658027143 | 2,498185745 | 0,159841399 | |
| 1564494_s_at | P4HB | 4,341158978 | 4,181349409 | 0,159809569 | |
| 1565845_at | - | 0,317354302 | 0,157549243 | 0,159805059 | |
| 211805_s_at | SLC8A1 | 0,317354302 | 0,157549243 | 0,159805059 | |
| 213657_s_at | - | 0,317354302 | 0,157549243 | 0,159805059 | |
| 231983_at | IBA57 | 0,317354302 | 0,157549243 | 0,159805059 | |
| 218204_s_at | FYCO1 | 2,243430036 | 2,083632167 | 0,159797869 | |

| | | | | | |
|---|---|---|---|---|---|
| 200752_s_at | CAPN1 | 1,965648824 | 1,805902329 | 0,159746495 | |
| 38447_at | ADRBK1 | 2,560901553 | 2,401164389 | 0,159737164 | |
| 227117_at | XPOT | 3,477451563 | 3,317734938 | 0,159716626 | |
| 218073_s_at | TMEM48 | 2,139623739 | 1,979940779 | 0,15968296 | |
| 225432_s_at | CSRP2BP | 1,621313976 | 1,461646549 | 0,159667427 | |
| 219148_at | PBK | 4,264153976 | 4,104540277 | 0,159613699 | |
| 229235_at | NFATC2IP | 3,479385765 | 3,319860027 | 0,159525737 | |
| 230165_at | SGOL2 | 3,805796305 | 3,646288546 | 0,15950776 | |
| 200823_x_at | RPL29 | 7,436247072 | 7,276778786 | 0,159468287 | |
| 221844_x_at | SPCS3 | 5,08686793 | 4,927455161 | 0,159412769 | |
| 1554771_at | - | -0,00073712 | -0,160136748 | 0,159399628 | |
| 220242_x_at | ZNF701 | -0,00073712 | -0,160136748 | 0,159399628 | |
| 237414_at | F7 | -0,00073712 | -0,160136748 | 0,159399628 | |
| 1556822_s_at | ZNF837 | -0,85085127 | -1,010192375 | 0,159341105 | |
| 1557906_at | MUC12 | -0,85085127 | -1,010192375 | 0,159341105 | |
| 205918_at | SLC4A3 | -0,85085127 | -1,010192375 | 0,159341105 | |
| 237840_at | LOC388948 | -0,85085127 | -1,010192375 | 0,159341105 | |
| 239837_at | ADAM11 | -0,85085127 | -1,010192375 | 0,159341105 | |
| 241389_at | CHRNB2 | -0,85085127 | -1,010192375 | 0,159341105 | |
| 204847_at | ZBTB11 | 3,992767659 | 3,833443169 | 0,15932449 | |
| 203211_s_at | MTMR2 | 3,755604375 | 3,596320795 | 0,15928358 | |
| 1563431_x_at | CALM3 | 1,774096855 | 1,614891062 | 0,159205794 | |
| 205369_x_at | DBT | 1,774096855 | 1,614891062 | 0,159205794 | |
| 225390_s_at | KLF13 | 1,774096855 | 1,614891062 | 0,159205794 | |
| 201831_s_at | USO1 | 2,14450783 | 1,98530622 | 0,15920161 | |
| 214198_s_at | DGCR2 | 1,940595304 | 1,781437557 | 0,159157747 | |
| 228282_at | MFSD8 | 1,940595304 | 1,781437557 | 0,159157747 | |
| 219731_at | CC2D2B | 3,017453847 | 2,858346012 | 0,159107834 | |
| 203983_at | TSNAX | 3,964737003 | 3,805658807 | 0,159078196 | |
| 1557256_a_at | - | -3,114791413 | -3,273861381 | 0,159069969 | |
| 1562054_at | SMEK3P | -3,114791413 | -3,273861381 | 0,159069969 | |
| 1563135_at | - | -3,114791413 | -3,273861381 | 0,159069969 | |
| 217229_at | ASB4 | -3,114791413 | -3,273861381 | 0,159069969 | |
| 220502_s_at | SLC13A1 | -3,114791413 | -3,273861381 | 0,159069969 | |
| 220786_s_at | SLC38A4 | -3,114791413 | -3,273861381 | 0,159069969 | |
| 238441_at | PRKAA2 | -3,114791413 | -3,273861381 | 0,159069969 | |
| 240447_at | - | -3,114791413 | -3,273861381 | 0,159069969 | |
| 241016_at | - | -3,114791413 | -3,273861381 | 0,159069969 | |
| 242952_at | - | -3,114791413 | -3,273861381 | 0,159069969 | |
| 243401_at | - | -3,114791413 | -3,273861381 | 0,159069969 | |
| 229742_at | C15orf61 | 3,598072957 | 3,439026336 | 0,159046621 | |
| 227807_at | PARP9 | 1,666156729 | 1,507116528 | 0,159040201 | |
| 242264_at | - | 1,119359127 | 0,960386719 | 0,158972408 | |
| 200798_x_at | MCL1 | 5,487980577 | 5,329035945 | 0,158944632 | |
| 203881_s_at | DMD | 4,034147209 | 3,875225653 | 0,158921556 | |
| 208591_s_at | PDE3B | 1,063722698 | 0,904843258 | 0,158879439 | |
| 208810_at | DNAJB6 /// TMEM | 4,278110569 | 4,119256941 | 0,158853628 | |
| 206889_at | PDIA2 | -0,259832361 | -0,41858459 | 0,158752228 | |
| 207527_at | KCNJ9 | -0,259832361 | -0,41858459 | 0,158752228 | |
| 220135_s_at | SLC7A9 | -0,259832361 | -0,41858459 | 0,158752228 | |
| 224374_s_at | EMILIN2 | -0,259832361 | -0,41858459 | 0,158752228 | |
| 228876_at | BAIAP2L2 | -0,259832361 | -0,41858459 | 0,158752228 | |
| 232405_at | - | -0,259832361 | -0,41858459 | 0,158752228 | |
| 238841_at | PTPDC1 | -0,259832361 | -0,41858459 | 0,158752228 | |
| 242781_at | FAM199X | -0,259832361 | -0,41858459 | 0,158752228 | |
| 207722_s_at | BTBD2 | 0,592200562 | 0,433557298 | 0,158643263 | |

| | | | | | |
|---|---|---|---|---|---|
| 238824_at | - | 0,592200562 | 0,433557298 | 0,158643263 | |
| 242813_at | - | 0,592200562 | 0,433557298 | 0,158643263 | |
| 208631_s_at | HADHA | 4,15705639 | 3,9984201 | 0,15863629 | |
| 217922_at | MAN1A2 | 2,898174856 | 2,739547836 | 0,15862702 | |
| 204853_at | ORC2 | 2,814842651 | 2,656223838 | 0,158618813 | |
| 225425_s_at | MRPL41 | 3,434229569 | 3,275657468 | 0,158572101 | |
| 210455_at | R3HCC1L | 0,882651021 | 0,724100169 | 0,158550852 | |
| 225761_at | PAPD4 | 3,191547726 | 3,033010968 | 0,158536758 | |
| 204900_x_at | SAP30 | 4,521870506 | 4,363380228 | 0,158490278 | |
| 200818_at | ATP5O | 6,793224861 | 6,634767296 | 0,158457565 | |
| 212335_at | GNS | 2,355089531 | 2,196649505 | 0,158440026 | |
| 214155_s_at | LARP4 | 2,86748657 | 2,709065209 | 0,158421361 | |
| 222574_s_at | DHX40 | 2,979690938 | 2,821324225 | 0,158366714 | |
| 209879_at | SELPLG | 0,425644434 | 0,26733076 | 0,158313674 | |
| 228798_x_at | MAZ | 0,425644434 | 0,26733076 | 0,158313674 | |
| 219899_x_at | NDOR1 | 0,747935635 | 0,589657387 | 0,158278249 | |
| 231927_at | ATF6 | 0,747935635 | 0,589657387 | 0,158278249 | |
| 216305_s_at | GCFC2 | 1,917954284 | 1,759685182 | 0,158269102 | |
| 227490_at | WDFY2 | 1,917954284 | 1,759685182 | 0,158269102 | |
| 1569039_s_at | ZNF677 | -1,173767127 | -1,332017329 | 0,158250202 | |
| 1569827_at | ATG7 | -1,173767127 | -1,332017329 | 0,158250202 | |
| 210847_x_at | TNFRSF25 | -1,173767127 | -1,332017329 | 0,158250202 | |
| 214069_at | ACSM2A /// ACS | -1,173767127 | -1,332017329 | 0,158250202 | |
| 214161_at | OSGIN2 | -1,173767127 | -1,332017329 | 0,158250202 | |
| 215365_at | CACNB2 | -1,173767127 | -1,332017329 | 0,158250202 | |
| 215417_at | EXOC6B | -1,173767127 | -1,332017329 | 0,158250202 | |
| 224505_s_at | PLCD4 | -1,173767127 | -1,332017329 | 0,158250202 | |
| 229830_at | - | -1,173767127 | -1,332017329 | 0,158250202 | |
| 230827_at | - | -1,173767127 | -1,332017329 | 0,158250202 | |
| 231184_at | DHX8 | -1,173767127 | -1,332017329 | 0,158250202 | |
| 233920_at | - | -1,173767127 | -1,332017329 | 0,158250202 | |
| 236171_at | ATP2C2 | -1,173767127 | -1,332017329 | 0,158250202 | |
| 237423_at | RSPO4 | -1,173767127 | -1,332017329 | 0,158250202 | |
| 239943_x_at | - | -1,173767127 | -1,332017329 | 0,158250202 | |
| 218092_s_at | AGFG1 | 3,775418946 | 3,617207412 | 0,158211534 | |
| 229198_at | USP35 | 0,334552598 | 0,176436073 | 0,158116525 | |
| 239422_at | GPC2 | 0,334552598 | 0,176436073 | 0,158116525 | |
| 217764_s_at | RAB31 | 4,455622373 | 4,297510916 | 0,158111457 | |
| 1554761_a_at | HEATR2 | 1,515954005 | 1,3579181 | 0,158035905 | |
| 213124_at | ZNF473 | 2,35929703 | 2,201267805 | 0,158029225 | |
| 58308_at | TRIM62 | 0,473009058 | 0,31498563 | 0,158023427 | |
| 221096_s_at | TMCO6 | 2,156646206 | 1,998633167 | 0,158013039 | |
| 225769_at | COG6 | 3,608715137 | 3,450709925 | 0,158005213 | |
| 209878_s_at | RELA | 1,473887017 | 1,315948728 | 0,157938289 | |
| 204170_s_at | CKS2 | 6,290925919 | 6,133021341 | 0,157904578 | |
| 205811_at | POLG2 | 2,102456302 | 1,944570415 | 0,157885887 | |
| 217880_at | CDC27 | 3,789518787 | 3,631821253 | 0,157697534 | |
| 222580_at | ZNF644 | 3,931919671 | 3,77423254 | 0,157687131 | |
| 201878_at | ARIH1 | 2,017152618 | 1,859476797 | 0,157675822 | |
| 225485_at | CEP41 | 1,789769151 | 1,632095957 | 0,157673194 | |
| 228853_at | STYX | 3,745192233 | 3,587527995 | 0,157664238 | |
| 225599_s_at | TRIQK | 1,187200746 | 1,029536893 | 0,157663853 | |
| 1553770_a_at | SLAMF9 | 0,133056859 | -0,024572586 | 0,157629445 | |
| 1558279_a_at | KDSR | 0,133056859 | -0,024572586 | 0,157629445 | |
| 203803_at | PCYOX1 | 0,133056859 | -0,024572586 | 0,157629445 | |
| 210325_at | CD1A | 0,133056859 | -0,024572586 | 0,157629445 | |

| | | | | | |
|---|---|---|---|---|---|
| 227361_at | HS3ST3B1 | 0,133056859 | -0,024572586 | 0,157629445 | |
| 244177_at | - | 0,133056859 | -0,024572586 | 0,157629445 | |
| 51192_at | SSH3 | 0,291167349 | 0,133587671 | 0,157579679 | |
| 228189_at | BAG4 | 4,552510269 | 4,394968439 | 0,157541831 | |
| 223403_s_at | POLR1B | 4,077568583 | 3,920040657 | 0,157527926 | |
| 209761_s_at | SP110 | 2,190100188 | 2,032717854 | 0,157382334 | |
| 216841_s_at | LOC100129518 / | 2,26825514 | 2,110887591 | 0,15736755 | |
| 222617_s_at | FAM204A | 3,177319542 | 3,019999252 | 0,157320289 | |
| 204548_at | STAR | 0,020670649 | -0,136643983 | 0,157314632 | |
| 210858_x_at | ATM | 0,020670649 | -0,136643983 | 0,157314632 | |
| 205663_at | PCBP3 | 2,605861638 | 2,448549499 | 0,157312139 | |
| 34858_at | KCTD2 | 2,424999855 | 2,267724404 | 0,157275452 | |
| 1557945_at | TCTE3 | 0,82905153 | 0,67181667 | 0,15723486 | |
| 222436_s_at | CHMP3 /// RNF1( | 3,081249977 | 2,924079008 | 0,15717097 | |
| 223140_s_at | DHX36 | 3,89689195 | 3,739727424 | 0,157164527 | |
| 1561581_at | - | -3,15396682 | -3,311072652 | 0,157105832 | |
| 1561912_at | - | -3,15396682 | -3,311072652 | 0,157105832 | |
| 203489_at | SIVA1 | 4,426844657 | 4,269747853 | 0,157096804 | |
| 212555_at | PRKAR1B | 0,962260738 | 0,805216064 | 0,157044674 | |
| 218241_at | GOLGA5 | 3,899061119 | 3,742106442 | 0,156954677 | |
| 1569949_at | GRK5 | 1,247462054 | 1,090553537 | 0,156908517 | |
| 238029_s_at | SLC16A14 | 1,247462054 | 1,090553537 | 0,156908517 | |
| 225159_s_at | ELK4 | 2,947914474 | 2,791007185 | 0,156907289 | |
| 218194_at | REXO2 | 5,681862829 | 5,525047463 | 0,156815367 | |
| 235759_at | - | 3,441193003 | 3,284389368 | 0,156803635 | |
| 200630_x_at | SET | 6,717147417 | 6,56038354 | 0,156763878 | |
| 226445_s_at | TRIM41 | 2,05655332 | 1,899812471 | 0,156740849 | |
| 1557918_s_at | SLC16A1 | 3,508091848 | 3,351366359 | 0,156725489 | |
| 226995_at | LOC642852 | 3,508091848 | 3,351366359 | 0,156725489 | |
| 201079_at | SYNGR2 | 3,966117062 | 3,809444866 | 0,156672196 | |
| 40420_at | STK10 | 1,950390104 | 1,793721801 | 0,156668303 | |
| 211707_s_at | IQCB1 | 2,710020346 | 2,553413592 | 0,156606754 | |
| 211439_at | SRSF7 | 1,143956113 | 0,987376598 | 0,156579516 | |
| 214753_at | N4BP2L2 | 1,143956113 | 0,987376598 | 0,156579516 | |
| 1569008_at | FAM201B /// LOC | 0,613492853 | 0,456918297 | 0,156574556 | |
| 201588_at | TXNL1 | 6,243628344 | 6,087128827 | 0,156499516 | |
| 226786_at | RFX1 | 2,654607516 | 2,498185745 | 0,156421772 | |
| 225386_s_at | HNRPLL | 4,712357174 | 4,555975485 | 0,156381689 | |
| 212299_at | NEK9 | 3,423218668 | 3,266872397 | 0,156346271 | |
| 236496_at | DEGS2 | -0,234278768 | -0,39061235 | 0,156333583 | |
| 201095_at | DAP | 2,352981171 | 2,196649505 | 0,156331666 | |
| 215239_x_at | ZNF273 | 2,352981171 | 2,196649505 | 0,156331666 | |
| 225719_s_at | MRPL55 | 2,352981171 | 2,196649505 | 0,156331666 | |
| 1553536_at | MBNL2 | -0,57445847 | -0,730778808 | 0,156320338 | |
| 1553691_at | B3GALNT2 | -0,57445847 | -0,730778808 | 0,156320338 | |
| 1556069_s_at | HIF3A | -0,57445847 | -0,730778808 | 0,156320338 | |
| 207832_at | BAIAP2 | -0,57445847 | -0,730778808 | 0,156320338 | |
| 210583_at | POLDIP3 | -0,57445847 | -0,730778808 | 0,156320338 | |
| 213106_at | ATP8A1 | -0,57445847 | -0,730778808 | 0,156320338 | |
| 216439_at | TNK2 | -0,57445847 | -0,730778808 | 0,156320338 | |
| 225612_s_at | B3GNT5 /// LOC1 | -0,57445847 | -0,730778808 | 0,156320338 | |
| 226187_at | CDS1 | -0,57445847 | -0,730778808 | 0,156320338 | |
| 232462_s_at | A1BG-AS1 | -0,57445847 | -0,730778808 | 0,156320338 | |
| 239350_at | MARVELD3 | -0,57445847 | -0,730778808 | 0,156320338 | |
| 239918_at | - | -0,57445847 | -0,730778808 | 0,156320338 | |
| 240206_at | TARS | -0,57445847 | -0,730778808 | 0,156320338 | |

| | | | | | |
|---|---|---|---|---|---|
| 244139_s_at | - | -0,57445847 | -0,730778808 | 0,156320338 | |
| 219581_at | TSEN2 | 2,768138014 | 2,611817827 | 0,156320187 | |
| 221000_s_at | KAZALD1 | 0,533835206 | 0,377530647 | 0,156304559 | |
| 219427_at | FAT4 | 2,55541096 | 2,39915583 | 0,15625513 | |
| 218461_at | GPN3 | 4,305625125 | 4,149442805 | 0,15618232 | |
| 207239_s_at | CDK16 | 1,805273023 | 1,64909809 | 0,156174933 | |
| 205717_x_at | PCDHGA1 /// PC | 1,40228911 | 1,246149888 | 0,156139221 | |
| 218478_s_at | ZCCHC8 | 3,217273782 | 3,061230246 | 0,156043536 | |
| 1552807_a_at | SIGLEC10 | 4,129607882 | 3,973663955 | 0,155943927 | |
| 220253_s_at | LRP12 | 2,755423794 | 2,599625088 | 0,155798706 | |
| 221781_s_at | DNAJC10 | 2,755423794 | 2,599625088 | 0,155798706 | |
| 218974_at | SOBP | 0,911589958 | 0,755840599 | 0,155749359 | |
| 222918_at | RAB9B | 0,911589958 | 0,755840599 | 0,155749359 | |
| 233350_s_at | TEX264 | 0,911589958 | 0,755840599 | 0,155749359 | |
| 205294_at | BAIAP2 | 0,359971644 | 0,204310755 | 0,155660888 | |
| 221811_at | PGAP3 | 0,359971644 | 0,204310755 | 0,155660888 | |
| 200669_s_at | UBE2D3 | 6,117965209 | 5,962345958 | 0,155619251 | |
| 208957_at | ERP44 | 1,261018186 | 1,105412836 | 0,15560535 | |
| 210044_s_at | LYL1 | 5,02431927 | 4,868790444 | 0,155528826 | |
| 225561_at | SELT | 4,206061174 | 4,050537457 | 0,155523717 | |
| 212754_s_at | MON2 | 2,127340679 | 1,971855016 | 0,155485663 | |
| 1558445_at | - | 1,210676994 | 1,055274826 | 0,155402168 | |
| 221752_at | SSH1 | 2,522021211 | 2,366632651 | 0,15538856 | |
| 1561726_s_at | - | -0,79540287 | -0,950686014 | 0,155283145 | |
| 1567030_at | SH3GL1P2 | -0,79540287 | -0,950686014 | 0,155283145 | |
| 206849_at | GABRG2 | -0,79540287 | -0,950686014 | 0,155283145 | |
| 207264_at | KDELR3 | -0,79540287 | -0,950686014 | 0,155283145 | |
| 207274_at | CHRNE | -0,79540287 | -0,950686014 | 0,155283145 | |
| 214990_at | PIGO | -0,79540287 | -0,950686014 | 0,155283145 | |
| 223858_at | ESRRB | -0,79540287 | -0,950686014 | 0,155283145 | |
| 223882_at | FAM172A | -0,79540287 | -0,950686014 | 0,155283145 | |
| 230330_at | PPM1D | -0,79540287 | -0,950686014 | 0,155283145 | |
| 231208_at | - | -0,79540287 | -0,950686014 | 0,155283145 | |
| 232055_at | SFXN1 | -0,79540287 | -0,950686014 | 0,155283145 | |
| 237316_at | ANKDD1A | -0,79540287 | -0,950686014 | 0,155283145 | |
| 237369_at | - | -0,79540287 | -0,950686014 | 0,155283145 | |
| 242305_at | LOC100507322 / | -0,79540287 | -0,950686014 | 0,155283145 | |
| 219292_at | THAP1 | 2,666540916 | 2,511262629 | 0,155278287 | |
| 65884_at | MAN1B1 | 1,339784194 | 1,184511019 | 0,155273175 | |
| 205621_at | ALKBH1 | 3,676882273 | 3,521663705 | 0,155218569 | |
| 200652_at | SSR2 | 5,249275201 | 5,094094626 | 0,155180575 | |
| 209523_at | TAF2 | 4,954075234 | 4,798904943 | 0,155170291 | |
| 1562628_at | KRT40 | -3,192553003 | -3,347710726 | 0,155157723 | |
| 219895_at | FAM70A | -3,192553003 | -3,347710726 | 0,155157723 | |
| 232305_at | HMGCLL1 | -3,192553003 | -3,347710726 | 0,155157723 | |
| 242118_x_at | - | -3,192553003 | -3,347710726 | 0,155157723 | |
| 243126_x_at | - | -3,192553003 | -3,347710726 | 0,155157723 | |
| 212338_at | MYO1D | 2,014487247 | 1,859476797 | 0,15501045 | |
| 201439_at | GBF1 | 1,414471577 | 1,259496994 | 0,154974583 | |
| 225900_at | EXOC6B | 2,708372067 | 2,553413592 | 0,154958475 | |
| 205704_s_at | ATP6V0A2 | 3,330897748 | 3,175947691 | 0,154950057 | |
| 226178_at | SOCS4 | 3,590934306 | 3,436090594 | 0,154843712 | |
| 211727_s_at | COX11 | 3,792633459 | 3,637795954 | 0,154837505 | |
| 232118_at | - | 1,709647566 | 1,55481039 | 0,154837175 | |
| 225766_s_at | TNPO1 | 3,89689195 | 3,742106442 | 0,154785508 | |
| 207268_x_at | ABI2 | 2,134723057 | 1,979940779 | 0,154782278 | |

| | | | | | |
|---|---|---|---|---|---|
| 224059_s_at | NUMBL | 1,04816921 | 0,893473268 | 0,154695943 | |
| 204838_s_at | MLH3 | 1,672933557 | 1,518263577 | 0,154669979 | |
| 1563808_at | MCF2L | 0,46522187 | 0,310717132 | 0,154504738 | |
| 206908_s_at | CLDN11 | 0,46522187 | 0,310717132 | 0,154504738 | |
| 222358_x_at | - | 0,46522187 | 0,310717132 | 0,154504738 | |
| 221509_at | DENR | 4,920655822 | 4,766161183 | 0,154494638 | |
| 218283_at | SS18L2 | 4,832231174 | 4,677791538 | 0,154439636 | |
| 218289_s_at | UBA5 | 3,373122675 | 3,218713651 | 0,154409024 | |
| 225523_at | CCDC142 /// MRI | 3,67603922 | 3,521663705 | 0,154375515 | |
| 200748_s_at | FTH1 | 6,661427392 | 6,507118179 | 0,154309212 | |
| 212805_at | PRUNE2 | 0,052199481 | -0,102100538 | 0,154300018 | |
| 219601_s_at | C10orf12 | 0,052199481 | -0,102100538 | 0,154300018 | |
| 234343_s_at | RASAL2 | 0,052199481 | -0,102100538 | 0,154300018 | |
| 243389_at | PRH1 /// PRH1-P | 0,052199481 | -0,102100538 | 0,154300018 | |
| 211942_x_at | RPL13A /// RPL1 | 7,589669872 | 7,435379856 | 0,154290016 | |
| 203451_at | LDB1 | 2,596981013 | 2,442710777 | 0,154270236 | |
| 222893_s_at | RPAP2 | 1,960118853 | 1,805902329 | 0,154216524 | |
| 205450_at | PHKA1 | 1,716223609 | 1,562009954 | 0,154213655 | |
| 222183_x_at | SNW1 | 1,716223609 | 1,562009954 | 0,154213655 | |
| 227111_at | ZBTB34 | 2,618203384 | 2,464004877 | 0,154198507 | |
| 209576_at | GNAI1 | 3,566577193 | 3,412387088 | 0,154190105 | |
| 217043_s_at | MFN1 | 2,827046023 | 2,672945617 | 0,154100406 | |
| 202587_s_at | AK1 | 0,376672223 | 0,222599095 | 0,154073129 | |
| 234695_x_at | FAM22A | 0,376672223 | 0,222599095 | 0,154073129 | |
| 202194_at | TMED5 | 5,110149007 | 4,956087342 | 0,154061664 | |
| 212914_at | CBX7 | 0,792179257 | 0,638150376 | 0,15402888 | |
| 220659_s_at | C7orf43 | 0,792179257 | 0,638150376 | 0,15402888 | |
| 205068_s_at | ARHGAP26 | 1,114389003 | 0,960386719 | 0,154002284 | |
| 223196_s_at | SESN2 | 1,114389003 | 0,960386719 | 0,154002284 | |
| 202597_at | IRF6 | 3,266269388 | 3,112391743 | 0,153877645 | |
| 244828_x_at | NAF1 | 3,266269388 | 3,112391743 | 0,153877645 | |
| 211392_s_at | PATZ1 | 1,426552034 | 1,272721751 | 0,153830283 | |
| 215942_s_at | GTSE1 | 1,331241794 | 1,177496821 | 0,153744973 | |
| 239133_at | CTDSPL2 | 1,331241794 | 1,177496821 | 0,153744973 | |
| 242300_at | - | 2,429008604 | 2,275409734 | 0,15359887 | |
| 1554470_s_at | ZBTB44 | 1,000476627 | 0,847075464 | 0,153401164 | |
| 218126_at | FAM82A2 | 2,626954721 | 2,473581079 | 0,153373642 | |
| 212352_s_at | TMED10 | 5,7855532 | 5,632180175 | 0,153373025 | |
| 208485_x_at | CFLAR | 1,726031811 | 1,572742379 | 0,153289432 | |
| 213934_s_at | ZNF23 | 1,726031811 | 1,572742379 | 0,153289432 | |
| 211587_x_at | CHRNA3 | 0,648296226 | 0,495031851 | 0,153264375 | |
| 214372_x_at | ERN2 | 0,648296226 | 0,495031851 | 0,153264375 | |
| 243115_at | - | 0,648296226 | 0,495031851 | 0,153264375 | |
| 211684_s_at | DYNC1I2 | 4,9941977 | 4,840945211 | 0,153252489 | |
| 242939_at | TFDP1 | 2,361396186 | 2,208167653 | 0,153228534 | |
| 224369_s_at | FBXO38 | 2,433006244 | 2,27978304 | 0,153223204 | |
| 231056_at | LOC339352 | 2,456762221 | 2,303602088 | 0,153160133 | |
| 223735_at | ARL6 | 0,181384709 | 0,028364014 | 0,153020695 | |
| 223778_at | KIF9 | 0,181384709 | 0,028364014 | 0,153020695 | |
| 230702_at | - | 0,181384709 | 0,028364014 | 0,153020695 | |
| 235043_at | FAM122A | 0,181384709 | 0,028364014 | 0,153020695 | |
| 216594_x_at | AKR1C1 | 4,509619341 | 4,356668262 | 0,152951079 | |
| 208831_x_at | SUPT6H | 1,973904239 | 1,820984782 | 0,152919457 | |
| 203512_at | TRAPPC3 | 3,302773593 | 3,149904996 | 0,152868598 | |
| 223275_at | PRMT6 | 3,183265014 | 3,030417996 | 0,152847018 | |
| 1557820_at | AFG3L2 | 5,410775419 | 5,257944504 | 0,152830915 | |

| | | | | | |
|---|---|---|---|---|---|
| 205679_x_at | ACAN | -0,196756757 | -0,34958478 | 0,152828023 | |
| 207176_s_at | CD80 | -0,196756757 | -0,34958478 | 0,152828023 | |
| 211808_s_at | CREBBP | -0,196756757 | -0,34958478 | 0,152828023 | |
| 214174_s_at | PDLIM4 | -0,196756757 | -0,34958478 | 0,152828023 | |
| 215974_at | - | -0,196756757 | -0,34958478 | 0,152828023 | |
| 243756_at | - | -0,196756757 | -0,34958478 | 0,152828023 | |
| 203859_s_at | PALM | 0,57058931 | 0,417770464 | 0,152818847 | |
| 205140_at | FPGT | 0,57058931 | 0,417770464 | 0,152818847 | |
| 231815_at | PHF12 | 0,57058931 | 0,417770464 | 0,152818847 | |
| 222654_at | IMPAD1 | 4,191091206 | 4,038328982 | 0,152762224 | |
| 1554380_at | NEK11 | -0,759588698 | -0,912331589 | 0,152742891 | |
| 1554857_at | ELMO2 | -0,759588698 | -0,912331589 | 0,152742891 | |
| 1557729_at | GRK5 | -0,759588698 | -0,912331589 | 0,152742891 | |
| 1559307_s_at | RBL1 | -0,759588698 | -0,912331589 | 0,152742891 | |
| 1569422_at | FAM129C | -0,759588698 | -0,912331589 | 0,152742891 | |
| 202178_at | PRKCZ | -0,759588698 | -0,912331589 | 0,152742891 | |
| 203726_s_at | LAMA3 | -0,759588698 | -0,912331589 | 0,152742891 | |
| 204694_at | AFP | -0,759588698 | -0,912331589 | 0,152742891 | |
| 205096_at | POM121 | -0,759588698 | -0,912331589 | 0,152742891 | |
| 207925_at | CST5 | -0,759588698 | -0,912331589 | 0,152742891 | |
| 211682_x_at | UGT2B28 | -0,759588698 | -0,912331589 | 0,152742891 | |
| 212272_at | LPIN1 | -0,759588698 | -0,912331589 | 0,152742891 | |
| 213991_s_at | HS3ST1 | -0,759588698 | -0,912331589 | 0,152742891 | |
| 226985_at | FGD5 | -0,759588698 | -0,912331589 | 0,152742891 | |
| 233619_at | - | -0,759588698 | -0,912331589 | 0,152742891 | |
| 239756_at | - | -0,759588698 | -0,912331589 | 0,152742891 | |
| 241230_at | CA12 | -0,759588698 | -0,912331589 | 0,152742891 | |
| 218297_at | FAM188A | 1,732533761 | 1,579853228 | 0,152680533 | |
| 201872_s_at | ABCE1 | 5,243596157 | 5,090987226 | 0,152608931 | |
| 218428_s_at | REV1 | 3,101484927 | 2,949006836 | 0,152478091 | |
| 216700_at | TRIO | 0,072842263 | -0,079522948 | 0,152365211 | |
| 219741_x_at | ZNF552 | 0,072842263 | -0,079522948 | 0,152365211 | |
| 223563_at | GNB1L | 0,072842263 | -0,079522948 | 0,152365211 | |
| 238656_at | - | 0,072842263 | -0,079522948 | 0,152365211 | |
| 241689_at | - | 0,072842263 | -0,079522948 | 0,152365211 | |
| 227470_at | ZNF48 | 1,880387527 | 1,728030156 | 0,152357371 | |
| 208146_s_at | CPVL | 2,243430036 | 2,091116676 | 0,152313361 | |
| 215143_at | DPY19L2P2 | 1,073999308 | 0,921731975 | 0,152267333 | |
| 225042_s_at | CSRNP2 | 1,073999308 | 0,921731975 | 0,152267333 | |
| 213554_s_at | CDV3 | 2,55541096 | 2,403170156 | 0,152240804 | |
| 213203_at | SNAPC5 | 3,231115193 | 3,078904771 | 0,152210422 | |
| 225973_at | TAP2 | 3,284083774 | 3,131875186 | 0,152208588 | |
| 227105_at | CSPP1 | 3,534378463 | 3,382199319 | 0,152179144 | |
| 1561775_at | - | -3,250025915 | -3,402185466 | 0,152159551 | |
| 244169_x_at | - | -3,250025915 | -3,402185466 | 0,152159551 | |
| 212125_at | RANGAP1 | 2,218170265 | 2,066015817 | 0,152154448 | |
| 200002_at | RPL35 | 7,612266859 | 7,46021759 | 0,152049269 | |
| 38043_at | FAM3A | 1,514068246 | 1,36204866 | 0,152019586 | |
| 202764_at | STIM1 | 1,446464021 | 1,294497133 | 0,151966888 | |
| 225514_at | NOP9 | 1,446464021 | 1,294497133 | 0,151966888 | |
| 1554553_s_at | YIF1B | 3,796517367 | 3,644594022 | 0,151923345 | |
| 218395_at | ACTR6 | 3,193905507 | 3,042049843 | 0,151855664 | |
| 201296_s_at | WSB1 | 4,376357713 | 4,224525141 | 0,151832572 | |
| 218961_s_at | PNKP | 1,742232072 | 1,590454215 | 0,151777857 | |
| 224601_at | LOC100507246 | 4,471743273 | 4,319980141 | 0,151763132 | |
| 1560089_at | LOC100289019 | 0,401366113 | 0,249604176 | 0,151761937 | |

| | | | | | |
|---|---|---|---|---|---|
| 227197_at | ARHGEF26 | 0,401366113 | 0,249604176 | 0,151761937 | |
| 243909_x_at | GUSBP4 | 0,401366113 | 0,249604176 | 0,151761937 | |
| 235497_at | LOC643837 | 1,139070151 | 0,987376598 | 0,151693553 | |
| 225387_at | TSPAN5 | 3,484209953 | 3,332545245 | 0,151664708 | |
| 219360_s_at | TRPM4 | 2,197169074 | 2,045615873 | 0,151553202 | |
| 201454_s_at | LOC100653042 / | 3,19743497 | 3,045906378 | 0,151528592 | |
| 243529_at | MARS2 | 2,082235068 | 1,930732138 | 0,15150293 | |
| 227488_at | MGC16121 /// MI | 0,95671774 | 0,805216064 | 0,151501676 | |
| 227563_at | FAM27E3 | 0,95671774 | 0,805216064 | 0,151501676 | |
| 207122_x_at | SULT1A2 | 1,305306849 | 1,153866761 | 0,151440088 | |
| 226370_at | KLHL15 | 3,747601714 | 3,596320795 | 0,151280919 | |
| 232296_s_at | GFM1 | 2,254767157 | 2,103505212 | 0,151261945 | |
| 219291_at | DTWD1 | 2,609398637 | 2,458228496 | 0,151170142 | |
| 226539_s_at | CCDC42B | 0,822971223 | 0,67181667 | 0,151154553 | |
| 1561571_at | LOC730139 | 0,30867765 | 0,157549243 | 0,151128407 | |
| 216577_at | - | 0,30867765 | 0,157549243 | 0,151128407 | |
| 232926_x_at | ANKRD19P | 1,027166904 | 0,876248481 | 0,150918423 | |
| 204648_at | NPR1 | 0,503742507 | 0,352836757 | 0,15090575 | |
| 206446_s_at | CELA2A /// CELA | 0,503742507 | 0,352836757 | 0,15090575 | |
| 229723_at | TAGAP | 0,503742507 | 0,352836757 | 0,15090575 | |
| 235239_at | QSOX2 | 0,503742507 | 0,352836757 | 0,15090575 | |
| 226430_at | RELL1 | 2,410880861 | 2,259997914 | 0,150882947 | |
| 225114_at | AGPS | 3,913439491 | 3,76256191 | 0,150877581 | |
| 201564_s_at | FSCN1 /// LOC10 | 3,32445605 | 3,173599504 | 0,150856546 | |
| 218018_at | PDXK | 4,393896578 | 4,243120651 | 0,150775927 | |
| 204347_at | AK4 /// LOC1005 | 2,845159691 | 2,694398145 | 0,150761547 | |
| 222427_s_at | LARS | 5,369653912 | 5,218906902 | 0,15074701 | |
| 212164_at | TMEM183A /// TM | 1,593009234 | 1,442269454 | 0,15073978 | |
| 212490_at | DNAJC8 | 1,593009234 | 1,442269454 | 0,15073978 | |
| 235215_at | ERCC4 | 1,67631007 | 1,525647381 | 0,15066269 | |
| 218782_s_at | ATAD2 | 5,044219854 | 4,89361379 | 0,150606064 | |
| 1554819_a_at | ITGA11 | -0,172274514 | -0,322870281 | 0,150595767 | |
| 1560509_at | - | -0,172274514 | -0,322870281 | 0,150595767 | |
| 203642_s_at | COBLL1 | -0,172274514 | -0,322870281 | 0,150595767 | |
| 206080_at | PLCH2 | -0,172274514 | -0,322870281 | 0,150595767 | |
| 206683_at | ZNF165 | -0,172274514 | -0,322870281 | 0,150595767 | |
| 206813_at | CTF1 | -0,172274514 | -0,322870281 | 0,150595767 | |
| 213157_s_at | LOC339166 /// W | -0,172274514 | -0,322870281 | 0,150595767 | |
| 220307_at | CD244 | -0,172274514 | -0,322870281 | 0,150595767 | |
| 220449_at | LOC79015 | -0,172274514 | -0,322870281 | 0,150595767 | |
| 235068_at | ZDHHC21 | -0,172274514 | -0,322870281 | 0,150595767 | |
| 238886_at | TMED10 | 0,209623097 | 0,059218869 | 0,150404229 | |
| 238977_at | MCM6 | 0,209623097 | 0,059218869 | 0,150404229 | |
| 243338_at | CSNK1A1 | 0,209623097 | 0,059218869 | 0,150404229 | |
| 200967_at | PPIB | 6,463055332 | 6,312753142 | 0,15030219 | |
| 223713_at | RSPH3 | 0,417596971 | 0,26733076 | 0,150266211 | |
| 231331_at | - | 0,417596971 | 0,26733076 | 0,150266211 | |
| 213927_at | MAP3K9 | 1,318332599 | 1,16809119 | 0,150241409 | |
| 214721_x_at | CDC42EP4 | 1,868628927 | 1,718396606 | 0,150232321 | |
| 221797_at | C17orf90 | 1,46610492 | 1,315948728 | 0,150156192 | |
| 220482_s_at | SERGEF | 0,682279745 | 0,532164371 | 0,150115375 | |
| 225210_s_at | FAM103A1 | 4,751298963 | 4,601232927 | 0,150066036 | |
| 200013_at | RPL24 | 7,789050711 | 7,639040799 | 0,150009912 | |
| 226700_at | U2AF1L4 | 1,940595304 | 1,790660532 | 0,149934772 | |
| 227407_at | TAPT1 | 1,940595304 | 1,790660532 | 0,149934772 | |
| 221825_at | ANGEL2 | 2,889473182 | 2,739547836 | 0,149925346 | |

| | | | | | |
|---|---|---|---|---|---|
| 1558250_s_at | - | 1,215326747 | 1,065442793 | 0,149883954 | |
| 224829_at | CPEB4 | 1,215326747 | 1,065442793 | 0,149883954 | |
| 201949_x_at | CAPZB | 4,859592153 | 4,709745554 | 0,149846598 | |
| 232902_s_at | RARS2 | 3,791855421 | 3,642048499 | 0,149806922 | |
| 226284_at | ZBTB2 | 2,446911392 | 2,297144887 | 0,149766505 | |
| 1567214_a_at | PNN | 4,342753543 | 4,192989865 | 0,149763678 | |
| 1553458_at | VSTM4 | -0,496991414 | -0,646746079 | 0,149754665 | |
| 207838_x_at | PBXIP1 | -0,496991414 | -0,646746079 | 0,149754665 | |
| 230715_at | ZNF518B | -0,496991414 | -0,646746079 | 0,149754665 | |
| 239059_at | DNAH1 | -0,496991414 | -0,646746079 | 0,149754665 | |
| 244015_at | - | -0,496991414 | -0,646746079 | 0,149754665 | |
| 60815_at | POLR2J4 | -0,777586431 | -0,927308905 | 0,149722473 | |
| 212590_at | RRAS2 | 3,821129803 | 3,671470423 | 0,149659379 | |
| 65493_at | HEATR6 | 1,375535468 | 1,225894807 | 0,149640661 | |
| 232003_at | PNMAL2 | 0,103263601 | -0,046302147 | 0,149565748 | |
| 242390_at | - | 0,103263601 | -0,046302147 | 0,149565748 | |
| 207595_s_at | BMP1 | 0,606430281 | 0,456918297 | 0,149511984 | |
| 224854_s_at | SLAIN2 | 0,606430281 | 0,456918297 | 0,149511984 | |
| 230046_at | - | 0,606430281 | 0,456918297 | 0,149511984 | |
| 237247_at | USP51 | 0,841135803 | 0,691645685 | 0,149490118 | |
| 212289_at | ANKRD12 | 1,473887017 | 1,324440845 | 0,149446172 | |
| 224827_at | UBTD2 | 1,841816345 | 1,692388143 | 0,149428203 | |
| 222700_at | ATL2 | 4,164288643 | 4,014909928 | 0,149378714 | |
| 1554875_at | CAMKMT | -1,888254346 | -2,037551727 | 0,149297381 | |
| 1562738_a_at | LOC100130855 | -1,888254346 | -2,037551727 | 0,149297381 | |
| 1563082_at | LINC00486 | -1,888254346 | -2,037551727 | 0,149297381 | |
| 1569235_a_at | - | -1,888254346 | -2,037551727 | 0,149297381 | |
| 202335_s_at | UBE2B | -1,888254346 | -2,037551727 | 0,149297381 | |
| 206990_at | TNR | -1,888254346 | -2,037551727 | 0,149297381 | |
| 209168_at | GPM6B | -1,888254346 | -2,037551727 | 0,149297381 | |
| 210412_at | GRIN2B | -1,888254346 | -2,037551727 | 0,149297381 | |
| 210536_s_at | SPAM1 | -1,888254346 | -2,037551727 | 0,149297381 | |
| 216592_at | MAGEC3 | -1,888254346 | -2,037551727 | 0,149297381 | |
| 217239_x_at | LOC100508797 | -1,888254346 | -2,037551727 | 0,149297381 | |
| 228950_s_at | WLS | -1,888254346 | -2,037551727 | 0,149297381 | |
| 231683_at | GLYAT | -1,888254346 | -2,037551727 | 0,149297381 | |
| 233637_at | DCAF8 | -1,888254346 | -2,037551727 | 0,149297381 | |
| 234019_at | - | -1,888254346 | -2,037551727 | 0,149297381 | |
| 237200_at | - | -1,888254346 | -2,037551727 | 0,149297381 | |
| 239778_x_at | - | -1,888254346 | -2,037551727 | 0,149297381 | |
| 241546_at | SPATA5 | -1,888254346 | -2,037551727 | 0,149297381 | |
| 217792_at | SNX5 | 4,481426562 | 4,332138021 | 0,149288541 | |
| 1553729_s_at | LRRC43 | -1,937711681 | -2,086984744 | 0,149273062 | |
| 1555401_at | SOHLH2 | -1,937711681 | -2,086984744 | 0,149273062 | |
| 1557798_at | CNPY2 | -1,937711681 | -2,086984744 | 0,149273062 | |
| 1560924_at | - | -1,937711681 | -2,086984744 | 0,149273062 | |
| 1561398_at | - | -1,937711681 | -2,086984744 | 0,149273062 | |
| 1561693_at | LOC400794 | -1,937711681 | -2,086984744 | 0,149273062 | |
| 1563064_at | - | -1,937711681 | -2,086984744 | 0,149273062 | |
| 1565882_at | - | -1,937711681 | -2,086984744 | 0,149273062 | |
| 202363_at | SPOCK1 | -1,937711681 | -2,086984744 | 0,149273062 | |
| 207359_at | CAMKK2 | -1,937711681 | -2,086984744 | 0,149273062 | |
| 209198_s_at | SYT11 | -1,937711681 | -2,086984744 | 0,149273062 | |
| 210349_at | CAMK4 | -1,937711681 | -2,086984744 | 0,149273062 | |
| 216163_at | - | -1,937711681 | -2,086984744 | 0,149273062 | |
| 216967_at | GAP43 | -1,937711681 | -2,086984744 | 0,149273062 | |

| | | | | | |
|---|---|---|---|---|---|
| 220434_at | ADCK4 | -1,937711681 | -2,086984744 | 0,149273062 | |
| 220718_at | HEXA-AS1 | -1,937711681 | -2,086984744 | 0,149273062 | |
| 221658_s_at | IL21R | -1,937711681 | -2,086984744 | 0,149273062 | |
| 225937_at | CUX1 | -1,937711681 | -2,086984744 | 0,149273062 | |
| 232599_at | EXOC6 | -1,937711681 | -2,086984744 | 0,149273062 | |
| 232824_at | - | -1,937711681 | -2,086984744 | 0,149273062 | |
| 233975_at | PRNT | -1,937711681 | -2,086984744 | 0,149273062 | |
| 235360_at | PLEKHM3 | -1,937711681 | -2,086984744 | 0,149273062 | |
| 237694_at | - | -1,937711681 | -2,086984744 | 0,149273062 | |
| 237704_at | - | -1,937711681 | -2,086984744 | 0,149273062 | |
| 237923_at | - | -1,937711681 | -2,086984744 | 0,149273062 | |
| 241309_at | - | -1,937711681 | -2,086984744 | 0,149273062 | |
| 241559_at | - | -1,937711681 | -2,086984744 | 0,149273062 | |
| 242391_at | - | -1,937711681 | -2,086984744 | 0,149273062 | |
| 219646_at | DEF8 | 3,5222335 | 3,373018423 | 0,149215077 | |
| 207035_at | SLC30A3 | 1,69307511 | 1,543943227 | 0,149131883 | |
| 228101_at | APBA1 | 1,69307511 | 1,543943227 | 0,149131883 | |
| 230570_at | EIF3H | 1,69307511 | 1,543943227 | 0,149131883 | |
| 1555515_a_at | FAM189B | -1,812418401 | -1,961547147 | 0,149128746 | |
| 1557633_at | POM121L8P | -1,812418401 | -1,961547147 | 0,149128746 | |
| 1560026_at | - | -1,812418401 | -1,961547147 | 0,149128746 | |
| 1569338_at | - | -1,812418401 | -1,961547147 | 0,149128746 | |
| 1569580_a_at | - | -1,812418401 | -1,961547147 | 0,149128746 | |
| 216021_s_at | GLRA3 | -1,812418401 | -1,961547147 | 0,149128746 | |
| 220706_at | ADAMTS7 | -1,812418401 | -1,961547147 | 0,149128746 | |
| 220832_at | TLR8 | -1,812418401 | -1,961547147 | 0,149128746 | |
| 224050_s_at | - | -1,812418401 | -1,961547147 | 0,149128746 | |
| 229151_at | SLC14A1 | -1,812418401 | -1,961547147 | 0,149128746 | |
| 232770_at | TUSC3 | -1,812418401 | -1,961547147 | 0,149128746 | |
| 233569_at | COPG2 | -1,812418401 | -1,961547147 | 0,149128746 | |
| 234027_at | CCDC129 | -1,812418401 | -1,961547147 | 0,149128746 | |
| 235956_at | KIAA1377 | -1,812418401 | -1,961547147 | 0,149128746 | |
| 240495_at | - | -1,812418401 | -1,961547147 | 0,149128746 | |
| 241192_at | - | -1,812418401 | -1,961547147 | 0,149128746 | |
| 242606_at | - | -1,812418401 | -1,961547147 | 0,149128746 | |
| 223680_at | ZNF607 | 2,827046023 | 2,677924589 | 0,149121434 | |
| 203493_s_at | CEP57 | 3,182077878 | 3,033010968 | 0,149066911 | |
| 204794_at | DUSP2 | 0,984222186 | 0,835239052 | 0,148983133 | |
| 230098_at | PHF20L1 | 0,984222186 | 0,835239052 | 0,148983133 | |
| 232087_at | CXorf23 | 0,984222186 | 0,835239052 | 0,148983133 | |
| 213269_at | ZNF248 | 0,917308734 | 0,768343791 | 0,148964944 | |
| 1553202_at | STOX1 | -2,01371626 | -2,162639771 | 0,148923511 | |
| 1553534_at | NLRP10 | -2,01371626 | -2,162639771 | 0,148923511 | |
| 1554041_at | TMEM239 | -2,01371626 | -2,162639771 | 0,148923511 | |
| 1557214_at | LOC100506107 | -2,01371626 | -2,162639771 | 0,148923511 | |
| 1561038_at | ZNF81 | -2,01371626 | -2,162639771 | 0,148923511 | |
| 1563659_at | HERC6 | -2,01371626 | -2,162639771 | 0,148923511 | |
| 1566194_at | - | -2,01371626 | -2,162639771 | 0,148923511 | |
| 1570635_at | - | -2,01371626 | -2,162639771 | 0,148923511 | |
| 206178_at | PLA2G5 | -2,01371626 | -2,162639771 | 0,148923511 | |
| 215397_x_at | - | -2,01371626 | -2,162639771 | 0,148923511 | |
| 223890_at | - | -2,01371626 | -2,162639771 | 0,148923511 | |
| 228224_at | PRELP | -2,01371626 | -2,162639771 | 0,148923511 | |
| 241143_at | - | -2,01371626 | -2,162639771 | 0,148923511 | |
| 225270_at | NEO1 | 1,774096855 | 1,6252386 | 0,148858255 | |
| 1554112_a_at | ULK2 | -1,763254337 | -1,912089812 | 0,148835475 | |

| | | | | | |
|---|---|---|---|---|---|
| 1554665_at | ZNF586 | -1,763254337 | -1,912089812 | 0,148835475 | |
| 1554882_at | ERCC8 | -1,763254337 | -1,912089812 | 0,148835475 | |
| 1556387_at | LOC100507389 | -1,763254337 | -1,912089812 | 0,148835475 | |
| 1557707_at | - | -1,763254337 | -1,912089812 | 0,148835475 | |
| 1560935_s_at | LOC284669 | -1,763254337 | -1,912089812 | 0,148835475 | |
| 1564786_at | LOC338667 | -1,763254337 | -1,912089812 | 0,148835475 | |
| 1570318_at | - | -1,763254337 | -1,912089812 | 0,148835475 | |
| 205828_at | MMP3 | -1,763254337 | -1,912089812 | 0,148835475 | |
| 206068_s_at | ACADL | -1,763254337 | -1,912089812 | 0,148835475 | |
| 207155_at | TBX5 | -1,763254337 | -1,912089812 | 0,148835475 | |
| 207299_s_at | GRM1 | -1,763254337 | -1,912089812 | 0,148835475 | |
| 207399_at | BFSP2 | -1,763254337 | -1,912089812 | 0,148835475 | |
| 207889_at | SARDH | -1,763254337 | -1,912089812 | 0,148835475 | |
| 211460_at | TTTY9A /// TTTY | -1,763254337 | -1,912089812 | 0,148835475 | |
| 216991_at | ZNF224 | -1,763254337 | -1,912089812 | 0,148835475 | |
| 217355_at | - | -1,763254337 | -1,912089812 | 0,148835475 | |
| 224048_at | USP44 | -1,763254337 | -1,912089812 | 0,148835475 | |
| 224208_at | PLEKHA8 | -1,763254337 | -1,912089812 | 0,148835475 | |
| 232458_at | COL3A1 | -1,763254337 | -1,912089812 | 0,148835475 | |
| 235243_at | CLIP3 | -1,763254337 | -1,912089812 | 0,148835475 | |
| 236206_at | FAM53A | -1,763254337 | -1,912089812 | 0,148835475 | |
| 237871_x_at | - | -1,763254337 | -1,912089812 | 0,148835475 | |
| 239052_at | - | -1,763254337 | -1,912089812 | 0,148835475 | |
| 241670_x_at | LOC729177 | -1,763254337 | -1,912089812 | 0,148835475 | |
| 242227_at | - | -1,763254337 | -1,912089812 | 0,148835475 | |
| 243104_at | - | -1,763254337 | -1,912089812 | 0,148835475 | |
| 243743_at | - | -1,763254337 | -1,912089812 | 0,148835475 | |
| 1563225_a_at | - | -0,706943342 | -0,855751026 | 0,148807684 | |
| 1566363_at | DNTT | -0,706943342 | -0,855751026 | 0,148807684 | |
| 206328_at | CDH15 | -0,706943342 | -0,855751026 | 0,148807684 | |
| 207933_at | ZP2 | -0,706943342 | -0,855751026 | 0,148807684 | |
| 212728_at | DLG3 | -0,706943342 | -0,855751026 | 0,148807684 | |
| 215761_at | DMXL2 | -0,706943342 | -0,855751026 | 0,148807684 | |
| 218413_s_at | ZNF639 | -0,706943342 | -0,855751026 | 0,148807684 | |
| 218880_at | FOSL2 | -0,706943342 | -0,855751026 | 0,148807684 | |
| 231072_at | MIDN | -0,706943342 | -0,855751026 | 0,148807684 | |
| 232677_at | - | -0,706943342 | -0,855751026 | 0,148807684 | |
| 232910_at | BBIP1 | -0,706943342 | -0,855751026 | 0,148807684 | |
| 233428_at | - | -0,706943342 | -0,855751026 | 0,148807684 | |
| 234200_at | - | -0,706943342 | -0,855751026 | 0,148807684 | |
| 234322_at | - | -0,706943342 | -0,855751026 | 0,148807684 | |
| 234380_x_at | - | -0,706943342 | -0,855751026 | 0,148807684 | |
| 236825_at | - | -0,706943342 | -0,855751026 | 0,148807684 | |
| 239384_at | FLJ44342 | -0,706943342 | -0,855751026 | 0,148807684 | |
| 74694_s_at | RABEP2 | 1,614289682 | 1,465490932 | 0,14879875 | |
| 218519_at | SLC35A5 | 2,227406889 | 2,078620836 | 0,148786053 | |
| 218239_s_at | GTPBP4 | 5,23446293 | 5,085689237 | 0,148773693 | |
| 209326_at | SLC35A2 | 4,707414703 | 4,558677353 | 0,14873735 | |
| 230327_at | LOC730098 | 0,228146222 | 0,079428135 | 0,148718086 | |
| 239774_at | - | 0,228146222 | 0,079428135 | 0,148718086 | |
| 208805_at | KIAA0391 /// PSM | 6,988791461 | 6,840082874 | 0,148708588 | |
| 202296_s_at | RER1 | 4,416786695 | 4,268097973 | 0,148688722 | |
| 203306_s_at | SLC35A1 | 4,476593042 | 4,327920804 | 0,148672238 | |
| 200084_at | C11orf58 | 5,649802912 | 5,501134613 | 0,148668298 | |
| 225164_s_at | EIF2AK4 | 1,850809354 | 1,702196344 | 0,14861301 | |
| 216913_s_at | RRP12 | 2,90394695 | 2,755336312 | 0,148610638 | |

| | | | | | |
|---|---|---|---|---|---|
| 1556829_at | TIPARP-AS1 | -2,063149277 | -2,211750327 | 0,14860105 | |
| 1557645_at | - | -2,063149277 | -2,211750327 | 0,14860105 | |
| 1558354_s_at | LOC100505725 | -2,063149277 | -2,211750327 | 0,14860105 | |
| 1558652_at | KANSL3 | -2,063149277 | -2,211750327 | 0,14860105 | |
| 1561370_at | LOC100505716 | -2,063149277 | -2,211750327 | 0,14860105 | |
| 206680_at | CD5L | -2,063149277 | -2,211750327 | 0,14860105 | |
| 214723_x_at | ANKRD36 | -2,063149277 | -2,211750327 | 0,14860105 | |
| 215710_at | ST3GAL4 | -2,063149277 | -2,211750327 | 0,14860105 | |
| 216209_at | LOC400084 | -2,063149277 | -2,211750327 | 0,14860105 | |
| 216405_at | - | -2,063149277 | -2,211750327 | 0,14860105 | |
| 219153_s_at | THSD4 | -2,063149277 | -2,211750327 | 0,14860105 | |
| 223801_s_at | APOL4 | -2,063149277 | -2,211750327 | 0,14860105 | |
| 229658_at | BAZ1B | -2,063149277 | -2,211750327 | 0,14860105 | |
| 232856_at | LRRC55 | -2,063149277 | -2,211750327 | 0,14860105 | |
| 234233_s_at | - | -2,063149277 | -2,211750327 | 0,14860105 | |
| 234411_x_at | CD44 | -2,063149277 | -2,211750327 | 0,14860105 | |
| 235567_at | RORA | -2,063149277 | -2,211750327 | 0,14860105 | |
| 236342_at | - | -2,063149277 | -2,211750327 | 0,14860105 | |
| 238017_at | SDR16C5 | -2,063149277 | -2,211750327 | 0,14860105 | |
| 242257_at | - | -2,063149277 | -2,211750327 | 0,14860105 | |
| 242284_at | LINC00466 | -2,063149277 | -2,211750327 | 0,14860105 | |
| 205412_at | ACAT1 | 4,208686884 | 4,060103278 | 0,148583606 | |
| 1556285_s_at | PPA2 | 4,045288912 | 3,896739828 | 0,148549084 | |
| 200892_s_at | TRA2B | 5,433900161 | 5,285389828 | 0,148510333 | |
| 202923_s_at | GCLC | 3,939667505 | 3,79118048 | 0,148487025 | |
| 1552580_at | TRIML2 | -1,71423189 | -1,862647763 | 0,148415873 | |
| 1556277_a_at | PAPD4 | -1,71423189 | -1,862647763 | 0,148415873 | |
| 1556437_at | LOC253805 | -1,71423189 | -1,862647763 | 0,148415873 | |
| 1558346_at | COX17 | -1,71423189 | -1,862647763 | 0,148415873 | |
| 1560881_a_at | LINC00112 | -1,71423189 | -1,862647763 | 0,148415873 | |
| 1567540_at | - | -1,71423189 | -1,862647763 | 0,148415873 | |
| 205792_at | WISP2 | -1,71423189 | -1,862647763 | 0,148415873 | |
| 209872_s_at | PKP3 | -1,71423189 | -1,862647763 | 0,148415873 | |
| 215523_at | ZNF391 | -1,71423189 | -1,862647763 | 0,148415873 | |
| 216410_at | - | -1,71423189 | -1,862647763 | 0,148415873 | |
| 229294_at | JPH3 | -1,71423189 | -1,862647763 | 0,148415873 | |
| 229639_s_at | SLC19A1 | -1,71423189 | -1,862647763 | 0,148415873 | |
| 231140_at | TPPP2 | -1,71423189 | -1,862647763 | 0,148415873 | |
| 232295_at | GFM1 | -1,71423189 | -1,862647763 | 0,148415873 | |
| 234507_at | - | -1,71423189 | -1,862647763 | 0,148415873 | |
| 236947_at | - | -1,71423189 | -1,862647763 | 0,148415873 | |
| 237818_at | LARP6 | -1,71423189 | -1,862647763 | 0,148415873 | |
| 241254_at | - | -1,71423189 | -1,862647763 | 0,148415873 | |
| 241712_at | CBY3 | -1,71423189 | -1,862647763 | 0,148415873 | |
| 241729_at | DOK6 | -1,71423189 | -1,862647763 | 0,148415873 | |
| 242060_x_at | PHF11 | -1,71423189 | -1,862647763 | 0,148415873 | |
| 242460_at | - | -1,71423189 | -1,862647763 | 0,148415873 | |
| 242936_at | - | -1,71423189 | -1,862647763 | 0,148415873 | |
| 243353_at | - | -1,71423189 | -1,862647763 | 0,148415873 | |
| 244198_at | RANBP17 | -1,71423189 | -1,862647763 | 0,148415873 | |
| 244546_at | CYCS | -1,71423189 | -1,862647763 | 0,148415873 | |
| 1558460_at | ABCC5 | 0,853119696 | 0,704715219 | 0,148404477 | |
| 201054_at | HNRNPA0 | 5,487139709 | 5,338762121 | 0,148377588 | |
| 1568935_at | - | -3,32387526 | -3,472251727 | 0,148376467 | |
| 208672_s_at | SRSF3 | 6,577519271 | 6,429166008 | 0,148353264 | |
| 218127_at | NFYB | 2,640847372 | 2,492544876 | 0,148302496 | |

| | | | | | |
|---|---|---|---|---|---|
| 205095_s_at | ATP6V0A1 | 0,702292136 | 0,553993624 | 0,148298512 | |
| 241817_at | C3orf62 /// MIR42 | 0,702292136 | 0,553993624 | 0,148298512 | |
| 201376_s_at | HNRNPF | 3,117719377 | 2,969455976 | 0,148263401 | |
| 225892_at | IREB2 | 4,673180244 | 4,524995365 | 0,148184879 | |
| 222230_s_at | ACTR10 | 5,220364653 | 5,072200946 | 0,148163706 | |
| 1553361_x_at | FBXL18 | 1,578645857 | 1,43051703 | 0,148128826 | |
| 201157_s_at | NMT1 | 3,86543671 | 3,717332003 | 0,148104707 | |
| 1566954_at | - | 0,44160593 | 0,293518836 | 0,148087094 | |
| 221527_s_at | PARD3 | 0,44160593 | 0,293518836 | 0,148087094 | |
| 223791_at | FAM27A /// FAM2 | 0,44160593 | 0,293518836 | 0,148087094 | |
| 224375_at | - | 0,44160593 | 0,293518836 | 0,148087094 | |
| 241958_at | - | 0,44160593 | 0,293518836 | 0,148087094 | |
| 37652_at | CABIN1 | 1,643909574 | 1,495882679 | 0,148026896 | |
| 220419_s_at | USP25 | 3,612245157 | 3,464222256 | 0,148022901 | |
| 214060_at | MIR5096 /// SSBI | 1,442503549 | 1,294497133 | 0,148006416 | |
| 204782_at | - | 0,995078803 | 0,847075464 | 0,148003339 | |
| 206081_at | SLC24A1 | 0,995078803 | 0,847075464 | 0,148003339 | |
| 242007_at | - | 0,995078803 | 0,847075464 | 0,148003339 | |
| 225053_at | CNOT7 | 6,154286309 | 6,006305764 | 0,147980546 | |
| 212276_at | LPIN1 | 1,394109935 | 1,246149888 | 0,147960047 | |
| 227778_at | HEATR7A /// LOC | 1,394109935 | 1,246149888 | 0,147960047 | |
| 236104_at | HNRPLL | 1,394109935 | 1,246149888 | 0,147960047 | |
| 217047_s_at | FAM13A | 2,344516784 | 2,196649505 | 0,147867279 | |
| 213801_x_at | RPSA /// RPSAP1 | 7,884373508 | 7,736568868 | 0,14780464 | |
| 1553887_at | C19orf75 | -2,138804305 | -2,286583451 | 0,147779145 | |
| 1554121_at | HSD17B12 | -2,138804305 | -2,286583451 | 0,147779145 | |
| 1555671_at | ICA1L | -2,138804305 | -2,286583451 | 0,147779145 | |
| 1556134_a_at | B3GNT5 /// LOC1 | -2,138804305 | -2,286583451 | 0,147779145 | |
| 1556472_s_at | SCML4 | -2,138804305 | -2,286583451 | 0,147779145 | |
| 1556996_at | - | -2,138804305 | -2,286583451 | 0,147779145 | |
| 1560525_at | - | -2,138804305 | -2,286583451 | 0,147779145 | |
| 1562162_at | - | -2,138804305 | -2,286583451 | 0,147779145 | |
| 1562630_at | - | -2,138804305 | -2,286583451 | 0,147779145 | |
| 1564122_at | LINC00514 /// LC | -2,138804305 | -2,286583451 | 0,147779145 | |
| 1566658_at | LOC100507654 | -2,138804305 | -2,286583451 | 0,147779145 | |
| 204529_s_at | TOX | -2,138804305 | -2,286583451 | 0,147779145 | |
| 207034_s_at | GLI2 | -2,138804305 | -2,286583451 | 0,147779145 | |
| 207569_at | ROS1 | -2,138804305 | -2,286583451 | 0,147779145 | |
| 208053_at | GUCY2F | -2,138804305 | -2,286583451 | 0,147779145 | |
| 210523_at | BMPR1B | -2,138804305 | -2,286583451 | 0,147779145 | |
| 213479_at | NPTX2 | -2,138804305 | -2,286583451 | 0,147779145 | |
| 216334_s_at | CYP2A7P1 | -2,138804305 | -2,286583451 | 0,147779145 | |
| 217481_x_at | - | -2,138804305 | -2,286583451 | 0,147779145 | |
| 227400_at | NFIX | -2,138804305 | -2,286583451 | 0,147779145 | |
| 229960_at | MAP3K6 | -2,138804305 | -2,286583451 | 0,147779145 | |
| 231998_at | SART1 | -2,138804305 | -2,286583451 | 0,147779145 | |
| 232327_at | THSD7B | -2,138804305 | -2,286583451 | 0,147779145 | |
| 233553_at | - | -2,138804305 | -2,286583451 | 0,147779145 | |
| 237057_at | - | -2,138804305 | -2,286583451 | 0,147779145 | |
| 238282_at | - | -2,138804305 | -2,286583451 | 0,147779145 | |
| 238426_at | TMEM130 | -2,138804305 | -2,286583451 | 0,147779145 | |
| 240182_at | - | -2,138804305 | -2,286583451 | 0,147779145 | |
| 240636_at | - | -2,138804305 | -2,286583451 | 0,147779145 | |
| 241917_at | - | -2,138804305 | -2,286583451 | 0,147779145 | |
| 242122_at | - | -2,138804305 | -2,286583451 | 0,147779145 | |
| 243375_at | - | -2,138804305 | -2,286583451 | 0,147779145 | |

| | | | | | |
|---|---|---|---|---|---|
| 244301_at | - | -2,138804305 | -2,286583451 | 0,147779145 | |
| 225254_at | CCDC97 | 0,123193849 | -0,024572586 | 0,147766436 | |
| 243624_at | PIAS2 | 0,123193849 | -0,024572586 | 0,147766436 | |
| 203635_at | DSCR3 | 3,123915073 | 2,976208448 | 0,147706625 | |
| 202616_s_at | MECP2 | 1,709647566 | 1,562009954 | 0,147637611 | |
| 227431_at | RSBN1L-AS1 | 1,709647566 | 1,562009954 | 0,147637611 | |
| 230009_at | FAM118B | 1,709647566 | 1,562009954 | 0,147637611 | |
| 201323_at | EBNA1BP2 | 4,734795999 | 4,58718675 | 0,147609248 | |
| 243444_at | SRD5A3 | 0,627515113 | 0,479907041 | 0,147608072 | |
| 227369_at | SERBP1 | 4,047898031 | 3,900294538 | 0,147603493 | |
| 212396_s_at | EMC1 | 3,170152481 | 3,022611001 | 0,14754148 | |
| 226692_at | C15orf63 /// MIR1 | 1,348276312 | 1,200746069 | 0,147530242 | |
| 229500_at | SLC30A9 | 1,348276312 | 1,200746069 | 0,147530242 | |
| 1564459_at | LOC100129427 | -1,639579028 | -1,787089803 | 0,147510775 | |
| 1569022_a_at | PIK3C2A | -1,639579028 | -1,787089803 | 0,147510775 | |
| 1569841_x_at | - | -1,639579028 | -1,787089803 | 0,147510775 | |
| 1570383_at | BMP1 | -1,639579028 | -1,787089803 | 0,147510775 | |
| 208360_s_at | ERVH-4 | -1,639579028 | -1,787089803 | 0,147510775 | |
| 209097_s_at | JAG1 | -1,639579028 | -1,787089803 | 0,147510775 | |
| 211876_x_at | PCDHGA10 /// PC | -1,639579028 | -1,787089803 | 0,147510775 | |
| 219465_at | APOA2 | -1,639579028 | -1,787089803 | 0,147510775 | |
| 227884_at | TAF15 | -1,639579028 | -1,787089803 | 0,147510775 | |
| 229221_at | CD44 | -1,639579028 | -1,787089803 | 0,147510775 | |
| 234820_at | MAS1L | -1,639579028 | -1,787089803 | 0,147510775 | |
| 241163_at | - | -1,639579028 | -1,787089803 | 0,147510775 | |
| 242058_at | - | -1,639579028 | -1,787089803 | 0,147510775 | |
| 205845_at | CACNA1H | 0,541261317 | 0,393761504 | 0,147499812 | |
| 219264_s_at | PPP2R3B | 0,541261317 | 0,393761504 | 0,147499812 | |
| 208777_s_at | PSMD11 | 5,15258632 | 5,005113369 | 0,14747295 | |
| 210794_s_at | MEG3 | 0,934330293 | 0,786897721 | 0,147432571 | |
| 1556909_at | LOC100505853 | -0,136301282 | -0,283667828 | 0,147366545 | |
| 1569696_at | LOC402779 | -0,136301282 | -0,283667828 | 0,147366545 | |
| 205332_at | RCE1 | -0,136301282 | -0,283667828 | 0,147366545 | |
| 216800_at | - | -0,136301282 | -0,283667828 | 0,147366545 | |
| 236713_at | - | -0,136301282 | -0,283667828 | 0,147366545 | |
| 203065_s_at | CAV1 | 5,208169782 | 5,060810624 | 0,147359158 | |
| 205435_s_at | AAK1 | -0,467091869 | -0,614371577 | 0,147279708 | |
| 208544_at | ADRA2B | -0,467091869 | -0,614371577 | 0,147279708 | |
| 227679_at | HDAC11 | -0,467091869 | -0,614371577 | 0,147279708 | |
| 231570_at | FAM154A | -0,467091869 | -0,614371577 | 0,147279708 | |
| 233069_at | PPP4R1L | -0,467091869 | -0,614371577 | 0,147279708 | |
| 238699_s_at | CASK | -0,467091869 | -0,614371577 | 0,147279708 | |
| 241351_at | - | -0,467091869 | -0,614371577 | 0,147279708 | |
| 212255_s_at | ATP2C1 | 2,566371329 | 2,419116683 | 0,147254646 | |
| 203683_s_at | VEGFB | 0,351548291 | 0,204310755 | 0,147237536 | |
| 209691_s_at | DOK4 | 0,351548291 | 0,204310755 | 0,147237536 | |
| 236609_at | PMS1 | 0,351548291 | 0,204310755 | 0,147237536 | |
| 205583_s_at | ALG13 | 2,159061673 | 2,011838132 | 0,147223541 | |
| 1552849_at | C2orf65 | -2,187914861 | -2,335104118 | 0,147189257 | |
| 1557627_at | LOC283745 | -2,187914861 | -2,335104118 | 0,147189257 | |
| 1557639_at | NFIA | -2,187914861 | -2,335104118 | 0,147189257 | |
| 1557704_a_at | 07.03.15 | -2,187914861 | -2,335104118 | 0,147189257 | |
| 1558497_a_at | LOC100505609 | -2,187914861 | -2,335104118 | 0,147189257 | |
| 1559034_at | SIRPB2 | -2,187914861 | -2,335104118 | 0,147189257 | |
| 1560029_a_at | C11orf57 | -2,187914861 | -2,335104118 | 0,147189257 | |
| 1565358_at | RARA | -2,187914861 | -2,335104118 | 0,147189257 | |

| | | | | | |
|---|---|---|---|---|---|
| 1568633_a_at | - | -2,187914861 | -2,335104118 | 0,147189257 | |
| 1570202_a_at | MKL2 | -2,187914861 | -2,335104118 | 0,147189257 | |
| 206377_at | FOXF2 | -2,187914861 | -2,335104118 | 0,147189257 | |
| 211556_at | MAPRE2 | -2,187914861 | -2,335104118 | 0,147189257 | |
| 216513_at | DCT | -2,187914861 | -2,335104118 | 0,147189257 | |
| 216826_at | - | -2,187914861 | -2,335104118 | 0,147189257 | |
| 219947_at | CLEC4A | -2,187914861 | -2,335104118 | 0,147189257 | |
| 229403_at | B4GALT1 | -2,187914861 | -2,335104118 | 0,147189257 | |
| 233516_s_at | SPAG17 | -2,187914861 | -2,335104118 | 0,147189257 | |
| 233947_s_at | TBX5-AS1 | -2,187914861 | -2,335104118 | 0,147189257 | |
| 235379_at | LOC100506114 | -2,187914861 | -2,335104118 | 0,147189257 | |
| 237771_s_at | - | -2,187914861 | -2,335104118 | 0,147189257 | |
| 239510_at | - | -2,187914861 | -2,335104118 | 0,147189257 | |
| 241025_at | UTP6 | -2,187914861 | -2,335104118 | 0,147189257 | |
| 242660_at | C10orf112 | -2,187914861 | -2,335104118 | 0,147189257 | |
| 242852_at | LOC285147 | -2,187914861 | -2,335104118 | 0,147189257 | |
| 243510_at | LOC100506418 | -2,187914861 | -2,335104118 | 0,147189257 | |
| 218705_s_at | SNX24 | 0,715481152 | 0,568365095 | 0,147116056 | |
| 238574_at | SLC25A51 | 0,715481152 | 0,568365095 | 0,147116056 | |
| 243475_at | CBL | 0,715481152 | 0,568365095 | 0,147116056 | |
| 219633_at | TTPAL | 2,886560922 | 2,739547836 | 0,147013086 | |
| 211752_s_at | NDUFS7 | 3,886725813 | 3,739727424 | 0,14699839 | |
| 212135_s_at | ATP2B4 | 2,696781084 | 2,549796788 | 0,146984296 | |
| 225910_at | HELZ | 2,132266459 | 1,98530622 | 0,146960239 | |
| 202228_s_at | NPTN | 5,918559225 | 5,771649566 | 0,146909659 | |
| 218446_s_at | FAM18B1 | 3,234554895 | 3,087661492 | 0,146893403 | |
| 212415_at | 38961 | 0,87091093 | 0,724100169 | 0,146810761 | |
| 232063_x_at | FARSB | 0,87091093 | 0,724100169 | 0,146810761 | |
| 1559915_at | - | -1,591296941 | -1,738067356 | 0,146770414 | |
| 1560002_at | FAM27A | -1,591296941 | -1,738067356 | 0,146770414 | |
| 1561487_at | - | -1,591296941 | -1,738067356 | 0,146770414 | |
| 1561853_a_at | IL23R | -1,591296941 | -1,738067356 | 0,146770414 | |
| 1563587_at | CDH4 | -1,591296941 | -1,738067356 | 0,146770414 | |
| 1568717_a_at | FKBP15 | -1,591296941 | -1,738067356 | 0,146770414 | |
| 1569283_at | ZNF891 | -1,591296941 | -1,738067356 | 0,146770414 | |
| 1570592_a_at | - | -1,591296941 | -1,738067356 | 0,146770414 | |
| 221160_s_at | CABP5 | -1,591296941 | -1,738067356 | 0,146770414 | |
| 221329_at | OR52A1 | -1,591296941 | -1,738067356 | 0,146770414 | |
| 232334_at | NXPH2 | -1,591296941 | -1,738067356 | 0,146770414 | |
| 233432_at | LINC00598 | -1,591296941 | -1,738067356 | 0,146770414 | |
| 234263_at | - | -1,591296941 | -1,738067356 | 0,146770414 | |
| 204205_at | APOBEC3G | 2,549899391 | 2,403170156 | 0,146729235 | |
| 222487_s_at | RPS27L | 3,408067074 | 3,261354437 | 0,146712637 | |
| 218120_s_at | HMOX2 | 2,433006244 | 2,286318242 | 0,146688003 | |
| 211160_x_at | ACTN1 | 3,769898242 | 3,623242814 | 0,146655428 | |
| 225585_at | RAP2A | 3,952256707 | 3,805658807 | 0,1465979 | |
| 200970_s_at | SERP1 | 4,265835914 | 4,119256941 | 0,146578972 | |
| 212527_at | DESI1 | 1,596577845 | 1,450051551 | 0,146526294 | |
| 1555522_s_at | DPY30 /// MEMO | 3,137452663 | 2,990953566 | 0,146499097 | |
| 232420_x_at | LOC100289341 | 1,874520207 | 1,728030156 | 0,14649005 | |
| 208885_at | LCP1 | 4,232392431 | 4,085930792 | 0,146461639 | |
| 203511_s_at | TRAPPC3 | 3,495722611 | 3,349287209 | 0,146435402 | |
| 204178_s_at | RBM14 | 3,308224834 | 3,161800893 | 0,146423941 | |
| 213243_at | VPS13B | 2,979690938 | 2,833274877 | 0,146416061 | |
| 220183_s_at | NUDT6 | 1,360921491 | 1,214517879 | 0,146403612 | |
| 223329_x_at | SUGT1 | 4,99030312 | 4,843901726 | 0,146401395 | |

| | | | | | |
|---|---|---|---|---|---|
| 240419_at | SLC6A15 | 0,641402394 | 0,495031851 | 0,146370544 | |
| 203161_s_at | RNF8 | -0,67289328 | -0,819238336 | 0,146345056 | |
| 206010_at | HABP2 | -0,67289328 | -0,819238336 | 0,146345056 | |
| 234688_x_at | CNTROB | -0,67289328 | -0,819238336 | 0,146345056 | |
| 235496_at | HRCT1 | -0,67289328 | -0,819238336 | 0,146345056 | |
| 235835_at | - | -0,67289328 | -0,819238336 | 0,146345056 | |
| 236321_at | FAM200B | -0,67289328 | -0,819238336 | 0,146345056 | |
| 236869_at | - | -0,67289328 | -0,819238336 | 0,146345056 | |
| 237323_at | HKDC1 | -0,67289328 | -0,819238336 | 0,146345056 | |
| 212579_at | SMCHD1 | 3,296204798 | 3,149904996 | 0,146299803 | |
| 219923_at | TRIM45 | 0,255492549 | 0,109221392 | 0,146271157 | |
| 222391_at | TMEM30A | 4,109766258 | 3,963505394 | 0,146260864 | |
| 219317_at | POLI | 1,079110292 | 0,932882274 | 0,146228018 | |
| 223325_at | TXNDC11 | 1,079110292 | 0,932882274 | 0,146228018 | |
| 225781_at | MAPK9 | 1,726031811 | 1,579853228 | 0,146178582 | |
| 231984_at | MTAP | 2,860082603 | 2,713921281 | 0,146161322 | |
| 208945_s_at | BECN1 | 2,983784569 | 2,837730973 | 0,146053596 | |
| 205462_s_at | HPCAL1 | 2,535098095 | 2,389070873 | 0,146027222 | |
| 219537_x_at | DLL3 | 1,880387527 | 1,73441697 | 0,145970557 | |
| 1561691_at | LINC00326 | -2,262747984 | -2,408705138 | 0,145957153 | |
| 1562866_at | - | -2,262747984 | -2,408705138 | 0,145957153 | |
| 1566339_at | SNORD8 | -2,262747984 | -2,408705138 | 0,145957153 | |
| 211741_x_at | PSG3 | -2,262747984 | -2,408705138 | 0,145957153 | |
| 213824_at | OLIG2 | -2,262747984 | -2,408705138 | 0,145957153 | |
| 216120_s_at | ATP2B2 | -2,262747984 | -2,408705138 | 0,145957153 | |
| 217360_x_at | IGHA1 /// IGHG1 | -2,262747984 | -2,408705138 | 0,145957153 | |
| 219524_s_at | NDUFAF5 | -2,262747984 | -2,408705138 | 0,145957153 | |
| 219645_at | CASQ1 | -2,262747984 | -2,408705138 | 0,145957153 | |
| 219928_s_at | CABYR | -2,262747984 | -2,408705138 | 0,145957153 | |
| 224343_x_at | YME1L1 | -2,262747984 | -2,408705138 | 0,145957153 | |
| 226755_at | MIR205HG | -2,262747984 | -2,408705138 | 0,145957153 | |
| 232477_at | - | -2,262747984 | -2,408705138 | 0,145957153 | |
| 236971_at | - | -2,262747984 | -2,408705138 | 0,145957153 | |
| 237086_at | FOXA1 | -2,262747984 | -2,408705138 | 0,145957153 | |
| 238217_at | - | -2,262747984 | -2,408705138 | 0,145957153 | |
| 239977_at | C12orf42 | -2,262747984 | -2,408705138 | 0,145957153 | |
| 242084_at | LOC339316 | -2,262747984 | -2,408705138 | 0,145957153 | |
| 242709_s_at | - | -2,262747984 | -2,408705138 | 0,145957153 | |
| 242906_at | SESN3 | -2,262747984 | -2,408705138 | 0,145957153 | |
| 244655_at | LOC100507311 | -2,262747984 | -2,408705138 | 0,145957153 | |
| 224615_x_at | HM13 | 2,466546243 | 2,320681318 | 0,145864925 | |
| 218898_at | FAM57A | 2,952099138 | 2,806245339 | 0,145853799 | |
| 237215_s_at | TFRC | 3,604290432 | 3,458446747 | 0,145843685 | |
| 214039_s_at | LAPTM4B | 6,781073846 | 6,63524734 | 0,145826506 | |
| 218437_s_at | LZTFL1 | 1,689737652 | 1,543943227 | 0,145794424 | |
| 228613_at | RAB11FIP3 | 1,261018186 | 1,115234684 | 0,145783501 | |
| 211612_s_at | IL13RA1 | 0,882651021 | 0,73688026 | 0,14577076 | |
| 203858_s_at | COX10 | 2,491678455 | 2,345926937 | 0,145751519 | |
| 223270_at | CTDSPL2 | 2,261526912 | 2,115788273 | 0,145738639 | |
| 228662_at | SOCS7 | 2,261526912 | 2,115788273 | 0,145738639 | |
| 212307_s_at | OGT | 2,901063789 | 2,755336312 | 0,145727478 | |
| 209675_s_at | HNRNPUL1 | 1,987559146 | 1,841838775 | 0,145720372 | |
| 205035_at | CTDP1 | 1,369290453 | 1,223626587 | 0,145663865 | |
| 227150_at | MTF1 | 1,369290453 | 1,223626587 | 0,145663865 | |
| 232981_s_at | SYNRG | 1,021868205 | 0,876248481 | 0,145619724 | |
| 223153_x_at | TMUB1 | 2,470441349 | 2,324919719 | 0,14552163 | |

| | | | | | |
|---|---|---|---|---|---|
| 228159_at | - | 2,713311265 | 2,567790814 | 0,145520451 | |
| 204691_x_at | PLA2G6 | 2,236584753 | 2,091116676 | 0,145468077 | |
| 202370_s_at | CBFB | 4,61981709 | 4,474382017 | 0,145435073 | |
| 1555735_a_at | BAP1 | 0,735041319 | 0,589657387 | 0,145383932 | |
| 202817_s_at | SS18 | 2,652894659 | 2,507538456 | 0,145356203 | |
| 1556400_at | - | -1,518072576 | -1,663414495 | 0,145341919 | |
| 1561460_at | - | -1,518072576 | -1,663414495 | 0,145341919 | |
| 1566868_at | - | -1,518072576 | -1,663414495 | 0,145341919 | |
| 205306_x_at | KMO | -1,518072576 | -1,663414495 | 0,145341919 | |
| 206831_s_at | ARSD | -1,518072576 | -1,663414495 | 0,145341919 | |
| 207101_at | VAMP1 | -1,518072576 | -1,663414495 | 0,145341919 | |
| 208607_s_at | SAA1 /// SAA2 /// | -1,518072576 | -1,663414495 | 0,145341919 | |
| 211171_s_at | PDE10A | -1,518072576 | -1,663414495 | 0,145341919 | |
| 213472_at | HNRNPH1 | -1,518072576 | -1,663414495 | 0,145341919 | |
| 213904_at | - | -1,518072576 | -1,663414495 | 0,145341919 | |
| 214865_at | DOT1L | -1,518072576 | -1,663414495 | 0,145341919 | |
| 223816_at | SLC46A2 | -1,518072576 | -1,663414495 | 0,145341919 | |
| 233054_at | CNOT2 | -1,518072576 | -1,663414495 | 0,145341919 | |
| 233314_at | - | -1,518072576 | -1,663414495 | 0,145341919 | |
| 236617_at | - | -1,518072576 | -1,663414495 | 0,145341919 | |
| 237223_at | - | -1,518072576 | -1,663414495 | 0,145341919 | |
| 1552432_at | MFSD6L | -0,112808516 | -0,258114234 | 0,145305717 | |
| 1556123_a_at | RAB11B-AS1 | -0,112808516 | -0,258114234 | 0,145305717 | |
| 210510_s_at | NRP1 | -0,112808516 | -0,258114234 | 0,145305717 | |
| 217644_s_at | SOS2 | -0,112808516 | -0,258114234 | 0,145305717 | |
| 228154_at | C19orf44 | -0,112808516 | -0,258114234 | 0,145305717 | |
| 232763_at | TLN1 | -0,112808516 | -0,258114234 | 0,145305717 | |
| 236482_at | - | -0,112808516 | -0,258114234 | 0,145305717 | |
| 238158_at | MEIG1 | -0,112808516 | -0,258114234 | 0,145305717 | |
| 223065_s_at | STARD3NL | 5,448784246 | 5,303490367 | 0,145293879 | |
| 205268_s_at | ADD2 | 1,210676994 | 1,065442793 | 0,145234201 | |
| 1554943_at | VIL1 | 0,152582607 | 0,007421914 | 0,145160693 | |
| 202700_s_at | TMEM63A | 0,152582607 | 0,007421914 | 0,145160693 | |
| 209237_s_at | SLC23A2 | 0,152582607 | 0,007421914 | 0,145160693 | |
| 231934_at | TRIM14 | 0,152582607 | 0,007421914 | 0,145160693 | |
| 232463_at | PPP2R3B-AS1 | 0,152582607 | 0,007421914 | 0,145160693 | |
| 226715_at | FOXK1 | 2,522021211 | 2,376875125 | 0,145146086 | |
| 222669_s_at | SBDS /// SBDSP1 | 4,628558674 | 4,483415907 | 0,145142766 | |
| 1556639_at | LOC439914 | -2,311268652 | -2,456406426 | 0,145137775 | |
| 1556913_a_at | GIT2 | -2,311268652 | -2,456406426 | 0,145137775 | |
| 1557672_s_at | LOC100133308 | -2,311268652 | -2,456406426 | 0,145137775 | |
| 1559848_at | NSUN4 | -2,311268652 | -2,456406426 | 0,145137775 | |
| 1560020_at | DNAJC13 | -2,311268652 | -2,456406426 | 0,145137775 | |
| 1570122_at | - | -2,311268652 | -2,456406426 | 0,145137775 | |
| 203397_s_at | GALNT3 | -2,311268652 | -2,456406426 | 0,145137775 | |
| 207497_s_at | MS4A2 | -2,311268652 | -2,456406426 | 0,145137775 | |
| 208214_at | ADRB1 | -2,311268652 | -2,456406426 | 0,145137775 | |
| 217280_x_at | GABRA5 | -2,311268652 | -2,456406426 | 0,145137775 | |
| 219738_s_at | PCDH9 | -2,311268652 | -2,456406426 | 0,145137775 | |
| 220211_at | FLJ13224 | -2,311268652 | -2,456406426 | 0,145137775 | |
| 229516_at | WDR31 | -2,311268652 | -2,456406426 | 0,145137775 | |
| 231614_at | NOVA1 | -2,311268652 | -2,456406426 | 0,145137775 | |
| 232654_s_at | UGT1A6 | -2,311268652 | -2,456406426 | 0,145137775 | |
| 234527_at | - | -2,311268652 | -2,456406426 | 0,145137775 | |
| 235362_at | LOC729970 | -2,311268652 | -2,456406426 | 0,145137775 | |
| 236659_x_at | - | -2,311268652 | -2,456406426 | 0,145137775 | |

| | | | | | |
|---|---|---|---|---|---|
| 239620_at | TTC23L | -2,311268652 | -2,456406426 | 0,145137775 | |
| 243728_at | LOC100505750 | -2,311268652 | -2,456406426 | 0,145137775 | |
| 244620_at | SLC8A1-AS1 | -2,311268652 | -2,456406426 | 0,145137775 | |
| 202187_s_at | PPP2R5A | 2,908260915 | 2,763166224 | 0,145094691 | |
| 219008_at | C2orf43 | 1,926486359 | 1,781437557 | 0,145048802 | |
| 222585_x_at | KRCC1 | 0,962260738 | 0,817300337 | 0,144960401 | |
| 1553952_at | ZDHHC19 | 1,094335495 | 0,949447784 | 0,144887711 | |
| 1556047_s_at | MAGEE1 | 1,094335495 | 0,949447784 | 0,144887711 | |
| 208748_s_at | FLOT1 | 1,094335495 | 0,949447784 | 0,144887711 | |
| 221194_s_at | RNFT1 | 1,894952274 | 1,750261389 | 0,144690885 | |
| 226069_at | PRICKLE1 | 2,573632254 | 2,428994491 | 0,144637763 | |
| 1555202_a_at | RPRD1A | 1,274448124 | 1,129843209 | 0,144604915 | |
| 238076_at | GATAD2B | 1,381753566 | 1,23718272 | 0,144570847 | |
| 1555306_a_at | ECE2 | 0,661985843 | 0,51742585 | 0,144559992 | |
| 205966_at | TAF13 | 0,661985843 | 0,51742585 | 0,144559992 | |
| 215260_s_at | TCF3 | 0,661985843 | 0,51742585 | 0,144559992 | |
| 238108_at | - | 0,661985843 | 0,51742585 | 0,144559992 | |
| 221925_s_at | CSPP1 | 1,481627361 | 1,337086025 | 0,144541336 | |
| 205672_at | XPA | 1,575032557 | 1,43051703 | 0,144515526 | |
| 201067_at | PSMC2 | 2,190100188 | 2,045615873 | 0,144484315 | |
| 204601_at | N4BP1 | 2,190100188 | 2,045615873 | 0,144484315 | |
| 225400_at | TSEN15 | 2,303618506 | 2,159161028 | 0,144457478 | |
| 225463_x_at | GPR89A /// GPR8 | 3,541801785 | 3,39737216 | 0,144429625 | |
| 201849_at | BNIP3 | 4,87068766 | 4,726262343 | 0,144425317 | |
| 219477_s_at | MRPS31P3 /// TH | 1,706348269 | 1,562009954 | 0,144338315 | |
| 1552903_at | B4GALNT2 | -1,470833215 | -1,615132408 | 0,144299192 | |
| 1556327_a_at | - | -1,470833215 | -1,615132408 | 0,144299192 | |
| 1561714_a_at | B3GALNT2 | -1,470833215 | -1,615132408 | 0,144299192 | |
| 1563119_at | HP09025 | -1,470833215 | -1,615132408 | 0,144299192 | |
| 1569676_at | TOR1AIP2 | -1,470833215 | -1,615132408 | 0,144299192 | |
| 227842_at | RAB30 | -1,470833215 | -1,615132408 | 0,144299192 | |
| 227899_at | VIT | -1,470833215 | -1,615132408 | 0,144299192 | |
| 228921_at | SLC25A42 | -1,470833215 | -1,615132408 | 0,144299192 | |
| 231739_at | LOC100507547 / | -1,470833215 | -1,615132408 | 0,144299192 | |
| 233804_at | LOC440131 | -1,470833215 | -1,615132408 | 0,144299192 | |
| 233863_at | CASZ1 | -1,470833215 | -1,615132408 | 0,144299192 | |
| 234051_at | - | -1,470833215 | -1,615132408 | 0,144299192 | |
| 237389_at | - | -1,470833215 | -1,615132408 | 0,144299192 | |
| 240942_at | MPHOSPH8 | -1,470833215 | -1,615132408 | 0,144299192 | |
| 241196_at | - | -1,470833215 | -1,615132408 | 0,144299192 | |
| 243167_at | ABCB5 | -1,470833215 | -1,615132408 | 0,144299192 | |
| 244424_at | LOC439938 | -1,470833215 | -1,615132408 | 0,144299192 | |
| 211675_s_at | MDFIC | 3,372081975 | 3,227795991 | 0,144285984 | |
| 219086_at | ZNF839 | 0,57782909 | 0,433557298 | 0,144271792 | |
| 209716_at | CSF1 | 0,747935635 | 0,603679647 | 0,144255989 | |
| 203718_at | PNPLA6 | 0,900083947 | 0,755840599 | 0,144243348 | |
| 1552701_a_at | CARD16 | -2,359436556 | -2,503677622 | 0,144241066 | |
| 1554643_at | RGS11 | -2,359436556 | -2,503677622 | 0,144241066 | |
| 205386_s_at | MDM2 | -2,359436556 | -2,503677622 | 0,144241066 | |
| 210426_x_at | RORA | -2,359436556 | -2,503677622 | 0,144241066 | |
| 216786_at | FAM224A | -2,359436556 | -2,503677622 | 0,144241066 | |
| 228850_s_at | SLIT2 | -2,359436556 | -2,503677622 | 0,144241066 | |
| 230372_at | HAS2 | -2,359436556 | -2,503677622 | 0,144241066 | |
| 232260_at | LOC100009676 | -2,359436556 | -2,503677622 | 0,144241066 | |
| 234098_at | SOBP | -2,359436556 | -2,503677622 | 0,144241066 | |
| 237734_s_at | LOC100506907 | -2,359436556 | -2,503677622 | 0,144241066 | |

| | | | | | |
|---|---|---|---|---|---|
| 240693_at | - | -2,359436556 | -2,503677622 | 0,144241066 | |
| 243711_at | DDAH1 | -2,359436556 | -2,503677622 | 0,144241066 | |
| 1554794_a_at | UBE3C | 1,037706275 | 0,893473268 | 0,144233007 | |
| 219284_at | HSPBAP1 | 1,037706275 | 0,893473268 | 0,144233007 | |
| 212269_s_at | MCM3AP | 5,299405017 | 5,155174723 | 0,144230294 | |
| 209123_at | QDPR | 4,600841383 | 4,456625687 | 0,144215696 | |
| 227521_at | FBXO33 | 3,164152611 | 3,019999252 | 0,144153359 | |
| 213083_at | SLC35D2 | 2,72965419 | 2,585563171 | 0,144091019 | |
| 218290_at | PLEKHJ1 | 2,72965419 | 2,585563171 | 0,144091019 | |
| 215307_at | ZNF529 | 3,709382903 | 3,565308644 | 0,144074259 | |
| 203097_s_at | RAPGEF2 | 4,354920338 | 4,210848982 | 0,144071357 | |
| 203156_at | AKAP11 | 2,938102794 | 2,794067719 | 0,144035075 | |
| 225767_at | RN45S | 3,523171367 | 3,379145507 | 0,14402586 | |
| 235117_at | CHAC2 | 3,523171367 | 3,379145507 | 0,14402586 | |
| 236240_at | C4orf29 | 1,224581536 | 1,080561626 | 0,14401991 | |
| 229664_at | MAPK8 | 1,104397092 | 0,960386719 | 0,144010373 | |
| 201498_at | USP7 | 4,181024227 | 4,037037849 | 0,143986378 | |
| 232814_x_at | APOPT1 | 3,166555554 | 3,022611001 | 0,143944554 | |
| 238122_at | RBM12B | 1,751865623 | 1,60795124 | 0,143914383 | |
| 225047_at | NUPL1 | 2,626954721 | 2,483094137 | 0,143860585 | |
| 202958_at | PTPN9 | 1,390002893 | 1,246149888 | 0,143853005 | |
| 209600_s_at | ACOX1 | 2,536956583 | 2,39311332 | 0,143843264 | |
| 1568286_at | HMGA2 | -3,41365796 | -3,557342128 | 0,143684168 | |
| 200598_s_at | HSP90B1 /// MIR | 4,9941977 | 4,850531814 | 0,143665886 | |
| 225673_at | MYADM | 6,102518102 | 5,958855849 | 0,143662253 | |
| 1557667_at | - | -0,423248115 | -0,566887459 | 0,143639344 | |
| 1560553_at | TIAF1 | -0,423248115 | -0,566887459 | 0,143639344 | |
| 224008_s_at | KCNK7 | -0,423248115 | -0,566887459 | 0,143639344 | |
| 227206_at | NDUFA10 | -0,423248115 | -0,566887459 | 0,143639344 | |
| 239499_at | DNAH2 | -0,423248115 | -0,566887459 | 0,143639344 | |
| 240310_at | - | -0,423248115 | -0,566887459 | 0,143639344 | |
| 242987_x_at | LOC100506553 | -0,423248115 | -0,566887459 | 0,143639344 | |
| 201699_at | PSMC6 | 5,562860257 | 5,419261348 | 0,143598909 | |
| 1570026_at | CPXM2 | 0,171847695 | 0,028364014 | 0,143483681 | |
| 217035_at | SKAP2 | 0,171847695 | 0,028364014 | 0,143483681 | |
| 221701_s_at | STRA6 | 0,171847695 | 0,028364014 | 0,143483681 | |
| 230202_at | RELA | 0,171847695 | 0,028364014 | 0,143483681 | |
| 237518_at | - | 0,171847695 | 0,028364014 | 0,143483681 | |
| 242739_at | - | 0,171847695 | 0,028364014 | 0,143483681 | |
| 244622_at | BRWD1 | 0,171847695 | 0,028364014 | 0,143483681 | |
| 213528_at | METTL18 | 1,90937145 | 1,765933685 | 0,143437765 | |
| 202095_s_at | BIRC5 | 4,451686997 | 4,308254202 | 0,143432795 | |
| 203602_s_at | ZBTB17 | 1,542099044 | 1,398700978 | 0,143398066 | |
| 212117_at | RHOQ | 3,032022986 | 2,888726553 | 0,143296432 | |
| 218995_s_at | EDN1 | 0,496120222 | 0,352836757 | 0,143283465 | |
| 243022_at | - | 0,496120222 | 0,352836757 | 0,143283465 | |
| 206323_x_at | OPHN1 | 4,750498305 | 4,607335387 | 0,143162918 | |
| 230875_s_at | ATP11A | 3,176127502 | 3,033010968 | 0,143116534 | |
| 222551_s_at | C8orf33 | 3,359534664 | 3,216434104 | 0,14310056 | |
| 203418_at | CCNA2 | 4,198742362 | 4,05564712 | 0,143095242 | |
| 214182_at | - | 3,191547726 | 3,048471686 | 0,14307604 | |
| 201334_s_at | ARHGEF12 | 4,726676749 | 4,583653734 | 0,143023015 | |
| 224899_s_at | MAGT1 | 4,674447146 | 4,531428237 | 0,143018909 | |
| 203261_at | DCTN6 | 4,767615283 | 4,624630085 | 0,142985199 | |
| 218090_s_at | WDR11 | 3,052955227 | 2,910041809 | 0,142913418 | |
| 202467_s_at | COPS2 | 5,735044187 | 5,592190191 | 0,142853996 | |

| | | | | | |
|---|---|---|---|---|---|
| 228590_at | PTCD3 | 2,571820446 | 2,428994491 | 0,142825955 | |
| 218555_at | ANAPC2 | 1,40228911 | 1,259496994 | 0,142792115 | |
| 225986_x_at | CPSF2 | 3,102740237 | 2,959949103 | 0,142791134 | |
| 209876_at | GIT2 | 1,119359127 | 0,976641161 | 0,142717966 | |
| 216316_x_at | - | 1,119359127 | 0,976641161 | 0,142717966 | |
| 202308_at | SREBF1 | 1,296557217 | 1,153866761 | 0,142690456 | |
| 213468_at | ERCC2 | 1,296557217 | 1,153866761 | 0,142690456 | |
| 223090_x_at | VEZT | 3,088871381 | 2,946258252 | 0,142613129 | |
| 202330_s_at | UNG | 4,112884557 | 3,970285709 | 0,142598847 | |
| 1557274_at | LOC100506494 | -0,622910612 | -0,765504029 | 0,142593417 | |
| 205093_at | PLEKHA6 | -0,622910612 | -0,765504029 | 0,142593417 | |
| 211197_s_at | ICOSLG | -0,622910612 | -0,765504029 | 0,142593417 | |
| 233135_at | LOC100506527 | -0,622910612 | -0,765504029 | 0,142593417 | |
| 237646_x_at | PLEKHG5 | -0,622910612 | -0,765504029 | 0,142593417 | |
| 204479_at | OSTF1 | 2,325288363 | 2,182705233 | 0,14258313 | |
| 212731_at | ANKRD46 | 3,467741468 | 3,325159096 | 0,142582372 | |
| 1553140_at | PELO | -2,43257096 | -2,575129729 | 0,142558769 | |
| 1554766_s_at | - | -2,43257096 | -2,575129729 | 0,142558769 | |
| 1555220_a_at | AKR1E2 | -2,43257096 | -2,575129729 | 0,142558769 | |
| 1556323_at | CELF2 | -2,43257096 | -2,575129729 | 0,142558769 | |
| 1559413_at | TCP11L2 | -2,43257096 | -2,575129729 | 0,142558769 | |
| 1560246_at | - | -2,43257096 | -2,575129729 | 0,142558769 | |
| 1561577_at | - | -2,43257096 | -2,575129729 | 0,142558769 | |
| 1564022_at | ZNF804B | -2,43257096 | -2,575129729 | 0,142558769 | |
| 1564676_a_at | - | -2,43257096 | -2,575129729 | 0,142558769 | |
| 1565242_at | TNXB | -2,43257096 | -2,575129729 | 0,142558769 | |
| 1568921_at | - | -2,43257096 | -2,575129729 | 0,142558769 | |
| 1570474_s_at | LOC338579 | -2,43257096 | -2,575129729 | 0,142558769 | |
| 201842_s_at | EFEMP1 | -2,43257096 | -2,575129729 | 0,142558769 | |
| 206425_s_at | TRPC3 | -2,43257096 | -2,575129729 | 0,142558769 | |
| 210016_at | MYT1L | -2,43257096 | -2,575129729 | 0,142558769 | |
| 210479_s_at | RORA | -2,43257096 | -2,575129729 | 0,142558769 | |
| 210712_at | LDHAL6B | -2,43257096 | -2,575129729 | 0,142558769 | |
| 213537_at | HLA-DPA1 | -2,43257096 | -2,575129729 | 0,142558769 | |
| 215422_at | - | -2,43257096 | -2,575129729 | 0,142558769 | |
| 217093_at | RNASE1 | -2,43257096 | -2,575129729 | 0,142558769 | |
| 220030_at | STYK1 | -2,43257096 | -2,575129729 | 0,142558769 | |
| 221363_x_at | GPR25 | -2,43257096 | -2,575129729 | 0,142558769 | |
| 223643_at | CRYGS | -2,43257096 | -2,575129729 | 0,142558769 | |
| 224426_s_at | ACTR3BP2 /// AC | -2,43257096 | -2,575129729 | 0,142558769 | |
| 231242_at | BHLHE41 | -2,43257096 | -2,575129729 | 0,142558769 | |
| 231668_x_at | IGHG1 | -2,43257096 | -2,575129729 | 0,142558769 | |
| 232791_at | - | -2,43257096 | -2,575129729 | 0,142558769 | |
| 234245_at | - | -2,43257096 | -2,575129729 | 0,142558769 | |
| 237676_at | - | -2,43257096 | -2,575129729 | 0,142558769 | |
| 237980_at | LINC00347 | -2,43257096 | -2,575129729 | 0,142558769 | |
| 238313_at | - | -2,43257096 | -2,575129729 | 0,142558769 | |
| 238576_at | MOCOS | -2,43257096 | -2,575129729 | 0,142558769 | |
| 241279_at | - | -2,43257096 | -2,575129729 | 0,142558769 | |
| 243729_at | - | -2,43257096 | -2,575129729 | 0,142558769 | |
| 243876_at | - | -2,43257096 | -2,575129729 | 0,142558769 | |
| 212675_s_at | CEP68 | 2,768138014 | 2,625627364 | 0,14251065 | |
| 212734_x_at | RPL13 /// SNORD | 7,509180604 | 7,366697803 | 0,142482801 | |
| 214435_x_at | RALA | 3,920575295 | 3,778101865 | 0,14247343 | |
| 224913_s_at | TIMM50 | 5,881739849 | 5,739266787 | 0,142473062 | |
| 214221_at | ALMS1 | 2,122398022 | 1,979940779 | 0,142457244 | |

| | | | | | |
|---|---|---|---|---|---|
| 1555286_at | CDKL1 | -1,399512875 | -1,541908042 | 0,142395167 | |
| 1570084_at | - | -1,399512875 | -1,541908042 | 0,142395167 | |
| 1570252_at | - | -1,399512875 | -1,541908042 | 0,142395167 | |
| 208341_x_at | CSH2 | -1,399512875 | -1,541908042 | 0,142395167 | |
| 209569_x_at | D4S234E | -1,399512875 | -1,541908042 | 0,142395167 | |
| 220425_x_at | ROPN1B | -1,399512875 | -1,541908042 | 0,142395167 | |
| 220532_s_at | TMEM176B | -1,399512875 | -1,541908042 | 0,142395167 | |
| 228102_at | - | -1,399512875 | -1,541908042 | 0,142395167 | |
| 228206_at | HS3ST4 | -1,399512875 | -1,541908042 | 0,142395167 | |
| 229046_s_at | PLEKHB1 | -1,399512875 | -1,541908042 | 0,142395167 | |
| 230828_at | GRAMD2 | -1,399512875 | -1,541908042 | 0,142395167 | |
| 232244_at | KIAA1161 | -1,399512875 | -1,541908042 | 0,142395167 | |
| 232704_s_at | LRRFIP2 | -1,399512875 | -1,541908042 | 0,142395167 | |
| 236928_at | - | -1,399512875 | -1,541908042 | 0,142395167 | |
| 238243_at | - | -1,399512875 | -1,541908042 | 0,142395167 | |
| 240944_at | - | -1,399512875 | -1,541908042 | 0,142395167 | |
| 243292_at | FAM132A | -1,399512875 | -1,541908042 | 0,142395167 | |
| 244049_at | - | -1,399512875 | -1,541908042 | 0,142395167 | |
| 244319_at | - | -1,399512875 | -1,541908042 | 0,142395167 | |
| 1555897_at | - | -0,078265071 | -0,220592223 | 0,142327152 | |
| 227055_at | METTL7B | -0,078265071 | -0,220592223 | 0,142327152 | |
| 233795_at | ODF3 | -0,078265071 | -0,220592223 | 0,142327152 | |
| 237109_at | TOM1L2 | -0,078265071 | -0,220592223 | 0,142327152 | |
| 241978_at | - | -0,078265071 | -0,220592223 | 0,142327152 | |
| 242545_at | TTLL11 | -0,078265071 | -0,220592223 | 0,142327152 | |
| 222550_at | ARMC1 | 4,810884483 | 4,668649411 | 0,142235072 | |
| 43934_at | GPR137 | 1,156099112 | 1,013870809 | 0,142228303 | |
| 200751_s_at | HNRNPC /// LOC | 6,528153377 | 6,386081529 | 0,142071848 | |
| 214895_s_at | ADAM10 | 1,247462054 | 1,105412836 | 0,142049218 | |
| 200867_at | RNF114 | 3,372081975 | 3,23005767 | 0,142024305 | |
| 224598_at | MGAT4B | 5,055446416 | 4,913450933 | 0,141995483 | |
| 1555106_a_at | CTDSPL2 | 1,063722698 | 0,921731975 | 0,141990722 | |
| 204633_s_at | RPS6KA5 | 1,512180019 | 1,370274469 | 0,14190555 | |
| 213150_at | HOXA10 | 1,129248302 | 0,987376598 | 0,141871704 | |
| 205452_at | PIGB | 2,220484971 | 2,078620836 | 0,141864135 | |
| 202561_at | TNKS | 2,438982012 | 2,297144887 | 0,141837125 | |
| 227151_at | SNX33 | 1,560488174 | 1,418668082 | 0,141820092 | |
| 205251_at | PER2 | 2,361396186 | 2,21959457 | 0,141801616 | |
| 217294_s_at | ENO1 | 6,854913809 | 6,71315288 | 0,141760929 | |
| 204730_at | RIMS3 | 1,414471577 | 1,272721751 | 0,141749827 | |
| 228988_at | ZNF711 | 4,177454337 | 4,035745559 | 0,141708778 | |
| 221932_s_at | GLRX5 | 6,224151084 | 6,082442605 | 0,141708479 | |
| 202173_s_at | VEZF1 | 0,695652136 | 0,553993624 | 0,141658512 | |
| 213561_at | MCM9 | 0,695652136 | 0,553993624 | 0,141658512 | |
| 224448_s_at | MNF1 | 4,571901478 | 4,430312399 | 0,14158908 | |
| 217965_s_at | SAP30BP | 3,01878441 | 2,877228323 | 0,141556087 | |
| 226538_at | MAN2A1 | 3,563845287 | 3,42231092 | 0,141534367 | |
| 209530_at | CACNB3 | 0,779676066 | 0,638150376 | 0,141525689 | |
| 1558474_at | - | -2,479842156 | -2,621331989 | 0,141489833 | |
| 1559259_at | - | -2,479842156 | -2,621331989 | 0,141489833 | |
| 1559450_at | - | -2,479842156 | -2,621331989 | 0,141489833 | |
| 1560317_s_at | HIP1 | -2,479842156 | -2,621331989 | 0,141489833 | |
| 1561877_at | - | -2,479842156 | -2,621331989 | 0,141489833 | |
| 1562275_at | - | -2,479842156 | -2,621331989 | 0,141489833 | |
| 1562591_a_at | OFCC1 | -2,479842156 | -2,621331989 | 0,141489833 | |
| 1564970_at | SETDB2 | -2,479842156 | -2,621331989 | 0,141489833 | |

| | | | | | |
|---|---|---|---|---|---|
| 1568612_at | GABRG2 | -2,479842156 | -2,621331989 | 0,141489833 | |
| 1569257_at | FMNL1 | -2,479842156 | -2,621331989 | 0,141489833 | |
| 205816_at | ITGB8 | -2,479842156 | -2,621331989 | 0,141489833 | |
| 206239_s_at | SPINK1 | -2,479842156 | -2,621331989 | 0,141489833 | |
| 216558_x_at | IGHA1 /// IGHD // | -2,479842156 | -2,621331989 | 0,141489833 | |
| 216989_at | SPAM1 | -2,479842156 | -2,621331989 | 0,141489833 | |
| 220880_at | - | -2,479842156 | -2,621331989 | 0,141489833 | |
| 221089_at | NOX3 | -2,479842156 | -2,621331989 | 0,141489833 | |
| 225540_at | MAP2 | -2,479842156 | -2,621331989 | 0,141489833 | |
| 233621_s_at | ARHGEF12 | -2,479842156 | -2,621331989 | 0,141489833 | |
| 234281_at | ESPN /// ESPNP | -2,479842156 | -2,621331989 | 0,141489833 | |
| 234629_at | - | -2,479842156 | -2,621331989 | 0,141489833 | |
| 236439_at | - | -2,479842156 | -2,621331989 | 0,141489833 | |
| 239539_at | - | -2,479842156 | -2,621331989 | 0,141489833 | |
| 240850_at | - | -2,479842156 | -2,621331989 | 0,141489833 | |
| 242309_at | - | -2,479842156 | -2,621331989 | 0,141489833 | |
| 243483_at | TRPM8 | -2,479842156 | -2,621331989 | 0,141489833 | |
| 244761_at | C5orf63 | -2,479842156 | -2,621331989 | 0,141489833 | |
| 201930_at | MCM6 | 5,55003372 | 5,408567805 | 0,141465915 | |
| 217955_at | BCL2L13 | 1,968405881 | 1,826973888 | 0,141431993 | |
| 1553113_s_at | CDK8 | 3,431234921 | 3,289820089 | 0,141414832 | |
| 228099_at | ZNF550 | 2,166283906 | 2,024923328 | 0,141360578 | |
| 1565254_s_at | ELL | -0,394749123 | -0,536087151 | 0,141338027 | |
| 207982_at | HIST1H1T | -0,394749123 | -0,536087151 | 0,141338027 | |
| 217212_s_at | IL9R | -0,394749123 | -0,536087151 | 0,141338027 | |
| 222595_s_at | DIDO1 | -0,394749123 | -0,536087151 | 0,141338027 | |
| 228814_at | RBBP6 | -0,394749123 | -0,536087151 | 0,141338027 | |
| 230293_at | - | -0,394749123 | -0,536087151 | 0,141338027 | |
| 232735_at | ANKRD34A | -0,394749123 | -0,536087151 | 0,141338027 | |
| 237659_at | FLJ40288 | -0,394749123 | -0,536087151 | 0,141338027 | |
| 202585_s_at | NFX1 | 0,518867317 | 0,377530647 | 0,14133667 | |
| 212205_at | H2AFV | 5,515811234 | 5,374726101 | 0,141085133 | |
| 1554386_at | CST9 | -1,353583928 | -1,494668682 | 0,141084754 | |
| 1556938_a_at | - | -1,353583928 | -1,494668682 | 0,141084754 | |
| 1559680_at | TTL | -1,353583928 | -1,494668682 | 0,141084754 | |
| 1560378_at | GRIK1-AS2 | -1,353583928 | -1,494668682 | 0,141084754 | |
| 1569188_s_at | - | -1,353583928 | -1,494668682 | 0,141084754 | |
| 206636_at | RASA2 | -1,353583928 | -1,494668682 | 0,141084754 | |
| 208425_s_at | TANC2 | -1,353583928 | -1,494668682 | 0,141084754 | |
| 211633_x_at | - | -1,353583928 | -1,494668682 | 0,141084754 | |
| 215550_at | SRGAP3 | -1,353583928 | -1,494668682 | 0,141084754 | |
| 222434_at | ENAH | -1,353583928 | -1,494668682 | 0,141084754 | |
| 222802_at | EDN1 | -1,353583928 | -1,494668682 | 0,141084754 | |
| 223232_s_at | CGN | -1,353583928 | -1,494668682 | 0,141084754 | |
| 224487_at | - | -1,353583928 | -1,494668682 | 0,141084754 | |
| 226086_at | SYT13 | -1,353583928 | -1,494668682 | 0,141084754 | |
| 231111_at | - | -1,353583928 | -1,494668682 | 0,141084754 | |
| 231510_at | - | -1,353583928 | -1,494668682 | 0,141084754 | |
| 235631_at | DDR2 | -1,353583928 | -1,494668682 | 0,141084754 | |
| 236315_at | - | -1,353583928 | -1,494668682 | 0,141084754 | |
| 237601_at | LOC100507277 | -1,353583928 | -1,494668682 | 0,141084754 | |
| 240518_at | LOC729291 | -1,353583928 | -1,494668682 | 0,141084754 | |
| 243245_at | LOC100506446 | -1,353583928 | -1,494668682 | 0,141084754 | |
| 218128_at | NFYB | 1,659347917 | 1,518263577 | 0,14108434 | |
| 230411_at | UBE2V2 | 1,422536444 | 1,281471383 | 0,141065061 | |
| 1553990_at | C16orf79 | 0,20027154 | 0,059218869 | 0,141052671 | |

| | | | | | |
|---|---|---|---|---|---|
| 238948_at | TM9SF1 | 0,20027154 | 0,059218869 | 0,141052671 | |
| 203028_s_at | CYBA | 5,672171472 | 5,531136224 | 0,141035248 | |
| 211758_x_at | TXNDC9 | 4,163687338 | 4,022758595 | 0,140928743 | |
| 223525_at | DLL4 | 0,317354302 | 0,176436073 | 0,140918229 | |
| 227883_at | CCDC71L | 0,317354302 | 0,176436073 | 0,140918229 | |
| 200050_at | ZNF146 | 6,681991566 | 6,541101365 | 0,140890202 | |
| 222402_at | POMP | 3,232262672 | 3,091398163 | 0,140864509 | |
| 221011_s_at | LBH | 3,930506493 | 3,789647959 | 0,140858534 | |
| 219522_at | FJX1 | 2,846658948 | 2,705818724 | 0,140840224 | |
| 33132_at | CPSF1 /// MIR12 | 3,908064381 | 3,767241497 | 0,140822883 | |
| 205548_s_at | BTG3 | 3,319065909 | 3,178292062 | 0,140773847 | |
| 203269_at | NSMAF | 5,281225703 | 5,140521187 | 0,140704516 | |
| 229766_at | ZNF445 | 1,789769151 | 1,64909809 | 0,140671061 | |
| 218228_s_at | TNKS2 | 4,453164021 | 4,312529215 | 0,140634806 | |
| 203847_s_at | AKAP8 | 2,708372067 | 2,567790814 | 0,140581253 | |
| 228370_at | LOC100506948 / | 1,666156729 | 1,525647381 | 0,140509348 | |
| 1552865_a_at | IFLTD1 | -0,055687482 | -0,19610998 | 0,140422498 | |
| 237293_at | - | -0,055687482 | -0,19610998 | 0,140422498 | |
| 208835_s_at | LUC7L3 | 4,138815532 | 3,9984201 | 0,140395432 | |
| 212957_s_at | LOC92249 | 0,945567441 | 0,805216064 | 0,140351377 | |
| 219392_x_at | PRR11 | 4,281994342 | 4,141655035 | 0,140339307 | |
| 211105_s_at | NFATC1 | 1,530951994 | 1,390636111 | 0,140315883 | |
| 224941_at | PAPPA | 1,530951994 | 1,390636111 | 0,140315883 | |
| 213828_x_at | H3F3A /// H3F3A | 7,213893795 | 7,073593152 | 0,140300643 | |
| 1561329_s_at | RPUSD3 | -0,59053611 | -0,730778808 | 0,140242698 | |
| 209603_at | GATA3 | -0,59053611 | -0,730778808 | 0,140242698 | |
| 211230_s_at | PIK3CD | -0,59053611 | -0,730778808 | 0,140242698 | |
| 215999_at | CDRT1 | -0,59053611 | -0,730778808 | 0,140242698 | |
| 232290_at | - | -0,59053611 | -0,730778808 | 0,140242698 | |
| 235582_at | E2F2 | -0,59053611 | -0,730778808 | 0,140242698 | |
| 238602_at | DIS3L2 | -0,59053611 | -0,730778808 | 0,140242698 | |
| 239335_at | ZNF710 | -0,59053611 | -0,730778808 | 0,140242698 | |
| 241187_at | - | -0,59053611 | -0,730778808 | 0,140242698 | |
| 1568678_s_at | FGFR1OP | 3,824937847 | 3,684723168 | 0,140214679 | |
| 201655_s_at | HSPG2 | 3,284083774 | 3,14392006 | 0,140163714 | |
| 1552800_at | ABHD11 | 1,269985355 | 1,129843209 | 0,140142146 | |
| 201584_s_at | DDX39A | 6,735572926 | 6,595453115 | 0,140119811 | |
| 210054_at | HAUS3 | 2,796341835 | 2,656223838 | 0,140117997 | |
| 202294_at | STAG1 | 1,874520207 | 1,73441697 | 0,140103236 | |
| 221813_at | FBXO42 | 1,874520207 | 1,73441697 | 0,140103236 | |
| 218402_s_at | HPS4 | 2,2992452 | 2,159161028 | 0,140084173 | |
| 217560_at | GGA1 | 0,533835206 | 0,393761504 | 0,140073702 | |
| 239013_at | SEC22C | 0,533835206 | 0,393761504 | 0,140073702 | |
| 213407_at | PHLPP2 | 1,434549838 | 1,294497133 | 0,140052705 | |
| 209005_at | FBXL5 | 0,798390451 | 0,658444279 | 0,139946172 | |
| 233779_x_at | - | 0,798390451 | 0,658444279 | 0,139946172 | |
| 221890_at | ZNF335 | 1,799091453 | 1,659204017 | 0,139887437 | |
| 223307_at | CDCA3 | 3,769107843 | 3,629253073 | 0,13985477 | |
| 229845_at | MAPKAP1 | 1,951783949 | 1,811954245 | 0,139829704 | |
| 222610_s_at | S100PBP | 1,153678675 | 1,013870809 | 0,139807866 | |
| 209143_s_at | CLNS1A | 5,256342713 | 5,116578126 | 0,139764586 | |
| 205437_at | ZNF211 | 3,18563636 | 3,045906378 | 0,139729981 | |
| 223231_at | TATDN1 | 4,482873466 | 4,343149918 | 0,139723548 | |
| 202930_s_at | SUCLA2 | 4,633995388 | 4,494276519 | 0,139718868 | |
| 201456_s_at | BUB3 | 3,69780007 | 3,558125564 | 0,139674506 | |
| 1561143_at | - | -2,551294263 | -2,690860744 | 0,139566481 | |

| | | | | | |
|---|---|---|---|---|---|
| 1561261_at | - | -2,551294263 | -2,690860744 | 0,139566481 | |
| 1562386_s_at | ZNF501 | -2,551294263 | -2,690860744 | 0,139566481 | |
| 1565804_at | - | -2,551294263 | -2,690860744 | 0,139566481 | |
| 1565895_at | - | -2,551294263 | -2,690860744 | 0,139566481 | |
| 1565976_at | - | -2,551294263 | -2,690860744 | 0,139566481 | |
| 1570101_at | - | -2,551294263 | -2,690860744 | 0,139566481 | |
| 1570316_at | - | -2,551294263 | -2,690860744 | 0,139566481 | |
| 1570484_at | - | -2,551294263 | -2,690860744 | 0,139566481 | |
| 206535_at | SLC2A2 | -2,551294263 | -2,690860744 | 0,139566481 | |
| 223869_at | SOST | -2,551294263 | -2,690860744 | 0,139566481 | |
| 228377_at | KLHL14 | -2,551294263 | -2,690860744 | 0,139566481 | |
| 232202_at | - | -2,551294263 | -2,690860744 | 0,139566481 | |
| 233379_at | PRR5L | -2,551294263 | -2,690860744 | 0,139566481 | |
| 235335_at | ABCA9 | -2,551294263 | -2,690860744 | 0,139566481 | |
| 240489_at | - | -2,551294263 | -2,690860744 | 0,139566481 | |
| 242901_at | - | -2,551294263 | -2,690860744 | 0,139566481 | |
| 244188_at | - | -2,551294263 | -2,690860744 | 0,139566481 | |
| 212343_at | YIPF6 | 2,305800198 | 2,166264721 | 0,139535476 | |
| 229234_at | ZC3H12B | 0,95671774 | 0,817300337 | 0,139417403 | |
| 219703_at | MNS1 | 1,805273023 | 1,665902186 | 0,139370838 | |
| 227295_at | IKBIP | 1,883312263 | 1,743944487 | 0,139367776 | |
| 222118_at | CENPN | 2,061726373 | 1,922365006 | 0,139361367 | |
| 222690_s_at | TMEM39A | 2,061726373 | 1,922365006 | 0,139361367 | |
| 202961_s_at | ATP5J2 | 6,76153603 | 6,622229752 | 0,139306278 | |
| 209448_at | HTATIP2 | 2,365585359 | 2,226407528 | 0,139177831 | |
| 225655_at | UHRF1 | 5,038819589 | 4,899664665 | 0,139154924 | |
| 200092_s_at | RPL37 | 7,924324991 | 7,785179709 | 0,139145282 | |
| 216323_x_at | TUBA3C /// TUBA | 1,496984567 | 1,3579181 | 0,139066467 | |
| 235711_at | PURB | 1,496984567 | 1,3579181 | 0,139066467 | |
| 209771_x_at | CD24 | 1,283332461 | 1,144305291 | 0,13902717 | |
| 209284_s_at | FAM208A | 2,790121796 | 2,651169275 | 0,13895252 | |
| 214716_at | BMP2K | 2,132266459 | 1,993317152 | 0,138949307 | |
| 203487_s_at | ARMC8 | 2,640847372 | 2,501934108 | 0,138913264 | |
| 208749_x_at | FLOT1 | 3,663333942 | 3,524430539 | 0,138903403 | |
| 204531_s_at | BRCA1 | 3,285189903 | 3,146317015 | 0,138872889 | |
| 1553395_a_at | CD200R1 | -1,2845686 | -1,423348341 | 0,138779741 | |
| 1564475_s_at | ZBED3-AS1 | -1,2845686 | -1,423348341 | 0,138779741 | |
| 1565671_a_at | - | -1,2845686 | -1,423348341 | 0,138779741 | |
| 1565935_at | C4orf21 | -1,2845686 | -1,423348341 | 0,138779741 | |
| 1570239_a_at | CLNK | -1,2845686 | -1,423348341 | 0,138779741 | |
| 209401_s_at | SLC12A4 | -1,2845686 | -1,423348341 | 0,138779741 | |
| 211148_s_at | ANGPT2 | -1,2845686 | -1,423348341 | 0,138779741 | |
| 215591_at | SATB2 | -1,2845686 | -1,423348341 | 0,138779741 | |
| 216055_at | PDGFB | -1,2845686 | -1,423348341 | 0,138779741 | |
| 224273_at | C3orf20 | -1,2845686 | -1,423348341 | 0,138779741 | |
| 234186_at | - | -1,2845686 | -1,423348341 | 0,138779741 | |
| 238119_at | - | -1,2845686 | -1,423348341 | 0,138779741 | |
| 241801_at | PGAP1 | -1,2845686 | -1,423348341 | 0,138779741 | |
| 242514_at | - | -1,2845686 | -1,423348341 | 0,138779741 | |
| 244241_x_at | - | -1,2845686 | -1,423348341 | 0,138779741 | |
| 221919_at | HNRNPA1 | 2,197169074 | 2,058399602 | 0,138769473 | |
| 226980_at | DEPDC1B | 3,751608593 | 3,6128809 | 0,138727693 | |
| 212684_at | ZNF3 | 2,069451339 | 1,930732138 | 0,138719201 | |
| 230588_s_at | LOC285074 | 2,501229299 | 2,362515214 | 0,138714086 | |
| 217771_at | GOLM1 | 3,069741844 | 2,93104672 | 0,138695125 | |
| 200958_s_at | SDCBP | 5,939084132 | 5,800470711 | 0,138613421 | |

| | | | | | |
|---|---|---|---|---|---|
| 221783_at | WIZ | 1,168140757 | 1,029536893 | 0,138603864 | |
| 204160_s_at | ENPP4 | 3,199783157 | 3,061230246 | 0,138552911 | |
| 45828_at | ATP5SL | 3,913081773 | 3,77461994 | 0,138461833 | |
| 224015_s_at | MRPS25 | 1,454352496 | 1,315948728 | 0,138403768 | |
| 220127_s_at | FBXL12 | 3,401961598 | 3,263564154 | 0,138397444 | |
| 218087_s_at | SORBS1 | 1,139070151 | 1,000684521 | 0,13838563 | |
| 221869_at | ZNF512B | 1,139070151 | 1,000684521 | 0,13838563 | |
| 1553471_at | SLC35G3 | -2,597496523 | -2,735862426 | 0,138365903 | |
| 1554560_at | PGM5 | -2,597496523 | -2,735862426 | 0,138365903 | |
| 1555240_s_at | GNG12 | -2,597496523 | -2,735862426 | 0,138365903 | |
| 1556541_s_at | - | -2,597496523 | -2,735862426 | 0,138365903 | |
| 1560373_a_at | - | -2,597496523 | -2,735862426 | 0,138365903 | |
| 1561700_at | - | -2,597496523 | -2,735862426 | 0,138365903 | |
| 1563277_at | - | -2,597496523 | -2,735862426 | 0,138365903 | |
| 208481_at | ASB4 | -2,597496523 | -2,735862426 | 0,138365903 | |
| 216669_at | - | -2,597496523 | -2,735862426 | 0,138365903 | |
| 219823_at | LIN28A | -2,597496523 | -2,735862426 | 0,138365903 | |
| 232721_at | TRIM55 | -2,597496523 | -2,735862426 | 0,138365903 | |
| 234227_at | - | -2,597496523 | -2,735862426 | 0,138365903 | |
| 237961_at | - | -2,597496523 | -2,735862426 | 0,138365903 | |
| 239460_at | LOC100652954 | -2,597496523 | -2,735862426 | 0,138365903 | |
| 240866_at | - | -2,597496523 | -2,735862426 | 0,138365903 | |
| 241589_at | - | -2,597496523 | -2,735862426 | 0,138365903 | |
| 243325_at | - | -2,597496523 | -2,735862426 | 0,138365903 | |
| 244582_at | - | -2,597496523 | -2,735862426 | 0,138365903 | |
| 224636_at | ZFP91 | 4,065387235 | 3,927027853 | 0,138359382 | |
| 217900_at | IARS2 | 4,771961618 | 4,63362748 | 0,138334138 | |
| 1560599_a_at | CEP89 | 1,735773781 | 1,59747851 | 0,138295271 | |
| 221669_s_at | ACAD8 | 1,735773781 | 1,59747851 | 0,138295271 | |
| 223671_x_at | DPH5 | 3,767525744 | 3,629253073 | 0,138272671 | |
| 201139_s_at | SSB | 5,16074523 | 5,022506227 | 0,138239003 | |
| 1556900_at | LOC149773 | 0,555999837 | 0,417770464 | 0,138229373 | |
| 218821_at | NPEPL1 /// STX1 | 0,555999837 | 0,417770464 | 0,138229373 | |
| 209781_s_at | KHDRBS3 | 2,580856819 | 2,442710777 | 0,138146041 | |
| 202956_at | ARFGEF1 | 3,205636958 | 3,067567462 | 0,138069496 | |
| 1555780_a_at | RHEB | 5,13582221 | 4,997831652 | 0,137990558 | |
| 1559922_at | - | -0,352951799 | -0,490927335 | 0,137975537 | |
| 220584_at | FLJ22184 | -0,352951799 | -0,490927335 | 0,137975537 | |
| 221375_at | OR1G1 | -0,352951799 | -0,490927335 | 0,137975537 | |
| 225757_s_at | CLMN | -0,352951799 | -0,490927335 | 0,137975537 | |
| 227529_s_at | AKAP12 | -0,352951799 | -0,490927335 | 0,137975537 | |
| 230582_at | HECA | -0,352951799 | -0,490927335 | 0,137975537 | |
| 239821_at | FLJ30064 | -0,352951799 | -0,490927335 | 0,137975537 | |
| 223402_at | DUSP23 | 4,128375743 | 3,990437532 | 0,137938212 | |
| 209221_s_at | OSBPL2 | 1,177702228 | 1,039887232 | 0,137814996 | |
| 227701_at | C10orf118 | 1,177702228 | 1,039887232 | 0,137814996 | |
| 203921_at | CHST2 | 2,536956583 | 2,39915583 | 0,137800753 | |
| 213080_x_at | RPL5 /// SNORD: | 7,861057199 | 7,72328437 | 0,13777283 | |
| 220083_x_at | UCHL5 | 2,327437554 | 2,189694216 | 0,137743338 | |
| 217935_s_at | UQCC | 2,959046717 | 2,821324225 | 0,137722492 | |
| 203192_at | ABCB6 | -0,022466681 | -0,160136748 | 0,137670068 | |
| 235584_at | LOC285033 | -0,022466681 | -0,160136748 | 0,137670068 | |
| 212607_at | AKT3 | 2,082235068 | 1,944570415 | 0,137664653 | |
| 200709_at | FKBP1A | 4,156452062 | 4,018839599 | 0,137612463 | |
| 218095_s_at | TMEM165 | 3,895444023 | 3,757867094 | 0,137576929 | |
| 227558_at | CBX4 | 2,905386372 | 2,767843855 | 0,137542517 | |

| | | | | | |
|---|---|---|---|---|---|
| 206833_s_at | ACYP2 | 2,272723257 | 2,135226207 | 0,13749705 | |
| 221575_at | SCLY | 2,38635068 | 2,248887791 | 0,137462889 | |
| 206809_s_at | HNRNPA3 /// HN | 3,934741882 | 3,797294336 | 0,137447546 | |
| 225522_at | AAK1 | 2,182996494 | 2,045615873 | 0,137380621 | |
| 239193_at | FUBP3 | 0,359971644 | 0,222599095 | 0,137372549 | |
| 235429_at | EIF3E | 1,567778694 | 1,43051703 | 0,137261664 | |
| 1552546_a_at | LETM2 | -1,240169874 | -1,377419394 | 0,13724952 | |
| 1552955_at | LINC00208 | -1,240169874 | -1,377419394 | 0,13724952 | |
| 1558995_at | ZNF547 | -1,240169874 | -1,377419394 | 0,13724952 | |
| 204803_s_at | RRAD | -1,240169874 | -1,377419394 | 0,13724952 | |
| 210637_at | TACR1 | -1,240169874 | -1,377419394 | 0,13724952 | |
| 211130_x_at | EDA | -1,240169874 | -1,377419394 | 0,13724952 | |
| 213948_x_at | CADM3 | -1,240169874 | -1,377419394 | 0,13724952 | |
| 215736_at | KCNV1 | -1,240169874 | -1,377419394 | 0,13724952 | |
| 216077_s_at | L3MBTL1 | -1,240169874 | -1,377419394 | 0,13724952 | |
| 229023_at | SFT2D3 | -1,240169874 | -1,377419394 | 0,13724952 | |
| 232829_at | OR52K3P | -1,240169874 | -1,377419394 | 0,13724952 | |
| 234754_at | SLC37A1 | -1,240169874 | -1,377419394 | 0,13724952 | |
| 236717_at | FAM179A | -1,240169874 | -1,377419394 | 0,13724952 | |
| 236767_at | IQCF2 | -1,240169874 | -1,377419394 | 0,13724952 | |
| 241149_at | - | -1,240169874 | -1,377419394 | 0,13724952 | |
| 243489_at | - | -1,240169874 | -1,377419394 | 0,13724952 | |
| 243741_at | - | -1,240169874 | -1,377419394 | 0,13724952 | |
| 244171_at | MKLN1 | -1,240169874 | -1,377419394 | 0,13724952 | |
| 204125_at | NDUFAF1 | 3,638448528 | 3,501209806 | 0,137238722 | |
| 222508_s_at | ARGLU1 | 1,305306849 | 1,16809119 | 0,137215659 | |
| 228309_at | - | 1,305306849 | 1,16809119 | 0,137215659 | |
| 214491_at | SSTR3 | 0,246434561 | 0,109221392 | 0,137213169 | |
| 241812_at | SPATS2L | 0,246434561 | 0,109221392 | 0,137213169 | |
| 228977_at | LOC729680 | 0,984222186 | 0,847075464 | 0,137146722 | |
| 1552939_at | ANGPT1 | -2,64318869 | -2,780313924 | 0,137125235 | |
| 1553296_at | GPR128 | -2,64318869 | -2,780313924 | 0,137125235 | |
| 1555869_a_at | LOC100507477 | -2,64318869 | -2,780313924 | 0,137125235 | |
| 1556623_at | - | -2,64318869 | -2,780313924 | 0,137125235 | |
| 1557018_a_at | - | -2,64318869 | -2,780313924 | 0,137125235 | |
| 1557136_at | ATP13A4 | -2,64318869 | -2,780313924 | 0,137125235 | |
| 1561507_at | FFAR1 | -2,64318869 | -2,780313924 | 0,137125235 | |
| 1561584_at | - | -2,64318869 | -2,780313924 | 0,137125235 | |
| 1564430_at | - | -2,64318869 | -2,780313924 | 0,137125235 | |
| 1568638_a_at | IDO2 | -2,64318869 | -2,780313924 | 0,137125235 | |
| 1569140_at | UBR2 | -2,64318869 | -2,780313924 | 0,137125235 | |
| 1569582_at | LOC201651 | -2,64318869 | -2,780313924 | 0,137125235 | |
| 205041_s_at | ORM1 /// ORM2 | -2,64318869 | -2,780313924 | 0,137125235 | |
| 211338_at | IFNA2 | -2,64318869 | -2,780313924 | 0,137125235 | |
| 211436_at | - | -2,64318869 | -2,780313924 | 0,137125235 | |
| 213316_at | KIAA1462 | -2,64318869 | -2,780313924 | 0,137125235 | |
| 215824_at | - | -2,64318869 | -2,780313924 | 0,137125235 | |
| 217583_at | PAH | -2,64318869 | -2,780313924 | 0,137125235 | |
| 234480_at | DKFZP761C1711 | -2,64318869 | -2,780313924 | 0,137125235 | |
| 237328_at | C14orf105 | -2,64318869 | -2,780313924 | 0,137125235 | |
| 241758_at | - | -2,64318869 | -2,780313924 | 0,137125235 | |
| 243106_at | CLEC12A | -2,64318869 | -2,780313924 | 0,137125235 | |
| 243347_at | - | -2,64318869 | -2,780313924 | 0,137125235 | |
| 244097_at | CR2 | -2,64318869 | -2,780313924 | 0,137125235 | |
| 214801_at | TOR1AIP2 | 3,321224383 | 3,184136378 | 0,137088005 | |
| 1556464_a_at | C2orf72 | 0,57058931 | 0,433557298 | 0,137032012 | |

| | | | | | |
|---|---|---|---|---|---|
| 225954_s_at | MIDN | 3,004080279 | 2,867091644 | 0,136988635 | |
| 208786_s_at | MAP1LC3B | 3,50998543 | 3,373018423 | 0,136967007 | |
| 225878_at | KIF1B | 2,834621043 | 2,697670403 | 0,13695064 | |
| 227095_at | LEPROT | 4,074372924 | 3,937445604 | 0,13692732 | |
| 227451_s_at | CCDC90A | 2,814842651 | 2,677924589 | 0,136918062 | |
| 219182_at | TMEM231 | 1,795990708 | 1,659204017 | 0,136786691 | |
| 213009_s_at | TRIM37 | 3,394805732 | 3,258033502 | 0,13677223 | |
| 228152_s_at | DDX60L | 2,644299732 | 2,507538456 | 0,136761276 | |
| 215954_s_at | CACTIN | 1,251994932 | 1,115234684 | 0,136760248 | |
| 212725_s_at | TUG1 | 4,571221501 | 4,434486264 | 0,136735237 | |
| 1566701_at | VRK3 | -0,543051993 | -0,679763839 | 0,136711846 | |
| 215449_at | TSPO2 | -0,543051993 | -0,679763839 | 0,136711846 | |
| 222248_s_at | SIRT4 | -0,543051993 | -0,679763839 | 0,136711846 | |
| 228111_s_at | DNAH1 | -0,543051993 | -0,679763839 | 0,136711846 | |
| 230725_at | ANKRD32 | -0,543051993 | -0,679763839 | 0,136711846 | |
| 231280_at | - | -0,543051993 | -0,679763839 | 0,136711846 | |
| 240164_at | - | -0,543051993 | -0,679763839 | 0,136711846 | |
| 222127_s_at | EXOC1 | 3,981211211 | 3,844556416 | 0,136654795 | |
| 236132_at | TLN1 | 1,191926657 | 1,055274826 | 0,136651831 | |
| 214543_x_at | QKI | 3,078700536 | 2,942125533 | 0,136575003 | |
| 223639_s_at | ZNRD1 | 4,362800281 | 4,226225581 | 0,1365747 | |
| 239047_at | FAM122C | 1,314003737 | 1,177496821 | 0,136506916 | |
| 225951_s_at | CHD2 /// LOC100 | 4,411731296 | 4,275233864 | 0,136497432 | |
| 208310_s_at | CCZ1 /// CCZ1B | 3,692807389 | 3,556324192 | 0,136483198 | |
| 203734_at | FOXJ2 | 1,880387527 | 1,743944487 | 0,136443039 | |
| 206430_at | CDX1 | 0,841135803 | 0,704715219 | 0,136420584 | |
| 210765_at | CSE1L | 0,841135803 | 0,704715219 | 0,136420584 | |
| 229194_at | PCGF5 | 2,194816625 | 2,058399602 | 0,136417024 | |
| 212577_at | SMCHD1 | 2,994644467 | 2,858346012 | 0,136298455 | |
| 232612_s_at | ATG16L1 | 1,672933557 | 1,536652708 | 0,136280849 | |
| 242602_x_at | ZNF254 | 1,672933557 | 1,536652708 | 0,136280849 | |
| 221818_at | INTS5 | 1,373456798 | 1,23718272 | 0,136274079 | |
| 218645_at | ZNF277 | 3,833280332 | 3,697038015 | 0,136242317 | |
| 55692_at | ELMO2 | 3,189186085 | 3,052950028 | 0,136236058 | |
| 210849_s_at | VPS41 | 2,782308844 | 2,646096941 | 0,136211903 | |
| 210154_at | ME2 | 2,718233611 | 2,582026171 | 0,136207439 | |
| 229803_s_at | NUDT3 /// RPS10 | 3,182077878 | 3,045906378 | 0,1361715 | |
| 218258_at | POLR1D | 5,197928051 | 5,06176326 | 0,136164791 | |
| 204628_s_at | ITGB3 | 1,534677255 | 1,398700978 | 0,135976277 | |
| 234987_at | SAMHD1 | 2,535098095 | 2,39915583 | 0,135942266 | |
| 209150_s_at | TM9SF1 | 4,291933439 | 4,155999779 | 0,13593366 | |
| 201604_s_at | PPP1R12A | 3,906268218 | 3,770352811 | 0,135915407 | |
| 1554059_at | SETMAR | -0,00073712 | -0,136643983 | 0,135906862 | |
| 228388_at | NFKBIB | -0,00073712 | -0,136643983 | 0,135906862 | |
| 231682_at | - | -0,00073712 | -0,136643983 | 0,135906862 | |
| 234921_at | ZNF470 | -0,00073712 | -0,136643983 | 0,135906862 | |
| 235762_at | TAS2R14 | -0,00073712 | -0,136643983 | 0,135906862 | |
| 215936_s_at | KIAA1033 | -0,325749314 | -0,461594427 | 0,135845112 | |
| 224348_s_at | - | -0,325749314 | -0,461594427 | 0,135845112 | |
| 232720_at | LINGO2 | -0,325749314 | -0,461594427 | 0,135845112 | |
| 238540_at | LOC401320 | -0,325749314 | -0,461594427 | 0,135845112 | |
| 201830_s_at | NET1 | 4,691650895 | 4,555975485 | 0,13567541 | |
| 218030_at | GIT1 | 2,487840343 | 2,352169913 | 0,13567043 | |
| 1553796_at | LOC100652811 / | -1,196375121 | -1,332017329 | 0,135642208 | |
| 1554280_a_at | C9orf43 | -1,196375121 | -1,332017329 | 0,135642208 | |
| 1556255_a_at | LOC400756 | -1,196375121 | -1,332017329 | 0,135642208 | |

| | | | | | |
|---|---|---|---|---|---|
| 1559154_at | - | -1,196375121 | -1,332017329 | 0,135642208 | |
| 1569703_a_at | - | -1,196375121 | -1,332017329 | 0,135642208 | |
| 205736_at | PGAM2 | -1,196375121 | -1,332017329 | 0,135642208 | |
| 207282_s_at | MYOG | -1,196375121 | -1,332017329 | 0,135642208 | |
| 219334_s_at | NABP1 | -1,196375121 | -1,332017329 | 0,135642208 | |
| 219874_at | SLC12A8 | -1,196375121 | -1,332017329 | 0,135642208 | |
| 223596_at | SLC12A6 | -1,196375121 | -1,332017329 | 0,135642208 | |
| 229404_at | TWIST2 | -1,196375121 | -1,332017329 | 0,135642208 | |
| 230675_at | - | -1,196375121 | -1,332017329 | 0,135642208 | |
| 230975_at | - | -1,196375121 | -1,332017329 | 0,135642208 | |
| 232413_at | - | -1,196375121 | -1,332017329 | 0,135642208 | |
| 237525_at | - | -1,196375121 | -1,332017329 | 0,135642208 | |
| 242694_at | IQSEC3 /// LOC1 | -1,196375121 | -1,332017329 | 0,135642208 | |
| 243038_at | RBM43 | -1,196375121 | -1,332017329 | 0,135642208 | |
| 243243_at | GPC3 | -1,196375121 | -1,332017329 | 0,135642208 | |
| 204054_at | PTEN | 1,381753566 | 1,246149888 | 0,135603678 | |
| 1557260_a_at | ZNF382 | 3,220746604 | 3,08516499 | 0,135581614 | |
| 225012_at | HDLBP | 2,355089531 | 2,21959457 | 0,135494961 | |
| 1553607_at | BACH1 /// GRIK1 | -3,212234735 | -3,347710726 | 0,135475991 | |
| 1555928_at | - | -3,212234735 | -3,347710726 | 0,135475991 | |
| 1559612_at | - | -3,212234735 | -3,347710726 | 0,135475991 | |
| 1564631_at | - | -3,212234735 | -3,347710726 | 0,135475991 | |
| 1554809_at | LOC389199 | -0,148196756 | -0,283667828 | 0,135471071 | |
| 1566923_at | - | -0,148196756 | -0,283667828 | 0,135471071 | |
| 211234_x_at | ESR1 | -0,148196756 | -0,283667828 | 0,135471071 | |
| 215235_at | SPTAN1 | -0,148196756 | -0,283667828 | 0,135471071 | |
| 222856_at | APLN | -0,148196756 | -0,283667828 | 0,135471071 | |
| 234795_at | - | -0,148196756 | -0,283667828 | 0,135471071 | |
| 237401_at | ACTN1 | -0,148196756 | -0,283667828 | 0,135471071 | |
| 226265_at | QSER1 | 4,754497159 | 4,619032017 | 0,135465142 | |
| 212004_at | C1orf144 | 2,854131948 | 2,718761062 | 0,135370886 | |
| 203268_s_at | DRG2 | 1,932146506 | 1,796776588 | 0,135369918 | |
| 207535_s_at | NFKB2 | 0,384950573 | 0,249604176 | 0,135346398 | |
| 209420_s_at | SMPD1 | 0,384950573 | 0,249604176 | 0,135346398 | |
| 203100_s_at | CDYL | 3,299492934 | 3,164168347 | 0,135324587 | |
| 228175_at | SLC4A8 | 0,592200562 | 0,456918297 | 0,135282264 | |
| 212113_at | ATXN7L3B | 2,043538949 | 1,908311039 | 0,13522791 | |
| 225692_at | CAMTA1 | 4,784922606 | 4,649767202 | 0,135155404 | |
| 219307_at | PDSS2 | 2,331726354 | 2,196649505 | 0,13507685 | |
| 1552316_a_at | GIMAP1 | -2,712026959 | -2,847001814 | 0,134974855 | |
| 1553823_a_at | RTP1 | -2,712026959 | -2,847001814 | 0,134974855 | |
| 1555991_s_at | C22orf42 | -2,712026959 | -2,847001814 | 0,134974855 | |
| 1557842_at | - | -2,712026959 | -2,847001814 | 0,134974855 | |
| 1560979_a_at | - | -2,712026959 | -2,847001814 | 0,134974855 | |
| 1561247_at | LOC283682 | -2,712026959 | -2,847001814 | 0,134974855 | |
| 1561662_at | - | -2,712026959 | -2,847001814 | 0,134974855 | |
| 1561998_at | - | -2,712026959 | -2,847001814 | 0,134974855 | |
| 1565557_at | - | -2,712026959 | -2,847001814 | 0,134974855 | |
| 1565732_at | - | -2,712026959 | -2,847001814 | 0,134974855 | |
| 1566544_at | - | -2,712026959 | -2,847001814 | 0,134974855 | |
| 1570130_at | SPATS2 | -2,712026959 | -2,847001814 | 0,134974855 | |
| 205259_at | NR3C2 | -2,712026959 | -2,847001814 | 0,134974855 | |
| 215094_at | SPICE1 | -2,712026959 | -2,847001814 | 0,134974855 | |
| 240144_at | DNASE1 | -2,712026959 | -2,847001814 | 0,134974855 | |
| 240657_at | - | -2,712026959 | -2,847001814 | 0,134974855 | |
| 241762_at | FBXO32 | -2,712026959 | -2,847001814 | 0,134974855 | |

| | | | | | |
|---|---|---|---|---|---|
| 242893_at | LOC100506267 | -2,712026959 | -2,847001814 | 0,134974855 | |
| 243863_at | ZCWPW2 | -2,712026959 | -2,847001814 | 0,134974855 | |
| 243121_x_at | - | 0,859074518 | 0,724100169 | 0,134974349 | |
| 232053_x_at | RHBDD2 | 1,689737652 | 1,55481039 | 0,134927261 | |
| 200653_s_at | CALM1 /// CALM | 5,83284143 | 5,697993921 | 0,134847508 | |
| 225099_at | FBXO45 | 3,691139316 | 3,556324192 | 0,134815124 | |
| 205389_s_at | ANK1 | 1,215326747 | 1,080561626 | 0,134765122 | |
| 205807_s_at | TUFT1 | 2,277177578 | 2,14244844 | 0,134729139 | |
| 219443_at | TASP1 | 2,277177578 | 2,14244844 | 0,134729139 | |
| 227208_at | CCDC84 | 2,084778276 | 1,950068772 | 0,134709504 | |
| 229504_at | - | 2,669932407 | 2,53523821 | 0,134694197 | |
| 222794_x_at | MTPAP | 4,051152809 | 3,916534328 | 0,134618481 | |
| 224250_s_at | SECISBP2 | 1,394109935 | 1,259496994 | 0,134612941 | |
| 223374_s_at | B3GALNT1 | 2,051361651 | 1,916759838 | 0,134601814 | |
| 1553717_at | KCTD7 /// RABG | -0,512251684 | -0,646746079 | 0,134494394 | |
| 1566038_at | DGCR7 | -0,512251684 | -0,646746079 | 0,134494394 | |
| 206754_s_at | CYP2B6 /// CYP2 | -0,512251684 | -0,646746079 | 0,134494394 | |
| 208069_x_at | CSH1 /// CSHL1 / | -0,512251684 | -0,646746079 | 0,134494394 | |
| 220353_at | FAM86C1 | -0,512251684 | -0,646746079 | 0,134494394 | |
| 234265_at | PPP1R12C | -0,512251684 | -0,646746079 | 0,134494394 | |
| 238515_at | NUDT16 | -0,512251684 | -0,646746079 | 0,134494394 | |
| 236194_at | - | 2,553576109 | 2,419116683 | 0,134459426 | |
| 37965_at | PARVB | 0,82601458 | 0,691645685 | 0,134368895 | |
| 220234_at | CA8 | 2,696781084 | 2,562416132 | 0,134364952 | |
| 59999_at | HIF1AN | 2,384287546 | 2,250002661 | 0,134284885 | |
| 222909_s_at | BAG4 | 1,742232072 | 1,60795124 | 0,134280832 | |
| 203567_s_at | TRIM38 | 1,946200473 | 1,811954245 | 0,134246228 | |
| 201256_at | COX7A2L | 4,849158785 | 4,715003086 | 0,134155699 | |
| 203833_s_at | TGOLN2 | 0,87091093 | 0,73688026 | 0,13403067 | |
| 211114_x_at | GEMIN2 | 3,655656719 | 3,521663705 | 0,133993015 | |
| 211976_at | - | 3,655656719 | 3,521663705 | 0,133993015 | |
| 208973_at | ERI3 | 2,31665944 | 2,182705233 | 0,133954207 | |
| 213612_x_at | LOC100506032 / | 5,450264243 | 5,316319697 | 0,133944546 | |
| 219449_s_at | TMEM70 | 4,553887643 | 4,419948427 | 0,133939216 | |
| 223101_s_at | ARPC5L | 2,37600538 | 2,242180416 | 0,133824964 | |
| 224427_s_at | PAPOLG | 1,163336153 | 1,029536893 | 0,13379926 | |
| 207749_s_at | PPP2R3A | 0,792179257 | 0,658444279 | 0,133734978 | |
| 237105_at | LOC100506831 | 0,792179257 | 0,658444279 | 0,133734978 | |
| 57532_at | DVL2 | 2,562727117 | 2,428994491 | 0,133732626 | |
| 202784_s_at | NNT | 1,512180019 | 1,378453643 | 0,133726376 | |
| 212167_s_at | SMARCB1 | 2,635653293 | 2,501934108 | 0,133719185 | |
| 224649_x_at | CCNY | 2,635653293 | 2,501934108 | 0,133719185 | |
| 212849_at | AXIN1 | 1,659347917 | 1,525647381 | 0,133700537 | |
| 222548_s_at | MAP4K4 | 1,659347917 | 1,525647381 | 0,133700537 | |
| 221448_s_at | TEX15 | 1,027166904 | 0,893473268 | 0,133693637 | |
| 1556099_at | LINC00290 | -2,756478458 | -2,89013181 | 0,133653352 | |
| 1556655_s_at | - | -2,756478458 | -2,89013181 | 0,133653352 | |
| 1557647_a_at | LOC100507240 | -2,756478458 | -2,89013181 | 0,133653352 | |
| 1560721_at | - | -2,756478458 | -2,89013181 | 0,133653352 | |
| 1560735_s_at | LOC727924 /// O | -2,756478458 | -2,89013181 | 0,133653352 | |
| 1561442_at | LOC283585 | -2,756478458 | -2,89013181 | 0,133653352 | |
| 1563053_at | LOC729083 | -2,756478458 | -2,89013181 | 0,133653352 | |
| 1563834_a_at | AKNAD1 | -2,756478458 | -2,89013181 | 0,133653352 | |
| 1564358_at | - | -2,756478458 | -2,89013181 | 0,133653352 | |
| 1564841_at | - | -2,756478458 | -2,89013181 | 0,133653352 | |
| 1564937_at | - | -2,756478458 | -2,89013181 | 0,133653352 | |

| | | | | | |
|---|---|---|---|---|---|
| 1569407_at | - | -2,756478458 | -2,89013181 | 0,133653352 | |
| 203850_s_at | KIF1A | -2,756478458 | -2,89013181 | 0,133653352 | |
| 206022_at | NDP | -2,756478458 | -2,89013181 | 0,133653352 | |
| 216632_at | NAV3 | -2,756478458 | -2,89013181 | 0,133653352 | |
| 221233_s_at | FAM135A | -2,756478458 | -2,89013181 | 0,133653352 | |
| 228038_at | SOX2 | -2,756478458 | -2,89013181 | 0,133653352 | |
| 229084_at | CNTN4 | -2,756478458 | -2,89013181 | 0,133653352 | |
| 229461_x_at | NEGR1 | -2,756478458 | -2,89013181 | 0,133653352 | |
| 234110_at | LOC283075 | -2,756478458 | -2,89013181 | 0,133653352 | |
| 234517_at | - | -2,756478458 | -2,89013181 | 0,133653352 | |
| 241656_at | - | -2,756478458 | -2,89013181 | 0,133653352 | |
| 242524_at | CBLN4 | -2,756478458 | -2,89013181 | 0,133653352 | |
| 243641_at | - | -2,756478458 | -2,89013181 | 0,133653352 | |
| 222627_at | VPS54 | 2,027765132 | 1,894118818 | 0,133646314 | |
| 1557331_at | POLR1B | 1,406361373 | 1,272721751 | 0,133639622 | |
| 222887_s_at | TMEM127 | 1,406361373 | 1,272721751 | 0,133639622 | |
| 228340_at | TLE3 | 1,406361373 | 1,272721751 | 0,133639622 | |
| 201238_s_at | CAPZA2 | 6,085317171 | 5,951678905 | 0,133638266 | |
| 224699_s_at | ESYT2 | 3,420201053 | 3,28656411 | 0,133636943 | |
| 212925_at | C19orf21 | 0,291166226 | 0,157549243 | 0,133616983 | |
| 214226_at | PRSS53 | 0,291166226 | 0,157549243 | 0,133616983 | |
| 235908_at | MMP11 | 0,291166226 | 0,157549243 | 0,133616983 | |
| 236883_at | - | 0,291166226 | 0,157549243 | 0,133616983 | |
| 203623_at | PLXNA3 | 0,613492853 | 0,479907041 | 0,133585812 | |
| 208042_at | AGGF1 | 0,613492853 | 0,479907041 | 0,133585812 | |
| 235686_at | TYW5 | 0,613492853 | 0,479907041 | 0,133585812 | |
| 208692_at | RPS3 | 7,64704569 | 7,513528164 | 0,133517526 | |
| 201780_s_at | RNF13 | 2,59162628 | 2,458228496 | 0,133397784 | |
| 225624_at | SNX29 | 0,03125738 | -0,102100538 | 0,133357918 | |
| 241795_at | RHEB | 0,03125738 | -0,102100538 | 0,133357918 | |
| 205128_x_at | PTGS1 | 3,786397376 | 3,653046807 | 0,133350569 | |
| 218066_at | SLC12A7 | 3,517535002 | 3,384231608 | 0,133303394 | |
| 204871_at | MTERF | 1,960118853 | 1,826973888 | 0,133144965 | |
| 227049_at | ZADH2 | 1,841816345 | 1,708698295 | 0,13311805 | |
| 1555823_at | PACS2 | 3,883077619 | 3,75000829 | 0,133069329 | |
| 201410_at | PLEKHB2 | 4,984696817 | 4,851633873 | 0,133062944 | |
| 203264_s_at | ARHGEF9 | 1,758252436 | 1,6252386 | 0,133013836 | |
| 229653_at | VPS53 | 1,758252436 | 1,6252386 | 0,133013836 | |
| 214442_s_at | PIAS2 | 2,173470163 | 2,040470494 | 0,132999669 | |
| 208655_at | CCNI | 4,123436638 | 3,990437532 | 0,132999106 | |
| 215017_s_at | FNBP1L | 2,2992452 | 2,166264721 | 0,132980479 | |
| 207738_s_at | NCKAP1 | 4,246090231 | 4,113143231 | 0,132947 | |
| 1554268_at | MORN1 | -1,131062212 | -1,264005341 | 0,132943128 | |
| 1557064_s_at | HGSNAT | -1,131062212 | -1,264005341 | 0,132943128 | |
| 1564446_at | LOC284930 | -1,131062212 | -1,264005341 | 0,132943128 | |
| 202198_s_at | MTMR3 | -1,131062212 | -1,264005341 | 0,132943128 | |
| 208477_at | KCNC1 | -1,131062212 | -1,264005341 | 0,132943128 | |
| 221102_s_at | TRPM6 | -1,131062212 | -1,264005341 | 0,132943128 | |
| 221332_at | BMP15 | -1,131062212 | -1,264005341 | 0,132943128 | |
| 222268_x_at | MUC5B | -1,131062212 | -1,264005341 | 0,132943128 | |
| 229177_at | C16orf89 | -1,131062212 | -1,264005341 | 0,132943128 | |
| 237945_at | - | -1,131062212 | -1,264005341 | 0,132943128 | |
| 241067_at | - | -1,131062212 | -1,264005341 | 0,132943128 | |
| 241690_at | FAM205B | -1,131062212 | -1,264005341 | 0,132943128 | |
| 242902_at | CYMP | -1,131062212 | -1,264005341 | 0,132943128 | |
| 243603_at | - | -1,131062212 | -1,264005341 | 0,132943128 | |

| | | | | | |
|---|---|---|---|---|---|
| 202008_s_at | NID1 | 1,037706275 | 0,904843258 | 0,132863017 | |
| 223626_x_at | IFI27L2 | 1,523472489 | 1,390636111 | 0,132836378 | |
| 224625_x_at | C15orf63 /// MIR1 | 6,64552699 | 6,512702464 | 0,132824527 | |
| 1563947_a_at | ERC1 | -0,285837228 | -0,41858459 | 0,132747361 | |
| 201850_at | CAPG | -0,285837228 | -0,41858459 | 0,132747361 | |
| 206474_at | CDK17 | -0,285837228 | -0,41858459 | 0,132747361 | |
| 220747_at | HSPC072 | -0,285837228 | -0,41858459 | 0,132747361 | |
| 225709_at | ARL6IP6 | -0,285837228 | -0,41858459 | 0,132747361 | |
| 229601_at | SCYL1 | -0,285837228 | -0,41858459 | 0,132747361 | |
| 231638_at | PRSS30P | -0,285837228 | -0,41858459 | 0,132747361 | |
| 232508_at | ZMIZ1 | -0,285837228 | -0,41858459 | 0,132747361 | |
| 239246_at | FARP1 | -0,285837228 | -0,41858459 | 0,132747361 | |
| 240849_at | - | -0,285837228 | -0,41858459 | 0,132747361 | |
| 221939_at | YIPF2 | 3,956428813 | 3,823741953 | 0,13268686 | |
| 212104_s_at | RBFOX2 | 2,718233611 | 2,585563171 | 0,13267044 | |
| 223115_at | MED17 | 0,888485433 | 0,755840599 | 0,132644834 | |
| 226013_at | TRAK1 | 0,888485433 | 0,755840599 | 0,132644834 | |
| 227305_s_at | SMCR8 | 4,281994342 | 4,149442805 | 0,132551537 | |
| 218008_at | TMEM248 | 3,939667505 | 3,807174423 | 0,132493082 | |
| 215833_s_at | SPPL2B | 0,627515113 | 0,495031851 | 0,132483262 | |
| 217023_x_at | TPSAB1 /// TPSE | 0,627515113 | 0,495031851 | 0,132483262 | |
| 229882_at | RPS15A | 0,627515113 | 0,495031851 | 0,132483262 | |
| 237473_at | PPIEL | 0,627515113 | 0,495031851 | 0,132483262 | |
| 212416_at | SCAMP1 | 3,663333942 | 3,530865925 | 0,132468018 | |
| 208980_s_at | UBC | 6,82042267 | 6,687974626 | 0,132448044 | |
| 203346_s_at | MTF2 | 3,503346991 | 3,370970266 | 0,132376726 | |
| 202527_s_at | SMAD4 | 2,87191078 | 2,739547836 | 0,132362944 | |
| 209248_at | GHITM | 5,578621938 | 5,446261552 | 0,132360386 | |
| 202670_at | MAP2K1 | 4,351756271 | 4,219411762 | 0,132344509 | |
| 200005_at | EIF3D | 5,711375436 | 5,579053664 | 0,132321772 | |
| 225784_s_at | ZC4H2 | 1,722769813 | 1,590454215 | 0,132315598 | |
| 208184_s_at | TRAPPC10 | 2,580856819 | 2,448549499 | 0,13230732 | |
| 226119_at | PCMTD1 | 2,605861638 | 2,473581079 | 0,132280558 | |
| 209595_at | GTF2F2 | 2,478200152 | 2,345926937 | 0,132273215 | |
| 1554508_at | PIK3AP1 | 0,30867765 | 0,176436073 | 0,132241577 | |
| 1558484_s_at | LRRC27 | 0,30867765 | 0,176436073 | 0,132241577 | |
| 209170_s_at | GPM6B | 0,30867765 | 0,176436073 | 0,132241577 | |
| 214731_at | - | 0,30867765 | 0,176436073 | 0,132241577 | |
| 229827_at | BUB3 | 0,30867765 | 0,176436073 | 0,132241577 | |
| 231161_x_at | - | 0,30867765 | 0,176436073 | 0,132241577 | |
| 210252_s_at | MADD | 2,531373922 | 2,39915583 | 0,132218092 | |
| 212749_s_at | RCHY1 | 4,046594062 | 3,914426433 | 0,132167629 | |
| 235094_at | - | 2,082235068 | 1,950068772 | 0,132166295 | |
| 209805_at | PMS2 /// PMS2C | 1,631786706 | 1,499637023 | 0,132149683 | |
| 211554_s_at | APAF1 | 0,425644434 | 0,293518836 | 0,132125598 | |
| 223589_at | ZNF416 | 1,426552034 | 1,294497133 | 0,132054901 | |
| 230021_at | C15orf42 | 1,426552034 | 1,294497133 | 0,132054901 | |
| 203081_at | CTNNBIP1 | 2,048758794 | 1,916759838 | 0,131998956 | |
| 209317_at | POLR1C | 4,235256884 | 4,103307085 | 0,131949799 | |
| 204550_x_at | GSTM1 | 1,187200746 | 1,055274826 | 0,13192592 | |
| 1555292_at | FAM40B | 1,897847661 | 1,765933685 | 0,131913976 | |
| 204420_at | FOSL1 | 1,897847661 | 1,765933685 | 0,131913976 | |
| 202159_at | FARSA | 3,251631457 | 3,119728903 | 0,131902554 | |
| 225936_at | EID2 | 3,827217859 | 3,695402096 | 0,131815762 | |
| 213005_s_at | KANK1 | 0,900083947 | 0,768343791 | 0,131740157 | |
| 214958_s_at | TMC6 | 0,900083947 | 0,768343791 | 0,131740157 | |

| | | | | | |
|---|---|---|---|---|---|
| 1558103_a_at | - | 0,052199481 | -0,079522948 | 0,131722429 | |
| 1569296_a_at | - | 0,052199481 | -0,079522948 | 0,131722429 | |
| 216658_at | - | 0,052199481 | -0,079522948 | 0,131722429 | |
| 230168_at | - | 0,052199481 | -0,079522948 | 0,131722429 | |
| 235699_at | REM2 | 0,052199481 | -0,079522948 | 0,131722429 | |
| 211529_x_at | HLA-G | 4,685380613 | 4,553720057 | 0,131660556 | |
| 201461_s_at | MAPKAPK2 | 1,05337236 | 0,921731975 | 0,131640384 | |
| 204316_at | RGS10 | 1,05337236 | 0,921731975 | 0,131640384 | |
| 213133_s_at | GCSH | 4,63941169 | 4,507854821 | 0,131556869 | |
| 200723_s_at | CAPRIN1 | 5,729772784 | 5,598221512 | 0,131551272 | |
| 223350_x_at | LIN7C | 3,999522577 | 3,86798237 | 0,131540207 | |
| 212243_at | GCOM1 /// POLR | 2,540666392 | 2,409170778 | 0,131495614 | |
| 201174_s_at | TERF2IP | 4,176858496 | 4,045409633 | 0,131448863 | |
| 1558139_at | FLJ39632 /// LOC | 1,434549838 | 1,303116084 | 0,131433753 | |
| 227228_s_at | CCDC88C | 1,434549838 | 1,303116084 | 0,131433753 | |
| 203194_s_at | NUP98 | 2,406821352 | 2,275409734 | 0,131411618 | |
| 1553739_at | IRAK2 | -2,823166348 | -2,954576755 | 0,131410407 | |
| 1554190_s_at | PLEKHS1 | -2,823166348 | -2,954576755 | 0,131410407 | |
| 1555403_a_at | CDH19 | -2,823166348 | -2,954576755 | 0,131410407 | |
| 1555623_at | - | -2,823166348 | -2,954576755 | 0,131410407 | |
| 1556448_a_at | LINC00606 | -2,823166348 | -2,954576755 | 0,131410407 | |
| 1556529_a_at | LOC285326 | -2,823166348 | -2,954576755 | 0,131410407 | |
| 1557735_at | - | -2,823166348 | -2,954576755 | 0,131410407 | |
| 1566882_at | - | -2,823166348 | -2,954576755 | 0,131410407 | |
| 1567361_at | BDNF-AS | -2,823166348 | -2,954576755 | 0,131410407 | |
| 210732_s_at | LGALS8 | -2,823166348 | -2,954576755 | 0,131410407 | |
| 210767_at | NF2 | -2,823166348 | -2,954576755 | 0,131410407 | |
| 211766_s_at | PNLIPRP2 | -2,823166348 | -2,954576755 | 0,131410407 | |
| 215763_at | - | -2,823166348 | -2,954576755 | 0,131410407 | |
| 219230_at | TMEM100 | -2,823166348 | -2,954576755 | 0,131410407 | |
| 220586_at | CHD9 | -2,823166348 | -2,954576755 | 0,131410407 | |
| 231690_at | - | -2,823166348 | -2,954576755 | 0,131410407 | |
| 237056_at | INSC | -2,823166348 | -2,954576755 | 0,131410407 | |
| 237873_s_at | - | -2,823166348 | -2,954576755 | 0,131410407 | |
| 240061_at | - | -2,823166348 | -2,954576755 | 0,131410407 | |
| 241482_at | EPHA1 | -2,823166348 | -2,954576755 | 0,131410407 | |
| 228595_at | HSD17B1 | 1,593009234 | 1,461646549 | 0,131362685 | |
| 219868_s_at | ANKFY1 | 0,735041319 | 0,603679647 | 0,131361673 | |
| 200638_s_at | YWHAZ | 6,473160945 | 6,34180139 | 0,131359555 | |
| 1558584_at | UBL4B | 0,822971223 | 0,691645685 | 0,131325538 | |
| 223260_s_at | POLK | 0,822971223 | 0,691645685 | 0,131325538 | |
| 211090_s_at | PRPF4B | 3,769107843 | 3,637795954 | 0,131311889 | |
| 214327_x_at | TPT1 | 7,246486658 | 7,115177681 | 0,131308978 | |
| 228397_at | TUG1 | 2,320980353 | 2,189694216 | 0,131286137 | |
| 229850_at | KDSR | 1,865674241 | 1,73441697 | 0,131257271 | |
| 57540_at | RBKS | 0,380817344 | 0,249604497 | 0,131212847 | |
| 228768_at | FNIP1 | 3,737131445 | 3,605931378 | 0,131200066 | |
| 207082_at | CSF1 | -1,089016378 | -1,220210588 | 0,13119421 | |
| 209887_at | SMAD6 | -1,089016378 | -1,220210588 | 0,13119421 | |
| 215892_at | ZNF440 | -1,089016378 | -1,220210588 | 0,13119421 | |
| 216247_at | RPS20 /// SNORI | -1,089016378 | -1,220210588 | 0,13119421 | |
| 220279_at | TRIM17 | -1,089016378 | -1,220210588 | 0,13119421 | |
| 220282_at | RIC3 | -1,089016378 | -1,220210588 | 0,13119421 | |
| 223798_at | SLC41A2 | -1,089016378 | -1,220210588 | 0,13119421 | |
| 227097_at | - | -1,089016378 | -1,220210588 | 0,13119421 | |
| 233375_at | EFCAB2 | -1,089016378 | -1,220210588 | 0,13119421 | |

| | | | | | |
|---|---|---|---|---|---|
| 235092_at | - | -1,089016378 | -1,220210588 | 0,13119421 | |
| 238500_at | EMP2 | -1,089016378 | -1,220210588 | 0,13119421 | |
| 239688_at | SMC1A | -1,089016378 | -1,220210588 | 0,13119421 | |
| 242303_at | NRG2 | -1,089016378 | -1,220210588 | 0,13119421 | |
| 243440_at | SIX3-AS1 | -1,089016378 | -1,220210588 | 0,13119421 | |
| 1554168_a_at | SH3KBP1 | 4,138203514 | 4,007018329 | 0,131185185 | |
| 214752_x_at | FLNA | 3,595400101 | 3,464222256 | 0,131177845 | |
| 37433_at | PIAS2 | 0,644853431 | 0,513717619 | 0,131135812 | |
| 57163_at | ELOVL1 | 2,908260915 | 2,777153862 | 0,131107053 | |
| 227267_at | POC5 | 1,69307511 | 1,562009954 | 0,131065156 | |
| 233049_x_at | STUB1 | 4,446258267 | 4,31519468 | 0,131063587 | |
| 228009_x_at | ZNRD1 | 4,953379586 | 4,822328446 | 0,13105114 | |
| 200025_s_at | RPL27 | 7,801279985 | 7,67024222 | 0,131037765 | |
| 218875_s_at | FBXO5 | 4,546987586 | 4,41598058 | 0,131007006 | |
| 201839_s_at | EPCAM | 5,635779871 | 5,504875333 | 0,130904538 | |
| 1553479_at | TMEM145 | -0,089689931 | -0,220592223 | 0,130902292 | |
| 208465_at | GRM2 | -0,089689931 | -0,220592223 | 0,130902292 | |
| 213748_at | TRIM66 | -0,089689931 | -0,220592223 | 0,130902292 | |
| 220600_at | C3orf75 | -0,089689931 | -0,220592223 | 0,130902292 | |
| 224049_at | KCNK17 | -0,089689931 | -0,220592223 | 0,130902292 | |
| 227544_at | TMEM229B | -0,089689931 | -0,220592223 | 0,130902292 | |
| 227573_s_at | OBSL1 | -0,089689931 | -0,220592223 | 0,130902292 | |
| 229313_at | ANO5 | -0,089689931 | -0,220592223 | 0,130902292 | |
| 229854_at | OBSCN | -0,089689931 | -0,220592223 | 0,130902292 | |
| 238082_at | - | -0,089689931 | -0,220592223 | 0,130902292 | |
| 1564112_at | FAM71A | 0,44160593 | 0,310717132 | 0,130888798 | |
| 213349_at | TMCC1 | 0,44160593 | 0,310717132 | 0,130888798 | |
| 216698_x_at | OR7E12P | 0,44160593 | 0,310717132 | 0,130888798 | |
| 35147_at | MCF2L | 0,496120222 | 0,365236551 | 0,130883671 | |
| 223454_at | CXCL16 | 0,648296226 | 0,51742585 | 0,130870375 | |
| 230634_x_at | ADAT3 | 0,648296226 | 0,51742585 | 0,130870375 | |
| 212120_at | RHOQ | 2,266015882 | 2,135226207 | 0,130789675 | |
| 212869_x_at | TPT1 | 8,130584911 | 7,999803985 | 0,130780926 | |
| 208609_s_at | TNXB | -0,259832361 | -0,39061235 | 0,130779989 | |
| 209261_s_at | NR2F6 | -0,259832361 | -0,39061235 | 0,130779989 | |
| 227169_at | DNAJC18 | -0,259832361 | -0,39061235 | 0,130779989 | |
| 234499_at | - | -0,259832361 | -0,39061235 | 0,130779989 | |
| 235845_at | SP5 | -0,259832361 | -0,39061235 | 0,130779989 | |
| 221989_at | RPL10 /// SNORA | 2,742596529 | 2,611817827 | 0,130778702 | |
| 229835_s_at | SLMO2 | 2,416948786 | 2,286318242 | 0,130630544 | |
| 227580_s_at | TECPR1 | 2,102456302 | 1,971855016 | 0,130601286 | |
| 200857_s_at | NCOR1 | 1,789769151 | 1,659204017 | 0,130565134 | |
| 216331_at | ITGA7 | 1,390002893 | 1,259496994 | 0,130505899 | |
| 224786_at | SCOC | 5,054797981 | 4,924316041 | 0,13048194 | |
| 204240_s_at | SMC2 | 3,617524043 | 3,487095824 | 0,130428219 | |
| 240307_at | - | 0,917308734 | 0,786897721 | 0,130411013 | |
| 32032_at | DGCR14 /// TSSK | 3,296204798 | 3,165941392 | 0,130263407 | |
| 1555705_a_at | CMTM3 | 2,997346717 | 2,867091644 | 0,130255072 | |
| 200003_s_at | RPL28 | 8,048117714 | 7,917867472 | 0,130250242 | |
| 239333_x_at | - | 0,334552598 | 0,204310755 | 0,130241843 | |
| 216508_x_at | - | 4,378950369 | 4,248708657 | 0,130241711 | |
| 202352_s_at | PSMD12 | 5,323562354 | 5,193345242 | 0,130217112 | |
| 215952_s_at | OAZ1 | 6,713043537 | 6,582844205 | 0,130199332 | |
| 1563983_at | TPT1-AS1 | 0,209623097 | 0,079428135 | 0,130194962 | |
| 228250_at | FNIP1 | 0,209623097 | 0,079428135 | 0,130194962 | |
| 214726_x_at | ADD1 | 1,274448124 | 1,144305291 | 0,130142833 | |

| | | | | | |
|---|---|---|---|---|---|
| 238898_at | LOC100505730 | 1,274448124 | 1,144305291 | 0,130142833 | |
| 202149_at | NEDD9 | 1,880387527 | 1,750261389 | 0,130126138 | |
| 227226_at | MRAP2 | 2,506929603 | 2,376875125 | 0,130054478 | |
| 225456_at | MED1 | 3,000043914 | 2,869995113 | 0,130048801 | |
| 217852_s_at | ARL8B | 5,656015641 | 5,525968169 | 0,130047472 | |
| 1553930_at | TAAR1 | -2,866296344 | -2,996335703 | 0,130039359 | |
| 1555307_at | TTF2 | -2,866296344 | -2,996335703 | 0,130039359 | |
| 1555369_at | BAGE | -2,866296344 | -2,996335703 | 0,130039359 | |
| 1558226_a_at | - | -2,866296344 | -2,996335703 | 0,130039359 | |
| 1559707_at | - | -2,866296344 | -2,996335703 | 0,130039359 | |
| 1560637_at | - | -2,866296344 | -2,996335703 | 0,130039359 | |
| 1562418_at | - | -2,866296344 | -2,996335703 | 0,130039359 | |
| 1564807_at | - | -2,866296344 | -2,996335703 | 0,130039359 | |
| 207307_at | HTR2C | -2,866296344 | -2,996335703 | 0,130039359 | |
| 211878_at | - | -2,866296344 | -2,996335703 | 0,130039359 | |
| 214012_at | ERAP1 | -2,866296344 | -2,996335703 | 0,130039359 | |
| 214685_at | NOP14-AS1 | -2,866296344 | -2,996335703 | 0,130039359 | |
| 215634_at | - | -2,866296344 | -2,996335703 | 0,130039359 | |
| 215878_at | ITGB1 | -2,866296344 | -2,996335703 | 0,130039359 | |
| 216440_at | - | -2,866296344 | -2,996335703 | 0,130039359 | |
| 231581_at | LOC400680 | -2,866296344 | -2,996335703 | 0,130039359 | |
| 233084_s_at | SYCE1 | -2,866296344 | -2,996335703 | 0,130039359 | |
| 233904_at | - | -2,866296344 | -2,996335703 | 0,130039359 | |
| 234134_at | - | -2,866296344 | -2,996335703 | 0,130039359 | |
| 234501_x_at | - | -2,866296344 | -2,996335703 | 0,130039359 | |
| 237729_at | LOC100505676 | -2,866296344 | -2,996335703 | 0,130039359 | |
| 238264_at | - | -2,866296344 | -2,996335703 | 0,130039359 | |
| 238957_at | PDXDC2P | -2,866296344 | -2,996335703 | 0,130039359 | |
| 240819_at | - | -2,866296344 | -2,996335703 | 0,130039359 | |
| 241206_at | - | -2,866296344 | -2,996335703 | 0,130039359 | |
| 241283_at | - | -2,866296344 | -2,996335703 | 0,130039359 | |
| 242252_at | - | -2,866296344 | -2,996335703 | 0,130039359 | |
| 242401_x_at | - | -2,866296344 | -2,996335703 | 0,130039359 | |
| 228536_at | PRMT10 | 2,109966786 | 1,979940779 | 0,130026007 | |
| 48117_at | CCDC101 | 1,367202759 | 1,23718272 | 0,13002004 | |
| 226285_at | CAPRIN1 | 2,683419202 | 2,553413592 | 0,130005609 | |
| 203255_at | FBXO11 | 3,396853889 | 3,266872397 | 0,129981493 | |
| 238389_s_at | - | 1,560488174 | 1,43051703 | 0,129971144 | |
| 203159_at | GLS | 2,277177578 | 2,147243253 | 0,129934326 | |
| 217913_at | VPS4A | 3,146001821 | 3,016072743 | 0,129929077 | |
| 226976_at | KPNA6 /// LOC10 | 3,076146581 | 2,946258252 | 0,129888329 | |
| 233873_x_at | MTPAP | 3,002736078 | 2,87289275 | 0,129843328 | |
| 202617_s_at | MECP2 | 0,661985843 | 0,532164371 | 0,129821472 | |
| 227777_at | - | 0,661985843 | 0,532164371 | 0,129821472 | |
| 231817_at | USP53 | 1,709647566 | 1,579853228 | 0,129794337 | |
| 213649_at | SRSF7 | 2,732900676 | 2,603119255 | 0,129781421 | |
| 224319_s_at | SMCR7L | 2,003776245 | 1,874012195 | 0,12976405 | |
| 224573_at | RNASEK /// RNA | 5,835675329 | 5,705937317 | 0,129738012 | |
| 217165_x_at | MT1F | 4,463949573 | 4,334242016 | 0,129707557 | |
| 220807_at | HBQ1 | 2,400710591 | 2,271023131 | 0,12968746 | |
| 227617_at | TMEM201 | 2,400710591 | 2,271023131 | 0,12968746 | |
| 212649_at | DHX29 | 1,079110292 | 0,949447784 | 0,129662508 | |
| 37590_g_at | - | -0,680266109 | -0,809921974 | 0,129655865 | |
| 225927_at | MAP3K1 | 3,907705327 | 3,778101865 | 0,129603462 | |
| 229957_at | TMEM91 | 1,40228911 | 1,272721751 | 0,129567359 | |
| 224943_at | BTBD7 | 1,283332461 | 1,153866761 | 0,129465699 | |

| | | | | | |
|---|---|---|---|---|---|
| 212071_s_at | SPTBN1 | 2,737756747 | 2,608344689 | 0,129412058 | |
| 206611_at | C2orf27A | 0,083054335 | -0,046302147 | 0,129356482 | |
| 217891_at | C16orf58 | 0,083054335 | -0,046302147 | 0,129356482 | |
| 222960_at | CACNA1H | 0,083054335 | -0,046302147 | 0,129356482 | |
| 1553959_a_at | B3GALT6 | 3,838564178 | 3,709248632 | 0,129315547 | |
| 226263_at | SNRNP48 | 2,912562019 | 2,783327323 | 0,129234696 | |
| 203419_at | MLL4 | 2,646022818 | 2,516830926 | 0,129191893 | |
| 221515_s_at | LCMT1 | 3,53158484 | 3,402394519 | 0,129190321 | |
| 224888_at | EPT1 | 2,348755185 | 2,21959457 | 0,129160615 | |
| 1553174_at | JPH2 | -0,43775896 | -0,566887459 | 0,129128499 | |
| 1561305_at | - | -0,43775896 | -0,566887459 | 0,129128499 | |
| 209223_at | NDUFA2 | -0,43775896 | -0,566887459 | 0,129128499 | |
| 217625_x_at | LOC100506190 | -0,43775896 | -0,566887459 | 0,129128499 | |
| 217691_x_at | SLC16A3 | -0,43775896 | -0,566887459 | 0,129128499 | |
| 227329_at | ZBTB46 | -0,43775896 | -0,566887459 | 0,129128499 | |
| 228034_x_at | ALKBH5 /// LOC1 | -0,43775896 | -0,566887459 | 0,129128499 | |
| 236736_at | - | -0,43775896 | -0,566887459 | 0,129128499 | |
| 238193_at | - | -0,43775896 | -0,566887459 | 0,129128499 | |
| 227854_at | - | 0,46522187 | 0,336136178 | 0,129085693 | |
| 201940_at | CPD | 1,894952274 | 1,765933685 | 0,129018589 | |
| 202246_s_at | CDK4 | 5,096666579 | 4,967654074 | 0,129012505 | |
| 223210_at | CHURC1 | 2,051361651 | 1,922365006 | 0,128996645 | |
| 201819_at | SCARB1 | 1,672933557 | 1,543943227 | 0,128990329 | |
| 219001_s_at | DCAF10 | 1,672933557 | 1,543943227 | 0,128990329 | |
| 210574_s_at | NUDC | 5,004644064 | 4,875668581 | 0,128975483 | |
| 223068_at | EML4 | 3,508091848 | 3,379145507 | 0,128946341 | |
| 242407_at | - | 0,767063567 | 0,638150376 | 0,128913191 | |
| 206097_at | SLC22A18AS | 1,089278259 | 0,960386719 | 0,12889154 | |
| 223440_at | C16orf70 | 2,194816625 | 2,066015817 | 0,128800808 | |
| 238070_at | CHD1L | 2,194816625 | 2,066015817 | 0,128800808 | |
| 201190_s_at | PITPNA | 3,67603922 | 3,547283438 | 0,128755782 | |
| 1553022_at | ZIM3 | -2,908843709 | -3,037500868 | 0,128657159 | |
| 1559953_at | DDX42 | -2,908843709 | -3,037500868 | 0,128657159 | |
| 1561196_at | - | -2,908843709 | -3,037500868 | 0,128657159 | |
| 1562736_at | LHX9 | -2,908843709 | -3,037500868 | 0,128657159 | |
| 1562880_at | LOC100288238 | -2,908843709 | -3,037500868 | 0,128657159 | |
| 1563611_at | - | -2,908843709 | -3,037500868 | 0,128657159 | |
| 1565026_a_at | OFCC1 | -2,908843709 | -3,037500868 | 0,128657159 | |
| 1566162_x_at | - | -2,908843709 | -3,037500868 | 0,128657159 | |
| 209465_x_at | LOC100287705 / | -2,908843709 | -3,037500868 | 0,128657159 | |
| 216619_at | GTPBP10 | -2,908843709 | -3,037500868 | 0,128657159 | |
| 228440_at | - | -2,908843709 | -3,037500868 | 0,128657159 | |
| 231648_at | - | -2,908843709 | -3,037500868 | 0,128657159 | |
| 233845_at | - | -2,908843709 | -3,037500868 | 0,128657159 | |
| 240179_at | LOC100505801 | -2,908843709 | -3,037500868 | 0,128657159 | |
| 240747_at | - | -2,908843709 | -3,037500868 | 0,128657159 | |
| 241172_at | GRIA2 | -2,908843709 | -3,037500868 | 0,128657159 | |
| 241436_at | SCNN1G | -2,908843709 | -3,037500868 | 0,128657159 | |
| 226453_at | RNASEH2C | 2,325288363 | 2,196649505 | 0,128638858 | |
| 225845_at | ZBTB44 | 3,539022513 | 3,410394103 | 0,12862841 | |
| 38149_at | ARHGAP25 | -0,142235148 | -0,270834454 | 0,128599306 | |
| 202891_at | NIT1 | 2,678376417 | 2,549796788 | 0,128579629 | |
| 225272_at | SAT2 | 1,129248302 | 1,000684521 | 0,128563781 | |
| 225377_at | RABL6 | 1,129248302 | 1,000684521 | 0,128563781 | |
| 223324_s_at | TRPM7 | 1,726031811 | 1,59747851 | 0,1285533 | |
| 238959_at | LARP4 | 1,726031811 | 1,59747851 | 0,1285533 | |

| | | | | | |
|---|---|---|---|---|---|
| 225306_s_at | SLC25A29 | 2,232003119 | 2,103505212 | 0,128497907 | |
| 206163_at | MAB21L1 | 3,643632572 | 3,515187047 | 0,128445525 | |
| 215629_s_at | DLEU2 /// DLEU2 | 2,022468633 | 1,894118818 | 0,128349816 | |
| 1560498_at | - | -1,026571149 | -1,154897679 | 0,12832653 | |
| 1570191_at | - | -1,026571149 | -1,154897679 | 0,12832653 | |
| 203807_x_at | CSH2 | -1,026571149 | -1,154897679 | 0,12832653 | |
| 208377_s_at | CACNA1F | -1,026571149 | -1,154897679 | 0,12832653 | |
| 214365_at | TPM3 | -1,026571149 | -1,154897679 | 0,12832653 | |
| 216249_at | PVT1 | -1,026571149 | -1,154897679 | 0,12832653 | |
| 232543_x_at | ARHGAP9 | -1,026571149 | -1,154897679 | 0,12832653 | |
| 232976_at | SNORD116-17 // | -1,026571149 | -1,154897679 | 0,12832653 | |
| 238420_at | TAOK1 | -1,026571149 | -1,154897679 | 0,12832653 | |
| 239401_at | - | -1,026571149 | -1,154897679 | 0,12832653 | |
| 244481_at | - | -1,026571149 | -1,154897679 | 0,12832653 | |
| 208376_at | CCR4 | 0,682279745 | 0,553993624 | 0,128286121 | |
| 228231_at | SNX8 | 0,945567441 | 0,817300337 | 0,128267105 | |
| 223043_at | EMC4 | 5,381596876 | 5,253365418 | 0,128231459 | |
| 205457_at | C6orf106 | 1,683039483 | 1,55481039 | 0,128229092 | |
| 239355_at | GMCL1 | 3,34687727 | 3,218713651 | 0,128163619 | |
| 218949_s_at | QRSL1 | 2,986507217 | 2,858346012 | 0,128161205 | |
| 212370_x_at | FAM21A /// FAM2 | 3,063308547 | 2,935211251 | 0,128097296 | |
| 201734_at | CLCN3 | 4,833365882 | 4,705281859 | 0,128084023 | |
| 208756_at | EIF3I | 6,126938787 | 5,998864294 | 0,128074493 | |
| 1555751_a_at | GEMIN7 | 3,451082821 | 3,323041804 | 0,128041017 | |
| 225069_at | PCYT1A | 2,908260915 | 2,780243895 | 0,12801702 | |
| 204235_s_at | GULP1 | 2,027765132 | 1,899812471 | 0,127952661 | |
| 1570375_at | LOC100506585 | -0,221656139 | -0,34958478 | 0,127928642 | |
| 206287_s_at | ITIH4 /// MUSTN1 | -0,221656139 | -0,34958478 | 0,127928642 | |
| 213602_s_at | MMP11 | -0,221656139 | -0,34958478 | 0,127928642 | |
| 221184_at | - | -0,221656139 | -0,34958478 | 0,127928642 | |
| 228404_at | IRX2 | -0,221656139 | -0,34958478 | 0,127928642 | |
| 229762_at | - | -0,221656139 | -0,34958478 | 0,127928642 | |
| 232748_at | PAPPA | -0,221656139 | -0,34958478 | 0,127928642 | |
| 1563638_at | FAM18A | 0,480753764 | 0,352836757 | 0,127917006 | |
| 203750_s_at | RARA | 0,480753764 | 0,352836757 | 0,127917006 | |
| 207006_s_at | CCDC106 | 0,480753764 | 0,352836757 | 0,127917006 | |
| 223177_at | NT5DC1 | 3,335176289 | 3,207279726 | 0,127896563 | |
| 226353_at | SPPL2A | 3,085065712 | 2,957221307 | 0,127844405 | |
| 202284_s_at | CDKN1A | 2,931053371 | 2,803210557 | 0,127842814 | |
| 206800_at | MTHFR | 0,103263601 | -0,024572586 | 0,127836188 | |
| 212817_at | DNAJB5 | 0,103263601 | -0,024572586 | 0,127836188 | |
| 215603_x_at | GGT1 /// GGT2 // | 0,103263601 | -0,024572586 | 0,127836188 | |
| 234882_at | - | 0,103263601 | -0,024572586 | 0,127836188 | |
| 235298_at | WDR27 | 0,103263601 | -0,024572586 | 0,127836188 | |
| 200888_s_at | RPL23 | 7,389826452 | 7,262003475 | 0,127822978 | |
| 1555697_at | KLK4 | 1,735773781 | 1,60795124 | 0,127822541 | |
| 54970_at | ZMIZ2 | 2,588941442 | 2,461119577 | 0,127821865 | |
| 204244_s_at | DBF4 | 4,848036436 | 4,720241528 | 0,127794908 | |
| 203820_s_at | IGF2BP3 | 3,274090262 | 3,146317015 | 0,127773248 | |
| 202970_at | DYRK2 | 1,104397092 | 0,976641161 | 0,127755931 | |
| 233999_s_at | TTC26 | 1,104397092 | 0,976641161 | 0,127755931 | |
| 235119_at | TAF3 | 1,104397092 | 0,976641161 | 0,127755931 | |
| 224621_at | MAPK1 | 4,329411337 | 4,201658983 | 0,127752355 | |
| 208752_x_at | NAP1L1 | 6,300667153 | 6,172950708 | 0,127716445 | |
| 212411_at | IMP4 | 4,087113308 | 3,959421856 | 0,127691452 | |
| 226502_at | ELMOD2 | 2,59162628 | 2,464004877 | 0,127621403 | |

| | | | | | |
|---|---|---|---|---|---|
| 208093_s_at | NDEL1 | 3,069741844 | 2,942125533 | 0,127616311 | |
| 1553587_a_at | POLE4 | 3,964737003 | 3,837157105 | 0,127579898 | |
| 200776_s_at | BZW1 | 5,521923587 | 5,3943954 | 0,127528187 | |
| 225404_at | C1orf212 | 3,914869478 | 3,787346121 | 0,127523358 | |
| 1553677_a_at | TIPRL | 2,59519831 | 2,467842989 | 0,12735532 | |
| 218578_at | CDC73 | 2,938102794 | 2,810785576 | 0,127317218 | |
| 221544_s_at | MED16 | 0,695652136 | 0,568365095 | 0,127287041 | |
| 226644_at | MIB2 | 0,695652136 | 0,568365095 | 0,127287041 | |
| 228382_at | FAM105B | 0,695652136 | 0,568365095 | 0,127287041 | |
| 54632_at | THADA | 2,433006244 | 2,305748082 | 0,127258163 | |
| 225864_at | FAM84B | 1,596577845 | 1,469325098 | 0,127252747 | |
| 228859_at | C4orf21 | 1,596577845 | 1,469325098 | 0,127252747 | |
| 207829_s_at | BNIP1 | 2,146943689 | 2,019703483 | 0,127240207 | |
| 203960_s_at | HSPB11 | 2,721505869 | 2,594367918 | 0,127137951 | |
| 208990_s_at | HNRNPH3 | 5,90459196 | 5,777460041 | 0,127131919 | |
| 217678_at | SLC7A11 | 2,37600538 | 2,248887791 | 0,127117589 | |
| 1563302_at | - | 0,376672223 | 0,249604176 | 0,127068048 | |
| 204405_x_at | DIMT1 | 4,674447146 | 4,547386035 | 0,127061111 | |
| 229342_at | C17orf85 | 0,962260738 | 0,835239052 | 0,127021686 | |
| 238121_at | GK5 | 1,114389003 | 0,987376598 | 0,127012406 | |
| 203499_at | EPHA2 | 1,318332599 | 1,191491281 | 0,126841318 | |
| 228311_at | BCL6B | 1,318332599 | 1,191491281 | 0,126841318 | |
| 238299_at | - | 0,882651021 | 0,755840599 | 0,126810421 | |
| 227158_at | C14orf126 | 3,062018438 | 2,935211251 | 0,126807187 | |
| 217750_s_at | UBE2Z | 4,311065622 | 4,18426834 | 0,126797282 | |
| 214003_x_at | RPS20 /// SNORI | 8,098027738 | 7,97123216 | 0,126795578 | |
| 224793_s_at | TGFBR1 | 3,985300536 | 3,858511405 | 0,126789131 | |
| 1552276_a_at | VPS18 | 0,255492549 | 0,128747141 | 0,126745409 | |
| 203932_at | HLA-DMB | 0,255492549 | 0,128747141 | 0,126745409 | |
| 222038_s_at | UTP18 | 0,255492549 | 0,128747141 | 0,126745409 | |
| 223410_s_at | - | 0,255492549 | 0,128747141 | 0,126745409 | |
| 229402_at | SAMD13 | 0,255492549 | 0,128747141 | 0,126745409 | |
| 241234_at | LOC100506797 | 0,255492549 | 0,128747141 | 0,126745409 | |
| 226611_s_at | CENPV | 3,904829675 | 3,778101865 | 0,126727811 | |
| 201688_s_at | TPD52 | 1,496984567 | 1,370274469 | 0,126710098 | |
| 1560477_a_at | SAMD11 | -0,033456564 | -0,160136748 | 0,126680184 | |
| 238259_at | ADSSL1 | -0,033456564 | -0,160136748 | 0,126680184 | |
| 240080_at | - | -0,033456564 | -0,160136748 | 0,126680184 | |
| 241930_x_at | - | -0,033456564 | -0,160136748 | 0,126680184 | |
| 228299_at | KCTD20 | 4,604390697 | 4,477716877 | 0,12667382 | |
| 1554432_x_at | FAM165B | 1,751865623 | 1,6252386 | 0,126627022 | |
| 204587_at | SLC25A14 | 2,605861638 | 2,479296438 | 0,1265652 | |
| 226596_x_at | LOC729852 | 0,606430281 | 0,479907041 | 0,126523241 | |
| 230976_at | AK8 | 0,606430281 | 0,479907041 | 0,126523241 | |
| 235535_x_at | FRG1 /// FRG1B | 0,606430281 | 0,479907041 | 0,126523241 | |
| 1555846_a_at | - | 1,706348269 | 1,579853228 | 0,126495041 | |
| 1555429_at | - | -0,986356909 | -1,112851844 | 0,126494935 | |
| 1558785_a_at | RGS5 | -0,986356909 | -1,112851844 | 0,126494935 | |
| 1560485_at | HIVEP1 | -0,986356909 | -1,112851844 | 0,126494935 | |
| 207851_s_at | INSR | -0,986356909 | -1,112851844 | 0,126494935 | |
| 233027_at | - | -0,986356909 | -1,112851844 | 0,126494935 | |
| 234063_at | - | -0,986356909 | -1,112851844 | 0,126494935 | |
| 234637_at | KRTAP4-5 | -0,986356909 | -1,112851844 | 0,126494935 | |
| 235854_x_at | ROCK1 | -0,986356909 | -1,112851844 | 0,126494935 | |
| 215735_s_at | TSC2 | 1,191926657 | 1,065442793 | 0,126483864 | |
| 219083_at | SHQ1 | 4,226646405 | 4,100219485 | 0,12642692 | |

| | | | | | |
|---|---|---|---|---|---|
| 235132_at | LOC254128 | 1,844820248 | 1,718396606 | 0,126423642 | |
| 202496_at | EDC4 | 2,048758794 | 1,922365006 | 0,126393788 | |
| 203971_at | SLC31A1 | 2,755423794 | 2,629059192 | 0,126364601 | |
| 1553462_at | - | -2,972500237 | -3,098858316 | 0,12635808 | |
| 1556282_at | FGFR1OP2 | -2,972500237 | -3,098858316 | 0,12635808 | |
| 1562235_s_at | - | -2,972500237 | -3,098858316 | 0,12635808 | |
| 1565834_a_at | - | -2,972500237 | -3,098858316 | 0,12635808 | |
| 1567059_at | - | -2,972500237 | -3,098858316 | 0,12635808 | |
| 1567696_at | - | -2,972500237 | -3,098858316 | 0,12635808 | |
| 1569669_at | FOXR2 | -2,972500237 | -3,098858316 | 0,12635808 | |
| 204463_s_at | EDNRA | -2,972500237 | -3,098858316 | 0,12635808 | |
| 210738_s_at | SLC4A4 | -2,972500237 | -3,098858316 | 0,12635808 | |
| 213458_at | FAM149B1 | -2,972500237 | -3,098858316 | 0,12635808 | |
| 215717_s_at | FBN2 | -2,972500237 | -3,098858316 | 0,12635808 | |
| 237788_at | - | -2,972500237 | -3,098858316 | 0,12635808 | |
| 239301_at | - | -2,972500237 | -3,098858316 | 0,12635808 | |
| 239349_at | C1QTNF7 | -2,972500237 | -3,098858316 | 0,12635808 | |
| 241560_at | - | -2,972500237 | -3,098858316 | 0,12635808 | |
| 243788_at | - | -2,972500237 | -3,098858316 | 0,12635808 | |
| 243998_at | KRT222 | -2,972500237 | -3,098858316 | 0,12635808 | |
| 244094_at | - | -2,972500237 | -3,098858316 | 0,12635808 | |
| 226776_at | ENY2 | 3,010782559 | 2,884425449 | 0,12635711 | |
| 207992_s_at | AMPD3 | 2,159061673 | 2,032717854 | 0,126343819 | |
| 202754_at | R3HDM1 | 4,372199749 | 4,245917359 | 0,126282389 | |
| 218831_s_at | FCGRT | 2,446911392 | 2,320681318 | 0,126230074 | |
| 204395_s_at | GRK5 | 0,503742507 | 0,377530647 | 0,12621186 | |
| 205211_s_at | RIN1 | 0,503742507 | 0,377530647 | 0,12621186 | |
| 211465_x_at | FUT6 | 0,503742507 | 0,377530647 | 0,12621186 | |
| 239307_at | - | 0,503742507 | 0,377530647 | 0,12621186 | |
| 203799_at | CD302 /// LY75-C | 1,326951551 | 1,200746069 | 0,126205481 | |
| 203437_at | TMEM11 | 2,993291442 | 2,867091644 | 0,126199798 | |
| 226316_at | RBM26 | 3,088871381 | 2,962671751 | 0,12619963 | |
| 218665_at | FZD4 | 1,758252436 | 1,632095957 | 0,12615648 | |
| 228002_at | GTPBP4 /// IDI2 | 1,758252436 | 1,632095957 | 0,12615648 | |
| 1553158_at | CEP19 | -0,196756757 | -0,322870281 | 0,126113524 | |
| 207834_at | FBLN1 | -0,196756757 | -0,322870281 | 0,126113524 | |
| 208344_x_at | IFNA1 /// IFNA13 | -0,196756757 | -0,322870281 | 0,126113524 | |
| 211809_x_at | COL13A1 | -0,196756757 | -0,322870281 | 0,126113524 | |
| 213159_at | PCNX | -0,196756757 | -0,322870281 | 0,126113524 | |
| 224169_at | NPFFR2 | -0,196756757 | -0,322870281 | 0,126113524 | |
| 239265_at | SLC35G1 | -0,196756757 | -0,322870281 | 0,126113524 | |
| 244182_at | - | -0,196756757 | -0,322870281 | 0,126113524 | |
| 222206_s_at | NCLN | 2,197169074 | 2,071071048 | 0,126098027 | |
| 1554918_a_at | ABCC4 | 3,359534664 | 3,233443554 | 0,12609111 | |
| 212828_at | SYNJ2 | 2,688444421 | 2,562416132 | 0,126028289 | |
| 202839_s_at | NDUFB7 | 4,919943867 | 4,79394496 | 0,125998908 | |
| 1559942_at | MDFIC | 1,614289682 | 1,488344553 | 0,125945129 | |
| 229553_at | PGM2L1 | 2,807162789 | 2,681234383 | 0,125928406 | |
| 225354_s_at | SH3BGRL2 | 2,535098095 | 2,409170778 | 0,125927317 | |
| 47069_at | PRR5 | 1,957345899 | 1,831449458 | 0,125896441 | |
| 38703_at | DNPEP | 3,910933598 | 3,785040604 | 0,125892995 | |
| 217871_s_at | MIF | 6,815502549 | 6,689618984 | 0,125883565 | |
| 1564207_at | FLJ35390 | 2,422991296 | 2,297144887 | 0,12584641 | |
| 1553789_a_at | C21orf58 | 0,715481152 | 0,589657387 | 0,125823765 | |
| 1562403_a_at | SLC8A3 | 0,715481152 | 0,589657387 | 0,125823765 | |
| 211373_s_at | PSEN2 | 0,715481152 | 0,589657387 | 0,125823765 | |

| | | | | | |
|---|---|---|---|---|---|
| 226633_at | RAB8B | 5,075404701 | 4,949582959 | 0,125821743 | |
| 212428_at | KIAA0368 | 2,896728217 | 2,770953871 | 0,125774345 | |
| 226031_at | CCDC132 | 3,482282213 | 3,356551164 | 0,125731049 | |
| 202776_at | DNTTIP2 | 4,725862305 | 4,600140481 | 0,125721824 | |
| 228745_at | SGTB | 2,333865983 | 2,208167653 | 0,12569833 | |
| 201535_at | UBL3 | 2,132266459 | 2,006570641 | 0,125695818 | |
| 242546_at | FLJ39632 /// LOC | 1,269985355 | 1,144305291 | 0,125680064 | |
| 225610_at | UHRF2 | 2,693452197 | 2,567790814 | 0,125661383 | |
| 1554382_at | LOC200261 | 0,133056859 | 0,007421914 | 0,125634945 | |
| 233475_at | MGC32805 | 0,133056859 | 0,007421914 | 0,125634945 | |
| 229333_at | - | 1,81142822 | 1,685812099 | 0,12561612 | |
| 1565269_s_at | ATF1 | 2,790121796 | 2,664608955 | 0,125512841 | |
| 228711_at | ZNF37A | 1,567778694 | 1,442269454 | 0,12550924 | |
| 221534_at | C11orf68 | 2,456762221 | 2,331254065 | 0,125508156 | |
| 228053_s_at | TOMM5 | 5,482445879 | 5,356985528 | 0,125460352 | |
| 1568983_a_at | - | 3,042527069 | 2,917077481 | 0,125449589 | |
| 218988_at | SLC35E3 | 2,983784569 | 2,858346012 | 0,125438557 | |
| 212034_s_at | EXOC7 | 2,696781084 | 2,571362843 | 0,12541824 | |
| 212129_at | NIPA2 | 4,94360513 | 4,818200317 | 0,125404813 | |
| 217908_s_at | DCAF6 | 4,83676466 | 4,711365298 | 0,125399362 | |
| 205281_s_at | PIGA | 3,50144467 | 3,376085218 | 0,125359452 | |
| 213655_at | - | 5,902791466 | 5,777460041 | 0,125331425 | |
| 227181_at | LNP1 | 1,859746653 | 1,73441697 | 0,125329683 | |
| 202557_at | HSPA13 | 3,838564178 | 3,713295978 | 0,1252682 | |
| 227974_at | - | 1,339784194 | 1,214517879 | 0,125266316 | |
| 239363_at | - | 1,339784194 | 1,214517879 | 0,125266316 | |
| 238622_at | RAP2B | 2,208874124 | 2,083632167 | 0,125241957 | |
| 200718_s_at | SKP1 | 7,109811822 | 6,984595957 | 0,125215865 | |
| 211963_s_at | ARPC5 | 5,144381006 | 5,019240565 | 0,125140441 | |
| 242317_at | HIGD1A | 1,462198073 | 1,337086025 | 0,125112049 | |
| 211417_x_at | GGT1 /// GGT2 // | 0,518867317 | 0,393761504 | 0,125105813 | |
| 218972_at | TTC17 | 0,518867317 | 0,393761504 | 0,125105813 | |
| 219172_at | UBTD1 | 0,518867317 | 0,393761504 | 0,125105813 | |
| 233110_s_at | BCL2L12 | 3,818840142 | 3,693764321 | 0,125075821 | |
| 202568_s_at | MARK3 | 3,462861802 | 3,337798009 | 0,125063793 | |
| 203553_s_at | MAP4K5 | 3,067171967 | 2,942125533 | 0,125046434 | |
| 219481_at | TTC13 | 2,819431021 | 2,694398145 | 0,125032876 | |
| 223526_at | C18orf21 | 3,177319542 | 3,052311115 | 0,125008427 | |
| 1552661_at | PCDHGB7 | -3,013665402 | -3,138626879 | 0,124961477 | |
| 1557883_a_at | - | -3,013665402 | -3,138626879 | 0,124961477 | |
| 1560048_at | - | -3,013665402 | -3,138626879 | 0,124961477 | |
| 1560111_at | - | -3,013665402 | -3,138626879 | 0,124961477 | |
| 1563696_at | HSD17B4 | -3,013665402 | -3,138626879 | 0,124961477 | |
| 1567380_at | - | -3,013665402 | -3,138626879 | 0,124961477 | |
| 1569941_at | - | -3,013665402 | -3,138626879 | 0,124961477 | |
| 1569981_at | - | -3,013665402 | -3,138626879 | 0,124961477 | |
| 1570469_at | - | -3,013665402 | -3,138626879 | 0,124961477 | |
| 210643_at | TNFSF11 | -3,013665402 | -3,138626879 | 0,124961477 | |
| 211119_at | ESR2 | -3,013665402 | -3,138626879 | 0,124961477 | |
| 213172_at | TTC9 | -3,013665402 | -3,138626879 | 0,124961477 | |
| 215619_at | - | -3,013665402 | -3,138626879 | 0,124961477 | |
| 216068_at | - | -3,013665402 | -3,138626879 | 0,124961477 | |
| 231898_x_at | SOX2-OT | -3,013665402 | -3,138626879 | 0,124961477 | |
| 233942_at | LOC613126 | -3,013665402 | -3,138626879 | 0,124961477 | |
| 237690_at | GPR115 | -3,013665402 | -3,138626879 | 0,124961477 | |
| 240713_s_at | - | -3,013665402 | -3,138626879 | 0,124961477 | |

| | | | | | |
|---|---|---|---|---|---|
| 241782_at | NEBL | -3,013665402 | -3,138626879 | 0,124961477 | |
| 243165_at | BTF3 | -3,013665402 | -3,138626879 | 0,124961477 | |
| 243605_at | - | -3,013665402 | -3,138626879 | 0,124961477 | |
| 243878_at | - | -3,013665402 | -3,138626879 | 0,124961477 | |
| 208776_at | PSMD11 | 2,247975581 | 2,123108223 | 0,124867358 | |
| 211702_s_at | USP32 | 2,577249059 | 2,452428893 | 0,124820165 | |
| 227374_at | EARS2 | 2,14450783 | 2,019703483 | 0,124804347 | |
| 1556015_a_at | MESP2 | 1,215326747 | 1,090553537 | 0,12477321 | |
| 235154_at | TAF3 | 1,215326747 | 1,090553537 | 0,12477321 | |
| 200983_x_at | CD59 | 4,631170853 | 4,506456081 | 0,124714773 | |
| 204522_at | DOM3Z | 0,911589958 | 0,786897721 | 0,124692237 | |
| 202354_s_at | GTF2F1 | 2,28383338 | 2,159161028 | 0,124672352 | |
| 232553_at | PCYT1B | 1,732533761 | 1,60795124 | 0,124582521 | |
| 65521_at | UBE2D4 | 1,870104004 | 1,745526308 | 0,124577696 | |
| 205300_s_at | SNRNP35 | 1,073999308 | 0,949447784 | 0,124551524 | |
| 216486_x_at | ZNF79 | 1,073999308 | 0,949447784 | 0,124551524 | |
| 230083_at | USP53 | 1,073999308 | 0,949447784 | 0,124551524 | |
| 202933_s_at | YES1 | 4,540517622 | 4,41598058 | 0,124537042 | |
| 209458_x_at | HBA1 /// HBA2 | 5,384696241 | 5,260159446 | 0,124536794 | |
| 209408_at | KIF2C | 4,32026764 | 4,195885363 | 0,124382278 | |
| 31637_s_at | NR1D1 /// THRA | 2,271607525 | 2,147243253 | 0,124364272 | |
| 231865_at | NCKAP5L | 0,82905153 | 0,704715219 | 0,124336311 | |
| 226524_at | C3orf38 | 3,115233629 | 2,990953566 | 0,124280063 | |
| 1560878_at | SYT15 | 1,874520207 | 1,750261389 | 0,124258818 | |
| 207105_s_at | PIK3R2 | 0,152582607 | 0,028364014 | 0,124218593 | |
| 239452_at | - | 0,152582607 | 0,028364014 | 0,124218593 | |
| 214943_s_at | ARID4B /// RBM3 | 1,960118853 | 1,835911187 | 0,124207667 | |
| 242356_at | VTI1A | 1,58584542 | 1,461646549 | 0,124198871 | |
| 1553750_a_at | FAM76B | -0,366776884 | -0,490927335 | 0,124150451 | |
| 205555_s_at | MSX2 | -0,366776884 | -0,490927335 | 0,124150451 | |
| 207255_at | LEPR | -0,366776884 | -0,490927335 | 0,124150451 | |
| 211266_s_at | GPR4 | -0,366776884 | -0,490927335 | 0,124150451 | |
| 228279_s_at | - | -0,366776884 | -0,490927335 | 0,124150451 | |
| 232211_at | PPP1R3F | -0,366776884 | -0,490927335 | 0,124150451 | |
| 233422_at | - | -0,366776884 | -0,490927335 | 0,124150451 | |
| 239599_at | - | -0,366776884 | -0,490927335 | 0,124150451 | |
| 242503_at | CHST13 | -0,366776884 | -0,490927335 | 0,124150451 | |
| 244202_at | - | -0,366776884 | -0,490927335 | 0,124150451 | |
| 244890_at | - | -0,366776884 | -0,490927335 | 0,124150451 | |
| 202470_s_at | CPSF6 | 1,153678675 | 1,029536893 | 0,124141782 | |
| 209067_s_at | HNRPDL | 5,820687187 | 5,696563542 | 0,124123645 | |
| 207020_at | HSF2BP | 0,417596971 | 0,293518836 | 0,124078135 | |
| 216069_at | PRMT2 | 0,417596971 | 0,293518836 | 0,124078135 | |
| 229437_at | MIR155 /// MIR15 | 0,417596971 | 0,293518836 | 0,124078135 | |
| 40560_at | TBX2 | 0,920159643 | 0,796085967 | 0,124073677 | |
| 236026_at | GPATCH2 | 1,292162421 | 1,16809119 | 0,12407123 | |
| 203919_at | TCEA2 | 2,259277178 | 2,135226207 | 0,12405097 | |
| 232218_at | - | 2,259277178 | 2,135226207 | 0,12405097 | |
| 219329_s_at | C2orf28 | 4,407166241 | 4,283151707 | 0,124014533 | |
| 220310_at | TUBAL3 | 0,641402394 | 0,51742585 | 0,123976544 | |
| 211097_s_at | PBX2 | 1,534677255 | 1,410714371 | 0,123962884 | |
| 213750_at | RSL1D1 | 2,227406889 | 2,103505212 | 0,123901676 | |
| 223501_at | TNFSF13B | 2,227406889 | 2,103505212 | 0,123901676 | |
| 200896_x_at | HDGF | 4,868844325 | 4,74496648 | 0,123877845 | |
| 225180_at | TTC14 | 1,965648824 | 1,841838775 | 0,12381005 | |
| 242263_at | TMED5 | 1,965648824 | 1,841838775 | 0,12381005 | |

| | | | | | |
|---|---|---|---|---|---|
| 223174_at | BTBD10 | 2,945117939 | 2,821324225 | 0,123793714 | |
| 221779_at | MICALL1 | 1,481627361 | 1,3579181 | 0,123709261 | |
| 31845_at | ELF4 | 1,394109935 | 1,270526026 | 0,123583909 | |
| 217927_at | SPCS1 | 6,038200748 | 5,914633633 | 0,123567115 | |
| 1563620_at | BTRC | -3,054236653 | -3,177802286 | 0,123565633 | |
| 216087_at | - | -3,054236653 | -3,177802286 | 0,123565633 | |
| 220884_at | - | -3,054236653 | -3,177802286 | 0,123565633 | |
| 243028_x_at | - | -3,054236653 | -3,177802286 | 0,123565633 | |
| 212877_at | KLC1 | 2,331726354 | 2,208167653 | 0,123558702 | |
| 1558738_at | NOL3 | -0,926850548 | -1,050406615 | 0,123556067 | |
| 1562267_s_at | ZNF709 | -0,926850548 | -1,050406615 | 0,123556067 | |
| 204421_s_at | FGF2 | -0,926850548 | -1,050406615 | 0,123556067 | |
| 206012_at | LEFTY2 | -0,926850548 | -1,050406615 | 0,123556067 | |
| 207548_at | GRM7 | -0,926850548 | -1,050406615 | 0,123556067 | |
| 209013_x_at | TRIO | -0,926850548 | -1,050406615 | 0,123556067 | |
| 209500_x_at | TNFSF12 /// TNF | -0,926850548 | -1,050406615 | 0,123556067 | |
| 213319_s_at | CSDA | -0,926850548 | -1,050406615 | 0,123556067 | |
| 216430_x_at | IGLV1-44 | -0,926850548 | -1,050406615 | 0,123556067 | |
| 216918_s_at | DST /// LOC1006 | -0,926850548 | -1,050406615 | 0,123556067 | |
| 220337_at | NGB | -0,926850548 | -1,050406615 | 0,123556067 | |
| 221680_s_at | ETV7 | -0,926850548 | -1,050406615 | 0,123556067 | |
| 223657_at | FAM167B | -0,926850548 | -1,050406615 | 0,123556067 | |
| 231982_at | C19orf77 | -0,926850548 | -1,050406615 | 0,123556067 | |
| 232133_at | ADAMTS10 | -0,926850548 | -1,050406615 | 0,123556067 | |
| 238400_at | ENDOV | -0,926850548 | -1,050406615 | 0,123556067 | |
| 240200_at | SULT1C2 | -0,926850548 | -1,050406615 | 0,123556067 | |
| 240500_at | - | -0,926850548 | -1,050406615 | 0,123556067 | |
| 244461_at | SPECC1 | -0,926850548 | -1,050406615 | 0,123556067 | |
| 224068_x_at | RBM22 | 3,540875956 | 3,417357537 | 0,123518419 | |
| 204169_at | IMPDH1 | 2,817903185 | 2,694398145 | 0,12350504 | |
| 206412_at | FER | 0,541261317 | 0,417770464 | 0,123490853 | |
| 211965_at | ZFP36L1 | 0,541261317 | 0,417770464 | 0,123490853 | |
| 222349_x_at | RNF126P1 | 0,541261317 | 0,417770464 | 0,123490853 | |
| 222566_at | SUV420H1 | 0,541261317 | 0,417770464 | 0,123490853 | |
| 232312_at | PPP6R3 | 0,541261317 | 0,417770464 | 0,123490853 | |
| 238740_at | AARSD1 /// PTGI | 0,541261317 | 0,417770464 | 0,123490853 | |
| 233852_at | POLH | 0,928678725 | 0,805216064 | 0,123462661 | |
| 235753_at | HOXA7 | 0,928678725 | 0,805216064 | 0,123462661 | |
| 227372_s_at | BAIAP2L1 | 1,649074067 | 1,525647381 | 0,123426686 | |
| 1554930_a_at | FUT8 | 2,365585359 | 2,242180416 | 0,123404943 | |
| 201132_at | HNRNPH2 /// RP | 4,301804532 | 4,17842456 | 0,123379973 | |
| 220651_s_at | MCM10 | 4,192860462 | 4,06960609 | 0,123254372 | |
| 229468_at | CDK3 /// TEN1-C | 1,889144008 | 1,765933685 | 0,123210323 | |
| 237563_s_at | - | 1,889144008 | 1,765933685 | 0,123210323 | |
| 212453_at | KIAA1279 | 2,845159691 | 2,721978587 | 0,123181105 | |
| 226503_at | RIF1 | 2,652894659 | 2,529740643 | 0,123154016 | |
| 212873_at | HMHA1 | 1,369290453 | 1,246149888 | 0,123140564 | |
| 213986_s_at | C19orf6 | 1,238353345 | 1,115234684 | 0,123118661 | |
| 217961_at | SLC25A38 | 2,933877275 | 2,810785576 | 0,123091699 | |
| 201532_at | PSMA3 | 6,354482896 | 6,231410697 | 0,123072199 | |
| 1554239_s_at | ZADH2 | 2,017152618 | 1,894118818 | 0,123033801 | |
| 209969_s_at | STAT1 | 3,602516743 | 3,479511525 | 0,123005218 | |
| 225887_at | PROSER1 | 3,562021138 | 3,439026336 | 0,122994803 | |
| 225284_at | DNAJC3 | 3,32445605 | 3,201528602 | 0,122927448 | |
| 1554899_s_at | FCER1G | -1,964176657 | -2,086984744 | 0,122808087 | |
| 1558888_x_at | ZNF321P /// ZNF | -1,964176657 | -2,086984744 | 0,122808087 | |

| | | | | | |
|---|---|---|---|---|---|
| 1560411_at | - | -1,964176657 | -2,086984744 | 0,122808087 | |
| 1560940_at | SACS-AS1 | -1,964176657 | -2,086984744 | 0,122808087 | |
| 1561232_at | LOC100270680 | -1,964176657 | -2,086984744 | 0,122808087 | |
| 1564472_at | - | -1,964176657 | -2,086984744 | 0,122808087 | |
| 1569203_at | CXCL2 | -1,964176657 | -2,086984744 | 0,122808087 | |
| 202438_x_at | IDS | -1,964176657 | -2,086984744 | 0,122808087 | |
| 204971_at | CSTA | -1,964176657 | -2,086984744 | 0,122808087 | |
| 205485_at | RYR1 | -1,964176657 | -2,086984744 | 0,122808087 | |
| 205525_at | CALD1 | -1,964176657 | -2,086984744 | 0,122808087 | |
| 210032_s_at | SPAG6 | -1,964176657 | -2,086984744 | 0,122808087 | |
| 217163_at | ESR1 | -1,964176657 | -2,086984744 | 0,122808087 | |
| 217478_s_at | HLA-DMA | -1,964176657 | -2,086984744 | 0,122808087 | |
| 221137_at | - | -1,964176657 | -2,086984744 | 0,122808087 | |
| 228781_at | OBFC1 | -1,964176657 | -2,086984744 | 0,122808087 | |
| 232289_at | KCNJ12 | -1,964176657 | -2,086984744 | 0,122808087 | |
| 232696_at | - | -1,964176657 | -2,086984744 | 0,122808087 | |
| 232994_s_at | RGNEF | -1,964176657 | -2,086984744 | 0,122808087 | |
| 233577_at | - | -1,964176657 | -2,086984744 | 0,122808087 | |
| 234408_at | IL17F | -1,964176657 | -2,086984744 | 0,122808087 | |
| 234772_s_at | KRTAP2-2 | -1,964176657 | -2,086984744 | 0,122808087 | |
| 235709_at | GAS2L3 | -1,964176657 | -2,086984744 | 0,122808087 | |
| 239537_at | ST8SIA2 | -1,964176657 | -2,086984744 | 0,122808087 | |
| 241329_s_at | - | -1,964176657 | -2,086984744 | 0,122808087 | |
| 242937_at | FOXK2 | -1,964176657 | -2,086984744 | 0,122808087 | |
| 203209_at | RFC5 | 3,136227209 | 3,013449121 | 0,122778088 | |
| 1554248_at | ZNF638 | 0,020670649 | -0,102100538 | 0,122771187 | |
| 206250_x_at | AVPR1A | 0,020670649 | -0,102100538 | 0,122771187 | |
| 212257_s_at | SMARCA2 | 0,020670649 | -0,102100538 | 0,122771187 | |
| 213261_at | TRANK1 | 0,020670649 | -0,102100538 | 0,122771187 | |
| 238130_at | NFATC2IP | 0,020670649 | -0,102100538 | 0,122771187 | |
| 241237_at | - | 0,020670649 | -0,102100538 | 0,122771187 | |
| 212907_at | SLC30A1 | 3,421207626 | 3,298466943 | 0,122740683 | |
| 1553809_a_at | C9orf71 | -1,838812296 | -1,961547147 | 0,122734851 | |
| 1560010_a_at | SATB2-AS1 | -1,838812296 | -1,961547147 | 0,122734851 | |
| 1561128_at | - | -1,838812296 | -1,961547147 | 0,122734851 | |
| 1563031_at | - | -1,838812296 | -1,961547147 | 0,122734851 | |
| 203700_s_at | DIO2 | -1,838812296 | -1,961547147 | 0,122734851 | |
| 207393_at | HCRTR2 | -1,838812296 | -1,961547147 | 0,122734851 | |
| 213797_at | RSAD2 | -1,838812296 | -1,961547147 | 0,122734851 | |
| 221052_at | TDRKH | -1,838812296 | -1,961547147 | 0,122734851 | |
| 222049_s_at | RBP4 | -1,838812296 | -1,961547147 | 0,122734851 | |
| 222888_at | CCNJ | -1,838812296 | -1,961547147 | 0,122734851 | |
| 223732_at | SLC23A1 | -1,838812296 | -1,961547147 | 0,122734851 | |
| 227108_at | STARD9 | -1,838812296 | -1,961547147 | 0,122734851 | |
| 229781_at | LOC100506725 | -1,838812296 | -1,961547147 | 0,122734851 | |
| 231167_at | - | -1,838812296 | -1,961547147 | 0,122734851 | |
| 231606_at | - | -1,838812296 | -1,961547147 | 0,122734851 | |
| 233157_x_at | CCDC114 | -1,838812296 | -1,961547147 | 0,122734851 | |
| 233865_at | NPAS3 | -1,838812296 | -1,961547147 | 0,122734851 | |
| 234532_at | - | -1,838812296 | -1,961547147 | 0,122734851 | |
| 234913_at | TTTY4 /// TTTY4 | -1,838812296 | -1,961547147 | 0,122734851 | |
| 235995_at | PUM2 | -1,838812296 | -1,961547147 | 0,122734851 | |
| 236938_at | - | -1,838812296 | -1,961547147 | 0,122734851 | |
| 237633_at | - | -1,838812296 | -1,961547147 | 0,122734851 | |
| 238730_at | ARHGEF11 | -1,838812296 | -1,961547147 | 0,122734851 | |
| 241250_at | - | -1,838812296 | -1,961547147 | 0,122734851 | |

| | | | | | |
|---|---|---|---|---|---|
| 242662_at | PCSK6 | -1,838812296 | -1,961547147 | 0,122734851 | |
| 244194_at | ADAM22 | -1,838812296 | -1,961547147 | 0,122734851 | |
| 227607_at | STAMBPL1 | 1,659347917 | 1,536652708 | 0,122695209 | |
| 223020_at | CLPTM1L | 5,109524703 | 4,986839697 | 0,122685006 | |
| 221597_s_at | TMEM208 | 3,722507547 | 3,599822964 | 0,122684583 | |
| 58696_at | EXOSC4 | 4,5960582 | 4,473427782 | 0,122630418 | |
| 218698_at | APIP | 3,807337009 | 3,684723168 | 0,122613841 | |
| 227913_at | EXOSC3 | 0,760715727 | 0,638150376 | 0,12256535 | |
| 228698_at | SOX7 | 4,582512819 | 4,460001797 | 0,122511022 | |
| 224949_at | YIPF5 | 3,102740237 | 2,980244813 | 0,122495425 | |
| 1552306_at | ALG10 | 0,555999837 | 0,433557298 | 0,122442539 | |
| 203860_at | PCCA | 1,177702228 | 1,055274826 | 0,122427402 | |
| 223151_at | DCUN1D5 | 5,713842013 | 5,591420757 | 0,122421257 | |
| 221540_x_at | GTF2H2 /// GTF2 | 3,085065712 | 2,962671751 | 0,122393961 | |
| 213476_x_at | TUBB3 | 5,670056013 | 5,547664017 | 0,122391996 | |
| 229097_at | DIAPH3 | 3,954344268 | 3,831954914 | 0,122389354 | |
| 1553364_at | PNPLA1 | -2,089474779 | -2,211750327 | 0,122275548 | |
| 1555022_at | RGS12 | -2,089474779 | -2,211750327 | 0,122275548 | |
| 1557637_at | - | -2,089474779 | -2,211750327 | 0,122275548 | |
| 1558968_at | - | -2,089474779 | -2,211750327 | 0,122275548 | |
| 1561092_at | - | -2,089474779 | -2,211750327 | 0,122275548 | |
| 1561906_at | - | -2,089474779 | -2,211750327 | 0,122275548 | |
| 1561963_at | MIR4296 | -2,089474779 | -2,211750327 | 0,122275548 | |
| 1565795_at | DUOX1 | -2,089474779 | -2,211750327 | 0,122275548 | |
| 1565830_at | - | -2,089474779 | -2,211750327 | 0,122275548 | |
| 1565838_at | - | -2,089474779 | -2,211750327 | 0,122275548 | |
| 1566142_at | - | -2,089474779 | -2,211750327 | 0,122275548 | |
| 1569596_at | - | -2,089474779 | -2,211750327 | 0,122275548 | |
| 1570541_s_at | - | -2,089474779 | -2,211750327 | 0,122275548 | |
| 207963_at | KIF25-AS1 | -2,089474779 | -2,211750327 | 0,122275548 | |
| 208589_at | TRPC7 | -2,089474779 | -2,211750327 | 0,122275548 | |
| 211463_at | ZIC4 | -2,089474779 | -2,211750327 | 0,122275548 | |
| 213880_at | LGR5 | -2,089474779 | -2,211750327 | 0,122275548 | |
| 215225_s_at | GPR17 | -2,089474779 | -2,211750327 | 0,122275548 | |
| 215472_at | PACRG | -2,089474779 | -2,211750327 | 0,122275548 | |
| 223620_at | GPR34 | -2,089474779 | -2,211750327 | 0,122275548 | |
| 228425_at | LOC654433 | -2,089474779 | -2,211750327 | 0,122275548 | |
| 229975_at | BMPR1B | -2,089474779 | -2,211750327 | 0,122275548 | |
| 235895_at | - | -2,089474779 | -2,211750327 | 0,122275548 | |
| 237671_at | - | -2,089474779 | -2,211750327 | 0,122275548 | |
| 238308_at | LOC100652798 / | -2,089474779 | -2,211750327 | 0,122275548 | |
| 240426_at | FAM47B | -2,089474779 | -2,211750327 | 0,122275548 | |
| 240611_at | - | -2,089474779 | -2,211750327 | 0,122275548 | |
| 241591_at | - | -2,089474779 | -2,211750327 | 0,122275548 | |
| 242232_at | - | -2,089474779 | -2,211750327 | 0,122275548 | |
| 242347_at | - | -2,089474779 | -2,211750327 | 0,122275548 | |
| 218151_x_at | SLC52A2 | 3,63931383 | 3,517040489 | 0,122273341 | |
| 211512_s_at | OGFR | 1,381753566 | 1,259496994 | 0,122256572 | |
| 227549_x_at | ZDHHC24 | 1,381753566 | 1,259496994 | 0,122256572 | |
| 209301_at | CA2 | 5,783402046 | 5,661173565 | 0,122228482 | |
| 241687_at | - | 0,859074518 | 0,73688026 | 0,122194258 | |
| 224660_at | PIGY | 6,031624929 | 5,909439342 | 0,122185587 | |
| 214149_s_at | LOC100652765 | 1,903621061 | 1,781437557 | 0,122183504 | |
| 228999_at | CHD2 | 1,903621061 | 1,781437557 | 0,122183504 | |
| 224465_s_at | WIBG | 2,790121796 | 2,667949403 | 0,122172393 | |
| 220500_s_at | RABL2A /// RABL | 0,181384709 | 0,059218869 | 0,122165841 | |

| | | | | | |
|---|---|---|---|---|---|
| 223923_at | C21orf62 | 0,181384709 | 0,059218869 | 0,122165841 | |
| 239998_at | C10orf53 | 0,181384709 | 0,059218869 | 0,122165841 | |
| 209112_at | CDKN1B | 3,234554895 | 3,112391743 | 0,122163152 | |
| 210074_at | CTSL2 | 2,742596529 | 2,620464265 | 0,122132263 | |
| 225576_at | C6orf72 | 3,431234921 | 3,309203139 | 0,122031782 | |
| 221822_at | CCDC101 | 1,446464021 | 1,324440845 | 0,122023175 | |
| 200056_s_at | C1D | 4,423834624 | 4,301817835 | 0,122016789 | |
| 218056_at | BFAR | 3,27186005 | 3,149904996 | 0,121955054 | |
| 201342_at | SNRPC | 5,90495179 | 5,783054676 | 0,121897114 | |
| 210153_s_at | ME2 | 3,591828571 | 3,469974737 | 0,121853835 | |
| 218197_s_at | OXR1 | 2,794789337 | 2,672945617 | 0,12184372 | |
| 1556172_at | - | -0,136301282 | -0,258114234 | 0,121812952 | |
| 211486_s_at | KCNQ2 | -0,136301282 | -0,258114234 | 0,121812952 | |
| 216344_at | NPHP4 | -0,136301282 | -0,258114234 | 0,121812952 | |
| 220910_at | FRAS1 | -0,136301282 | -0,258114234 | 0,121812952 | |
| 224732_at | CHTF8 | 4,065387235 | 3,943660335 | 0,121726899 | |
| 206136_at | FZD5 | -0,888496123 | -1,010192375 | 0,121696253 | |
| 216627_s_at | B4GALT1 | -0,888496123 | -1,010192375 | 0,121696253 | |
| 221674_s_at | CHRD | -0,888496123 | -1,010192375 | 0,121696253 | |
| 221679_s_at | ABHD6 | -0,888496123 | -1,010192375 | 0,121696253 | |
| 225500_x_at | SCAF1 | -0,888496123 | -1,010192375 | 0,121696253 | |
| 231463_at | CNTD1 | -0,888496123 | -1,010192375 | 0,121696253 | |
| 231602_at | LOC100506231 | -0,888496123 | -1,010192375 | 0,121696253 | |
| 233368_s_at | DNAJC27 | -0,888496123 | -1,010192375 | 0,121696253 | |
| 236330_at | - | -0,888496123 | -1,010192375 | 0,121696253 | |
| 238648_at | - | -0,888496123 | -1,010192375 | 0,121696253 | |
| 244865_at | HAX1 | -0,888496123 | -1,010192375 | 0,121696253 | |
| 215482_s_at | EIF2B4 | 3,899783451 | 3,778101865 | 0,121681587 | |
| 223466_x_at | COL4A3BP | 1,995690482 | 1,874012195 | 0,121678288 | |
| 226762_at | PURB | 3,358484112 | 3,236821511 | 0,121662602 | |
| 218506_x_at | GLYR1 | 3,056846434 | 2,935211251 | 0,121635183 | |
| 200894_s_at | FKBP4 | 4,539590968 | 4,417965868 | 0,121625101 | |
| 223081_at | PHF23 | 3,844579258 | 3,722963534 | 0,121615724 | |
| 212157_at | SDC2 | 1,512180019 | 1,390636111 | 0,121543908 | |
| 1565951_s_at | CHML | 0,95671774 | 0,835239052 | 0,121478688 | |
| 202907_s_at | NBN | 4,506303097 | 4,384854065 | 0,121449032 | |
| 202964_s_at | RFX5 | 1,394109935 | 1,272721751 | 0,121388185 | |
| 218642_s_at | CHCHD7 | 2,544366685 | 2,423075926 | 0,12129076 | |
| 224373_s_at | ND4 | 8,066770579 | 7,945484878 | 0,121285701 | |
| 1557849_at | - | -3,114791413 | -3,236070201 | 0,121278788 | |
| 1558388_a_at | UG0898H09 | -3,114791413 | -3,236070201 | 0,121278788 | |
| 1564015_at | PIEZO2 | -3,114791413 | -3,236070201 | 0,121278788 | |
| 1568871_at | - | -3,114791413 | -3,236070201 | 0,121278788 | |
| 1569873_at | LIPJ | -3,114791413 | -3,236070201 | 0,121278788 | |
| 203991_s_at | KDM6A | -3,114791413 | -3,236070201 | 0,121278788 | |
| 206504_at | CYP24A1 | -3,114791413 | -3,236070201 | 0,121278788 | |
| 208175_s_at | DMP1 | -3,114791413 | -3,236070201 | 0,121278788 | |
| 212915_at | PDZRN3 | -3,114791413 | -3,236070201 | 0,121278788 | |
| 230760_at | ZFY | -3,114791413 | -3,236070201 | 0,121278788 | |
| 244163_at | SEMA3A | -3,114791413 | -3,236070201 | 0,121278788 | |
| 212137_at | LARP1 | 5,686056242 | 5,5647888 | 0,121267441 | |
| 213681_at | CYHR1 | 0,457392764 | 0,336136178 | 0,121256587 | |
| 232291_at | MIR17HG /// MIR | 0,457392764 | 0,336136178 | 0,121256587 | |
| 1553603_s_at | ATL2 | 0,779676066 | 0,658444279 | 0,121231786 | |
| 203909_at | SLC9A6 | 2,827046023 | 2,705818724 | 0,1212273 | |
| 223598_at | RAD23B | 3,545499171 | 3,424287521 | 0,121211649 | |

| | | | | | |
|---|---|---|---|---|---|
| 224582_s_at | NUCKS1 | 3,348994562 | 3,227795991 | 0,121198572 | |
| 226891_at | XXYLT1 | 1,780386218 | 1,659204017 | 0,121182202 | |
| 227133_at | FAM199X | 1,780386218 | 1,659204017 | 0,121182202 | |
| 217902_s_at | HERC2 | 3,121439986 | 3,000258961 | 0,121181026 | |
| 209391_at | DPM2 | 3,024094427 | 2,902971658 | 0,121122769 | |
| 212688_at | PIK3CB | 5,004979787 | 4,883951109 | 0,121028678 | |
| 209249_s_at | GHITM | 5,874316981 | 5,753290379 | 0,121026602 | |
| 212880_at | WDR7 | 2,26825514 | 2,147243253 | 0,121011888 | |
| 213101_s_at | ACTR3 | 6,832200789 | 6,711231616 | 0,120969173 | |
| 1555467_a_at | CELF1 | 2,400710591 | 2,27978304 | 0,120927551 | |
| 223551_at | PKIB | 2,303618506 | 2,182705233 | 0,120913273 | |
| 233467_s_at | TSPAN32 | 0,57782909 | 0,456918297 | 0,120910793 | |
| 208709_s_at | NRD1 | 4,455131038 | 4,334242016 | 0,120889022 | |
| 225578_at | MZT1 | 3,76038475 | 3,639498476 | 0,120886274 | |
| 218222_x_at | ARNT | 0,20027154 | 0,079428135 | 0,120843404 | |
| 219278_at | MAP3K6 | 0,20027154 | 0,079428135 | 0,120843404 | |
| 223515_s_at | COQ3 | 3,620156261 | 3,499335901 | 0,12082036 | |
| 222547_at | MAP4K4 | 1,40228911 | 1,281471383 | 0,120817726 | |
| 210132_at | EFNA3 | 1,201332287 | 1,080561626 | 0,120770661 | |
| 222147_s_at | ACTR5 | 1,201332287 | 1,080561626 | 0,120770661 | |
| 201707_at | PEX19 | 2,127340679 | 2,006570641 | 0,120770038 | |
| 209242_at | PEG3 | 5,426400135 | 5,305636527 | 0,120763608 | |
| 219556_at | C16orf59 | 2,166283906 | 2,045615873 | 0,120668033 | |
| 208078_s_at | SIK1 | 2,688444421 | 2,567790814 | 0,120653607 | |
| 1552312_a_at | MFAP3 | 1,274448124 | 1,153866761 | 0,120581363 | |
| 202697_at | NUDT21 | 4,474655091 | 4,354078399 | 0,120576691 | |
| 226495_at | MAVS | 2,926807124 | 2,806245339 | 0,120561785 | |
| 212030_at | RBM25 | 4,490086298 | 4,369548294 | 0,120538004 | |
| 201408_at | PPP1CB | 2,406821352 | 2,286318242 | 0,120503111 | |
| 201567_s_at | GOLGA4 | 3,556534813 | 3,436090594 | 0,120444218 | |
| 202168_at | TAF9 | 4,521870506 | 4,401504959 | 0,120365547 | |
| 201894_s_at | SSR1 | 4,673180244 | 4,552816898 | 0,120363346 | |
| 205683_x_at | TPSAB1 | 0,792179257 | 0,67181667 | 0,120362587 | |
| 213785_at | IPO9 | 0,792179257 | 0,67181667 | 0,120362587 | |
| 203957_at | E2F6 | 3,599852114 | 3,479511525 | 0,120340589 | |
| 228857_at | GNL1 | 1,69307511 | 1,572742379 | 0,120332731 | |
| 1555783_x_at | PQLC2 | 1,530951994 | 1,410714371 | 0,120237622 | |
| 219758_at | TTC26 | 1,530951994 | 1,410714371 | 0,120237622 | |
| 216954_x_at | ATP5O | 4,11599613 | 3,995764148 | 0,120231981 | |
| 237441_at | - | 2,410880861 | 2,290658654 | 0,120222207 | |
| 201063_at | RCN1 | 3,757996542 | 3,637795954 | 0,120200589 | |
| 204373_s_at | CEP350 | 2,842156496 | 2,721978587 | 0,120177909 | |
| 202963_at | RFX5 | 2,503131904 | 2,382985886 | 0,120146018 | |
| 1554397_s_at | UEVLD | 0,888485433 | 0,768343791 | 0,120141643 | |
| 211375_s_at | ILF3 | 6,519719809 | 6,399582123 | 0,120137686 | |
| 206468_s_at | METTL13 | 2,771299138 | 2,651169275 | 0,120129863 | |
| 1565558_at | - | 1,210676994 | 1,090553537 | 0,120123457 | |
| 228729_at | CCNB1 | 3,933331467 | 3,813221015 | 0,120110451 | |
| 1555417_a_at | TAS1R1 | -1,543331864 | -1,663414495 | 0,12008263 | |
| 1556398_a_at | - | -1,543331864 | -1,663414495 | 0,12008263 | |
| 1561979_at | LOC100505635 | -1,543331864 | -1,663414495 | 0,12008263 | |
| 217512_at | KNG1 | -1,543331864 | -1,663414495 | 0,12008263 | |
| 219516_at | TRPV4 | -1,543331864 | -1,663414495 | 0,12008263 | |
| 221231_s_at | C14orf102 | -1,543331864 | -1,663414495 | 0,12008263 | |
| 225151_at | RTKN | -1,543331864 | -1,663414495 | 0,12008263 | |
| 231230_at | KCNK10 | -1,543331864 | -1,663414495 | 0,12008263 | |

| | | | | | |
|---|---|---|---|---|---|
| 232694_at | ZNF395 | -1,543331864 | -1,663414495 | 0,12008263 | |
| 236397_at | - | -1,543331864 | -1,663414495 | 0,12008263 | |
| 237025_at | - | -1,543331864 | -1,663414495 | 0,12008263 | |
| 239450_at | - | -1,543331864 | -1,663414495 | 0,12008263 | |
| 241515_at | - | -1,543331864 | -1,663414495 | 0,12008263 | |
| 242287_at | CLIP1 | -1,543331864 | -1,663414495 | 0,12008263 | |
| 242799_at | - | -1,543331864 | -1,663414495 | 0,12008263 | |
| 243722_at | PYDC1 | -1,543331864 | -1,663414495 | 0,12008263 | |
| 243831_at | - | -1,543331864 | -1,663414495 | 0,12008263 | |
| 210338_s_at | HSPA8 /// SNOR | 7,720280366 | 7,600227024 | 0,120053342 | |
| 226446_at | HES6 | 2,618203384 | 2,498185745 | 0,120017639 | |
| 200763_s_at | RPLP1 | 7,998907246 | 7,878896491 | 0,120010755 | |
| 235099_at | CMTM8 | 1,414471577 | 1,294497133 | 0,119974445 | |
| 228208_x_at | ZNF354C | 2,646022818 | 2,526063925 | 0,119958893 | |
| 211956_s_at | EIF1 | 7,257729446 | 7,137789285 | 0,119940161 | |
| 1552837_at | ICK | -3,15396682 | -3,273861381 | 0,119894561 | |
| 1552974_at | - | -3,15396682 | -3,273861381 | 0,119894561 | |
| 1553474_at | LOC100288966 / | -3,15396682 | -3,273861381 | 0,119894561 | |
| 1562364_at | GVINP1 | -3,15396682 | -3,273861381 | 0,119894561 | |
| 1569663_at | - | -3,15396682 | -3,273861381 | 0,119894561 | |
| 209909_s_at | TGFB2 | -3,15396682 | -3,273861381 | 0,119894561 | |
| 210999_s_at | GRB10 | 1,699726954 | 1,579853228 | 0,119873726 | |
| 226979_at | MAP3K2 | 3,102740237 | 2,982929462 | 0,119810775 | |
| 218093_s_at | ANKRD10 | 3,043834719 | 2,924079008 | 0,119755711 | |
| 214816_x_at | C19orf40 | 2,320980353 | 2,201267805 | 0,119712548 | |
| 209089_at | RAB5A | 5,231023008 | 5,111373454 | 0,119649554 | |
| 217737_x_at | C20orf43 | 4,288076367 | 4,168435485 | 0,119640882 | |
| 226662_at | STX17 | 1,940595304 | 1,820984782 | 0,119610522 | |
| 204883_s_at | HUS1 | 2,450859799 | 2,331254065 | 0,119605734 | |
| 223748_at | SLC4A11 | 0,984222186 | 0,864649967 | 0,119572218 | |
| 208460_at | GJC1 | -0,299034815 | -0,41858459 | 0,119549775 | |
| 213388_at | LOC728802 /// PI | -0,299034815 | -0,41858459 | 0,119549775 | |
| 214301_s_at | DPYSL4 | -0,299034815 | -0,41858459 | 0,119549775 | |
| 215661_at | MAST2 | -0,299034815 | -0,41858459 | 0,119549775 | |
| 215886_x_at | USP12 | -0,299034815 | -0,41858459 | 0,119549775 | |
| 216555_at | PRR14L | -0,299034815 | -0,41858459 | 0,119549775 | |
| 218523_at | LHPP | -0,299034815 | -0,41858459 | 0,119549775 | |
| 227664_at | FLJ37453 | -0,299034815 | -0,41858459 | 0,119549775 | |
| 232566_at | NOL6 | -0,299034815 | -0,41858459 | 0,119549775 | |
| 236143_at | - | -0,299034815 | -0,41858459 | 0,119549775 | |
| 236392_at | CUL9 | -0,299034815 | -0,41858459 | 0,119549775 | |
| 237307_at | - | -0,299034815 | -0,41858459 | 0,119549775 | |
| 213334_x_at | HAUS7 | 2,254767157 | 2,135226207 | 0,11954095 | |
| 228477_at | ARGLU1 | 3,541801785 | 3,42231092 | 0,119490864 | |
| 208549_x_at | PTMA | 5,843580761 | 5,724103011 | 0,11947775 | |
| 1557984_s_at | RPAP3 | 2,027765132 | 1,908311039 | 0,119454093 | |
| 218858_at | DEPTOR | 1,422536444 | 1,303116084 | 0,11942036 | |
| 201518_at | CBX1 | 4,275330031 | 4,155999779 | 0,119330252 | |
| 204692_at | LRCH4 /// SAP25 | 3,572931593 | 3,453616097 | 0,119315496 | |
| 228597_at | MIS18A | 4,746086726 | 4,626777417 | 0,119309309 | |
| 222119_s_at | FBXO11 | 3,050355245 | 2,93104672 | 0,119308526 | |
| 1555326_a_at | ADAM9 | 1,224581536 | 1,105412836 | 0,1191687 | |
| 211100_x_at | LILRA2 | 0,072842263 | -0,046302147 | 0,11914441 | |
| 223948_s_at | TMPRSS3 | 0,072842263 | -0,046302147 | 0,11914441 | |
| 239203_at | C7orf53 | 0,072842263 | -0,046302147 | 0,11914441 | |
| 244356_at | - | 0,072842263 | -0,046302147 | 0,11914441 | |

| | | | | | |
|---|---|---|---|---|---|
| 205497_at | ZNF175 | 2,947914474 | 2,828804976 | 0,119109498 | |
| 1552362_a_at | LEAP2 | 0,810733188 | 0,691645685 | 0,119087502 | |
| 1558393_at | KRT7 | 0,810733188 | 0,691645685 | 0,119087502 | |
| 226114_at | ZNF436 | 0,810733188 | 0,691645685 | 0,119087502 | |
| 238581_at | GBP5 | 0,810733188 | 0,691645685 | 0,119087502 | |
| 209812_x_at | CASP2 | 2,151803104 | 2,032717854 | 0,11908525 | |
| 204018_x_at | HBA1 /// HBA2 | 5,420120192 | 5,301072115 | 0,119048077 | |
| 1554344_s_at | AQP12A /// AQP1 | 2,190100188 | 2,071071048 | 0,11902914 | |
| 202033_s_at | RB1CC1 | 4,253455927 | 4,13442882 | 0,119027107 | |
| 224782_at | ZMAT2 | 4,279776324 | 4,160749839 | 0,119026485 | |
| 212600_s_at | UQCRC2 | 5,402771752 | 5,28375775 | 0,119014002 | |
| 227935_s_at | PCGF5 | 1,549482847 | 1,43051703 | 0,118965817 | |
| 1558174_at | FAM43A | 0,228146222 | 0,109221392 | 0,118924829 | |
| 1565852_at | - | 0,228146222 | 0,109221392 | 0,118924829 | |
| 229789_at | TIGD3 | 0,228146222 | 0,109221392 | 0,118924829 | |
| 232299_at | C2orf82 | 0,228146222 | 0,109221392 | 0,118924829 | |
| 234841_x_at | OBP2A | 0,228146222 | 0,109221392 | 0,118924829 | |
| 200764_s_at | CTNNA1 | 2,035673598 | 1,916759838 | 0,118913761 | |
| 228127_at | KCNK3 | 0,995078803 | 0,876248481 | 0,118830322 | |
| 77508_r_at | RABEP2 | 0,995078803 | 0,876248481 | 0,118830322 | |
| 1552296_at | BEST4 | -2,384869671 | -2,503677622 | 0,118807951 | |
| 1553894_at | CCDC122 | -2,384869671 | -2,503677622 | 0,118807951 | |
| 1561139_at | - | -2,384869671 | -2,503677622 | 0,118807951 | |
| 1563643_at | - | -2,384869671 | -2,503677622 | 0,118807951 | |
| 204726_at | CDH13 | -2,384869671 | -2,503677622 | 0,118807951 | |
| 207329_at | MMP8 | -2,384869671 | -2,503677622 | 0,118807951 | |
| 217578_at | - | -2,384869671 | -2,503677622 | 0,118807951 | |
| 221092_at | IKZF3 | -2,384869671 | -2,503677622 | 0,118807951 | |
| 221149_at | GPR77 | -2,384869671 | -2,503677622 | 0,118807951 | |
| 224146_s_at | ABCC11 | -2,384869671 | -2,503677622 | 0,118807951 | |
| 229471_s_at | SRSF8 | -2,384869671 | -2,503677622 | 0,118807951 | |
| 230244_at | C2orf82 | -2,384869671 | -2,503677622 | 0,118807951 | |
| 232303_at | ZNF608 | -2,384869671 | -2,503677622 | 0,118807951 | |
| 237139_at | PDE9A | -2,384869671 | -2,503677622 | 0,118807951 | |
| 239693_at | SNX24 | -2,384869671 | -2,503677622 | 0,118807951 | |
| 241864_x_at | - | -2,384869671 | -2,503677622 | 0,118807951 | |
| 243386_at | CASZ1 | -2,384869671 | -2,503677622 | 0,118807951 | |
| 244573_at | - | -2,384869671 | -2,503677622 | 0,118807951 | |
| 244634_at | - | -2,384869671 | -2,503677622 | 0,118807951 | |
| 1553545_at | ILDR1 | -0,83191556 | -0,950686014 | 0,118770455 | |
| 1557688_at | - | -0,83191556 | -0,950686014 | 0,118770455 | |
| 1566102_at | TTLL5 | -0,83191556 | -0,950686014 | 0,118770455 | |
| 1569368_at | LOC283693 | -0,83191556 | -0,950686014 | 0,118770455 | |
| 1569383_s_at | ZFYVE28 | -0,83191556 | -0,950686014 | 0,118770455 | |
| 1570038_at | ZNF595 | -0,83191556 | -0,950686014 | 0,118770455 | |
| 205388_at | TNNC2 | -0,83191556 | -0,950686014 | 0,118770455 | |
| 207687_at | INHBC | -0,83191556 | -0,950686014 | 0,118770455 | |
| 207732_s_at | DLG3 | -0,83191556 | -0,950686014 | 0,118770455 | |
| 210219_at | SP100 | -0,83191556 | -0,950686014 | 0,118770455 | |
| 213914_s_at | SPTBN1 | -0,83191556 | -0,950686014 | 0,118770455 | |
| 228506_at | NSMCE4A | -0,83191556 | -0,950686014 | 0,118770455 | |
| 234721_s_at | CYP26B1 | -0,83191556 | -0,950686014 | 0,118770455 | |
| 240187_at | PPP1R3C | -0,83191556 | -0,950686014 | 0,118770455 | |
| 244859_at | - | -0,83191556 | -0,950686014 | 0,118770455 | |
| 214293_at | 40787 | 1,716223609 | 1,59747851 | 0,118745099 | |
| 1557226_a_at | ASPG | 1,079110292 | 0,960386719 | 0,118723573 | |

| | | | | | |
|---|---|---|---|---|---|
| 1557689_at | - | 1,079110292 | 0,960386719 | 0,118723573 | |
| 218928_s_at | SLC37A1 | 1,079110292 | 0,960386719 | 0,118723573 | |
| 217749_at | COPG1 | 3,231115193 | 3,112391743 | 0,11872345 | |
| 241373_at | - | 1,119359127 | 1,000684521 | 0,118674606 | |
| 1555968_a_at | - | 0,496120222 | 0,377530647 | 0,118589575 | |
| 236301_at | IKZF3 | 0,496120222 | 0,377530647 | 0,118589575 | |
| 223047_at | CMTM6 | 4,111014386 | 3,992437318 | 0,118577068 | |
| 223044_at | SLC40A1 | 6,807778103 | 6,68920807 | 0,118570033 | |
| 1561525_at | - | -3,192553003 | -3,311072652 | 0,118519649 | |
| 1566865_at | FAM200A | -3,192553003 | -3,311072652 | 0,118519649 | |
| 220656_at | - | -3,192553003 | -3,311072652 | 0,118519649 | |
| 203237_s_at | NOTCH3 | 0,613492853 | 0,495031851 | 0,118461002 | |
| 203626_s_at | SKP2 | 0,613492853 | 0,495031851 | 0,118461002 | |
| 223048_at | SDHAF2 | 3,199783157 | 3,081412119 | 0,118371038 | |
| 233124_s_at | ECHDC1 | 4,218563327 | 4,100219485 | 0,118343842 | |
| 214246_x_at | MINK1 | 3,002736078 | 2,884425449 | 0,11831063 | |
| 228728_at | CPED1 | 2,438982012 | 2,320681318 | 0,118300694 | |
| 211325_x_at | DSTNP2 | 1,960118853 | 1,841838775 | 0,118280079 | |
| 200010_at | RPL11 | 7,523253047 | 7,404986363 | 0,118266684 | |
| 242583_at | STON2 | 0,822971223 | 0,704715219 | 0,118256004 | |
| 211340_s_at | MCAM | 4,88279496 | 4,764601839 | 0,118193121 | |
| 218439_s_at | COMMD10 | 3,046446467 | 2,928263672 | 0,118182795 | |
| 203175_at | RHOG | 3,674351634 | 3,556324192 | 0,118027442 | |
| 232909_s_at | BPTF /// LOC146 | 2,277177578 | 2,159161028 | 0,118016551 | |
| 212265_at | QKI | 5,563544186 | 5,445531846 | 0,11801234 | |
| 225674_at | BCAP29 | 2,089851283 | 1,971855016 | 0,117996267 | |
| 227110_at | HNRNPC /// LOC | 4,007586951 | 3,889604025 | 0,117982926 | |
| 225834_at | FAM72A /// FAM7 | 5,360237789 | 5,242343956 | 0,117893833 | |
| 1552338_at | GSC | -1,424023738 | -1,541908042 | 0,117884304 | |
| 1555203_s_at | SLC44A4 | -1,424023738 | -1,541908042 | 0,117884304 | |
| 1561221_x_at | LOC728099 | -1,424023738 | -1,541908042 | 0,117884304 | |
| 202796_at | SYNPO | -1,424023738 | -1,541908042 | 0,117884304 | |
| 204007_at | FCGR3B | -1,424023738 | -1,541908042 | 0,117884304 | |
| 205143_at | NCAN | -1,424023738 | -1,541908042 | 0,117884304 | |
| 205376_at | INPP4B | -1,424023738 | -1,541908042 | 0,117884304 | |
| 210731_s_at | LGALS8 | -1,424023738 | -1,541908042 | 0,117884304 | |
| 210876_at | ANXA2P1 | -1,424023738 | -1,541908042 | 0,117884304 | |
| 211347_at | CDC14B | -1,424023738 | -1,541908042 | 0,117884304 | |
| 212831_at | MEGF9 | -1,424023738 | -1,541908042 | 0,117884304 | |
| 233735_at | - | -1,424023738 | -1,541908042 | 0,117884304 | |
| 236405_at | FBXO41 | -1,424023738 | -1,541908042 | 0,117884304 | |
| 237612_at | - | -1,424023738 | -1,541908042 | 0,117884304 | |
| 237940_s_at | - | -1,424023738 | -1,541908042 | 0,117884304 | |
| 238292_at | - | -1,424023738 | -1,541908042 | 0,117884304 | |
| 239387_at | - | -1,424023738 | -1,541908042 | 0,117884304 | |
| 239719_at | CD109 | -1,424023738 | -1,541908042 | 0,117884304 | |
| 241085_at | - | -1,424023738 | -1,541908042 | 0,117884304 | |
| 242963_at | SGMS2 | -1,424023738 | -1,541908042 | 0,117884304 | |
| 202130_at | RIOK3 | 4,288076367 | 4,170203296 | 0,117873071 | |
| 207507_s_at | ATP5G3 | 6,592619964 | 6,474761006 | 0,117858958 | |
| 1565604_at | PWP2 | -0,078265071 | -0,19610998 | 0,117844909 | |
| 214797_s_at | CDK18 | -0,078265071 | -0,19610998 | 0,117844909 | |
| 229243_at | - | -0,078265071 | -0,19610998 | 0,117844909 | |
| 234960_at | HIST1H4A /// HIS | -0,078265071 | -0,19610998 | 0,117844909 | |
| 243531_at | ORAOV1 | -0,078265071 | -0,19610998 | 0,117844909 | |
| 237561_x_at | - | 1,011212064 | 0,893473268 | 0,117738796 | |

| | | | | | |
|---|---|---|---|---|---|
| 223886_s_at | RNF146 | 3,58196103 | 3,464222256 | 0,117738774 | |
| 201828_x_at | FAM127A | 3,22766727 | 3,109937707 | 0,117729563 | |
| 235394_at | PLAA | 1,567778694 | 1,450051551 | 0,117727143 | |
| 231947_at | MYCT1 | 3,818840142 | 3,701119714 | 0,117720427 | |
| 219644_at | CCDC41 | 1,62481334 | 1,507116528 | 0,117696812 | |
| 222689_at | ACER3 | 1,094335495 | 0,976641161 | 0,117694334 | |
| 1552375_at | ZNF333 | 0,246434561 | 0,128747141 | 0,11768742 | |
| 204002_s_at | ICA1 | 0,246434561 | 0,128747141 | 0,11768742 | |
| 222317_at | PDE3B | 0,246434561 | 0,128747141 | 0,11768742 | |
| 224574_at | C17orf49 /// RNA | 3,568395593 | 3,450709925 | 0,117685669 | |
| 232524_x_at | ANAPC4 | 3,50998543 | 3,392332256 | 0,117653175 | |
| 218633_x_at | ABHD10 | 3,525045272 | 3,407399455 | 0,117645818 | |
| 1559490_at | LRCH3 | 0,384950573 | 0,26733076 | 0,117619814 | |
| 232392_at | SRSF3 | 0,384950573 | 0,26733076 | 0,117619814 | |
| 219401_at | XYLT2 | 1,318332599 | 1,200746069 | 0,11758653 | |
| 213184_at | SENP5 | 2,734521183 | 2,617011906 | 0,117509277 | |
| 200079_s_at | KARS | 5,388046388 | 5,270634231 | 0,117412157 | |
| 218206_x_at | SCAND1 | 4,268634793 | 4,151234028 | 0,117400765 | |
| 208707_at | EIF5 | 1,835789711 | 1,718396606 | 0,117393105 | |
| 214739_at | LRCH3 | 1,835789711 | 1,718396606 | 0,117393105 | |
| 1558814_s_at | TMED5 | -0,496991414 | -0,614371577 | 0,117380163 | |
| 1563048_at | - | -0,496991414 | -0,614371577 | 0,117380163 | |
| 202440_s_at | ST5 | -0,496991414 | -0,614371577 | 0,117380163 | |
| 204960_at | PTPRCAP | -0,496991414 | -0,614371577 | 0,117380163 | |
| 206828_at | TXK | -0,496991414 | -0,614371577 | 0,117380163 | |
| 209948_at | KCNMB1 | -0,496991414 | -0,614371577 | 0,117380163 | |
| 220763_at | HSPA12A | -0,496991414 | -0,614371577 | 0,117380163 | |
| 236770_at | TPTE2P5 | -0,496991414 | -0,614371577 | 0,117380163 | |
| 238499_at | SLC45A3 | -0,496991414 | -0,614371577 | 0,117380163 | |
| 239189_at | CASKIN1 | -0,496991414 | -0,614371577 | 0,117380163 | |
| 241696_at | CNTLN | -0,496991414 | -0,614371577 | 0,117380163 | |
| 227294_at | ZNF689 | 2,420979937 | 2,303602088 | 0,117377849 | |
| 229119_s_at | ZSWIM7 | 2,763383302 | 2,646096941 | 0,117286361 | |
| 200614_at | CLTC | 6,832957376 | 6,715676968 | 0,117280407 | |
| 235334_at | ST6GALNAC3 | 1,454352496 | 1,337086025 | 0,117266472 | |
| 242110_at | - | 1,454352496 | 1,337086025 | 0,117266472 | |
| 215722_s_at | SNRPA1 | 3,121439986 | 3,004228687 | 0,1172113 | |
| 121_at | PAX8 | 1,862713491 | 1,745526308 | 0,117187183 | |
| 223113_at | TMEM138 | 3,659926886 | 3,542741726 | 0,11718516 | |
| 223647_x_at | HSCB | 2,392522438 | 2,275409734 | 0,117112703 | |
| 218671_s_at | ATPIF1 | 3,988699475 | 3,871608557 | 0,117090918 | |
| 203747_at | AQP3 | 2,605861638 | 2,488772006 | 0,117089632 | |
| 201239_s_at | LOC653566 /// SI | 5,119171181 | 5,002138926 | 0,117032255 | |
| 1554014_at | CHD2 | 1,021868205 | 0,904843258 | 0,117024947 | |
| 223442_at | NICN1 | 1,578645857 | 1,461646549 | 0,116999307 | |
| 218034_at | FIS1 | 3,729838025 | 3,6128809 | 0,116957126 | |
| 1558081_at | SNORA65 | -0,79540287 | -0,912331589 | 0,116928719 | |
| 1561400_at | - | -0,79540287 | -0,912331589 | 0,116928719 | |
| 206801_at | NPPB | -0,79540287 | -0,912331589 | 0,116928719 | |
| 208443_x_at | SHOX2 | -0,79540287 | -0,912331589 | 0,116928719 | |
| 213868_s_at | DHRS7 | -0,79540287 | -0,912331589 | 0,116928719 | |
| 215291_at | MKL1 | -0,79540287 | -0,912331589 | 0,116928719 | |
| 223904_at | PRKAG3 | -0,79540287 | -0,912331589 | 0,116928719 | |
| 226646_at | KLF2 | -0,79540287 | -0,912331589 | 0,116928719 | |
| 228227_at | ITGB1BP1 | -0,79540287 | -0,912331589 | 0,116928719 | |
| 229142_s_at | BBS1 | -0,79540287 | -0,912331589 | 0,116928719 | |

| | | | | | |
|---|---|---|---|---|---|
| 233597_at | PNPLA5 | -0,79540287 | -0,912331589 | 0,116928719 | |
| 238931_at | METTL16 | -0,79540287 | -0,912331589 | 0,116928719 | |
| 242946_at | CD53 | -0,79540287 | -0,912331589 | 0,116928719 | |
| 228135_at | C1orf52 | 4,13575284 | 4,018839599 | 0,116913241 | |
| 222601_at | UBA6 | 3,506195778 | 3,38929984 | 0,116895937 | |
| 202862_at | FAH | 2,819431021 | 2,702564916 | 0,116866105 | |
| 219055_at | SRBD1 | 2,609398637 | 2,492544876 | 0,116853762 | |
| 201041_s_at | DUSP1 | 2,259277178 | 2,14244844 | 0,116828738 | |
| 227731_at | CNBP | 1,937784532 | 1,820984782 | 0,11679975 | |
| 202599_s_at | NRIP1 | 3,492853048 | 3,376085218 | 0,11676783 | |
| 62987_r_at | CACNG4 | 3,904109866 | 3,787346121 | 0,116763745 | |
| 1555192_at | ZNF277 | -2,504600601 | -2,621331989 | 0,116731388 | |
| 1560864_at | - | -2,504600601 | -2,621331989 | 0,116731388 | |
| 1561274_at | - | -2,504600601 | -2,621331989 | 0,116731388 | |
| 1562803_at | LRRC37A5P | -2,504600601 | -2,621331989 | 0,116731388 | |
| 1562873_at | PWRN1 | -2,504600601 | -2,621331989 | 0,116731388 | |
| 1564241_at | ATP1A4 | -2,504600601 | -2,621331989 | 0,116731388 | |
| 1566666_at | - | -2,504600601 | -2,621331989 | 0,116731388 | |
| 1568897_at | - | -2,504600601 | -2,621331989 | 0,116731388 | |
| 207161_at | KIAA0087 | -2,504600601 | -2,621331989 | 0,116731388 | |
| 207550_at | MPL | -2,504600601 | -2,621331989 | 0,116731388 | |
| 208147_s_at | CYP2C8 | -2,504600601 | -2,621331989 | 0,116731388 | |
| 209493_at | PDZD2 | -2,504600601 | -2,621331989 | 0,116731388 | |
| 221040_at | CAPN10 | -2,504600601 | -2,621331989 | 0,116731388 | |
| 222455_s_at | PARVA | -2,504600601 | -2,621331989 | 0,116731388 | |
| 229045_at | SNX20 | -2,504600601 | -2,621331989 | 0,116731388 | |
| 231544_s_at | POLR3G | -2,504600601 | -2,621331989 | 0,116731388 | |
| 232741_at | - | -2,504600601 | -2,621331989 | 0,116731388 | |
| 234504_at | SUN5 | -2,504600601 | -2,621331989 | 0,116731388 | |
| 235957_at | GRIP1 | -2,504600601 | -2,621331989 | 0,116731388 | |
| 236010_at | - | -2,504600601 | -2,621331989 | 0,116731388 | |
| 236712_at | - | -2,504600601 | -2,621331989 | 0,116731388 | |
| 236740_at | - | -2,504600601 | -2,621331989 | 0,116731388 | |
| 238162_at | - | -2,504600601 | -2,621331989 | 0,116731388 | |
| 238748_at | RAD18 | -2,504600601 | -2,621331989 | 0,116731388 | |
| 240645_at | - | -2,504600601 | -2,621331989 | 0,116731388 | |
| 243236_at | - | -2,504600601 | -2,621331989 | 0,116731388 | |
| 244739_at | RDX | -2,504600601 | -2,621331989 | 0,116731388 | |
| 244854_at | - | -2,504600601 | -2,621331989 | 0,116731388 | |
| 242557_at | ZNRD1-AS1 | 1,331241794 | 1,214517879 | 0,116723915 | |
| 204336_s_at | RGS19 | 3,69530589 | 3,578681277 | 0,116624613 | |
| 222104_x_at | GTF2H3 | 3,668429488 | 3,551810897 | 0,116618591 | |
| 201969_at | NASP | 2,87191078 | 2,755336312 | 0,116574469 | |
| 210008_s_at | MRPS12 | 3,997499421 | 3,880994209 | 0,116505212 | |
| 218740_s_at | CDK5RAP3 | 3,171349466 | 3,05486507 | 0,116484397 | |
| 226631_at | METTL10 | 2,369762403 | 2,253342112 | 0,116420291 | |
| 206632_s_at | APOBEC3B | 4,506303097 | 4,389920115 | 0,116382982 | |
| 218839_at | HEY1 | 6,765999734 | 6,649627688 | 0,116372046 | |
| 221504_s_at | ATP6V1H | 3,815781597 | 3,69948842 | 0,116293176 | |
| 37860_at | ZNF337 | 1,560488174 | 1,444218917 | 0,116269257 | |
| 210235_s_at | PPFIA1 | 1,339784194 | 1,223626587 | 0,116157607 | |
| 223444_at | SENP7 | 1,339784194 | 1,223626587 | 0,116157607 | |
| 210886_x_at | TP53TG1 | 0,648296226 | 0,532164371 | 0,116131855 | |
| 237110_at | - | 0,648296226 | 0,532164371 | 0,116131855 | |
| 241731_x_at | ZNF440 | 3,340506684 | 3,22439681 | 0,116109874 | |
| 216320_x_at | MST1 | 0,533835206 | 0,417770464 | 0,116064743 | |

| | | | | | |
|---|---|---|---|---|---|
| 203214_x_at | CDK1 | 3,472604685 | 3,356551164 | 0,116053521 | |
| 202375_at | SEC24D | 2,342392905 | 2,226407528 | 0,115985377 | |
| 209093_s_at | GBA /// GBAP1 | 1,037706275 | 0,921731975 | 0,1159743 | |
| 55616_at | PGAP3 | 0,995078803 | 0,879133598 | 0,115945205 | |
| 212302_at | RTF1 | 2,59519831 | 2,479296438 | 0,115901872 | |
| 230282_at | TSPAN3 | 0,273439642 | 0,157549243 | 0,115890399 | |
| 230676_s_at | TMEM19 | 0,273439642 | 0,157549243 | 0,115890399 | |
| 235645_at | ESCO1 | 0,273439642 | 0,157549243 | 0,115890399 | |
| 217317_s_at | HERC2P2 /// HEF | 4,070528765 | 3,954643074 | 0,115885691 | |
| 214328_s_at | HSP90AA1 | 7,834857791 | 7,719013403 | 0,115844388 | |
| 222533_at | CRBN | 3,237986416 | 3,122166354 | 0,115820062 | |
| 235056_at | ETV6 | 1,859746653 | 1,743944487 | 0,115802165 | |
| 201521_s_at | NCBP2 | 3,102740237 | 2,986947093 | 0,115793144 | |
| 215440_s_at | BEX4 | 4,632475172 | 4,516682156 | 0,115793016 | |
| 224919_at | MRPS6 | 4,293033569 | 4,177252958 | 0,115780611 | |
| 201322_at | ATP5B | 6,43305539 | 6,317280805 | 0,115774586 | |
| 1564413_at | FLJ36116 | 0,123193849 | 0,007421914 | 0,115771936 | |
| 215683_at | LOC100506070 | 0,123193849 | 0,007421914 | 0,115771936 | |
| 227360_at | RDH13 | 0,123193849 | 0,007421914 | 0,115771936 | |
| 226128_at | BROX | 3,523171367 | 3,407399455 | 0,115771912 | |
| 212168_at | RBM12 | 4,618940011 | 4,50318707 | 0,115752942 | |
| 210371_s_at | RBBP4 | 5,481963596 | 5,366268691 | 0,115694905 | |
| 201022_s_at | DSTN | 6,091495221 | 5,975801468 | 0,115693753 | |
| 212711_at | CAMSAP1 | 2,446911392 | 2,331254065 | 0,115657327 | |
| 203396_at | PSMA4 | 6,005521189 | 5,889993621 | 0,115527568 | |
| 202233_s_at | UQCRH /// UQCF | 6,983562613 | 6,868035364 | 0,115527249 | |
| 202659_at | PSMB10 | 1,912238072 | 1,796776588 | 0,115461484 | |
| 202798_at | SEC24B | 4,261908338 | 4,146452482 | 0,115455857 | |
| 222728_s_at | MIR1304 /// SNO | 3,828735867 | 3,713295978 | 0,115439889 | |
| 204576_s_at | CLUAP1 | 2,281618189 | 2,166264721 | 0,115353468 | |
| 1568639_a_at | LOC100506995 | -0,234278768 | -0,34958478 | 0,115306013 | |
| 204595_s_at | STC1 | -0,234278768 | -0,34958478 | 0,115306013 | |
| 206241_at | KPNA5 | -0,234278768 | -0,34958478 | 0,115306013 | |
| 211082_x_at | MARK2 | -0,234278768 | -0,34958478 | 0,115306013 | |
| 211631_x_at | B4GALT1 | -0,234278768 | -0,34958478 | 0,115306013 | |
| 214671_s_at | ABR | -0,234278768 | -0,34958478 | 0,115306013 | |
| 224198_at | CELA1 | -0,234278768 | -0,34958478 | 0,115306013 | |
| 229237_s_at | - | -0,234278768 | -0,34958478 | 0,115306013 | |
| 229930_at | LOC100134361 | -0,234278768 | -0,34958478 | 0,115306013 | |
| 230428_at | - | -0,234278768 | -0,34958478 | 0,115306013 | |
| 244625_at | - | -0,234278768 | -0,34958478 | 0,115306013 | |
| 1554128_at | ADIG | 1,04816921 | 0,932882274 | 0,115286936 | |
| 232780_s_at | ZNF691 | 1,04816921 | 0,932882274 | 0,115286936 | |
| 226626_at | THOC2 | 3,561108198 | 3,445853256 | 0,115254942 | |
| 223013_at | TBL1XR1 | 5,288138786 | 5,172917458 | 0,115221327 | |
| 1556062_at | RPP30 | -1,308181863 | -1,423348341 | 0,115166478 | |
| 1561042_at | LOC286109 | -1,308181863 | -1,423348341 | 0,115166478 | |
| 201510_at | ELF3 | -1,308181863 | -1,423348341 | 0,115166478 | |
| 202994_s_at | FBLN1 | -1,308181863 | -1,423348341 | 0,115166478 | |
| 210518_at | CDH8 | -1,308181863 | -1,423348341 | 0,115166478 | |
| 221403_s_at | INSL6 | -1,308181863 | -1,423348341 | 0,115166478 | |
| 224551_s_at | SPTBN4 | -1,308181863 | -1,423348341 | 0,115166478 | |
| 234670_at | - | -1,308181863 | -1,423348341 | 0,115166478 | |
| 235975_at | MTO1 | -1,308181863 | -1,423348341 | 0,115166478 | |
| 241768_at | - | -1,308181863 | -1,423348341 | 0,115166478 | |
| 224844_at | SLAIN2 | 2,796341835 | 2,681234383 | 0,115107452 | |

| | | | | | |
|---|---|---|---|---|---|
| 225264_at | RARS2 | 4,585658616 | 4,470561285 | 0,115097331 | |
| 229790_at | TERF2 | 2,051361651 | 1,936283387 | 0,115078264 | |
| 214045_at | LIAS | 2,846658948 | 2,731588328 | 0,11507062 | |
| 1552411_at | DEFB106A /// DE | 0,87091093 | 0,755840599 | 0,115070331 | |
| 225193_at | KIAA1967 | 0,87091093 | 0,755840599 | 0,115070331 | |
| 200677_at | PTTG1IP | 6,553277031 | 6,43824208 | 0,115034951 | |
| 219691_at | SAMD9 | 2,009141686 | 1,894118818 | 0,115022868 | |
| 202818_s_at | TCEB3 | 2,134723057 | 2,019703483 | 0,115019574 | |
| 218909_at | RPS6KC1 | 2,134723057 | 2,019703483 | 0,115019574 | |
| 201263_at | TARS | 5,282610972 | 5,167617512 | 0,114993461 | |
| 243436_at | - | -3,287237186 | -3,402185466 | 0,11494828 | |
| 1553304_at | LSM14B | 0,425644434 | 0,310717132 | 0,114927302 | |
| 1562081_a_at | LINC00424 | 0,425644434 | 0,310717132 | 0,114927302 | |
| 204657_s_at | SHB | 0,425644434 | 0,310717132 | 0,114927302 | |
| 211231_x_at | CYP4A11 | 0,425644434 | 0,310717132 | 0,114927302 | |
| 222463_s_at | BACE1 | 0,425644434 | 0,310717132 | 0,114927302 | |
| 201857_at | ZFR | 5,513217422 | 5,398297283 | 0,114920139 | |
| 219753_at | STAG3 | 1,774096855 | 1,659204017 | 0,114892839 | |
| 201657_at | ARL1 | 2,898174856 | 2,783327323 | 0,114847532 | |
| 221219_s_at | KLHDC4 /// LOC1 | 3,097712429 | 2,982929462 | 0,114782967 | |
| 203497_at | MED1 | 3,899783451 | 3,785040604 | 0,114742848 | |
| 213171_s_at | MMP24 | 0,291166226 | 0,176436073 | 0,114730152 | |
| 220195_at | MBD5 | 0,291166226 | 0,176436073 | 0,114730152 | |
| 220370_s_at | USP36 | 0,291166226 | 0,176436073 | 0,114730152 | |
| 236692_at | - | 0,291166226 | 0,176436073 | 0,114730152 | |
| 226628_at | THOC2 | 2,429008604 | 2,314300277 | 0,114708327 | |
| 218363_at | EXD2 | 1,493160564 | 1,378453643 | 0,114706921 | |
| 207181_s_at | CASP7 | 2,139623739 | 2,024923328 | 0,114700411 | |
| 227793_at | MIRLET7D | 1,292162421 | 1,177496821 | 0,1146656 | |
| 218956_s_at | ATP5J2-PTCD1 / | 2,292660284 | 2,17802703 | 0,114633254 | |
| 202497_x_at | SLC2A3 | 5,560121297 | 5,445531846 | 0,114589452 | |
| 222608_s_at | ANLN | 4,209852334 | 4,09526554 | 0,114586794 | |
| 241484_x_at | - | 1,556829052 | 1,442269454 | 0,114559598 | |
| 229563_s_at | RPL10A | 7,938674672 | 7,824134717 | 0,114539955 | |
| 238156_at | - | 3,182077878 | 3,067567462 | 0,114510416 | |
| 219363_s_at | MTERFD1 | 4,611022217 | 4,496626742 | 0,114395475 | |
| 635_s_at | PPP2R5B | 0,55966116 | 0,445285082 | 0,114376078 | |
| 201483_s_at | SUPT4H1 | 4,279221286 | 4,164893351 | 0,114327935 | |
| 228114_x_at | C16orf13 | 1,67631007 | 1,562009954 | 0,114300116 | |
| 1561937_x_at | - | 1,063722698 | 0,949447784 | 0,114274914 | |
| 227531_at | CLOCK | 1,063722698 | 0,949447784 | 0,114274914 | |
| 200685_at | SRSF11 | 2,333865983 | 2,21959457 | 0,114271413 | |
| 226825_s_at | TMEM165 | 4,205184875 | 4,090916839 | 0,114268035 | |
| 225320_at | MCU | 2,705069849 | 2,590852465 | 0,114217385 | |
| 224523_s_at | C3orf26 | 4,718920906 | 4,604723207 | 0,114197699 | |
| 1558140_at | PLXNA1 | -0,022466681 | -0,136643983 | 0,114177302 | |
| 206513_at | AIM2 | -0,022466681 | -0,136643983 | 0,114177302 | |
| 208044_s_at | PPARD | -0,022466681 | -0,136643983 | 0,114177302 | |
| 240826_at | - | -0,022466681 | -0,136643983 | 0,114177302 | |
| 242788_at | KDM4D | -0,022466681 | -0,136643983 | 0,114177302 | |
| 1555257_a_at | MYO3B | -0,741668563 | -0,855751026 | 0,114082463 | |
| 215961_at | F12 | -0,741668563 | -0,855751026 | 0,114082463 | |
| 217004_s_at | MCF2 | -0,741668563 | -0,855751026 | 0,114082463 | |
| 224483_s_at | MFSD9 | -0,741668563 | -0,855751026 | 0,114082463 | |
| 232672_x_at | SLC24A5 | -0,741668563 | -0,855751026 | 0,114082463 | |
| 237558_at | - | -0,741668563 | -0,855751026 | 0,114082463 | |

| | | | | | |
|---|---|---|---|---|---|
| 236978_at | - | 1,504602299 | 1,390636111 | 0,113966188 | |
| 238477_at | KIF1C | 2,303618506 | 2,189694216 | 0,11392429 | |
| 207455_at | P2RY1 | 0,682279745 | 0,568365095 | 0,11391465 | |
| 202983_at | HLTF | 5,074765177 | 4,960861351 | 0,113903826 | |
| 224830_at | NUDT21 | 5,434648023 | 5,320836197 | 0,113811826 | |
| 225710_at | GNB4 | 5,107650169 | 4,99384426 | 0,113805909 | |
| 221934_s_at | DALRD3 | 1,153678675 | 1,039887232 | 0,113791444 | |
| 227910_at | XPNPEP3 | 1,153678675 | 1,039887232 | 0,113791444 | |
| 218329_at | PRDM4 | 2,739371812 | 2,625627364 | 0,113744449 | |
| 224838_at | FOXP1 | 2,739371812 | 2,625627364 | 0,113744449 | |
| 225446_at | BRWD1 | 3,394805732 | 3,281121096 | 0,113684636 | |
| 221636_s_at | MARC2 | 0,57058931 | 0,456918297 | 0,113671013 | |
| 225536_at | TMEM54 | 0,57058931 | 0,456918297 | 0,113671013 | |
| 231828_at | LOC253039 | 2,030406107 | 1,916759838 | 0,11364627 | |
| 224581_s_at | NUCKS1 | 4,931823006 | 4,818200317 | 0,113622689 | |
| 230329_s_at | NUDT6 | 1,940595304 | 1,826973888 | 0,113621416 | |
| 1563040_s_at | LOC100506195 | -3,32387526 | -3,437493426 | 0,113618166 | |
| 226372_at | CHST11 | 2,986507217 | 2,87289275 | 0,113614467 | |
| 206147_x_at | SCML2 | 3,659073863 | 3,545468469 | 0,113605394 | |
| 226255_at | ZBTB33 | 3,715139632 | 3,601570866 | 0,113568766 | |
| 222825_at | OTUD6B | 4,042675065 | 3,92911743 | 0,113557635 | |
| 209647_s_at | SOCS5 | 2,197169074 | 2,083632167 | 0,113536907 | |
| 228564_at | LOC375295 | 1,631786706 | 1,518263577 | 0,113523129 | |
| 239995_at | - | 2,310153708 | 2,196649505 | 0,113504203 | |
| 225106_s_at | OGFOD1 | 3,920575295 | 3,807174423 | 0,113400872 | |
| 1552919_at | C4orf36 | -2,667025278 | -2,780313924 | 0,113288647 | |
| 1553076_at | OFCC1 | -2,667025278 | -2,780313924 | 0,113288647 | |
| 1555002_at | MGC39545 | -2,667025278 | -2,780313924 | 0,113288647 | |
| 1560483_at | - | -2,667025278 | -2,780313924 | 0,113288647 | |
| 1561556_at | - | -2,667025278 | -2,780313924 | 0,113288647 | |
| 1562049_at | LOC100507959 | -2,667025278 | -2,780313924 | 0,113288647 | |
| 1563333_at | - | -2,667025278 | -2,780313924 | 0,113288647 | |
| 1569072_s_at | ABCB5 | -2,667025278 | -2,780313924 | 0,113288647 | |
| 1569391_at | TNKS | -2,667025278 | -2,780313924 | 0,113288647 | |
| 1569753_at | - | -2,667025278 | -2,780313924 | 0,113288647 | |
| 1570645_at | - | -2,667025278 | -2,780313924 | 0,113288647 | |
| 205535_s_at | PCDH7 | -2,667025278 | -2,780313924 | 0,113288647 | |
| 209227_at | TUSC3 | -2,667025278 | -2,780313924 | 0,113288647 | |
| 214416_at | - | -2,667025278 | -2,780313924 | 0,113288647 | |
| 229302_at | TMEM178A | -2,667025278 | -2,780313924 | 0,113288647 | |
| 238392_at | - | -2,667025278 | -2,780313924 | 0,113288647 | |
| 238553_at | AGAP9 /// BMS1I | -2,667025278 | -2,780313924 | 0,113288647 | |
| 239287_at | - | -2,667025278 | -2,780313924 | 0,113288647 | |
| 239468_at | MKX | -2,667025278 | -2,780313924 | 0,113288647 | |
| 240750_at | - | -2,667025278 | -2,780313924 | 0,113288647 | |
| 241278_at | - | -2,667025278 | -2,780313924 | 0,113288647 | |
| 243733_at | - | -2,667025278 | -2,780313924 | 0,113288647 | |
| 244372_at | - | -2,667025278 | -2,780313924 | 0,113288647 | |
| 244444_at | LOC100653085 / | -2,667025278 | -2,780313924 | 0,113288647 | |
| 244734_at | MTHFSD | -2,667025278 | -2,780313924 | 0,113288647 | |
| 200633_at | UBB | 8,152949307 | 8,039715419 | 0,113233889 | |
| 226729_at | USP37 | 1,69307511 | 1,579853228 | 0,113221882 | |
| 225932_s_at | HNRNPA2B1 | 4,376357713 | 4,263136983 | 0,11322073 | |
| 56829_at | TRAPPC9 | 2,278289013 | 2,165083199 | 0,113205814 | |
| 212674_s_at | DHX30 | 2,993291442 | 2,880111483 | 0,113179959 | |
| 219575_s_at | COG8 /// PDF | 2,993291442 | 2,880111483 | 0,113179959 | |

| | | | | | |
|---|---|---|---|---|---|
| 205304_s_at | KCNJ8 | 0,317354302 | 0,204310755 | 0,113043546 | |
| 217275_at | TSSK2 | 0,317354302 | 0,204310755 | 0,113043546 | |
| 217758_s_at | TM9SF3 | 4,872528643 | 4,759522298 | 0,113006345 | |
| 211566_x_at | BRE | 2,166283906 | 2,053299695 | 0,11298421 | |
| 216026_s_at | POLE | 1,903621061 | 1,790660532 | 0,11296053 | |
| 1557809_a_at | - | -0,423248115 | -0,536087151 | 0,112839035 | |
| 205864_at | SLC7A4 | -0,423248115 | -0,536087151 | 0,112839035 | |
| 215675_at | - | -0,423248115 | -0,536087151 | 0,112839035 | |
| 219034_at | PARP16 | -0,423248115 | -0,536087151 | 0,112839035 | |
| 235724_at | ACSS1 | -0,423248115 | -0,536087151 | 0,112839035 | |
| 243849_at | TMEM37 | -0,423248115 | -0,536087151 | 0,112839035 | |
| 244234_at | - | -0,423248115 | -0,536087151 | 0,112839035 | |
| 226059_at | TOMM40L | 1,954567604 | 1,841838775 | 0,112728829 | |
| 231853_at | TUBD1 | 1,954567604 | 1,841838775 | 0,112728829 | |
| 224985_at | NRAS | 4,593269972 | 4,480569206 | 0,112700766 | |
| 209479_at | CCDC28A | 3,171349466 | 3,058687544 | 0,112661922 | |
| 214941_s_at | PRPF40A | 3,171349466 | 3,058687544 | 0,112661922 | |
| 1553728_at | LRRC43 | 2,834621043 | 2,721978587 | 0,112642456 | |
| 235749_at | UGGT2 | 1,394109935 | 1,281471383 | 0,112638552 | |
| 1552417_a_at | NEDD1 | 0,702292136 | 0,589657387 | 0,112634749 | |
| 226735_at | TAPT1 | 0,702292136 | 0,589657387 | 0,112634749 | |
| 1568859_a_at | SLC8A3 | 0,171847695 | 0,059218869 | 0,112628827 | |
| 220501_at | ACTL7A | 0,171847695 | 0,059218869 | 0,112628827 | |
| 226857_at | ARHGEF19 | 0,171847695 | 0,059218869 | 0,112628827 | |
| 231360_at | C20orf141 | 0,171847695 | 0,059218869 | 0,112628827 | |
| 229624_at | OPA3 | 1,90937145 | 1,796776588 | 0,112594862 | |
| 213698_at | ZMYM6 /// ZMYM | 2,933877275 | 2,821324225 | 0,11255305 | |
| 1556024_at | EME2 | 0,46522187 | 0,352836757 | 0,112385113 | |
| 201330_at | RARS | 5,150463493 | 5,038079323 | 0,11238417 | |
| 211071_s_at | MLLT11 | 4,79776819 | 4,685434648 | 0,112333542 | |
| 209567_at | RRS1 | 5,023656689 | 4,911338525 | 0,112318164 | |
| 1553169_at | LRRN4 | -0,706943342 | -0,819238336 | 0,112294994 | |
| 1562144_at | - | -0,706943342 | -0,819238336 | 0,112294994 | |
| 206252_s_at | AVPR1A | -0,706943342 | -0,819238336 | 0,112294994 | |
| 208585_at | BTN2A3P | -0,706943342 | -0,819238336 | 0,112294994 | |
| 211224_s_at | ABCB11 | -0,706943342 | -0,819238336 | 0,112294994 | |
| 215039_at | HS2ST1 /// LOC3 | -0,706943342 | -0,819238336 | 0,112294994 | |
| 215337_at | MED24 | -0,706943342 | -0,819238336 | 0,112294994 | |
| 216090_x_at | - | -0,706943342 | -0,819238336 | 0,112294994 | |
| 222782_s_at | GMIP | -0,706943342 | -0,819238336 | 0,112294994 | |
| 223734_at | MGARP | -0,706943342 | -0,819238336 | 0,112294994 | |
| 230002_at | GPSM2 | -0,706943342 | -0,819238336 | 0,112294994 | |
| 232698_at | BPIFB2 | -0,706943342 | -0,819238336 | 0,112294994 | |
| 234369_at | OR4F16 /// OR4F | -0,706943342 | -0,819238336 | 0,112294994 | |
| 235670_at | STX11 | -0,706943342 | -0,819238336 | 0,112294994 | |
| 241823_at | - | -0,706943342 | -0,819238336 | 0,112294994 | |
| 203101_s_at | MGAT2 | 0,592200562 | 0,479907041 | 0,112293521 | |
| 225628_s_at | MLLT6 | 0,592200562 | 0,479907041 | 0,112293521 | |
| 227699_at | C14orf149 | 0,592200562 | 0,479907041 | 0,112293521 | |
| 222673_x_at | FAM122B | 4,392357579 | 4,2801535 | 0,112204078 | |
| 235381_at | RSF1 | 1,709647566 | 1,59747851 | 0,112169055 | |
| 208577_at | HIST1H3A /// HIS | 2,331726354 | 2,21959457 | 0,112131784 | |
| 216547_at | - | 1,917954284 | 1,805902329 | 0,112051955 | |
| 223152_at | PPP1R12C | 1,917954284 | 1,805902329 | 0,112051955 | |
| 227640_s_at | RP9 /// RP9P | 2,259277178 | 2,147243253 | 0,112033925 | |
| 201718_s_at | EPB41L2 | 1,655931423 | 1,543943227 | 0,111988196 | |

| | | | | | |
|---|---|---|---|---|---|
| 226607_at | C20orf194 | 1,655931423 | 1,543943227 | 0,111988196 | |
| 1553538_s_at | COX1 | 8,447104578 | 8,335141653 | 0,111962926 | |
| 211604_x_at | HAP1 | 0,334552598 | 0,222599095 | 0,111953504 | |
| 213085_s_at | WWC1 | 0,334552598 | 0,222599095 | 0,111953504 | |
| 226369_at | LOC338799 | 0,334552598 | 0,222599095 | 0,111953504 | |
| 228856_at | ZNF747 | 0,334552598 | 0,222599095 | 0,111953504 | |
| 225451_at | GRIPAP1 | 1,820612054 | 1,708698295 | 0,111913759 | |
| 204068_at | STK3 | 3,299492934 | 3,187631636 | 0,111861298 | |
| 200594_x_at | HNRNPU | 6,387174329 | 6,275463655 | 0,111710674 | |
| 215093_at | NSDHL | 2,061726373 | 1,950068772 | 0,111657601 | |
| 221079_s_at | METTL2A /// MET | 2,825526235 | 2,713921281 | 0,111604954 | |
| 213066_at | RUSC2 | 1,542099044 | 1,43051703 | 0,111582013 | |
| 211537_x_at | MAP3K7 | 3,42923503 | 3,317734938 | 0,111500092 | |
| 201192_s_at | PITPNA | 3,042527069 | 2,93104672 | 0,11148035 | |
| 229577_at | AGPAT6 | 0,606430281 | 0,495031851 | 0,111398431 | |
| 219132_at | PELI2 | -0,172274514 | -0,283667828 | 0,111393314 | |
| 226991_at | NFATC2 | -0,172274514 | -0,283667828 | 0,111393314 | |
| 239718_at | - | -0,172274514 | -0,283667828 | 0,111393314 | |
| 243285_at | LOC283335 | -0,172274514 | -0,283667828 | 0,111393314 | |
| 238652_at | - | 0,928678725 | 0,817300337 | 0,111378388 | |
| 213235_at | C16orf88 | 1,481627361 | 1,370274469 | 0,111352892 | |
| 213344_s_at | H2AFX | 1,829737795 | 1,718396606 | 0,111341189 | |
| 238541_at | C21orf58 | 1,829737795 | 1,718396606 | 0,111341189 | |
| 229980_s_at | SNX5 | 5,63729661 | 5,525968169 | 0,111328441 | |
| 223258_s_at | G2E3 | 2,905386372 | 2,794067719 | 0,111318653 | |
| 219598_s_at | RWDD1 | 4,244385102 | 4,133220924 | 0,111164178 | |
| 223188_at | SMG9 | 1,726031811 | 1,614891062 | 0,111140749 | |
| 228743_at | TXNDC17 | 1,726031811 | 1,614891062 | 0,111140749 | |
| 201829_at | NET1 | 4,497501866 | 4,386375749 | 0,111126117 | |
| 202125_s_at | TRAK2 | 2,549899391 | 2,438805127 | 0,111094264 | |
| 208478_s_at | BAX | 1,348276312 | 1,23718272 | 0,111093592 | |
| 226350_at | CHML | 3,90985831 | 3,798818761 | 0,11103955 | |
| 219211_at | USP18 | 2,156646206 | 2,045615873 | 0,111030333 | |
| 201935_s_at | EIF4G3 | 2,277177578 | 2,166264721 | 0,110912857 | |
| 209117_at | WBP2 | 1,937784532 | 1,826973888 | 0,110810644 | |
| 202142_at | COPS8 | 3,406034785 | 3,295230443 | 0,110804342 | |
| 205244_s_at | SLC13A3 | 0,03125738 | -0,079522948 | 0,110780328 | |
| 238864_at | VWA3A | 0,03125738 | -0,079522948 | 0,110780328 | |
| 244368_x_at | - | 0,03125738 | -0,079522948 | 0,110780328 | |
| 226381_at | LOC100506748 | 2,077135162 | 1,966439221 | 0,110695941 | |
| 211185_s_at | SF3B1 | 5,986946417 | 5,876251039 | 0,110695378 | |
| 1552816_at | NXNL2 | -2,779459409 | -2,89013181 | 0,110672402 | |
| 1554676_at | SRGN | -2,779459409 | -2,89013181 | 0,110672402 | |
| 1555517_at | GABRG3 | -2,779459409 | -2,89013181 | 0,110672402 | |
| 1556883_a_at | LOC440896 | -2,779459409 | -2,89013181 | 0,110672402 | |
| 1561137_s_at | GYPE | -2,779459409 | -2,89013181 | 0,110672402 | |
| 1561737_at | - | -2,779459409 | -2,89013181 | 0,110672402 | |
| 1562587_at | CLNK | -2,779459409 | -2,89013181 | 0,110672402 | |
| 1565699_at | HECTD2 | -2,779459409 | -2,89013181 | 0,110672402 | |
| 1566267_at | - | -2,779459409 | -2,89013181 | 0,110672402 | |
| 1567856_x_at | ZNF29P | -2,779459409 | -2,89013181 | 0,110672402 | |
| 1569272_at | PIK3C3 | -2,779459409 | -2,89013181 | 0,110672402 | |
| 1570289_at | LOC646736 | -2,779459409 | -2,89013181 | 0,110672402 | |
| 202508_s_at | SNAP25 | -2,779459409 | -2,89013181 | 0,110672402 | |
| 203798_s_at | VSNL1 | -2,779459409 | -2,89013181 | 0,110672402 | |
| 206545_at | CD28 | -2,779459409 | -2,89013181 | 0,110672402 | |

| | | | | | |
|---|---|---|---|---|---|
| 231717_s_at | ZNF226 | -2,779459409 | -2,89013181 | 0,110672402 | |
| 233120_at | - | -2,779459409 | -2,89013181 | 0,110672402 | |
| 233346_at | - | -2,779459409 | -2,89013181 | 0,110672402 | |
| 233615_at | CGA | -2,779459409 | -2,89013181 | 0,110672402 | |
| 233853_at | - | -2,779459409 | -2,89013181 | 0,110672402 | |
| 235955_at | MARVELD2 | -2,779459409 | -2,89013181 | 0,110672402 | |
| 238224_at | - | -2,779459409 | -2,89013181 | 0,110672402 | |
| 240749_at | LOC100505862 | -2,779459409 | -2,89013181 | 0,110672402 | |
| 241744_x_at | - | -2,779459409 | -2,89013181 | 0,110672402 | |
| 242631_x_at | DLC1 | -2,779459409 | -2,89013181 | 0,110672402 | |
| 242762_s_at | FAM171B | -2,779459409 | -2,89013181 | 0,110672402 | |
| 244405_s_at | - | -2,779459409 | -2,89013181 | 0,110672402 | |
| 244503_at | - | -2,779459409 | -2,89013181 | 0,110672402 | |
| 244657_at | - | -2,779459409 | -2,89013181 | 0,110672402 | |
| 1553167_a_at | SEPSECS | 0,735041319 | 0,624460759 | 0,11058056 | |
| 207547_s_at | FAM107A /// LOC | 0,735041319 | 0,624460759 | 0,11058056 | |
| 230019_s_at | PTCHD2 | 0,735041319 | 0,624460759 | 0,11058056 | |
| 232860_x_at | RBM41 | 0,735041319 | 0,624460759 | 0,11058056 | |
| 238333_s_at | MTG1 | 0,735041319 | 0,624460759 | 0,11058056 | |
| 214501_s_at | H2AFY | 3,842326541 | 3,731768909 | 0,110557633 | |
| 229949_at | - | 2,589836943 | 2,479296438 | 0,110540506 | |
| 212484_at | FAM89B | 1,735773781 | 1,6252386 | 0,110535181 | |
| 228775_at | EMC3 | 1,844820248 | 1,73441697 | 0,110403278 | |
| 1561421_a_at | - | 0,359971644 | 0,249604176 | 0,110367468 | |
| 1569321_at | - | 0,359971644 | 0,249604176 | 0,110367468 | |
| 205232_s_at | PAFAH2 | 0,359971644 | 0,249604176 | 0,110367468 | |
| 219617_at | CAMKMT | 0,359971644 | 0,249604176 | 0,110367468 | |
| 238590_x_at | TMEM107 | 0,359971644 | 0,249604176 | 0,110367468 | |
| 37966_at | PARVB | 1,398205319 | 1,287998959 | 0,11020636 | |
| 47608_at | TJAP1 | 3,458946151 | 3,348766952 | 0,110179198 | |
| 1558954_at | SNORA78 | 1,795990708 | 1,685812099 | 0,110178608 | |
| 200946_x_at | GLUD1 | 4,206936942 | 4,096815476 | 0,110121466 | |
| 218785_s_at | RABL5 | 0,627515113 | 0,51742585 | 0,110089263 | |
| 229541_at | - | 0,627515113 | 0,51742585 | 0,110089263 | |
| 205321_at | EIF2S3 | 5,739893061 | 5,629826771 | 0,11006629 | |
| 201512_s_at | TOMM70A | 3,997499421 | 3,887456383 | 0,110043037 | |
| 209314_s_at | HBS1L | 4,444279108 | 4,334242016 | 0,110037092 | |
| 203856_at | VRK1 | 4,895163777 | 4,785127613 | 0,110036164 | |
| 235109_at | ZBED3 | 0,503742507 | 0,393761504 | 0,109981003 | |
| 244758_at | SCAND3 | 0,503742507 | 0,393761504 | 0,109981003 | |
| 201834_at | PRKAB1 | 2,568189989 | 2,458228496 | 0,109961494 | |
| 1552646_at | IL11RA | -0,148196756 | -0,258114234 | 0,109917477 | |
| 207281_x_at | VCX2 | -0,148196756 | -0,258114234 | 0,109917477 | |
| 224393_s_at | CECR6 | -0,148196756 | -0,258114234 | 0,109917477 | |
| 231246_at | - | -0,148196756 | -0,258114234 | 0,109917477 | |
| 243305_at | - | -0,148196756 | -0,258114234 | 0,109917477 | |
| 202430_s_at | PLSCR1 | 3,773843756 | 3,663961904 | 0,109881852 | |
| 218240_at | NKIRAS2 | 1,85379461 | 1,743944487 | 0,109850122 | |
| 203149_at | PVRL2 | 2,134723057 | 2,024923328 | 0,109799729 | |
| 228070_at | PPP2R5E | 1,369290453 | 1,259496994 | 0,109793458 | |
| 209599_s_at | PRUNE | 0,747935635 | 0,638150376 | 0,109785259 | |
| 212437_at | CENPB | 0,747935635 | 0,638150376 | 0,109785259 | |
| 239411_at | - | 0,747935635 | 0,638150376 | 0,109785259 | |
| 202620_s_at | PLOD2 | 3,617524043 | 3,507749374 | 0,10977467 | |
| 208953_at | LARP4B | 3,19743497 | 3,087661492 | 0,109773478 | |
| 215040_at | RNASEH2B | 0,95671774 | 0,847075464 | 0,109642276 | |

| | | | | | |
|---|---|---|---|---|---|
| 235546_at | SPINT1 | 0,95671774 | 0,847075464 | 0,109642276 | |
| 202118_s_at | CPNE3 | 3,072307152 | 2,962671751 | 0,109635401 | |
| 212377_s_at | NOTCH2 | 2,573632254 | 2,464004877 | 0,109627377 | |
| 1554097_a_at | MIR31HG | -0,655928373 | -0,765504029 | 0,109575656 | |
| 1568734_a_at | HS1BP3 | -0,655928373 | -0,765504029 | 0,109575656 | |
| 1570171_at | - | -0,655928373 | -0,765504029 | 0,109575656 | |
| 205049_s_at | CD79A | -0,655928373 | -0,765504029 | 0,109575656 | |
| 206150_at | CD27 | -0,655928373 | -0,765504029 | 0,109575656 | |
| 206171_at | ADORA3 | -0,655928373 | -0,765504029 | 0,109575656 | |
| 210746_s_at | EPB42 | -0,655928373 | -0,765504029 | 0,109575656 | |
| 213195_at | C17orf108 | -0,655928373 | -0,765504029 | 0,109575656 | |
| 213949_s_at | DOHH | -0,655928373 | -0,765504029 | 0,109575656 | |
| 214535_s_at | ADAMTS2 | -0,655928373 | -0,765504029 | 0,109575656 | |
| 222677_x_at | - | -0,655928373 | -0,765504029 | 0,109575656 | |
| 229556_at | LOC100288893 | -0,655928373 | -0,765504029 | 0,109575656 | |
| 232973_at | DOCK1 | -0,655928373 | -0,765504029 | 0,109575656 | |
| 234832_at | ANTXR1 | -0,655928373 | -0,765504029 | 0,109575656 | |
| 239198_at | EZH1 | -0,655928373 | -0,765504029 | 0,109575656 | |
| 203519_s_at | UPF2 | 3,373122675 | 3,263564154 | 0,109558521 | |
| 237403_at | GFI1B | 5,051551426 | 4,942014748 | 0,109536678 | |
| 211999_at | H3F3A /// H3F3B | 6,498417648 | 6,388994204 | 0,109423444 | |
| 204343_at | ABCA3 | 1,224581536 | 1,115234684 | 0,109346851 | |
| 1566146_x_at | - | 0,376672223 | 0,26733076 | 0,109341464 | |
| 219541_at | LIME1 /// SLC2A4 | 0,376672223 | 0,26733076 | 0,109341464 | |
| 227385_at | PPAPDC2 | 0,376672223 | 0,26733076 | 0,109341464 | |
| 239490_at | COA1 | 0,376672223 | 0,26733076 | 0,109341464 | |
| 202969_at | DYRK2 | 1,578645857 | 1,469325098 | 0,109320759 | |
| 232149_s_at | NSMAF | 3,579258128 | 3,469974737 | 0,109283391 | |
| 210529_s_at | FAM115A /// LOC | 1,699726954 | 1,590454215 | 0,109272739 | |
| 214315_x_at | CALR | 4,898781575 | 4,789543023 | 0,109238552 | |
| 222470_s_at | UQCC | 0,641402394 | 0,532164371 | 0,109238024 | |
| 221521_s_at | GINS2 | 4,360703168 | 4,251494565 | 0,109208603 | |
| 204930_s_at | BNIP1 | 2,102456302 | 1,993317152 | 0,10913915 | |
| 203357_s_at | CAPN7 | 2,840652551 | 2,731588328 | 0,109064223 | |
| 201671_x_at | USP14 | 3,689469311 | 3,580454966 | 0,109014345 | |
| 209580_s_at | MBD4 | 4,732770466 | 4,623770256 | 0,10900021 | |
| 219080_s_at | CTPS2 | 1,868628927 | 1,759685182 | 0,108943745 | |
| 223054_at | DNAJB11 | 4,768801946 | 4,659868459 | 0,108933488 | |
| 227919_at | UCA1 | 6,017883727 | 5,908998287 | 0,10888544 | |
| 225145_at | NCOA5 | 3,085065712 | 2,976208448 | 0,108857264 | |
| 202337_at | PMF1 | 2,896728217 | 2,787940144 | 0,108788072 | |
| 222730_s_at | ZDHHC2 | 3,292909151 | 3,184136378 | 0,108772774 | |
| 1552618_at | STX6 | 2,491678455 | 2,382985886 | 0,108692569 | |
| 204603_at | EXO1 | 2,422991296 | 2,314300277 | 0,108691019 | |
| 218936_s_at | CCDC59 | 4,05440026 | 3,945725977 | 0,108674283 | |
| 1554701_a_at | TBC1D16 | -0,352951799 | -0,461594427 | 0,108642628 | |
| 1561037_a_at | - | -0,352951799 | -0,461594427 | 0,108642628 | |
| 216741_at | - | -0,352951799 | -0,461594427 | 0,108642628 | |
| 232042_at | TTYH2 | -0,352951799 | -0,461594427 | 0,108642628 | |
| 1560019_at | MGC11082 | 0,767063567 | 0,658444279 | 0,108619288 | |
| 228663_x_at | FIZ1 | 0,767063567 | 0,658444279 | 0,108619288 | |
| 218117_at | RBX1 | 5,837467257 | 5,728908813 | 0,108558445 | |
| 201245_s_at | OTUB1 | 3,850569363 | 3,742106442 | 0,108462921 | |
| 202442_at | AP3S1 | 4,939746973 | 4,831294479 | 0,108452494 | |
| 201737_s_at | 06.03.15 | 3,976425635 | 3,86798237 | 0,108443265 | |
| 217379_at | - | 5,161347762 | 5,05300713 | 0,108340632 | |

| | | | | | |
|---|---|---|---|---|---|
| 223342_at | RRM2B | 2,879254447 | 2,770953871 | 0,108300576 | |
| 207428_x_at | CDK11A /// CDK1 | 1,716223609 | 1,60795124 | 0,108272369 | |
| 224366_s_at | REPS1 | 3,394805732 | 3,28656411 | 0,108241622 | |
| 222044_at | PCIF1 | 1,596577845 | 1,488344553 | 0,108233293 | |
| 208540_x_at | - | 3,050355245 | 2,942125533 | 0,108229712 | |
| 242608_x_at | FAM161B | 3,004080279 | 2,895866687 | 0,108213592 | |
| 226314_at | CHST14 | 1,774096855 | 1,665902186 | 0,10819467 | |
| 201166_s_at | PUM1 | 5,492057836 | 5,38389617 | 0,108161666 | |
| 207002_s_at | PLAGL1 | 4,550671723 | 4,442554164 | 0,108117559 | |
| 1554170_a_at | MBLAC1 | 1,163336153 | 1,055274826 | 0,108061327 | |
| 212619_at | TMEM194A | 0,984222186 | 0,876248481 | 0,107973705 | |
| 229210_at | RNASEH2B | 0,984222186 | 0,876248481 | 0,107973705 | |
| 204256_at | ELOVL6 | 4,511983428 | 4,404011141 | 0,107972287 | |
| 1555032_at | RGS12 | -0,622910612 | -0,730778808 | 0,107868196 | |
| 1559902_at | MKL1 | -0,622910612 | -0,730778808 | 0,107868196 | |
| 1562436_at | CARS-AS1 | -0,622910612 | -0,730778808 | 0,107868196 | |
| 233100_at | EEPD1 | -0,622910612 | -0,730778808 | 0,107868196 | |
| 233342_at | LOC100134040 | -0,622910612 | -0,730778808 | 0,107868196 | |
| 236298_at | PDSS1 | -0,622910612 | -0,730778808 | 0,107868196 | |
| 237594_at | - | -0,622910612 | -0,730778808 | 0,107868196 | |
| 240430_at | KCMF1 | -0,622910612 | -0,730778808 | 0,107868196 | |
| 243151_at | LOC100506282 | -0,622910612 | -0,730778808 | 0,107868196 | |
| 244876_at | - | -0,622910612 | -0,730778808 | 0,107868196 | |
| 223331_s_at | DDX20 | 3,166555554 | 3,058687544 | 0,10786801 | |
| 235143_at | SLC10A7 | 0,779676066 | 0,67181667 | 0,107859396 | |
| 224555_x_at | IL37 | 0,401366113 | 0,293518836 | 0,107847277 | |
| 226894_at | SLC35A3 | 2,571820446 | 2,464004877 | 0,107815569 | |
| 212574_x_at | C19orf6 | 1,40228911 | 1,294497133 | 0,107791977 | |
| 201497_x_at | MYH11 | -0,112808516 | -0,220592223 | 0,107783707 | |
| 205853_at | ZBTB7B | -0,112808516 | -0,220592223 | 0,107783707 | |
| 236424_at | CEP250 | -0,112808516 | -0,220592223 | 0,107783707 | |
| 243953_at | FADS2 | -0,112808516 | -0,220592223 | 0,107783707 | |
| 244178_at | COMMD7 | -0,112808516 | -0,220592223 | 0,107783707 | |
| 232033_at | USP37 | 1,835789711 | 1,728030156 | 0,107759554 | |
| 207129_at | CA5B | 0,541261317 | 0,433557298 | 0,107704019 | |
| 224950_at | PTGFRN | 0,541261317 | 0,433557298 | 0,107704019 | |
| 242600_at | FRMD3 | 0,541261317 | 0,433557298 | 0,107704019 | |
| 228131_at | ERCC1 | 1,251994932 | 1,144305291 | 0,107689641 | |
| 1569030_s_at | NUB1 | 2,605861638 | 2,498185745 | 0,107675893 | |
| 203076_s_at | SMAD2 | 3,232262672 | 3,124599694 | 0,107662977 | |
| 217911_s_at | BAG3 | 3,013454777 | 2,905803877 | 0,1076509 | |
| 232991_at | - | 0,083054335 | -0,024572586 | 0,107626921 | |
| 239596_at | SLC30A7 | 0,083054335 | -0,024572586 | 0,107626921 | |
| 228622_s_at | DNAJC4 | 1,331241794 | 1,223626587 | 0,107615206 | |
| 215691_x_at | HSPB11 | 5,197340607 | 5,08974239 | 0,107598218 | |
| 221803_s_at | NRBF2 | 1,607231019 | 1,499637023 | 0,107593996 | |
| 204749_at | NAP1L3 | 4,255150375 | 4,147649355 | 0,107501021 | |
| 225617_at | ODF2 | 2,173470163 | 2,066015817 | 0,107454346 | |
| 212558_at | SPRY1 | 3,408067074 | 3,300620584 | 0,10744649 | |
| 227447_at | SKIV2L2 | 3,408067074 | 3,300620584 | 0,10744649 | |
| 224561_s_at | MORF4L1 | 6,082774913 | 5,975380244 | 0,107394669 | |
| 209130_at | SNAP23 | 5,535001352 | 5,427660949 | 0,107340403 | |
| 213398_s_at | SDR39U1 | 3,480351894 | 3,373018423 | 0,10733347 | |
| 201272_at | AKR1B1 | 5,391902301 | 5,284574019 | 0,107328282 | |
| 207467_x_at | CAST | 4,144312047 | 4,037037849 | 0,107274198 | |
| 219037_at | RRP15 | 3,857278636 | 3,75000829 | 0,107270345 | |

| | | | | | |
|---|---|---|---|---|---|
| 209120_at | NR2F2 | 3,674351634 | 3,56709884 | 0,107252794 | |
| 1557502_at | PCCB | -1,047650486 | -1,154897679 | 0,107247193 | |
| 1559550_s_at | - | -1,047650486 | -1,154897679 | 0,107247193 | |
| 1564074_at | - | -1,047650486 | -1,154897679 | 0,107247193 | |
| 1568706_s_at | LOC100653271 / | -1,047650486 | -1,154897679 | 0,107247193 | |
| 1569097_at | TP53BP1 | -1,047650486 | -1,154897679 | 0,107247193 | |
| 206882_at | SLC1A6 | -1,047650486 | -1,154897679 | 0,107247193 | |
| 224297_s_at | SPTBN4 | -1,047650486 | -1,154897679 | 0,107247193 | |
| 228874_at | PHLDB3 | -1,047650486 | -1,154897679 | 0,107247193 | |
| 230622_at | MLLT4 | -1,047650486 | -1,154897679 | 0,107247193 | |
| 232709_at | - | -1,047650486 | -1,154897679 | 0,107247193 | |
| 240314_at | - | -1,047650486 | -1,154897679 | 0,107247193 | |
| 240357_at | - | -1,047650486 | -1,154897679 | 0,107247193 | |
| 242011_at | - | -1,047650486 | -1,154897679 | 0,107247193 | |
| 214017_s_at | DHX34 | 1,549482847 | 1,442269454 | 0,107213393 | |
| 222644_s_at | GLT25D1 | 1,897847661 | 1,790660532 | 0,107187129 | |
| 213343_s_at | GDPD5 | 0,67554678 | 0,568365095 | 0,107181685 | |
| 244738_at | BRWD3 | 0,67554678 | 0,568365095 | 0,107181685 | |
| 218481_at | EXOSC5 | 3,260656977 | 3,153484076 | 0,107172901 | |
| 236067_at | - | 1,261018186 | 1,153866761 | 0,107151424 | |
| 225376_at | C20orf11 | 4,575296564 | 4,468168179 | 0,107128385 | |
| 226371_at | KDM5A | 2,261526912 | 2,154405725 | 0,107121186 | |
| 214864_s_at | GRHPR | 3,466766854 | 3,359653127 | 0,107113728 | |
| 203025_at | NAA10 | 3,069741844 | 2,962671751 | 0,107070094 | |
| 226290_at | BDP1 | 3,450096885 | 3,343031718 | 0,107065167 | |
| 201566_x_at | ID2 | 1,485482016 | 1,378453643 | 0,107028372 | |
| 204867_at | GCHFR | 1,485482016 | 1,378453643 | 0,107028372 | |
| 236129_at | GALNT5 | 2,520143371 | 2,413157372 | 0,106985999 | |
| 230257_s_at | TSEN15 | 2,303618506 | 2,196649505 | 0,106969001 | |
| 204044_at | QPRT | 2,001086023 | 1,894118818 | 0,106967205 | |
| 227324_at | ADCK4 | 1,094335495 | 0,987376598 | 0,106958897 | |
| 200821_at | LAMP2 | 4,037433122 | 3,930508802 | 0,10692432 | |
| 221579_s_at | NUDT3 /// RPS1( | 2,710020346 | 2,603119255 | 0,106901091 | |
| 230138_at | NCS1 | 0,417596971 | 0,310717132 | 0,106879839 | |
| 238061_at | LGI3 | 0,417596971 | 0,310717132 | 0,106879839 | |
| 223022_s_at | VTA1 | 4,265835914 | 4,158970399 | 0,106865515 | |
| 1553191_at | DST /// LOC1006 | -2,930741289 | -3,037500868 | 0,106759579 | |
| 1555519_at | - | -2,930741289 | -3,037500868 | 0,106759579 | |
| 1556158_at | FAM154B | -2,930741289 | -3,037500868 | 0,106759579 | |
| 1556491_at | - | -2,930741289 | -3,037500868 | 0,106759579 | |
| 1559341_at | - | -2,930741289 | -3,037500868 | 0,106759579 | |
| 1560098_at | - | -2,930741289 | -3,037500868 | 0,106759579 | |
| 1560526_at | MAGI2-IT1 | -2,930741289 | -3,037500868 | 0,106759579 | |
| 1561264_at | - | -2,930741289 | -3,037500868 | 0,106759579 | |
| 1561328_at | - | -2,930741289 | -3,037500868 | 0,106759579 | |
| 1562566_at | TRAF3IP2-AS1 | -2,930741289 | -3,037500868 | 0,106759579 | |
| 1563332_at | - | -2,930741289 | -3,037500868 | 0,106759579 | |
| 1567378_x_at | DNAH1 | -2,930741289 | -3,037500868 | 0,106759579 | |
| 1569408_at | EIF2C4 | -2,930741289 | -3,037500868 | 0,106759579 | |
| 1569788_at | ST8SIA1 | -2,930741289 | -3,037500868 | 0,106759579 | |
| 1569969_a_at | UNC13C | -2,930741289 | -3,037500868 | 0,106759579 | |
| 1570584_at | MPZL3 | -2,930741289 | -3,037500868 | 0,106759579 | |
| 207479_at | - | -2,930741289 | -3,037500868 | 0,106759579 | |
| 210330_at | SGCD | -2,930741289 | -3,037500868 | 0,106759579 | |
| 211054_at | INVS | -2,930741289 | -3,037500868 | 0,106759579 | |
| 224363_at | - | -2,930741289 | -3,037500868 | 0,106759579 | |

| | | | | | |
|---|---|---|---|---|---|
| 225571_at | LIFR | -2,930741289 | -3,037500868 | 0,106759579 | |
| 232673_at | LRRFIP2 | -2,930741289 | -3,037500868 | 0,106759579 | |
| 236532_at | C11orf87 | -2,930741289 | -3,037500868 | 0,106759579 | |
| 237116_at | LOC646903 | -2,930741289 | -3,037500868 | 0,106759579 | |
| 237374_at | - | -2,930741289 | -3,037500868 | 0,106759579 | |
| 238149_at | ZNF818P | -2,930741289 | -3,037500868 | 0,106759579 | |
| 244004_at | - | -2,930741289 | -3,037500868 | 0,106759579 | |
| 244888_at | - | -2,930741289 | -3,037500868 | 0,106759579 | |
| 209628_at | NXT2 | 3,345817457 | 3,239069096 | 0,106748361 | |
| 214438_at | HLX | 0,798390451 | 0,691645685 | 0,106744766 | |
| 210933_s_at | FSCN1 /// LOC10 | 2,559073676 | 2,452428893 | 0,106644783 | |
| 221777_at | C12orf52 | 2,882181479 | 2,775606361 | 0,106575118 | |
| 1559755_at | C21orf49 | -0,089689931 | -0,19610998 | 0,106420049 | |
| 1569095_at | LOC731424 | -0,089689931 | -0,19610998 | 0,106420049 | |
| 210476_s_at | PRLR | -0,089689931 | -0,19610998 | 0,106420049 | |
| 213278_at | MTMR9 | 2,688444421 | 2,582026171 | 0,10641825 | |
| 234980_at | TMEM56 | 0,911589958 | 0,805216064 | 0,106373894 | |
| 203442_x_at | EML3 | 1,011212064 | 0,904843258 | 0,106368805 | |
| 203705_s_at | FZD7 | 1,011212064 | 0,904843258 | 0,106368805 | |
| 212305_s_at | MIA3 | 1,011212064 | 0,904843258 | 0,106368805 | |
| 206005_s_at | KIAA1009 | 1,352503698 | 1,246149888 | 0,10635381 | |
| 222502_s_at | UFM1 | 2,427005622 | 2,320681318 | 0,106324304 | |
| 219258_at | TIPIN | 3,059434754 | 2,953119918 | 0,106314836 | |
| 201628_s_at | RRAGA | 4,024244326 | 3,917937882 | 0,106306444 | |
| 203622_s_at | PNO1 | 4,777867415 | 4,671564559 | 0,106302856 | |
| 223144_s_at | AKIRIN2 | 4,060873315 | 3,954643074 | 0,106230241 | |
| 35436_at | GOLGA2 | 0,692320641 | 0,586130423 | 0,106190218 | |
| 228927_at | ZNF397 | 2,151803104 | 2,045615873 | 0,106187231 | |
| 225788_at | RRP36 | 4,119101006 | 4,012941072 | 0,106159934 | |
| 228661_s_at | LOC100506029 / | 1,567778694 | 1,461646549 | 0,106132144 | |
| 215832_x_at | PICALM | 2,752227654 | 2,646096941 | 0,106130713 | |
| 219855_at | NUDT11 | 3,086335384 | 2,980244813 | 0,106090571 | |
| 1557910_at | HSP90AB1 | 6,919648195 | 6,813558862 | 0,106089334 | |
| 201327_s_at | CCT6A | 5,775355733 | 5,669321722 | 0,106034011 | |
| 211028_s_at | KHK | 0,810733188 | 0,704715219 | 0,106017969 | |
| 233065_at | RNF207 | 0,810733188 | 0,704715219 | 0,106017969 | |
| 1555851_s_at | SEPW1 | 5,612394725 | 5,506392234 | 0,106002491 | |
| 1560451_at | - | 0,695652136 | 0,589657387 | 0,105994749 | |
| 211656_x_at | HLA-DQB1 /// LO | 0,695652136 | 0,589657387 | 0,105994749 | |
| 219032_x_at | OPN3 | 0,695652136 | 0,589657387 | 0,105994749 | |
| 239024_at | ZNF148 | 0,695652136 | 0,589657387 | 0,105994749 | |
| 40225_at | GAK | 3,659500438 | 3,553617909 | 0,105882528 | |
| 225524_at | ANTXR2 | 3,112743591 | 3,006869115 | 0,105874476 | |
| 227846_at | GPR176 | 1,283332461 | 1,177496821 | 0,10583564 | |
| 235134_at | - | 2,159061673 | 2,053299695 | 0,105761978 | |
| 203693_s_at | E2F3 | 4,654470045 | 4,548745669 | 0,105724376 | |
| 210457_x_at | HMGA1 /// HMGA | 1,575032557 | 1,469325098 | 0,105707459 | |
| 223662_x_at | DDX59 | 1,575032557 | 1,469325098 | 0,105707459 | |
| 208927_at | SPOP | 3,508091848 | 3,402394519 | 0,105697329 | |
| 1554868_s_at | PCNP | 4,402076958 | 4,296432174 | 0,105644784 | |
| 219981_x_at | ZNF587 /// ZNF5 | 2,402750389 | 2,297144887 | 0,105605502 | |
| 205077_s_at | PIGF | 3,747601714 | 3,642048499 | 0,105553215 | |
| 238453_at | FGFBP3 | 2,288253583 | 2,182705233 | 0,10554835 | |
| 214210_at | SLC25A17 | 1,926486359 | 1,820984782 | 0,105501578 | |
| 224975_at | NFIA | 1,119359127 | 1,013870809 | 0,105488318 | |
| 226600_at | TMTC3 | 1,119359127 | 1,013870809 | 0,105488318 | |

| | | | | | |
|---|---|---|---|---|---|
| 212330_at | TFDP1 | 4,763255815 | 4,657769855 | 0,10548596 | |
| 211528_x_at | HLA-G | 5,196458993 | 5,090987226 | 0,105471767 | |
| 240318_at | AFMID | 0,44160593 | 0,336136178 | 0,105469752 | |
| 1554251_at | HP1BP3 | 2,734521183 | 2,629059192 | 0,105461991 | |
| 200948_at | MLF2 | 4,131454119 | 4,026016313 | 0,105437806 | |
| 207198_s_at | LIMS1 | 4,538199871 | 4,432769076 | 0,105430795 | |
| 218111_s_at | CMAS | 1,442503549 | 1,337086025 | 0,105417524 | |
| 221029_s_at | WNT5B | 1,442503549 | 1,337086025 | 0,105417524 | |
| 209054_s_at | WHSC1 | 3,209137871 | 3,103784293 | 0,105353579 | |
| 1556361_s_at | ANKRD13C | -0,57445847 | -0,679763839 | 0,105305369 | |
| 1559477_s_at | MEIS1 | -0,57445847 | -0,679763839 | 0,105305369 | |
| 1564897_at | TNR | -0,57445847 | -0,679763839 | 0,105305369 | |
| 225165_at | PPP1R1B | -0,57445847 | -0,679763839 | 0,105305369 | |
| 226655_at | STX17 | -0,57445847 | -0,679763839 | 0,105305369 | |
| 231032_at | LOC286071 | -0,57445847 | -0,679763839 | 0,105305369 | |
| 235681_at | - | -0,57445847 | -0,679763839 | 0,105305369 | |
| 238235_at | - | -0,57445847 | -0,679763839 | 0,105305369 | |
| 243343_at | - | -0,57445847 | -0,679763839 | 0,105305369 | |
| 244191_at | RPLP1 | -0,57445847 | -0,679763839 | 0,105305369 | |
| 231094_s_at | MTHFD1L | 1,210676994 | 1,105412836 | 0,105264158 | |
| 208808_s_at | HMGB2 | 7,085335131 | 6,980109941 | 0,10522519 | |
| 204132_s_at | FOXO3 /// FOXO | 1,932146506 | 1,826973888 | 0,105172617 | |
| 216338_s_at | YIPF3 | 2,294858597 | 2,189694216 | 0,105164381 | |
| 212683_at | SLC25A44 | 1,649074067 | 1,543943227 | 0,105130839 | |
| 226180_at | WDR36 | 3,617524043 | 3,512402411 | 0,105121632 | |
| 201252_at | PSMC4 | 5,963472883 | 5,858361335 | 0,105111548 | |
| 225890_at | C20orf72 | 4,60172953 | 4,496626742 | 0,105102788 | |
| 204361_s_at | SKAP2 | 1,296557217 | 1,191491281 | 0,105065936 | |
| 203007_x_at | LYPLA1 | 4,788632316 | 4,683579237 | 0,105053079 | |
| 219274_at | TSPAN12 | 2,553576109 | 2,448549499 | 0,10502661 | |
| 238183_at | - | 0,82905153 | 0,724100169 | 0,104951361 | |
| 204238_s_at | C6orf108 | 2,035673598 | 1,930732138 | 0,104941461 | |
| 226509_at | ZNF641 | 2,035673598 | 1,930732138 | 0,104941461 | |
| 212282_at | TMEM97 | 4,760076998 | 4,655247494 | 0,104829504 | |
| 225967_s_at | C17orf89 | 4,548830831 | 4,44401622 | 0,104814611 | |
| 206844_at | FBP2 | -0,285837228 | -0,39061235 | 0,104775122 | |
| 207355_at | SLC1A7 | -0,285837228 | -0,39061235 | 0,104775122 | |
| 229054_at | ZFP36L1 | -0,285837228 | -0,39061235 | 0,104775122 | |
| 242443_at | EML5 | -0,285837228 | -0,39061235 | 0,104775122 | |
| 208731_at | RAB2A | 4,647605722 | 4,542844647 | 0,104761075 | |
| 219626_at | MAP7D3 | 1,454352496 | 1,349621332 | 0,104731164 | |
| 1552409_a_at | ODF4 | 0,133056859 | 0,028364014 | 0,104692844 | |
| 219852_s_at | MORN1 | 0,133056859 | 0,028364014 | 0,104692844 | |
| 222830_at | GRHL1 | 0,133056859 | 0,028364014 | 0,104692844 | |
| 232915_at | DDX49 | 0,133056859 | 0,028364014 | 0,104692844 | |
| 215285_s_at | PHTF1 | 1,940595304 | 1,835911187 | 0,104684117 | |
| 227551_at | FAM108B1 | 3,704430214 | 3,599822964 | 0,10460725 | |
| 228126_x_at | CTXN1 | 0,457392764 | 0,352836757 | 0,104556007 | |
| 218284_at | SMAD3 | 2,089851283 | 1,98530622 | 0,104545063 | |
| 212371_at | DESI2 | 3,930506493 | 3,825986493 | 0,104520001 | |
| 226712_at | SSR1 | 2,562727117 | 2,458228496 | 0,104498621 | |
| 222438_at | MED4 | 1,780386218 | 1,675891488 | 0,10449473 | |
| 1554151_at | OGDH | -0,055687482 | -0,160136748 | 0,104449266 | |
| 207743_at | - | -0,055687482 | -0,160136748 | 0,104449266 | |
| 208407_s_at | CTNND1 /// TMX | -0,055687482 | -0,160136748 | 0,104449266 | |
| 211663_x_at | PTGDS | -0,055687482 | -0,160136748 | 0,104449266 | |

| | | | | | |
|---|---|---|---|---|---|
| 216088_s_at | PSMA7 | 4,486724797 | 4,382314353 | 0,104410444 | |
| 205817_at | SIX1 | 0,30867765 | 0,204310755 | 0,104366894 | |
| 211267_at | HESX1 | 0,30867765 | 0,204310755 | 0,104366894 | |
| 209268_at | VPS45 | 3,288503211 | 3,184136378 | 0,104366833 | |
| 222702_x_at | CRIPT | 3,997499421 | 3,893176339 | 0,104323082 | |
| 231855_at | KIAA1524 | 1,894952274 | 1,790660532 | 0,104291742 | |
| 1554415_at | TAF5L | 1,462198073 | 1,3579181 | 0,104279973 | |
| 203615_x_at | SULT1A1 | 1,462198073 | 1,3579181 | 0,104279973 | |
| 213494_s_at | YY1 | 1,462198073 | 1,3579181 | 0,104279973 | |
| 1553292_s_at | SGK494 | 0,841135803 | 0,73688026 | 0,104255543 | |
| 1555639_a_at | RBM14 | 0,841135803 | 0,73688026 | 0,104255543 | |
| 210544_s_at | ALDH3A2 | 0,841135803 | 0,73688026 | 0,104255543 | |
| 218673_s_at | ATG7 | 0,841135803 | 0,73688026 | 0,104255543 | |
| 222013_x_at | FAM86A | 0,841135803 | 0,73688026 | 0,104255543 | |
| 224479_s_at | MRPL45 | 3,649657172 | 3,545468469 | 0,104188704 | |
| 201978_s_at | KIAA0141 | 2,048758794 | 1,944570415 | 0,104188379 | |
| 218745_x_at | TMEM161A | 2,976955384 | 2,87289275 | 0,104062634 | |
| 200839_s_at | CTSB | 4,002215714 | 3,898162764 | 0,10405295 | |
| 1558147_a_at | FLJ90757 | 2,14450783 | 2,040470494 | 0,104037336 | |
| 223214_s_at | ZHX1 | 3,554701391 | 3,450709925 | 0,103991467 | |
| 227239_at | FAM126A | 4,365417393 | 4,261479522 | 0,103937871 | |
| 211318_s_at | RAE1 | 4,512927979 | 4,409010481 | 0,103917498 | |
| 218014_at | NUP85 | 3,914869478 | 3,810956512 | 0,103912966 | |
| 205543_at | HSPA4L | 3,101484927 | 2,997606395 | 0,103878531 | |
| 202114_at | SNX2 | 3,469688722 | 3,365837114 | 0,103851608 | |
| 227696_at | EXOSC6 | 2,763383302 | 2,659583735 | 0,103799567 | |
| 244786_at | SNHG10 | 1,104397092 | 1,000684521 | 0,103712571 | |
| 1555786_s_at | LINC00520 | 2,194816625 | 2,091116676 | 0,103699949 | |
| 1561061_at | LOC729506 | -0,543051993 | -0,646746079 | 0,103694085 | |
| 204462_s_at | SLC16A2 | -0,543051993 | -0,646746079 | 0,103694085 | |
| 206026_s_at | TNFAIP6 | -0,543051993 | -0,646746079 | 0,103694085 | |
| 212404_s_at | UBE3B | -0,543051993 | -0,646746079 | 0,103694085 | |
| 213618_at | ARAP2 | -0,543051993 | -0,646746079 | 0,103694085 | |
| 220529_at | FLJ11710 | -0,543051993 | -0,646746079 | 0,103694085 | |
| 221171_at | CCDC30 | -0,543051993 | -0,646746079 | 0,103694085 | |
| 225473_at | SOGA1 | -0,543051993 | -0,646746079 | 0,103694085 | |
| 228192_at | MNF1 | -0,543051993 | -0,646746079 | 0,103694085 | |
| 231145_at | PAX9 | -0,543051993 | -0,646746079 | 0,103694085 | |
| 243513_at | - | -0,543051993 | -0,646746079 | 0,103694085 | |
| 222417_s_at | SNX5 | 4,831095574 | 4,727463497 | 0,103632077 | |
| 201169_s_at | BHLHE40 | 1,473887017 | 1,370274469 | 0,103612548 | |
| 1556216_s_at | - | -0,946847173 | -1,050406615 | 0,103559442 | |
| 1556633_at | C1orf204 | -0,946847173 | -1,050406615 | 0,103559442 | |
| 1558856_at | DMRTA2 | -0,946847173 | -1,050406615 | 0,103559442 | |
| 204368_at | SLCO2A1 | -0,946847173 | -1,050406615 | 0,103559442 | |
| 206230_at | LHX1 | -0,946847173 | -1,050406615 | 0,103559442 | |
| 213956_at | CEP350 | -0,946847173 | -1,050406615 | 0,103559442 | |
| 220870_at | - | -0,946847173 | -1,050406615 | 0,103559442 | |
| 227588_s_at | GET4 | -0,946847173 | -1,050406615 | 0,103559442 | |
| 230436_s_at | CENPV | -0,946847173 | -1,050406615 | 0,103559442 | |
| 232577_at | LOC145945 | -0,946847173 | -1,050406615 | 0,103559442 | |
| 234211_at | - | -0,946847173 | -1,050406615 | 0,103559442 | |
| 236137_at | - | -0,946847173 | -1,050406615 | 0,103559442 | |
| 239261_s_at | CORIN | -0,946847173 | -1,050406615 | 0,103559442 | |
| 239660_at | RALGAPA2 | -0,946847173 | -1,050406615 | 0,103559442 | |
| 241153_at | - | -0,946847173 | -1,050406615 | 0,103559442 | |

| | | | | | |
|---|---|---|---|---|---|
| 243135_x_at | SNX8 | -0,946847173 | -1,050406615 | 0,103559442 | |
| 217814_at | CCDC47 | 3,811181587 | 3,707626508 | 0,103555079 | |
| 202382_s_at | GNPDA1 | 3,492853048 | 3,38929984 | 0,103553207 | |
| 224755_at | TM9SF3 | 3,421207626 | 3,317734938 | 0,103472688 | |
| 1553570_x_at | COX2 | 8,399953048 | 8,296512904 | 0,103440144 | |
| 1553906_s_at | FGD2 | 3,013454777 | 2,910041809 | 0,103412968 | |
| 207563_s_at | OGT | 2,109966786 | 2,006570641 | 0,103396145 | |
| 230352_at | PRPS2 | 3,285189903 | 3,181801492 | 0,103388412 | |
| 211972_x_at | RPLP0 | 7,843565687 | 7,740193324 | 0,103372363 | |
| 239134_at | - | 1,063722698 | 0,960386719 | 0,103335978 | |
| 226393_at | CYP2U1 | 1,326951551 | 1,223626587 | 0,103324963 | |
| 1553101_a_at | ALKBH5 /// LOC1 | 3,327680494 | 3,22439681 | 0,103283684 | |
| 200847_s_at | TMEM66 | 5,617673065 | 5,514416888 | 0,103256177 | |
| 206668_s_at | SCAMP1 | 0,480753764 | 0,377530647 | 0,103223117 | |
| 225808_at | METTL23 | 3,462861802 | 3,359653127 | 0,103208675 | |
| 206008_at | TGM1 | -0,033456564 | -0,136643983 | 0,103187418 | |
| 212321_at | SGPL1 | -0,033456564 | -0,136643983 | 0,103187418 | |
| 213268_at | CAMTA1 | -0,033456564 | -0,136643983 | 0,103187418 | |
| 221695_s_at | MAP3K2 | -0,033456564 | -0,136643983 | 0,103187418 | |
| 225595_at | CREBZF | -0,033456564 | -0,136643983 | 0,103187418 | |
| 208949_s_at | LGALS3 | 1,481627361 | 1,378453643 | 0,103173718 | |
| 228011_at | FAM92A1 | 1,481627361 | 1,378453643 | 0,103173718 | |
| 231718_at | SLU7 | 2,861566439 | 2,758473378 | 0,103093061 | |
| 223556_at | HELLS | 2,68509622 | 2,582026171 | 0,103070049 | |
| 202937_x_at | RRP7A | 2,292660284 | 2,189694216 | 0,102966068 | |
| 211058_x_at | LOC100288366 / | 7,744156625 | 7,641219915 | 0,102936711 | |
| 222792_s_at | CCDC59 | 4,912447057 | 4,809530416 | 0,102916641 | |
| 213063_at | ZC3H14 | 3,375201825 | 3,272369332 | 0,102832493 | |
| 202974_at | MPP1 | 5,148945274 | 5,046124219 | 0,102821055 | |
| 205812_s_at | TMED9 | 5,697733364 | 5,594934827 | 0,102798537 | |
| 1553362_at | DNAH6 | -3,07502285 | -3,177802286 | 0,102779436 | |
| 1560980_a_at | - | -3,07502285 | -3,177802286 | 0,102779436 | |
| 1561644_x_at | - | -3,07502285 | -3,177802286 | 0,102779436 | |
| 1561789_at | - | -3,07502285 | -3,177802286 | 0,102779436 | |
| 1569772_x_at | - | -3,07502285 | -3,177802286 | 0,102779436 | |
| 206426_at | MLANA | -3,07502285 | -3,177802286 | 0,102779436 | |
| 206664_at | SI | -3,07502285 | -3,177802286 | 0,102779436 | |
| 217194_at | - | -3,07502285 | -3,177802286 | 0,102779436 | |
| 224101_x_at | MRS2 | -3,07502285 | -3,177802286 | 0,102779436 | |
| 235801_at | TUSC3 | -3,07502285 | -3,177802286 | 0,102779436 | |
| 236277_at | - | -3,07502285 | -3,177802286 | 0,102779436 | |
| 237608_at | - | -3,07502285 | -3,177802286 | 0,102779436 | |
| 239317_at | CEACAM21 | -3,07502285 | -3,177802286 | 0,102779436 | |
| 203348_s_at | ETV5 | 1,751865623 | 1,64909809 | 0,102767532 | |
| 226516_at | MFSD12 | 1,81142822 | 1,708698295 | 0,102729925 | |
| 200810_s_at | CIRBP | 4,685380613 | 4,582769126 | 0,102611487 | |
| 227668_at | C17orf56 | 1,339784194 | 1,23718272 | 0,102601475 | |
| 218295_s_at | NUP50 | 2,2992452 | 2,196649505 | 0,102595696 | |
| 221887_s_at | DFNB31 | 0,87091093 | 0,768343791 | 0,102567139 | |
| 230884_s_at | SPG7 | 0,87091093 | 0,768343791 | 0,102567139 | |
| 204663_at | ME3 | 1,493160564 | 1,390636111 | 0,102524453 | |
| 211849_s_at | RNGTT | 1,493160564 | 1,390636111 | 0,102524453 | |
| 212509_s_at | MXRA7 | 3,438212789 | 3,335699198 | 0,102513591 | |
| 223494_at | MGEA5 | 3,512821151 | 3,410394103 | 0,102427048 | |
| 222604_at | GTF3C3 | 2,173470163 | 2,071071048 | 0,102399115 | |
| 210784_x_at | LILRA6 /// LILRB3 | 0,496120222 | 0,393761504 | 0,102358718 | |

| | | | | | |
|---|---|---|---|---|---|
| 237287_at | HMGA1P4 | 0,496120222 | 0,393761504 | 0,102358718 | |
| 242466_at | - | 0,496120222 | 0,393761504 | 0,102358718 | |
| 200777_s_at | BZW1 | 5,795096834 | 5,692776415 | 0,10232042 | |
| 223195_s_at | SESN2 | 2,082235068 | 1,979940779 | 0,102294289 | |
| 214398_s_at | IKBKE | 0,760715727 | 0,658444279 | 0,102271448 | |
| 226292_at | CAPN5 | 0,760715727 | 0,658444279 | 0,102271448 | |
| 224748_at | DCAF7 | 4,305079945 | 4,202810939 | 0,102269006 | |
| 201546_at | TRIP12 | 4,937989849 | 4,835756686 | 0,102233162 | |
| 212931_at | TCF20 | 1,820612054 | 1,718396606 | 0,102215448 | |
| 227649_s_at | SRGAP2 | 1,820612054 | 1,718396606 | 0,102215448 | |
| 205324_s_at | FTSJ1 | 4,326728025 | 4,224525141 | 0,102202884 | |
| 1555789_s_at | PHF23 | 3,132544588 | 3,030417996 | 0,102126592 | |
| 203318_s_at | ZNF148 | 1,348276312 | 1,246149888 | 0,102126423 | |
| 211924_s_at | PLAUR | 1,348276312 | 1,246149888 | 0,102126423 | |
| 1568869_at | - | -0,512251684 | -0,614371577 | 0,102119892 | |
| 206324_s_at | DAPK2 | -0,512251684 | -0,614371577 | 0,102119892 | |
| 210711_at | LINC00260 | -0,512251684 | -0,614371577 | 0,102119892 | |
| 216082_at | NEU3 | -0,512251684 | -0,614371577 | 0,102119892 | |
| 219249_s_at | FKBP10 | -0,512251684 | -0,614371577 | 0,102119892 | |
| 221183_at | LOC100507388 | -0,512251684 | -0,614371577 | 0,102119892 | |
| 223504_at | DNAJC27 | -0,512251684 | -0,614371577 | 0,102119892 | |
| 232724_at | MS4A6A | -0,512251684 | -0,614371577 | 0,102119892 | |
| 234452_at | HIBADH | -0,512251684 | -0,614371577 | 0,102119892 | |
| 239086_at | - | -0,512251684 | -0,614371577 | 0,102119892 | |
| 239842_x_at | - | -0,512251684 | -0,614371577 | 0,102119892 | |
| 241173_at | - | -0,512251684 | -0,614371577 | 0,102119892 | |
| 242156_at | - | -0,512251684 | -0,614371577 | 0,102119892 | |
| 227229_at | VPS53 | 1,426552034 | 1,324440845 | 0,102111189 | |
| 212552_at | HPCAL1 | 4,43733059 | 4,335292864 | 0,102037726 | |
| 1555989_at | - | 2,038300149 | 1,936283387 | 0,102016762 | |
| 217301_x_at | RBBP4 | 5,111084956 | 5,009069778 | 0,102015177 | |
| 1557605_a_at | LOC401312 | 0,181384709 | 0,079428135 | 0,101956574 | |
| 228960_at | NARG2 | 0,181384709 | 0,079428135 | 0,101956574 | |
| 202849_x_at | GRK6 | 2,705069849 | 2,603119255 | 0,101950595 | |
| 219877_at | ZMAT4 | 0,351548291 | 0,249604176 | 0,101944115 | |
| 236119_s_at | SPRR2G | 0,351548291 | 0,249604176 | 0,101944115 | |
| 1552664_at | FLCN | 1,089278259 | 0,987376598 | 0,101901662 | |
| 1553858_at | ZBTB3 | 1,089278259 | 0,987376598 | 0,101901662 | |
| 224180_x_at | WDPCP | 1,089278259 | 0,987376598 | 0,101901662 | |
| 227006_at | PPP1R14A | 3,183265014 | 3,081412119 | 0,101852895 | |
| 211284_s_at | GRN | 2,912562019 | 2,810785576 | 0,101776443 | |
| 200973_s_at | TSPAN3 | 3,829494273 | 3,727773127 | 0,101721146 | |
| 227187_at | CBLL1 | 2,710020346 | 2,608344689 | 0,101675657 | |
| 212003_at | C1orf144 | 2,678376417 | 2,576704359 | 0,101672058 | |
| 202550_s_at | VAPB | 3,678566904 | 3,576905405 | 0,101661499 | |
| 228542_at | MRS2 | 2,544366685 | 2,442710777 | 0,101655908 | |
| 224647_at | CCNY | 4,406149819 | 4,304503145 | 0,101646674 | |
| 207524_at | ST7 /// ST7-OT3 | 0,888485433 | 0,786897721 | 0,101587712 | |
| 243188_at | ZNF283 | 0,888485433 | 0,786897721 | 0,101587712 | |
| 203015_s_at | SSX2IP | 1,995690482 | 1,894118818 | 0,101571664 | |
| 201111_at | CSE1L | 5,089402992 | 4,987842433 | 0,101560558 | |
| 202636_at | RNF103 | 2,580856819 | 2,479296438 | 0,101560381 | |
| 227864_s_at | FAM125A | 2,774453351 | 2,672945617 | 0,101507734 | |
| 215208_x_at | RPL35A | 1,512180019 | 1,410714371 | 0,101465647 | |
| 226040_at | - | 1,191926657 | 1,090553537 | 0,10137312 | |
| 1552995_at | IL27 | -0,00073712 | -0,102100538 | 0,101363417 | |

| | | | | | |
|---|---|---|---|---|---|
| 208100_x_at | SEMA6C | -0,00073712 | -0,102100538 | 0,101363417 | |
| 211405_x_at | IFNA17 | -0,00073712 | -0,102100538 | 0,101363417 | |
| 211506_s_at | IL8 | -0,00073712 | -0,102100538 | 0,101363417 | |
| 229411_at | PNCK | -0,00073712 | -0,102100538 | 0,101363417 | |
| 233888_s_at | SRGAP1 | -0,00073712 | -0,102100538 | 0,101363417 | |
| 239783_at | - | -0,00073712 | -0,102100538 | 0,101363417 | |
| 225227_at | SKIL | 3,279650757 | 3,178292062 | 0,101358695 | |
| 226597_at | REEP6 | 2,146943689 | 2,045615873 | 0,101327817 | |
| 202104_s_at | SPG7 | 2,478200152 | 2,376875125 | 0,101325027 | |
| 203714_s_at | TBCE | 3,949468585 | 3,848241888 | 0,101226696 | |
| 205474_at | CRLF3 | 3,101484927 | 3,000258961 | 0,101225966 | |
| 201447_at | TIA1 | 2,922548342 | 2,821324225 | 0,101224117 | |
| 1559582_at | RHOQ | -0,221656139 | -0,322870281 | 0,101214143 | |
| 213539_at | CD3D | -0,221656139 | -0,322870281 | 0,101214143 | |
| 219318_x_at | MED31 | -0,221656139 | -0,322870281 | 0,101214143 | |
| 227365_at | ATCAY | -0,221656139 | -0,322870281 | 0,101214143 | |
| 233673_at | - | -0,221656139 | -0,322870281 | 0,101214143 | |
| 234528_at | - | -0,221656139 | -0,322870281 | 0,101214143 | |
| 235865_at | CELF1 | -0,221656139 | -0,322870281 | 0,101214143 | |
| 242376_at | - | -0,221656139 | -0,322870281 | 0,101214143 | |
| 211676_s_at | IFNGR1 | 3,054253462 | 2,953119918 | 0,101133544 | |
| 223513_at | CENPJ | 2,840652551 | 2,739547836 | 0,101104715 | |
| 226464_at | C3orf58 | 4,527957295 | 4,426866007 | 0,101091288 | |
| 223040_at | NAA20 | 5,091933607 | 4,990846468 | 0,101087139 | |
| 208645_s_at | RPS14 | 8,023970784 | 7,9229005 | 0,101070284 | |
| 206790_s_at | NDUFB1 | 6,010044129 | 5,908998287 | 0,101045842 | |
| 234762_x_at | NLN | 2,926807124 | 2,825817328 | 0,100989795 | |
| 213775_x_at | ZNF638 | 4,710299868 | 4,609508596 | 0,100791272 | |
| 218527_at | APTX | 2,726400382 | 2,625627364 | 0,100773018 | |
| 223032_x_at | PRELID1 | 5,637729671 | 5,536971059 | 0,100758613 | |
| 208811_s_at | DNAJB6 /// TMEM | 4,654470045 | 4,553720057 | 0,100749988 | |
| 212522_at | PDE8A | 1,373456798 | 1,272721751 | 0,100735047 | |
| 225129_at | CPNE2 | 1,373456798 | 1,272721751 | 0,100735047 | |
| 1555864_s_at | PDHA1 | 5,265058615 | 5,164369044 | 0,100689571 | |
| 229587_at | UBA2 | 2,790121796 | 2,689475799 | 0,100645997 | |
| 242195_x_at | NUMBL | 2,933877275 | 2,833274877 | 0,100602398 | |
| 219069_at | ANKRD49 | 3,228817493 | 3,128242026 | 0,100575467 | |
| 1552329_at | RBBP6 | 1,114389003 | 1,013870809 | 0,100518194 | |
| 201103_x_at | LOC100506032 / | 5,422384122 | 5,321896852 | 0,10048727 | |
| 210174_at | NR5A2 | 2,700102307 | 2,599625088 | 0,100477219 | |
| 222239_s_at | INTS6 | 1,732533761 | 1,632095957 | 0,100437804 | |
| 218684_at | LRRC8D | 5,38907564 | 5,288648454 | 0,100427185 | |
| 210423_s_at | SLC11A1 | 0,209623097 | 0,109221392 | 0,100401705 | |
| 224088_at | NMUR2 | 0,209623097 | 0,109221392 | 0,100401705 | |
| 227504_s_at | - | 0,209623097 | 0,109221392 | 0,100401705 | |
| 200043_at | ERH | 6,924093067 | 6,823703003 | 0,100390064 | |
| 233827_s_at | SUPT16H | 3,276317033 | 3,175947691 | 0,100369342 | |
| 214965_at | SPATA2L | 1,912238072 | 1,811954245 | 0,100283827 | |
| 235574_at | GBP4 | 1,912238072 | 1,811954245 | 0,100283827 | |
| 227541_at | WDR20 | 2,11992033 | 2,019703483 | 0,100216847 | |
| 1555447_at | GPR114 | 0,020670649 | -0,079522948 | 0,100193598 | |
| 1569501_at | - | 0,020670649 | -0,079522948 | 0,100193598 | |
| 240229_at | - | 0,020670649 | -0,079522948 | 0,100193598 | |
| 243715_at | - | 0,020670649 | -0,079522948 | 0,100193598 | |
| 218795_at | ACP6 | 3,046446467 | 2,946258252 | 0,100188215 | |
| 228676_at | ORAOV1 | 1,021868205 | 0,921731975 | 0,10013623 | |

| | | | | | |
|---|---|---|---|---|---|
| 227304_at | SMCR8 | 1,607231019 | 1,507116528 | 0,100114492 | |
| 229074_at | EHD4 | 1,607231019 | 1,507116528 | 0,100114492 | |
| 229618_at | SNX16 | 1,607231019 | 1,507116528 | 0,100114492 | |
| 212082_s_at | MYL6 | 6,889418081 | 6,78932054 | 0,100097542 | |
| 218569_s_at | KBTBD4 | 1,215326747 | 1,115234684 | 0,100092063 | |
| 1553138_a_at | ANKLE1 | 1,859746653 | 1,759685182 | 0,100061471 | |
| 228007_at | CEP85L | 2,573632254 | 2,473581079 | 0,100051175 | |
| 230223_at | ZNF839 | 0,917308734 | 0,817300337 | 0,100008397 | |
| 218856_at | TNFRSF21 | 2,802535172 | 2,702564916 | 0,099970256 | |
| 229732_at | ZNF823 | 3,693640704 | 3,593688577 | 0,099952127 | |
| 210759_s_at | PSMA1 | 5,178117298 | 5,078176444 | 0,099940854 | |
| 222754_at | TRNT1 | 3,467741468 | 3,367892567 | 0,099848901 | |
| 1554828_at | PDGFRA | -1,937711681 | -2,037551727 | 0,099840045 | |
| 1555144_at | ARL17A /// ARL1 | -1,937711681 | -2,037551727 | 0,099840045 | |
| 1555798_at | LOC400692 | -1,937711681 | -2,037551727 | 0,099840045 | |
| 1557504_at | - | -1,937711681 | -2,037551727 | 0,099840045 | |
| 1561469_at | - | -1,937711681 | -2,037551727 | 0,099840045 | |
| 1561757_a_at | LOC283352 | -1,937711681 | -2,037551727 | 0,099840045 | |
| 1562280_at | - | -1,937711681 | -2,037551727 | 0,099840045 | |
| 1566141_at | - | -1,937711681 | -2,037551727 | 0,099840045 | |
| 1570087_at | - | -1,937711681 | -2,037551727 | 0,099840045 | |
| 207575_at | GOLGA6A | -1,937711681 | -2,037551727 | 0,099840045 | |
| 213802_at | PRSS12 | -1,937711681 | -2,037551727 | 0,099840045 | |
| 215378_at | - | -1,937711681 | -2,037551727 | 0,099840045 | |
| 217504_at | ABCA6 | -1,937711681 | -2,037551727 | 0,099840045 | |
| 219671_at | HPCAL4 | -1,937711681 | -2,037551727 | 0,099840045 | |
| 220527_at | MRPL20 | -1,937711681 | -2,037551727 | 0,099840045 | |
| 223999_at | PPIL2 | -1,937711681 | -2,037551727 | 0,099840045 | |
| 224488_s_at | SPON1 | -1,937711681 | -2,037551727 | 0,099840045 | |
| 231588_at | PRCP | -1,937711681 | -2,037551727 | 0,099840045 | |
| 233225_at | - | -1,937711681 | -2,037551727 | 0,099840045 | |
| 234013_at | YME1L1 | -1,937711681 | -2,037551727 | 0,099840045 | |
| 235230_at | PLCXD2 | -1,937711681 | -2,037551727 | 0,099840045 | |
| 236002_at | - | -1,937711681 | -2,037551727 | 0,099840045 | |
| 236939_at | PTPLAD2 | -1,937711681 | -2,037551727 | 0,099840045 | |
| 239338_x_at | - | -1,937711681 | -2,037551727 | 0,099840045 | |
| 239567_at | - | -1,937711681 | -2,037551727 | 0,099840045 | |
| 1555542_at | AFAP1L1 | -0,85085127 | -0,950686014 | 0,099834745 | |
| 1560489_at | - | -0,85085127 | -0,950686014 | 0,099834745 | |
| 205674_x_at | FXYD2 | -0,85085127 | -0,950686014 | 0,099834745 | |
| 219431_at | ARHGAP10 | -0,85085127 | -0,950686014 | 0,099834745 | |
| 221103_s_at | WDR52 | -0,85085127 | -0,950686014 | 0,099834745 | |
| 223586_at | ARNTL2 | -0,85085127 | -0,950686014 | 0,099834745 | |
| 228584_at | SGCB | -0,85085127 | -0,950686014 | 0,099834745 | |
| 237255_at | LOC100506115 | -0,85085127 | -0,950686014 | 0,099834745 | |
| 239845_at | - | -0,85085127 | -0,950686014 | 0,099834745 | |
| 242848_x_at | - | -0,85085127 | -0,950686014 | 0,099834745 | |
| 224869_s_at | MRPS25 | 3,920575295 | 3,820743792 | 0,099831503 | |
| 1555906_s_at | C3orf23 | 1,542099044 | 1,442269454 | 0,09982959 | |
| 236198_at | - | 1,542099044 | 1,442269454 | 0,09982959 | |
| 207412_x_at | CELP | -0,467091869 | -0,566887459 | 0,099795591 | |
| 216983_s_at | ZNF224 | -0,467091869 | -0,566887459 | 0,099795591 | |
| 217227_x_at | IGLV1-44 | -0,467091869 | -0,566887459 | 0,099795591 | |
| 221546_at | PRPF18 | -0,467091869 | -0,566887459 | 0,099795591 | |
| 221731_x_at | VCAN | -0,467091869 | -0,566887459 | 0,099795591 | |
| 224145_s_at | SPTBN4 | -0,467091869 | -0,566887459 | 0,099795591 | |

| | | | | | |
|---|---|---|---|---|---|
| 230462_at | NUMB | -0,467091869 | -0,566887459 | 0,099795591 | |
| 231451_s_at | - | -0,467091869 | -0,566887459 | 0,099795591 | |
| 238106_at | - | -0,467091869 | -0,566887459 | 0,099795591 | |
| 218855_at | TPRA1 | 1,865674241 | 1,765933685 | 0,099740556 | |
| 201373_at | PLEC | 1,129248302 | 1,029536893 | 0,099711409 | |
| 218201_at | NDUFB2 | 5,830001953 | 5,730307495 | 0,099694458 | |
| 214170_x_at | FH | 4,402586695 | 4,302892559 | 0,099694136 | |
| 224641_at | FYTTD1 | 4,055697197 | 3,956010056 | 0,09968714 | |
| 1552523_a_at | TIGD4 | -1,812418401 | -1,912089812 | 0,099671411 | |
| 1555602_a_at | ELAVL3 | -1,812418401 | -1,912089812 | 0,099671411 | |
| 1562693_at | - | -1,812418401 | -1,912089812 | 0,099671411 | |
| 1563023_at | - | -1,812418401 | -1,912089812 | 0,099671411 | |
| 1570339_x_at | - | -1,812418401 | -1,912089812 | 0,099671411 | |
| 204581_at | CD22 | -1,812418401 | -1,912089812 | 0,099671411 | |
| 204851_s_at | DCX | -1,812418401 | -1,912089812 | 0,099671411 | |
| 205509_at | CPB1 | -1,812418401 | -1,912089812 | 0,099671411 | |
| 206276_at | LY6D | -1,812418401 | -1,912089812 | 0,099671411 | |
| 207751_at | - | -1,812418401 | -1,912089812 | 0,099671411 | |
| 211138_s_at | KMO | -1,812418401 | -1,912089812 | 0,099671411 | |
| 213744_at | ATRNL1 | -1,812418401 | -1,912089812 | 0,099671411 | |
| 215612_at | - | -1,812418401 | -1,912089812 | 0,099671411 | |
| 215934_at | - | -1,812418401 | -1,912089812 | 0,099671411 | |
| 221030_s_at | ARHGAP24 | -1,812418401 | -1,912089812 | 0,099671411 | |
| 221053_s_at | TDRKH | -1,812418401 | -1,912089812 | 0,099671411 | |
| 235928_at | - | -1,812418401 | -1,912089812 | 0,099671411 | |
| 210163_at | CXCL11 | 2,352981171 | 2,253342112 | 0,099639059 | |
| 201175_at | TMX2 | 4,764049426 | 4,66447467 | 0,099574756 | |
| 233320_at | TCAM1P | 1,748661581 | 1,64909809 | 0,099563491 | |
| 224758_at | C7orf73 | 2,582657321 | 2,483094137 | 0,099563184 | |
| 1556789_a_at | GCFC1-AS1 | -2,063149277 | -2,162639771 | 0,099490494 | |
| 1561761_x_at | LOC100507322 / | -2,063149277 | -2,162639771 | 0,099490494 | |
| 1563882_a_at | RAPGEF5 | -2,063149277 | -2,162639771 | 0,099490494 | |
| 1566715_at | - | -2,063149277 | -2,162639771 | 0,099490494 | |
| 1569620_s_at | LOC100128079 | -2,063149277 | -2,162639771 | 0,099490494 | |
| 207694_at | POU3F4 | -2,063149277 | -2,162639771 | 0,099490494 | |
| 209800_at | KRT16 | -2,063149277 | -2,162639771 | 0,099490494 | |
| 214304_x_at | - | -2,063149277 | -2,162639771 | 0,099490494 | |
| 216494_at | - | -2,063149277 | -2,162639771 | 0,099490494 | |
| 216517_at | IGKC | -2,063149277 | -2,162639771 | 0,099490494 | |
| 216884_at | PTPN12 | -2,063149277 | -2,162639771 | 0,099490494 | |
| 219584_at | PLA1A | -2,063149277 | -2,162639771 | 0,099490494 | |
| 221840_at | PTPRE | -2,063149277 | -2,162639771 | 0,099490494 | |
| 224170_s_at | TULP4 | -2,063149277 | -2,162639771 | 0,099490494 | |
| 227359_at | OSCP1 | -2,063149277 | -2,162639771 | 0,099490494 | |
| 229667_s_at | HOXB8 | -2,063149277 | -2,162639771 | 0,099490494 | |
| 231179_at | IP6K3 | -2,063149277 | -2,162639771 | 0,099490494 | |
| 231235_at | NKTR | -2,063149277 | -2,162639771 | 0,099490494 | |
| 231373_at | RNF133 | -2,063149277 | -2,162639771 | 0,099490494 | |
| 233316_at | - | -2,063149277 | -2,162639771 | 0,099490494 | |
| 233383_at | - | -2,063149277 | -2,162639771 | 0,099490494 | |
| 236047_at | XKR6 | -2,063149277 | -2,162639771 | 0,099490494 | |
| 236113_at | - | -2,063149277 | -2,162639771 | 0,099490494 | |
| 236534_at | BNIPL | -2,063149277 | -2,162639771 | 0,099490494 | |
| 238989_at | C1GALT1C1 | -2,063149277 | -2,162639771 | 0,099490494 | |
| 240997_at | - | -2,063149277 | -2,162639771 | 0,099490494 | |
| 214354_x_at | SFTPB | 1,314003737 | 1,214517879 | 0,099485858 | |

| | | | | | |
|---|---|---|---|---|---|
| 228583_at | LIN52 | 2,512607472 | 2,413157372 | 0,0994501 | |
| 219828_at | RABL6 | 3,158127685 | 3,058687544 | 0,099440141 | |
| 217741_s_at | ZFAND5 | 3,602516743 | 3,503081281 | 0,099435462 | |
| 200057_s_at | NONO | 7,10590457 | 7,006489352 | 0,099415218 | |
| 211172_x_at | AKAP7 | 0,228146222 | 0,128747141 | 0,099399081 | |
| 214617_at | PRF1 | 0,228146222 | 0,128747141 | 0,099399081 | |
| 217576_x_at | SOS2 | 0,228146222 | 0,128747141 | 0,099399081 | |
| 1553586_at | FBXL19-AS1 | -1,763254337 | -1,862647763 | 0,099393426 | |
| 1558118_at | LOC100287576 / | -1,763254337 | -1,862647763 | 0,099393426 | |
| 1558868_a_at | DSE | -1,763254337 | -1,862647763 | 0,099393426 | |
| 1565150_at | - | -1,763254337 | -1,862647763 | 0,099393426 | |
| 1568782_at | RP2 | -1,763254337 | -1,862647763 | 0,099393426 | |
| 206700_s_at | KDM5D | -1,763254337 | -1,862647763 | 0,099393426 | |
| 213695_at | PON3 | -1,763254337 | -1,862647763 | 0,099393426 | |
| 214577_at | MAP1B | -1,763254337 | -1,862647763 | 0,099393426 | |
| 215289_at | ZNF749 | -1,763254337 | -1,862647763 | 0,099393426 | |
| 220686_s_at | PIWIL2 | -1,763254337 | -1,862647763 | 0,099393426 | |
| 227848_at | PEBP4 | -1,763254337 | -1,862647763 | 0,099393426 | |
| 232842_at | DOCK8 | -1,763254337 | -1,862647763 | 0,099393426 | |
| 234422_at | - | -1,763254337 | -1,862647763 | 0,099393426 | |
| 234840_s_at | OR5V1 | -1,763254337 | -1,862647763 | 0,099393426 | |
| 236586_at | - | -1,763254337 | -1,862647763 | 0,099393426 | |
| 236657_at | LOC100288911 | -1,763254337 | -1,862647763 | 0,099393426 | |
| 239271_at | SMAD2 | -1,763254337 | -1,862647763 | 0,099393426 | |
| 240808_at | ESD | -1,763254337 | -1,862647763 | 0,099393426 | |
| 241966_at | MYO5A | -1,763254337 | -1,862647763 | 0,099393426 | |
| 242658_at | - | -1,763254337 | -1,862647763 | 0,099393426 | |
| 243231_at | SLC38A11 | -1,763254337 | -1,862647763 | 0,099393426 | |
| 231763_at | POLR3A | 2,783874823 | 2,684536601 | 0,099338222 | |
| 1555910_at | PTCD2 | 1,689737652 | 1,590454215 | 0,099283436 | |
| 201258_at | RPS16 | 7,931053997 | 7,831787108 | 0,099266889 | |
| 218465_at | TMEM33 | 3,38038663 | 3,281121096 | 0,099265535 | |
| 215091_s_at | GTF3A | 6,06790868 | 5,968708536 | 0,099200144 | |
| 213091_at | CRTC1 | 1,40228911 | 1,303116084 | 0,099173025 | |
| 209278_s_at | TFPI2 | 6,490407486 | 6,391269595 | 0,099137892 | |
| 1557325_at | - | 0,555999837 | 0,456918297 | 0,09908154 | |
| 211970_x_at | ACTG1 | 7,821863345 | 7,722782559 | 0,099080786 | |
| 212672_at | ATM | 1,758252436 | 1,659204017 | 0,09904842 | |
| 229826_at | C3orf78 | 2,758612869 | 2,659583735 | 0,099029133 | |
| 201068_s_at | PSMC2 | 6,098748309 | 5,999775868 | 0,098972441 | |
| 1554127_s_at | MSRB3 | -1,688127507 | -1,787089803 | 0,098962296 | |
| 1554708_s_at | SPATA6L | -1,688127507 | -1,787089803 | 0,098962296 | |
| 1556740_at | EGFLAM-AS2 | -1,688127507 | -1,787089803 | 0,098962296 | |
| 1557193_at | - | -1,688127507 | -1,787089803 | 0,098962296 | |
| 1558859_at | LOC401320 | -1,688127507 | -1,787089803 | 0,098962296 | |
| 1564227_at | - | -1,688127507 | -1,787089803 | 0,098962296 | |
| 1569376_s_at | - | -1,688127507 | -1,787089803 | 0,098962296 | |
| 201372_s_at | CUL3 | -1,688127507 | -1,787089803 | 0,098962296 | |
| 206708_at | FOXN2 | -1,688127507 | -1,787089803 | 0,098962296 | |
| 210965_x_at | CDK13 | -1,688127507 | -1,787089803 | 0,098962296 | |
| 216847_at | - | -1,688127507 | -1,787089803 | 0,098962296 | |
| 220997_s_at | DIAPH3 | -1,688127507 | -1,787089803 | 0,098962296 | |
| 225626_at | PAG1 | -1,688127507 | -1,787089803 | 0,098962296 | |
| 228057_at | DDIT4L | -1,688127507 | -1,787089803 | 0,098962296 | |
| 229523_at | - | -1,688127507 | -1,787089803 | 0,098962296 | |
| 230547_at | KCNC1 | -1,688127507 | -1,787089803 | 0,098962296 | |

| | | | | | |
|---|---|---|---|---|---|
| 232107_at | - | -1,688127507 | -1,787089803 | 0,098962296 | |
| 232588_at | STAG1 | -1,688127507 | -1,787089803 | 0,098962296 | |
| 234962_at | - | -1,688127507 | -1,787089803 | 0,098962296 | |
| 236187_s_at | - | -1,688127507 | -1,787089803 | 0,098962296 | |
| 239173_at | INADL | -1,688127507 | -1,787089803 | 0,098962296 | |
| 239746_at | MESDC2 | -1,688127507 | -1,787089803 | 0,098962296 | |
| 240486_at | HELZ | -1,688127507 | -1,787089803 | 0,098962296 | |
| 242993_at | - | -1,688127507 | -1,787089803 | 0,098962296 | |
| 243995_at | PTAR1 | -1,688127507 | -1,787089803 | 0,098962296 | |
| 218268_at | TBC1D15 | 3,236843482 | 3,137910193 | 0,098933289 | |
| 212094_at | PEG10 | 2,14450783 | 2,045615873 | 0,098891957 | |
| 210449_x_at | MAPK14 | 1,560488174 | 1,461646549 | 0,098841625 | |
| 218296_x_at | MSTO1 /// MSTO | 1,560488174 | 1,461646549 | 0,098841625 | |
| 1552870_s_at | AXDND1 | -3,212234735 | -3,311072652 | 0,098837917 | |
| 1559353_at | - | -3,212234735 | -3,311072652 | 0,098837917 | |
| 1559545_at | LOC100506948 / | -3,212234735 | -3,311072652 | 0,098837917 | |
| 1562301_at | C8orf34 | -3,212234735 | -3,311072652 | 0,098837917 | |
| 1570244_at | - | -3,212234735 | -3,311072652 | 0,098837917 | |
| 236995_x_at | TFEC | -3,212234735 | -3,311072652 | 0,098837917 | |
| 203137_at | WTAP | 5,606212153 | 5,507441463 | 0,09877069 | |
| 209512_at | HSDL2 | 1,04816921 | 0,949447784 | 0,098721427 | |
| 209380_s_at | ABCC5 | 3,469688722 | 3,370970266 | 0,098718457 | |
| 212195_at | IL6ST | 5,612394725 | 5,513720861 | 0,098673863 | |
| 1552673_at | RFX6 | -2,187914861 | -2,286583451 | 0,098668589 | |
| 1554779_s_at | PHLDB2 | -2,187914861 | -2,286583451 | 0,098668589 | |
| 1557222_at | - | -2,187914861 | -2,286583451 | 0,098668589 | |
| 1559597_at | - | -2,187914861 | -2,286583451 | 0,098668589 | |
| 1562384_at | - | -2,187914861 | -2,286583451 | 0,098668589 | |
| 1562457_at | NCKAP5L | -2,187914861 | -2,286583451 | 0,098668589 | |
| 1568887_at | - | -2,187914861 | -2,286583451 | 0,098668589 | |
| 1569573_at | - | -2,187914861 | -2,286583451 | 0,098668589 | |
| 204959_at | MNDA | -2,187914861 | -2,286583451 | 0,098668589 | |
| 206385_s_at | ANK3 | -2,187914861 | -2,286583451 | 0,098668589 | |
| 206651_s_at | CPB2 | -2,187914861 | -2,286583451 | 0,098668589 | |
| 207197_at | ZIC3 | -2,187914861 | -2,286583451 | 0,098668589 | |
| 209687_at | CXCL12 | -2,187914861 | -2,286583451 | 0,098668589 | |
| 209830_s_at | SLC9A3R2 | -2,187914861 | -2,286583451 | 0,098668589 | |
| 212344_at | SULF1 | -2,187914861 | -2,286583451 | 0,098668589 | |
| 216850_at | LOC100506948 / | -2,187914861 | -2,286583451 | 0,098668589 | |
| 224495_at | TMEM107 | -2,187914861 | -2,286583451 | 0,098668589 | |
| 226769_at | FIBIN | -2,187914861 | -2,286583451 | 0,098668589 | |
| 228086_at | STK33 | -2,187914861 | -2,286583451 | 0,098668589 | |
| 229979_x_at | - | -2,187914861 | -2,286583451 | 0,098668589 | |
| 230366_at | LOC100505683 | -2,187914861 | -2,286583451 | 0,098668589 | |
| 231350_at | - | -2,187914861 | -2,286583451 | 0,098668589 | |
| 231358_at | MRO | -2,187914861 | -2,286583451 | 0,098668589 | |
| 232651_at | LOC100287813 | -2,187914861 | -2,286583451 | 0,098668589 | |
| 234385_at | - | -2,187914861 | -2,286583451 | 0,098668589 | |
| 239900_x_at | - | -2,187914861 | -2,286583451 | 0,098668589 | |
| 242117_at | - | -2,187914861 | -2,286583451 | 0,098668589 | |
| 242331_x_at | LOC642236 | -2,187914861 | -2,286583451 | 0,098668589 | |
| 242432_at | LRRC37A2 | -2,187914861 | -2,286583451 | 0,098668589 | |
| 242899_at | SESN3 | -2,187914861 | -2,286583451 | 0,098668589 | |
| 243155_at | - | -2,187914861 | -2,286583451 | 0,098668589 | |
| 243854_at | - | -2,187914861 | -2,286583451 | 0,098668589 | |
| 229083_at | - | 2,52764011 | 2,428994491 | 0,09864562 | |

| | | | | | |
|---|---|---|---|---|---|
| 1552873_s_at | ASMTL-AS1 | 0,702292136 | 0,603679647 | 0,098612489 | |
| 218812_s_at | ORAI2 | 0,702292136 | 0,603679647 | 0,098612489 | |
| 202401_s_at | SRF | 2,600539826 | 2,501934108 | 0,098605717 | |
| 202413_s_at | USP1 | 4,96550526 | 4,866974944 | 0,098530315 | |
| 223382_s_at | ZNRF1 | 2,566371329 | 2,467842989 | 0,09852834 | |
| 243852_at | LUC7L2 | 3,102740237 | 3,004228687 | 0,098511551 | |
| 1553843_at | C10orf67 | 0,052199481 | -0,046302147 | 0,098501627 | |
| 203421_at | TP53I11 | 0,052199481 | -0,046302147 | 0,098501627 | |
| 206553_at | OAS2 | 0,052199481 | -0,046302147 | 0,098501627 | |
| 221119_at | ARHGEF38 | 0,052199481 | -0,046302147 | 0,098501627 | |
| 244409_at | CCDC154 | 0,052199481 | -0,046302147 | 0,098501627 | |
| 209189_at | FOS | 0,945567441 | 0,847075464 | 0,098491978 | |
| 213878_at | PYROXD1 | 0,945567441 | 0,847075464 | 0,098491978 | |
| 215412_x_at | LOC100132832 / | 0,945567441 | 0,847075464 | 0,098491978 | |
| 226591_at | LOC100506965 | 0,945567441 | 0,847075464 | 0,098491978 | |
| 1553285_s_at | RAD9B | -1,639579028 | -1,738067356 | 0,098488328 | |
| 1562007_at | - | -1,639579028 | -1,738067356 | 0,098488328 | |
| 1562682_at | RORA | -1,639579028 | -1,738067356 | 0,098488328 | |
| 1567527_at | - | -1,639579028 | -1,738067356 | 0,098488328 | |
| 1569944_at | - | -1,639579028 | -1,738067356 | 0,098488328 | |
| 208442_s_at | ATM | -1,639579028 | -1,738067356 | 0,098488328 | |
| 208797_s_at | GOLGA8A | -1,639579028 | -1,738067356 | 0,098488328 | |
| 216416_at | - | -1,639579028 | -1,738067356 | 0,098488328 | |
| 226490_at | NHSL1 | -1,639579028 | -1,738067356 | 0,098488328 | |
| 228390_at | RAB30 | -1,639579028 | -1,738067356 | 0,098488328 | |
| 228973_at | DLG2 | -1,639579028 | -1,738067356 | 0,098488328 | |
| 231601_at | LOC100507224 | -1,639579028 | -1,738067356 | 0,098488328 | |
| 232378_at | SLC5A9 | -1,639579028 | -1,738067356 | 0,098488328 | |
| 232512_at | - | -1,639579028 | -1,738067356 | 0,098488328 | |
| 233831_at | LOC100291666 | -1,639579028 | -1,738067356 | 0,098488328 | |
| 234553_at | - | -1,639579028 | -1,738067356 | 0,098488328 | |
| 235563_at | GPRC5A | -1,639579028 | -1,738067356 | 0,098488328 | |
| 235676_at | - | -1,639579028 | -1,738067356 | 0,098488328 | |
| 235951_s_at | ZNF688 | -1,639579028 | -1,738067356 | 0,098488328 | |
| 237469_at | TOP2A | -1,639579028 | -1,738067356 | 0,098488328 | |
| 238033_at | HEXDC | -1,639579028 | -1,738067356 | 0,098488328 | |
| 239140_at | - | -1,639579028 | -1,738067356 | 0,098488328 | |
| 242634_at | GATAD1 | -1,639579028 | -1,738067356 | 0,098488328 | |
| 243835_at | ZDHHC21 | -1,639579028 | -1,738067356 | 0,098488328 | |
| 214707_x_at | ALMS1 | 1,567778694 | 1,469325098 | 0,098453596 | |
| 226131_s_at | RPS16 | 8,380322394 | 8,281905277 | 0,098417117 | |
| 202081_at | IER2 | 5,13582221 | 5,037433789 | 0,098388421 | |
| 1555893_at | - | -2,236765673 | -2,335104118 | 0,098338445 | |
| 1556700_a_at | - | -2,236765673 | -2,335104118 | 0,098338445 | |
| 1556711_at | FAM216B | -2,236765673 | -2,335104118 | 0,098338445 | |
| 1557063_at | DICER1-AS1 | -2,236765673 | -2,335104118 | 0,098338445 | |
| 1558947_at | - | -2,236765673 | -2,335104118 | 0,098338445 | |
| 1561169_at | - | -2,236765673 | -2,335104118 | 0,098338445 | |
| 1566440_at | CTNND2 | -2,236765673 | -2,335104118 | 0,098338445 | |
| 1566571_at | - | -2,236765673 | -2,335104118 | 0,098338445 | |
| 201859_at | SRGN | -2,236765673 | -2,335104118 | 0,098338445 | |
| 207470_at | - | -2,236765673 | -2,335104118 | 0,098338445 | |
| 211064_at | ZNF493 | -2,236765673 | -2,335104118 | 0,098338445 | |
| 214160_at | - | -2,236765673 | -2,335104118 | 0,098338445 | |
| 216642_at | - | -2,236765673 | -2,335104118 | 0,098338445 | |
| 217513_at | MILR1 | -2,236765673 | -2,335104118 | 0,098338445 | |

| | | | | | |
|---|---|---|---|---|---|
| 224153_s_at | - | -2,236765673 | -2,335104118 | 0,098338445 | |
| 230539_at | FAM182A | -2,236765673 | -2,335104118 | 0,098338445 | |
| 231093_at | FCRL3 | -2,236765673 | -2,335104118 | 0,098338445 | |
| 232131_at | LOC100506874 | -2,236765673 | -2,335104118 | 0,098338445 | |
| 233671_at | - | -2,236765673 | -2,335104118 | 0,098338445 | |
| 234178_at | - | -2,236765673 | -2,335104118 | 0,098338445 | |
| 239472_at | - | -2,236765673 | -2,335104118 | 0,098338445 | |
| 241031_at | C2CD4A | -2,236765673 | -2,335104118 | 0,098338445 | |
| 241543_at | - | -2,236765673 | -2,335104118 | 0,098338445 | |
| 218704_at | RNF43 | -0,43775896 | -0,536087151 | 0,09832819 | |
| 230035_at | BOC | -0,43775896 | -0,536087151 | 0,09832819 | |
| 230989_s_at | TSSK6 | -0,43775896 | -0,536087151 | 0,09832819 | |
| 235668_at | PRDM1 | -0,43775896 | -0,536087151 | 0,09832819 | |
| 239372_at | - | -0,43775896 | -0,536087151 | 0,09832819 | |
| 239892_at | - | -0,43775896 | -0,536087151 | 0,09832819 | |
| 239917_at | - | -0,43775896 | -0,536087151 | 0,09832819 | |
| 243209_at | KCNQ4 | -0,43775896 | -0,536087151 | 0,09832819 | |
| 203469_s_at | CDK10 | 1,496984567 | 1,398700978 | 0,098283589 | |
| 204226_at | STAU2 | 1,496984567 | 1,398700978 | 0,098283589 | |
| 203659_s_at | TRIM13 | 2,804079361 | 2,705818724 | 0,098260638 | |
| 212708_at | MSL1 | 3,109000457 | 3,010820719 | 0,098179738 | |
| 203412_at | LZTR1 | 1,251994932 | 1,153866761 | 0,098128171 | |
| 202062_s_at | SEL1L | 1,422536444 | 1,324440845 | 0,098095599 | |
| 202699_s_at | TMEM63A | 0,255492549 | 0,157549243 | 0,097943306 | |
| 211059_s_at | GOLGA2 | 0,255492549 | 0,157549243 | 0,097943306 | |
| 220346_at | MTHFD2L | 0,255492549 | 0,157549243 | 0,097943306 | |
| 1552846_s_at | RAB42 | 0,57782909 | 0,479907041 | 0,09792205 | |
| 201939_at | PLK2 | 0,57782909 | 0,479907041 | 0,09792205 | |
| 218783_at | INTS7 | 0,57782909 | 0,479907041 | 0,09792205 | |
| 229251_s_at | TPCN2 | 0,57782909 | 0,479907041 | 0,09792205 | |
| 235475_at | SERP1 | 0,57782909 | 0,479907041 | 0,09792205 | |
| 226488_at | RCCD1 | 3,088871381 | 2,990953566 | 0,097917815 | |
| 229200_at | - | 1,578645857 | 1,480766833 | 0,097879024 | |
| 218741_at | CENPM | 2,81177561 | 2,713921281 | 0,097854329 | |
| 224693_at | FAM210B | 2,901063789 | 2,803210557 | 0,097853232 | |
| 214363_s_at | MATR3 /// SNHG | 6,802380078 | 6,704537798 | 0,097842281 | |
| 213460_x_at | NSUN5P2 | 2,506929603 | 2,409170778 | 0,097758825 | |
| 210524_x_at | - | 3,21379258 | 3,116064987 | 0,097727592 | |
| 202591_s_at | MIR5096 /// SSBI | 6,913223426 | 6,815536928 | 0,097686498 | |
| 1560992_at | LOC400590 | -3,250025915 | -3,347710726 | 0,097684811 | |
| 1562780_at | - | -3,250025915 | -3,347710726 | 0,097684811 | |
| 1568925_at | MYLK3 | -3,250025915 | -3,347710726 | 0,097684811 | |
| 1569577_x_at | - | -3,250025915 | -3,347710726 | 0,097684811 | |
| 240734_at | LOC100507221 | -3,250025915 | -3,347710726 | 0,097684811 | |
| 223370_at | PLEKHA3 | 3,304956562 | 3,207279726 | 0,097676835 | |
| 203743_s_at | TDG | 4,968262591 | 4,870603662 | 0,097658929 | |
| 209905_at | HOXA10-HOXA9 | 0,962260738 | 0,864649967 | 0,097610771 | |
| 237202_at | PGPEP1 | 0,962260738 | 0,864649967 | 0,097610771 | |
| 202939_at | ZMPSTE24 | 4,729929934 | 4,632345568 | 0,097584366 | |
| 207974_s_at | SKP1 | 5,68207279 | 5,584587791 | 0,097484999 | |
| 227715_at | LOC100233156 / | 1,434549838 | 1,337086025 | 0,097463813 | |
| 218488_at | EIF2B3 | 3,512821151 | 3,415371412 | 0,09744974 | |
| 1552853_at | VWA5B1 | -2,311268652 | -2,408705138 | 0,097436486 | |
| 1562966_at | KIAA1217 | -2,311268652 | -2,408705138 | 0,097436486 | |
| 1569089_a_at | FLJ35390 /// LOC | -2,311268652 | -2,408705138 | 0,097436486 | |
| 1570476_at | - | -2,311268652 | -2,408705138 | 0,097436486 | |

| | | | | | |
|---|---|---|---|---|---|
| 204619_s_at | VCAN | -2,311268652 | -2,408705138 | 0,097436486 | |
| 207994_s_at | OPRM1 | -2,311268652 | -2,408705138 | 0,097436486 | |
| 214053_at | ERBB4 | -2,311268652 | -2,408705138 | 0,097436486 | |
| 215752_at | SIK2 | -2,311268652 | -2,408705138 | 0,097436486 | |
| 217953_at | PHF3 | -2,311268652 | -2,408705138 | 0,097436486 | |
| 220673_s_at | PPP4R4 | -2,311268652 | -2,408705138 | 0,097436486 | |
| 220874_at | - | -2,311268652 | -2,408705138 | 0,097436486 | |
| 227314_at | ITGA2 | -2,311268652 | -2,408705138 | 0,097436486 | |
| 230749_s_at | CAMK2D | -2,311268652 | -2,408705138 | 0,097436486 | |
| 231248_at | CST6 | -2,311268652 | -2,408705138 | 0,097436486 | |
| 233882_s_at | SEMA6D | -2,311268652 | -2,408705138 | 0,097436486 | |
| 235511_at | RBM4 | -2,311268652 | -2,408705138 | 0,097436486 | |
| 238516_at | BMPR2 | -2,311268652 | -2,408705138 | 0,097436486 | |
| 240212_at | - | -2,311268652 | -2,408705138 | 0,097436486 | |
| 240301_at | DPPA2 | -2,311268652 | -2,408705138 | 0,097436486 | |
| 240931_s_at | - | -2,311268652 | -2,408705138 | 0,097436486 | |
| 223707_at | RPL27A /// SNOF | 0,072842263 | -0,024572586 | 0,097414849 | |
| 230239_at | ROCK1 | 0,072842263 | -0,024572586 | 0,097414849 | |
| 242422_at | G3BP1 | 0,072842263 | -0,024572586 | 0,097414849 | |
| 208695_s_at | LOC100652821 / | 7,992995898 | 7,8956002 | 0,097395698 | |
| 213515_x_at | HBG1 /// HBG2 // | 8,11768112 | 8,020326558 | 0,097354561 | |
| 210667_s_at | C21orf33 | 3,981211211 | 3,883869861 | 0,09734135 | |
| 227577_at | EXOC8 | 2,726400382 | 2,629059192 | 0,09734119 | |
| 219097_x_at | C19orf42 | 4,642435962 | 4,545117128 | 0,097318834 | |
| 1553274_a_at | SNRNP48 | 2,589836943 | 2,492544876 | 0,097292068 | |
| 210916_s_at | CD44 | 1,515954005 | 1,418668082 | 0,097285923 | |
| 227159_at | GHDC | 0,853119696 | 0,755840599 | 0,097279097 | |
| 219192_at | UBAP2 | 4,257970041 | 4,160749839 | 0,097220202 | |
| 203727_at | SKIV2L | 2,077135162 | 1,979940779 | 0,097194383 | |
| 212178_s_at | POM121 /// POM | 2,55541096 | 2,458228496 | 0,097182464 | |
| 201462_at | SCRN1 | 0,592200562 | 0,495031851 | 0,097168711 | |
| 216064_s_at | AGA | 0,592200562 | 0,495031851 | 0,097168711 | |
| 236030_at | RCOR2 | 0,592200562 | 0,495031851 | 0,097168711 | |
| 209797_at | CNPY2 | 3,358484112 | 3,261354437 | 0,097129675 | |
| 1553567_s_at | ATP6 | 8,056442163 | 7,959353442 | 0,097088721 | |
| 218046_s_at | LOC100652993 / | 3,664184451 | 3,56709884 | 0,097085611 | |
| 221972_s_at | SDF4 | 3,000043914 | 2,902971658 | 0,097072257 | |
| 1553453_at | ASB14 | -1,518072576 | -1,615132408 | 0,097059832 | |
| 1554689_a_at | NLGN4X | -1,518072576 | -1,615132408 | 0,097059832 | |
| 1561480_a_at | - | -1,518072576 | -1,615132408 | 0,097059832 | |
| 1564536_at | ASB10 | -1,518072576 | -1,615132408 | 0,097059832 | |
| 207695_s_at | IGSF1 | -1,518072576 | -1,615132408 | 0,097059832 | |
| 208601_s_at | TUBB1 | -1,518072576 | -1,615132408 | 0,097059832 | |
| 216900_s_at | CHRNA4 | -1,518072576 | -1,615132408 | 0,097059832 | |
| 220481_at | GPR75 | -1,518072576 | -1,615132408 | 0,097059832 | |
| 222198_at | - | -1,518072576 | -1,615132408 | 0,097059832 | |
| 225667_s_at | FAM84A | -1,518072576 | -1,615132408 | 0,097059832 | |
| 229259_at | GFAP | -1,518072576 | -1,615132408 | 0,097059832 | |
| 230824_at | 10.03.15 | -1,518072576 | -1,615132408 | 0,097059832 | |
| 231083_at | ETV5 | -1,518072576 | -1,615132408 | 0,097059832 | |
| 236654_s_at | - | -1,518072576 | -1,615132408 | 0,097059832 | |
| 237055_at | LOC100506174 | -1,518072576 | -1,615132408 | 0,097059832 | |
| 238756_at | GAS2L3 | -1,518072576 | -1,615132408 | 0,097059832 | |
| 239619_at | - | -1,518072576 | -1,615132408 | 0,097059832 | |
| 239812_s_at | IQCH | -1,518072576 | -1,615132408 | 0,097059832 | |
| 240437_at | CASP9 | -1,518072576 | -1,615132408 | 0,097059832 | |

| | | | | | |
|---|---|---|---|---|---|
| 241660_at | - | -1,518072576 | -1,615132408 | 0,097059832 | |
| 203078_at | CUL2 | 0,97328325 | 0,876248481 | 0,097034769 | |
| 1557135_at | - | 0,273439642 | 0,176436073 | 0,097003569 | |
| 220019_s_at | ZNF224 | 0,273439642 | 0,176436073 | 0,097003569 | |
| 228427_at | FBXO16 | 0,273439642 | 0,176436073 | 0,097003569 | |
| 238920_at | - | 0,273439642 | 0,176436073 | 0,097003569 | |
| 1555723_at | - | -2,359436556 | -2,456406426 | 0,09696987 | |
| 1556571_at | LOC100506538 / | -2,359436556 | -2,456406426 | 0,09696987 | |
| 1557052_at | - | -2,359436556 | -2,456406426 | 0,09696987 | |
| 1557068_at | LOC100505782 | -2,359436556 | -2,456406426 | 0,09696987 | |
| 1560340_s_at | RP9P | -2,359436556 | -2,456406426 | 0,09696987 | |
| 1560863_a_at | - | -2,359436556 | -2,456406426 | 0,09696987 | |
| 1562216_at | - | -2,359436556 | -2,456406426 | 0,09696987 | |
| 1566582_x_at | - | -2,359436556 | -2,456406426 | 0,09696987 | |
| 205850_s_at | GABRB3 | -2,359436556 | -2,456406426 | 0,09696987 | |
| 207731_at | - | -2,359436556 | -2,456406426 | 0,09696987 | |
| 211273_s_at | TBX1 | -2,359436556 | -2,456406426 | 0,09696987 | |
| 213071_at | DPT | -2,359436556 | -2,456406426 | 0,09696987 | |
| 215615_x_at | - | -2,359436556 | -2,456406426 | 0,09696987 | |
| 217385_at | - | -2,359436556 | -2,456406426 | 0,09696987 | |
| 220075_s_at | CDHR5 | -2,359436556 | -2,456406426 | 0,09696987 | |
| 221469_at | GPR32 | -2,359436556 | -2,456406426 | 0,09696987 | |
| 231622_at | ASB17 | -2,359436556 | -2,456406426 | 0,09696987 | |
| 232927_at | - | -2,359436556 | -2,456406426 | 0,09696987 | |
| 237261_at | ANGPT2 | -2,359436556 | -2,456406426 | 0,09696987 | |
| 237999_at | - | -2,359436556 | -2,456406426 | 0,09696987 | |
| 238984_at | REG4 | -2,359436556 | -2,456406426 | 0,09696987 | |
| 241579_at | - | -2,359436556 | -2,456406426 | 0,09696987 | |
| 241967_at | - | -2,359436556 | -2,456406426 | 0,09696987 | |
| 223880_x_at | C20orf24 /// TGIF | 6,372808442 | 6,2758742 | 0,096934242 | |
| 229716_at | - | 1,976645579 | 1,879785595 | 0,096859984 | |
| 208697_s_at | EIF3E | 7,317944519 | 7,221159724 | 0,096784795 | |
| 227018_at | DPP8 | 1,923647937 | 1,826973888 | 0,096674049 | |
| 225795_at | C22orf32 | 3,469688722 | 3,373018423 | 0,096670299 | |
| 203805_s_at | FANCA | 1,187200746 | 1,090553537 | 0,096647209 | |
| 211043_s_at | CLTB | 3,188003814 | 3,091398163 | 0,096605651 | |
| 214751_at | ZNF468 | 3,451082821 | 3,354479478 | 0,096603343 | |
| 225904_at | CCSAP | 3,164152611 | 3,067567462 | 0,096585149 | |
| 207417_s_at | ZNF177 /// ZNF5 | 1,805273023 | 1,708698295 | 0,096574728 | |
| 1554905_x_at | FRMD8 | 1,369290453 | 1,272721751 | 0,096568702 | |
| 215749_s_at | GORASP1 | 1,369290453 | 1,272721751 | 0,096568702 | |
| 219409_at | SNIP1 | 2,089851283 | 1,993317152 | 0,096534131 | |
| 48030_i_at | C5orf4 | 3,41414682 | 3,317734938 | 0,096411882 | |
| 218178_s_at | CHMP1B | 3,352164686 | 3,255815291 | 0,096349395 | |
| 201960_s_at | MYCBP2 | 4,286420174 | 4,190088544 | 0,09633163 | |
| 230304_at | - | 1,932146506 | 1,835911187 | 0,096235319 | |
| 1554522_at | CNNM2 | -0,394749123 | -0,490927335 | 0,096178212 | |
| 1563903_x_at | - | -0,394749123 | -0,490927335 | 0,096178212 | |
| 1569393_at | MGC15885 | -0,394749123 | -0,490927335 | 0,096178212 | |
| 209138_x_at | IGLC1 | -0,394749123 | -0,490927335 | 0,096178212 | |
| 214462_at | SOCS6 | -0,394749123 | -0,490927335 | 0,096178212 | |
| 216504_s_at | SLC39A8 | -0,394749123 | -0,490927335 | 0,096178212 | |
| 223905_at | CCDC135 | -0,394749123 | -0,490927335 | 0,096178212 | |
| 241166_at | - | -0,394749123 | -0,490927335 | 0,096178212 | |
| 242437_at | - | -0,394749123 | -0,490927335 | 0,096178212 | |
| 218195_at | C6orf211 | 4,344876891 | 4,248708657 | 0,096168234 | |

| | | | | | |
|---|---|---|---|---|---|
| 1566480_x_at | C17orf104 | -0,759588698 | -0,855751026 | 0,096162328 | |
| 203666_at | CXCL12 | -0,759588698 | -0,855751026 | 0,096162328 | |
| 211107_s_at | AURKC | -0,759588698 | -0,855751026 | 0,096162328 | |
| 222897_s_at | ZFP64 | -0,759588698 | -0,855751026 | 0,096162328 | |
| 223447_at | REG4 | -0,759588698 | -0,855751026 | 0,096162328 | |
| 233535_at | ATP13A4 | -0,759588698 | -0,855751026 | 0,096162328 | |
| 238252_at | - | -0,759588698 | -0,855751026 | 0,096162328 | |
| 239485_at | CDH4 | -0,759588698 | -0,855751026 | 0,096162328 | |
| 239925_at | - | -0,759588698 | -0,855751026 | 0,096162328 | |
| 241517_at | - | -0,759588698 | -0,855751026 | 0,096162328 | |
| 205135_s_at | NUFIP1 | 2,963199262 | 2,867091644 | 0,096107618 | |
| 229759_s_at | VEPH1 | 1,814495998 | 1,718396606 | 0,096099392 | |
| 208794_s_at | SMARCA4 | 6,70402539 | 6,60795278 | 0,09607261 | |
| 210912_x_at | GSTM4 | 0,613492853 | 0,51742585 | 0,096067003 | |
| 221889_at | KCTD13 | 0,613492853 | 0,51742585 | 0,096067003 | |
| 230134_s_at | RC3H2 | 0,613492853 | 0,51742585 | 0,096067003 | |
| 208433_s_at | LRP8 | 1,614289682 | 1,518263577 | 0,096026104 | |
| 202268_s_at | NAE1 | 5,627082064 | 5,531136224 | 0,095945841 | |
| 1570237_at | - | 1,937784532 | 1,841838775 | 0,095945757 | |
| 227160_s_at | NDUFAF5 | 3,27186005 | 3,175947691 | 0,095912359 | |
| 224691_at | UHMK1 | 5,554513056 | 5,458610413 | 0,095902643 | |
| 213653_at | METTL3 | 2,721505869 | 2,625627364 | 0,095878505 | |
| 206185_at | CRYBB1 | 0,103263601 | 0,007421914 | 0,095841688 | |
| 221785_at | WIZ | 0,103263601 | 0,007421914 | 0,095841688 | |
| 236504_x_at | C6orf52 | 1,46610492 | 1,370274469 | 0,095830451 | |
| 218728_s_at | CNIH4 | 3,405017566 | 3,309203139 | 0,095814427 | |
| 230243_at | TRMT10A | 1,296557217 | 1,200746069 | 0,095811148 | |
| 213935_at | ABHD5 | 0,882651021 | 0,786897721 | 0,095753299 | |
| 228490_at | ABHD2 | 3,508091848 | 3,412387088 | 0,095704761 | |
| 244599_at | - | 2,854131948 | 2,758473378 | 0,09565857 | |
| 231851_at | RAVER2 | 1,000476627 | 0,904843258 | 0,095633369 | |
| 202906_s_at | NBN | 3,986661073 | 3,891034012 | 0,09562706 | |
| 218537_at | HCFC1R1 | 1,69307511 | 1,59747851 | 0,0955966 | |
| 225443_at | DCP1A | 2,886560922 | 2,791007185 | 0,095553737 | |
| 217347_at | - | 1,946200473 | 1,85068474 | 0,095515732 | |
| 225930_at | NKIRAS1 | 1,946200473 | 1,85068474 | 0,095515732 | |
| 204065_at | CHST10 | 2,003776245 | 1,908311039 | 0,095465206 | |
| 45749_at | FAM65A | 3,018119282 | 2,922681418 | 0,095437864 | |
| 226794_at | STXBP5 | 5,321407378 | 5,226006411 | 0,095400967 | |
| 223309_x_at | PNPLA8 | 2,392522438 | 2,297144887 | 0,095377551 | |
| 225428_s_at | DDX54 | 0,627515113 | 0,532164371 | 0,095350742 | |
| 228144_at | ZNF300 | 0,627515113 | 0,532164371 | 0,095350742 | |
| 239979_at | - | 0,627515113 | 0,532164371 | 0,095350742 | |
| 1553935_at | MGC2848 | -2,479842156 | -2,575129729 | 0,095287573 | |
| 1554810_at | PLA2G4C | -2,479842156 | -2,575129729 | 0,095287573 | |
| 1556235_at | LOC100506560 / | -2,479842156 | -2,575129729 | 0,095287573 | |
| 1558796_a_at | - | -2,479842156 | -2,575129729 | 0,095287573 | |
| 1559598_at | - | -2,479842156 | -2,575129729 | 0,095287573 | |
| 1562000_at | LOC400620 | -2,479842156 | -2,575129729 | 0,095287573 | |
| 1565554_at | LOC127841 | -2,479842156 | -2,575129729 | 0,095287573 | |
| 1565912_at | LOC283693 | -2,479842156 | -2,575129729 | 0,095287573 | |
| 1567390_at | - | -2,479842156 | -2,575129729 | 0,095287573 | |
| 1568854_at | LINC00240 | -2,479842156 | -2,575129729 | 0,095287573 | |
| 1569080_at | RNF165 | -2,479842156 | -2,575129729 | 0,095287573 | |
| 1569287_at | LINC00458 | -2,479842156 | -2,575129729 | 0,095287573 | |
| 207452_s_at | CNTN5 | -2,479842156 | -2,575129729 | 0,095287573 | |

| | | | | | |
|---|---|---|---|---|---|
| 210836_x_at | PDE4D | -2,479842156 | -2,575129729 | 0,095287573 | |
| 211632_at | IGHD /// IGHG1 /, | -2,479842156 | -2,575129729 | 0,095287573 | |
| 214566_at | SMR3A | -2,479842156 | -2,575129729 | 0,095287573 | |
| 216723_at | - | -2,479842156 | -2,575129729 | 0,095287573 | |
| 219642_s_at | PEX5L | -2,479842156 | -2,575129729 | 0,095287573 | |
| 223965_at | TRNAU1AP | -2,479842156 | -2,575129729 | 0,095287573 | |
| 224528_s_at | KCNIP2 | -2,479842156 | -2,575129729 | 0,095287573 | |
| 230913_at | - | -2,479842156 | -2,575129729 | 0,095287573 | |
| 232881_at | GNAS-AS1 | -2,479842156 | -2,575129729 | 0,095287573 | |
| 233038_at | - | -2,479842156 | -2,575129729 | 0,095287573 | |
| 233907_s_at | SERTAD4 | -2,479842156 | -2,575129729 | 0,095287573 | |
| 234793_at | - | -2,479842156 | -2,575129729 | 0,095287573 | |
| 235561_at | TXNL1 | -2,479842156 | -2,575129729 | 0,095287573 | |
| 237556_at | - | -2,479842156 | -2,575129729 | 0,095287573 | |
| 237949_at | - | -2,479842156 | -2,575129729 | 0,095287573 | |
| 240192_at | FLJ45983 | -2,479842156 | -2,575129729 | 0,095287573 | |
| 217748_at | ADIPOR1 | 3,436222554 | 3,340940512 | 0,095282042 | |
| 203186_s_at | S100A4 | 2,114952142 | 2,019703483 | 0,095248659 | |
| 236267_at | ZNF346 | 0,767063567 | 0,67181667 | 0,095246897 | |
| 200078_s_at | ATP6V0B | 5,437635603 | 5,342425264 | 0,095210338 | |
| 235204_at | ENTPD7 | 1,556829052 | 1,461646549 | 0,095182502 | |
| 1552609_s_at | IL28A /// IL28B | -1,399512875 | -1,494668682 | 0,095155806 | |
| 1560455_at | LOC339166 /// W | -1,399512875 | -1,494668682 | 0,095155806 | |
| 1564084_at | CAD | -1,399512875 | -1,494668682 | 0,095155806 | |
| 1564851_at | - | -1,399512875 | -1,494668682 | 0,095155806 | |
| 1569683_at | XYLB | -1,399512875 | -1,494668682 | 0,095155806 | |
| 206995_x_at | SCARF1 | -1,399512875 | -1,494668682 | 0,095155806 | |
| 210363_s_at | SCN2B | -1,399512875 | -1,494668682 | 0,095155806 | |
| 211794_at | FYB | -1,399512875 | -1,494668682 | 0,095155806 | |
| 215753_at | - | -1,399512875 | -1,494668682 | 0,095155806 | |
| 216707_at | - | -1,399512875 | -1,494668682 | 0,095155806 | |
| 217174_s_at | APC2 | -1,399512875 | -1,494668682 | 0,095155806 | |
| 220451_s_at | BIRC7 | -1,399512875 | -1,494668682 | 0,095155806 | |
| 232914_s_at | SYTL2 | -1,399512875 | -1,494668682 | 0,095155806 | |
| 237284_at | DNAJB8 | -1,399512875 | -1,494668682 | 0,095155806 | |
| 241000_at | - | -1,399512875 | -1,494668682 | 0,095155806 | |
| 244077_at | C10orf113 | -1,399512875 | -1,494668682 | 0,095155806 | |
| 244847_at | - | -1,399512875 | -1,494668682 | 0,095155806 | |
| 223165_s_at | IP6K2 | 3,217273782 | 3,122166354 | 0,095107427 | |
| 226848_at | - | 1,954567604 | 1,859476797 | 0,095090807 | |
| 209482_at | POP7 | 4,577555523 | 4,482467631 | 0,095087892 | |
| 201511_at | AAMP | 4,569406661 | 4,474382017 | 0,095024644 | |
| 1561417_x_at | - | -3,342483913 | -3,437493426 | 0,095009513 | |
| 1563055_at | - | -3,342483913 | -3,437493426 | 0,095009513 | |
| 206091_at | MATN3 | -3,342483913 | -3,437493426 | 0,095009513 | |
| 65086_at | YIPF2 | 3,443176398 | 3,348246509 | 0,094929889 | |
| 226043_at | GPSM1 | 2,35929703 | 2,264418116 | 0,094878913 | |
| 240239_at | ZNF566 | 2,35929703 | 2,264418116 | 0,094878913 | |
| 219577_s_at | ABCA7 | 0,900083947 | 0,805216064 | 0,094867883 | |
| 1552651_a_at | RAD51L3-RFFL / | 0,123193849 | 0,028364014 | 0,094829835 | |
| 203578_s_at | SLC7A6 | 0,123193849 | 0,028364014 | 0,094829835 | |
| 205243_at | SLC13A3 | 0,123193849 | 0,028364014 | 0,094829835 | |
| 230273_at | C6orf165 | 0,123193849 | 0,028364014 | 0,094829835 | |
| 1555165_a_at | PGPEP1 | -0,366776884 | -0,461594427 | 0,094817543 | |
| 1564674_a_at | CDC20B | -0,366776884 | -0,461594427 | 0,094817543 | |
| 214063_s_at | TF | -0,366776884 | -0,461594427 | 0,094817543 | |

| | | | | | |
|---|---|---|---|---|---|
| 220961_s_at | TBRG4 | -0,366776884 | -0,461594427 | 0,094817543 | |
| 233197_at | KLHL9 | -0,366776884 | -0,461594427 | 0,094817543 | |
| 203526_s_at | APC | 0,317354302 | 0,222599095 | 0,094755207 | |
| 222716_s_at | SNX24 | 0,317354302 | 0,222599095 | 0,094755207 | |
| 223769_x_at | HYI | 1,318332599 | 1,223626587 | 0,094706012 | |
| 227972_at | TOR2A | 1,318332599 | 1,223626587 | 0,094706012 | |
| 223381_at | NUF2 | 3,489018063 | 3,394350331 | 0,094667733 | |
| 206936_x_at | - | 1,780386218 | 1,685812099 | 0,094574119 | |
| 205420_at | PEX7 | 2,320980353 | 2,226407528 | 0,094572825 | |
| 221634_at | LOC100287195 / | 2,994644467 | 2,900133867 | 0,0945106 | |
| 205193_at | MAFF | 1,493160564 | 1,398700978 | 0,094459586 | |
| 235158_at | TMEM209 | 2,783874823 | 2,689475799 | 0,094399024 | |
| 43511_s_at | ARRB1 | 2,025119313 | 1,930732138 | 0,094387176 | |
| 221498_at | SNX27 | 3,025418883 | 2,93104672 | 0,094372163 | |
| 201562_s_at | SORD | 2,369762403 | 2,275409734 | 0,094352669 | |
| 201242_s_at | ATP1B1 | 0,648296226 | 0,553993624 | 0,094302601 | |
| 235961_at | GPR161 | 0,648296226 | 0,553993624 | 0,094302601 | |
| 229806_at | QRICH1 | 0,911589958 | 0,817300337 | 0,094289621 | |
| 201530_x_at | EIF4A1 /// SENP3 | 7,536318821 | 7,442041109 | 0,094277711 | |
| 203837_at | MAP3K5 | 4,014272998 | 3,920040657 | 0,094232341 | |
| 204983_s_at | GPC4 | 2,236584753 | 2,14244844 | 0,094136313 | |
| 225584_at | HCG18 | 1,973904239 | 1,879785595 | 0,094118643 | |
| 225173_at | ARHGAP18 | 2,945117939 | 2,851017268 | 0,094100671 | |
| 212313_at | CHMP7 | 3,24026957 | 3,146317015 | 0,093952556 | |
| 222661_at | AGGF1 | 2,857110344 | 2,763166224 | 0,093944119 | |
| 37384_at | PPM1F | 2,034358528 | 1,940432851 | 0,093925676 | |
| 212795_at | KIAA1033 | 3,378314944 | 3,284389368 | 0,093925576 | |
| 216177_at | - | 1,655931423 | 1,562009954 | 0,093921469 | |
| 227539_at | GNA13 | 1,504602299 | 1,410714371 | 0,093887927 | |
| 201788_at | DDX42 | 3,620156261 | 3,526272152 | 0,093884109 | |
| 231839_at | PDE12 | 3,904829675 | 3,810956512 | 0,093873163 | |
| 201339_s_at | SCP2 | 4,454147865 | 4,360286277 | 0,093861588 | |
| 228766_at | CD36 | 4,972388731 | 4,878554857 | 0,093833875 | |
| 207163_s_at | AKT1 | 2,796341835 | 2,702564916 | 0,093776919 | |
| 201585_s_at | SFPQ | 4,763255815 | 4,669482912 | 0,093772903 | |
| 228013_at | PPP2R2A | 2,466546243 | 2,372786857 | 0,093759386 | |
| 212438_at | SNRNP27 | 3,527851575 | 3,434130109 | 0,093721466 | |
| 218431_at | C14orf133 | 2,623460554 | 2,529740643 | 0,093719911 | |
| 226240_at | TADA2B | 1,982112682 | 1,888402606 | 0,093710077 | |
| 205252_at | ZNF174 | 1,094335495 | 1,000684521 | 0,093650974 | |
| 206968_s_at | NFRKB | 1,094335495 | 1,000684521 | 0,093650974 | |
| 235081_x_at | TRIM65 | 2,146943689 | 2,053299695 | 0,093643994 | |
| 237333_at | RBBP4 | 1,339784194 | 1,246149888 | 0,093634306 | |
| 213499_at | CLCN2 | 0,661985843 | 0,568365095 | 0,093620747 | |
| 214828_s_at | RRP7A /// RRP7B | 0,661985843 | 0,568365095 | 0,093620747 | |
| 220153_at | ENTPD7 | 1,247462054 | 1,153866761 | 0,093595292 | |
| 221291_at | ULBP2 | 1,247462054 | 1,153866761 | 0,093595292 | |
| 200726_at | PPP1CC | 6,28609959 | 6,192508474 | 0,093591116 | |
| 226917_s_at | ANAPC4 | 3,408067074 | 3,314541423 | 0,093525651 | |
| 31846_at | RHOD | 2,751427511 | 2,657904765 | 0,093522746 | |
| 209998_at | PIGO | 1,512180019 | 1,418668082 | 0,093511937 | |
| 218375_at | NUDT9 | 2,450859799 | 2,357351836 | 0,093507962 | |
| 225176_at | LNPEP | 2,512607472 | 2,419116683 | 0,093490789 | |
| 224967_at | UGCG | 4,015606497 | 3,922140371 | 0,093466125 | |
| 214924_s_at | TRAK1 | 1,735773781 | 1,642321262 | 0,093452519 | |
| 1557964_at | EIF4G2 | 0,928678725 | 0,835239052 | 0,093439673 | |

| | | | | | |
|---|---|---|---|---|---|
| 207265_s_at | KDELR3 | 0,928678725 | 0,835239052 | 0,093439673 | |
| 232347_x_at | - | 0,928678725 | 0,835239052 | 0,093439673 | |
| 225948_at | APOPT1 /// KLC1 | 3,298397722 | 3,204982027 | 0,093415695 | |
| 218867_s_at | C12orf49 | 1,666156729 | 1,572742379 | 0,09341435 | |
| 223545_at | FANCD2 | 1,666156729 | 1,572742379 | 0,09341435 | |
| 217877_s_at | GPBP1L1 | 3,388643755 | 3,295230443 | 0,093413312 | |
| 201191_at | PITPNA | 2,151803104 | 2,058399602 | 0,093403502 | |
| 1553126_a_at | SLC39A12 | -2,597496523 | -2,690860744 | 0,093364221 | |
| 1554308_s_at | GABRA2 | -2,597496523 | -2,690860744 | 0,093364221 | |
| 1555046_at | CENPI | -2,597496523 | -2,690860744 | 0,093364221 | |
| 1559587_at | SYMPK | -2,597496523 | -2,690860744 | 0,093364221 | |
| 1569176_at | TMPRSS12 | -2,597496523 | -2,690860744 | 0,093364221 | |
| 1569674_at | LOC100287765 | -2,597496523 | -2,690860744 | 0,093364221 | |
| 205638_at | BAI3 | -2,597496523 | -2,690860744 | 0,093364221 | |
| 207182_at | GABRA6 | -2,597496523 | -2,690860744 | 0,093364221 | |
| 208582_s_at | DUX1 /// DUX3 // | -2,597496523 | -2,690860744 | 0,093364221 | |
| 210147_at | ART3 | -2,597496523 | -2,690860744 | 0,093364221 | |
| 210262_at | CRISP2 | -2,597496523 | -2,690860744 | 0,093364221 | |
| 220177_s_at | TMPRSS3 | -2,597496523 | -2,690860744 | 0,093364221 | |
| 223698_at | SLC25A36 | -2,597496523 | -2,690860744 | 0,093364221 | |
| 226535_at | ITGB6 | -2,597496523 | -2,690860744 | 0,093364221 | |
| 227478_at | SETBP1 | -2,597496523 | -2,690860744 | 0,093364221 | |
| 230993_s_at | C6orf118 | -2,597496523 | -2,690860744 | 0,093364221 | |
| 231750_at | PCDHB4 | -2,597496523 | -2,690860744 | 0,093364221 | |
| 231789_at | PCDHB15 | -2,597496523 | -2,690860744 | 0,093364221 | |
| 235326_at | - | -2,597496523 | -2,690860744 | 0,093364221 | |
| 236029_at | FAT3 | -2,597496523 | -2,690860744 | 0,093364221 | |
| 240596_at | - | -2,597496523 | -2,690860744 | 0,093364221 | |
| 241509_at | - | -2,597496523 | -2,690860744 | 0,093364221 | |
| 1554208_at | MEI1 | 0,152582607 | 0,059218869 | 0,093363738 | |
| 221718_s_at | AKAP13 | 0,152582607 | 0,059218869 | 0,093363738 | |
| 228312_at | PI16 | 0,152582607 | 0,059218869 | 0,093363738 | |
| 234963_s_at | FA2H | 0,152582607 | 0,059218869 | 0,093363738 | |
| 213111_at | PIKFYVE | 3,495722611 | 3,402394519 | 0,093328092 | |
| 209704_at | MTF2 | 3,436222554 | 3,343031718 | 0,093190836 | |
| 211780_x_at | DCTN1 | 2,840652551 | 2,747463672 | 0,093188879 | |
| 224744_at | IMPAD1 | 3,209137871 | 3,116064987 | 0,093072884 | |
| 213047_x_at | SET | 5,998625791 | 5,905553422 | 0,093072368 | |
| 233123_at | SLC40A1 | 2,159061673 | 2,066015817 | 0,093045856 | |
| 1555565_s_at | TAPBP | 1,05337236 | 0,960386719 | 0,09298564 | |
| 58780_s_at | ARHGEF40 | 1,712939334 | 1,620074108 | 0,092865226 | |
| 1552261_at | WFDC2 | -1,2845686 | -1,377419394 | 0,092850794 | |
| 1559042_at | NDUFB6 | -1,2845686 | -1,377419394 | 0,092850794 | |
| 1561367_a_at | LINC00540 | -1,2845686 | -1,377419394 | 0,092850794 | |
| 1562903_at | FLJ10661 | -1,2845686 | -1,377419394 | 0,092850794 | |
| 1563814_at | C2orf50 | -1,2845686 | -1,377419394 | 0,092850794 | |
| 206803_at | PDYN | -1,2845686 | -1,377419394 | 0,092850794 | |
| 211494_s_at | SLC4A4 | -1,2845686 | -1,377419394 | 0,092850794 | |
| 215270_at | LFNG | -1,2845686 | -1,377419394 | 0,092850794 | |
| 215757_at | PRKDC | -1,2845686 | -1,377419394 | 0,092850794 | |
| 219813_at | LATS1 | -1,2845686 | -1,377419394 | 0,092850794 | |
| 220400_at | VPS13B | -1,2845686 | -1,377419394 | 0,092850794 | |
| 220687_at | - | -1,2845686 | -1,377419394 | 0,092850794 | |
| 235308_at | ZBTB20 | -1,2845686 | -1,377419394 | 0,092850794 | |
| 241249_at | LOC253039 | -1,2845686 | -1,377419394 | 0,092850794 | |
| 242822_at | MGC39584 | -1,2845686 | -1,377419394 | 0,092850794 | |

| | | | | | |
|---|---|---|---|---|---|
| 204991_s_at | NF2 | -0,325749314 | -0,41858459 | 0,092835276 | |
| 205983_at | DPEP1 | -0,325749314 | -0,41858459 | 0,092835276 | |
| 206570_s_at | PSG11 | -0,325749314 | -0,41858459 | 0,092835276 | |
| 217966_s_at | FAM129A | -0,325749314 | -0,41858459 | 0,092835276 | |
| 220561_at | IGF2-AS | -0,325749314 | -0,41858459 | 0,092835276 | |
| 220705_s_at | ADAMTS7 /// LO( | -0,325749314 | -0,41858459 | 0,092835276 | |
| 228331_at | C11orf31 | -0,325749314 | -0,41858459 | 0,092835276 | |
| 234912_at | - | -0,325749314 | -0,41858459 | 0,092835276 | |
| 213053_at | HAUS5 | 2,001086023 | 1,908311039 | 0,092774984 | |
| 223879_s_at | OXR1 | 2,001086023 | 1,908311039 | 0,092774984 | |
| 221485_at | B4GALT5 | 4,324039713 | 4,231314909 | 0,092724804 | |
| 203011_at | IMPA1 | 4,391844214 | 4,299127518 | 0,092716696 | |
| 227456_s_at | C6orf136 | 3,059434754 | 2,966746113 | 0,092688641 | |
| 202573_at | CSNK1G2 | 3,217273782 | 3,124599694 | 0,092674087 | |
| 209682_at | CBLB | 3,217273782 | 3,124599694 | 0,092674087 | |
| 1557275_a_at | TLCD2 | -2,64318869 | -2,735862426 | 0,092673736 | |
| 1558592_at | - | -2,64318869 | -2,735862426 | 0,092673736 | |
| 1559288_at | - | -2,64318869 | -2,735862426 | 0,092673736 | |
| 1559479_at | LOC285540 | -2,64318869 | -2,735862426 | 0,092673736 | |
| 1561365_at | NRP1 | -2,64318869 | -2,735862426 | 0,092673736 | |
| 1562321_at | PDK4 | -2,64318869 | -2,735862426 | 0,092673736 | |
| 215241_at | ANO3 | -2,64318869 | -2,735862426 | 0,092673736 | |
| 217334_at | OR2J3 | -2,64318869 | -2,735862426 | 0,092673736 | |
| 221874_at | KIAA1324 | -2,64318869 | -2,735862426 | 0,092673736 | |
| 223878_at | INPP4B | -2,64318869 | -2,735862426 | 0,092673736 | |
| 228707_at | CLDN23 | -2,64318869 | -2,735862426 | 0,092673736 | |
| 230121_at | C1orf133 | -2,64318869 | -2,735862426 | 0,092673736 | |
| 231442_at | ZPBP2 | -2,64318869 | -2,735862426 | 0,092673736 | |
| 232606_at | ANK2 | -2,64318869 | -2,735862426 | 0,092673736 | |
| 233139_at | - | -2,64318869 | -2,735862426 | 0,092673736 | |
| 233552_at | - | -2,64318869 | -2,735862426 | 0,092673736 | |
| 234994_at | TMEM200A | -2,64318869 | -2,735862426 | 0,092673736 | |
| 238272_at | TPMT | -2,64318869 | -2,735862426 | 0,092673736 | |
| 238355_at | - | -2,64318869 | -2,735862426 | 0,092673736 | |
| 238874_at | LOC100506860 | -2,64318869 | -2,735862426 | 0,092673736 | |
| 239622_at | TRAP1 | -2,64318869 | -2,735862426 | 0,092673736 | |
| 240370_at | - | -2,64318869 | -2,735862426 | 0,092673736 | |
| 241586_at | - | -2,64318869 | -2,735862426 | 0,092673736 | |
| 244303_at | - | -2,64318869 | -2,735862426 | 0,092673736 | |
| 1557411_s_at | SLC25A43 | 1,751865623 | 1,659204017 | 0,092661606 | |
| 1559461_at | - | 0,359971644 | 0,26733076 | 0,092640884 | |
| 1563629_a_at | ERVK13-1 | 0,359971644 | 0,26733076 | 0,092640884 | |
| 202192_s_at | GAS7 | 0,359971644 | 0,26733076 | 0,092640884 | |
| 239061_at | TPRXL | 0,359971644 | 0,26733076 | 0,092640884 | |
| 235456_at | - | 2,883642771 | 2,791007185 | 0,092635586 | |
| 213149_at | DLAT | 3,50998543 | 3,417357537 | 0,092627894 | |
| 227343_at | - | -0,67289328 | -0,765504029 | 0,092610749 | |
| 232605_s_at | SPG20OS | -0,67289328 | -0,765504029 | 0,092610749 | |
| 236541_at | ARSA | -0,67289328 | -0,765504029 | 0,092610749 | |
| 237082_at | - | -0,67289328 | -0,765504029 | 0,092610749 | |
| 222757_s_at | ZAK | 1,820612054 | 1,728030156 | 0,092581897 | |
| 215667_x_at | LOC100132832 / | 2,531373922 | 2,438805127 | 0,092568795 | |
| 37170_at | BMP2K | 1,976645579 | 1,884100534 | 0,092545045 | |
| 214919_s_at | ANKHD1 /// ANKI | 1,269985355 | 1,177496821 | 0,092488534 | |
| 214257_s_at | - | 3,807337009 | 3,714911743 | 0,092425266 | |
| 217235_x_at | IGLL5 | 0,171847695 | 0,079428135 | 0,09241956 | |

| | | | | | |
|---|---|---|---|---|---|
| 232051_at | CCDC102A | 0,171847695 | 0,079428135 | 0,09241956 | |
| 232357_at | TTLL9 | 0,171847695 | 0,079428135 | 0,09241956 | |
| 234436_x_at | OBP2A | 0,171847695 | 0,079428135 | 0,09241956 | |
| 215004_s_at | SUGP1 | 1,534677255 | 1,442269454 | 0,092407801 | |
| 229091_s_at | CCNJ | 1,758252436 | 1,665902186 | 0,092350251 | |
| 224628_at | ERLEC1 | 3,983257322 | 3,891034012 | 0,09222331 | |
| 207305_s_at | TRAPPC8 | 3,598072957 | 3,505883949 | 0,092189008 | |
| 223741_s_at | TTYH2 | 0,82905153 | 0,73688026 | 0,09217127 | |
| 229810_at | - | 0,82905153 | 0,73688026 | 0,09217127 | |
| 222938_x_at | ENPP3 | 4,977187694 | 4,885027942 | 0,092159752 | |
| 226842_at | FBXL20 | 2,540666392 | 2,448549499 | 0,092116893 | |
| 1566509_s_at | FBXO9 | 4,043328971 | 3,951219941 | 0,09210903 | |
| 214902_x_at | - | 0,95671774 | 0,864649967 | 0,092067773 | |
| 228628_at | SRGAP2C | 0,95671774 | 0,864649967 | 0,092067773 | |
| 225792_at | HOOK1 | 3,746799001 | 3,654731437 | 0,092067563 | |
| 227525_at | GLCCI1 | 2,501229299 | 2,409170778 | 0,092058521 | |
| 210473_s_at | GPR125 | 1,542099044 | 1,450051551 | 0,092047493 | |
| 222200_s_at | BSDC1 | 2,416948786 | 2,324919719 | 0,092029067 | |
| 203682_s_at | IVD | 1,373456798 | 1,281471383 | 0,091985415 | |
| 211177_s_at | TXNRD2 | 1,373456798 | 1,281471383 | 0,091985415 | |
| 227248_at | PLEKHH3 | 0,695652136 | 0,603679647 | 0,091972489 | |
| 224076_s_at | WHSC1L1 | 2,621710292 | 2,529740643 | 0,091969649 | |
| 204899_s_at | SAP30 | 1,897847661 | 1,805902329 | 0,091945332 | |
| 232432_s_at | SLC30A5 | 3,750808107 | 3,658934427 | 0,09187368 | |
| 207808_s_at | PROS1 | 4,84391367 | 4,752066034 | 0,091847635 | |
| 1559361_at | MACC1 | -1,240169874 | -1,332017329 | 0,091847455 | |
| 1560891_a_at | LOC100505875 | -1,240169874 | -1,332017329 | 0,091847455 | |
| 1561293_at | - | -1,240169874 | -1,332017329 | 0,091847455 | |
| 1562447_a_at | LOC100506599 | -1,240169874 | -1,332017329 | 0,091847455 | |
| 1563063_at | - | -1,240169874 | -1,332017329 | 0,091847455 | |
| 1565406_a_at | LHX9 | -1,240169874 | -1,332017329 | 0,091847455 | |
| 213845_at | GRIK2 | -1,240169874 | -1,332017329 | 0,091847455 | |
| 215238_s_at | DOCK9 | -1,240169874 | -1,332017329 | 0,091847455 | |
| 217614_at | - | -1,240169874 | -1,332017329 | 0,091847455 | |
| 218903_s_at | NABP2 | -1,240169874 | -1,332017329 | 0,091847455 | |
| 223624_at | ZFAND4 | -1,240169874 | -1,332017329 | 0,091847455 | |
| 227809_at | ZC3H6 | -1,240169874 | -1,332017329 | 0,091847455 | |
| 230059_at | DEAF1 | -1,240169874 | -1,332017329 | 0,091847455 | |
| 231046_at | HP07349 /// LOC | -1,240169874 | -1,332017329 | 0,091847455 | |
| 233715_at | - | -1,240169874 | -1,332017329 | 0,091847455 | |
| 235715_at | SPRNP1 | -1,240169874 | -1,332017329 | 0,091847455 | |
| 236753_at | LOC154822 | -1,240169874 | -1,332017329 | 0,091847455 | |
| 238268_at | - | -1,240169874 | -1,332017329 | 0,091847455 | |
| 241630_at | - | -1,240169874 | -1,332017329 | 0,091847455 | |
| 207120_at | ZNF667 | 1,835789711 | 1,743944487 | 0,091845223 | |
| 229388_at | LOC100507212 | 1,283332461 | 1,191491281 | 0,09184118 | |
| 231723_at | SNX12 | 2,077135162 | 1,98530622 | 0,091828941 | |
| 229519_at | FXR1 | 3,330897748 | 3,239069096 | 0,091828652 | |
| 210774_s_at | NCOA4 | 4,625068394 | 4,533260944 | 0,091807449 | |
| 225308_s_at | TANC1 | 4,916735717 | 4,8249493 | 0,091786418 | |
| 233898_s_at | FGFR1OP2 | 2,663141434 | 2,571362843 | 0,091778591 | |
| 1555037_a_at | IDH1 | 3,378314944 | 3,28656411 | 0,091750834 | |
| 213762_x_at | RBMX /// SNORD | 5,858705693 | 5,766984279 | 0,091721415 | |
| 209307_at | SWAP70 | 2,626954721 | 2,53523821 | 0,091716511 | |
| 225209_s_at | UBE2J2 | 4,037433122 | 3,945725977 | 0,091707145 | |
| 211159_s_at | PPP2R5D | 2,549899391 | 2,458228496 | 0,091670895 | |

| | | | | | |
|---|---|---|---|---|---|
| 203609_s_at | ALDH5A1 | -0,299034815 | -0,39061235 | 0,091577535 | |
| 207770_x_at | CSH2 | -0,299034815 | -0,39061235 | 0,091577535 | |
| 211620_x_at | LOC100506403 / | -0,299034815 | -0,39061235 | 0,091577535 | |
| 216603_at | SLC7A8 | -0,299034815 | -0,39061235 | 0,091577535 | |
| 222089_s_at | C16orf71 | -0,299034815 | -0,39061235 | 0,091577535 | |
| 227405_s_at | FZD8 | -0,299034815 | -0,39061235 | 0,091577535 | |
| 238591_at | HEXDC | -0,299034815 | -0,39061235 | 0,091577535 | |
| 241150_at | SPTAN1 | -0,299034815 | -0,39061235 | 0,091577535 | |
| 243195_s_at | ZNF551 | -0,299034815 | -0,39061235 | 0,091577535 | |
| 213376_at | ZBTB1 | 3,137452663 | 3,045906378 | 0,091546285 | |
| 214484_s_at | SIGMAR1 | 3,111496958 | 3,019999252 | 0,091497706 | |
| 219767_s_at | CRYZL1 | 2,084778276 | 1,993317152 | 0,091461124 | |
| 233168_s_at | SELO | 2,084778276 | 1,993317152 | 0,091461124 | |
| 244038_at | WDR89 | 2,084778276 | 1,993317152 | 0,091461124 | |
| 202381_at | ADAM9 | 3,843828743 | 3,75237043 | 0,091458314 | |
| 216446_at | - | 0,384950573 | 0,293518836 | 0,091431738 | |
| 238527_at | KIAA0664L3 | 0,384950573 | 0,293518836 | 0,091431738 | |
| 209563_x_at | CALM1 /// CALM2 | 6,270964743 | 6,179548226 | 0,091416517 | |
| 213088_s_at | DNAJC9 | 5,834164609 | 5,742834983 | 0,091329626 | |
| 213485_s_at | ABCC10 | 2,635653293 | 2,544354523 | 0,09129877 | |
| 227245_at | NAA25 | 2,635653293 | 2,544354523 | 0,09129877 | |
| 1553581_s_at | SREK1IP1 | 3,957816838 | 3,866529339 | 0,091287499 | |
| 218817_at | SPCS3 | 2,559073676 | 2,467842989 | 0,091230687 | |
| 208754_s_at | NAP1L1 | 4,841283944 | 4,750097438 | 0,091186506 | |
| 219187_at | FKBPL | 2,344516784 | 2,253342112 | 0,091174672 | |
| 202559_x_at | CHTOP | 3,676882273 | 3,585762986 | 0,091119288 | |
| 223125_s_at | C1orf21 | 2,035673598 | 1,944570415 | 0,091103183 | |
| 202524_s_at | SPOCK2 | 0,20027154 | 0,109221392 | 0,091050147 | |
| 227358_at | ZBTB46 | 0,20027154 | 0,109221392 | 0,091050147 | |
| 224851_at | CDK6 | 3,008105382 | 2,917077481 | 0,091027902 | |
| 1557409_at | - | 0,715481152 | 0,624460759 | 0,091020392 | |
| 223833_at | WDR55 | 0,715481152 | 0,624460759 | 0,091020392 | |
| 228263_at | GRASP | 0,715481152 | 0,624460759 | 0,091020392 | |
| 230921_s_at | - | 0,715481152 | 0,624460759 | 0,091020392 | |
| 209906_at | C3AR1 | 1,481627361 | 1,390636111 | 0,09099125 | |
| 200803_s_at | TMBIM6 | 5,967957176 | 5,876972858 | 0,090984319 | |
| 239205_s_at | CR1 /// CR1L | 2,201862497 | 2,110887591 | 0,090974906 | |
| 209762_x_at | SP110 | 2,854131948 | 2,763166224 | 0,090965724 | |
| 200668_s_at | UBE2D3 | 5,890669844 | 5,799709572 | 0,090960271 | |
| 224564_s_at | RTN3 | 3,994797458 | 3,903840511 | 0,090956947 | |
| 212933_x_at | RPL13 /// SNORD | 7,111293807 | 7,020358154 | 0,090935653 | |
| 1553551_s_at | ND2 | 7,920503842 | 7,829574887 | 0,090928955 | |
| 200032_s_at | RPL9 | 7,766209003 | 7,675282385 | 0,090926618 | |
| 201726_at | ELAVL1 | 6,047030826 | 5,956125984 | 0,090904842 | |
| 232141_at | U2AF1 | 3,682769893 | 3,591931093 | 0,090838799 | |
| 211558_s_at | DHPS | 3,952952896 | 3,862161446 | 0,090791451 | |
| 212399_s_at | VGLL4 | 2,206540699 | 2,115788273 | 0,090752427 | |
| 203168_at | ATF6B | 0,984222186 | 0,893473268 | 0,090748918 | |
| 1554249_a_at | ZNF638 | 0,859074518 | 0,768343791 | 0,090730728 | |
| 213584_s_at | CREBZF | 0,57058931 | 0,479907041 | 0,090682269 | |
| 220685_at | FAM120C | 0,57058931 | 0,479907041 | 0,090682269 | |
| 221193_s_at | ZCCHC10 | 2,827046023 | 2,7363693 | 0,090676723 | |
| 205909_at | POLE2 | 4,509619341 | 4,418957488 | 0,090661853 | |
| 204779_s_at | HOXB7 | 0,401366113 | 0,310717132 | 0,090648981 | |
| 227851_s_at | - | 0,401366113 | 0,310717132 | 0,090648981 | |
| 241976_at | TCEA3 | 0,401366113 | 0,310717132 | 0,090648981 | |

| | | | | | |
|---|---|---|---|---|---|
| 202802_at | DHPS | 3,234554895 | 3,14392006 | 0,090634835 | |
| 210543_s_at | PRKDC | 3,972997622 | 3,882432751 | 0,09056487 | |
| 214144_at | POLR2D | 1,104397092 | 1,013870809 | 0,090526283 | |
| 241017_at | RPL31 /// TBC1D | 1,104397092 | 1,013870809 | 0,090526283 | |
| 1560690_at | - | -2,756478458 | -2,847001814 | 0,090523356 | |
| 1560758_at | - | -2,756478458 | -2,847001814 | 0,090523356 | |
| 1560810_at | - | -2,756478458 | -2,847001814 | 0,090523356 | |
| 1563107_at | - | -2,756478458 | -2,847001814 | 0,090523356 | |
| 1564656_at | - | -2,756478458 | -2,847001814 | 0,090523356 | |
| 1570206_at | - | -2,756478458 | -2,847001814 | 0,090523356 | |
| 210771_at | PPARA | -2,756478458 | -2,847001814 | 0,090523356 | |
| 211655_at | IGLC1 | -2,756478458 | -2,847001814 | 0,090523356 | |
| 216070_at | - | -2,756478458 | -2,847001814 | 0,090523356 | |
| 219864_s_at | RCAN3 | -2,756478458 | -2,847001814 | 0,090523356 | |
| 231173_at | PYROXD1 | -2,756478458 | -2,847001814 | 0,090523356 | |
| 231504_at | CCDC148 | -2,756478458 | -2,847001814 | 0,090523356 | |
| 231644_at | - | -2,756478458 | -2,847001814 | 0,090523356 | |
| 232424_at | PRDM16 | -2,756478458 | -2,847001814 | 0,090523356 | |
| 236948_x_at | SRSF11 | -2,756478458 | -2,847001814 | 0,090523356 | |
| 237573_at | - | -2,756478458 | -2,847001814 | 0,090523356 | |
| 240867_at | - | -2,756478458 | -2,847001814 | 0,090523356 | |
| 242088_at | KLHL24 | -2,756478458 | -2,847001814 | 0,090523356 | |
| 214553_s_at | ARPP19 | 1,865674241 | 1,775255987 | 0,090418254 | |
| 212076_at | MLL | 0,728550685 | 0,638150376 | 0,090400309 | |
| 217550_at | ATF6 | 0,728550685 | 0,638150376 | 0,090400309 | |
| 242369_x_at | - | 0,728550685 | 0,638150376 | 0,090400309 | |
| 224468_s_at | C19orf48 | 3,764356332 | 3,673964604 | 0,090391728 | |
| 203026_at | ZBTB5 | 3,161745659 | 3,071356473 | 0,090389186 | |
| 1554471_a_at | ANKRD13C | 1,314003737 | 1,223626587 | 0,090377149 | |
| 201815_s_at | TBC1D5 | 1,314003737 | 1,223626587 | 0,090377149 | |
| 218969_at | PAM16 | 2,799441827 | 2,709065209 | 0,090376618 | |
| 202580_x_at | FOXM1 | 2,361396186 | 2,271023131 | 0,090373055 | |
| 209251_x_at | TUBA1C | 7,651341382 | 7,561013979 | 0,090327404 | |
| 210830_s_at | PON2 | 2,963199262 | 2,87289275 | 0,090306511 | |
| 229356_x_at | INO80 | 3,081249977 | 2,990953566 | 0,090296411 | |
| 223455_at | TCHP | 2,31665944 | 2,226407528 | 0,090251912 | |
| 1564194_a_at | MS4A15 | -1,173767127 | -1,264005341 | 0,090238214 | |
| 200785_s_at | LRP1 | -1,173767127 | -1,264005341 | 0,090238214 | |
| 205610_at | MYOM1 | -1,173767127 | -1,264005341 | 0,090238214 | |
| 215557_at | - | -1,173767127 | -1,264005341 | 0,090238214 | |
| 216029_at | - | -1,173767127 | -1,264005341 | 0,090238214 | |
| 222712_s_at | MUC13 | -1,173767127 | -1,264005341 | 0,090238214 | |
| 223600_s_at | KIAA1683 | -1,173767127 | -1,264005341 | 0,090238214 | |
| 229423_at | CHEK1 | -1,173767127 | -1,264005341 | 0,090238214 | |
| 230211_at | TRIP11 | -1,173767127 | -1,264005341 | 0,090238214 | |
| 230433_at | LOC729970 | -1,173767127 | -1,264005341 | 0,090238214 | |
| 230855_at | GATA4 | -1,173767127 | -1,264005341 | 0,090238214 | |
| 223084_s_at | CCNDBP1 | 3,497632487 | 3,407399455 | 0,090233032 | |
| 211628_x_at | FTH1P5 | 6,796477013 | 6,706264935 | 0,090212078 | |
| 225392_at | GFM2 | 3,80348216 | 3,713295978 | 0,090186181 | |
| 238474_at | NUP43 | 2,365585359 | 2,275409734 | 0,090175625 | |
| 208764_s_at | ATP5G2 | 6,062757787 | 5,972597079 | 0,090160707 | |
| 203284_s_at | HS2ST1 /// LOC3 | 4,233538894 | 4,143455947 | 0,090082947 | |
| 214920_at | THSD7A | 1,414471577 | 1,324440845 | 0,090030732 | |
| 218670_at | PUS1 | 1,414471577 | 1,324440845 | 0,090030732 | |
| 219861_at | DNAJC17 | 1,414471577 | 1,324440845 | 0,090030732 | |

| | | | | | |
|---|---|---|---|---|---|
| 207017_at | RAB27B | 3,700289946 | 3,61027875 | 0,090011196 | |
| 200945_s_at | SEC31A | 4,851400868 | 4,761478085 | 0,089922783 | |
| 212351_at | EIF2B5 | 2,940912947 | 2,851017268 | 0,089895679 | |
| 230032_at | OSGEPL1 | 1,119359127 | 1,029536893 | 0,089822234 | |
| 235698_at | ZFP90 | 1,119359127 | 1,029536893 | 0,089822234 | |
| 237306_at | ZNF829 | 1,119359127 | 1,029536893 | 0,089822234 | |
| 203738_at | C5orf22 | 3,277429131 | 3,187631636 | 0,089797494 | |
| 1557164_a_at | - | -0,259832361 | -0,34958478 | 0,089752419 | |
| 208269_s_at | ADAM28 | -0,259832361 | -0,34958478 | 0,089752419 | |
| 210628_x_at | LTBP4 | -0,259832361 | -0,34958478 | 0,089752419 | |
| 216641_s_at | LAD1 | -0,259832361 | -0,34958478 | 0,089752419 | |
| 234015_at | - | -0,259832361 | -0,34958478 | 0,089752419 | |
| 236832_at | ADCY10P1 | -0,259832361 | -0,34958478 | 0,089752419 | |
| 242683_at | LOC400643 | -0,259832361 | -0,34958478 | 0,089752419 | |
| 224664_at | ANAPC16 | 4,539590968 | 4,449849671 | 0,089741297 | |
| 224372_at | ND4 | 8,236486636 | 8,146766531 | 0,089720106 | |
| 201684_s_at | TOX4 | 3,706908684 | 3,617207412 | 0,089701272 | |
| 45714_at | HCFC1R1 | 2,036987472 | 1,947322213 | 0,089665259 | |
| 1553984_s_at | DTYMK | 4,41122478 | 4,321571774 | 0,089653006 | |
| 227164_at | SRSF1 | 2,745814053 | 2,656223838 | 0,089590215 | |
| 201316_at | PSMA2 | 3,205636958 | 3,116064987 | 0,089571971 | |
| 212751_at | UBE2N | 4,563948403 | 4,474382017 | 0,089566386 | |
| 218038_at | ATP5SL | 2,446911392 | 2,357351836 | 0,089559555 | |
| 226981_at | MLL | 2,446911392 | 2,357351836 | 0,089559555 | |
| 205327_s_at | ACVR2A | 0,425644434 | 0,336136178 | 0,089508256 | |
| 213667_at | LOC100862671 / | 0,425644434 | 0,336136178 | 0,089508256 | |
| 238746_at | PXMP4 | 0,425644434 | 0,336136178 | 0,089508256 | |
| 1556551_s_at | SLC39A6 | 0,747935635 | 0,658444279 | 0,089491356 | |
| 231550_at | XYLT2 | 0,747935635 | 0,658444279 | 0,089491356 | |
| 216317_x_at | RHCE | 1,011212064 | 0,921731975 | 0,089480089 | |
| 219811_at | DGCR8 /// MIR13 | 1,233777334 | 1,144305291 | 0,089472043 | |
| 219880_at | LOC100507619 | 1,233777334 | 1,144305291 | 0,089472043 | |
| 229035_s_at | KLHDC4 /// LOC1 | 1,426552034 | 1,337086025 | 0,089466009 | |
| 35150_at | CD40 | 2,801762457 | 2,712304406 | 0,089458051 | |
| 203060_s_at | PAPSS2 | 1,748661581 | 1,659204017 | 0,089457565 | |
| 210978_s_at | TAGLN2 | 5,327862673 | 5,238417317 | 0,089445356 | |
| 209644_x_at | CDKN2A | 6,570844973 | 6,48142129 | 0,089423683 | |
| 228285_at | TDRD9 | 3,837056482 | 3,747642277 | 0,089414205 | |
| 213374_x_at | HIBCH | 4,273659132 | 4,18426834 | 0,089390792 | |
| 203669_s_at | DGAT1 | 2,600539826 | 2,511262629 | 0,089277196 | |
| 215136_s_at | EXOSC8 | 4,529358299 | 4,440114107 | 0,089244192 | |
| 1554958_at | ZNF641 | -0,59053611 | -0,679763839 | 0,089227729 | |
| 202516_s_at | DLG1 | -0,59053611 | -0,679763839 | 0,089227729 | |
| 211403_x_at | VCX2 | -0,59053611 | -0,679763839 | 0,089227729 | |
| 214331_at | TSFM | -0,59053611 | -0,679763839 | 0,089227729 | |
| 224194_at | FCRL2 | -0,59053611 | -0,679763839 | 0,089227729 | |
| 228376_at | GGTA1P | -0,59053611 | -0,679763839 | 0,089227729 | |
| 229264_at | - | -0,59053611 | -0,679763839 | 0,089227729 | |
| 233614_at | - | -0,59053611 | -0,679763839 | 0,089227729 | |
| 235665_at | PTOV1 | -0,59053611 | -0,679763839 | 0,089227729 | |
| 239910_at | PSG6 | -0,59053611 | -0,679763839 | 0,089227729 | |
| 244332_at | - | -0,59053611 | -0,679763839 | 0,089227729 | |
| 1555339_at | RAP1A | -1,131062212 | -1,220210588 | 0,089148375 | |
| 1555425_x_at | SSH2 | -1,131062212 | -1,220210588 | 0,089148375 | |
| 1559033_at | LOC255167 | -1,131062212 | -1,220210588 | 0,089148375 | |
| 1560117_at | ABHD1 | -1,131062212 | -1,220210588 | 0,089148375 | |

| | | | | | |
|---|---|---|---|---|---|
| 1562103_at | - | -1,131062212 | -1,220210588 | 0,089148375 | |
| 211057_at | ROR1 | -1,131062212 | -1,220210588 | 0,089148375 | |
| 214342_at | ATXN7L1 | -1,131062212 | -1,220210588 | 0,089148375 | |
| 219090_at | SLC24A3 | -1,131062212 | -1,220210588 | 0,089148375 | |
| 220369_at | SMEK1 | -1,131062212 | -1,220210588 | 0,089148375 | |
| 221947_at | - | -1,131062212 | -1,220210588 | 0,089148375 | |
| 223426_s_at | EPB41L4B | -1,131062212 | -1,220210588 | 0,089148375 | |
| 227614_at | HKDC1 | -1,131062212 | -1,220210588 | 0,089148375 | |
| 234004_at | TTC28 | -1,131062212 | -1,220210588 | 0,089148375 | |
| 241859_at | PLCL1 | -1,131062212 | -1,220210588 | 0,089148375 | |
| 244420_at | - | -1,131062212 | -1,220210588 | 0,089148375 | |
| 218347_at | TYW1 /// TYW1B | 2,562727117 | 2,473581079 | 0,089146038 | |
| 225226_at | FAM40A | 2,791679321 | 2,702564916 | 0,089114406 | |
| 223477_s_at | C12orf65 | 2,522021211 | 2,432926755 | 0,089094456 | |
| 208906_at | BSCL2 /// HNRNI | 2,243430036 | 2,154405725 | 0,089024311 | |
| 226576_at | ARHGAP26 | 0,606430281 | 0,51742585 | 0,089004431 | |
| 230670_at | IGSF10 | 0,606430281 | 0,51742585 | 0,089004431 | |
| 222614_at | RWDD2B | 1,021868205 | 0,932882274 | 0,088985931 | |
| 231952_at | - | 1,021868205 | 0,932882274 | 0,088985931 | |
| 200033_at | DDX5 | 6,704180463 | 6,61521708 | 0,088963382 | |
| 204546_at | KIAA0513 | 0,760715727 | 0,67181667 | 0,088899057 | |
| 234799_at | ADARB1 | 0,760715727 | 0,67181667 | 0,088899057 | |
| 237895_at | - | 0,760715727 | 0,67181667 | 0,088899057 | |
| 223141_at | UCK1 | 2,568189989 | 2,479296438 | 0,088893552 | |
| 210442_at | IL1RL1 | 0,246434561 | 0,157549243 | 0,088885318 | |
| 212504_at | DIP2C | 0,246434561 | 0,157549243 | 0,088885318 | |
| 238423_at | SYTL3 | 0,246434561 | 0,157549243 | 0,088885318 | |
| 205228_at | RBMS2 | 0,44160593 | 0,352836757 | 0,088769173 | |
| 217580_x_at | - | 0,44160593 | 0,352836757 | 0,088769173 | |
| 219227_at | CCNJL | 0,44160593 | 0,352836757 | 0,088769173 | |
| 219699_at | LGI2 | 0,44160593 | 0,352836757 | 0,088769173 | |
| 229637_at | RIC8B | 0,44160593 | 0,352836757 | 0,088769173 | |
| 233764_s_at | LARP1B | 0,44160593 | 0,352836757 | 0,088769173 | |
| 212636_at | QKI | 3,223057183 | 3,134292219 | 0,088764964 | |
| 219373_at | DPM3 | 3,170152481 | 3,081412119 | 0,088740361 | |
| 209366_x_at | CYB5A | 1,764611101 | 1,675891488 | 0,088719613 | |
| 226952_at | EAF1 | 3,325531666 | 3,236821511 | 0,088710155 | |
| 214056_at | MCL1 | 2,487840343 | 2,39915583 | 0,088684513 | |
| 238865_at | PABPC4L | 4,171484821 | 4,082805739 | 0,088679082 | |
| 227363_s_at | EMC8 | 4,663430187 | 4,574783153 | 0,088647034 | |
| 204557_s_at | DZIP1 | 1,968405881 | 1,879785595 | 0,088620286 | |
| 227576_at | - | 1,089278259 | 1,000684521 | 0,088593738 | |
| 218309_at | CAMK2N1 | -0,234278768 | -0,322870281 | 0,088591514 | |
| 233440_at | - | -0,234278768 | -0,322870281 | 0,088591514 | |
| 235257_at | ODF3B | -0,234278768 | -0,322870281 | 0,088591514 | |
| 240537_s_at | LOC440356 | -0,234278768 | -0,322870281 | 0,088591514 | |
| 243189_at | NRF1 | -0,234278768 | -0,322870281 | 0,088591514 | |
| 202686_s_at | AXL | 4,576652364 | 4,488147962 | 0,088504402 | |
| 203500_at | GCDH | 2,254767157 | 2,166264721 | 0,088502436 | |
| 224640_at | SPPL3 | 2,618203384 | 2,529740643 | 0,088462741 | |
| 212170_at | RBM12 | 2,402750389 | 2,314300277 | 0,088450112 | |
| 210612_s_at | SYNJ2 | 2,094906514 | 2,006570641 | 0,088335873 | |
| 213064_at | ZC3H14 | 4,08075718 | 3,992437318 | 0,088319861 | |
| 201393_s_at | IGF2R | 3,680249569 | 3,591931093 | 0,088318475 | |
| 224695_at | C2orf29 | 3,406034785 | 3,317734938 | 0,088299847 | |
| 214585_s_at | VPS52 | 3,474545391 | 3,386261038 | 0,088284353 | |

| | | | | | |
|---|---|---|---|---|---|
| 1556457_s_at | FLJ39739 | -2,866296344 | -2,954576755 | 0,088280411 | |
| 1560869_a_at | - | -2,866296344 | -2,954576755 | 0,088280411 | |
| 1570030_at | ZNF396 | -2,866296344 | -2,954576755 | 0,088280411 | |
| 208317_at | XYLB | -2,866296344 | -2,954576755 | 0,088280411 | |
| 232445_at | SYT9 | -2,866296344 | -2,954576755 | 0,088280411 | |
| 237458_at | - | -2,866296344 | -2,954576755 | 0,088280411 | |
| 242025_at | - | -2,866296344 | -2,954576755 | 0,088280411 | |
| 244224_x_at | LYNX1 | -2,866296344 | -2,954576755 | 0,088280411 | |
| 207098_s_at | MFN1 | 3,037284587 | 2,949006836 | 0,088277751 | |
| 1552378_s_at | RDH10 | 1,037706275 | 0,949447784 | 0,088258491 | |
| 213070_at | PIK3C2A | 2,497416546 | 2,409170778 | 0,088245768 | |
| 203441_s_at | CDH2 | 2,038300149 | 1,950068772 | 0,088231377 | |
| 218356_at | FTSJ2 | 3,62365843 | 3,535445117 | 0,088213313 | |
| 53987_at | RANBP10 | 1,311834425 | 1,223626587 | 0,088207837 | |
| 212773_s_at | TOMM20 | 4,385669548 | 4,297510916 | 0,088158632 | |
| 213127_s_at | MED8 | 2,314494121 | 2,226407528 | 0,088086593 | |
| 209657_s_at | HSF2 | 3,565667132 | 3,477609204 | 0,088057928 | |
| 205700_at | HSD17B6 | 0,779676066 | 0,691645685 | 0,08803038 | |
| 209623_at | MCCC2 | 4,743272317 | 4,655247494 | 0,088024824 | |
| 223099_s_at | LONP2 | 1,982112682 | 1,894118818 | 0,087993865 | |
| 224364_s_at | PPIL3 | 4,648465551 | 4,560475791 | 0,08798976 | |
| 1554441_a_at | WAPAL | 2,546213279 | 2,458228496 | 0,087984783 | |
| 225004_at | TMEM101 | 1,85379461 | 1,765933685 | 0,087860925 | |
| 223697_x_at | C9orf64 | 3,854300588 | 3,766462619 | 0,087837969 | |
| 218161_s_at | CLN6 | 1,549482847 | 1,461646549 | 0,087836298 | |
| 224970_at | NFIA | 1,549482847 | 1,461646549 | 0,087836298 | |
| 209971_x_at | AIMP2 | 5,196752925 | 5,108917684 | 0,087835241 | |
| 216945_x_at | PASK | 1,369290453 | 1,281471383 | 0,08781907 | |
| 202779_s_at | UBE2S | 5,50848942 | 5,420747189 | 0,087742231 | |
| 215460_x_at | BRD1 | 1,923647937 | 1,835911187 | 0,087736751 | |
| 209889_at | SEC31B | 0,46522187 | 0,377530647 | 0,087691223 | |
| 1552978_a_at | SCAMP1 | 1,168140757 | 1,080561626 | 0,087579132 | |
| 222793_at | DDX58 | 1,168140757 | 1,080561626 | 0,087579132 | |
| 212456_at | KIAA0664 | 3,249366227 | 3,161800893 | 0,087565333 | |
| 201361_at | TMEM109 | 2,960432227 | 2,87289275 | 0,087539477 | |
| 1559006_at | - | 1,556829052 | 1,469325098 | 0,087503954 | |
| 1560349_at | - | -2,908843709 | -2,996335703 | 0,087491994 | |
| 1561493_at | - | -2,908843709 | -2,996335703 | 0,087491994 | |
| 1561857_at | - | -2,908843709 | -2,996335703 | 0,087491994 | |
| 1563858_at | - | -2,908843709 | -2,996335703 | 0,087491994 | |
| 1566253_at | SH3GL1P1 | -2,908843709 | -2,996335703 | 0,087491994 | |
| 1566952_at | - | -2,908843709 | -2,996335703 | 0,087491994 | |
| 1569054_at | SLC1A3 | -2,908843709 | -2,996335703 | 0,087491994 | |
| 204530_s_at | TOX | -2,908843709 | -2,996335703 | 0,087491994 | |
| 205466_s_at | HS3ST1 | -2,908843709 | -2,996335703 | 0,087491994 | |
| 215647_at | - | -2,908843709 | -2,996335703 | 0,087491994 | |
| 220772_at | BPESC1 | -2,908843709 | -2,996335703 | 0,087491994 | |
| 222330_at | - | -2,908843709 | -2,996335703 | 0,087491994 | |
| 226425_at | CLIP4 | -2,908843709 | -2,996335703 | 0,087491994 | |
| 227874_at | EMCN | -2,908843709 | -2,996335703 | 0,087491994 | |
| 228863_at | PCDH17 | -2,908843709 | -2,996335703 | 0,087491994 | |
| 234325_at | C2orf65 | -2,908843709 | -2,996335703 | 0,087491994 | |
| 236773_at | - | -2,908843709 | -2,996335703 | 0,087491994 | |
| 240472_at | - | -2,908843709 | -2,996335703 | 0,087491994 | |
| 241761_at | - | -2,908843709 | -2,996335703 | 0,087491994 | |
| 242313_at | LOC728730 | -2,908843709 | -2,996335703 | 0,087491994 | |

| | | | | | |
|---|---|---|---|---|---|
| 243307_at | - | -2,908843709 | -2,996335703 | 0,087491994 | |
| 221570_s_at | METTL5 | 5,453465706 | 5,366011631 | 0,087454075 | |
| 202535_at | FADD | 2,640847372 | 2,553413592 | 0,08743378 | |
| 218133_s_at | NIF3L1 | 3,401961598 | 3,314541423 | 0,087420175 | |
| 204341_at | TRIM16 | 0,641402394 | 0,553993624 | 0,08740877 | |
| 216711_s_at | TAF1 | 0,641402394 | 0,553993624 | 0,08740877 | |
| 219236_at | PAQR6 | 0,641402394 | 0,553993624 | 0,08740877 | |
| 222343_at | BCL2L11 | 0,641402394 | 0,553993624 | 0,08740877 | |
| 230792_at | FAAH2 | 0,641402394 | 0,553993624 | 0,08740877 | |
| 208369_s_at | GCDH | 1,995690482 | 1,908311039 | 0,087379443 | |
| 242253_at | LOC100506012 | 0,934330293 | 0,847075464 | 0,087254829 | |
| 223198_x_at | COMMD5 | 3,999522577 | 3,912315453 | 0,087207124 | |
| 225181_at | ARID1B | 3,148435161 | 3,061230246 | 0,087204915 | |
| 202129_s_at | RIOK3 | 2,28383338 | 2,196649505 | 0,087183875 | |
| 205891_at | ADORA2B /// LO( | 2,834621043 | 2,747463672 | 0,087157371 | |
| 217456_x_at | HLA-E | 4,309979162 | 4,222822694 | 0,087156468 | |
| 224569_s_at | IRF2BP2 | 5,099184497 | 5,012029981 | 0,087154515 | |
| 225616_at | SPRYD4 | 1,177702228 | 1,090553537 | 0,08714869 | |
| 204852_s_at | PTPN7 | 2,003776245 | 1,916759838 | 0,087016407 | |
| 228776_at | GJC1 | 1,567778694 | 1,480766833 | 0,087011861 | |
| 1557944_s_at | CTNND1 /// TMX | 0,480753764 | 0,393761504 | 0,086992259 | |
| 207883_s_at | TFR2 | 0,480753764 | 0,393761504 | 0,086992259 | |
| 219417_s_at | C17orf59 | 0,480753764 | 0,393761504 | 0,086992259 | |
| 228994_at | CCDC24 | 0,480753764 | 0,393761504 | 0,086992259 | |
| 202073_at | OPTN | -0,196756757 | -0,283667828 | 0,08691107 | |
| 211384_s_at | CASR | -0,196756757 | -0,283667828 | 0,08691107 | |
| 230039_at | - | -0,196756757 | -0,283667828 | 0,08691107 | |
| 239012_at | RNF144B | -0,196756757 | -0,283667828 | 0,08691107 | |
| 239064_at | - | -0,196756757 | -0,283667828 | 0,08691107 | |
| 212864_at | CDS2 | 2,768138014 | 2,681234383 | 0,086903631 | |
| 227568_at | HECTD2 | 1,390002893 | 1,303116084 | 0,086886809 | |
| 238316_at | ZNF567 | 0,291166226 | 0,204310755 | 0,08685547 | |
| 202047_s_at | CBX6 | 2,654607516 | 2,567790814 | 0,086816703 | |
| 212556_at | SCRIB | 4,171484821 | 4,084681583 | 0,086803238 | |
| 208924_at | RNF11 | 4,085209408 | 3,9984201 | 0,086789308 | |
| 222468_at | KIAA0319L | 1,485482016 | 1,398700978 | 0,086781037 | |
| 211434_s_at | CCRL2 | 1,575032557 | 1,488344553 | 0,086688004 | |
| 202057_at | KPNA1 | 1,073999308 | 0,987376598 | 0,086622711 | |
| 244411_at | LOC100507316 | 1,659347917 | 1,572742379 | 0,086605538 | |
| 203564_at | FANCG | 1,883312263 | 1,796776588 | 0,086535676 | |
| 226089_at | RABL3 | 1,951783949 | 1,865308541 | 0,086475407 | |
| 218515_at | GCFC1 | 4,493918448 | 4,407512498 | 0,08640595 | |
| 200052_s_at | ILF2 | 5,770427329 | 5,684038418 | 0,086388911 | |
| 211136_s_at | CLPTM1 | 3,049053496 | 2,962671751 | 0,086381745 | |
| 222526_at | GATAD2A | 3,302773593 | 3,216434104 | 0,086339489 | |
| 61874_at | CACFD1 | 2,354035736 | 2,267724404 | 0,086311332 | |
| 203302_at | DCK | 4,004232272 | 3,917937882 | 0,086294389 | |
| 1558647_at | SH3D19 | -1,026571149 | -1,112851844 | 0,086280695 | |
| 1564138_at | FBXW12 | -1,026571149 | -1,112851844 | 0,086280695 | |
| 207715_at | CRYGB | -1,026571149 | -1,112851844 | 0,086280695 | |
| 210348_at | 38231 | -1,026571149 | -1,112851844 | 0,086280695 | |
| 213592_at | APLNR | -1,026571149 | -1,112851844 | 0,086280695 | |
| 217475_s_at | LIMK2 | -1,026571149 | -1,112851844 | 0,086280695 | |
| 222084_s_at | SBF1 | -1,026571149 | -1,112851844 | 0,086280695 | |
| 227498_at | SOX6 | -1,026571149 | -1,112851844 | 0,086280695 | |
| 227956_at | ITPRIPL2 | -1,026571149 | -1,112851844 | 0,086280695 | |

| | | | | | |
|---|---|---|---|---|---|
| 230279_at | LOC100507540 | -1,026571149 | -1,112851844 | 0,086280695 | |
| 242995_at | - | -1,026571149 | -1,112851844 | 0,086280695 | |
| 202085_at | TJP2 | 4,082030648 | 3,995764148 | 0,086266499 | |
| 218414_s_at | NDE1 | 2,022468633 | 1,936283387 | 0,086185246 | |
| 229570_at | - | 0,822971223 | 0,73688026 | 0,086090963 | |
| 238651_at | - | 0,822971223 | 0,73688026 | 0,086090963 | |
| 1557120_at | EEF1A1 | 0,30867765 | 0,222599095 | 0,086078555 | |
| 220819_at | FRMD1 | 0,30867765 | 0,222599095 | 0,086078555 | |
| 236924_at | - | 0,30867765 | 0,222599095 | 0,086078555 | |
| 239498_at | - | 0,30867765 | 0,222599095 | 0,086078555 | |
| 208909_at | UQCRFS1 | 7,614028608 | 7,527961716 | 0,086066892 | |
| 204009_s_at | KRAS | 3,651373876 | 3,565308644 | 0,086065232 | |
| 227034_at | SOWAHC | 3,310399576 | 3,22439681 | 0,086002766 | |
| 1563030_at | - | 0,503742507 | 0,417770464 | 0,085972044 | |
| 221577_x_at | GDF15 | 0,503742507 | 0,417770464 | 0,085972044 | |
| 1570266_x_at | ERVH-1 | 1,751865623 | 1,665902186 | 0,085963437 | |
| 204258_at | CHD1 | 3,3865839 | 3,300620584 | 0,085963316 | |
| 1565817_at | IKZF1 | 0,67554678 | 0,589657387 | 0,085889393 | |
| 212735_at | KIAA0226 | 0,67554678 | 0,589657387 | 0,085889393 | |
| 224728_at | ATPAF1 | 1,67631007 | 1,590454215 | 0,085855855 | |
| 1554078_s_at | DNAJA3 | 4,076291169 | 3,990437532 | 0,085853637 | |
| 201705_at | PSMD7 | 4,43733059 | 4,351483879 | 0,085846711 | |
| 1555021_a_at | SCARF1 | -0,172274514 | -0,258114234 | 0,08583972 | |
| 1563802_at | LOC284551 | -0,172274514 | -0,258114234 | 0,08583972 | |
| 222107_x_at | LZTS1 | -0,172274514 | -0,258114234 | 0,08583972 | |
| 230785_at | - | -0,172274514 | -0,258114234 | 0,08583972 | |
| 238143_at | LOC646627 | -0,172274514 | -0,258114234 | 0,08583972 | |
| 221952_x_at | TRMT5 | 6,535143884 | 6,449325479 | 0,085818405 | |
| 1553910_at | NBPF4 | 1,829737795 | 1,743944487 | 0,085793308 | |
| 212436_at | TRIM33 | 2,208874124 | 2,123108223 | 0,085765901 | |
| 203614_at | UTP14C | 3,919150955 | 3,833443169 | 0,085707786 | |
| 209695_at | PTP4A3 | 2,680059304 | 2,594367918 | 0,085691387 | |
| 228379_at | LOC128322 /// NU | 2,035673598 | 1,950068772 | 0,085604826 | |
| 227060_at | RELT | 1,683039483 | 1,59747851 | 0,085560973 | |
| 200054_at | ZNF259 | 3,600740871 | 3,515187047 | 0,085553824 | |
| 210968_s_at | RTN4 | 5,528477288 | 5,442974965 | 0,085502323 | |
| 238154_at | CEP70 | 1,215326747 | 1,129843209 | 0,085483538 | |
| 204742_s_at | PDS5B | 1,422536444 | 1,337086025 | 0,085450419 | |
| 220072_at | CSPP1 | 1,603688695 | 1,518263577 | 0,085425117 | |
| 215847_at | HERC2P3 | 0,518867317 | 0,433557298 | 0,085310019 | |
| 201211_s_at | DDX3X | 3,191547726 | 3,106248809 | 0,085298917 | |
| 202138_x_at | AIMP2 | 5,027297124 | 4,942014748 | 0,085282376 | |
| 226223_at | PAWR | 2,427005622 | 2,341749893 | 0,085255729 | |
| 212072_s_at | CSNK2A1 /// CSN | 5,308953409 | 5,223738367 | 0,085215042 | |
| 1555120_at | CD96 | -3,013665402 | -3,098858316 | 0,085192914 | |
| 1556583_a_at | SLC8A1 | -3,013665402 | -3,098858316 | 0,085192914 | |
| 1556904_at | - | -3,013665402 | -3,098858316 | 0,085192914 | |
| 1557631_at | - | -3,013665402 | -3,098858316 | 0,085192914 | |
| 1560424_at | - | -3,013665402 | -3,098858316 | 0,085192914 | |
| 1561707_at | LOC150185 | -3,013665402 | -3,098858316 | 0,085192914 | |
| 1563519_at | - | -3,013665402 | -3,098858316 | 0,085192914 | |
| 1564965_at | - | -3,013665402 | -3,098858316 | 0,085192914 | |
| 1565693_at | DTYMK | -3,013665402 | -3,098858316 | 0,085192914 | |
| 204938_s_at | PLN | -3,013665402 | -3,098858316 | 0,085192914 | |
| 217394_at | - | -3,013665402 | -3,098858316 | 0,085192914 | |
| 224126_at | SLC10A7 | -3,013665402 | -3,098858316 | 0,085192914 | |

| | | | | | |
|---|---|---|---|---|---|
| 233102_at | - | -3,013665402 | -3,098858316 | 0,085192914 | |
| 233499_at | LRRC7 | -3,013665402 | -3,098858316 | 0,085192914 | |
| 242549_at | PRKD3 | -3,013665402 | -3,098858316 | 0,085192914 | |
| 244288_s_at | - | -3,013665402 | -3,098858316 | 0,085192914 | |
| 244560_at | - | -3,013665402 | -3,098858316 | 0,085192914 | |
| 214710_s_at | CCNB1 | 5,335891383 | 5,250722366 | 0,085169017 | |
| 208751_at | NAPA | 1,844820248 | 1,759685182 | 0,085135066 | |
| 201378_s_at | UBAP2L | 3,225364068 | 3,140317145 | 0,085046923 | |
| 210554_s_at | CTBP2 | 3,408067074 | 3,323041804 | 0,08502527 | |
| 220181_x_at | SLC30A5 | 2,227406889 | 2,14244844 | 0,084958449 | |
| 232593_at | NEURL3 | 0,334552598 | 0,249604176 | 0,084948422 | |
| 205446_s_at | ATF2 | 1,850809354 | 1,765933685 | 0,08487567 | |
| 203821_at | HBEGF | 1,114389003 | 1,029536893 | 0,08485211 | |
| 213207_s_at | GOSR2 | 1,114389003 | 1,029536893 | 0,08485211 | |
| 219888_at | SPAG4 | 1,114389003 | 1,029536893 | 0,08485211 | |
| 226093_at | DCP1B | 1,114389003 | 1,029536893 | 0,08485211 | |
| 204151_x_at | AKR1C1 | 5,151980116 | 5,06714968 | 0,084830436 | |
| 214773_x_at | TIPRL | 2,922548342 | 2,837730973 | 0,084817369 | |
| 209061_at | NCOA3 | 2,338135744 | 2,253342112 | 0,084793631 | |
| 225597_at | SLC45A4 | 2,338135744 | 2,253342112 | 0,084793631 | |
| 221897_at | TRIM52 | 0,853119696 | 0,768343791 | 0,084775905 | |
| 204977_at | DDX10 | 4,131454119 | 4,046693299 | 0,08476082 | |
| 215383_x_at | SPG21 | 1,621313976 | 1,536652708 | 0,084661269 | |
| 52731_at | AMBRA1 | 1,892051064 | 1,80741769 | 0,084633374 | |
| 209651_at | TGFB1I1 | 1,534677255 | 1,450051551 | 0,084625704 | |
| 201771_at | SCAMP3 | 3,76356289 | 3,678940066 | 0,084622823 | |
| 200720_s_at | ACTR1A | 3,927675977 | 3,843079586 | 0,084596391 | |
| 217094_s_at | ITCH | 2,623460554 | 2,538891651 | 0,084568903 | |
| 226143_at | RAI1 | 2,666540916 | 2,582026171 | 0,084514745 | |
| 222582_at | PRKAG2 | 1,238353345 | 1,153866761 | 0,084486584 | |
| 207809_s_at | ATP6AP1 | 3,562021138 | 3,477609204 | 0,084411934 | |
| 230733_at | - | 1,709647566 | 1,6252386 | 0,084408965 | |
| 1552480_s_at | PTPRC | -3,054236653 | -3,138626879 | 0,084390226 | |
| 1555572_at | CA6 | -3,054236653 | -3,138626879 | 0,084390226 | |
| 1559606_at | GBP6 | -3,054236653 | -3,138626879 | 0,084390226 | |
| 1560990_a_at | - | -3,054236653 | -3,138626879 | 0,084390226 | |
| 205501_at | PDE10A | -3,054236653 | -3,138626879 | 0,084390226 | |
| 207256_at | MBL2 | -3,054236653 | -3,138626879 | 0,084390226 | |
| 207272_at | ZNF80 | -3,054236653 | -3,138626879 | 0,084390226 | |
| 214981_at | POSTN | -3,054236653 | -3,138626879 | 0,084390226 | |
| 216435_at | MAP2K5 | -3,054236653 | -3,138626879 | 0,084390226 | |
| 217147_s_at | TRAT1 | -3,054236653 | -3,138626879 | 0,084390226 | |
| 221805_at | NEFL | -3,054236653 | -3,138626879 | 0,084390226 | |
| 227702_at | CYP4X1 | -3,054236653 | -3,138626879 | 0,084390226 | |
| 237530_at | - | -3,054236653 | -3,138626879 | 0,084390226 | |
| 241635_at | - | -3,054236653 | -3,138626879 | 0,084390226 | |
| 242791_at | FBXO3 | -3,054236653 | -3,138626879 | 0,084390226 | |
| 243159_x_at | - | -3,054236653 | -3,138626879 | 0,084390226 | |
| 210231_x_at | SET | 6,212860491 | 6,128482074 | 0,084378417 | |
| 205756_s_at | F8 | 0,541261317 | 0,456918297 | 0,084343019 | |
| 210291_s_at | ZNF174 | 0,541261317 | 0,456918297 | 0,084343019 | |
| 212557_at | ZNF451 | 3,899061119 | 3,814728712 | 0,084332407 | |
| 206612_at | CACNG1 | -0,136301282 | -0,220592223 | 0,084290941 | |
| 209488_s_at | RBPMS | -0,136301282 | -0,220592223 | 0,084290941 | |
| 223607_x_at | ZSWIM1 | 2,752227654 | 2,667949403 | 0,084278251 | |
| 212115_at | HN1L | 4,791165053 | 4,706906619 | 0,084258434 | |

| | | | | | |
|---|---|---|---|---|---|
| 225793_at | LIX1L | 3,401961598 | 3,317734938 | 0,08422666 | |
| 212967_x_at | NAP1L1 | 6,230624919 | 6,146404405 | 0,084220514 | |
| 202665_s_at | WIPF1 | 0,351548291 | 0,26733076 | 0,084217531 | |
| 208742_s_at | SAP18 | 6,395776901 | 6,311619003 | 0,084157898 | |
| 204004_at | PAWR | 1,716223609 | 1,632095957 | 0,084127652 | |
| 208802_at | SRP72 | 4,190500971 | 4,106388091 | 0,08411288 | |
| 235615_at | PGGT1B | 2,59162628 | 2,507538456 | 0,084087824 | |
| 202593_s_at | GDE1 | 3,132544588 | 3,048471686 | 0,084072902 | |
| 1552633_at | ZNF101 | 0,87091093 | 0,786897721 | 0,084013209 | |
| 204893_s_at | ZFYVE9 | 0,87091093 | 0,786897721 | 0,084013209 | |
| 1554768_a_at | MAD2L1 | 5,149552753 | 5,065567526 | 0,083985228 | |
| 226108_at | ZC3H18 | 2,250242994 | 2,166264721 | 0,083978273 | |
| 203378_at | PCF11 | 2,456762221 | 2,372786857 | 0,083975363 | |
| 1558201_s_at | SLC4A1AP | 2,87191078 | 2,787940144 | 0,083970636 | |
| 228220_at | FCHO2 | 2,194816625 | 2,110887591 | 0,083929034 | |
| 226583_at | C12orf76 | 2,014487247 | 1,930732138 | 0,083755109 | |
| 229182_at | MIR4723 /// TME | 1,462198073 | 1,378453643 | 0,08374443 | |
| 202556_s_at | MCRS1 | 2,646022818 | 2,562416132 | 0,083606686 | |
| 201594_s_at | PPP4R1 | 5,547270277 | 5,463664675 | 0,083605603 | |
| 223465_at | COL4A3BP | 1,261018186 | 1,177496821 | 0,083521365 | |
| 203445_s_at | CTDSP2 | 3,467741468 | 3,384231608 | 0,08350986 | |
| 202444_s_at | ERLIN1 | 1,732533761 | 1,64909809 | 0,083435671 | |
| 222561_at | LANCL2 | 1,732533761 | 1,64909809 | 0,083435671 | |
| 201444_s_at | ATP6AP2 | 5,277617766 | 5,194214288 | 0,083403478 | |
| 213798_s_at | CAP1 | 6,023694176 | 5,940328216 | 0,083365961 | |
| 223277_at | C3orf75 | 3,236843482 | 3,153484076 | 0,083359407 | |
| 220046_s_at | CCNL1 | 3,868391882 | 3,785040604 | 0,083351278 | |
| 1560457_x_at | PLIN5 | -0,926850548 | -1,010192375 | 0,083341827 | |
| 205438_at | PTPN21 | -0,926850548 | -1,010192375 | 0,083341827 | |
| 206266_at | GPLD1 | -0,926850548 | -1,010192375 | 0,083341827 | |
| 215590_x_at | ACVR2B-AS1 | -0,926850548 | -1,010192375 | 0,083341827 | |
| 220568_at | - | -0,926850548 | -1,010192375 | 0,083341827 | |
| 228957_at | ZNF362 | -0,926850548 | -1,010192375 | 0,083341827 | |
| 237735_at | LOC100506907 | -0,926850548 | -1,010192375 | 0,083341827 | |
| 242497_at | TRAFD1 | -0,926850548 | -1,010192375 | 0,083341827 | |
| 243583_at | - | -0,926850548 | -1,010192375 | 0,083341827 | |
| 217010_s_at | CDC25C | -0,112808516 | -0,19610998 | 0,083301464 | |
| 220089_at | L2HGDH | -0,112808516 | -0,19610998 | 0,083301464 | |
| 230386_at | - | -0,112808516 | -0,19610998 | 0,083301464 | |
| 237892_at | - | -0,112808516 | -0,19610998 | 0,083301464 | |
| 243763_x_at | - | -0,112808516 | -0,19610998 | 0,083301464 | |
| 1566079_at | RPS16P5 | 0,888485433 | 0,805216064 | 0,083269369 | |
| 220052_s_at | TINF2 | 0,888485433 | 0,805216064 | 0,083269369 | |
| 212348_s_at | KDM1A | 3,815015946 | 3,731768909 | 0,083247038 | |
| 225849_s_at | SFT2D1 | 5,557377128 | 5,474197485 | 0,083179643 | |
| 210290_at | ZNF174 | 0,376672223 | 0,293518836 | 0,083153388 | |
| 236269_at | ZNF628 | 0,376672223 | 0,293518836 | 0,083153388 | |
| 208864_s_at | TXN | 6,443496441 | 6,360373874 | 0,083122567 | |
| 225017_at | CCDC14 | 3,962664437 | 3,879554231 | 0,083110206 | |
| 200708_at | GOT2 | 4,78999665 | 4,706906619 | 0,08309003 | |
| 212757_s_at | CAMK2G | 2,272723257 | 2,189694216 | 0,083029041 | |
| 205687_at | UBFD1 | 3,158127685 | 3,075135559 | 0,082992127 | |
| 217909_s_at | MLX | 2,931053371 | 2,848075314 | 0,082978057 | |
| 1554486_a_at | GFOD1 | 3,000043914 | 2,917077481 | 0,082966434 | |
| 203265_s_at | MAP2K4 | 1,481627361 | 1,398700978 | 0,082926383 | |
| 234032_at | - | 1,481627361 | 1,398700978 | 0,082926383 | |

| | | | | | |
|---|---|---|---|---|---|
| 202329_at | CSK | 3,598072957 | 3,515187047 | 0,08288591 | |
| 222858_s_at | DAPP1 | 0,57782909 | 0,495031851 | 0,08279724 | |
| 213367_at | LOC155060 | 0,900083947 | 0,817300337 | 0,08278361 | |
| 244774_at | PHACTR2 | 0,900083947 | 0,817300337 | 0,08278361 | |
| 221221_s_at | KLHL3 | 1,163336153 | 1,080561626 | 0,082774528 | |
| 208997_s_at | UCP2 | 1,748661581 | 1,665902186 | 0,082759396 | |
| 1557067_s_at | LUC7L | 2,485917452 | 2,403170156 | 0,082747296 | |
| 214086_s_at | PARP2 | 3,461883885 | 3,379145507 | 0,082738378 | |
| 217457_s_at | RAP1GDS1 | 2,938102794 | 2,855418981 | 0,082683814 | |
| 224768_at | IWS1 | 3,903389697 | 3,820743792 | 0,082645905 | |
| 217496_s_at | IDE | 3,105247586 | 3,022611001 | 0,082636585 | |
| 212898_at | TTI1 | 3,363729238 | 3,281121096 | 0,082608142 | |
| 1554053_at | SPTLC1 | 1,283332461 | 1,200746069 | 0,082586391 | |
| 216551_x_at | PLCG1 | 1,283332461 | 1,200746069 | 0,082586391 | |
| 239035_at | MTHFR | 1,283332461 | 1,200746069 | 0,082586391 | |
| 46323_at | CANT1 | 3,32445605 | 3,241873662 | 0,082582388 | |
| 216835_s_at | DOK1 | 2,28383338 | 2,201267805 | 0,082565575 | |
| 233268_s_at | CHURC1 | 4,441801334 | 4,359253484 | 0,08254785 | |
| 213876_x_at | ZRSR2 | 1,976645579 | 1,894118818 | 0,082526761 | |
| 226684_at | ATG2B | 3,010782559 | 2,928263672 | 0,082518887 | |
| 222499_at | LOC100652993 / | 2,491678455 | 2,409170778 | 0,082507677 | |
| 203020_at | RABGAP1L | 3,366867184 | 3,284389368 | 0,082477816 | |
| 228323_at | CASC5 | 3,540875956 | 3,458446747 | 0,082429209 | |
| 200714_x_at | OS9 | 3,717599787 | 3,635238397 | 0,08236139 | |
| 219336_s_at | ASCC1 | 2,396622324 | 2,314300277 | 0,082322046 | |
| 225677_at | BCAP29 | 2,048758794 | 1,966439221 | 0,082319573 | |
| 238005_s_at | SIN3A | 1,982112682 | 1,899812471 | 0,082300211 | |
| 200655_s_at | CALM1 /// CALM2 | 6,349600574 | 6,267365756 | 0,082234818 | |
| 217912_at | DUS1L | 3,399920684 | 3,317734938 | 0,082185746 | |
| 225741_at | THUMPD3 | 2,236584753 | 2,154405725 | 0,082179027 | |
| 230777_s_at | PRDM15 | 2,915422315 | 2,833274877 | 0,082147438 | |
| 204089_x_at | MAP3K4 | 3,117719377 | 3,035599287 | 0,082120089 | |
| 1555364_at | C11orf58 | -3,15396682 | -3,236070201 | 0,082103381 | |
| 1560800_at | - | -3,15396682 | -3,236070201 | 0,082103381 | |
| 1565915_at | - | -3,15396682 | -3,236070201 | 0,082103381 | |
| 216697_at | TRIO | -3,15396682 | -3,236070201 | 0,082103381 | |
| 217505_at | KLHL23 /// PHOS | -3,15396682 | -3,236070201 | 0,082103381 | |
| 229057_at | SCN2A | -3,15396682 | -3,236070201 | 0,082103381 | |
| 241098_at | CLEC7A | -3,15396682 | -3,236070201 | 0,082103381 | |
| 243390_at | - | -3,15396682 | -3,236070201 | 0,082103381 | |
| 235837_at | SNIP1 | 0,917308734 | 0,835239052 | 0,082069682 | |
| 226049_at | ERC1 | 1,767779954 | 1,685812099 | 0,081967854 | |
| 216194_s_at | TBCB | 5,752744279 | 5,670779353 | 0,081964926 | |
| 204747_at | IFIT3 | 3,866915053 | 3,785040604 | 0,081874449 | |
| 206539_s_at | CYP4F12 | -0,078265071 | -0,160136748 | 0,081871677 | |
| 208851_s_at | THY1 | -0,078265071 | -0,160136748 | 0,081871677 | |
| 215649_s_at | MVK | -0,078265071 | -0,160136748 | 0,081871677 | |
| 228845_at | PLBD2 | -0,078265071 | -0,160136748 | 0,081871677 | |
| 244145_at | - | -0,078265071 | -0,160136748 | 0,081871677 | |
| 218553_s_at | KCTD15 | 2,185368281 | 2,103505212 | 0,081863068 | |
| 222839_s_at | PAPOLG | 2,55541096 | 2,473581079 | 0,081829881 | |
| 209210_s_at | FERMT2 | 4,69955433 | 4,617737065 | 0,081817265 | |
| 202019_s_at | LANCL1 | 1,689737652 | 1,60795124 | 0,081786412 | |
| 202433_at | SLC35B1 | 3,711030028 | 3,629253073 | 0,081776955 | |
| 202216_x_at | NFYC | 2,127340679 | 2,045615873 | 0,081724806 | |
| 244881_at | LMLN | 1,305306849 | 1,223626587 | 0,081680262 | |

| | | | | | |
|---|---|---|---|---|---|
| 235926_at | ANAPC5 | 2,64946283 | 2,567790814 | 0,081672016 | |
| 244803_at | - | 0,928678725 | 0,847075464 | 0,081603261 | |
| 47083_at | C7orf26 | 3,207388477 | 3,125814827 | 0,081573649 | |
| 208107_s_at | LOC81691 | 2,779171778 | 2,697670403 | 0,081501375 | |
| 226335_at | RPS6KA3 | 4,480461153 | 4,398994417 | 0,081466736 | |
| 229267_at | ANAPC1 /// LOC1 | 3,133773173 | 3,052311115 | 0,081462058 | |
| 1553105_s_at | DSG2 | 0,417596971 | 0,336136178 | 0,081460793 | |
| 205209_at | ACVR1B | 0,417596971 | 0,336136178 | 0,081460793 | |
| 211087_x_at | MAPK14 | 0,417596971 | 0,336136178 | 0,081460793 | |
| 208737_at | ATP6V1G1 | 5,468393796 | 5,386939953 | 0,081453844 | |
| 219297_at | WDR44 | 3,565667132 | 3,484256382 | 0,08141075 | |
| 226120_at | TTC8 | 3,821129803 | 3,739727424 | 0,081402379 | |
| 1560694_at | SFT2D1 | 0,613492853 | 0,532164371 | 0,081328483 | |
| 220000_at | SIGLEC5 | 0,613492853 | 0,532164371 | 0,081328483 | |
| 222650_s_at | SLC2A4RG | 0,613492853 | 0,532164371 | 0,081328483 | |
| 234254_at | - | 0,613492853 | 0,532164371 | 0,081328483 | |
| 241993_x_at | - | 1,783520648 | 1,702196344 | 0,081324304 | |
| 1557762_at | - | -3,192553003 | -3,273861381 | 0,081308379 | |
| 1561939_at | DYNC2H1 | -3,192553003 | -3,273861381 | 0,081308379 | |
| 1565563_at | - | -3,192553003 | -3,273861381 | 0,081308379 | |
| 207276_at | CDR1 | -3,192553003 | -3,273861381 | 0,081308379 | |
| 207750_at | - | -3,192553003 | -3,273861381 | 0,081308379 | |
| 215514_at | - | -3,192553003 | -3,273861381 | 0,081308379 | |
| 204269_at | PIM2 | 2,701760055 | 2,620464265 | 0,08129579 | |
| 212418_at | ELF1 | 4,794664596 | 4,713387424 | 0,081277172 | |
| 217941_s_at | ERBB2IP | 4,091545995 | 4,010311743 | 0,081234252 | |
| 202829_s_at | VAMP7 | 4,649324867 | 4,56809424 | 0,081230627 | |
| 208771_s_at | LTA4H | 4,522808608 | 4,441578636 | 0,081229972 | |
| 225551_at | CNST | 1,523472489 | 1,442269454 | 0,081203035 | |
| 229558_at | C16orf88 | 1,523472489 | 1,442269454 | 0,081203035 | |
| 243492_at | THEM4 | 1,523472489 | 1,442269454 | 0,081203035 | |
| 209782_s_at | DBP | 1,318332599 | 1,23718272 | 0,081149879 | |
| 200074_s_at | RPL14 | 6,976903525 | 6,895812729 | 0,081090796 | |
| 219312_s_at | ZBTB10 | 1,789769151 | 1,708698295 | 0,081070856 | |
| 227858_at | PCNXL3 | 1,789769151 | 1,708698295 | 0,081070856 | |
| 221699_s_at | DDX50 | 4,278110569 | 4,197041937 | 0,081068633 | |
| 34449_at | CASP2 | 1,97938172 | 1,898391163 | 0,080990558 | |
| 1566775_at | DNAH1 | -0,055687482 | -0,136643983 | 0,080956501 | |
| 205589_at | MYL3 | -0,055687482 | -0,136643983 | 0,080956501 | |
| 216646_at | DSCC1 | -0,055687482 | -0,136643983 | 0,080956501 | |
| 224069_x_at | P2RX2 | -0,055687482 | -0,136643983 | 0,080956501 | |
| 231306_at | LYZL4 | -0,055687482 | -0,136643983 | 0,080956501 | |
| 231372_at | SLC25A48 | -0,055687482 | -0,136643983 | 0,080956501 | |
| 244795_at | UQCC | -0,055687482 | -0,136643983 | 0,080956501 | |
| 228167_at | KLHL6 | 2,208874124 | 2,127967638 | 0,080906486 | |
| 218106_s_at | MRPS10 | 4,282548314 | 4,201658983 | 0,080889331 | |
| 1554937_x_at | EXOC3L1 | 0,209623097 | 0,128747141 | 0,080875957 | |
| 229443_at | MNF1 | 0,209623097 | 0,128747141 | 0,080875957 | |
| 230146_s_at | NCS1 | 0,209623097 | 0,128747141 | 0,080875957 | |
| 218199_s_at | NOL6 | 1,62481334 | 1,543943227 | 0,080870113 | |
| 227805_at | METAP1D | 1,62481334 | 1,543943227 | 0,080870113 | |
| 229846_s_at | MAPKAP1 | 2,794789337 | 2,713921281 | 0,080868056 | |
| 205004_at | NKRF | 3,148435161 | 3,067567462 | 0,080867699 | |
| 1559226_x_at | LCE1E | 1,210676994 | 1,129843209 | 0,080833785 | |
| 225560_at | POMT2 | 1,326951551 | 1,246149888 | 0,080801662 | |
| 204172_at | CPOX | 4,771961618 | 4,691191826 | 0,080769791 | |

| | | | | | |
|---|---|---|---|---|---|
| 208925_at | CLDND1 | 3,27186005 | 3,191118448 | 0,080741602 | |
| 219079_at | CYB5R4 | 3,27186005 | 3,191118448 | 0,080741602 | |
| 203042_at | LAMP2 | 3,48613512 | 3,405399563 | 0,080735557 | |
| 221547_at | PRPF18 | 3,120200849 | 3,039473081 | 0,080727768 | |
| 222512_at | NUB1 | 3,152077493 | 3,071356473 | 0,08072102 | |
| 204521_at | FAM216A | 4,301804532 | 4,221118236 | 0,080686296 | |
| 219342_at | CASD1 | 2,630440446 | 2,549796788 | 0,080643658 | |
| 210980_s_at | ASAH1 | 4,718102071 | 4,637466396 | 0,080635675 | |
| 201799_s_at | OSBP | 2,879254447 | 2,798646381 | 0,080608066 | |
| 202451_at | GTF2H1 | 3,801937331 | 3,721356767 | 0,080580564 | |
| 226177_at | GLTP | 3,540875956 | 3,460374487 | 0,080501469 | |
| 1554167_a_at | GOLGA7 | 4,764049426 | 4,683579237 | 0,080470189 | |
| 206829_x_at | ZNF430 | 2,493593689 | 2,413157372 | 0,080436317 | |
| 233849_s_at | ARHGAP5 | 3,159334685 | 3,078904771 | 0,080429914 | |
| 1553815_a_at | TCEANC | -0,83191556 | -0,912331589 | 0,080416029 | |
| 1558299_at | - | -0,83191556 | -0,912331589 | 0,080416029 | |
| 209270_at | LAMB3 | -0,83191556 | -0,912331589 | 0,080416029 | |
| 213259_s_at | SARM1 | -0,83191556 | -0,912331589 | 0,080416029 | |
| 224323_s_at | MRO | -0,83191556 | -0,912331589 | 0,080416029 | |
| 225013_at | LZTS2 | -0,83191556 | -0,912331589 | 0,080416029 | |
| 234076_at | - | -0,83191556 | -0,912331589 | 0,080416029 | |
| 234225_at | - | -0,83191556 | -0,912331589 | 0,080416029 | |
| 239109_at | - | -0,83191556 | -0,912331589 | 0,080416029 | |
| 212717_at | PLEKHM1 | 2,544366685 | 2,464004877 | 0,080361808 | |
| 211536_x_at | MAP3K7 | 3,161745659 | 3,081412119 | 0,08033354 | |
| 49111_at | ARRB1 | 1,448440187 | 1,36822241 | 0,080217777 | |
| 213668_s_at | SOX4 | 1,64218396 | 1,562009954 | 0,080174006 | |
| 203374_s_at | TPP2 | 3,771477743 | 3,691304166 | 0,080173577 | |
| 200790_at | ODC1 | 6,689947949 | 6,609799427 | 0,080148522 | |
| 226747_at | TXNDC16 | 4,955812888 | 4,875668581 | 0,080144307 | |
| 209377_s_at | HMGN3 | 6,728271603 | 6,648148518 | 0,080123085 | |
| 212861_at | MFSD5 | 2,503131904 | 2,423075926 | 0,080055978 | |
| 225267_at | KPNA4 | 4,169090081 | 4,08904909 | 0,08004099 | |
| 33646_g_at | GM2A | 1,855284924 | 1,775255987 | 0,080028937 | |
| 201047_x_at | RAB6A | 4,875469339 | 4,795472925 | 0,079996413 | |
| 202560_s_at | CHTOP | 4,415271934 | 4,335292864 | 0,079979071 | |
| 217408_at | MRPS18B | 4,755694657 | 4,675718863 | 0,079975793 | |
| 209784_s_at | JAG2 | 0,648296226 | 0,568365095 | 0,07993113 | |
| 208837_at | TMED3 | 4,124054951 | 4,044124824 | 0,079930127 | |
| 235940_at | C9orf64 | 3,692807389 | 3,6128809 | 0,07992649 | |
| 201896_s_at | PSRC1 | 1,233777334 | 1,153866761 | 0,079910572 | |
| 200097_s_at | HNRNPK /// MIR | 5,456414609 | 5,376513741 | 0,079900869 | |
| 204467_s_at | SNCA | 2,456762221 | 2,376875125 | 0,079887096 | |
| 219549_s_at | RTN3 | 4,234684448 | 4,154809816 | 0,079874632 | |
| 204270_at | SKI | 0,457392764 | 0,377530647 | 0,079862118 | |
| 1553510_s_at | RQCD1 | 3,294008537 | 3,21415095 | 0,079857587 | |
| 228822_s_at | USP16 | 3,917011805 | 3,837157105 | 0,0798547 | |
| 211530_x_at | HLA-G | 4,779830653 | 4,699988715 | 0,079841938 | |
| 214756_x_at | PMS2P1 | 3,008105382 | 2,928263672 | 0,079841711 | |
| 205017_s_at | MBNL2 | 0,97328325 | 0,893473268 | 0,079809982 | |
| 1556646_at | - | 1,973904239 | 1,894118818 | 0,079785421 | |
| 202649_x_at | RPS19 | 7,834905002 | 7,755140714 | 0,079764289 | |
| 235061_at | PPM1K | 1,823660386 | 1,743944487 | 0,079715899 | |
| 225187_at | KIAA1967 | 3,046446467 | 2,966746113 | 0,079700354 | |
| 227598_at | C7orf29 | 3,046446467 | 2,966746113 | 0,079700354 | |
| 205877_s_at | ZC3H7B | 2,178241192 | 2,098562556 | 0,079678635 | |

| | | | | | |
|---|---|---|---|---|---|
| 201195_s_at | SLC7A5 | 4,082030648 | 4,002394882 | 0,079635766 | |
| 220977_x_at | EPB41L5 | -0,022466681 | -0,102100538 | 0,079633857 | |
| 222339_x_at | - | -0,022466681 | -0,102100538 | 0,079633857 | |
| 222655_s_at | IMPAD1 | -0,022466681 | -0,102100538 | 0,079633857 | |
| 224342_x_at | LOC96610 | -0,022466681 | -0,102100538 | 0,079633857 | |
| 243615_at | - | -0,022466681 | -0,102100538 | 0,079633857 | |
| 212640_at | PTPLB | 3,681090166 | 3,601570866 | 0,0795193 | |
| 215926_x_at | SNAPC4 | 2,35929703 | 2,27978304 | 0,07951399 | |
| 213477_x_at | EEF1A1 /// LOC1 | 8,21190393 | 8,132404793 | 0,079499137 | |
| 229189_s_at | LOC100507376 | 1,119359127 | 1,039887232 | 0,079471896 | |
| 202074_s_at | OPTN | 1,360921491 | 1,281471383 | 0,079450108 | |
| 228880_at | NAT8L | 1,567778694 | 1,488344553 | 0,079434141 | |
| 1568718_at | SLC22A23 | 0,984222186 | 0,904843258 | 0,079378927 | |
| 212086_x_at | LMNA | 3,021441862 | 2,942125533 | 0,079316328 | |
| 219924_s_at | ZMYM6 | 2,522021211 | 2,442710777 | 0,079310434 | |
| 226466_s_at | FAM58A /// FAM5 | 2,752227654 | 2,672945617 | 0,079282036 | |
| 204031_s_at | PCBP2 | 5,71034645 | 5,631110922 | 0,079235528 | |
| 214080_x_at | PRKCSH | 3,755604375 | 3,67645448 | 0,079149895 | |
| 203062_s_at | MDC1 | 3,50144467 | 3,42231092 | 0,07913375 | |
| 201214_s_at | PPP1R7 | 3,575646363 | 3,496520471 | 0,079125892 | |
| 201686_x_at | API5 | 2,369762403 | 2,290658654 | 0,079103748 | |
| 1553943_at | NS3BP | 0,255492549 | 0,176436073 | 0,079056476 | |
| 205026_at | STAT5B | 0,255492549 | 0,176436073 | 0,079056476 | |
| 205326_at | RAMP3 | 0,255492549 | 0,176436073 | 0,079056476 | |
| 210144_at | TBC1D22A | 0,255492549 | 0,176436073 | 0,079056476 | |
| 214367_at | RASGRP2 | 0,255492549 | 0,176436073 | 0,079056476 | |
| 215153_at | NOS1AP | 0,255492549 | 0,176436073 | 0,079056476 | |
| 212162_at | KIDINS220 | 2,064305961 | 1,98530622 | 0,078999741 | |
| 204313_s_at | CREB1 | 2,760204767 | 2,681234383 | 0,078970383 | |
| 238650_x_at | WDR89 | 2,31665944 | 2,237691445 | 0,078967994 | |
| 209214_s_at | EWSR1 | 4,519052534 | 4,440114107 | 0,078938427 | |
| 205162_at | ERCC8 | 2,482063962 | 2,403170156 | 0,078893806 | |
| 1557965_at | MTERFD2 | 2,721505869 | 2,64270545 | 0,078800419 | |
| 225725_at | ZMAT3 | 2,320980353 | 2,242180416 | 0,078799937 | |
| 209711_at | SLC35D1 | 1,764611101 | 1,685812099 | 0,078799001 | |
| 218542_at | CEP55 | 3,069741844 | 2,990953566 | 0,078788278 | |
| 207582_at | PIN1P1 | -0,00073712 | -0,079522948 | 0,078785828 | |
| 209122_at | LOC100509484 / | 5,070280549 | 4,991513183 | 0,078767367 | |
| 213726_x_at | TUBB4B | 5,869070493 | 5,790352889 | 0,078717604 | |
| 211475_s_at | BAG1 | 3,512821151 | 3,434130109 | 0,078691042 | |
| 221226_s_at | ASIC4 | 1,381753566 | 1,303116084 | 0,078637482 | |
| 1555181_a_at | ST3GAL3 | 2,382221458 | 2,303602088 | 0,07861937 | |
| 200627_at | LOC100506732 / | 6,795992082 | 6,717390829 | 0,078601253 | |
| 1556320_at | STOML1 | 0,682279745 | 0,603679647 | 0,078600099 | |
| 210001_s_at | SOCS1 | 0,682279745 | 0,603679647 | 0,078600099 | |
| 213238_at | ATP10D | 0,682279745 | 0,603679647 | 0,078600099 | |
| 203537_at | PRPSAP2 | 3,852809256 | 3,77423254 | 0,078576716 | |
| 222414_at | MLL3 | 2,206540699 | 2,127967638 | 0,078573061 | |
| 211755_s_at | ATP5F1 | 6,882856958 | 6,804291857 | 0,078565101 | |
| 218099_at | TEX2 | 2,854131948 | 2,775606361 | 0,078525588 | |
| 215948_x_at | ZMYM5 | 1,143956113 | 1,065442793 | 0,07851332 | |
| 206067_s_at | WT1 | 3,517535002 | 3,439026336 | 0,078508666 | |
| 213941_x_at | RPS7 | 7,725433715 | 7,646997217 | 0,078436498 | |
| 240114_s_at | TMEM174 | 1,079110292 | 1,000684521 | 0,078425771 | |
| 222113_s_at | EPS15L1 | 2,331726354 | 2,253342112 | 0,078384242 | |
| 207196_s_at | TNIP1 | 2,59519831 | 2,516830926 | 0,078367384 | |

| | | | | | |
|---|---|---|---|---|---|
| 239729_at | - | 0,496120222 | 0,417770464 | 0,078349758 | |
| 228449_at | MORC2-AS1 | 1,011212064 | 0,932882274 | 0,07832979 | |
| 234241_at | SCAMP5 | 1,011212064 | 0,932882274 | 0,07832979 | |
| 238609_at | FAM200A | 1,011212064 | 0,932882274 | 0,07832979 | |
| 208961_s_at | KLF6 | 1,596577845 | 1,518263577 | 0,078314268 | |
| 1555382_at | POF1B | -3,32387526 | -3,402185466 | 0,078310206 | |
| 1557079_at | ITGBL1 | -3,32387526 | -3,402185466 | 0,078310206 | |
| 233875_at | - | -3,32387526 | -3,402185466 | 0,078310206 | |
| 203665_at | HMOX1 | 1,859746653 | 1,781437557 | 0,078309096 | |
| 1555743_s_at | ERVH-6 | 2,497416546 | 2,419116683 | 0,078299862 | |
| 205063_at | GEMIN2 | 3,548266005 | 3,469974737 | 0,078291269 | |
| 209256_s_at | KLHDC10 | 2,861566439 | 2,783327323 | 0,078239116 | |
| 207700_s_at | NCOA3 | 2,338135744 | 2,259997914 | 0,07813783 | |
| 200827_at | PLOD1 | 2,90394695 | 2,825817328 | 0,078129621 | |
| 212132_at | LSM14A | 6,043930105 | 5,965827644 | 0,078102461 | |
| 1556329_a_at | - | 1,603688695 | 1,525647381 | 0,078041314 | |
| 219100_at | OBFC1 | 2,156646206 | 2,078620836 | 0,07802537 | |
| 200828_s_at | ZNF207 | 5,938908435 | 5,860917599 | 0,077990837 | |
| 220967_s_at | ZNF696 | 2,022468633 | 1,944570415 | 0,077898218 | |
| 201453_x_at | RHEB | 6,375277638 | 6,297400219 | 0,077877419 | |
| 226277_at | COL4A3BP | 2,745814053 | 2,667949403 | 0,07786465 | |
| 217728_at | S100A6 | 3,316904202 | 3,239069096 | 0,077835106 | |
| 236613_at | RBM25 | 0,702292136 | 0,624460759 | 0,077831377 | |
| 34697_at | LRP6 | -0,690806998 | -0,768633105 | 0,077826107 | |
| 217816_s_at | PCNP | 5,145904033 | 5,068098141 | 0,077805893 | |
| 200717_x_at | RPL7 | 8,150902865 | 8,073169078 | 0,077733787 | |
| 214195_at | TPP1 | 1,027166904 | 0,949447784 | 0,077719121 | |
| 232134_at | - | 1,027166904 | 0,949447784 | 0,077719121 | |
| 235174_s_at | LOC100128822 | 2,876321464 | 2,798646381 | 0,077675084 | |
| 215130_s_at | IQCK | 1,292162421 | 1,214517879 | 0,077644542 | |
| 200937_s_at | RPL5 /// SNORD: | 7,277456502 | 7,199827195 | 0,077629307 | |
| 1560656_at | - | -0,741668563 | -0,819238336 | 0,077569773 | |
| 1570186_at | GRASPOS | -0,741668563 | -0,819238336 | 0,077569773 | |
| 204733_at | KLK6 | -0,741668563 | -0,819238336 | 0,077569773 | |
| 206049_at | SELP | -0,741668563 | -0,819238336 | 0,077569773 | |
| 206487_at | SUN1 | -0,741668563 | -0,819238336 | 0,077569773 | |
| 210982_s_at | HLA-DRA | -0,741668563 | -0,819238336 | 0,077569773 | |
| 215527_at | KHDRBS2 | -0,741668563 | -0,819238336 | 0,077569773 | |
| 220508_at | CCT8L2 | -0,741668563 | -0,819238336 | 0,077569773 | |
| 233939_at | REXO1 | -0,741668563 | -0,819238336 | 0,077569773 | |
| 237447_at | C22orf45 | -0,741668563 | -0,819238336 | 0,077569773 | |
| 243956_at | SUSD3 | -0,741668563 | -0,819238336 | 0,077569773 | |
| 244085_at | ZNF653 | -0,741668563 | -0,819238336 | 0,077569773 | |
| 244516_at | - | -0,741668563 | -0,819238336 | 0,077569773 | |
| 222110_at | SENP5 | 0,03125738 | -0,046302147 | 0,077559527 | |
| 226978_at | PPARA | 0,03125738 | -0,046302147 | 0,077559527 | |
| 244561_at | SLC9A1 | 0,03125738 | -0,046302147 | 0,077559527 | |
| 224474_x_at | SMEK2 | 2,102456302 | 2,024923328 | 0,077532974 | |
| 225030_at | BOD1 | 2,840652551 | 2,763166224 | 0,077486326 | |
| 220350_at | ZNF235 | 0,882651021 | 0,805216064 | 0,077434957 | |
| 209759_s_at | ECI1 | 2,520143371 | 2,442710777 | 0,077432594 | |
| 201810_s_at | SH3BP5 | 1,414471577 | 1,337086025 | 0,077385552 | |
| 206507_at | ZSCAN12 | 1,414471577 | 1,337086025 | 0,077385552 | |
| 212585_at | OSBPL8 | 4,769592513 | 4,692217481 | 0,077375033 | |
| 1555216_a_at | LOC645722 | 1,621313976 | 1,543943227 | 0,077370749 | |
| 203098_at | CDYL | 3,884538004 | 3,807174423 | 0,077363581 | |

| | | | | | |
|---|---|---|---|---|---|
| 218403_at | TRIAP1 | 4,714822074 | 4,637466396 | 0,077355679 | |
| 219720_s_at | C14orf118 | 0,715481152 | 0,638150376 | 0,077330775 | |
| 228356_at | ANKRD11 | 0,715481152 | 0,638150376 | 0,077330775 | |
| 239376_at | - | 0,715481152 | 0,638150376 | 0,077330775 | |
| 209325_s_at | RGS16 | 1,037706275 | 0,960386719 | 0,077319556 | |
| 221010_s_at | SIRT5 | 1,037706275 | 0,960386719 | 0,077319556 | |
| 226439_s_at | NBEA | 1,037706275 | 0,960386719 | 0,077319556 | |
| 213881_x_at | SUMO2 | 7,001339637 | 6,924026094 | 0,077313542 | |
| 200091_s_at | RPS25 | 7,282034778 | 7,204722872 | 0,077311906 | |
| 202011_at | TJP1 | 4,242108456 | 4,164893351 | 0,077215106 | |
| 212284_x_at | TPT1 | 8,106030794 | 8,028836191 | 0,077194603 | |
| 209394_at | ASMTL | 1,889144008 | 1,811954245 | 0,077189763 | |
| 203848_at | AKAP8 | 2,626954721 | 2,549796788 | 0,077157933 | |
| 56197_at | C17orf61-PLSCR | 3,435226406 | 3,358102979 | 0,077123427 | |
| 218147_s_at | GLT8D1 | 3,046446467 | 2,969455976 | 0,076990491 | |
| 222668_at | KCTD15 | 4,010933848 | 3,933981372 | 0,076952476 | |
| 203557_s_at | PCBD1 | 0,533835206 | 0,456918297 | 0,076916909 | |
| 211112_at | SLC12A4 | 0,533835206 | 0,456918297 | 0,076916909 | |
| 225325_at | MFSD6 | 0,533835206 | 0,456918297 | 0,076916909 | |
| 226396_at | TEN1 /// TEN1-C | 0,533835206 | 0,456918297 | 0,076916909 | |
| 212260_at | GIGYF2 | 1,897847661 | 1,820984782 | 0,076862879 | |
| 208158_s_at | OSBPL1A | 1,314003737 | 1,23718272 | 0,076821017 | |
| 222057_at | NOL12 | 0,052199481 | -0,024572586 | 0,076772067 | |
| 230590_at | - | 0,052199481 | -0,024572586 | 0,076772067 | |
| 235431_s_at | PELI3 | 0,052199481 | -0,024572586 | 0,076772067 | |
| 243272_at | - | 0,052199481 | -0,024572586 | 0,076772067 | |
| 204278_s_at | EBAG9 | 3,563845287 | 3,487095824 | 0,076749463 | |
| 227413_at | UBLCP1 | 3,223057183 | 3,146317015 | 0,076740169 | |
| 238026_at | RPL35A | 1,191926657 | 1,115234684 | 0,076691972 | |
| 208617_s_at | PTP4A2 | 5,892666962 | 5,815986205 | 0,076680757 | |
| 218802_at | CCDC109B | 2,732900676 | 2,656223838 | 0,076676837 | |
| 204769_s_at | TAP2 | 1,820612054 | 1,743944487 | 0,076667566 | |
| 222017_x_at | LRCH4 | 0,735041319 | 0,658444279 | 0,07659704 | |
| 207079_s_at | MED6 | 4,569406661 | 4,492864546 | 0,076542116 | |
| 1555797_a_at | ARPC5 | 4,00557508 | 3,92911743 | 0,07645765 | |
| 220305_at | MAVS | 2,596981013 | 2,520531219 | 0,076449794 | |
| 209432_s_at | CREB3 | 2,693452197 | 2,617011906 | 0,076440291 | |
| 212923_s_at | PXDC1 | 0,911589958 | 0,835239052 | 0,076350906 | |
| 242141_at | HDAC2 | 0,911589958 | 0,835239052 | 0,076350906 | |
| 235997_at | ZNF74 | 1,063722698 | 0,987376598 | 0,0763461 | |
| 225230_at | DRAM2 | 2,266015882 | 2,189694216 | 0,076321666 | |
| 226740_x_at | LOC100506032 / | 5,454818035 | 5,378554049 | 0,076263986 | |
| 209795_at | CD69 | 4,102254727 | 4,026016313 | 0,076238414 | |
| 224572_s_at | IRF2BP2 | 2,790121796 | 2,713921281 | 0,076200515 | |
| 213534_s_at | PASK | 1,446464021 | 1,370274469 | 0,076189551 | |
| 237209_s_at | NFRKB | 0,747935635 | 0,67181667 | 0,076118965 | |
| 202170_s_at | AASDHPPT | 3,877221251 | 3,801102381 | 0,07611887 | |
| 201165_s_at | PUM1 | 4,223764788 | 4,147649355 | 0,076115433 | |
| 220189_s_at | MGAT4B | 3,24026957 | 3,164168347 | 0,076101223 | |
| 1552535_at | CLDN19 | 0,555999837 | 0,479907041 | 0,076092796 | |
| 223006_s_at | TMEM245 | 3,606946883 | 3,530865925 | 0,076080958 | |
| 215728_s_at | ACOT7 | 1,655931423 | 1,579853228 | 0,076078195 | |
| 212199_at | MRFAP1L1 | 4,160677048 | 4,084681583 | 0,075995465 | |
| 223007_s_at | TMEM245 | 3,425226911 | 3,349287209 | 0,075939703 | |
| 223394_at | SERTAD1 | 3,076146581 | 3,000258961 | 0,075887621 | |
| 218156_s_at | TSR1 | 4,076291169 | 4,00040886 | 0,075882309 | |

| | | | | | |
|---|---|---|---|---|---|
| 211998_at | H3F3A /// H3F3B | 2,146943689 | 2,071071048 | 0,075872641 | |
| 222907_x_at | TMEM50B | 2,614687931 | 2,538891651 | 0,07579628 | |
| 224934_at | YIPF5 | 4,387215694 | 4,311461649 | 0,075754045 | |
| 214683_s_at | CLK1 | 2,802535172 | 2,726791458 | 0,075743714 | |
| 207419_s_at | RAC2 | 3,251631457 | 3,175947691 | 0,075683766 | |
| 201128_s_at | ACLY | 5,5868312 | 5,511165886 | 0,075665314 | |
| 225045_at | CCDC88A | 2,082235068 | 2,006570641 | 0,075664427 | |
| 206737_at | WNT11 | 0,083054335 | 0,007421914 | 0,075632421 | |
| 226694_at | AKAP2 /// PALM2 | 0,083054335 | 0,007421914 | 0,075632421 | |
| 227168_at | MIAT | 0,083054335 | 0,007421914 | 0,075632421 | |
| 230234_at | FXN | 0,083054335 | 0,007421914 | 0,075632421 | |
| 225010_at | CCDC6 | 3,805796305 | 3,730171924 | 0,075624382 | |
| 225783_at | UBE2F | 3,91129185 | 3,835672678 | 0,075619172 | |
| 231892_at | C9orf100 | 0,57058931 | 0,495031851 | 0,07555746 | |
| 220311_at | N6AMT1 | 1,348276312 | 1,272721751 | 0,075554561 | |
| 223142_s_at | UCK1 | 1,348276312 | 1,272721751 | 0,075554561 | |
| 228046_at | ZNF827 | 1,348276312 | 1,272721751 | 0,075554561 | |
| 209234_at | KIF1B | 2,288253583 | 2,212749286 | 0,075504296 | |
| 211759_x_at | TBCB | 6,250723505 | 6,17529995 | 0,075423555 | |
| 223684_s_at | SMUG1 | 2,410880861 | 2,335461563 | 0,075419297 | |
| 217346_at | - | 1,089278259 | 1,013870809 | 0,07540745 | |
| 220200_s_at | SETD8 | 1,089278259 | 1,013870809 | 0,07540745 | |
| 224883_at | PLDN | 2,896728217 | 2,821324225 | 0,075403992 | |
| 200969_at | SERP1 | 4,650183672 | 4,574783153 | 0,075400519 | |
| 222931_s_at | THNSL1 | 2,675004741 | 2,599625088 | 0,075379654 | |
| 212955_s_at | POLR2I | 5,710758133 | 5,635383188 | 0,075374944 | |
| 200728_at | ACTR2 | 5,429404826 | 5,35413705 | 0,075267776 | |
| 222789_at | RSBN1 | 2,678376417 | 2,603119255 | 0,075257162 | |
| 209344_at | TPM4 | 2,726400382 | 2,651169275 | 0,075231107 | |
| 1555656_at | CD300LG | 2,094906514 | 2,019703483 | 0,075203031 | |
| 205658_s_at | SNAPC4 | 1,473887017 | 1,398700978 | 0,075186039 | |
| 209475_at | USP15 | 3,021441862 | 2,946258252 | 0,075183609 | |
| 217786_at | PRMT5 | 5,131216168 | 5,056037987 | 0,075178182 | |
| 233750_s_at | TRMT1L | 2,166283906 | 2,091116676 | 0,07516723 | |
| 220258_s_at | WRAP53 | 1,683039483 | 1,60795124 | 0,075088243 | |
| 336_at | TBXA2R | -0,430183988 | -0,505249144 | 0,075065156 | |
| 1556395_at | - | 6,195242402 | 6,120275227 | 0,074967175 | |
| 1557814_a_at | - | 0,779676066 | 0,704715219 | 0,074960847 | |
| 236241_at | MED31 | 0,779676066 | 0,704715219 | 0,074960847 | |
| 236721_at | ALKBH1 | 0,779676066 | 0,704715219 | 0,074960847 | |
| 204892_x_at | EEF1A1 /// LOC1 | 8,441689547 | 8,36673719 | 0,074952356 | |
| 218443_s_at | DAZAP1 | 3,480351894 | 3,405399563 | 0,07495233 | |
| 213359_at | HNRNPD | 2,173470163 | 2,098562556 | 0,074907607 | |
| 202756_s_at | GPC1 | 0,103263601 | 0,028364014 | 0,074899587 | |
| 207184_at | SLC6A13 | 0,103263601 | 0,028364014 | 0,074899587 | |
| 210499_s_at | PQBP1 | 0,103263601 | 0,028364014 | 0,074899587 | |
| 213641_at | ZNF500 | 0,103263601 | 0,028364014 | 0,074899587 | |
| 240897_at | - | 0,103263601 | 0,028364014 | 0,074899587 | |
| 234797_at | - | 1,104397092 | 1,029536893 | 0,074860199 | |
| 1553592_x_at | BCRP3 | -0,655928373 | -0,730778808 | 0,074850435 | |
| 1554367_at | CALHM1 | -0,655928373 | -0,730778808 | 0,074850435 | |
| 1554487_a_at | ATF6B | -0,655928373 | -0,730778808 | 0,074850435 | |
| 1558938_at | - | -0,655928373 | -0,730778808 | 0,074850435 | |
| 1569386_at | - | -0,655928373 | -0,730778808 | 0,074850435 | |
| 205487_s_at | VGLL1 | -0,655928373 | -0,730778808 | 0,074850435 | |
| 208003_s_at | NFAT5 | -0,655928373 | -0,730778808 | 0,074850435 | |

| | | | | | |
|---|---|---|---|---|---|
| 215484_at | - | -0,655928373 | -0,730778808 | 0,074850435 | |
| 222238_s_at | POLM | -0,655928373 | -0,730778808 | 0,074850435 | |
| 222711_s_at | RHBDF1 | -0,655928373 | -0,730778808 | 0,074850435 | |
| 237096_at | - | -0,655928373 | -0,730778808 | 0,074850435 | |
| 222902_s_at | DEM1 | 1,689737652 | 1,614891062 | 0,07484659 | |
| 203073_at | COG2 | 2,912562019 | 2,837730973 | 0,074831046 | |
| 209115_at | UBA3 | 3,891817836 | 3,816987307 | 0,074830529 | |
| 204728_s_at | WDHD1 | 2,59162628 | 2,516830926 | 0,074795355 | |
| 226017_at | CMTM7 | 3,142344098 | 3,067567462 | 0,074776636 | |
| 225592_at | NRM | 2,691784869 | 2,617011906 | 0,074772963 | |
| 228928_x_at | BANP | 3,072307152 | 2,997606395 | 0,074700757 | |
| 223027_at | SNX9 | 3,146001821 | 3,071356473 | 0,074645348 | |
| 213583_x_at | EEF1A1 /// LOC1 | 8,244967699 | 8,170383416 | 0,074584284 | |
| 201426_s_at | VIM | 7,213675956 | 7,139143803 | 0,074532153 | |
| 37950_at | PREP | 2,723955201 | 2,649480478 | 0,074474723 | |
| 200670_at | XBP1 | 3,002736078 | 2,928263672 | 0,074472407 | |
| 209388_at | PAPOLA | 4,877670934 | 4,803278451 | 0,074392483 | |
| 202243_s_at | PSMB4 | 6,134169759 | 6,059813854 | 0,074355906 | |
| 220746_s_at | UIMC1 | 2,382221458 | 2,307890888 | 0,074330569 | |
| 242082_at | MMAB | 0,798390451 | 0,724100169 | 0,074290282 | |
| 215778_x_at | HAB1 | 0,606430281 | 0,532164371 | 0,074265911 | |
| 226616_s_at | NDUFV3 | 5,450757238 | 5,376513741 | 0,074243498 | |
| 1552766_at | HS6ST2 | 0,384950573 | 0,310717132 | 0,074233441 | |
| 206956_at | BGLAP /// PMF1- | 0,384950573 | 0,310717132 | 0,074233441 | |
| 209588_at | EPHB2 | 0,384950573 | 0,310717132 | 0,074233441 | |
| 212965_at | HIC2 | 0,384950573 | 0,310717132 | 0,074233441 | |
| 242657_at | - | 0,384950573 | 0,310717132 | 0,074233441 | |
| 242697_at | ZNF540 | 0,384950573 | 0,310717132 | 0,074233441 | |
| 226006_at | PET100 | 4,671066265 | 4,59685817 | 0,074208095 | |
| 201832_s_at | USO1 | 4,416281951 | 4,34210478 | 0,074177171 | |
| 205872_x_at | PDE4DIP | 2,609398637 | 2,53523821 | 0,074160427 | |
| 228987_at | FAM49B | 2,446911392 | 2,372786857 | 0,074124535 | |
| 212569_at | SMCHD1 | 3,388643755 | 3,314541423 | 0,074102332 | |
| 211252_x_at | PTCRA | 1,504602299 | 1,43051703 | 0,074085269 | |
| 214651_s_at | HOXA10-HOXA9 | 1,390002893 | 1,315948728 | 0,074054165 | |
| 219402_s_at | DERL1 | 4,945355432 | 4,871328311 | 0,074027121 | |
| 221931_s_at | SEH1L | 5,520750144 | 5,446747817 | 0,074002327 | |
| 209585_s_at | MINPP1 | 5,191452978 | 5,117494652 | 0,073958326 | |
| 212409_s_at | TOR1AIP1 | 2,132266459 | 2,058399602 | 0,073866858 | |
| 214787_at | DENND4A | 0,810733188 | 0,73688026 | 0,073852927 | |
| 229370_at | - | 0,810733188 | 0,73688026 | 0,073852927 | |
| 211279_at | NRF1 | 0,133056859 | 0,059218869 | 0,07383799 | |
| 225453_x_at | CCDC124 | 0,133056859 | 0,059218869 | 0,07383799 | |
| 236660_at | - | 0,133056859 | 0,059218869 | 0,07383799 | |
| 210285_x_at | WTAP | 3,568395593 | 3,49464046 | 0,073755133 | |
| 33736_at | STOML1 | 0,273440755 | 0,199702333 | 0,073738422 | |
| 226963_at | BTF3L4 | 4,779830653 | 4,706094468 | 0,073736185 | |
| 201429_s_at | RPL37A | 8,269522091 | 8,195791132 | 0,073730959 | |
| 225729_at | C6orf89 | 1,274448124 | 1,200746069 | 0,073702055 | |
| 232171_x_at | KLHDC4 | 1,515954005 | 1,442269454 | 0,073684551 | |
| 222759_at | SUV420H1 | 3,207971844 | 3,134292219 | 0,073679625 | |
| 202529_at | PRPSAP1 | 2,623460554 | 2,549796788 | 0,073663766 | |
| 226666_at | DAAM1 | 3,598072957 | 3,524430539 | 0,073642418 | |
| 207060_at | EN2 | 1,139070151 | 1,065442793 | 0,073627358 | |
| 217526_at | NFATC2IP | 2,861566439 | 2,787940144 | 0,073626295 | |
| 221700_s_at | UBA52 | 7,466364266 | 7,392798805 | 0,073565461 | |

| | | | | | |
|---|---|---|---|---|---|
| 204119_s_at | ADK | 4,574391988 | 4,500847519 | 0,07354447 | |
| 204552_at | INPP4A | 0,627515113 | 0,553993624 | 0,073521489 | |
| 227219_x_at | MAP1LC3A | 0,627515113 | 0,553993624 | 0,073521489 | |
| 231831_at | COX19 | 0,627515113 | 0,553993624 | 0,073521489 | |
| 213194_at | ROBO1 | 1,90937145 | 1,835911187 | 0,073460263 | |
| 221230_s_at | ARID4B | 3,377277984 | 3,303845028 | 0,073432956 | |
| 1553037_a_at | SYN2 | -1,964176657 | -2,037551727 | 0,07337507 | |
| 1554032_at | ARSB | -1,964176657 | -2,037551727 | 0,07337507 | |
| 1554655_a_at | RPRML | -1,964176657 | -2,037551727 | 0,07337507 | |
| 1554908_at | HYDIN | -1,964176657 | -2,037551727 | 0,07337507 | |
| 1556262_at | PWRN1 | -1,964176657 | -2,037551727 | 0,07337507 | |
| 1558247_s_at | NTN5 | -1,964176657 | -2,037551727 | 0,07337507 | |
| 1561454_at | - | -1,964176657 | -2,037551727 | 0,07337507 | |
| 1562562_at | ANKUB1 | -1,964176657 | -2,037551727 | 0,07337507 | |
| 1563034_at | GPD1 | -1,964176657 | -2,037551727 | 0,07337507 | |
| 205675_at | MTTP | -1,964176657 | -2,037551727 | 0,07337507 | |
| 207433_at | IL10 | -1,964176657 | -2,037551727 | 0,07337507 | |
| 213683_at | ACSL6 /// LOC10 | -1,964176657 | -2,037551727 | 0,07337507 | |
| 213929_at | EXPH5 | -1,964176657 | -2,037551727 | 0,07337507 | |
| 215795_at | MYH7B | -1,964176657 | -2,037551727 | 0,07337507 | |
| 216514_at | - | -1,964176657 | -2,037551727 | 0,07337507 | |
| 216922_x_at | DAZ1 /// DAZ2 /// | -1,964176657 | -2,037551727 | 0,07337507 | |
| 222177_s_at | SCAND2 | -1,964176657 | -2,037551727 | 0,07337507 | |
| 223484_at | C15orf48 | -1,964176657 | -2,037551727 | 0,07337507 | |
| 223529_at | SYT4 | -1,964176657 | -2,037551727 | 0,07337507 | |
| 223710_at | CCL26 | -1,964176657 | -2,037551727 | 0,07337507 | |
| 224192_at | FCRL2 | -1,964176657 | -2,037551727 | 0,07337507 | |
| 227440_at | ANKS1B | -1,964176657 | -2,037551727 | 0,07337507 | |
| 233288_at | ATR | -1,964176657 | -2,037551727 | 0,07337507 | |
| 233330_s_at | - | -1,964176657 | -2,037551727 | 0,07337507 | |
| 238058_at | LOC150381 | -1,964176657 | -2,037551727 | 0,07337507 | |
| 238060_s_at | B4GALNT4 | -1,964176657 | -2,037551727 | 0,07337507 | |
| 244784_at | DHX57 | -1,964176657 | -2,037551727 | 0,07337507 | |
| 222715_s_at | SYNRG | 0,995078803 | 0,921731975 | 0,073346827 | |
| 213564_x_at | LDHB | 6,955177044 | 6,881856054 | 0,07332099 | |
| 1554339_a_at | COG3 | -1,888254346 | -1,961547147 | 0,073292802 | |
| 1559394_a_at | - | -1,888254346 | -1,961547147 | 0,073292802 | |
| 1560078_at | LAMA3 | -1,888254346 | -1,961547147 | 0,073292802 | |
| 1561820_at | SCN8A | -1,888254346 | -1,961547147 | 0,073292802 | |
| 1562785_at | - | -1,888254346 | -1,961547147 | 0,073292802 | |
| 1569792_a_at | METTL20 | -1,888254346 | -1,961547147 | 0,073292802 | |
| 1569854_at | - | -1,888254346 | -1,961547147 | 0,073292802 | |
| 1570630_at | - | -1,888254346 | -1,961547147 | 0,073292802 | |
| 206345_s_at | PON1 | -1,888254346 | -1,961547147 | 0,073292802 | |
| 211044_at | TRIM14 | -1,888254346 | -1,961547147 | 0,073292802 | |
| 211302_s_at | PDE4B | -1,888254346 | -1,961547147 | 0,073292802 | |
| 214936_at | LRCH1 | -1,888254346 | -1,961547147 | 0,073292802 | |
| 215351_at | - | -1,888254346 | -1,961547147 | 0,073292802 | |
| 220783_at | MMP27 | -1,888254346 | -1,961547147 | 0,073292802 | |
| 224293_at | TTTY10 | -1,888254346 | -1,961547147 | 0,073292802 | |
| 224310_s_at | BCL11B | -1,888254346 | -1,961547147 | 0,073292802 | |
| 224421_x_at | PMCHL1 | -1,888254346 | -1,961547147 | 0,073292802 | |
| 229187_at | LOC283788 | -1,888254346 | -1,961547147 | 0,073292802 | |
| 230232_at | SEL1L3 | -1,888254346 | -1,961547147 | 0,073292802 | |
| 232531_at | EMX2OS | -1,888254346 | -1,961547147 | 0,073292802 | |
| 235133_at | ZDHHC14 | -1,888254346 | -1,961547147 | 0,073292802 | |

| | | | | | |
|---|---|---|---|---|---|
| 237164_at | - | -1,888254346 | -1,961547147 | 0,073292802 | |
| 238928_at | - | -1,888254346 | -1,961547147 | 0,073292802 | |
| 240828_at | JARID2-AS1 | -1,888254346 | -1,961547147 | 0,073292802 | |
| 66053_at | HNRNPUL2 /// HI | 0,634475464 | 0,561197255 | 0,073278209 | |
| 1560175_at | PPP4R1L | -1,838812296 | -1,912089812 | 0,073277516 | |
| 205630_at | CRH | -1,838812296 | -1,912089812 | 0,073277516 | |
| 208039_at | - | -1,838812296 | -1,912089812 | 0,073277516 | |
| 209245_s_at | KIF1C | -1,838812296 | -1,912089812 | 0,073277516 | |
| 213895_at | EMP1 | -1,838812296 | -1,912089812 | 0,073277516 | |
| 214534_at | HIST1H1B | -1,838812296 | -1,912089812 | 0,073277516 | |
| 219229_at | SLCO3A1 | -1,838812296 | -1,912089812 | 0,073277516 | |
| 219232_s_at | EGLN3 | -1,838812296 | -1,912089812 | 0,073277516 | |
| 227697_at | SOCS3 | -1,838812296 | -1,912089812 | 0,073277516 | |
| 229497_at | ANKDD1A | -1,838812296 | -1,912089812 | 0,073277516 | |
| 229947_at | PI15 | -1,838812296 | -1,912089812 | 0,073277516 | |
| 233390_at | - | -1,838812296 | -1,912089812 | 0,073277516 | |
| 235941_s_at | FAM224A | -1,838812296 | -1,912089812 | 0,073277516 | |
| 244654_at | MYO1G | -1,838812296 | -1,912089812 | 0,073277516 | |
| 1560679_at | LOC100506328 | -2,01371626 | -2,086984744 | 0,073268483 | |
| 1564282_a_at | LINC00491 | -2,01371626 | -2,086984744 | 0,073268483 | |
| 1570335_at | - | -2,01371626 | -2,086984744 | 0,073268483 | |
| 206773_at | LY6H | -2,01371626 | -2,086984744 | 0,073268483 | |
| 210467_x_at | MAGEA12 | -2,01371626 | -2,086984744 | 0,073268483 | |
| 211132_at | INTS3 | -2,01371626 | -2,086984744 | 0,073268483 | |
| 220088_at | C5AR1 | -2,01371626 | -2,086984744 | 0,073268483 | |
| 222696_at | AXIN2 | -2,01371626 | -2,086984744 | 0,073268483 | |
| 232453_at | - | -2,01371626 | -2,086984744 | 0,073268483 | |
| 234537_at | - | -2,01371626 | -2,086984744 | 0,073268483 | |
| 236185_at | NHLRC2 | -2,01371626 | -2,086984744 | 0,073268483 | |
| 236295_s_at | MIR3929 /// NLR( | -2,01371626 | -2,086984744 | 0,073268483 | |
| 237828_at | SRRM4 | -2,01371626 | -2,086984744 | 0,073268483 | |
| 238986_at | FLJ43663 | -2,01371626 | -2,086984744 | 0,073268483 | |
| 243486_at | - | -2,01371626 | -2,086984744 | 0,073268483 | |
| 244691_at | SETD5 | -2,01371626 | -2,086984744 | 0,073268483 | |
| 218196_at | OSTM1 | 3,444167073 | 3,370970266 | 0,073196808 | |
| 1557389_at | LOC100505839 | -2,089474779 | -2,162639771 | 0,073164992 | |
| 1559526_at | - | -2,089474779 | -2,162639771 | 0,073164992 | |
| 1559737_at | - | -2,089474779 | -2,162639771 | 0,073164992 | |
| 1561723_at | - | -2,089474779 | -2,162639771 | 0,073164992 | |
| 1564828_at | KIAA1211 | -2,089474779 | -2,162639771 | 0,073164992 | |
| 1564972_x_at | SETDB2 | -2,089474779 | -2,162639771 | 0,073164992 | |
| 204400_at | EFS | -2,089474779 | -2,162639771 | 0,073164992 | |
| 206458_s_at | WNT2B | -2,089474779 | -2,162639771 | 0,073164992 | |
| 207581_s_at | MAGEB4 | -2,089474779 | -2,162639771 | 0,073164992 | |
| 208421_at | - | -2,089474779 | -2,162639771 | 0,073164992 | |
| 214529_at | TSHB | -2,089474779 | -2,162639771 | 0,073164992 | |
| 214985_at | EXT1 | -2,089474779 | -2,162639771 | 0,073164992 | |
| 217068_at | - | -2,089474779 | -2,162639771 | 0,073164992 | |
| 219528_s_at | BCL11B | -2,089474779 | -2,162639771 | 0,073164992 | |
| 219750_at | TMEM144 | -2,089474779 | -2,162639771 | 0,073164992 | |
| 220184_at | NANOG | -2,089474779 | -2,162639771 | 0,073164992 | |
| 221075_s_at | NCR2 | -2,089474779 | -2,162639771 | 0,073164992 | |
| 228534_s_at | - | -2,089474779 | -2,162639771 | 0,073164992 | |
| 228969_at | AGR2 | -2,089474779 | -2,162639771 | 0,073164992 | |
| 229877_at | NEO1 | -2,089474779 | -2,162639771 | 0,073164992 | |
| 230928_at | - | -2,089474779 | -2,162639771 | 0,073164992 | |

| | | | | | |
|---|---|---|---|---|---|
| 232485_at | SNX29 | -2,089474779 | -2,162639771 | 0,073164992 | |
| 232689_at | LOC284561 | -2,089474779 | -2,162639771 | 0,073164992 | |
| 232734_at | TTC23 | -2,089474779 | -2,162639771 | 0,073164992 | |
| 236786_at | FAM135B | -2,089474779 | -2,162639771 | 0,073164992 | |
| 237787_at | - | -2,089474779 | -2,162639771 | 0,073164992 | |
| 238226_at | FAM70B | -2,089474779 | -2,162639771 | 0,073164992 | |
| 238354_x_at | - | -2,089474779 | -2,162639771 | 0,073164992 | |
| 238846_at | TNFRSF11A | -2,089474779 | -2,162639771 | 0,073164992 | |
| 239653_at | - | -2,089474779 | -2,162639771 | 0,073164992 | |
| 244238_at | - | -2,089474779 | -2,162639771 | 0,073164992 | |
| 244796_at | - | -2,089474779 | -2,162639771 | 0,073164992 | |
| 1564333_a_at | PSAPL1 | 0,152582607 | 0,079428135 | 0,073154472 | |
| 232534_at | LIN37 | 0,152582607 | 0,079428135 | 0,073154472 | |
| 200693_at | YWHAQ | 6,134630082 | 6,061481705 | 0,073148377 | |
| 208923_at | CYFIP1 | 4,322962984 | 4,249823666 | 0,073139318 | |
| 203352_at | ORC4 | 1,153678675 | 1,080561626 | 0,073117049 | |
| 208947_s_at | UPF1 | 1,153678675 | 1,080561626 | 0,073117049 | |
| 200016_x_at | HNRNPA1 | 7,680585549 | 7,607475867 | 0,073109682 | |
| 217811_at | SELT | 6,035409686 | 5,962345958 | 0,073063728 | |
| 215587_x_at | - | 0,641402394 | 0,568365095 | 0,073037299 | |
| 223091_x_at | MFF | 0,641402394 | 0,568365095 | 0,073037299 | |
| 209835_x_at | CD44 | 1,534677255 | 1,461646549 | 0,073030706 | |
| 213110_s_at | COL4A5 | 1,534677255 | 1,461646549 | 0,073030706 | |
| 203107_x_at | RPS2 /// SNORA( | 8,124340598 | 8,051312814 | 0,073027785 | |
| 215982_s_at | DOM3Z | 2,227406889 | 2,154405725 | 0,073001164 | |
| 201015_s_at | JUP | 2,923969333 | 2,851017268 | 0,072952065 | |
| 1553157_at | LHX4 | -2,138804305 | -2,211750327 | 0,072946022 | |
| 1553335_x_at | LOC285696 | -2,138804305 | -2,211750327 | 0,072946022 | |
| 1555580_at | - | -2,138804305 | -2,211750327 | 0,072946022 | |
| 1556725_a_at | LOC100288490 | -2,138804305 | -2,211750327 | 0,072946022 | |
| 1558682_at | HMGA2 | -2,138804305 | -2,211750327 | 0,072946022 | |
| 1564148_at | - | -2,138804305 | -2,211750327 | 0,072946022 | |
| 1569122_at | - | -2,138804305 | -2,211750327 | 0,072946022 | |
| 206030_at | ASPA | -2,138804305 | -2,211750327 | 0,072946022 | |
| 214557_at | PTTG2 | -2,138804305 | -2,211750327 | 0,072946022 | |
| 215262_at | - | -2,138804305 | -2,211750327 | 0,072946022 | |
| 215284_at | - | -2,138804305 | -2,211750327 | 0,072946022 | |
| 217424_at | - | -2,138804305 | -2,211750327 | 0,072946022 | |
| 218182_s_at | CLDN1 | -2,138804305 | -2,211750327 | 0,072946022 | |
| 219965_s_at | MAGIX | -2,138804305 | -2,211750327 | 0,072946022 | |
| 223926_at | KIF2B | -2,138804305 | -2,211750327 | 0,072946022 | |
| 223975_at | TRIM51 /// TRIM5 | -2,138804305 | -2,211750327 | 0,072946022 | |
| 231589_at | - | -2,138804305 | -2,211750327 | 0,072946022 | |
| 231696_x_at | TMEM50B | -2,138804305 | -2,211750327 | 0,072946022 | |
| 231762_at | FGF10 | -2,138804305 | -2,211750327 | 0,072946022 | |
| 235523_at | CTC1 | -2,138804305 | -2,211750327 | 0,072946022 | |
| 238165_at | PDZRN3 | -2,138804305 | -2,211750327 | 0,072946022 | |
| 241110_at | - | -2,138804305 | -2,211750327 | 0,072946022 | |
| 241759_at | - | -2,138804305 | -2,211750327 | 0,072946022 | |
| 243770_at | ZNF483 | -2,138804305 | -2,211750327 | 0,072946022 | |
| 243784_s_at | LOC100272217 | -2,138804305 | -2,211750327 | 0,072946022 | |
| 200975_at | PPT1 | 5,158634383 | 5,085689237 | 0,072945147 | |
| 1555860_x_at | LOC440944 | 1,296557217 | 1,223626587 | 0,07293063 | |
| 221979_at | LOC100129250 | 2,745814053 | 2,672945617 | 0,072868435 | |
| 1552965_a_at | B3GNT7 | -1,71423189 | -1,787089803 | 0,072857913 | |
| 1560787_at | QDPR | -1,71423189 | -1,787089803 | 0,072857913 | |

| | | | | | |
|---|---|---|---|---|---|
| 1562590_at | PRTG | -1,71423189 | -1,787089803 | 0,072857913 | |
| 1562625_at | FRYL | -1,71423189 | -1,787089803 | 0,072857913 | |
| 1565608_at | - | -1,71423189 | -1,787089803 | 0,072857913 | |
| 205968_at | KCNS3 | -1,71423189 | -1,787089803 | 0,072857913 | |
| 206684_s_at | ATF7 | -1,71423189 | -1,787089803 | 0,072857913 | |
| 207558_s_at | PITX2 | -1,71423189 | -1,787089803 | 0,072857913 | |
| 215144_at | - | -1,71423189 | -1,787089803 | 0,072857913 | |
| 236163_at | LIX1 | -1,71423189 | -1,787089803 | 0,072857913 | |
| 243823_at | - | -1,71423189 | -1,787089803 | 0,072857913 | |
| 243894_at | SLC41A2 | -1,71423189 | -1,787089803 | 0,072857913 | |
| 244747_at | NPNT | -1,71423189 | -1,787089803 | 0,072857913 | |
| 210288_at | KLRG1 | 0,425644434 | 0,352836757 | 0,072807676 | |
| 234993_at | ABHD13 | 0,425644434 | 0,352836757 | 0,072807676 | |
| 213324_at | SRC | 0,841135803 | 0,768343791 | 0,072792012 | |
| 201961_s_at | RNF41 | 1,163336153 | 1,090553537 | 0,072782616 | |
| 227157_at | CCDC111 | 1,542099044 | 1,469325098 | 0,072773946 | |
| 201813_s_at | TBC1D5 | 1,748661581 | 1,675891488 | 0,072770093 | |
| 204866_at | PHF16 | 3,456984333 | 3,384231608 | 0,072752725 | |
| 204319_s_at | RGS10 | 4,749296484 | 4,676548291 | 0,072748194 | |
| 208783_s_at | CD46 | 4,689563828 | 4,616873117 | 0,072690711 | |
| 225530_at | MOB3A | 1,932146506 | 1,859476797 | 0,072669709 | |
| 212103_at | KPNA6 /// LOC10 | 3,014789032 | 2,942125533 | 0,072663499 | |
| 201073_s_at | SMARCC1 | 2,546213279 | 2,473581079 | 0,0726322 | |
| 209806_at | HIST1H2BK | 7,706206571 | 7,633605262 | 0,072601309 | |
| 212093_s_at | MTUS1 | 2,097427503 | 2,024923328 | 0,072504175 | |
| 223506_at | ZC3H8 | 3,523171367 | 3,450709925 | 0,072461442 | |
| 225600_at | TRIQK | 1,758252436 | 1,685812099 | 0,072440337 | |
| 207061_at | ERN1 | -0,148196756 | -0,220592223 | 0,072395467 | |
| 211605_s_at | RARA | -0,148196756 | -0,220592223 | 0,072395467 | |
| 231006_at | SPATA8 | -0,148196756 | -0,220592223 | 0,072395467 | |
| 231531_at | C3orf24 | -0,148196756 | -0,220592223 | 0,072395467 | |
| 1553299_at | DUSP5P | -2,262747984 | -2,335104118 | 0,072356134 | |
| 1553793_a_at | KIAA1109 | -2,262747984 | -2,335104118 | 0,072356134 | |
| 1558306_at | THADA | -2,262747984 | -2,335104118 | 0,072356134 | |
| 1559624_at | STK32A | -2,262747984 | -2,335104118 | 0,072356134 | |
| 1565588_at | SP140L | -2,262747984 | -2,335104118 | 0,072356134 | |
| 1566301_at | PPP1R11 | -2,262747984 | -2,335104118 | 0,072356134 | |
| 1570360_s_at | DDX3Y | -2,262747984 | -2,335104118 | 0,072356134 | |
| 205314_x_at | SNTB2 | -2,262747984 | -2,335104118 | 0,072356134 | |
| 206218_at | MAGEB2 | -2,262747984 | -2,335104118 | 0,072356134 | |
| 210560_at | GBX2 | -2,262747984 | -2,335104118 | 0,072356134 | |
| 214796_at | KIAA1456 | -2,262747984 | -2,335104118 | 0,072356134 | |
| 220696_at | - | -2,262747984 | -2,335104118 | 0,072356134 | |
| 231308_at | - | -2,262747984 | -2,335104118 | 0,072356134 | |
| 232941_at | TMPRSS6 | -2,262747984 | -2,335104118 | 0,072356134 | |
| 233291_at | - | -2,262747984 | -2,335104118 | 0,072356134 | |
| 234556_at | - | -2,262747984 | -2,335104118 | 0,072356134 | |
| 236944_at | - | -2,262747984 | -2,335104118 | 0,072356134 | |
| 237543_at | - | -2,262747984 | -2,335104118 | 0,072356134 | |
| 237627_at | - | -2,262747984 | -2,335104118 | 0,072356134 | |
| 240545_at | LOC286382 | -2,262747984 | -2,335104118 | 0,072356134 | |
| 240715_at | TBX5 | -2,262747984 | -2,335104118 | 0,072356134 | |
| 242656_at | GTF2H1 | -2,262747984 | -2,335104118 | 0,072356134 | |
| 243431_at | - | -2,262747984 | -2,335104118 | 0,072356134 | |
| 243990_at | - | -2,262747984 | -2,335104118 | 0,072356134 | |
| 201308_s_at | 40787 | 0,661985843 | 0,589657387 | 0,072328456 | |

| | | | | | |
|---|---|---|---|---|---|
| 217363_x_at | - | 1,177702228 | 1,105412836 | 0,072289392 | |
| 1563045_at | - | -0,57445847 | -0,646746079 | 0,072287608 | |
| 205844_at | VNN1 | -0,57445847 | -0,646746079 | 0,072287608 | |
| 211469_s_at | CXCR6 | -0,57445847 | -0,646746079 | 0,072287608 | |
| 223971_at | OR2A20P /// OR2 | -0,57445847 | -0,646746079 | 0,072287608 | |
| 229272_at | FNBP4 | -0,57445847 | -0,646746079 | 0,072287608 | |
| 229591_at | LRP5 | -0,57445847 | -0,646746079 | 0,072287608 | |
| 236899_at | - | -0,57445847 | -0,646746079 | 0,072287608 | |
| 243299_at | - | -0,57445847 | -0,646746079 | 0,072287608 | |
| 226165_at | C8orf59 | 6,082297741 | 6,01009441 | 0,07220333 | |
| 201680_x_at | SRRT | 4,935173991 | 4,862972786 | 0,072201205 | |
| 228024_at | VPS37A | 1,318332599 | 1,246149888 | 0,072182711 | |
| 219778_at | ZFPM2 | 0,181384709 | 0,109221392 | 0,072163317 | |
| 229024_at | - | 0,181384709 | 0,109221392 | 0,072163317 | |
| 229033_s_at | MUM1 | 0,181384709 | 0,109221392 | 0,072163317 | |
| 222977_at | SURF4 | 4,252325187 | 4,180180181 | 0,072145007 | |
| 242873_at | - | 2,030406107 | 1,958277216 | 0,072128891 | |
| 1552455_at | PRUNE2 | -1,591296941 | -1,663414495 | 0,072117553 | |
| 1553282_at | C21orf128 | -1,591296941 | -1,663414495 | 0,072117553 | |
| 1553443_at | FER1L6-AS1 | -1,591296941 | -1,663414495 | 0,072117553 | |
| 1563404_at | - | -1,591296941 | -1,663414495 | 0,072117553 | |
| 1565065_at | OFCC1 | -1,591296941 | -1,663414495 | 0,072117553 | |
| 1566514_at | CWF19L2 | -1,591296941 | -1,663414495 | 0,072117553 | |
| 205158_at | RNASE4 | -1,591296941 | -1,663414495 | 0,072117553 | |
| 205500_at | C5 | -1,591296941 | -1,663414495 | 0,072117553 | |
| 206375_s_at | HSPB3 | -1,591296941 | -1,663414495 | 0,072117553 | |
| 211889_x_at | CEACAM1 | -1,591296941 | -1,663414495 | 0,072117553 | |
| 214871_x_at | LOC100287590 | -1,591296941 | -1,663414495 | 0,072117553 | |
| 215862_at | - | -1,591296941 | -1,663414495 | 0,072117553 | |
| 216773_at | - | -1,591296941 | -1,663414495 | 0,072117553 | |
| 221668_s_at | DNAI2 | -1,591296941 | -1,663414495 | 0,072117553 | |
| 223745_at | C16orf95 | -1,591296941 | -1,663414495 | 0,072117553 | |
| 225021_at | ZNF532 | -1,591296941 | -1,663414495 | 0,072117553 | |
| 228212_at | ISM2 | -1,591296941 | -1,663414495 | 0,072117553 | |
| 229004_at | ADAMTS15 | -1,591296941 | -1,663414495 | 0,072117553 | |
| 229993_at | ZCCHC3 | -1,591296941 | -1,663414495 | 0,072117553 | |
| 233442_at | - | -1,591296941 | -1,663414495 | 0,072117553 | |
| 233850_s_at | EBF4 | -1,591296941 | -1,663414495 | 0,072117553 | |
| 234965_at | - | -1,591296941 | -1,663414495 | 0,072117553 | |
| 237856_at | RAP1GDS1 | -1,591296941 | -1,663414495 | 0,072117553 | |
| 237862_at | LOC100506189 | -1,591296941 | -1,663414495 | 0,072117553 | |
| 239826_at | - | -1,591296941 | -1,663414495 | 0,072117553 | |
| 242572_at | - | -1,591296941 | -1,663414495 | 0,072117553 | |
| 242598_at | - | -1,591296941 | -1,663414495 | 0,072117553 | |
| 243137_at | - | -1,591296941 | -1,663414495 | 0,072117553 | |
| 212191_x_at | RPL13 /// SNORD | 6,932412531 | 6,860299605 | 0,072112927 | |
| 201487_at | CTSC | 3,671816548 | 3,599822964 | 0,071993583 | |
| 206291_at | NTS | 7,484474452 | 7,412504958 | 0,071969494 | |
| 222537_s_at | CDC42SE1 | 1,187200746 | 1,115234684 | 0,071966062 | |
| 223663_at | CCDC88B | 1,187200746 | 1,115234684 | 0,071966062 | |
| 224892_at | PLDN | 4,954075234 | 4,8821546 | 0,071920634 | |
| 229460_at | FAM126B | 2,912562019 | 2,840694075 | 0,071867944 | |
| 1552291_at | PIGX | 0,67554678 | 0,603679647 | 0,071867133 | |
| 1554160_a_at | ZNF446 | 0,67554678 | 0,603679647 | 0,071867133 | |
| 212026_s_at | EXOC7 | 0,67554678 | 0,603679647 | 0,071867133 | |
| 225905_s_at | ST3GAL3 | 0,67554678 | 0,603679647 | 0,071867133 | |

| | | | | | |
|---|---|---|---|---|---|
| 222748_s_at | TXNL4B | 1,868628927 | 1,796776588 | 0,071852339 | |
| 1562274_at | - | -1,543331864 | -1,615132408 | 0,071800543 | |
| 205911_at | PTH1R | -1,543331864 | -1,615132408 | 0,071800543 | |
| 214497_s_at | NHLH2 | -1,543331864 | -1,615132408 | 0,071800543 | |
| 218044_x_at | PTMS | -1,543331864 | -1,615132408 | 0,071800543 | |
| 231456_at | - | -1,543331864 | -1,615132408 | 0,071800543 | |
| 232526_at | ITPKB | -1,543331864 | -1,615132408 | 0,071800543 | |
| 232956_at | - | -1,543331864 | -1,615132408 | 0,071800543 | |
| 235046_at | INPP4B | -1,543331864 | -1,615132408 | 0,071800543 | |
| 236621_at | RPS27 | -1,543331864 | -1,615132408 | 0,071800543 | |
| 238173_at | TCEA2 | -1,543331864 | -1,615132408 | 0,071800543 | |
| 239593_at | TMEM213 | -1,543331864 | -1,615132408 | 0,071800543 | |
| 242489_at | - | -1,543331864 | -1,615132408 | 0,071800543 | |
| 242778_at | LPXN | -1,543331864 | -1,615132408 | 0,071800543 | |
| 221474_at | MYL12B | 6,263267197 | 6,191493336 | 0,071773861 | |
| 214288_s_at | PSMB1 | 7,230712925 | 7,158939524 | 0,071773401 | |
| 203781_at | MRPL33 | 4,671912228 | 4,600140481 | 0,071771747 | |
| 204174_at | ALOX5AP | 1,331241794 | 1,259496994 | 0,071744799 | |
| 238190_at | TUFM | 1,331241794 | 1,259496994 | 0,071744799 | |
| 222103_at | ATF1 | 4,60394751 | 4,532344882 | 0,071602628 | |
| 208986_at | TCF12 | 3,899783451 | 3,828227545 | 0,071555906 | |
| 201458_s_at | BUB3 | 4,28697245 | 4,215422121 | 0,071550328 | |
| 1556749_at | - | -2,384869671 | -2,456406426 | 0,071536755 | |
| 1557398_at | - | -2,384869671 | -2,456406426 | 0,071536755 | |
| 1564856_s_at | LOC727924 | -2,384869671 | -2,456406426 | 0,071536755 | |
| 1566486_at | - | -2,384869671 | -2,456406426 | 0,071536755 | |
| 1566605_at | TEX9 | -2,384869671 | -2,456406426 | 0,071536755 | |
| 209309_at | AZGP1 | -2,384869671 | -2,456406426 | 0,071536755 | |
| 211736_at | SP2 | -2,384869671 | -2,456406426 | 0,071536755 | |
| 214982_at | SNRNP200 | -2,384869671 | -2,456406426 | 0,071536755 | |
| 215290_at | - | -2,384869671 | -2,456406426 | 0,071536755 | |
| 216972_at | SPAM1 | -2,384869671 | -2,456406426 | 0,071536755 | |
| 217623_at | MYLK3 | -2,384869671 | -2,456406426 | 0,071536755 | |
| 229942_at | BNC2 | -2,384869671 | -2,456406426 | 0,071536755 | |
| 233047_at | FRMD7 | -2,384869671 | -2,456406426 | 0,071536755 | |
| 233704_at | - | -2,384869671 | -2,456406426 | 0,071536755 | |
| 239724_at | - | -2,384869671 | -2,456406426 | 0,071536755 | |
| 240814_at | MGC39584 | -2,384869671 | -2,456406426 | 0,071536755 | |
| 243200_at | - | -2,384869671 | -2,456406426 | 0,071536755 | |
| 1554102_a_at | TMTC4 | 0,20027154 | 0,128747141 | 0,071524399 | |
| 204746_s_at | PICK1 | 0,20027154 | 0,128747141 | 0,071524399 | |
| 221443_x_at | PRLH | 0,20027154 | 0,128747141 | 0,071524399 | |
| 242974_at | CD47 | 0,20027154 | 0,128747141 | 0,071524399 | |
| 229654_at | - | 3,270743649 | 3,199221717 | 0,071521932 | |
| 210149_s_at | ATP5H | 6,36275821 | 6,291253623 | 0,071504588 | |
| 203774_at | MTR | 3,606946883 | 3,535445117 | 0,071501766 | |
| 230268_at | - | 1,201332287 | 1,129843209 | 0,071489078 | |
| 232181_at | PPARGC1B | 1,201332287 | 1,129843209 | 0,071489078 | |
| 223042_s_at | FUNDC2 | 4,846163912 | 4,77470758 | 0,071456332 | |
| 224655_at | AK3 | 3,738747209 | 3,667303849 | 0,07144336 | |
| 222503_s_at | WDR41 | 3,495722611 | 3,424287521 | 0,071435089 | |
| 219785_s_at | C16orf95 | 2,051361651 | 1,979940779 | 0,071420873 | |
| 214243_s_at | SERHL /// SERHI | -0,543051993 | -0,614371577 | 0,071319583 | |
| 227537_s_at | SP3 | -0,543051993 | -0,614371577 | 0,071319583 | |
| 228458_at | C6orf226 | -0,543051993 | -0,614371577 | 0,071319583 | |
| 231991_at | C20orf160 | -0,543051993 | -0,614371577 | 0,071319583 | |

| | | | | | |
|---|---|---|---|---|---|
| 232016_at | - | -0,543051993 | -0,614371577 | 0,071319583 | |
| 237583_at | - | -0,543051993 | -0,614371577 | 0,071319583 | |
| 203010_at | STAT5A | 3,132544588 | 3,061230246 | 0,071314343 | |
| 1564232_at | LOC100506453 / | 0,695652136 | 0,624460759 | 0,071191377 | |
| 210236_at | PPFIA1 | 0,888485433 | 0,817300337 | 0,071185097 | |
| 208882_s_at | UBR5 | 4,040056475 | 3,968932193 | 0,071124282 | |
| 1553127_a_at | RNF168 | -2,43257096 | -2,503677622 | 0,071106662 | |
| 1554624_a_at | LOC100653194 / | -2,43257096 | -2,503677622 | 0,071106662 | |
| 1555261_at | - | -2,43257096 | -2,503677622 | 0,071106662 | |
| 1557874_at | GRID1-AS1 | -2,43257096 | -2,503677622 | 0,071106662 | |
| 1560422_at | - | -2,43257096 | -2,503677622 | 0,071106662 | |
| 1560960_at | - | -2,43257096 | -2,503677622 | 0,071106662 | |
| 1565898_at | METTL15 | -2,43257096 | -2,503677622 | 0,071106662 | |
| 1566465_at | KCNK1 | -2,43257096 | -2,503677622 | 0,071106662 | |
| 1570290_at | - | -2,43257096 | -2,503677622 | 0,071106662 | |
| 1570601_at | - | -2,43257096 | -2,503677622 | 0,071106662 | |
| 205619_s_at | MEOX1 | -2,43257096 | -2,503677622 | 0,071106662 | |
| 209988_s_at | ASCL1 | -2,43257096 | -2,503677622 | 0,071106662 | |
| 217577_at | - | -2,43257096 | -2,503677622 | 0,071106662 | |
| 220380_at | DNASE2B | -2,43257096 | -2,503677622 | 0,071106662 | |
| 220639_at | TM4SF20 | -2,43257096 | -2,503677622 | 0,071106662 | |
| 223986_x_at | DMRT2 | -2,43257096 | -2,503677622 | 0,071106662 | |
| 224295_at | - | -2,43257096 | -2,503677622 | 0,071106662 | |
| 231405_at | SPACA7 | -2,43257096 | -2,503677622 | 0,071106662 | |
| 233094_at | - | -2,43257096 | -2,503677622 | 0,071106662 | |
| 233556_at | CRYBB2P1 | -2,43257096 | -2,503677622 | 0,071106662 | |
| 234764_x_at | CKAP2 /// IGLC1 | -2,43257096 | -2,503677622 | 0,071106662 | |
| 234778_at | - | -2,43257096 | -2,503677622 | 0,071106662 | |
| 235199_at | RNF125 | -2,43257096 | -2,503677622 | 0,071106662 | |
| 235778_s_at | ANKRD44 | -2,43257096 | -2,503677622 | 0,071106662 | |
| 238258_at | WBSCR28 | -2,43257096 | -2,503677622 | 0,071106662 | |
| 239829_at | - | -2,43257096 | -2,503677622 | 0,071106662 | |
| 240838_s_at | LOC145837 | -2,43257096 | -2,503677622 | 0,071106662 | |
| 241621_at | SMCHD1 | -2,43257096 | -2,503677622 | 0,071106662 | |
| 243525_at | - | -2,43257096 | -2,503677622 | 0,071106662 | |
| 244225_x_at | LMNA | -2,43257096 | -2,503677622 | 0,071106662 | |
| 1553723_at | GPR97 | -1,470833215 | -1,541908042 | 0,071074826 | |
| 1555738_at | CMTM1 | -1,470833215 | -1,541908042 | 0,071074826 | |
| 1558053_s_at | TMED4 | -1,470833215 | -1,541908042 | 0,071074826 | |
| 1566476_at | - | -1,470833215 | -1,541908042 | 0,071074826 | |
| 1568864_at | LOC100131691 | -1,470833215 | -1,541908042 | 0,071074826 | |
| 209847_at | CDH17 | -1,470833215 | -1,541908042 | 0,071074826 | |
| 215750_at | - | -1,470833215 | -1,541908042 | 0,071074826 | |
| 222776_at | MAGOHB | -1,470833215 | -1,541908042 | 0,071074826 | |
| 223691_at | RGS22 | -1,470833215 | -1,541908042 | 0,071074826 | |
| 229064_s_at | RCAN3 | -1,470833215 | -1,541908042 | 0,071074826 | |
| 232625_at | TLN2 | -1,470833215 | -1,541908042 | 0,071074826 | |
| 232805_at | - | -1,470833215 | -1,541908042 | 0,071074826 | |
| 232954_at | - | -1,470833215 | -1,541908042 | 0,071074826 | |
| 235754_at | HFE | -1,470833215 | -1,541908042 | 0,071074826 | |
| 236508_at | SLC6A5 | -1,470833215 | -1,541908042 | 0,071074826 | |
| 237971_at | - | -1,470833215 | -1,541908042 | 0,071074826 | |
| 240834_at | FAM105B | -1,470833215 | -1,541908042 | 0,071074826 | |
| 241663_at | C3orf23 | -1,470833215 | -1,541908042 | 0,071074826 | |
| 244252_at | LOC399884 | -1,470833215 | -1,541908042 | 0,071074826 | |
| 244402_at | - | -1,470833215 | -1,541908042 | 0,071074826 | |

| | | | | | |
|---|---|---|---|---|---|
| 208759_at | NCSTN | 2,416948786 | 2,345926937 | 0,071021849 | |
| 212262_at | QKI | 3,024094427 | 2,953119918 | 0,070974509 | |
| 64883_at | MOSPD2 | 1,143956113 | 1,073022014 | 0,070934099 | |
| 218572_at | CHMP4A /// TM9 | 2,596981013 | 2,526063925 | 0,070917088 | |
| 228738_at | D2HGDH | 1,481627361 | 1,410714371 | 0,07091299 | |
| 203041_s_at | LAMP2 | 4,291933439 | 4,221118236 | 0,070815203 | |
| 217773_s_at | NDUFA4 | 6,257643072 | 6,18684359 | 0,070799482 | |
| 213792_s_at | INSR | 2,807162789 | 2,7363693 | 0,070793489 | |
| 202348_s_at | TOR1A | 3,292909151 | 3,222126232 | 0,070782919 | |
| 218607_s_at | SDAD1 | 3,425226911 | 3,354479478 | 0,070747434 | |
| 1553011_at | TAF1L | -1,424023738 | -1,494668682 | 0,070644943 | |
| 1564639_at | LOC100291323 | -1,424023738 | -1,494668682 | 0,070644943 | |
| 1568698_at | TMEM232 | -1,424023738 | -1,494668682 | 0,070644943 | |
| 217154_s_at | EDN3 | -1,424023738 | -1,494668682 | 0,070644943 | |
| 228570_at | BTBD11 | -1,424023738 | -1,494668682 | 0,070644943 | |
| 234082_at | - | -1,424023738 | -1,494668682 | 0,070644943 | |
| 234775_at | OR51B5 | -1,424023738 | -1,494668682 | 0,070644943 | |
| 237263_at | - | -1,424023738 | -1,494668682 | 0,070644943 | |
| 238188_at | LOC100506642 | -1,424023738 | -1,494668682 | 0,070644943 | |
| 239351_at | - | -1,424023738 | -1,494668682 | 0,070644943 | |
| 241380_at | ARHGEF37 | -1,424023738 | -1,494668682 | 0,070644943 | |
| 241778_at | NT5C2 | -1,424023738 | -1,494668682 | 0,070644943 | |
| 244517_x_at | - | -1,424023738 | -1,494668682 | 0,070644943 | |
| 211595_s_at | MRPS11 | 3,787958926 | 3,717332003 | 0,070626923 | |
| 1562257_x_at | NLRP1 | 0,228146222 | 0,157549243 | 0,070596979 | |
| 242159_at | LOC100506684 | 0,228146222 | 0,157549243 | 0,070596979 | |
| 207039_at | CDKN2A | 5,206127244 | 5,135553145 | 0,070574099 | |
| 200599_s_at | HSP90B1 /// MIR | 6,505019418 | 6,434446889 | 0,070572529 | |
| 200874_s_at | MIR1292 /// NOP | 2,861566439 | 2,791007185 | 0,070559254 | |
| 223411_at | MIF4GD | 1,814495998 | 1,743944487 | 0,070551511 | |
| 235683_at | SESN3 | 1,814495998 | 1,743944487 | 0,070551511 | |
| 1555454_at | LITAF | -2,504600601 | -2,575129729 | 0,070529128 | |
| 1557197_a_at | LGALS3 | -2,504600601 | -2,575129729 | 0,070529128 | |
| 1557309_at | DENND1B | -2,504600601 | -2,575129729 | 0,070529128 | |
| 1559412_at | LINC00478 | -2,504600601 | -2,575129729 | 0,070529128 | |
| 1560051_at | - | -2,504600601 | -2,575129729 | 0,070529128 | |
| 1561027_at | LOC646241 | -2,504600601 | -2,575129729 | 0,070529128 | |
| 1561180_at | - | -2,504600601 | -2,575129729 | 0,070529128 | |
| 1562791_at | MYCBPAP | -2,504600601 | -2,575129729 | 0,070529128 | |
| 1562941_at | - | -2,504600601 | -2,575129729 | 0,070529128 | |
| 1563420_at | XG /// XGPY2 | -2,504600601 | -2,575129729 | 0,070529128 | |
| 1564389_at | - | -2,504600601 | -2,575129729 | 0,070529128 | |
| 1566442_at | - | -2,504600601 | -2,575129729 | 0,070529128 | |
| 1569819_at | PWRN1 | -2,504600601 | -2,575129729 | 0,070529128 | |
| 206581_at | BNC1 | -2,504600601 | -2,575129729 | 0,070529128 | |
| 207261_at | CNGA3 | -2,504600601 | -2,575129729 | 0,070529128 | |
| 207655_s_at | BLNK | -2,504600601 | -2,575129729 | 0,070529128 | |
| 211351_at | KIF25-AS1 | -2,504600601 | -2,575129729 | 0,070529128 | |
| 212328_at | LIMCH1 | -2,504600601 | -2,575129729 | 0,070529128 | |
| 213285_at | TMEM30B | -2,504600601 | -2,575129729 | 0,070529128 | |
| 214586_at | GPR37 | -2,504600601 | -2,575129729 | 0,070529128 | |
| 215561_s_at | IL1R1 | -2,504600601 | -2,575129729 | 0,070529128 | |
| 227646_at | EBF1 | -2,504600601 | -2,575129729 | 0,070529128 | |
| 232252_at | DUSP27 | -2,504600601 | -2,575129729 | 0,070529128 | |
| 235599_at | LOC339535 | -2,504600601 | -2,575129729 | 0,070529128 | |
| 237898_at | - | -2,504600601 | -2,575129729 | 0,070529128 | |

| | | | | | |
|---|---|---|---|---|---|
| 241272_at | - | -2,504600601 | -2,575129729 | 0,070529128 | |
| 241463_at | EIF2AK3 | -2,504600601 | -2,575129729 | 0,070529128 | |
| 241890_at | - | -2,504600601 | -2,575129729 | 0,070529128 | |
| 243655_x_at | - | -2,504600601 | -2,575129729 | 0,070529128 | |
| 243935_at | FRAS1 | -2,504600601 | -2,575129729 | 0,070529128 | |
| 244009_at | - | -2,504600601 | -2,575129729 | 0,070529128 | |
| 244105_at | LOC100652840 / | -2,504600601 | -2,575129729 | 0,070529128 | |
| 1569933_at | - | -0,089689931 | -0,160136748 | 0,070446817 | |
| 205170_at | STAT2 | -0,089689931 | -0,160136748 | 0,070446817 | |
| 213247_at | SVEP1 | -0,089689931 | -0,160136748 | 0,070446817 | |
| 219997_s_at | COPS7B | 1,912238072 | 1,841838775 | 0,070399297 | |
| 214383_x_at | KLHDC3 | 3,668429488 | 3,598072942 | 0,070356546 | |
| 223714_at | ZNF256 | 1,820612054 | 1,750261389 | 0,070350665 | |
| 205090_s_at | NAGPA | 1,373456798 | 1,303116084 | 0,070340714 | |
| 214624_at | UPK1A | 0,917308734 | 0,847075464 | 0,07023327 | |
| 225574_at | RWDD4 | 3,377277984 | 3,307062282 | 0,070215702 | |
| 209243_s_at | PEG3 | 2,825526235 | 2,755336312 | 0,070189923 | |
| 204819_at | FGD1 | 0,503742507 | 0,433557298 | 0,070185209 | |
| 205351_at | GGCX | 0,503742507 | 0,433557298 | 0,070185209 | |
| 241741_at | CRLS1 | 0,503742507 | 0,433557298 | 0,070185209 | |
| 215792_s_at | DNAJC11 | 2,44295215 | 2,372786857 | 0,070165292 | |
| 209900_s_at | SLC16A1 | 3,850569363 | 3,780418488 | 0,070150875 | |
| 235210_s_at | SBSPON | 2,089851283 | 2,019703483 | 0,0701478 | |
| 224709_s_at | CDC42SE2 | 3,877221251 | 3,807174423 | 0,070046828 | |
| 218116_at | C9orf78 | 3,62365843 | 3,553617909 | 0,070040521 | |
| 224535_s_at | MRP63 | 3,62365843 | 3,553617909 | 0,070040521 | |
| 1554224_at | - | -2,551294263 | -2,621331989 | 0,070037726 | |
| 1557359_at | LOC285758 | -2,551294263 | -2,621331989 | 0,070037726 | |
| 1560021_at | - | -2,551294263 | -2,621331989 | 0,070037726 | |
| 1560776_at | - | -2,551294263 | -2,621331989 | 0,070037726 | |
| 1562311_at | - | -2,551294263 | -2,621331989 | 0,070037726 | |
| 1563606_a_at | LOC286359 | -2,551294263 | -2,621331989 | 0,070037726 | |
| 1564485_at | LOC100131551 | -2,551294263 | -2,621331989 | 0,070037726 | |
| 1566642_at | - | -2,551294263 | -2,621331989 | 0,070037726 | |
| 1570411_at | LOC100133131 | -2,551294263 | -2,621331989 | 0,070037726 | |
| 211508_s_at | GH2 | -2,551294263 | -2,621331989 | 0,070037726 | |
| 213025_at | THUMPD1 | -2,551294263 | -2,621331989 | 0,070037726 | |
| 217387_at | CAPN6 | -2,551294263 | -2,621331989 | 0,070037726 | |
| 227949_at | PHACTR3 | -2,551294263 | -2,621331989 | 0,070037726 | |
| 230883_at | NXPH2 | -2,551294263 | -2,621331989 | 0,070037726 | |
| 231104_at | TDRD5 | -2,551294263 | -2,621331989 | 0,070037726 | |
| 231383_at | - | -2,551294263 | -2,621331989 | 0,070037726 | |
| 234358_at | - | -2,551294263 | -2,621331989 | 0,070037726 | |
| 234474_x_at | IL6ST | -2,551294263 | -2,621331989 | 0,070037726 | |
| 234961_at | - | -2,551294263 | -2,621331989 | 0,070037726 | |
| 236576_at | - | -2,551294263 | -2,621331989 | 0,070037726 | |
| 239594_at | LOC145837 | -2,551294263 | -2,621331989 | 0,070037726 | |
| 240107_at | - | -2,551294263 | -2,621331989 | 0,070037726 | |
| 240158_at | - | -2,551294263 | -2,621331989 | 0,070037726 | |
| 240762_at | - | -2,551294263 | -2,621331989 | 0,070037726 | |
| 241494_at | - | -2,551294263 | -2,621331989 | 0,070037726 | |
| 242054_s_at | SIX3 | -2,551294263 | -2,621331989 | 0,070037726 | |
| 242520_s_at | C1orf228 | -2,551294263 | -2,621331989 | 0,070037726 | |
| 243013_at | - | -2,551294263 | -2,621331989 | 0,070037726 | |
| 218412_s_at | GTF2IRD1 | 1,62481334 | 1,55481039 | 0,07000295 | |
| 1554586_a_at | RHOBTB2 | 0,246434561 | 0,176436073 | 0,069998488 | |

| | | | | | |
|---|---|---|---|---|---|
| 1558334_a_at | C22orf15 | 0,246434561 | 0,176436073 | 0,069998488 | |
| 217192_s_at | PRDM1 | 0,246434561 | 0,176436073 | 0,069998488 | |
| 221251_x_at | INO80B /// INO80 | 0,246434561 | 0,176436073 | 0,069998488 | |
| 236629_at | IBA57 | 0,246434561 | 0,176436073 | 0,069998488 | |
| 209624_s_at | MCCC2 | 2,094906514 | 2,024923328 | 0,069983186 | |
| 1554245_x_at | ARL17A /// ARL1 | 1,247462054 | 1,177496821 | 0,069965233 | |
| 1556582_at | PVR | 1,247462054 | 1,177496821 | 0,069965233 | |
| 213608_s_at | SRRD | 3,62627949 | 3,556324192 | 0,069955298 | |
| 237626_at | - | 1,512180019 | 1,442269454 | 0,069910565 | |
| 223617_x_at | ATAD3B | 3,628024221 | 3,558125564 | 0,069898657 | |
| 1556734_at | - | -0,496991414 | -0,566887459 | 0,069896046 | |
| 1566821_at | LOC100505862 | -0,496991414 | -0,566887459 | 0,069896046 | |
| 206970_at | CNTN2 | -0,496991414 | -0,566887459 | 0,069896046 | |
| 207302_at | SGCG | -0,496991414 | -0,566887459 | 0,069896046 | |
| 207787_at | KRT33B | -0,496991414 | -0,566887459 | 0,069896046 | |
| 214704_at | TCF25 | -0,496991414 | -0,566887459 | 0,069896046 | |
| 243495_s_at | ZNF652 | -0,496991414 | -0,566887459 | 0,069896046 | |
| 225014_at | C4orf52 | 3,655656719 | 3,585762986 | 0,069893733 | |
| 218166_s_at | RSF1 | 1,735773781 | 1,665902186 | 0,069871595 | |
| 222979_s_at | SURF4 | 4,389788927 | 4,319980141 | 0,069808786 | |
| 208097_s_at | TMX1 | 5,149552753 | 5,079744839 | 0,069807914 | |
| 1554303_at | HNMT | -1,353583928 | -1,423348341 | 0,069764413 | |
| 1555678_at | ST3GAL3 | -1,353583928 | -1,423348341 | 0,069764413 | |
| 1556779_s_at | - | -1,353583928 | -1,423348341 | 0,069764413 | |
| 1560154_a_at | - | -1,353583928 | -1,423348341 | 0,069764413 | |
| 1562831_a_at | WDR11-AS1 | -1,353583928 | -1,423348341 | 0,069764413 | |
| 205023_at | RAD51 | -1,353583928 | -1,423348341 | 0,069764413 | |
| 210380_s_at | CACNA1G | -1,353583928 | -1,423348341 | 0,069764413 | |
| 210506_at | FUT7 | -1,353583928 | -1,423348341 | 0,069764413 | |
| 214292_at | ITGB4 | -1,353583928 | -1,423348341 | 0,069764413 | |
| 217020_at | RARB | -1,353583928 | -1,423348341 | 0,069764413 | |
| 230700_at | RTN4RL1 | -1,353583928 | -1,423348341 | 0,069764413 | |
| 233211_at | MTBP | -1,353583928 | -1,423348341 | 0,069764413 | |
| 235430_at | C14orf43 | -1,353583928 | -1,423348341 | 0,069764413 | |
| 236452_at | - | -1,353583928 | -1,423348341 | 0,069764413 | |
| 236687_at | - | -1,353583928 | -1,423348341 | 0,069764413 | |
| 237611_at | - | -1,353583928 | -1,423348341 | 0,069764413 | |
| 242104_at | - | -1,353583928 | -1,423348341 | 0,069764413 | |
| 242941_x_at | TBX1 | -1,353583928 | -1,423348341 | 0,069764413 | |
| 200985_s_at | CD59 | 3,63237684 | 3,562619178 | 0,069757661 | |
| 224130_s_at | SRA1 | 3,102740237 | 3,033010968 | 0,06972927 | |
| 221064_s_at | UNKL | 0,934330293 | 0,864649967 | 0,069680326 | |
| 228480_at | VAPA | 1,394109935 | 1,324440845 | 0,06966909 | |
| 222664_at | KCTD15 | 3,394805732 | 3,325159096 | 0,069646636 | |
| 227964_at | FRMD8 | 3,842326541 | 3,7726819 | 0,069644641 | |
| 208002_s_at | ACOT7 | 3,890364806 | 3,820743792 | 0,069621014 | |
| 212689_s_at | KDM3A | 4,106641205 | 4,037037849 | 0,069603356 | |
| 222633_at | TBL1XR1 | 2,52764011 | 2,458228496 | 0,069411615 | |
| 224675_at | MESDC2 | 4,724639777 | 4,655247494 | 0,069392283 | |
| 218340_s_at | UBA6 | 3,191547726 | 3,122166354 | 0,069381372 | |
| 200044_at | GATC /// SRSF9 | 5,869070493 | 5,799709572 | 0,069360921 | |
| 235037_at | TMEM41A | 3,266269388 | 3,196911138 | 0,06935825 | |
| 201675_at | AKAP1 | 3,115233629 | 3,045906378 | 0,069327251 | |
| 238105_x_at | WNT7B | 0,945567441 | 0,876248481 | 0,06931896 | |
| 202338_at | TK1 | 3,193905507 | 3,124599694 | 0,069305813 | |
| 224784_at | MLLT6 | 1,269985355 | 1,200746069 | 0,069239285 | |

| | | | | | |
|---|---|---|---|---|---|
| 1554755_a_at | MTUS2 | -1,308181863 | -1,377419394 | 0,069237531 | |
| 1555569_a_at | KCTD7 /// RABGI | -1,308181863 | -1,377419394 | 0,069237531 | |
| 1561608_at | - | -1,308181863 | -1,377419394 | 0,069237531 | |
| 1566609_at | - | -1,308181863 | -1,377419394 | 0,069237531 | |
| 207092_at | LEP | -1,308181863 | -1,377419394 | 0,069237531 | |
| 215042_at | BMP6 | -1,308181863 | -1,377419394 | 0,069237531 | |
| 215052_at | FRMPD4 | -1,308181863 | -1,377419394 | 0,069237531 | |
| 215433_at | DPY19L1 | -1,308181863 | -1,377419394 | 0,069237531 | |
| 220626_at | SERPINA10 | -1,308181863 | -1,377419394 | 0,069237531 | |
| 223757_at | DIO3OS | -1,308181863 | -1,377419394 | 0,069237531 | |
| 230249_at | KHDRBS3 | -1,308181863 | -1,377419394 | 0,069237531 | |
| 231286_at | C9orf47 | -1,308181863 | -1,377419394 | 0,069237531 | |
| 233777_at | - | -1,308181863 | -1,377419394 | 0,069237531 | |
| 234153_at | - | -1,308181863 | -1,377419394 | 0,069237531 | |
| 239157_at | ZSCAN12P1 | -1,308181863 | -1,377419394 | 0,069237531 | |
| 240116_at | - | -1,308181863 | -1,377419394 | 0,069237531 | |
| 241050_at | - | -1,308181863 | -1,377419394 | 0,069237531 | |
| 205667_at | WRN | 3,782486091 | 3,713295978 | 0,069190113 | |
| 53968_at | INTS5 | 2,705896113 | 2,636751089 | 0,069145024 | |
| 203311_s_at | ARF6 | 3,536237877 | 3,467101363 | 0,069136514 | |
| 1554559_at | GPR62 | 0,273439642 | 0,204310755 | 0,069128887 | |
| 1568627_at | SMEK2 | 0,273439642 | 0,204310755 | 0,069128887 | |
| 1569150_x_at | PDLIM7 | 0,273439642 | 0,204310755 | 0,069128887 | |
| 221421_s_at | ADAMTS12 | 0,273439642 | 0,204310755 | 0,069128887 | |
| 234554_at | KCNK16 | 0,273439642 | 0,204310755 | 0,069128887 | |
| 218350_s_at | GMNN | 5,878079008 | 5,809005963 | 0,069073045 | |
| 218560_s_at | JMJD4 | 0,760715727 | 0,691645685 | 0,069070041 | |
| 227382_at | CYB5B | 0,760715727 | 0,691645685 | 0,069070041 | |
| 202826_at | SPINT1 | -0,467091869 | -0,536087151 | 0,068995282 | |
| 212822_at | HEG1 | -0,467091869 | -0,536087151 | 0,068995282 | |
| 215232_at | ARHGAP44 | -0,467091869 | -0,536087151 | 0,068995282 | |
| 205273_s_at | PITRM1 | 3,604290432 | 3,535445117 | 0,068845315 | |
| 1553797_a_at | LOC100652811 / | -2,667025278 | -2,735862426 | 0,068837148 | |
| 1557538_at | - | -2,667025278 | -2,735862426 | 0,068837148 | |
| 1557914_s_at | ERVFH21-1 | -2,667025278 | -2,735862426 | 0,068837148 | |
| 1562337_at | OR7D2 | -2,667025278 | -2,735862426 | 0,068837148 | |
| 1562365_at | LOC286177 | -2,667025278 | -2,735862426 | 0,068837148 | |
| 1563128_at | - | -2,667025278 | -2,735862426 | 0,068837148 | |
| 1565546_at | RNF141 | -2,667025278 | -2,735862426 | 0,068837148 | |
| 1565810_at | - | -2,667025278 | -2,735862426 | 0,068837148 | |
| 1570284_x_at | - | -2,667025278 | -2,735862426 | 0,068837148 | |
| 1570422_at | SNTG2 | -2,667025278 | -2,735862426 | 0,068837148 | |
| 202833_s_at | SERPINA1 | -2,667025278 | -2,735862426 | 0,068837148 | |
| 208088_s_at | CFHR5 | -2,667025278 | -2,735862426 | 0,068837148 | |
| 211776_s_at | EPB41L3 | -2,667025278 | -2,735862426 | 0,068837148 | |
| 214932_at | KIDINS220 | -2,667025278 | -2,735862426 | 0,068837148 | |
| 215626_at | - | -2,667025278 | -2,735862426 | 0,068837148 | |
| 220540_at | KCNK15 | -2,667025278 | -2,735862426 | 0,068837148 | |
| 221872_at | RARRES1 | -2,667025278 | -2,735862426 | 0,068837148 | |
| 229695_at | - | -2,667025278 | -2,735862426 | 0,068837148 | |
| 231098_at | - | -2,667025278 | -2,735862426 | 0,068837148 | |
| 232261_at | - | -2,667025278 | -2,735862426 | 0,068837148 | |
| 235746_s_at | PLA2R1 | -2,667025278 | -2,735862426 | 0,068837148 | |
| 240053_x_at | PEX5L | -2,667025278 | -2,735862426 | 0,068837148 | |
| 240088_at | PDE5A | -2,667025278 | -2,735862426 | 0,068837148 | |
| 241026_at | - | -2,667025278 | -2,735862426 | 0,068837148 | |

| | | | | | |
|---|---|---|---|---|---|
| 242643_x_at | - | -2,667025278 | -2,735862426 | 0,068837148 | |
| 235536_at | SNORD89 | 1,283332461 | 1,214517879 | 0,068814582 | |
| 203544_s_at | STAM | 3,423218668 | 3,354479478 | 0,068739191 | |
| 201493_s_at | PUM2 | 4,175069494 | 4,106388091 | 0,068681403 | |
| 1552355_s_at | C19orf26 | -0,033456564 | -0,102100538 | 0,068643973 | |
| 1569426_at | - | -0,033456564 | -0,102100538 | 0,068643973 | |
| 203601_s_at | ZBTB17 | -0,033456564 | -0,102100538 | 0,068643973 | |
| 207892_at | CD40LG | -0,033456564 | -0,102100538 | 0,068643973 | |
| 211232_x_at | GLP1R | -0,033456564 | -0,102100538 | 0,068643973 | |
| 216980_s_at | SPN | -0,033456564 | -0,102100538 | 0,068643973 | |
| 221407_at | GJD2 | -0,033456564 | -0,102100538 | 0,068643973 | |
| 219335_at | ARMCX5 | 1,874520207 | 1,805902329 | 0,068617877 | |
| 202002_at | ACAA2 | 0,291166226 | 0,222599095 | 0,068567131 | |
| 215908_at | - | 0,291166226 | 0,222599095 | 0,068567131 | |
| 224356_x_at | MS4A6A | 0,291166226 | 0,222599095 | 0,068567131 | |
| 239939_at | - | 0,291166226 | 0,222599095 | 0,068567131 | |
| 221764_at | R3HDM4 | 2,923969333 | 2,855418981 | 0,068550353 | |
| 202512_s_at | ATG5 | 1,292162421 | 1,223626587 | 0,068535833 | |
| 220768_s_at | CSNK1G3 | 3,101484927 | 3,033010968 | 0,068473959 | |
| 220547_s_at | FAM35A | 4,972732044 | 4,904274741 | 0,068457303 | |
| 221483_s_at | ARPP19 | 5,154100714 | 5,085689237 | 0,068411478 | |
| 209868_s_at | RBMS1 | 2,061726373 | 1,993317152 | 0,068409221 | |
| 212150_at | EFR3A | 3,647078281 | 3,578681277 | 0,068397004 | |
| 219137_s_at | MFF | 2,782308844 | 2,713921281 | 0,068387563 | |
| 222841_s_at | TIMM22 | 2,882181479 | 2,813804482 | 0,068376996 | |
| 206837_at | ALX1 | 1,67631007 | 1,60795124 | 0,068358831 | |
| 1553622_a_at | FSIP1 | -2,712026959 | -2,780313924 | 0,068286965 | |
| 1553851_at | SPIC | -2,712026959 | -2,780313924 | 0,068286965 | |
| 1554781_at | MGC32805 | -2,712026959 | -2,780313924 | 0,068286965 | |
| 1556666_a_at | LOC100652860 / | -2,712026959 | -2,780313924 | 0,068286965 | |
| 1557778_at | - | -2,712026959 | -2,780313924 | 0,068286965 | |
| 1559077_at | ABI3BP | -2,712026959 | -2,780313924 | 0,068286965 | |
| 1560018_at | ARPP21 | -2,712026959 | -2,780313924 | 0,068286965 | |
| 1560848_at | - | -2,712026959 | -2,780313924 | 0,068286965 | |
| 1561899_at | CLECL1 | -2,712026959 | -2,780313924 | 0,068286965 | |
| 1562670_at | - | -2,712026959 | -2,780313924 | 0,068286965 | |
| 1562879_at | - | -2,712026959 | -2,780313924 | 0,068286965 | |
| 1569675_at | POU2AF1 | -2,712026959 | -2,780313924 | 0,068286965 | |
| 1569942_at | - | -2,712026959 | -2,780313924 | 0,068286965 | |
| 1570039_at | TMSB15A /// TMS | -2,712026959 | -2,780313924 | 0,068286965 | |
| 211412_at | PADI4 | -2,712026959 | -2,780313924 | 0,068286965 | |
| 214615_at | P2RY10 | -2,712026959 | -2,780313924 | 0,068286965 | |
| 215586_at | - | -2,712026959 | -2,780313924 | 0,068286965 | |
| 215911_x_at | ATP2B3 | -2,712026959 | -2,780313924 | 0,068286965 | |
| 216244_at | IL1RN | -2,712026959 | -2,780313924 | 0,068286965 | |
| 219840_s_at | TCL6 | -2,712026959 | -2,780313924 | 0,068286965 | |
| 222951_s_at | ANKRD5 | -2,712026959 | -2,780313924 | 0,068286965 | |
| 224354_at | - | -2,712026959 | -2,780313924 | 0,068286965 | |
| 231490_at | - | -2,712026959 | -2,780313924 | 0,068286965 | |
| 233282_at | - | -2,712026959 | -2,780313924 | 0,068286965 | |
| 235351_at | - | -2,712026959 | -2,780313924 | 0,068286965 | |
| 236297_at | - | -2,712026959 | -2,780313924 | 0,068286965 | |
| 241821_at | - | -2,712026959 | -2,780313924 | 0,068286965 | |
| 243697_at | MOXD1 | -2,712026959 | -2,780313924 | 0,068286965 | |
| 244066_at | - | -2,712026959 | -2,780313924 | 0,068286965 | |
| 244553_at | HOTTIP | -2,712026959 | -2,780313924 | 0,068286965 | |

| | | | | | |
|---|---|---|---|---|---|
| 202782_s_at | INPP5K | 1,567778694 | 1,499637023 | 0,068141671 | |
| 216483_s_at | C19orf10 | 3,232262672 | 3,164168347 | 0,068094324 | |
| 242922_at | NOMO3 | 0,792179257 | 0,724100169 | 0,068079088 | |
| 202164_s_at | CNOT8 | 3,685285822 | 3,617207412 | 0,06807841 | |
| 1559051_s_at | MB21D1 | 1,446464021 | 1,378453643 | 0,068010377 | |
| 208635_x_at | NACA | 7,54900834 | 7,481078695 | 0,067929645 | |
| 200642_at | SOD1 | 7,137176564 | 7,069257481 | 0,067919083 | |
| 215994_x_at | TBC1D9B | 1,575032557 | 1,507116528 | 0,067916029 | |
| 205085_at | ORC1 | 1,990274687 | 1,922365006 | 0,067909681 | |
| 201153_s_at | MBNL1 | 5,200568591 | 5,132684978 | 0,067883614 | |
| 203671_at | TPMT | 1,314003737 | 1,246149888 | 0,067853848 | |
| 218620_s_at | HEMK1 | 1,69307511 | 1,6252386 | 0,06783651 | |
| 201538_s_at | DUSP3 | 0,317354302 | 0,249604176 | 0,067750126 | |
| 203761_at | SLA | -0,423248115 | -0,490927335 | 0,06767922 | |
| 203768_s_at | STS | -0,423248115 | -0,490927335 | 0,06767922 | |
| 206705_at | TULP1 | -0,423248115 | -0,490927335 | 0,06767922 | |
| 210248_at | WNT7A | -0,423248115 | -0,490927335 | 0,06767922 | |
| 211864_s_at | MYOF | -0,423248115 | -0,490927335 | 0,06767922 | |
| 213467_at | RND2 | -0,423248115 | -0,490927335 | 0,06767922 | |
| 217231_s_at | MAST1 | -0,423248115 | -0,490927335 | 0,06767922 | |
| 221016_s_at | TCF7L1 | -0,423248115 | -0,490927335 | 0,06767922 | |
| 221602_s_at | FAIM3 | -0,423248115 | -0,490927335 | 0,06767922 | |
| 222355_at | - | -0,423248115 | -0,490927335 | 0,06767922 | |
| 229276_at | IGSF9 | -0,423248115 | -0,490927335 | 0,06767922 | |
| 229840_at | IQSEC2 | -0,423248115 | -0,490927335 | 0,06767922 | |
| 227373_at | ATXN1L | 2,952099138 | 2,884425449 | 0,067673689 | |
| 1553641_a_at | TSGA13 | -1,196375121 | -1,264005341 | 0,067630219 | |
| 1555852_at | LOC100507463 | -1,196375121 | -1,264005341 | 0,067630219 | |
| 1558631_at | PPARA | -1,196375121 | -1,264005341 | 0,067630219 | |
| 1558773_s_at | RANBP10 | -1,196375121 | -1,264005341 | 0,067630219 | |
| 1561065_at | - | -1,196375121 | -1,264005341 | 0,067630219 | |
| 1563597_at | - | -1,196375121 | -1,264005341 | 0,067630219 | |
| 1564733_at | - | -1,196375121 | -1,264005341 | 0,067630219 | |
| 207135_at | HTR2A | -1,196375121 | -1,264005341 | 0,067630219 | |
| 207755_at | - | -1,196375121 | -1,264005341 | 0,067630219 | |
| 234042_at | TAS2R43 /// TAS | -1,196375121 | -1,264005341 | 0,067630219 | |
| 234649_at | - | -1,196375121 | -1,264005341 | 0,067630219 | |
| 236828_at | - | -1,196375121 | -1,264005341 | 0,067630219 | |
| 237155_at | LOC100506502 | -1,196375121 | -1,264005341 | 0,067630219 | |
| 239597_at | - | -1,196375121 | -1,264005341 | 0,067630219 | |
| 240119_at | TEPP | -1,196375121 | -1,264005341 | 0,067630219 | |
| 242246_x_at | MEG3 | -1,196375121 | -1,264005341 | 0,067630219 | |
| 210973_s_at | FGFR1 | 1,000476627 | 0,932882274 | 0,067594353 | |
| 221896_s_at | HIGD1A | 5,772203495 | 5,704616451 | 0,067587043 | |
| 206494_s_at | ITGA2B | 1,58584542 | 1,518263577 | 0,067581843 | |
| 206397_x_at | CERS1 /// GDF1 | 2,171078719 | 2,103505212 | 0,067573507 | |
| 1554301_at | LHFPL3-AS1 | -2,779459409 | -2,847001814 | 0,067542406 | |
| 1556954_at | LOC283854 | -2,779459409 | -2,847001814 | 0,067542406 | |
| 1559333_at | SRGAP3-AS2 | -2,779459409 | -2,847001814 | 0,067542406 | |
| 1569241_a_at | ZNF93 | -2,779459409 | -2,847001814 | 0,067542406 | |
| 1570021_at | - | -2,779459409 | -2,847001814 | 0,067542406 | |
| 206378_at | SCGB2A2 | -2,779459409 | -2,847001814 | 0,067542406 | |
| 219687_at | HHAT | -2,779459409 | -2,847001814 | 0,067542406 | |
| 222379_at | KCNE4 | -2,779459409 | -2,847001814 | 0,067542406 | |
| 222950_at | NIPAL2 | -2,779459409 | -2,847001814 | 0,067542406 | |
| 223618_at | FMN2 | -2,779459409 | -2,847001814 | 0,067542406 | |

| | | | | | |
|---|---|---|---|---|---|
| 228441_s_at | - | -2,779459409 | -2,847001814 | 0,067542406 | |
| 232817_at | - | -2,779459409 | -2,847001814 | 0,067542406 | |
| 234420_at | PIH2 | -2,779459409 | -2,847001814 | 0,067542406 | |
| 235481_at | COMMD10 | -2,779459409 | -2,847001814 | 0,067542406 | |
| 235489_at | RHOJ | -2,779459409 | -2,847001814 | 0,067542406 | |
| 241271_at | - | -2,779459409 | -2,847001814 | 0,067542406 | |
| 242703_at | - | -2,779459409 | -2,847001814 | 0,067542406 | |
| 243424_at | - | -2,779459409 | -2,847001814 | 0,067542406 | |
| 223037_at | PDZD11 | 3,702775533 | 3,635238397 | 0,067537136 | |
| 202464_s_at | PFKFB3 | 3,251631457 | 3,184136378 | 0,067495079 | |
| 226580_at | BRMS1L | 2,64946283 | 2,582026171 | 0,067436658 | |
| 213588_x_at | RPL14 | 7,549382416 | 7,481968578 | 0,067413838 | |
| 217948_at | FAM127B | 1,46610492 | 1,398700978 | 0,067403942 | |
| 209257_s_at | SMC3 | 1,709647566 | 1,642321262 | 0,067326303 | |
| 211139_s_at | NAB1 | 1,709647566 | 1,642321262 | 0,067326303 | |
| 226329_s_at | MITD1 | 3,964046478 | 3,896739828 | 0,06730665 | |
| 209180_at | RABGGTB | 4,05440026 | 3,987098381 | 0,067301879 | |
| 34221_at | HMGXB3 | 2,436992838 | 2,369713036 | 0,067279802 | |
| 242073_at | - | 1,917954284 | 1,85068474 | 0,067269544 | |
| 223649_s_at | SLC25A39 | 3,220746604 | 3,153484076 | 0,067262528 | |
| 219365_s_at | CAMKV | 0,334552598 | 0,26733076 | 0,067221839 | |
| 224281_s_at | NGRN | 3,142344098 | 3,075135559 | 0,067208539 | |
| 224410_s_at | LMBR1 | 3,472604685 | 3,405399563 | 0,067205121 | |
| 214544_s_at | SNAP23 | 3,657366303 | 3,590171466 | 0,067194837 | |
| 218711_s_at | SDPR | 3,506195778 | 3,439026336 | 0,067169442 | |
| 209653_at | KPNA4 | 2,342392905 | 2,275409734 | 0,066983171 | |
| 1562263_at | LOC100507156 | 0,020670649 | -0,046302147 | 0,066972796 | |
| 210241_s_at | TP53TG1 | 0,020670649 | -0,046302147 | 0,066972796 | |
| 218416_s_at | SLC48A1 | 0,020670649 | -0,046302147 | 0,066972796 | |
| 240837_at | FNDC7 | 0,020670649 | -0,046302147 | 0,066972796 | |
| 1555244_at | LOC554207 | -2,823166348 | -2,89013181 | 0,066965462 | |
| 1555356_a_at | SCML4 | -2,823166348 | -2,89013181 | 0,066965462 | |
| 1557133_at | RP1-177G6.2 | -2,823166348 | -2,89013181 | 0,066965462 | |
| 1561200_at | VWA3B | -2,823166348 | -2,89013181 | 0,066965462 | |
| 1562193_at | - | -2,823166348 | -2,89013181 | 0,066965462 | |
| 1562398_at | - | -2,823166348 | -2,89013181 | 0,066965462 | |
| 1563899_at | LCTL | -2,823166348 | -2,89013181 | 0,066965462 | |
| 1565837_at | - | -2,823166348 | -2,89013181 | 0,066965462 | |
| 1569323_at | PTPRG | -2,823166348 | -2,89013181 | 0,066965462 | |
| 1569831_at | - | -2,823166348 | -2,89013181 | 0,066965462 | |
| 205767_at | EREG | -2,823166348 | -2,89013181 | 0,066965462 | |
| 207638_at | TMPRSS15 | -2,823166348 | -2,89013181 | 0,066965462 | |
| 214942_at | RBM34 | -2,823166348 | -2,89013181 | 0,066965462 | |
| 233828_at | - | -2,823166348 | -2,89013181 | 0,066965462 | |
| 237597_at | LOC100507525 | -2,823166348 | -2,89013181 | 0,066965462 | |
| 237802_at | XKR4 | -2,823166348 | -2,89013181 | 0,066965462 | |
| 237911_at | - | -2,823166348 | -2,89013181 | 0,066965462 | |
| 238073_at | ELAVL4 | -2,823166348 | -2,89013181 | 0,066965462 | |
| 240386_at | - | -2,823166348 | -2,89013181 | 0,066965462 | |
| 241590_at | - | -2,823166348 | -2,89013181 | 0,066965462 | |
| 244173_at | MIS18BP1 | -2,823166348 | -2,89013181 | 0,066965462 | |
| 217933_s_at | LAP3 | 4,977187694 | 4,91028116 | 0,066906535 | |
| 212341_at | YIPF6 | 4,328338611 | 4,261479522 | 0,066859089 | |
| 217770_at | PIGT | 3,609598452 | 3,542741726 | 0,066856726 | |
| 1553239_at | FAM124A | -0,394749123 | -0,461594427 | 0,066845303 | |
| 204149_s_at | GSTM4 | -0,394749123 | -0,461594427 | 0,066845303 | |

| | | | | | |
|---|---|---|---|---|---|
| 208333_at | LHX5 | -0,394749123 | -0,461594427 | 0,066845303 | |
| 210677_at | SOAT2 | -0,394749123 | -0,461594427 | 0,066845303 | |
| 223832_s_at | CAPNS2 | -0,394749123 | -0,461594427 | 0,066845303 | |
| 230245_s_at | LOC283663 | -0,394749123 | -0,461594427 | 0,066845303 | |
| 234356_at | DKFZP434K028 | -0,394749123 | -0,461594427 | 0,066845303 | |
| 235756_at | - | -0,394749123 | -0,461594427 | 0,066845303 | |
| 209455_at | FBXW11 | 3,234554895 | 3,16771226 | 0,066842635 | |
| 225028_at | LOC550643 | 1,726031811 | 1,659204017 | 0,066827794 | |
| 202171_at | VEZF1 | 2,669932407 | 2,603119255 | 0,066813152 | |
| 221223_x_at | CISH | 1,027166904 | 0,960386719 | 0,066780185 | |
| 222511_x_at | FAF1 | 1,027166904 | 0,960386719 | 0,066780185 | |
| 202696_at | OXSR1 | 3,700289946 | 3,633530837 | 0,06675911 | |
| 46167_at | HEATR8 /// HEAT | 3,910933598 | 3,84418735 | 0,066746248 | |
| 210453_x_at | ATP5L | 6,826988486 | 6,760273516 | 0,06671497 | |
| 216883_x_at | PDE6D | 3,316904202 | 3,250254796 | 0,066649405 | |
| 204372_s_at | KHSRP | 4,372199749 | 4,305575871 | 0,066623878 | |
| 1557905_s_at | CD44 | 2,11992033 | 2,053299695 | 0,066620634 | |
| 200959_at | FUS | 5,354716535 | 5,288105861 | 0,066610674 | |
| 200876_s_at | PSMB1 | 6,923471608 | 6,856919273 | 0,066552335 | |
| 232309_at | LOC202181 | 0,359971644 | 0,293518836 | 0,066452808 | |
| 235949_at | TTC26 | 0,359971644 | 0,293518836 | 0,066452808 | |
| 201425_at | ALDH2 | 1,210676994 | 1,144305291 | 0,066371703 | |
| 216985_s_at | STX3 | 1,210676994 | 1,144305291 | 0,066371703 | |
| 224779_s_at | FAM96A | 5,47349744 | 5,40731921 | 0,06617823 | |
| 228014_at | PTRH1 | 1,369290453 | 1,303116084 | 0,066174368 | |
| 224522_s_at | DCAKD | 1,954567604 | 1,888402606 | 0,066164998 | |
| 221437_s_at | MRPS15 | 3,36999832 | 3,303845028 | 0,066153292 | |
| 212160_at | XPOT | 4,618940011 | 4,552816898 | 0,066123114 | |
| 201172_x_at | ATP6V0E1 | 5,209918231 | 5,143823736 | 0,066094494 | |
| 201757_at | NDUFS5 | 6,862436922 | 6,79634875 | 0,066088171 | |
| 227307_at | TSPAN18 | 1,751865623 | 1,685812099 | 0,066053523 | |
| 1555238_at | PTH2 | 1,05337236 | 0,987376598 | 0,065995762 | |
| 202991_at | STARD3 | 1,05337236 | 0,987376598 | 0,065995762 | |
| 223240_at | FBXO8 | 1,515954005 | 1,450051551 | 0,065902454 | |
| 200024_at | RPS5 | 7,883095607 | 7,817193884 | 0,065901723 | |
| 1552854_a_at | VWA5B1 | -1,089016378 | -1,154897679 | 0,065881301 | |
| 1559759_at | KIFC3 | -1,089016378 | -1,154897679 | 0,065881301 | |
| 1563042_at | LOC338694 | -1,089016378 | -1,154897679 | 0,065881301 | |
| 202454_s_at | ERBB3 | -1,089016378 | -1,154897679 | 0,065881301 | |
| 206724_at | CBX4 | -1,089016378 | -1,154897679 | 0,065881301 | |
| 215623_x_at | SMC4 | -1,089016378 | -1,154897679 | 0,065881301 | |
| 225355_at | NEURL1B | -1,089016378 | -1,154897679 | 0,065881301 | |
| 227130_s_at | TLE1 | -1,089016378 | -1,154897679 | 0,065881301 | |
| 228887_x_at | CCDC61 | -1,089016378 | -1,154897679 | 0,065881301 | |
| 231640_at | - | -1,089016378 | -1,154897679 | 0,065881301 | |
| 232009_at | EMR2 | -1,089016378 | -1,154897679 | 0,065881301 | |
| 233044_at | - | -1,089016378 | -1,154897679 | 0,065881301 | |
| 234157_at | - | -1,089016378 | -1,154897679 | 0,065881301 | |
| 234552_at | EPPK1 | -1,089016378 | -1,154897679 | 0,065881301 | |
| 236231_at | ZNF271 | -1,089016378 | -1,154897679 | 0,065881301 | |
| 238946_at | - | -1,089016378 | -1,154897679 | 0,065881301 | |
| 239046_at | - | -1,089016378 | -1,154897679 | 0,065881301 | |
| 239534_at | - | -1,089016378 | -1,154897679 | 0,065881301 | |
| 239773_at | GTPBP10 | -1,089016378 | -1,154897679 | 0,065881301 | |
| 239964_at | TCL6 | -1,089016378 | -1,154897679 | 0,065881301 | |
| 240243_at | - | -1,089016378 | -1,154897679 | 0,065881301 | |

| | | | | | |
|---|---|---|---|---|---|
| 216241_s_at | TCEA1 | 6,439155183 | 6,373284962 | 0,065870221 | |
| 201404_x_at | PSMB2 | 1,965648824 | 1,899812471 | 0,065836353 | |
| 213358_at | SOGA2 | 1,381753566 | 1,315948728 | 0,065804838 | |
| 227262_at | HAPLN3 | 1,381753566 | 1,315948728 | 0,065804838 | |
| 228809_at | CXorf40A /// CXo | 1,381753566 | 1,315948728 | 0,065804838 | |
| 237188_x_at | SUN5 | 1,381753566 | 1,315948728 | 0,065804838 | |
| 225647_s_at | CTSC | 3,48613512 | 3,420331607 | 0,065803513 | |
| 225082_at | CPSF3 | 4,418803905 | 4,353041151 | 0,065762754 | |
| 207243_s_at | CALM1 /// CALM: | 7,092224248 | 7,026551816 | 0,065672432 | |
| 226243_at | PTRHD1 | 4,524682985 | 4,459038 | 0,065644985 | |
| 201972_at | ATP6V1A | 4,752099177 | 4,686464402 | 0,065634775 | |
| 1556034_s_at | MTMR11 | -0,352951799 | -0,41858459 | 0,065632791 | |
| 216061_x_at | PDGFB | -0,352951799 | -0,41858459 | 0,065632791 | |
| 216493_s_at | IGF2BP3 | -0,352951799 | -0,41858459 | 0,065632791 | |
| 237350_at | TTC36 | -0,352951799 | -0,41858459 | 0,065632791 | |
| 241974_at | - | -0,352951799 | -0,41858459 | 0,065632791 | |
| 204092_s_at | AURKA | 5,043076031 | 4,97744706 | 0,065628971 | |
| 212229_s_at | FBXO21 | 2,314494121 | 2,248887791 | 0,06560633 | |
| 1556096_s_at | UNC13C | -2,930741289 | -2,996335703 | 0,065594414 | |
| 1557895_at | FLJ35934 | -2,930741289 | -2,996335703 | 0,065594414 | |
| 1562491_at | - | -2,930741289 | -2,996335703 | 0,065594414 | |
| 1563465_at | PKD1L1 | -2,930741289 | -2,996335703 | 0,065594414 | |
| 1564707_x_at | GLS2 | -2,930741289 | -2,996335703 | 0,065594414 | |
| 1565873_at | - | -2,930741289 | -2,996335703 | 0,065594414 | |
| 1566428_at | - | -2,930741289 | -2,996335703 | 0,065594414 | |
| 1569783_at | LRRD1 | -2,930741289 | -2,996335703 | 0,065594414 | |
| 1570023_at | - | -2,930741289 | -2,996335703 | 0,065594414 | |
| 1570141_at | MYO5B | -2,930741289 | -2,996335703 | 0,065594414 | |
| 203170_at | RRP8 | -2,930741289 | -2,996335703 | 0,065594414 | |
| 207875_at | - | -2,930741289 | -2,996335703 | 0,065594414 | |
| 209348_s_at | MAF | -2,930741289 | -2,996335703 | 0,065594414 | |
| 211149_at | UTY | -2,930741289 | -2,996335703 | 0,065594414 | |
| 216030_s_at | SEMG2 | -2,930741289 | -2,996335703 | 0,065594414 | |
| 216731_s_at | - | -2,930741289 | -2,996335703 | 0,065594414 | |
| 221153_s_at | - | -2,930741289 | -2,996335703 | 0,065594414 | |
| 221933_at | NLGN4X | -2,930741289 | -2,996335703 | 0,065594414 | |
| 223863_at | WDR26 | -2,930741289 | -2,996335703 | 0,065594414 | |
| 232383_at | TFEC | -2,930741289 | -2,996335703 | 0,065594414 | |
| 233372_at | - | -2,930741289 | -2,996335703 | 0,065594414 | |
| 233744_at | - | -2,930741289 | -2,996335703 | 0,065594414 | |
| 237225_at | ZFY-AS1 | -2,930741289 | -2,996335703 | 0,065594414 | |
| 237649_at | COPS4 | -2,930741289 | -2,996335703 | 0,065594414 | |
| 240204_at | LOC100506948 / | -2,930741289 | -2,996335703 | 0,065594414 | |
| 241264_at | - | -2,930741289 | -2,996335703 | 0,065594414 | |
| 203257_s_at | C11orf49 | 1,973904239 | 1,908311039 | 0,065593199 | |
| 222765_x_at | ESF1 | 1,973904239 | 1,908311039 | 0,065593199 | |
| 228301_x_at | NDUFB10 | 4,133297997 | 4,067710529 | 0,065587468 | |
| 201076_at | NHP2L1 | 4,196980309 | 4,131407181 | 0,065573127 | |
| 231925_at | - | 3,952952896 | 3,887456383 | 0,065496513 | |
| 226833_at | CYB5D1 | 2,59519831 | 2,529740643 | 0,065457667 | |
| 242077_x_at | MB21D1 | 0,072842263 | 0,007421914 | 0,065420349 | |
| 208716_s_at | TMCO1 | 5,34068725 | 5,275295809 | 0,065391442 | |
| 201180_s_at | GNAI3 | 5,682492619 | 5,617138029 | 0,06535459 | |
| 226943_at | C12orf73 | 2,886560922 | 2,821324225 | 0,065236697 | |
| 212656_at | TSFM | 3,807337009 | 3,742106442 | 0,065230567 | |
| 1556056_at | SIK2 | 0,401366113 | 0,336136178 | 0,065229935 | |

| | | | | | |
|---|---|---|---|---|---|
| 230807_at | CCDC151 | 0,401366113 | 0,336136178 | 0,065229935 | |
| 231808_at | OIP5-AS1 | 3,123915073 | 3,058687544 | 0,065227529 | |
| 222615_s_at | LOC100630923 / | 1,40228911 | 1,337086025 | 0,065203085 | |
| 1556899_at | PRMT5-AS1 | -1,047650486 | -1,112851844 | 0,065201358 | |
| 1558712_at | TMEM134 | -1,047650486 | -1,112851844 | 0,065201358 | |
| 1566157_x_at | - | -1,047650486 | -1,112851844 | 0,065201358 | |
| 203398_s_at | GALNT3 | -1,047650486 | -1,112851844 | 0,065201358 | |
| 207183_at | GPR19 | -1,047650486 | -1,112851844 | 0,065201358 | |
| 210742_at | CDC14A | -1,047650486 | -1,112851844 | 0,065201358 | |
| 217217_at | IGHA1 /// IGHA2 | -1,047650486 | -1,112851844 | 0,065201358 | |
| 220711_at | - | -1,047650486 | -1,112851844 | 0,065201358 | |
| 238320_at | LOC100653017 / | -1,047650486 | -1,112851844 | 0,065201358 | |
| 242736_at | - | -1,047650486 | -1,112851844 | 0,065201358 | |
| 201038_s_at | ANP32A | 4,105389286 | 4,040263518 | 0,065125768 | |
| 53076_at | B4GALT7 | 0,891393815 | 0,826297576 | 0,065096239 | |
| 236192_at | HOOK3 | 2,084778276 | 2,019703483 | 0,065074794 | |
| 204514_at | DPH2 | 2,406821352 | 2,341749893 | 0,06507146 | |
| 1553646_at | HDX | -2,972500237 | -3,037500868 | 0,065000632 | |
| 1555778_a_at | POSTN | -2,972500237 | -3,037500868 | 0,065000632 | |
| 1557181_s_at | C11orf87 | -2,972500237 | -3,037500868 | 0,065000632 | |
| 1557472_a_at | FLJ30838 | -2,972500237 | -3,037500868 | 0,065000632 | |
| 1559433_at | LOC149773 | -2,972500237 | -3,037500868 | 0,065000632 | |
| 1562270_at | ARHGEF7 | -2,972500237 | -3,037500868 | 0,065000632 | |
| 201110_s_at | THBS1 | -2,972500237 | -3,037500868 | 0,065000632 | |
| 207406_at | CYP7A1 | -2,972500237 | -3,037500868 | 0,065000632 | |
| 216659_at | LOC1720 | -2,972500237 | -3,037500868 | 0,065000632 | |
| 217489_s_at | IL6R | -2,972500237 | -3,037500868 | 0,065000632 | |
| 224226_at | PKD2L2 | -2,972500237 | -3,037500868 | 0,065000632 | |
| 232368_at | BET3L | -2,972500237 | -3,037500868 | 0,065000632 | |
| 234242_at | - | -2,972500237 | -3,037500868 | 0,065000632 | |
| 234641_at | - | -2,972500237 | -3,037500868 | 0,065000632 | |
| 238906_s_at | RHOJ | -2,972500237 | -3,037500868 | 0,065000632 | |
| 239655_at | - | -2,972500237 | -3,037500868 | 0,065000632 | |
| 240112_at | - | -2,972500237 | -3,037500868 | 0,065000632 | |
| 240914_at | - | -2,972500237 | -3,037500868 | 0,065000632 | |
| 244742_at | PAH | -2,972500237 | -3,037500868 | 0,065000632 | |
| 217774_s_at | TRMT112 | 6,050612827 | 5,985623155 | 0,064989673 | |
| 218554_s_at | ASH1L | -0,325749314 | -0,39061235 | 0,064863036 | |
| 220120_s_at | EPB41L4A | -0,325749314 | -0,39061235 | 0,064863036 | |
| 229607_at | LOC100652912 | -0,325749314 | -0,39061235 | 0,064863036 | |
| 230402_at | DUSP15 | -0,325749314 | -0,39061235 | 0,064863036 | |
| 230667_at | FAM204A | -0,325749314 | -0,39061235 | 0,064863036 | |
| 231824_at | LARP1B | -0,325749314 | -0,39061235 | 0,064863036 | |
| 241091_at | - | -0,325749314 | -0,39061235 | 0,064863036 | |
| 225825_at | C20orf194 | 0,900083947 | 0,835239052 | 0,064844895 | |
| 202325_s_at | ATP5J | 6,132787907 | 6,067975866 | 0,064812041 | |
| 230840_at | SMIM1 | 1,094335495 | 1,029536893 | 0,064798602 | |
| 238092_at | LRFN4 | 1,094335495 | 1,029536893 | 0,064798602 | |
| 211257_x_at | ZNF638 | 4,244953702 | 4,180180181 | 0,064773522 | |
| 1568853_at | - | 0,417596971 | 0,352836757 | 0,064760213 | |
| 202572_s_at | DLGAP4 | 0,417596971 | 0,352836757 | 0,064760213 | |
| 209770_at | BTN3A1 | 0,417596971 | 0,352836757 | 0,064760213 | |
| 227127_at | MUSTN1 /// TME | 0,417596971 | 0,352836757 | 0,064760213 | |
| 229489_at | - | 0,417596971 | 0,352836757 | 0,064760213 | |
| 242097_at | - | 0,417596971 | 0,352836757 | 0,064760213 | |
| 218320_s_at | NDUFB11 | 6,064530481 | 5,999775868 | 0,064754613 | |

| | | | | | |
|---|---|---|---|---|---|
| 227517_s_at | GAS5 /// SNORD | 4,589693159 | 4,524995365 | 0,064697795 | |
| 218136_s_at | SLC25A37 | 3,525045272 | 3,460374487 | 0,064670786 | |
| 204114_at | NID2 | 3,143564369 | 3,078904771 | 0,064659598 | |
| 222481_at | FXC1 | 2,68509622 | 2,620464265 | 0,064631955 | |
| 242621_at | ZNF498 | 1,422536444 | 1,3579181 | 0,064618344 | |
| 212631_at | STX7 | 2,908260915 | 2,843651104 | 0,064609811 | |
| 212665_at | TIPARP | 2,493593689 | 2,428994491 | 0,064599198 | |
| 202544_at | GMFB | 5,145904033 | 5,08131153 | 0,064592503 | |
| 238197_at | GATA5 | 2,009141686 | 1,944570415 | 0,064571271 | |
| 215501_s_at | DUSP10 | 2,960432227 | 2,895866687 | 0,06456554 | |
| 226230_at | SMEK2 | 2,497416546 | 2,432926755 | 0,064489791 | |
| 213366_x_at | ATP5C1 | 6,100791503 | 6,036339961 | 0,064451542 | |
| 202012_s_at | EXT2 | 3,316904202 | 3,252481567 | 0,064422635 | |
| 209095_at | DLD | 5,698044848 | 5,6336758 | 0,064369048 | |
| 218888_s_at | NETO2 | 3,638448528 | 3,574237491 | 0,064211037 | |
| 222408_s_at | YPEL5 | 3,508091848 | 3,443906002 | 0,064185847 | |
| 204352_at | TRAF5 | 2,640847372 | 2,576704359 | 0,064143013 | |
| 1560115_a_at | KIAA1217 | 0,702292136 | 0,638150376 | 0,06414176 | |
| 206085_s_at | CTH | 0,702292136 | 0,638150376 | 0,06414176 | |
| 216473_x_at | DUX2 /// DUX4 // | 0,702292136 | 0,638150376 | 0,06414176 | |
| 215160_x_at | LOC100289097 | 3,288503211 | 3,22439681 | 0,064106401 | |
| 235374_at | MDH1 | 0,44160593 | 0,377530647 | 0,064075283 | |
| 243780_at | - | 0,44160593 | 0,377530647 | 0,064075283 | |
| 1555157_at | - | -0,986356909 | -1,050406615 | 0,064049706 | |
| 202403_s_at | COL1A2 | -0,986356909 | -1,050406615 | 0,064049706 | |
| 207967_at | VPS45 | -0,986356909 | -1,050406615 | 0,064049706 | |
| 221670_s_at | LHX3 | -0,986356909 | -1,050406615 | 0,064049706 | |
| 228058_at | ZG16B | -0,986356909 | -1,050406615 | 0,064049706 | |
| 237591_at | LINC00173 | -0,986356909 | -1,050406615 | 0,064049706 | |
| 238971_at | - | -0,986356909 | -1,050406615 | 0,064049706 | |
| 241743_at | - | -0,986356909 | -1,050406615 | 0,064049706 | |
| 218482_at | ENY2 | 6,66562745 | 6,60158002 | 0,06404743 | |
| 205246_at | PEX13 | 1,706348269 | 1,642321262 | 0,064027007 | |
| 1563549_a_at | ANO8 | 0,123193849 | 0,059218869 | 0,063974981 | |
| 228727_at | ANXA11 | 0,123193849 | 0,059218869 | 0,063974981 | |
| 236507_at | ZDHHC3 | 0,123193849 | 0,059218869 | 0,063974981 | |
| 239212_at | LTV1 | 0,123193849 | 0,059218869 | 0,063974981 | |
| 236715_x_at | UACA | 2,030406107 | 1,966439221 | 0,063966887 | |
| 218288_s_at | CCDC90B | 3,933331467 | 3,869433939 | 0,063897528 | |
| 204026_s_at | ZWINT | 4,961359357 | 4,897531959 | 0,063827398 | |
| 1556129_at | LOC642533 | 1,129248302 | 1,065442793 | 0,063805509 | |
| 235181_at | TYW5 | 1,129248302 | 1,065442793 | 0,063805509 | |
| 226416_at | ERI1 | 3,857278636 | 3,793476213 | 0,063802422 | |
| 200089_s_at | RPL4 /// SNORD: | 7,093171894 | 7,029395242 | 0,063776652 | |
| 222148_s_at | RHOT1 | 3,745192233 | 3,681421378 | 0,063770856 | |
| 213379_at | COQ2 | 2,452829957 | 2,389070873 | 0,063759084 | |
| 222363_at | - | -0,285837228 | -0,34958478 | 0,063747552 | |
| 237062_at | - | -0,285837228 | -0,34958478 | 0,063747552 | |
| 237766_at | ATP9B | -0,285837228 | -0,34958478 | 0,063747552 | |
| 218026_at | CCDC56 | 4,671066265 | 4,607335387 | 0,063730877 | |
| 219960_s_at | UCHL5 | 4,472714532 | 4,409010481 | 0,063704051 | |
| 203952_at | ATF6 | 2,658027143 | 2,594367918 | 0,063659226 | |
| 203471_s_at | PLEK | 0,457392764 | 0,393761504 | 0,06363126 | |
| 1554678_s_at | HNRPDL | 4,891173693 | 4,827565401 | 0,063608292 | |
| 1552858_at | MAGEB6 | -3,07502285 | -3,138626879 | 0,063604029 | |
| 1553344_at | PCDH15 | -3,07502285 | -3,138626879 | 0,063604029 | |

| | | | | | |
|---|---|---|---|---|---|
| 1560086_at | - | -3,07502285 | -3,138626879 | 0,063604029 | |
| 1560182_at | - | -3,07502285 | -3,138626879 | 0,063604029 | |
| 1565876_x_at | - | -3,07502285 | -3,138626879 | 0,063604029 | |
| 1568644_at | ZNF208 | -3,07502285 | -3,138626879 | 0,063604029 | |
| 1568866_at | - | -3,07502285 | -3,138626879 | 0,063604029 | |
| 210073_at | ST8SIA1 | -3,07502285 | -3,138626879 | 0,063604029 | |
| 233502_at | - | -3,07502285 | -3,138626879 | 0,063604029 | |
| 234022_at | - | -3,07502285 | -3,138626879 | 0,063604029 | |
| 239443_at | PCDHB6 | -3,07502285 | -3,138626879 | 0,063604029 | |
| 240027_at | LIN7A | -3,07502285 | -3,138626879 | 0,063604029 | |
| 241510_at | PLSCR2 | -3,07502285 | -3,138626879 | 0,063604029 | |
| 242198_at | - | -3,07502285 | -3,138626879 | 0,063604029 | |
| 221791_s_at | TMA7 | 7,348425445 | 7,284892157 | 0,063533288 | |
| 202797_at | SACM1L | 4,117238882 | 4,053733117 | 0,063505765 | |
| 214085_x_at | GLIPR1 | 2,048758794 | 1,98530622 | 0,063452574 | |
| 219081_at | ANKHD1 /// ANKI | 2,666540916 | 2,603119255 | 0,063421661 | |
| 208670_s_at | EID1 | 3,843828743 | 3,780418488 | 0,063410256 | |
| 201306_s_at | ANP32B | 7,174889267 | 7,111502174 | 0,063387093 | |
| 202272_s_at | FBXO28 | 4,002215714 | 3,938828971 | 0,063386743 | |
| 202960_s_at | MUT | 3,571118904 | 3,507749374 | 0,06336953 | |
| 210869_s_at | MCAM | 5,076043943 | 5,01268698 | 0,063356962 | |
| 200020_at | TARDBP | 5,179010189 | 5,115661018 | 0,063349171 | |
| 1556662_at | LOC100506142 | -0,946847173 | -1,010192375 | 0,063345202 | |
| 1557615_a_at | SLIT1 | -0,946847173 | -1,010192375 | 0,063345202 | |
| 202687_s_at | TNFSF10 | -0,946847173 | -1,010192375 | 0,063345202 | |
| 203770_s_at | STS | -0,946847173 | -1,010192375 | 0,063345202 | |
| 209866_s_at | LPHN3 | -0,946847173 | -1,010192375 | 0,063345202 | |
| 229910_at | SHE | -0,946847173 | -1,010192375 | 0,063345202 | |
| 236581_at | - | -0,946847173 | -1,010192375 | 0,063345202 | |
| 240317_at | PCDHB4 | -0,946847173 | -1,010192375 | 0,063345202 | |
| 45297_at | EHD2 | 1,05337236 | 0,990048021 | 0,063324339 | |
| 225198_at | VAPA | 4,317026567 | 4,253719424 | 0,063307143 | |
| 214930_at | SLITRK5 | 1,607231019 | 1,543943227 | 0,063287792 | |
| 226998_at | NAA15 | 3,277429131 | 3,21415095 | 0,063278181 | |
| 215329_s_at | CDK11A /// CDK1 | 0,95671774 | 0,893473268 | 0,063244472 | |
| 244455_at | KCNT2 | 0,95671774 | 0,893473268 | 0,063244472 | |
| 205622_at | SMPD2 | 0,735041319 | 0,67181667 | 0,063224649 | |
| 228753_at | LOC100128737 | 1,473887017 | 1,410714371 | 0,063172645 | |
| 204842_x_at | PRKAR2A | 3,32445605 | 3,261354437 | 0,063101613 | |
| 218636_s_at | MAN1B1 | 2,061726373 | 1,998633167 | 0,063093206 | |
| 222035_s_at | PAPOLA | 5,648514173 | 5,585471285 | 0,063042888 | |
| 219593_at | SLC15A3 | -0,259832361 | -0,322870281 | 0,06303792 | |
| 227985_at | LOC100506098 | -0,259832361 | -0,322870281 | 0,06303792 | |
| 231447_at | DZANK1-AS1 | -0,259832361 | -0,322870281 | 0,06303792 | |
| 239531_at | - | -0,259832361 | -0,322870281 | 0,06303792 | |
| 244827_at | - | -0,259832361 | -0,322870281 | 0,06303792 | |
| 1554764_a_at | LINC00301 | -3,114791413 | -3,177802286 | 0,063010873 | |
| 1558519_at | KANSL1L | -3,114791413 | -3,177802286 | 0,063010873 | |
| 1562396_at | - | -3,114791413 | -3,177802286 | 0,063010873 | |
| 1563704_at | - | -3,114791413 | -3,177802286 | 0,063010873 | |
| 215516_at | LAMB4 | -3,114791413 | -3,177802286 | 0,063010873 | |
| 224362_at | LOC100128922 | -3,114791413 | -3,177802286 | 0,063010873 | |
| 228071_at | GIMAP7 | -3,114791413 | -3,177802286 | 0,063010873 | |
| 233025_at | PDZD2 | -3,114791413 | -3,177802286 | 0,063010873 | |
| 242622_x_at | PTEN | -3,114791413 | -3,177802286 | 0,063010873 | |
| 205565_s_at | FXN | 2,965960999 | 2,902971658 | 0,062989342 | |

| | | | | | |
|---|---|---|---|---|---|
| 235067_at | MKLN1 | 2,802535172 | 2,739547836 | 0,062987336 | |
| 204952_at | LYPD3 | 0,480753764 | 0,417770464 | 0,0629833 | |
| 205189_s_at | FANCC | 0,480753764 | 0,417770464 | 0,0629833 | |
| 230762_at | - | 0,480753764 | 0,417770464 | 0,0629833 | |
| 221196_x_at | BRCC3 | 1,859746653 | 1,796776588 | 0,062970065 | |
| 37005_at | C1orf151-NBL1 /// | 1,481627368 | 1,418668082 | 0,062959286 | |
| 200926_at | RPS23 | 8,077205192 | 8,014270097 | 0,062935095 | |
| 222473_s_at | ERBB2IP | 2,55541096 | 2,492544876 | 0,062866084 | |
| 206846_s_at | HDAC6 | 3,209137871 | 3,146317015 | 0,062820857 | |
| 225972_at | TMEM64 | 2,074578432 | 2,011838132 | 0,0627403 | |
| 205255_x_at | TCF7 | 1,168140757 | 1,105412836 | 0,062727922 | |
| 211027_s_at | IKBKB | 0,171847695 | 0,109221392 | 0,062626303 | |
| 219963_at | DUSP13 | 0,171847695 | 0,109221392 | 0,062626303 | |
| 230191_at | TTBK1 | 0,171847695 | 0,109221392 | 0,062626303 | |
| 230672_at | - | 0,171847695 | 0,109221392 | 0,062626303 | |
| 234773_x_at | - | 0,171847695 | 0,109221392 | 0,062626303 | |
| 243791_at | - | 0,171847695 | 0,109221392 | 0,062626303 | |
| 218793_s_at | SCML1 | 2,173470163 | 2,110887591 | 0,062582572 | |
| 221914_at | SYN1 | 0,496120222 | 0,433557298 | 0,062562924 | |
| 229668_at | - | 0,496120222 | 0,433557298 | 0,062562924 | |
| 212517_at | ATRN | 2,082235068 | 2,019703483 | 0,062531585 | |
| 209476_at | TMX1 | 5,790819745 | 5,728308962 | 0,062510783 | |
| 215913_s_at | GULP1 | 0,984222186 | 0,921731975 | 0,06249021 | |
| 1560916_a_at | DPY19L1 | 1,177702228 | 1,115234684 | 0,062467543 | |
| 235233_s_at | GMEB1 | 1,177702228 | 1,115234684 | 0,062467543 | |
| 228499_at | PFKFB4 | 2,501229299 | 2,438805127 | 0,062424172 | |
| 218830_at | RPL26L1 | 4,866629209 | 4,804227461 | 0,062401747 | |
| 208620_at | PCBP1 | 5,158634383 | 5,096265831 | 0,062368552 | |
| 223532_at | ANKRD39 | 1,504602299 | 1,442269454 | 0,062332845 | |
| 225024_at | RPRD1B | 3,541801785 | 3,479511525 | 0,06229026 | |
| 202141_s_at | COPS8 | 4,453164021 | 4,390931194 | 0,062232827 | |
| 203832_at | SNRPF | 5,947929114 | 5,885696298 | 0,062232816 | |
| 222464_s_at | MCMBP | 4,045288912 | 3,983081173 | 0,062207739 | |
| 213945_s_at | NUP210 | 0,995078803 | 0,932882274 | 0,062196529 | |
| 236915_at | C4orf47 | 0,995078803 | 0,932882274 | 0,062196529 | |
| 1554037_a_at | ZBTB24 | -0,888496123 | -0,950686014 | 0,062189892 | |
| 1563113_at | UBR4 | -0,888496123 | -0,950686014 | 0,062189892 | |
| 1563792_at | AMN | -0,888496123 | -0,950686014 | 0,062189892 | |
| 204713_s_at | F5 | -0,888496123 | -0,950686014 | 0,062189892 | |
| 206674_at | FLT3 | -0,888496123 | -0,950686014 | 0,062189892 | |
| 207298_at | SLC17A3 | -0,888496123 | -0,950686014 | 0,062189892 | |
| 211167_s_at | GCK | -0,888496123 | -0,950686014 | 0,062189892 | |
| 216141_at | - | -0,888496123 | -0,950686014 | 0,062189892 | |
| 216911_s_at | HIC2 | -0,888496123 | -0,950686014 | 0,062189892 | |
| 221350_at | HOXC8 | -0,888496123 | -0,950686014 | 0,062189892 | |
| 226185_at | CDS1 | -0,888496123 | -0,950686014 | 0,062189892 | |
| 229246_at | FLJ44342 | -0,888496123 | -0,950686014 | 0,062189892 | |
| 230489_at | CD5 | -0,888496123 | -0,950686014 | 0,062189892 | |
| 236592_at | - | -0,888496123 | -0,950686014 | 0,062189892 | |
| 243157_at | - | -0,888496123 | -0,950686014 | 0,062189892 | |
| 226936_at | CENPW | 5,265619128 | 5,20345168 | 0,062167447 | |
| 224605_at | C4orf3 | 2,833109217 | 2,770953871 | 0,062155346 | |
| 228361_at | E2F2 | 2,190100188 | 2,127967638 | 0,06213255 | |
| 235020_at | TAF4B | 2,190100188 | 2,127967638 | 0,06213255 | |
| 208532_x_at | KRTAP5-8 | 1,512180019 | 1,450051551 | 0,062128468 | |
| 213848_at | DUSP7 | 2,582657321 | 2,520531219 | 0,062126102 | |

| | | | | | |
|---|---|---|---|---|---|
| 1555754_s_at | ATN1 | 1,191926657 | 1,129843209 | 0,062083448 | |
| 215905_s_at | SNRNP40 | 4,66810148 | 4,606029888 | 0,062071591 | |
| 201152_s_at | MBNL1 | 4,994704915 | 4,932671897 | 0,062033018 | |
| 217256_x_at | - | 5,956025826 | 5,894010769 | 0,062015057 | |
| 1560382_at | GAB1 | -0,221656139 | -0,283667828 | 0,062011689 | |
| 206366_x_at | XCL1 | -0,221656139 | -0,283667828 | 0,062011689 | |
| 213182_x_at | CDKN1C | -0,221656139 | -0,283667828 | 0,062011689 | |
| 217251_x_at | - | -0,221656139 | -0,283667828 | 0,062011689 | |
| 224271_x_at | FRMD8P1 | -0,221656139 | -0,283667828 | 0,062011689 | |
| 229425_at | - | -0,221656139 | -0,283667828 | 0,062011689 | |
| 229451_at | GALNT9 | -0,221656139 | -0,283667828 | 0,062011689 | |
| 230982_at | SOX1 | -0,221656139 | -0,283667828 | 0,062011689 | |
| 209036_s_at | MDH2 | 6,026259918 | 5,964300133 | 0,061959784 | |
| 215822_x_at | MYT1 | 0,518867317 | 0,456918297 | 0,06194902 | |
| 222556_at | ALG5 | 0,518867317 | 0,456918297 | 0,06194902 | |
| 225313_at | FAM217B | 3,897615369 | 3,835672678 | 0,061942691 | |
| 201997_s_at | SPEN | 3,753208234 | 3,691304166 | 0,061904068 | |
| 207157_s_at | GNG5 | 6,322919735 | 6,261020454 | 0,061899281 | |
| 203752_s_at | JUND | 5,379008977 | 5,317117754 | 0,061891223 | |
| 221245_s_at | FZD5 | 2,369762403 | 2,307890888 | 0,061871515 | |
| 238528_at | UBR1 | 1,659347917 | 1,59747851 | 0,061869407 | |
| 216397_s_at | BOP1 | 2,661438684 | 2,599625088 | 0,061813596 | |
| 238722_x_at | NAPEPLD | 1,011212064 | 0,949447784 | 0,06176428 | |
| 221046_s_at | GTPBP8 | 3,354274239 | 3,292527802 | 0,061746437 | |
| 218047_at | OSBPL9 | 5,037507384 | 4,97576335 | 0,061744034 | |
| 225125_at | MMGT1 | 3,06588531 | 3,004228687 | 0,061656623 | |
| 202789_at | PLCG1 | 2,600539826 | 2,538891651 | 0,061648175 | |
| 214463_x_at | HIST1H4J | 2,600539826 | 2,538891651 | 0,061648175 | |
| 1553261_x_at | ALS2CR11 | -3,212234735 | -3,273861381 | 0,061626647 | |
| 1556598_at | ARPP21 /// LOC1 | -3,212234735 | -3,273861381 | 0,061626647 | |
| 1560144_at | - | -3,212234735 | -3,273861381 | 0,061626647 | |
| 1560581_at | - | -3,212234735 | -3,273861381 | 0,061626647 | |
| 1569592_a_at | F11 | -3,212234735 | -3,273861381 | 0,061626647 | |
| 216974_at | - | -3,212234735 | -3,273861381 | 0,061626647 | |
| 241909_at | TNKS2 | -3,212234735 | -3,273861381 | 0,061626647 | |
| 244435_at | FAM196A | -3,212234735 | -3,273861381 | 0,061626647 | |
| 200683_s_at | UBE2L3 | 3,497632487 | 3,436090594 | 0,061541892 | |
| 230739_at | FAM210A | 2,535098095 | 2,473581079 | 0,061517016 | |
| 1556341_s_at | MAPK12 | 0,798390451 | 0,73688026 | 0,06151019 | |
| 214441_at | STX6 | 0,798390451 | 0,73688026 | 0,06151019 | |
| 226787_at | ZNF18 | 0,798390451 | 0,73688026 | 0,06151019 | |
| 221685_s_at | CCDC99 | 4,606604592 | 4,545117128 | 0,061487464 | |
| 1569302_at | KIAA1731 | 1,021868205 | 0,960386719 | 0,061481486 | |
| 1553237_x_at | PCDHAC1 | -0,85085127 | -0,912331589 | 0,061480319 | |
| 1556224_a_at | LOC155060 /// ZI | -0,85085127 | -0,912331589 | 0,061480319 | |
| 1570536_at | - | -0,85085127 | -0,912331589 | 0,061480319 | |
| 202018_s_at | LTF | -0,85085127 | -0,912331589 | 0,061480319 | |
| 206870_at | PPARA | -0,85085127 | -0,912331589 | 0,061480319 | |
| 207552_at | ATP5G2 | -0,85085127 | -0,912331589 | 0,061480319 | |
| 207921_x_at | PAX8 | -0,85085127 | -0,912331589 | 0,061480319 | |
| 208189_s_at | MYO7A | -0,85085127 | -0,912331589 | 0,061480319 | |
| 216157_at | - | -0,85085127 | -0,912331589 | 0,061480319 | |
| 223736_at | IFT81 | -0,85085127 | -0,912331589 | 0,061480319 | |
| 236719_at | - | -0,85085127 | -0,912331589 | 0,061480319 | |
| 238145_at | ZNF496 | -0,85085127 | -0,912331589 | 0,061480319 | |
| 240853_at | - | -0,85085127 | -0,912331589 | 0,061480319 | |

| | | | | | |
|---|---|---|---|---|---|
| 244751_at | PCP2 | -0,85085127 | -0,912331589 | 0,061480319 | |
| 215165_x_at | UMPS | 4,097225224 | 4,035745559 | 0,061479664 | |
| 212897_at | CDK19 | 1,67631007 | 1,614891062 | 0,061419009 | |
| 1552450_a_at | DNAJC5G | -0,196756757 | -0,258114234 | 0,061357477 | |
| 1557382_x_at | AGAP11 | -0,196756757 | -0,258114234 | 0,061357477 | |
| 205793_x_at | TNK1 | -0,196756757 | -0,258114234 | 0,061357477 | |
| 226096_at | FNDC5 | -0,196756757 | -0,258114234 | 0,061357477 | |
| 234430_at | ANTXR1 | -0,196756757 | -0,258114234 | 0,061357477 | |
| 204642_at | S1PR1 | 0,541261317 | 0,479907041 | 0,061354276 | |
| 207130_at | ZMYND8 | 0,541261317 | 0,479907041 | 0,061354276 | |
| 225018_at | SPIRE1 | 0,541261317 | 0,479907041 | 0,061354276 | |
| 203039_s_at | NDUFS1 | 4,196980309 | 4,135635705 | 0,061344604 | |
| 221005_s_at | PTDSS2 | 1,542099044 | 1,480766833 | 0,061332211 | |
| 243750_x_at | FAM207A | 2,220484971 | 2,159161028 | 0,061323943 | |
| 201611_s_at | ICMT | 2,392522438 | 2,331254065 | 0,061268373 | |
| 224738_x_at | RPL7L1 | 6,229619784 | 6,168388135 | 0,061231649 | |
| 41512_at | BRAP | 2,317740882 | 2,256673851 | 0,061067031 | |
| 1552377_s_at | FAM18B2 | 1,037706275 | 0,976641161 | 0,061065114 | |
| 207585_s_at | RPL36AL | 6,804406705 | 6,74335226 | 0,061054446 | |
| 1563660_at | - | -3,250025915 | -3,311072652 | 0,061046737 | |
| 207447_s_at | MGAT4C | -3,250025915 | -3,311072652 | 0,061046737 | |
| 209292_at | ID4 | -3,250025915 | -3,311072652 | 0,061046737 | |
| 241868_at | - | -3,250025915 | -3,311072652 | 0,061046737 | |
| 218143_s_at | SCAMP2 | 3,319065909 | 3,258033502 | 0,061032407 | |
| 204741_at | BICD1 | 0,555999837 | 0,495031851 | 0,060967986 | |
| 231017_at | STK11 | 1,820612054 | 1,759685182 | 0,060926872 | |
| 202257_s_at | CD2BP2 | 2,406821352 | 2,345926937 | 0,060894416 | |
| 212102_s_at | KPNA6 /// LOC10 | 2,14450783 | 2,083632167 | 0,060875663 | |
| 222852_at | C10orf88 | 1,238353345 | 1,177496821 | 0,060856524 | |
| 224846_at | SHKBP1 | 1,238353345 | 1,177496821 | 0,060856524 | |
| 203899_s_at | CRCP | 1,560488174 | 1,499637023 | 0,060851151 | |
| 203382_s_at | APOE | 6,121220712 | 6,060370018 | 0,060850694 | |
| 46256_at | SPSB3 | 3,367911651 | 3,307062282 | 0,06084937 | |
| 212982_at | ZDHHC17 | 2,940912947 | 2,880111483 | 0,060801464 | |
| 229269_x_at | SSBP4 | 3,285189903 | 3,22439681 | 0,060793093 | |
| 226088_at | ZDHHC12 | 1,04816921 | 0,987376598 | 0,060792613 | |
| 208688_x_at | EIF3B | 5,125360659 | 5,064617399 | 0,060743259 | |
| 238880_at | GTF3A | 2,243430036 | 2,182705233 | 0,060724803 | |
| 224413_s_at | TM2D2 | 5,052201321 | 4,991513183 | 0,060688139 | |
| 219979_s_at | C11orf73 | 3,000043914 | 2,939363796 | 0,060680119 | |
| 201845_s_at | RYBP | 5,595105514 | 5,534456965 | 0,060648549 | |
| 1560587_s_at | PRDX5 | 4,961359357 | 4,900729837 | 0,06062952 | |
| 228201_at | ARL13B | 3,336243945 | 3,275657468 | 0,060586477 | |
| 212433_x_at | RPS2 /// SNORA | 7,926763454 | 7,866183754 | 0,060579699 | |
| 218720_x_at | SEZ6L2 | 1,709647566 | 1,64909809 | 0,060549475 | |
| 218679_s_at | VPS28 | 4,032830746 | 3,972313606 | 0,06051714 | |
| 217818_s_at | ARPC4 | 3,006764929 | 2,946258252 | 0,060506676 | |
| 209724_s_at | ZFP161 | 1,251994932 | 1,191491281 | 0,060503651 | |
| 217956_s_at | ENOPH1 | 4,442792954 | 4,382314353 | 0,060478601 | |
| 233822_x_at | - | -3,287237186 | -3,347710726 | 0,06047354 | |
| 244586_x_at | - | -3,287237186 | -3,347710726 | 0,06047354 | |
| 202364_at | MXI1 | 3,253893136 | 3,193438316 | 0,06045482 | |
| 216354_at | - | 0,57782909 | 0,51742585 | 0,06040324 | |
| 219473_at | GDAP2 | 0,57782909 | 0,51742585 | 0,06040324 | |
| 227684_at | S1PR2 | 1,578645857 | 1,518263577 | 0,060382279 | |
| 209531_at | GSTZ1 | 2,716594694 | 2,656223838 | 0,060370856 | |

| | | | | | |
|---|---|---|---|---|---|
| 1562603_at | RAD51L3-RFFL / | -0,79540287 | -0,855751026 | 0,060348156 | |
| 210954_s_at | TSC22D2 | -0,79540287 | -0,855751026 | 0,060348156 | |
| 217138_x_at | IGLC1 | -0,79540287 | -0,855751026 | 0,060348156 | |
| 217203_at | - | -0,79540287 | -0,855751026 | 0,060348156 | |
| 219383_at | PRR5L | -0,79540287 | -0,855751026 | 0,060348156 | |
| 223464_at | OSBPL5 | -0,79540287 | -0,855751026 | 0,060348156 | |
| 227948_at | FGD4 | -0,79540287 | -0,855751026 | 0,060348156 | |
| 232328_at | ZNF552 | -0,79540287 | -0,855751026 | 0,060348156 | |
| 239098_at | KCNRG | -0,79540287 | -0,855751026 | 0,060348156 | |
| 243367_at | - | -0,79540287 | -0,855751026 | 0,060348156 | |
| 201071_x_at | SF3B1 | 6,00770067 | 5,947389902 | 0,060310769 | |
| 209081_s_at | COL18A1 | 1,261018186 | 1,200746069 | 0,060272116 | |
| 221290_s_at | MUM1 | 1,261018186 | 1,200746069 | 0,060272116 | |
| 202143_s_at | COPS8 | 4,119101006 | 4,058831494 | 0,060269512 | |
| 211509_s_at | RTN4 | 5,592203727 | 5,531938482 | 0,060265245 | |
| 201473_at | JUNB | 2,261526912 | 2,201267805 | 0,060259107 | |
| 237289_at | CREB1 | 1,58584542 | 1,525647381 | 0,06019804 | |
| 226520_at | LCOR | 2,171078719 | 2,110887591 | 0,060191128 | |
| 211799_x_at | HLA-C | 3,992767659 | 3,932593347 | 0,060174312 | |
| 223353_at | MOB2 | 1,073999308 | 1,013870809 | 0,060128499 | |
| 226273_at | CLCN5 | 1,073999308 | 1,013870809 | 0,060128499 | |
| 221789_x_at | RHOT2 | 1,968405881 | 1,908311039 | 0,060094842 | |
| 201356_at | SF3A1 | 3,791855421 | 3,731768909 | 0,060086513 | |
| 209814_at | ZNF330 | 4,419307767 | 4,359253484 | 0,060054283 | |
| 211938_at | EIF4B /// LOC100 | 4,777867415 | 4,717826149 | 0,060041266 | |
| 219748_at | TREML2 | 0,592200562 | 0,532164371 | 0,060036191 | |
| 222684_s_at | NOL10 | 4,293033569 | 4,23300737 | 0,060026199 | |
| 217989_at | HSD17B11 | 3,664184451 | 3,604188755 | 0,059995696 | |
| 224759_s_at | C12orf23 | 4,297973795 | 4,238072872 | 0,059900923 | |
| 228908_s_at | LOC642852 | 2,799441827 | 2,739547836 | 0,059893991 | |
| 200663_at | CD63 | 5,864822529 | 5,804934308 | 0,05988822 | |
| 214224_s_at | PIN4 | 3,598072957 | 3,538185672 | 0,059887285 | |
| 228910_at | CD82 | 1,735773781 | 1,675891488 | 0,059882293 | |
| 225296_at | ZNF317 | 2,976955384 | 2,917077481 | 0,059877903 | |
| 212610_at | PTPN11 | 5,621619186 | 5,561761707 | 0,059857479 | |
| 1554759_at | SRCIN1 | -0,136301282 | -0,19610998 | 0,059808698 | |
| 205754_at | F2 | -0,136301282 | -0,19610998 | 0,059808698 | |
| 207313_x_at | KIR3DL1 /// KIR3 | -0,136301282 | -0,19610998 | 0,059808698 | |
| 213669_at | FCHO1 | -0,136301282 | -0,19610998 | 0,059808698 | |
| 214128_at | DAGLA | -0,136301282 | -0,19610998 | 0,059808698 | |
| 228172_at | - | -0,136301282 | -0,19610998 | 0,059808698 | |
| 236676_at | - | -0,136301282 | -0,19610998 | 0,059808698 | |
| 202801_at | PRKACA | 2,52764011 | 2,467842989 | 0,059797121 | |
| 203525_s_at | APC | 3,188003814 | 3,128242026 | 0,059761787 | |
| 202618_s_at | MECP2 | 1,089278259 | 1,029536893 | 0,059741366 | |
| 212691_at | NUP188 | 2,604089881 | 2,544354523 | 0,059735357 | |
| 222978_at | SURF4 | 4,904731178 | 4,845008859 | 0,059722319 | |
| 206145_at | RHAG | 7,378599585 | 7,318890442 | 0,059709144 | |
| 1558668_s_at | SPATA22 | -3,342483913 | -3,402185466 | 0,059701552 | |
| 208932_at | PPP4C | 4,284208955 | 4,224525141 | 0,059683814 | |
| 1559713_at | LOC100507150 | -0,759588698 | -0,819238336 | 0,059649638 | |
| 1562549_at | - | -0,759588698 | -0,819238336 | 0,059649638 | |
| 1569681_at | - | -0,759588698 | -0,819238336 | 0,059649638 | |
| 202638_s_at | ICAM1 | -0,759588698 | -0,819238336 | 0,059649638 | |
| 206781_at | DNAJC4 | -0,759588698 | -0,819238336 | 0,059649638 | |
| 208712_at | CCND1 | -0,759588698 | -0,819238336 | 0,059649638 | |

| | | | | | |
|---|---|---|---|---|---|
| 217115_at | MKRN7P | -0,759588698 | -0,819238336 | 0,059649638 | |
| 219107_at | BCAN | -0,759588698 | -0,819238336 | 0,059649638 | |
| 225956_at | CREBRF | -0,759588698 | -0,819238336 | 0,059649638 | |
| 228380_at | SENP2 | -0,759588698 | -0,819238336 | 0,059649638 | |
| 230183_at | EXT1 | -0,759588698 | -0,819238336 | 0,059649638 | |
| 240728_at | - | -0,759588698 | -0,819238336 | 0,059649638 | |
| 242745_at | CASR | -0,759588698 | -0,819238336 | 0,059649638 | |
| 244542_at | BCDIN3D-AS1 | -0,759588698 | -0,819238336 | 0,059649638 | |
| 222338_x_at | LOC646808 | 2,929639344 | 2,869995113 | 0,05964423 | |
| 201060_x_at | STOM | 5,001955461 | 4,94235962 | 0,059595841 | |
| 217873_at | CAB39 | 4,699139437 | 4,639594721 | 0,059544716 | |
| 213007_at | FANCI | 3,813483425 | 3,753943043 | 0,059540382 | |
| 218490_s_at | ZNF302 | 4,097854871 | 4,038328982 | 0,059525889 | |
| 229666_s_at | CSTF3 | 3,19743497 | 3,137910193 | 0,059524777 | |
| 201725_at | CDC123 | 4,920655822 | 4,861149946 | 0,059505876 | |
| 240393_at | - | 0,613492853 | 0,553993624 | 0,059499229 | |
| 219817_at | MAPKAPK5-AS1 | 1,614289682 | 1,55481039 | 0,059479291 | |
| 203944_x_at | BTN2A1 | 2,817903185 | 2,758473378 | 0,059429807 | |
| 218716_x_at | MTO1 | 3,720873481 | 3,661450356 | 0,059423126 | |
| 201360_at | CST3 | 3,464815649 | 3,405399563 | 0,059416086 | |
| 203454_s_at | ATOX1 | 4,600397104 | 4,541024081 | 0,059373023 | |
| 212979_s_at | FAM115A /// LOC | 3,105247586 | 3,045906378 | 0,059341208 | |
| 200816_s_at | PAFAH1B1 | 3,999522577 | 3,940211012 | 0,059311565 | |
| 225550_at | CNST | 2,206540699 | 2,147243253 | 0,059297446 | |
| 200941_at | HSBP1 | 3,471633352 | 3,412387088 | 0,059246265 | |
| 202387_at | BAG1 | 3,471633352 | 3,412387088 | 0,059246265 | |
| 224790_at | ASAP1 | 3,827217859 | 3,768019955 | 0,059197904 | |
| 221998_s_at | VRK3 | 2,947914474 | 2,888726553 | 0,059187921 | |
| 213363_at | CA5BP1 | 1,305306849 | 1,246149888 | 0,059156961 | |
| 213859_x_at | SMARCA5 | 2,764969948 | 2,705818724 | 0,059151224 | |
| 1557169_x_at | HCG11 | 0,627515113 | 0,568365095 | 0,059150018 | |
| 209241_x_at | MINK1 | 0,627515113 | 0,568365095 | 0,059150018 | |
| 216860_s_at | GDF11 | 0,627515113 | 0,568365095 | 0,059150018 | |
| 216246_at | - | 2,213529682 | 2,154405725 | 0,059123957 | |
| 224025_s_at | ATG7 | 1,114389003 | 1,055274826 | 0,059114178 | |
| 227135_at | NAAA | 1,114389003 | 1,055274826 | 0,059114178 | |
| 214196_s_at | TPP1 | 0,30867765 | 0,249604176 | 0,059073474 | |
| 226440_at | DUSP22 /// LOC1 | 2,009141686 | 1,950068772 | 0,059072914 | |
| 232052_at | LOC440944 | 1,631786706 | 1,572742379 | 0,059044327 | |
| 223457_at | COPG2 | 3,120200849 | 3,061230246 | 0,058970603 | |
| 215171_s_at | TIMM17A | 5,960360187 | 5,90139096 | 0,058969227 | |
| 210622_x_at | CDK10 | 1,638726528 | 1,579853228 | 0,058873299 | |
| 212434_at | GRPEL1 | 3,903389697 | 3,844556416 | 0,058833282 | |
| 225052_at | TMEM203 | 3,680249569 | 3,621520989 | 0,05872858 | |
| 238842_at | - | 2,846658948 | 2,787940144 | 0,058718803 | |
| 212644_s_at | MAPK1IP1L | 4,495352883 | 4,436691079 | 0,058661804 | |
| 236685_at | - | 0,648296226 | 0,589657387 | 0,058638839 | |
| 1564272_a_at | KLHDC1 | -3,41365796 | -3,472251727 | 0,058593767 | |
| 1561509_at | - | -0,706943342 | -0,765504029 | 0,058560687 | |
| 1566760_at | FLJ34208 | -0,706943342 | -0,765504029 | 0,058560687 | |
| 207521_s_at | ATP2A3 | -0,706943342 | -0,765504029 | 0,058560687 | |
| 216472_at | - | -0,706943342 | -0,765504029 | 0,058560687 | |
| 217603_at | ATP6V0A2 | -0,706943342 | -0,765504029 | 0,058560687 | |
| 217658_at | THAP3 | -0,706943342 | -0,765504029 | 0,058560687 | |
| 221166_at | FGF23 | -0,706943342 | -0,765504029 | 0,058560687 | |
| 223729_at | CECR2 | -0,706943342 | -0,765504029 | 0,058560687 | |

| | | | | | |
|---|---|---|---|---|---|
| 229811_at | - | -0,706943342 | -0,765504029 | 0,058560687 | |
| 231371_at | TDRD10 | -0,706943342 | -0,765504029 | 0,058560687 | |
| 237228_at | ZDHHC1 | -0,706943342 | -0,765504029 | 0,058560687 | |
| 239202_at | RAB3B | -0,706943342 | -0,765504029 | 0,058560687 | |
| 241693_at | - | -0,706943342 | -0,765504029 | 0,058560687 | |
| 223640_at | HCST | 1,139070151 | 1,080561626 | 0,058508525 | |
| 201384_s_at | NBR1 | 2,658027143 | 2,599625088 | 0,058402056 | |
| 234630_at | - | -0,078265071 | -0,136643983 | 0,058378911 | |
| 234681_s_at | CHD6 | -0,078265071 | -0,136643983 | 0,058378911 | |
| 239187_at | DCAF16 | -0,078265071 | -0,136643983 | 0,058378911 | |
| 243921_at | - | -0,078265071 | -0,136643983 | 0,058378911 | |
| 201082_s_at | DCTN1 | 0,661985843 | 0,603679647 | 0,058306196 | |
| 202739_s_at | PHKB | 0,661985843 | 0,603679647 | 0,058306196 | |
| 224182_x_at | SEMA6B | 0,661985843 | 0,603679647 | 0,058306196 | |
| 223547_at | JKAMP | 2,344516784 | 2,286318242 | 0,058198542 | |
| 218939_at | LETM1 | 1,932146506 | 1,874012195 | 0,058134311 | |
| 221858_at | TBC1D12 | 1,932146506 | 1,874012195 | 0,058134311 | |
| 208872_s_at | REEP5 | 4,537271727 | 4,479143747 | 0,05812798 | |
| 204640_s_at | SPOP | 3,741167469 | 3,683073218 | 0,058094251 | |
| 217045_x_at | NCR2 | 0,934330293 | 0,876248481 | 0,058081812 | |
| 230740_at | EHD3 | 0,934330293 | 0,876248481 | 0,058081812 | |
| 239835_at | KBTBD8 | 3,158127685 | 3,100079607 | 0,058048079 | |
| 1557303_at | NT5C | 0,351548291 | 0,293518836 | 0,058029455 | |
| 1568666_at | PLIN5 | 0,351548291 | 0,293518836 | 0,058029455 | |
| 219980_at | C4orf29 | 0,351548291 | 0,293518836 | 0,058029455 | |
| 202973_x_at | FAM13A | 2,938102794 | 2,880111483 | 0,057991311 | |
| 218079_s_at | GGNBP2 | 3,21379258 | 3,155865206 | 0,057927374 | |
| 242550_at | EIF3B | 1,163336153 | 1,105412836 | 0,057923318 | |
| 234672_s_at | TMEM48 | 4,864040601 | 4,806123612 | 0,057916989 | |
| 1553977_a_at | CYP39A1 | -0,67289328 | -0,730778808 | 0,057885528 | |
| 1554796_at | DPP6 | -0,67289328 | -0,730778808 | 0,057885528 | |
| 1557518_a_at | - | -0,67289328 | -0,730778808 | 0,057885528 | |
| 1559117_at | - | -0,67289328 | -0,730778808 | 0,057885528 | |
| 1559119_at | - | -0,67289328 | -0,730778808 | 0,057885528 | |
| 205312_at | SPI1 | -0,67289328 | -0,730778808 | 0,057885528 | |
| 206671_at | SAG | -0,67289328 | -0,730778808 | 0,057885528 | |
| 222072_at | LOC100505933 | -0,67289328 | -0,730778808 | 0,057885528 | |
| 223616_at | ZNF649 | -0,67289328 | -0,730778808 | 0,057885528 | |
| 229361_at | SLC25A27 | -0,67289328 | -0,730778808 | 0,057885528 | |
| 230994_at | FAM181B | -0,67289328 | -0,730778808 | 0,057885528 | |
| 235605_at | CASZ1 | -0,67289328 | -0,730778808 | 0,057885528 | |
| 240493_at | - | -0,67289328 | -0,730778808 | 0,057885528 | |
| 203024_s_at | C5orf15 | 3,568395593 | 3,510542997 | 0,057852597 | |
| 217520_x_at | LOC283683 /// L( | 2,266015882 | 2,208167653 | 0,057848229 | |
| 203428_s_at | ASF1A | 3,645356455 | 3,587527995 | 0,05782846 | |
| 244352_at | CD84 | 0,682279745 | 0,624460759 | 0,057818986 | |
| 214316_x_at | CALR | 1,360921491 | 1,303116084 | 0,057805407 | |
| 216250_s_at | LPXN | 1,360921491 | 1,303116084 | 0,057805407 | |
| 218596_at | TBC1D13 | 1,360921491 | 1,303116084 | 0,057805407 | |
| 224187_x_at | HSPA8 /// SNOR | 7,633728589 | 7,575927406 | 0,057801182 | |
| 224430_s_at | MTO1 | 3,170152481 | 3,112391743 | 0,057760738 | |
| 224932_at | CHCHD10 | 3,950863319 | 3,893176339 | 0,057686981 | |
| 1552472_a_at | ACAP2 | 1,373456798 | 1,315948728 | 0,05750807 | |
| 225341_at | MTERFD3 | 1,373456798 | 1,315948728 | 0,05750807 | |
| 235745_at | ERN1 | 0,695652136 | 0,638150376 | 0,05750176 | |
| 209024_s_at | SYNCRIP | 5,168258855 | 5,110759903 | 0,057498952 | |

| | | | | | |
|---|---|---|---|---|---|
| 200725_x_at | RPL10 /// SNORA | 7,286702126 | 7,229237793 | 0,057464333 | |
| 212391_x_at | RPS3A /// SNOR | 8,179552493 | 8,122090785 | 0,057461708 | |
| 212179_at | PNISR | 2,901063789 | 2,843651104 | 0,057412685 | |
| 205094_at | PEX12 | 2,185368281 | 2,127967638 | 0,057400643 | |
| 218958_at | C19orf60 | 1,187200746 | 1,129843209 | 0,057357537 | |
| 218683_at | PTBP2 | 2,288253583 | 2,230931691 | 0,057321891 | |
| 221689_s_at | PIGP | 4,490086298 | 4,432769076 | 0,057317222 | |
| 205881_at | ZNF74 | 1,381753566 | 1,324440845 | 0,057312721 | |
| 221135_s_at | ASTE1 | 1,381753566 | 1,324440845 | 0,057312721 | |
| 224722_at | MIB1 | 1,381753566 | 1,324440845 | 0,057312721 | |
| 207831_x_at | DHPS | 4,136978698 | 4,079673902 | 0,057304796 | |
| 217994_x_at | CPSF3L | 2,382221458 | 2,324919719 | 0,057301739 | |
| 203974_at | HDHD1 | 4,55296954 | 4,495687112 | 0,057282428 | |
| 218005_at | ZNF22 | 2,845159691 | 2,787940144 | 0,057219547 | |
| 231931_at | PRDM15 | 1,556829052 | 1,499637023 | 0,057192029 | |
| 225733_at | B3GALT6 | 2,294858597 | 2,237691445 | 0,057167152 | |
| 239102_s_at | - | 2,089851283 | 2,032717854 | 0,057133429 | |
| 225520_at | MTHFD1L | 2,2992452 | 2,242180416 | 0,057064785 | |
| 236594_at | LLGL1 | 2,2992452 | 2,242180416 | 0,057064785 | |
| 211613_s_at | GPD2 | -0,022466681 | -0,079522948 | 0,057056268 | |
| 217684_at | TYMS | -0,022466681 | -0,079522948 | 0,057056268 | |
| 206139_at | PI4KB | 0,715481152 | 0,658444279 | 0,057036872 | |
| 214177_s_at | PBXIP1 | 0,715481152 | 0,658444279 | 0,057036872 | |
| 1552689_at | CASKIN1 | 1,201332287 | 1,144305291 | 0,057026996 | |
| 202725_at | POLR2A | 2,305800198 | 2,248887791 | 0,056912407 | |
| 1552490_at | LACE1 | -0,622910612 | -0,679763839 | 0,056853227 | |
| 1557479_at | FAM212B | -0,622910612 | -0,679763839 | 0,056853227 | |
| 1564391_at | - | -0,622910612 | -0,679763839 | 0,056853227 | |
| 211263_s_at | PCSK6 | -0,622910612 | -0,679763839 | 0,056853227 | |
| 213143_at | C2orf72 | -0,622910612 | -0,679763839 | 0,056853227 | |
| 214147_at | HEATR8 | -0,622910612 | -0,679763839 | 0,056853227 | |
| 214619_at | CRHR1 | -0,622910612 | -0,679763839 | 0,056853227 | |
| 215243_s_at | GJB3 | -0,622910612 | -0,679763839 | 0,056853227 | |
| 215536_at | HLA-DQB2 | -0,622910612 | -0,679763839 | 0,056853227 | |
| 216220_s_at | ADORA1 | -0,622910612 | -0,679763839 | 0,056853227 | |
| 216615_s_at | HTR3A | -0,622910612 | -0,679763839 | 0,056853227 | |
| 223706_at | C22orf23 | -0,622910612 | -0,679763839 | 0,056853227 | |
| 226852_at | MTA3 | -0,622910612 | -0,679763839 | 0,056853227 | |
| 227666_at | DCLK2 | -0,622910612 | -0,679763839 | 0,056853227 | |
| 228140_s_at | PPP2R2C | -0,622910612 | -0,679763839 | 0,056853227 | |
| 238192_at | - | -0,622910612 | -0,679763839 | 0,056853227 | |
| 241326_at | AK7 | -0,622910612 | -0,679763839 | 0,056853227 | |
| 241840_at | - | -0,622910612 | -0,679763839 | 0,056853227 | |
| 201541_s_at | ZNHIT1 | 4,209852334 | 4,15302303 | 0,056829304 | |
| 217905_at | MCMBP | 1,210676994 | 1,153866761 | 0,056810233 | |
| 226598_s_at | GTPBP5 | 1,210676994 | 1,153866761 | 0,056810233 | |
| 228093_at | ZNF599 | 2,796341835 | 2,739547836 | 0,056793999 | |
| 1554016_a_at | C16orf57 | 1,575032557 | 1,518263577 | 0,056768979 | |
| 40640_at | NCAPH2 | 0,405440979 | 0,348681268 | 0,056759711 | |
| 218257_s_at | UGGT1 | 1,406361373 | 1,349621332 | 0,056740041 | |
| 225277_at | SLC39A13 | 0,728550685 | 0,67181667 | 0,056734015 | |
| 212652_s_at | SNX4 | 3,994121175 | 3,937445604 | 0,056675572 | |
| 1561969_at | ZPLD1 | 4,713590151 | 4,656929558 | 0,056660593 | |
| 224936_at | EIF2S3 | 5,713123031 | 5,656556785 | 0,056566246 | |
| 229573_at | USP9X | 1,414471577 | 1,3579181 | 0,056553477 | |
| 213951_s_at | PSMC3IP | 2,114952142 | 2,058399602 | 0,05655254 | |

| | | | | | |
|---|---|---|---|---|---|
| 31874_at | GAS2L1 | 2,700931419 | 2,644402192 | 0,056529227 | |
| 1553613_s_at | FOXC1 | 1,224581536 | 1,16809119 | 0,056490345 | |
| 220476_s_at | FAM212B | 1,224581536 | 1,16809119 | 0,056490345 | |
| 217740_x_at | RPL7A /// SNORI | 7,741036171 | 7,684588869 | 0,056447302 | |
| 219937_at | TRHDE | 2,327437554 | 2,271023131 | 0,056414423 | |
| 229394_s_at | ARHGAP35 | 3,913439491 | 3,857048799 | 0,056390692 | |
| 202197_at | MTMR3 | 2,422991296 | 2,366632651 | 0,056358645 | |
| 203696_s_at | RFC2 | 3,123915073 | 3,067567462 | 0,056347611 | |
| 232219_x_at | USP21 | 1,883312263 | 1,826973888 | 0,056338375 | |
| 214061_at | WDR67 | 2,745814053 | 2,689475799 | 0,056338254 | |
| 212042_x_at | RPL7 | 7,801859754 | 7,745538886 | 0,056320868 | |
| 209811_at | CASP2 | 2,331726354 | 2,275409734 | 0,05631662 | |
| 205208_at | ALDH1L1 | 0,747935635 | 0,691645685 | 0,05628995 | |
| 235855_at | COX15 | 0,747935635 | 0,691645685 | 0,05628995 | |
| 202476_s_at | LOC100506167 / | 1,426552034 | 1,370274469 | 0,056277565 | |
| 222554_s_at | NOL6 | 1,426552034 | 1,370274469 | 0,056277565 | |
| 234084_x_at | - | 1,426552034 | 1,370274469 | 0,056277565 | |
| 222199_s_at | BIN3 | 3,127619759 | 3,071356473 | 0,056263286 | |
| 230029_x_at | UBR3 | 4,802411098 | 4,746152168 | 0,05625893 | |
| 221524_s_at | RRAGD | 4,637680685 | 4,581441196 | 0,056239488 | |
| 225820_at | PHF17 | 2,014487247 | 1,958277216 | 0,056210031 | |
| 1553063_at | GPR78 | -0,59053611 | -0,646746079 | 0,056209968 | |
| 1562219_at | FLJ41649 | -0,59053611 | -0,646746079 | 0,056209968 | |
| 1569959_at | - | -0,59053611 | -0,646746079 | 0,056209968 | |
| 205701_at | IPO8 | -0,59053611 | -0,646746079 | 0,056209968 | |
| 205785_at | ITGAM | -0,59053611 | -0,646746079 | 0,056209968 | |
| 210699_at | - | -0,59053611 | -0,646746079 | 0,056209968 | |
| 215530_at | FANCA | -0,59053611 | -0,646746079 | 0,056209968 | |
| 227620_at | SLC44A1 | -0,59053611 | -0,646746079 | 0,056209968 | |
| 235653_s_at | THAP6 | -0,59053611 | -0,646746079 | 0,056209968 | |
| 239076_at | - | -0,59053611 | -0,646746079 | 0,056209968 | |
| 240005_at | - | -0,59053611 | -0,646746079 | 0,056209968 | |
| 241443_at | - | -0,59053611 | -0,646746079 | 0,056209968 | |
| 228956_at | UGT8 | 1,434549838 | 1,378453643 | 0,056096194 | |
| 227983_at | RILPL2 | 2,758612869 | 2,702564916 | 0,056047953 | |
| 229743_at | ZNF438 | 0,760715727 | 0,704715219 | 0,056000508 | |
| 202692_s_at | UBTF | 1,247462054 | 1,191491281 | 0,055970773 | |
| 227396_at | LOC100287223 / | 1,247462054 | 1,191491281 | 0,055970773 | |
| 232983_s_at | SERGEF | 1,247462054 | 1,191491281 | 0,055970773 | |
| 204362_at | SKAP2 | 3,249366227 | 3,193438316 | 0,055927911 | |
| 212937_s_at | COL6A1 | 0,03125738 | -0,024572586 | 0,055829966 | |
| 215413_at | EXOC7 | 0,03125738 | -0,024572586 | 0,055829966 | |
| 227251_at | DCAF5 | 0,03125738 | -0,024572586 | 0,055829966 | |
| 235285_at | - | 0,03125738 | -0,024572586 | 0,055829966 | |
| 239056_at | SEC22C | 0,03125738 | -0,024572586 | 0,055829966 | |
| 1555411_a_at | CCNL1 | 3,354274239 | 3,298466943 | 0,055807295 | |
| 222395_s_at | UBE2Z | 3,149650293 | 3,09388391 | 0,055766383 | |
| 200064_at | HSP90AB1 | 7,236871635 | 7,181245959 | 0,055625676 | |
| 233943_x_at | PLEKHA8 | 0,779676066 | 0,724100169 | 0,055575896 | |
| 213105_s_at | TSR3 | 2,26825514 | 2,212749286 | 0,055505854 | |
| 226055_at | ARRDC2 | 1,269985355 | 1,214517879 | 0,055467476 | |
| 1555793_a_at | ZFP82 | 3,462861802 | 3,407399455 | 0,055462347 | |
| 217168_s_at | HERPUD1 | 3,36999832 | 3,314541423 | 0,055456897 | |
| 202300_at | HBXIP | 5,156973692 | 5,101525193 | 0,055448499 | |
| 227284_at | ZNF766 | 3,32445605 | 3,269073685 | 0,055382365 | |
| 214715_x_at | ZNF160 | 3,926967479 | 3,871608557 | 0,055358922 | |

| | | | | | |
|---|---|---|---|---|---|
| 221918_at | CDK17 | 3,467741468 | 3,412387088 | 0,05535438 | |
| 226233_at | B3GALNT2 | 3,052955227 | 2,997606395 | 0,055348831 | |
| 236814_at | MDM4 | 3,598072957 | 3,542741726 | 0,055331231 | |
| 221648_s_at | AGMAT | 3,423218668 | 3,367892567 | 0,055326101 | |
| 225098_at | ABI2 | 2,37600538 | 2,320681318 | 0,055324062 | |
| 203459_s_at | VPS16 | 2,791679321 | 2,7363693 | 0,055310021 | |
| 211899_s_at | TRAF4 | 0,792179257 | 0,73688026 | 0,055298996 | |
| 213616_at | TPGS2 | 2,171078719 | 2,115788273 | 0,055290446 | |
| 203984_s_at | CASP9 | 3,171349466 | 3,116064987 | 0,055284479 | |
| 222809_x_at | CCDC85C | 1,473887017 | 1,418668082 | 0,055218935 | |
| 233140_s_at | C14orf133 | 1,473887017 | 1,418668082 | 0,055218935 | |
| 225716_at | BRI3BP | 3,967495801 | 3,912315453 | 0,055180348 | |
| 201514_s_at | G3BP1 | 4,076291169 | 4,021126974 | 0,055164195 | |
| 209412_at | TRAPPC10 | 3,062018438 | 3,006869115 | 0,055149323 | |
| 222099_s_at | LSM14A | 3,686960673 | 3,631821253 | 0,05513942 | |
| 201066_at | CYC1 | 5,754941599 | 5,699830898 | 0,055110701 | |
| 209658_at | CDC16 | 3,837056482 | 3,781960839 | 0,055095643 | |
| 209358_at | TAF11 | 4,412743794 | 4,357702907 | 0,055040887 | |
| 225794_s_at | C22orf32 | 3,611363461 | 3,556324192 | 0,055039269 | |
| 228158_at | LOC645166 /// LC | 2,182996494 | 2,127967638 | 0,055028856 | |
| 225312_at | COMMD6 | 5,278173421 | 5,223170799 | 0,055002622 | |
| 210958_s_at | MAST4 | 1,485482016 | 1,43051703 | 0,054964985 | |
| 213144_at | GOSR2 | 1,485482016 | 1,43051703 | 0,054964985 | |
| 218765_at | SIDT2 | 1,485482016 | 1,43051703 | 0,054964985 | |
| 91580_at | LRTM1 | -2,887413183 | -2,942256703 | 0,05484352 | |
| 202884_s_at | PPP2R1B | 1,814495998 | 1,759685182 | 0,054810816 | |
| 208640_at | RAC1 | 6,0549968 | 6,00019003 | 0,05480677 | |
| 226820_at | ZNF362 | 1,954567604 | 1,899812471 | 0,054755133 | |
| 227698_s_at | RAB40C | 1,954567604 | 1,899812471 | 0,054755133 | |
| 212605_s_at | - | 2,954882186 | 2,900133867 | 0,054748319 | |
| 206016_at | CCDC22 | 2,817903185 | 2,763166224 | 0,054736961 | |
| 208952_s_at | LARP4B | 2,817903185 | 2,763166224 | 0,054736961 | |
| 208515_at | HIST1H2BM | 0,083054335 | 0,028364014 | 0,05469032 | |
| 217555_at | SMC1A | 0,083054335 | 0,028364014 | 0,05469032 | |
| 238642_at | ANKRD13D | 0,083054335 | 0,028364014 | 0,05469032 | |
| 200657_at | SLC25A5 | 6,783815538 | 6,72913132 | 0,054684218 | |
| 1552665_at | LOC84989 | -0,512251684 | -0,566887459 | 0,054635775 | |
| 1556732_at | EML4 | -0,512251684 | -0,566887459 | 0,054635775 | |
| 1559121_s_at | - | -0,512251684 | -0,566887459 | 0,054635775 | |
| 204431_at | TLE2 | -0,512251684 | -0,566887459 | 0,054635775 | |
| 212551_at | CAP2 | -0,512251684 | -0,566887459 | 0,054635775 | |
| 213484_at | - | -0,512251684 | -0,566887459 | 0,054635775 | |
| 215169_at | SLC35E2 | -0,512251684 | -0,566887459 | 0,054635775 | |
| 230184_at | LOC100506421 | -0,512251684 | -0,566887459 | 0,054635775 | |
| 230898_at | - | -0,512251684 | -0,566887459 | 0,054635775 | |
| 233189_at | - | -0,512251684 | -0,566887459 | 0,054635775 | |
| 237948_at | - | -0,512251684 | -0,566887459 | 0,054635775 | |
| 243297_at | - | -0,512251684 | -0,566887459 | 0,054635775 | |
| 244486_at | PINK1-AS | -0,512251684 | -0,566887459 | 0,054635775 | |
| 229989_at | FDXACB1 | 0,822971223 | 0,768343791 | 0,054627433 | |
| 215273_s_at | TADA3 | 2,825526235 | 2,770953871 | 0,054572364 | |
| 214538_x_at | RGS6 | 1,504602299 | 1,450051551 | 0,054550748 | |
| 226048_at | MAPK8 | 1,504602299 | 1,450051551 | 0,054550748 | |
| 215933_s_at | HHEX | 5,074765177 | 5,02022104 | 0,054544137 | |
| 207253_s_at | UBN1 | 1,829737795 | 1,775255987 | 0,054481808 | |
| 212733_at | KIAA0226 | 3,508091848 | 3,453616097 | 0,054475751 | |

| | | | | | |
|---|---|---|---|---|---|
| 214805_at | EIF4A1 | 2,094906514 | 2,040470494 | 0,05443602 | |
| 211212_s_at | ORC5 | 2,213529682 | 2,159161028 | 0,054368654 | |
| 203095_at | MTIF2 | 4,346467354 | 4,292109123 | 0,054358231 | |
| 200829_x_at | ZNF207 | 5,704983859 | 5,650659434 | 0,054324425 | |
| 224736_at | CCAR1 | 4,244385102 | 4,190088544 | 0,054296558 | |
| 225149_at | PCID2 | 2,604089881 | 2,549796788 | 0,054293093 | |
| 205322_s_at | MTF1 | 0,841135803 | 0,786897721 | 0,054238082 | |
| 219931_s_at | KLHL12 | 0,841135803 | 0,786897721 | 0,054238082 | |
| 210022_at | PCGF1 | 1,326951551 | 1,272721751 | 0,0542298 | |
| 211902_x_at | YME1L1 | 1,326951551 | 1,272721751 | 0,0542298 | |
| 244033_at | CEP128 | 2,220484971 | 2,166264721 | 0,05422025 | |
| 211997_x_at | H3F3A /// H3F3B | 6,729643419 | 6,675478115 | 0,054165304 | |
| 218231_at | NAGK | 3,109000457 | 3,05486507 | 0,054135387 | |
| 225414_at | RNF149 | 3,433232044 | 3,379145507 | 0,054086537 | |
| 204441_s_at | POLA2 | 2,696781084 | 2,64270545 | 0,054075634 | |
| 200787_s_at | PEA15 | 1,850809354 | 1,796776588 | 0,054032767 | |
| 224450_s_at | RIOK1 | 4,256842836 | 4,202810939 | 0,054031897 | |
| 204427_s_at | TMED2 | 4,461505382 | 4,407512498 | 0,053992885 | |
| 201682_at | PMPCB | 4,79776819 | 4,743779817 | 0,053988373 | |
| 226091_s_at | MRFAP1 | 6,509226384 | 6,455258381 | 0,053968003 | |
| 204000_at | GNB5 | 0,533835206 | 0,479907041 | 0,053928165 | |
| 229514_at | C14orf118 | 0,533835206 | 0,479907041 | 0,053928165 | |
| 1555460_a_at | SLC39A6 | 1,119359127 | 1,065442793 | 0,053916334 | |
| 220558_x_at | TSPAN32 | 1,534677255 | 1,480766833 | 0,053910422 | |
| 225064_at | RABEP1 | 2,44295215 | 2,389070873 | 0,053881276 | |
| 205120_s_at | SGCB | 0,859074518 | 0,805216064 | 0,053858454 | |
| 222772_at | MYEF2 | 0,859074518 | 0,805216064 | 0,053858454 | |
| 222947_at | LOC100379224 | 0,859074518 | 0,805216064 | 0,053858454 | |
| 209085_x_at | RFC1 | 2,931053371 | 2,877228323 | 0,053825048 | |
| 221742_at | CELF1 | 2,630440446 | 2,576704359 | 0,053736087 | |
| 226431_at | FAM117B | 3,298397722 | 3,244672787 | 0,053724935 | |
| 202252_at | RAB13 | 5,271770432 | 5,218052609 | 0,053717823 | |
| 216570_x_at | - | 5,599558436 | 5,545849527 | 0,053708908 | |
| 200659_s_at | PHB | 4,099113343 | 4,045409633 | 0,05370371 | |
| 200018_at | LOC100508408 / | 7,798934377 | 7,745291843 | 0,053642534 | |
| 212493_s_at | SETD2 | 3,191547726 | 3,137910193 | 0,053637533 | |
| 1557656_at | LOC400238 | 0,133056859 | 0,079428135 | 0,053628723 | |
| 1560049_at | - | 0,133056859 | 0,079428135 | 0,053628723 | |
| 210106_at | BLOC1S1-RDH5 | 0,133056859 | 0,079428135 | 0,053628723 | |
| 1556283_s_at | FGFR1OP2 | 0,87091093 | 0,817300337 | 0,053610593 | |
| 202390_s_at | HTT | 0,87091093 | 0,817300337 | 0,053610593 | |
| 213931_at | ID2 /// ID2B | 0,87091093 | 0,817300337 | 0,053610593 | |
| 223289_s_at | USP38 | 2,250242994 | 2,196649505 | 0,053593489 | |
| 212629_s_at | PKN2 | 1,874520207 | 1,820984782 | 0,053535425 | |
| 217760_at | TRIM44 | 3,19743497 | 3,14392006 | 0,05351491 | |
| 202374_s_at | AURKAPS1 /// R/ | 2,361396186 | 2,307890888 | 0,053505298 | |
| 200012_x_at | RPL21 /// RPL21l | 7,878888799 | 7,825397356 | 0,053491443 | |
| 218592_s_at | CECR5 | 2,55541096 | 2,501934108 | 0,053476852 | |
| 218027_at | MRPL15 | 5,533838503 | 5,480385464 | 0,053453039 | |
| 238856_s_at | PANK2 | 1,369290453 | 1,315948728 | 0,053341725 | |
| 202388_at | RGS2 | 5,207586495 | 5,154281832 | 0,053304663 | |
| 218134_s_at | RBM22 | 4,236401075 | 4,183101476 | 0,053299599 | |
| 225676_s_at | DCAF13 | 6,085475914 | 6,032214748 | 0,053261166 | |
| 201759_at | TBCD | 0,888485433 | 0,835239052 | 0,053246381 | |
| 212830_at | MEGF9 | 0,888485433 | 0,835239052 | 0,053246381 | |
| 1566084_at | - | -0,43775896 | -0,490927335 | 0,053168375 | |

| | | | | | |
|---|---|---|---|---|---|
| 204055_s_at | CTAGE5 | -0,43775896 | -0,490927335 | 0,053168375 | |
| 221892_at | H6PD | -0,43775896 | -0,490927335 | 0,053168375 | |
| 240975_x_at | RBFOX3 | -0,43775896 | -0,490927335 | 0,053168375 | |
| 234156_at | - | 0,57058931 | 0,51742585 | 0,05316346 | |
| 238975_at | MMAB | 0,57058931 | 0,51742585 | 0,05316346 | |
| 227109_at | CYP2R1 | 3,383488593 | 3,330438773 | 0,05304982 | |
| 202873_at | ATP6V1C1 | 0,900083947 | 0,847075464 | 0,053008484 | |
| 220438_at | QPCTL | 0,900083947 | 0,847075464 | 0,053008484 | |
| 242371_x_at | - | 1,390002893 | 1,337086025 | 0,052916869 | |
| 230030_at | HS6ST2 | 2,976955384 | 2,924079008 | 0,052876376 | |
| 223259_at | ORMDL3 | 1,912238072 | 1,859476797 | 0,052761275 | |
| 223097_at | ADPRHL2 | 3,234554895 | 3,181801492 | 0,052753403 | |
| 204305_at | MIPEP | 1,05337236 | 1,000684521 | 0,052687838 | |
| 215038_s_at | SETD2 | 1,40228911 | 1,349621332 | 0,052667778 | |
| 222557_at | STMN3 | 3,595400101 | 3,542741726 | 0,052658374 | |
| 210063_at | SARDH | 0,181384709 | 0,128747141 | 0,052637569 | |
| 226401_at | PARP10 | 0,181384709 | 0,128747141 | 0,052637569 | |
| 239437_at | - | 0,181384709 | 0,128747141 | 0,052637569 | |
| 242826_at | - | 0,181384709 | 0,128747141 | 0,052637569 | |
| 226575_at | ZNF462 | 1,596577845 | 1,543943227 | 0,052634618 | |
| 213435_at | SATB2 | 4,154032218 | 4,101455318 | 0,0525769 | |
| 222552_at | GOLT1B | 3,245961699 | 3,193438316 | 0,052523383 | |
| 209345_s_at | PI4K2A | 3,462861802 | 3,410394103 | 0,052467699 | |
| 1569346_a_at | - | 0,606430281 | 0,553993624 | 0,052436657 | |
| 203151_at | MAP1A | 0,928678725 | 0,876248481 | 0,052430244 | |
| 222114_x_at | WDR55 | 0,928678725 | 0,876248481 | 0,052430244 | |
| 228883_at | TUB | 0,928678725 | 0,876248481 | 0,052430244 | |
| 225707_at | ARL6IP6 | 4,099113343 | 4,046693299 | 0,052420044 | |
| 202731_at | MIR4680 /// PDC | 3,251631457 | 3,199221717 | 0,05240974 | |
| 201821_s_at | TIMM17A | 4,987757501 | 4,935446463 | 0,052311038 | |
| 222848_at | CENPK | 3,420201053 | 3,367892567 | 0,052308486 | |
| 208636_at | ACTN1 | 6,105025837 | 6,05272386 | 0,052301977 | |
| 214211_at | FTH1 | 4,136978698 | 4,084681583 | 0,052297115 | |
| 202626_s_at | LYN | 4,341690695 | 4,289400623 | 0,052290072 | |
| 202323_s_at | ACBD3 | 1,614289682 | 1,562009954 | 0,052279728 | |
| 211713_x_at | KIAA0101 | 2,31665944 | 2,264418116 | 0,052241323 | |
| 222921_s_at | HEY2 | 1,940595304 | 1,888402606 | 0,052192698 | |
| 226563_at | SMAD2 | 1,940595304 | 1,888402606 | 0,052192698 | |
| 216231_s_at | B2M | 7,210913811 | 7,158791135 | 0,052122676 | |
| 226451_at | FDX1L | 4,55296954 | 4,500847519 | 0,052122022 | |
| 223306_at | EBPL | 5,194399796 | 5,142323515 | 0,052076281 | |
| 214951_at | SLC26A10 | 0,209623097 | 0,157549243 | 0,052073854 | |
| 223726_at | KCNH3 | 0,209623097 | 0,157549243 | 0,052073854 | |
| 228402_at | ZBED3 | 0,209623097 | 0,157549243 | 0,052073854 | |
| 239101_at | ITCH | 0,209623097 | 0,157549243 | 0,052073854 | |
| 239740_at | ETV6 | 0,209623097 | 0,157549243 | 0,052073854 | |
| 211355_x_at | LEPR | 1,62481334 | 1,572742379 | 0,052070961 | |
| 201694_s_at | EGR1 | 4,458566876 | 4,406512977 | 0,0520539 | |
| 226720_at | PWWP2A | 2,535098095 | 2,483094137 | 0,052003959 | |
| 201804_x_at | TBCB | 6,175319908 | 6,123320246 | 0,051999662 | |
| 208854_s_at | STK24 | 3,584658878 | 3,532699346 | 0,051959532 | |
| 225194_at | PLRG1 | 4,440809032 | 4,388908328 | 0,051900704 | |
| 211542_x_at | RPS10 | 8,200113584 | 8,14822477 | 0,051888814 | |
| 221549_at | GRWD1 | 2,804079361 | 2,752192409 | 0,051886952 | |
| 220387_s_at | HHLA3 | 0,95671774 | 0,904843258 | 0,051874482 | |
| 223049_at | GRB2 | 3,805796305 | 3,753943043 | 0,051853262 | |

| | | | | | |
|---|---|---|---|---|---|
| 201379_s_at | TPD52L2 | 4,128375743 | 4,076535251 | 0,051840492 | |
| 210456_at | PCYT1B | -0,366776884 | -0,41858459 | 0,051807706 | |
| 234811_at | CENPN | -0,366776884 | -0,41858459 | 0,051807706 | |
| 237993_at | CHCHD5 | -0,366776884 | -0,41858459 | 0,051807706 | |
| 242997_at | - | -0,366776884 | -0,41858459 | 0,051807706 | |
| 208930_s_at | ILF3 | 4,311608545 | 4,259820154 | 0,051788391 | |
| 218626_at | EIF4ENIF1 | 3,032022986 | 2,980244813 | 0,051778173 | |
| 203141_s_at | AP3B1 | 0,641402394 | 0,589657387 | 0,051745008 | |
| 205019_s_at | VIPR1 | 0,641402394 | 0,589657387 | 0,051745008 | |
| 222451_s_at | ZDHHC9 | 0,641402394 | 0,589657387 | 0,051745008 | |
| 241799_x_at | - | 0,641402394 | 0,589657387 | 0,051745008 | |
| 227000_at | C7orf41 | 0,228146222 | 0,176436073 | 0,051710148 | |
| 233941_at | C14orf166B | 0,228146222 | 0,176436073 | 0,051710148 | |
| 235226_at | CDK19 | 0,228146222 | 0,176436073 | 0,051710148 | |
| 218096_at | AGPAT5 | 6,113145999 | 6,061481705 | 0,051664294 | |
| 204617_s_at | ACD | 2,646022818 | 2,594367918 | 0,0516549 | |
| 221711_s_at | BABAM1 | 4,265835914 | 4,214280195 | 0,051555719 | |
| 218471_s_at | BBS1 | 0,97328325 | 0,921731975 | 0,051551275 | |
| 229926_at | MIR3682 | 0,97328325 | 0,921731975 | 0,051551275 | |
| 209497_s_at | RBM4B | 1,973904239 | 1,922365006 | 0,051539232 | |
| 210555_s_at | NFATC3 | 2,822481847 | 2,770953871 | 0,051527976 | |
| 226041_at | NAPEPLD | 1,462198073 | 1,410714371 | 0,051483702 | |
| 218896_s_at | C17orf85 | 2,562727117 | 2,511262629 | 0,051464488 | |
| 212165_at | TMEM183A /// TM | 4,179835245 | 4,128379201 | 0,051456044 | |
| 209861_s_at | METAP2 | 5,491099517 | 5,439680874 | 0,051418643 | |
| 223256_at | G2E3 | 2,745814053 | 2,694398145 | 0,051415908 | |
| 235198_at | OSTM1 | 1,982112682 | 1,930732138 | 0,051380545 | |
| 208867_s_at | CSNK1A1 | 3,789518787 | 3,738139229 | 0,051379558 | |
| 242335_at | SLC25A37 | 3,518475927 | 3,467101363 | 0,051374563 | |
| 213875_x_at | C6orf62 | 3,949468585 | 3,898162764 | 0,051305821 | |
| 202630_at | APPBP2 | 1,987559146 | 1,936283387 | 0,051275759 | |
| 202824_s_at | TCEB1 | 6,387174329 | 6,335907096 | 0,051267233 | |
| 224908_s_at | TTL | 1,666156729 | 1,614891062 | 0,051265667 | |
| 225370_at | PYGO2 | 1,251994932 | 1,200746069 | 0,051248863 | |
| 228929_at | DNASE1 | 0,255492549 | 0,204310755 | 0,051181794 | |
| 225480_at | C1orf122 | 3,715139632 | 3,663961904 | 0,051177727 | |
| 209989_at | ZNF268 | 1,841816345 | 1,790660532 | 0,051155813 | |
| 200761_s_at | ARL6IP5 | 3,842326541 | 3,79118048 | 0,051146061 | |
| 209149_s_at | TM9SF1 | 2,997346717 | 2,946258252 | 0,051088465 | |
| 213632_at | DHODH | 0,67554678 | 0,624460759 | 0,051086021 | |
| 221218_s_at | TPK1 | 0,67554678 | 0,624460759 | 0,051086021 | |
| 232520_s_at | NSFL1C | 4,653613789 | 4,602542772 | 0,051071017 | |
| 211445_x_at | NACAP1 | 4,830716841 | 4,779734028 | 0,050982813 | |
| 1552486_s_at | LACTB | 2,382221458 | 2,331254065 | 0,050967393 | |
| 201738_at | EIF1B | 6,032613215 | 5,981685722 | 0,050927492 | |
| 201987_at | MED13 | 4,006916639 | 3,956010056 | 0,050906583 | |
| 208929_x_at | RPL13 /// SNORD | 7,201350934 | 7,150456898 | 0,050894036 | |
| 206485_at | CD5 | 0,273439642 | 0,222599095 | 0,050840547 | |
| 209913_x_at | AP5Z1 | 0,273439642 | 0,222599095 | 0,050840547 | |
| 224035_s_at | BCL2L13 | 0,273439642 | 0,222599095 | 0,050840547 | |
| 232785_at | RGAG1 | 0,273439642 | 0,222599095 | 0,050840547 | |
| 242079_at | RGS12 | 0,273439642 | 0,222599095 | 0,050840547 | |
| 200620_at | TMEM59 | 4,181024227 | 4,130196752 | 0,050827475 | |
| 232221_x_at | RSBN1L | 1,274448124 | 1,223626587 | 0,050821537 | |
| 226331_at | BBX | 2,277177578 | 2,226407528 | 0,05077005 | |
| 209331_s_at | MAX | 3,647078281 | 3,596320795 | 0,050757486 | |

| | | | | | |
|---|---|---|---|---|---|
| 238765_at | ATP6V1G1 | 1,69307511 | 1,642321262 | 0,050753848 | |
| 231215_at | - | 2,503131904 | 2,452428893 | 0,050703011 | |
| 212462_at | KAT6B | 3,396853889 | 3,346162854 | 0,050691036 | |
| 231940_at | ZNF529 | 5,123506602 | 5,072831113 | 0,050675489 | |
| 203551_s_at | COX11 | 2,022468633 | 1,971855016 | 0,050613617 | |
| 207966_s_at | GLG1 | 2,022468633 | 1,971855016 | 0,050613617 | |
| 224332_s_at | MRPL43 | 4,482391326 | 4,431786907 | 0,050604419 | |
| 201184_s_at | CHD4 | 3,831009889 | 3,780418488 | 0,050591401 | |
| 208112_x_at | EHD1 | 2,609398637 | 2,558821855 | 0,050576783 | |
| 201820_at | KRT5 | -0,299034815 | -0,34958478 | 0,050549965 | |
| 204401_at | KCNN4 | -0,299034815 | -0,34958478 | 0,050549965 | |
| 211577_s_at | IGF1 | -0,299034815 | -0,34958478 | 0,050549965 | |
| 211637_x_at | IGH@ /// IGHA1 / | -0,299034815 | -0,34958478 | 0,050549965 | |
| 226489_at | TMCC3 | -0,299034815 | -0,34958478 | 0,050549965 | |
| 237345_at | - | -0,299034815 | -0,34958478 | 0,050549965 | |
| 240098_at | RIF1 | -0,299034815 | -0,34958478 | 0,050549965 | |
| 225433_at | GTF2A1 | 4,404623843 | 4,354078399 | 0,050545444 | |
| 210820_x_at | COQ7 | 1,512180019 | 1,461646549 | 0,05053347 | |
| 236918_s_at | LRRC34 | 1,027166904 | 0,976641161 | 0,050525743 | |
| 239240_at | - | 1,027166904 | 0,976641161 | 0,050525743 | |
| 204257_at | FADS3 | 2,030406107 | 1,979940779 | 0,050465329 | |
| 240592_at | LCORL | 2,030406107 | 1,979940779 | 0,050465329 | |
| 1555950_a_at | CD55 | 4,030853795 | 3,980396806 | 0,05045699 | |
| 218097_s_at | CUEDC2 | 3,410096504 | 3,359653127 | 0,050443377 | |
| 219428_s_at | PXMP4 | 3,170152481 | 3,119728903 | 0,050423578 | |
| 210754_s_at | LYN | 4,329411337 | 4,279061697 | 0,050349641 | |
| 203456_at | PRAF2 | 2,626954721 | 2,576704359 | 0,050250362 | |
| 234944_s_at | FAM54A | 2,890927111 | 2,840694075 | 0,050233035 | |
| 1552402_at | CALML6 | -1,812418401 | -1,862647763 | 0,050229361 | |
| 1554045_at | ZNF24 | -1,812418401 | -1,862647763 | 0,050229361 | |
| 1556663_s_at | LOC100506142 | -1,812418401 | -1,862647763 | 0,050229361 | |
| 1558608_a_at | PAPPA | -1,812418401 | -1,862647763 | 0,050229361 | |
| 1560832_at | - | -1,812418401 | -1,862647763 | 0,050229361 | |
| 1563379_at | - | -1,812418401 | -1,862647763 | 0,050229361 | |
| 1566702_at | - | -1,812418401 | -1,862647763 | 0,050229361 | |
| 206294_at | HSD3B2 | -1,812418401 | -1,862647763 | 0,050229361 | |
| 221624_at | TCL6 | -1,812418401 | -1,862647763 | 0,050229361 | |
| 224419_x_at | PMCHL1 | -1,812418401 | -1,862647763 | 0,050229361 | |
| 231307_at | - | -1,812418401 | -1,862647763 | 0,050229361 | |
| 232719_at | CELF4 | -1,812418401 | -1,862647763 | 0,050229361 | |
| 234744_x_at | - | -1,812418401 | -1,862647763 | 0,050229361 | |
| 235656_s_at | - | -1,812418401 | -1,862647763 | 0,050229361 | |
| 236993_at | - | -1,812418401 | -1,862647763 | 0,050229361 | |
| 237807_at | SPATA12 | -1,812418401 | -1,862647763 | 0,050229361 | |
| 241594_at | - | -1,812418401 | -1,862647763 | 0,050229361 | |
| 244040_at | KCNN3 | -1,812418401 | -1,862647763 | 0,050229361 | |
| 214784_x_at | XPO6 | 3,477451563 | 3,427247355 | 0,050204209 | |
| 206445_s_at | PRMT1 | 6,345364659 | 6,295174259 | 0,050190401 | |
| 209163_at | CYB561 | 1,726031811 | 1,675891488 | 0,050140323 | |
| 206994_at | CST4 | 0,317354302 | 0,26733076 | 0,050023542 | |
| 230018_at | DPP9 | 0,317354302 | 0,26733076 | 0,050023542 | |
| 1554833_at | MCTP2 | -1,688127507 | -1,738067356 | 0,049939849 | |
| 1561702_at | - | -1,688127507 | -1,738067356 | 0,049939849 | |
| 1562974_at | - | -1,688127507 | -1,738067356 | 0,049939849 | |
| 1567285_at | OR5L2 | -1,688127507 | -1,738067356 | 0,049939849 | |
| 206404_at | FGF9 | -1,688127507 | -1,738067356 | 0,049939849 | |

| | | | | | |
|---|---|---|---|---|---|
| 214396_s_at | MBD2 | -1,688127507 | -1,738067356 | 0,049939849 | |
| 215362_at | - | -1,688127507 | -1,738067356 | 0,049939849 | |
| 228371_s_at | - | -1,688127507 | -1,738067356 | 0,049939849 | |
| 232766_at | BANF2 | -1,688127507 | -1,738067356 | 0,049939849 | |
| 236963_at | - | -1,688127507 | -1,738067356 | 0,049939849 | |
| 238478_at | BNC2 | -1,688127507 | -1,738067356 | 0,049939849 | |
| 241096_at | - | -1,688127507 | -1,738067356 | 0,049939849 | |
| 242174_at | - | -1,688127507 | -1,738067356 | 0,049939849 | |
| 243448_at | - | -1,688127507 | -1,738067356 | 0,049939849 | |
| 243782_at | - | -1,688127507 | -1,738067356 | 0,049939849 | |
| 202722_s_at | GFPT1 | 3,260656977 | 3,210719429 | 0,049937548 | |
| 207769_s_at | PQBP1 | 4,570314367 | 4,520382831 | 0,049931536 | |
| 201937_s_at | DNPEP | 2,197169074 | 2,147243253 | 0,049925822 | |
| 1569180_at | - | -1,912771092 | -1,962679142 | 0,04990805 | |
| 212112_s_at | STX12 | 2,325288363 | 2,275409734 | 0,049878629 | |
| 223917_s_at | SLC39A3 | 4,455131038 | 4,405262601 | 0,049868436 | |
| 212201_at | ANKLE2 | 1,063722698 | 1,013870809 | 0,049851889 | |
| 214502_at | HIST1H2BJ | 1,742232072 | 1,692388143 | 0,049843929 | |
| 206182_at | ZNF134 | 2,64946283 | 2,599625088 | 0,049837742 | |
| 1556416_s_at | - | -2,236765673 | -2,286583451 | 0,049817778 | |
| 1556777_a_at | - | -2,236765673 | -2,286583451 | 0,049817778 | |
| 1557767_at | NANOS2 | -2,236765673 | -2,286583451 | 0,049817778 | |
| 1561669_at | - | -2,236765673 | -2,286583451 | 0,049817778 | |
| 1569042_at | LRP1 | -2,236765673 | -2,286583451 | 0,049817778 | |
| 1570052_at | MIPOL1 | -2,236765673 | -2,286583451 | 0,049817778 | |
| 213033_s_at | NFIB | -2,236765673 | -2,286583451 | 0,049817778 | |
| 213862_at | - | -2,236765673 | -2,286583451 | 0,049817778 | |
| 215257_at | ERGIC3 | -2,236765673 | -2,286583451 | 0,049817778 | |
| 216121_at | - | -2,236765673 | -2,286583451 | 0,049817778 | |
| 220293_at | CATSPERB | -2,236765673 | -2,286583451 | 0,049817778 | |
| 224459_at | L2HGDH | -2,236765673 | -2,286583451 | 0,049817778 | |
| 229829_at | LINC00526 | -2,236765673 | -2,286583451 | 0,049817778 | |
| 231425_at | PLCZ1 | -2,236765673 | -2,286583451 | 0,049817778 | |
| 233213_at | - | -2,236765673 | -2,286583451 | 0,049817778 | |
| 234320_at | CD244 | -2,236765673 | -2,286583451 | 0,049817778 | |
| 237126_at | - | -2,236765673 | -2,286583451 | 0,049817778 | |
| 238011_at | - | -2,236765673 | -2,286583451 | 0,049817778 | |
| 240250_at | - | -2,236765673 | -2,286583451 | 0,049817778 | |
| 240514_at | PGA3 | -2,236765673 | -2,286583451 | 0,049817778 | |
| 243360_at | - | -2,236765673 | -2,286583451 | 0,049817778 | |
| 1561048_at | RARS2 | 1,556829052 | 1,507116528 | 0,049712524 | |
| 209066_x_at | UQCRB | 6,465008917 | 6,415319716 | 0,049689201 | |
| 225081_s_at | CDCA7L | 2,840652551 | 2,791007185 | 0,049645366 | |
| 212578_x_at | RPS17 /// RPS17 | 7,928534296 | 7,878896491 | 0,049637805 | |
| 203307_at | GNL1 | 2,755423794 | 2,705818724 | 0,04960507 | |
| 37831_at | SIPA1L3 | 2,800989329 | 2,751405362 | 0,049583967 | |
| 209186_at | ATP2A2 | 4,280885759 | 4,231314909 | 0,04957085 | |
| 217930_s_at | TOLLIP | 1,758252436 | 1,708698295 | 0,049554141 | |
| 208744_x_at | HSPH1 | 4,082030648 | 4,032509762 | 0,049520886 | |
| 202282_at | HSD17B10 | 5,690863646 | 5,641343186 | 0,049520459 | |
| 209607_x_at | SLX1A-SULT1A3 | 1,567778694 | 1,518263577 | 0,049515116 | |
| 218611_at | IER5 | 3,406034785 | 3,356551164 | 0,049483621 | |
| 218824_at | PNMAL1 | 2,933877275 | 2,884425449 | 0,049451826 | |
| 211061_s_at | MGAT2 | 4,086478954 | 4,037037849 | 0,049441105 | |
| 202126_at | PRPF4B | 4,647605722 | 4,59817199 | 0,049433732 | |
| 1554589_at | LENG9 | -0,234278768 | -0,283667828 | 0,04938906 | |

| | | | | | |
|---|---|---|---|---|---|
| 202835_at | TXNL4A | -0,234278768 | -0,283667828 | 0,04938906 | |
| 216909_at | RRP12 | -0,234278768 | -0,283667828 | 0,04938906 | |
| 220923_s_at | PNMA3 | -0,234278768 | -0,283667828 | 0,04938906 | |
| 227009_at | - | -0,234278768 | -0,283667828 | 0,04938906 | |
| 227669_at | BRP44 | -0,234278768 | -0,283667828 | 0,04938906 | |
| 227839_at | MBD5 | -0,234278768 | -0,283667828 | 0,04938906 | |
| 230770_at | - | -0,234278768 | -0,283667828 | 0,04938906 | |
| 233008_at | LOC100507284 | -0,234278768 | -0,283667828 | 0,04938906 | |
| 235883_at | - | -0,234278768 | -0,283667828 | 0,04938906 | |
| 237144_at | LTBP3 | -0,234278768 | -0,283667828 | 0,04938906 | |
| 206653_at | POLR3G | 1,352503698 | 1,303116084 | 0,049387614 | |
| 227638_at | EPG5 | 1,575032557 | 1,525647381 | 0,049385176 | |
| 209384_at | PROSC | 4,495830712 | 4,446449692 | 0,04938102 | |
| 212379_at | GART | 4,196980309 | 4,147649355 | 0,049330954 | |
| 1558217_at | SLFN13 | 2,094906514 | 2,045615873 | 0,049290641 | |
| 228073_at | NANP | 3,902669169 | 3,853385784 | 0,049283384 | |
| 1559634_at | CHRM3 | -2,359436556 | -2,408705138 | 0,049268582 | |
| 1562230_at | - | -2,359436556 | -2,408705138 | 0,049268582 | |
| 1563656_at | - | -2,359436556 | -2,408705138 | 0,049268582 | |
| 1564985_a_at | SLC8A1 | -2,359436556 | -2,408705138 | 0,049268582 | |
| 208167_s_at | MMP16 | -2,359436556 | -2,408705138 | 0,049268582 | |
| 208350_at | CSN1S1 | -2,359436556 | -2,408705138 | 0,049268582 | |
| 211083_s_at | MAP3K13 | -2,359436556 | -2,408705138 | 0,049268582 | |
| 211606_at | - | -2,359436556 | -2,408705138 | 0,049268582 | |
| 212942_s_at | KIAA1199 | -2,359436556 | -2,408705138 | 0,049268582 | |
| 213438_at | NFASC | -2,359436556 | -2,408705138 | 0,049268582 | |
| 216827_at | - | -2,359436556 | -2,408705138 | 0,049268582 | |
| 222919_at | TRDN | -2,359436556 | -2,408705138 | 0,049268582 | |
| 222943_at | GBA3 | -2,359436556 | -2,408705138 | 0,049268582 | |
| 222969_at | B3GALT1 | -2,359436556 | -2,408705138 | 0,049268582 | |
| 223633_s_at | - | -2,359436556 | -2,408705138 | 0,049268582 | |
| 230563_at | RASGEF1A | -2,359436556 | -2,408705138 | 0,049268582 | |
| 231381_at | ESRG | -2,359436556 | -2,408705138 | 0,049268582 | |
| 231981_at | PRLR | -2,359436556 | -2,408705138 | 0,049268582 | |
| 233490_at | DCTN4 | -2,359436556 | -2,408705138 | 0,049268582 | |
| 236184_at | - | -2,359436556 | -2,408705138 | 0,049268582 | |
| 237972_at | - | -2,359436556 | -2,408705138 | 0,049268582 | |
| 240868_at | LOC100129406 | -2,359436556 | -2,408705138 | 0,049268582 | |
| 243321_at | - | -2,359436556 | -2,408705138 | 0,049268582 | |
| 1556053_at | - | 1,58584542 | 1,536652708 | 0,049192713 | |
| 219507_at | RSRC1 | 2,102456302 | 2,053299695 | 0,049156607 | |
| 226001_at | KLHL5 | 3,173740462 | 3,124599694 | 0,049140768 | |
| 218497_s_at | RNASEH1 | 3,641042878 | 3,591931093 | 0,049111785 | |
| 202345_s_at | FABP5 | 4,913162719 | 4,864065385 | 0,049097334 | |
| 204675_at | SRD5A1 | 2,369762403 | 2,320681318 | 0,049081085 | |
| 228129_at | SERBP1 | 2,107467633 | 2,058399602 | 0,049068032 | |
| 224754_at | SP1 | 2,874852734 | 2,825817328 | 0,049035406 | |
| 208905_at | CYCS | 6,673146317 | 6,624111969 | 0,049034348 | |
| 213274_s_at | CTSB | 1,373456798 | 1,324440845 | 0,049015953 | |
| 219060_at | WDYHV1 | 2,959046717 | 2,910041809 | 0,049004908 | |
| 201776_s_at | KIAA0494 | 2,250242994 | 2,201267805 | 0,048975189 | |
| 217768_at | C14orf166 | 6,723485451 | 6,674544283 | 0,048941168 | |
| 200770_s_at | LAMC1 | 2,380152406 | 2,331254065 | 0,048898341 | |
| 217499_x_at | OR7E37P | 1,965648824 | 1,916759838 | 0,048888987 | |
| 209430_at | BTAF1 | 3,657366303 | 3,608541373 | 0,04882493 | |
| 209219_at | MIR1236 /// RDB | 3,843828743 | 3,795004675 | 0,048824068 | |

| | | | | | |
|---|---|---|---|---|---|
| 212764_at | ZEB1 | 0,384950573 | 0,336136178 | 0,048814396 | |
| 213955_at | MYOZ3 | 0,384950573 | 0,336136178 | 0,048814396 | |
| 215068_s_at | FBXL18 | 0,384950573 | 0,336136178 | 0,048814396 | |
| 222624_s_at | ZNF639 | 2,261526912 | 2,212749286 | 0,048777625 | |
| 209549_s_at | DGUOK | 4,758484959 | 4,709745554 | 0,048739405 | |
| 203380_x_at | SRSF5 | 4,559384026 | 4,51064824 | 0,048735786 | |
| 218702_at | FBXO17 /// SARS | 2,713311265 | 2,664608955 | 0,04870231 | |
| 205130_at | MOK | 1,129248302 | 1,080561626 | 0,048686676 | |
| 222276_at | METTL2B | 1,129248302 | 1,080561626 | 0,048686676 | |
| 200740_s_at | SUMO3 | 6,50887134 | 6,460203967 | 0,048667373 | |
| 225367_at | PGM2 | 4,74286981 | 4,694266604 | 0,048603206 | |
| 225848_at | ZNF746 | 2,512607472 | 2,464004877 | 0,048602595 | |
| 203054_s_at | TCTA | 1,621313976 | 1,572742379 | 0,048571597 | |
| 214849_at | KCTD20 | 1,814495998 | 1,765933685 | 0,048562313 | |
| 206751_s_at | PCYT1B | 0,401366113 | 0,352836757 | 0,048529356 | |
| 219337_at | C1orf159 | 0,401366113 | 0,352836757 | 0,048529356 | |
| 229445_at | CYBA | 0,401366113 | 0,352836757 | 0,048529356 | |
| 202389_s_at | HTT | 1,139070151 | 1,090553537 | 0,048516614 | |
| 212203_x_at | IFITM3 | 6,224439428 | 6,175959986 | 0,048479442 | |
| 238974_at | C2orf69 | 4,952683603 | 4,904274741 | 0,048408861 | |
| 239377_at | EIF1AD | 2,911129742 | 2,862725455 | 0,048404287 | |
| 227428_at | GABPA | 2,52764011 | 2,479296438 | 0,048343672 | |
| 1563147_at | - | -0,172274514 | -0,220592223 | 0,04831771 | |
| 225742_at | MDM4 | -0,172274514 | -0,220592223 | 0,04831771 | |
| 231932_at | - | -0,172274514 | -0,220592223 | 0,04831771 | |
| 240608_at | - | -0,172274514 | -0,220592223 | 0,04831771 | |
| 217188_s_at | C14orf1 | 3,41414682 | 3,365837114 | 0,048309706 | |
| 200858_s_at | RPS8 /// SNORD | 6,865490682 | 6,817183247 | 0,048307435 | |
| 229129_at | HNRNPD | 2,151803104 | 2,103505212 | 0,048297892 | |
| 217979_at | TSPAN13 | 5,896110031 | 5,847814633 | 0,048295398 | |
| 227713_at | KATNAL1 | 1,638726528 | 1,590454215 | 0,048272312 | |
| 1554183_s_at | TRIM73 /// TRIM7 | 1,153678675 | 1,105412836 | 0,048265839 | |
| 1562002_at | MYOZ3 | 1,153678675 | 1,105412836 | 0,048265839 | |
| 202415_s_at | HSPBP1 | 3,158127685 | 3,109937707 | 0,048189978 | |
| 1557302_at | ZNF585B | 4,229522279 | 4,181349409 | 0,04817287 | |
| 217466_x_at | RPS2 /// SNORA | 5,732208132 | 5,684038418 | 0,048169714 | |
| 209033_s_at | DYRK1A | 5,247006265 | 5,198840377 | 0,048165888 | |
| 224643_at | PRRC1 | 4,232392431 | 4,18426834 | 0,048124091 | |
| 200068_s_at | CANX | 6,135703599 | 6,087596613 | 0,048106986 | |
| 1558775_s_at | NSMAF | 1,163336153 | 1,115234684 | 0,048101469 | |
| 202397_at | LOC128322 /// NU | 5,443592265 | 5,395529292 | 0,048062972 | |
| 213327_s_at | USP12 | 2,750626925 | 2,702564916 | 0,048062009 | |
| 224927_at | PPP1R18 | 3,702775533 | 3,654731437 | 0,048044095 | |
| 201270_x_at | NUDCD3 | 2,546213279 | 2,498185745 | 0,048027534 | |
| 201064_s_at | PABPC4 | 5,519575746 | 5,471571483 | 0,048004263 | |
| 235025_at | WDR89 | 1,655931423 | 1,60795124 | 0,047980183 | |
| 219940_s_at | PCID2 | 4,240968785 | 4,192989865 | 0,04797892 | |
| 1555553_a_at | SLC22A7 | -0,148196756 | -0,19610998 | 0,047913224 | |
| 1563842_at | PIGG | -0,148196756 | -0,19610998 | 0,047913224 | |
| 205291_at | IL2RB | -0,148196756 | -0,19610998 | 0,047913224 | |
| 217074_at | SMOX | -0,148196756 | -0,19610998 | 0,047913224 | |
| 237010_at | - | -0,148196756 | -0,19610998 | 0,047913224 | |
| 239804_at | - | -0,148196756 | -0,19610998 | 0,047913224 | |
| 238737_at | TCEANC2 | 0,853119696 | 0,805216064 | 0,047903632 | |
| 239078_at | BROX | 0,853119696 | 0,805216064 | 0,047903632 | |
| 242434_at | - | 0,853119696 | 0,805216064 | 0,047903632 | |

| | | | | | |
|---|---|---|---|---|---|
| 218031_s_at | FOXN3 | 1,85379461 | 1,805902329 | 0,047892281 | |
| 202092_s_at | ARL2BP | 3,024094427 | 2,976208448 | 0,047885979 | |
| 225361_x_at | FAM122B | 4,280885759 | 4,23300737 | 0,047878389 | |
| 230708_at | PRICKLE1 | 2,55541096 | 2,507538456 | 0,047872504 | |
| 204626_s_at | ITGB3 | 1,177702228 | 1,129843209 | 0,047859019 | |
| 207771_at | SLC5A2 | 0,44160593 | 0,393761504 | 0,047844426 | |
| 203303_at | DYNLT3 | 4,214504741 | 4,166665505 | 0,047839237 | |
| 235260_s_at | PACRGL | 2,027765132 | 1,979940779 | 0,047824353 | |
| 210878_s_at | KDM3B | 3,188003814 | 3,140317145 | 0,047686668 | |
| 1552322_at | FAM122C | -2,64318869 | -2,690860744 | 0,047672054 | |
| 1555581_a_at | TP63 | -2,64318869 | -2,690860744 | 0,047672054 | |
| 1556238_at | - | -2,64318869 | -2,690860744 | 0,047672054 | |
| 1558852_at | - | -2,64318869 | -2,690860744 | 0,047672054 | |
| 1561479_at | - | -2,64318869 | -2,690860744 | 0,047672054 | |
| 1563581_at | RPL34-AS1 | -2,64318869 | -2,690860744 | 0,047672054 | |
| 1564198_a_at | C10orf90 | -2,64318869 | -2,690860744 | 0,047672054 | |
| 1565826_at | - | -2,64318869 | -2,690860744 | 0,047672054 | |
| 1565833_at | - | -2,64318869 | -2,690860744 | 0,047672054 | |
| 1566271_x_at | RALGAPA1 | -2,64318869 | -2,690860744 | 0,047672054 | |
| 206290_s_at | RGS7 | -2,64318869 | -2,690860744 | 0,047672054 | |
| 211466_at | NFIB | -2,64318869 | -2,690860744 | 0,047672054 | |
| 214633_at | SOX3 | -2,64318869 | -2,690860744 | 0,047672054 | |
| 219764_at | FZD10 | -2,64318869 | -2,690860744 | 0,047672054 | |
| 224400_s_at | CHST9 | -2,64318869 | -2,690860744 | 0,047672054 | |
| 226281_at | DNER | -2,64318869 | -2,690860744 | 0,047672054 | |
| 227629_at | PRLR | -2,64318869 | -2,690860744 | 0,047672054 | |
| 228934_x_at | - | -2,64318869 | -2,690860744 | 0,047672054 | |
| 229494_s_at | PHLDA2 | -2,64318869 | -2,690860744 | 0,047672054 | |
| 231439_at | FAM84A | -2,64318869 | -2,690860744 | 0,047672054 | |
| 232444_at | CEP85L | -2,64318869 | -2,690860744 | 0,047672054 | |
| 234217_at | - | -2,64318869 | -2,690860744 | 0,047672054 | |
| 240186_at | - | -2,64318869 | -2,690860744 | 0,047672054 | |
| 240989_at | - | -2,64318869 | -2,690860744 | 0,047672054 | |
| 244678_at | - | -2,64318869 | -2,690860744 | 0,047672054 | |
| 244684_at | - | -2,64318869 | -2,690860744 | 0,047672054 | |
| 212296_at | PSMD14 | 5,497078594 | 5,449419352 | 0,047659242 | |
| 221735_at | WDR48 | 2,190100188 | 2,14244844 | 0,047651748 | |
| 225637_at | DEF8 | 2,190100188 | 2,14244844 | 0,047651748 | |
| 213258_at | TFPI | 4,916378816 | 4,868790444 | 0,047588372 | |
| 226193_x_at | CBWD1 /// CBWI | 5,403917855 | 5,35633864 | 0,047579215 | |
| 235484_at | PTAR1 | 1,874520207 | 1,826973888 | 0,047546318 | |
| 228264_at | ACCS | 1,04816921 | 1,000684521 | 0,047484689 | |
| 213385_at | CHN2 | 0,46522187 | 0,417770464 | 0,047451407 | |
| 227886_at | IFITM10 | 0,46522187 | 0,417770464 | 0,047451407 | |
| 1555174_at | - | -1,2845686 | -1,332017329 | 0,047448729 | |
| 1558768_at | DNAH1 | -1,2845686 | -1,332017329 | 0,047448729 | |
| 1563243_at | - | -1,2845686 | -1,332017329 | 0,047448729 | |
| 1564008_at | COL27A1 | -1,2845686 | -1,332017329 | 0,047448729 | |
| 201525_at | APOD | -1,2845686 | -1,332017329 | 0,047448729 | |
| 207708_at | ALOXE3 | -1,2845686 | -1,332017329 | 0,047448729 | |
| 208178_x_at | TRIO | -1,2845686 | -1,332017329 | 0,047448729 | |
| 209519_at | NCBP1 | -1,2845686 | -1,332017329 | 0,047448729 | |
| 214680_at | NTRK2 | -1,2845686 | -1,332017329 | 0,047448729 | |
| 215185_at | LOC100128077 / | -1,2845686 | -1,332017329 | 0,047448729 | |
| 221023_s_at | KCNH6 | -1,2845686 | -1,332017329 | 0,047448729 | |
| 222289_at | KCNC2 | -1,2845686 | -1,332017329 | 0,047448729 | |

| | | | | | |
|---|---|---|---|---|---|
| 224211_at | FOXP3 | -1,2845686 | -1,332017329 | 0,047448729 | |
| 226690_at | ADCYAP1R1 | -1,2845686 | -1,332017329 | 0,047448729 | |
| 228687_at | ZNF777 | -1,2845686 | -1,332017329 | 0,047448729 | |
| 229185_at | LIFR | -1,2845686 | -1,332017329 | 0,047448729 | |
| 231323_at | PSMB2 | -1,2845686 | -1,332017329 | 0,047448729 | |
| 237589_at | - | -1,2845686 | -1,332017329 | 0,047448729 | |
| 240705_at | CYP19A1 | -1,2845686 | -1,332017329 | 0,047448729 | |
| 241413_at | - | -1,2845686 | -1,332017329 | 0,047448729 | |
| 205768_s_at | SLC27A2 | 1,689737652 | 1,642321262 | 0,04741639 | |
| 1557105_a_at | ZSCAN30 | 0,882651021 | 0,835239052 | 0,047411969 | |
| 205390_s_at | ANK1 | 1,883312263 | 1,835911187 | 0,047401076 | |
| 214820_at | BRWD1 | 1,883312263 | 1,835911187 | 0,047401076 | |
| 203967_at | CDC6 | 3,205636958 | 3,158242412 | 0,047394546 | |
| 208966_x_at | IFI16 | 2,206540699 | 2,159161028 | 0,047379671 | |
| 227622_at | PCF11 | 2,791679321 | 2,744302548 | 0,047376774 | |
| 207425_s_at | 40057 | -0,112808516 | -0,160136748 | 0,047328232 | |
| 210827_s_at | ELF3 | -0,112808516 | -0,160136748 | 0,047328232 | |
| 212543_at | AIM1 | -0,112808516 | -0,160136748 | 0,047328232 | |
| 216371_at | - | -0,112808516 | -0,160136748 | 0,047328232 | |
| 230950_at | CNDP2 | -0,112808516 | -0,160136748 | 0,047328232 | |
| 237507_at | KRT73 | -0,112808516 | -0,160136748 | 0,047328232 | |
| 241956_at | - | -0,112808516 | -0,160136748 | 0,047328232 | |
| 242635_s_at | NAPEPLD | -0,112808516 | -0,160136748 | 0,047328232 | |
| 208800_at | SRP72 | 4,443783893 | 4,396479497 | 0,047304396 | |
| 222421_at | UBE2H | 2,59162628 | 2,544354523 | 0,047271757 | |
| 227037_at | PLD6 | 2,59162628 | 2,544354523 | 0,047271757 | |
| 209018_s_at | PINK1 | 1,215326747 | 1,16809119 | 0,047235557 | |
| 222651_s_at | TRPS1 | 1,215326747 | 1,16809119 | 0,047235557 | |
| 226220_at | METTL9 | 1,215326747 | 1,16809119 | 0,047235557 | |
| 209429_x_at | EIF2B4 | 3,938261899 | 3,891034012 | 0,047227887 | |
| 202356_s_at | GTF2F1 | 2,355089531 | 2,307890888 | 0,047198643 | |
| 223689_at | IGF2BP1 | 2,355089531 | 2,307890888 | 0,047198643 | |
| 202271_at | FBXO28 | 2,596981013 | 2,549796788 | 0,047184225 | |
| 200596_s_at | EIF3A | 4,108517049 | 4,061373942 | 0,047143107 | |
| 209665_at | CYB561D2 | 2,485917452 | 2,438805127 | 0,047112325 | |
| 213687_s_at | RPL35A | 7,38944077 | 7,342404279 | 0,047036491 | |
| 223105_s_at | TMEM14B /// TM | 6,931441452 | 6,884415932 | 0,04702552 | |
| 1553713_a_at | RHEBL1 | 2,609398637 | 2,562416132 | 0,046982505 | |
| 218940_at | METTL21D | 3,231115193 | 3,184136378 | 0,046978815 | |
| 226556_at | MAP3K13 | 3,231115193 | 3,184136378 | 0,046978815 | |
| 1556586_x_at | - | -0,089689931 | -0,136643983 | 0,046954051 | |
| 215087_at | C15orf39 | -0,089689931 | -0,136643983 | 0,046954051 | |
| 219637_at | ARMC9 | -0,089689931 | -0,136643983 | 0,046954051 | |
| 223385_at | CYP2S1 | -0,089689931 | -0,136643983 | 0,046954051 | |
| 234068_s_at | AP2A1 | -0,089689931 | -0,136643983 | 0,046954051 | |
| 204605_at | CGRRF1 | 2,371846398 | 2,324919719 | 0,046926679 | |
| 226757_at | IFIT2 | 3,086335384 | 3,039473081 | 0,046862303 | |
| 203174_s_at | ARFRP1 | 1,238353345 | 1,191491281 | 0,046862064 | |
| 201004_at | SSR4 | 5,531743008 | 5,484890723 | 0,046852286 | |
| 1566906_at | LOC100129722 | 0,503742507 | 0,456918297 | 0,04682421 | |
| 218310_at | KCTD7 /// RABGI | 2,830080806 | 2,783327323 | 0,046753482 | |
| 201507_at | PFDN1 | 3,731461974 | 3,684723168 | 0,046738806 | |
| 223585_x_at | KBTBD2 | 1,732533761 | 1,685812099 | 0,046721662 | |
| 206245_s_at | IVNS1ABP | 3,882346871 | 3,835672678 | 0,046674194 | |
| 223452_s_at | ATL3 | 3,251631457 | 3,204982027 | 0,04664943 | |
| 226793_at | LINC00294 | 1,515954005 | 1,469325098 | 0,046628907 | |

| | | | | | |
|---|---|---|---|---|---|
| 202100_at | RALB | 4,065387235 | 4,018839599 | 0,046547635 | |
| 1552677_a_at | DIP2A | 1,261018186 | 1,214517879 | 0,046500307 | |
| 225287_s_at | TMEM55B | 3,333038605 | 3,28656411 | 0,046474495 | |
| 202441_at | ERLIN1 | 3,985300536 | 3,938828971 | 0,046471565 | |
| 1555613_a_at | ZAP70 | -1,173767127 | -1,220210588 | 0,046443461 | |
| 1556374_s_at | LOC100506817 | -1,173767127 | -1,220210588 | 0,046443461 | |
| 1561269_at | RAPGEF5 | -1,173767127 | -1,220210588 | 0,046443461 | |
| 1564386_at | TXNDC8 | -1,173767127 | -1,220210588 | 0,046443461 | |
| 1570282_at | - | -1,173767127 | -1,220210588 | 0,046443461 | |
| 205595_at | DSG3 | -1,173767127 | -1,220210588 | 0,046443461 | |
| 208122_x_at | KIR2DS3 | -1,173767127 | -1,220210588 | 0,046443461 | |
| 209594_x_at | PSG9 | -1,173767127 | -1,220210588 | 0,046443461 | |
| 210381_s_at | CCKBR | -1,173767127 | -1,220210588 | 0,046443461 | |
| 220114_s_at | STAB2 | -1,173767127 | -1,220210588 | 0,046443461 | |
| 222139_at | ERV3-2 | -1,173767127 | -1,220210588 | 0,046443461 | |
| 231229_at | HILS1 | -1,173767127 | -1,220210588 | 0,046443461 | |
| 231592_at | TSIX | -1,173767127 | -1,220210588 | 0,046443461 | |
| 231838_at | PABPC1L | -1,173767127 | -1,220210588 | 0,046443461 | |
| 232456_at | C10orf71 | -1,173767127 | -1,220210588 | 0,046443461 | |
| 235624_at | HDLBP | -1,173767127 | -1,220210588 | 0,046443461 | |
| 235789_at | KDM4B /// LOC1( | -1,173767127 | -1,220210588 | 0,046443461 | |
| 239248_at | LOC100507495 | -1,173767127 | -1,220210588 | 0,046443461 | |
| 242188_at | - | -1,173767127 | -1,220210588 | 0,046443461 | |
| 210833_at | PTGER3 | -0,055687482 | -0,102100538 | 0,046413056 | |
| 225663_at | ACBD5 | 2,752227654 | 2,705818724 | 0,04640893 | |
| 202299_s_at | HBXIP | 5,321676926 | 5,275295809 | 0,046381117 | |
| 222532_at | SRPRB | 3,903389697 | 3,857048799 | 0,046340898 | |
| 234950_s_at | RFWD2 | 2,857110344 | 2,810785576 | 0,046324767 | |
| 202185_at | PLOD3 | 3,857278636 | 3,810956512 | 0,046322124 | |
| 37577_at | ARHGAP19 | 3,03794094 | 2,991620231 | 0,046320709 | |
| 226287_at | CCDC34 | 3,595400101 | 3,549096127 | 0,046303974 | |
| 219212_at | HSPA14 | 4,446258267 | 4,399999158 | 0,046259109 | |
| 212885_at | MPHOSPH10 | 3,998848507 | 3,952590169 | 0,046258338 | |
| 233274_at | - | 0,541261317 | 0,495031851 | 0,046229466 | |
| 204214_s_at | RAB32 | 2,864529541 | 2,81832103 | 0,046208512 | |
| 1569459_a_at | - | 2,544366685 | 2,498185745 | 0,046180941 | |
| 236923_x_at | - | 2,768138014 | 2,721978587 | 0,046159427 | |
| 224484_s_at | BRMS1L | 1,283332461 | 1,23718272 | 0,046149741 | |
| 224217_s_at | FAF1 | 3,868391882 | 3,822243651 | 0,04614823 | |
| 219002_at | FASTKD1 | 3,869867201 | 3,823741953 | 0,046125248 | |
| 202703_at | DUSP11 | 3,285189903 | 3,239069096 | 0,046120807 | |
| 210935_s_at | WDR1 | 3,288503211 | 3,242433922 | 0,04606929 | |
| 1562841_at | LOC339666 | -0,033456564 | -0,079522948 | 0,046066384 | |
| 1563816_at | GAB4 | -0,033456564 | -0,079522948 | 0,046066384 | |
| 1569868_s_at | EME2 | -0,033456564 | -0,079522948 | 0,046066384 | |
| 213380_x_at | MST1P9 | -0,033456564 | -0,079522948 | 0,046066384 | |
| 215540_at | - | -0,033456564 | -0,079522948 | 0,046066384 | |
| 238501_at | - | -0,033456564 | -0,079522948 | 0,046066384 | |
| 240294_at | HIPK2 | -0,033456564 | -0,079522948 | 0,046066384 | |
| 225507_at | PNISR | 3,667581479 | 3,621520989 | 0,046060491 | |
| 1569594_a_at | NEMF | 3,923419763 | 3,877391565 | 0,046028197 | |
| 1558836_at | - | 1,292162421 | 1,246149888 | 0,046012533 | |
| 219854_at | ZNF14 | 1,292162421 | 1,246149888 | 0,046012533 | |
| 228722_at | PRMT2 | 2,671625168 | 2,625627364 | 0,045997804 | |
| 202041_s_at | FIBP | 3,831009889 | 3,785040604 | 0,045969285 | |
| 223088_x_at | ECHDC1 | 4,400036207 | 4,354078399 | 0,045957808 | |

| | | | | | |
|---|---|---|---|---|---|
| 227421_at | YBEY | 3,729025365 | 3,683073218 | 0,045952147 | |
| 210996_s_at | YWHAE | 5,144381006 | 5,098433773 | 0,045947233 | |
| 218773_s_at | MSRB2 | 3,062018438 | 3,016072743 | 0,045945695 | |
| 225375_at | MIR4723 /// TME | 2,680059304 | 2,634191677 | 0,045867627 | |
| 219253_at | TMEM185B | 2,886560922 | 2,840694075 | 0,045866846 | |
| 201298_s_at | MOB1A | 4,192270951 | 4,146452482 | 0,045818469 | |
| 209164_s_at | CYB561 | 1,305306849 | 1,259496994 | 0,045809855 | |
| 208683_at | CAPN2 | 6,196198265 | 6,150440013 | 0,045758252 | |
| 1554384_at | PADI2 | -2,908843709 | -2,954576755 | 0,045733046 | |
| 1554751_at | - | -2,908843709 | -2,954576755 | 0,045733046 | |
| 1555097_a_at | PTGFR | -2,908843709 | -2,954576755 | 0,045733046 | |
| 1556095_at | UNC13C | -2,908843709 | -2,954576755 | 0,045733046 | |
| 1561364_at | - | -2,908843709 | -2,954576755 | 0,045733046 | |
| 1563357_at | - | -2,908843709 | -2,954576755 | 0,045733046 | |
| 1565875_at | - | -2,908843709 | -2,954576755 | 0,045733046 | |
| 1567859_at | - | -2,908843709 | -2,954576755 | 0,045733046 | |
| 1570306_at | ST18 | -2,908843709 | -2,954576755 | 0,045733046 | |
| 207599_at | MMP20 | -2,908843709 | -2,954576755 | 0,045733046 | |
| 216007_at | - | -2,908843709 | -2,954576755 | 0,045733046 | |
| 216719_s_at | - | -2,908843709 | -2,954576755 | 0,045733046 | |
| 219857_at | PLEKHS1 | -2,908843709 | -2,954576755 | 0,045733046 | |
| 230319_at | - | -2,908843709 | -2,954576755 | 0,045733046 | |
| 234844_at | ZNF407 | -2,908843709 | -2,954576755 | 0,045733046 | |
| 236262_at | MMRN2 | -2,908843709 | -2,954576755 | 0,045733046 | |
| 236870_at | IQCF4 | -2,908843709 | -2,954576755 | 0,045733046 | |
| 237664_at | - | -2,908843709 | -2,954576755 | 0,045733046 | |
| 239129_at | - | -2,908843709 | -2,954576755 | 0,045733046 | |
| 240949_x_at | - | -2,908843709 | -2,954576755 | 0,045733046 | |
| 241547_at | A1CF | -2,908843709 | -2,954576755 | 0,045733046 | |
| 243632_at | - | -2,908843709 | -2,954576755 | 0,045733046 | |
| 244067_x_at | - | -2,908843709 | -2,954576755 | 0,045733046 | |
| 203139_at | DAPK1 | 4,796992917 | 4,751278918 | 0,045713999 | |
| 210097_s_at | NOL7 | 5,975086044 | 5,929409519 | 0,045676525 | |
| 243267_x_at | EFNA2 | 0,57782909 | 0,532164371 | 0,04566472 | |
| 211615_s_at | LRPPRC | 5,020671303 | 4,975089315 | 0,045581988 | |
| 243829_at | BRAF | -0,00073712 | -0,046302147 | 0,045565027 | |
| 222821_s_at | GEMIN7 | 3,647078281 | 3,601570866 | 0,045507414 | |
| 213271_s_at | DOPEY1 | 2,173470163 | 2,127967638 | 0,045502525 | |
| 221619_s_at | MTCH1 | 6,144263096 | 6,098778216 | 0,04548488 | |
| 227520_at | TXLNG | 4,362800281 | 4,317323511 | 0,04547677 | |
| 242429_at | ZNF567 | 2,59519831 | 2,549796788 | 0,045401522 | |
| 204439_at | IFI44L | 3,097712429 | 3,052311115 | 0,045401314 | |
| 225363_at | PTEN | 4,437828024 | 4,392446485 | 0,045381538 | |
| 203791_at | DMXL1 | 4,099113343 | 4,053733117 | 0,045380226 | |
| 224467_s_at | PDCD2L | 4,567134896 | 4,52176814 | 0,045366756 | |
| 225762_x_at | RN45S | 3,405017566 | 3,359653127 | 0,045364439 | |
| 200006_at | PARK7 | 6,967976658 | 6,922628453 | 0,045348205 | |
| 221860_at | HNRNPL | 2,922548342 | 2,877228323 | 0,045320019 | |
| 212149_at | EFR3A | 4,206061174 | 4,160749839 | 0,045311336 | |
| 41856_at | UNC5B | 2,104964143 | 2,059671766 | 0,045292377 | |
| 200943_at | HMGN1 | 7,475901967 | 7,430655592 | 0,045246375 | |
| 1552274_at | PXK | 0,020670649 | -0,024572586 | 0,045243236 | |
| 1554193_s_at | MANEA | 0,020670649 | -0,024572586 | 0,045243236 | |
| 1566908_at | C9orf173 | 0,020670649 | -0,024572586 | 0,045243236 | |
| 207356_at | DEFB4A /// DEFE | 0,020670649 | -0,024572586 | 0,045243236 | |
| 213283_s_at | SALL2 | 0,020670649 | -0,024572586 | 0,045243236 | |

| | | | | | |
|---|---|---|---|---|---|
| 232162_at | ZNF696 | 0,020670649 | -0,024572586 | 0,045243236 | |
| 232736_s_at | POM121L9P | 0,020670649 | -0,024572586 | 0,045243236 | |
| 207727_s_at | MUTYH | 1,021868205 | 0,976641161 | 0,045227044 | |
| 210547_x_at | ICA1 | 1,021868205 | 0,976641161 | 0,045227044 | |
| 212236_x_at | JUP /// KRT17 | 0,613492853 | 0,568365095 | 0,045127758 | |
| 211933_s_at | HNRNPA3 /// HN | 5,438132931 | 5,39300832 | 0,045124611 | |
| 202136_at | ZMYND11 | 3,421207626 | 3,376085218 | 0,045122408 | |
| 209362_at | MED21 | 2,72965419 | 2,684536601 | 0,045117589 | |
| 226149_at | SNHG11 /// SNO | 2,72965419 | 2,684536601 | 0,045117589 | |
| 226723_at | CCDC23 | 2,030406107 | 1,98530622 | 0,045099887 | |
| 229582_at | INO80C | 4,893714114 | 4,848693177 | 0,045020937 | |
| 1598_g_at | GAS6 | 1,498892774 | 1,453926914 | 0,04496586 | |
| 228217_s_at | PSMG4 | 5,084646091 | 5,039691895 | 0,044954196 | |
| 218926_at | MYNN | 2,043538949 | 1,998633167 | 0,044905782 | |
| 211072_x_at | LOC100288366 / | 7,743452596 | 7,698590113 | 0,044862483 | |
| 203047_at | STK10 | 1,369290453 | 1,324440845 | 0,044849607 | |
| 213805_at | ABHD5 | 1,369290453 | 1,324440845 | 0,044849607 | |
| 206154_at | RLBP1 | 0,052199481 | 0,007421914 | 0,044777567 | |
| 207042_at | E2F2 | 0,052199481 | 0,007421914 | 0,044777567 | |
| 208198_x_at | KIR2DS1 | 0,052199481 | 0,007421914 | 0,044777567 | |
| 212707_s_at | RASA4 /// RASA4 | 0,052199481 | 0,007421914 | 0,044777567 | |
| 214643_x_at | BIN1 | 0,052199481 | 0,007421914 | 0,044777567 | |
| 226212_s_at | INSR | 0,052199481 | 0,007421914 | 0,044777567 | |
| 229663_at | LONP2 | 0,052199481 | 0,007421914 | 0,044777567 | |
| 235729_at | ZNF514 | 0,052199481 | 0,007421914 | 0,044777567 | |
| 242245_at | - | 0,052199481 | 0,007421914 | 0,044777567 | |
| 200898_s_at | MGEA5 | 3,375201825 | 3,330438773 | 0,044763053 | |
| 225651_at | UBE2E2 | 3,75400739 | 3,709248632 | 0,044758758 | |
| 227280_s_at | CCNYL1 | 2,37600538 | 2,331254065 | 0,044751315 | |
| 218421_at | CERK | 1,64218396 | 1,59747851 | 0,04470545 | |
| 231846_at | FOXRED2 | 1,64218396 | 1,59747851 | 0,04470545 | |
| 218042_at | COPS4 | 4,657462947 | 4,612762285 | 0,044700662 | |
| 207941_s_at | RBM39 | 5,55003372 | 5,505342242 | 0,044691479 | |
| 203501_at | CPQ | 2,380152406 | 2,335461563 | 0,044690843 | |
| 209895_at | PTPN11 | 1,865674241 | 1,820984782 | 0,044689459 | |
| 215966_x_at | GK3P | 1,381753566 | 1,337086025 | 0,044667542 | |
| 1553579_a_at | SPAG11A /// SPA | -3,054236653 | -3,098858316 | 0,044621664 | |
| 1553886_at | CCDC108 | -3,054236653 | -3,098858316 | 0,044621664 | |
| 1557437_a_at | - | -3,054236653 | -3,098858316 | 0,044621664 | |
| 1569712_at | - | -3,054236653 | -3,098858316 | 0,044621664 | |
| 1570578_at | - | -3,054236653 | -3,098858316 | 0,044621664 | |
| 204111_at | HNMT | -3,054236653 | -3,098858316 | 0,044621664 | |
| 204469_at | PTPRZ1 | -3,054236653 | -3,098858316 | 0,044621664 | |
| 206693_at | IL7 | -3,054236653 | -3,098858316 | 0,044621664 | |
| 215441_at | - | -3,054236653 | -3,098858316 | 0,044621664 | |
| 215962_at | - | -3,054236653 | -3,098858316 | 0,044621664 | |
| 220504_at | KERA | -3,054236653 | -3,098858316 | 0,044621664 | |
| 231790_at | DMGDH | -3,054236653 | -3,098858316 | 0,044621664 | |
| 234432_at | - | -3,054236653 | -3,098858316 | 0,044621664 | |
| 234583_at | CHL1 | -3,054236653 | -3,098858316 | 0,044621664 | |
| 237489_at | - | -3,054236653 | -3,098858316 | 0,044621664 | |
| 238378_at | - | -3,054236653 | -3,098858316 | 0,044621664 | |
| 243847_at | - | -3,054236653 | -3,098858316 | 0,044621664 | |
| 208812_x_at | HLA-C | 6,771732655 | 6,727231231 | 0,044501424 | |
| 222297_x_at | - | 1,394109935 | 1,349621332 | 0,044488603 | |
| 203236_s_at | LGALS9 | 0,072842263 | 0,028364014 | 0,044478248 | |

| | | | | | |
|---|---|---|---|---|---|
| 210239_at | IRX5 | 0,072842263 | 0,028364014 | 0,044478248 | |
| 238827_at | - | 0,072842263 | 0,028364014 | 0,044478248 | |
| 214116_at | BTD | 1,073999308 | 1,029536893 | 0,044462415 | |
| 202644_s_at | TNFAIP3 | 1,659347917 | 1,614891062 | 0,044456856 | |
| 212620_at | ZNF609 | 1,659347917 | 1,614891062 | 0,044456856 | |
| 205928_at | ZNF443 | 3,325531666 | 3,281121096 | 0,04441057 | |
| 213322_at | C6orf130 | 3,249366227 | 3,204982027 | 0,0443842 | |
| 229173_at | KIAA1715 | 1,40228911 | 1,3579181 | 0,044371009 | |
| 52975_at | FAM125B | -0,325601563 | -0,369933821 | 0,044332258 | |
| 218844_at | ACSF2 | 1,894952274 | 1,85068474 | 0,044267533 | |
| 218378_s_at | LOC100630923 / | 2,410880861 | 2,366632651 | 0,04424821 | |
| 225928_at | VTI1B | 1,67631007 | 1,632095957 | 0,044214114 | |
| 208720_s_at | RBM39 | 5,113889166 | 5,069677524 | 0,044211642 | |
| 224712_x_at | C19orf42 | 1,414471577 | 1,370274469 | 0,044197108 | |
| 219938_s_at | PSTPIP2 | 2,678376417 | 2,634191677 | 0,04418474 | |
| 1567035_at | - | 0,682279745 | 0,638150376 | 0,044129369 | |
| 229337_at | USP2 | 0,682279745 | 0,638150376 | 0,044129369 | |
| 218797_s_at | SIRT7 | 2,097427503 | 2,053299695 | 0,044127807 | |
| 219007_at | NUP43 | 5,345732318 | 5,301609855 | 0,044122463 | |
| 201241_at | DDX1 | 5,786920447 | 5,742834983 | 0,044085464 | |
| 202424_at | MAP2K2 | 3,492853048 | 3,448769219 | 0,044083829 | |
| 214679_x_at | GNA11 | 2,102456302 | 2,058399602 | 0,0440567 | |
| 1553153_at | ATP6V0D2 | 0,103263601 | 0,059218869 | 0,044044733 | |
| 205975_s_at | HOXD1 | 0,103263601 | 0,059218869 | 0,044044733 | |
| 209558_s_at | HIP1R | 0,103263601 | 0,059218869 | 0,044044733 | |
| 216275_at | BUB1 | 0,103263601 | 0,059218869 | 0,044044733 | |
| 232548_at | GALNTL1 | 0,103263601 | 0,059218869 | 0,044044733 | |
| 217916_s_at | FAM49B | 4,838649416 | 4,794709145 | 0,043940271 | |
| 226004_at | CABLES2 | 2,281618189 | 2,237691445 | 0,043926744 | |
| 203584_at | EMC2 | 4,539590968 | 4,495687112 | 0,043903856 | |
| 218387_s_at | PGLS | 3,436222554 | 3,392332256 | 0,043890298 | |
| 213589_s_at | B3GNTL1 | 0,702292136 | 0,658444279 | 0,043847857 | |
| 209193_at | PIM1 | 1,442503549 | 1,398700978 | 0,04380257 | |
| 224509_s_at | RTN4IP1 | 2,44295215 | 2,39915583 | 0,04379632 | |
| 1565660_at | FUT6 | 0,123193849 | 0,079428135 | 0,043765714 | |
| 208364_at | INPP4A | 0,123193849 | 0,079428135 | 0,043765714 | |
| 220186_s_at | CDHR2 | 0,123193849 | 0,079428135 | 0,043765714 | |
| 228651_at | VWA1 | 0,123193849 | 0,079428135 | 0,043765714 | |
| 236080_at | LOC100507670 | 0,123193849 | 0,079428135 | 0,043765714 | |
| 240391_at | NDUFB2 | 0,123193849 | 0,079428135 | 0,043765714 | |
| 205078_at | PIGF | 2,827046023 | 2,783327323 | 0,0437187 | |
| 218276_s_at | SAV1 | 2,127340679 | 2,083632167 | 0,043708512 | |
| 216591_s_at | SDHC | 3,299492934 | 3,255815291 | 0,043677643 | |
| 224394_at | RNF7 | 0,715481152 | 0,67181667 | 0,043664482 | |
| 225140_at | KLF3 | 1,454352496 | 1,410714371 | 0,043638125 | |
| 201134_x_at | COX7C | 6,963362865 | 6,919741536 | 0,04362133 | |
| 224850_at | ATAD1 | 3,992767659 | 3,949162156 | 0,043605504 | |
| 227921_at | - | 3,043834719 | 3,000258961 | 0,043575758 | |
| 228846_at | MXD1 | 2,724770722 | 2,681234383 | 0,043536339 | |
| 206492_at | FHIT | 1,462198073 | 1,418668082 | 0,043529991 | |
| 210191_s_at | PHTF1 | 1,462198073 | 1,418668082 | 0,043529991 | |
| 227776_at | ACER3 | 2,462640593 | 2,419116683 | 0,04352391 | |
| 1553180_at | ADAMTS19 | -3,192553003 | -3,236070201 | 0,043517198 | |
| 1553519_at | LINC00314 | -3,192553003 | -3,236070201 | 0,043517198 | |
| 1560075_at | - | -3,192553003 | -3,236070201 | 0,043517198 | |
| 1561021_at | - | -3,192553003 | -3,236070201 | 0,043517198 | |

| | | | | | |
|---|---|---|---|---|---|
| 1563173_at | - | -3,192553003 | -3,236070201 | 0,043517198 | |
| 206415_at | TLL1 | -3,192553003 | -3,236070201 | 0,043517198 | |
| 207815_at | PF4V1 | -3,192553003 | -3,236070201 | 0,043517198 | |
| 211093_at | PDE6C | -3,192553003 | -3,236070201 | 0,043517198 | |
| 220700_at | - | -3,192553003 | -3,236070201 | 0,043517198 | |
| 240167_at | LOC152742 | -3,192553003 | -3,236070201 | 0,043517198 | |
| 241188_at | - | -3,192553003 | -3,236070201 | 0,043517198 | |
| 221532_s_at | WDR61 | 5,534071148 | 5,49056154 | 0,043509608 | |
| 225468_at | PATL1 | 2,466546243 | 2,423075926 | 0,043470318 | |
| 225730_s_at | THUMPD3 | 2,466546243 | 2,423075926 | 0,043470318 | |
| 1556301_at | LOC100287015 | 0,735041319 | 0,691645685 | 0,043395634 | |
| 212423_at | ZCCHC24 | 0,735041319 | 0,691645685 | 0,043395634 | |
| 241538_at | CXorf30 | 0,735041319 | 0,691645685 | 0,043395634 | |
| 238004_at | PGBD2 | 1,735773781 | 1,692388143 | 0,043385638 | |
| 213286_at | ZFR | 1,473887017 | 1,43051703 | 0,043369987 | |
| 232658_at | - | 0,152582607 | 0,109221392 | 0,043361215 | |
| 236513_at | - | 0,152582607 | 0,109221392 | 0,043361215 | |
| 243888_at | - | 0,152582607 | 0,109221392 | 0,043361215 | |
| 223312_at | PRADC1 | 2,47626436 | 2,432926755 | 0,043337605 | |
| 57739_at | DND1 | 1,859746653 | 1,816476579 | 0,043270074 | |
| 239442_at | CEP68 | 0,747935635 | 0,704715219 | 0,043220416 | |
| 200772_x_at | LOC100506248 / | 7,514083259 | 7,470895038 | 0,043188222 | |
| 201380_at | CRTAP /// LOC1( | 2,166283906 | 2,123108223 | 0,043175683 | |
| 209390_at | TSC1 | 2,491678455 | 2,448549499 | 0,043128957 | |
| 218965_s_at | TUT1 | 0,171847695 | 0,128747141 | 0,043100555 | |
| 221201_s_at | ZNF155 | 0,171847695 | 0,128747141 | 0,043100555 | |
| 233300_at | - | 0,171847695 | 0,128747141 | 0,043100555 | |
| 221449_s_at | ITFG1 | 3,343695493 | 3,300620584 | 0,04307491 | |
| 223245_at | STRBP | 2,876321464 | 2,833274877 | 0,043046587 | |
| 212367_at | FEM1B | 3,085065712 | 3,042049843 | 0,043015869 | |
| 212040_at | TGOLN2 | 1,987559146 | 1,944570415 | 0,042988731 | |
| 1557649_at | GALE | 0,767063567 | 0,724100169 | 0,042963398 | |
| 206015_s_at | FOXJ3 | 3,885996912 | 3,843079586 | 0,042917326 | |
| 1294_at | UBA7 | 0,186131111 | 0,143220285 | 0,042910826 | |
| 210655_s_at | FOXO3 /// FOXO | 1,187200746 | 1,144305291 | 0,042895455 | |
| 200645_at | GABARAP | 4,887900851 | 4,845008859 | 0,042891993 | |
| 37020_at | CRP | -2,572231155 | -2,61510413 | 0,042872975 | |
| 208758_at | ATIC | 5,001282527 | 4,958476321 | 0,042806206 | |
| 222525_s_at | CCDC25 | 4,194038762 | 4,151234028 | 0,042804734 | |
| 204523_at | ZNF140 | 2,516380342 | 2,473581079 | 0,042799262 | |
| 212567_s_at | MAP4 | 0,779676066 | 0,73688026 | 0,042795805 | |
| 232680_at | HDGFL1 | 0,779676066 | 0,73688026 | 0,042795805 | |
| 243918_at | - | 0,779676066 | 0,73688026 | 0,042795805 | |
| 225797_at | MRPL54 | 3,367911651 | 3,325159096 | 0,042752555 | |
| 209240_at | OGT | 4,642867483 | 4,600140481 | 0,042727002 | |
| 206351_s_at | PEX10 | 0,20027154 | 0,157549243 | 0,042722297 | |
| 239763_at | LOC100507338 / | 0,20027154 | 0,157549243 | 0,042722297 | |
| 226319_s_at | ALYREF | 5,661137055 | 5,618433519 | 0,042703536 | |
| 201774_s_at | NCAPD2 | 2,201862497 | 2,159161028 | 0,042701469 | |
| 222719_s_at | PDGFC | 2,525769574 | 2,483094137 | 0,042675438 | |
| 213656_s_at | KLC1 | 4,253455927 | 4,210848982 | 0,042606945 | |
| 208025_s_at | HMGA2 | 3,45992606 | 3,417357537 | 0,042568524 | |
| 210686_x_at | SLC25A16 | 3,534378463 | 3,491815843 | 0,04256262 | |
| 229470_at | - | 0,798390451 | 0,755840599 | 0,042549852 | |
| 218504_at | FAHD2A | 2,915422315 | 2,87289275 | 0,042529565 | |
| 207108_s_at | NIPBL | 2,027765132 | 1,98530622 | 0,042458912 | |

| | | | | | |
|---|---|---|---|---|---|
| 222745_s_at | C15orf29 | 2,027765132 | 1,98530622 | 0,042458912 | |
| 1554572_a_at | SUV39H2 | 3,127619759 | 3,08516499 | 0,042454768 | |
| 208845_at | VDAC3 | 6,530195776 | 6,487755648 | 0,042440127 | |
| 219166_at | DNAAF2 | 4,648465551 | 4,606029888 | 0,042435662 | |
| 234915_s_at | DENR | 3,130084275 | 3,087661492 | 0,042422783 | |
| 203733_at | DEXI | 2,225103271 | 2,182705233 | 0,042398038 | |
| 200766_at | CTSD | 0,810733188 | 0,768343791 | 0,042389397 | |
| 227437_at | LOC100506668 | 0,810733188 | 0,768343791 | 0,042389397 | |
| 211316_x_at | CFLAR | 2,232003119 | 2,189694216 | 0,042308903 | |
| 210422_x_at | SLC11A1 | 1,233777334 | 1,191491281 | 0,042286053 | |
| 227745_at | LOC100507018 | 1,233777334 | 1,191491281 | 0,042286053 | |
| 224737_x_at | CCAR1 | 2,559073676 | 2,516830926 | 0,042242751 | |
| 226020_s_at | DAB1 /// OMA1 | 2,238870125 | 2,196649505 | 0,04222062 | |
| 200947_s_at | GLUD1 | 4,527022536 | 4,484837154 | 0,042185382 | |
| 222037_at | MCM4 | 3,634114217 | 3,591931093 | 0,042183123 | |
| 221539_at | EIF4EBP1 | 4,914235548 | 4,872052597 | 0,042182952 | |
| 213044_at | ROCK1 | 3,943876123 | 3,901713973 | 0,04216215 | |
| 202304_at | FNDC3A | 3,243687542 | 3,201528602 | 0,04215894 | |
| 200848_at | AHCYL1 | 3,152077493 | 3,109937707 | 0,042139785 | |
| 207025_at | GJC2 | 0,246434561 | 0,204310755 | 0,042123805 | |
| 213700_s_at | - | 0,246434561 | 0,204310755 | 0,042123805 | |
| 215556_at | - | 0,246434561 | 0,204310755 | 0,042123805 | |
| 230673_at | PKHD1L1 | 0,246434561 | 0,204310755 | 0,042123805 | |
| 243736_at | - | 0,246434561 | 0,204310755 | 0,042123805 | |
| 223384_s_at | TRIM4 | 2,947914474 | 2,905803877 | 0,042110597 | |
| 219031_s_at | NIP7 | 4,006916639 | 3,964864009 | 0,04205263 | |
| 220927_s_at | HPSE2 | 1,578645857 | 1,536652708 | 0,041993149 | |
| 219281_at | MSRA | 2,580856819 | 2,538891651 | 0,041965168 | |
| 223433_at | YAE1D1 | 2,074578432 | 2,032717854 | 0,041860578 | |
| 224916_at | TMEM173 | 2,074578432 | 2,032717854 | 0,041860578 | |
| 218367_x_at | USP21 | 1,85379461 | 1,811954245 | 0,041840365 | |
| 216693_x_at | HDGFRP3 | 2,272723257 | 2,230931691 | 0,041791566 | |
| 224926_at | EXOC4 | 3,182077878 | 3,140317145 | 0,041760733 | |
| 1552264_a_at | MAPK1 | 2,450859799 | 2,409170778 | 0,04168902 | |
| 201021_s_at | DSTN | 5,325445321 | 5,28375775 | 0,041687571 | |
| 208899_x_at | ATP6V1D | 4,566680113 | 4,524995365 | 0,041684749 | |
| 200935_at | CALR | 1,603688695 | 1,562009954 | 0,04167874 | |
| 202827_s_at | MMP14 | 0,291166226 | 0,249604176 | 0,04156205 | |
| 225776_at | RBMS2 | 0,291166226 | 0,249604176 | 0,04156205 | |
| 214075_at | NENF | 1,614289682 | 1,572742379 | 0,041547303 | |
| 220238_s_at | KLHL7 | 1,614289682 | 1,572742379 | 0,041547303 | |
| 233868_x_at | ADAM33 | 1,614289682 | 1,572742379 | 0,041547303 | |
| 201881_s_at | ARIH1 | 2,882181479 | 2,840694075 | 0,041487404 | |
| 228723_at | - | 1,883312263 | 1,841838775 | 0,041473488 | |
| 211801_x_at | MFN1 | 1,621313976 | 1,579853228 | 0,041460748 | |
| 40829_at | WDTC1 | 2,301433511 | 2,259997914 | 0,041435597 | |
| 212929_s_at | FAM21A /// FAM2 | 2,623460554 | 2,582026171 | 0,041434382 | |
| 211968_s_at | HSP90AA1 | 7,405519523 | 7,364125898 | 0,041393625 | |
| 208068_x_at | CSH1 /// CSHL1 / | 0,30867765 | 0,26733076 | 0,04134689 | |
| 221985_at | KLHL24 | 0,30867765 | 0,26733076 | 0,04134689 | |
| 231873_at | BMPR2 | 0,30867765 | 0,26733076 | 0,04134689 | |
| 204332_s_at | AGA | 1,631786706 | 1,590454215 | 0,041332491 | |
| 201098_at | COPB2 | 4,872528643 | 4,831294479 | 0,041234164 | |
| 216977_x_at | SNRPA1 | 4,206061174 | 4,164893351 | 0,041167824 | |
| 223055_s_at | XPO5 | 2,132266459 | 2,091116676 | 0,041149783 | |
| 223157_at | NOA1 | 3,327680494 | 3,28656411 | 0,041116384 | |

| | | | | | |
|---|---|---|---|---|---|
| 222899_at | ITGA11 | 0,917308734 | 0,876248481 | 0,041060253 | |
| 1566671_a_at | PDXK | 0,334552598 | 0,293518836 | 0,041033763 | |
| 213758_at | COX4I1 | 0,334552598 | 0,293518836 | 0,041033763 | |
| 219464_at | CA14 | 0,334552598 | 0,293518836 | 0,041033763 | |
| 224252_s_at | FXYD5 | 5,343875661 | 5,302953328 | 0,040922333 | |
| 204137_at | GPR137B | 2,344516784 | 2,303602088 | 0,040914696 | |
| 1555301_a_at | DIP2A | 2,666540916 | 2,625627364 | 0,040913553 | |
| 205996_s_at | AK2 | 2,929639344 | 2,888726553 | 0,04091279 | |
| 212948_at | CAMTA2 | 0,934330293 | 0,893473268 | 0,040857025 | |
| 219344_at | SLC29A3 | 0,934330293 | 0,893473268 | 0,040857025 | |
| 1553584_at | CXorf36 | 0,351548291 | 0,310717132 | 0,040831159 | |
| 239816_at | POLD3 | 0,351548291 | 0,310717132 | 0,040831159 | |
| 210376_x_at | ELK1 | 2,81177561 | 2,770953871 | 0,040821739 | |
| 210250_x_at | ADSL | 5,06851491 | 5,027715959 | 0,040798951 | |
| 204566_at | PPM1D | 2,355089531 | 2,314300277 | 0,040789254 | |
| 230972_at | ANKRD9 | 3,358484112 | 3,317734938 | 0,040749175 | |
| 238177_at | SLC6A19 | 0,945567441 | 0,904843258 | 0,040724183 | |
| 226392_at | RASA2 | 3,067171967 | 3,026519779 | 0,040652188 | |
| 205542_at | STEAP1 | 3,274090262 | 3,233443554 | 0,040646708 | |
| 222806_s_at | C11orf30 | 1,689737652 | 1,64909809 | 0,040639561 | |
| 201696_at | SRSF4 | 4,226646405 | 4,186016868 | 0,040629537 | |
| 203150_at | RABEPK | 2,954882186 | 2,914267328 | 0,040614858 | |
| 228026_at | SIKE1 | 2,371846398 | 2,331254065 | 0,040592334 | |
| 201666_at | TIMP1 | 8,08296092 | 8,042372185 | 0,040588735 | |
| 218270_at | MRPL24 | 3,279650757 | 3,239069096 | 0,040581662 | |
| 202537_s_at | CHMP2B | 3,373122675 | 3,332545245 | 0,04057743 | |
| 207717_s_at | PKP2 | 0,376672223 | 0,336136178 | 0,040536046 | |
| 219005_at | TMEM59L | 0,376672223 | 0,336136178 | 0,040536046 | |
| 239269_at | LOC100653017 / | 0,376672223 | 0,336136178 | 0,040536046 | |
| 202060_at | CTR9 | 4,290281671 | 4,249823666 | 0,040458005 | |
| 205480_s_at | UGP2 | 4,706176435 | 4,665728361 | 0,040448074 | |
| 204331_s_at | MRPS12 | 6,114235615 | 6,073811461 | 0,040424154 | |
| 218088_s_at | RRAGC | 3,193905507 | 3,153484076 | 0,040421432 | |
| 221706_s_at | USE1 | 2,194816625 | 2,154405725 | 0,0404109 | |
| 1552812_a_at | SENP1 | 0,97328325 | 0,932882274 | 0,040400976 | |
| 228799_at | LOC100507239 | 0,97328325 | 0,932882274 | 0,040400976 | |
| 202915_s_at | FAM20B | 3,389672581 | 3,349287209 | 0,040385372 | |
| 204115_at | GNG11 | 1,390002893 | 1,349621332 | 0,040381561 | |
| 212118_at | TRIM27 | 3,391728033 | 3,351366359 | 0,040361674 | |
| 217968_at | TSSC1 | 2,721505869 | 2,681234383 | 0,040271486 | |
| 213461_at | NUDT21 | 2,402750389 | 2,362515214 | 0,040235175 | |
| 211296_x_at | UBC | 7,60939942 | 7,569187982 | 0,040211438 | |
| 201248_s_at | SREBF2 | 1,000476627 | 0,960386719 | 0,040089908 | |
| 221946_at | C9orf116 | 1,000476627 | 0,960386719 | 0,040089908 | |
| 227954_at | ITPRIPL2 | 1,000476627 | 0,960386719 | 0,040089908 | |
| 239091_at | - | 1,000476627 | 0,960386719 | 0,040089908 | |
| 221592_at | TBC1D8 | 0,417596971 | 0,377530647 | 0,040066324 | |
| 219816_s_at | RBM23 | 3,665883965 | 3,625821706 | 0,040062259 | |
| 218669_at | RAP2C | 3,941773349 | 3,901713973 | 0,040059376 | |
| 201570_at | SAMM50 | 4,511983428 | 4,471995246 | 0,039988182 | |
| 200757_s_at | CALU | 4,918518904 | 4,878554857 | 0,039964047 | |
| 204265_s_at | GPSM3 | 1,748661581 | 1,708698295 | 0,039963286 | |
| 222678_s_at | DCUN1D1 | 2,429008604 | 2,389070873 | 0,039937731 | |
| 1563753_at | BPI /// LOC14968 | -0,57445847 | -0,614371577 | 0,039913106 | |
| 209637_s_at | RGS12 | -0,57445847 | -0,614371577 | 0,039913106 | |
| 230266_at | RAB7B | -0,57445847 | -0,614371577 | 0,039913106 | |

| | | | | | |
|---|---|---|---|---|---|
| 231137_at | IDH3A | -0,57445847 | -0,614371577 | 0,039913106 | |
| 231949_at | - | -0,57445847 | -0,614371577 | 0,039913106 | |
| 237131_at | RIIAD1 | -0,57445847 | -0,614371577 | 0,039913106 | |
| 239224_at | FBXL20 | -0,57445847 | -0,614371577 | 0,039913106 | |
| 243420_at | - | -0,57445847 | -0,614371577 | 0,039913106 | |
| 226572_at | SOCS7 | 1,758252436 | 1,718396606 | 0,039855831 | |
| 231870_s_at | NMD3 | 4,895888063 | 4,856033707 | 0,039854356 | |
| 230811_at | C16orf55 | 1,027166904 | 0,987376598 | 0,039790307 | |
| 243224_at | - | 1,027166904 | 0,987376598 | 0,039790307 | |
| 212809_at | NFATC2IP | 3,034656185 | 2,994948944 | 0,039707241 | |
| 218656_s_at | LHFP | 2,259277178 | 2,21959457 | 0,039682607 | |
| 204219_s_at | PSMC1 | 6,128326249 | 6,088687525 | 0,039638725 | |
| 209825_s_at | MIR3658 /// UCK | 3,456984333 | 3,417357537 | 0,039626796 | |
| 206343_s_at | NRG1 | 0,457392764 | 0,417770464 | 0,039622301 | |
| 213038_at | RNF19B | 0,457392764 | 0,417770464 | 0,039622301 | |
| 212057_at | KIAA0182 | 2,787001691 | 2,747463672 | 0,039538019 | |
| 234302_s_at | ALKBH5 /// LOC1 | 3,713497195 | 3,673964604 | 0,039532591 | |
| 213420_at | DHX57 | 1,789769151 | 1,750261389 | 0,039507762 | |
| 214749_s_at | ARMCX6 | 4,67908296 | 4,639594721 | 0,039488239 | |
| 203905_at | PARN | 3,726584632 | 3,687194562 | 0,039390071 | |
| 209659_s_at | CDC16 | 4,485762928 | 4,446449692 | 0,039313236 | |
| 222994_at | PRDX5 | 4,396969658 | 4,357702907 | 0,039266752 | |
| 216031_x_at | HN1L | 2,2992452 | 2,259997914 | 0,039247287 | |
| 210775_x_at | CASP9 | 1,814495998 | 1,775255987 | 0,039240011 | |
| 230194_at | LRPPRC | 2,814842651 | 2,775606361 | 0,03923629 | |
| 1553263_at | USH1G | 1,079110292 | 1,039887232 | 0,039223061 | |
| 224079_at | IL17C | 1,079110292 | 1,039887232 | 0,039223061 | |
| 226357_at | USP19 | 3,302773593 | 3,263564154 | 0,039209439 | |
| 201344_at | UBE2D2 | 0,496120222 | 0,456918297 | 0,039201925 | |
| 219198_at | GTF3C4 | 2,503131904 | 2,464004877 | 0,039127027 | |
| 208629_s_at | HADHA | 3,410096504 | 3,370970266 | 0,039126238 | |
| 201114_x_at | PSMA7 | 6,387560618 | 6,348452607 | 0,039108011 | |
| 1556385_at | - | -0,496991414 | -0,536087151 | 0,039095737 | |
| 204086_at | PRAME | -0,496991414 | -0,536087151 | 0,039095737 | |
| 212668_at | SMURF1 | -0,496991414 | -0,536087151 | 0,039095737 | |
| 228924_s_at | UBE2J1 | -0,496991414 | -0,536087151 | 0,039095737 | |
| 233089_at | QRSL1 | -0,496991414 | -0,536087151 | 0,039095737 | |
| 1557961_s_at | LOC100127983 | 2,314494121 | 2,275409734 | 0,039084386 | |
| 218220_at | C12orf10 | 2,314494121 | 2,275409734 | 0,039084386 | |
| 220926_s_at | EDEM3 | 2,967339888 | 2,928263672 | 0,039076217 | |
| 235197_s_at | OSTM1 | 0,518867317 | 0,479907041 | 0,038960276 | |
| 236453_at | - | 0,518867317 | 0,479907041 | 0,038960276 | |
| 1553269_at | ZNF718 | 1,104397092 | 1,065442793 | 0,038954299 | |
| 209158_s_at | CYTH2 | 2,109966786 | 2,071071048 | 0,038895738 | |
| 202758_s_at | RFXANK | 3,702775533 | 3,663961904 | 0,038813628 | |
| 225909_at | LOC728743 /// ZN | 0,533835206 | 0,495031851 | 0,038803356 | |
| 216520_s_at | TPT1 | 7,50610055 | 7,46730472 | 0,03879583 | |
| 64486_at | CORO1B | 3,058788109 | 3,019999252 | 0,038788857 | |
| 202656_s_at | SERTAD2 | 1,859746653 | 1,820984782 | 0,038761871 | |
| 223548_at | SWT1 | 1,859746653 | 1,820984782 | 0,038761871 | |
| 37793_r_at | RAD51D | -0,437435603 | -0,476186332 | 0,03875073 | |
| 201084_s_at | BCLAF1 | 5,968301546 | 5,929583479 | 0,038718067 | |
| 201237_at | CAPZA2 | 5,298309737 | 5,25960603 | 0,038703708 | |
| 205788_s_at | ZC3H11A | 4,674447146 | 4,635761472 | 0,038685674 | |
| 238653_at | - | 2,352981171 | 2,314300277 | 0,038680894 | |
| 227611_at | TARSL2 | 2,546213279 | 2,507538456 | 0,038674823 | |

| | | | | | |
|---|---|---|---|---|---|
| 203401_at | PRPS2 | 4,404623843 | 4,365953461 | 0,038670383 | |
| 204084_s_at | CLN5 | 2,549899391 | 2,511262629 | 0,038636762 | |
| 210425_x_at | GOLGA8A /// GO | 2,549899391 | 2,511262629 | 0,038636762 | |
| 208775_at | XPO1 | 5,552332553 | 5,513720861 | 0,038611692 | |
| 203241_at | UVRAG | 1,874520207 | 1,835911187 | 0,03860902 | |
| 226835_s_at | ZNFX1-AS1 | 6,477703621 | 6,439097679 | 0,038605943 | |
| 231110_at | - | 0,555999837 | 0,51742585 | 0,038573986 | |
| 232177_at | - | 0,555999837 | 0,51742585 | 0,038573986 | |
| 238065_at | TPM3 | 0,555999837 | 0,51742585 | 0,038573986 | |
| 201437_s_at | EIF4E | 4,443783893 | 4,405262601 | 0,038521292 | |
| 223021_x_at | VTA1 | 4,317567252 | 4,279061697 | 0,038505555 | |
| 201644_at | TSTA3 | 3,812716554 | 3,77423254 | 0,038484013 | |
| 209087_x_at | MCAM | 4,657462947 | 4,619032017 | 0,03843093 | |
| 1553221_at | ZNF583 | 0,57058931 | 0,532164371 | 0,03842494 | |
| 227891_s_at | TAF15 | 0,57058931 | 0,532164371 | 0,03842494 | |
| 213531_s_at | RAB3GAP1 | 4,723824182 | 4,685434648 | 0,038389534 | |
| 232150_at | - | 1,897847661 | 1,859476797 | 0,038370864 | |
| 201398_s_at | TRAM1 | 5,162251088 | 5,12389408 | 0,038357008 | |
| 206459_s_at | WNT2B | -0,423248115 | -0,461594427 | 0,038346311 | |
| 206630_at | TYR | -0,423248115 | -0,461594427 | 0,038346311 | |
| 209885_at | RHOD | -0,423248115 | -0,461594427 | 0,038346311 | |
| 229130_at | - | -0,423248115 | -0,461594427 | 0,038346311 | |
| 230353_at | LOC284112 /// M | -0,423248115 | -0,461594427 | 0,038346311 | |
| 232555_at | CREB5 | -0,423248115 | -0,461594427 | 0,038346311 | |
| 236150_at | AGPHD1 | -0,423248115 | -0,461594427 | 0,038346311 | |
| 242913_at | CLIC6 | -0,423248115 | -0,461594427 | 0,038346311 | |
| 209239_at | NFKB1 | 1,903621061 | 1,865308541 | 0,03831252 | |
| 226510_at | HEATR5A | 2,752227654 | 2,713921281 | 0,038306373 | |
| 229106_at | DYNLL2 | 1,168140757 | 1,129843209 | 0,038297548 | |
| 1558143_a_at | BCL2L11 | 2,908260915 | 2,869995113 | 0,038265802 | |
| 213646_x_at | LOC100288366 / | 7,942053631 | 7,903813681 | 0,03823995 | |
| 232682_at | MREG | 1,593009234 | 1,55481039 | 0,038198844 | |
| 220486_x_at | TMEM164 | 2,400710591 | 2,362515214 | 0,038195378 | |
| 230738_at | - | 0,606430281 | 0,568365095 | 0,038065186 | |
| 64440_at | IL17RC | 0,606430282 | 0,568365096 | 0,038065186 | |
| 221265_s_at | C15orf44 | 2,933877275 | 2,895866687 | 0,038010588 | |
| 225120_at | PURB | 2,933877275 | 2,895866687 | 0,038010588 | |
| 214201_x_at | PRRC2A | 2,790121796 | 2,752192409 | 0,037929387 | |
| 235121_at | ZNF542 | 2,943717636 | 2,905803877 | 0,037913759 | |
| 205353_s_at | PEBP1 | 4,430348443 | 4,392446485 | 0,037901958 | |
| 221090_s_at | OGFOD1 | 2,947914474 | 2,910041809 | 0,037872665 | |
| 214471_x_at | LHB | 0,627515113 | 0,589657387 | 0,037857726 | |
| 200869_at | RPL18A | 7,752578285 | 7,714729755 | 0,03784853 | |
| 207069_s_at | SMAD6 | 1,215326747 | 1,177496821 | 0,037829927 | |
| 235424_at | - | 1,215326747 | 1,177496821 | 0,037829927 | |
| 235457_at | MAML2 | 1,215326747 | 1,177496821 | 0,037829927 | |
| 223847_s_at | ERGIC1 | 2,220484971 | 2,182705233 | 0,037779738 | |
| 212687_at | LIMS1 | 5,846393664 | 5,808627689 | 0,037765974 | |
| 225844_at | POLE4 | 4,161279609 | 4,123521175 | 0,037758434 | |
| 214110_s_at | - | 1,960118853 | 1,922365006 | 0,037753847 | |
| 242560_at | FANCD2 | 3,098971025 | 3,061230246 | 0,037740779 | |
| 1552663_a_at | ERC1 | 0,641402394 | 0,603679647 | 0,037722748 | |
| 210203_at | CNOT4 | 0,641402394 | 0,603679647 | 0,037722748 | |
| 214169_at | SUN1 | 0,641402394 | 0,603679647 | 0,037722748 | |
| 219047_s_at | ZNF668 | 0,641402394 | 0,603679647 | 0,037722748 | |
| 226663_at | ANKRD10 /// ANK | 0,641402394 | 0,603679647 | 0,037722748 | |

| | | | | | |
|---|---|---|---|---|---|
| 227682_at | - | 0,641402394 | 0,603679647 | 0,037722748 | |
| 213908_at | WHAMMP2 /// W | -0,352951799 | -0,39061235 | 0,037660552 | |
| 215788_at | KIAA1751 | -0,352951799 | -0,39061235 | 0,037660552 | |
| 228424_at | NAALADL1 | -0,352951799 | -0,39061235 | 0,037660552 | |
| 229515_at | PAWR | -0,352951799 | -0,39061235 | 0,037660552 | |
| 226018_at | C7orf41 | 2,817903185 | 2,780243895 | 0,03765929 | |
| 217863_at | PIAS1 | 2,825526235 | 2,787940144 | 0,037586091 | |
| 200028_s_at | STARD7 | 5,441361398 | 5,403817384 | 0,037544014 | |
| 213474_at | KCTD7 /// RABGI | 0,661985843 | 0,624460759 | 0,037525083 | |
| 236615_at | - | 0,661985843 | 0,624460759 | 0,037525083 | |
| 238519_at | DDI2 /// RSC1A1 | 2,250242994 | 2,212749286 | 0,037493708 | |
| 217973_at | DCXR | 4,309979162 | 4,272493466 | 0,037485697 | |
| 212406_s_at | PCMTD2 | 4,190500971 | 4,15302303 | 0,037477941 | |
| 235244_at | CCDC58 | 2,47626436 | 2,438805127 | 0,037459233 | |
| 209288_s_at | CDC42EP3 | 3,917011805 | 3,879554231 | 0,037457574 | |
| 204827_s_at | CCNF | 0,67554678 | 0,638150376 | 0,037396404 | |
| 214335_at | RPL18 | 0,67554678 | 0,638150376 | 0,037396404 | |
| 229325_at | ZZZ3 | 1,261018186 | 1,223626587 | 0,037391598 | |
| 243747_at | ZNF599 | 1,261018186 | 1,223626587 | 0,037391598 | |
| 224859_at | CD276 | 2,485917452 | 2,448549499 | 0,037367953 | |
| 1552670_a_at | PPP1R3B | 1,274448124 | 1,23718272 | 0,037265404 | |
| 1555088_x_at | STAT5B | 1,274448124 | 1,23718272 | 0,037265404 | |
| 211363_s_at | MTAP | 1,274448124 | 1,23718272 | 0,037265404 | |
| 214764_at | RRP15 | 1,274448124 | 1,23718272 | 0,037265404 | |
| 224952_at | TANC2 | 1,274448124 | 1,23718272 | 0,037265404 | |
| 208833_s_at | ATXN10 | 5,574216947 | 5,536971059 | 0,037245888 | |
| 226159_at | C5orf51 | 3,017453847 | 2,980244813 | 0,037209034 | |
| 238711_s_at | ZNF148 | 0,695652136 | 0,658444279 | 0,037207857 | |
| 224928_at | SETD7 | 1,283332461 | 1,246149888 | 0,037182572 | |
| 239295_at | SRSF12 | 1,283332461 | 1,246149888 | 0,037182572 | |
| 218291_at | LAMTOR2 | 3,161745659 | 3,124599694 | 0,037145965 | |
| 211653_x_at | AKR1C2 /// LOC1 | 3,61576656 | 3,578681277 | 0,037085283 | |
| 223377_x_at | CISH | 1,296557217 | 1,259496994 | 0,037060223 | |
| 202874_s_at | ATP6V1C1 | 4,712768283 | 4,675718863 | 0,03704942 | |
| 1563229_at | DLEU2 | -0,285837228 | -0,322870281 | 0,037033053 | |
| 211416_x_at | GGTLC1 | -0,285837228 | -0,322870281 | 0,037033053 | |
| 215395_x_at | PRSS3P2 | -0,285837228 | -0,322870281 | 0,037033053 | |
| 230371_at | HPS6 | -0,285837228 | -0,322870281 | 0,037033053 | |
| 233006_at | 38231 | -0,285837228 | -0,322870281 | 0,037033053 | |
| 200802_at | SARS | 4,473685138 | 4,436691079 | 0,036994059 | |
| 55872_at | ZNF512B | 3,765149338 | 3,728173203 | 0,036976135 | |
| 212041_at | ATP6V0D1 | 4,311065622 | 4,274138329 | 0,036927293 | |
| 1554516_at | LINC00537 | 0,728550685 | 0,691645685 | 0,036905 | |
| 218010_x_at | PPDPF | 0,728550685 | 0,691645685 | 0,036905 | |
| 212497_at | MAPK1IP1L | 1,318332599 | 1,281471383 | 0,036861216 | |
| 229751_s_at | PUS7L | 1,318332599 | 1,281471383 | 0,036861216 | |
| 218777_at | REEP4 | 2,90394695 | 2,867091644 | 0,036855305 | |
| 212010_s_at | CDV3 | 5,825066914 | 5,788244042 | 0,036822872 | |
| 202098_s_at | PRMT2 | 4,496785894 | 4,460001797 | 0,036784098 | |
| 210817_s_at | CALCOCO2 | 3,444167073 | 3,407399455 | 0,036767619 | |
| 226360_at | ZNRF3 | 1,331241794 | 1,294497133 | 0,036744661 | |
| 210596_at | MAGT1 | 2,750626925 | 2,713921281 | 0,036705643 | |
| 211855_s_at | SLC25A14 | 2,077135162 | 2,040470494 | 0,036664667 | |
| 227064_at | ANKRD40 | 2,082235068 | 2,045615873 | 0,036619195 | |
| 1563853_at | LOC283045 | 0,760715727 | 0,724100169 | 0,036615557 | |
| 200897_s_at | PALLD | 0,760715727 | 0,724100169 | 0,036615557 | |

| | | | | | |
|---|---|---|---|---|---|
| 239549_at | - | 0,760715727 | 0,724100169 | 0,036615557 | |
| 1555760_a_at | RBM15 | 3,467741468 | 3,431184377 | 0,036557091 | |
| 205441_at | OCEL1 | 2,089851283 | 2,053299695 | 0,036551588 | |
| 222804_x_at | DCAF10 | 2,089851283 | 2,053299695 | 0,036551588 | |
| 212439_at | IP6K1 | 2,580856819 | 2,544354523 | 0,036502295 | |
| 219352_at | HERC6 | 2,580856819 | 2,544354523 | 0,036502295 | |
| 1562695_at | FOXN4 | -0,221656139 | -0,258114234 | 0,036458095 | |
| 204150_at | STAB1 | -0,221656139 | -0,258114234 | 0,036458095 | |
| 206746_at | BFSP1 | -0,221656139 | -0,258114234 | 0,036458095 | |
| 212611_at | DTX4 | -0,221656139 | -0,258114234 | 0,036458095 | |
| 222847_s_at | EGLN3 | -0,221656139 | -0,258114234 | 0,036458095 | |
| 229918_at | CCDC40 | -0,221656139 | -0,258114234 | 0,036458095 | |
| 231054_at | SPACA4 | -0,221656139 | -0,258114234 | 0,036458095 | |
| 233719_s_at | TASP1 | -0,221656139 | -0,258114234 | 0,036458095 | |
| 240328_at | - | -0,221656139 | -0,258114234 | 0,036458095 | |
| 208878_s_at | PAK2 | 3,101484927 | 3,065035915 | 0,036449012 | |
| 200656_s_at | P4HB | 4,376876617 | 4,340535652 | 0,036340965 | |
| 212264_s_at | WAPAL | 4,040056475 | 4,003717379 | 0,036339095 | |
| 215741_x_at | AKAP8L | 0,792179257 | 0,755840599 | 0,036338657 | |
| 206855_s_at | HYAL2 | 2,114952142 | 2,078620836 | 0,036331306 | |
| 226867_at | DENND4C | 2,382221458 | 2,345926937 | 0,036294521 | |
| 1554747_a_at | 37500 | 3,506195778 | 3,469974737 | 0,036221041 | |
| 202753_at | PSMD6 | 5,615256223 | 5,579053664 | 0,036202559 | |
| 209084_s_at | RAB28 | 1,81142822 | 1,775255987 | 0,036172233 | |
| 227784_s_at | COG1 | 1,81142822 | 1,775255987 | 0,036172233 | |
| 213161_at | TMOD1 /// TSTD | 2,139623739 | 2,103505212 | 0,036118527 | |
| 215505_s_at | STRN3 | 2,402750389 | 2,366632651 | 0,036117738 | |
| 209620_s_at | ABCB7 | 3,139900454 | 3,103784293 | 0,036116161 | |
| 203304_at | BAMBI | 3,988699475 | 3,952590169 | 0,036109306 | |
| 200626_s_at | MATR3 /// SNHG | 6,072400675 | 6,036339961 | 0,036060714 | |
| 201841_s_at | HSPB1 | 4,781007315 | 4,74496648 | 0,036040835 | |
| 212154_at | SDC2 | 3,418185797 | 3,382199319 | 0,035986478 | |
| 201010_s_at | TXNIP | 5,642700556 | 5,606732016 | 0,035968541 | |
| 223538_at | SERF1A /// SERF | 3,158127685 | 3,122166354 | 0,035961331 | |
| 212309_at | CLASP2 | 2,644299732 | 2,608344689 | 0,035955043 | |
| 225402_at | TP53RK | 4,08838118 | 4,052455702 | 0,035925478 | |
| 224458_at | TMEM246 | 4,162483976 | 4,126559358 | 0,035924618 | |
| 204972_at | OAS2 | 0,841135803 | 0,805216064 | 0,035919739 | |
| 212190_at | SERPINE2 | 0,841135803 | 0,805216064 | 0,035919739 | |
| 221917_s_at | GRSF1 | 0,841135803 | 0,805216064 | 0,035919739 | |
| 223089_at | VEZT | 0,841135803 | 0,805216064 | 0,035919739 | |
| 243806_at | - | 0,841135803 | 0,805216064 | 0,035919739 | |
| 227686_at | OXNAD1 | 3,01878441 | 2,982929462 | 0,035854948 | |
| 223522_at | MIR600 /// MIR60 | 0,853119696 | 0,817300337 | 0,035819359 | |
| 206284_x_at | CLTB | 2,661438684 | 2,625627364 | 0,03581132 | |
| 206364_at | KIF14 | 2,178241192 | 2,14244844 | 0,035792752 | |
| 203730_s_at | ZKSCAN5 | 1,446464021 | 1,410714371 | 0,035749649 | |
| 203834_s_at | TGOLN2 | 1,446464021 | 1,410714371 | 0,035749649 | |
| 204474_at | ZNF142 | 1,446464021 | 1,410714371 | 0,035749649 | |
| 225656_at | EFHC1 | 1,446464021 | 1,410714371 | 0,035749649 | |
| 209902_at | ATR | 1,454352496 | 1,418668082 | 0,035684414 | |
| 222874_s_at | CLN8 | 1,454352496 | 1,418668082 | 0,035684414 | |
| 52078_at | TMEM222 | 2,870437551 | 2,834761772 | 0,035675778 | |
| 223818_s_at | RSF1 | 0,87091093 | 0,835239052 | 0,035671878 | |
| 228482_at | CDRT4 /// FAM18 | 0,87091093 | 0,835239052 | 0,035671878 | |
| 235076_at | CALCOCO2 | 0,87091093 | 0,835239052 | 0,035671878 | |

| | | | | | |
|---|---|---|---|---|---|
| 1555630_a_at | NARR /// RAB34 | 3,96128107 | 3,925633118 | 0,035647951 | |
| 224914_s_at | SARNP | 4,400036207 | 4,364410072 | 0,035626135 | |
| 222714_s_at | LACTB2 | 4,208686884 | 4,173144843 | 0,035542041 | |
| 215780_s_at | SET /// SETP4 | 5,642268985 | 5,606732016 | 0,035536969 | |
| 200035_at | CTDNEP1 | 3,474545391 | 3,439026336 | 0,035519055 | |
| 226173_at | TBC1D25 | 1,894952274 | 1,859476797 | 0,035475477 | |
| 204080_at | TOE1 | 2,218170265 | 2,182705233 | 0,035465032 | |
| 204155_s_at | SIK3 | 0,900083947 | 0,864649967 | 0,03543398 | |
| 229069_at | SARNP | 0,900083947 | 0,864649967 | 0,03543398 | |
| 228346_at | ZNF844 | 1,485482016 | 1,450051551 | 0,035430465 | |
| 212287_at | SUZ12 | 4,608815095 | 4,573447849 | 0,035367246 | |
| 212875_s_at | C2CD2 | 2,493593689 | 2,458228496 | 0,035365193 | |
| 227700_x_at | ATAD3A | 3,815781597 | 3,780418488 | 0,035363109 | |
| 212794_s_at | KIAA1033 | 0,911589958 | 0,876248481 | 0,035341477 | |
| 219375_at | CEPT1 | 2,724770722 | 2,689475799 | 0,035294923 | |
| 201988_s_at | CREBL2 | 1,504602299 | 1,469325098 | 0,035277201 | |
| 227871_at | CHM | 1,504602299 | 1,469325098 | 0,035277201 | |
| 203258_at | DRAP1 | 2,919702154 | 2,884425449 | 0,035276705 | |
| 226619_at | SENP1 | 2,243430036 | 2,208167653 | 0,035262384 | |
| 220755_s_at | C6orf48 | 5,857776633 | 5,822558197 | 0,035218436 | |
| 226987_at | RBM15B | 0,928678725 | 0,893473268 | 0,035205457 | |
| 204767_s_at | FEN1 | 4,929879398 | 4,894683437 | 0,035195961 | |
| 226823_at | PHACTR4 | 3,643632572 | 3,608541373 | 0,035091198 | |
| 223082_at | SH3KBP1 | 4,757289776 | 4,72225126 | 0,035038516 | |
| 213701_at | C12orf29 | 3,858765357 | 3,823741953 | 0,035023404 | |
| 215566_x_at | LYPLA2 | 2,952099138 | 2,917077481 | 0,035021657 | |
| 214988_s_at | SON | 5,444830147 | 5,409815321 | 0,035014826 | |
| 205346_at | ST3GAL2 | 0,95671774 | 0,921731975 | 0,034985765 | |
| 214780_s_at | MYO9B | 0,95671774 | 0,921731975 | 0,034985765 | |
| 222991_s_at | UBQLN1 | 3,866915053 | 3,831954914 | 0,034960139 | |
| 209246_at | ABCF2 | 2,546213279 | 2,511262629 | 0,03495065 | |
| 1554577_a_at | PSMD10 | 3,868391882 | 3,833443169 | 0,034948713 | |
| 200081_s_at | RPS6 | 7,708630465 | 7,673725451 | 0,034905014 | |
| 201100_s_at | USP9X | 5,415581646 | 5,38071872 | 0,034862927 | |
| 208898_at | ATP6V1D | 4,83185274 | 4,796999275 | 0,034853466 | |
| 205519_at | WDR76 | 1,560488174 | 1,525647381 | 0,034840793 | |
| 222056_s_at | FAHD2A | 2,787001691 | 2,752192409 | 0,034809282 | |
| 224954_at | SHMT1 | 2,305800198 | 2,271023131 | 0,034777067 | |
| 204059_s_at | ME1 | 0,984222186 | 0,949447784 | 0,034774402 | |
| 214625_s_at | MINK1 | 0,984222186 | 0,949447784 | 0,034774402 | |
| 218021_at | DHRS4 /// DHRS | 2,994644467 | 2,959949103 | 0,034695365 | |
| 218324_s_at | SPATS2 | 0,995078803 | 0,960386719 | 0,034692083 | |
| 209898_x_at | ITSN2 | 3,004080279 | 2,969455976 | 0,034624303 | |
| 209218_at | SQLE | 6,093075084 | 6,058462275 | 0,03461281 | |
| 227259_at | CD47 | 1,011212064 | 0,976641161 | 0,034570903 | |
| 222801_s_at | STAG3L4 | 1,596577845 | 1,562009954 | 0,034567891 | |
| 223805_at | OSBPL6 | 4,019599615 | 3,985091175 | 0,034508439 | |
| 206123_at | LLGL1 | 1,021868205 | 0,987376598 | 0,034491608 | |
| 219333_s_at | CAPN10 | 1,021868205 | 0,987376598 | 0,034491608 | |
| 228364_at | ZNF784 | 1,021868205 | 0,987376598 | 0,034491608 | |
| 222010_at | SNORA29 /// TCF | 3,608715137 | 3,574237491 | 0,034477646 | |
| 220099_s_at | LUC7L2 | 5,025312571 | 4,990846468 | 0,034466103 | |
| 229863_s_at | C3orf75 | 2,611163891 | 2,576704359 | 0,034459532 | |
| 212435_at | TRIM33 | 2,837639949 | 2,803210557 | 0,034429391 | |
| 225153_at | GFM1 | 4,20284547 | 4,168435485 | 0,034409986 | |
| 214106_s_at | GMDS | 1,631786706 | 1,59747851 | 0,034308196 | |

| | | | | | |
|---|---|---|---|---|---|
| 225160_x_at | MDM2 | 1,04816921 | 1,013870809 | 0,034298401 | |
| 229018_at | C12orf26 | 1,64218396 | 1,60795124 | 0,03423272 | |
| 226703_at | NEURL4 | 1,063722698 | 1,029536893 | 0,034185804 | |
| 206167_s_at | ARHGAP6 | 4,159471171 | 4,125344852 | 0,034126319 | |
| 223580_at | SPSB2 | 1,073999308 | 1,039887232 | 0,034112077 | |
| 225040_s_at | LOC729020 /// RI | 3,882346871 | 3,848241888 | 0,034104983 | |
| 202562_s_at | C14orf1 | 3,776205896 | 3,742106442 | 0,034099454 | |
| 209536_s_at | EHD4 | 3,248232277 | 3,21415095 | 0,034081327 | |
| 218862_at | ASB13 | 1,666156729 | 1,632095957 | 0,034060772 | |
| 223507_at | CLPX | 2,410880861 | 2,376875125 | 0,034005736 | |
| 228347_at | SIX1 | 2,410880861 | 2,376875125 | 0,034005736 | |
| 207627_s_at | TFCP2 | 1,67631007 | 1,642321262 | 0,033988808 | |
| 90610_at | LRCH4 | 2,683419202 | 2,649480478 | 0,033938724 | |
| 1553569_at | COX2 | 8,22705635 | 8,193185275 | 0,033871075 | |
| 208896_at | DDX18 | 4,35912833 | 4,32527877 | 0,03384956 | |
| 203869_at | USP46 | 1,699726954 | 1,665902186 | 0,033824769 | |
| 225902_at | PPIG | 1,699726954 | 1,665902186 | 0,033824769 | |
| 224626_at | SLC35A4 | 2,922548342 | 2,888726553 | 0,033821789 | |
| 203189_s_at | MIR4691 /// NDU | 3,823415835 | 3,789647959 | 0,033767877 | |
| 217928_s_at | PPP6R3 | 2,132266459 | 2,098562556 | 0,033703903 | |
| 213427_at | RPP40 | 4,657462947 | 4,623770256 | 0,03369269 | |
| 204507_s_at | PPP3R1 | 4,22203305 | 4,188344947 | 0,033688103 | |
| 206063_x_at | PPIL2 | 1,139070151 | 1,105412836 | 0,033657315 | |
| 225059_at | AGTRAP | 1,726031811 | 1,692388143 | 0,033643668 | |
| 218055_s_at | WDR41 | 2,954882186 | 2,921282473 | 0,033599713 | |
| 223273_at | C14orf142 | 3,734704399 | 3,701119714 | 0,033584685 | |
| 224978_s_at | USP36 | 1,735773781 | 1,702196344 | 0,033577437 | |
| 221823_at | C5orf30 | 2,156646206 | 2,123108223 | 0,033537983 | |
| 223079_s_at | GLS | 2,156646206 | 2,123108223 | 0,033537983 | |
| 203597_s_at | WBP4 | 1,163336153 | 1,129843209 | 0,033492944 | |
| 225851_at | CHURC1-FNTB / | 2,485917452 | 2,452428893 | 0,033488558 | |
| 222458_s_at | AKIRIN1 | 2,979690938 | 2,946258252 | 0,033432686 | |
| 233746_x_at | C15orf63 /// MIR1 | 4,176858496 | 4,143455947 | 0,033402549 | |
| 231770_x_at | WDPCP | 1,177702228 | 1,144305291 | 0,033396937 | |
| 219600_s_at | TMEM50B | 4,178645282 | 4,145254615 | 0,033390668 | |
| 226343_at | DPP8 | 2,501229299 | 2,467842989 | 0,03338631 | |
| 218069_at | DCTPP1 | 4,993182736 | 4,959839678 | 0,033343058 | |
| 207785_s_at | RBPJ | 3,901227032 | 3,86798237 | 0,033244662 | |
| 219296_at | ZDHHC13 | 1,201332287 | 1,16809119 | 0,033241096 | |
| 200046_at | DAD1 | 6,26782071 | 6,234582224 | 0,033238485 | |
| 201596_x_at | KRT18 | 1,210676994 | 1,177496821 | 0,033180173 | |
| 212000_at | SUGP2 | 1,210676994 | 1,177496821 | 0,033180173 | |
| 230048_at | IFRD1 | 1,210676994 | 1,177496821 | 0,033180173 | |
| 221554_at | STRADA | 1,799091453 | 1,765933685 | 0,033157769 | |
| 1565162_s_at | MGST1 | 2,804079361 | 2,770953871 | 0,03312549 | |
| 228420_at | PDCD2 | 1,224581536 | 1,191491281 | 0,033090255 | |
| 218233_s_at | PRICKLE4 /// TO | 6,912507794 | 6,87942671 | 0,033081084 | |
| 215113_s_at | SENP3 /// SENP3 | 1,814495998 | 1,781437557 | 0,033058441 | |
| 238729_x_at | SAV1 | 1,233777334 | 1,200746069 | 0,033031264 | |
| 212710_at | CAMSAP1 | 1,823660386 | 1,790660532 | 0,032999854 | |
| 217882_at | EMC3 | 4,327801949 | 4,294812548 | 0,032989401 | |
| 202107_s_at | MCM2 | 4,329411337 | 4,296432174 | 0,032979163 | |
| 200008_s_at | GDI2 | 5,288690404 | 5,255726156 | 0,032964248 | |
| 220633_s_at | HP1BP3 | 1,829737795 | 1,796776588 | 0,032961208 | |
| 235443_at | LOC100131067 | 1,247462054 | 1,214517879 | 0,032944175 | |
| 236077_at | GANC | 0,255492549 | 0,222599095 | 0,032893455 | |

| | | | | | |
|---|---|---|---|---|---|
| 239493_at | RPL7 | 0,255492549 | 0,222599095 | 0,032893455 | |
| 239106_at | - | 3,262904562 | 3,23005767 | 0,032846892 | |
| 222845_x_at | TMBIM4 | 4,725862305 | 4,69303748 | 0,032824826 | |
| 203308_x_at | HPS1 | 1,269985355 | 1,23718272 | 0,032802635 | |
| 200070_at | CNPPD1 | 1,859746653 | 1,826973888 | 0,032772765 | |
| 202810_at | DRG1 | 4,86589008 | 4,83315541 | 0,032734671 | |
| 225247_at | C19orf6 | 1,874520207 | 1,841838775 | 0,032681432 | |
| 238965_at | C21orf2 | 1,292162421 | 1,259496994 | 0,032665426 | |
| 227941_at | LOC339803 | 2,292660284 | 2,259997914 | 0,03266237 | |
| 210706_s_at | RNF24 | 3,75400739 | 3,721356767 | 0,032650623 | |
| 212859_x_at | LOC100505584 / | 1,883312263 | 1,85068474 | 0,032627523 | |
| 201364_s_at | OAZ2 | 2,632180155 | 2,599625088 | 0,032555068 | |
| 214314_s_at | EIF5B | 3,117719377 | 3,08516499 | 0,032554386 | |
| 221511_x_at | CCPG1 /// DYX1( | 1,314003737 | 1,281471383 | 0,032532354 | |
| 231270_at | CA13 /// LOC100 | 1,314003737 | 1,281471383 | 0,032532354 | |
| 200027_at | NARS | 6,145329472 | 6,112864255 | 0,032465217 | |
| 218842_at | RPAP3 | 3,923419763 | 3,891034012 | 0,032385751 | |
| 200977_s_at | TAX1BP1 | 4,868844325 | 4,836499048 | 0,032345277 | |
| 222028_at | ZNF45 | 3,154500615 | 3,122166354 | 0,032334261 | |
| 1570253_a_at | RHEBL1 | 1,932146506 | 1,899812471 | 0,032334035 | |
| 243943_x_at | C6orf52 | 1,954567604 | 1,922365006 | 0,032202598 | |
| 209501_at | CDR2 | 2,956271699 | 2,924079008 | 0,032192692 | |
| 228875_at | FAM162B | 2,956271699 | 2,924079008 | 0,032192692 | |
| 215034_s_at | TM4SF1 | 2,967339888 | 2,935211251 | 0,032128637 | |
| 225429_at | PPP6C | 2,967339888 | 2,935211251 | 0,032128637 | |
| 225411_at | TMEM87B | 2,705069849 | 2,672945617 | 0,032124232 | |
| 1564746_at | SLC9B2 | 0,384950573 | 0,352836757 | 0,032113816 | |
| 225089_at | USP40 | 0,384950573 | 0,352836757 | 0,032113816 | |
| 226764_at | ZNF827 | 0,384950573 | 0,352836757 | 0,032113816 | |
| 201733_at | CLCN3 | 1,390002893 | 1,3579181 | 0,032084793 | |
| 210180_s_at | TRA2B | 2,981056773 | 2,949006836 | 0,032049937 | |
| 214414_x_at | HBA1 /// HBA2 | 6,650471128 | 6,618565837 | 0,03190529 | |
| 223630_at | C7orf13 | 2,745814053 | 2,713921281 | 0,031892772 | |
| 207812_s_at | GORASP2 | 4,332089668 | 4,300204247 | 0,031885421 | |
| 1553354_a_at | FLJ31958 | 0,425644434 | 0,393761504 | 0,031882929 | |
| 237994_at | - | 0,425644434 | 0,393761504 | 0,031882929 | |
| 242475_at | - | 0,425644434 | 0,393761504 | 0,031882929 | |
| 224819_at | TCEAL8 | 3,01878441 | 2,986947093 | 0,031837317 | |
| 203729_at | EMP3 | 5,652376942 | 5,620590087 | 0,031786855 | |
| 208659_at | CLIC1 | 6,751066697 | 6,719303915 | 0,031762782 | |
| 207388_s_at | PTGES | 0,46522187 | 0,433557298 | 0,031664572 | |
| 214368_at | RASGRP2 | 0,46522187 | 0,433557298 | 0,031664572 | |
| 224065_at | HIPK2 | 0,46522187 | 0,433557298 | 0,031664572 | |
| 211797_s_at | NFYC | 2,051361651 | 2,019703483 | 0,031658169 | |
| 218597_s_at | CISD1 | 3,054253462 | 3,022611001 | 0,031642462 | |
| 209592_s_at | DCAF7 | 2,470441349 | 2,438805127 | 0,031636222 | |
| 217977_at | MSRB1 | 3,795741421 | 3,76412346 | 0,031617962 | |
| 217608_at | SREK1IP1 | 1,473887017 | 1,442269454 | 0,031617563 | |
| 212447_at | KBTBD2 | 3,062018438 | 3,030417996 | 0,031600442 | |
| 200062_s_at | RPL30 | 7,740481836 | 7,708964585 | 0,031517251 | |
| 35776_at | ITSN1 | 2,083507233 | 2,052021897 | 0,031485336 | |
| 213507_s_at | KPNB1 | 6,467203544 | 6,435733475 | 0,031470068 | |
| 226457_at | - | 1,512180019 | 1,480766833 | 0,031413186 | |
| 223219_s_at | CNOT10 | 2,097427503 | 2,066015817 | 0,031411685 | |
| 204266_s_at | CHKA | 2,837639949 | 2,806245339 | 0,031394609 | |
| 211270_x_at | PTBP1 | 5,97996145 | 5,948592109 | 0,031369341 | |

| | | | | | |
|---|---|---|---|---|---|
| 212073_at | CSNK2A1 /// CSN | 3,329826127 | 3,298466943 | 0,031359184 | |
| 1553715_s_at | FAM195A | 3,857278636 | 3,825986493 | 0,031292143 | |
| 235256_s_at | GALM | 3,343695493 | 3,312408479 | 0,031287015 | |
| 203925_at | GCLM | 3,348994562 | 3,317734938 | 0,031259625 | |
| 208848_at | ADH5 | 3,359534664 | 3,32832922 | 0,031205444 | |
| 208714_at | NDUFV1 | 4,361752105 | 4,330558009 | 0,031194096 | |
| 204053_x_at | PTEN | 3,363729238 | 3,332545245 | 0,031183993 | |
| 224769_at | TAOK1 | 3,895444023 | 3,864347045 | 0,031096978 | |
| 224455_s_at | ADPGK | 1,58584542 | 1,55481039 | 0,03103503 | |
| 208732_at | RAB2A | 2,173470163 | 2,14244844 | 0,031021724 | |
| 231988_x_at | ZNF490 | 2,173470163 | 2,14244844 | 0,031021724 | |
| 206583_at | ZNF673 | 2,589836943 | 2,558821855 | 0,031015089 | |
| 226753_at | FAM76B | 2,589836943 | 2,558821855 | 0,031015089 | |
| 204094_s_at | TSC22D2 | 2,919702154 | 2,888726553 | 0,0309756 | |
| 211943_x_at | TPT1 | 7,750427418 | 7,719466222 | 0,030961196 | |
| 219321_at | MPP5 | 1,603688695 | 1,572742379 | 0,030946316 | |
| 223470_at | PIGM | 2,933877275 | 2,902971658 | 0,030905617 | |
| 201194_at | SEPW1 | 4,204015647 | 4,173144843 | 0,030870804 | |
| 203625_x_at | SKP2 | 3,428234044 | 3,39737216 | 0,030861884 | |
| 200014_s_at | HNRNPC /// LOC | 5,98252085 | 5,951678905 | 0,030841945 | |
| 49878_at | PEX16 | 2,636520272 | 2,605734338 | 0,030785935 | |
| 230787_at | LOC100505481 | 1,638726528 | 1,60795124 | 0,030775288 | |
| 239071_at | RBBP4 | 1,638726528 | 1,60795124 | 0,030775288 | |
| 201503_at | G3BP1 | 5,651304988 | 5,620590087 | 0,030714901 | |
| 214696_at | MIR22 /// MIR22H | 2,243430036 | 2,212749286 | 0,03068075 | |
| 64432_at | MAPKAPK5-AS1 | 1,247462054 | 1,216800453 | 0,030661601 | |
| 211040_x_at | GTSE1 | 3,471633352 | 3,440980183 | 0,030653169 | |
| 204300_at | PET112 | 2,261526912 | 2,230931691 | 0,03059522 | |
| 204887_s_at | PLK4 | 3,000043914 | 2,969455976 | 0,030587938 | |
| 201587_s_at | IRAK1 | 4,00557508 | 3,975013041 | 0,030562039 | |
| 40189_at | SET | 5,496123605 | 5,46558546 | 0,030538146 | |
| 203530_s_at | STX4 | 3,010782559 | 2,980244813 | 0,030537746 | |
| 215699_x_at | SFI1 | 0,702292136 | 0,67181667 | 0,030475466 | |
| 227979_at | RBM4 | 0,702292136 | 0,67181667 | 0,030475466 | |
| 230271_at | ONECUT2 | 0,702292136 | 0,67181667 | 0,030475466 | |
| 225870_s_at | TRAPPC5 | 3,704430214 | 3,673964604 | 0,03046561 | |
| 209953_s_at | CDC37 | 6,256938512 | 6,226604901 | 0,030333611 | |
| 224890_s_at | C7orf59 | 3,056846434 | 3,026519779 | 0,030326655 | |
| 221471_at | SERINC3 | 4,986397998 | 4,956087342 | 0,030310656 | |
| 202176_at | ERCC3 | 2,344516784 | 2,314300277 | 0,030216507 | |
| 205741_s_at | DTNA | 0,767063567 | 0,73688026 | 0,030183306 | |
| 229841_at | - | 0,767063567 | 0,73688026 | 0,030183306 | |
| 210186_s_at | FKBP1A | 4,366985384 | 4,336867701 | 0,030117683 | |
| 201046_s_at | RAD23A | 3,109000457 | 3,078904771 | 0,030095686 | |
| 216804_s_at | PDLIM5 | 3,111496958 | 3,081412119 | 0,030084839 | |
| 232201_at | NKD2 | 0,798390451 | 0,768343791 | 0,03004666 | |
| 232865_at | AFF4 | 0,798390451 | 0,768343791 | 0,03004666 | |
| 225318_at | - | 3,606946883 | 3,576905405 | 0,030041479 | |
| 229690_at | FAM109A | 1,81142822 | 1,781437557 | 0,029990663 | |
| 201528_at | RPA1 | 5,47616359 | 5,446261552 | 0,029902038 | |
| 203038_at | PTPRK | 1,835789711 | 1,805902329 | 0,029887382 | |
| 203267_s_at | DRG2 | 1,835789711 | 1,805902329 | 0,029887382 | |
| 216873_s_at | ATP8B2 | 1,841816345 | 1,811954245 | 0,029862101 | |
| 221475_s_at | RPL15 | 7,302732218 | 7,272944883 | 0,029787335 | |
| 219351_at | TRAPPC2 | 3,18563636 | 3,155865206 | 0,029771154 | |
| 203142_s_at | AP3B1 | 3,188003814 | 3,158242412 | 0,029761401 | |

| | | | | | |
|---|---|---|---|---|---|
| 222699_s_at | PLEKHF2 | 3,886725813 | 3,857048799 | 0,029677014 | |
| 241786_at | - | 1,894952274 | 1,865308541 | 0,029643732 | |
| 201722_s_at | GALNT1 | 5,287586957 | 5,257944504 | 0,029642452 | |
| 201599_at | OAT | 5,55279188 | 5,523204285 | 0,029587595 | |
| 1558942_at | ZNF765 | 3,236843482 | 3,207279726 | 0,029563756 | |
| 217833_at | SYNCRIP | 4,501552344 | 4,471995246 | 0,029557098 | |
| 218165_at | MEAF6 | 4,08838118 | 4,058831494 | 0,029549686 | |
| 210466_s_at | SERBP1 | 6,382530778 | 6,352999488 | 0,02953129 | |
| 210844_x_at | CTNNA1 | 4,246090231 | 4,216563145 | 0,029527086 | |
| 220637_at | FAM124B | 0,934330293 | 0,904843258 | 0,029487034 | |
| 202894_at | EPHB4 | 2,522021211 | 2,492544876 | 0,029476335 | |
| 203366_at | POLG | 1,937784532 | 1,908311039 | 0,029473492 | |
| 226527_at | RPRD2 | 1,946200473 | 1,916759838 | 0,029440635 | |
| 211370_s_at | MAP2K5 | 0,962260738 | 0,932882274 | 0,029378464 | |
| 214077_x_at | MEIS3P1 | 0,962260738 | 0,932882274 | 0,029378464 | |
| 224673_at | LENG8 | 0,962260738 | 0,932882274 | 0,029378464 | |
| 235124_at | LOC645212 | 0,962260738 | 0,932882274 | 0,029378464 | |
| 231901_at | C19orf52 | 4,294682191 | 4,265343974 | 0,029338217 | |
| 202353_s_at | PSMD12 | 3,310399576 | 3,281121096 | 0,02927848 | |
| 222523_at | SENP2 | 2,582657321 | 2,553413592 | 0,029243728 | |
| 217393_x_at | UBE2NL | 4,461994552 | 4,432769076 | 0,029225476 | |
| 202301_s_at | RSRC2 | 3,002736078 | 2,973511251 | 0,029224828 | |
| 36994_at | ATP6V0C | 5,050901237 | 5,021690504 | 0,029210733 | |
| 214670_at | ZKSCAN1 | 2,014487247 | 1,98530622 | 0,029181027 | |
| 242648_at | KLHL8 | 3,343695493 | 3,314541423 | 0,029154071 | |
| 233757_x_at | - | 3,024094427 | 2,994948944 | 0,029145483 | |
| 226505_x_at | USP32 | 3,348994562 | 3,319860027 | 0,029134535 | |
| 201713_s_at | RANBP2 | 4,217404896 | 4,188344947 | 0,029059949 | |
| 208928_at | POR | 2,061726373 | 2,032717854 | 0,029008519 | |
| 320_at | PEX6 | 0,062557792 | 0,033557811 | 0,028999981 | |
| 227013_at | LATS2 | 2,658027143 | 2,629059192 | 0,028967951 | |
| 228588_s_at | UBE2B | 3,081249977 | 3,052311115 | 0,028938862 | |
| 1558700_s_at | ZNF260 | 4,253455927 | 4,224525141 | 0,028930786 | |
| 209184_s_at | IRS2 | 1,094335495 | 1,065442793 | 0,028892702 | |
| 227663_at | - | 2,094906514 | 2,066015817 | 0,028890697 | |
| 200881_s_at | DNAJA1 | 6,384983404 | 6,356109089 | 0,028874315 | |
| 211074_at | - | 2,701760055 | 2,672945617 | 0,028814438 | |
| 214698_at | PTBP3 | 1,119359127 | 1,090553537 | 0,02880559 | |
| 223252_at | HDGFRP2 | 2,11992033 | 2,091116676 | 0,028803654 | |
| 213670_x_at | NSUN5P1 | 2,139623739 | 2,110887591 | 0,028736148 | |
| 205084_at | BCAP29 | 1,143956113 | 1,115234684 | 0,028721429 | |
| 227492_at | LOC647859 /// O | 2,171078719 | 2,14244844 | 0,02863028 | |
| 213246_at | C14orf109 | 3,979845523 | 3,951219941 | 0,028625582 | |
| 227932_at | ARIH2 | 2,764969948 | 2,7363693 | 0,028600648 | |
| 204448_s_at | PDCL | 2,194816625 | 2,166264721 | 0,028551904 | |
| 223222_at | SLC25A19 | 2,804079361 | 2,775606361 | 0,028473 | |
| 202633_at | TOPBP1 | 4,036776538 | 4,008336597 | 0,028439941 | |
| 203392_s_at | CTBP1 | 4,404623843 | 4,376200741 | 0,028423102 | |
| 204766_s_at | NUDT1 | 2,827046023 | 2,798646381 | 0,028399643 | |
| 218104_at | TEX10 | 4,247793348 | 4,219411762 | 0,028381586 | |
| 224891_at | FOXO3 | 4,575748639 | 4,547386035 | 0,028362604 | |
| 1555874_x_at | MGC21881 | 2,254767157 | 2,226407528 | 0,028359629 | |
| 222995_s_at | RHBDD2 | 2,259277178 | 2,230931691 | 0,028345486 | |
| 208908_s_at | CAST | 2,849652795 | 2,821324225 | 0,02832857 | |
| 224623_at | LOC728554 /// Tl | 3,852063011 | 3,823741953 | 0,028321058 | |
| 217887_s_at | EPS15 | 3,590934306 | 3,562619178 | 0,028315128 | |

| | | | | | |
|---|---|---|---|---|---|
| 214283_at | TMEM97 | 2,281618189 | 2,253342112 | 0,028276077 | |
| 1555847_a_at | LOC284454 | 2,288253583 | 2,259997914 | 0,028255669 | |
| 209939_x_at | CFLAR | 5,199102221 | 5,170858681 | 0,02824354 | |
| 225629_s_at | ZBTB4 | 2,292660284 | 2,264418116 | 0,028242168 | |
| 217828_at | SLTM | 4,117238882 | 4,08904909 | 0,028189792 | |
| 1555193_a_at | ZNF277 | 1,331241794 | 1,303116084 | 0,028125709 | |
| 212031_at | RBM25 | 4,814336575 | 4,786280762 | 0,028055812 | |
| 202853_s_at | RYK | 3,358484112 | 3,330438773 | 0,02804534 | |
| 201359_at | COPB1 | 4,725454911 | 4,697539161 | 0,02791575 | |
| 203361_s_at | GJA9-MYCBP /// | 2,427005622 | 2,39915583 | 0,027849792 | |
| 209862_s_at | CEP57 | 2,436992838 | 2,409170778 | 0,02782206 | |
| 211761_s_at | CACYBP | 5,347321839 | 5,319509282 | 0,027812557 | |
| 208671_at | SERINC1 | 4,768801946 | 4,741007135 | 0,027794812 | |
| 203046_s_at | TIMELESS | 3,034656185 | 3,006869115 | 0,02778707 | |
| 200031_s_at | RPS11 | 8,145050721 | 8,117326049 | 0,027724672 | |
| 213892_s_at | APRT | 3,067171967 | 3,039473081 | 0,027698886 | |
| 227125_at | IFNAR2 | 3,067171967 | 3,039473081 | 0,027698886 | |
| 218883_s_at | MLF1IP | 5,011176618 | 4,983492199 | 0,027684419 | |
| 208827_at | PSMB6 | 5,744524656 | 5,716864158 | 0,027660498 | |
| 219240_s_at | C10orf88 | 1,496984567 | 1,469325098 | 0,027659469 | |
| 214305_s_at | SF3B1 | 2,510717331 | 2,483094137 | 0,027623194 | |
| 206158_s_at | CNBP | 6,106278071 | 6,078682636 | 0,027595435 | |
| 204367_at | SP2 | 1,534677255 | 1,507116528 | 0,027560727 | |
| 224472_x_at | SDF4 | 3,863216352 | 3,835672678 | 0,027543674 | |
| 217827_s_at | SPG21 | 4,360703168 | 4,333190402 | 0,027512766 | |
| 1569110_x_at | LOC728613 | 3,901227032 | 3,873779903 | 0,027447129 | |
| 201035_s_at | HADH | 2,586251598 | 2,558821855 | 0,027429744 | |
| 209522_s_at | CRAT | 1,607231019 | 1,579853228 | 0,027377791 | |
| 223568_s_at | PPAPDC1B | 1,607231019 | 1,579853228 | 0,027377791 | |
| 223839_s_at | SCD | 3,19743497 | 3,170070041 | 0,027364929 | |
| 225413_at | USMG5 | 7,27502272 | 7,247703285 | 0,027319435 | |
| 201257_x_at | RPS3A /// SNOR | 8,148283735 | 8,120986768 | 0,027296968 | |
| 213214_x_at | ACTG1 | 7,840606193 | 7,813333862 | 0,027272331 | |
| 201606_s_at | PWP1 | 4,250061047 | 4,222822694 | 0,027238353 | |
| 202154_x_at | TUBB3 | 4,891899985 | 4,864793325 | 0,027106659 | |
| 204415_at | IFI6 | 6,591558544 | 6,564478117 | 0,027080428 | |
| 218354_at | TRAPPC2L | 4,08838118 | 4,061373942 | 0,027007238 | |
| 203746_s_at | HCCS | 5,252954628 | 5,226006411 | 0,026948216 | |
| 213347_x_at | RPS4X | 7,726553789 | 7,699636024 | 0,026917766 | |
| 231049_at | LMO2 | 3,397876879 | 3,370970266 | 0,026906613 | |
| 222047_s_at | SRRT | 5,277061897 | 5,250165317 | 0,02689658 | |
| 210110_x_at | HNRNPH3 | 4,426844657 | 4,399999158 | 0,026845499 | |
| 201767_s_at | ELAC2 | 3,84757742 | 3,820743792 | 0,026833628 | |
| 222026_at | RBM3 | 1,868628927 | 1,841838775 | 0,026790152 | |
| 223334_at | TMEM126A | 4,676134621 | 4,649344778 | 0,026789843 | |
| 212506_at | PICALM | 5,578283569 | 5,551512249 | 0,026771321 | |
| 218631_at | AVPI1 | 2,882181479 | 2,855418981 | 0,026762498 | |
| 200716_x_at | RPL13A /// RPL1 | 7,972907267 | 7,946172884 | 0,026734384 | |
| 202732_at | PKIG | 4,90958085 | 4,882873472 | 0,026707378 | |
| 231059_x_at | SCAND1 | 4,235256884 | 4,208556964 | 0,02669992 | |
| 212334_at | GNS | 3,498586477 | 3,471887144 | 0,026699333 | |
| 202767_at | ACP2 | 1,926486359 | 1,899812471 | 0,026673889 | |
| 208595_s_at | MBD1 | 1,926486359 | 1,899812471 | 0,026673889 | |
| 227729_at | - | 1,926486359 | 1,899812471 | 0,026673889 | |
| 218516_s_at | IMPAD1 | 2,926807124 | 2,900133867 | 0,026673257 | |
| 210006_at | ABHD14A | 2,943717636 | 2,917077481 | 0,026640156 | |

| | | | | | |
|---|---|---|---|---|---|
| 201074_at | SMARCC1 | 4,767615283 | 4,741007135 | 0,026608149 | |
| 221523_s_at | RRAGD | 3,575646363 | 3,549096127 | 0,026550236 | |
| 201145_at | HAX1 | 4,336364681 | 4,309858816 | 0,026505865 | |
| 1557624_at | - | 1,027166904 | 1,000684521 | 0,026482383 | |
| 209751_s_at | TRAPPC2 /// TR/ | 3,614006933 | 3,587527995 | 0,026478938 | |
| 232032_x_at | SDF4 | 4,866629209 | 4,840205135 | 0,026424074 | |
| 201388_at | PSMD3 | 3,741167469 | 3,714911743 | 0,026255726 | |
| 201476_s_at | RRM1 | 3,811181587 | 3,785040604 | 0,026140983 | |
| 200950_at | ARPC1A | 3,831767101 | 3,805658807 | 0,026108294 | |
| 223160_s_at | SMEK1 | 4,431347964 | 4,405262601 | 0,026085362 | |
| 226278_at | SVIP | 3,852063011 | 3,825986493 | 0,026076519 | |
| 217947_at | CMTM6 | 4,660449652 | 4,634481456 | 0,025968196 | |
| 209860_s_at | ANXA7 | 4,352811731 | 4,326864571 | 0,02594716 | |
| 227527_at | MLL2 | 3,366867184 | 3,340940512 | 0,025926672 | |
| 224788_at | ARF6 | 4,741661615 | 4,71581024 | 0,025851376 | |
| 220597_s_at | ARL6IP4 | 2,429008604 | 2,403170156 | 0,025838448 | |
| 225002_s_at | SUMF2 | 2,438982012 | 2,413157372 | 0,02582464 | |
| 218203_at | ALG5 | 4,093441556 | 4,067710529 | 0,025731028 | |
| 207071_s_at | ACO1 | 3,5222335 | 3,496520471 | 0,02571303 | |
| 219000_s_at | DSCC1 | 4,598173655 | 4,572556959 | 0,025616696 | |
| 236305_at | RFESD | 3,625406332 | 3,599822964 | 0,025583368 | |
| 221895_at | MOSPD2 | 2,637386731 | 2,611817827 | 0,025568905 | |
| 211921_x_at | LOC100506248 / | 7,325558139 | 7,300142615 | 0,025415524 | |
| 202003_s_at | ACAA2 | 3,914869478 | 3,889604025 | 0,025265453 | |
| 212802_s_at | GAPVD1 | 3,964046478 | 3,938828971 | 0,025217508 | |
| 203160_s_at | RNF8 | 2,967339888 | 2,942125533 | 0,025214355 | |
| 201817_at | UBE3C | 4,622007457 | 4,59685817 | 0,025149287 | |
| 206461_x_at | MT1H | 4,659596938 | 4,634481456 | 0,025115483 | |
| 212397_at | RDX | 4,722599924 | 4,697539161 | 0,025060764 | |
| 46270_at | UBAP1 | 2,166283906 | 2,141247242 | 0,025036664 | |
| 200807_s_at | HSPD1 | 7,687033587 | 7,662046928 | 0,02498666 | |
| 223155_at | HDHD2 | 3,27186005 | 3,246908183 | 0,024951867 | |
| 218366_x_at | METTL17 | 4,903471189 | 4,878554857 | 0,024916333 | |
| 204709_s_at | KIF23 | 2,382221458 | 2,357351836 | 0,024869621 | |
| 225515_s_at | LOC220906 /// RI | 4,583412318 | 4,558677353 | 0,024734966 | |
| 223305_at | TMEM216 | 3,709382903 | 3,684723168 | 0,024659734 | |
| 229450_at | IFIT3 | 5,331079521 | 5,306440515 | 0,024639006 | |
| 201735_s_at | CLCN3 | 3,915583941 | 3,891034012 | 0,024549929 | |
| 212066_s_at | USP34 | 4,075012622 | 4,050537457 | 0,024475165 | |
| 201550_x_at | ACTG1 | 7,914708405 | 7,8903368 | 0,024371605 | |
| 218226_s_at | NDUFB4 | 5,875878037 | 5,851583612 | 0,024294425 | |
| 211747_s_at | LSM5 | 5,659218655 | 5,634956531 | 0,024262124 | |
| 1552452_at | WDR88 | -2,43257096 | -2,456406426 | 0,023835466 | |
| 1552506_at | CRB2 | -2,063149277 | -2,086984744 | 0,023835466 | |
| 1552734_at | RICTOR | -2,311268652 | -2,335104118 | 0,023835466 | |
| 1552872_at | ASMTL-AS1 | -1,399512875 | -1,423348341 | 0,023835466 | |
| 1552906_at | FMR1NB | -3,07502285 | -3,098858316 | 0,023835466 | |
| 1552922_at | RIMS1 | -3,013665402 | -3,037500868 | 0,023835466 | |
| 1552972_at | LOC100507431 | -3,448416261 | -3,472251727 | 0,023835466 | |
| 1553177_at | SH2D1B | -2,063149277 | -2,086984744 | 0,023835466 | |
| 1553326_at | RXFP2 | -2,311268652 | -2,335104118 | 0,023835466 | |
| 1553329_at | C7orf45 | -2,597496523 | -2,621331989 | 0,023835466 | |
| 1553449_at | LINC00304 | -2,01371626 | -2,037551727 | 0,023835466 | |
| 1553541_at | LMX1A | -1,888254346 | -1,912089812 | 0,023835466 | |
| 1553547_at | - | -2,43257096 | -2,456406426 | 0,023835466 | |
| 1553550_at | VN1R5 | -2,063149277 | -2,086984744 | 0,023835466 | |

| | | | | | |
|---|---|---|---|---|---|
| 1553682_at | FBXL14 | -2,063149277 | -2,086984744 | 0,023835466 | |
| 1553785_at | RASGEF1B | -2,384869671 | -2,408705138 | 0,023835466 | |
| 1553873_at | KLHL34 | -2,823166348 | -2,847001814 | 0,023835466 | |
| 1553878_at | GOT1L1 | -2,712026959 | -2,735862426 | 0,023835466 | |
| 1554143_a_at | SUGT1P3 | -2,712026959 | -2,735862426 | 0,023835466 | |
| 1554334_a_at | DNAJA4 | -2,01371626 | -2,037551727 | 0,023835466 | |
| 1554506_x_at | NAALAD2 | -3,114791413 | -3,138626879 | 0,023835466 | |
| 1554540_at | DNAH14 | -3,07502285 | -3,098858316 | 0,023835466 | |
| 1554737_at | FBN2 | -2,01371626 | -2,037551727 | 0,023835466 | |
| 1554745_at | RALGPS1 | -2,262747984 | -2,286583451 | 0,023835466 | |
| 1554785_at | CCDC82 | -2,712026959 | -2,735862426 | 0,023835466 | |
| 1554874_at | MITF | -2,930741289 | -2,954576755 | 0,023835466 | |
| 1555034_at | CLRN1 | -2,930741289 | -2,954576755 | 0,023835466 | |
| 1555071_at | TLL1 | -2,756478458 | -2,780313924 | 0,023835466 | |
| 1555111_at | C1orf114 | -3,41365796 | -3,437493426 | 0,023835466 | |
| 1555118_at | ENTPD3 | -2,597496523 | -2,621331989 | 0,023835466 | |
| 1555313_a_at | MCF2 | -2,823166348 | -2,847001814 | 0,023835466 | |
| 1555538_s_at | FAM9B | -3,114791413 | -3,138626879 | 0,023835466 | |
| 1555543_a_at | CLCC1 | 1,46610492 | 1,442269454 | 0,023835466 | |
| 1555585_a_at | FAM71B | -1,888254346 | -1,912089812 | 0,023835466 | |
| 1555591_at | PIF1 | -2,712026959 | -2,735862426 | 0,023835466 | |
| 1555801_s_at | ZNF385B | -2,823166348 | -2,847001814 | 0,023835466 | |
| 1556111_s_at | - | -1,888254346 | -1,912089812 | 0,023835466 | |
| 1556187_at | ZNF555 | -3,114791413 | -3,138626879 | 0,023835466 | |
| 1556240_at | - | -2,01371626 | -2,037551727 | 0,023835466 | |
| 1556404_a_at | ZNRF2P1 | -2,756478458 | -2,780313924 | 0,023835466 | |
| 1556488_s_at | C3orf15 /// LOC1 | -3,15396682 | -3,177802286 | 0,023835466 | |
| 1556500_a_at | - | -2,712026959 | -2,735862426 | 0,023835466 | |
| 1556578_a_at | FLJ31945 | -1,399512875 | -1,423348341 | 0,023835466 | |
| 1556595_at | - | -2,43257096 | -2,456406426 | 0,023835466 | |
| 1556671_s_at | LOC100505624 | -1,089016378 | -1,112851844 | 0,023835466 | |
| 1556696_s_at | FLJ42709 | -3,013665402 | -3,037500868 | 0,023835466 | |
| 1556697_at | GPRIN3 | -3,15396682 | -3,177802286 | 0,023835466 | |
| 1556713_at | - | -2,930741289 | -2,954576755 | 0,023835466 | |
| 1556817_a_at | - | -3,15396682 | -3,177802286 | 0,023835466 | |
| 1556895_a_at | NT5DC2 | -1,937711681 | -1,961547147 | 0,023835466 | |
| 1556958_at | - | -1,937711681 | -1,961547147 | 0,023835466 | |
| 1556964_s_at | - | -3,013665402 | -3,037500868 | 0,023835466 | |
| 1556980_at | - | -2,384869671 | -2,408705138 | 0,023835466 | |
| 1557026_at | - | -1,089016378 | -1,112851844 | 0,023835466 | |
| 1557488_at | CBX3P2 | -3,07502285 | -3,098858316 | 0,023835466 | |
| 1557531_a_at | C10orf55 | -2,597496523 | -2,621331989 | 0,023835466 | |
| 1557570_a_at | LOC285084 | -1,888254346 | -1,912089812 | 0,023835466 | |
| 1557776_at | - | -2,384869671 | -2,408705138 | 0,023835466 | |
| 1557837_a_at | ELMOD2 | -2,712026959 | -2,735862426 | 0,023835466 | |
| 1557843_at | - | -3,287237186 | -3,311072652 | 0,023835466 | |
| 1558082_at | CCDC171 | -2,597496523 | -2,621331989 | 0,023835466 | |
| 1558708_at | NRXN1 | -3,114791413 | -3,138626879 | 0,023835466 | |
| 1558937_s_at | - | -1,518072576 | -1,541908042 | 0,023835466 | |
| 1559036_at | C4A /// C4B /// LC | -2,384869671 | -2,408705138 | 0,023835466 | |
| 1559063_at | - | -2,756478458 | -2,780313924 | 0,023835466 | |
| 1559092_at | SLC9A5 | -2,311268652 | -2,335104118 | 0,023835466 | |
| 1559125_at | - | -3,07502285 | -3,098858316 | 0,023835466 | |
| 1559201_a_at | - | -2,187914861 | -2,211750327 | 0,023835466 | |
| 1559276_at | LOC728606 | -1,888254346 | -1,912089812 | 0,023835466 | |
| 1559369_at | C5orf44 | -2,597496523 | -2,621331989 | 0,023835466 | |

| | | | | | |
|---|---|---|---|---|---|
| 1559648_at | LOC100128420 | -2,384869671 | -2,408705138 | 0,023835466 | |
| 1559706_at | RGNEF | -2,712026959 | -2,735862426 | 0,023835466 | |
| 1559747_at | SPG11 | -1,888254346 | -1,912089812 | 0,023835466 | |
| 1559849_at | ZNF605 | -3,41365796 | -3,437493426 | 0,023835466 | |
| 1560060_s_at | VPS37C | 3,571118904 | 3,547283438 | 0,023835466 | |
| 1560169_at | - | -3,15396682 | -3,177802286 | 0,023835466 | |
| 1560318_at | ARHGAP29 | -2,930741289 | -2,954576755 | 0,023835466 | |
| 1560558_at | INIP | -2,756478458 | -2,780313924 | 0,023835466 | |
| 1560566_at | PCDH20 | -2,756478458 | -2,780313924 | 0,023835466 | |
| 1560570_a_at | - | -3,114791413 | -3,138626879 | 0,023835466 | |
| 1560683_at | NBEAP1 | -3,013665402 | -3,037500868 | 0,023835466 | |
| 1560707_at | LOC283856 | -2,01371626 | -2,037551727 | 0,023835466 | |
| 1560720_at | MKNK1 | -2,823166348 | -2,847001814 | 0,023835466 | |
| 1560734_at | LOC727924 /// O | -2,823166348 | -2,847001814 | 0,023835466 | |
| 1560746_at | NEXN-AS1 | -3,15396682 | -3,177802286 | 0,023835466 | |
| 1560884_at | SLC17A1 | -3,07502285 | -3,098858316 | 0,023835466 | |
| 1560987_a_at | - | -2,597496523 | -2,621331989 | 0,023835466 | |
| 1561030_at | TMC7 | -3,114791413 | -3,138626879 | 0,023835466 | |
| 1561105_at | CLDN11 | -2,823166348 | -2,847001814 | 0,023835466 | |
| 1561230_at | - | -3,15396682 | -3,177802286 | 0,023835466 | |
| 1561331_at | ERVMER61-1 | -1,089016378 | -1,112851844 | 0,023835466 | |
| 1561471_at | LOC441009 | -2,43257096 | -2,456406426 | 0,023835466 | |
| 1561534_at | - | -1,888254346 | -1,912089812 | 0,023835466 | |
| 1561543_at | - | -2,063149277 | -2,086984744 | 0,023835466 | |
| 1561550_at | - | -2,823166348 | -2,847001814 | 0,023835466 | |
| 1561561_x_at | - | -1,518072576 | -1,541908042 | 0,023835466 | |
| 1561635_at | - | -2,712026959 | -2,735862426 | 0,023835466 | |
| 1561813_at | - | -2,597496523 | -2,621331989 | 0,023835466 | |
| 1562110_at | - | -3,114791413 | -3,138626879 | 0,023835466 | |
| 1562223_at | LOC642426 | -3,41365796 | -3,437493426 | 0,023835466 | |
| 1562310_at | - | -2,930741289 | -2,954576755 | 0,023835466 | |
| 1562317_at | FAM186A | -2,930741289 | -2,954576755 | 0,023835466 | |
| 1562511_at | LYST | -2,063149277 | -2,086984744 | 0,023835466 | |
| 1562648_at | CCDC88A | -2,063149277 | -2,086984744 | 0,023835466 | |
| 1562722_at | PRR20A /// PRR2 | -2,187914861 | -2,211750327 | 0,023835466 | |
| 1562932_at | - | -2,756478458 | -2,780313924 | 0,023835466 | |
| 1562949_at | - | -2,756478458 | -2,780313924 | 0,023835466 | |
| 1562982_at | - | -2,01371626 | -2,037551727 | 0,023835466 | |
| 1563091_at | - | -2,823166348 | -2,847001814 | 0,023835466 | |
| 1563189_at | - | -2,384869671 | -2,408705138 | 0,023835466 | |
| 1563217_at | - | -2,930741289 | -2,954576755 | 0,023835466 | |
| 1563376_at | - | -2,756478458 | -2,780313924 | 0,023835466 | |
| 1563821_at | LOC170425 | -2,262747984 | -2,286583451 | 0,023835466 | |
| 1563876_at | C1orf220 | -3,013665402 | -3,037500868 | 0,023835466 | |
| 1564121_at | - | -2,597496523 | -2,621331989 | 0,023835466 | |
| 1564139_at | A2M-AS1 | -1,937711681 | -1,961547147 | 0,023835466 | |
| 1564287_at | LINC00410 | -2,01371626 | -2,037551727 | 0,023835466 | |
| 1564424_at | - | -2,01371626 | -2,037551727 | 0,023835466 | |
| 1564567_at | - | -3,013665402 | -3,037500868 | 0,023835466 | |
| 1564690_at | - | -3,013665402 | -3,037500868 | 0,023835466 | |
| 1565002_at | - | -1,888254346 | -1,912089812 | 0,023835466 | |
| 1565192_at | - | -2,43257096 | -2,456406426 | 0,023835466 | |
| 1565562_at | - | -1,888254346 | -1,912089812 | 0,023835466 | |
| 1565610_at | - | -1,888254346 | -1,912089812 | 0,023835466 | |
| 1565673_at | FCGR2A | -2,756478458 | -2,780313924 | 0,023835466 | |
| 1565685_at | LOC400940 | -3,114791413 | -3,138626879 | 0,023835466 | |

| | | | | | |
|---|---|---|---|---|---|
| 1565755_at | METAP1D | -3,114791413 | -3,138626879 | 0,023835466 | |
| 1565787_at | - | -2,43257096 | -2,456406426 | 0,023835466 | |
| 1565803_at | - | -1,196375121 | -1,220210588 | 0,023835466 | |
| 1566087_at | - | -3,07502285 | -3,098858316 | 0,023835466 | |
| 1566203_at | - | -2,823166348 | -2,847001814 | 0,023835466 | |
| 1566228_at | - | -1,196375121 | -1,220210588 | 0,023835466 | |
| 1566269_at | RALGAPA1 | -2,712026959 | -2,735862426 | 0,023835466 | |
| 1566445_at | - | -2,930741289 | -2,954576755 | 0,023835466 | |
| 1566581_at | - | -2,384869671 | -2,408705138 | 0,023835466 | |
| 1566645_at | - | -2,756478458 | -2,780313924 | 0,023835466 | |
| 1566691_at | - | -2,43257096 | -2,456406426 | 0,023835466 | |
| 1566726_at | - | -2,262747984 | -2,286583451 | 0,023835466 | |
| 1566935_at | TYRO3P | -2,01371626 | -2,037551727 | 0,023835466 | |
| 1567576_at | - | -3,013665402 | -3,037500868 | 0,023835466 | |
| 1567705_at | - | -2,930741289 | -2,954576755 | 0,023835466 | |
| 1568673_s_at | EAF2 | -3,114791413 | -3,138626879 | 0,023835466 | |
| 1568723_at | ATP9B | -2,01371626 | -2,037551727 | 0,023835466 | |
| 1568751_at | RGS13 | -3,15396682 | -3,177802286 | 0,023835466 | |
| 1568801_at | C15orf44 | -2,187914861 | -2,211750327 | 0,023835466 | |
| 1568932_at | - | -2,756478458 | -2,780313924 | 0,023835466 | |
| 1569061_at | IQGAP3 | -2,063149277 | -2,086984744 | 0,023835466 | |
| 1569156_at | ZNF846 | -2,384869671 | -2,408705138 | 0,023835466 | |
| 1569311_at | - | -2,384869671 | -2,408705138 | 0,023835466 | |
| 1569342_at | GLI3 | -2,187914861 | -2,211750327 | 0,023835466 | |
| 1569417_at | - | -1,518072576 | -1,541908042 | 0,023835466 | |
| 1569552_at | PTPN18 | -2,930741289 | -2,954576755 | 0,023835466 | |
| 1569610_at | IQCH | -2,823166348 | -2,847001814 | 0,023835466 | |
| 1569764_at | - | -2,43257096 | -2,456406426 | 0,023835466 | |
| 1569818_at | - | -2,823166348 | -2,847001814 | 0,023835466 | |
| 1569916_at | - | -3,114791413 | -3,138626879 | 0,023835466 | |
| 1570013_at | - | -2,311268652 | -2,335104118 | 0,023835466 | |
| 1570058_at | SUV420H2 | -2,43257096 | -2,456406426 | 0,023835466 | |
| 1570093_at | - | -2,187914861 | -2,211750327 | 0,023835466 | |
| 1570271_at | - | -2,384869671 | -2,408705138 | 0,023835466 | |
| 1570320_at | - | -2,712026959 | -2,735862426 | 0,023835466 | |
| 1570361_a_at | - | -2,311268652 | -2,335104118 | 0,023835466 | |
| 1570572_at | LOC729291 | -2,187914861 | -2,211750327 | 0,023835466 | |
| 200924_s_at | SLC3A2 | 3,010782559 | 2,986947093 | 0,023835466 | |
| 200930_s_at | VCL | -2,311268652 | -2,335104118 | 0,023835466 | |
| 200952_s_at | CCND2 | -2,262747984 | -2,286583451 | 0,023835466 | |
| 200986_at | SERPING1 | 2,069451339 | 2,045615873 | 0,023835466 | |
| 200995_at | IPO7 | 4,027552846 | 4,003717379 | 0,023835466 | |
| 201058_s_at | MYL9 | -1,518072576 | -1,541908042 | 0,023835466 | |
| 201348_at | GPX3 | -1,937711681 | -1,961547147 | 0,023835466 | |
| 201782_s_at | AIP | 3,262904562 | 3,239069096 | 0,023835466 | |
| 201811_x_at | SH3BP5 | 2,225103271 | 2,201267805 | 0,023835466 | |
| 201843_s_at | EFEMP1 | -3,114791413 | -3,138626879 | 0,023835466 | |
| 201936_s_at | EIF4G3 | 1,523472489 | 1,499637023 | 0,023835466 | |
| 202189_x_at | PTBP1 | 6,069354061 | 6,045518595 | 0,023835466 | |
| 202281_at | GAK | 1,649074067 | 1,6252386 | 0,023835466 | |
| 202493_x_at | CSH1 | -2,187914861 | -2,211750327 | 0,023835466 | |
| 202935_s_at | SOX9 | -2,823166348 | -2,847001814 | 0,023835466 | |
| 203188_at | B3GNT1 | 1,850809354 | 1,826973888 | 0,023835466 | |
| 203316_s_at | SNRPE | 6,704490558 | 6,680655092 | 0,023835466 | |
| 203717_at | DPP4 | -2,311268652 | -2,335104118 | 0,023835466 | |
| 203772_at | BLVRA | -2,063149277 | -2,086984744 | 0,023835466 | |

| | | | | | |
|---|---|---|---|---|---|
| 204030_s_at | IQCJ-SCHIP1 /// | -2,311268652 | -2,335104118 | 0,023835466 | |
| 204464_s_at | EDNRA | -2,712026959 | -2,735862426 | 0,023835466 | |
| 204584_at | L1CAM | -1,937711681 | -1,961547147 | 0,023835466 | |
| 204714_s_at | F5 | -1,888254346 | -1,912089812 | 0,023835466 | |
| 204758_s_at | C2CD2L | -1,888254346 | -1,912089812 | 0,023835466 | |
| 204913_s_at | SOX11 | -2,823166348 | -2,847001814 | 0,023835466 | |
| 205174_s_at | QPCT | -2,930741289 | -2,954576755 | 0,023835466 | |
| 205234_at | SLC16A4 | -3,013665402 | -3,037500868 | 0,023835466 | |
| 205488_at | GZMA | -1,937711681 | -1,961547147 | 0,023835466 | |
| 205556_at | MSX2 | -2,597496523 | -2,621331989 | 0,023835466 | |
| 205593_s_at | PDE9A | -1,089016378 | -1,112851844 | 0,023835466 | |
| 205999_x_at | CYP3A4 | -1,518072576 | -1,541908042 | 0,023835466 | |
| 206007_at | PRG4 | -2,930741289 | -2,954576755 | 0,023835466 | |
| 206083_at | BAI1 | -1,399512875 | -1,423348341 | 0,023835466 | |
| 206168_at | ZC3H7B | -1,937711681 | -1,961547147 | 0,023835466 | |
| 206214_at | PLA2G7 | -2,756478458 | -2,780313924 | 0,023835466 | |
| 206311_s_at | PLA2G1B | -2,311268652 | -2,335104118 | 0,023835466 | |
| 206330_s_at | SHC3 | -1,196375121 | -1,220210588 | 0,023835466 | |
| 206356_s_at | GNAL | -2,01371626 | -2,037551727 | 0,023835466 | |
| 206382_s_at | BDNF | -2,063149277 | -2,086984744 | 0,023835466 | |
| 206577_at | VIP | -2,712026959 | -2,735862426 | 0,023835466 | |
| 206584_at | LY96 | -2,597496523 | -2,621331989 | 0,023835466 | |
| 206840_at | AFM | -2,384869671 | -2,408705138 | 0,023835466 | |
| 206964_at | NAT8B | -1,518072576 | -1,541908042 | 0,023835466 | |
| 206984_s_at | RIT2 | -2,823166348 | -2,847001814 | 0,023835466 | |
| 207210_at | GABRA3 | -1,888254346 | -1,912089812 | 0,023835466 | |
| 207218_at | F9 | -2,712026959 | -2,735862426 | 0,023835466 | |
| 207250_at | SIX6 | -1,888254346 | -1,912089812 | 0,023835466 | |
| 207288_at | ASMTL-AS1 | -1,196375121 | -1,220210588 | 0,023835466 | |
| 207294_at | AGTR2 | -2,311268652 | -2,335104118 | 0,023835466 | |
| 207333_at | NMBR | -1,888254346 | -1,912089812 | 0,023835466 | |
| 207363_at | RS1 | -2,712026959 | -2,735862426 | 0,023835466 | |
| 207404_s_at | HTR1E | -1,518072576 | -1,541908042 | 0,023835466 | |
| 207422_at | ADAM20 | -1,399512875 | -1,423348341 | 0,023835466 | |
| 207472_at | RARS2 | -2,712026959 | -2,735862426 | 0,023835466 | |
| 207500_at | CASP5 | -1,518072576 | -1,541908042 | 0,023835466 | |
| 207577_at | HTR4 | -2,756478458 | -2,780313924 | 0,023835466 | |
| 207596_at | - | -2,712026959 | -2,735862426 | 0,023835466 | |
| 207603_at | SLC4A7 | -3,07502285 | -3,098858316 | 0,023835466 | |
| 207607_at | ASCL2 | -2,930741289 | -2,954576755 | 0,023835466 | |
| 207696_at | FUT9 | -3,114791413 | -3,138626879 | 0,023835466 | |
| 207728_at | ATF7IP | -2,756478458 | -2,780313924 | 0,023835466 | |
| 207799_x_at | ARMCX4 | -1,089016378 | -1,112851844 | 0,023835466 | |
| 207896_s_at | DLEC1 | -1,518072576 | -1,541908042 | 0,023835466 | |
| 207909_x_at | DAZ1 /// DAZ2 /// | -2,823166348 | -2,847001814 | 0,023835466 | |
| 208083_s_at | ITGB6 | -2,712026959 | -2,735862426 | 0,023835466 | |
| 208166_at | MMP16 | -2,823166348 | -2,847001814 | 0,023835466 | |
| 208233_at | PDPN | -2,43257096 | -2,456406426 | 0,023835466 | |
| 208338_at | P2RX3 | -2,262747984 | -2,286583451 | 0,023835466 | |
| 208348_s_at | CBLB | -2,597496523 | -2,621331989 | 0,023835466 | |
| 208448_x_at | IFNA16 | -2,384869671 | -2,408705138 | 0,023835466 | |
| 208561_at | ABCC9 | -2,930741289 | -2,954576755 | 0,023835466 | |
| 208574_at | SOX14 | -2,262747984 | -2,286583451 | 0,023835466 | |
| 209082_s_at | COL18A1 | 1,523472489 | 1,499637023 | 0,023835466 | |
| 209304_x_at | GADD45B | 1,104397092 | 1,080561626 | 0,023835466 | |
| 209630_s_at | FBXW2 | 3,234554895 | 3,210719429 | 0,023835466 | |

| | | | | | |
|---|---|---|---|---|---|
| 209720_s_at | SERPINB3 | -3,15396682 | -3,177802286 | 0,023835466 | |
| 209819_at | HABP4 | -2,823166348 | -2,847001814 | 0,023835466 | |
| 210278_s_at | AP4S1 | 2,082235068 | 2,058399602 | 0,023835466 | |
| 210459_at | PSMD4 | -1,196375121 | -1,220210588 | 0,023835466 | |
| 210558_at | AKR1C4 | -2,311268652 | -2,335104118 | 0,023835466 | |
| 211026_s_at | MGLL | -1,196375121 | -1,220210588 | 0,023835466 | |
| 211032_at | COBLL1 | -2,930741289 | -2,954576755 | 0,023835466 | |
| 211117_x_at | ESR2 | -1,399512875 | -1,423348341 | 0,023835466 | |
| 211239_s_at | ADAM7 | -3,013665402 | -3,037500868 | 0,023835466 | |
| 211275_s_at | GYG1 | 3,526916747 | 3,503081281 | 0,023835466 | |
| 211330_s_at | HFE | -2,187914861 | -2,211750327 | 0,023835466 | |
| 211337_s_at | TUBGCP4 | 2,35929703 | 2,335461563 | 0,023835466 | |
| 211361_s_at | SERPINB13 | -3,15396682 | -3,177802286 | 0,023835466 | |
| 211440_x_at | CYP3A43 | -2,712026959 | -2,735862426 | 0,023835466 | |
| 211525_s_at | GP5 | -2,597496523 | -2,621331989 | 0,023835466 | |
| 211585_at | NPAT | -2,311268652 | -2,335104118 | 0,023835466 | |
| 211782_at | IDS | -2,930741289 | -2,954576755 | 0,023835466 | |
| 212385_at | TCF4 | 1,104397092 | 1,080561626 | 0,023835466 | |
| 212479_s_at | RMND5A | 3,63237684 | 3,608541373 | 0,023835466 | |
| 212939_at | COL6A1 | -1,937711681 | -1,961547147 | 0,023835466 | |
| 213098_at | RQCD1 | 2,220484971 | 2,196649505 | 0,023835466 | |
| 213596_at | CASP4 | -2,063149277 | -2,086984744 | 0,023835466 | |
| 213912_at | TBC1D30 | -1,937711681 | -1,961547147 | 0,023835466 | |
| 214341_at | AP1G2 | -1,196375121 | -1,220210588 | 0,023835466 | |
| 214370_at | S100A8 | -2,43257096 | -2,456406426 | 0,023835466 | |
| 214410_at | - | -2,756478458 | -2,780313924 | 0,023835466 | |
| 214525_x_at | MLH3 | 1,104397092 | 1,080561626 | 0,023835466 | |
| 214735_at | IPCEF1 | -2,384869671 | -2,408705138 | 0,023835466 | |
| 214918_at | HNRNPM | -1,888254346 | -1,912089812 | 0,023835466 | |
| 214984_at | LOC100271836 / | -2,384869671 | -2,408705138 | 0,023835466 | |
| 215103_at | CYP2C18 | -2,756478458 | -2,780313924 | 0,023835466 | |
| 215164_at | - | -2,384869671 | -2,408705138 | 0,023835466 | |
| 215192_at | PMS2P4 | -2,384869671 | -2,408705138 | 0,023835466 | |
| 215308_at | XRCC6 | -1,888254346 | -1,912089812 | 0,023835466 | |
| 215671_at | PDE4B | -3,15396682 | -3,177802286 | 0,023835466 | |
| 215673_at | TEF | -3,013665402 | -3,037500868 | 0,023835466 | |
| 215817_at | SERPINB13 | -2,063149277 | -2,086984744 | 0,023835466 | |
| 216084_at | FAM205B | -2,712026959 | -2,735862426 | 0,023835466 | |
| 216097_at | - | -3,013665402 | -3,037500868 | 0,023835466 | |
| 216098_s_at | HTR7 /// HTR7P1 | -2,01371626 | -2,037551727 | 0,023835466 | |
| 216131_at | FRMD4B | -3,114791413 | -3,138626879 | 0,023835466 | |
| 216259_at | LOC100506699 | -2,01371626 | -2,037551727 | 0,023835466 | |
| 216403_at | SP3P | -2,43257096 | -2,456406426 | 0,023835466 | |
| 216415_at | DNAH3 | -2,311268652 | -2,335104118 | 0,023835466 | |
| 216433_s_at | PRDM2 | -2,187914861 | -2,211750327 | 0,023835466 | |
| 216541_x_at | IGHG1 /// IGHM | -2,187914861 | -2,211750327 | 0,023835466 | |
| 216709_at | - | -2,43257096 | -2,456406426 | 0,023835466 | |
| 216748_at | PYHIN1 | -1,399512875 | -1,423348341 | 0,023835466 | |
| 216828_at | MLLT10P1 | -2,384869671 | -2,408705138 | 0,023835466 | |
| 216871_at | - | -1,937711681 | -1,961547147 | 0,023835466 | |
| 217215_s_at | - | -2,01371626 | -2,037551727 | 0,023835466 | |
| 217271_at | - | -2,187914861 | -2,211750327 | 0,023835466 | |
| 217443_at | - | -2,712026959 | -2,735862426 | 0,023835466 | |
| 217639_at | - | -2,01371626 | -2,037551727 | 0,023835466 | |
| 218025_s_at | ECI2 | 5,067068688 | 5,043233221 | 0,023835466 | |
| 218191_s_at | LMBRD1 | 3,146001821 | 3,122166354 | 0,023835466 | |

| | | | | | |
|---|---|---|---|---|---|
| 218530_at | FHOD1 | 2,696781084 | 2,672945617 | 0,023835466 | |
| 218532_s_at | FAM134B | -2,597496523 | -2,621331989 | 0,023835466 | |
| 218612_s_at | TSSC4 | 1,631786706 | 1,60795124 | 0,023835466 | |
| 218676_s_at | PCTP | 2,577249059 | 2,553413592 | 0,023835466 | |
| 218906_x_at | KLC2 | 1,504602299 | 1,480766833 | 0,023835466 | |
| 219162_s_at | MRPL11 | 3,416167722 | 3,392332256 | 0,023835466 | |
| 219341_at | CLN8 | -1,196375121 | -1,220210588 | 0,023835466 | |
| 219568_x_at | SOX18 | -2,384869671 | -2,408705138 | 0,023835466 | |
| 219647_at | POPDC2 | -2,063149277 | -2,086984744 | 0,023835466 | |
| 220229_s_at | AP4E1 | -2,187914861 | -2,211750327 | 0,023835466 | |
| 220575_at | FAM106A | -2,384869671 | -2,408705138 | 0,023835466 | |
| 220625_s_at | ELF5 | -2,187914861 | -2,211750327 | 0,023835466 | |
| 220640_at | CSNK1G1 | -1,089016378 | -1,112851844 | 0,023835466 | |
| 220680_at | RAVER2 | -2,597496523 | -2,621331989 | 0,023835466 | |
| 220767_at | - | -2,311268652 | -2,335104118 | 0,023835466 | |
| 220883_at | PRO2012 | -2,597496523 | -2,621331989 | 0,023835466 | |
| 220893_at | LOC57399 | -3,07502285 | -3,098858316 | 0,023835466 | |
| 221200_at | ERVK3-2 | -2,187914861 | -2,211750327 | 0,023835466 | |
| 221425_s_at | ISCA1 | 2,355089531 | 2,331254065 | 0,023835466 | |
| 221587_s_at | C19orf24 | 2,540666392 | 2,516830926 | 0,023835466 | |
| 221886_at | DENND2A | -3,07502285 | -3,098858316 | 0,023835466 | |
| 222340_at | - | -2,063149277 | -2,086984744 | 0,023835466 | |
| 222867_s_at | MED31 | 3,784051878 | 3,760216412 | 0,023835466 | |
| 223606_x_at | KIAA1704 | 3,364775978 | 3,340940512 | 0,023835466 | |
| 223667_at | FKBP7 | -2,43257096 | -2,456406426 | 0,023835466 | |
| 223673_at | RFX4 | -2,823166348 | -2,847001814 | 0,023835466 | |
| 224014_at | ZNF160 | -1,196375121 | -1,220210588 | 0,023835466 | |
| 224213_at | - | -2,823166348 | -2,847001814 | 0,023835466 | |
| 224227_s_at | BDP1 | -2,384869671 | -2,408705138 | 0,023835466 | |
| 224278_at | CYP4F30P | -3,114791413 | -3,138626879 | 0,023835466 | |
| 224351_at | LOC100131508 | -3,15396682 | -3,177802286 | 0,023835466 | |
| 224402_s_at | FCRL4 | -2,823166348 | -2,847001814 | 0,023835466 | |
| 224590_at | XIST | -3,15396682 | -3,177802286 | 0,023835466 | |
| 225299_at | MYO5B | -2,384869671 | -2,408705138 | 0,023835466 | |
| 225353_s_at | C1QC | -2,063149277 | -2,086984744 | 0,023835466 | |
| 225381_at | MIR100HG | -2,311268652 | -2,335104118 | 0,023835466 | |
| 226122_at | PLEKHG1 | -2,43257096 | -2,456406426 | 0,023835466 | |
| 226682_at | RORA | -2,262747984 | -2,286583451 | 0,023835466 | |
| 226940_at | FAM69B | -2,01371626 | -2,037551727 | 0,023835466 | |
| 227441_s_at | ANKS1B | -2,823166348 | -2,847001814 | 0,023835466 | |
| 227708_at | EEF1A1 /// LOC1 | 3,046446467 | 3,022611001 | 0,023835466 | |
| 227779_at | - | -1,518072576 | -1,541908042 | 0,023835466 | |
| 227955_s_at | EFNA5 | -2,384869671 | -2,408705138 | 0,023835466 | |
| 228115_at | FAM59A | -2,187914861 | -2,211750327 | 0,023835466 | |
| 228148_at | ZNF584 | 1,638726528 | 1,614891062 | 0,023835466 | |
| 228166_at | ALG2 | -1,196375121 | -1,220210588 | 0,023835466 | |
| 228237_at | PAPPA2 | -2,384869671 | -2,408705138 | 0,023835466 | |
| 228459_at | FAM84A | -2,262747984 | -2,286583451 | 0,023835466 | |
| 228470_at | RNF187 | -2,187914861 | -2,211750327 | 0,023835466 | |
| 228608_at | NALCN | -2,311268652 | -2,335104118 | 0,023835466 | |
| 228818_at | - | -2,311268652 | -2,335104118 | 0,023835466 | |
| 229163_at | - | -2,063149277 | -2,086984744 | 0,023835466 | |
| 229327_s_at | - | -3,15396682 | -3,177802286 | 0,023835466 | |
| 229339_at | MYOCD | -1,518072576 | -1,541908042 | 0,023835466 | |
| 229478_x_at | BIVM | -2,712026959 | -2,735862426 | 0,023835466 | |
| 229620_at | SEPP1 | -2,823166348 | -2,847001814 | 0,023835466 | |

| | | | | | |
|---|---|---|---|---|---|
| 229649_at | NRXN3 | -2,384869671 | -2,408705138 | 0,023835466 | |
| 230045_at | CNTN2 | -2,187914861 | -2,211750327 | 0,023835466 | |
| 230074_s_at | - | -1,196375121 | -1,220210588 | 0,023835466 | |
| 230122_at | MLLT10 | 1,05337236 | 1,029536893 | 0,023835466 | |
| 230127_at | - | -1,888254346 | -1,912089812 | 0,023835466 | |
| 230144_at | GRIA3 | -2,756478458 | -2,780313924 | 0,023835466 | |
| 230331_at | METTL21C | -2,823166348 | -2,847001814 | 0,023835466 | |
| 230550_at | MS4A6A | -2,43257096 | -2,456406426 | 0,023835466 | |
| 230573_at | SGK2 | -2,823166348 | -2,847001814 | 0,023835466 | |
| 230620_at | USP27X | 1,742232072 | 1,718396606 | 0,023835466 | |
| 230644_at | LRFN5 | -2,01371626 | -2,037551727 | 0,023835466 | |
| 230746_s_at | - | -2,756478458 | -2,780313924 | 0,023835466 | |
| 230914_at | HNF4A | -1,196375121 | -1,220210588 | 0,023835466 | |
| 231473_at | LOC100506530 | -2,930741289 | -2,954576755 | 0,023835466 | |
| 231486_x_at | - | -2,823166348 | -2,847001814 | 0,023835466 | |
| 231579_s_at | TIMP2 | -2,311268652 | -2,335104118 | 0,023835466 | |
| 231613_at | - | -2,756478458 | -2,780313924 | 0,023835466 | |
| 231617_at | TEX33 | -2,384869671 | -2,408705138 | 0,023835466 | |
| 231662_at | ARG1 | -2,384869671 | -2,408705138 | 0,023835466 | |
| 231771_at | GJB6 | -2,597496523 | -2,621331989 | 0,023835466 | |
| 231775_at | TNFRSF10A | 2,540666392 | 2,516830926 | 0,023835466 | |
| 231933_at | 08.03.15 | -1,089016378 | -1,112851844 | 0,023835466 | |
| 231967_at | PHF20L1 | 1,46610492 | 1,442269454 | 0,023835466 | |
| 232039_at | KIAA1383 | -2,930741289 | -2,954576755 | 0,023835466 | |
| 232090_at | DNM3OS | -2,823166348 | -2,847001814 | 0,023835466 | |
| 232163_at | WDR19 | -1,937711681 | -1,961547147 | 0,023835466 | |
| 232275_s_at | HS6ST3 | -3,114791413 | -3,138626879 | 0,023835466 | |
| 232314_at | MTFR1 | -2,712026959 | -2,735862426 | 0,023835466 | |
| 232738_at | FAM190A | -2,187914861 | -2,211750327 | 0,023835466 | |
| 232743_at | - | -2,262747984 | -2,286583451 | 0,023835466 | |
| 232826_at | - | -1,518072576 | -1,541908042 | 0,023835466 | |
| 232958_at | - | -2,187914861 | -2,211750327 | 0,023835466 | |
| 233016_at | LOC100506546 | -2,187914861 | -2,211750327 | 0,023835466 | |
| 233040_at | PLEKHA5 | -3,15396682 | -3,177802286 | 0,023835466 | |
| 233046_at | - | -3,114791413 | -3,138626879 | 0,023835466 | |
| 233092_s_at | LOC100271840 | -2,823166348 | -2,847001814 | 0,023835466 | |
| 233113_at | ADAMTS9-AS2 | -2,756478458 | -2,780313924 | 0,023835466 | |
| 233166_at | DEFB126 | -3,114791413 | -3,138626879 | 0,023835466 | |
| 233709_at | - | -2,43257096 | -2,456406426 | 0,023835466 | |
| 233798_at | - | -2,756478458 | -2,780313924 | 0,023835466 | |
| 234035_at | HNRNPM | -1,399512875 | -1,423348341 | 0,023835466 | |
| 234165_at | PTGDR | -2,597496523 | -2,621331989 | 0,023835466 | |
| 234350_at | IGLC1 | -2,187914861 | -2,211750327 | 0,023835466 | |
| 234392_at | - | -2,262747984 | -2,286583451 | 0,023835466 | |
| 234404_at | CAND2 | -1,888254346 | -1,912089812 | 0,023835466 | |
| 234503_at | - | -2,712026959 | -2,735862426 | 0,023835466 | |
| 234548_at | - | -2,823166348 | -2,847001814 | 0,023835466 | |
| 234627_at | FLJ21408 | -2,01371626 | -2,037551727 | 0,023835466 | |
| 234849_at | YME1L1 | -2,712026959 | -2,735862426 | 0,023835466 | |
| 235030_at | NXPE3 | -2,063149277 | -2,086984744 | 0,023835466 | |
| 235236_at | - | -2,712026959 | -2,735862426 | 0,023835466 | |
| 235251_at | - | -2,187914861 | -2,211750327 | 0,023835466 | |
| 235267_at | MAGI2-AS3 | -1,888254346 | -1,912089812 | 0,023835466 | |
| 235287_at | CDK6 | -1,888254346 | -1,912089812 | 0,023835466 | |
| 235328_at | PLXNC1 | 1,104397092 | 1,080561626 | 0,023835466 | |
| 235468_at | RBFOX3 | -1,937711681 | -1,961547147 | 0,023835466 | |

| | | | | | |
|---|---|---|---|---|---|
| 235633_at | - | -1,196375121 | -1,220210588 | 0,023835466 | |
| 235636_at | - | -1,196375121 | -1,220210588 | 0,023835466 | |
| 235818_at | VSTM1 | -2,384869671 | -2,408705138 | 0,023835466 | |
| 235838_at | NLGN2 | -1,518072576 | -1,541908042 | 0,023835466 | |
| 235986_at | - | -1,888254346 | -1,912089812 | 0,023835466 | |
| 235999_at | - | -2,01371626 | -2,037551727 | 0,023835466 | |
| 236003_x_at | - | -1,937711681 | -1,961547147 | 0,023835466 | |
| 236138_at | LMBRD2 | -3,07502285 | -3,098858316 | 0,023835466 | |
| 236141_at | NBLA00301 | -2,384869671 | -2,408705138 | 0,023835466 | |
| 236234_at | PDE1A | -2,712026959 | -2,735862426 | 0,023835466 | |
| 236363_at | LSM3 | -1,399512875 | -1,423348341 | 0,023835466 | |
| 236466_at | - | -2,597496523 | -2,621331989 | 0,023835466 | |
| 236523_at | LOC285556 | -2,756478458 | -2,780313924 | 0,023835466 | |
| 236694_at | TXLNG2P | -3,013665402 | -3,037500868 | 0,023835466 | |
| 237047_at | LINC00281 | -2,262747984 | -2,286583451 | 0,023835466 | |
| 237070_at | TRPM1 | -3,07502285 | -3,098858316 | 0,023835466 | |
| 237121_at | - | -2,063149277 | -2,086984744 | 0,023835466 | |
| 237130_at | - | -1,196375121 | -1,220210588 | 0,023835466 | |
| 237182_at | MRPL45P2 | -3,15396682 | -3,177802286 | 0,023835466 | |
| 237235_at | - | -1,518072576 | -1,541908042 | 0,023835466 | |
| 237339_at | - | -1,937711681 | -1,961547147 | 0,023835466 | |
| 237356_at | - | -2,311268652 | -2,335104118 | 0,023835466 | |
| 237436_at | - | -2,063149277 | -2,086984744 | 0,023835466 | |
| 237462_at | - | -2,384869671 | -2,408705138 | 0,023835466 | |
| 237490_at | - | -2,597496523 | -2,621331989 | 0,023835466 | |
| 237514_at | LOC100507274 | -2,597496523 | -2,621331989 | 0,023835466 | |
| 237519_at | LOC100506880 | -2,43257096 | -2,456406426 | 0,023835466 | |
| 237533_at | - | -1,399512875 | -1,423348341 | 0,023835466 | |
| 237576_x_at | LOC100506480 | -1,888254346 | -1,912089812 | 0,023835466 | |
| 237805_at | - | -2,384869671 | -2,408705138 | 0,023835466 | |
| 237819_at | CREB3L2 | -1,518072576 | -1,541908042 | 0,023835466 | |
| 237877_at | - | -2,756478458 | -2,780313924 | 0,023835466 | |
| 237917_at | NBPF8 | -2,597496523 | -2,621331989 | 0,023835466 | |
| 237937_x_at | - | -3,15396682 | -3,177802286 | 0,023835466 | |
| 237950_s_at | - | -2,262747984 | -2,286583451 | 0,023835466 | |
| 238098_at | - | -1,888254346 | -1,912089812 | 0,023835466 | |
| 238133_at | - | -2,063149277 | -2,086984744 | 0,023835466 | |
| 238138_at | CANX | -1,399512875 | -1,423348341 | 0,023835466 | |
| 238236_at | - | -2,01371626 | -2,037551727 | 0,023835466 | |
| 238283_at | LOC151658 | -2,823166348 | -2,847001814 | 0,023835466 | |
| 238372_s_at | - | -2,43257096 | -2,456406426 | 0,023835466 | |
| 238615_at | ERLIN2 | -2,384869671 | -2,408705138 | 0,023835466 | |
| 238644_at | MYSM1 | -1,196375121 | -1,220210588 | 0,023835466 | |
| 238670_at | RAD18 | -1,089016378 | -1,112851844 | 0,023835466 | |
| 238674_at | - | -2,063149277 | -2,086984744 | 0,023835466 | |
| 238731_at | SETDB2 | -2,063149277 | -2,086984744 | 0,023835466 | |
| 238805_at | C11orf52 /// HSP | -1,937711681 | -1,961547147 | 0,023835466 | |
| 238847_at | HOXD10 | -2,01371626 | -2,037551727 | 0,023835466 | |
| 238958_at | UBE2E1 | -2,756478458 | -2,780313924 | 0,023835466 | |
| 239298_at | - | -2,384869671 | -2,408705138 | 0,023835466 | |
| 239305_at | - | -2,311268652 | -2,335104118 | 0,023835466 | |
| 239356_at | DOCK9-AS2 | -2,187914861 | -2,211750327 | 0,023835466 | |
| 239454_at | SCARF2 | -2,187914861 | -2,211750327 | 0,023835466 | |
| 239455_at | - | -2,01371626 | -2,037551727 | 0,023835466 | |
| 239507_at | LINC00608 | -2,187914861 | -2,211750327 | 0,023835466 | |
| 239541_at | LOC100505817 | -2,311268652 | -2,335104118 | 0,023835466 | |

| | | | | | |
|---|---|---|---|---|---|
| 239797_at | - | -2,01371626 | -2,037551727 | 0,023835466 | |
| 239878_at | IPMK | -2,43257096 | -2,456406426 | 0,023835466 | |
| 240016_at | - | -1,399512875 | -1,423348341 | 0,023835466 | |
| 240039_at | PLA2R1 | -1,888254346 | -1,912089812 | 0,023835466 | |
| 240295_at | - | -1,089016378 | -1,112851844 | 0,023835466 | |
| 240312_at | - | -2,384869671 | -2,408705138 | 0,023835466 | |
| 240414_at | - | -1,937711681 | -1,961547147 | 0,023835466 | |
| 240629_at | - | -2,384869671 | -2,408705138 | 0,023835466 | |
| 240714_at | - | -2,756478458 | -2,780313924 | 0,023835466 | |
| 240718_at | - | -2,756478458 | -2,780313924 | 0,023835466 | |
| 240789_at | - | -2,384869671 | -2,408705138 | 0,023835466 | |
| 240904_at | - | -2,712026959 | -2,735862426 | 0,023835466 | |
| 240994_at | - | -2,712026959 | -2,735862426 | 0,023835466 | |
| 241044_x_at | - | -2,930741289 | -2,954576755 | 0,023835466 | |
| 241082_at | ASNS | -2,43257096 | -2,456406426 | 0,023835466 | |
| 241211_at | - | -2,01371626 | -2,037551727 | 0,023835466 | |
| 241226_at | - | -1,888254346 | -1,912089812 | 0,023835466 | |
| 241242_at | - | -2,01371626 | -2,037551727 | 0,023835466 | |
| 241405_at | LOC400604 | -1,518072576 | -1,541908042 | 0,023835466 | |
| 241421_at | - | -2,597496523 | -2,621331989 | 0,023835466 | |
| 241647_x_at | - | -3,287237186 | -3,311072652 | 0,023835466 | |
| 241685_x_at | - | -3,013665402 | -3,037500868 | 0,023835466 | |
| 241711_at | - | -1,399512875 | -1,423348341 | 0,023835466 | |
| 241735_at | RASSF8 | -1,888254346 | -1,912089812 | 0,023835466 | |
| 241760_x_at | RORA | -3,114791413 | -3,138626879 | 0,023835466 | |
| 241987_x_at | SNX31 | -2,823166348 | -2,847001814 | 0,023835466 | |
| 241990_at | RHOV | -1,089016378 | -1,112851844 | 0,023835466 | |
| 242001_at | - | -2,063149277 | -2,086984744 | 0,023835466 | |
| 242050_at | LRRC7 | -1,196375121 | -1,220210588 | 0,023835466 | |
| 242152_at | - | -2,384869671 | -2,408705138 | 0,023835466 | |
| 242230_at | ATXN1 | -2,187914861 | -2,211750327 | 0,023835466 | |
| 242236_at | - | -3,013665402 | -3,037500868 | 0,023835466 | |
| 242319_at | DGKG | -2,063149277 | -2,086984744 | 0,023835466 | |
| 242415_at | FBRSL1 | -1,399512875 | -1,423348341 | 0,023835466 | |
| 242453_at | - | -2,063149277 | -2,086984744 | 0,023835466 | |
| 242544_x_at | - | -3,013665402 | -3,037500868 | 0,023835466 | |
| 242591_at | DNAH1 | -2,43257096 | -2,456406426 | 0,023835466 | |
| 242625_at | RSAD2 | -2,262747984 | -2,286583451 | 0,023835466 | |
| 242690_at | ATP11C | -2,384869671 | -2,408705138 | 0,023835466 | |
| 242991_at | TJP2 | -2,597496523 | -2,621331989 | 0,023835466 | |
| 243015_at | CYP3A5 | -3,013665402 | -3,037500868 | 0,023835466 | |
| 243082_at | - | -2,823166348 | -2,847001814 | 0,023835466 | |
| 243251_at | - | -1,518072576 | -1,541908042 | 0,023835466 | |
| 243329_at | - | -1,196375121 | -1,220210588 | 0,023835466 | |
| 243337_at | FREM3 | -3,013665402 | -3,037500868 | 0,023835466 | |
| 243421_at | - | -3,114791413 | -3,138626879 | 0,023835466 | |
| 243441_at | HOXB-AS4 | -1,089016378 | -1,112851844 | 0,023835466 | |
| 243689_s_at | FRG1B | -2,712026959 | -2,735862426 | 0,023835466 | |
| 243708_at | TMEM132E | -2,063149277 | -2,086984744 | 0,023835466 | |
| 243723_at | - | -2,384869671 | -2,408705138 | 0,023835466 | |
| 243805_at | CCBE1 | -2,187914861 | -2,211750327 | 0,023835466 | |
| 243871_at | LOC100130476 | -2,597496523 | -2,621331989 | 0,023835466 | |
| 243933_at | - | -2,311268652 | -2,335104118 | 0,023835466 | |
| 243974_at | - | -3,07502285 | -3,098858316 | 0,023835466 | |
| 244117_at | - | -2,756478458 | -2,780313924 | 0,023835466 | |
| 244138_at | - | -2,597496523 | -2,621331989 | 0,023835466 | |

| | | | | | |
|---|---|---|---|---|---|
| 244189_at | TTC28-AS1 | 2,2992452 | 2,275409734 | 0,023835466 | |
| 244228_at | - | -2,823166348 | -2,847001814 | 0,023835466 | |
| 244345_at | CADM1 | -2,187914861 | -2,211750327 | 0,023835466 | |
| 244407_at | CYP39A1 | -3,013665402 | -3,037500868 | 0,023835466 | |
| 244440_at | - | -2,262747984 | -2,286583451 | 0,023835466 | |
| 244451_x_at | - | -2,311268652 | -2,335104118 | 0,023835466 | |
| 244507_at | - | -2,712026959 | -2,735862426 | 0,023835466 | |
| 244596_at | - | -1,888254346 | -1,912089812 | 0,023835466 | |
| 244651_at | - | -2,262747984 | -2,286583451 | 0,023835466 | |
| 244664_at | - | 1,774096855 | 1,750261389 | 0,023835466 | |
| 244723_at | ACSM2A | -3,013665402 | -3,037500868 | 0,023835466 | |
| 244728_at | - | -1,196375121 | -1,220210588 | 0,023835466 | |
| 244781_x_at | - | -2,262747984 | -2,286583451 | 0,023835466 | |
| 48531_at | TNIP2 | 2,279399592 | 2,255564126 | 0,023835466 | |
| 58916_at | KCTD14 /// NDUI | -2,222460895 | -2,246296361 | 0,023835466 | |
| 1570382_at | - | -0,234278768 | -0,258114234 | 0,023835466 | |
| 202912_at | ADM | 0,518867317 | 0,495031851 | 0,023835466 | |
| 204896_s_at | PTGER4 | -0,234278768 | -0,258114234 | 0,023835466 | |
| 208435_s_at | AQP6 | -0,299034815 | -0,322870281 | 0,023835466 | |
| 210307_s_at | KLHL25 | 0,984222186 | 0,960386719 | 0,023835466 | |
| 217207_s_at | BTNL3 | 0,715481152 | 0,691645685 | 0,023835466 | |
| 225168_at | FRMD4A | 0,518867317 | 0,495031851 | 0,023835466 | |
| 226403_at | TMC4 | -0,299034815 | -0,322870281 | 0,023835466 | |
| 227423_at | LRRC28 | 0,984222186 | 0,960386719 | 0,023835466 | |
| 229161_at | - | -0,234278768 | -0,258114234 | 0,023835466 | |
| 236094_at | TCF7L2 | -0,234278768 | -0,258114234 | 0,023835466 | |
| 239424_at | - | -0,299034815 | -0,322870281 | 0,023835466 | |
| 243061_at | C14orf23 | -0,234278768 | -0,258114234 | 0,023835466 | |
| 244160_at | - | -0,299034815 | -0,322870281 | 0,023835466 | |
| 1553246_a_at | SGK196 | -0,136301282 | -0,160136748 | 0,023835466 | |
| 1562789_at | ZNF229 | -0,136301282 | -0,160136748 | 0,023835466 | |
| 1563800_at | LOC100506870 / | -0,136301282 | -0,160136748 | 0,023835466 | |
| 200907_s_at | PALLD | -0,196756757 | -0,220592223 | 0,023835466 | |
| 204685_s_at | ATP2B2 | -0,136301282 | -0,160136748 | 0,023835466 | |
| 215217_at | IGKC | -0,136301282 | -0,160136748 | 0,023835466 | |
| 216265_x_at | MYH7 | -0,196756757 | -0,220592223 | 0,023835466 | |
| 217253_at | SH3BP2 | -0,196756757 | -0,220592223 | 0,023835466 | |
| 225615_at | IFFO2 | -0,136301282 | -0,160136748 | 0,023835466 | |
| 228419_at | METRN | -0,196756757 | -0,220592223 | 0,023835466 | |
| 229135_at | FASTKD2 | -0,196756757 | -0,220592223 | 0,023835466 | |
| 229699_at | LOC100129550 | -0,136301282 | -0,160136748 | 0,023835466 | |
| 232370_at | LOC254057 | -0,136301282 | -0,160136748 | 0,023835466 | |
| 235792_x_at | PIK3C2A | -0,136301282 | -0,160136748 | 0,023835466 | |
| 1559789_a_at | ZNF781 | 0,503742507 | 0,479907041 | 0,023835466 | |
| 1561270_at | - | -0,325749314 | -0,34958478 | 0,023835466 | |
| 1566257_at | GPR180 | 0,44160593 | 0,417770464 | 0,023835466 | |
| 202439_s_at | IDS | -0,325749314 | -0,34958478 | 0,023835466 | |
| 203058_s_at | PAPSS2 | -0,325749314 | -0,34958478 | 0,023835466 | |
| 203894_at | TUBG2 | -0,325749314 | -0,34958478 | 0,023835466 | |
| 219772_s_at | SMPX | -0,325749314 | -0,34958478 | 0,023835466 | |
| 227499_at | FZD3 | 0,44160593 | 0,417770464 | 0,023835466 | |
| 231912_s_at | TECPR1 | 0,44160593 | 0,417770464 | 0,023835466 | |
| 232965_at | LOC400684 | -0,325749314 | -0,34958478 | 0,023835466 | |
| 235131_at | RHOJ | -0,325749314 | -0,34958478 | 0,023835466 | |
| 242012_at | - | -0,325749314 | -0,34958478 | 0,023835466 | |
| 1552395_at | TSSK3 | 1,560488174 | 1,536652708 | 0,023835466 | |

| | | | | | |
|---|---|---|---|---|---|
| 1552473_at | GAMT | 1,799091453 | 1,775255987 | 0,023835466 | |
| 1552485_at | LACTB | 1,716223609 | 1,692388143 | 0,023835466 | |
| 1553023_a_at | NOX5 | -1,308181863 | -1,332017329 | 0,023835466 | |
| 1553031_at | GPR115 | -1,308181863 | -1,332017329 | 0,023835466 | |
| 1553512_at | HOXC12 | -1,470833215 | -1,494668682 | 0,023835466 | |
| 1553881_at | MGC16142 | -1,763254337 | -1,787089803 | 0,023835466 | |
| 1554276_at | SART3 | -1,838812296 | -1,862647763 | 0,023835466 | |
| 1554411_at | CTNNB1 | 1,238353345 | 1,214517879 | 0,023835466 | |
| 1554584_at | LRRC48 | -0,706943342 | -0,730778808 | 0,023835466 | |
| 1554666_at | LOC100130950 | -0,79540287 | -0,819238336 | 0,023835466 | |
| 1555096_at | C4orf37 | -0,79540287 | -0,819238336 | 0,023835466 | |
| 1555437_at | EARS2 | -1,353583928 | -1,377419394 | 0,023835466 | |
| 1555620_a_at | PTGIR | 1,683039483 | 1,659204017 | 0,023835466 | |
| 1556063_s_at | RPP30 | 0,747935635 | 0,724100169 | 0,023835466 | |
| 1556338_at | - | -1,026571149 | -1,050406615 | 0,023835466 | |
| 1556344_at | LOC150051 | -1,353583928 | -1,377419394 | 0,023835466 | |
| 1556608_a_at | EHD4 | -1,470833215 | -1,494668682 | 0,023835466 | |
| 1556676_a_at | - | -0,706943342 | -0,730778808 | 0,023835466 | |
| 1557162_at | C16orf92 | -1,591296941 | -1,615132408 | 0,023835466 | |
| 1557287_at | - | -1,838812296 | -1,862647763 | 0,023835466 | |
| 1557405_at | LOC100130111 | -1,71423189 | -1,738067356 | 0,023835466 | |
| 1557466_at | FLJ31104 | -1,639579028 | -1,663414495 | 0,023835466 | |
| 1557575_at | STBD1 | -1,353583928 | -1,377419394 | 0,023835466 | |
| 1557693_at | LOC100507299 | -1,240169874 | -1,264005341 | 0,023835466 | |
| 1558447_at | - | -1,71423189 | -1,738067356 | 0,023835466 | |
| 1558512_at | - | -1,131062212 | -1,154897679 | 0,023835466 | |
| 1558739_at | - | -1,470833215 | -1,494668682 | 0,023835466 | |
| 1558758_s_at | - | -0,622910612 | -0,646746079 | 0,023835466 | |
| 1559384_at | - | -1,639579028 | -1,663414495 | 0,023835466 | |
| 1559891_at | HMGA2 | -1,240169874 | -1,264005341 | 0,023835466 | |
| 1559948_at | - | -1,838812296 | -1,862647763 | 0,023835466 | |
| 1560316_s_at | GLCCI1 | -0,622910612 | -0,646746079 | 0,023835466 | |
| 1560747_at | - | -1,838812296 | -1,862647763 | 0,023835466 | |
| 1561060_at | LOC100506439 | -0,706943342 | -0,730778808 | 0,023835466 | |
| 1561273_at | - | -1,240169874 | -1,264005341 | 0,023835466 | |
| 1561500_at | CTU2 | -1,131062212 | -1,154897679 | 0,023835466 | |
| 1562005_at | RIN3 | -1,591296941 | -1,615132408 | 0,023835466 | |
| 1562158_at | - | -1,591296941 | -1,615132408 | 0,023835466 | |
| 1563036_at | - | -1,71423189 | -1,738067356 | 0,023835466 | |
| 1563691_at | LOC285300 | -1,71423189 | -1,738067356 | 0,023835466 | |
| 1564352_at | CECR3 | -1,639579028 | -1,663414495 | 0,023835466 | |
| 1564426_x_at | LOC729732 | -1,763254337 | -1,787089803 | 0,023835466 | |
| 1565620_at | AGAP4 | -1,240169874 | -1,264005341 | 0,023835466 | |
| 1565738_at | SCTR | -1,639579028 | -1,663414495 | 0,023835466 | |
| 1565751_at | FGD2 | -0,79540287 | -0,819238336 | 0,023835466 | |
| 1566235_at | DGCR12 | -1,470833215 | -1,494668682 | 0,023835466 | |
| 1566776_at | DNAH1 | -1,131062212 | -1,154897679 | 0,023835466 | |
| 1566986_at | - | -1,470833215 | -1,494668682 | 0,023835466 | |
| 1566999_at | - | -1,308181863 | -1,332017329 | 0,023835466 | |
| 1567559_s_at | TREML4 | -1,763254337 | -1,787089803 | 0,023835466 | |
| 1568730_at | - | -1,240169874 | -1,264005341 | 0,023835466 | |
| 1568889_at | FANCD2 | -1,639579028 | -1,663414495 | 0,023835466 | |
| 1569182_at | - | -1,838812296 | -1,862647763 | 0,023835466 | |
| 1569207_s_at | TCP11L2 | -1,71423189 | -1,738067356 | 0,023835466 | |
| 1569237_at | - | -1,639579028 | -1,663414495 | 0,023835466 | |
| 1569629_x_at | LOC389906 | 1,709647566 | 1,685812099 | 0,023835466 | |

| | | | | | |
|---|---|---|---|---|---|
| 1569668_at | - | -1,308181863 | -1,332017329 | 0,023835466 | |
| 1569791_at | STK4 | -1,763254337 | -1,787089803 | 0,023835466 | |
| 1569991_at | KY | -1,026571149 | -1,050406615 | 0,023835466 | |
| 1570040_at | - | -1,308181863 | -1,332017329 | 0,023835466 | |
| 1570329_at | - | -1,71423189 | -1,738067356 | 0,023835466 | |
| 201354_s_at | BAZ2A | 1,215326747 | 1,191491281 | 0,023835466 | |
| 201884_at | CEACAM5 | -1,470833215 | -1,494668682 | 0,023835466 | |
| 202685_s_at | AXL | 1,261018186 | 1,23718272 | 0,023835466 | |
| 203691_at | PI3 | -1,026571149 | -1,050406615 | 0,023835466 | |
| 204097_s_at | RBMX2 | 1,549482847 | 1,525647381 | 0,023835466 | |
| 204509_at | CA12 | -1,470833215 | -1,494668682 | 0,023835466 | |
| 204542_at | ST6GALNAC2 | -1,240169874 | -1,264005341 | 0,023835466 | |
| 204799_at | ZBED4 | 1,542099044 | 1,518263577 | 0,023835466 | |
| 204875_s_at | GMDS | 1,296557217 | 1,272721751 | 0,023835466 | |
| 205098_at | CCR1 | -1,470833215 | -1,494668682 | 0,023835466 | |
| 205287_s_at | TFAP2C | -1,763254337 | -1,787089803 | 0,023835466 | |
| 205454_at | HPCA | -1,308181863 | -1,332017329 | 0,023835466 | |
| 205651_x_at | RAPGEF4 | -1,353583928 | -1,377419394 | 0,023835466 | |
| 205921_s_at | SLC6A6 | -1,763254337 | -1,787089803 | 0,023835466 | |
| 206316_s_at | KNTC1 | 1,40228911 | 1,378453643 | 0,023835466 | |
| 206601_s_at | HOXD3 | -1,838812296 | -1,862647763 | 0,023835466 | |
| 206750_at | MAFK | -1,026571149 | -1,050406615 | 0,023835466 | |
| 206866_at | CDH4 | -1,131062212 | -1,154897679 | 0,023835466 | |
| 206887_at | CCBP2 | -0,622910612 | -0,646746079 | 0,023835466 | |
| 207077_at | CELA2B | -1,470833215 | -1,494668682 | 0,023835466 | |
| 207152_at | NTRK2 | -1,308181863 | -1,332017329 | 0,023835466 | |
| 207304_at | ZNF45 | 1,820612054 | 1,796776588 | 0,023835466 | |
| 207505_at | PRKG2 | -1,763254337 | -1,787089803 | 0,023835466 | |
| 207723_s_at | KLRC3 | -1,838812296 | -1,862647763 | 0,023835466 | |
| 207995_s_at | CLEC4M | -0,706943342 | -0,730778808 | 0,023835466 | |
| 208004_at | PROL1 | -1,71423189 | -1,738067356 | 0,023835466 | |
| 208594_x_at | LILRA6 | -1,71423189 | -1,738067356 | 0,023835466 | |
| 209521_s_at | AMOT | -1,591296941 | -1,615132408 | 0,023835466 | |
| 210304_at | PDE6B | -1,026571149 | -1,050406615 | 0,023835466 | |
| 210652_s_at | TTC39A | -1,639579028 | -1,663414495 | 0,023835466 | |
| 210674_s_at | PCDHA1 /// PCD | -1,240169874 | -1,264005341 | 0,023835466 | |
| 210998_s_at | HGF | -1,763254337 | -1,787089803 | 0,023835466 | |
| 211249_at | GPR68 | -1,470833215 | -1,494668682 | 0,023835466 | |
| 211650_x_at | IGH@ /// IGHA1 / | -1,353583928 | -1,377419394 | 0,023835466 | |
| 211836_s_at | MOG | -0,706943342 | -0,730778808 | 0,023835466 | |
| 211915_s_at | TUBB7P | -1,591296941 | -1,615132408 | 0,023835466 | |
| 212444_at | GPRC5A | -0,79540287 | -0,819238336 | 0,023835466 | |
| 213421_x_at | PRSS3 | -0,622910612 | -0,646746079 | 0,023835466 | |
| 213821_s_at | IDS | -1,353583928 | -1,377419394 | 0,023835466 | |
| 213954_at | FAM169A | -1,353583928 | -1,377419394 | 0,023835466 | |
| 214357_at | C1orf105 | -1,639579028 | -1,663414495 | 0,023835466 | |
| 214492_at | SGCD | -1,470833215 | -1,494668682 | 0,023835466 | |
| 214555_at | SSTR5 | -1,591296941 | -1,615132408 | 0,023835466 | |
| 214792_x_at | VAMP2 | -1,71423189 | -1,738067356 | 0,023835466 | |
| 214817_at | UNC13A | -0,622910612 | -0,646746079 | 0,023835466 | |
| 215024_at | CCZ1B | -1,639579028 | -1,663414495 | 0,023835466 | |
| 215035_at | IGLV6-57 | -1,639579028 | -1,663414495 | 0,023835466 | |
| 215252_at | - | -1,470833215 | -1,494668682 | 0,023835466 | |
| 215576_at | - | -1,353583928 | -1,377419394 | 0,023835466 | |
| 215610_at | ALG1L2 | -1,026571149 | -1,050406615 | 0,023835466 | |
| 215873_x_at | ABCC10 | 1,689737652 | 1,665902186 | 0,023835466 | |

| | | | | | |
|---|---|---|---|---|---|
| 216010_x_at | FUT3 | -1,591296941 | -1,615132408 | 0,023835466 | |
| 216192_at | FABP7 | -1,838812296 | -1,862647763 | 0,023835466 | |
| 216230_x_at | SMPD1 | -1,308181863 | -1,332017329 | 0,023835466 | |
| 216418_at | - | -1,71423189 | -1,738067356 | 0,023835466 | |
| 216428_x_at | KIR3DX1 | -1,71423189 | -1,738067356 | 0,023835466 | |
| 216436_at | PIK3R4 | -1,308181863 | -1,332017329 | 0,023835466 | |
| 216489_at | TRPM3 | -1,308181863 | -1,332017329 | 0,023835466 | |
| 216564_at | - | -1,470833215 | -1,494668682 | 0,023835466 | |
| 216576_x_at | IGK@ /// IGKC | -1,639579028 | -1,663414495 | 0,023835466 | |
| 216763_at | - | -1,470833215 | -1,494668682 | 0,023835466 | |
| 216768_x_at | TTC38 | -1,131062212 | -1,154897679 | 0,023835466 | |
| 217049_x_at | PCDH11X /// PCI | -1,639579028 | -1,663414495 | 0,023835466 | |
| 217157_x_at | IGK@ /// IGKC | -1,71423189 | -1,738067356 | 0,023835466 | |
| 217464_at | - | -1,308181863 | -1,332017329 | 0,023835466 | |
| 217563_at | CLOCK | -1,71423189 | -1,738067356 | 0,023835466 | |
| 218417_s_at | SLC48A1 | 1,177702228 | 1,153866761 | 0,023835466 | |
| 218632_at | HECTD3 | 1,089278259 | 1,065442793 | 0,023835466 | |
| 219796_s_at | CDHR5 | -1,131062212 | -1,154897679 | 0,023835466 | |
| 219994_at | APBB1IP | -1,71423189 | -1,738067356 | 0,023835466 | |
| 220002_at | KIF26B | -1,591296941 | -1,615132408 | 0,023835466 | |
| 220012_at | ERO1LB | -1,591296941 | -1,615132408 | 0,023835466 | |
| 220049_s_at | PDCD1LG2 | -1,838812296 | -1,862647763 | 0,023835466 | |
| 221325_at | KCNK13 | -1,763254337 | -1,787089803 | 0,023835466 | |
| 221414_s_at | DEFB126 | -1,763254337 | -1,787089803 | 0,023835466 | |
| 221455_s_at | WNT3 | -1,71423189 | -1,738067356 | 0,023835466 | |
| 221962_s_at | UBE2H | 1,982112682 | 1,958277216 | 0,023835466 | |
| 222059_at | ZNF335 | -1,240169874 | -1,264005341 | 0,023835466 | |
| 222300_at | - | -1,71423189 | -1,738067356 | 0,023835466 | |
| 222454_s_at | PARVA | -1,639579028 | -1,663414495 | 0,023835466 | |
| 222813_at | ZNF668 | -1,71423189 | -1,738067356 | 0,023835466 | |
| 223045_at | EGLN1 | -1,763254337 | -1,787089803 | 0,023835466 | |
| 223121_s_at | SFRP2 | -1,639579028 | -1,663414495 | 0,023835466 | |
| 223453_s_at | ATL3 | -1,71423189 | -1,738067356 | 0,023835466 | |
| 223943_s_at | - | -1,026571149 | -1,050406615 | 0,023835466 | |
| 226271_at | GDAP1 | 1,215326747 | 1,191491281 | 0,023835466 | |
| 226498_at | FLT1 | -1,240169874 | -1,264005341 | 0,023835466 | |
| 226945_at | RHBDD1 | 1,58584542 | 1,562009954 | 0,023835466 | |
| 227076_at | NCAPH2 | 1,261018186 | 1,23718272 | 0,023835466 | |
| 227123_at | RAB3B | -1,353583928 | -1,377419394 | 0,023835466 | |
| 227571_at | - | -0,622910612 | -0,646746079 | 0,023835466 | |
| 227799_at | MYO1G | -1,131062212 | -1,154897679 | 0,023835466 | |
| 228136_s_at | C17orf70 | -0,706943342 | -0,730778808 | 0,023835466 | |
| 228215_at | LOC100505933 | -1,591296941 | -1,615132408 | 0,023835466 | |
| 228233_at | FREM1 | -1,591296941 | -1,615132408 | 0,023835466 | |
| 228345_at | CHIC1 | 0,747935635 | 0,724100169 | 0,023835466 | |
| 228603_at | ACTR3 | 1,940595304 | 1,916759838 | 0,023835466 | |
| 228784_at | ST3GAL2 | -1,131062212 | -1,154897679 | 0,023835466 | |
| 228878_s_at | CFC1B | -1,591296941 | -1,615132408 | 0,023835466 | |
| 229118_at | PRRG3 | -1,838812296 | -1,862647763 | 0,023835466 | |
| 229271_x_at | COL11A1 | -1,591296941 | -1,615132408 | 0,023835466 | |
| 229678_at | LOC728431 | -1,240169874 | -1,264005341 | 0,023835466 | |
| 229772_at | DEFB123 | -0,622910612 | -0,646746079 | 0,023835466 | |
| 230073_at | LOC100131564 | -1,131062212 | -1,154897679 | 0,023835466 | |
| 230432_at | LOC100422737 | -1,591296941 | -1,615132408 | 0,023835466 | |
| 230595_at | LOC100653336 / | -0,622910612 | -0,646746079 | 0,023835466 | |
| 230997_at | TTC21A | -1,131062212 | -1,154897679 | 0,023835466 | |

| | | | | | |
|---|---|---|---|---|---|
| 231013_at | - | -1,71423189 | -1,738067356 | 0,023835466 | |
| 231540_at | LOC100130691 | -1,639579028 | -1,663414495 | 0,023835466 | |
| 231742_at | CRX | -1,591296941 | -1,615132408 | 0,023835466 | |
| 231745_at | GPR55 | -1,131062212 | -1,154897679 | 0,023835466 | |
| 231994_at | CHDH | -1,353583928 | -1,377419394 | 0,023835466 | |
| 232158_x_at | NIPAL1 | -1,470833215 | -1,494668682 | 0,023835466 | |
| 232301_at | UBE3B | -1,71423189 | -1,738067356 | 0,023835466 | |
| 232408_at | ZFYVE28 | -0,706943342 | -0,730778808 | 0,023835466 | |
| 232434_at | DIRC3 | -1,353583928 | -1,377419394 | 0,023835466 | |
| 232576_at | - | -1,591296941 | -1,615132408 | 0,023835466 | |
| 232830_at | RNF32 | -1,639579028 | -1,663414495 | 0,023835466 | |
| 232845_at | CDH23 /// LOC10 | -0,706943342 | -0,730778808 | 0,023835466 | |
| 232894_at | - | -0,79540287 | -0,819238336 | 0,023835466 | |
| 232979_at | MIR10A | -1,763254337 | -1,787089803 | 0,023835466 | |
| 233043_at | LOC221814 | -1,763254337 | -1,787089803 | 0,023835466 | |
| 233067_at | PRDM11 | -1,591296941 | -1,615132408 | 0,023835466 | |
| 233072_at | NTNG2 | -1,353583928 | -1,377419394 | 0,023835466 | |
| 233179_x_at | SIRT6 | 1,58584542 | 1,562009954 | 0,023835466 | |
| 233229_at | SCFD1 | -1,591296941 | -1,615132408 | 0,023835466 | |
| 233393_at | - | -1,639579028 | -1,663414495 | 0,023835466 | |
| 233437_at | GABRA4 | -1,639579028 | -1,663414495 | 0,023835466 | |
| 233453_at | - | -1,71423189 | -1,738067356 | 0,023835466 | |
| 233593_at | - | -1,026571149 | -1,050406615 | 0,023835466 | |
| 233883_at | SNX24 | -1,308181863 | -1,332017329 | 0,023835466 | |
| 233909_at | - | -1,470833215 | -1,494668682 | 0,023835466 | |
| 233967_at | - | -1,353583928 | -1,377419394 | 0,023835466 | |
| 234010_at | - | -1,131062212 | -1,154897679 | 0,023835466 | |
| 234044_at | - | -0,79540287 | -0,819238336 | 0,023835466 | |
| 234574_at | - | -1,026571149 | -1,050406615 | 0,023835466 | |
| 234640_x_at | - | -1,591296941 | -1,615132408 | 0,023835466 | |
| 234869_at | - | -1,308181863 | -1,332017329 | 0,023835466 | |
| 234900_at | - | -1,838812296 | -1,862647763 | 0,023835466 | |
| 234910_at | RASAL2 | -0,706943342 | -0,730778808 | 0,023835466 | |
| 235053_at | - | -0,622910612 | -0,646746079 | 0,023835466 | |
| 235152_at | - | -1,639579028 | -1,663414495 | 0,023835466 | |
| 235195_at | FBXW2 | -1,639579028 | -1,663414495 | 0,023835466 | |
| 235255_at | ATP6V0A2 | 1,177702228 | 1,153866761 | 0,023835466 | |
| 235268_at | - | -1,838812296 | -1,862647763 | 0,023835466 | |
| 235364_at | TSC22D3 | -1,131062212 | -1,154897679 | 0,023835466 | |
| 235612_at | - | 1,549482847 | 1,525647381 | 0,023835466 | |
| 235639_at | CDH19 | -1,763254337 | -1,787089803 | 0,023835466 | |
| 235654_at | TMEM218 | -1,308181863 | -1,332017329 | 0,023835466 | |
| 235671_at | - | -1,308181863 | -1,332017329 | 0,023835466 | |
| 235842_at | - | -1,763254337 | -1,787089803 | 0,023835466 | |
| 235853_at | C3orf58 | -0,79540287 | -0,819238336 | 0,023835466 | |
| 235863_at | JSRP1 | -1,639579028 | -1,663414495 | 0,023835466 | |
| 235904_at | UGT3A1 | -1,763254337 | -1,787089803 | 0,023835466 | |
| 235982_at | FCRL1 | -1,838812296 | -1,862647763 | 0,023835466 | |
| 236086_at | - | -1,308181863 | -1,332017329 | 0,023835466 | |
| 236310_at | ZNF37BP | -1,353583928 | -1,377419394 | 0,023835466 | |
| 236579_at | NKX2-1-AS1 | -1,763254337 | -1,787089803 | 0,023835466 | |
| 236720_at | - | -1,838812296 | -1,862647763 | 0,023835466 | |
| 236790_s_at | - | -1,026571149 | -1,050406615 | 0,023835466 | |
| 236840_at | C12orf56 | -1,71423189 | -1,738067356 | 0,023835466 | |
| 237005_at | LOC442075 | 1,360921491 | 1,337086025 | 0,023835466 | |
| 237008_at | POU3F3 | -1,131062212 | -1,154897679 | 0,023835466 | |

| | | | | | |
|---|---|---|---|---|---|
| 237113_at | - | -1,763254337 | -1,787089803 | 0,023835466 | |
| 237191_x_at | - | -1,838812296 | -1,862647763 | 0,023835466 | |
| 237848_at | - | -1,838812296 | -1,862647763 | 0,023835466 | |
| 237915_at | AMPD3 /// LOC1( | -0,622910612 | -0,646746079 | 0,023835466 | |
| 237985_at | - | -1,71423189 | -1,738067356 | 0,023835466 | |
| 238210_at | - | -1,838812296 | -1,862647763 | 0,023835466 | |
| 238212_at | - | -1,470833215 | -1,494668682 | 0,023835466 | |
| 238244_at | LINC00535 | -1,763254337 | -1,787089803 | 0,023835466 | |
| 238293_at | - | -1,763254337 | -1,787089803 | 0,023835466 | |
| 238322_s_at | TEAD2 | -0,622910612 | -0,646746079 | 0,023835466 | |
| 238936_at | TMEM178B | -0,706943342 | -0,730778808 | 0,023835466 | |
| 238961_s_at | FNDC3A | -1,353583928 | -1,377419394 | 0,023835466 | |
| 239022_at | - | -0,622910612 | -0,646746079 | 0,023835466 | |
| 239030_at | - | -1,470833215 | -1,494668682 | 0,023835466 | |
| 239156_at | - | -1,131062212 | -1,154897679 | 0,023835466 | |
| 239192_at | PARD3B | -1,763254337 | -1,787089803 | 0,023835466 | |
| 239306_at | - | -1,470833215 | -1,494668682 | 0,023835466 | |
| 239656_at | LHFPL3-AS2 | -1,838812296 | -1,862647763 | 0,023835466 | |
| 239686_at | - | -1,026571149 | -1,050406615 | 0,023835466 | |
| 240033_at | PLG | -0,622910612 | -0,646746079 | 0,023835466 | |
| 240228_at | CSMD3 | -1,470833215 | -1,494668682 | 0,023835466 | |
| 240273_at | - | -0,622910612 | -0,646746079 | 0,023835466 | |
| 240346_at | - | -1,591296941 | -1,615132408 | 0,023835466 | |
| 240390_at | GALNT5 | 1,296557217 | 1,272721751 | 0,023835466 | |
| 240653_at | - | -1,639579028 | -1,663414495 | 0,023835466 | |
| 240661_at | LOC100506459 | -1,026571149 | -1,050406615 | 0,023835466 | |
| 240833_at | - | -1,353583928 | -1,377419394 | 0,023835466 | |
| 241013_at | FAM124A | -0,79540287 | -0,819238336 | 0,023835466 | |
| 241140_at | LMO7 | -1,639579028 | -1,663414495 | 0,023835466 | |
| 241174_at | AP4E1 | -1,026571149 | -1,050406615 | 0,023835466 | |
| 241308_at | - | -1,639579028 | -1,663414495 | 0,023835466 | |
| 241487_at | - | -1,639579028 | -1,663414495 | 0,023835466 | |
| 241584_at | - | -1,639579028 | -1,663414495 | 0,023835466 | |
| 241728_at | - | -1,763254337 | -1,787089803 | 0,023835466 | |
| 242678_at | - | -1,470833215 | -1,494668682 | 0,023835466 | |
| 242681_at | - | -1,838812296 | -1,862647763 | 0,023835466 | |
| 242700_at | PON2 | -1,240169874 | -1,264005341 | 0,023835466 | |
| 242785_at | EML6 | -1,838812296 | -1,862647763 | 0,023835466 | |
| 242828_at | FIGN | -1,639579028 | -1,663414495 | 0,023835466 | |
| 242836_at | - | -1,763254337 | -1,787089803 | 0,023835466 | |
| 242876_at | AKT3 | -1,353583928 | -1,377419394 | 0,023835466 | |
| 243091_at | - | -0,706943342 | -0,730778808 | 0,023835466 | |
| 243113_at | - | -1,240169874 | -1,264005341 | 0,023835466 | |
| 243123_at | - | -1,838812296 | -1,862647763 | 0,023835466 | |
| 243403_x_at | CPM | -1,639579028 | -1,663414495 | 0,023835466 | |
| 243787_at | - | -1,71423189 | -1,738067356 | 0,023835466 | |
| 244137_at | KIAA0317 | -1,353583928 | -1,377419394 | 0,023835466 | |
| 244152_at | - | -0,79540287 | -0,819238336 | 0,023835466 | |
| 244187_at | - | -0,79540287 | -0,819238336 | 0,023835466 | |
| 244355_at | AVL9 | -1,591296941 | -1,615132408 | 0,023835466 | |
| 244662_at | MBD5 | -1,131062212 | -1,154897679 | 0,023835466 | |
| 244683_at | - | -1,308181863 | -1,332017329 | 0,023835466 | |
| 1552367_a_at | SCIN | -2,667025278 | -2,690860744 | 0,023835466 | |
| 1552522_at | TIGD4 | -2,972500237 | -2,996335703 | 0,023835466 | |
| 1552732_at | ABRA | -3,212234735 | -3,236070201 | 0,023835466 | |
| 1553265_at | SLC23A3 | -2,667025278 | -2,690860744 | 0,023835466 | |

| | | | | | |
|---|---|---|---|---|---|
| 1553434_at | CYP4Z2P | -2,551294263 | -2,575129729 | 0,023835466 | |
| 1553757_at | IQCG | -2,138804305 | -2,162639771 | 0,023835466 | |
| 1554072_s_at | CCDC67 | -2,866296344 | -2,89013181 | 0,023835466 | |
| 1554789_a_at | PDE8B | -2,866296344 | -2,89013181 | 0,023835466 | |
| 1554920_at | SCEL | -3,250025915 | -3,273861381 | 0,023835466 | |
| 1554960_at | C1orf110 | -2,667025278 | -2,690860744 | 0,023835466 | |
| 1555346_at | CDC20B | -2,551294263 | -2,575129729 | 0,023835466 | |
| 1555489_at | - | -2,551294263 | -2,575129729 | 0,023835466 | |
| 1555513_at | PIAS2 | -3,250025915 | -3,273861381 | 0,023835466 | |
| 1555648_at | FOXP2 | -2,972500237 | -2,996335703 | 0,023835466 | |
| 1555749_at | SF1 | -2,551294263 | -2,575129729 | 0,023835466 | |
| 1555755_at | - | -2,667025278 | -2,690860744 | 0,023835466 | |
| 1555774_at | ZAR1 | -2,551294263 | -2,575129729 | 0,023835466 | |
| 1555876_at | SRGAP1 | -2,667025278 | -2,690860744 | 0,023835466 | |
| 1556081_at | - | -2,972500237 | -2,996335703 | 0,023835466 | |
| 1557057_a_at | C5orf47 | -3,212234735 | -3,236070201 | 0,023835466 | |
| 1557519_at | - | -2,551294263 | -2,575129729 | 0,023835466 | |
| 1558185_at | CLLU1 /// LOC10 | -3,212234735 | -3,236070201 | 0,023835466 | |
| 1558234_at | KCNJ2-AS1 | -3,212234735 | -3,236070201 | 0,023835466 | |
| 1558293_at | KIAA1107 | -2,138804305 | -2,162639771 | 0,023835466 | |
| 1558295_a_at | PPFIA2 | -2,138804305 | -2,162639771 | 0,023835466 | |
| 1558411_at | EGFEM1P | -2,972500237 | -2,996335703 | 0,023835466 | |
| 1558603_at | PLGLB2 | -2,667025278 | -2,690860744 | 0,023835466 | |
| 1559280_a_at | LOC100506013 | -2,866296344 | -2,89013181 | 0,023835466 | |
| 1559506_x_at | MIR137HG /// MI | -3,250025915 | -3,273861381 | 0,023835466 | |
| 1559510_at | LOC100287765 | -2,138804305 | -2,162639771 | 0,023835466 | |
| 1560108_at | - | -2,138804305 | -2,162639771 | 0,023835466 | |
| 1560161_at | CCNB2 | -2,551294263 | -2,575129729 | 0,023835466 | |
| 1560174_at | SPAG16 | -2,667025278 | -2,690860744 | 0,023835466 | |
| 1560359_at | PELO | -2,866296344 | -2,89013181 | 0,023835466 | |
| 1561198_at | LOC100506127 | -2,972500237 | -2,996335703 | 0,023835466 | |
| 1561615_s_at | SLC8A1 | -2,551294263 | -2,575129729 | 0,023835466 | |
| 1561765_at | - | -3,250025915 | -3,273861381 | 0,023835466 | |
| 1562076_at | - | -3,250025915 | -3,273861381 | 0,023835466 | |
| 1562839_at | LOC100272228 | -2,866296344 | -2,89013181 | 0,023835466 | |
| 1562991_at | ZNF292 | -3,250025915 | -3,273861381 | 0,023835466 | |
| 1563110_at | - | -2,972500237 | -2,996335703 | 0,023835466 | |
| 1563610_at | LOC157273 | -2,972500237 | -2,996335703 | 0,023835466 | |
| 1563927_a_at | LOC401463 | -2,667025278 | -2,690860744 | 0,023835466 | |
| 1564192_at | - | -2,972500237 | -2,996335703 | 0,023835466 | |
| 1564299_at | LOC100127940 | -2,866296344 | -2,89013181 | 0,023835466 | |
| 1564323_at | - | -2,551294263 | -2,575129729 | 0,023835466 | |
| 1564855_at | LOC727924 | -2,551294263 | -2,575129729 | 0,023835466 | |
| 1565937_a_at | LMO3 | -2,866296344 | -2,89013181 | 0,023835466 | |
| 1566096_x_at | - | -2,972500237 | -2,996335703 | 0,023835466 | |
| 1566888_at | THADA | -2,551294263 | -2,575129729 | 0,023835466 | |
| 1567303_at | - | -3,250025915 | -3,273861381 | 0,023835466 | |
| 1568736_s_at | - | -2,551294263 | -2,575129729 | 0,023835466 | |
| 1569716_at | - | -2,866296344 | -2,89013181 | 0,023835466 | |
| 1569960_at | BRD7P3 | -2,667025278 | -2,690860744 | 0,023835466 | |
| 1570160_at | - | -2,972500237 | -2,996335703 | 0,023835466 | |
| 1570594_at | - | -2,866296344 | -2,89013181 | 0,023835466 | |
| 1570632_at | - | -2,667025278 | -2,690860744 | 0,023835466 | |
| 203570_at | LOXL1 | -2,667025278 | -2,690860744 | 0,023835466 | |
| 204712_at | WIF1 | -2,667025278 | -2,690860744 | 0,023835466 | |
| 204985_s_at | TRAPPC6A | 3,109000457 | 3,08516499 | 0,023835466 | |

| | | | | | |
|---|---|---|---|---|---|
| 205496_at | KIAA0408 | -2,972500237 | -2,996335703 | 0,023835466 | |
| 206165_s_at | CLCA2 | -2,866296344 | -2,89013181 | 0,023835466 | |
| 207119_at | PRKG1 | -2,972500237 | -2,996335703 | 0,023835466 | |
| 207843_x_at | CYB5A | 1,614289682 | 1,590454215 | 0,023835466 | |
| 209537_at | EXTL2 | 2,834621043 | 2,810785576 | 0,023835466 | |
| 210277_at | AP4S1 | 2,705069849 | 2,681234383 | 0,023835466 | |
| 210525_x_at | EFCAB11 | -2,551294263 | -2,575129729 | 0,023835466 | |
| 212489_at | COL5A1 | -2,138804305 | -2,162639771 | 0,023835466 | |
| 214258_x_at | KAT5 | 2,44295215 | 2,419116683 | 0,023835466 | |
| 214702_at | FN1 | -2,866296344 | -2,89013181 | 0,023835466 | |
| 215324_at | SEMA3D | -2,866296344 | -2,89013181 | 0,023835466 | |
| 215361_at | DOCK5 | -2,667025278 | -2,690860744 | 0,023835466 | |
| 215597_x_at | - | -2,667025278 | -2,690860744 | 0,023835466 | |
| 215653_at | - | -3,212234735 | -3,236070201 | 0,023835466 | |
| 215793_at | MTMR7 | -2,667025278 | -2,690860744 | 0,023835466 | |
| 216015_s_at | NLRP3 | -2,138804305 | -2,162639771 | 0,023835466 | |
| 216167_at | LRRN2 | -2,667025278 | -2,690860744 | 0,023835466 | |
| 216169_at | - | -2,551294263 | -2,575129729 | 0,023835466 | |
| 216752_at | PIK3R4 | -2,972500237 | -2,996335703 | 0,023835466 | |
| 216865_at | COL14A1 | -2,551294263 | -2,575129729 | 0,023835466 | |
| 217245_at | EPAG | -2,138804305 | -2,162639771 | 0,023835466 | |
| 218860_at | NOC4L | 2,303618506 | 2,27978304 | 0,023835466 | |
| 219091_s_at | MMRN2 | -2,138804305 | -2,162639771 | 0,023835466 | |
| 219995_s_at | ZNF750 | -3,32387526 | -3,347710726 | 0,023835466 | |
| 220247_at | CASC5 | -2,972500237 | -2,996335703 | 0,023835466 | |
| 220518_at | ABI3BP | -2,972500237 | -2,996335703 | 0,023835466 | |
| 220564_at | RNLS | -2,551294263 | -2,575129729 | 0,023835466 | |
| 222025_s_at | OPLAH | -2,138804305 | -2,162639771 | 0,023835466 | |
| 222999_s_at | CCNL2 | 3,620156261 | 3,596320795 | 0,023835466 | |
| 223843_at | SCARA3 | -2,866296344 | -2,89013181 | 0,023835466 | |
| 224127_at | - | -2,667025278 | -2,690860744 | 0,023835466 | |
| 224403_at | FCRL4 | -2,667025278 | -2,690860744 | 0,023835466 | |
| 224520_s_at | BEST3 | -2,551294263 | -2,575129729 | 0,023835466 | |
| 224925_at | PREX1 | -2,138804305 | -2,162639771 | 0,023835466 | |
| 226225_at | MCC | -2,138804305 | -2,162639771 | 0,023835466 | |
| 226938_at | DCAF4 | 3,308224834 | 3,284389368 | 0,023835466 | |
| 228051_at | KIAA1244 | -2,866296344 | -2,89013181 | 0,023835466 | |
| 228063_s_at | NAP1L5 | 2,146943689 | 2,123108223 | 0,023835466 | |
| 228503_at | RPS6KA6 | -2,551294263 | -2,575129729 | 0,023835466 | |
| 229266_at | SHISA6 | -2,667025278 | -2,690860744 | 0,023835466 | |
| 229779_at | COL4A4 | -2,866296344 | -2,89013181 | 0,023835466 | |
| 230225_x_at | - | -3,212234735 | -3,236070201 | 0,023835466 | |
| 230718_at | HSF5 | -2,667025278 | -2,690860744 | 0,023835466 | |
| 231773_at | ANGPTL1 | -2,972500237 | -2,996335703 | 0,023835466 | |
| 232603_at | DCDC5 | -3,212234735 | -3,236070201 | 0,023835466 | |
| 232818_at | - | -2,551294263 | -2,575129729 | 0,023835466 | |
| 233273_at | - | -2,972500237 | -2,996335703 | 0,023835466 | |
| 233680_at | - | -2,667025278 | -2,690860744 | 0,023835466 | |
| 233856_at | - | -2,667025278 | -2,690860744 | 0,023835466 | |
| 233884_at | HIVEP3 | -2,866296344 | -2,89013181 | 0,023835466 | |
| 234064_at | - | -2,667025278 | -2,690860744 | 0,023835466 | |
| 234473_at | - | -2,667025278 | -2,690860744 | 0,023835466 | |
| 234678_at | KRTAP4-3 | -2,551294263 | -2,575129729 | 0,023835466 | |
| 234861_at | LOC93463 | -2,972500237 | -2,996335703 | 0,023835466 | |
| 235247_at | - | -2,866296344 | -2,89013181 | 0,023835466 | |
| 235971_at | - | -2,138804305 | -2,162639771 | 0,023835466 | |

| | | | | | |
|---|---|---|---|---|---|
| 236984_at | C4orf26 | -2,551294263 | -2,575129729 | 0,023835466 | |
| 237332_at | - | -2,138804305 | -2,162639771 | 0,023835466 | |
| 237496_at | - | -2,972500237 | -2,996335703 | 0,023835466 | |
| 238079_at | TPM3 | -2,866296344 | -2,89013181 | 0,023835466 | |
| 238626_at | ANKS6 | -2,551294263 | -2,575129729 | 0,023835466 | |
| 238680_at | METTL12 /// SNC | -2,138804305 | -2,162639771 | 0,023835466 | |
| 238751_at | - | -2,667025278 | -2,690860744 | 0,023835466 | |
| 238858_at | TIFA | -2,551294263 | -2,575129729 | 0,023835466 | |
| 239650_at | NCKAP5 | -3,250025915 | -3,273861381 | 0,023835466 | |
| 239827_at | RGCC | -2,138804305 | -2,162639771 | 0,023835466 | |
| 239830_at | - | -2,551294263 | -2,575129729 | 0,023835466 | |
| 239890_s_at | SERP2 | -2,667025278 | -2,690860744 | 0,023835466 | |
| 239962_at | - | -3,212234735 | -3,236070201 | 0,023835466 | |
| 240132_at | - | -2,667025278 | -2,690860744 | 0,023835466 | |
| 240356_s_at | - | -2,972500237 | -2,996335703 | 0,023835466 | |
| 241384_x_at | GK5 | -2,138804305 | -2,162639771 | 0,023835466 | |
| 241390_at | - | -3,212234735 | -3,236070201 | 0,023835466 | |
| 241507_x_at | - | -2,972500237 | -2,996335703 | 0,023835466 | |
| 241554_at | - | -2,667025278 | -2,690860744 | 0,023835466 | |
| 241587_at | NAALADL2-AS3 | -3,212234735 | -3,236070201 | 0,023835466 | |
| 242144_at | - | -2,138804305 | -2,162639771 | 0,023835466 | |
| 242299_at | - | -2,551294263 | -2,575129729 | 0,023835466 | |
| 242488_at | CHRM3 | -2,866296344 | -2,89013181 | 0,023835466 | |
| 242706_s_at | MED23 | 2,44295215 | 2,419116683 | 0,023835466 | |
| 242849_at | - | -2,667025278 | -2,690860744 | 0,023835466 | |
| 242948_x_at | - | -2,866296344 | -2,89013181 | 0,023835466 | |
| 243533_x_at | - | -2,138804305 | -2,162639771 | 0,023835466 | |
| 243535_at | - | -2,138804305 | -2,162639771 | 0,023835466 | |
| 243813_at | LOC100507632 | -2,667025278 | -2,690860744 | 0,023835466 | |
| 243940_at | TSHZ2 | -3,212234735 | -3,236070201 | 0,023835466 | |
| 244608_at | - | -2,551294263 | -2,575129729 | 0,023835466 | |
| 244874_at | CHST15 | -2,551294263 | -2,575129729 | 0,023835466 | |
| 205458_at | MC1R | 0,083054335 | 0,059218869 | 0,023835466 | |
| 210123_s_at | CHRFAM7A /// C | 0,083054335 | 0,059218869 | 0,023835466 | |
| 220612_at | - | 0,083054335 | 0,059218869 | 0,023835466 | |
| 227119_at | CNOT6L | 0,083054335 | 0,059218869 | 0,023835466 | |
| 237016_at | TMEM217 | 0,083054335 | 0,059218869 | 0,023835466 | |
| 206617_s_at | RENBP | -0,022466681 | -0,046302147 | 0,023835466 | |
| 226250_at | - | -0,022466681 | -0,046302147 | 0,023835466 | |
| 228903_at | CES4A | -0,022466681 | -0,046302147 | 0,023835466 | |
| 225535_s_at | TIMM23 | 5,186133489 | 5,162298023 | 0,023835466 | |
| 224144_at | SPTBN4 | 0,03125738 | 0,007421914 | 0,023835466 | |
| 225929_s_at | RNF213 | 0,03125738 | 0,007421914 | 0,023835466 | |
| 231004_s_at | H1FX | 0,03125738 | 0,007421914 | 0,023835466 | |
| 231065_at | PDE6D | 0,03125738 | 0,007421914 | 0,023835466 | |
| 204740_at | CNKSR1 | -0,055687482 | -0,079522948 | 0,023835466 | |
| 204864_s_at | IL6ST | -0,055687482 | -0,079522948 | 0,023835466 | |
| 209598_at | PNMA2 | -0,055687482 | -0,079522948 | 0,023835466 | |
| 216530_at | - | -0,055687482 | -0,079522948 | 0,023835466 | |
| 232890_at | - | -0,055687482 | -0,079522948 | 0,023835466 | |
| 236598_at | - | -0,055687482 | -0,079522948 | 0,023835466 | |
| 243528_at | - | -0,055687482 | -0,079522948 | 0,023835466 | |
| 207528_s_at | SLC7A11 | -0,00073712 | -0,024572586 | 0,023835466 | |
| 210994_x_at | TRIM23 | 0,052199481 | 0,028364014 | 0,023835466 | |
| 219542_at | NEK11 | -0,00073712 | -0,024572586 | 0,023835466 | |
| 221161_at | ASCL3 | 0,052199481 | 0,028364014 | 0,023835466 | |

| | | | | | |
|---|---|---|---|---|---|
| 224794_s_at | CERCAM /// LOC | -0,00073712 | -0,024572586 | 0,023835466 | |
| 227371_at | BAIAP2L1 | 0,052199481 | 0,028364014 | 0,023835466 | |
| 235966_at | DAB2IP | 0,052199481 | 0,028364014 | 0,023835466 | |
| 236421_at | ANKRD45 | 0,052199481 | 0,028364014 | 0,023835466 | |
| 241095_at | - | -0,00073712 | -0,024572586 | 0,023835466 | |
| 1552315_at | GIMAP1 | -0,83191556 | -0,855751026 | 0,023835466 | |
| 1552792_at | SOCS4 | -0,59053611 | -0,614371577 | 0,023835466 | |
| 1552964_at | TTC40 | -0,59053611 | -0,614371577 | 0,023835466 | |
| 1553673_at | STK35 | -0,888496123 | -0,912331589 | 0,023835466 | |
| 1554322_a_at | HDAC4 | -0,926850548 | -0,950686014 | 0,023835466 | |
| 1554588_a_at | TTC30B | -0,512251684 | -0,536087151 | 0,023835466 | |
| 1554592_a_at | SLC1A6 | -0,394749123 | -0,41858459 | 0,023835466 | |
| 1554773_at | WFIKKN2 | -0,59053611 | -0,614371577 | 0,023835466 | |
| 1554907_a_at | HYDIN /// HYDIN | -0,366776884 | -0,39061235 | 0,023835466 | |
| 1555979_at | LOC100130417 | -0,926850548 | -0,950686014 | 0,023835466 | |
| 1557147_a_at | - | -0,467091869 | -0,490927335 | 0,023835466 | |
| 1557595_at | GINS2 | -0,366776884 | -0,39061235 | 0,023835466 | |
| 1559796_at | EBF3 | -0,655928373 | -0,679763839 | 0,023835466 | |
| 1560636_a_at | HSPC081 | -0,888496123 | -0,912331589 | 0,023835466 | |
| 1561039_a_at | ZNF81 | 0,480753764 | 0,456918297 | 0,023835466 | |
| 1562272_at | - | -0,59053611 | -0,614371577 | 0,023835466 | |
| 1562697_at | LOC339988 | 0,541261317 | 0,51742585 | 0,023835466 | |
| 1564482_at | ATP5O | -0,83191556 | -0,855751026 | 0,023835466 | |
| 1564823_at | CDC42BPG | -0,543051993 | -0,566887459 | 0,023835466 | |
| 1566183_at | - | -0,926850548 | -0,950686014 | 0,023835466 | |
| 1568675_at | CHRNA10 | -0,366776884 | -0,39061235 | 0,023835466 | |
| 1568913_at | NSUN3 | -0,655928373 | -0,679763839 | 0,023835466 | |
| 1569009_s_at | FAM201B /// LOC | -0,83191556 | -0,855751026 | 0,023835466 | |
| 1569776_at | - | -0,543051993 | -0,566887459 | 0,023835466 | |
| 1570336_at | BDH1 | -0,741668563 | -0,765504029 | 0,023835466 | |
| 201579_at | FAT1 | -0,83191556 | -0,855751026 | 0,023835466 | |
| 203129_s_at | KIF5C | -0,741668563 | -0,765504029 | 0,023835466 | |
| 203579_s_at | SLC7A6 | 0,401366113 | 0,377530647 | 0,023835466 | |
| 203809_s_at | AKT2 | 0,695652136 | 0,67181667 | 0,023835466 | |
| 204705_x_at | ALDOB | -0,59053611 | -0,614371577 | 0,023835466 | |
| 204912_at | IL10RA | -0,467091869 | -0,490927335 | 0,023835466 | |
| 205040_at | ORM1 | -0,888496123 | -0,912331589 | 0,023835466 | |
| 205835_s_at | YTHDC2 | -0,655928373 | -0,679763839 | 0,023835466 | |
| 206038_s_at | NR2C2 | -0,926850548 | -0,950686014 | 0,023835466 | |
| 206274_s_at | CROCC | -0,888496123 | -0,912331589 | 0,023835466 | |
| 206533_at | CHRNA5 | -0,512251684 | -0,536087151 | 0,023835466 | |
| 206694_at | PNLIPRP1 | -0,467091869 | -0,490927335 | 0,023835466 | |
| 207051_at | SLC17A4 | -0,655928373 | -0,679763839 | 0,023835466 | |
| 208033_s_at | ZFHX3 | -0,655928373 | -0,679763839 | 0,023835466 | |
| 208106_x_at | PSG6 | -0,366776884 | -0,39061235 | 0,023835466 | |
| 208285_at | OR7A5 | -0,888496123 | -0,912331589 | 0,023835466 | |
| 208365_s_at | GRK4 | -0,83191556 | -0,855751026 | 0,023835466 | |
| 208560_at | KCNA10 | -0,888496123 | -0,912331589 | 0,023835466 | |
| 208590_x_at | GJA3 | -0,741668563 | -0,765504029 | 0,023835466 | |
| 209052_s_at | WHSC1 | 0,810733188 | 0,786897721 | 0,023835466 | |
| 210364_at | SCN2B | -0,543051993 | -0,566887459 | 0,023835466 | |
| 210599_at | ZNF614 | -0,394749123 | -0,41858459 | 0,023835466 | |
| 211143_x_at | NR4A1 | 0,401366113 | 0,377530647 | 0,023835466 | |
| 213016_at | BBX | 0,859074518 | 0,835239052 | 0,023835466 | |
| 214286_at | GNAT1 | -0,83191556 | -0,855751026 | 0,023835466 | |
| 214360_at | SMARCA4 | -0,83191556 | -0,855751026 | 0,023835466 | |

| | | | | | |
|---|---|---|---|---|---|
| 214740_at | POLR2J2 /// POL | -0,543051993 | -0,566887459 | 0,023835466 | |
| 214825_at | FAM155A | 0,541261317 | 0,51742585 | 0,023835466 | |
| 214972_at | MGEA5 | -0,394749123 | -0,41858459 | 0,023835466 | |
| 215131_at | IQCK | -0,543051993 | -0,566887459 | 0,023835466 | |
| 215414_at | FARS2 | -0,83191556 | -0,855751026 | 0,023835466 | |
| 215459_at | CTNS | -0,83191556 | -0,855751026 | 0,023835466 | |
| 216014_s_at | ZXDA /// ZXDB | -0,59053611 | -0,614371577 | 0,023835466 | |
| 216684_s_at | SS18 | 0,888485433 | 0,864649967 | 0,023835466 | |
| 216915_s_at | PTPN12 | 0,695652136 | 0,67181667 | 0,023835466 | |
| 218029_at | FAM65A | 0,900083947 | 0,876248481 | 0,023835466 | |
| 219020_at | HS1BP3 | 0,661985843 | 0,638150376 | 0,023835466 | |
| 219501_at | ENOX1 | -0,394749123 | -0,41858459 | 0,023835466 | |
| 219760_at | LIN7B | 0,480753764 | 0,456918297 | 0,023835466 | |
| 219838_at | TTC23 | 0,917308734 | 0,893473268 | 0,023835466 | |
| 220567_at | IKZF2 | -0,83191556 | -0,855751026 | 0,023835466 | |
| 220770_s_at | C5orf54 | 0,376672223 | 0,352836757 | 0,023835466 | |
| 220987_s_at | AKIP1 /// NUAK2 | -0,741668563 | -0,765504029 | 0,023835466 | |
| 221128_at | ADAM19 | -0,83191556 | -0,855751026 | 0,023835466 | |
| 221348_at | NPPC | -0,83191556 | -0,855751026 | 0,023835466 | |
| 221415_s_at | GJA9 /// GJA9-M | -0,259832361 | -0,283667828 | 0,023835466 | |
| 221769_at | SPSB3 | 0,661985843 | 0,638150376 | 0,023835466 | |
| 221942_s_at | GUCY1A3 | -0,888496123 | -0,912331589 | 0,023835466 | |
| 222928_s_at | FAM160B2 | -0,512251684 | -0,536087151 | 0,023835466 | |
| 223530_at | TDRKH | 0,728550685 | 0,704715219 | 0,023835466 | |
| 223595_at | TMEM133 | -0,467091869 | -0,490927335 | 0,023835466 | |
| 223621_at | PNMA3 | 0,928678725 | 0,904843258 | 0,023835466 | |
| 223787_s_at | C3orf19 | 0,917308734 | 0,893473268 | 0,023835466 | |
| 223920_s_at | TP53AIP1 | -0,512251684 | -0,536087151 | 0,023835466 | |
| 224490_s_at | KANSL1 | 0,592200562 | 0,568365095 | 0,023835466 | |
| 224580_at | SLC38A1 | -0,467091869 | -0,490927335 | 0,023835466 | |
| 226328_at | KLF16 | 0,888485433 | 0,864649967 | 0,023835466 | |
| 226429_at | KIAA1704 | 0,592200562 | 0,568365095 | 0,023835466 | |
| 227057_at | ARHGAP27 | -0,926850548 | -0,950686014 | 0,023835466 | |
| 227355_at | RBM26 | -0,59053611 | -0,614371577 | 0,023835466 | |
| 227401_at | IL17D | 0,480753764 | 0,456918297 | 0,023835466 | |
| 227720_at | ANKRD13B | -0,512251684 | -0,536087151 | 0,023835466 | |
| 227879_at | ALKBH7 | -0,83191556 | -0,855751026 | 0,023835466 | |
| 227914_s_at | UBE3B | -0,655928373 | -0,679763839 | 0,023835466 | |
| 228117_at | LOC100132273 | -0,543051993 | -0,566887459 | 0,023835466 | |
| 229136_s_at | - | 0,480753764 | 0,456918297 | 0,023835466 | |
| 229415_at | CYCS | 0,810733188 | 0,786897721 | 0,023835466 | |
| 229585_at | ADAMTSL1 | -0,926850548 | -0,950686014 | 0,023835466 | |
| 229940_at | SETD3 | -0,543051993 | -0,566887459 | 0,023835466 | |
| 230079_at | ST7L | 0,695652136 | 0,67181667 | 0,023835466 | |
| 230974_at | DDX19B | 0,401366113 | 0,377530647 | 0,023835466 | |
| 232283_at | LYSMD1 | -0,512251684 | -0,536087151 | 0,023835466 | |
| 232358_at | KIAA1328 | -0,543051993 | -0,566887459 | 0,023835466 | |
| 232433_at | KIAA1683 | -0,259832361 | -0,283667828 | 0,023835466 | |
| 232532_at | QRICH2 | -0,888496123 | -0,912331589 | 0,023835466 | |
| 232921_at | KIAA1549 | -0,741668563 | -0,765504029 | 0,023835466 | |
| 233145_at | CSMD2 | -0,741668563 | -0,765504029 | 0,023835466 | |
| 233224_at | - | -0,59053611 | -0,614371577 | 0,023835466 | |
| 233258_at | - | -0,259832361 | -0,283667828 | 0,023835466 | |
| 233787_at | C6orf163 | -0,741668563 | -0,765504029 | 0,023835466 | |
| 235412_at | ARHGEF7 | -0,543051993 | -0,566887459 | 0,023835466 | |
| 235415_at | RPRD2 | -0,83191556 | -0,855751026 | 0,023835466 | |

| | | | | | |
|---|---|---|---|---|---|
| 235706_at | CPM | -0,512251684 | -0,536087151 | 0,023835466 | |
| 235738_at | - | -0,888496123 | -0,912331589 | 0,023835466 | |
| 235889_at | - | 0,888485433 | 0,864649967 | 0,023835466 | |
| 236147_at | - | -0,741668563 | -0,765504029 | 0,023835466 | |
| 236498_s_at | C1orf86 | -0,83191556 | -0,855751026 | 0,023835466 | |
| 236585_at | - | -0,512251684 | -0,536087151 | 0,023835466 | |
| 236708_at | - | -0,655928373 | -0,679763839 | 0,023835466 | |
| 236916_at | - | -0,926850548 | -0,950686014 | 0,023835466 | |
| 237495_at | MPP7 | -0,83191556 | -0,855751026 | 0,023835466 | |
| 237600_at | - | -0,888496123 | -0,912331589 | 0,023835466 | |
| 237666_at | XRRA1 | -0,888496123 | -0,912331589 | 0,023835466 | |
| 237887_at | - | 0,810733188 | 0,786897721 | 0,023835466 | |
| 237899_at | LOC100506374 | -0,512251684 | -0,536087151 | 0,023835466 | |
| 238053_at | DHRSX | -0,59053611 | -0,614371577 | 0,023835466 | |
| 238486_at | FRS2 | -0,259832361 | -0,283667828 | 0,023835466 | |
| 238953_at | LOC100506325 | -0,366776884 | -0,39061235 | 0,023835466 | |
| 239342_at | DGKZ | -0,741668563 | -0,765504029 | 0,023835466 | |
| 239347_at | - | -0,926850548 | -0,950686014 | 0,023835466 | |
| 239833_at | - | -0,59053611 | -0,614371577 | 0,023835466 | |
| 240368_at | - | -0,83191556 | -0,855751026 | 0,023835466 | |
| 240528_s_at | EXOC4 | 0,859074518 | 0,835239052 | 0,023835466 | |
| 241241_at | - | -0,543051993 | -0,566887459 | 0,023835466 | |
| 242114_at | - | -0,83191556 | -0,855751026 | 0,023835466 | |
| 242201_at | PMS2P5 | 0,917308734 | 0,893473268 | 0,023835466 | |
| 242394_at | CNKSR3 | -0,926850548 | -0,950686014 | 0,023835466 | |
| 242447_at | C3orf70 | -0,512251684 | -0,536087151 | 0,023835466 | |
| 242561_at | IPO9 | -0,655928373 | -0,679763839 | 0,023835466 | |
| 242645_at | - | -0,926850548 | -0,950686014 | 0,023835466 | |
| 242808_at | - | -0,512251684 | -0,536087151 | 0,023835466 | |
| 242983_at | - | -0,83191556 | -0,855751026 | 0,023835466 | |
| 243349_at | KIAA1324 | -0,888496123 | -0,912331589 | 0,023835466 | |
| 243439_at | ZNF418 | -0,259832361 | -0,283667828 | 0,023835466 | |
| 243589_at | KANSL1 | 0,87091093 | 0,847075464 | 0,023835466 | |
| 243595_at | SHB | -0,543051993 | -0,566887459 | 0,023835466 | |
| 244121_at | UBR3 | -0,926850548 | -0,950686014 | 0,023835466 | |
| 244215_at | - | -0,888496123 | -0,912331589 | 0,023835466 | |
| 244788_at | - | -0,543051993 | -0,566887459 | 0,023835466 | |
| 1554001_at | TRIM37 | -0,112808516 | -0,136643983 | 0,023835466 | |
| 1557892_at | LOC100144597 | -0,112808516 | -0,136643983 | 0,023835466 | |
| 1560396_at | KLHL6 | -0,112808516 | -0,136643983 | 0,023835466 | |
| 1560689_s_at | AKT2 | -0,112808516 | -0,136643983 | 0,023835466 | |
| 210932_s_at | RNF6 | -0,112808516 | -0,136643983 | 0,023835466 | |
| 213837_at | L3MBTL1 | -0,112808516 | -0,136643983 | 0,023835466 | |
| 241447_at | ING5 | 0,133056859 | 0,109221392 | 0,023835466 | |
| 1554074_s_at | SLFNL1 | 0,152582607 | 0,128747141 | 0,023835466 | |
| 1554919_s_at | C7orf63 | 0,20027154 | 0,176436073 | 0,023835466 | |
| 1560652_at | - | 0,181384709 | 0,157549243 | 0,023835466 | |
| 1561908_a_at | HS3ST3B1 | 0,20027154 | 0,176436073 | 0,023835466 | |
| 1562810_at | - | -0,078265071 | -0,102100538 | 0,023835466 | |
| 1569519_at | LOC100506032 / | -0,172274514 | -0,19610998 | 0,023835466 | |
| 201668_x_at | MARCKS | 0,152582607 | 0,128747141 | 0,023835466 | |
| 202456_s_at | ZER1 | 0,20027154 | 0,176436073 | 0,023835466 | |
| 203365_s_at | MMP15 | 0,181384709 | 0,157549243 | 0,023835466 | |
| 204356_at | LIMK1 | -0,078265071 | -0,102100538 | 0,023835466 | |
| 204862_s_at | NME3 | 0,246434561 | 0,222599095 | 0,023835466 | |
| 204944_at | PTPRG | 0,228146222 | 0,204310755 | 0,023835466 | |

| | | | | | |
|---|---|---|---|---|---|
| 206748_s_at | SPAG9 | 0,181384709 | 0,157549243 | 0,023835466 | |
| 206857_s_at | FKBP1B | 0,20027154 | 0,176436073 | 0,023835466 | |
| 207473_at | MLN | -0,172274514 | -0,19610998 | 0,023835466 | |
| 210360_s_at | MTSS1 | 0,181384709 | 0,157549243 | 0,023835466 | |
| 210431_at | ALPPL2 | -0,078265071 | -0,102100538 | 0,023835466 | |
| 210750_s_at | DLGAP1 | -0,172274514 | -0,19610998 | 0,023835466 | |
| 214616_at | HIST1H3A /// HIS | 0,152582607 | 0,128747141 | 0,023835466 | |
| 214980_at | UBE3A | 0,152582607 | 0,128747141 | 0,023835466 | |
| 216219_at | AQP6 | 0,152582607 | 0,128747141 | 0,023835466 | |
| 216425_at | - | -0,078265071 | -0,102100538 | 0,023835466 | |
| 221387_at | NPFFR1 | 0,152582607 | 0,128747141 | 0,023835466 | |
| 224203_at | SUFU | 0,228146222 | 0,204310755 | 0,023835466 | |
| 224543_at | SVEP1 | 0,246434561 | 0,222599095 | 0,023835466 | |
| 225389_at | BTBD6 | -0,078265071 | -0,102100538 | 0,023835466 | |
| 227438_at | ALPK1 | -0,078265071 | -0,102100538 | 0,023835466 | |
| 228556_at | YTHDC1 | 0,246434561 | 0,222599095 | 0,023835466 | |
| 230689_at | - | -0,172274514 | -0,19610998 | 0,023835466 | |
| 231884_at | CNTROB | -0,172274514 | -0,19610998 | 0,023835466 | |
| 232044_at | RBBP6 | 0,228146222 | 0,204310755 | 0,023835466 | |
| 232467_at | KIRREL | -0,172274514 | -0,19610998 | 0,023835466 | |
| 233743_x_at | S1PR5 | 0,246434561 | 0,222599095 | 0,023835466 | |
| 238089_at | MAN2C1 | -0,078265071 | -0,102100538 | 0,023835466 | |
| 241756_at | - | 0,152582607 | 0,128747141 | 0,023835466 | |
| 244580_at | - | -0,172274514 | -0,19610998 | 0,023835466 | |
| 1559218_s_at | NFYC | 0,359971644 | 0,336136178 | 0,023835466 | |
| 1563273_at | - | 0,273439642 | 0,249604176 | 0,023835466 | |
| 1570238_at | ZNF527 | 0,273439642 | 0,249604176 | 0,023835466 | |
| 205855_at | ZNF197 | 0,359971644 | 0,336136178 | 0,023835466 | |
| 206234_s_at | MMP17 | 0,359971644 | 0,336136178 | 0,023835466 | |
| 212820_at | DMXL2 | 0,291166226 | 0,26733076 | 0,023835466 | |
| 214050_at | - | -0,43775896 | -0,461594427 | 0,023835466 | |
| 215743_at | NMT2 | 0,457392764 | 0,433557298 | 0,023835466 | |
| 219422_at | ESPN | 0,317354302 | 0,293518836 | 0,023835466 | |
| 220454_s_at | SEMA6A | -0,43775896 | -0,461594427 | 0,023835466 | |
| 222008_at | COL9A1 | -0,43775896 | -0,461594427 | 0,023835466 | |
| 223103_at | STARD10 | 0,291166226 | 0,26733076 | 0,023835466 | |
| 236180_at | - | 0,273439642 | 0,249604176 | 0,023835466 | |
| 241944_x_at | - | -0,43775896 | -0,461594427 | 0,023835466 | |
| 244082_at | - | -0,43775896 | -0,461594427 | 0,023835466 | |
| 1569250_at | ZNF333 | -0,986356909 | -1,010192375 | 0,023835466 | |
| 202229_s_at | CHERP | 0,627515113 | 0,603679647 | 0,023835466 | |
| 205513_at | TCN1 | -0,986356909 | -1,010192375 | 0,023835466 | |
| 208575_at | HIST1H3A /// HIS | -0,986356909 | -1,010192375 | 0,023835466 | |
| 220280_s_at | ANKMY1 | -0,986356909 | -1,010192375 | 0,023835466 | |
| 222594_s_at | SPATS2 | 0,792179257 | 0,768343791 | 0,023835466 | |
| 227845_s_at | SHD | -0,986356909 | -1,010192375 | 0,023835466 | |
| 228124_at | ABHD12 | 0,792179257 | 0,768343791 | 0,023835466 | |
| 232730_at | C19orf44 | 0,648296226 | 0,624460759 | 0,023835466 | |
| 233256_at | - | -0,986356909 | -1,010192375 | 0,023835466 | |
| 234002_at | ABHD16B | -0,986356909 | -1,010192375 | 0,023835466 | |
| 240298_at | LOC147791 | -0,986356909 | -1,010192375 | 0,023835466 | |
| 241301_at | - | -0,986356909 | -1,010192375 | 0,023835466 | |
| 241948_at | - | -0,986356909 | -1,010192375 | 0,023835466 | |
| 201910_at | FARP1 | 0,103263601 | 0,079428135 | 0,023835466 | |
| 236867_at | - | 0,103263601 | 0,079428135 | 0,023835466 | |
| 215170_s_at | CEP152 | 1,000476627 | 0,976641161 | 0,023835466 | |

| | | | | | |
|---|---|---|---|---|---|
| 238903_at | UBXN2B | 1,011212064 | 0,987376598 | 0,023835466 | |
| 1556917_a_at | - | -2,479842156 | -2,503677622 | 0,023835466 | |
| 1557511_at | - | -2,479842156 | -2,503677622 | 0,023835466 | |
| 1558815_at | SORBS2 | -2,479842156 | -2,503677622 | 0,023835466 | |
| 1562481_at | - | -2,479842156 | -2,503677622 | 0,023835466 | |
| 1562895_at | - | -2,479842156 | -2,503677622 | 0,023835466 | |
| 1564533_at | LOC643711 | -2,479842156 | -2,503677622 | 0,023835466 | |
| 207173_x_at | CDH11 | -2,479842156 | -2,503677622 | 0,023835466 | |
| 210039_s_at | PRKCQ | 2,213529682 | 2,189694216 | 0,023835466 | |
| 213652_at | PCSK5 | -2,479842156 | -2,503677622 | 0,023835466 | |
| 215405_at | - | -2,479842156 | -2,503677622 | 0,023835466 | |
| 216992_s_at | GRM8 | -2,479842156 | -2,503677622 | 0,023835466 | |
| 217123_x_at | PMCHL1 | -2,479842156 | -2,503677622 | 0,023835466 | |
| 220539_at | TTC40 | -2,479842156 | -2,503677622 | 0,023835466 | |
| 224532_at | USP26 | -2,479842156 | -2,503677622 | 0,023835466 | |
| 226955_at | AFAP1L1 | -2,479842156 | -2,503677622 | 0,023835466 | |
| 231084_at | WDR96 | -2,479842156 | -2,503677622 | 0,023835466 | |
| 231315_at | NKX2-1 | -2,479842156 | -2,503677622 | 0,023835466 | |
| 232060_at | ROR1 | -2,479842156 | -2,503677622 | 0,023835466 | |
| 233668_at | - | -2,479842156 | -2,503677622 | 0,023835466 | |
| 237631_at | - | -2,479842156 | -2,503677622 | 0,023835466 | |
| 237951_at | - | -2,479842156 | -2,503677622 | 0,023835466 | |
| 238134_at | - | -2,479842156 | -2,503677622 | 0,023835466 | |
| 240018_at | - | -2,479842156 | -2,503677622 | 0,023835466 | |
| 241962_at | - | -2,479842156 | -2,503677622 | 0,023835466 | |
| 242430_at | - | -2,479842156 | -2,503677622 | 0,023835466 | |
| 215156_at | WDR61 | 1,224581536 | 1,200746069 | 0,023835466 | |
| 238549_at | CBFA2T2 | 1,767779954 | 1,743944487 | 0,023835466 | |
| 204087_s_at | SLC5A6 | 3,63931383 | 3,615478364 | 0,023835466 | |
| 220949_s_at | C7orf49 /// LOC6 | 3,621908408 | 3,598072942 | 0,023835466 | |
| 225666_at | TMTC4 | 1,318332599 | 1,294497133 | 0,023835466 | |
| 226951_at | C2orf49 | 2,201862497 | 2,17802703 | 0,023835466 | |
| 238511_at | LOC440288 | 1,318332599 | 1,294497133 | 0,023835466 | |
| 243366_s_at | - | 3,225364068 | 3,201528602 | 0,023835466 | |
| 50221_at | TFEB | -0,083957829 | -0,107775957 | 0,023818128 | |
| 244740_at | - | 4,592823355 | 4,569434506 | 0,023388849 | |
| 225419_at | MPLKIP | 4,496785894 | 4,473427782 | 0,023358112 | |
| 201201_at | CSTB | 7,341197789 | 7,318093364 | 0,023104425 | |
| 203931_s_at | MRPL12 | 4,417795652 | 4,394968439 | 0,022827214 | |
| 200817_x_at | RPS10 | 8,233875279 | 8,211059878 | 0,022815401 | |
| 224670_at | SYS1 | 2,380152406 | 2,357351836 | 0,02280057 | |
| 235207_at | - | 3,219589925 | 3,196911138 | 0,022678787 | |
| 218150_at | ARL5A | 4,114752306 | 4,092160663 | 0,022591642 | |
| 214047_s_at | MBD4 | 5,087185056 | 5,064617399 | 0,022567657 | |
| 200780_x_at | GNAS | 6,933912006 | 6,911354273 | 0,022557733 | |
| 208739_x_at | SUMO2 | 5,647224281 | 5,624678706 | 0,022545575 | |
| 202583_s_at | RANBP9 | 3,042527069 | 3,019999252 | 0,022527817 | |
| 208722_s_at | ANAPC5 | 3,945975838 | 3,923538485 | 0,022437353 | |
| 218059_at | ZNF706 | 5,247573834 | 5,225156313 | 0,022417521 | |
| 209444_at | RAP1GDS1 | 3,848325987 | 3,825986493 | 0,022339495 | |
| 205880_at | PRKD1 | 2,840652551 | 2,81832103 | 0,022331521 | |
| 202872_at | ATP6V1C1 | 3,800390846 | 3,778101865 | 0,022288982 | |
| 201545_s_at | BCL2L2-PABPN1 | 3,75400739 | 3,731768909 | 0,022238481 | |
| 222240_s_at | ISYNA1 | 2,716594694 | 2,694398145 | 0,02219655 | |
| 200944_s_at | HMGN1 | 7,381511472 | 7,35942032 | 0,022091151 | |
| 219765_at | ZNF329 | 2,621710292 | 2,599625088 | 0,022085204 | |

| | | | | | |
|---|---|---|---|---|---|
| 202774_s_at | SFSWAP | 2,589836943 | 2,567790814 | 0,022046129 | |
| 212837_at | FAM175B | 2,589836943 | 2,567790814 | 0,022046129 | |
| 202121_s_at | CHMP2A | 3,582860873 | 3,560823412 | 0,022037462 | |
| 212798_s_at | ANKMY2 | 4,155242648 | 4,133220924 | 0,022021724 | |
| 209503_s_at | PSMC5 | 4,873632107 | 4,851633873 | 0,021998234 | |
| 201230_s_at | ARIH2 | 2,450859799 | 2,428994491 | 0,021865308 | |
| 202172_at | VEZF1 | 2,410880861 | 2,389070873 | 0,021809988 | |
| 40472_at | LPCAT4 | 1,400248659 | 1,378453643 | 0,021795016 | |
| 233665_x_at | MTO1 | 3,92199823 | 3,900294538 | 0,021703692 | |
| 1552767_a_at | HS6ST2 | 3,336243945 | 3,314541423 | 0,021702522 | |
| 225236_at | RBM18 | 3,336243945 | 3,314541423 | 0,021702522 | |
| 224791_at | ASAP1 | 5,304868977 | 5,283213313 | 0,021655663 | |
| 213490_s_at | MAP2K2 | 2,259277178 | 2,237691445 | 0,021585732 | |
| 214394_x_at | EEF1D | 6,754764566 | 6,733322659 | 0,021441907 | |
| 218434_s_at | AACS | 3,067171967 | 3,045906378 | 0,021265589 | |
| 202016_at | MEST | 6,817080726 | 6,795824013 | 0,021256713 | |
| 220452_x_at | - | 2,027765132 | 2,006570641 | 0,021194491 | |
| 210987_x_at | TPM1 | 3,595400101 | 3,574237491 | 0,02116261 | |
| 229128_s_at | ANP32E | 3,586454644 | 3,565308644 | 0,021146001 | |
| 219553_at | NME7 | 3,574742007 | 3,553617909 | 0,021124098 | |
| 220248_x_at | NSFL1C | 4,305625125 | 4,284512471 | 0,021112653 | |
| 209042_s_at | UBE2G2 | 5,413307008 | 5,392251169 | 0,021055839 | |
| 210215_at | TFR2 | 2,938102794 | 2,917077481 | 0,021025314 | |
| 225307_at | ZNF511 | 3,512821151 | 3,491815843 | 0,021005308 | |
| 224602_at | C4orf3 | 5,245302218 | 5,224305713 | 0,020996506 | |
| 202077_at | NDUFAB1 | 6,682463892 | 6,661511506 | 0,020952387 | |
| 212862_at | CDS2 | 2,893830579 | 2,87289275 | 0,020937829 | |
| 209111_at | RNF5 | 5,02431927 | 5,003461658 | 0,020857612 | |
| 208866_at | CSNK1A1 | 2,849652795 | 2,828804976 | 0,020847819 | |
| 216941_s_at | TAF1B | 1,841816345 | 1,820984782 | 0,020831564 | |
| 51176_at | MED27 | 3,419697503 | 3,398880704 | 0,020816798 | |
| 203648_at | TATDN2 | 2,834621043 | 2,813804482 | 0,02081656 | |
| 222900_at | NRIP3 | 1,795990708 | 1,775255987 | 0,02073472 | |
| 235420_at | HAPLN4 | 1,795990708 | 1,775255987 | 0,02073472 | |
| 222632_s_at | LZTFL1 | 2,783874823 | 2,763166224 | 0,020708599 | |
| 225785_at | REEP3 | 1,780386218 | 1,759685182 | 0,020701037 | |
| 224810_s_at | ANKRD13A | 4,099113343 | 4,078419261 | 0,020694082 | |
| 1555781_at | PQLC2 | 1,748661581 | 1,728030156 | 0,020631425 | |
| 226719_at | DERL2 | 2,701760055 | 2,681234383 | 0,020525672 | |
| 222143_s_at | MTMR14 | 2,671625168 | 2,651169275 | 0,020455892 | |
| 206572_x_at | ZNF85 | 3,248232277 | 3,227795991 | 0,020436286 | |
| 230285_at | SVIP | 2,654607516 | 2,634191677 | 0,020415839 | |
| 225445_at | UBN2 | 2,614687931 | 2,594367918 | 0,020320013 | |
| 200680_x_at | HMGB1 | 7,393677617 | 7,373363377 | 0,02031424 | |
| 214931_s_at | SRPK2 | 1,593009234 | 1,572742379 | 0,020266855 | |
| 212472_at | MICAL2 | 3,906268218 | 3,886022844 | 0,020245374 | |
| 225221_at | ZKSCAN1 | 3,152077493 | 3,131875186 | 0,020202307 | |
| 224676_at | TMED4 | 2,559073676 | 2,538891651 | 0,020182025 | |
| 204020_at | PURA | 3,142344098 | 3,122166354 | 0,020177743 | |
| 1558685_a_at | LOC158960 | 2,536956583 | 2,516830926 | 0,020125658 | |
| 226050_at | TMCO3 | 3,117719377 | 3,09760452 | 0,020114857 | |
| 201346_at | ADIPOR2 | 3,532516649 | 3,512402411 | 0,020114238 | |
| 222992_s_at | NDUFB9 | 7,251659322 | 7,231567765 | 0,020091557 | |
| 200041_s_at | ATP6V1G2-DDX | 5,606212153 | 5,58613355 | 0,020078603 | |
| 217167_x_at | GK | 1,481627361 | 1,461646549 | 0,019980812 | |
| 223242_s_at | MFSD11 | 1,462198073 | 1,442269454 | 0,019928619 | |

| | | | | | |
|---|---|---|---|---|---|
| 202946_s_at | BTBD3 | 3,024094427 | 3,004228687 | 0,01986574 | |
| 209165_at | AATF | 3,425226911 | 3,405399563 | 0,019827348 | |
| 211935_at | ARL6IP1 | 5,643131999 | 5,623388815 | 0,019743184 | |
| 230085_at | PDK3 | 1,390002893 | 1,370274469 | 0,019728424 | |
| 221591_s_at | FAM64A | 2,38635068 | 2,366632651 | 0,019718029 | |
| 219251_s_at | WDR60 | 2,355089531 | 2,335461563 | 0,019627968 | |
| 213614_x_at | EEF1A1 /// LOC1 | 8,043099891 | 8,023589766 | 0,019510125 | |
| 215333_x_at | GSTM1 | 1,314003737 | 1,294497133 | 0,019506604 | |
| 228392_at | ZNF302 | 2,896728217 | 2,877228323 | 0,019499894 | |
| 227628_at | GPX8 | 2,305800198 | 2,286318242 | 0,019481956 | |
| 204162_at | NDC80 | 3,62365843 | 3,604188755 | 0,019469676 | |
| 222985_at | YWHAG | 6,103302238 | 6,083850071 | 0,019452167 | |
| 228797_at | NLN | 1,292162421 | 1,272721751 | 0,01944067 | |
| 201250_s_at | SLC2A1 | 2,860082603 | 2,840694075 | 0,019388528 | |
| 200697_at | HK1 | 5,429154674 | 5,409815321 | 0,019339353 | |
| 208785_s_at | MAP1LC3B | 4,426844657 | 4,407512498 | 0,01933216 | |
| 224377_s_at | RAB18 | 4,253455927 | 4,234134575 | 0,019321352 | |
| 212363_x_at | ACTG1 | 7,647744611 | 7,628470687 | 0,019273924 | |
| 211222_s_at | HAP1 | 1,233777334 | 1,214517879 | 0,019259455 | |
| 229094_at | ATP6V0E2-AS1 | 1,233777334 | 1,214517879 | 0,019259455 | |
| 203880_at | COX17 | 4,032172063 | 4,012941072 | 0,019230991 | |
| 224359_s_at | HOOK3 | 1,210676994 | 1,191491281 | 0,019185713 | |
| 201678_s_at | C3orf37 | 3,506195778 | 3,487095824 | 0,019099954 | |
| 204423_at | MKLN1 | 2,750626925 | 2,731588328 | 0,019038597 | |
| 209096_at | UBE2V2 | 5,331079521 | 5,312055917 | 0,019023603 | |
| 221920_s_at | SLC25A37 | 3,744388178 | 3,725370335 | 0,019017843 | |
| 204224_s_at | GCH1 | 2,122398022 | 2,103505212 | 0,01889281 | |
| 213372_at | PAQR3 | 3,684447666 | 3,665633845 | 0,018813822 | |
| 220892_s_at | PSAT1 | 2,683419202 | 2,664608955 | 0,018810247 | |
| 204641_at | NEK2 | 3,086335384 | 3,067567462 | 0,018767922 | |
| 212881_at | PIAS4 | 2,084778276 | 2,066015817 | 0,018762459 | |
| 217756_x_at | C15orf63 /// MIR1 | 6,435797263 | 6,417057788 | 0,018739476 | |
| 208632_at | RNF10 | 2,077135162 | 2,058399602 | 0,01873556 | |
| 204458_at | PLA2G15 | 1,073999308 | 1,055274826 | 0,018724482 | |
| 213297_at | RMND5B | 1,073999308 | 1,055274826 | 0,018724482 | |
| 227634_at | STK32C | 1,073999308 | 1,055274826 | 0,018724482 | |
| 200927_s_at | RAB14 | 3,875753437 | 3,857048799 | 0,018704637 | |
| 225855_at | EPB41L5 | 2,064305961 | 2,045615873 | 0,018690088 | |
| 218581_at | ABHD4 | 2,621710292 | 2,603119255 | 0,018591037 | |
| 218771_at | PANK4 | 2,030406107 | 2,011838132 | 0,018567975 | |
| 224584_at | TMEM230 | 6,387818086 | 6,369250622 | 0,018567465 | |
| 226274_at | CLCN5 | 3,833280332 | 3,814728712 | 0,01855162 | |
| 211787_s_at | EIF4A1 /// SENP3 | 7,54471392 | 7,526179779 | 0,01853414 | |
| 225155_at | SNHG5 /// SNOR | 6,24916556 | 6,230634374 | 0,018531187 | |
| 201013_s_at | PAICS | 6,059205849 | 6,040695015 | 0,018510834 | |
| 202647_s_at | NRAS | 3,597182555 | 3,578681277 | 0,018501278 | |
| 238504_at | C6orf57 | 2,003776245 | 1,98530622 | 0,018470025 | |
| 1566502_at | - | 0,995078803 | 0,976641161 | 0,018437642 | |
| 204389_at | MAOA | 2,577249059 | 2,558821855 | 0,018427204 | |
| 218580_x_at | AURKAIP1 | 4,938692955 | 4,920294977 | 0,018397978 | |
| 203012_x_at | RPL23A | 8,145431452 | 8,127067399 | 0,018364053 | |
| 225621_at | ALG2 | 3,943876123 | 3,925633118 | 0,018243005 | |
| 214052_x_at | PRRC2C | 1,940595304 | 1,922365006 | 0,018230297 | |
| 240344_x_at | LYRM7 | 1,926486359 | 1,908311039 | 0,01817532 | |
| 212075_s_at | CSNK2A1 /// CSN | 3,91129185 | 3,893176339 | 0,018115511 | |
| 217719_at | EIF3L | 6,452509245 | 6,434446889 | 0,018062356 | |

| | | | | | |
|---|---|---|---|---|---|
| 212193_s_at | LARP1 | 2,890927111 | 2,87289275 | 0,01803436 | |
| 200682_s_at | UBE2L3 | 5,800038555 | 5,78209163 | 0,017946926 | |
| 1555996_s_at | EIF4A2 /// MIR12 | 1,868628927 | 1,85068474 | 0,017944186 | |
| 205562_at | RPP38 | 2,427005622 | 2,409170778 | 0,017834844 | |
| 201104_x_at | LOC100506032 / | 3,634982121 | 3,617207412 | 0,017774709 | |
| 1553102_a_at | CCDC69 | 0,822971223 | 0,805216064 | 0,017755159 | |
| 212482_at | RMND5A | 3,812716554 | 3,795004675 | 0,017711878 | |
| 203972_s_at | PEX3 | 3,396853889 | 3,379145507 | 0,017708382 | |
| 217831_s_at | NSFL1C | 3,133773173 | 3,116064987 | 0,017708186 | |
| 218761_at | RNF111 | 2,81177561 | 2,794067719 | 0,017707892 | |
| 201548_s_at | KDM5B | 3,127619759 | 3,109937707 | 0,017682052 | |
| 227947_at | PHACTR2 | 4,591035503 | 4,573447849 | 0,017587654 | |
| 221012_s_at | TRIM8 | 4,103509368 | 4,085930792 | 0,017578576 | |
| 224841_x_at | GAS5 /// SNORD | 6,653793515 | 6,636260242 | 0,017533273 | |
| 211773_s_at | ZKSCAN3 | 1,767779954 | 1,750261389 | 0,017518565 | |
| 225460_at | SEC22C | 3,088871381 | 3,071356473 | 0,017514908 | |
| 216215_s_at | RBFOX2 | 3,082523011 | 3,065035915 | 0,017487096 | |
| 233749_at | - | 1,751865623 | 1,73441697 | 0,017448652 | |
| 225344_at | NCOA7 | 6,847725652 | 6,830286602 | 0,017439051 | |
| 230097_at | GART | 2,320980353 | 2,303602088 | 0,017378265 | |
| 201112_s_at | CSE1L | 6,407760362 | 6,390385147 | 0,017375215 | |
| 228106_at | DCAF16 | 3,537166687 | 3,519816196 | 0,017350491 | |
| 210691_s_at | CACYBP | 5,020671303 | 5,003461658 | 0,017209645 | |
| 200834_s_at | RPS21 | 7,963286258 | 7,946129893 | 0,017156365 | |
| 218756_s_at | DHRS11 | 1,67631007 | 1,659204017 | 0,017106054 | |
| 206907_at | TNFSF9 | 0,67554678 | 0,658444279 | 0,017102501 | |
| 203765_at | GCA | 2,997346717 | 2,980244813 | 0,017101904 | |
| 209642_at | BUB1 | 4,671066265 | 4,653984658 | 0,017081607 | |
| 235123_at | - | 1,666156729 | 1,64909809 | 0,017058638 | |
| 201729_s_at | KIAA0100 | 2,983784569 | 2,966746113 | 0,017038456 | |
| 227322_s_at | BCCIP | 4,661301862 | 4,644266013 | 0,017035849 | |
| 227328_at | CAMTA1 | 2,243430036 | 2,226407528 | 0,017022508 | |
| 202275_at | G6PD | 1,649074067 | 1,632095957 | 0,01697811 | |
| 231069_at | - | 1,64218396 | 1,6252386 | 0,01694536 | |
| 200784_s_at | LRP1 | 0,641402394 | 0,624460759 | 0,016941635 | |
| 225632_s_at | ISY1-RAB43 /// R | 0,641402394 | 0,624460759 | 0,016941635 | |
| 238431_at | - | 2,637386731 | 2,620464265 | 0,016922466 | |
| 208095_s_at | SRP72 | 4,723824182 | 4,706906619 | 0,016917562 | |
| 210070_s_at | CHKB-CPT1B /// | 0,606430281 | 0,589657387 | 0,016772894 | |
| 216515_x_at | - | 6,791814937 | 6,775063757 | 0,016751179 | |
| 213761_at | MDM1 | 3,170152481 | 3,153484076 | 0,016668405 | |
| 224604_at | C4orf3 | 4,480461153 | 4,463850559 | 0,016610594 | |
| 200009_at | GDI2 | 6,417629071 | 6,401025631 | 0,016603439 | |
| 219058_x_at | TINAGL1 | 0,57058931 | 0,553993624 | 0,016595686 | |
| 219223_at | CACFD1 | 0,57058931 | 0,553993624 | 0,016595686 | |
| 229158_at | WNK4 | 0,57058931 | 0,553993624 | 0,016595686 | |
| 229517_at | PTPDC1 | 0,57058931 | 0,553993624 | 0,016595686 | |
| 227812_at | TNFRSF19 | 1,560488174 | 1,543943227 | 0,016544947 | |
| 201827_at | SMARCD2 | 2,14450783 | 2,127967638 | 0,016540192 | |
| 219570_at | KIF16B | 0,533835206 | 0,51742585 | 0,016409356 | |
| 243514_at | - | 0,533835206 | 0,51742585 | 0,016409356 | |
| 200641_s_at | YWHAZ | 5,814762961 | 5,798376613 | 0,016386348 | |
| 235716_at | - | 1,523472489 | 1,507116528 | 0,016355961 | |
| 214629_x_at | RTN4 | 5,766472448 | 5,75014201 | 0,016330438 | |
| 208821_at | SNRPB | 5,619537856 | 5,603246598 | 0,016291258 | |
| 212791_at | C1orf216 | 3,095191939 | 3,078904771 | 0,016287168 | |

| | | | | | |
|---|---|---|---|---|---|
| 226044_at | TDP1 | 1,504602299 | 1,488344553 | 0,016257746 | |
| 218163_at | MCTS1 | 4,750097809 | 4,733852814 | 0,016244995 | |
| 203202_at | KRR1 | 3,500492568 | 3,484256382 | 0,016236186 | |
| 233825_s_at | CD99L2 | 0,496120222 | 0,479907041 | 0,016213181 | |
| 227072_at | RTTN | 2,074578432 | 2,058399602 | 0,01617883 | |
| 212697_at | FAM134C | 2,796341835 | 2,780243895 | 0,01609794 | |
| 211928_at | DYNC1H1 | 3,471633352 | 3,455550298 | 0,016083054 | |
| 224973_at | FAM46A | 3,63931383 | 3,623242814 | 0,016071016 | |
| 1554489_a_at | CEP70 | 1,46610492 | 1,450051551 | 0,01605337 | |
| 203263_s_at | ARHGEF9 | 2,771299138 | 2,755336312 | 0,015962826 | |
| 211951_at | NOLC1 | 6,576051313 | 6,560102664 | 0,015948649 | |
| 209566_at | INSIG2 | 2,027765132 | 2,011838132 | 0,015927 | |
| 213720_s_at | SMARCA4 | 6,139225255 | 6,123320246 | 0,015905009 | |
| 1553704_x_at | ZNF791 | 3,895444023 | 3,879554231 | 0,015889792 | |
| 238273_at | C7orf73 /// LOC1 | 1,434549838 | 1,418668082 | 0,015881755 | |
| 201659_s_at | ARL1 | 3,421207626 | 3,405399563 | 0,015808063 | |
| 204544_at | HPS5 | 2,001086023 | 1,98530622 | 0,015779803 | |
| 200690_at | HSPA9 | 3,220746604 | 3,204982027 | 0,015764577 | |
| 226877_at | RPL32P3 | 1,394109935 | 1,378453643 | 0,015656292 | |
| 221002_s_at | TSPAN14 | 2,710020346 | 2,694398145 | 0,015622202 | |
| 204478_s_at | RABIF | 2,972842302 | 2,957221307 | 0,015620995 | |
| 228275_at | LOC100505687 | 1,960118853 | 1,944570415 | 0,015548438 | |
| 203744_at | HMGB3 | 3,828735867 | 3,813221015 | 0,015514852 | |
| 218321_x_at | STYXL1 | 3,537166687 | 3,521663705 | 0,015502982 | |
| 218448_at | C20orf11 | 4,166691359 | 4,151234028 | 0,015457331 | |
| 222674_at | C9orf114 | 1,352503698 | 1,337086025 | 0,015417674 | |
| 212107_s_at | DHX9 | 3,159334685 | 3,14392006 | 0,015414625 | |
| 1552868_at | CIRBP-AS1 | 0,351548291 | 0,336136178 | 0,015412113 | |
| 213096_at | TMCC2 | 0,351548291 | 0,336136178 | 0,015412113 | |
| 218380_at | LOC728392 | 0,351548291 | 0,336136178 | 0,015412113 | |
| 239391_at | FAM120AOS | 0,351548291 | 0,336136178 | 0,015412113 | |
| 243075_at | - | 0,351548291 | 0,336136178 | 0,015412113 | |
| 218118_s_at | TIMM23 | 3,670124012 | 3,654731437 | 0,015392575 | |
| 229796_at | SIX4 | 1,932146506 | 1,916759838 | 0,015386668 | |
| 202911_at | MSH6 | 4,862929774 | 4,847588869 | 0,015340905 | |
| 218302_at | PSENEN | 4,715232482 | 4,699988715 | 0,015243766 | |
| 211871_x_at | GNB5 | 0,30867765 | 0,293518836 | 0,015158814 | |
| 214014_at | CDC42EP2 | 0,30867765 | 0,293518836 | 0,015158814 | |
| 220938_s_at | GMEB1 | 0,30867765 | 0,293518836 | 0,015158814 | |
| 239699_s_at | PMS2P1 /// PMS2 | 0,30867765 | 0,293518836 | 0,015158814 | |
| 1555790_a_at | TMEM192 /// ZNF | 3,619279389 | 3,604188755 | 0,015090634 | |
| 203973_s_at | CEBPD | 1,296557217 | 1,281471383 | 0,015085834 | |
| 219889_at | FRAT1 | 1,296557217 | 1,281471383 | 0,015085834 | |
| 208950_s_at | ALDH7A1 | 1,880387527 | 1,865308541 | 0,015078986 | |
| 232035_at | HIST1H4A /// HIS | 5,200861687 | 5,185791503 | 0,015070183 | |
| 202866_at | DNAJB12 | 2,614687931 | 2,599625088 | 0,015062843 | |
| 226434_at | PPP1R35 | 2,609398637 | 2,594367918 | 0,01503072 | |
| 214143_x_at | RPL24 | 7,758762889 | 7,743759232 | 0,015003657 | |
| 229076_s_at | - | 1,274448124 | 1,259496994 | 0,01495113 | |
| 202868_s_at | POP4 | 6,315089089 | 6,30016103 | 0,014928058 | |
| 212077_at | CALD1 | 1,261018186 | 1,246149888 | 0,014868298 | |
| 219690_at | IGFLR1 | 2,582657321 | 2,567790814 | 0,014866507 | |
| 223100_s_at | NUDT5 | 4,779830653 | 4,764991833 | 0,01483882 | |
| 203945_at | ARG2 | 2,568189989 | 2,553413592 | 0,014776397 | |
| 208897_s_at | DDX18 | 4,150394824 | 4,135635705 | 0,014759119 | |
| 201773_at | ADNP | 4,701212708 | 4,686464402 | 0,014748306 | |

| | | | | | |
|---|---|---|---|---|---|
| 218613_at | PSD3 | 1,238353345 | 1,223626587 | 0,014726758 | |
| 213574_s_at | - | 4,231245055 | 4,216563145 | 0,01468191 | |
| 213179_at | RQCD1 | 3,393780562 | 3,379145507 | 0,014635055 | |
| 59433_at | LOC100506453 / | 0,084173957 | 0,069557789 | 0,014616168 | |
| 203123_s_at | SLC11A2 | 1,805273023 | 1,790660532 | 0,014612491 | |
| 208919_s_at | NADK | 3,67603922 | 3,661450356 | 0,014588864 | |
| 1554903_at | FRMD8 | 1,215326747 | 1,200746069 | 0,014580678 | |
| 228043_at | UTP15 | 1,215326747 | 1,200746069 | 0,014580678 | |
| 1555039_a_at | ABCC4 | 1,795990708 | 1,781437557 | 0,01455315 | |
| 219406_at | C1orf50 | 1,795990708 | 1,781437557 | 0,01455315 | |
| 201727_s_at | ELAVL1 | 3,014789032 | 3,000258961 | 0,014530072 | |
| 214695_at | UBAP2L | 1,789769151 | 1,775255987 | 0,014513164 | |
| 214113_s_at | RBM8A | 4,368551674 | 4,354078399 | 0,014473274 | |
| 217391_x_at | - | 1,191926657 | 1,177496821 | 0,014429836 | |
| 218871_x_at | CSGALNACT2 | 1,191926657 | 1,177496821 | 0,014429836 | |
| 236090_at | - | 2,506929603 | 2,492544876 | 0,014384727 | |
| 228690_s_at | NDUFA11 | 5,896291018 | 5,881925611 | 0,014365407 | |
| 225156_at | ELOF1 | 5,605769525 | 5,591420757 | 0,014348769 | |
| 212083_at | TEX261 | 2,983784569 | 2,969455976 | 0,014328593 | |
| 224429_x_at | - | 2,758612869 | 2,744302548 | 0,014310321 | |
| 220935_s_at | CDK5RAP2 | 1,758252436 | 1,743944487 | 0,014307949 | |
| 1557059_at | LOC254128 | 0,171847695 | 0,157549243 | 0,014298453 | |
| 1558327_at | ZDHHC18 | 0,171847695 | 0,157549243 | 0,014298453 | |
| 227982_at | SEPSECS | 0,171847695 | 0,157549243 | 0,014298453 | |
| 229058_at | ANKRD16 | 1,168140757 | 1,153866761 | 0,014273996 | |
| 229208_at | HAUS2 | 1,168140757 | 1,153866761 | 0,014273996 | |
| 201436_at | EIF4E | 3,489018063 | 3,474751011 | 0,014267052 | |
| 222884_at | ZNF346 | 1,742232072 | 1,728030156 | 0,014201916 | |
| 211152_s_at | HTRA2 | 2,478200152 | 2,464004877 | 0,014195275 | |
| 201094_at | RPS29 | 7,860129653 | 7,845934684 | 0,014194969 | |
| 221267_s_at | FAM108A1 | 3,467741468 | 3,453616097 | 0,014125371 | |
| 202070_s_at | IDH3A | 2,466546243 | 2,452428893 | 0,01411735 | |
| 214908_s_at | TRRAP | 1,143956113 | 1,129843209 | 0,014112904 | |
| 228430_at | BOLA3-AS1 | 1,143956113 | 1,129843209 | 0,014112904 | |
| 228660_x_at | SEMA4F | 1,143956113 | 1,129843209 | 0,014112904 | |
| 1553588_at | ND3 /// SH3KBP1 | 8,330596909 | 8,316538669 | 0,01405824 | |
| 201569_s_at | SAMM50 | 4,585658616 | 4,571665519 | 0,013993097 | |
| 220741_s_at | PPA2 | 4,032830746 | 4,018839599 | 0,013991146 | |
| 206942_s_at | PMCH | 0,123193849 | 0,109221392 | 0,013972457 | |
| 209310_s_at | CASP4 | 0,123193849 | 0,109221392 | 0,013972457 | |
| 216611_s_at | SLC6A2 | 0,123193849 | 0,109221392 | 0,013972457 | |
| 229216_s_at | - | 0,123193849 | 0,109221392 | 0,013972457 | |
| 236600_at | SPG20 | 0,123193849 | 0,109221392 | 0,013972457 | |
| 218292_s_at | PRKAG2 | 1,119359127 | 1,105412836 | 0,013946292 | |
| 200001_at | CAPNS1 | 5,688357434 | 5,674417 | 0,013940434 | |
| 201762_s_at | PSME2 | 5,022330613 | 5,00841113 | 0,013919483 | |
| 226558_at | LOC389834 /// M | 2,436992838 | 2,423075926 | 0,013916913 | |
| 219639_x_at | PARP6 | 2,691784869 | 2,677924589 | 0,01386028 | |
| 1554429_a_at | DMWD | 1,104397092 | 1,090553537 | 0,013843555 | |
| 212080_at | MLL | 1,104397092 | 1,090553537 | 0,013843555 | |
| 244185_at | - | 1,104397092 | 1,090553537 | 0,013843555 | |
| 225000_at | PRKAR2A | 3,687797371 | 3,673964604 | 0,013832767 | |
| 238538_at | ANKRD11 | 2,422991296 | 2,409170778 | 0,013820518 | |
| 204282_s_at | FARS2 | 2,097427503 | 2,083632167 | 0,013795336 | |
| 1558914_at | DESI2 | 1,094335495 | 1,080561626 | 0,013773869 | |
| 218985_at | SLC2A8 | 1,094335495 | 1,080561626 | 0,013773869 | |

| | | | | | |
|---|---|---|---|---|---|
| 230020_at | NAA38 | 1,094335495 | 1,080561626 | 0,013773869 | |
| 202162_s_at | CNOT8 | 3,092667038 | 3,078904771 | 0,013762267 | |
| 221848_at | ZGPAT | 1,079110292 | 1,065442793 | 0,013667499 | |
| 228669_x_at | PARP10 | 0,072842263 | 0,059218869 | 0,013623394 | |
| 232167_at | SLC2A11 | 0,072842263 | 0,059218869 | 0,013623394 | |
| 225113_at | AGPS | 3,072307152 | 3,058687544 | 0,013619608 | |
| 201874_at | MPZL1 | 1,638726528 | 1,6252386 | 0,013487928 | |
| 210470_x_at | NONO | 5,456414609 | 5,442974965 | 0,013439644 | |
| 235014_at | LOC147727 | 4,499171087 | 4,485783875 | 0,013387213 | |
| 219831_at | CDKL3 | 1,027166904 | 1,013870809 | 0,013296095 | |
| 232841_at | - | 1,027166904 | 1,013870809 | 0,013296095 | |
| 208113_x_at | PABPC3 | 7,043008909 | 7,029719849 | 0,01328906 | |
| 221692_s_at | MRPL34 | 5,640325313 | 5,627040516 | 0,013284797 | |
| 203893_at | TAF9 | 5,112331944 | 5,099052587 | 0,013279357 | |
| 1554393_a_at | ADC | 0,020670649 | 0,007421914 | 0,013248736 | |
| 209119_x_at | NR2F2 | 0,020670649 | 0,007421914 | 0,013248736 | |
| 213464_at | SHC2 | 0,020670649 | 0,007421914 | 0,013248736 | |
| 227453_at | UNC13A | 0,020670649 | 0,007421914 | 0,013248736 | |
| 240257_at | SYNJ2 | 0,020670649 | 0,007421914 | 0,013248736 | |
| 216620_s_at | ARHGEF10 | 3,341570404 | 3,32832922 | 0,013241184 | |
| 208971_at | UROD | 5,016681155 | 5,003461658 | 0,013219497 | |
| 217924_at | C6orf106 | 2,338135744 | 2,324919719 | 0,013216025 | |
| 218181_s_at | MAP4K4 | 2,338135744 | 2,324919719 | 0,013216025 | |
| 228612_at | LOC100506233 | 2,338135744 | 2,324919719 | 0,013216025 | |
| 57082_at | LDLRAP1 | 0,592200562 | 0,579050522 | 0,01315004 | |
| 211716_x_at | ARHGDIA | 2,589836943 | 2,576704359 | 0,013132584 | |
| 239669_at | - | 3,325531666 | 3,312408479 | 0,013123187 | |
| 209329_x_at | HIGD2A | 4,8851678 | 4,872052597 | 0,013115203 | |
| 238821_at | CSTF2 | 1,000476627 | 0,987376598 | 0,01310003 | |
| 213220_at | BBIP1 | 3,000043914 | 2,986947093 | 0,013096821 | |
| 202788_at | MAPKAPK3 | 2,31665944 | 2,303602088 | 0,013057352 | |
| 209584_x_at | APOBEC3C | 3,308224834 | 3,295230443 | 0,012994391 | |
| 234311_s_at | GTPBP10 | 2,303618506 | 2,290658654 | 0,012959852 | |
| 201947_s_at | CCT2 | 6,701386602 | 6,68848869 | 0,012897912 | |
| 203034_s_at | RPL27A /// SNOF | 8,029179258 | 8,016319044 | 0,012860214 | |
| 202045_s_at | ARHGAP35 | -0,033456564 | -0,046302147 | 0,012845583 | |
| 204572_s_at | PIN4 | -0,033456564 | -0,046302147 | 0,012845583 | |
| 215296_at | CDC42BPA | -0,033456564 | -0,046302147 | 0,012845583 | |
| 203128_at | SPTLC2 | 1,549482847 | 1,536652708 | 0,012830139 | |
| 229304_s_at | MLF1IP | 1,549482847 | 1,536652708 | 0,012830139 | |
| 1555281_x_at | ARMC8 | 2,28383338 | 2,271023131 | 0,012810249 | |
| 201803_at | POLR2B | 5,867963534 | 5,855159624 | 0,01280391 | |
| 213315_x_at | CXorf40A | 2,768138014 | 2,755336312 | 0,012801702 | |
| 202860_at | DENND4B | 2,764969948 | 2,752192409 | 0,012777539 | |
| 33322_i_at | SFN | 2,755423794 | 2,742719384 | 0,01270441 | |
| 204189_at | RARG | 0,945567441 | 0,932882274 | 0,012685168 | |
| 232106_s_at | CEP89 | 0,945567441 | 0,932882274 | 0,012685168 | |
| 222875_at | DHX33 | 3,388643755 | 3,376085218 | 0,012558537 | |
| 212228_s_at | COQ9 | 3,513765154 | 3,501209806 | 0,012555348 | |
| 235593_at | ZEB2 | 2,250242994 | 2,237691445 | 0,012551549 | |
| 1557165_s_at | KLHL18 | 1,512180019 | 1,499637023 | 0,012542996 | |
| 203916_at | NDST2 | 1,512180019 | 1,499637023 | 0,012542996 | |
| 49327_at | SIRT3 | 2,243430036 | 2,230931691 | 0,012498345 | |
| 1554316_at | PGLS | -0,089689931 | -0,102100538 | 0,012410606 | |
| 1559654_s_at | - | -0,089689931 | -0,102100538 | 0,012410606 | |
| 215161_at | CAMK1G | -0,089689931 | -0,102100538 | 0,012410606 | |

| | | | | | |
|---|---|---|---|---|---|
| 225868_at | TRIM47 | -0,089689931 | -0,102100538 | 0,012410606 | |
| 226565_at | TMEM99 | -0,089689931 | -0,102100538 | 0,012410606 | |
| 240746_s_at | - | -0,089689931 | -0,102100538 | 0,012410606 | |
| 222701_s_at | CHCHD7 | 4,230671025 | 4,21827299 | 0,012398035 | |
| 200053_at | SPAG7 | 3,484209953 | 3,471887144 | 0,012322808 | |
| 209411_s_at | GGA3 | 1,481627361 | 1,469325098 | 0,012302263 | |
| 203689_s_at | FMR1 | 2,47626436 | 2,464004877 | 0,012259482 | |
| 204451_at | FZD1 | 0,888485433 | 0,876248481 | 0,012236952 | |
| 207232_s_at | DZIP3 | 0,888485433 | 0,876248481 | 0,012236952 | |
| 218658_s_at | ACTR8 | 0,888485433 | 0,876248481 | 0,012236952 | |
| 200893_at | TRA2B | 6,58168915 | 6,569454465 | 0,012234684 | |
| 224162_s_at | FBXO31 | 2,208874124 | 2,196649505 | 0,012224619 | |
| 202148_s_at | PYCR1 | 3,199783157 | 3,187631636 | 0,012151521 | |
| 219968_at | ZNF589 | 1,462198073 | 1,450051551 | 0,012146523 | |
| 224981_at | TMEM219 | 2,680059304 | 2,667949403 | 0,012109902 | |
| 203032_s_at | FH | 2,190100188 | 2,17802703 | 0,012073157 | |
| 231847_at | ANKRD54 | 2,190100188 | 2,17802703 | 0,012073157 | |
| 220789_s_at | SNORA5B /// TBI | 3,453052675 | 3,440980183 | 0,012072492 | |
| 235509_at | LOC100506538 / | 4,773538862 | 4,761478085 | 0,012060778 | |
| 223993_s_at | CNIH4 | 5,111708585 | 5,099671136 | 0,012037449 | |
| 225441_x_at | LSMD1 | 3,769898242 | 3,757867094 | 0,012031148 | |
| 218115_at | ASF1B | 4,252325187 | 4,24031851 | 0,012006678 | |
| 1553253_at | ASB16 | 0,859074518 | 0,847075464 | 0,011999055 | |
| 243618_s_at | ZNF827 | 1,442503549 | 1,43051703 | 0,011986518 | |
| 204504_s_at | HIRIP3 | 2,178241192 | 2,166264721 | 0,01197647 | |
| 236271_at | - | -0,148196756 | -0,160136748 | 0,011939992 | |
| 238819_at | ZNF347 | -0,148196756 | -0,160136748 | 0,011939992 | |
| 201674_s_at | AKAP1 | 2,166283906 | 2,154405725 | 0,01187818 | |
| 238835_at | AVPR1A | 2,837639949 | 2,825817328 | 0,01182262 | |
| 228535_at | RAD1 | 1,422536444 | 1,410714371 | 0,011822073 | |
| 203040_s_at | HMBS | 2,420979937 | 2,409170778 | 0,011809159 | |
| 213294_at | EIF2AK2 | 4,000869774 | 3,989102799 | 0,011766975 | |
| 227041_at | SESTD1 | 0,82905153 | 0,817300337 | 0,011751194 | |
| 218039_at | NUSAP1 | 4,685380613 | 4,673643206 | 0,011737407 | |
| 225643_at | MAPK1IP1L | 4,461505382 | 4,449849671 | 0,011655711 | |
| 201051_at | ANP32A | 5,102639473 | 5,090987226 | 0,011652247 | |
| 204949_at | ICAM3 | 5,30704878 | 5,295413712 | 0,011635067 | |
| 227087_at | INPP4A | 2,814842651 | 2,803210557 | 0,011632094 | |
| 210346_s_at | CLK4 | 2,134723057 | 2,123108223 | 0,011614834 | |
| 229905_at | RAP1GDS1 | 1,390002893 | 1,378453643 | 0,01154925 | |
| 235595_at | ARHGEF2 | 1,390002893 | 1,378453643 | 0,01154925 | |
| 211745_x_at | HBA1 /// HBA2 | 5,532907547 | 5,521358749 | 0,011548798 | |
| 213304_at | FAM179B | 3,121439986 | 3,109937707 | 0,011502279 | |
| 216833_x_at | GYPB | 0,798390451 | 0,786897721 | 0,01149273 | |
| 224980_at | LEMD2 | 0,798390451 | 0,786897721 | 0,01149273 | |
| 232019_at | ZKSCAN2 | 1,381753566 | 1,370274469 | 0,011479097 | |
| 200845_s_at | PRDX6 | 5,883749359 | 5,872274565 | 0,011474793 | |
| 45633_at | GINS3 | 3,545960679 | 3,534530441 | 0,011430238 | |
| 220864_s_at | NDUFA13 | 6,309799654 | 6,298410885 | 0,011388769 | |
| 225295_at | SLC39A10 | 3,785615967 | 3,77423254 | 0,011383427 | |
| 235407_at | - | 1,369290453 | 1,3579181 | 0,011372353 | |
| 201624_at | DARS | 4,175069494 | 4,163710704 | 0,01135879 | |
| 223387_at | ZFYVE1 | 2,950705598 | 2,939363796 | 0,011341803 | |
| 1558369_at | MPHOSPH9 | 2,102456302 | 2,091116676 | 0,011339626 | |
| 235008_at | - | 0,779676066 | 0,768343791 | 0,011332275 | |
| 241996_at | - | 0,779676066 | 0,768343791 | 0,011332275 | |

| | | | | | |
|---|---|---|---|---|---|
| 219326_s_at | B3GNT2 | 3,22766727 | 3,216434104 | 0,011233165 | |
| 222777_s_at | WHSC1 | 0,767063567 | 0,755840599 | 0,011222968 | |
| 217835_x_at | C20orf24 /// TGIF | 6,441265426 | 6,430088484 | 0,011176941 | |
| 230391_at | CD84 | 2,342392905 | 2,331254065 | 0,01113884 | |
| 223339_at | ATPIF1 | 3,072307152 | 3,061230246 | 0,011076906 | |
| 1556228_a_at | VCPIP1 | 0,747935635 | 0,73688026 | 0,011055375 | |
| 206672_at | AQP2 | 0,747935635 | 0,73688026 | 0,011055375 | |
| 217962_at | NOP10 | 6,540132993 | 6,529097199 | 0,011035794 | |
| 219293_s_at | OLA1 | 5,931156513 | 5,920159511 | 0,010997002 | |
| 204076_at | ENTPD4 | 3,325531666 | 3,314541423 | 0,010990243 | |
| 208289_s_at | EI24 | 3,642769857 | 3,631821253 | 0,010948604 | |
| 212961_x_at | CXorf40B | 3,056846434 | 3,045906378 | 0,010940056 | |
| 209680_s_at | KIFC1 | 3,421207626 | 3,410394103 | 0,010813523 | |
| 239277_at | - | 1,305306849 | 1,294497133 | 0,010809717 | |
| 207782_s_at | PSEN1 | 2,716594694 | 2,705818724 | 0,010775971 | |
| 220172_at | DCAF17 | 0,715481152 | 0,704715219 | 0,010765932 | |
| 227627_at | C8orf44-SGK3 /// | 0,715481152 | 0,704715219 | 0,010765932 | |
| 208308_s_at | GPI | 6,149283408 | 6,138524926 | 0,010758482 | |
| 200090_at | FNTA | 5,758729106 | 5,747973514 | 0,010755592 | |
| 219029_at | C5orf28 | 2,030406107 | 2,019703483 | 0,010702625 | |
| 229744_at | SSFA2 | 2,030406107 | 2,019703483 | 0,010702625 | |
| 230077_at | LOC220729 /// SI | 1,283332461 | 1,272721751 | 0,01061071 | |
| 233506_at | - | 1,283332461 | 1,272721751 | 0,01061071 | |
| 219356_s_at | CHMP5 | 4,963779248 | 4,953181111 | 0,010598137 | |
| 205036_at | LSM6 | 4,422829883 | 4,412250787 | 0,010579096 | |
| 205103_at | C1orf61 | 1,011212064 | 1,000684521 | 0,010527543 | |
| 201349_at | SLC9A3R1 | 1,269985355 | 1,259496994 | 0,01048836 | |
| 214473_x_at | PMS2P3 | 2,683419202 | 2,672945617 | 0,010473584 | |
| 203680_at | PRKAR2B | 5,726519244 | 5,716057595 | 0,010461649 | |
| 214527_s_at | PQBP1 | 5,231023008 | 5,220613974 | 0,010409034 | |
| 216242_x_at | POLR2J2 /// POL | 1,67631007 | 1,665902186 | 0,010407885 | |
| 206734_at | JRKL | 1,990274687 | 1,979940779 | 0,010333908 | |
| 222734_at | WARS2 | 1,990274687 | 1,979940779 | 0,010333908 | |
| 218571_s_at | CHMP4A | 3,915583941 | 3,905256463 | 0,010327478 | |
| 227186_s_at | MRPL41 | 4,749296484 | 4,73902338 | 0,010273105 | |
| 223758_s_at | GTF2H2 | 3,744388178 | 3,734161076 | 0,010227102 | |
| 203890_s_at | DAPK3 | 1,233777334 | 1,223626587 | 0,010150746 | |
| 210130_s_at | TM7SF2 | 0,648296226 | 0,638150376 | 0,010145849 | |
| 212879_x_at | PIAS4 | 1,968405881 | 1,958277216 | 0,010128665 | |
| 224502_s_at | KIAA1191 | 3,815781597 | 3,805658807 | 0,01012279 | |
| 204825_at | MELK | 4,855129945 | 4,845008859 | 0,010121087 | |
| 1560303_at | FKBP4 | 1,224581536 | 1,214517879 | 0,010063657 | |
| 202615_at | GNAQ | 3,807337009 | 3,797294336 | 0,010042673 | |
| 210719_s_at | HMG20B | 2,804079361 | 2,794067719 | 0,010011642 | |
| 212002_at | C1orf144 | 1,954567604 | 1,944570415 | 0,009997189 | |
| 225831_at | LUZP1 | 1,954567604 | 1,944570415 | 0,009997189 | |
| 219590_x_at | DPH5 | 3,719237563 | 3,709248632 | 0,009988931 | |
| 214687_x_at | ALDOA | 7,114253219 | 7,104277067 | 0,009976152 | |
| 220797_at | METTL16 | 1,210676994 | 1,200746069 | 0,009930925 | |
| 235848_x_at | - | 1,210676994 | 1,200746069 | 0,009930925 | |
| 200747_s_at | NUMA1 | 1,946200473 | 1,936283387 | 0,009917086 | |
| 236283_x_at | LOC646214 | 1,940595304 | 1,930732138 | 0,009863166 | |
| 213918_s_at | NIPBL | 3,319065909 | 3,309203139 | 0,009862771 | |
| 211672_s_at | ARPC4 | 3,203298287 | 3,193438316 | 0,009859971 | |
| 203581_at | RAB4A /// SPHAI | 3,525045272 | 3,515187047 | 0,009858225 | |
| 227481_at | CNKSR3 | 1,201332287 | 1,191491281 | 0,009841006 | |

| | | | | | |
|---|---|---|---|---|---|
| 228790_at | FAM110B | 1,201332287 | 1,191491281 | 0,009841006 | |
| 1554173_at | CD300LB | 0,613492853 | 0,603679647 | 0,009813206 | |
| 219259_at | SEMA4A | 0,613492853 | 0,603679647 | 0,009813206 | |
| 226523_at | TAGLN | 0,613492853 | 0,603679647 | 0,009813206 | |
| 239790_s_at | - | 0,613492853 | 0,603679647 | 0,009813206 | |
| 220254_at | LRP12 | 1,932146506 | 1,922365006 | 0,009781499 | |
| 242705_x_at | LRPAP1 | 1,932146506 | 1,922365006 | 0,009781499 | |
| 218389_s_at | APH1A | 3,780134221 | 3,770352811 | 0,00978141 | |
| 226881_at | GRPEL2 | 3,308224834 | 3,298466943 | 0,009757891 | |
| 222431_at | SPIN1 | 4,41122478 | 4,401504959 | 0,009719821 | |
| 217125_at | - | 1,187200746 | 1,177496821 | 0,009703926 | |
| 202318_s_at | SENP6 | 3,914869478 | 3,905256463 | 0,009613016 | |
| 210270_at | RGS6 | 1,177702228 | 1,16809119 | 0,009611037 | |
| 219927_at | FCF1 | 1,90937145 | 1,899812471 | 0,009558979 | |
| 223184_s_at | AGPAT3 | 3,391728033 | 3,382199319 | 0,009528715 | |
| 225516_at | SLC7A2 | 2,901063789 | 2,891586849 | 0,009476941 | |
| 217821_s_at | WBP11 | 2,38635068 | 2,376875125 | 0,009475555 | |
| 208830_s_at | SUPT6H | 1,163336153 | 1,153866761 | 0,009469392 | |
| 1561546_at | FGD5-AS1 | 0,57782909 | 0,568365095 | 0,009463995 | |
| 203636_at | MID1 | 0,57782909 | 0,568365095 | 0,009463995 | |
| 200713_s_at | MAPRE1 | 6,339120524 | 6,32979092 | 0,009329604 | |
| 1555971_s_at | FBXO28 | 3,651373876 | 3,642048499 | 0,009325377 | |
| 232649_at | GLDN | 0,541261317 | 0,532164371 | 0,009096946 | |
| 1556291_at | POM121L12 | 1,859746653 | 1,85068474 | 0,009061913 | |
| 204925_at | CTNS | 1,534677255 | 1,525647381 | 0,009029875 | |
| 209820_s_at | TBL3 | 1,534677255 | 1,525647381 | 0,009029875 | |
| 218108_at | UBR7 | 4,696231843 | 4,687287676 | 0,008944167 | |
| 204848_x_at | HBG1 /// HBG2 // | 8,123490517 | 8,114574402 | 0,008916115 | |
| 216942_s_at | CD58 | 2,107467633 | 2,098562556 | 0,008905077 | |
| 224710_at | NARR /// RAB34 | 3,842326541 | 3,833443169 | 0,008883372 | |
| 1554126_at | MSRB3 | 1,515954005 | 1,507116528 | 0,008837478 | |
| 216060_s_at | DAAM1 | 3,416167722 | 3,407399455 | 0,008768267 | |
| 201898_s_at | UBE2A | 4,315944589 | 4,307183466 | 0,008761123 | |
| 204348_s_at | AK4 /// LOC1005 | 4,315944589 | 4,307183466 | 0,008761123 | |
| 226148_at | ZBTB44 | 3,829494273 | 3,820743792 | 0,008750481 | |
| 203460_s_at | PSEN1 | 3,312571045 | 3,303845028 | 0,008726017 | |
| 1556064_at | LOC284926 | 1,089278259 | 1,080561626 | 0,008716634 | |
| 1557478_at | - | 0,503742507 | 0,495031851 | 0,008710656 | |
| 243294_at | ZNF780B | 0,503742507 | 0,495031851 | 0,008710656 | |
| 202146_at | IFRD1 | 4,825404123 | 4,816696246 | 0,008707877 | |
| 78383_at | LOC100129250 | 2,08731701 | 2,078620836 | 0,008696174 | |
| 234111_at | - | 2,305800198 | 2,297144887 | 0,008655311 | |
| 227346_at | IKZF1 | 2,819431021 | 2,810785576 | 0,008645445 | |
| 203911_at | RAP1GAP | 1,496984567 | 1,488344553 | 0,008640014 | |
| 211341_at | POU4F1 | 1,496984567 | 1,488344553 | 0,008640014 | |
| 201920_at | SLC20A1 | 6,66414022 | 6,655529249 | 0,008610971 | |
| 229377_at | GRTP1 | 2,074578432 | 2,066015817 | 0,008562615 | |
| 208676_s_at | PA2G4 | 4,861076493 | 4,852551612 | 0,008524881 | |
| 211456_x_at | MT1P2 | 4,801251771 | 4,792797923 | 0,008453849 | |
| 243759_at | SCAF4 | 1,063722698 | 1,055274826 | 0,008447872 | |
| 206302_s_at | NUDT4 /// NUDT | 4,148572682 | 4,140152555 | 0,008420127 | |
| 212987_at | FBXO9 | 5,257470309 | 5,249050572 | 0,008419736 | |
| 32137_at | JAG2 | 1,278897131 | 1,270526026 | 0,008371105 | |
| 1560156_at | - | 0,46522187 | 0,456918297 | 0,008303573 | |
| 207083_s_at | CC2D1A | 0,46522187 | 0,456918297 | 0,008303573 | |
| 214364_at | MTERFD2 | 0,46522187 | 0,456918297 | 0,008303573 | |

| | | | | | |
|---|---|---|---|---|---|
| 217196_s_at | CAMSAP2 | 0,46522187 | 0,456918297 | 0,008303573 | |
| 239930_at | GALNT2 | 0,46522187 | 0,456918297 | 0,008303573 | |
| 241722_x_at | - | 0,46522187 | 0,456918297 | 0,008303573 | |
| 203981_s_at | ABCD4 | 2,26825514 | 2,259997914 | 0,008257227 | |
| 235072_s_at | KIF13A | 2,779171778 | 2,770953871 | 0,008217907 | |
| 222014_x_at | MTO1 | 3,625406332 | 3,617207412 | 0,00819892 | |
| 223005_s_at | TMEM245 | 3,154500615 | 3,146317015 | 0,008183601 | |
| 219717_at | DCAF16 | 1,037706275 | 1,029536893 | 0,008169382 | |
| 212511_at | PICALM | 4,205184875 | 4,197041937 | 0,008142938 | |
| 226771_at | ATP8B2 | 3,534378463 | 3,526272152 | 0,008106311 | |
| 222570_at | NCS1 | 0,44160593 | 0,433557298 | 0,008048632 | |
| 213473_at | BRAP | 2,763383302 | 2,755336312 | 0,008046991 | |
| 218024_at | BRP44L | 3,831767101 | 3,823741953 | 0,008025147 | |
| 1552610_a_at | JAK1 | 3,024094427 | 3,016072743 | 0,008021684 | |
| 201692_at | SIGMAR1 | 3,024094427 | 3,016072743 | 0,008021684 | |
| 200711_s_at | SKP1 | 4,192270951 | 4,18426834 | 0,008002611 | |
| 213037_x_at | STAU1 | 5,233890179 | 5,226006411 | 0,007883767 | |
| 225008_at | ASPH | 0,425644434 | 0,417770464 | 0,00787397 | |
| 1555500_s_at | SLC2A4RG | 1,742232072 | 1,73441697 | 0,007815102 | |
| 217720_at | CHCHD2 | 6,361972127 | 6,354166374 | 0,007805753 | |
| 226011_at | CCDC12 | 4,166691359 | 4,158970399 | 0,007720961 | |
| 91952_at | DCAF15 | 2,92893181 | 2,921282473 | 0,007649337 | |
| 244044_at | - | 4,49104529 | 4,483415907 | 0,007629383 | |
| 206090_s_at | DISC1 /// TSNAX | 0,401366113 | 0,393761504 | 0,007604609 | |
| 207188_at | CDK3 /// TEN1-C | 0,401366113 | 0,393761504 | 0,007604609 | |
| 227849_at | RP9 | 0,401366113 | 0,393761504 | 0,007604609 | |
| 209515_s_at | RAB27A | 2,400710591 | 2,39311332 | 0,007597272 | |
| 219397_at | COQ10B | 3,792633459 | 3,785040604 | 0,007592855 | |
| 207459_x_at | GYPB | 0,984222186 | 0,976641161 | 0,007581025 | |
| 208769_at | EIF4EBP2 | 0,984222186 | 0,976641161 | 0,007581025 | |
| 210813_s_at | XRCC4 | 0,984222186 | 0,976641161 | 0,007581025 | |
| 212626_x_at | HNRNPC /// LOC | 6,146395059 | 6,138825886 | 0,007569173 | |
| 223172_s_at | MTFP1 | 3,396853889 | 3,38929984 | 0,007554049 | |
| 211563_s_at | URI1 | 5,254649665 | 5,247097694 | 0,00755197 | |
| 200055_at | TAF10 | 3,981211211 | 3,973663955 | 0,007547256 | |
| 201125_s_at | ITGB5 | 2,981056773 | 2,973511251 | 0,007545522 | |
| 218655_s_at | CWC25 | 1,973904239 | 1,966439221 | 0,007465018 | |
| 202443_x_at | NOTCH2 | 3,634982121 | 3,62753841 | 0,007443712 | |
| 204813_at | MAPK10 | 0,384950573 | 0,377530647 | 0,007419927 | |
| 212947_at | SLC9A8 | 0,384950573 | 0,377530647 | 0,007419927 | |
| 222045_s_at | PCIF1 | 0,384950573 | 0,377530647 | 0,007419927 | |
| 226433_at | RNF157 | 0,384950573 | 0,377530647 | 0,007419927 | |
| 232438_at | EPS15L1 | 0,384950573 | 0,377530647 | 0,007419927 | |
| 236535_at | SMC6 | 0,384950573 | 0,377530647 | 0,007419927 | |
| 219076_s_at | PXMP2 | 2,190100188 | 2,182705233 | 0,007394955 | |
| 49329_at | KLHL22 | 1,381753566 | 1,374369853 | 0,007383714 | |
| 226668_at | WDSUB1 | 2,380152406 | 2,372786857 | 0,007365549 | |
| 216969_s_at | KIF22 | 1,699726954 | 1,692388143 | 0,007338812 | |
| 209021_x_at | ATG13 | 2,696781084 | 2,689475799 | 0,007305285 | |
| 203067_at | PDHX | 4,075012622 | 4,067710529 | 0,007302094 | |
| 223216_x_at | FBXO16 /// ZNF3 | 0,95671774 | 0,949447784 | 0,007269956 | |
| 227256_at | USP31 | 0,95671774 | 0,949447784 | 0,007269956 | |
| 218483_s_at | IFT46 | 2,178241192 | 2,170981159 | 0,007260033 | |
| 228195_at | C2orf88 | 2,178241192 | 2,170981159 | 0,007260033 | |
| 228764_s_at | MDP1 /// NEDD8- | 2,178241192 | 2,170981159 | 0,007260033 | |
| 226456_at | RMI2 | 2,691784869 | 2,684536601 | 0,007248268 | |

| | | | | | |
|---|---|---|---|---|---|
| 564_at | GNA11 | 3,412629284 | 3,405399563 | 0,00722972 | |
| 203417_at | MFAP2 | 0,359971644 | 0,352836757 | 0,007134887 | |
| 213557_at | - | 0,359971644 | 0,352836757 | 0,007134887 | |
| 217040_x_at | SOX15 | 0,359971644 | 0,352836757 | 0,007134887 | |
| 226253_at | LRRC45 | 0,359971644 | 0,352836757 | 0,007134887 | |
| 233984_at | - | 0,359971644 | 0,352836757 | 0,007134887 | |
| 241268_x_at | - | 0,359971644 | 0,352836757 | 0,007134887 | |
| 218192_at | IP6K2 | 2,35929703 | 2,352169913 | 0,007127116 | |
| 232676_x_at | MYEF2 | 2,166283906 | 2,159161028 | 0,007122878 | |
| 208718_at | DDX17 | 5,994320507 | 5,987211837 | 0,00710867 | |
| 219220_x_at | MRPS22 | 4,912089092 | 4,904982678 | 0,007106414 | |
| 215158_s_at | DEDD | 2,675004741 | 2,667949403 | 0,007055339 | |
| 203416_at | CD53 | 2,352981171 | 2,345926937 | 0,007054234 | |
| 212702_s_at | BICD2 | 1,937784532 | 1,930732138 | 0,007052394 | |
| 207525_s_at | GIPC1 | 3,600740871 | 3,593688577 | 0,007052294 | |
| 204067_at | SUOX | 1,672933557 | 1,665902186 | 0,007031371 | |
| 229232_at | LRRC57 | 1,666156729 | 1,659204017 | 0,006952712 | |
| 219298_at | ECHDC3 | 0,928678725 | 0,921731975 | 0,006946749 | |
| 225565_at | CREB1 | 0,928678725 | 0,921731975 | 0,006946749 | |
| 223066_at | SNAPIN | 3,66163142 | 3,654731437 | 0,006899983 | |
| 1557360_at | LRPPRC | 2,338135744 | 2,331254065 | 0,006881679 | |
| 226196_s_at | IFT43 | 2,338135744 | 2,331254065 | 0,006881679 | |
| 226813_at | NTPCR | 2,338135744 | 2,331254065 | 0,006881679 | |
| 1565580_s_at | TATDN2 | -0,67289328 | -0,679763839 | 0,006870559 | |
| 1566927_at | LINC00527 | -0,67289328 | -0,679763839 | 0,006870559 | |
| 210030_at | - | -0,67289328 | -0,679763839 | 0,006870559 | |
| 213755_s_at | - | -0,67289328 | -0,679763839 | 0,006870559 | |
| 213866_at | SAMD14 | -0,67289328 | -0,679763839 | 0,006870559 | |
| 214049_x_at | CD7 | -0,67289328 | -0,679763839 | 0,006870559 | |
| 232043_at | GNG7 | -0,67289328 | -0,679763839 | 0,006870559 | |
| 237360_at | ACTRT2 | -0,67289328 | -0,679763839 | 0,006870559 | |
| 239463_at | - | -0,67289328 | -0,679763839 | 0,006870559 | |
| 240774_at | - | -0,67289328 | -0,679763839 | 0,006870559 | |
| 208942_s_at | SEC62 | 4,091545995 | 4,084681583 | 0,006864412 | |
| 203827_at | WIPI1 | 1,331241794 | 1,324440845 | 0,006800949 | |
| 242268_at | CELF2 | 1,331241794 | 1,324440845 | 0,006800949 | |
| 225331_at | CCDC50 | 3,717599787 | 3,710868933 | 0,006730854 | |
| 202377_at | LEPROT | 4,132069007 | 4,125344852 | 0,006724155 | |
| 233952_s_at | ZNF295 | 3,569303935 | 3,562619178 | 0,006684757 | |
| 224807_at | GRAMD1A | 2,640847372 | 2,634191677 | 0,006655695 | |
| 226861_at | ASB8 | 2,640847372 | 2,634191677 | 0,006655695 | |
| 218154_at | GSDMD | 0,317354302 | 0,310717132 | 0,00663717 | |
| 1560527_at | NFE4 | 4,173278271 | 4,166665505 | 0,006612766 | |
| 1556006_s_at | CSNK1A1 | 0,900083947 | 0,893473268 | 0,006610679 | |
| 203644_s_at | MON1B | 0,900083947 | 0,893473268 | 0,006610679 | |
| 228759_at | CREB3L2 | 0,900083947 | 0,893473268 | 0,006610679 | |
| 208915_s_at | GGA2 | 2,314494121 | 2,307890888 | 0,006603233 | |
| 218277_s_at | DHX40 | 2,635653293 | 2,629059192 | 0,0065941 | |
| 225297_at | HAUS1 | 4,065387235 | 4,058831494 | 0,006555741 | |
| 226259_at | EXOC6 | 3,69780007 | 3,691304166 | 0,006495905 | |
| 203667_at | TBCA | 6,484280248 | 6,477797627 | 0,006482621 | |
| 209579_s_at | MBD4 | 4,551591289 | 4,545117128 | 0,006474161 | |
| 201934_at | WDR82 | 5,220798283 | 5,214344813 | 0,00645347 | |
| 202501_at | MAPRE2 | 3,76038475 | 3,753943043 | 0,006441707 | |
| 1564053_a_at | YTHDF3 | 2,470441349 | 2,464004877 | 0,006436472 | |
| 202968_s_at | DYRK2 | 0,882651021 | 0,876248481 | 0,00640254 | |

| | | | | | |
|---|---|---|---|---|---|
| 216680_s_at | EPHB4 | 0,882651021 | 0,876248481 | 0,00640254 | |
| 48580_at | CXXC1 | 3,618401983 | 3,612014038 | 0,006387945 | |
| 1554767_s_at | CRYZL1 | 1,880387527 | 1,874012195 | 0,006375332 | |
| 223701_s_at | USP47 | 2,614687931 | 2,608344689 | 0,006343242 | |
| 219299_at | TRMT12 | 3,45992606 | 3,453616097 | 0,006309963 | |
| 1568597_at | LOC646762 | 2,609398637 | 2,603119255 | 0,006279383 | |
| 212340_at | YIPF6 | 1,603688695 | 1,59747851 | 0,006210184 | |
| 223083_s_at | EGLN2 /// RAB4E | 1,865674241 | 1,859476797 | 0,006197444 | |
| 235114_x_at | HOOK3 | 1,865674241 | 1,859476797 | 0,006197444 | |
| 228487_s_at | RREB1 | 3,085065712 | 3,078904771 | 0,006160941 | |
| 1562641_at | FAM122C | 0,273439642 | 0,26733076 | 0,006108882 | |
| 219205_at | SRR | 0,273439642 | 0,26733076 | 0,006108882 | |
| 227738_s_at | ARMC5 | 0,273439642 | 0,26733076 | 0,006108882 | |
| 242996_at | MTRF1 | 0,273439642 | 0,26733076 | 0,006108882 | |
| 212581_x_at | GAPDH | 7,896140746 | 7,890068656 | 0,00607209 | |
| 238677_at | WDR36 | 2,077135162 | 2,071071048 | 0,006064114 | |
| 235235_s_at | PATL1 | 0,853119696 | 0,847075464 | 0,006044232 | |
| 215016_x_at | DST /// LOC1006 | 2,266015882 | 2,259997914 | 0,006017969 | |
| 212098_at | MGAT5 | 4,123436638 | 4,117425547 | 0,006011091 | |
| 218361_at | GOLPH3L | 3,653945107 | 3,647981081 | 0,005964026 | |
| 1559646_a_at | LINC00184 | -0,759588698 | -0,765504029 | 0,005915331 | |
| 1570105_at | - | -0,759588698 | -0,765504029 | 0,005915331 | |
| 201261_x_at | BGN | -0,759588698 | -0,765504029 | 0,005915331 | |
| 206313_at | HLA-DOA | -0,759588698 | -0,765504029 | 0,005915331 | |
| 210760_x_at | TRIP11 | -0,759588698 | -0,765504029 | 0,005915331 | |
| 211845_at | PVRL1 | -0,759588698 | -0,765504029 | 0,005915331 | |
| 221065_s_at | CHST8 | -0,759588698 | -0,765504029 | 0,005915331 | |
| 228076_s_at | NOL6 | -0,759588698 | -0,765504029 | 0,005915331 | |
| 231454_at | PLAC4 | -0,759588698 | -0,765504029 | 0,005915331 | |
| 238892_at | - | -0,759588698 | -0,765504029 | 0,005915331 | |
| 242567_at | ABTB1 | -0,759588698 | -0,765504029 | 0,005915331 | |
| 201225_s_at | SRRM1 | 5,323562354 | 5,317649548 | 0,005912806 | |
| 202717_s_at | CDC16 | 3,716780201 | 3,710868933 | 0,005911268 | |
| 1554483_at | TMEM37 | 1,578645857 | 1,572742379 | 0,005903478 | |
| 212281_s_at | TMEM97 | 4,779830653 | 4,773932724 | 0,00589793 | |
| 203206_at | FAM53B | 0,841135803 | 0,835239052 | 0,005896751 | |
| 225275_at | EDIL3 | 0,841135803 | 0,835239052 | 0,005896751 | |
| 235553_at | GAPVD1 | 0,841135803 | 0,835239052 | 0,005896751 | |
| 214452_at | BCAT1 | 0,255492549 | 0,249604176 | 0,005888373 | |
| 224835_at | GPCPD1 | 2,254767157 | 2,248887791 | 0,005879367 | |
| 209225_x_at | TNPO1 | 3,839317436 | 3,833443169 | 0,005874267 | |
| 213694_at | RSBN1 | 2,834621043 | 2,828804976 | 0,005816067 | |
| 219066_at | PPCDC | 2,834621043 | 2,828804976 | 0,005816067 | |
| 217865_at | RNF130 | 3,330897748 | 3,325159096 | 0,005738652 | |
| 201752_s_at | ADD3 | 3,634982121 | 3,629253073 | 0,005729048 | |
| 218145_at | TRIB3 | 2,827046023 | 2,821324225 | 0,005721798 | |
| 238704_at | - | 0,822971223 | 0,817300337 | 0,005670886 | |
| 211983_x_at | ACTG1 | 7,834102201 | 7,828432519 | 0,005669682 | |
| 225126_at | MRRF | 2,236584753 | 2,230931691 | 0,005653061 | |
| 222467_s_at | PPP6R3 | 3,988699475 | 3,983081173 | 0,005618302 | |
| 1559989_at | TPM4 | 0,228146222 | 0,222599095 | 0,005547127 | |
| 206823_at | L3MBTL1 | 0,228146222 | 0,222599095 | 0,005547127 | |
| 207131_x_at | GGT1 /// GGT2 // | 0,228146222 | 0,222599095 | 0,005547127 | |
| 209794_at | SRGAP3 | 0,228146222 | 0,222599095 | 0,005547127 | |
| 231181_at | - | 0,228146222 | 0,222599095 | 0,005547127 | |
| 201802_at | SLC29A1 | 1,549482847 | 1,543943227 | 0,005539619 | |

| | | | | | |
|---|---|---|---|---|---|
| 228006_at | PTEN | 1,549482847 | 1,543943227 | 0,005539619 | |
| 224804_s_at | FAM219B | 2,225103271 | 2,21959457 | 0,005508701 | |
| 223819_x_at | COMMD5 | 3,923419763 | 3,917937882 | 0,00548188 | |
| 222390_at | WAC | 5,073485277 | 5,068098141 | 0,005387136 | |
| 200851_s_at | IST1 | 4,858849409 | 4,853468768 | 0,005380641 | |
| 203977_at | TAZ | 0,209623097 | 0,204310755 | 0,005312342 | |
| 226798_at | BCL2L13 | 0,209623097 | 0,204310755 | 0,005312342 | |
| 228298_at | PCED1B | 0,209623097 | 0,204310755 | 0,005312342 | |
| 239256_at | - | 0,209623097 | 0,204310755 | 0,005312342 | |
| 212473_s_at | MICAL2 | 6,040658973 | 6,035370383 | 0,00528859 | |
| 225215_s_at | MTRF1L | 3,792633459 | 3,787346121 | 0,005287338 | |
| 211913_s_at | MERTK | 0,792179257 | 0,786897721 | 0,005281535 | |
| 226713_at | CCDC50 | 0,792179257 | 0,786897721 | 0,005281535 | |
| 212173_at | AK2 | 2,206540699 | 2,201267805 | 0,005272895 | |
| 226541_at | FBXO30 | 3,96128107 | 3,956010056 | 0,005271014 | |
| 1562553_at | - | -3,342483913 | -3,347710726 | 0,005226813 | |
| 207090_x_at | ZFP30 | 2,371846398 | 2,366632651 | 0,005213747 | |
| 218602_s_at | HAUS6 | 2,201862497 | 2,196649505 | 0,005212992 | |
| 221851_at | DCAF15 | 1,523472489 | 1,518263577 | 0,005208912 | |
| 200880_at | DNAJA1 | 5,328667557 | 5,323486373 | 0,005181185 | |
| 225391_at | LOC93622 | 3,102740237 | 3,09760452 | 0,005135717 | |
| 208912_s_at | CNP | 3,779349411 | 3,77423254 | 0,005116871 | |
| 229028_s_at | ARL17A /// ARL1 | 1,512180019 | 1,507116528 | 0,005063491 | |
| 214771_x_at | MPRIP | 2,889473182 | 2,884425449 | 0,005047733 | |
| 213061_s_at | NTAN1 | 2,182996494 | 2,17802703 | 0,004969464 | |
| 213117_at | KLHL9 | 2,182996494 | 2,17802703 | 0,004969464 | |
| 213822_s_at | UBE3B | 1,990274687 | 1,98530622 | 0,004968467 | |
| 235985_at | - | 1,504602299 | 1,499637023 | 0,004965276 | |
| 220984_s_at | SLCO5A1 | 0,181384709 | 0,176436073 | 0,004948636 | |
| 233528_s_at | GATSL3 | 0,181384709 | 0,176436073 | 0,004948636 | |
| 234026_at | LOC100287177 | 0,181384709 | 0,176436073 | 0,004948636 | |
| 212449_s_at | LYPLA1 | 5,764887454 | 5,759957937 | 0,004929517 | |
| 202584_at | NFX1 | 1,764611101 | 1,759685182 | 0,004925919 | |
| 1562892_at | CHCHD5 | -0,85085127 | -0,855751026 | 0,004899756 | |
| 207536_s_at | TNFRSF9 | -0,85085127 | -0,855751026 | 0,004899756 | |
| 211165_x_at | EPHB2 | -0,85085127 | -0,855751026 | 0,004899756 | |
| 216285_at | DGCR14 /// TSSK | -0,85085127 | -0,855751026 | 0,004899756 | |
| 229995_at | SYCN | -0,85085127 | -0,855751026 | 0,004899756 | |
| 230745_s_at | TOX3 | -0,85085127 | -0,855751026 | 0,004899756 | |
| 231691_at | C3P1 | -0,85085127 | -0,855751026 | 0,004899756 | |
| 232153_at | SPEG | -0,85085127 | -0,855751026 | 0,004899756 | |
| 236127_at | - | -0,85085127 | -0,855751026 | 0,004899756 | |
| 240323_at | - | -0,85085127 | -0,855751026 | 0,004899756 | |
| 240909_at | - | -0,85085127 | -0,855751026 | 0,004899756 | |
| 242553_at | ABCC3 | -0,85085127 | -0,855751026 | 0,004899756 | |
| 243340_at | - | -0,85085127 | -0,855751026 | 0,004899756 | |
| 1552528_at | C21orf67 | 0,760715727 | 0,755840599 | 0,004875127 | |
| 233924_s_at | EXOC6 | 2,497416546 | 2,492544876 | 0,00487167 | |
| 1552477_a_at | IRF6 | 2,874852734 | 2,869995113 | 0,004857621 | |
| 218138_at | MKKS | 2,874852734 | 2,869995113 | 0,004857621 | |
| 232760_at | TEX15 | 2,874852734 | 2,869995113 | 0,004857621 | |
| 202396_at | TCERG1 | 4,817780426 | 4,812929194 | 0,004851232 | |
| 210831_s_at | PTGER3 | 1,493160564 | 1,488344553 | 0,004816011 | |
| 200634_at | PFN1 | 6,737796535 | 6,733023681 | 0,004772854 | |
| 200793_s_at | ACO2 | 3,072307152 | 3,067567462 | 0,00473969 | |
| 208787_at | MRPL3 | 6,373978588 | 6,369250622 | 0,004727966 | |

| | | | | | |
|---|---|---|---|---|---|
| 228357_at | UNK | 3,742778722 | 3,738139229 | 0,004639493 | |
| 202483_s_at | RANBP1 | 5,7855532 | 5,780935126 | 0,004618074 | |
| 214271_x_at | RPL12 | 6,925246493 | 6,920660727 | 0,004585766 | |
| 221478_at | BNIP3L | 5,08337493 | 5,078804007 | 0,004570923 | |
| 203293_s_at | LMAN1 | 1,473887017 | 1,469325098 | 0,004561919 | |
| 224486_s_at | C15orf41 | 3,056846434 | 3,052311115 | 0,004535319 | |
| 219004_s_at | MIS18A | 3,904829675 | 3,900294538 | 0,004535137 | |
| 219061_s_at | LAGE3 | 3,734704399 | 3,730171924 | 0,004532476 | |
| 211504_x_at | ROCK2 | 0,728550685 | 0,724100169 | 0,004450516 | |
| 226578_s_at | DUSP1 | 0,728550685 | 0,724100169 | 0,004450516 | |
| 212455_at | YTHDC1 | 4,757289776 | 4,752852722 | 0,004437055 | |
| 203050_at | TP53BP1 | 3,786397376 | 3,781960839 | 0,004436537 | |
| 218768_at | NUP107 | 4,974790208 | 4,970362234 | 0,004427974 | |
| 225731_at | ANKRD50 | 3,046446467 | 3,042049843 | 0,004396624 | |
| 1553262_a_at | UTS2R | 1,722769813 | 1,718396606 | 0,004373208 | |
| 213356_x_at | HNRNPA1 /// HN | 7,533819819 | 7,529483243 | 0,004336576 | |
| 1562409_s_at | - | 0,133056859 | 0,128747141 | 0,004309718 | |
| 219330_at | VANGL1 | 0,133056859 | 0,128747141 | 0,004309718 | |
| 220095_at | CNTLN | 0,133056859 | 0,128747141 | 0,004309718 | |
| 222050_at | - | 0,133056859 | 0,128747141 | 0,004309718 | |
| 243579_at | MSI2 | 0,133056859 | 0,128747141 | 0,004309718 | |
| 222444_at | ARMCX3 | 1,454352496 | 1,450051551 | 0,004300946 | |
| 221063_x_at | RNF123 | 1,446464021 | 1,442269454 | 0,004194567 | |
| 213793_s_at | HOMER1 | 3,123915073 | 3,119728903 | 0,00418617 | |
| 221727_at | SUB1 | 3,582860873 | 3,578681277 | 0,004179597 | |
| 219918_s_at | ASPM | 4,208686884 | 4,204537151 | 0,004149733 | |
| 1561243_at | TMEM105 | 1,119359127 | 1,115234684 | 0,004124443 | |
| 218148_at | CENPT | 1,119359127 | 1,115234684 | 0,004124443 | |
| 241724_x_at | - | 1,119359127 | 1,115234684 | 0,004124443 | |
| 204234_s_at | ZNF195 | 1,926486359 | 1,922365006 | 0,004121353 | |
| 215215_s_at | LOC81691 | 1,926486359 | 1,922365006 | 0,004121353 | |
| 208296_x_at | TNFAIP8 | 4,6074892 | 4,603415342 | 0,004073858 | |
| 214062_x_at | NFKBIB | 2,28383338 | 2,27978304 | 0,00405034 | |
| 220086_at | IKZF5 | 1,434549838 | 1,43051703 | 0,004032808 | |
| 221100_at | C6orf15 | 0,695652136 | 0,691645685 | 0,004006451 | |
| 218159_at | DDRGK1 | 3,017453847 | 3,013449121 | 0,004004726 | |
| 225074_at | RAB2B | 3,749205801 | 3,745272377 | 0,003933424 | |
| 202693_s_at | STK17A | 3,495722611 | 3,491815843 | 0,003906767 | |
| 215884_s_at | UBQLN2 | 3,95851035 | 3,954643074 | 0,003867276 | |
| 1554044_a_at | MRAP | -0,946847173 | -0,950686014 | 0,003838841 | |
| 1560476_at | - | -0,946847173 | -0,950686014 | 0,003838841 | |
| 214732_at | SP1 | -0,946847173 | -0,950686014 | 0,003838841 | |
| 228094_at | AMICA1 | -0,946847173 | -0,950686014 | 0,003838841 | |
| 236547_at | - | -0,946847173 | -0,950686014 | 0,003838841 | |
| 238610_s_at | - | -0,946847173 | -0,950686014 | 0,003838841 | |
| 242944_at | FAM83A | -0,946847173 | -0,950686014 | 0,003838841 | |
| 244229_at | PARVG | -0,946847173 | -0,950686014 | 0,003838841 | |
| 244466_at | ZNF544 | -0,946847173 | -0,950686014 | 0,003838841 | |
| 244729_at | - | -0,946847173 | -0,950686014 | 0,003838841 | |
| 227144_at | KIAA0930 | 0,67554678 | 0,67181667 | 0,00373011 | |
| 231002_s_at | - | 0,67554678 | 0,67181667 | 0,00373011 | |
| 209001_s_at | ANAPC13 | 4,579810951 | 4,576117222 | 0,003693729 | |
| 214274_s_at | ACAA1 | 3,406034785 | 3,402394519 | 0,003640266 | |
| 205601_s_at | HOXB5 | 0,083054335 | 0,079428135 | 0,0036262 | |
| 231005_at | - | 0,083054335 | 0,079428135 | 0,0036262 | |
| 35201_at | HNRNPL | 5,137966674 | 5,134346191 | 0,003620483 | |

| | | | | | |
|---|---|---|---|---|---|
| 202094_at | BIRC5 | 3,082523011 | 3,078904771 | 0,00361824 | |
| 220015_at | CASZ1 | 1,40228911 | 1,398700978 | 0,003588131 | |
| 223490_s_at | EXOSC3 | 3,780134221 | 3,77655538 | 0,003578841 | |
| 208361_s_at | POLR3D | 2,779171778 | 2,775606361 | 0,003565417 | |
| 209204_at | LMO4 | 0,661985843 | 0,658444279 | 0,003541563 | |
| 228330_at | ZUFSP | 3,074867907 | 3,071356473 | 0,003511434 | |
| 45572_s_at | GGA1 | 3,979503898 | 3,976024028 | 0,00347987 | |
| 212886_at | CCDC69 | 1,394109935 | 1,390636111 | 0,003473824 | |
| 201994_at | MORF4L2 | 6,317929234 | 6,314485986 | 0,003443248 | |
| 208863_s_at | SRSF1 | 5,317628408 | 5,314189382 | 0,003439026 | |
| 223583_at | TNFAIP8L2 | 2,069451339 | 2,066015817 | 0,003435522 | |
| 216396_s_at | EI24 | 4,107892039 | 4,104540277 | 0,003351762 | |
| 33768_at | DMWD | 1,761435272 | 1,758118818 | 0,003316454 | |
| 231806_s_at | STK36 | 1,381753566 | 1,378453643 | 0,003299923 | |
| 228650_at | - | 2,380152406 | 2,376875125 | 0,003277281 | |
| 204770_at | TAP2 | 0,641402394 | 0,638150376 | 0,003252018 | |
| 213215_at | AP3S2 /// C15orf: | 0,641402394 | 0,638150376 | 0,003252018 | |
| 212602_at | WDFY3 | 1,373456798 | 1,370274469 | 0,003182329 | |
| 224207_x_at | MMP28 | 1,373456798 | 1,370274469 | 0,003182329 | |
| 225559_at | C3orf19 | 1,373456798 | 1,370274469 | 0,003182329 | |
| 202778_s_at | ZMYM2 | 3,51093129 | 3,507749374 | 0,003181916 | |
| 229323_at | LOC387723 | 0,627515113 | 0,624460759 | 0,003054354 | |
| 219798_s_at | MEPCE | 3,434229569 | 3,431184377 | 0,003045192 | |
| 224596_at | SLC44A1 | 2,846658948 | 2,843651104 | 0,003007844 | |
| 227689_at | ZNF227 | 2,846658948 | 2,843651104 | 0,003007844 | |
| 201395_at | RBM5 | 4,08266696 | 4,079673902 | 0,002993059 | |
| 212202_s_at | TMEM87A | 2,945117939 | 2,942125533 | 0,002992406 | |
| 219578_s_at | CPEB1 | 1,844820248 | 1,841838775 | 0,002981473 | |
| 214662_at | WDR43 | 3,354274239 | 3,351366359 | 0,00290788 | |
| 229146_at | C7orf31 | 0,03125738 | 0,028364014 | 0,002893366 | |
| 233867_at | - | 0,03125738 | 0,028364014 | 0,002893366 | |
| 218107_at | WDR26 | 4,519522578 | 4,516682156 | 0,002840422 | |
| 215806_x_at | TARP /// TRGC2 | 3,348994562 | 3,346162854 | 0,002831709 | |
| 200833_s_at | LOC100506390 / | 7,312292598 | 7,309496854 | 0,002795744 | |
| 224878_at | UBFD1 | 3,608715137 | 3,605931378 | 0,002783759 | |
| 209235_at | CLCN7 | 1,829737795 | 1,826973888 | 0,002763907 | |
| 1557626_at | - | -1,047650486 | -1,050406615 | 0,002756129 | |
| 1557850_at | INHBA-AS1 | -1,047650486 | -1,050406615 | 0,002756129 | |
| 1561886_a_at | - | -1,047650486 | -1,050406615 | 0,002756129 | |
| 1562289_at | - | -1,047650486 | -1,050406615 | 0,002756129 | |
| 1570568_at | LOC100130502 | -1,047650486 | -1,050406615 | 0,002756129 | |
| 205971_s_at | CTRB1 /// CTRB2 | -1,047650486 | -1,050406615 | 0,002756129 | |
| 207907_at | TNFSF14 | -1,047650486 | -1,050406615 | 0,002756129 | |
| 214511_x_at | FCGR1B | -1,047650486 | -1,050406615 | 0,002756129 | |
| 214664_at | PAICS | -1,047650486 | -1,050406615 | 0,002756129 | |
| 216936_at | - | -1,047650486 | -1,050406615 | 0,002756129 | |
| 222296_at | - | -1,047650486 | -1,050406615 | 0,002756129 | |
| 237207_at | - | -1,047650486 | -1,050406615 | 0,002756129 | |
| 238839_at | OTX1 | -1,047650486 | -1,050406615 | 0,002756129 | |
| 241684_at | - | -1,047650486 | -1,050406615 | 0,002756129 | |
| 243051_at | CNIH4 | -1,047650486 | -1,050406615 | 0,002756129 | |
| 244051_at | - | -1,047650486 | -1,050406615 | 0,002756129 | |
| 213682_at | NUP50 | 4,692484877 | 4,689754684 | 0,002730193 | |
| 226486_at | MTERFD2 | 1,339784194 | 1,337086025 | 0,002698169 | |
| 201912_s_at | GSPT1 | 4,74085559 | 4,738229113 | 0,002626476 | |
| 221430_s_at | RNF146 | 1,593009234 | 1,590454215 | 0,002555019 | |

| | | | | | |
|---|---|---|---|---|---|
| 201219_at | CTBP2 | 0,592200562 | 0,589657387 | 0,002543175 | |
| 207440_at | SLC35A2 | 0,592200562 | 0,589657387 | 0,002543175 | |
| 213248_at | LOC730101 | 0,592200562 | 0,589657387 | 0,002543175 | |
| 209141_at | UBE2G1 | 4,248360607 | 4,245917359 | 0,002443248 | |
| 203109_at | UBE2M | 4,906708939 | 4,904274741 | 0,002434197 | |
| 219448_at | TMEM70 | 1,318332599 | 1,315948728 | 0,002383871 | |
| 209020_at | C20orf111 | 2,31665944 | 2,314300277 | 0,002359162 | |
| 217976_s_at | DYNC1LI1 | 2,310153708 | 2,307890888 | 0,00226282 | |
| 218122_s_at | SENP2 | 2,310153708 | 2,307890888 | 0,00226282 | |
| 202505_at | SNRPB2 | 6,142738335 | 6,140480048 | 0,002258287 | |
| 219027_s_at | MYO9A | 1,987559146 | 1,98530622 | 0,002252926 | |
| 1558236_at | - | 0,57058931 | 0,568365095 | 0,002224215 | |
| 205938_at | PPM1E | 0,57058931 | 0,568365095 | 0,002224215 | |
| 206044_s_at | BRAF | 0,57058931 | 0,568365095 | 0,002224215 | |
| 227755_at | - | 0,57058931 | 0,568365095 | 0,002224215 | |
| 243707_at | - | 0,57058931 | 0,568365095 | 0,002224215 | |
| 204510_at | CDC7 | 3,148435161 | 3,146317015 | 0,002118146 | |
| 211166_at | FAM153A | -0,022466681 | -0,024572586 | 0,002105906 | |
| 243731_at | - | -0,022466681 | -0,024572586 | 0,002105906 | |
| 243976_at | - | -0,022466681 | -0,024572586 | 0,002105906 | |
| 210053_at | TAF5 | 3,498586477 | 3,496520471 | 0,002066007 | |
| 222729_at | FBXW7 | 1,556829052 | 1,55481039 | 0,002018661 | |
| 226166_x_at | STK36 | 1,556829052 | 1,55481039 | 0,002018661 | |
| 201229_s_at | ARIH2 | 2,292660284 | 2,290658654 | 0,00200163 | |
| 225455_at | TADA1 | 2,55541096 | 2,553413592 | 0,001997368 | |
| 228334_x_at | CEP44 | 2,55541096 | 2,553413592 | 0,001997368 | |
| 65770_at | RHOT2 | 2,724770722 | 2,722781848 | 0,001988874 | |
| 226610_at | CENPV | 3,875753437 | 3,873779903 | 0,001973534 | |
| 201490_s_at | PPIF | 3,612245157 | 3,61027875 | 0,001966406 | |
| 222400_s_at | ADI1 | 5,522158161 | 5,52020409 | 0,001954072 | |
| 200933_x_at | RPS4X | 7,793284449 | 7,791342925 | 0,001941524 | |
| 225229_at | AFF4 | 3,133773173 | 3,131875186 | 0,001897988 | |
| 201508_at | IGFBP4 | 0,962260738 | 0,960386719 | 0,001874019 | |
| 226605_at | DGKQ | 0,962260738 | 0,960386719 | 0,001874019 | |
| 235472_at | FUT10 | 0,962260738 | 0,960386719 | 0,001874019 | |
| 239300_at | PIK3C3 | 0,962260738 | 0,960386719 | 0,001874019 | |
| 234295_at | DBR1 | 2,420979937 | 2,419116683 | 0,001863253 | |
| 201253_s_at | CDIPT | 4,224629877 | 4,222822694 | 0,001807183 | |
| 202361_at | SEC24C | 2,864529541 | 2,862725455 | 0,001804086 | |
| 1556613_s_at | DPY19L4 | 2,658027143 | 2,656223838 | 0,001803305 | |
| 209132_s_at | COMMD4 | 3,80887607 | 3,807174423 | 0,001701647 | |
| 1555653_at | - | 6,298888458 | 6,297198001 | 0,001690458 | |
| 208406_s_at | GRAP2 | 0,533835206 | 0,532164371 | 0,001670836 | |
| 78047_s_at | - | 2,034358528 | 2,032717854 | 0,001640674 | |
| 242617_at | TMED8 | 2,531373922 | 2,529740643 | 0,001633279 | |
| 218545_at | CCDC91 | 2,261526912 | 2,259997914 | 0,001528998 | |
| 209994_s_at | ABCB1 /// ABCB4 | 1,261018186 | 1,259496994 | 0,001521191 | |
| 227249_at | NDE1 | 3,024094427 | 3,022611001 | 0,001483426 | |
| 208679_s_at | ARPC2 | 5,900447459 | 5,898994315 | 0,001453144 | |
| 203950_s_at | CLCN6 | 0,934330293 | 0,932882274 | 0,001448019 | |
| 235126_at | FLVCR1-AS1 | 0,934330293 | 0,932882274 | 0,001448019 | |
| 213558_at | PCLO | 0,518867317 | 0,51742585 | 0,001441467 | |
| 239793_at | - | 0,518867317 | 0,51742585 | 0,001441467 | |
| 214937_x_at | PCM1 | 5,101070055 | 5,099671136 | 0,001398919 | |
| 218567_x_at | DPP3 | 2,929639344 | 2,928263672 | 0,001375672 | |
| 213310_at | EIF2C2 /// LOC28 | 1,735773781 | 1,73441697 | 0,001356811 | |

| | | | | | |
|---|---|---|---|---|---|
| 212563_at | BOP1 | 4,092178125 | 4,090916839 | 0,001261286 | |
| 1557700_at | POLH | -0,078265071 | -0,079522948 | 0,001257877 | |
| 1563318_s_at | MAGIX | -0,078265071 | -0,079522948 | 0,001257877 | |
| 1565697_at | - | -0,078265071 | -0,079522948 | 0,001257877 | |
| 1566700_at | VRK3 | -0,078265071 | -0,079522948 | 0,001257877 | |
| 222282_at | - | -0,078265071 | -0,079522948 | 0,001257877 | |
| 229141_at | SFT2D3 /// WDR | -0,078265071 | -0,079522948 | 0,001257877 | |
| 239911_at | ONECUT2 | -0,078265071 | -0,079522948 | 0,001257877 | |
| 226843_s_at | PAPD5 | 1,917954284 | 1,916759838 | 0,001194447 | |
| 201460_at | MAPKAPK2 | 3,722507547 | 3,721356767 | 0,00115078 | |
| 217954_s_at | PHF3 | 3,999522577 | 3,9984201 | 0,001102477 | |
| 203051_at | BAHD1 | 1,90937145 | 1,908311039 | 0,001060411 | |
| 213132_s_at | MCAT | 2,814842651 | 2,813804482 | 0,001038169 | |
| 213509_x_at | CES2 | 2,227406889 | 2,226407528 | 0,000999361 | |
| 231370_at | - | 2,227406889 | 2,226407528 | 0,000999361 | |
| 227547_at | - | 2,81177561 | 2,810785576 | 0,000990034 | |
| 208579_x_at | H2BFS /// HIST1I | 7,376849629 | 7,375886965 | 0,000962664 | |
| 210681_s_at | USP15 | 4,736009955 | 4,735047668 | 0,000962287 | |
| 213452_at | ZNF184 | 2,710020346 | 2,709065209 | 0,000955137 | |
| 231990_at | USP15 | 1,709647566 | 1,708698295 | 0,000949271 | |
| 209069_s_at | H3F3A /// H3F3B | 6,832957376 | 6,832009248 | 0,000948128 | |
| 1554027_a_at | SLC4A4 | -2,779459409 | -2,780313924 | 0,000854516 | |
| 1554418_s_at | SPOCK3 | -2,779459409 | -2,780313924 | 0,000854516 | |
| 1556805_at | - | -2,779459409 | -2,780313924 | 0,000854516 | |
| 1557475_at | LINC00507 /// LC | -2,779459409 | -2,780313924 | 0,000854516 | |
| 1557863_at | - | -2,779459409 | -2,780313924 | 0,000854516 | |
| 1559470_at | D21S2088E | -2,779459409 | -2,780313924 | 0,000854516 | |
| 1560657_at | TIE1 | -2,779459409 | -2,780313924 | 0,000854516 | |
| 1562107_at | - | -2,779459409 | -2,780313924 | 0,000854516 | |
| 1562623_at | LOC146513 | -2,779459409 | -2,780313924 | 0,000854516 | |
| 1565924_a_at | - | -2,779459409 | -2,780313924 | 0,000854516 | |
| 1568896_at | STXBP5L | -2,779459409 | -2,780313924 | 0,000854516 | |
| 1570268_at | - | -2,779459409 | -2,780313924 | 0,000854516 | |
| 213382_at | MST1 /// MST1P2 | -2,779459409 | -2,780313924 | 0,000854516 | |
| 215002_at | 61E3.4 /// LOC10 | -2,779459409 | -2,780313924 | 0,000854516 | |
| 217460_at | TNP2 | -2,779459409 | -2,780313924 | 0,000854516 | |
| 232715_at | - | -2,779459409 | -2,780313924 | 0,000854516 | |
| 234038_at | - | -2,779459409 | -2,780313924 | 0,000854516 | |
| 234185_at | - | -2,779459409 | -2,780313924 | 0,000854516 | |
| 236793_at | - | -2,779459409 | -2,780313924 | 0,000854516 | |
| 237066_at | - | -2,779459409 | -2,780313924 | 0,000854516 | |
| 240361_at | LOC100507603 | -2,779459409 | -2,780313924 | 0,000854516 | |
| 240706_at | - | -2,779459409 | -2,780313924 | 0,000854516 | |
| 240907_at | - | -2,779459409 | -2,780313924 | 0,000854516 | |
| 241168_at | - | -2,779459409 | -2,780313924 | 0,000854516 | |
| 241485_at | MYT1L | -2,779459409 | -2,780313924 | 0,000854516 | |
| 242127_at | - | -2,779459409 | -2,780313924 | 0,000854516 | |
| 244390_at | - | -2,779459409 | -2,780313924 | 0,000854516 | |
| 242417_at | LOC283278 | 0,480753764 | 0,479907041 | 0,000846723 | |
| 212039_x_at | RNU86 /// RPL3 / | 7,662876265 | 7,662046928 | 0,000829337 | |
| 227703_s_at | SYTL4 | 2,981056773 | 2,980244813 | 0,00081196 | |
| 48808_at | DHFR | 3,700289946 | 3,69948842 | 0,000801526 | |
| 202727_s_at | IFNGR1 | 4,249494456 | 4,248708657 | 0,000785799 | |
| 218333_at | DERL2 | 3,977794563 | 3,977034308 | 0,000760255 | |
| 227712_at | LYRM2 | 3,211467103 | 3,210719429 | 0,000747674 | |
| 202303_x_at | SMARCA5 | 3,794188277 | 3,793476213 | 0,000712064 | |

| | | | | | |
|---|---|---|---|---|---|
| 50314_i_at | C20orf27 | 2,586251598 | 2,585563171 | 0,000688427 | |
| 207030_s_at | CSRP2 | 1,69307511 | 1,692388143 | 0,000686968 | |
| 207153_s_at | GLMN | 3,052955227 | 3,052311115 | 0,000644112 | |
| 200040_at | KHDRBS1 | 5,825447133 | 5,824804578 | 0,000642555 | |
| 218256_s_at | NUP54 | 5,071563295 | 5,070939787 | 0,000623508 | |
| 220173_at | C14orf45 | 1,201332287 | 1,200746069 | 0,000586217 | |
| 208778_s_at | SNORA29 /// TCI | 6,510350114 | 6,50979668 | 0,000553435 | |
| 226555_at | INO80D | 1,462198073 | 1,461646549 | 0,000551524 | |
| 204349_at | MED7 | 2,577249059 | 2,576704359 | 0,000544699 | |
| 201714_at | TUBG1 | 3,828735867 | 3,828227545 | 0,000508322 | |
| 207618_s_at | BCS1L | 2,960432227 | 2,959949103 | 0,000483125 | |
| 227597_at | DIS3L2 | 0,457392764 | 0,456918297 | 0,000474467 | |
| 208774_at | CSNK1D | 3,872077355 | 3,871608557 | 0,000468797 | |
| 233841_s_at | SUDS3 | 2,678376417 | 2,677924589 | 0,000451828 | |
| 221458_at | HTR1F | 1,191926657 | 1,191491281 | 0,000435376 | |
| 234941_s_at | GPHN | 1,191926657 | 1,191491281 | 0,000435376 | |
| 209421_at | MSH2 | 4,46638963 | 4,466010984 | 0,000378646 | |
| 1560671_at | KIAA1652 | -0,136301282 | -0,136643983 | 0,0003427 | |
| 206152_at | AGAP2 | -0,136301282 | -0,136643983 | 0,0003427 | |
| 206862_at | ZNF254 | -0,136301282 | -0,136643983 | 0,0003427 | |
| 206869_at | CHAD | -0,136301282 | -0,136643983 | 0,0003427 | |
| 222957_at | NEU4 | -0,136301282 | -0,136643983 | 0,0003427 | |
| 224923_at | TTC7A | -0,136301282 | -0,136643983 | 0,0003427 | |
| 239220_at | - | -0,136301282 | -0,136643983 | 0,0003427 | |
| 202716_at | PTPN1 | 3,508091848 | 3,507749374 | 0,000342475 | |
| 226116_at | DFFA | 3,61576656 | 3,615478364 | 0,000288196 | |
| 228500_at | THAP8 | 1,859746653 | 1,859476797 | 0,000269856 | |
| 212274_at | LPIN1 | 1,666156729 | 1,665902186 | 0,000254543 | |
| 223947_s_at | MED23 | 2,559073676 | 2,558821855 | 0,000251822 | |
| 202951_at | STK38 | 4,834877439 | 4,834642428 | 0,000235011 | |
| 213977_s_at | CIZ1 | 1,442503549 | 1,442269454 | 0,000234095 | |
| 219743_at | HEY2 | 1,177702228 | 1,177496821 | 0,000205407 | |
| 202201_at | BLVRB | 5,280393903 | 5,280215235 | 0,000178668 | |
| 203484_at | SEC61G | 5,854054399 | 5,853876947 | 0,000177452 | |
| 201721_s_at | LAPTM5 | 6,710575593 | 6,710421896 | 0,000153697 | |
| 223266_at | STRADB | 2,758612869 | 2,758473378 | 0,000139491 | |
| 200800_s_at | HSPA1A /// HSPA | 5,406207333 | 5,406069533 | 0,0001378 | |
| 223383_at | ZNRF1 | 2,171078719 | 2,170981159 | 9,75601E-05 | |
| 233878_s_at | XRN2 | 4,089014699 | 4,08904909 | -3,4391E-05 | |
| 201558_at | RAE1 | 5,28177997 | 5,281851323 | -7,1352E-05 | |
| 225036_at | TOMM5 | 5,733019003 | 5,733100797 | -8,1794E-05 | |
| 244519_at | ASXL1 | 3,691139316 | 3,691304166 | -0,00016485 | |
| 210537_s_at | TADA2A | 0,417596971 | 0,417770464 | -0,00017349 | |
| 221418_s_at | MED16 | 0,417596971 | 0,417770464 | -0,00017349 | |
| 213890_x_at | RPS16 | 8,173815646 | 8,174003891 | -0,00018824 | |
| 222744_s_at | TMLHE | 1,000476627 | 1,000684521 | -0,00020789 | |
| 205292_s_at | HNRNPA2B1 | 6,75331653 | 6,753558289 | -0,00024176 | |
| 222237_s_at | ZFP112 /// ZNF28 | 1,631786706 | 1,632095957 | -0,00030925 | |
| 238034_at | CANX | 2,520143371 | 2,520531219 | -0,00038785 | |
| 223301_s_at | CCDC82 | 2,726400382 | 2,726791458 | -0,00039108 | |
| 211423_s_at | SC5DL | 2,986507217 | 2,986947093 | -0,00043988 | |
| 216306_x_at | PTBP1 | 6,033929877 | 6,034400153 | -0,00047028 | |
| 213109_at | TNIK | 2,134723057 | 2,135226207 | -0,00050315 | |
| 221517_s_at | MED17 | 3,455019843 | 3,455550298 | -0,00053046 | |
| 201890_at | RRM2 | 6,024025496 | 6,024580369 | -0,00055487 | |
| 209194_at | CETN2 | 3,97847854 | 3,979052747 | -0,00057421 | |

| | | | | | |
|---|---|---|---|---|---|
| 204711_at | KIAA0753 | 2,392522438 | 2,39311332 | -0,00059088 | |
| 36019_at | STK19 | 2,614687931 | 2,615282623 | -0,00059469 | |
| 220330_s_at | SAMSN1 | 2,506929603 | 2,507538456 | -0,00060885 | |
| 1566549_at | - | -0,196756757 | -0,19610998 | -0,00064678 | |
| 219316_s_at | FLVCR2 | -0,196756757 | -0,19610998 | -0,00064678 | |
| 243786_at | ZDHHC20 | -0,196756757 | -0,19610998 | -0,00064678 | |
| 201854_s_at | ATMIN | 2,890927111 | 2,891586849 | -0,00065974 | |
| 1555704_at | CMTM3 | -1,424023738 | -1,423348341 | -0,0006754 | |
| 1565759_at | RPL13 | -1,424023738 | -1,423348341 | -0,0006754 | |
| 203461_at | CHD2 | -1,424023738 | -1,423348341 | -0,0006754 | |
| 207418_s_at | DDO | -1,424023738 | -1,423348341 | -0,0006754 | |
| 208559_at | PDX1 | -1,424023738 | -1,423348341 | -0,0006754 | |
| 210042_s_at | CTSZ | -1,424023738 | -1,423348341 | -0,0006754 | |
| 210889_s_at | FCGR2B | -1,424023738 | -1,423348341 | -0,0006754 | |
| 215503_at | SPINT3 | -1,424023738 | -1,423348341 | -0,0006754 | |
| 216581_at | - | -1,424023738 | -1,423348341 | -0,0006754 | |
| 220010_at | KCNE1L | -1,424023738 | -1,423348341 | -0,0006754 | |
| 228580_at | HTRA3 | -1,424023738 | -1,423348341 | -0,0006754 | |
| 232331_at | - | -1,424023738 | -1,423348341 | -0,0006754 | |
| 232944_at | - | -1,424023738 | -1,423348341 | -0,0006754 | |
| 235293_at | - | -1,424023738 | -1,423348341 | -0,0006754 | |
| 236820_at | - | -1,424023738 | -1,423348341 | -0,0006754 | |
| 237409_at | - | -1,424023738 | -1,423348341 | -0,0006754 | |
| 243101_x_at | - | -1,424023738 | -1,423348341 | -0,0006754 | |
| 224585_x_at | ACTG1 | 7,976656474 | 7,977345414 | -0,00068894 | |
| 224078_at | HIATL2 | 2,122398022 | 2,123108223 | -0,0007102 | |
| 44783_s_at | HEY1 | 7,205446641 | 7,206159621 | -0,00071298 | |
| 208719_s_at | DDX17 | 1,607231019 | 1,60795124 | -0,00072022 | |
| 221311_x_at | LYRM2 | 3,25727902 | 3,258033502 | -0,00075448 | |
| 235175_at | GBP4 | 1,795990708 | 1,796776588 | -0,00078588 | |
| 204775_at | CHAF1B | 1,965648824 | 1,966439221 | -0,0007904 | |
| 228512_at | PTCD3 | 2,491678455 | 2,492544876 | -0,00086642 | |
| 211986_at | AHNAK | 2,37600538 | 2,376875125 | -0,00086975 | |
| 223221_at | SCO1 | 3,375201825 | 3,376085218 | -0,00088339 | |
| 201329_s_at | ETS2 | 2,790121796 | 2,791007185 | -0,00088539 | |
| 220840_s_at | C1orf112 | 1,789769151 | 1,790660532 | -0,00089138 | |
| 1554033_at | FRMD4A | -2,504600601 | -2,503677622 | -0,00092298 | |
| 1555430_at | TSTD2 | -2,504600601 | -2,503677622 | -0,00092298 | |
| 1557050_at | HOTAIRM1 | -2,504600601 | -2,503677622 | -0,00092298 | |
| 1557560_at | LOC100506496 | -2,504600601 | -2,503677622 | -0,00092298 | |
| 1560284_at | - | -2,504600601 | -2,503677622 | -0,00092298 | |
| 1560728_at | - | -2,504600601 | -2,503677622 | -0,00092298 | |
| 1562694_at | C3orf65 | -2,504600601 | -2,503677622 | -0,00092298 | |
| 1563154_at | - | -2,504600601 | -2,503677622 | -0,00092298 | |
| 1566046_at | - | -2,504600601 | -2,503677622 | -0,00092298 | |
| 1568930_at | EFCAB1 | -2,504600601 | -2,503677622 | -0,00092298 | |
| 206449_s_at | MASP1 | -2,504600601 | -2,503677622 | -0,00092298 | |
| 207935_s_at | KRT13 | -2,504600601 | -2,503677622 | -0,00092298 | |
| 207952_at | IL5 | -2,504600601 | -2,503677622 | -0,00092298 | |
| 210435_at | ARL17A /// ARL1 | -2,504600601 | -2,503677622 | -0,00092298 | |
| 211298_s_at | ALB | -2,504600601 | -2,503677622 | -0,00092298 | |
| 216636_at | - | -2,504600601 | -2,503677622 | -0,00092298 | |
| 216898_s_at | COL4A3 | -2,504600601 | -2,503677622 | -0,00092298 | |
| 220384_at | NME8 | -2,504600601 | -2,503677622 | -0,00092298 | |
| 220704_at | IKZF1 | -2,504600601 | -2,503677622 | -0,00092298 | |
| 224253_at | - | -2,504600601 | -2,503677622 | -0,00092298 | |

| | | | | | |
|---|---|---|---|---|---|
| 224275_at | GPR98 | -2,504600601 | -2,503677622 | -0,00092298 | |
| 233086_at | FAM209A | -2,504600601 | -2,503677622 | -0,00092298 | |
| 233305_at | NECAB1 | -2,504600601 | -2,503677622 | -0,00092298 | |
| 236894_at | L1TD1 | -2,504600601 | -2,503677622 | -0,00092298 | |
| 238294_at | - | -2,504600601 | -2,503677622 | -0,00092298 | |
| 238941_at | TRIM50 | -2,504600601 | -2,503677622 | -0,00092298 | |
| 239336_at | THBS1 | -2,504600601 | -2,503677622 | -0,00092298 | |
| 241079_at | - | -2,504600601 | -2,503677622 | -0,00092298 | |
| 242845_at | RUNX1T1 | -2,504600601 | -2,503677622 | -0,00092298 | |
| 244206_at | ZFAND4 | -2,504600601 | -2,503677622 | -0,00092298 | |
| 214212_x_at | FERMT2 | 1,369290453 | 1,370274469 | -0,00098402 | |
| 203350_at | AP1G1 | 3,032022986 | 3,033010968 | -0,00098798 | |
| 217763_s_at | RAB31 | 3,784051878 | 3,785040604 | -0,00098873 | |
| 207266_x_at | RBMS1 | 2,482063962 | 2,483094137 | -0,00103017 | |
| 201574_at | ETF1 | 5,084646091 | 5,085689237 | -0,00104315 | |
| 230296_at | C16orf52 | 2,102456302 | 2,103505212 | -0,00104891 | |
| 1560213_at | HCP5B | -0,221656139 | -0,220592223 | -0,00106392 | |
| 233526_at | - | -0,221656139 | -0,220592223 | -0,00106392 | |
| 202536_at | CHMP2B | 3,586454644 | 3,587527995 | -0,00107335 | |
| 221787_at | C6orf120 | 3,237986416 | 3,239069096 | -0,00108268 | |
| 201120_s_at | PGRMC1 | 3,907705327 | 3,908790275 | -0,00108495 | |
| 213947_s_at | NUP210 | 2,361396186 | 2,362515214 | -0,00111903 | |
| 218574_s_at | LMCD1 | 1,774096855 | 1,775255987 | -0,00115913 | |
| 228230_at | PRIC285 | 1,774096855 | 1,775255987 | -0,00115913 | |
| 224648_at | GPBP1 | 3,232262672 | 3,233443554 | -0,00118088 | |
| 212586_at | CAST | 4,05440026 | 4,05564712 | -0,00124686 | |
| 212591_at | ARID4B /// RBM3 | 4,382572276 | 4,383838717 | -0,00126644 | |
| 239007_at | ZNF616 | 0,767063567 | 0,768343791 | -0,00128022 | |
| 1562627_at | LOC100130078 | 0,351548291 | 0,352836757 | -0,00128847 | |
| 219156_at | SYNJ2BP /// SYN | 0,351548291 | 0,352836757 | -0,00128847 | |
| 221567_at | NOL3 | 0,351548291 | 0,352836757 | -0,00128847 | |
| 223408_s_at | - | 0,351548291 | 0,352836757 | -0,00128847 | |
| 232867_at | - | 0,351548291 | 0,352836757 | -0,00128847 | |
| 224624_at | LRRC8A | 4,318648014 | 4,319980141 | -0,00133213 | |
| 235013_at | SLC31A1 | 1,348276312 | 1,349621332 | -0,00134502 | |
| 89948_at | PCIF1 | 1,667853922 | 1,669239644 | -0,00138572 | |
| 233080_s_at | PRPF40A | 4,251193561 | 4,252607423 | -0,00141386 | |
| 1562621_at | - | -1,543331864 | -1,541908042 | -0,00142382 | |
| 1565535_x_at | - | -1,543331864 | -1,541908042 | -0,00142382 | |
| 205818_at | DBC1 | -1,543331864 | -1,541908042 | -0,00142382 | |
| 206423_at | ANGPTL7 | -1,543331864 | -1,541908042 | -0,00142382 | |
| 206978_at | CCR2 | -1,543331864 | -1,541908042 | -0,00142382 | |
| 217146_at | JRK | -1,543331864 | -1,541908042 | -0,00142382 | |
| 219190_s_at | EIF2C4 | -1,543331864 | -1,541908042 | -0,00142382 | |
| 230104_s_at | TPPP | -1,543331864 | -1,541908042 | -0,00142382 | |
| 238823_at | FMNL3 | -1,543331864 | -1,541908042 | -0,00142382 | |
| 241131_at | - | -1,543331864 | -1,541908042 | -0,00142382 | |
| 241267_at | EHD3 | -1,543331864 | -1,541908042 | -0,00142382 | |
| 241977_s_at | RAB3C | -1,543331864 | -1,541908042 | -0,00142382 | |
| 242315_at | - | -1,543331864 | -1,541908042 | -0,00142382 | |
| 244064_at | - | -1,543331864 | -1,541908042 | -0,00142382 | |
| 226507_at | PAK1 | 1,079110292 | 1,080561626 | -0,00145133 | |
| 237868_x_at | - | 1,079110292 | 1,080561626 | -0,00145133 | |
| 37028_at | PPP1R15A | 3,34210197 | 3,343554046 | -0,00145208 | |
| 223625_at | FAM126A | 2,926807124 | 2,928263672 | -0,00145655 | |
| 200729_s_at | ACTR2 | 5,384180142 | 5,38567249 | -0,00149235 | |

| | | | | | |
|---|---|---|---|---|---|
| 224880_at | RALA | 4,659596938 | 4,661126158 | -0,00152922 | |
| 204905_s_at | EEF1E1 | 5,95202668 | 5,953562035 | -0,00153535 | |
| 208965_s_at | IFI16 | 0,334552598 | 0,336136178 | -0,00158358 | |
| 236286_at | PROCA1 | 0,334552598 | 0,336136178 | -0,00158358 | |
| 225253_s_at | METTL2A /// MET | 4,224629877 | 4,226225581 | -0,0015957 | |
| 209043_at | PAPSS1 | 3,957816838 | 3,959421856 | -0,00160502 | |
| 214766_s_at | AHCTF1 | 3,069741844 | 3,071356473 | -0,00161463 | |
| 225563_at | PAN3 | 3,994121175 | 3,995764148 | -0,00164297 | |
| 208264_s_at | EIF3J | 2,915422315 | 2,917077481 | -0,00165517 | |
| 1559861_at | - | -0,259832361 | -0,258114234 | -0,00171813 | |
| 1566452_at | - | -0,259832361 | -0,258114234 | -0,00171813 | |
| 222515_x_at | TMEM165 | -0,259832361 | -0,258114234 | -0,00171813 | |
| 228307_at | EMILIN3 | -0,259832361 | -0,258114234 | -0,00171813 | |
| 228602_at | SGCD | -0,259832361 | -0,258114234 | -0,00171813 | |
| 229496_at | CLP1 | -0,259832361 | -0,258114234 | -0,00171813 | |
| 236159_x_at | - | -0,259832361 | -0,258114234 | -0,00171813 | |
| 240432_x_at | KLF7 | -0,259832361 | -0,258114234 | -0,00171813 | |
| 203185_at | RASSF2 | 1,063722698 | 1,065442793 | -0,0017201 | |
| 227332_at | PXN-AS1 | 1,063722698 | 1,065442793 | -0,0017201 | |
| 218435_at | DNAJC15 | 3,265148651 | 3,266872397 | -0,00172375 | |
| 202825_at | SLC25A4 | 2,737756747 | 2,739547836 | -0,00179109 | |
| 203491_s_at | CEP57 | 2,194816625 | 2,196649505 | -0,00183288 | |
| 225753_at | ZNF513 | 0,735041319 | 0,73688026 | -0,00183894 | |
| 227201_at | LOC643837 | 0,735041319 | 0,73688026 | -0,00183894 | |
| 228019_s_at | LOC100509751 / | 5,141940812 | 5,143823736 | -0,00188292 | |
| 217122_s_at | SLC35E2 /// SLC | 3,191547726 | 3,193438316 | -0,00189059 | |
| 217832_at | SYNCRIP | 4,731959456 | 4,733852814 | -0,00189336 | |
| 225310_at | RBMX /// SNORD | 5,123506602 | 5,125413579 | -0,00190698 | |
| 224986_s_at | PDPK1 | 3,685285822 | 3,687194562 | -0,00190874 | |
| 225029_at | LOC550643 | 4,749296484 | 4,751278918 | -0,00198243 | |
| 208728_s_at | CDC42 | 4,770777552 | 4,772769658 | -0,00199211 | |
| 213187_x_at | FTL | 7,7146981 | 7,716696773 | -0,00199867 | |
| 1555889_a_at | CRTAP /// LOC10 | 4,480461153 | 4,482467631 | -0,00200648 | |
| 206438_x_at | TCTN2 | 2,724770722 | 2,726791458 | -0,00202074 | |
| 216212_s_at | DKC1 /// SNORA | 3,893269405 | 3,895315489 | -0,00204608 | |
| 210275_s_at | ZFAND5 | 4,951987283 | 4,954036493 | -0,00204921 | |
| 200794_x_at | DAZAP2 | 5,711786826 | 5,713837217 | -0,00205039 | |
| 210216_x_at | RAD1 | 3,420201053 | 3,42231092 | -0,00210987 | |
| 1555865_at | LOC255512 | -0,285837228 | -0,283667828 | -0,0021694 | |
| 215660_s_at | MAST2 | -0,285837228 | -0,283667828 | -0,0021694 | |
| 228203_at | B3GNT1 | -0,285837228 | -0,283667828 | -0,0021694 | |
| 228422_at | LHFPL4 | -0,285837228 | -0,283667828 | -0,0021694 | |
| 230987_at | - | -0,285837228 | -0,283667828 | -0,0021694 | |
| 232022_at | - | -0,285837228 | -0,283667828 | -0,0021694 | |
| 233870_at | NAV1 | -0,285837228 | -0,283667828 | -0,0021694 | |
| 237045_at | FAM91A1 | -0,285837228 | -0,283667828 | -0,0021694 | |
| 1556499_s_at | COL1A1 | 2,038300149 | 2,040470494 | -0,00217034 | |
| 212851_at | DCUN1D4 | 1,716223609 | 1,718396606 | -0,002173 | |
| 244563_at | QSER1 | 1,523472489 | 1,525647381 | -0,00217489 | |
| 212624_s_at | CHN1 | 1,037706275 | 1,039887232 | -0,00218096 | |
| 208891_at | DUSP6 | 4,169090081 | 4,171380634 | -0,00229055 | |
| 226775_at | ENY2 | 4,118170245 | 4,12047658 | -0,00230634 | |
| 226924_at | LOC400657 | 2,030406107 | 2,032717854 | -0,00231175 | |
| 205144_at | MFSD7 | 0,291166226 | 0,293518836 | -0,00235261 | |
| 209414_at | FZR1 | 0,291166226 | 0,293518836 | -0,00235261 | |
| 231422_x_at | TYSND1 | 0,291166226 | 0,293518836 | -0,00235261 | |

| | | | | | |
|---|---|---|---|---|---|
| 244078_at | - | 0,291166226 | 0,293518836 | -0,00235261 | |
| 227001_at | NIPAL2 | 1,027166904 | 1,029536893 | -0,00236999 | |
| 218212_s_at | MOCS2 | 2,791679321 | 2,794067719 | -0,0023884 | |
| 201406_at | RPL36A /// RPL3 | 7,07437963 | 7,076856008 | -0,00247638 | |
| 206103_at | RAC3 | 2,605861638 | 2,608344689 | -0,00248305 | |
| 1552827_s_at | SLC26A7 | -2,089474779 | -2,086984744 | -0,00249004 | |
| 204468_s_at | TIE1 | -2,089474779 | -2,086984744 | -0,00249004 | |
| 205703_at | - | -2,089474779 | -2,086984744 | -0,00249004 | |
| 206271_at | TLR3 | -2,089474779 | -2,086984744 | -0,00249004 | |
| 214567_s_at | XCL1 /// XCL2 | -2,089474779 | -2,086984744 | -0,00249004 | |
| 214595_at | KCNG1 | -2,089474779 | -2,086984744 | -0,00249004 | |
| 217530_at | SLC34A1 | -2,089474779 | -2,086984744 | -0,00249004 | |
| 220383_at | ABCG5 | -2,089474779 | -2,086984744 | -0,00249004 | |
| 222853_at | FLRT3 | -2,089474779 | -2,086984744 | -0,00249004 | |
| 234570_at | ZNF71 | -2,089474779 | -2,086984744 | -0,00249004 | |
| 235524_at | RALGAPA1 | -2,089474779 | -2,086984744 | -0,00249004 | |
| 237927_at | - | -2,089474779 | -2,086984744 | -0,00249004 | |
| 238148_s_at | ZNF818P | -2,089474779 | -2,086984744 | -0,00249004 | |
| 238934_at | - | -2,089474779 | -2,086984744 | -0,00249004 | |
| 204969_s_at | RDX | 1,504602299 | 1,507116528 | -0,00251423 | |
| 207805_s_at | PSMD9 | 4,053102157 | 4,05564712 | -0,00254496 | |
| 223512_at | SAR1B | 2,017152618 | 2,019703483 | -0,00255086 | |
| 223016_x_at | ZRANB2 | 4,049851779 | 4,052455702 | -0,00260392 | |
| 205195_at | AP1S1 | 2,277177578 | 2,27978304 | -0,00260546 | |
| 1553405_a_at | CSMD1 | -1,964176657 | -1,961547147 | -0,00262951 | |
| 1556999_at | LOC100271832 | -1,964176657 | -1,961547147 | -0,00262951 | |
| 1558760_at | - | -1,964176657 | -1,961547147 | -0,00262951 | |
| 1560285_at | - | -1,964176657 | -1,961547147 | -0,00262951 | |
| 1561560_at | - | -1,964176657 | -1,961547147 | -0,00262951 | |
| 1562720_at | - | -1,964176657 | -1,961547147 | -0,00262951 | |
| 1565325_at | MATR3 /// SNHG | -1,964176657 | -1,961547147 | -0,00262951 | |
| 1566897_at | - | -1,964176657 | -1,961547147 | -0,00262951 | |
| 1569614_s_at | LCN8 | -1,964176657 | -1,961547147 | -0,00262951 | |
| 203639_s_at | FGFR2 | -1,964176657 | -1,961547147 | -0,00262951 | |
| 204154_at | CDO1 | -1,964176657 | -1,961547147 | -0,00262951 | |
| 206088_at | LRRC37A3 | -1,964176657 | -1,961547147 | -0,00262951 | |
| 206420_at | IGSF6 | -1,964176657 | -1,961547147 | -0,00262951 | |
| 210713_at | ITSN1 | -1,964176657 | -1,961547147 | -0,00262951 | |
| 211790_s_at | MLL2 | -1,964176657 | -1,961547147 | -0,00262951 | |
| 215254_at | RCAN1 | -1,964176657 | -1,961547147 | -0,00262951 | |
| 215578_at | - | -1,964176657 | -1,961547147 | -0,00262951 | |
| 220613_s_at | SYTL2 | -1,964176657 | -1,961547147 | -0,00262951 | |
| 229049_at | LOC100506655 | -1,964176657 | -1,961547147 | -0,00262951 | |
| 231566_at | NOXRED1 | -1,964176657 | -1,961547147 | -0,00262951 | |
| 232316_at | SAMD15 | -1,964176657 | -1,961547147 | -0,00262951 | |
| 235155_at | BDH2 | -1,964176657 | -1,961547147 | -0,00262951 | |
| 235813_at | - | -1,964176657 | -1,961547147 | -0,00262951 | |
| 236418_at | TTLL10 | -1,964176657 | -1,961547147 | -0,00262951 | |
| 239478_x_at | C14orf118 | -1,964176657 | -1,961547147 | -0,00262951 | |
| 240892_at | - | -1,964176657 | -1,961547147 | -0,00262951 | |
| 244541_x_at | - | -1,964176657 | -1,961547147 | -0,00262951 | |
| 244840_x_at | DOCK4 | -1,964176657 | -1,961547147 | -0,00262951 | |
| 210010_s_at | SLC25A1 | 2,596981013 | 2,599625088 | -0,00264407 | |
| 209303_at | NDUFS4 | 3,493810203 | 3,496520471 | -0,00271027 | |
| 224713_at | MKI67IP | 4,568952594 | 4,571665519 | -0,00271292 | |
| 204090_at | STK19 | 2,38635068 | 2,389070873 | -0,00272019 | |

| | | | | | |
|---|---|---|---|---|---|
| 205707_at | IL17RA | 1,269985355 | 1,272721751 | -0,0027364 | |
| 225944_at | NLN | 1,269985355 | 1,272721751 | -0,0027364 | |
| 214095_at | SHMT2 | 2,26825514 | 2,271023131 | -0,00276799 | |
| 217286_s_at | NDRG3 | 3,202127528 | 3,204982027 | -0,0028545 | |
| 209478_at | STRA13 | 4,806654012 | 4,809530416 | -0,0028764 | |
| 1553701_a_at | DUSP18 | -0,325749314 | -0,322870281 | -0,00287903 | |
| 1554287_at | TRIM4 | -0,325749314 | -0,322870281 | -0,00287903 | |
| 207367_at | ATP12A | -0,325749314 | -0,322870281 | -0,00287903 | |
| 215138_s_at | KAZN | -0,325749314 | -0,322870281 | -0,00287903 | |
| 216881_x_at | PRB4 | -0,325749314 | -0,322870281 | -0,00287903 | |
| 232623_at | LOC100128751 | -0,325749314 | -0,322870281 | -0,00287903 | |
| 235249_at | RDH13 | -0,325749314 | -0,322870281 | -0,00287903 | |
| 238646_at | - | -0,325749314 | -0,322870281 | -0,00287903 | |
| 243594_x_at | SPIRE2 | -0,325749314 | -0,322870281 | -0,00287903 | |
| 44669_at | SDHAF1 | 3,924839896 | 3,927724715 | -0,00288482 | |
| 200703_at | DYNLL1 | 6,611097066 | 6,614026941 | -0,00292987 | |
| 59644_at | BMP2K | 5,247148178 | 5,250095671 | -0,00294749 | |
| 205097_at | SLC26A2 | 2,994644467 | 2,997606395 | -0,00296193 | |
| 221937_at | SYNRG | 2,840652551 | 2,843651104 | -0,00299855 | |
| 1567080_s_at | CLN6 | 2,837639949 | 2,840694075 | -0,00305413 | |
| 226736_at | CHURC1 | 3,9480725 | 3,951219941 | -0,00314744 | |
| 216379_x_at | CD24 | 0,984222186 | 0,987376598 | -0,00315441 | |
| 1556046_a_at | LINC00599 /// MI | 0,246434561 | 0,249604176 | -0,00316961 | |
| 223362_s_at | 37865 | 0,246434561 | 0,249604176 | -0,00316961 | |
| 243982_at | - | 0,246434561 | 0,249604176 | -0,00316961 | |
| 219806_s_at | C11orf75 | 1,982112682 | 1,98530622 | -0,00319354 | |
| 212804_s_at | GAPVD1 | 2,830080806 | 2,833274877 | -0,00319407 | |
| 1554250_s_at | TRIM73 | 1,46610492 | 1,469325098 | -0,00322018 | |
| 220113_x_at | POLR1B | 1,46610492 | 1,469325098 | -0,00322018 | |
| 229822_at | - | 1,46610492 | 1,469325098 | -0,00322018 | |
| 1553857_at | IGSF22 | -0,352951799 | -0,34958478 | -0,00336702 | |
| 1554607_at | CNKSR2 | -0,352951799 | -0,34958478 | -0,00336702 | |
| 1554835_a_at | B3GNT5 /// LOC1 | -0,352951799 | -0,34958478 | -0,00336702 | |
| 209684_at | RIN2 | -0,352951799 | -0,34958478 | -0,00336702 | |
| 214025_at | DDX28 | -0,352951799 | -0,34958478 | -0,00336702 | |
| 217468_at | CYP2D6 | -0,352951799 | -0,34958478 | -0,00336702 | |
| 219118_at | FKBP11 | -0,352951799 | -0,34958478 | -0,00336702 | |
| 220296_at | - | -0,352951799 | -0,34958478 | -0,00336702 | |
| 221633_at | NCAPH2 | -0,352951799 | -0,34958478 | -0,00336702 | |
| 223517_at | FBXO44 | -0,352951799 | -0,34958478 | -0,00336702 | |
| 236709_at | - | -0,352951799 | -0,34958478 | -0,00336702 | |
| 237654_at | PPP1R36 | -0,352951799 | -0,34958478 | -0,00336702 | |
| 203218_at | MAPK9 | 3,109000457 | 3,112391743 | -0,00339129 | |
| 206176_at | BMP6 | 1,233777334 | 1,23718272 | -0,00340539 | |
| 227406_at | GABPB1-AS1 | 1,233777334 | 1,23718272 | -0,00340539 | |
| 227103_s_at | ECE2 | 1,968405881 | 1,971855016 | -0,00344913 | |
| 1555618_s_at | SAE1 | 4,552510269 | 4,555975485 | -0,00346522 | |
| 207988_s_at | ARPC2 | 4,965850214 | 4,96934727 | -0,00349706 | |
| 212088_at | PMPCA | 3,889637741 | 3,893176339 | -0,0035386 | |
| 1552295_a_at | SLC39A13 | 0,95671774 | 0,960386719 | -0,00366898 | |
| 201561_s_at | CLSTN1 | 0,95671774 | 0,960386719 | -0,00366898 | |
| 202179_at | BLMH | 3,277429131 | 3,281121096 | -0,00369197 | |
| 201897_s_at | CKS1B | 6,202505765 | 6,206215175 | -0,00370941 | |
| 218082_s_at | UBP1 | 3,719237563 | 3,722963534 | -0,00372597 | |
| 219348_at | USE1 | 2,331726354 | 2,335461563 | -0,00373521 | |
| 204218_at | ANAPC15 | 2,630440446 | 2,634191677 | -0,00375123 | |

| | | | | | |
|---|---|---|---|---|---|
| 212788_x_at | FTL | 7,940738094 | 7,944538334 | -0,00380024 | |
| 224739_at | PIM3 | 3,149650293 | 3,153484076 | -0,00383378 | |
| 203245_s_at | LINC00094 | 1,946200473 | 1,950068772 | -0,0038683 | |
| 221562_s_at | SIRT3 | 0,945567441 | 0,949447784 | -0,00388034 | |
| 208856_x_at | RPLP0 | 7,905611789 | 7,909513373 | -0,00390158 | |
| 218575_at | ANAPC1 /// LOC1 | 3,480351894 | 3,484256382 | -0,00390449 | |
| 221988_at | C19orf42 | 3,142344098 | 3,146317015 | -0,00397292 | |
| 1569445_at | LOC100287576 / | 0,20027154 | 0,204310755 | -0,00403922 | |
| 215479_at | - | 0,20027154 | 0,204310755 | -0,00403922 | |
| 229531_at | - | 0,20027154 | 0,204310755 | -0,00403922 | |
| 1560943_s_at | PGD | -0,394749123 | -0,39061235 | -0,00413677 | |
| 228897_at | DERL3 | -0,394749123 | -0,39061235 | -0,00413677 | |
| 237878_at | - | -0,394749123 | -0,39061235 | -0,00413677 | |
| 208689_s_at | RPN2 | 5,950110119 | 5,954331698 | -0,00422158 | |
| 225437_s_at | BRAT1 | 1,603688695 | 1,60795124 | -0,00426255 | |
| 226385_s_at | MALSU1 | 5,763102253 | 5,767373629 | -0,00427138 | |
| 218269_at | DROSHA | 4,624194503 | 4,628492984 | -0,00429848 | |
| 201403_s_at | LOC100505828 / | 4,866998631 | 4,871328311 | -0,00432968 | |
| 226027_at | SWI5 | 2,506929603 | 2,511262629 | -0,00433303 | |
| 228287_at | ING5 | 2,352981171 | 2,357351836 | -0,00437067 | |
| 223247_at | MED10 | 3,056846434 | 3,061230246 | -0,00438381 | |
| 223294_at | CXorf26 | 4,377913865 | 4,382314353 | -0,00440049 | |
| 208996_s_at | POLR2C | 4,179835245 | 4,18426834 | -0,00443309 | |
| 211987_at | TOP2B | 5,252954628 | 5,257390237 | -0,00443561 | |
| 209685_s_at | PRKCB | 4,762858846 | 4,767329587 | -0,00447074 | |
| 213449_at | POP1 | 2,292660284 | 2,297144887 | -0,0044846 | |
| 200689_x_at | EEF1G /// MIR36 | 7,483812203 | 7,488300539 | -0,00448834 | |
| 200048_s_at | JTB | 5,411282092 | 5,415788424 | -0,00450633 | |
| 226923_at | SCFD2 | 2,912562019 | 2,917077481 | -0,00451546 | |
| 220924_s_at | SLC38A2 | 7,107390573 | 7,111962124 | -0,00457155 | |
| 1557710_at | - | 0,171847695 | 0,176436073 | -0,00458838 | |
| 214950_at | - | 0,171847695 | 0,176436073 | -0,00458838 | |
| 219922_s_at | LTBP3 | 0,171847695 | 0,176436073 | -0,00458838 | |
| 230169_at | THAP6 | 0,171847695 | 0,176436073 | -0,00458838 | |
| 236648_at | GMPS | 0,171847695 | 0,176436073 | -0,00458838 | |
| 219679_s_at | WAC | 1,58584542 | 1,590454215 | -0,0046088 | |
| 206102_at | GINS1 | 4,775114384 | 4,779734028 | -0,00461964 | |
| 1553542_at | CCDC125 | -0,423248115 | -0,41858459 | -0,00466353 | |
| 205137_x_at | USH1C | -0,423248115 | -0,41858459 | -0,00466353 | |
| 213650_at | GOLGA8A /// GO | -0,423248115 | -0,41858459 | -0,00466353 | |
| 243070_at | INO80D | -0,423248115 | -0,41858459 | -0,00466353 | |
| 243387_at | MESP1 | -0,423248115 | -0,41858459 | -0,00466353 | |
| 243749_s_at | - | -0,423248115 | -0,41858459 | -0,00466353 | |
| 210871_x_at | SSX2IP | 1,903621061 | 1,908311039 | -0,00468998 | |
| 203865_s_at | ADARB1 | 2,166283906 | 2,170981159 | -0,00469725 | |
| 213505_s_at | SUGP2 | 2,747420128 | 2,752192409 | -0,00477228 | |
| 213625_at | ZKSCAN4 | 2,035673598 | 2,040470494 | -0,0047969 | |
| 204208_at | RNGTT | 3,859508144 | 3,864347045 | -0,0048389 | |
| 211666_x_at | RNU86 /// RPL3 / | 7,345711032 | 7,35061262 | -0,00490159 | |
| 201552_at | LAMP1 | 4,591035503 | 4,595981624 | -0,00494612 | |
| 216870_x_at | DLEU2 | 2,027765132 | 2,032717854 | -0,00495272 | |
| 227724_at | LOC728190 | 2,027765132 | 2,032717854 | -0,00495272 | |
| 1555491_a_at | C19orf66 | 0,152582607 | 0,157549243 | -0,00496664 | |
| 232192_at | LOC153811 | 0,152582607 | 0,157549243 | -0,00496664 | |
| 211594_s_at | MRPL9 | 3,566577193 | 3,571564634 | -0,00498744 | |
| 223443_s_at | AMZ2P1 | 0,888485433 | 0,893473268 | -0,00498783 | |

| | | | | | |
|---|---|---|---|---|---|
| 1554556_a_at | ATP11B | 1,373456798 | 1,378453643 | -0,00499685 | |
| 224302_s_at | MRPS36 | 4,371158382 | 4,376200741 | -0,00504236 | |
| 208826_x_at | HINT1 | 4,866998631 | 4,872052597 | -0,00505397 | |
| 229120_s_at | CDC42SE1 | 4,018934862 | 4,024062565 | -0,0051277 | |
| 200909_s_at | RPLP2 /// SNOR/ | 7,735887788 | 7,741085628 | -0,00519784 | |
| 218185_s_at | ARMC1 | 4,664280639 | 4,669482912 | -0,00520227 | |
| 212529_at | LSM12 | 2,254767157 | 2,259997914 | -0,00523076 | |
| 223405_at | NPL | 1,139070151 | 1,144305291 | -0,00523514 | |
| 221481_x_at | HNRNPD | 6,703353216 | 6,708598363 | -0,00524515 | |
| 201358_s_at | COPB1 | 5,829243814 | 5,834498675 | -0,00525486 | |
| 209832_s_at | CDT1 | 1,722769813 | 1,728030156 | -0,00526034 | |
| 212744_at | BBS4 | 1,722769813 | 1,728030156 | -0,00526034 | |
| 200595_s_at | EIF3A | 5,398050406 | 5,403316429 | -0,00526602 | |
| 1556821_x_at | DLEU2 | 0,87091093 | 0,876248481 | -0,00533755 | |
| 205851_at | NME6 | 0,87091093 | 0,876248481 | -0,00533755 | |
| 200688_at | SF3B3 | 1,868628927 | 1,874012195 | -0,00538327 | |
| 203278_s_at | PHF21A | 1,868628927 | 1,874012195 | -0,00538327 | |
| 35617_at | MAPK7 | 1,003167985 | 1,008610748 | -0,00544276 | |
| 218722_s_at | CCDC51 | 2,933877275 | 2,939363796 | -0,00548652 | |
| 223498_at | SPECC1 | -0,467091869 | -0,461594427 | -0,00549744 | |
| 224218_s_at | TRPS1 | -0,467091869 | -0,461594427 | -0,00549744 | |
| 226622_at | MUC20 | -0,467091869 | -0,461594427 | -0,00549744 | |
| 227789_at | MRS2 | -0,467091869 | -0,461594427 | -0,00549744 | |
| 231571_at | LOC100507362 | -0,467091869 | -0,461594427 | -0,00549744 | |
| 234054_at | LOC143188 | -0,467091869 | -0,461594427 | -0,00549744 | |
| 237502_at | CRLS1 | -0,467091869 | -0,461594427 | -0,00549744 | |
| 208948_s_at | STAU1 | 4,949896306 | 4,95540405 | -0,00550774 | |
| 202705_at | CCNB2 | 5,014681934 | 5,02022104 | -0,00553911 | |
| 214674_at | USP19 | 0,123193849 | 0,128747141 | -0,00555329 | |
| 221588_x_at | ALDH6A1 | 0,123193849 | 0,128747141 | -0,00555329 | |
| 239234_at | - | 0,123193849 | 0,128747141 | -0,00555329 | |
| 239944_at | - | 0,123193849 | 0,128747141 | -0,00555329 | |
| 203464_s_at | EPN2 | 0,995078803 | 1,000684521 | -0,00560572 | |
| 203620_s_at | FCHSD2 | 0,995078803 | 1,000684521 | -0,00560572 | |
| 229410_at | SLC35E1 | 4,177454337 | 4,183101476 | -0,00564714 | |
| 203122_at | TRAPPC12 | 1,85379461 | 1,859476797 | -0,00568219 | |
| 222962_s_at | MCM10 | 2,849652795 | 2,855418981 | -0,00576619 | |
| 226669_at | USP42 | 3,255587071 | 3,261354437 | -0,00576737 | |
| 222600_s_at | UBA6 | 2,614687931 | 2,620464265 | -0,00577633 | |
| 208638_at | PDIA6 | 5,654304455 | 5,660125593 | -0,00582114 | |
| 238829_at | - | 1,331241794 | 1,337086025 | -0,00584423 | |
| 218709_s_at | IFT52 | 3,693640704 | 3,69948842 | -0,00584772 | |
| 209694_at | PTS | 4,012938265 | 4,018839599 | -0,00590133 | |
| 202234_s_at | SLC16A1 | 3,164152611 | 3,170070041 | -0,00591743 | |
| 212008_at | UBXN4 | 2,520143371 | 2,526063925 | -0,00592055 | |
| 218848_at | THOC6 | 2,220484971 | 2,226407528 | -0,00592256 | |
| 228870_at | FAM84B | 0,841135803 | 0,847075464 | -0,00593966 | |
| 1554493_s_at | THADA | 0,103263601 | 0,109221392 | -0,00595779 | |
| 220965_s_at | RSPH6A | 0,103263601 | 0,109221392 | -0,00595779 | |
| 241468_at | - | 0,103263601 | 0,109221392 | -0,00595779 | |
| 241921_x_at | - | 0,103263601 | 0,109221392 | -0,00595779 | |
| 241364_at | TMEM57 | 2,325288363 | 2,331254065 | -0,0059657 | |
| 225340_s_at | CAPRIN1 | 5,335357524 | 5,341379601 | -0,00602208 | |
| 212573_at | ENDOD1 | 1,973904239 | 1,979940779 | -0,00603654 | |
| 210326_at | AGXT | -0,496991414 | -0,490927335 | -0,00606408 | |
| 215124_at | ZNF550 | -0,496991414 | -0,490927335 | -0,00606408 | |

| | | | | | |
|---|---|---|---|---|---|
| 221967_at | NXPH4 | -0,496991414 | -0,490927335 | -0,00606408 | |
| 227275_at | MEX3C | -0,496991414 | -0,490927335 | -0,00606408 | |
| 230938_x_at | ATF5 | -0,496991414 | -0,490927335 | -0,00606408 | |
| 214469_at | HIST1H2AB /// H | 3,097712429 | 3,103784293 | -0,00607186 | |
| 223401_at | C17orf48 | 1,318332599 | 1,324440845 | -0,00610825 | |
| 223412_at | KBTBD7 | 1,318332599 | 1,324440845 | -0,00610825 | |
| 1553103_at | NFX1 | 2,596981013 | 2,603119255 | -0,00613824 | |
| 221258_s_at | KIF18A | 1,829737795 | 1,835911187 | -0,00617339 | |
| 243527_at | - | 0,82905153 | 0,835239052 | -0,00618752 | |
| 208642_s_at | XRCC5 | 5,8716501 | 5,877874624 | -0,00622452 | |
| 221484_at | B4GALT5 | 3,361633475 | 3,367892567 | -0,00625909 | |
| 223351_at | C17orf80 | 2,893830579 | 2,900133867 | -0,00630329 | |
| 226208_at | ZSWIM6 | 2,084778276 | 2,091116676 | -0,0063384 | |
| 223003_at | C19orf43 | 6,121220712 | 6,127572504 | -0,00635179 | |
| 225123_at | SESN3 | 3,406034785 | 3,412387088 | -0,0063523 | |
| 212015_x_at | PTBP1 | 5,405190236 | 5,411560032 | -0,0063698 | |
| 219762_s_at | RPL36 | 6,495314506 | 6,501746214 | -0,00643171 | |
| 212286_at | ANKRD12 | 1,493160564 | 1,499637023 | -0,00647646 | |
| 225278_at | PRKAB2 | 2,077135162 | 2,083632167 | -0,00649701 | |
| 223852_s_at | STK40 | 2,661438684 | 2,667949403 | -0,00651072 | |
| 204326_x_at | MT1X | 5,259160052 | 5,26568197 | -0,00652192 | |
| 236562_at | ZNF439 | 3,013454777 | 3,019999252 | -0,00654448 | |
| 223167_s_at | USP25 | 1,659347917 | 1,665902186 | -0,00655427 | |
| 226566_at | TRIM11 | 1,659347917 | 1,665902186 | -0,00655427 | |
| 201728_s_at | KIAA0100 | 1,073999308 | 1,080561626 | -0,00656232 | |
| 219657_s_at | KLF3 | 0,810733188 | 0,817300337 | -0,00656715 | |
| 229753_at | POU2F1 | 0,810733188 | 0,817300337 | -0,00656715 | |
| 231848_x_at | ZNF207 | 0,810733188 | 0,817300337 | -0,00656715 | |
| 225283_at | ARRDC4 | 4,806268808 | 4,812929194 | -0,00666039 | |
| 216153_x_at | RECK | 2,652894659 | 2,659583735 | -0,00668908 | |
| 218836_at | RPP21 /// TRIM3 | 4,839779088 | 4,846483715 | -0,00670463 | |
| 200650_s_at | LDHA | 7,280648955 | 7,287405531 | -0,00675658 | |
| 227379_at | MBOAT1 | 3,336243945 | 3,343031718 | -0,00678777 | |
| 244317_at | KIAA1324L | 0,798390451 | 0,805216064 | -0,00682561 | |
| 204955_at | SRPX | 2,382221458 | 2,389070873 | -0,00684942 | |
| 202777_at | SHOC2 | 3,75958912 | 3,766462619 | -0,0068735 | |
| 37278_at | TAZ | 1,172929414 | 1,179838678 | -0,00690926 | |
| 207258_at | DSCR4 | -0,543051993 | -0,536087151 | -0,00696484 | |
| 211248_s_at | CHRD | -0,543051993 | -0,536087151 | -0,00696484 | |
| 216197_at | ATF7IP | -0,543051993 | -0,536087151 | -0,00696484 | |
| 223976_at | FUT10 | -0,543051993 | -0,536087151 | -0,00696484 | |
| 231342_at | LOC646778 | -0,543051993 | -0,536087151 | -0,00696484 | |
| 232062_at | ADCY1 | -0,543051993 | -0,536087151 | -0,00696484 | |
| 233417_at | - | -0,543051993 | -0,536087151 | -0,00696484 | |
| 236250_at | AFG3L1P | -0,543051993 | -0,536087151 | -0,00696484 | |
| 240780_at | - | -0,543051993 | -0,536087151 | -0,00696484 | |
| 210178_x_at | SRSF10 | 3,926967479 | 3,933981372 | -0,00701389 | |
| 212706_at | RASA4 /// RASA4 | 0,052199481 | 0,059218869 | -0,00701939 | |
| 214490_at | ARSF | 0,052199481 | 0,059218869 | -0,00701939 | |
| 217711_at | TEK | 0,052199481 | 0,059218869 | -0,00701939 | |
| 227876_at | ARHGAP39 | 0,052199481 | 0,059218869 | -0,00701939 | |
| 228860_at | UBE3B | 0,052199481 | 0,059218869 | -0,00701939 | |
| 237453_at | ZNF529 | 0,052199481 | 0,059218869 | -0,00701939 | |
| 237799_at | SLC22A12 | 0,052199481 | 0,059218869 | -0,00701939 | |
| 244523_at | MMD | 0,052199481 | 0,059218869 | -0,00701939 | |
| 214938_x_at | HMGB1 | 7,454460331 | 7,461481408 | -0,00702108 | |

| | | | | | |
|---|---|---|---|---|---|
| 201553_s_at | LAMP1 | 5,815911485 | 5,822932837 | -0,00702135 | |
| 218742_at | NARFL | 1,274448124 | 1,281471383 | -0,00702326 | |
| 223207_x_at | PHPT1 | 4,656608465 | 4,66363827 | -0,00702981 | |
| 236371_s_at | TGS1 | 1,04816921 | 1,055274826 | -0,00710562 | |
| 202653_s_at | 07.03.15 | 4,074372924 | 4,08155382 | -0,0071809 | |
| 217869_at | HSD17B12 | 5,117309148 | 5,124502072 | -0,00719292 | |
| 238417_at | PGM2L1 | 0,779676066 | 0,786897721 | -0,00722166 | |
| 201350_at | FLOT2 | 2,456762221 | 2,464004877 | -0,00724266 | |
| 201763_s_at | DAXX | 3,211467103 | 3,218713651 | -0,00724655 | |
| 223486_at | GTPBP8 | 3,848325987 | 3,855584709 | -0,00725872 | |
| 218286_s_at | RNF7 | 3,312571045 | 3,319860027 | -0,00728898 | |
| 218920_at | FAM193B | 2,038300149 | 2,045615873 | -0,00731572 | |
| 200015_s_at | 37500 | 5,967440469 | 5,974790325 | -0,00734986 | |
| 212855_at | DCUN1D4 | 3,69780007 | 3,705189898 | -0,00738983 | |
| 200877_at | CCT4 | 6,511591115 | 6,519016859 | -0,00742574 | |
| 78495_at | ZNF783 | 3,378314944 | 3,385753948 | -0,007439 | |
| 235023_at | VPS13C | 1,251994932 | 1,259496994 | -0,00750206 | |
| 215293_s_at | PGAP2 | 2,965960999 | 2,973511251 | -0,00755025 | |
| 203136_at | RABAC1 | 4,571221501 | 4,578781665 | -0,00756016 | |
| 203670_at | ARPC4-TTLL3 /// | -0,57445847 | -0,566887459 | -0,00757101 | |
| 207611_at | HIST1H2BL | -0,57445847 | -0,566887459 | -0,00757101 | |
| 216914_at | CDC25C | -0,57445847 | -0,566887459 | -0,00757101 | |
| 229817_at | ZNF608 | -0,57445847 | -0,566887459 | -0,00757101 | |
| 230837_at | LOC100132057 | -0,57445847 | -0,566887459 | -0,00757101 | |
| 1559388_a_at | LYNX1 | 0,760715727 | 0,768343791 | -0,00762806 | |
| 213645_at | ENOSF1 | 0,760715727 | 0,768343791 | -0,00762806 | |
| 222015_at | CSNK1E | 1,607231019 | 1,614891062 | -0,00766004 | |
| 1559443_s_at | IL21R-AS1 | 1,021868205 | 1,029536893 | -0,00766869 | |
| 201028_s_at | CD99 | 1,021868205 | 1,029536893 | -0,00766869 | |
| 214391_x_at | PTGER1 | 1,021868205 | 1,029536893 | -0,00766869 | |
| 1554388_at | VPS29 | 0,020670649 | 0,028364014 | -0,00769336 | |
| 1569149_at | PDLIM7 | 0,020670649 | 0,028364014 | -0,00769336 | |
| 214104_at | GPR161 | 0,020670649 | 0,028364014 | -0,00769336 | |
| 225379_at | MAPT | 0,020670649 | 0,028364014 | -0,00769336 | |
| 229617_x_at | AP2A1 | 0,020670649 | 0,028364014 | -0,00769336 | |
| 214686_at | ZNF266 | 3,389672581 | 3,39737216 | -0,00769958 | |
| 219065_s_at | DPY30 /// MEMO | 4,584311257 | 4,592030564 | -0,00771931 | |
| 206288_at | PGGT1B | 1,434549838 | 1,442269454 | -0,00771962 | |
| 1553218_a_at | ZNF512 | 2,338135744 | 2,345926937 | -0,00779119 | |
| 224587_at | SUB1 | 4,770777552 | 4,778575632 | -0,00779808 | |
| 210811_s_at | DDX49 | 3,18563636 | 3,193438316 | -0,00780196 | |
| 230735_at | - | 0,425644434 | 0,433557298 | -0,00791286 | |
| 231857_s_at | AGBL5 | 0,425644434 | 0,433557298 | -0,00791286 | |
| 232228_at | ZNF530 | 0,425644434 | 0,433557298 | -0,00791286 | |
| 224820_at | COX20 | 3,850569363 | 3,858511405 | -0,00794204 | |
| 223478_at | TIMM8B | 2,739371812 | 2,747463672 | -0,00809186 | |
| 37986_at | EPOR | 3,604290432 | 3,612447534 | -0,0081571 | |
| 209502_s_at | BAIAP2 | -0,00073712 | 0,007421914 | -0,00815903 | |
| 222347_at | - | -0,00073712 | 0,007421914 | -0,00815903 | |
| 225778_at | RBMS2 | -0,00073712 | 0,007421914 | -0,00815903 | |
| 229894_s_at | RAB43 | -0,00073712 | 0,007421914 | -0,00815903 | |
| 232859_s_at | MAGI1 | -0,00073712 | 0,007421914 | -0,00815903 | |
| 242113_at | - | -0,00073712 | 0,007421914 | -0,00815903 | |
| 225343_at | TMED8 | 4,012938265 | 4,021126974 | -0,00818871 | |
| 208887_at | EIF3G | 7,112306921 | 7,120521066 | -0,00821415 | |
| 1555315_a_at | MAK | 2,218170265 | 2,226407528 | -0,00823726 | |

| | | | | | |
|---|---|---|---|---|---|
| 38964_r_at | WAS | 2,053959821 | 2,062212735 | -0,00825291 | |
| 236402_at | BRAF | 1,215326747 | 1,223626587 | -0,00829984 | |
| 47773_at | FBXO42 | 1,314003737 | 1,322322498 | -0,00831876 | |
| 211692_s_at | BBC3 | 0,728550685 | 0,73688026 | -0,00832958 | |
| 213489_at | MAPRE2 | 0,728550685 | 0,73688026 | -0,00832958 | |
| 209271_at | BPTF | 2,493593689 | 2,501934108 | -0,00834042 | |
| 218349_s_at | ZWILCH | 3,53158484 | 3,540009821 | -0,00842498 | |
| 225681_at | CTHRC1 | 2,400710591 | 2,409170778 | -0,00846019 | |
| 200846_s_at | PPP1CA | 4,984696817 | 4,993178622 | -0,00848181 | |
| 210017_at | MALT1 | -0,622910612 | -0,614371577 | -0,00853904 | |
| 211706_s_at | CDK19 | -0,622910612 | -0,614371577 | -0,00853904 | |
| 230905_at | - | -0,622910612 | -0,614371577 | -0,00853904 | |
| 234476_at | DNAH7 | -0,622910612 | -0,614371577 | -0,00853904 | |
| 239769_at | CDH11 | -0,622910612 | -0,614371577 | -0,00853904 | |
| 242328_at | RAB3C | -0,622910612 | -0,614371577 | -0,00853904 | |
| 244248_at | TTC27 | -0,622910612 | -0,614371577 | -0,00853904 | |
| 223067_at | CWC15 | 4,700383757 | 4,708935 | -0,00855124 | |
| 217791_s_at | ALDH18A1 | 4,010265089 | 4,018839599 | -0,00857451 | |
| 226227_x_at | ZNFX1-AS1 | 6,895768471 | 6,904379077 | -0,00861061 | |
| 209008_x_at | KRT8 | 0,715481152 | 0,724100169 | -0,00861902 | |
| 226640_at | DAGLB | 0,715481152 | 0,724100169 | -0,00861902 | |
| 224991_at | CMIP | 2,089851283 | 2,098562556 | -0,00871127 | |
| 202595_s_at | LEPROTL1 | 3,844579258 | 3,853385784 | -0,00880653 | |
| 232586_x_at | OR7E126P | 0,384950573 | 0,393761504 | -0,00881093 | |
| 239898_x_at | ZNF286A | 0,384950573 | 0,393761504 | -0,00881093 | |
| 211085_s_at | STK4 | 1,191926657 | 1,200746069 | -0,00881941 | |
| 225596_at | KLC4 | 1,191926657 | 1,200746069 | -0,00881941 | |
| 203970_s_at | PEX3 | 2,470441349 | 2,479296438 | -0,00885509 | |
| 209846_s_at | BTN3A2 | 3,137452663 | 3,146317015 | -0,00886435 | |
| 212988_x_at | ACTG1 | 7,81904989 | 7,827919325 | -0,00886943 | |
| 242839_at | - | 1,381753566 | 1,390636111 | -0,00888254 | |
| 1552739_s_at | ST7L | -0,033456564 | -0,024572586 | -0,00888398 | |
| 201141_at | GPNMB | -0,033456564 | -0,024572586 | -0,00888398 | |
| 207854_at | GYPE | -0,033456564 | -0,024572586 | -0,00888398 | |
| 212292_at | SLC7A1 | -0,033456564 | -0,024572586 | -0,00888398 | |
| 221936_x_at | MRPL41 | -0,033456564 | -0,024572586 | -0,00888398 | |
| 239068_at | GNL1 | -0,033456564 | -0,024572586 | -0,00888398 | |
| 201059_at | CTTN | 3,38038663 | 3,38929984 | -0,00891321 | |
| 212375_at | EP400 | 2,771299138 | 2,780243895 | -0,00894476 | |
| 218667_at | PJA1 | 3,508091848 | 3,517040489 | -0,00894864 | |
| 210434_x_at | JTB | 5,758032154 | 5,766984279 | -0,00895212 | |
| 218058_at | CXXC1 | 2,074578432 | 2,083632167 | -0,00905374 | |
| 219729_at | PRRX2 | 0,695652136 | 0,704715219 | -0,00906308 | |
| 224316_at | - | 0,695652136 | 0,704715219 | -0,00906308 | |
| 228455_at | - | 0,695652136 | 0,704715219 | -0,00906308 | |
| 234999_at | C19orf47 | 0,695652136 | 0,704715219 | -0,00906308 | |
| 201505_at | LAMB1 | 1,69307511 | 1,702196344 | -0,00912123 | |
| 234952_s_at | PHRF1 | 1,369290453 | 1,378453643 | -0,00916319 | |
| 1554652_s_at | MAST4 | -0,655928373 | -0,646746079 | -0,00918229 | |
| 1559002_at | LOC340544 | -0,655928373 | -0,646746079 | -0,00918229 | |
| 1560754_at | CMTM7 | -0,655928373 | -0,646746079 | -0,00918229 | |
| 203915_at | CXCL9 | -0,655928373 | -0,646746079 | -0,00918229 | |
| 211461_at | CSPG4P1Y | -0,655928373 | -0,646746079 | -0,00918229 | |
| 212226_s_at | PPAP2B | -0,655928373 | -0,646746079 | -0,00918229 | |
| 214916_x_at | IGHA1 /// IGHA2 | -0,655928373 | -0,646746079 | -0,00918229 | |
| 215490_at | IBA57 | -0,655928373 | -0,646746079 | -0,00918229 | |

| | | | | | |
|---|---|---|---|---|---|
| 221615_at | BMP8B | -0,655928373 | -0,646746079 | -0,00918229 | |
| 234946_at | ENTPD6 | -0,655928373 | -0,646746079 | -0,00918229 | |
| 238682_at | CCDC96 | -0,655928373 | -0,646746079 | -0,00918229 | |
| 221622_s_at | TMEM126B | 5,274557833 | 5,28375775 | -0,00919992 | |
| 238015_at | C4orf46 | 2,611163891 | 2,620464265 | -0,00930037 | |
| 1729_at | TRADD | 1,610764667 | 1,620074108 | -0,00930944 | |
| 218941_at | FBXW2 | 1,683039483 | 1,692388143 | -0,00934866 | |
| 227433_at | KIAA2018 | 1,360921491 | 1,370274469 | -0,00935298 | |
| 206410_at | NR0B2 | 0,682279745 | 0,691645685 | -0,00936594 | |
| 222352_at | - | 0,682279745 | 0,691645685 | -0,00936594 | |
| 228130_at | ZSCAN30 | 0,682279745 | 0,691645685 | -0,00936594 | |
| 223564_s_at | GNB1L | -0,055687482 | -0,046302147 | -0,00938534 | |
| 223666_at | SNX5 | 3,266269388 | 3,275657468 | -0,00938808 | |
| 217846_at | QARS | 5,031587647 | 5,040980656 | -0,00939301 | |
| 223310_x_at | PNPLA8 | 3,446146386 | 3,455550298 | -0,00940391 | |
| 90265_at | ADAP1 | 1,525346004 | 1,534824307 | -0,0094783 | |
| 202893_at | UNC13B | 2,261526912 | 2,271023131 | -0,00949622 | |
| 232101_s_at | PIGN | 2,438982012 | 2,448549499 | -0,00956749 | |
| 208847_s_at | ADH5 | 5,182279401 | 5,191895668 | -0,00961627 | |
| 224150_s_at | CEP70 | 2,156646206 | 2,166264721 | -0,00961852 | |
| 212544_at | ZNHIT3 | 4,61586603 | 4,625489401 | -0,00962337 | |
| 202151_s_at | UBAC1 | 3,049053496 | 3,058687544 | -0,00963405 | |
| 227872_at | POLR3A | 2,048758794 | 2,058399602 | -0,00964081 | |
| 1561093_at | SLC22A25 | 1,348276312 | 1,3579181 | -0,00964179 | |
| 203452_at | B3GAT3 | 1,666156729 | 1,675891488 | -0,00973476 | |
| 239329_at | RAB21 | 1,666156729 | 1,675891488 | -0,00973476 | |
| 244598_at | LCP2 | 0,661985843 | 0,67181667 | -0,00983083 | |
| 200852_x_at | GNB2 | 3,893994642 | 3,903840511 | -0,00984587 | |
| 223063_at | C1orf198 | 3,548266005 | 3,558125564 | -0,00985956 | |
| 200968_s_at | PPIB | 5,498509893 | 5,50837347 | -0,00986358 | |
| 241402_at | TSEN54 | 1,143956113 | 1,153866761 | -0,00991065 | |
| 49485_at | PRDM4 | 1,506500464 | 1,516411707 | -0,00991124 | |
| 218643_s_at | CRIPT | 2,243430036 | 2,253342112 | -0,00991208 | |
| 226889_at | WDR35 | 2,422991296 | 2,432926755 | -0,00993546 | |
| 37012_at | CAPZB | 5,021335256 | 5,031286769 | -0,00995151 | |
| 218967_s_at | PTER | 1,655931423 | 1,665902186 | -0,00997076 | |
| 218036_x_at | NMD3 | 3,191547726 | 3,201528602 | -0,00998088 | |
| 219275_at | PDCD5 | 4,933411285 | 4,943393743 | -0,00998246 | |
| 204627_s_at | ITGB3 | 2,976955384 | 2,986947093 | -0,00999171 | |
| 218973_at | EFTUD1 | 2,976955384 | 2,986947093 | -0,00999171 | |
| 244861_at | ZNF527 | 2,654607516 | 2,664608955 | -0,01000144 | |
| 225534_at | C8orf40 | 3,886725813 | 3,896739828 | -0,01001402 | |
| 201908_at | DVL3 | 2,331726354 | 2,341749893 | -0,01002354 | |
| 211700_s_at | TRO | 2,030406107 | 2,040470494 | -0,01006439 | |
| 217926_at | C19orf53 | 6,50981793 | 6,519883654 | -0,01006572 | |
| 200631_s_at | SET | 6,294912682 | 6,304996536 | -0,01008385 | |
| 217805_at | ILF3 | 5,096666579 | 5,106765451 | -0,01009887 | |
| 1554509_a_at | FAM188A | 1,912238072 | 1,922365006 | -0,01012693 | |
| 217903_at | STRN4 | 0,911589958 | 0,921731975 | -0,01014202 | |
| 243642_x_at | - | 0,648296226 | 0,658444279 | -0,01014805 | |
| 222756_s_at | ARRB1 | -0,089689931 | -0,079522948 | -0,01016698 | |
| 231412_at | LOC100506342 | -0,089689931 | -0,079522948 | -0,01016698 | |
| 236980_at | STRN | -0,089689931 | -0,079522948 | -0,01016698 | |
| 242087_x_at | MFI2-AS1 | -0,089689931 | -0,079522948 | -0,01016698 | |
| 222445_at | SLC39A9 | 4,256278903 | 4,266446205 | -0,0101673 | |
| 221563_at | DUSP10 | 3,183265014 | 3,193438316 | -0,0101733 | |

| | | | | | |
|---|---|---|---|---|---|
| 225359_at | DNAJC19 | 3,647078281 | 3,6572547 | -0,01017642 | |
| 221569_at | AHI1 | 2,132266459 | 2,14244844 | -0,01018198 | |
| 229981_at | SNX5 | 1,780386218 | 1,790660532 | -0,01027431 | |
| 203764_at | DLGAP5 | 4,585658616 | 4,595981624 | -0,01032301 | |
| 226009_at | DPCD | 4,339030146 | 4,349404898 | -0,01037475 | |
| 221125_s_at | KCNMB3 | 1,314003737 | 1,324440845 | -0,01043711 | |
| 200750_s_at | RAN | 6,845102872 | 6,855546609 | -0,01044374 | |
| 213506_at | F2RL1 | 2,482063962 | 2,492544876 | -0,01048091 | |
| 220157_x_at | PLEKHA8P1 | 1,119359127 | 1,129843209 | -0,01048408 | |
| 205690_s_at | BUD31 | 5,024981547 | 5,035495453 | -0,01051391 | |
| 203135_at | TBP | 3,217273782 | 3,227795991 | -0,01052221 | |
| 219620_x_at | TOR4A | 1,305306849 | 1,315948728 | -0,01064188 | |
| 230934_at | LOC100506057 / | 1,305306849 | 1,315948728 | -0,01064188 | |
| 242451_x_at | RPS19 | 1,305306849 | 1,315948728 | -0,01064188 | |
| 238654_at | VSIG10L | 2,626954721 | 2,637603218 | -0,0106485 | |
| 204565_at | ACOT13 | 4,111638046 | 4,122304108 | -0,01066606 | |
| 222221_x_at | EHD1 | 1,889144008 | 1,899812471 | -0,01066846 | |
| 212723_at | JMJD6 | 3,858765357 | 3,869433939 | -0,01066858 | |
| 1552619_a_at | ANLN | 3,111496958 | 3,122166354 | -0,0106694 | |
| 213318_s_at | BAG6 | 4,827683398 | 4,838353281 | -0,01066988 | |
| 213812_s_at | CAMKK2 | 3,729025365 | 3,739727424 | -0,01070206 | |
| 206766_at | ITGA10 | -0,112808516 | -0,102100538 | -0,01070798 | |
| 211192_s_at | CD84 | -0,112808516 | -0,102100538 | -0,01070798 | |
| 219641_at | DET1 | -0,112808516 | -0,102100538 | -0,01070798 | |
| 223719_s_at | RTBDN | -0,112808516 | -0,102100538 | -0,01070798 | |
| 230944_at | C6orf223 | -0,112808516 | -0,102100538 | -0,01070798 | |
| 241355_at | HR | -0,112808516 | -0,102100538 | -0,01070798 | |
| 244425_at | - | -0,112808516 | -0,102100538 | -0,01070798 | |
| 225170_at | WDR5 | 2,472384965 | 2,483094137 | -0,01070917 | |
| 1553535_a_at | RANGAP1 | 2,001086023 | 2,011838132 | -0,01075211 | |
| 231917_at | GFM2 | 2,001086023 | 2,011838132 | -0,01075211 | |
| 212747_at | ANKS1A | 3,000043914 | 3,010820719 | -0,0107768 | |
| 229366_at | - | 1,621313976 | 1,632095957 | -0,01078198 | |
| 212059_s_at | TRPC4AP | 1,883312263 | 1,894118818 | -0,01080655 | |
| 203344_s_at | RBBP8 | 4,228372617 | 4,239196128 | -0,01082351 | |
| 200065_s_at | ARF1 /// MIR362( | 6,10102707 | 6,111868133 | -0,01084106 | |
| 220302_at | MAK | 3,787958926 | 3,798818761 | -0,01085983 | |
| 1570241_at | SPATA21 | -0,741668563 | -0,730778808 | -0,01088975 | |
| 215310_at | APC | -0,741668563 | -0,730778808 | -0,01088975 | |
| 216589_at | - | -0,741668563 | -0,730778808 | -0,01088975 | |
| 217310_s_at | FOXJ3 | -0,741668563 | -0,730778808 | -0,01088975 | |
| 230242_at | NFASC | -0,741668563 | -0,730778808 | -0,01088975 | |
| 230641_at | LOC100505938 | -0,741668563 | -0,730778808 | -0,01088975 | |
| 234521_at | OR51I2 | -0,741668563 | -0,730778808 | -0,01088975 | |
| 236623_at | ATP1A1OS | -0,741668563 | -0,730778808 | -0,01088975 | |
| 238741_at | FAM83A | -0,741668563 | -0,730778808 | -0,01088975 | |
| 1556442_x_at | - | 2,382221458 | 2,39311332 | -0,01089186 | |
| 205618_at | PRRG1 | 2,817903185 | 2,828804976 | -0,01090179 | |
| 230200_at | NSUN6 | 2,292660284 | 2,303602088 | -0,0109418 | |
| 219551_at | EAF2 | 1,292162421 | 1,303116084 | -0,01095366 | |
| 222786_at | CHST12 | 0,613492853 | 0,624460759 | -0,01096791 | |
| 242756_at | - | 0,613492853 | 0,624460759 | -0,01096791 | |
| 1552615_at | ACACB | 1,748661581 | 1,759685182 | -0,0110236 | |
| 220703_at | IDI2-AS1 | 1,094335495 | 1,105412836 | -0,01107734 | |
| 225057_at | SLC15A4 | 1,094335495 | 1,105412836 | -0,01107734 | |
| 201400_at | PSMB3 | 6,372548281 | 6,383670962 | -0,01112268 | |

| | | | | | |
|---|---|---|---|---|---|
| 209402_s_at | SLC12A4 | 1,283332461 | 1,294497133 | -0,01116467 | |
| 201007_at | HADHB | 5,219930892 | 5,231096511 | -0,01116562 | |
| 203196_at | ABCC4 | 5,010675163 | 5,021853686 | -0,01117852 | |
| 201240_s_at | LOC653566 /// SI | 5,576477597 | 5,587677654 | -0,01120006 | |
| 236381_s_at | WRAP73 | 1,982112682 | 1,993317152 | -0,01120447 | |
| 210541_s_at | TRIM27 | 3,831767101 | 3,843079586 | -0,01131249 | |
| 201482_at | QSOX1 | 2,446911392 | 2,458228496 | -0,0113171 | |
| 200742_s_at | TPP1 | 1,732533761 | 1,743944487 | -0,01141073 | |
| 218726_at | HJURP | 3,630637368 | 3,642048499 | -0,01141113 | |
| 201892_s_at | IMPDH2 | 6,00014224 | 6,01157422 | -0,01143198 | |
| 228211_at | ERCC6L2 | 1,079110292 | 1,090553537 | -0,01144325 | |
| 219546_at | BMP2K | 5,293370663 | 5,304832091 | -0,01146143 | |
| 200036_s_at | RPL10A | 7,30150281 | 7,312968279 | -0,01146547 | |
| 206599_at | SLC16A5 | 0,592200562 | 0,603679647 | -0,01147909 | |
| 232710_at | - | 0,592200562 | 0,603679647 | -0,01147909 | |
| 211261_at | NUP214 | 1,269985355 | 1,281471383 | -0,01148603 | |
| 213230_at | CDR2L | 1,269985355 | 1,281471383 | -0,01148603 | |
| 224783_at | FAM100B | 1,85379461 | 1,865308541 | -0,01151393 | |
| 230492_s_at | GPCPD1 | 2,26825514 | 2,27978304 | -0,0115279 | |
| 1552672_a_at | IGSF3 | -0,148196756 | -0,136643983 | -0,01155277 | |
| 1566784_at | NSF | -0,148196756 | -0,136643983 | -0,01155277 | |
| 209636_at | NFKB2 | -0,148196756 | -0,136643983 | -0,01155277 | |
| 228983_at | - | -0,148196756 | -0,136643983 | -0,01155277 | |
| 238331_at | SPRN | -0,148196756 | -0,136643983 | -0,01155277 | |
| 240497_at | - | -0,148196756 | -0,136643983 | -0,01155277 | |
| 241599_at | LSM11 | -0,148196756 | -0,136643983 | -0,01155277 | |
| 220980_s_at | ADPGK | 3,789518787 | 3,801102381 | -0,01158359 | |
| 203874_s_at | SMARCA1 | 1,722769813 | 1,73441697 | -0,01164716 | |
| 224787_s_at | RAB18 | 4,017604437 | 4,029266691 | -0,01166225 | |
| 213829_x_at | RTEL1 | 1,261018186 | 1,272721751 | -0,01170356 | |
| 223050_s_at | FBXW5 | 1,261018186 | 1,272721751 | -0,01170356 | |
| 224931_at | SLC41A3 | 1,261018186 | 1,272721751 | -0,01170356 | |
| 204948_s_at | FST | 2,166283906 | 2,17802703 | -0,01174312 | |
| 222418_s_at | TMEM43 | 2,259277178 | 2,271023131 | -0,01174595 | |
| 222474_s_at | TOMM22 | 4,632475172 | 4,644266013 | -0,01179084 | |
| 1570153_at | CCDC169 | 0,57782909 | 0,589657387 | -0,0118283 | |
| 201976_s_at | MYO10 | 0,57782909 | 0,589657387 | -0,0118283 | |
| 210018_x_at | MALT1 | 0,57782909 | 0,589657387 | -0,0118283 | |
| 226171_at | ZDHHC3 | 0,57782909 | 0,589657387 | -0,0118283 | |
| 203885_at | RAB21 | 3,870604295 | 3,882432751 | -0,01182846 | |
| 219453_at | KLHL36 | 0,255492549 | 0,26733076 | -0,01183821 | |
| 219611_s_at | CEP85 | 1,954567604 | 1,966439221 | -0,01187162 | |
| 203814_s_at | NQO2 | 5,097611313 | 5,109532018 | -0,0119207 | |
| 222453_at | CYBRD1 | 2,644299732 | 2,656223838 | -0,01192411 | |
| 227002_at | FAM78A | 3,158127685 | 3,170070041 | -0,01194236 | |
| 202530_at | MAPK14 | 3,676882273 | 3,688839808 | -0,01195753 | |
| 201049_s_at | RPS18 | 8,198316508 | 8,210362016 | -0,01204551 | |
| 226871_s_at | ATG4D | 2,333865983 | 2,345926937 | -0,01206095 | |
| 1554345_a_at | GIN1 | 1,05337236 | 1,065442793 | -0,01207043 | |
| 228453_at | EPG5 | 1,567778694 | 1,579853228 | -0,01207453 | |
| 1569105_at | SETD5 | 1,829737795 | 1,841838775 | -0,01210098 | |
| 211378_x_at | LOC100288602 / | 7,648604357 | 7,660737638 | -0,01213328 | |
| 1559979_at | SYF2 | -0,172274514 | -0,160136748 | -0,01213777 | |
| 1568598_at | KAZALD1 | -0,172274514 | -0,160136748 | -0,01213777 | |
| 207343_at | LYZL6 | -0,172274514 | -0,160136748 | -0,01213777 | |
| 207936_x_at | RFPL3 | -0,172274514 | -0,160136748 | -0,01213777 | |

| | | | | | |
|---|---|---|---|---|---|
| 210281_s_at | ZMYM2 | -0,172274514 | -0,160136748 | -0,01213777 | |
| 211635_x_at | IGHA1 /// IGHA2 | -0,172274514 | -0,160136748 | -0,01213777 | |
| 228900_at | SPECC1 | -0,172274514 | -0,160136748 | -0,01213777 | |
| 229505_at | - | -0,172274514 | -0,160136748 | -0,01213777 | |
| 230522_s_at | C9orf100 | -0,172274514 | -0,160136748 | -0,01213777 | |
| 239469_at | - | -0,172274514 | -0,160136748 | -0,01213777 | |
| 241935_at | SHROOM1 | -0,172274514 | -0,160136748 | -0,01213777 | |
| 244686_at | TCOF1 | -0,172274514 | -0,160136748 | -0,01213777 | |
| 208398_s_at | TBPL1 | 4,900226157 | 4,912395116 | -0,01216896 | |
| 227711_at | GTSF1 | 4,306714867 | 4,318918076 | -0,01220321 | |
| 237367_x_at | CFLAR | 2,146943689 | 2,159161028 | -0,01221734 | |
| 202857_at | CNPY2 | 5,158634383 | 5,170858681 | -0,0122243 | |
| 203701_s_at | TRMT1 | 2,994644467 | 3,006869115 | -0,01222465 | |
| 228725_x_at | PRMT2 | 5,108900129 | 5,121154943 | -0,01225481 | |
| 202175_at | CHPF | 0,822971223 | 0,835239052 | -0,01226783 | |
| 210513_s_at | VEGFA | 0,822971223 | 0,835239052 | -0,01226783 | |
| 222657_s_at | UBE2W | 2,559073676 | 2,571362843 | -0,01228917 | |
| 209352_s_at | SIN3B | 2,406821352 | 2,419116683 | -0,01229533 | |
| 225857_s_at | LOC388796 | 4,464437915 | 4,476764847 | -0,01232693 | |
| 213383_at | SBF1 | 0,555999837 | 0,568365095 | -0,01236526 | |
| 201018_at | EIF1AX | 3,848325987 | 3,860702537 | -0,01237655 | |
| 212571_at | CHD8 | 2,134723057 | 2,147243253 | -0,0125202 | |
| 229111_at | - | 1,549482847 | 1,562009954 | -0,01252711 | |
| 227840_at | C2orf76 | 2,396622324 | 2,409170778 | -0,01254845 | |
| 204669_s_at | RNF24 | 4,08266696 | 4,09526554 | -0,01259858 | |
| 211023_at | PDHB | 5,200568591 | 5,213202033 | -0,01263344 | |
| 1552425_a_at | KLHL10 | -0,83191556 | -0,819238336 | -0,01267722 | |
| 1556205_at | - | -0,83191556 | -0,819238336 | -0,01267722 | |
| 1562227_at | PDE5A | -0,83191556 | -0,819238336 | -0,01267722 | |
| 1569067_at | - | -0,83191556 | -0,819238336 | -0,01267722 | |
| 204134_at | PDE2A | -0,83191556 | -0,819238336 | -0,01267722 | |
| 206194_at | HOXC4 | -0,83191556 | -0,819238336 | -0,01267722 | |
| 206243_at | TIMP4 | -0,83191556 | -0,819238336 | -0,01267722 | |
| 217182_at | MUC5AC | -0,83191556 | -0,819238336 | -0,01267722 | |
| 217341_at | DNM1 | -0,83191556 | -0,819238336 | -0,01267722 | |
| 221409_at | OR2S2 | -0,83191556 | -0,819238336 | -0,01267722 | |
| 222812_s_at | RHOF | -0,83191556 | -0,819238336 | -0,01267722 | |
| 228895_s_at | - | -0,83191556 | -0,819238336 | -0,01267722 | |
| 233525_s_at | LINC00475 | -0,83191556 | -0,819238336 | -0,01267722 | |
| 238859_at | - | -0,83191556 | -0,819238336 | -0,01267722 | |
| 217960_s_at | TOMM22 | 4,308891884 | 4,321571774 | -0,01267989 | |
| 223300_s_at | CCDC82 | 3,127619759 | 3,140317145 | -0,01269739 | |
| 218912_at | GCC1 | 2,864529541 | 2,877228323 | -0,01269878 | |
| 201119_s_at | COX8A | 6,065979249 | 6,078682636 | -0,01270339 | |
| 224233_s_at | MSTO1 | 1,027166904 | 1,039887232 | -0,01272033 | |
| 224998_at | CMTM4 | 1,027166904 | 1,039887232 | -0,01272033 | |
| 229328_at | LOC100507433 | 1,027166904 | 1,039887232 | -0,01272033 | |
| 218339_at | MRPL22 | 4,408689531 | 4,42143356 | -0,01274403 | |
| 203651_at | ZFYVE16 | 2,218170265 | 2,230931691 | -0,01276143 | |
| 1555939_at | - | -3,32387526 | -3,311072652 | -0,01280261 | |
| 1561564_at | - | -3,32387526 | -3,311072652 | -0,01280261 | |
| 213764_s_at | MFAP5 | -3,32387526 | -3,311072652 | -0,01280261 | |
| 227630_at | PPP2R5E | 2,122398022 | 2,135226207 | -0,01282818 | |
| 226683_at | SNX18 | 2,536956583 | 2,549796788 | -0,0128402 | |
| 239206_at | CR1L | 1,799091453 | 1,811954245 | -0,01286279 | |
| 226738_at | WDR81 | 2,213529682 | 2,226407528 | -0,01287785 | |

| | | | | | |
|---|---|---|---|---|---|
| 226297_at | HIPK3 | 4,55296954 | 4,565857693 | -0,01288815 | |
| 225703_at | FBRSL1 | 3,671816548 | 3,684723168 | -0,01290662 | |
| 211069_s_at | SUMO1 | 5,412041769 | 5,424948779 | -0,01290701 | |
| 219520_s_at | WWC3 | 1,210676994 | 1,223626587 | -0,01294959 | |
| 224558_s_at | LOC100507645 / | 4,549751571 | 4,562720691 | -0,01296912 | |
| 227173_s_at | BACH2 | 0,209623097 | 0,222599095 | -0,012976 | |
| 213126_at | MED8 | 1,530951994 | 1,543943227 | -0,01299123 | |
| 236117_at | - | 2,206540699 | 2,21959457 | -0,01305387 | |
| 236075_s_at | LOC100506676 | 2,849652795 | 2,862725455 | -0,01307266 | |
| 218388_at | PGLS | 3,526916747 | 3,540009821 | -0,01309307 | |
| 212237_at | ASXL1 | 2,109966786 | 2,123108223 | -0,01314144 | |
| 213360_s_at | POM121 /// POM | 4,725862305 | 4,73902338 | -0,01316107 | |
| 1554451_s_at | DNAJC14 | 1,201332287 | 1,214517879 | -0,01318559 | |
| 204027_s_at | METTL1 | 1,201332287 | 1,214517879 | -0,01318559 | |
| 211584_s_at | NPAT | 1,201332287 | 1,214517879 | -0,01318559 | |
| 212402_at | ZC3H13 | 3,982575608 | 3,995764148 | -0,01318854 | |
| 222606_at | ZWILCH | 4,770777552 | 4,783973541 | -0,01319599 | |
| 216434_at | TTC38 | 0,518867317 | 0,532164371 | -0,01329705 | |
| 217593_at | ZSCAN18 | 0,518867317 | 0,532164371 | -0,01329705 | |
| 238775_at | ITFG1 | 0,518867317 | 0,532164371 | -0,01329705 | |
| 203246_s_at | NPRL2 | 3,102740237 | 3,116064987 | -0,01332475 | |
| 226154_at | DNM1L | 4,195216101 | 4,208556964 | -0,01334086 | |
| 204435_at | NUPL1 | 2,194816625 | 2,208167653 | -0,01335103 | |
| 1561959_x_at | - | -3,287237186 | -3,273861381 | -0,0133758 | |
| 1570561_at | - | -3,287237186 | -3,273861381 | -0,0133758 | |
| 220845_at | ACOXL | -3,287237186 | -3,273861381 | -0,0133758 | |
| 214522_x_at | HIST1H2AD /// H | 4,362800281 | 4,376200741 | -0,01340046 | |
| 224657_at | ERRFI1 | 2,512607472 | 2,526063925 | -0,01345645 | |
| 225440_at | AGPAT3 | 4,047898031 | 4,061373942 | -0,01347591 | |
| 211762_s_at | KPNA2 | 6,394368053 | 6,407847031 | -0,01347898 | |
| 228839_s_at | LOC642361 | 3,316904202 | 3,330438773 | -0,01353457 | |
| 212496_s_at | KDM4B | 3,396853889 | 3,410394103 | -0,01354021 | |
| 232737_s_at | ENPP3 | 2,577249059 | 2,590852465 | -0,01360341 | |
| 218168_s_at | ADCK3 | 3,506195778 | 3,519816196 | -0,01362042 | |
| 212450_at | SECISBP2L | 2,089851283 | 2,103505212 | -0,01365393 | |
| 231402_at | - | 0,503742507 | 0,51742585 | -0,01368334 | |
| 1569807_at | - | -0,234278768 | -0,220592223 | -0,01368654 | |
| 205382_s_at | CFD | -0,234278768 | -0,220592223 | -0,01368654 | |
| 217647_at | - | -0,234278768 | -0,220592223 | -0,01368654 | |
| 223685_s_at | PRPF18 | -0,234278768 | -0,220592223 | -0,01368654 | |
| 229612_at | - | -0,234278768 | -0,220592223 | -0,01368654 | |
| 231818_x_at | - | -0,234278768 | -0,220592223 | -0,01368654 | |
| 232633_at | XRCC5 | -0,234278768 | -0,220592223 | -0,01368654 | |
| 225365_at | ZDHHC20 | 3,96128107 | 3,975013041 | -0,01373197 | |
| 228733_at | PUSL1 | 2,084778276 | 2,098562556 | -0,01378428 | |
| 213410_at | C10orf137 | 3,345817457 | 3,359653127 | -0,01383567 | |
| 1565717_s_at | FUS | 4,34540724 | 4,359253484 | -0,01384624 | |
| 242323_at | PLA2G12A | 3,032022986 | 3,045906378 | -0,01388339 | |
| 216602_s_at | FARSA | 3,493810203 | 3,507749374 | -0,01393917 | |
| 1560372_at | - | -3,250025915 | -3,236070201 | -0,01395571 | |
| 1561069_at | - | -3,250025915 | -3,236070201 | -0,01395571 | |
| 1562800_at | - | -3,250025915 | -3,236070201 | -0,01395571 | |
| 222299_x_at | - | -3,250025915 | -3,236070201 | -0,01395571 | |
| 240469_at | - | -3,250025915 | -3,236070201 | -0,01395571 | |
| 241147_at | - | -3,250025915 | -3,236070201 | -0,01395571 | |
| 205125_at | PLCD1 | 1,493160564 | 1,507116528 | -0,01395596 | |

| | | | | | |
|---|---|---|---|---|---|
| 209654_at | KIAA0947 | 3,630637368 | 3,644594022 | -0,01395665 | |
| 222619_at | ZNF281 | 3,170152481 | 3,184136378 | -0,0139839 | |
| 208870_x_at | ATP5C1 | 5,902611293 | 5,916653471 | -0,01404218 | |
| 201052_s_at | PSMF1 | 3,413135307 | 3,427247355 | -0,01411205 | |
| 203679_at | TMED1 | 4,779830653 | 4,79394496 | -0,01411431 | |
| 227211_at | PHF19 | 3,62365843 | 3,637795954 | -0,01413752 | |
| 226770_at | MAGI3 | 1,163336153 | 1,177496821 | -0,01416067 | |
| 223120_at | FUCA2 | 4,520462208 | 4,534633949 | -0,01417174 | |
| 204725_s_at | NCK1 | 2,250242994 | 2,264418116 | -0,01417512 | |
| 226790_at | MORN2 | 2,069451339 | 2,083632167 | -0,01418083 | |
| 224947_at | RNF26 | 2,331726354 | 2,345926937 | -0,01420058 | |
| 201037_at | PFKP | 4,519052534 | 4,533260944 | -0,01420841 | |
| 217140_s_at | VDAC1 | 4,389788927 | 4,404011141 | -0,01422221 | |
| 221940_at | RPUSD2 | 2,064305961 | 2,078620836 | -0,01431488 | |
| 205217_at | TIMM8A | 2,156646206 | 2,170981159 | -0,01433495 | |
| 201600_at | PHB2 | 6,275424489 | 6,289763693 | -0,0143392 | |
| 218905_at | INTS8 | 4,725862305 | 4,74021396 | -0,01435165 | |
| 1555374_at | - | 0,962260738 | 0,976641161 | -0,01438042 | |
| 203663_s_at | COX5A | 6,489927858 | 6,504376305 | -0,01444845 | |
| 207438_s_at | SNUPN | 3,735513868 | 3,75000829 | -0,01449442 | |
| 200088_x_at | LOC648771 /// NI | 7,673686286 | 7,688191905 | -0,01450562 | |
| 1560806_at | LOC646743 /// TI | -0,926850548 | -0,912331589 | -0,01451896 | |
| 1561459_at | - | -0,926850548 | -0,912331589 | -0,01451896 | |
| 214028_x_at | TDRD3 | -0,926850548 | -0,912331589 | -0,01451896 | |
| 214236_at | - | -0,926850548 | -0,912331589 | -0,01451896 | |
| 216329_at | - | -0,926850548 | -0,912331589 | -0,01451896 | |
| 216762_at | - | -0,926850548 | -0,912331589 | -0,01451896 | |
| 222046_at | SRRT | -0,926850548 | -0,912331589 | -0,01451896 | |
| 228387_at | - | -0,926850548 | -0,912331589 | -0,01451896 | |
| 230354_at | - | -0,926850548 | -0,912331589 | -0,01451896 | |
| 231210_at | C11orf85 | -0,926850548 | -0,912331589 | -0,01451896 | |
| 232243_at | MCPH1 | -0,926850548 | -0,912331589 | -0,01451896 | |
| 233418_at | LOC91450 | -0,926850548 | -0,912331589 | -0,01451896 | |
| 235055_x_at | MUC4 | -0,926850548 | -0,912331589 | -0,01451896 | |
| 235212_at | C14orf102 | -0,926850548 | -0,912331589 | -0,01451896 | |
| 218528_s_at | RNF38 | 3,358484112 | 3,373018423 | -0,01453431 | |
| 200681_at | GLO1 | 6,661906497 | 6,676514997 | -0,0146085 | |
| 225298_at | PNKD | 2,952099138 | 2,966746113 | -0,01464698 | |
| 221788_at | PGM3 | 2,14450783 | 2,159161028 | -0,0146532 | |
| 204370_at | CLP1 | 4,166691359 | 4,181349409 | -0,01465805 | |
| 206077_at | KEL | 1,46610492 | 1,480766833 | -0,01466191 | |
| 228306_at | CNIH4 | 1,46610492 | 1,480766833 | -0,01466191 | |
| 225003_at | TMEM205 | 5,273443519 | 5,288105861 | -0,01466234 | |
| 204391_x_at | TRIM24 | 4,570314367 | 4,584979629 | -0,01466526 | |
| 203016_s_at | SSX2IP | 3,143564369 | 3,158242412 | -0,01467804 | |
| 211328_x_at | HFE | 0,46522187 | 0,479907041 | -0,01468517 | |
| 1552379_at | RDH10 | -3,192553003 | -3,177802286 | -0,01475072 | |
| 1555756_a_at | CLEC7A | -3,192553003 | -3,177802286 | -0,01475072 | |
| 1555926_a_at | - | -3,192553003 | -3,177802286 | -0,01475072 | |
| 1560066_at | - | -3,192553003 | -3,177802286 | -0,01475072 | |
| 1560383_at | LOC100506387 | -3,192553003 | -3,177802286 | -0,01475072 | |
| 1565923_at | - | -3,192553003 | -3,177802286 | -0,01475072 | |
| 1570355_a_at | DPY19L1 | -3,192553003 | -3,177802286 | -0,01475072 | |
| 214254_at | MAGEA4 | -3,192553003 | -3,177802286 | -0,01475072 | |
| 244621_x_at | - | -3,192553003 | -3,177802286 | -0,01475072 | |
| 218171_at | VPS4B | 3,310399576 | 3,325159096 | -0,01475952 | |

| | | | | | |
|---|---|---|---|---|---|
| 204245_s_at | RPP14 | 2,783874823 | 2,798646381 | -0,01477156 | |
| 225995_x_at | WASH1 /// WASH | 2,724770722 | 2,739547836 | -0,01477711 | |
| 225777_at | SAPCD2 | 2,663141434 | 2,677924589 | -0,01478316 | |
| 235793_at | - | 1,139070151 | 1,153866761 | -0,01479661 | |
| 215037_s_at | BCL2L1 | 0,945567441 | 0,960386719 | -0,01481928 | |
| 225245_x_at | H2AFJ | 4,06796029 | 4,082805739 | -0,01484545 | |
| 217815_at | SUPT16H | 5,30704878 | 5,321896852 | -0,01484807 | |
| 217982_s_at | MORF4L1 | 6,798705596 | 6,813558862 | -0,01485327 | |
| 217329_x_at | - | 4,34540724 | 4,360286277 | -0,01487904 | |
| 225324_at | CRLS1 | 3,992090425 | 4,007018329 | -0,0149279 | |
| 207431_s_at | DEGS1 | 3,49093683 | 3,505883949 | -0,01494712 | |
| 204798_at | MYB | 6,341515089 | 6,356497318 | -0,01498223 | |
| 229348_at | UBIAD1 | 2,2992452 | 2,314300277 | -0,01505508 | |
| 1556852_a_at | LOC100506609 | 1,129248302 | 1,144305291 | -0,01505699 | |
| 1564356_at | ZNF568 | 1,129248302 | 1,144305291 | -0,01505699 | |
| 212976_at | LRRC8B | 1,129248302 | 1,144305291 | -0,01505699 | |
| 211036_x_at | ANAPC5 | 4,883890602 | 4,898954113 | -0,01506351 | |
| 208336_s_at | TECR | 5,345732318 | 5,360860774 | -0,01512846 | |
| 210104_at | MED6 | 4,148572682 | 4,163710704 | -0,01513802 | |
| 41858_at | PGAP2 | 2,768928944 | 2,784097152 | -0,01516821 | |
| 213969_x_at | RPL29 | 7,658187996 | 7,673361925 | -0,01517393 | |
| 1555226_s_at | C1orf43 | 4,332089668 | 4,347322916 | -0,01523325 | |
| 222409_at | CORO1C | 5,9738003 | 5,989049182 | -0,01524888 | |
| 201351_s_at | YME1L1 | 5,261409968 | 5,276663999 | -0,01525403 | |
| 212312_at | BCL2L1 | 3,612245157 | 3,62753841 | -0,01529325 | |
| 210254_at | MS4A3 | 1,820612054 | 1,835911187 | -0,01529913 | |
| 202975_s_at | RHOBTB3 | 3,734704399 | 3,75000829 | -0,01530389 | |
| 202328_s_at | PKD1 | 0,44160593 | 0,456918297 | -0,01531237 | |
| 1556849_at | - | -3,15396682 | -3,138626879 | -0,01533994 | |
| 1557014_a_at | FAM201A | -3,15396682 | -3,138626879 | -0,01533994 | |
| 1559584_a_at | C16orf54 | -3,15396682 | -3,138626879 | -0,01533994 | |
| 1566266_at | - | -3,15396682 | -3,138626879 | -0,01533994 | |
| 1566637_at | - | -3,15396682 | -3,138626879 | -0,01533994 | |
| 1569494_at | MIPOL1 | -3,15396682 | -3,138626879 | -0,01533994 | |
| 1569886_a_at | GLB1L3 | -3,15396682 | -3,138626879 | -0,01533994 | |
| 1570575_at | - | -3,15396682 | -3,138626879 | -0,01533994 | |
| 207678_s_at | SOX30 | -3,15396682 | -3,138626879 | -0,01533994 | |
| 216623_x_at | TOX3 | -3,15396682 | -3,138626879 | -0,01533994 | |
| 216672_s_at | MYT1L | -3,15396682 | -3,138626879 | -0,01533994 | |
| 235118_at | CADM2 | -3,15396682 | -3,138626879 | -0,01533994 | |
| 236608_at | GPR113 | -3,15396682 | -3,138626879 | -0,01533994 | |
| 236637_at | TAS2R14 | -3,15396682 | -3,138626879 | -0,01533994 | |
| 237253_at | IGSF11-AS1 | -3,15396682 | -3,138626879 | -0,01533994 | |
| 239667_at | SLC3A1 | -3,15396682 | -3,138626879 | -0,01533994 | |
| 240433_x_at | CADM2 | -3,15396682 | -3,138626879 | -0,01533994 | |
| 240442_at | - | -3,15396682 | -3,138626879 | -0,01533994 | |
| 242248_at | PHKB | -3,15396682 | -3,138626879 | -0,01533994 | |
| 242795_at | - | -3,15396682 | -3,138626879 | -0,01533994 | |
| 1554119_at | C16orf57 | -0,299034815 | -0,283667828 | -0,01536699 | |
| 1558975_at | FAM100A | -0,299034815 | -0,283667828 | -0,01536699 | |
| 1569349_at | C11orf30 | -0,299034815 | -0,283667828 | -0,01536699 | |
| 217646_at | SURF1 | -0,299034815 | -0,283667828 | -0,01536699 | |
| 238811_at | ATP11B | -0,299034815 | -0,283667828 | -0,01536699 | |
| 239112_at | - | -0,299034815 | -0,283667828 | -0,01536699 | |
| 243255_at | - | -0,299034815 | -0,283667828 | -0,01536699 | |
| 201753_s_at | ADD3 | 4,343815608 | 4,359253484 | -0,01543788 | |

| | | | | | |
|---|---|---|---|---|---|
| 1568987_at | MGC57346 | 1,114389003 | 1,129843209 | -0,01545421 | |
| 242692_at | - | 1,114389003 | 1,129843209 | -0,01545421 | |
| 224630_at | ERLEC1 | 3,943876123 | 3,959421856 | -0,01554573 | |
| 219242_at | CEP63 | 1,69307511 | 1,708698295 | -0,01562318 | |
| 209702_at | FTO | 3,152077493 | 3,16771226 | -0,01563477 | |
| 217777_s_at | PTPLAD1 | 3,888182512 | 3,903840511 | -0,015658 | |
| 210139_s_at | PMP22 | 5,08337493 | 5,099052587 | -0,01567766 | |
| 209080_x_at | GLRX3 | 5,858148329 | 5,873902629 | -0,0157543 | |
| 202926_at | NBAS | 2,857110344 | 2,87289275 | -0,01578241 | |
| 208852_s_at | CANX | 4,758883134 | 4,77470758 | -0,01582445 | |
| 202391_at | BASP1 | 3,744388178 | 3,760216412 | -0,01582823 | |
| 225276_at | GSPT1 | 2,003776245 | 2,019703483 | -0,01592724 | |
| 1553915_at | C10orf126 | -3,114791413 | -3,098858316 | -0,0159331 | |
| 1555103_s_at | FGF7 | -3,114791413 | -3,098858316 | -0,0159331 | |
| 1560494_a_at | CPXCR1 | -3,114791413 | -3,098858316 | -0,0159331 | |
| 1561562_at | - | -3,114791413 | -3,098858316 | -0,0159331 | |
| 1566324_a_at | MAF | -3,114791413 | -3,098858316 | -0,0159331 | |
| 206950_at | SCN9A | -3,114791413 | -3,098858316 | -0,0159331 | |
| 212681_at | EPB41L3 | -3,114791413 | -3,098858316 | -0,0159331 | |
| 215663_at | MBNL1 | -3,114791413 | -3,098858316 | -0,0159331 | |
| 216462_at | - | -3,114791413 | -3,098858316 | -0,0159331 | |
| 220810_at | CLCA3P | -3,114791413 | -3,098858316 | -0,0159331 | |
| 222940_at | SULT1E1 | -3,114791413 | -3,098858316 | -0,0159331 | |
| 224167_at | SPZ1 | -3,114791413 | -3,098858316 | -0,0159331 | |
| 233789_at | - | -3,114791413 | -3,098858316 | -0,0159331 | |
| 236335_at | GUCY1A2 | -3,114791413 | -3,098858316 | -0,0159331 | |
| 240915_at | IGHV1-69 | -3,114791413 | -3,098858316 | -0,0159331 | |
| 241796_x_at | - | -3,114791413 | -3,098858316 | -0,0159331 | |
| 225232_at | MTMR12 | 3,588248178 | 3,604188755 | -0,01594058 | |
| 1554424_at | FIP1L1 | 0,417596971 | 0,433557298 | -0,01596033 | |
| 229633_at | INTS10 | 0,417596971 | 0,433557298 | -0,01596033 | |
| 218341_at | PPCS | 2,094906514 | 2,110887591 | -0,01598108 | |
| 227129_x_at | FLJ45340 | 2,901063789 | 2,917077481 | -0,01601369 | |
| 214857_at | LOC100505761 | 1,414471577 | 1,43051703 | -0,01604545 | |
| 220761_s_at | TAOK3 | 4,114129991 | 4,130196752 | -0,01606676 | |
| 210480_s_at | MYO6 | 0,67554678 | 0,691645685 | -0,01609891 | |
| 210379_s_at | TLK1 | 1,089278259 | 1,105412836 | -0,01613458 | |
| 221610_s_at | STAP2 | 1,089278259 | 1,105412836 | -0,01613458 | |
| 200940_s_at | RERE | 3,298397722 | 3,314541423 | -0,0161437 | |
| 219078_at | GPATCH2 | 1,995690482 | 2,011838132 | -0,01614765 | |
| 219983_at | HRASLS | 2,787001691 | 2,803210557 | -0,01620887 | |
| 231041_at | POLR1E | 0,888485433 | 0,904843258 | -0,01635783 | |
| 1560128_x_at | C5orf56 | -1,026571149 | -1,010192375 | -0,01637877 | |
| 203948_s_at | MPO | -1,026571149 | -1,010192375 | -0,01637877 | |
| 204072_s_at | FRY | -1,026571149 | -1,010192375 | -0,01637877 | |
| 206259_at | PROC | -1,026571149 | -1,010192375 | -0,01637877 | |
| 207466_at | GAL | -1,026571149 | -1,010192375 | -0,01637877 | |
| 210961_s_at | ADRA1D | -1,026571149 | -1,010192375 | -0,01637877 | |
| 211900_x_at | CD6 | -1,026571149 | -1,010192375 | -0,01637877 | |
| 213874_at | SERPINA4 | -1,026571149 | -1,010192375 | -0,01637877 | |
| 214815_at | TRIM33 | -1,026571149 | -1,010192375 | -0,01637877 | |
| 215839_at | LILRA5 | -1,026571149 | -1,010192375 | -0,01637877 | |
| 220272_at | BNC2 | -1,026571149 | -1,010192375 | -0,01637877 | |
| 227706_at | SPIRE2 | -1,026571149 | -1,010192375 | -0,01637877 | |
| 236428_at | - | -1,026571149 | -1,010192375 | -0,01637877 | |
| 236990_at | LOC100507360 | -1,026571149 | -1,010192375 | -0,01637877 | |

| | | | | | |
|---|---|---|---|---|---|
| 241466_at | - | -1,026571149 | -1,010192375 | -0,01637877 | |
| 243382_at | CCDC85A | -1,026571149 | -1,010192375 | -0,01637877 | |
| 244100_at | - | -1,026571149 | -1,010192375 | -0,01637877 | |
| 244603_at | - | -1,026571149 | -1,010192375 | -0,01637877 | |
| 201390_s_at | CSNK2B | 5,861489288 | 5,877874624 | -0,01638534 | |
| 202540_s_at | HMGCR | 4,034147209 | 4,050537457 | -0,01639025 | |
| 238773_at | METTL15 | 1,780386218 | 1,796776588 | -0,01639037 | |
| 200819_s_at | RPS15 | 7,961298109 | 7,977724004 | -0,0164259 | |
| 242655_at | BARD1 | 0,984222186 | 1,000684521 | -0,01646234 | |
| 228992_at | MED28 | 3,600740871 | 3,617207412 | -0,01646654 | |
| 221842_s_at | LOC100506639 / | 2,983784569 | 3,000258961 | -0,01647439 | |
| 205664_at | KIN | 3,32445605 | 3,340940512 | -0,01648446 | |
| 204263_s_at | CPT2 | 1,883312263 | 1,899812471 | -0,01650021 | |
| 227131_at | MAP3K3 | 2,774453351 | 2,791007185 | -0,01655383 | |
| 228714_at | - | 1,073999308 | 1,090553537 | -0,01655423 | |
| 231769_at | FBXO6 | 1,073999308 | 1,090553537 | -0,01655423 | |
| 206951_at | HIST1H4A /// HIS | 1,394109935 | 1,410714371 | -0,01660444 | |
| 209799_at | PRKAA1 | 1,394109935 | 1,410714371 | -0,01660444 | |
| 201848_s_at | BNIP3 | 2,874852734 | 2,891586849 | -0,01673411 | |
| 1555718_x_at | - | -3,054236653 | -3,037500868 | -0,01673578 | |
| 1559965_at | ZFHX4-AS1 | -3,054236653 | -3,037500868 | -0,01673578 | |
| 1560791_at | - | -3,054236653 | -3,037500868 | -0,01673578 | |
| 1561213_at | - | -3,054236653 | -3,037500868 | -0,01673578 | |
| 1563335_at | IRGM | -3,054236653 | -3,037500868 | -0,01673578 | |
| 1569784_at | - | -3,054236653 | -3,037500868 | -0,01673578 | |
| 207928_s_at | GLRA3 | -3,054236653 | -3,037500868 | -0,01673578 | |
| 211155_s_at | THPO | -3,054236653 | -3,037500868 | -0,01673578 | |
| 211164_at | EPHA3 | -3,054236653 | -3,037500868 | -0,01673578 | |
| 217345_at | - | -3,054236653 | -3,037500868 | -0,01673578 | |
| 228821_at | ST6GAL2 | -3,054236653 | -3,037500868 | -0,01673578 | |
| 234808_x_at | - | -3,054236653 | -3,037500868 | -0,01673578 | |
| 236999_at | PPWD1 | -3,054236653 | -3,037500868 | -0,01673578 | |
| 237984_x_at | - | -3,054236653 | -3,037500868 | -0,01673578 | |
| 239806_at | - | -3,054236653 | -3,037500868 | -0,01673578 | |
| 241265_x_at | - | -3,054236653 | -3,037500868 | -0,01673578 | |
| 243040_at | - | -3,054236653 | -3,037500868 | -0,01673578 | |
| 243257_at | - | -3,054236653 | -3,037500868 | -0,01673578 | |
| 244111_at | KRT222 | -3,054236653 | -3,037500868 | -0,01673578 | |
| 244412_at | - | -3,054236653 | -3,037500868 | -0,01673578 | |
| 201613_s_at | AP1G2 | 2,314494121 | 2,331254065 | -0,01675994 | |
| 226875_at | DOCK11 | 3,55745065 | 3,574237491 | -0,01678684 | |
| 202014_at | PPP1R15A | 2,922548342 | 2,939363796 | -0,01681545 | |
| 203753_at | TCF4 | 1,764611101 | 1,781437557 | -0,01682646 | |
| 1554468_s_at | MRPL38 | 1,063722698 | 1,080561626 | -0,01683893 | |
| 217894_at | KCTD3 | 1,063722698 | 1,080561626 | -0,01683893 | |
| 232478_at | MIR181A2HG | 1,063722698 | 1,080561626 | -0,01683893 | |
| 202061_s_at | SEL1L | 3,676882273 | 3,693764321 | -0,01688205 | |
| 223180_s_at | TIMM21 | 5,027297124 | 5,044197531 | -0,01690041 | |
| 210183_x_at | PNN | 6,351449883 | 6,368352567 | -0,01690268 | |
| 227936_at | TMEM68 | 2,918276951 | 2,935211251 | -0,0169343 | |
| 213026_at | ATG12 | 2,450859799 | 2,467842989 | -0,01698319 | |
| 218089_at | C20orf4 | 3,22766727 | 3,244672787 | -0,01700552 | |
| 228089_x_at | TMEM179B | 2,81177561 | 2,828804976 | -0,01702937 | |
| 215190_at | EIF3M | 0,641402394 | 0,658444279 | -0,01704188 | |
| 216694_at | - | 0,641402394 | 0,658444279 | -0,01704188 | |
| 217007_s_at | ADAM15 | 0,641402394 | 0,658444279 | -0,01704188 | |

| | | | | | |
|---|---|---|---|---|---|
| 230541_at | LOC149134 | 0,641402394 | 0,658444279 | -0,01704188 | |
| 208798_x_at | GOLGA8A | 3,700289946 | 3,717332003 | -0,01704206 | |
| 1553499_s_at | SERPINA9 | 0,376672223 | 0,393761504 | -0,01708928 | |
| 219801_at | ZNF34 | 0,376672223 | 0,393761504 | -0,01708928 | |
| 220465_at | LOC80054 | 0,376672223 | 0,393761504 | -0,01708928 | |
| 229405_at | KIF7 | 0,376672223 | 0,393761504 | -0,01708928 | |
| 230376_at | GTF2A2 | 0,376672223 | 0,393761504 | -0,01708928 | |
| 201751_at | JOSD1 | 3,098971025 | 3,116064987 | -0,01709396 | |
| 212936_at | FAM172A | 2,37600538 | 2,39311332 | -0,01710794 | |
| 209155_s_at | NT5C2 | 3,834791978 | 3,85191797 | -0,01712599 | |
| 218280_x_at | HIST2H2AA3 /// H | 6,467873458 | 6,485035874 | -0,01716242 | |
| 1570167_at | - | -0,366776884 | -0,34958478 | -0,0171921 | |
| 206847_s_at | HOXA7 | -0,366776884 | -0,34958478 | -0,0171921 | |
| 207360_s_at | NTSR1 | -0,366776884 | -0,34958478 | -0,0171921 | |
| 219471_at | KIAA0226L | -0,366776884 | -0,34958478 | -0,0171921 | |
| 224554_at | GHSR | -0,366776884 | -0,34958478 | -0,0171921 | |
| 228625_at | CITED4 | -0,366776884 | -0,34958478 | -0,0171921 | |
| 243411_at | POLDIP3 | -0,366776884 | -0,34958478 | -0,0171921 | |
| 220233_at | FBXO17 /// SARS | 2,220484971 | 2,237691445 | -0,01720647 | |
| 210983_s_at | MCM7 | 4,389274648 | 4,406512977 | -0,01723833 | |
| 204109_s_at | NFYA | 2,048758794 | 2,066015817 | -0,01725702 | |
| 214993_at | ASPHD1 | 1,04816921 | 1,065442793 | -0,01727358 | |
| 203198_at | CDK9 | 1,631786706 | 1,64909809 | -0,01731138 | |
| 208972_s_at | ATP5G1 | 4,854012234 | 4,871328311 | -0,01731608 | |
| 1559285_at | - | -3,013665402 | -2,996335703 | -0,0173297 | |
| 1559678_s_at | LOC100652791 / | -3,013665402 | -2,996335703 | -0,0173297 | |
| 1567861_at | DNAH14 | -3,013665402 | -2,996335703 | -0,0173297 | |
| 1569599_at | SAMSN1 | -3,013665402 | -2,996335703 | -0,0173297 | |
| 1569955_at | - | -3,013665402 | -2,996335703 | -0,0173297 | |
| 206898_at | CDH19 | -3,013665402 | -2,996335703 | -0,0173297 | |
| 208143_s_at | EDDM3A | -3,013665402 | -2,996335703 | -0,0173297 | |
| 222150_s_at | PION | -3,013665402 | -2,996335703 | -0,0173297 | |
| 227827_at | - | -3,013665402 | -2,996335703 | -0,0173297 | |
| 231630_at | - | -3,013665402 | -2,996335703 | -0,0173297 | |
| 237390_at | - | -3,013665402 | -2,996335703 | -0,0173297 | |
| 237859_at | - | -3,013665402 | -2,996335703 | -0,0173297 | |
| 237962_x_at | - | -3,013665402 | -2,996335703 | -0,0173297 | |
| 240348_at | - | -3,013665402 | -2,996335703 | -0,0173297 | |
| 241260_at | - | -3,013665402 | -2,996335703 | -0,0173297 | |
| 242321_at | PTPN14 | -3,013665402 | -2,996335703 | -0,0173297 | |
| 223367_at | DNAJC30 | 2,365585359 | 2,382985886 | -0,01740053 | |
| 223728_at | CENPBD1 | 2,213529682 | 2,230931691 | -0,01740201 | |
| 225424_at | GPAM | 2,213529682 | 2,230931691 | -0,01740201 | |
| 213377_x_at | RPS12 | 7,918634015 | 7,936077964 | -0,01744395 | |
| 229050_s_at | SNHG7 /// SNOR | 2,945117939 | 2,962671751 | -0,01755381 | |
| 1553845_x_at | C10orf67 | 0,359971644 | 0,377530647 | -0,017559 | |
| 221552_at | ABHD6 | 0,359971644 | 0,377530647 | -0,017559 | |
| 232004_at | HNRNPR | 1,037706275 | 1,055274826 | -0,01756855 | |
| 201375_s_at | PPP2CB | 5,614596381 | 5,632180175 | -0,01758379 | |
| 214023_x_at | TUBB2B | 3,461883885 | 3,479511525 | -0,01762764 | |
| 210840_s_at | IQGAP1 | 2,493593689 | 2,511262629 | -0,01766894 | |
| 201122_x_at | EIF5A | 5,940488937 | 5,958173867 | -0,01768493 | |
| 229676_at | MTPAP | 2,840652551 | 2,858346012 | -0,01769346 | |
| 219053_s_at | VPS37C | 3,030704581 | 3,048471686 | -0,0177671 | |
| 1559382_at | C19orf42 | 1,352503698 | 1,370274469 | -0,01777077 | |
| 211851_x_at | BRCA1 | 1,352503698 | 1,370274469 | -0,01777077 | |

| | | | | | |
|---|---|---|---|---|---|
| 216678_at | IFT122 | 1,352503698 | 1,370274469 | -0,01777077 | |
| 218202_x_at | MRPL44 | 1,352503698 | 1,370274469 | -0,01777077 | |
| 225860_at | - | 1,352503698 | 1,370274469 | -0,01777077 | |
| 229741_at | MAVS | 1,352503698 | 1,370274469 | -0,01777077 | |
| 202726_at | LIG1 | 2,553576109 | 2,571362843 | -0,01778673 | |
| 202259_s_at | N4BP2L2 | 2,549899391 | 2,567790814 | -0,01789142 | |
| 207469_s_at | PIR | 1,932146506 | 1,950068772 | -0,01792227 | |
| 1552439_s_at | MEGF11 | -2,972500237 | -2,954576755 | -0,01792348 | |
| 1554564_a_at | SPPL3 | -2,972500237 | -2,954576755 | -0,01792348 | |
| 1556625_a_at | - | -2,972500237 | -2,954576755 | -0,01792348 | |
| 1557717_at | LINC00550 | -2,972500237 | -2,954576755 | -0,01792348 | |
| 1558797_at | - | -2,972500237 | -2,954576755 | -0,01792348 | |
| 1560419_at | LOC100127974 | -2,972500237 | -2,954576755 | -0,01792348 | |
| 1560426_at | C12orf55 /// C12c | -2,972500237 | -2,954576755 | -0,01792348 | |
| 1562613_at | - | -2,972500237 | -2,954576755 | -0,01792348 | |
| 1563797_at | - | -2,972500237 | -2,954576755 | -0,01792348 | |
| 1564359_a_at | LOC339260 | -2,972500237 | -2,954576755 | -0,01792348 | |
| 1568936_a_at | - | -2,972500237 | -2,954576755 | -0,01792348 | |
| 1570115_at | PP12708 | -2,972500237 | -2,954576755 | -0,01792348 | |
| 205413_at | MPPED2 | -2,972500237 | -2,954576755 | -0,01792348 | |
| 207867_at | PAX4 | -2,972500237 | -2,954576755 | -0,01792348 | |
| 210035_s_at | RPL5 /// SNORA6 | -2,972500237 | -2,954576755 | -0,01792348 | |
| 211179_at | LOC100506403 / | -2,972500237 | -2,954576755 | -0,01792348 | |
| 214034_at | ERAP1 | -2,972500237 | -2,954576755 | -0,01792348 | |
| 217220_at | LOC100287387 | -2,972500237 | -2,954576755 | -0,01792348 | |
| 217536_x_at | - | -2,972500237 | -2,954576755 | -0,01792348 | |
| 221170_at | HRH4 | -2,972500237 | -2,954576755 | -0,01792348 | |
| 222073_at | COL4A3 | -2,972500237 | -2,954576755 | -0,01792348 | |
| 223841_s_at | SPATA9 | -2,972500237 | -2,954576755 | -0,01792348 | |
| 233238_s_at | - | -2,972500237 | -2,954576755 | -0,01792348 | |
| 235385_at | 01.03.15 | -2,972500237 | -2,954576755 | -0,01792348 | |
| 236936_at | - | -2,972500237 | -2,954576755 | -0,01792348 | |
| 237449_at | SP8 | -2,972500237 | -2,954576755 | -0,01792348 | |
| 237956_s_at | C5orf58 | -2,972500237 | -2,954576755 | -0,01792348 | |
| 238399_x_at | - | -2,972500237 | -2,954576755 | -0,01792348 | |
| 238899_at | - | -2,972500237 | -2,954576755 | -0,01792348 | |
| 240563_at | - | -2,972500237 | -2,954576755 | -0,01792348 | |
| 240676_at | - | -2,972500237 | -2,954576755 | -0,01792348 | |
| 241186_at | - | -2,972500237 | -2,954576755 | -0,01792348 | |
| 241643_at | TLK1 | -2,972500237 | -2,954576755 | -0,01792348 | |
| 205956_x_at | PSMC3IP | 2,194816625 | 2,212749286 | -0,01793266 | |
| 201967_at | RBM6 | 2,882181479 | 2,900133867 | -0,01795239 | |
| 206752_s_at | DFFB | 2,022468633 | 2,040470494 | -0,01800186 | |
| 225358_at | DNAJC19 | 2,022468633 | 2,040470494 | -0,01800186 | |
| 235693_at | - | 2,022468633 | 2,040470494 | -0,01800186 | |
| 204483_at | ENO3 | 1,021868205 | 1,039887232 | -0,01801903 | |
| 227581_at | TECPR1 | 1,021868205 | 1,039887232 | -0,01801903 | |
| 228919_at | - | 0,82905153 | 0,847075464 | -0,01802393 | |
| 1558459_s_at | LOC401320 | 0,606430281 | 0,624460759 | -0,01803048 | |
| 210224_at | MR1 | 0,606430281 | 0,624460759 | -0,01803048 | |
| 231419_at | LOC100505555 | 0,606430281 | 0,624460759 | -0,01803048 | |
| 200615_s_at | AP2B1 | 3,41111015 | 3,429217209 | -0,01810706 | |
| 201267_s_at | PSMC3 | 4,586556157 | 4,604723207 | -0,01816705 | |
| 203250_at | SCAF8 | 4,586556157 | 4,604723207 | -0,01816705 | |
| 1553171_x_at | LRRN4 | -1,131062212 | -1,112851844 | -0,01821037 | |
| 1553204_at | C20orf166-AS1 | -1,131062212 | -1,112851844 | -0,01821037 | |

| | | | | | |
|---|---|---|---|---|---|
| 1553396_a_at | CCDC13 | -1,131062212 | -1,112851844 | -0,01821037 | |
| 1553917_at | PIKFYVE | -1,131062212 | -1,112851844 | -0,01821037 | |
| 1555464_at | IFIH1 | -1,131062212 | -1,112851844 | -0,01821037 | |
| 1561539_at | LOC100506368 | -1,131062212 | -1,112851844 | -0,01821037 | |
| 1563081_at | - | -1,131062212 | -1,112851844 | -0,01821037 | |
| 1564291_at | - | -1,131062212 | -1,112851844 | -0,01821037 | |
| 202921_s_at | ANK2 | -1,131062212 | -1,112851844 | -0,01821037 | |
| 214516_at | HIST1H4A /// HIS | -1,131062212 | -1,112851844 | -0,01821037 | |
| 220404_at | GPR97 | -1,131062212 | -1,112851844 | -0,01821037 | |
| 220521_s_at | ATG16L1 | -1,131062212 | -1,112851844 | -0,01821037 | |
| 226218_at | IL7R | -1,131062212 | -1,112851844 | -0,01821037 | |
| 231667_at | SLC39A5 | -1,131062212 | -1,112851844 | -0,01821037 | |
| 232109_at | UBXN10 | -1,131062212 | -1,112851844 | -0,01821037 | |
| 232479_at | FERMT1 | -1,131062212 | -1,112851844 | -0,01821037 | |
| 233121_at | - | -1,131062212 | -1,112851844 | -0,01821037 | |
| 234037_s_at | - | -1,131062212 | -1,112851844 | -0,01821037 | |
| 234053_at | - | -1,131062212 | -1,112851844 | -0,01821037 | |
| 236986_at | LINC00582 | -1,131062212 | -1,112851844 | -0,01821037 | |
| 239160_at | - | -1,131062212 | -1,112851844 | -0,01821037 | |
| 243055_at | - | -1,131062212 | -1,112851844 | -0,01821037 | |
| 201833_at | HDAC2 | 5,582563715 | 5,60084548 | -0,01828176 | |
| 203861_s_at | ACTN2 | 0,334552598 | 0,352836757 | -0,01828416 | |
| 206701_x_at | EDNRB | 0,334552598 | 0,352836757 | -0,01828416 | |
| 222060_at | KRT8P12 | 0,334552598 | 0,352836757 | -0,01828416 | |
| 238902_at | - | 0,334552598 | 0,352836757 | -0,01828416 | |
| 222469_s_at | TOLLIP | 1,011212064 | 1,029536893 | -0,01832483 | |
| 213226_at | CCNA2 | 4,580712136 | 4,599047207 | -0,01833507 | |
| 208627_s_at | YBX1 | 7,182997545 | 7,201340869 | -0,01834332 | |
| 227388_at | TUSC1 | 4,368029766 | 4,386375749 | -0,01834598 | |
| 212166_at | XPO7 | 3,097712429 | 3,116064987 | -0,01835256 | |
| 222688_at | ACER3 | 1,331241794 | 1,349621332 | -0,01837954 | |
| 200771_at | LAMC1 | 3,681090166 | 3,69948842 | -0,01839825 | |
| 225836_s_at | C12orf32 | 2,178241192 | 2,196649505 | -0,01840831 | |
| 225862_at | SLC25A26 | 2,531373922 | 2,549796788 | -0,01842287 | |
| 217810_x_at | LARS | 4,047898031 | 4,066445436 | -0,0185474 | |
| 202554_s_at | GSTM3 | 5,452973635 | 5,471571483 | -0,01859785 | |
| 213708_s_at | MLX | 3,729025365 | 3,747642277 | -0,01861691 | |
| 221427_s_at | CCNL2 | 2,001086023 | 2,019703483 | -0,01861746 | |
| 236957_at | CDCA2 | 2,001086023 | 2,019703483 | -0,01861746 | |
| 211501_s_at | EIF3B | 3,582860873 | 3,601570866 | -0,01870999 | |
| 1556454_a_at | LOC100506274 | -2,908843709 | -2,89013181 | -0,0187119 | |
| 1559221_at | LOC100507254 | -2,908843709 | -2,89013181 | -0,0187119 | |
| 1560142_at | GRIK2 | -2,908843709 | -2,89013181 | -0,0187119 | |
| 1563963_at | - | -2,908843709 | -2,89013181 | -0,0187119 | |
| 1569988_at | - | -2,908843709 | -2,89013181 | -0,0187119 | |
| 1570621_at | - | -2,908843709 | -2,89013181 | -0,0187119 | |
| 202015_x_at | - | -2,908843709 | -2,89013181 | -0,0187119 | |
| 208411_x_at | PPEF2 | -2,908843709 | -2,89013181 | -0,0187119 | |
| 211170_s_at | PDE10A | -2,908843709 | -2,89013181 | -0,0187119 | |
| 212560_at | SORL1 | -2,908843709 | -2,89013181 | -0,0187119 | |
| 214591_at | KLHL4 | -2,908843709 | -2,89013181 | -0,0187119 | |
| 216443_at | - | -2,908843709 | -2,89013181 | -0,0187119 | |
| 229459_at | FAM19A5 | -2,908843709 | -2,89013181 | -0,0187119 | |
| 231294_at | - | -2,908843709 | -2,89013181 | -0,0187119 | |
| 232618_at | TXLNG2P | -2,908843709 | -2,89013181 | -0,0187119 | |
| 233415_at | - | -2,908843709 | -2,89013181 | -0,0187119 | |

| | | | | | |
|---|---|---|---|---|---|
| 234478_at | - | -2,908843709 | -2,89013181 | -0,0187119 | |
| 236644_at | RNF180 | -2,908843709 | -2,89013181 | -0,0187119 | |
| 237206_at | MYOCD | -2,908843709 | -2,89013181 | -0,0187119 | |
| 240326_at | - | -2,908843709 | -2,89013181 | -0,0187119 | |
| 241070_at | - | -2,908843709 | -2,89013181 | -0,0187119 | |
| 242840_at | - | -2,908843709 | -2,89013181 | -0,0187119 | |
| 243427_at | - | -2,908843709 | -2,89013181 | -0,0187119 | |
| 243753_at | C1orf173 | -2,908843709 | -2,89013181 | -0,0187119 | |
| 244119_at | LINC00551 | -2,908843709 | -2,89013181 | -0,0187119 | |
| 1555390_at | NOP9 | 0,317354302 | 0,336136178 | -0,01878188 | |
| 219472_at | CENPO | 0,317354302 | 0,336136178 | -0,01878188 | |
| 224055_x_at | KCNK7 | 0,317354302 | 0,336136178 | -0,01878188 | |
| 215404_x_at | FGFR1 | 0,995078803 | 1,013870809 | -0,01879201 | |
| 223407_at | C16orf48 | 0,995078803 | 1,013870809 | -0,01879201 | |
| 227599_at | MB21D2 | 0,995078803 | 1,013870809 | -0,01879201 | |
| 238949_at | RNF145 | 0,995078803 | 1,013870809 | -0,01879201 | |
| 203253_s_at | PPIP5K2 | 4,609698349 | 4,628492984 | -0,01879464 | |
| 1554664_at | NAIF1 | -1,173767127 | -1,154897679 | -0,01886945 | |
| 1559507_at | LOC100130357 | -1,173767127 | -1,154897679 | -0,01886945 | |
| 1561126_at | - | -1,173767127 | -1,154897679 | -0,01886945 | |
| 208564_at | KCNA2 | -1,173767127 | -1,154897679 | -0,01886945 | |
| 210763_x_at | NCR3 | -1,173767127 | -1,154897679 | -0,01886945 | |
| 215678_at | LOC440792 | -1,173767127 | -1,154897679 | -0,01886945 | |
| 221345_at | FFAR2 | -1,173767127 | -1,154897679 | -0,01886945 | |
| 227480_at | SUSD2 | -1,173767127 | -1,154897679 | -0,01886945 | |
| 232487_at | SFT2D1 | -1,173767127 | -1,154897679 | -0,01886945 | |
| 233811_at | RIN2 | -1,173767127 | -1,154897679 | -0,01886945 | |
| 233963_at | H2BFXP | -1,173767127 | -1,154897679 | -0,01886945 | |
| 236437_at | - | -1,173767127 | -1,154897679 | -0,01886945 | |
| 237168_at | - | -1,173767127 | -1,154897679 | -0,01886945 | |
| 238831_at | TMEM33 | -1,173767127 | -1,154897679 | -0,01886945 | |
| 239241_at | LOC727869 | -1,173767127 | -1,154897679 | -0,01886945 | |
| 239249_at | - | -1,173767127 | -1,154897679 | -0,01886945 | |
| 240532_at | SLC32A1 | -1,173767127 | -1,154897679 | -0,01886945 | |
| 241491_at | - | -1,173767127 | -1,154897679 | -0,01886945 | |
| 241837_at | - | -1,173767127 | -1,154897679 | -0,01886945 | |
| 201336_at | VAMP3 | 4,837895809 | 4,85676571 | -0,0188699 | |
| 226038_at | LONRF1 | 3,078700536 | 3,09760452 | -0,01890398 | |
| 229848_at | ZNF10 | 0,798390451 | 0,817300337 | -0,01890989 | |
| 238785_at | - | 0,798390451 | 0,817300337 | -0,01890989 | |
| 219412_at | RAB38 | 3,199783157 | 3,218713651 | -0,01893049 | |
| 242761_s_at | ZNF420 | 4,449221925 | 4,468168179 | -0,01894625 | |
| 1560339_s_at | NAP1L4 | 3,84757742 | 3,866529339 | -0,01895192 | |
| 227728_at | PPM1A | 1,987559146 | 2,006570641 | -0,01901149 | |
| 208727_s_at | CDC42 | 4,361752105 | 4,380788377 | -0,01903627 | |
| 219613_s_at | SIRT6 | 0,57058931 | 0,589657387 | -0,01906808 | |
| 207956_x_at | PDS5B | 3,891817836 | 3,910906416 | -0,01908858 | |
| 211982_x_at | XPO6 | 3,443176398 | 3,462299654 | -0,01912326 | |
| 227442_at | COX18 | 2,683419202 | 2,702564916 | -0,01914571 | |
| 222939_s_at | SLC16A10 | 3,889637741 | 3,908790275 | -0,01915253 | |
| 201945_at | FURIN | 1,683039483 | 1,702196344 | -0,01915686 | |
| 1554348_s_at | CDKN2AIPNL | -0,43775896 | -0,41858459 | -0,01917437 | |
| 1557810_at | - | -0,43775896 | -0,41858459 | -0,01917437 | |
| 1561770_at | - | -0,43775896 | -0,41858459 | -0,01917437 | |
| 1565848_at | ZNF428 | -0,43775896 | -0,41858459 | -0,01917437 | |
| 203158_s_at | GLS | -0,43775896 | -0,41858459 | -0,01917437 | |

| | | | | | |
|---|---|---|---|---|---|
| 219824_at | SLC13A4 | -0,43775896 | -0,41858459 | -0,01917437 | |
| 223889_at | - | -0,43775896 | -0,41858459 | -0,01917437 | |
| 228320_x_at | CCDC64 | -0,43775896 | -0,41858459 | -0,01917437 | |
| 228703_at | P4HA3 | -0,43775896 | -0,41858459 | -0,01917437 | |
| 219112_at | FNIP1 /// RAPGE | 3,188003814 | 3,207279726 | -0,01927591 | |
| 1553183_at | UMODL1 | -2,866296344 | -2,847001814 | -0,01929453 | |
| 1554500_a_at | RGS7 | -2,866296344 | -2,847001814 | -0,01929453 | |
| 1556599_s_at | LOC100130503 | -2,866296344 | -2,847001814 | -0,01929453 | |
| 1557726_at | ARMCX4 | -2,866296344 | -2,847001814 | -0,01929453 | |
| 1557882_at | - | -2,866296344 | -2,847001814 | -0,01929453 | |
| 1558661_at | LOC100506207 | -2,866296344 | -2,847001814 | -0,01929453 | |
| 1558667_at | SPATA22 | -2,866296344 | -2,847001814 | -0,01929453 | |
| 1558827_a_at | ZNF831 | -2,866296344 | -2,847001814 | -0,01929453 | |
| 1561383_at | LOC284661 | -2,866296344 | -2,847001814 | -0,01929453 | |
| 1562229_at | - | -2,866296344 | -2,847001814 | -0,01929453 | |
| 1564688_a_at | RAD9B | -2,866296344 | -2,847001814 | -0,01929453 | |
| 1566485_at | - | -2,866296344 | -2,847001814 | -0,01929453 | |
| 1566539_at | - | -2,866296344 | -2,847001814 | -0,01929453 | |
| 1568852_x_at | - | -2,866296344 | -2,847001814 | -0,01929453 | |
| 1570473_at | LOC338579 | -2,866296344 | -2,847001814 | -0,01929453 | |
| 1570618_at | - | -2,866296344 | -2,847001814 | -0,01929453 | |
| 215465_at | ABCA12 | -2,866296344 | -2,847001814 | -0,01929453 | |
| 216288_at | CYSLTR1 | -2,866296344 | -2,847001814 | -0,01929453 | |
| 218730_s_at | OGN | -2,866296344 | -2,847001814 | -0,01929453 | |
| 230698_at | CALN1 | -2,866296344 | -2,847001814 | -0,01929453 | |
| 231380_at | C8orf34 | -2,866296344 | -2,847001814 | -0,01929453 | |
| 232020_at | SMURF2 | -2,866296344 | -2,847001814 | -0,01929453 | |
| 233838_at | - | -2,866296344 | -2,847001814 | -0,01929453 | |
| 234112_at | - | -2,866296344 | -2,847001814 | -0,01929453 | |
| 234582_at | - | -2,866296344 | -2,847001814 | -0,01929453 | |
| 234794_at | - | -2,866296344 | -2,847001814 | -0,01929453 | |
| 236065_at | - | -2,866296344 | -2,847001814 | -0,01929453 | |
| 236536_at | GALNT13 | -2,866296344 | -2,847001814 | -0,01929453 | |
| 238160_at | ACOT12 | -2,866296344 | -2,847001814 | -0,01929453 | |
| 239312_at | - | -2,866296344 | -2,847001814 | -0,01929453 | |
| 240539_at | - | -2,866296344 | -2,847001814 | -0,01929453 | |
| 240879_x_at | - | -2,866296344 | -2,847001814 | -0,01929453 | |
| 241032_at | ANKRD40 | -2,866296344 | -2,847001814 | -0,01929453 | |
| 241412_at | BTC | -2,866296344 | -2,847001814 | -0,01929453 | |
| 241502_x_at | - | -2,866296344 | -2,847001814 | -0,01929453 | |
| 225478_at | MFHAS1 | 2,562727117 | 2,582026171 | -0,01929905 | |
| 227814_at | WDR53 | 1,296557217 | 1,315948728 | -0,01939151 | |
| 225208_s_at | FAM103A1 | 3,78170256 | 3,801102381 | -0,01939982 | |
| 222155_s_at | SLC52A2 | 2,618203384 | 2,637603218 | -0,01939983 | |
| 228066_at | C17orf96 | 1,880387527 | 1,899812471 | -0,01942494 | |
| 226785_at | ATP11C | 2,294858597 | 2,314300277 | -0,01944168 | |
| 229812_at | USP48 | 2,429008604 | 2,448549499 | -0,01954089 | |
| 225728_at | SORBS2 | 0,291166226 | 0,310717132 | -0,01955091 | |
| 227196_at | RHPN2 | 3,823415835 | 3,843079586 | -0,01966375 | |
| 230954_at | C20orf112 | 2,134723057 | 2,154405725 | -0,01968267 | |
| 222707_s_at | ACTR8 | 2,051361651 | 2,071071048 | -0,0197094 | |
| 206180_x_at | ZNF747 | 1,283332461 | 1,303116084 | -0,01978362 | |
| 217929_s_at | KIAA0319L | 1,283332461 | 1,303116084 | -0,01978362 | |
| 230023_at | NSUN4 | 1,283332461 | 1,303116084 | -0,01978362 | |
| 236236_at | WNK3 | 2,817903185 | 2,837730973 | -0,01982779 | |
| 201601_x_at | IFITM1 /// IFITM2 | 7,065715864 | 7,085586106 | -0,01987024 | |

| | | | | | |
|---|---|---|---|---|---|
| 209108_at | TSPAN6 | 4,002888213 | 4,022758595 | -0,01987038 | |
| 1552617_a_at | RFWD2 | 2,864529541 | 2,884425449 | -0,01989591 | |
| 200597_at | EIF3A | 2,658027143 | 2,677924589 | -0,01989745 | |
| 1554690_a_at | TACC1 | 0,95671774 | 0,976641161 | -0,01992342 | |
| 225037_at | SLC35C2 | 3,12638592 | 3,146317015 | -0,01993109 | |
| 1552639_at | KLHDC7B | -1,240169874 | -1,220210588 | -0,01995929 | |
| 1556554_at | TRIM50 | -1,240169874 | -1,220210588 | -0,01995929 | |
| 1559521_at | - | -1,240169874 | -1,220210588 | -0,01995929 | |
| 1562239_at | SGSM1 | -1,240169874 | -1,220210588 | -0,01995929 | |
| 1569219_at | - | -1,240169874 | -1,220210588 | -0,01995929 | |
| 202914_s_at | ARHGEF11 | -1,240169874 | -1,220210588 | -0,01995929 | |
| 206443_at | RORB | -1,240169874 | -1,220210588 | -0,01995929 | |
| 210670_at | PPY | -1,240169874 | -1,220210588 | -0,01995929 | |
| 210689_at | CLDN14 | -1,240169874 | -1,220210588 | -0,01995929 | |
| 215368_at | NEB | -1,240169874 | -1,220210588 | -0,01995929 | |
| 224508_at | MGC12916 | -1,240169874 | -1,220210588 | -0,01995929 | |
| 225189_s_at | RAPH1 | -1,240169874 | -1,220210588 | -0,01995929 | |
| 228629_s_at | WWTR1-AS1 | -1,240169874 | -1,220210588 | -0,01995929 | |
| 229536_at | REC8 | -1,240169874 | -1,220210588 | -0,01995929 | |
| 231159_at | CXorf51A /// CXo | -1,240169874 | -1,220210588 | -0,01995929 | |
| 236510_at | - | -1,240169874 | -1,220210588 | -0,01995929 | |
| 238927_at | - | -1,240169874 | -1,220210588 | -0,01995929 | |
| 239180_at | LOC100507419 / | -1,240169874 | -1,220210588 | -0,01995929 | |
| 239239_at | BANCR | -1,240169874 | -1,220210588 | -0,01995929 | |
| 240300_at | TK2 | -1,240169874 | -1,220210588 | -0,01995929 | |
| 244711_at | - | -1,240169874 | -1,220210588 | -0,01995929 | |
| 201955_at | CCNC | 5,372258632 | 5,392251169 | -0,01999254 | |
| 238912_x_at | C9orf85 | 3,202127528 | 3,222126232 | -0,0199987 | |
| 211005_at | LAT | 4,040056475 | 4,060103278 | -0,0200468 | |
| 220035_at | NUP210 | 0,273439642 | 0,293518836 | -0,02007919 | |
| 206240_s_at | ZNF136 | 2,857110344 | 2,877228323 | -0,02011798 | |
| 220201_at | RC3H2 | 0,533835206 | 0,553993624 | -0,02015842 | |
| 228894_at | LOC253842 /// NI | 0,533835206 | 0,553993624 | -0,02015842 | |
| 201603_at | PPP1R12A | 4,606604592 | 4,626777417 | -0,02017282 | |
| 213853_at | DNAJC24 | 3,117719377 | 3,137910193 | -0,02019082 | |
| 42361_g_at | CCHCR1 | 1,116876206 | 1,137092371 | -0,02021617 | |
| 201658_at | ARL1 | 3,111496958 | 3,131875186 | -0,02037823 | |
| 50374_at | C17orf90 | 2,070734822 | 2,091116676 | -0,02038185 | |
| 204085_s_at | CLN5 | 0,747935635 | 0,768343791 | -0,02040816 | |
| 215044_s_at | STAM2 | 0,747935635 | 0,768343791 | -0,02040816 | |
| 225380_at | PKDCC | 0,747935635 | 0,768343791 | -0,02040816 | |
| 205802_at | TRPC1 | 1,638726528 | 1,659204017 | -0,02047749 | |
| 216248_s_at | NR4A2 | 1,844820248 | 1,865308541 | -0,02048829 | |
| 228374_at | R3HCC1L | 2,107467633 | 2,127967638 | -0,0205 | |
| 1554970_at | PDILT | -1,2845686 | -1,264005341 | -0,02056326 | |
| 1555112_a_at | C1orf114 | -1,2845686 | -1,264005341 | -0,02056326 | |
| 1558154_at | - | -1,2845686 | -1,264005341 | -0,02056326 | |
| 1558238_at | TNRC18 | -1,2845686 | -1,264005341 | -0,02056326 | |
| 1560149_at | SLC29A2 | -1,2845686 | -1,264005341 | -0,02056326 | |
| 206325_at | SERPINA6 | -1,2845686 | -1,264005341 | -0,02056326 | |
| 222933_at | MORN1 | -1,2845686 | -1,264005341 | -0,02056326 | |
| 226071_at | ADAMTSL4 | -1,2845686 | -1,264005341 | -0,02056326 | |
| 227317_at | LMCD1 | -1,2845686 | -1,264005341 | -0,02056326 | |
| 228296_at | YPEL1 | -1,2845686 | -1,264005341 | -0,02056326 | |
| 233774_at | - | -1,2845686 | -1,264005341 | -0,02056326 | |
| 234119_at | - | -1,2845686 | -1,264005341 | -0,02056326 | |

| | | | | | |
|---|---|---|---|---|---|
| 235669_at | AGXT2L2 | -1,2845686 | -1,264005341 | -0,02056326 | |
| 236153_at | - | -1,2845686 | -1,264005341 | -0,02056326 | |
| 236470_at | - | -1,2845686 | -1,264005341 | -0,02056326 | |
| 239858_at | - | -1,2845686 | -1,264005341 | -0,02056326 | |
| 242925_at | RNF148 | -1,2845686 | -1,264005341 | -0,02056326 | |
| 244208_at | - | -1,2845686 | -1,264005341 | -0,02056326 | |
| 201251_at | PKM | 3,361633475 | 3,382199319 | -0,02056584 | |
| 215737_x_at | USF2 | 2,889473182 | 2,910041809 | -0,02056863 | |
| 212061_at | U2SURP | 2,635653293 | 2,656223838 | -0,02057055 | |
| 1552878_at | ATOH7 | -2,756478458 | -2,735862426 | -0,02061603 | |
| 1553024_at | DAOA-AS1 | -2,756478458 | -2,735862426 | -0,02061603 | |
| 1554816_at | ASTN2 | -2,756478458 | -2,735862426 | -0,02061603 | |
| 1555079_at | SPATA6L | -2,756478458 | -2,735862426 | -0,02061603 | |
| 1564627_at | DNAJC13 | -2,756478458 | -2,735862426 | -0,02061603 | |
| 1564628_at | - | -2,756478458 | -2,735862426 | -0,02061603 | |
| 1570470_at | CATSPERB | -2,756478458 | -2,735862426 | -0,02061603 | |
| 201789_at | DHRS7 | -2,756478458 | -2,735862426 | -0,02061603 | |
| 207471_at | - | -2,756478458 | -2,735862426 | -0,02061603 | |
| 208170_s_at | TRIM31 | -2,756478458 | -2,735862426 | -0,02061603 | |
| 208586_s_at | SSX4 /// SSX4B | -2,756478458 | -2,735862426 | -0,02061603 | |
| 215494_at | - | -2,756478458 | -2,735862426 | -0,02061603 | |
| 215901_at | ZNF81 | -2,756478458 | -2,735862426 | -0,02061603 | |
| 216104_at | LOC100505960 | -2,756478458 | -2,735862426 | -0,02061603 | |
| 216444_at | - | -2,756478458 | -2,735862426 | -0,02061603 | |
| 217371_s_at | IL15 | -2,756478458 | -2,735862426 | -0,02061603 | |
| 219909_at | MMP28 | -2,756478458 | -2,735862426 | -0,02061603 | |
| 227174_at | WDR72 | -2,756478458 | -2,735862426 | -0,02061603 | |
| 228547_at | NRXN1 | -2,756478458 | -2,735862426 | -0,02061603 | |
| 229778_at | C12orf39 | -2,756478458 | -2,735862426 | -0,02061603 | |
| 234780_at | - | -2,756478458 | -2,735862426 | -0,02061603 | |
| 235494_at | LSAMP | -2,756478458 | -2,735862426 | -0,02061603 | |
| 236349_at | - | -2,756478458 | -2,735862426 | -0,02061603 | |
| 237741_at | SLC25A36 | -2,756478458 | -2,735862426 | -0,02061603 | |
| 237814_at | - | -2,756478458 | -2,735862426 | -0,02061603 | |
| 237963_x_at | - | -2,756478458 | -2,735862426 | -0,02061603 | |
| 240218_at | DSCAM | -2,756478458 | -2,735862426 | -0,02061603 | |
| 242173_at | - | -2,756478458 | -2,735862426 | -0,02061603 | |
| 243241_at | - | -2,756478458 | -2,735862426 | -0,02061603 | |
| 243419_at | - | -2,756478458 | -2,735862426 | -0,02061603 | |
| 244316_at | C17orf98 | -2,756478458 | -2,735862426 | -0,02061603 | |
| 214437_s_at | SHMT2 | 3,423218668 | 3,443906002 | -0,02068733 | |
| 227516_at | SF3A1 | 1,515954005 | 1,536652708 | -0,0206987 | |
| 225747_at | COQ10A | 1,390002893 | 1,410714371 | -0,02071148 | |
| 229864_at | C3orf75 | 1,390002893 | 1,410714371 | -0,02071148 | |
| 221653_x_at | APOL2 | 1,251994932 | 1,272721751 | -0,02072682 | |
| 207318_s_at | CDK13 | 2,320980353 | 2,341749893 | -0,02076954 | |
| 211014_s_at | PML | 0,735041319 | 0,755840599 | -0,02079928 | |
| 219917_at | ZCCHC4 | 0,735041319 | 0,755840599 | -0,02079928 | |
| 212221_x_at | IDS | 2,094906514 | 2,115788273 | -0,02088176 | |
| 213517_at | PCBP2 | 0,246434561 | 0,26733076 | -0,0208962 | |
| 204472_at | GEM | 1,094335495 | 1,115234684 | -0,02089919 | |
| 226750_at | LARP1B | 2,243430036 | 2,264418116 | -0,02098808 | |
| 206688_s_at | CPSF4 | 3,34687727 | 3,367892567 | -0,0210153 | |
| 1557915_s_at | GSTO1 | 6,961331573 | 6,982375655 | -0,02104408 | |
| 204335_at | CCDC94 | 1,504602299 | 1,525647381 | -0,02104508 | |
| 222752_s_at | TMEM206 | 3,088871381 | 3,109937707 | -0,02106633 | |

| | | | | | |
|---|---|---|---|---|---|
| 1552733_at | KLHDC1 | -2,712026959 | -2,690860744 | -0,02116622 | |
| 1553197_at | DCAF4L2 | -2,712026959 | -2,690860744 | -0,02116622 | |
| 1553912_at | FLJ35424 | -2,712026959 | -2,690860744 | -0,02116622 | |
| 1557177_at | LOC100506258 | -2,712026959 | -2,690860744 | -0,02116622 | |
| 1557699_x_at | - | -2,712026959 | -2,690860744 | -0,02116622 | |
| 1558075_at | LOC399491 | -2,712026959 | -2,690860744 | -0,02116622 | |
| 1559807_at | - | -2,712026959 | -2,690860744 | -0,02116622 | |
| 1560834_a_at | RMST | -2,712026959 | -2,690860744 | -0,02116622 | |
| 1560973_a_at | - | -2,712026959 | -2,690860744 | -0,02116622 | |
| 1561297_at | WARS2 | -2,712026959 | -2,690860744 | -0,02116622 | |
| 1562071_at | - | -2,712026959 | -2,690860744 | -0,02116622 | |
| 1563487_at | - | -2,712026959 | -2,690860744 | -0,02116622 | |
| 1566867_at | - | -2,712026959 | -2,690860744 | -0,02116622 | |
| 1568840_at | DCAF13 | -2,712026959 | -2,690860744 | -0,02116622 | |
| 210853_at | SCN11A | -2,712026959 | -2,690860744 | -0,02116622 | |
| 221086_s_at | FEZF2 | -2,712026959 | -2,690860744 | -0,02116622 | |
| 221802_s_at | KIAA1598 | -2,712026959 | -2,690860744 | -0,02116622 | |
| 227265_at | FGL2 | -2,712026959 | -2,690860744 | -0,02116622 | |
| 231361_at | NLGN1 | -2,712026959 | -2,690860744 | -0,02116622 | |
| 231578_at | GBP1 | -2,712026959 | -2,690860744 | -0,02116622 | |
| 232277_at | SLC28A3 | -2,712026959 | -2,690860744 | -0,02116622 | |
| 233722_at | - | -2,712026959 | -2,690860744 | -0,02116622 | |
| 233879_at | - | -2,712026959 | -2,690860744 | -0,02116622 | |
| 234418_x_at | CD44 | -2,712026959 | -2,690860744 | -0,02116622 | |
| 234467_at | - | -2,712026959 | -2,690860744 | -0,02116622 | |
| 235462_at | CPEB2 | -2,712026959 | -2,690860744 | -0,02116622 | |
| 237516_at | - | -2,712026959 | -2,690860744 | -0,02116622 | |
| 238220_at | KDM6A | -2,712026959 | -2,690860744 | -0,02116622 | |
| 239587_at | - | -2,712026959 | -2,690860744 | -0,02116622 | |
| 240211_at | LOC100130468 | -2,712026959 | -2,690860744 | -0,02116622 | |
| 241122_s_at | MUSK | -2,712026959 | -2,690860744 | -0,02116622 | |
| 242410_s_at | CACNA1E | -2,712026959 | -2,690860744 | -0,02116622 | |
| 244645_at | - | -2,712026959 | -2,690860744 | -0,02116622 | |
| 1555446_s_at | TRAPPC10 | 2,86748657 | 2,888726553 | -0,02123998 | |
| 39402_at | IL1B | 0,083058036 | 0,104298896 | -0,02124086 | |
| 212053_at | PDXDC1 | 2,819431021 | 2,840694075 | -0,02126305 | |
| 209565_at | RNF113A | 2,371846398 | 2,39311332 | -0,02126692 | |
| 233369_at | - | 0,496120222 | 0,51742585 | -0,02130563 | |
| 238681_at | GDPD1 | 0,496120222 | 0,51742585 | -0,02130563 | |
| 221676_s_at | CORO1C | 6,120291318 | 6,141606798 | -0,02131548 | |
| 1561530_at | - | -0,512251684 | -0,490927335 | -0,02132435 | |
| 202504_at | TRIM29 | -0,512251684 | -0,490927335 | -0,02132435 | |
| 203425_s_at | IGFBP5 | -0,512251684 | -0,490927335 | -0,02132435 | |
| 204922_at | C11orf80 | -0,512251684 | -0,490927335 | -0,02132435 | |
| 206650_at | IQCC | -0,512251684 | -0,490927335 | -0,02132435 | |
| 206735_at | CHRNA4 | -0,512251684 | -0,490927335 | -0,02132435 | |
| 213622_at | COL9A2 | -0,512251684 | -0,490927335 | -0,02132435 | |
| 214541_s_at | QKI | -0,512251684 | -0,490927335 | -0,02132435 | |
| 232374_s_at | - | -0,512251684 | -0,490927335 | -0,02132435 | |
| 241490_s_at | PGBD2 | -0,512251684 | -0,490927335 | -0,02132435 | |
| 1553955_at | PPP1R21 | 1,369290453 | 1,390636111 | -0,02134566 | |
| 220051_at | PRSS21 | 4,099113343 | 4,12047658 | -0,02136324 | |
| 238760_at | YARS | 0,715481152 | 0,73688026 | -0,02139911 | |
| 204809_at | CLPX | 3,42923503 | 3,450709925 | -0,02147489 | |
| 224960_at | SCYL2 | 3,787958926 | 3,809444866 | -0,02148594 | |
| 215179_x_at | PGF | 2,947914474 | 2,969455976 | -0,0215415 | |

| | | | | | |
|---|---|---|---|---|---|
| 1555881_s_at | LZTS2 | 2,427005622 | 2,448549499 | -0,02154388 | |
| 202959_at | MUT | 1,603688695 | 1,6252386 | -0,02154991 | |
| 1554763_at | UBE2DNL | -1,353583928 | -1,332017329 | -0,0215666 | |
| 1564400_at | C18orf62 | -1,353583928 | -1,332017329 | -0,0215666 | |
| 207334_s_at | TGFBR2 | -1,353583928 | -1,332017329 | -0,0215666 | |
| 214890_s_at | FAM149A | -1,353583928 | -1,332017329 | -0,0215666 | |
| 225908_at | IAH1 | -1,353583928 | -1,332017329 | -0,0215666 | |
| 231404_at | SPTY2D1-AS1 | -1,353583928 | -1,332017329 | -0,0215666 | |
| 232887_at | PIRT | -1,353583928 | -1,332017329 | -0,0215666 | |
| 235787_at | CDC37L1 | -1,353583928 | -1,332017329 | -0,0215666 | |
| 236059_at | LOC100130428 | -1,353583928 | -1,332017329 | -0,0215666 | |
| 237535_x_at | NRXN1 | -1,353583928 | -1,332017329 | -0,0215666 | |
| 238373_at | H1FNT | -1,353583928 | -1,332017329 | -0,0215666 | |
| 240470_at | - | -1,353583928 | -1,332017329 | -0,0215666 | |
| 242850_at | - | -1,353583928 | -1,332017329 | -0,0215666 | |
| 244857_at | HBEGF | -1,353583928 | -1,332017329 | -0,0215666 | |
| 201883_s_at | B4GALT1 | 2,710020346 | 2,731588328 | -0,02156798 | |
| 228495_at | CCDC75 | 2,710020346 | 2,731588328 | -0,02156798 | |
| 203554_x_at | PTTG1 | 5,47954976 | 5,501134613 | -0,02158485 | |
| 211200_s_at | EFCAB2 | 0,900083947 | 0,921731975 | -0,02164803 | |
| 201012_at | ANXA1 | 7,047130775 | 7,06878371 | -0,02165293 | |
| 218891_at | C10orf76 | 2,069451339 | 2,091116676 | -0,02166534 | |
| 212175_s_at | AK2 | 4,626378238 | 4,648076762 | -0,02169852 | |
| 242695_at | - | 1,805273023 | 1,826973888 | -0,02170086 | |
| 213610_s_at | KLHL23 /// PHOS | 2,705069849 | 2,726791458 | -0,02172161 | |
| 234897_s_at | LY6G6D /// LY6G | 2,355089531 | 2,376875125 | -0,02178559 | |
| 211765_x_at | PPIA | 7,80826997 | 7,830064196 | -0,02179423 | |
| 229061_s_at | SLC25A13 | 2,938102794 | 2,959949103 | -0,02184631 | |
| 222190_s_at | C16orf58 | 1,215326747 | 1,23718272 | -0,02185597 | |
| 1552703_s_at | CARD16 /// CASP | -2,64318869 | -2,621331989 | -0,0218567 | |
| 1553787_at | C11orf45 | -2,64318869 | -2,621331989 | -0,0218567 | |
| 1553888_at | LDHAL6A | -2,64318869 | -2,621331989 | -0,0218567 | |
| 1556476_at | LOC283485 | -2,64318869 | -2,621331989 | -0,0218567 | |
| 1557443_s_at | LOC100505811 | -2,64318869 | -2,621331989 | -0,0218567 | |
| 1557736_at | NKTR | -2,64318869 | -2,621331989 | -0,0218567 | |
| 1558449_at | - | -2,64318869 | -2,621331989 | -0,0218567 | |
| 1560490_at | FAT3 | -2,64318869 | -2,621331989 | -0,0218567 | |
| 1561084_at | - | -2,64318869 | -2,621331989 | -0,0218567 | |
| 1561482_at | LOC100133308 | -2,64318869 | -2,621331989 | -0,0218567 | |
| 1561592_at | - | -2,64318869 | -2,621331989 | -0,0218567 | |
| 1564882_at | - | -2,64318869 | -2,621331989 | -0,0218567 | |
| 1569023_a_at | LINC00458 | -2,64318869 | -2,621331989 | -0,0218567 | |
| 1569677_a_at | MCMDC2 | -2,64318869 | -2,621331989 | -0,0218567 | |
| 206326_at | GRP | -2,64318869 | -2,621331989 | -0,0218567 | |
| 206676_at | CEACAM8 | -2,64318869 | -2,621331989 | -0,0218567 | |
| 212230_at | PPAP2B | -2,64318869 | -2,621331989 | -0,0218567 | |
| 214807_at | - | -2,64318869 | -2,621331989 | -0,0218567 | |
| 216573_at | IGLV1-44 | -2,64318869 | -2,621331989 | -0,0218567 | |
[truncated: 2,343,465 more chars]
